# Estimates and 25-year trends of the global burden of disease attributable to ambient air pollution: an analysis of data from the Global Burden of Diseases Study 2015

**DOI:** 10.1016/S0140-6736(17)30505-6

**Published:** 2017-05-13

**Authors:** Aaron J Cohen, Michael Brauer, Richard Burnett, H Ross Anderson, Joseph Frostad, Kara Estep, Kalpana Balakrishnan, Bert Brunekreef, Lalit Dandona, Rakhi Dandona, Valery Feigin, Greg Freedman, Bryan Hubbell, Amelia Jobling, Haidong Kan, Luke Knibbs, Yang Liu, Randall Martin, Lidia Morawska, C Arden Pope, Hwashin Shin, Kurt Straif, Gavin Shaddick, Matthew Thomas, Rita van Dingenen, Aaron van Donkelaar, Theo Vos, Christopher J L Murray, Mohammad H Forouzanfar

**Affiliations:** a Health Effects Institute, Boston, MA, USA; b University of British Columbia, Vancouver, BC, Canada; c Health Canada, Ottawa, ON, Canada; d St George's, University of London, London, UK; e Institute for Health Metrics and Evaluation, Seattle, WA, USA; f Sri Ramachandra University, Chennai, Tamil Nadu, India; g University of Utrecht, Utrecht, Netherlands; h Public Health Foundation of India, New Delhi, India; i Auckland University of Technology, Auckland, New Zealand; j United States Environmental Protection Agency, Washington, DC, USA; k University of Bath, Bath, UK; l Fudan University, Yangpu Qu, Shanghai, China; m University of Queensland, St Lucia, QLD, Australia; n Emory University, Atlanta, GA, USA; o Dalhousie University, Halifax, NS, Canada; p Queensland University of Technology, Brisbane, QLD, Australia; q Brigham Young University, Provo, UT, USA; r International Agency for Research on Cancer, Lyon, France; s European Commission, Brussels, Belgium

## Abstract

**Background:**

Exposure to ambient air pollution increases morbidity and mortality, and is a leading contributor to global disease burden. We explored spatial and temporal trends in mortality and burden of disease attributable to ambient air pollution from 1990 to 2015 at global, regional, and country levels.

**Methods:**

We estimated global population-weighted mean concentrations of particle mass with aerodynamic diameter less than 2·5 μm (PM_2·5_) and ozone at an approximate 11 km × 11 km resolution with satellite-based estimates, chemical transport models, and ground-level measurements. Using integrated exposure–response functions for each cause of death, we estimated the relative risk of mortality from ischaemic heart disease, cerebrovascular disease, chronic obstructive pulmonary disease, lung cancer, and lower respiratory infections from epidemiological studies using non-linear exposure–response functions spanning the global range of exposure.

**Findings:**

Ambient PM_2·5_ was the fifth-ranking mortality risk factor in 2015. Exposure to PM_2·5_ caused 4·2 million (95% uncertainty interval [UI] 3·7 million to 4·8 million) deaths and 103·1 million (90·8 million 115·1 million) disability-adjusted life-years (DALYs) in 2015, representing 7·6% of total global deaths and 4·2% of global DALYs, 59% of these in east and south Asia. Deaths attributable to ambient PM_2·5_ increased from 3·5 million (95% UI 3·0 million to 4·0 million) in 1990 to 4·2 million (3·7 million to 4·8 million) in 2015. Exposure to ozone caused an additional 254 000 (95% UI 97 000–422 000) deaths and a loss of 4·1 million (1·6 million to 6·8 million) DALYs from chronic obstructive pulmonary disease in 2015.

**Interpretation:**

Ambient air pollution contributed substantially to the global burden of disease in 2015, which increased over the past 25 years, due to population ageing, changes in non-communicable disease rates, and increasing air pollution in low-income and middle-income countries. Modest reductions in burden will occur in the most polluted countries unless PM_2·5_ values are decreased substantially, but there is potential for substantial health benefits from exposure reduction.

**Funding:**

Bill & Melinda Gates Foundation and Health Effects Institute.

## Introduction

Exposure to ambient air pollution increases mortality and morbidity and shortens life expectancy.^1^, ^2^ The Global Burden of Diseases, Injuries, and Risk Factors Study 2015 (GBD 2015) estimated the burden of disease attributable to 79 risk factors in 195 countries from 1990 to 2015. GBD 2015 identified air pollution as a leading cause of global disease burden, especially in low-income and middle-income countries.^3^ In view of the important role of public policy in mitigating this risk and the potential for substantial health benefits related to efforts to reduce emissions of climate-forcing agents, we explored spatial and temporal trends in mortality and burden of disease attributable to ambient air pollution from 1990 to 2015 at global, regional, and country levels.

## Methods

### Overview

Attributing deaths and disability-adjusted life-years (DALYs) to ambient air pollution requires spatially and temporally resolved estimates of population-weighted exposure, specification of a theoretical minimum risk exposure level (TMREL), estimation of relative risks across the exposure distribution, and estimates of the deaths and DALYs for diseases linked causally to air pollution. We combined estimates of exposure and relative risk to estimate the population-attributable fraction (PAF), the proportion of deaths and DALYs attributable to exposure above the TMREL. The numbers of deaths and DALYs for specific diseases were multiplied by the PAF to estimate the burden attributable to exposure. A more general description of the methods used to estimate the PAF and attributable burdens in GBD 2015 has been reported previously;^3^ here, we present details specific to air pollution.

**Research in context**

**Evidence before this study**

Literature reviews done by the US Environmental Protection Agency, WHO, and others have shown that long-term exposure to ambient air pollution increases mortality and morbidity from cardiovascular and respiratory disease and lung cancer and shortens life expectancy. Based on this evidence, the Global Burden of Diseases, Injuries, and Risk Factors Study 2015 estimated the burden of disease attributable to 79 risk factors, including ambient air pollution, in 195 countries and territories from 1990 to 2015. The Global Burden of Diseases, Injuries, and Risk Factors Study 2015 identified air pollution as a leading cause of global disease burden, especially in low-income and middle-income countries.

**Added value of this study**

In this Article, we show the crucial part played by broad demographic and epidemiological trends. We estimated that long-term exposure to ambient fine particle air pollution (PM_2·5_) caused 4·2 million deaths and 103·1 million lost years of healthy life in 2015, representing 7·6% of total global mortality, making it the fifth-ranked global risk factor in 2015. Exposure to ozone was responsible for an additional 254 000 deaths. Although global rates of mortality due to PM_2·5_ exposure decreased from 1990 to 2015, the absolute numbers of attributable deaths and disability-adjusted life-years increased because of rising levels of pollution and increasing numbers of deaths from non-communicable diseases in the largest low-income and middle-income countries in east and south Asia, where populations are growing and ageing. This research places the burden of disease as a result of ambient air pollution within the context of other common potentially modifiable risk factors at a national level, helping to prioritise air pollution from a population health perspective. In the analysis of trends in the burden of disease caused by ambient air pollution, we show where, and the extent to which, ambient air pollution is changing as a contributor to disease burden, and the extent to which the trends in the attributable burden reflect progress, or absence of it, in reducing exposure versus changes in demographic factors. In so doing, we were able to elucidate the challenges that must be overcome to reduce the public health effects of exposure to air pollution.

**Implications of all the available evidence**

Ambient air pollution was a leading risk factor for the global burden of disease in 2015 and has remained stable, and its contribution to the global burden of disease has remained relatively stable, from 1990 to 2015. Trends in attributable deaths reflect both demographic and epidemiological trends and increasing levels of air pollution in low-income and middle-income countries. Should these trends continue, they will lead to increasing burdens if major reductions are not made in pollution levels. Non-linear exposure–response functions suggest modest reductions in burden in the most polluted countries unless PM_2·5_ levels markedly decline. As a result, the challenges for future reductions in the burden of disease attributable to air pollution are substantial. International experience has shown that exposure to ambient air pollution and its associated burden of disease can be lowered for entire populations via policy action at the national and subnational levels via aggressive air quality management programmes, focused on major sources of air pollution.

GBD 2015 extended the methods and datasets used in GBD 2013 to estimate the burden of disease attributable to ambient air pollution for 1990–2015.^4^, ^5^ Estimates of exposure to ambient air pollution were updated with additional air pollution measurements and improved estimation methods,^3^, ^6^ relative risk estimates were updated using more recent epidemiological studies and refined statistical estimation techniques,^3^ and new methods were developed and applied to identify key drivers of global trends in the burden of disease attributable to ambient air pollution. The GBD 2015 estimates update the entire time series beginning in 1990, so the changes in methodology affect the estimates for each year in the 1990–2015 interval and supersede estimates previously released.

### Estimation of exposure

Air pollution is a complex mixture of gases and particles whose sources and composition vary spatially and temporally. Population-weighted annual mean concentrations of particle mass with aerodynamic diameter less than 2·5 μm (PM_2·5_) and tropospheric ozone are the two indicators used to quantify exposure to air pollution. PM_2·5_ is the most consistent and robust predictor of mortality in studies of long-term exposure.^7^, ^8^ Ozone, a gas produced via atmospheric reactions of precursor emissions, is associated with respiratory disease independent of PM_2·5_ exposure.^9^, ^10^ We estimated exposure to PM_2·5_ and ozone for the global population at spatial scales relevant to human exposure (appendix pp 2–4).^3^, ^6^ Global annual mean exposure to PM_2·5_ was estimated in 5-year intervals from 1990 to 2015, at 0·1 × 0·1° (∼11 km × 11 km at the equator) resolution using estimates from satellites combined with a chemical transport model, surface measurements, and geographical data. We aggregated gridded exposure concentrations to national-level population-weighted means using the corresponding grid cell population value.^11^ National-level population-weighted mean concentrations and the 95% uncertainty interval (95% UI) around this mean were estimated by sampling 1000 draws of each grid cell value and its uncertainty distribution.

As in previous assessments,^4^, ^5^ we used a chemical transport model to calculate a running 3-month mean (of daily 1 h maximum values) ozone concentration for each grid cell over 1 year, from which we selected the maximum of these values, consistent with epidemiological studies that use a seasonal (summer) mean, while accounting for global variation in the timing of the ozone (summer) season. We estimated population-weighted mean ozone concentrations and 95% UIs for each country as described for PM_2·5_, assuming a normal distribution with a 95% UI within 6% either side of the estimated mean concentration.

### Theoretical minimum risk exposure level

TMREL was assigned a uniform distribution of 2·4–5·9 μg/m^3^ for PM_2·5_ and 33·3–41·9 parts per billion for ozone, bounded by the minimum and fifth percentiles of exposure distributions from outdoor air pollution cohort studies (appendix pp 7, 11–14). The uniform distribution represents the uncertainty regarding adverse effects of low-level exposure.^3^, ^12^, ^13^

### Risk estimation

We estimated the burden attributable to PM_2·5_ for ischaemic heart disease (IHD), cerebrovascular disease (ischaemic stroke and haemorrhagic stroke), lung cancer, chronic obstructive pulmonary disease (COPD), and lower respiratory infections (LRI), and the burden attributable to ozone for COPD.^3^, ^14^ Evidence linking these diseases with exposure to ambient air pollution was judged to be consistent with a causal relationship on the basis of criteria specified for GBD risk factors.^3^

We developed integrated exposure–response functions (IERs) for each cause of death to estimate the relative risk of mortality over the entire global range of ambient annual mean PM_2·5_ concentrations using risk estimates from studies of ambient air pollution, household air pollution, and second-hand smoke exposure and active smoking (appendix pp 8–14).^12^, ^14^ IERs assign concentrations of PM_2·5_ to each type of exposure on an equivalent μg/m^3^ basis assuming that risk is determined by the 24-h PM_2·5_ inhaled dose regardless of the exposure source, consistent with previous findings.^15^, ^16^ We updated IERs from those used in GBD 2013 by adding additional risk estimates for air pollution (appendix pp 11–14) and active smoking.^17^ An alternative method to estimate exposure to second-hand smoke was used that incorporated estimates of PM_2·5_ attributable to exposure per cigarette, breathing rate, and number of cigarettes smoked in the country where each study was done. Further details are provided in the appendix (pp 8–14).

The IER has the mathematical form:

$$IER\left(z\right)=1+\alpha \times \left(1-e^{\beta {\left(z-z_{cf}\right)}^{\gamma }+}\right)$$

where *z* is the level of PM_2·5_ and *z*_cf_ is the TMREL, below which no additional risk is assumed, with

$${\left(z-z_{cf}\right)}_{+}=\left(z-z_{cf}\right)$$

if *z* is greater than *z*_cf_ and zero otherwise. Here, 1 + α is the maximum risk, β is the ratio of the IER at low to high concentrations, and ^γ^ is the power of PM_2·5_ concentration.

Epidemiological evidence suggests that the relative risks for IHD and stroke decline with age.^18^ We modified the particulate matter source-specific relative risk for both IHD and stroke mortality as described by Burnett and colleagues^12^ and applied this age modification to the relative risks, fitting the IER model for each age group separately.

Observed relative risks were related to the IER within a Bayesian framework using the STAN fitting algorithm, as described in the appendix (pp 15–17). Given the true values of the four parameters (α, β, γ, *z*_cf_), we assumed that the logarithm of each study's observed relative risk was normally distributed, with mean defined by the IER and variance given by the square of the observed SE of the study-specific log-relative risk estimate plus an additional variance term for each of the four sources on PM_2·5_ exposure (outdoor air pollution, second-hand smoke, household air pollution, and active smoking). Details regarding model fitting and code are provided in the appendix (pp 15–17).

We calculated 1000 predicted values of the IER for each PM_2·5_ concentration based on the posterior distributions of (α, β, γ) and the prespecified uniform distribution of TMREL to characterise uncertainty in the estimates of the IER. The mean of the 1000 IER predictions at each concentration was used as the central estimate, with uncertainty defined by 95% UIs.

We estimated the relative risk of COPD mortality from ozone exposure using a linear exposure–response function for respiratory mortality from Jerrett and colleagues.^10^ Additional details are provided in the appendix (p 14).

### Estimation of PAF and burden

We calculated DALYs and deaths attributable to ambient air pollution by applying the year-specific, location-specific, age-specific, and sex-specific PAF to the numbers of DALYs and deaths, as described in detail elsewhere.^19^, ^20^ PAF estimation methods are summarised in the appendix (pp 20–21).

### Role of the funding source

The funders of the study had no role in study design, data collection, data analysis, data interpretation, or writing of the report. All authors had full access to all the data in the study and AJC and MB had final responsibility for the decision to submit for publication.

## Results

**Figure 1:**
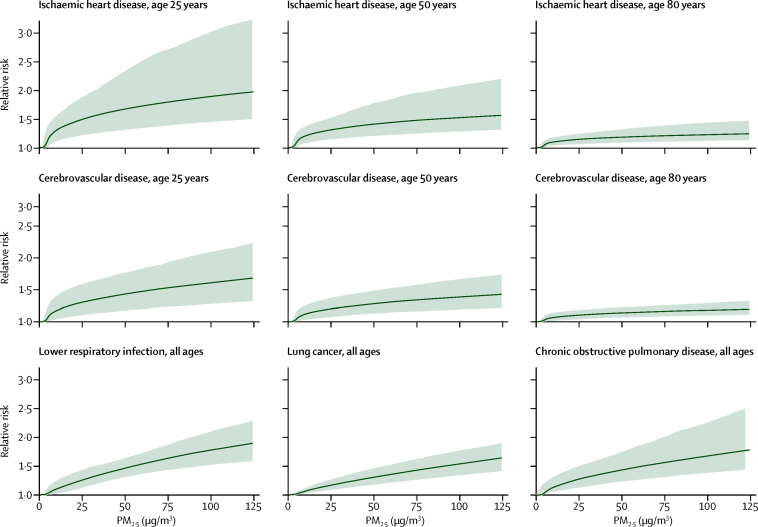
Integrated exposure–response functions Curves show the central estimate of the integrated exposure–response (solid lines) and their 95% uncertainty intervals (shaded areas). The relative risk equals 1 for PM_2·5_ concentrations of 0–2·4 μg/m^3^ (ie, lower bound of the theoretical minimum risk exposure level uncertainty distribution). Additional details are provided in the appendix (pp 7–15). PM_2·5_=particle mass with aerodynamic diameter less than 2·5 μm.

Figure 1 shows IERs for the five causes of death. The functions are all non-linear, with a greater change in relative risk for lower concentrations compared with higher values. We fit age-specific functions for IHD and cerebrovascular disease, and estimated decreasing relative risks as age increased from 25 years to 80 years.

**Figure 2:**
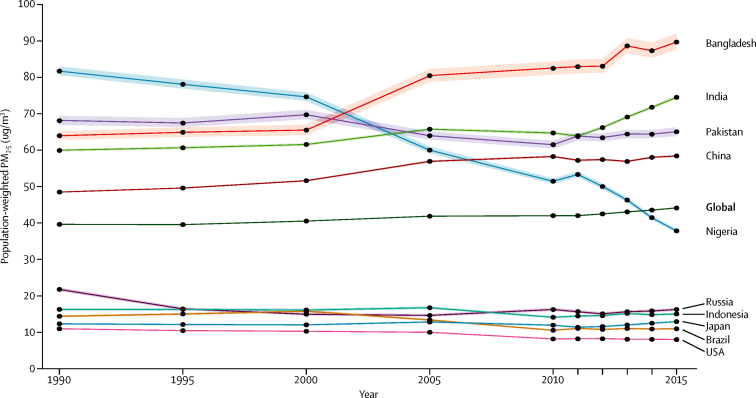
Trends in population-weighted mean concentrations of particle mass with aerodynamic diameter less than 2·5 μm Global data and data from the ten most populous countries are shown. Shaded areas are 95% uncertainty intervals. PM_2·5_=particle mass with aerodynamic diameter less than 2·5 μm.

Global population-weighted PM_2·5_ increased by 11·2% from 1990 (39·7 μg/m^3^) to 2015 (44·2 μg/m^3^), increasing most rapidly from 2010 to 2015 (figure 2). Among the world's ten most populous countries, exposures since 2010 increased in Bangladesh and India and were stable but remained high in Pakistan and China. Exposures decreased substantially in Nigeria and were low and slightly decreased in the USA, Brazil, and Russia. Population-weighted concentrations were low and stable in Japan and Indonesia.

The highest estimated population-weighted mean concentrations in 2015 were for Qatar (107·3 μg/m^3^), Saudi Arabia (106·2 μg/m^3^), and Egypt (104·7 μg/m^3^), followed by Bangladesh (89·4 μg/m^3^), Mauritania (85·1 μg/m^3^), Libya (79·2 μg/m^3^), Nepal (75·0 μg/m^3^), and India (74·3 μg/m^3^). The population-weighted mean PM_2·5_ in China was 58·4 μg/m^3^, with provincial population-weighted means ranging from 19·1 μg/m^3^ to 79·3 μg/m^3^. The lowest estimated population-weighted means were in several Pacific island nations and territories (Kiribati, American Samoa, Samoa, Tonga, Solomon Islands, Fiji, and Guam), Brunei, Sweden, Greenland, New Zealand, Australia, Finland, Iceland, Liberia, and Canada (all ≤8·0 μg/m^3^).

Population-weighted ozone levels increased by 7·2% globally from 1990 (56·8 parts per billion [ppb]) to 2015 (60·9 ppb). Within the world's ten most populous countries, increases of 14–25% were noted in China, India, Pakistan, Bangladesh, and Brazil, with smaller increases in Japan and negligible changes in Russia and Nigeria (data not shown). Decreases in population-weighted concentrations were noted in the USA (5·2%; from 70·2 ppb to 66·5 ppb) and Indonesia (12·9%; from 50·2 ppb to 43·7 ppb).

**Figure 3:**
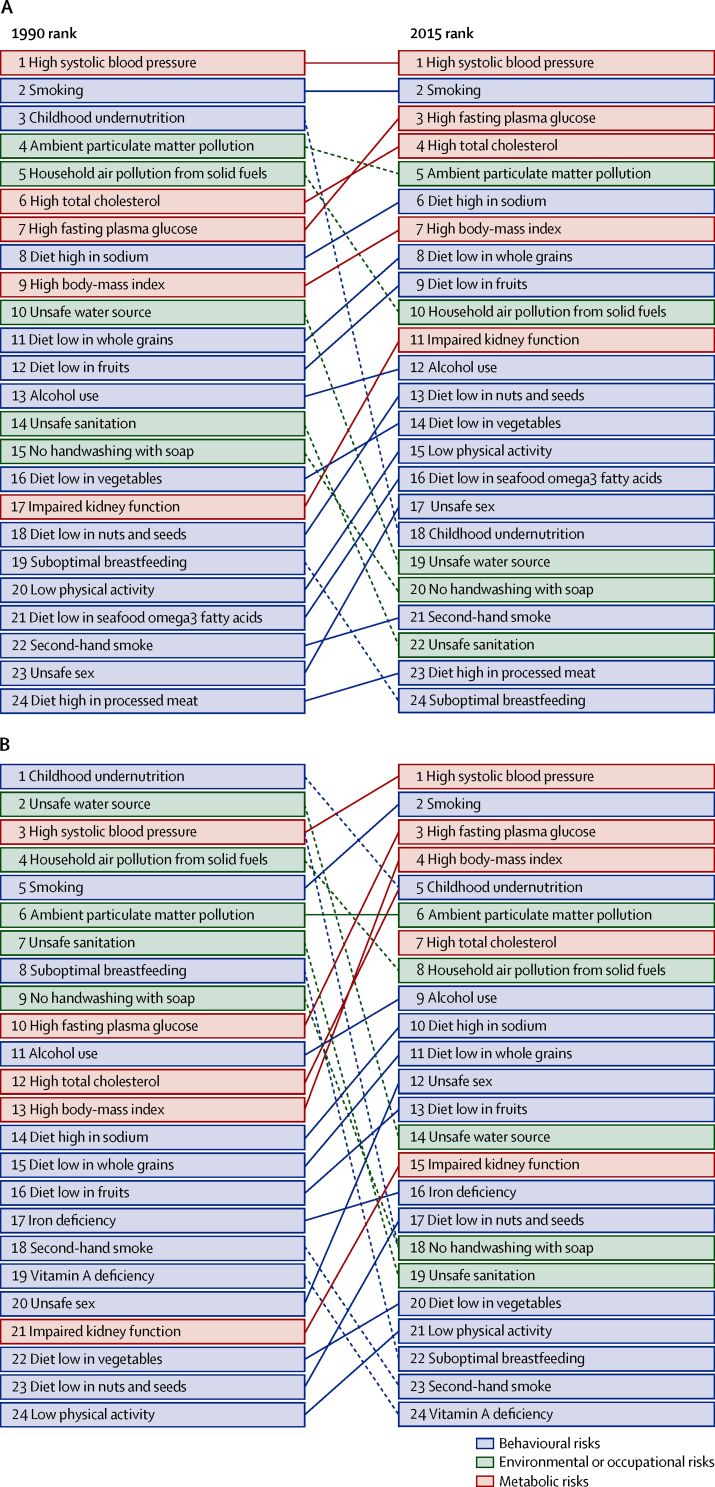
Leading level 3 Global Burden of Diseases global risk factors for deaths (A) and disability-adjusted life-years (B), 1990 and 2015 Risks are connected by lines between years; solid lines show risks that have stayed the same or moved higher in the ranking and dashed lines show risks that have moved lower.

Long-term exposure to PM_2·5_ contributed to 4·2 million (95% UI 3·7 million to 4·8 million) deaths and to a loss of 103·1 million (90·8 million to 115·1 million) DALYs in 2015, representing 7·6% of total global deaths and 4·2% of global DALYs, which is an increase from 1990. In 2015, ambient PM_2·5_ was the fifth-ranked risk factor for global deaths and sixth-ranked risk factor for DALYs among the risk factors included in GBD 2015 (figure 3). DALYs attributable to long-term exposure to PM_2·5_ consisted of 99·2 million (95% UI 87·7 million to 111·0 million) years of life lost and 3·9 million (2·6 million to 5·2 million) years lived with disability in 2015.

Household air pollution from solid fuel use was responsible for 2·8 million (95% UI 2·2 million to 3·6 million) deaths and 85·6 million (66·7 million to 106·1 million) DALYs in 2015. Together, ambient and household air pollution were estimated to have caused 6·4 million (5·7 million to 7·3 million) deaths in 2015.

**Figure 4:**
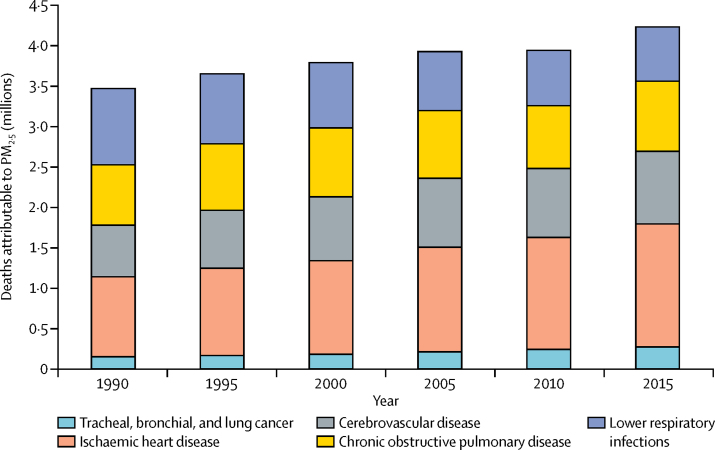
Deaths attributable to ambient particulate matter pollution by year and cause PM_2·5_=particle mass with aerodynamic diameter less than 2·5 μm.

Mortality from cardiovascular disease (IHD and cerebrovascular disease) accounted for most deaths and DALYs attributable to ambient PM_2·5_ air pollution (figure 4; table 1). Ambient PM_2·5_ air pollution contributed to 17·1% of IHD, 14·2% of cerebrovascular disease, 16·5% of lung cancer, 24·7% of LRI, and 27·1% of COPD mortality in 2015 according to GBD compare.^21^

**Table 1:** Global deaths, disability-adjusted life-years, and age-standardised rates attributable to ambient particulate matter pollution in 2015

		**Deaths, in thousands (95% UI)**	**Age-standardised deaths per 100 000 people (95% UI)**	**DALYs, in thousands (95% UI)**	**Age-standardised DALYs per 100 000 people (95% UI)**
All causes		4241·1 (3698·0–4776·7)	66·0 (57·2–74·8)	103 066·2 (90 829·6–115 072·6)	1490·9 (1312·4–1665·6)
Disease					
	Lower respiratory infection	675·0 (491·9–889·0)	10·1 (7·4–13·4)	28 359·9 (21 141·8–35 796·9)	390·9 (290·9–494·3)
	Lung cancer	283·3 (178·4–398·7)	4·4 (2·7–6·1)	6209·1 (3934·9–8689·3)	90·9 (57·5–127·3)
	Ischaemic heart disease	1521·1 (1231·7–1821·2)	23·6 (18·9–28·5)	32 406·0 (27 078·2–37 427·4)	470·7 (394·6–543·0)
	Cerebrovascular disease	898·1 (717·6–1083·6)	14·0 (11·0–17·1)	19 242·8 (16 095·9–22 679·7)	281·2 (234·4–331·4)
	Chronic obstructive pulmonary disease	863·6 (538·5–1212·8)	14·0 (8·7–19·6)	16 848·2 (10 517·4–23 590·0)	257·2 (160·3–360·6)
Sex					
	Male	2455·4 (2140·2–2752·9)	83·9 (72·5–94·7)	62 894·7 (55 545·7–70 098·2)	1888·8 (1659·4–2113·6)
	Female	1785·7 (1546·2–2049·2)	50·8 (44·0–58·4)	40 171·5 (35 205·5–45 382·8)	1127·4 (986·6–1275·4)
Age					
	Children <5 years	202·6 (152·7–254·6)	30·1 (22·7–37·8)	17 431·1 (13 139·7–21 906·3)	2585·9 (1949·1–3249·5)
	Elderly >70 years	2228·3 (1842·0–2653·9)	562·7 (465·1–670·8)	25 073·0 (20 775·2–29 511·1)	6302·2 (5226·3–7419·8)

DALY=disability-adjusted life-year. UI=uncertainty interval.

Age-standardised death and DALY rates due to exposure to PM_2·5_ were higher in males than females (table 1), as a result of higher all-cause mortality rates in males (1018·6 per 100 000 males *vs* 703·4 per 100 000 females^21^). They were also higher in elderly people (age >70 years) than in children (age <5 years; table 1), mainly because of age-related differences in mortality from non-communicable diseases (41·4 per 100 000 children aged 1–5 years *vs* 2914·4 per 100 000 adults aged 70–74 years^21^). Ambient PM_2·5_ contributed to 202 000 (95% UI 152 700–254 600) deaths and 17·4 million (13·1 million to 21·9 million) DALYs from LRI in children younger than 5 years.

**Figure 5:**
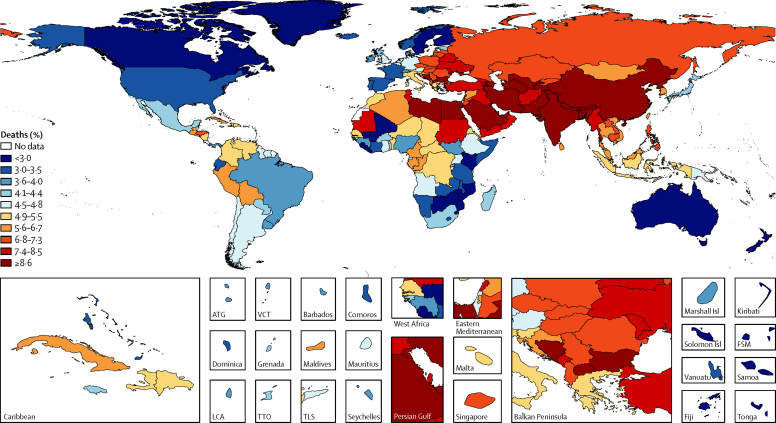
Deaths attributable to ambient particulate matter pollution in 2015 ATG=Antigua and Barbuda. FSM=Federated States of Micronesia. Isl=Island. LCA=Saint Lucia. TLS=Timor-Leste. TTO=Trinidad and Tobago. VCT=Saint Vincent and the Grenadines.

Deaths attributable to long-term exposure to PM_2·5_ in 2015 varied substantially among countries (figure 5). South and east Asia contributed 59% of the 4·2 million global deaths attributable to ambient PM_2·5_ in 2015 (1·36 million deaths [95% UI 1·19 million to 1·56 million] in south Asia and 1·14 million deaths [0·97 million to 1·31 million] in east Asia). In World Bank high-income countries, exposure to ambient PM_2·5_ contributed to 4·3% of total deaths in 2015 versus 9·0% in upper-middle-income, 8·7% in lower-middle-income, and 4·9% in low-income countries. These differences in attributable mortality mostly reflect the fraction of total deaths from cardiovascular disease among countries.^3^ The highest age-standardised rates of death due to PM_2·5_ exposure were in southern Asia (133·4 per 100 000 population, 95% UI 114·2–152·6), central Asia (85·2 per 100 000 population, 72·0–98·9), and eastern Asia (83·2 per 100 000 population, 70·4–95·6). Rates in high-income North American (USA, Canada, and Greenland; 17·8 per 100 000 people [95% UI 13·6–22·9]), Asian (18·7 per 100 000 people [14·6–23·7]), and western European countries (19·9 per 100 000 [15·9–24·8]) were four to eight times lower (appendix pp 26–1078).

Table 2 provides 2015 mortality and DALY estimates for the world's ten most populous countries in 2015. Ambient PM_2·5_ ranked among the top ten risk factors for mortality in each of the world's most populous countries. China and India combined had the largest numbers of attributable deaths and DALYs: 52% and 50% of the respective global totals. Pakistan, India, and Bangladesh had the highest age-adjusted mortality rates, more than seven times higher than those of Japan and the USA (table 2; appendix pp 26–1078).

**Table 2:** 2015 estimates of mortality and disability-adjusted life-years attributable to ambient particulate matter pollution and population-weighted mean particulate matter pollution in the world's ten most populous countries

	**Deaths, in thousands (95% UI)**	**Risk factor rank for deaths**	**Deaths per 100 000 people (95% UI)**	**DALYs, in thousands (95% UI)**	**DALYs per 100 000 people (95% UI)**	**Population-weighted mean PM**_2·5_**(μg/m^3^; 95% UI)**
China	1108·1 (948·7–1272·8)	1	84·3 (71·5–96·7)	21 778·7 (18 903·5–24 584·2)	1478·6 (1275·9–1675·6)	58·4 (58·1–58·7)
India	1090·4 (936·6–1254·8)	2	133·5 (112·8–154·9)	29 609·6 (25 923·3–33 562·7)	2922·1 (2527·3–3327·5)	74·3 (73·9–74·8)
USA	88·4 (66·8–115·0)	6	18·5 (14·2–23·7)	1485·9 (1166·3–1841·7)	337·1 (265·0–416·8)	8·4 (8·4–8·5)
Indonesia	78·6 (62·0–96·7)	7	49·9 (38·5–61·6)	2185·0 (1730·4–2716·2)	1081·1 (860·4–1324·2)	15·4 (15·1–15·7)
Brazil	52·3 (41·9–65·1)	9	30·9 (24·2–39·0)	1083·9 (884·0–1322·7)	573·7 (467·3–702·3)	11·4 (11·2–11·5)
Pakistan	135·1 (114·3–159·2)	4	136·3 (113·7–163·5)	4217·3 (3545·1–4916·3)	3114·2 (2651·3–3657·7)	65·0 (63·8–66·2)
Nigeria	50·9 (35·7–73·2)	10	68·9 (48·5–101·7)	2410·0 (1640·4–3387·0)	1581·0 (1107·6–2237·2)	38·0 (37·5–38·5)
Bangladesh	122·4 (103·2–144·4)	5	133·2 (111·8–158·4)	3408·0 (2920·3–3945·8)	2972·0 (2533·4–3469·1)	89·4 (87·3–91·7)
Russia	136·9 (111·3–161·1)	3	62·6 (51·8–73·2)	2601·6 (2194·8–3007·2)	1255·0 (1077·8–1431·1)	16·6 (16·2–17·0)
Japan	60·6 (44·5–81·4)	8	16·8 (12·8–21·9)	705·8 (561·2–891·0)	261·7 (212·8–319·2)	13·3 (13·1–13·6)

Countries are shown in order of population size in 2015. DALY=disability-adjusted life-year. PM_2·5_=particle mass with aerodynamic diameter less than 2·5 μm. UI=uncertainty interval.

Global mortality due to ambient PM_2·5_ increased from 1990 to 2015. Attributable deaths rose from 3·5 million (95% UI 3·0 million to 4·0 million) in 1990 to 3·8 million (3·3 million to 4·3 million) in 2000, and 4·2 million (3·7 million to 4·8 million) in 2015 (figure 4). However, age-standardised PM_2·5_ mortality rates decreased from 65·6 per 100 000 people (95% UI 56·9–74·9) in 1990 to 57·5 per 100 000 people (50·2–64·8) in 2015.

Trends in PM_2·5_-attributable mortality among countries largely reflect changes in PM_2·5_-attributable mortality from cardiovascular disease (appendix pp 24, 26–559). In World Bank high-income countries, the all-age proportion of PM_2·5_-attributable cardiovascular disease deaths decreased from 10·0% to 8·1% as a result of reductions in cardiovascular mortality and decreasing levels of PM_2·5_. By contrast, in World Bank low-income countries, it increased from 13·1% to 13·2%, and in lower-middle-income countries from 15·9% to 16·5%, between 1990 and 2015.

**Figure 6:**
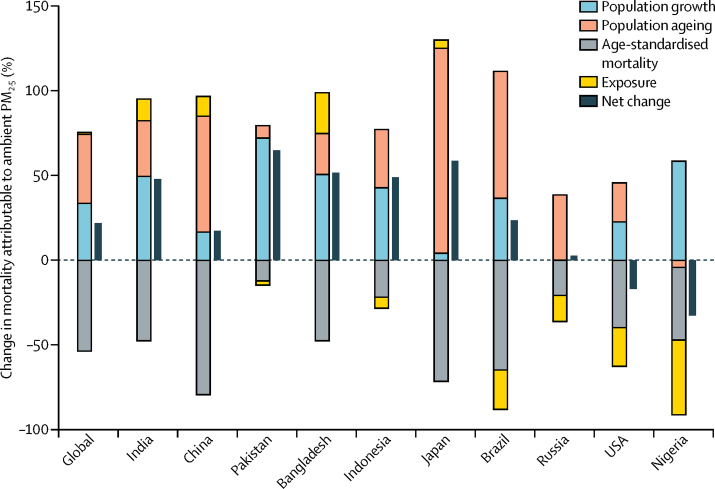
Changes in mortality attributable to ambient particulate matter pollution according to population-level determinants by country from 1990 to 2015 PM_2·5_=particle mass with aerodynamic diameter less than 2·5 μm.

Trends in PM_2·5_-attributable mortality at the global and national levels reflect the influence not only of changing air quality, but also of demography and underlying mortality rates. We calculated the contribution of changes in each of four factors—population growth, population ageing, age-standardised rates of mortality (IHD, cerebrovascular disease, COPD, lung cancer, and LRI), and exposure to ambient PM_2·5_—to the net change in mortality attributable to ambient PM_2·5_ between 1990 and 2015 globally and for the ten most populous countries (appendix pp 22–23). Figure 6 shows the changes in mortality attributable to ambient PM_2·5_ from 1990 to 2015 according to the contributions of these four factors. Age-standardised mortality decreased in all ten countries, with Nigeria, Russia, Brazil, Indonesia, Pakistan, and the USA also experiencing decreases in exposure. These decreases were offset by increases in population growth and population ageing in most countries. Consequently, net increases in attributable mortality were noted in all countries except Nigeria and the USA. In China, India, Bangladesh, and Japan, increases in exposure combined with increases in population growth and ageing resulted in net increases in attributable mortality. In Brazil, Russia, Indonesia, and Pakistan, despite decreasing exposure, population growth (except in the case of Russia) and the ageing of the population led to a net increase in attributable mortality. In the USA, reductions in exposure offset increases in population and ageing, leading to a net decrease in attributable burden.

GBD 2015^19^ estimated disease burden and mortality rates at the subnational level for China, the USA, and several other countries. When these data were combined with PM_2·5_ exposures estimated globally at fine (0·1 × 0·1°) resolution, we were able to estimate subnational burden attributable to PM_2·5_ exposure. In China, where ambient PM_2·5_ contributed to 1·1 million (95% UI 1·0 million to 1·8 million) deaths in 2015, the provincial-level PM_2·5_-attributable age-standardised rates varied by more than three times, from 132·1 deaths per 100 000 people (95% UI 97·6–172·0) in Qinghai to 40·6 deaths per 100 000 people (30·2–50·4) in Hong Kong. In the USA, where ambient PM_2·5_ contributed to 88 400 (95% UI 66 800–115 000) deaths in 2015, state-level PM_2·5_-attributable age-standardised death rates also varied by about three times, from 27·1 deaths per 100 000 (95% UI 21·2–34·1) in Mississippi to 8·1 deaths per 100 000 (5·1–11·7) in Hawaii.

**Figure 7:**
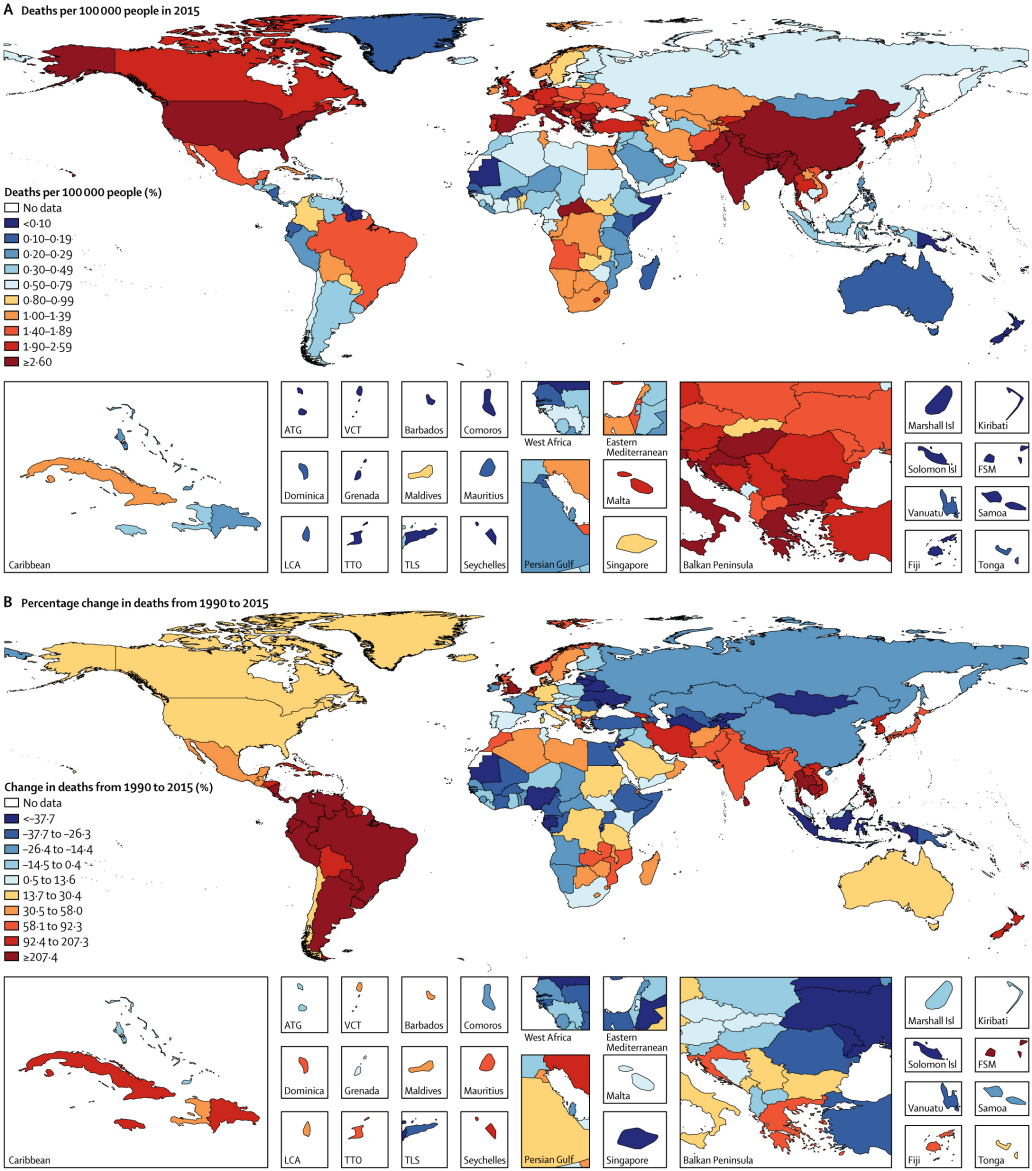
Proportion of deaths attributable to ozone (A) in 2015 and percentage change from 1990 (B) ATG=Antigua and Barbuda. FSM=Federated States of Micronesia. Isl=Island. LCA=Saint Lucia. TLS=Timor-Leste. TTO=Trinidad and Tobago. VCT=Saint Vincent and the Grenadines.

Exposure to ozone contributed to 254 000 (95% UI 97 000–422 000) deaths globally and a loss of 4·1 million (1·6 million to 6·8 million) DALYs from COPD in 2015. In 2015, ambient ozone was the 34th-ranked risk factor for global deaths and 42nd-ranked risk factor for DALYs among the 79 risk factors assessed in GBD 2015. Exposure to ozone contributed to an estimated 8·0% (95% UI 3·0–13.3) of global COPD mortality in 2015, with China, India, and the USA experiencing some of the highest mortality rates (figure 7A; appendix pp 1079–1362). The ozone-attributable COPD mortality rate increased in many countries from 1990 to 2015. Global deaths and DALYs attributable to ozone exposure increased from 1990 to 2015, as a result of increases in both levels of ozone and COPD mortality (figure 7B; appendix pp 1079–1362).

## Discussion

In this Article, we present, to our knowledge, the most comprehensive assessment so far of the global status and trends in the burden of disease attributable to ambient air pollution, highlighting the crucial part played by broad demographic and epidemiological trends. Estimation of spatially resolved trends over a 25-year period and assessment of the contributions of exposure and epidemiological and demographic factors to the attributable disease burden provides important insights for the development of policies to reduce the health effects of air pollution. These estimates are made in the context of assessment of burden attributable to other risk factors, allowing for direct comparisons and priority setting.

We estimated that long-term exposure to ambient PM_2·5_ caused 4·2 million deaths and 103·1 million lost years of healthy life in 2015, and exposure to ozone caused an additional 254 000 deaths. PM_2·5_ caused an estimated 7·6% of total global mortality in 2015 and was the fifth-ranking global mortality risk factor. Although global rates of mortality due to PM_2·5_ exposure decreased from 1990 to 2015 as a result of improved air quality in high-income countries and declining mortality rates for cardiovascular diseases, the absolute numbers of attributable deaths and DALYs increased as a result of increases in pollution and the absolute numbers of deaths from non-communicable diseases, especially in China and India, where populations are both growing and ageing. Household air pollution from the burning of solid fuels is also a major cause of mortality in low-income and middle-income countries, and together with ambient air pollution poses a substantial public health challenge.

Absolute numbers of attributable deaths and DALYs were higher in GBD 2015^3^ than estimated in GBD 2013.^4^ These differences are mainly a result of changes in the underlying disease burden estimates^3^ and to updates to the IER (appendix pp 8–14), which estimated higher relative risks in 2015 than in 2013 for IHD, cerebrovascular disease, LRI, COPD, and lung cancer. National-level population-weighted exposure estimates also increased between GBD 2013 and GBD 2015 (appendix pp 2–6). Because of the updated data and methods described earlier, we consider the current estimates to be more accurate.

Our results assume that the toxicity of ambient PM_2·5_ depends only on the magnitude of concentration, but not on the source, such as coal burning or vehicular emissions, or chemical composition, which vary among and within countries.^22^, ^23^ However, despite substantial effort, neither epidemiological nor toxicological research has identified particular sources or components that uniquely determine the toxicity of the PM_2·5_ mixture, and therefore the evidence does not support the development and application of source-specific relative risk functions for burden estimation.^15^, ^16^ This issue remains an active area of research and is a source of uncertainty in our estimates.

In the past few years, other researchers have estimated the burden of disease due to air pollution using different data and methods. Recent estimates from WHO^24^ of 3·0 million deaths in 2012 used the same exposure estimates as presented here, but an earlier (GBD 2013) version of the IER and somewhat different baseline disease burden estimates. Lelieveld and colleagues^25^ analysed source sector contributions to air pollution and the resulting disease burden in 2010 and estimated the burden in 2050. These estimates used an older (GBD 2010) IER. Furthermore, the coarse spatial resolution (∼100 × 100 km) of the exposure estimates introduced errors via spatial misalignment between exposure and population density compared with our estimates.

As in any assessment of this scope, this study has limitations. Since the GBD will be regularly updated, we anticipate enhancements to the methodology in the future to address them. First, we have probably underestimated the complete burden of disease attributable to air pollution. Although the causes of mortality we included make up four of the five leading global causes of death in 2015,^3^ findings from systematic reviews in the past 10 years have shown that PM_2·5_ exposure is also associated with low birthweight and preterm birth,^26^ asthma,^9^ and type 2 diabetes.^27^ Future updates of GBD estimates will consider these other causes of mortality and morbidity should they meet GBD inclusion criteria.

Second, our estimate of the importance of ambient PM_2·5_ assumes that exposure does not affect the prevalence of other mortality risk factors. However, if long-term exposure to PM_2·5_ causes high blood pressure, then some amount of the PM_2·5_ burden would be mediated by its effect on high blood pressure. Mediation analysis was used in GBD 2015 to more accurately apportion the burden attributable to other risk factors such as diet and high blood pressure, but an absence of longitudinal studies precludes such analyses for ambient PM_2·5_.^3^

Third, because large-scale cohort studies of PM_2·5_ and mortality are absent in the most polluted countries, the IERs were developed to estimate the effects of exposure at levels above those observed in air pollution cohort studies done in the USA, Canada, and western Europe, but the magnitude of the excess relative risk from PM_2·5_ exposure at high levels of PM_2·5_ remains uncertain. In Chinese cohort studies from the past 5 years, other metrics were used, such as total suspended particles and PM_10_,^28^, ^29^ and findings from a few analyses that converted these metrics to PM_2·5_ suggest that the IERs provide reasonable estimates of effects at high levels of ambient pollution.^12^, ^30^

Fourth, although we included estimates of the effect of seasonal ozone exposure on COPD mortality, less evidence is available for this relationship than that linking PM_2·5_ with COPD or the other causes of mortality. However, a causal link between increased COPD mortality and long-term exposure to ozone is, in our view, supported by a large body of evidence linking ozone exposure mortality to adverse effects on the respiratory system, including chronic changes in lung structure and function in human beings and non-human primates, and increased morbidity and mortality from COPD due to short-term and long-term exposure, especially in the warmer seasons.^10^, ^31^, ^32^

In conclusion, ambient air pollution contributes substantially to the global burden of disease, which has increased over the past 25 years, as a result of both demographic and epidemiological trends and increasing levels of air pollution in low-income and middle-income countries. Should these trends continue, major reductions in pollution levels will be needed to avoid increases in disease burden. Moreover, the non-linear IERs imply modest reductions in burden in the most polluted countries unless PM_2·5_ concentrations decline markedly.^3^, ^33^, ^34^ As a result, the challenges for future reductions in the burden of disease attributable to air pollution are substantial. For example, using earlier attributable burden estimates and future mortality predictions, Apte and colleagues^33^ estimated that air pollution levels in 2030 in China would need to decline by 29%, and those in India by 20%, to maintain per-person mortality at 2010 levels, although the economic^35^ and public health benefits of even incremental reductions would probably be substantial in view of the large populations affected.^34^ Exposure to ambient air pollution and its associated burden of disease can potentially be lowered for entire populations via policy action at the national and subnational levels. As the experience in the USA suggests,^36^ changes in ambient PM_2·5_ associated with aggressive air quality management programmes, focused on major sources^23^ of air pollution including coal combustion, household burning of solid fuels, and road transport, can lead to increased life expectancy over short timeframes.

**Contributors**

**Declaration of interests**
