# Department of Error

**DOI:** 10.1016/S0140-6736(17)31221-7

**Published:** 2017-06-17

*Cohen AJ, Brauer M, Burnett R, et al. Estimates and 25-year trends of the global burden of disease attributable to ambient air pollution: an analysis of data from the Global Burden of Diseases Study 2015.* Lancet *2016; **389:** 1907–18*—In this Article, the following changes have been made to the supplementary appendix. In table 1, for the Pinault et al^11^ study, the country, the CEV (Stroke)-Relative Risk (95% CI), and the IHD-Relative Risk (95% CI) have been corrected (p 11); the second Pinault et al^11^ reference has been changed to Thurston et al (2016; p 11); a footnote has been added stating that the indicated data were from “Additional, unpublished analyses, provided by the principal investigator at the authors' request” (p 12); the first Miller et al^15^ reference has been changed to Puett et al (2011); the LC-Relative Risk for Turner et al has been corrected (p 12); and the reference list has been updated. These corrections have been made to the online version as of May 23, 2017.

