# Department of Error

**DOI:** 10.1016/S0140-6736(18)30900-0

**Published:** 2018-04-21

*Cohen AJ, Brauer M, Burnett R, et al. Estimates and 25-year trends of the global burden of disease attributable to ambient air pollution: an analysis of data from the Global Burden of Diseases Study 2015.* Lancet *2017; **389:** 1907–18*—In this Article (published online first on April 10, 2017), the mathematical form for the IER has been corrected. This correction has been made to the online version as of April 19, 2018.

