# Supplementary material for: Estimates and 25-year trends of the global burden of disease attributable to ambient air pollution: an analysis of data from the Global Burden of Diseases Study 2015
Source: Lancet. 2017 May 13;389(10082):1907–18. doi: 10.1016/S0140-6736(17)30505-6 (PMC5439030; doi:10.1016/S0140-6736(17)30505-6)
Supplement: Supplementary appendix [file mmc1.pdf]

THE LANCET

Supplementary appendix

This appendix formed part of the original submission and has been peer reviewed. We post it as supplied by the authors.

Supplement to: Cohen AJ, Brauer M, Burnett R, et al. Estimates and 25-year trends of the global burden of disease attributable to ambient air pollution: an analysis of data from the Global Burden of Diseases Study 2015. *Lancet* 2017; published online April 10. http://dx.doi.org/10.1016/S0140-6736(17)30505-6.

Appendix to: The Global Burden of Disease attributable to ambient air pollution: Estimates of current burden and 25-year trends from the GBD 2015 study

Contents

Estimation of exposure…… 2

$PM_{2.5}$…… 2

Ozone…… 4

Theoretical minimum risk exposure level…… 7

Estimation of risk…… 8

Integrated exposure response parameter estimation…… 8

Relative risk estimate for ozone…… 14

STAN code for fitting IER…… 15

$PM_{2.5}$ attributable YLD estimation…… 18

Estimation of population attributable fraction (PAF) and burden…… 20

Decomposition of global trends in $PM_{2.5}$-attributable mortality…… 22

Web figures

Web Figure 1: Population-weighted PM2.5 estimates for 195 countries from GBD 2013 vs. GBD 2015…… 6

Web Figure 2: Percent change in population attributable fraction (PAF) for cardiovascular disease (IHD and stroke) attributable to PM2.5 in all countries from 1990 to 2015…… 24

Web tables

Web Table 1: Deaths attributable to ambient particulate matter pollution for all ages and age-standardized (rate per 100,000) for both sexes combined, by cause and 95% uncertainty intervals (UI)…… 26

Web Table 2: DALYs attributable to ambient particulate matter pollution for all ages and age-standardized (rate per 100,000) for both sexes combined, by cause and 95% uncertainty intervals (UI)…… 560

Web Table 3: DALYs attributable to ambient ozone pollution for all ages and age-standardized (rate per 100,000) for both sexes combined, by cause and 95% uncertainty intervals (UI)…… 1079

Web Table 4: Deaths attributable to ambient ozone pollution for all ages and age-standardized (rate per 100,000) for both sexes combined, by cause and 95% uncertainty intervals (UI)…… 1217

ESTIMATION OF EXPOSURE

The $PM_{2.5}$ and ozone exposure estimates used in GBD 2015 are described in detail elsewhere (GBD 2015 Risk Factor Collaborators 2016; Shaddick G. et al. Submitted).

$PM_{2.5}$ We estimated annual average exposure for five year intervals from 1990 – 2015 at 0.1 x 0.1 ° (~ 11 x 11 km at the equator) resolution throughout the world using a combination of satellite-derived estimates, surface measurements and geographic information. The satellite-based $PM_{2.5}$ estimates use multiple (MISR, MODIS Dark Target, MODIS and SeaWiFS Deep Blue, and MODIS MAIAC) satellite aerosol optical depth (AOD) retrievals to estimate near-surface $PM_{2.5}$ by applying the relationship of $PM_{2.5}$ to AOD simulated by the GEOS-Chem chemical transport model (Van Donkelaar et al. 2016). Estimates for 1995 and 1990 were based on the ratio of GEOS-Chem simulations using anthropogenic emissions between 2005 and the respective year of interest, but constant meteorology. Satellite-based estimates, and quantities estimated using GEOS-Chem were available for 1990, 1995, 2000, 2005, 2010, 2011, 2012, 2013 and 2014.

The GBD 2015 update incorporated more recent ground measurement data, and also including additional locations where measurement data have become available. These updates were made in collaboration with the World Health Organization (WHO) and included within the 2016 release of the WHO Global Urban Ambient Air Pollution Database. Monitor-specific measurements (rather than the city averages as reported in the WHO database) were used, resulting in measurements of annual average concentrations of PM_{10} and $PM_{2.5}$ from 6,003 ground monitors. The majority of measurements were recorded in 2014 (as there is a lag in reporting measurements, little data from 2015 were available). Where data were not available for 2014 (2760 monitors), data were used from 2015 (18 monitors), 2013 (2155), 2012 (564), 2011 (60), 2010 (375), 2009 (49), 2008 (21) and 2006 (1). For locations measuring only PM_{10}, $PM_{2.5}$ measurements were estimated from PM_{10}. This was performed using a locally derived conversion factor ($PM_{2.5}/PM_{10}$ ratio, for stations where measurements are available for the same year) that was estimated using population-weighted averages of location-specific conversion factors for the country. If country-level conversion factors were not available, the average of country-level conversion factors within a region were used. As in the GBD2013 database, additional information related to the ground measurements was also included where available (e.g. monitor geo coordinates, monitor site type).

Significant advances were incorporated in the methodology used to combine data from multiple sources since GBD 2013. In GBD 2010 and GBD 2013, exposure estimates were obtained using a single global function to calibrate available ground measurements to a 'fused' estimate of $PM_{2.5}$; the mean of satellite-based estimates and those from the TM5 chemical transport model, calculated for each $0.1^o \times 0.1^o$ grid cell. This was recognized to represent a trade-off between accuracy (see discussion in Brauer et al. 2016) and computationally efficiency when utilising available data sources.

In GBD 2015 we conducted formal model evaluation to compare the single global calibration function as in GBD 2013, a geographically-weighted regression (van Donkelaar et al. 2016), and a Bayesian Hierarchical Model (BHM), with the BHM ultimately used for analysis based on formal model evaluation results. The BHM framework allows for the estimation of country-specific coefficients in the calibration

model. Where data were insufficient within a country, information was `borrowed’ from a higher aggregation (region or super-region). Uncertainty is also be propagated through the model to comprise a posterior distribution for each grid cell, rather than just a single point estimate. Due to both the complexity of the models and the number of spatial predictions that were required, ‘approximate’ Bayesian inference based on integrated nested Laplace approximations (INLA) were used (R-INLA).

Model development and evaluation was performed using within- and out-of-sample assessment. In the evaluation, cross validation was performed using 25 combinations of training (80%) and validation (20%) datasets. Validation sets were obtained by taking a stratified random sample, using sampling probabilities based on the cross-tabulation of PM2.5 concentration measurement categories (0-24.9, 25-49.9, 50-74.9, 75-99.9, 100+ µg/m3) and super-regions, resulting in them having the same distribution of $PM_{2.5}$ concentrations and super-regions as the overall set of measurement sites. Modelling was performed on the log-scale with the unit of measurement being a grid cell. Variable selection was based on contribution to model fit (DIC, a measure of the relative quality of a model and closely related to that of AIC but for Bayesian models) and predictive ability.

Variables and model structures that were considered in model development included:

(SAT) Estimate of $PM_{2.5}$ (in $\mu g/m^3$) for 2014 from satellite remote sensing on the log-scale.

(CTM) Estimate of $PM_{2.5}$ (in $\mu g/m^3$) for 2014 from the TM5 chemical transport model on the log-scale.

(POP) Estimate of population for 2014 on the log-scale.

(SNAOC) Estimate of the sum of sulfate, nitrate, ammonium and organic carbon simulated using the GEOS Chem chemical transport model

(DST) Estimate of compositional concentrations of for mineral dust simulated using the GEOS Chem chemical transport model

(EDxDU) The log of the elevation difference between the elevation at the ground measurement location and the mean elevation within the GEOS Chem simulation grid cell multiplied by the inverse distance to the nearest urban land surface

(LOC) Binary variable indicting whether the exact location of ground measurement was known

(TYPE) Binary variable indicting whether exact type of ground monitoring site was known

(CONV) Binary variable indicting whether ground measurement was $PM_{2.5}$ or converted from PM_{10}

Random Effects were included for the intercept to allow for multiple ground monitors in a grid cell, country-region-super-region hierarchical random effects for the intercept, country-region-super-region hierarchical random effects for the coefficient associated with SAT, country-region-super-region hierarchical random effects for the coefficient associated with the difference between estimates from CTM and SAT, country-region-super-region hierarchical random effects for the coefficient associated

with POP, country level random effects for population using a neighbourhood structure allowing specific borrowing of information from neighbouring countries. Within a region, country level effects of SAT and the difference between SAT AND CTM were assumed to be independent and identically distributed. Within a super-region, region level random effects were assumed to be independent and identically distributed. Super-region random effects were assumed to be independent and identically distributed. Interactions between the binary variables and the effects of SAT and CTM were also included.

The final model contained the following variables: SAT, POP, SNAOC, DST, EDxDU, LOC, TYPE, and CONV, together with interactions between SAT and each of LOC, TYPE and CONV. The model structure contained grid cell random effects on the intercept to allow for multiple ground monitors in a grid cell, country-region-super-region hierarchical random effects for intercepts and SAT and country level random effects for population using a neighbourhood structure allowing specific borrowing of information from neighbouring countries together with region-super-region hierarchical random effects for POP. Compared to the model used in GBD 2013, this model showed improved predictions of ground measurements in all super regions and an improvement in both within-sample fit; with an increase in R^2 from 0.64 (reported in GBD 2013) to 0.91, and out-of-sample predictive ability; with a global population-weighted RMSE of 12.1 µg/m3 compared to 23.1 $\mu g/m^3$ when using the GBD 2013 model. Including estimates from the TM5 chemical transport model used in GBD 2013 did not improve the predictive ability of the model and was therefore not included. Because the TM5 simulations used in GBD 2013 included a constant level of contribution from sea salt and windblown mineral dust, these prior estimates obscured temporal trends in dust emissions. This difference, along with a lag in incorporating changes in levels of emissions into the emissions inventories which are inputs to CTM simulations, has led to changes in both the magnitude of exposure and slopes of trends in some countries.

As satellite estimates, populations and quantities estimated using the GEOS-Chem model were only available up to 2014, estimates for 2015, (and associated measures of uncertainty) were obtained by fitting smoothing splines within each cell to the medians and limits of the 95% credible intervals for each cell from 2010 to 2014 and extrapolating to 2015. The smoothing splines were fit within a Generalised Additive Modelling framework with the degrees of freedom calculated for each cell by multivariate generalized cross validation.

Ozone As in GBD2013 (Brauer et al. 2016) we used TM5-FASST TM5-FASST (Van Dingenen et al. 2014) (FAst Scenario Screening Tool, a reduced-form version of TM5) simulations for 1990, 2000 and 2010, including an updated set of internally consistent emissions inventories and “typical” (year 2001) meteorology, to simulate a running 3-month average (of daily 1 h max values) ozone concentrations for each grid cell over a full year from which we selected the maximum of these values. This metric was chosen to align with epidemiologic studies of chronic exposure which typically employ a seasonal (summer) average, and to account for global variation in the timing of the ozone (summer) season. These estimates were simulated with TM5-FASST at 0.1° x 0.1° for 1990, 2000 and 2010 using the same emissions and meteorological inputs as for the $PM_{2.5}$ simulations. Estimates for 2015 and 2005 were generated by fitting a natural cubic spline to the 1990, 2000 and 2010 estimates and then interpolating (in the case of 2005) and extrapolating (for 2015) from the fitted values.

References

Brauer et al. Ambient Air Pollution Exposure Estimation for the Global Burden of Disease 2013. Environ Sci Technol. 2016 Jan 5;50(1):79-88. doi: 10.1021/acs.est.5b03709.

GBD 2015 Risk Factor Collaborators. 2016. Appendix.

Shaddick G, Thomas ML, Jobling A, Brauer M, van Donkelaar A, Burnett R, Chang HH, Cohen A, Van Dingenen R, Dora C, Gumy S, Liu Y, Martin RV, Waller LA, West J, Zidek JV, Prüss-Ustün A. 2016. Data Integration Model for Air Quality: A Hierarchical Approach to the Global Estimation of Exposures to Ambient Air Pollution. Submitted.

Van Dingenen, R.; Leitao, J.; Dentener, F. A multi-metric global source-receptor model for integrated impact assessment of climate and air quality policy scenarios. EGU Gen. Assem. 2014.

van Donkelaar A, Martin RV, Brauer M, Hsu NC, Kahn RA, Levy RC, Lyapustin A, Sayer AM, Winker DM. Global Estimates of Fine Particulate Matter using a Combined Geophysical-Statistical Method with Information from Satellites, Models, and Monitors. Environ Sci Technol. 2016 Apr 5;50(7):3762-72. doi: 10.1021/acs.est.5b05833

World Health Organization. WHO Global Urban Ambient Air Pollution Database (update 2016). http://www.who.int/entity/phe/health_topics/outdoorair/databases/WHO_AAP_database_May2016_v3web.xlsx?ua=1 (accessed August 25, 2016)

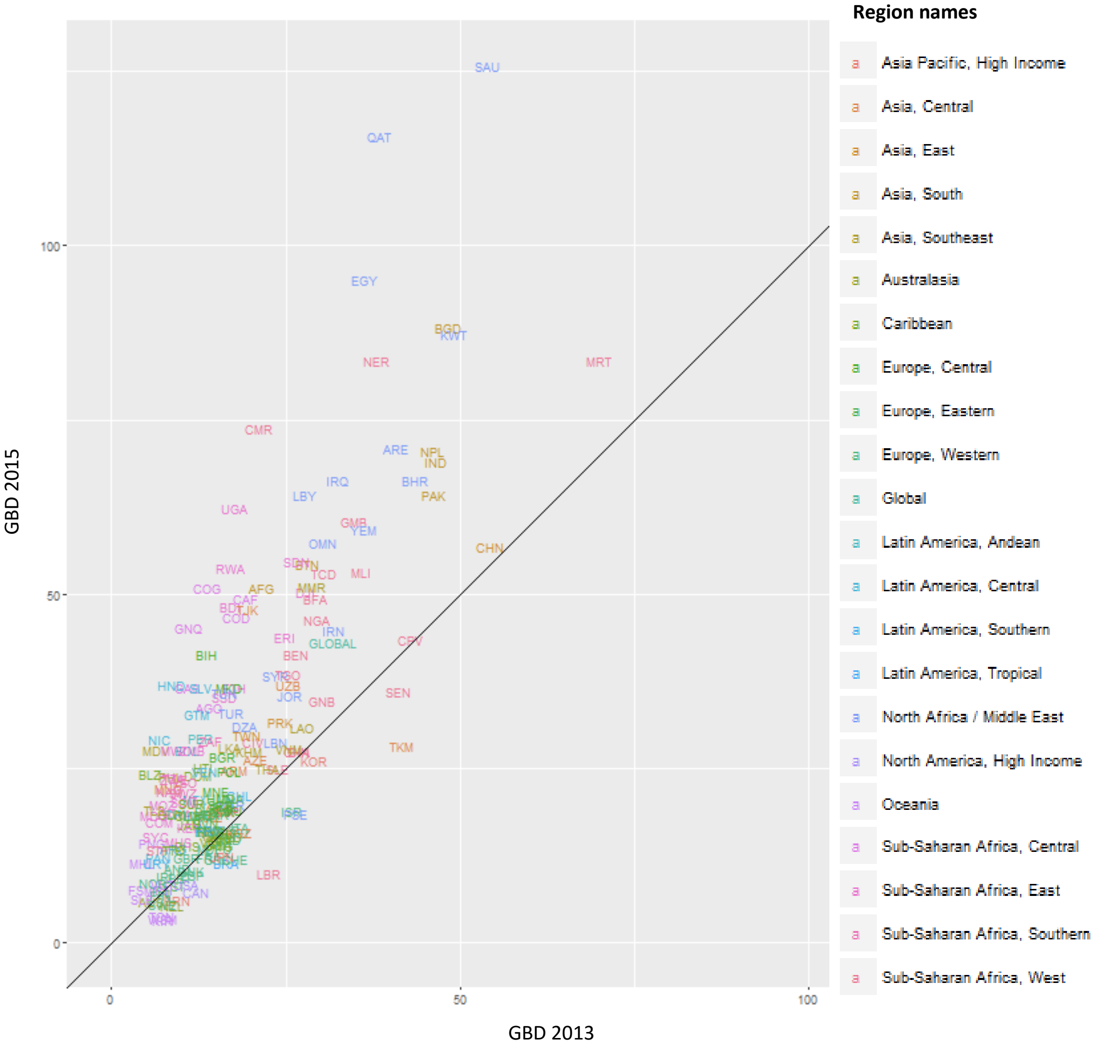

Web Figure 1. Population-weighted $PM_{2.5}$ estimates for 195 countries from GBD 2013 vs. GBD 2015.

Theoretical Minimum Risk Exposure Level

The TMREL was assigned a uniform distribution with lower/upper bounds given by the average of the minimum and 5th percentiles of outdoor air pollution cohort studies exposure distributions conducted in North America (Supplement Appendix Table 1 – selected studies indicated in red), with the assumption that current evidence was insufficient to precisely characterize the shape of the concentration-response function below the 5th percentile of the exposure distributions. The TMREL was defined as a uniform distribution rather than a fixed value in order to represent the uncertainty regarding the level at which the scientific evidence was consistent with adverse effects of exposure (Lim et al. 2012 Table 1; GBD 2015 Risk Factors Collaborators 2016).

The specific outdoor air pollution cohort studies selected for this averaging were based on the criteria that their 5th percentiles were less than that of the American Cancer Society Cancer Prevention II (CPS II) cohort's 5th percentile of 8.2 $\mu g / m^3$ based on Turner et al. (2016). This criteria was selected since GBD2010 (Lim et al., 2012) used the minimum, 5.8 $\mu g / m^3$, and 5th percentile solely from the CPS II cohort. The resulting lower/upper bounds of the distribution for GBD 2015 were 2.4 $\mu g / m^3$ and 5.9 $\mu g / m^3$.

The TMREL for ozone was defined similarly using a uniform distribution 33.3–41.9 parts per billion (Lim et al. 2012 Table 1).

References

Lim SS, Vos T, Flaxman AD, et al. 2012. A comparative risk assessment of burden of disease and injury attributable to 67 risk factors and risk factor clusters in 21 regions, 1990–2010: a systematic analysis for the Global Burden of Disease Study 2010. Lancet 380:2224-2260.

GBD 2015 Risk Factors Collaborators. Global, regional, and national comparative risk assessment of 79 behavioural, environmental and occupational, and metabolic risks or clusters of risks, 1990–2015: a systematic analysis for the Global Burden of Disease Study 2015. *Lance t*2016; **388:** 1659–724.

Turner MC, Jerrett M, Pope III CA, Krewski D, Gapstur SM, Diver WR, Beckerman BS, Marshall JD, Su J, Crouse DL, Burnett RT. (2015). Long-Term Ozone Exposure and Mortality in a Large Prospective Study. Am J Respir Crit Care Med Vol 193, Iss 10, pp 1134–1142, May 15, 2016

ESTIMATION OF RISK

We estimated the Ambient Air Pollution-attributable burden of disease based on the relation of long-term exposure to $PM_{2.5}$ with Ischemic Heart Disease, stroke (ischemic and hemorrhagic), COPD, lung cancer and acute lower respiratory infection and on the relation of long-term exposure to ozone with COPD. These were also the pollutant-outcome pairs used to estimate the Ambient Air Pollution attributable burden in GBD 2010 and 2013. For GBD 2015, we used results from all cohort studies conducted on subjects living in the US and Western Europe (Appendix Table 1). These studies comprised all studies published as of mid-2016 that reported cause-specific relative risk estimates based on measured or modeled $PM_{2.5}$ and that adjusted for potential confounding due to other major risk factors such as tobacco smoking using data for each study participant. One exception was (Crouse et al. 2012) that adjusted indirectly for potential confounding effects of smoking and obesity (Appendix Table 1).

For GBD 2013 we added health loss from LRI in adults to the five outcomes previously included in GBD 2010. This decision was taken based on the association between lower respiratory infection and exposure to indoor and ambient particulate air pollution in young children and the results of time-series studies reporting increased morbidity and mortality in adults associated with short-term exposure to ambient particulate air pollution. There is also evidence of adverse effects of exposure to air pollution on host defences and immune function with respect to respiratory infection in adults. The relative risk of LRI in adults was estimated by adding relative risk estimates for active smoking from the study by Doll et al. (1994) to the LRI IER, and this IER was used to estimate the LRI burden attributable to $PM_{2.5}$ in both children and adults. For GBD 2015 we added studies on active smoking risk from Bjartveit et al. 2005, Teo et al. 2006, and Pirie et al. 2013.

Integrated Exposure Response Parameter Estimation

The unknown IER model parameters were estimated in GBD 2010 by first simulating 1000 draws of the input relative risks based on the assumption that their logarithms followed a normal distribution with mean given by the logarithm of the observed relative risk and standard deviation by the corresponding observed standard error. The IER model was fit separately to the 1000 sets of simulated relative risk data by non-linear least squares. This approach was described in detail in Burnett et al. (2014).

A limitation of this method is that the non-linear least squares estimation routine did not converge for all 1000 sets of simulated input relative risk data, raising concerns that only certain patterns in the simulated data would be kept such that the estimation routine would converge. This approach also resulted in a wide range of predicted risk for any given concentration across the 1000 simulated values.

To address these limitations, in GBD 2013, we selected an alternative method to estimate the unknown IER parameters and their uncertainty that also incorporated the uncertainty in the TMREL. The observed relative risks were related to the IER within a Bayesian framework using the computer software package STAN (Stan, 2015). Given the true values of the four parameters $(\alpha, \beta, \gamma, z_{cf})$ we assumed that the logarithm of the each study's observed relative risk was normally distributed with mean defined by the

IER function, and variance given by the square of the observed standard error of the study-specific log-relative risk estimate. The parameters (α, β, γ) were each assumed to have a non-informative gamma distribution independent of each other, each with mean 100 and variance ten thousand. The STAN code used to fit the IER model is given below. However, in order to achieve convergence of the MCMC routine we needed to divide the exposure concentration by 10 billion which resulted in unrealistically narrow confidence intervals. To address this issue for GBD 2015, in addition to adding several new relative risk estimates we expanded the error specification for each logarithm of the observed relative risk to include the sum of the observed sampling error and an additional error parameter that was allowed to vary by source of $PM_{2.5}$ (outdoor air, second hand smoke, indoor air, and active smoking). Under these new conditions convergence was achieved without the need to divide concentration by 10 billion. As with GBD 2013, we assume a non-informative gamma prior on the IER parameters with mean 100 and standard deviation of 100.

The specification of the relative risks used as data input to the IER parameter fitting process was also changed from GBD 2013 to GBD 2015. In GBD 2010 and GBD 2013, we constructed a relative risk for outdoor air pollution studies based on the unit risk evaluated at an exposure contrast of the study mean to the TMREL. In GBD 2015 we altered the contrast to represent the 95th to 5th percentiles of the exposure distribution as for some studies their 5th percentiles was much larger than the TMREL. We therefore were not extrapolating evidence from any study beyond their observed exposure range.

References

Bjartveit K, Tverdal A. Health consequences of smoking 1-4 cigarettes per day. *Tob Control*. 2005; 14(5): 315–20.

Brauer et al. Ambient Air Pollution Exposure Estimation for the Global Burden of Disease 2013. Environmental Science and Technology. 2015. doi: 10.1021/acs.est.5b03709

Burnett RT, Pope CA III, Ezzati M, Olives C, Lim SS, Mehta S, Shin HH, Singh G, Hubbell B, Brauer M, Anderson HR, Smith KR, Balmes J, Bruce N, Kan H, Laden F, Prüss-Ustün A, Turner MC, Gapstur SM, Diver WR, Cohen A. 2014. An Integrated Risk Function for Estimating the Global Burden of Disease Attributable to Ambient Fine Particulate Matter Exposure. Environmental Health Perspectives http://dx.doi.org/10.1289/ehp.1307049

Crouse, D.L., Peters, P.A., van Donkelaar, A., Goldberg, M.S., Villeneuve, P.J., Brion, O., Khan, S., Atari, D.O., Jerrett, M., Pope, C.A., Brauer, M., Brook, J.R., Martin, R.V., Stieb, D., Burnett, R.T.Risk of nonaccidental and cardiovascular mortality in relation to long-term exposure to low concentrations of fine particulate matter: a Canadian national-level cohort study. Environ Health Perspect, 2012; 120:708-714.

Doll R, Peto R, Wheatley K, Gray R, Sutherland I. 1994. Mortality in relation to smoking: 40 years' observations on male British doctors. British Medical Journal. 309 (8): 901-11.

Lim SS, Vos T, Flaxman AD, et al. 2012. A comparative risk assessment of burden of disease and injury attributable to 67 risk factors and risk factor clusters in 21 regions, 1990–2010: a systematic analysis for the Global Burden of Disease Study 2010. Lancet 380:2224-2260.

Pirie K, Peto R, Reeves GK, Green J, Beral V, Million Women Study Collaborators. The 21st century hazards of smoking and benefits of stopping: a prospective study of one million women in the UK. *Lancet*. 2013; 381(9861): 133–41.

Stan Development Team. 2015. Stan Modeling Language Users Guide and Reference Manual, Version 2.8.0.

Teo KK, Ounpuu S, Hawken S, Pandey MR, Valentin V, Hunt D, Diaz R, Rashed W, Freeman R, Jiang L, Zhang X, Yusuf S, INTERHEART Study Investigators. Tobacco use and risk of myocardial infarction in 52 countries in the INTERHEART study: a case-control study. *Lancet*. 2006; 368(9536): 647–58.

Appendix Table 1: Descriptive statistics (mean, minimum, 5th percentile) for $PM_{2.5}$ exposure, hazard ratio for a 10 μg/m^3 change, and number of cause-specific deaths by study (N represents the number of cause specific deaths).

Study	Location	Number of Study Subjects	Mean Annual Average $PM_{2.5}$	5th/95th Percentile of Annual Average $PM_{2.5}$	$PM_{2.5}$ Increment	Outcome	LRI-Relative Risk (95% CI); number of deaths or incident cases	CEV (Stroke)-Relative Risk (95% CI); number of deaths	COPD-Relative Risk (95% CI); number of deaths or incident cases	IHD-Relative Risk (95% CI); number of deaths or incident cases	LC- Relative Risk (95% CI); number of deaths or incident cases
Brauer et al.[1]	Netherlands	4,000	16.9	14/19.1	3.2	Incidence	1.04 (0.85-1.26); N = 2,986	NA	NA	NA	NA
Crouse et al.[2]	Canada	2,521,525	8.9	3.6/15.1	5	Mortality	NA	0.969 (0.948-0.991); N = 19,725	1.008 (0.981-1.036); N = 14,170	1.099 (1.084-1.113); N = 63,050	1.064 (1.04-1.088); N = 30,545
Carey et al.[3]	UK	835,607	12.9	10.6/14.9	10	Mortality	NA	1 (0.81-1.29); N = 5,458	1.43 (1-1.79); N = 4,105	1.05 (0.81-1.29); N = 8,168	1.11 (0.88-1.43); N = 5,244
Hystad et al.[4]	Canada	2,390 cases 3,507 controls	11.9	6.7/17.1	10	Mortality	NA	NA	NA	NA	1.29 (0.95-1.76); N = 2,154
Katanoda et al.[5]	Japan	63,520	25	16.8/34	10	Mortality	NA	NA	0.89 (0.7-1.12); N = 64	NA	1.24 (1.14-1.37); N = 518
Gan et al.[6]	Canada	465,360	4.1	NA/NA	1.68	Mortality	NA	NA	1 (0.92-1.09); N = 541	NA	NA
Cesaroni et al.[7]	Italy	1,265,058	23	NA/NA	10	Mortality	NA	1.08 (1.04-1.13); N = 13,576	NA	1.1 (1.06-1.13); N = 22,562	1.05 (1.01-1.1); N = 12,208
Hertz-Picciotto et al.[8]	Czech Republic	26,214	22.3	NA/NA	25	Incidence	1.3 (1.08-1.58); N = 1,429	NA	NA	NA	NA
Karr, Lumley et al.[9]	United States	56,166	23	10.8/36	10	Incidence	1.09 (1.04-1.14); N = 5,350	NA	NA	NA	NA
Karr, Rudra, et al.[10]	United States	27,340	12.2	NA/NA	10	Incidence	1.04 (0.83-1.29); N = 3,124	NA	NA	NA	NA
Pinault et al.[11]	US	299,500	6.3	3/11.3	10	Mortality	NA	1.28 (0.98-1.57); N = 1,500	1.4 (1.09-1.8); N = 1,400	1.29 (1.3-1.48); N = 4,700	1.17 (0.98-1.4); N = 2,700
Pinault et al.[11]	US	517,041	13.6	8.8/20.1	10	Mortality	NA	1.08 (0.97-1.21); N = 3,793	1.06 (0.97-1.16); N = 5,330	1.14 (1.08-1.21); N = 14,815	0.96 (0.89-1.03); N = 2,913
Naess et al.[12]	Norway	19,072	NA	11.46/18.43	6.97	Mortality	NA	NA	1.1 (1-1.21); N = 503	NA	1.07 (0.97-1.18); N = 424
Naess et al.[12]	Norway	37,797	NA	11.46/18.43	6.97	Mortality	NA	NA	1.27 (1.11-1.47); N = 223	NA	1.07 (0.97-1.18); N = 449
Naess et al.[12]	Norway	38,014	NA	11.46/18.43	6.97	Mortality	NA	NA	1.05 (0.96-1.16); N = 516	NA	1.16 (1.02-1.32); N = 285
Naess et al.[12]	Norway	44,094	NA	11.46/18.43	6.97	Mortality	NA	NA	1.09 (0.94-1.25); N = 203	NA	1.27 (1.13-1.43); N = 295
Puett et al.[13]	US	66,250	13.9	10/17.8	10	Mortality	NA	NA	NA	2.02 (1.07-3.78); N = 379	NA

Lipsett et al.[14]	US	73,498	15.6	8.3/23	10	Mortality	NA	1.16 (0.92-1.46); N = 382	1.21 (0.88-1.68); N = 196	1.2 (1.02-1.41); N = 773	0.95 (0.7-1.28); N = 234
Miller et al.[15]	United States	17,454	17.9	12.3/22.4	10	Mortality	NA	NA	NA	0.98 (0.71-1.36); N = 746	NA
Miller et al.[15]	United States	65,893	13.5	7.4/19.6	10	Mortality	NA	1.83 (1.11-3); N = 122	NA	2.21 (1.17-4.16); N = 80	NA
Villeneuve et al.[16]	Canada	89,292	9.5	4.8/14.7	10	Mortality	NA	1.31 (0.97-1.78); N = 434	NA	1.34 (1.09-1.66); N = 903	NA
Weichenthal et al.[17]	United States	83,610	9.2	7.3/15	10	Mortality	NA	1.78 (0.72-4.42); N =242	NA	2.68 (1.04-6.87); N = 178	0.75 (0.34-1.65); N =337
Beelen et al.[18]	Nether-lands	120,852	28.3	24.8/31.8	10	Mortality	NA	1.62 (1.07-2.44); N = 1,175	NA	0.96 (0.75-1.22); N = 3,521	1.06 (0.82-1.38); N = 1,670
Chen et al.[19]	US	3,239	29	15/45.1	10	Mortality	NA	NA	NA	1 (0.87-1.15); N = 145	NA
Lepeule et al.[20]	US	8,096	17.8	10.2/23.6	10	Mortality	NA	0.89 (0.67-1.18); N = 317	1.17 (0.85-1.62); N = 247	1.33 (1.16-1.52); N = 1,065	1.37 (1.07-1.75); N = 351
Turner et al.[21]	US	669,046	12.6	8.2/20	10	Mortality	NA	1.11 (1.05-1.17); N = 17,085	1.1 (1.01-1.19); N = 9,967	1.14 (1.1-1.18); N = 45,644	1.03-1.16); N = 16,432
Atkinson et al.[22]	United Kingdom	807,401	12.9	NA/NA	10	Incidence	NA	NA	1.31 (0.92-1.86); N = 2,910	NA	NA
Raaschou-Nielsen et al.[23]	ESCAPE – DCH	37,447	11.3	NA/NA	5	Incidence	NA	NA	NA	NA	0.91 (0.52-1.6); N = 638
Raaschou-Nielsen et al.[23]	ESCAPE - EPIC-Athens	4,096	20.4	NA/NA	5	Incidence	NA	NA	NA	NA	0.9 (0.34-2.4); N = 18
Raaschou-Nielsen et al.[23]	ESCAPE - EPIC-MORGEN	15,993	16.9	NA/NA	5	Incidence	NA	NA	NA	NA	0.49 (0.08-3.21); N = 92
Raaschou-Nielsen et al.[23]	ESCAPE - EPIC-Oxford	36,832	9.8	NA/NA	5	Incidence	NA	NA	NA	NA	0.53 (0.15-1.91); N = 78
Raaschou-Nielsen et al.[23]	ESCAPE - EPIC-PROSPECT	14,630	16.8	NA/NA	5	Incidence	NA	NA	NA	NA	1.09 (0.17-6.99); N = 112
Raaschou-Nielsen et al.[23]	ESCAPE - EPIC-Turin	7,216	30.1	NA/NA	5	Incidence	NA	NA	NA	NA	1.6 (0.67-3.81); N = 48
Raaschou-Nielsen et al.[23]	ESCAPE – HUBRO	17,640	8.9	NA/NA	5	Incidence	NA	NA	NA	NA	0.83 (0.35-2); N = 75
Raaschou-Nielsen et al.[23]	ESCAPE - SALT	4,732	7.3	NA/NA	5	Incidence	NA	NA	NA	NA	1.24 (0.23-6.76); N = 29
Raaschou-Nielsen et al.[23]	ESCAPE - SDPP	7,116	6.6	NA/NA	5	Incidence	NA	NA	NA	NA	2.01 (0.4-10.01); N = 35
Raaschou-Nielsen et al.[23]	ESCAPE - SIDRIA-Rome	9,105	19.4	NA/NA	5	Incidence	NA	NA	NA	NA	1.33 (0.69-2.58); N = 53
Raaschou-Nielsen et al.[23]	ESCAPE - SIDRIA-Turin	4,816	31	NA/NA	5	Incidence	NA	NA	NA	NA	1.94 (0.54-7); N = 19
Raaschou-	ESCAPE -	3,813	8.3	NA/NA	5	Incidence	NA	NA	NA	NA	1.56 (0.41-

Nielsen et al.[23]	Sixty										5.98); N = 38
Raaschou-Nielsen et al.[23]	ESCAPE - SNAC-K	2,384	8	NA/NA	5	Incidence	NA	NA	NA	NA	0.73 (0.12-4.37); N = 18
Raaschou-Nielsen et al.[23]	ESCAPE - VHM&PP	108,018	13.6	NA/NA	5	Incidence	NA	NA	NA	NA	1.32 (0.97-1.81); N = 678
Gan et al.[24]	Van-couver	465,360	4.1	NA/NA	5	Incidence	NA	NA	1.02 (0.98-1.07); N = 2,299	NA	NA

1. Brauer M, Hoek G, Van Vliet P, Meliefste K, Fischer PH, Wijga A, Koopman LP, Neijens HJ, Gerritsen J, Kerkhof M, Heinrich J, Bellander T, Brunekreef B. Air pollution from traffic and the development of respiratory infections and asthmatic and allergic symptoms in children. *Am J Respir Crit Care Med.* 2002; 166(8): 1092-8.
2. Crouse DL, Peters PA, van Donkelaar A, Goldberg MS, Villeneuve PJ, Brion O, Khan S, Atari DO, Jerrett M, Pope CA, Brauer M, Brook JR, Martin RV, Stieb D, Burnett RT. Risk of nonaccidental and cardiovascular mortality in relation to long-term exposure to low concentrations of fine particulate matter: a Canadian national-level cohort study. *Environ Health Perspect*. 2012; 120(5): 708-14.
3. Carey IM, Atkinson RW, Kent AJ, van Staa T, Cook DG, Anderson HR. Mortality associations with long-term exposure to outdoor air pollution in a national English cohort. *Am J Respir Crit Care Med*. 2013; 187(11): 1226-33.
4. Hystad P, Demers PA, Johnson KC, Carpiano RM, Brauer M. Long-term residential exposure to air pollution and lung cancer risk. Epidemiology. 2013; 24(5): 762-72.
5. Katanoda K, Sobue T, Satoh H, Tajima K, Suzuki T, Nakatsuka H, Takezaki T, Nakayama T, Nitta H, Tanabe K, Tominaga S. An association between long-term exposure to ambient air pollution and mortality from lung cancer and respiratory diseases in Japan. <i>J Epidemiol</i>. 2011; 21(2): 132-43.
6. Gan WQ, FitzGerald JM, Carlsten C, Sadatsafavi M, Brauer M. Associations of ambient air pollution with chronic obstructive pulmonary disease hospitalization and mortality. *Am J Respir Crit Care Med*. 2013; 187(7): 721-7.
7. Cesaroni G, Badaloni C, Gariazzo C, Stafoggia M, Sozzi R, Davoli M, Forastiere F. Long-term exposure to urban air pollution and mortality in a cohort of more than a million adults in Rome. *Environ Health Perspect*. 2013; 121(3): 324-31.
8. Hertz-Picciotto I, Baker RJ, Yap P-S, Dostál M, Joad JP, Lipsett M, Greenfield T, Herr CEW, Benes I, Shumway RH, Pinkerton KE, Srám R. Early childhood lower respiratory illness and air pollution. Environ Health Perspect. 2007; 115(10): 1510-8.
9. Karr C, Lumley T, Schreuder A, Davis R, Larson T, Ritz B, Kaufman J. Effects of subchronic and chronic exposure to ambient air pollutants on infant bronchiolitis. *Am J Epidemiol.* 2007; 165(5): 553-60.
10. Karr CJ, Rudra CB, Miller KA, Gould TR, Larson T, Sathyanarayana S, Koenig JQ. Infant exposure to fine particulate matter and traffic and risk of hospitalization for RSV bronchiolitis in a region with lower ambient air pollution. *Environ Res.* 2009; 109(3): 321-7.
11. Pinault L, Tjepkema M, Crouse DL, Weichenthal S, van Donkelaar A, Martin RV, Brauer M, Chen H, Burnett RT. Risk estimates of mortality attributed to low concentrations of ambient fine particulate matter in the Canadian community health survey cohort. *Environ Health*. 2016; 15(1): 18.
12. Naess Ã˜, Nafstad P, Aamodt G, Claussen B, Rosland P. Relation between concentration of air pollution and cause-specific mortality: four-year exposures to nitrogen dioxide and particulate matter pollutants in 470 neighborhoods in Oslo, Norway. *Am J Epidemiol.* 2007; 165(4): 435-43.
13. Puett RC, Hart JE, Yanosky JD, Paciorek C, Schwartz J, Suh H, Speizer FE, Laden F. Chronic fine and coarse particulate exposure, mortality, and coronary heart disease in the Nurses' Health Study [Unpublished data]. *Environ Health Perspect*. 2009; 117(11): 1697-701.
14. Lipsett MJ, Ostro BD, Reynolds P, Goldberg D, Hertz A, Jerrett M, Smith DF, Garcia C, Chang ET, Bernstein L. Long-term exposure to air pollution and cardiorespiratory disease in the California teachers study cohort [Unpublished data]. *Am J Respir Crit Care Med*. 2011; 184(7): 828-35.
15. Miller KA, Siscovick DS, Sheppard L, Shepherd K, Sullivan JH, Anderson GL, Kaufman JD. Long-term exposure to air pollution and incidence of cardiovascular events in women [Unpublished data]. *N Engl J Med*. 2007; 356(5): 447-58.
16. Villeneuve PJ, Weichenthal SA, Crouse D, Miller AB, To T, Martin RV, van Donkelaar A, Wall C, Burnett RT. Long-term exposure to fine particulate matter air pollution and mortality among Canadian women. *Epidemiology*. 2015; 26(4): 536-45.
17. Weichenthal S, Hoppin JA, Reeves F. Obesity and cardiovascular health effects of fine particulate air pollution. *Obesity (Silver Spring).* 2014; 22(7): 1580-9.
18. Beelen R, Hoek G, van den Brandt PA, Goldbohm RA, Fischer P, Schouten LJ, Jerrett M, Hughes E, Armstrong B, Brunekreef B. Long-Term Effects of Traffic-Related Air Pollution on Mortality in a Dutch Cohort (NLCS-AIR Study) [Unpublished data]. *Environ Health Perspect*. 2008; 116(2): 196-202.
19. Chen LH, Knutsen SF, Shavlik D, Beeson WL, Petersen F, Ghamsary M, Abbey D. The association between fatal coronary heart disease and ambient particulate air pollution: Are females at greater risk? [Unpublished data]. *Environ Health Perspect*. 2005; 113(12): 1723-9.
20. Lepeule J, Laden F, Dockery D, Schwartz J. Chronic exposure to fine particles and mortality: an extended follow-up of the Harvard Six Cities study from 1974 to 2009 - Unpublished data. *Environ Health Perspect*. 2012; 120(7): 965-70.
21. Turner MC,Jerrett M, Pope CA 3rd, Krewski D, Gapstur SM, Diver WR, Beckerman BS, Marshall JD, Su J, Crouse DL, Burnett RT. Long-term ozone exposure and mortality in a large prospective study [Unpublished data]. *Am J Respir Crit Care Med*. 2016; 193(10): 1134-42.
22. Atkinson RW, Carey IM, Kent AJ, van Staa TP, Anderson HR, Cook DG. Long-term exposure to outdoor air pollution and the incidence of chronic obstructive pulmonary disease in a national English cohort. *Occup Environ Med*. 2015; 72(1): 42–8.
23. Raaschou-Nielsen O, Andersen ZJ, Beelen R, Samoli E, Stafoggia M, Weinmayr G, Hoffmann B, Fischer P, Nieuwenhuijsen MJ, Brunekreef B, Xun WW, Katsouyanni K, Dimakopoulou K, Sommar J, Forsberg B, Modig L, Oudin A, Oftedal B, Schwarze PE, Nafstad P, De Faire U, Pedersen NL, Ostenson C-G, Fratiglioni L, Penell J, Korek M, Pershagen G, Eriksen KT, Sørensen M, Tjønneland A, Ellermann T, Eeftens M, Peeters PH, Meliefste K, Wang M, Bueno-de-Mesquita B, Key TJ, de Hoogh K, Concin H, Nagel G, Vilier A, Grioni S, Krogh V, Tsai M-Y, Ricceri F, Sacerdote C, Galassi C, Migliore E, Ranzi A, Cesaroni G, Badaloni C, Forastiere F, Tamayo I, Amiano P, Dorronsoro M, Trichopoulou A, Bamia C, Vineis P, Hoek G. Air pollution and lung cancer

incidence in 17 European cohorts: prospective analyses from the European Study of Cohorts for Air Pollution Effects (ESCAPE). *Lancet Oncol.* 2013; 14(9): 813–22.

24. Gan WQ, FitzGerald JM, Carlsten C, Sadatsafavi M, Brauer M. Associations of ambient air pollution with chronic obstructive pulmonary disease hospitalization and mortality. *Am J Respir Crit Care Med*. 2013; 187(7): 721–7.

Relative risk estimate for Ozone

The GBD 2013 estimate (and the earlier GBD 2010 estimate reported by Lim S et al. 2012) used a log-linear concentration-response relationship for aggregated respiratory causes of death (460-519 ICD 9) reported by Jerrett et al. (2009) for the American Cancer Society (ACS) study that was robust to inclusion of $PM_{2.5}$, confounders and choice of analytic model. Estimates of annual average exposure to ozone were based on daily average maximum ozone levels from April through September reported by US EPA ground-level monitors. We used the estimate from a single pollutant model: RR=1.029 per 10 ppb (95% CI 1.010-1.048) (Jerrett et al. 2009, Table 3). In a subsequent unpublished analysis of conducted by Jerrett and colleagues the estimate for COPD ([490-496 ICD 9) in these data was: RR=1.027 per 10 ppb (95%CI 1.002-1.053) (Richard T Burnett, personal communication).

Reference

Jerrett M, Burnett RT, Pope CA 3rd, et al. Long-term ozone exposure and mortality. *N Engl J Med* 2009; **360:** 1085–95.

STAN code for fitting IER

Stan Development Team. 2015. Stan Modeling Language Users Guide and Reference Manual, Version 2.8.0.

```
##### RUN STAN MODEL #####

# Create the stan model object

# stan model

// specify the input data

data {

  // # observations

  int<lower=1> N;


  // log(RR) and sd(log(RR))

  vector[N] log_rr;

  vector[N] log_rr_sd;


  // sources

  int<lower=1> S;

  int<lower=1,upper=S> source[N];


  // observed exposure level

  vector[N] exposure;


  // counterfactual exposure level

  vector[N] cf_exposure;


  // test exposure values

  int<lower=1> T;            // number of test points
```

```
  vector[T] test_exposure;    // values at which to predict RR

  // theoretical minimum
  real tmrel;
}

// specify model parameters
parameters {
  // we're assuming that these values have to be positive
  // lower=1e-9 forces positive values in case we use a prior distribution that allows for negatives
  real<lower=1e-9> alpha;
  real<lower=1e-9> beta;
  real<lower=1e-9> gamma;
  real<lower=1e-9> delta[S];
}

// specify priors and data likelihood function
model {
  // make a temporary vector to store the predicted log(RR)
  vector[N] pred_log_rr;

  // non-informative prior on parameters
  alpha ~ gamma(1.0, 0.01);
  beta ~ gamma(1.0, 0.01);
  gamma ~ gamma(1.0, 0.01);
  delta ~ gamma(1.0, 0.01);
```

```
  // make predictions for log(RR) using alpha/beta/gamma
  for (n in 1:N) {
  pred_log_rr[n] <- log(
                    (1 + alpha * (1 - exp(-1 * beta * pow((exposure[n] - tmrel), gamma)))) /
                    (1 + alpha * (1 - exp(-1 * beta * pow((cf_exposure[n] - tmrel), gamma))))
                 );
  }

  // setup the data likelihood
  // our data is distributed with sd of sd(log(RR)) and mean being our predicted log(RR)
  for (n in 1:N) {
   log_rr[n] ~ normal(pred_log_rr[n], sqrt(pow(log_rr_sd[n],2) + pow(delta[source[n]], 2)));
  }
}

// generate values of RR at the test exposure levels
generated quantities {
  vector[T] predicted_RR;

  // generate predictions
  for (t in 1:T) {
  predicted_RR[t] <- if_else(test_exposure[t] > tmrel,
                   1 + alpha * (1 - exp(-1 * beta * pow((test_exposure[t] - tmrel), gamma))),
                   1.0);
  }
}
```

$PM_{2.5}$ Attributable YLD estimation

In order to estimate the DALYs attributable to ambient air pollution the fitted IERs were used to estimate the attributable YLL for all causes of health loss related to ambient air pollution. Since relative risks of both incidence and mortality were used to fit the IER curves for LRI, lung cancer, and COPD, RRs generated from these curves were applied evenly to produce estimates of YLL and Years of Life with Disability (YLD). For cardiovascular outcomes, the effect of air pollution is known to be significantly different for morbidity and mortality, therefore to estimate the YLD the fitted IERs were then re-scaled for IHD and stroke based on the ratio of excess relative risk of IHD and stroke incidence (as indicator of morbidity) and mortality in several major cohort studies.

In order to scale the YLDs for ambient air pollution, we used a ratio of the excess relative risk reported in three cohort studies (Miller et al. 2007; Lipsett et al. 2011; Puett et al. 2009) that reported mortality and incidence relative risks for IHD and stroke.

The ratio was applied as:

RR = ratio * IER(PM2.5) – ratio + 1

Or more simply:

RRc = ratio * RRm – ratio + 1

Stroke morbidity ratio: 0.553
IHD morbidity ratio: 0.141

All others: 1

Formula:
(RRc – 1) / (RRm – 1) = Ratio

<u>Stroke</u>
Miller et al. (2007): (1.28 – 1) / (1.82 – 1) = 0.341
Lipsett et al. (2011): (1.13 – 1) / (1.17 – 1) = 0.765
Avg: 0.553

<u>IHD</u>
Miller et al. (2007): (1.21 – 1) / (2.21 – 1) = 0.174
Puett et al. (2009): (1.11 – 1) / (2.02 – 1) = 0.108
Avg: 0.141

References

Lipsett MJ, Ostro BD, Reynolds P, Goldberg D, Hertz A, Jerrett M, Smith DF, Garcia C, Chang ET , Bernstein L. 2011. Long-Term Exposure to Air Pollution and Cardiorespiratory Disease in the California Teachers Study Cohort. Am J Respir Crit Care Med Vol 184. pp 828–835.

Miller KA, Siscovick DS, Sheppard L, Shepherd K, Sullivan JH, Anderson GL, Kaufman JD. 2007. Long-term exposure to air pollution and incidence of cardiovascular events in women. N Engl J Med .356:447–458.

Puett RC, Hart JE, Yanosky JD, Paciorek CJ, Schwartz J, Suh H, Speizer FE, Laden F. Chronic fine and coarse particulate exposure, mortality, and coronary heart disease in the Nurses' Health Study. 2009. Environ Health Perspect. 117:1697–1701.

ESTIMATION OF POPULATION ATTRIBUTABLE FRACTION (PAF) AND BURDEN

The numbers of DALYs and deaths attributable to ambient air pollution were calculated by applying the year-, location-, age- and sex-specific PAF to the numbers of DALYS and deaths, which were estimated as part of GBD 2015 (GBD 2015 Risk Factor Collaborators 2016). GBD 2015 included an expansion of countries for which we disaggregated our estimates at the subnational level. At the first level of subnational division, 256 geographic units were included in GBD 2015. For this paper we present results for the 195 national and territory-level geographies.

Cause-specific mortality for IHD, stroke, COPD, lung cancer and acute respiratory infections were estimated from vital registration and verbal autopsy data. YLLs due to each cause death were calculated by summing the number of years lost by due to death at a given age compared with reference standard population life expectancy. In addition, the prevalence of more than 1000 sequelae of the different causes were estimated by a Bayesian hierarchical model. YLDs were calculated as a product of prevalence of each sequela and the sequela's disability weight. Total burden was summarized in terms of DALYs by adding YLLs and total YLDs for each cause. Detailed descriptions of death and DALY estimation methods and results and the calculation of the PAF have been published elsewhere (GBD 2015 Mortality and Cause of Death Collaborators, 2016; GBD 2015 DALYs and HALE Collaborators, 2016; GBD 2015 Risk Factor Collaborators, 2016; GBD 2015 Disease and Injury Incidence and Prevalence Collaborators, 2016).

The relative risk for each cause was calculated at the $PM_{2.5}$ exposure grid-cell level from the IER.

The location-specific PAF is the proportion of outcomes, for example deaths from lung cancer, attributable to long-term exposure to ambient air pollution at a level greater than the TMREL. Because estimated death and DALYs were not available at the grid-cell level the country level (or subnational unit) PAFs were calculated using gridded population data and estimated as in Equation 1 below:

Eq. 1: $PAF = 1 - 1 \Big/ \frac{\sum Pop_i * (1/(1-paf_i)}{\sum Pop_i}$

Where Pop_{io} is population and paf_{io} is the PAF of outcome *o* in grid cell *i.*

To calculate total burden attributable to ambient air pollution is:

$$AB \quad = \sum_{o=1}^{w} DALY_o \, PAF_o$$

To compare the level of burden over time and among countries, different measures were calculated and reported, including: total number for all-age and by age, by sex and combined, all-age per person rate and also age standardized rate of death, DALYS, YLLs and YLDs. The WHO standard population was used to calculate age standardized deaths and DALYs per capita for every country. To preserve uncertainty of estimates for each step of analysis, we kept 1000 samples of posterior distribution for each step and used in later steps up to final results.

References

GBD 2015 Mortality and Causes of Death Collaborators. Global, regional, and national life expectancy, all-cause mortality, and cause-specific mortality for 249 causes of death, 1980–2015: a systematic analysis for the Global Burden of Disease Study 2015. *Lancet* 2016; **388:**1459–544.

GBD 2015 Disease and Injury Incidence and Prevalence Collaborators. Global, regional, and national incidence, prevalence, and years lived with disability for 310 diseases and injuries, 1990–2015: a systematic analysis for the Global Burden of Disease Study 2015. *Lancet* 2016; **388:** 1545–602.

GBD 2015 DALYs and HALE Collaborators. Global, regional, and national disability-adjusted life-years (DALYs) for 315 diseases and injuries and healthy life expectancy (HALE), 1990–2015: a systematic analysis for the Global Burden of Disease Study 2015. *Lancet* 2016; **388:**1603–58.

GBD 2015 Risk Factors Collaborators. Global, regional, and national comparative risk assessment of 79 behavioural, environmental and occupational, and metabolic risks or clusters of risks, 1990–2015: a systematic analysis for the Global Burden of Disease Study 2015. *Lancet*2016; **388:** 1659–724.

DECOMPOSITION OF GLOBAL TRENDS IN $PM_{2.5}$-ATTRIBUTABLE MORTALITY

Four factors - population growth, population aging, mortality rates independent of exposure to $PM_{2.5}$, and exposure to ambient $PM_{2.5}$ - contribute to the total mortality attributable to ambient $PM_{2.5}$ and the changes in these factors contributes to the overall change in attributable mortality**.** We calculated the contribution of changes in each of these factors to the net change in mortality attributable to ambient $PM_{2.5}$ between 1990 and 2015. The algorithm estimates the contribution from each factor incrementally. Following GBD 2015 decomposition of attributable burden, we used a method developed by Das Gupta to calculate average of each factor effect through all sequences of the four factors. For example for a sequence: first the increase due to population size (population growth), then the change in the age structure of the population (population aging), followed by the change in the rate of mortality unrelated to ambient $PM_{2.5}$ exposure (change in rate due to changes in access to care, treatment and other risk factors), and finally the change in exposure were calculated. The difference between every consecutive step is an estimate of the contribution of each factor.

$$A = \sum_{a=1}^{80+} Pop2015_a * \frac{\sum_{a=1}^{80+}(R1990_a * Pop1990_a)}{\sum_{a=1}^{80+}(Pop1990_a)} * All_{agePaf_{1990}})$$

$$B = \sum_{a=1}^{80+}(R1990_a * Pop2015_a * Paf1990_a)$$

$$C = \sum_{a=1}^{80+}\left(Pop2015_a * R2015_a * \left(\frac{(1 - Paf2015_a)}{1 - Paf1990_a}\right) * Paf1990_a\right)$$

Where, R=the cause-specific rate of mortality; PAF=the population attributable fraction; Pop: Population; a=age group

Population growth effect (%) = (A – total attributable burden in 1990) / total attributable burden in 1990

Population aging effect (%) = (B – A) / total attributable burden in 1990

Baseline rate change effect (%) = (C – B) / total attributable burden in 1990

Exposure change effect (%) = (total attributable burden in 2015 – C) / total attributable burden in 1990

Total change (%) = (total attributable burden in 2015 - total attributable burden in 1990)/ total attributable burden in 1990

References

GBD 2015 Risk Factors Collaborators. Global, regional, and national comparative risk assessment of 79 behavioural, environmental and occupational, and metabolic risks or clusters of risks in 195 countries, 1990–2015: a systematic analysis for the Global Burden of Disease Study 2015. Lancet 388: 1659–724.

Das Gupta P. Standardization and decomposition of rates: a user's manual. Washington, DC: US Bureau of the Census, 1993.

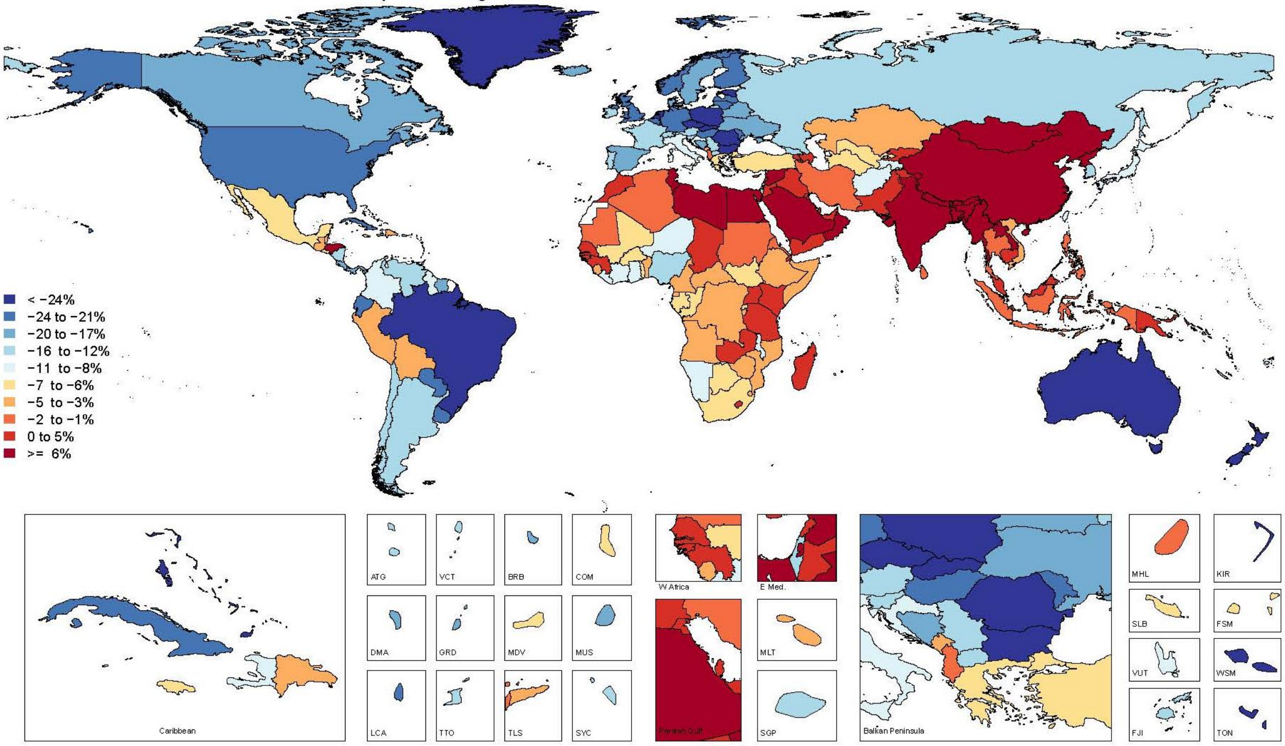

Web Figure 2. Percent change in population attributable fraction (PAF) for cardiovascular disease (IHD and stroke) attributable to PM2.5 in all countries from 1990 to 2015 for all ages and both sexes combined. ATG = Antigua and Barbuda. VCT = Saint Vincent and the Grenadines. BRB = Barbados. COM = Comoros. DMA = Dominica. GRD = Grenada. MDV = Maldives. MUS = Mauritius. LCA = Saint Lucia. TTO = Trinidad and Tobago. SYC = Seychelles. MLT = Malta. SGP = Singapore. MHL = Marshall Islands. KIR = Kiribati. SLB = Solomon Islands. FSM = Federated States of Micronesia. VUT = Vanuatu. WSM = Samoa. FJI = Fiji. TON = Tonga.

WEB Tables GLOBAL AND COUNTRY-LEVEL ESTIMATES OF DEATHS AND DALYS ATTRIBUTABLE TO $PM_{2.5}$ AND OZONE 1990-2015

Web Table 1: Deaths attributable to ambient particulate matter pollution for all ages and age-standardized (rate per 100,000) for both sexes combined, by cause and 95% uncertainty intervals (UI).

Web Table 2: DALYs attributable to ambient particulate matter pollution for all ages and age-standardized (rate per 100,000) for both sexes combined, by cause and 95% uncertainty intervals (UI).

Web Table 3: DALYs attributable to ambient ozone pollution for all ages and age-standardized (rate per 100,000) for both sexes combined, by cause and 95% uncertainty intervals (UI).

Web Table 4: Deaths attributable to ambient ozone pollution for all ages and age-standardized (rate per 100,000) for both sexes combined, by cause and 95% uncertainty intervals (UI).

Web Table 1: Deaths attributable to ambient particulate matter pollution for all ages and age-standardized (rate per 100,000) for both sexes combined, by cause and 95% uncertainty intervals (UI).						
**Cause name**	**Location**	**Year**	**Age group**	**Number (in thousands)**	**PAF**	**Rate (per 100,000)**
All causes	Global	1990	All Ages	3,476.7 (3,012.5 to 3,974.7)	7.3% (6.3 to 8.3%)	65.6 (56.8 to 74.9)
All causes	Global	1995	All Ages	3,654.8 (3,152.9 to 4,186.0)	7.3% (6.3 to 8.3%)	63.7 (54.9 to 72.9)
All causes	Global	2000	All Ages	3,794.0 (3,287.9 to 4,311.2)	7.3% (6.3 to 8.3%)	61.9 (53.6 to 70.3)
All causes	Global	2005	All Ages	3,934.3 (3,437.0 to 4,448.3)	7.3% (6.4 to 8.3%)	60.3 (52.7 to 68.2)
All causes	Global	2010	All Ages	3,944.7 (3,442.3 to 4,459.1)	7.3% (6.4 to 8.3%)	56.9 (49.7 to 64.3)
All causes	Global	2015	All Ages	4,241.1 (3,698.0 to 4,776.7)	7.6% (6.7 to 8.6%)	57.5 (50.2 to 64.8)
All causes	Global	1990	Age-standardized		7.9% (6.8 to 9.1%)	94.4 (80.8 to 108.9)
All causes	Global	1995	Age-standardized		7.8% (6.7 to 9.0%)	90.8 (77.8 to 104.5)
All causes	Global	2000	Age-standardized		7.8% (6.7 to 8.9%)	85.8 (73.9 to 98.3)
All causes	Global	2005	Age-standardized		7.7% (6.7 to 8.8%)	79.3 (68.7 to 90.1)
All causes	Global	2010	Age-standardized		7.6% (6.6 to 8.6%)	70.0 (60.8 to 79.7)
All causes	Global	2015	Age-standardized		7.8% (6.8 to 8.8%)	66.0 (57.2 to 74.8)
Lower respiratory infections	Global	1990	All Ages	941.7 (713.8 to 1,195.6)	27.7% (21.0 to 35.0%)	17.8 (13.5 to 22.5)
Lower respiratory infections	Global	1995	All Ages	865.2 (654.6 to 1,101.1)	27.2% (20.5 to 34.4%)	15.1 (11.4 to 19.2)
Lower respiratory infections	Global	2000	All Ages	803.0 (605.7 to 1,032.9)	26.8% (20.2 to 34.1%)	13.1 (9.9 to 16.8)
Lower respiratory infections	Global	2005	All Ages	736.1 (548.6 to 956.8)	26.0% (19.4 to 33.4%)	11.3 (8.4 to 14.7)
Lower respiratory infections	Global	2010	All Ages	679.9 (499.7 to 898.8)	24.8% (18.4 to 32.5%)	9.8 (7.2 to 13.0)
Lower respiratory infections	Global	2015	All Ages	675.0 (491.9 to 889.0)	24.7% (18.3 to 32.4%)	9.2 (6.7 to 12.1)
Lower respiratory infections	Global	1990	Age-standardized		26.1% (19.5 to 33.6%)	17.7 (13.2 to 23.0)
Lower respiratory infections	Global	1995	Age-standardized		25.7% (19.0 to 33.1%)	16.2 (12.1 to 21.1)
Lower respiratory infections	Global	2000	Age-standardized		25.5% (19.0 to 33.1%)	14.9 (11.1 to 19.5)
Lower respiratory infections	Global	2005	Age-standardized		25.1% (18.6 to 32.8%)	13.0 (9.6 to 17.1)
Lower respiratory infections	Global	2010	Age-standardized		24.2% (17.8 to 32.1%)	11.1 (8.1 to 14.9)
Lower respiratory infections	Global	2015	Age-standardized		24.3% (17.9 to 32.1%)	10.1 (7.4 to 13.4)
Tracheal, bronchus, and lung cancer	Global	1990	All Ages	150.7 (89.8 to 216.6)	14.7% (8.9 to 21.2%)	2.8 (1.7 to 4.1)
Tracheal, bronchus, and lung cancer	Global	1995	All Ages	165.8 (98.9 to 241.7)	14.3% (8.6 to 20.7%)	2.9 (1.7 to 4.2)
Tracheal, bronchus, and lung cancer	Global	2000	All Ages	191.6 (116.5 to 274.5)	14.8% (9.0 to 21.3%)	3.1 (1.9 to 4.5)
Tracheal, bronchus, and lung cancer	Global	2005	All Ages	225.4 (140.5 to 318.4)	15.7% (9.8 to 22.2%)	3.5 (2.2 to 4.9)
Tracheal, bronchus, and lung cancer	Global	2010	All Ages	249.7 (156.2 to 350.0)	16.0% (10.0 to 22.4%)	3.6 (2.3 to 5.0)
Tracheal, bronchus, and lung cancer	Global	2015	All Ages	283.3 (178.4 to 398.7)	16.4% (10.4 to 23.0%)	3.8 (2.4 to 5.4)
Tracheal, bronchus, and lung cancer	Global	1990	Age-standardized		14.6% (8.8 to 21.0%)	4.3 (2.5 to 6.1)
Tracheal, bronchus, and lung cancer	Global	1995	Age-standardized		14.1% (8.5 to 20.5%)	4.2 (2.5 to 6.1)
Tracheal, bronchus, and lung cancer	Global	2000	Age-standardized		14.6% (8.9 to 21.1%)	4.3 (2.6 to 6.2)
Tracheal, bronchus, and lung cancer	Global	2005	Age-standardized		15.6% (9.6 to 22.0%)	4.5 (2.8 to 6.4)
Tracheal, bronchus, and lung cancer	Global	2010	Age-standardized		15.8% (9.9 to 22.3%)	4.4 (2.8 to 6.2)

Cause name	Location	Year	Age group	Number<br>(in thousands)	PAF	Rate<br>(per 100,000)
Tracheal, bronchus, and lung cancer	Global	2015	Age-standardized		16.3%<br>(10.3 to 22.9%)	4.4<br>(2.7 to 6.1)
Cardiovascular diseases	Global	1990	All Ages	1,640.0<br>(1,417.0 to 1,867.7)	13.0%<br>(11.2 to 14.8%)	30.9<br>(26.7 to 35.2)
Cardiovascular diseases	Global	1995	All Ages	1,801.7<br>(1,558.8 to 2,056.1)	12.9%<br>(11.1 to 14.7%)	31.4<br>(27.2 to 35.8)
Cardiovascular diseases	Global	2000	All Ages	1,948.4<br>(1,678.7 to 2,219.7)	13.1%<br>(11.3 to 14.9%)	31.8<br>(27.4 to 36.2)
Cardiovascular diseases	Global	2005	All Ages	2,135.8<br>(1,844.3 to 2,426.2)	13.4%<br>(11.6 to 15.2%)	32.8<br>(28.3 to 37.2)
Cardiovascular diseases	Global	2010	All Ages	2,231.5<br>(1,915.7 to 2,553.2)	13.4%<br>(11.6 to 15.3%)	32.2<br>(27.6 to 36.8)
Cardiovascular diseases	Global	2015	All Ages	2,419.2<br>(2,068.9 to 2,784.7)	13.5%<br>(11.6 to 15.5%)	32.8<br>(28.1 to 37.8)
Cardiovascular diseases	Global	1990	Age-standardized		12.5%<br>(10.6 to 14.4%)	49.1<br>(41.8 to 56.9)
Cardiovascular diseases	Global	1995	Age-standardized		12.4%<br>(10.5 to 14.2%)	47.5<br>(40.5 to 54.9)
Cardiovascular diseases	Global	2000	Age-standardized		12.5%<br>(10.6 to 14.4%)	45.5<br>(38.7 to 52.4)
Cardiovascular diseases	Global	2005	Age-standardized		12.9%<br>(11.0 to 14.8%)	43.7<br>(37.2 to 50.1)
Cardiovascular diseases	Global	2010	Age-standardized		13.0%<br>(11.1 to 15.0%)	39.9<br>(33.9 to 46.0)
Cardiovascular diseases	Global	2015	Age-standardized		13.1%<br>(11.2 to 15.2%)	37.5<br>(31.9 to 43.5)
Ischemic heart disease	Global	1990	All Ages	996.0<br>(820.5 to 1,186.6)	16.6%<br>(13.8 to 19.7%)	18.8<br>(15.5 to 22.4)
Ischemic heart disease	Global	1995	All Ages	1,088.7<br>(891.5 to 1,295.1)	16.4%<br>(13.4 to 19.5%)	19.0<br>(15.5 to 22.6)
Ischemic heart disease	Global	2000	All Ages	1,160.6<br>(954.7 to 1,382.2)	16.6%<br>(13.6 to 19.6%)	18.9<br>(15.6 to 22.5)
Ischemic heart disease	Global	2005	All Ages	1,283.8<br>(1,060.1 to 1,530.4)	16.8%<br>(13.8 to 19.9%)	19.7<br>(16.3 to 23.5)
Ischemic heart disease	Global	2010	All Ages	1,380.3<br>(1,128.8 to 1,639.8)	16.9%<br>(13.9 to 20.1%)	19.9<br>(16.3 to 23.7)
Ischemic heart disease	Global	2015	All Ages	1,521.1<br>(1,231.7 to 1,821.2)	17.1%<br>(13.9 to 20.3%)	20.6<br>(16.7 to 24.7)
Ischemic heart disease	Global	1990	Age-standardized		15.7%<br>(12.5 to 19.0%)	29.9<br>(23.7 to 36.2)
Ischemic heart disease	Global	1995	Age-standardized		15.6%<br>(12.4 to 18.7%)	28.7<br>(22.9 to 34.7)
Ischemic heart disease	Global	2000	Age-standardized		15.7%<br>(12.5 to 18.9%)	27.1<br>(21.6 to 32.7)
Ischemic heart disease	Global	2005	Age-standardized		16.1%<br>(12.9 to 19.2%)	26.2<br>(21.1 to 31.6)
Ischemic heart disease	Global	2010	Age-standardized		16.3%<br>(13.1 to 19.6%)	24.6<br>(19.7 to 29.7)
Ischemic heart disease	Global	2015	Age-standardized		16.6%<br>(13.3 to 19.9%)	23.6<br>(18.9 to 28.5)
Ischemic stroke	Global	1990	All Ages	264.0<br>(197.9 to 329.8)	12.5%<br>(9.4 to 15.5%)	5.0<br>(3.7 to 6.2)
Ischemic stroke	Global	1995	All Ages	286.4<br>(214.0 to 359.3)	12.1%<br>(9.1 to 15.2%)	5.0<br>(3.7 to 6.3)
Ischemic stroke	Global	2000	All Ages	311.4<br>(232.1 to 389.5)	12.2%<br>(9.1 to 15.3%)	5.1<br>(3.8 to 6.4)
Ischemic stroke	Global	2005	All Ages	347.2<br>(259.8 to 431.6)	12.6%<br>(9.5 to 15.7%)	5.3<br>(4.0 to 6.6)
Ischemic stroke	Global	2010	All Ages	357.8<br>(264.5 to 450.6)	12.7%<br>(9.6 to 15.9%)	5.2<br>(3.8 to 6.5)
Ischemic stroke	Global	2015	All Ages	381.5<br>(282.8 to 482.6)	12.8%<br>(9.6 to 16.1%)	5.2<br>(3.8 to 6.5)
Ischemic stroke	Global	1990	Age-standardized		11.9%<br>(8.7 to 15.2%)	8.4<br>(6.1 to 10.8)
Ischemic stroke	Global	1995	Age-standardized		11.6%<br>(8.4 to 14.9%)	8.0<br>(5.8 to 10.3)
Ischemic stroke	Global	2000	Age-standardized		11.7%<br>(8.5 to 15.0%)	7.7<br>(5.6 to 9.9)
Ischemic stroke	Global	2005	Age-standardized		12.2%<br>(9.0 to 15.4%)	7.5<br>(5.5 to 9.5)
Ischemic stroke	Global	2010	Age-standardized		12.4%<br>(9.2 to 15.7%)	6.7<br>(4.9 to 8.5)

Cause name	Location	Year	Age group	Number (in thousands)	PAF	Rate (per 100,000)
Ischemic stroke	Global	2015	Age-standardized		12.6% (9.3 to 15.9%)	6.1 (4.5 to 7.8)
Hemorrhagic stroke	Global	1990	All Ages	380.0 (312.3 to 448.6)	15.1% (12.4 to 17.8%)	7.2 (5.9 to 8.5)
Hemorrhagic stroke	Global	1995	All Ages	426.6 (348.6 to 503.8)	15.1% (12.3 to 17.8%)	7.4 (6.1 to 8.8)
Hemorrhagic stroke	Global	2000	All Ages	476.4 (389.4 to 565.3)	15.2% (12.5 to 17.9%)	7.8 (6.4 to 9.2)
Hemorrhagic stroke	Global	2005	All Ages	504.9 (417.5 to 598.9)	15.5% (12.7 to 18.2%)	7.7 (6.4 to 9.2)
Hemorrhagic stroke	Global	2010	All Ages	493.5 (405.9 to 583.6)	15.4% (12.6 to 18.0%)	7.1 (5.9 to 8.4)
Hemorrhagic stroke	Global	2015	All Ages	516.6 (425.0 to 613.8)	15.4% (12.7 to 18.0%)	7.0 (5.8 to 8.3)
Hemorrhagic stroke	Global	1990	Age-standardized		14.6% (11.9 to 17.4%)	10.8 (8.7 to 12.8)
Hemorrhagic stroke	Global	1995	Age-standardized		14.5% (11.8 to 17.3%)	10.7 (8.7 to 12.8)
Hemorrhagic stroke	Global	2000	Age-standardized		14.6% (11.9 to 17.4%)	10.7 (8.7 to 12.7)
Hemorrhagic stroke	Global	2005	Age-standardized		15.0% (12.2 to 17.7%)	10.0 (8.1 to 11.9)
Hemorrhagic stroke	Global	2010	Age-standardized		14.9% (12.1 to 17.6%)	8.6 (7.0 to 10.2)
Hemorrhagic stroke	Global	2015	Age-standardized		15.0% (12.2 to 17.8%)	7.8 (6.4 to 9.3)
Chronic obstructive pulmonary disease	Global	1990	All Ages	744.3 (456.3 to 1,049.7)	26.1% (15.8 to 37.1%)	14.0 (8.6 to 19.8)
Chronic obstructive pulmonary disease	Global	1995	All Ages	822.2 (502.9 to 1,164.7)	26.2% (15.9 to 37.3%)	14.3 (8.8 to 20.3)
Chronic obstructive pulmonary disease	Global	2000	All Ages	851.0 (519.0 to 1,197.8)	26.4% (16.2 to 37.4%)	13.9 (8.5 to 19.5)
Chronic obstructive pulmonary disease	Global	2005	All Ages	837.0 (522.5 to 1,174.5)	27.0% (16.8 to 38.1%)	12.8 (8.0 to 18.0)
Chronic obstructive pulmonary disease	Global	2010	All Ages	783.6 (482.7 to 1,108.0)	26.4% (16.4 to 37.3%)	11.3 (7.0 to 16.0)
Chronic obstructive pulmonary disease	Global	2015	All Ages	863.6 (538.5 to 1,212.8)	27.1% (16.9 to 38.0%)	11.7 (7.3 to 16.5)
Chronic obstructive pulmonary disease	Global	1990	Age-standardized		26.4% (16.0 to 37.5%)	23.4 (14.3 to 33.0)
Chronic obstructive pulmonary disease	Global	1995	Age-standardized		26.3% (16.0 to 37.5%)	22.9 (14.1 to 32.5)
Chronic obstructive pulmonary disease	Global	2000	Age-standardized		26.5% (16.2 to 37.5%)	21.1 (12.8 to 29.8)
Chronic obstructive pulmonary disease	Global	2005	Age-standardized		27.0% (16.8 to 38.0%)	18.1 (11.3 to 25.4)
Chronic obstructive pulmonary disease	Global	2010	Age-standardized		26.3% (16.3 to 37.3%)	14.7 (9.0 to 20.7)
Chronic obstructive pulmonary disease	Global	2015	Age-standardized		27.0% (16.9 to 38.0%)	14.0 (8.7 to 19.6)
All causes	Canada	1990	All Ages	7.0 (5.4 to 8.8)	3.7% (2.8 to 4.6%)	25.6 (19.5 to 31.9)
All causes	Canada	1995	All Ages	7.2 (5.3 to 9.3)	3.4% (2.5 to 4.4%)	24.7 (18.3 to 31.8)
All causes	Canada	2000	All Ages	7.1 (5.2 to 9.3)	3.2% (2.4 to 4.2%)	23.1 (17.1 to 30.2)
All causes	Canada	2005	All Ages	7.2 (5.3 to 9.4)	3.1% (2.3 to 4.0%)	22.2 (16.5 to 29.2)
All causes	Canada	2010	All Ages	6.4 (4.6 to 8.6)	2.6% (1.8 to 3.5%)	18.8 (13.4 to 25.3)
All causes	Canada	2015	All Ages	7.1 (4.9 to 9.6)	2.6% (1.8 to 3.5%)	19.6 (13.6 to 26.6)
All causes	Canada	1990	Age-standardized		3.6% (2.8 to 4.5%)	23.9 (18.2 to 29.9)
All causes	Canada	1995	Age-standardized		3.4% (2.5 to 4.3%)	21.3 (15.9 to 27.2)
All causes	Canada	2000	Age-standardized		3.2% (2.4 to 4.1%)	18.3 (13.7 to 23.8)
All causes	Canada	2005	Age-standardized		3.0% (2.3 to 3.9%)	15.9 (11.9 to 20.6)
All causes	Canada	2010	Age-standardized		2.5% (1.8 to 3.3%)	12.3 (8.9 to 16.3)

Cause name	Location	Year	Age group	Number (in thousands)	PAF	Rate (per 100,000)
All causes	Canada	2015	Age-standardized		2.5% (1.8 to 3.3%)	11.9 (8.5 to 15.8)
Lower respiratory infections	Canada	1990	All Ages	0.4 (0.1 to 1.1)	6.3% (1.1 to 16.5%)	1.5 (0.3 to 4.1)
Lower respiratory infections	Canada	1995	All Ages	0.5 (0.1 to 1.2)	6.1% (1.0 to 16.3%)	1.6 (0.3 to 4.2)
Lower respiratory infections	Canada	2000	All Ages	0.4 (0.1 to 1.2)	6.1% (1.0 to 16.4%)	1.4 (0.2 to 4.0)
Lower respiratory infections	Canada	2005	All Ages	0.5 (0.1 to 1.2)	6.5% (1.1 to 16.9%)	1.4 (0.2 to 3.7)
Lower respiratory infections	Canada	2010	All Ages	0.4 (0.1 to 1.2)	5.5% (0.8 to 15.7%)	1.2 (0.2 to 3.4)
Lower respiratory infections	Canada	2015	All Ages	0.5 (0.1 to 1.3)	5.4% (0.7 to 15.4%)	1.3 (0.2 to 3.7)
Lower respiratory infections	Canada	1990	Age-standardized		6.3% (1.1 to 16.5%)	1.4 (0.2 to 3.9)
Lower respiratory infections	Canada	1995	Age-standardized		6.1% (1.0 to 16.3%)	1.3 (0.2 to 3.6)
Lower respiratory infections	Canada	2000	Age-standardized		6.1% (1.0 to 16.4%)	1.1 (0.2 to 3.1)
Lower respiratory infections	Canada	2005	Age-standardized		6.5% (1.1 to 16.9%)	1.0 (0.2 to 2.6)
Lower respiratory infections	Canada	2010	Age-standardized		5.5% (0.8 to 15.7%)	0.8 (0.1 to 2.2)
Lower respiratory infections	Canada	2015	Age-standardized		5.4% (0.7 to 15.4%)	0.8 (0.1 to 2.1)
Tracheal, bronchus, and lung cancer	Canada	1990	All Ages	0.6 (0.2 to 1.1)	4.2% (1.6 to 7.9%)	2.0 (0.8 to 3.8)
Tracheal, bronchus, and lung cancer	Canada	1995	All Ages	0.6 (0.2 to 1.2)	4.0% (1.5 to 7.7%)	2.1 (0.8 to 4.0)
Tracheal, bronchus, and lung cancer	Canada	2000	All Ages	0.7 (0.3 to 1.3)	4.0% (1.5 to 7.7%)	2.1 (0.8 to 4.1)
Tracheal, bronchus, and lung cancer	Canada	2005	All Ages	0.8 (0.3 to 1.4)	4.3% (1.6 to 8.2%)	2.4 (0.9 to 4.5)
Tracheal, bronchus, and lung cancer	Canada	2010	All Ages	0.7 (0.2 to 1.4)	3.6% (1.2 to 7.2%)	2.0 (0.7 to 4.1)
Tracheal, bronchus, and lung cancer	Canada	2015	All Ages	0.7 (0.3 to 1.5)	3.5% (1.2 to 7.0%)	2.0 (0.7 to 4.1)
Tracheal, bronchus, and lung cancer	Canada	1990	Age-standardized		4.2% (1.6 to 7.9%)	1.9 (0.7 to 3.5)
Tracheal, bronchus, and lung cancer	Canada	1995	Age-standardized		4.0% (1.5 to 7.7%)	1.8 (0.7 to 3.5)
Tracheal, bronchus, and lung cancer	Canada	2000	Age-standardized		4.0% (1.5 to 7.7%)	1.8 (0.7 to 3.4)
Tracheal, bronchus, and lung cancer	Canada	2005	Age-standardized		4.3% (1.6 to 8.1%)	1.8 (0.7 to 3.4)
Tracheal, bronchus, and lung cancer	Canada	2010	Age-standardized		3.6% (1.2 to 7.2%)	1.4 (0.5 to 2.8)
Tracheal, bronchus, and lung cancer	Canada	2015	Age-standardized		3.5% (1.2 to 7.0%)	1.3 (0.5 to 2.7)
Cardiovascular diseases	Canada	1990	All Ages	5.5 (4.0 to 6.9)	7.4% (5.5 to 9.3%)	19.7 (14.7 to 24.9)
Cardiovascular diseases	Canada	1995	All Ages	5.4 (3.9 to 7.0)	7.0% (5.1 to 9.0%)	18.5 (13.4 to 24.0)
Cardiovascular diseases	Canada	2000	All Ages	5.2 (3.7 to 6.8)	6.8% (4.9 to 8.9%)	16.8 (12.0 to 22.1)
Cardiovascular diseases	Canada	2005	All Ages	5.0 (3.5 to 6.6)	6.9% (4.9 to 9.1%)	15.5 (10.9 to 20.5)
Cardiovascular diseases	Canada	2010	All Ages	4.4 (3.0 to 6.1)	6.2% (4.2 to 8.5%)	13.0 (8.7 to 17.9)
Cardiovascular diseases	Canada	2015	All Ages	4.9 (3.3 to 7.0)	6.0% (4.0 to 8.5%)	13.6 (9.0 to 19.3)
Cardiovascular diseases	Canada	1990	Age-standardized		7.4% (5.5 to 9.3%)	18.5 (13.6 to 23.4)
Cardiovascular diseases	Canada	1995	Age-standardized		7.1% (5.2 to 9.1%)	15.9 (11.6 to 20.6)
Cardiovascular diseases	Canada	2000	Age-standardized		6.9% (5.0 to 9.0%)	13.3 (9.7 to 17.4)
Cardiovascular diseases	Canada	2005	Age-standardized		7.0% (5.1 to 9.1%)	11.0 (7.9 to 14.4)
Cardiovascular diseases	Canada	2010	Age-standardized		6.4% (4.5 to 8.4%)	8.5 (5.9 to 11.3)

Cause name	Location	Year	Age group	Number (in thousands)	PAF	Rate (per 100,000)
Cardiovascular diseases	Canada	2015	Age-standardized		6.2% (4.3 to 8.3%)	8.2 (5.7 to 11.2)
Ischemic heart disease	Canada	1990	All Ages	4.6 (3.2 to 6.0)	9.0% (6.4 to 11.8%)	16.6 (11.6 to 21.9)
Ischemic heart disease	Canada	1995	All Ages	4.5 (3.1 to 6.0)	8.7% (6.1 to 11.7%)	15.3 (10.6 to 20.5)
Ischemic heart disease	Canada	2000	All Ages	4.2 (2.9 to 5.8)	8.6% (5.8 to 11.6%)	13.8 (9.3 to 18.8)
Ischemic heart disease	Canada	2005	All Ages	4.0 (2.6 to 5.6)	8.8% (5.7 to 12.1%)	12.6 (8.2 to 17.3)
Ischemic heart disease	Canada	2010	All Ages	3.6 (2.2 to 5.1)	8.0% (5.0 to 11.4%)	10.5 (6.5 to 14.9)
Ischemic heart disease	Canada	2015	All Ages	4.0 (2.4 to 5.9)	7.8% (4.7 to 11.3%)	11.1 (6.7 to 16.3)
Ischemic heart disease	Canada	1990	Age-standardized		9.0% (6.4 to 11.8%)	15.5 (10.9 to 20.4)
Ischemic heart disease	Canada	1995	Age-standardized		8.8% (6.1 to 11.7%)	13.2 (9.2 to 17.7)
Ischemic heart disease	Canada	2000	Age-standardized		8.7% (5.9 to 11.7%)	11.0 (7.5 to 14.7)
Ischemic heart disease	Canada	2005	Age-standardized		9.0% (6.1 to 12.0%)	9.0 (6.1 to 12.1)
Ischemic heart disease	Canada	2010	Age-standardized		8.3% (5.5 to 11.3%)	6.9 (4.5 to 9.5)
Ischemic heart disease	Canada	2015	Age-standardized		8.0% (5.2 to 11.1%)	6.7 (4.3 to 9.4)
Ischemic stroke	Canada	1990	All Ages	0.6 (0.3 to 1.0)	5.5% (2.6 to 9.5%)	2.1 (1.0 to 3.5)
Ischemic stroke	Canada	1995	All Ages	0.6 (0.3 to 1.1)	5.4% (2.4 to 9.4%)	2.1 (0.9 to 3.7)
Ischemic stroke	Canada	2000	All Ages	0.6 (0.3 to 1.1)	5.4% (2.3 to 9.5%)	2.0 (0.9 to 3.7)
Ischemic stroke	Canada	2005	All Ages	0.6 (0.3 to 1.2)	5.5% (2.3 to 10.1%)	1.9 (0.8 to 3.6)
Ischemic stroke	Canada	2010	All Ages	0.6 (0.2 to 1.1)	5.0% (1.9 to 9.6%)	1.7 (0.7 to 3.3)
Ischemic stroke	Canada	2015	All Ages	0.6 (0.2 to 1.2)	4.9% (1.8 to 9.7%)	1.7 (0.6 to 3.3)
Ischemic stroke	Canada	1990	Age-standardized		5.5% (2.6 to 9.6%)	1.9 (0.9 to 3.3)
Ischemic stroke	Canada	1995	Age-standardized		5.4% (2.4 to 9.4%)	1.8 (0.8 to 3.1)
Ischemic stroke	Canada	2000	Age-standardized		5.4% (2.4 to 9.5%)	1.6 (0.7 to 2.8)
Ischemic stroke	Canada	2005	Age-standardized		5.6% (2.5 to 9.9%)	1.3 (0.6 to 2.3)
Ischemic stroke	Canada	2010	Age-standardized		5.1% (2.1 to 9.4%)	1.0 (0.4 to 1.9)
Ischemic stroke	Canada	2015	Age-standardized		4.9% (2.0 to 9.4%)	1.0 (0.4 to 1.8)
Hemorrhagic stroke	Canada	1990	All Ages	0.3 (0.2 to 0.4)	6.6% (4.2 to 9.2%)	1.1 (0.7 to 1.5)
Hemorrhagic stroke	Canada	1995	All Ages	0.3 (0.2 to 0.4)	6.3% (4.0 to 9.1%)	1.1 (0.7 to 1.5)
Hemorrhagic stroke	Canada	2000	All Ages	0.3 (0.2 to 0.5)	6.3% (3.9 to 9.2%)	1.0 (0.6 to 1.5)
Hemorrhagic stroke	Canada	2005	All Ages	0.3 (0.2 to 0.5)	6.4% (3.9 to 9.6%)	1.0 (0.6 to 1.4)
Hemorrhagic stroke	Canada	2010	All Ages	0.3 (0.2 to 0.4)	5.8% (3.3 to 9.0%)	0.8 (0.5 to 1.3)
Hemorrhagic stroke	Canada	2015	All Ages	0.3 (0.2 to 0.5)	5.6% (3.1 to 9.0%)	0.8 (0.4 to 1.3)
Hemorrhagic stroke	Canada	1990	Age-standardized		6.6% (4.3 to 9.2%)	1.0 (0.6 to 1.4)
Hemorrhagic stroke	Canada	1995	Age-standardized		6.4% (4.1 to 9.1%)	0.9 (0.6 to 1.3)
Hemorrhagic stroke	Canada	2000	Age-standardized		6.3% (4.0 to 9.2%)	0.8 (0.5 to 1.1)
Hemorrhagic stroke	Canada	2005	Age-standardized		6.6% (4.2 to 9.4%)	0.7 (0.4 to 1.0)
Hemorrhagic stroke	Canada	2010	Age-standardized		5.9% (3.6 to 8.8%)	0.6 (0.3 to 0.8)

Cause name	Location	Year	Age group	Number (in thousands)	PAF	Rate (per 100,000)
Hemorrhagic stroke	Canada	2015	Age-standardized		5.7% (3.4 to 8.6%)	0.5 (0.3 to 0.8)
Chronic obstructive pulmonary disease	Canada	1990	All Ages	0.6 (0.2 to 1.3)	8.4% (2.3 to 17.1%)	2.3 (0.6 to 4.8)
Chronic obstructive pulmonary disease	Canada	1995	All Ages	0.8 (0.2 to 1.6)	8.2% (2.1 to 17.1%)	2.6 (0.7 to 5.4)
Chronic obstructive pulmonary disease	Canada	2000	All Ages	0.8 (0.2 to 1.7)	8.3% (2.1 to 17.0%)	2.7 (0.7 to 5.7)
Chronic obstructive pulmonary disease	Canada	2005	All Ages	0.9 (0.3 to 1.9)	8.7% (2.4 to 17.6%)	2.9 (0.8 to 5.9)
Chronic obstructive pulmonary disease	Canada	2010	All Ages	0.9 (0.2 to 1.9)	7.6% (1.8 to 16.2%)	2.5 (0.6 to 5.4)
Chronic obstructive pulmonary disease	Canada	2015	All Ages	0.9 (0.2 to 2.1)	7.4% (1.6 to 16.3%)	2.6 (0.6 to 5.7)
Chronic obstructive pulmonary disease	Canada	1990	Age-standardized		8.4% (2.3 to 17.1%)	2.1 (0.6 to 4.4)
Chronic obstructive pulmonary disease	Canada	1995	Age-standardized		8.2% (2.1 to 17.1%)	2.2 (0.6 to 4.6)
Chronic obstructive pulmonary disease	Canada	2000	Age-standardized		8.2% (2.1 to 17.0%)	2.1 (0.5 to 4.4)
Chronic obstructive pulmonary disease	Canada	2005	Age-standardized		8.6% (2.4 to 17.6%)	2.0 (0.5 to 4.1)
Chronic obstructive pulmonary disease	Canada	2010	Age-standardized		7.6% (1.8 to 16.2%)	1.6 (0.4 to 3.5)
Chronic obstructive pulmonary disease	Canada	2015	Age-standardized		7.4% (1.6 to 16.3%)	1.6 (0.4 to 3.4)
All causes	Greenland	1990	All Ages	0.0 (0.0 to 0.0)	1.7% (1.1 to 2.3%)	13.3 (8.7 to 18.7)
All causes	Greenland	1995	All Ages	0.0 (0.0 to 0.0)	1.8% (1.2 to 2.4%)	12.5 (8.3 to 17.1)
All causes	Greenland	2000	All Ages	0.0 (0.0 to 0.0)	1.8% (1.2 to 2.6%)	12.7 (8.4 to 17.8)
All causes	Greenland	2005	All Ages	0.0 (0.0 to 0.0)	1.6% (1.0 to 2.3%)	11.0 (6.7 to 15.8)
All causes	Greenland	2010	All Ages	0.0 (0.0 to 0.0)	1.2% (0.6 to 1.8%)	8.0 (4.3 to 12.4)
All causes	Greenland	2015	All Ages	0.0 (0.0 to 0.0)	1.5% (0.9 to 2.3%)	9.7 (5.5 to 14.7)
All causes	Greenland	1990	Age-standardized		2.3% (1.5 to 3.2%)	32.8 (21.2 to 46.1)
All causes	Greenland	1995	Age-standardized		2.2% (1.5 to 3.1%)	29.2 (19.1 to 41.2)
All causes	Greenland	2000	Age-standardized		2.2% (1.4 to 3.1%)	26.9 (17.3 to 38.5)
All causes	Greenland	2005	Age-standardized		1.8% (1.1 to 2.7%)	20.7 (12.0 to 31.4)
All causes	Greenland	2010	Age-standardized		1.3% (0.7 to 2.1%)	13.7 (7.3 to 22.0)
All causes	Greenland	2015	Age-standardized		1.6% (0.9 to 2.4%)	14.9 (8.1 to 22.8)
Lower respiratory infections	Greenland	1990	All Ages	0.0 (0.0 to 0.0)	4.0% (0.4 to 13.7%)	0.9 (0.1 to 3.2)
Lower respiratory infections	Greenland	1995	All Ages	0.0 (0.0 to 0.0)	3.9% (0.4 to 13.1%)	0.7 (0.1 to 2.5)
Lower respiratory infections	Greenland	2000	All Ages	0.0 (0.0 to 0.0)	3.9% (0.4 to 12.9%)	0.6 (0.1 to 2.3)
Lower respiratory infections	Greenland	2005	All Ages	0.0 (0.0 to 0.0)	3.2% (0.2 to 11.7%)	0.5 (0.0 to 2.1)
Lower respiratory infections	Greenland	2010	All Ages	0.0 (0.0 to 0.0)	2.3% (0.1 to 9.9%)	0.4 (0.0 to 1.7)
Lower respiratory infections	Greenland	2015	All Ages	0.0 (0.0 to 0.0)	2.9% (0.2 to 11.5%)	0.5 (0.0 to 2.1)
Lower respiratory infections	Greenland	1990	Age-standardized		4.0% (0.4 to 13.7%)	2.0 (0.2 to 6.9)
Lower respiratory infections	Greenland	1995	Age-standardized		3.9% (0.4 to 13.1%)	1.7 (0.1 to 6.0)
Lower respiratory infections	Greenland	2000	Age-standardized		3.9% (0.4 to 12.9%)	1.6 (0.1 to 5.5)
Lower respiratory infections	Greenland	2005	Age-standardized		3.2% (0.2 to 11.7%)	1.2 (0.1 to 4.9)
Lower respiratory infections	Greenland	2010	Age-standardized		2.3% (0.1 to 9.9%)	0.8 (0.0 to 3.7)

Cause name	Location	Year	Age group	Number (in thousands)	PAF	Rate (per 100,000)
Lower respiratory infections	Greenland	2015	Age-standardized		2.9% (0.2 to 11.5%)	1.0 (0.0 to 4.1)
Tracheal, bronchus, and lung cancer	Greenland	1990	All Ages	0.0 (0.0 to 0.0)	2.5% (0.6 to 6.1%)	1.5 (0.4 to 3.6)
Tracheal, bronchus, and lung cancer	Greenland	1995	All Ages	0.0 (0.0 to 0.0)	2.4% (0.5 to 5.8%)	1.5 (0.3 to 3.5)
Tracheal, bronchus, and lung cancer	Greenland	2000	All Ages	0.0 (0.0 to 0.0)	2.4% (0.5 to 5.8%)	1.6 (0.4 to 3.8)
Tracheal, bronchus, and lung cancer	Greenland	2005	All Ages	0.0 (0.0 to 0.0)	2.0% (0.4 to 5.1%)	1.4 (0.2 to 3.6)
Tracheal, bronchus, and lung cancer	Greenland	2010	All Ages	0.0 (0.0 to 0.0)	1.4% (0.2 to 4.1%)	1.0 (0.1 to 3.0)
Tracheal, bronchus, and lung cancer	Greenland	2015	All Ages	0.0 (0.0 to 0.0)	1.8% (0.2 to 4.8%)	1.4 (0.2 to 3.8)
Tracheal, bronchus, and lung cancer	Greenland	1990	Age-standardized		2.5% (0.6 to 6.1%)	3.4 (0.8 to 8.0)
Tracheal, bronchus, and lung cancer	Greenland	1995	Age-standardized		2.4% (0.5 to 5.8%)	3.0 (0.6 to 7.0)
Tracheal, bronchus, and lung cancer	Greenland	2000	Age-standardized		2.4% (0.5 to 5.8%)	2.9 (0.7 to 6.9)
Tracheal, bronchus, and lung cancer	Greenland	2005	Age-standardized		2.0% (0.4 to 5.1%)	2.2 (0.4 to 5.6)
Tracheal, bronchus, and lung cancer	Greenland	2010	Age-standardized		1.4% (0.2 to 4.1%)	1.4 (0.2 to 4.2)
Tracheal, bronchus, and lung cancer	Greenland	2015	Age-standardized		1.8% (0.2 to 4.8%)	1.7 (0.2 to 4.8)
Cardiovascular diseases	Greenland	1990	All Ages	0.0 (0.0 to 0.0)	6.0% (4.1 to 8.0%)	9.5 (6.4 to 12.7)
Cardiovascular diseases	Greenland	1995	All Ages	0.0 (0.0 to 0.0)	5.9% (4.1 to 7.7%)	8.9 (6.2 to 12.0)
Cardiovascular diseases	Greenland	2000	All Ages	0.0 (0.0 to 0.0)	5.7% (3.8 to 7.7%)	9.0 (5.9 to 12.2)
Cardiovascular diseases	Greenland	2005	All Ages	0.0 (0.0 to 0.0)	4.9% (3.1 to 6.8%)	7.7 (4.9 to 10.9)
Cardiovascular diseases	Greenland	2010	All Ages	0.0 (0.0 to 0.0)	3.7% (2.1 to 5.5%)	5.6 (3.1 to 8.5)
Cardiovascular diseases	Greenland	2015	All Ages	0.0 (0.0 to 0.0)	4.4% (2.6 to 6.3%)	6.5 (3.9 to 9.5)
Cardiovascular diseases	Greenland	1990	Age-standardized		5.2% (3.4 to 7.1%)	23.1 (15.0 to 32.2)
Cardiovascular diseases	Greenland	1995	Age-standardized		5.0% (3.3 to 6.8%)	20.6 (13.6 to 28.7)
Cardiovascular diseases	Greenland	2000	Age-standardized		4.9% (3.1 to 6.8%)	18.7 (12.0 to 26.7)
Cardiovascular diseases	Greenland	2005	Age-standardized		4.1% (2.5 to 6.1%)	14.3 (8.4 to 21.1)
Cardiovascular diseases	Greenland	2010	Age-standardized		3.2% (1.7 to 4.9%)	9.4 (4.9 to 14.6)
Cardiovascular diseases	Greenland	2015	Age-standardized		3.7% (2.0 to 5.6%)	10.0 (5.5 to 15.1)
Ischemic heart disease	Greenland	1990	All Ages	0.0 (0.0 to 0.0)	8.5% (5.4 to 11.7%)	6.6 (4.2 to 9.1)
Ischemic heart disease	Greenland	1995	All Ages	0.0 (0.0 to 0.0)	8.2% (5.3 to 11.3%)	6.2 (4.0 to 8.7)
Ischemic heart disease	Greenland	2000	All Ages	0.0 (0.0 to 0.0)	8.1% (5.1 to 11.5%)	6.2 (3.8 to 8.8)
Ischemic heart disease	Greenland	2005	All Ages	0.0 (0.0 to 0.0)	7.0% (4.0 to 10.4%)	5.3 (3.0 to 7.9)
Ischemic heart disease	Greenland	2010	All Ages	0.0 (0.0 to 0.0)	5.5% (2.6 to 8.7%)	3.9 (1.8 to 6.2)
Ischemic heart disease	Greenland	2015	All Ages	0.0 (0.0 to 0.0)	6.5% (3.4 to 9.8%)	4.7 (2.4 to 7.1)
Ischemic heart disease	Greenland	1990	Age-standardized		7.2% (4.5 to 10.0%)	15.9 (9.7 to 22.8)
Ischemic heart disease	Greenland	1995	Age-standardized		6.9% (4.1 to 9.8%)	14.1 (8.5 to 20.7)
Ischemic heart disease	Greenland	2000	Age-standardized		6.8% (3.9 to 9.7%)	12.7 (7.3 to 18.5)
Ischemic heart disease	Greenland	2005	Age-standardized		5.9% (3.1 to 9.1%)	9.7 (5.1 to 15.1)
Ischemic heart disease	Greenland	2010	Age-standardized		4.7% (2.1 to 7.7%)	6.4 (2.8 to 10.6)

Cause name	Location	Year	Age group	Number (in thousands)	PAF	Rate (per 100,000)
Ischemic heart disease	Greenland	2015	Age-standardized		5.5% (2.7 to 8.5%)	6.9 (3.2 to 10.7)
Ischemic stroke	Greenland	1990	All Ages	0.0 (0.0 to 0.0)	4.6% (2.5 to 7.4%)	0.8 (0.4 to 1.3)
Ischemic stroke	Greenland	1995	All Ages	0.0 (0.0 to 0.0)	4.5% (2.3 to 7.0%)	0.7 (0.4 to 1.1)
Ischemic stroke	Greenland	2000	All Ages	0.0 (0.0 to 0.0)	4.6% (2.3 to 7.4%)	0.8 (0.4 to 1.3)
Ischemic stroke	Greenland	2005	All Ages	0.0 (0.0 to 0.0)	3.9% (1.9 to 6.7%)	0.7 (0.3 to 1.2)
Ischemic stroke	Greenland	2010	All Ages	0.0 (0.0 to 0.0)	3.0% (1.2 to 5.7%)	0.5 (0.2 to 0.9)
Ischemic stroke	Greenland	2015	All Ages	0.0 (0.0 to 0.0)	3.7% (1.6 to 6.5%)	0.6 (0.3 to 1.0)
Ischemic stroke	Greenland	1990	Age-standardized		4.2% (1.7 to 7.8%)	3.0 (1.2 to 5.6)
Ischemic stroke	Greenland	1995	Age-standardized		4.0% (1.7 to 7.4%)	2.7 (1.1 to 5.1)
Ischemic stroke	Greenland	2000	Age-standardized		4.0% (1.7 to 7.5%)	2.6 (1.0 to 5.0)
Ischemic stroke	Greenland	2005	Age-standardized		3.5% (1.2 to 7.2%)	2.0 (0.7 to 4.2)
Ischemic stroke	Greenland	2010	Age-standardized		2.7% (0.8 to 6.3%)	1.3 (0.4 to 3.1)
Ischemic stroke	Greenland	2015	Age-standardized		3.2% (1.0 to 6.9%)	1.4 (0.4 to 3.1)
Hemorrhagic stroke	Greenland	1990	All Ages	0.0 (0.0 to 0.0)	5.7% (3.3 to 8.7%)	2.1 (1.2 to 3.2)
Hemorrhagic stroke	Greenland	1995	All Ages	0.0 (0.0 to 0.0)	5.6% (3.2 to 8.2%)	1.9 (1.1 to 2.9)
Hemorrhagic stroke	Greenland	2000	All Ages	0.0 (0.0 to 0.0)	5.6% (3.1 to 8.4%)	2.0 (1.1 to 3.2)
Hemorrhagic stroke	Greenland	2005	All Ages	0.0 (0.0 to 0.0)	4.7% (2.4 to 7.5%)	1.7 (0.9 to 2.7)
Hemorrhagic stroke	Greenland	2010	All Ages	0.0 (0.0 to 0.0)	3.6% (1.6 to 6.3%)	1.2 (0.5 to 2.1)
Hemorrhagic stroke	Greenland	2015	All Ages	0.0 (0.0 to 0.0)	4.3% (2.2 to 7.3%)	1.3 (0.6 to 2.3)
Hemorrhagic stroke	Greenland	1990	Age-standardized		5.0% (2.8 to 7.7%)	4.2 (2.3 to 6.8)
Hemorrhagic stroke	Greenland	1995	Age-standardized		4.8% (2.6 to 7.4%)	3.7 (2.0 to 5.9)
Hemorrhagic stroke	Greenland	2000	Age-standardized		4.8% (2.6 to 7.4%)	3.4 (1.8 to 5.5)
Hemorrhagic stroke	Greenland	2005	Age-standardized		4.1% (2.0 to 6.8%)	2.6 (1.3 to 4.5)
Hemorrhagic stroke	Greenland	2010	Age-standardized		3.2% (1.3 to 5.8%)	1.7 (0.7 to 3.1)
Hemorrhagic stroke	Greenland	2015	Age-standardized		3.8% (1.8 to 6.3%)	1.7 (0.8 to 3.0)
Chronic obstructive pulmonary disease	Greenland	1990	All Ages	0.0 (0.0 to 0.0)	5.7% (0.8 to 13.6%)	1.5 (0.2 to 3.5)
Chronic obstructive pulmonary disease	Greenland	1995	All Ages	0.0 (0.0 to 0.0)	5.5% (0.9 to 13.4%)	1.4 (0.2 to 3.6)
Chronic obstructive pulmonary disease	Greenland	2000	All Ages	0.0 (0.0 to 0.0)	5.5% (0.9 to 13.6%)	1.5 (0.3 to 3.7)
Chronic obstructive pulmonary disease	Greenland	2005	All Ages	0.0 (0.0 to 0.0)	4.6% (0.5 to 12.3%)	1.4 (0.2 to 3.9)
Chronic obstructive pulmonary disease	Greenland	2010	All Ages	0.0 (0.0 to 0.0)	3.5% (0.3 to 10.7%)	1.0 (0.1 to 3.2)
Chronic obstructive pulmonary disease	Greenland	2015	All Ages	0.0 (0.0 to 0.0)	4.3% (0.5 to 11.9%)	1.2 (0.1 to 3.5)
Chronic obstructive pulmonary disease	Greenland	1990	Age-standardized		5.7% (0.8 to 13.6%)	4.4 (0.6 to 10.6)
Chronic obstructive pulmonary disease	Greenland	1995	Age-standardized		5.5% (0.9 to 13.5%)	4.0 (0.6 to 10.0)
Chronic obstructive pulmonary disease	Greenland	2000	Age-standardized		5.5% (0.9 to 13.6%)	3.8 (0.6 to 9.2)
Chronic obstructive pulmonary disease	Greenland	2005	Age-standardized		4.6% (0.5 to 12.3%)	3.1 (0.3 to 8.5)
Chronic obstructive pulmonary disease	Greenland	2010	Age-standardized		3.5% (0.3 to 10.7%)	2.0 (0.2 to 6.2)

Cause name	Location	Year	Age group	Number (in thousands)	PAF	Rate (per 100,000)
Chronic obstructive pulmonary disease	Greenland	2015	Age-standardized		4.3% (0.5 to 11.9%)	2.2 (0.2 to 6.2)
All causes	United States	1990	All Ages	106.0 (84.4 to 129.2)	4.9% (3.9 to 6.0%)	42.4 (33.8 to 51.7)
All causes	United States	1995	All Ages	107.2 (84.8 to 132.8)	4.6% (3.6 to 5.7%)	40.3 (31.8 to 49.8)
All causes	United States	2000	All Ages	106.2 (83.8 to 132.9)	4.4% (3.5 to 5.6%)	37.6 (29.7 to 47.1)
All causes	United States	2005	All Ages	100.0 (78.2 to 127.0)	4.1% (3.2 to 5.2%)	33.8 (26.5 to 42.9)
All causes	United States	2010	All Ages	83.4 (63.6 to 107.8)	3.4% (2.6 to 4.4%)	26.9 (20.5 to 34.8)
All causes	United States	2015	All Ages	88.4 (66.8 to 115.0)	3.3% (2.5 to 4.3%)	27.3 (20.7 to 35.6)
All causes	United States	1990	Age-standardized		4.9% (3.9 to 5.9%)	36.2 (29.1 to 43.9)
All causes	United States	1995	Age-standardized		4.6% (3.7 to 5.5%)	33.1 (26.5 to 40.3)
All causes	United States	2000	Age-standardized		4.4% (3.5 to 5.4%)	30.0 (24.0 to 37.1)
All causes	United States	2005	Age-standardized		4.0% (3.2 to 5.0%)	25.7 (20.4 to 32.0)
All causes	United States	2010	Age-standardized		3.3% (2.6 to 4.2%)	19.3 (14.9 to 24.7)
All causes	United States	2015	Age-standardized		3.2% (2.5 to 4.1%)	18.5 (14.2 to 23.7)
Lower respiratory infections	United States	1990	All Ages	7.7 (2.2 to 16.3)	9.9% (2.8 to 20.8%)	3.1 (0.9 to 6.5)
Lower respiratory infections	United States	1995	All Ages	8.3 (2.2 to 18.1)	9.4% (2.5 to 20.3%)	3.1 (0.8 to 6.8)
Lower respiratory infections	United States	2000	All Ages	8.2 (2.1 to 18.1)	9.3% (2.4 to 20.2%)	2.9 (0.8 to 6.4)
Lower respiratory infections	United States	2005	All Ages	7.7 (1.9 to 17.2)	9.1% (2.2 to 20.0%)	2.6 (0.6 to 5.8)
Lower respiratory infections	United States	2010	All Ages	6.0 (1.1 to 15.0)	7.2% (1.4 to 17.8%)	1.9 (0.4 to 4.8)
Lower respiratory infections	United States	2015	All Ages	6.4 (1.2 to 16.2)	6.9% (1.3 to 17.6%)	2.0 (0.4 to 5.0)
Lower respiratory infections	United States	1990	Age-standardized		9.9% (2.8 to 20.8%)	2.6 (0.7 to 5.4)
Lower respiratory infections	United States	1995	Age-standardized		9.4% (2.5 to 20.3%)	2.4 (0.6 to 5.3)
Lower respiratory infections	United States	2000	Age-standardized		9.3% (2.4 to 20.2%)	2.2 (0.6 to 4.9)
Lower respiratory infections	United States	2005	Age-standardized		9.1% (2.2 to 20.0%)	1.9 (0.5 to 4.3)
Lower respiratory infections	United States	2010	Age-standardized		7.2% (1.4 to 17.8%)	1.4 (0.3 to 3.4)
Lower respiratory infections	United States	2015	Age-standardized		6.9% (1.3 to 17.6%)	1.3 (0.2 to 3.3)
Tracheal, bronchus, and lung cancer	United States	1990	All Ages	10.1 (4.6 to 17.1)	6.8% (3.1 to 11.5%)	4.0 (1.9 to 6.9)
Tracheal, bronchus, and lung cancer	United States	1995	All Ages	10.5 (4.8 to 18.0)	6.4% (2.9 to 11.0%)	3.9 (1.8 to 6.8)
Tracheal, bronchus, and lung cancer	United States	2000	All Ages	10.5 (4.7 to 18.0)	6.3% (2.8 to 10.8%)	3.7 (1.7 to 6.4)
Tracheal, bronchus, and lung cancer	United States	2005	All Ages	10.3 (4.6 to 17.9)	6.1% (2.7 to 10.6%)	3.5 (1.5 to 6.0)
Tracheal, bronchus, and lung cancer	United States	2010	All Ages	8.1 (3.2 to 14.8)	4.7% (1.9 to 8.6%)	2.6 (1.0 to 4.8)
Tracheal, bronchus, and lung cancer	United States	2015	All Ages	8.5 (3.4 to 15.6)	4.5% (1.8 to 8.4%)	2.6 (1.0 to 4.8)
Tracheal, bronchus, and lung cancer	United States	1990	Age-standardized		6.8% (3.1 to 11.5%)	3.6 (1.6 to 6.1)
Tracheal, bronchus, and lung cancer	United States	1995	Age-standardized		6.4% (2.9 to 11.0%)	3.4 (1.5 to 5.9)
Tracheal, bronchus, and lung cancer	United States	2000	Age-standardized		6.3% (2.8 to 10.8%)	3.1 (1.4 to 5.4)
Tracheal, bronchus, and lung cancer	United States	2005	Age-standardized		6.1% (2.7 to 10.6%)	2.8 (1.2 to 4.9)
Tracheal, bronchus, and lung cancer	United States	2010	Age-standardized		4.7% (1.9 to 8.6%)	2.0 (0.8 to 3.6)

Cause name	Location	Year	Age group	Number (in thousands)	PAF	Rate (per 100,000)
Tracheal, bronchus, and lung cancer	United States	2015	Age-standardized		4.5% (1.8 to 8.4%)	1.9 (0.7 to 3.4)
Cardiovascular diseases	United States	1990	All Ages	78.0 (59.9 to 96.9)	9.0% (6.9 to 11.1%)	31.2 (24.0 to 38.8)
Cardiovascular diseases	United States	1995	All Ages	76.6 (57.7 to 96.0)	8.5% (6.4 to 10.7%)	28.8 (21.7 to 36.1)
Cardiovascular diseases	United States	2000	All Ages	73.8 (55.0 to 93.1)	8.3% (6.2 to 10.4%)	26.1 (19.5 to 33.0)
Cardiovascular diseases	United States	2005	All Ages	67.4 (50.1 to 85.2)	8.1% (6.0 to 10.2%)	22.8 (17.0 to 28.8)
Cardiovascular diseases	United States	2010	All Ages	56.4 (40.9 to 72.7)	7.1% (5.2 to 9.1%)	18.2 (13.2 to 23.5)
Cardiovascular diseases	United States	2015	All Ages	59.8 (42.8 to 77.5)	6.9% (5.0 to 9.0%)	18.5 (13.2 to 24.0)
Cardiovascular diseases	United States	1990	Age-standardized		9.2% (7.1 to 11.2%)	26.6 (20.6 to 32.6)
Cardiovascular diseases	United States	1995	Age-standardized		8.7% (6.7 to 10.8%)	23.6 (18.1 to 29.2)
Cardiovascular diseases	United States	2000	Age-standardized		8.5% (6.5 to 10.6%)	20.8 (15.9 to 25.7)
Cardiovascular diseases	United States	2005	Age-standardized		8.3% (6.3 to 10.3%)	17.2 (13.1 to 21.3)
Cardiovascular diseases	United States	2010	Age-standardized		7.2% (5.4 to 9.1%)	13.0 (9.7 to 16.3)
Cardiovascular diseases	United States	2015	Age-standardized		7.1% (5.3 to 9.0%)	12.4 (9.3 to 15.8)
Ischemic heart disease	United States	1990	All Ages	66.3 (48.8 to 83.8)	11.2% (8.2 to 14.2%)	26.5 (19.5 to 33.5)
Ischemic heart disease	United States	1995	All Ages	64.4 (46.3 to 83.0)	10.9% (7.8 to 13.9%)	24.2 (17.4 to 31.2)
Ischemic heart disease	United States	2000	All Ages	61.1 (43.0 to 79.7)	10.7% (7.5 to 13.8%)	21.7 (15.2 to 28.2)
Ischemic heart disease	United States	2005	All Ages	55.6 (38.6 to 72.6)	10.6% (7.4 to 13.8%)	18.8 (13.1 to 24.5)
Ischemic heart disease	United States	2010	All Ages	46.6 (31.5 to 62.1)	9.5% (6.4 to 12.7%)	15.1 (10.2 to 20.0)
Ischemic heart disease	United States	2015	All Ages	49.6 (33.3 to 66.7)	9.3% (6.3 to 12.6%)	15.3 (10.3 to 20.6)
Ischemic heart disease	United States	1990	Age-standardized		11.5% (8.6 to 14.3%)	22.7 (17.0 to 28.5)
Ischemic heart disease	United States	1995	Age-standardized		11.2% (8.3 to 14.1%)	20.0 (14.8 to 25.2)
Ischemic heart disease	United States	2000	Age-standardized		11.0% (8.1 to 14.0%)	17.3 (12.7 to 22.1)
Ischemic heart disease	United States	2005	Age-standardized		10.9% (8.0 to 13.9%)	14.3 (10.4 to 18.2)
Ischemic heart disease	United States	2010	Age-standardized		9.8% (7.0 to 12.6%)	10.8 (7.6 to 14.0)
Ischemic heart disease	United States	2015	Age-standardized		9.6% (6.8 to 12.5%)	10.4 (7.3 to 13.5)
Ischemic stroke	United States	1990	All Ages	7.5 (4.1 to 11.9)	7.2% (4.0 to 11.3%)	3.0 (1.7 to 4.7)
Ischemic stroke	United States	1995	All Ages	7.8 (4.1 to 12.6)	7.0% (3.6 to 11.3%)	2.9 (1.5 to 4.7)
Ischemic stroke	United States	2000	All Ages	8.3 (4.2 to 13.6)	6.9% (3.5 to 11.3%)	2.9 (1.5 to 4.8)
Ischemic stroke	United States	2005	All Ages	7.6 (3.8 to 12.6)	6.8% (3.4 to 11.4%)	2.6 (1.3 to 4.3)
Ischemic stroke	United States	2010	All Ages	6.2 (2.9 to 10.8)	5.9% (2.7 to 10.5%)	2.0 (0.9 to 3.5)
Ischemic stroke	United States	2015	All Ages	6.5 (2.9 to 11.5)	5.8% (2.6 to 10.4%)	2.0 (0.9 to 3.6)
Ischemic stroke	United States	1990	Age-standardized		7.3% (4.1 to 11.3%)	2.5 (1.4 to 3.8)
Ischemic stroke	United States	1995	Age-standardized		7.1% (3.8 to 11.2%)	2.3 (1.2 to 3.6)
Ischemic stroke	United States	2000	Age-standardized		7.0% (3.7 to 11.2%)	2.2 (1.2 to 3.5)
Ischemic stroke	United States	2005	Age-standardized		6.9% (3.6 to 11.2%)	1.8 (1.0 to 3.0)
Ischemic stroke	United States	2010	Age-standardized		6.0% (3.0 to 10.2%)	1.4 (0.7 to 2.3)

Cause name	Location	Year	Age group	Number (in thousands)	PAF	Rate (per 100,000)
Ischemic stroke	United States	2015	Age-standardized		5.9% (2.9 to 10.1%)	1.3 (0.6 to 2.2)
Hemorrhagic stroke	United States	1990	All Ages	4.2 (3.0 to 5.5)	8.7% (6.3 to 11.4%)	1.7 (1.2 to 2.2)
Hemorrhagic stroke	United States	1995	All Ages	4.4 (3.1 to 5.8)	8.4% (5.9 to 11.2%)	1.6 (1.1 to 2.2)
Hemorrhagic stroke	United States	2000	All Ages	4.4 (3.0 to 5.9)	8.3% (5.7 to 11.2%)	1.6 (1.1 to 2.1)
Hemorrhagic stroke	United States	2005	All Ages	4.2 (2.9 to 5.7)	8.2% (5.6 to 11.2%)	1.4 (1.0 to 1.9)
Hemorrhagic stroke	United States	2010	All Ages	3.5 (2.3 to 5.0)	7.1% (4.6 to 10.1%)	1.1 (0.7 to 1.6)
Hemorrhagic stroke	United States	2015	All Ages	3.7 (2.3 to 5.3)	6.9% (4.4 to 9.9%)	1.1 (0.7 to 1.6)
Hemorrhagic stroke	United States	1990	Age-standardized		8.9% (6.4 to 11.6%)	1.5 (1.0 to 1.9)
Hemorrhagic stroke	United States	1995	Age-standardized		8.6% (6.1 to 11.3%)	1.4 (1.0 to 1.9)
Hemorrhagic stroke	United States	2000	Age-standardized		8.4% (5.9 to 11.2%)	1.3 (0.9 to 1.7)
Hemorrhagic stroke	United States	2005	Age-standardized		8.3% (5.8 to 11.1%)	1.1 (0.8 to 1.5)
Hemorrhagic stroke	United States	2010	Age-standardized		7.3% (4.9 to 10.0%)	0.9 (0.6 to 1.2)
Hemorrhagic stroke	United States	2015	Age-standardized		7.1% (4.7 to 9.9%)	0.8 (0.5 to 1.1)
Chronic obstructive pulmonary disease	United States	1990	All Ages	10.1 (3.5 to 18.5)	12.0% (4.2 to 21.7%)	4.1 (1.4 to 7.4)
Chronic obstructive pulmonary disease	United States	1995	All Ages	11.9 (4.1 to 21.8)	11.6% (4.0 to 21.1%)	4.5 (1.5 to 8.2)
Chronic obstructive pulmonary disease	United States	2000	All Ages	13.8 (4.7 to 25.3)	11.4% (3.9 to 20.9%)	4.9 (1.7 to 9.0)
Chronic obstructive pulmonary disease	United States	2005	All Ages	14.6 (4.9 to 27.1)	11.2% (3.8 to 20.7%)	4.9 (1.7 to 9.2)
Chronic obstructive pulmonary disease	United States	2010	All Ages	13.0 (3.8 to 25.7)	9.2% (2.8 to 18.3%)	4.2 (1.2 to 8.3)
Chronic obstructive pulmonary disease	United States	2015	All Ages	13.8 (3.9 to 27.7)	9.0% (2.6 to 17.9%)	4.3 (1.2 to 8.6)
Chronic obstructive pulmonary disease	United States	1990	Age-standardized		12.0% (4.2 to 21.7%)	3.4 (1.2 to 6.2)
Chronic obstructive pulmonary disease	United States	1995	Age-standardized		11.6% (4.0 to 21.1%)	3.6 (1.2 to 6.6)
Chronic obstructive pulmonary disease	United States	2000	Age-standardized		11.4% (3.9 to 20.9%)	3.9 (1.3 to 7.1)
Chronic obstructive pulmonary disease	United States	2005	Age-standardized		11.2% (3.8 to 20.6%)	3.8 (1.3 to 7.0)
Chronic obstructive pulmonary disease	United States	2010	Age-standardized		9.2% (2.8 to 18.2%)	3.0 (0.9 to 5.9)
Chronic obstructive pulmonary disease	United States	2015	Age-standardized		9.0% (2.6 to 17.9%)	2.9 (0.8 to 5.8)
All causes	Australia	1990	All Ages	4.0 (3.0 to 5.3)	3.4% (2.5 to 4.4%)	23.8 (17.3 to 31.0)
All causes	Australia	1995	All Ages	4.0 (2.8 to 5.4)	3.2% (2.2 to 4.3%)	22.1 (15.6 to 29.6)
All causes	Australia	2000	All Ages	3.8 (2.6 to 5.1)	2.9% (2.0 to 4.0%)	19.9 (13.8 to 26.8)
All causes	Australia	2005	All Ages	3.5 (2.3 to 4.9)	2.6% (1.7 to 3.6%)	17.1 (11.4 to 24.0)
All causes	Australia	2010	All Ages	3.0 (1.8 to 4.4)	2.1% (1.3 to 3.0%)	13.5 (8.2 to 19.8)
All causes	Australia	2015	All Ages	3.4 (2.0 to 5.0)	2.0% (1.2 to 3.1%)	13.8 (8.2 to 20.7)
All causes	Australia	1990	Age-standardized		3.3% (2.4 to 4.4%)	22.7 (16.4 to 29.7)
All causes	Australia	1995	Age-standardized		3.1% (2.2 to 4.2%)	19.2 (13.5 to 25.8)
All causes	Australia	2000	Age-standardized		2.9% (2.0 to 3.8%)	15.8 (11.1 to 21.1)
All causes	Australia	2005	Age-standardized		2.5% (1.7 to 3.5%)	12.3 (8.4 to 16.9)
All causes	Australia	2010	Age-standardized		2.0% (1.3 to 2.8%)	9.1 (5.8 to 13.0)

Cause name	Location	Year	Age group	Number (in thousands)	PAF	Rate (per 100,000)
All causes	Australia	2015	Age-standardized		2.0% (1.2 to 2.9%)	8.9 (5.5 to 12.9)
Lower respiratory infections	Australia	1990	All Ages	0.1 (0.0 to 0.3)	4.8% (0.6 to 14.8%)	0.6 (0.1 to 1.9)
Lower respiratory infections	Australia	1995	All Ages	0.1 (0.0 to 0.4)	4.8% (0.5 to 14.9%)	0.7 (0.1 to 2.1)
Lower respiratory infections	Australia	2000	All Ages	0.2 (0.0 to 0.5)	4.9% (0.6 to 14.9%)	0.9 (0.1 to 2.6)
Lower respiratory infections	Australia	2005	All Ages	0.2 (0.0 to 0.5)	4.7% (0.5 to 14.6%)	0.9 (0.1 to 2.7)
Lower respiratory infections	Australia	2010	All Ages	0.1 (0.0 to 0.5)	3.7% (0.3 to 13.5%)	0.7 (0.1 to 2.3)
Lower respiratory infections	Australia	2015	All Ages	0.2 (0.0 to 0.6)	3.7% (0.3 to 13.3%)	0.7 (0.1 to 2.4)
Lower respiratory infections	Australia	1990	Age-standardized		4.8% (0.6 to 14.8%)	0.6 (0.1 to 1.9)
Lower respiratory infections	Australia	1995	Age-standardized		4.8% (0.5 to 14.9%)	0.6 (0.1 to 1.8)
Lower respiratory infections	Australia	2000	Age-standardized		4.9% (0.6 to 14.9%)	0.7 (0.1 to 2.1)
Lower respiratory infections	Australia	2005	Age-standardized		4.7% (0.5 to 14.6%)	0.6 (0.1 to 1.9)
Lower respiratory infections	Australia	2010	Age-standardized		3.7% (0.3 to 13.5%)	0.4 (0.0 to 1.5)
Lower respiratory infections	Australia	2015	Age-standardized		3.7% (0.3 to 13.3%)	0.4 (0.0 to 1.5)
Tracheal, bronchus, and lung cancer	Australia	1990	All Ages	0.2 (0.1 to 0.4)	3.1% (1.0 to 6.5%)	1.1 (0.3 to 2.4)
Tracheal, bronchus, and lung cancer	Australia	1995	All Ages	0.2 (0.1 to 0.4)	3.1% (1.0 to 6.6%)	1.2 (0.4 to 2.5)
Tracheal, bronchus, and lung cancer	Australia	2000	All Ages	0.2 (0.1 to 0.5)	3.2% (1.0 to 6.7%)	1.2 (0.4 to 2.4)
Tracheal, bronchus, and lung cancer	Australia	2005	All Ages	0.2 (0.1 to 0.5)	3.0% (0.9 to 6.4%)	1.1 (0.3 to 2.3)
Tracheal, bronchus, and lung cancer	Australia	2010	All Ages	0.2 (0.1 to 0.5)	2.4% (0.6 to 5.6%)	0.9 (0.2 to 2.0)
Tracheal, bronchus, and lung cancer	Australia	2015	All Ages	0.2 (0.1 to 0.5)	2.3% (0.6 to 5.5%)	0.9 (0.2 to 2.0)
Tracheal, bronchus, and lung cancer	Australia	1990	Age-standardized		3.1% (1.0 to 6.5%)	1.1 (0.3 to 2.3)
Tracheal, bronchus, and lung cancer	Australia	1995	Age-standardized		3.1% (1.0 to 6.6%)	1.0 (0.3 to 2.2)
Tracheal, bronchus, and lung cancer	Australia	2000	Age-standardized		3.2% (1.0 to 6.7%)	1.0 (0.3 to 2.0)
Tracheal, bronchus, and lung cancer	Australia	2005	Age-standardized		3.0% (0.9 to 6.4%)	0.8 (0.3 to 1.8)
Tracheal, bronchus, and lung cancer	Australia	2010	Age-standardized		2.4% (0.6 to 5.6%)	0.6 (0.2 to 1.5)
Tracheal, bronchus, and lung cancer	Australia	2015	Age-standardized		2.3% (0.6 to 5.5%)	0.6 (0.1 to 1.5)
Cardiovascular diseases	Australia	1990	All Ages	3.3 (2.4 to 4.4)	6.2% (4.4 to 8.1%)	19.5 (14.0 to 25.6)
Cardiovascular diseases	Australia	1995	All Ages	3.2 (2.2 to 4.3)	6.0% (4.1 to 8.0%)	17.6 (12.0 to 23.6)
Cardiovascular diseases	Australia	2000	All Ages	3.0 (2.0 to 4.0)	5.9% (3.9 to 8.1%)	15.4 (10.3 to 21.1)
Cardiovascular diseases	Australia	2005	All Ages	2.6 (1.7 to 3.8)	5.5% (3.5 to 7.9%)	13.0 (8.3 to 18.6)
Cardiovascular diseases	Australia	2010	All Ages	2.3 (1.3 to 3.5)	4.6% (2.6 to 7.2%)	10.2 (5.7 to 15.8)
Cardiovascular diseases	Australia	2015	All Ages	2.5 (1.4 to 4.0)	4.5% (2.5 to 7.1%)	10.4 (5.8 to 16.3)
Cardiovascular diseases	Australia	1990	Age-standardized		6.2% (4.4 to 8.2%)	18.7 (13.2 to 24.6)
Cardiovascular diseases	Australia	1995	Age-standardized		6.1% (4.2 to 8.1%)	15.4 (10.5 to 20.5)
Cardiovascular diseases	Australia	2000	Age-standardized		6.0% (4.1 to 8.1%)	12.2 (8.4 to 16.6)
Cardiovascular diseases	Australia	2005	Age-standardized		5.6% (3.8 to 7.8%)	9.3 (6.2 to 12.8)
Cardiovascular diseases	Australia	2010	Age-standardized		4.7% (2.9 to 7.0%)	6.9 (4.2 to 10.1)

Cause name	Location	Year	Age group	Number (in thousands)	PAF	Rate (per 100,000)
Cardiovascular diseases	Australia	2015	Age-standardized		4.6% (2.8 to 6.9%)	6.7 (4.0 to 9.9)
Ischemic heart disease	Australia	1990	All Ages	2.7 (1.8 to 3.7)	7.9% (5.3 to 10.7%)	15.8 (10.7 to 21.5)
Ischemic heart disease	Australia	1995	All Ages	2.6 (1.7 to 3.6)	7.7% (5.0 to 10.7%)	14.1 (9.2 to 19.7)
Ischemic heart disease	Australia	2000	All Ages	2.3 (1.4 to 3.3)	7.6% (4.7 to 10.9%)	12.1 (7.5 to 17.2)
Ischemic heart disease	Australia	2005	All Ages	2.0 (1.2 to 3.0)	7.3% (4.2 to 10.8%)	10.0 (5.8 to 14.9)
Ischemic heart disease	Australia	2010	All Ages	1.7 (0.9 to 2.8)	6.2% (3.3 to 9.9%)	7.8 (4.1 to 12.6)
Ischemic heart disease	Australia	2015	All Ages	2.0 (1.0 to 3.2)	6.1% (3.1 to 10.0%)	8.0 (4.0 to 13.1)
Ischemic heart disease	Australia	1990	Age-standardized		7.8% (5.3 to 10.7%)	15.1 (10.2 to 20.7)
Ischemic heart disease	Australia	1995	Age-standardized		7.7% (5.1 to 10.7%)	12.3 (8.1 to 17.1)
Ischemic heart disease	Australia	2000	Age-standardized		7.7% (4.9 to 10.9%)	9.6 (6.1 to 13.5)
Ischemic heart disease	Australia	2005	Age-standardized		7.4% (4.6 to 10.7%)	7.2 (4.4 to 10.4)
Ischemic heart disease	Australia	2010	Age-standardized		6.4% (3.6 to 9.9%)	5.3 (3.0 to 8.2)
Ischemic heart disease	Australia	2015	Age-standardized		6.3% (3.4 to 9.9%)	5.2 (2.8 to 8.1)
Ischemic stroke	Australia	1990	All Ages	0.3 (0.1 to 0.6)	4.8% (2.1 to 8.7%)	1.8 (0.8 to 3.4)
Ischemic stroke	Australia	1995	All Ages	0.3 (0.1 to 0.6)	4.7% (1.9 to 8.8%)	1.7 (0.7 to 3.3)
Ischemic stroke	Australia	2000	All Ages	0.3 (0.1 to 0.6)	4.7% (1.8 to 9.0%)	1.6 (0.6 to 3.1)
Ischemic stroke	Australia	2005	All Ages	0.3 (0.1 to 0.6)	4.5% (1.6 to 9.1%)	1.4 (0.5 to 3.0)
Ischemic stroke	Australia	2010	All Ages	0.3 (0.1 to 0.6)	3.8% (1.1 to 8.6%)	1.2 (0.3 to 2.6)
Ischemic stroke	Australia	2015	All Ages	0.3 (0.1 to 0.7)	3.8% (1.0 to 8.7%)	1.1 (0.3 to 2.7)
Ischemic stroke	Australia	1990	Age-standardized		4.7% (2.0 to 8.7%)	1.8 (0.8 to 3.3)
Ischemic stroke	Australia	1995	Age-standardized		4.7% (1.9 to 8.8%)	1.5 (0.6 to 2.8)
Ischemic stroke	Australia	2000	Age-standardized		4.7% (1.9 to 9.0%)	1.2 (0.5 to 2.4)
Ischemic stroke	Australia	2005	Age-standardized		4.5% (1.7 to 9.0%)	1.0 (0.4 to 2.0)
Ischemic stroke	Australia	2010	Age-standardized		3.9% (1.3 to 8.4%)	0.7 (0.2 to 1.6)
Ischemic stroke	Australia	2015	Age-standardized		3.8% (1.2 to 8.4%)	0.7 (0.2 to 1.5)
Hemorrhagic stroke	Australia	1990	All Ages	0.3 (0.2 to 0.5)	5.2% (2.8 to 8.2%)	1.9 (1.0 to 3.0)
Hemorrhagic stroke	Australia	1995	All Ages	0.3 (0.2 to 0.5)	5.1% (2.6 to 8.5%)	1.8 (0.9 to 2.9)
Hemorrhagic stroke	Australia	2000	All Ages	0.3 (0.2 to 0.6)	5.1% (2.5 to 8.7%)	1.7 (0.8 to 2.9)
Hemorrhagic stroke	Australia	2005	All Ages	0.3 (0.1 to 0.6)	4.8% (2.2 to 8.8%)	1.5 (0.7 to 2.9)
Hemorrhagic stroke	Australia	2010	All Ages	0.3 (0.1 to 0.6)	4.1% (1.6 to 8.2%)	1.2 (0.5 to 2.5)
Hemorrhagic stroke	Australia	2015	All Ages	0.3 (0.1 to 0.6)	4.0% (1.5 to 8.3%)	1.2 (0.5 to 2.7)
Hemorrhagic stroke	Australia	1990	Age-standardized		5.2% (2.8 to 8.3%)	1.8 (1.0 to 2.9)
Hemorrhagic stroke	Australia	1995	Age-standardized		5.1% (2.7 to 8.5%)	1.5 (0.8 to 2.6)
Hemorrhagic stroke	Australia	2000	Age-standardized		5.1% (2.6 to 8.7%)	1.4 (0.7 to 2.3)
Hemorrhagic stroke	Australia	2005	Age-standardized		4.9% (2.4 to 8.6%)	1.1 (0.5 to 1.9)
Hemorrhagic stroke	Australia	2010	Age-standardized		4.2% (1.9 to 7.9%)	0.8 (0.4 to 1.6)

Cause name	Location	Year	Age group	Number (in thousands)	PAF	Rate (per 100,000)
Hemorrhagic stroke	Australia	2015	Age-standardized		4.1% (1.8 to 8.0%)	0.8 (0.3 to 1.6)
Chronic obstructive pulmonary disease	Australia	1990	All Ages	0.4 (0.1 to 1.0)	6.7% (1.4 to 15.2%)	2.5 (0.5 to 5.7)
Chronic obstructive pulmonary disease	Australia	1995	All Ages	0.5 (0.1 to 1.1)	6.8% (1.3 to 15.3%)	2.6 (0.5 to 5.8)
Chronic obstructive pulmonary disease	Australia	2000	All Ages	0.5 (0.1 to 1.1)	6.9% (1.5 to 15.4%)	2.5 (0.5 to 5.6)
Chronic obstructive pulmonary disease	Australia	2005	All Ages	0.4 (0.1 to 1.0)	6.6% (1.3 to 15.1%)	2.2 (0.4 to 5.0)
Chronic obstructive pulmonary disease	Australia	2010	All Ages	0.4 (0.1 to 1.0)	5.4% (0.8 to 13.7%)	1.8 (0.3 to 4.4)
Chronic obstructive pulmonary disease	Australia	2015	All Ages	0.5 (0.1 to 1.2)	5.4% (0.9 to 13.5%)	1.9 (0.3 to 4.7)
Chronic obstructive pulmonary disease	Australia	1990	Age-standardized		6.7% (1.4 to 15.2%)	2.4 (0.5 to 5.4)
Chronic obstructive pulmonary disease	Australia	1995	Age-standardized		6.8% (1.3 to 15.3%)	2.2 (0.4 to 5.0)
Chronic obstructive pulmonary disease	Australia	2000	Age-standardized		6.9% (1.5 to 15.4%)	1.9 (0.4 to 4.4)
Chronic obstructive pulmonary disease	Australia	2005	Age-standardized		6.6% (1.3 to 15.1%)	1.6 (0.3 to 3.6)
Chronic obstructive pulmonary disease	Australia	2010	Age-standardized		5.4% (0.8 to 13.6%)	1.2 (0.2 to 3.0)
Chronic obstructive pulmonary disease	Australia	2015	Age-standardized		5.4% (0.9 to 13.5%)	1.2 (0.2 to 3.1)
All causes	New Zealand	1990	All Ages	0.8 (0.6 to 1.1)	3.1% (2.2 to 4.0%)	23.9 (17.0 to 31.3)
All causes	New Zealand	1995	All Ages	0.8 (0.6 to 1.1)	2.9% (2.0 to 3.9%)	21.6 (15.1 to 29.0)
All causes	New Zealand	2000	All Ages	0.8 (0.5 to 1.0)	2.7% (1.8 to 3.7%)	19.4 (13.0 to 26.7)
All causes	New Zealand	2005	All Ages	0.7 (0.5 to 1.0)	2.4% (1.6 to 3.5%)	16.8 (10.8 to 23.9)
All causes	New Zealand	2010	All Ages	0.6 (0.3 to 0.9)	1.8% (1.1 to 2.9%)	12.7 (7.3 to 19.7)
All causes	New Zealand	2015	All Ages	0.6 (0.4 to 0.9)	1.9% (1.1 to 2.9%)	13.4 (7.7 to 20.6)
All causes	New Zealand	1990	Age-standardized		3.1% (2.2 to 4.0%)	22.8 (16.3 to 30.0)
All causes	New Zealand	1995	Age-standardized		2.9% (2.0 to 3.8%)	19.5 (13.7 to 25.8)
All causes	New Zealand	2000	Age-standardized		2.7% (1.8 to 3.6%)	16.3 (11.1 to 22.3)
All causes	New Zealand	2005	Age-standardized		2.4% (1.6 to 3.4%)	13.2 (8.6 to 18.4)
All causes	New Zealand	2010	Age-standardized		1.8% (1.1 to 2.7%)	9.1 (5.4 to 13.9)
All causes	New Zealand	2015	Age-standardized		1.9% (1.1 to 2.8%)	9.0 (5.4 to 13.5)
Lower respiratory infections	New Zealand	1990	All Ages	0.0 (0.0 to 0.1)	4.2% (0.4 to 13.9%)	1.3 (0.1 to 4.3)
Lower respiratory infections	New Zealand	1995	All Ages	0.0 (0.0 to 0.1)	4.2% (0.4 to 13.8%)	1.1 (0.1 to 3.4)
Lower respiratory infections	New Zealand	2000	All Ages	0.0 (0.0 to 0.1)	4.1% (0.4 to 13.8%)	0.7 (0.1 to 2.2)
Lower respiratory infections	New Zealand	2005	All Ages	0.0 (0.0 to 0.1)	4.0% (0.3 to 13.6%)	0.6 (0.0 to 1.9)
Lower respiratory infections	New Zealand	2010	All Ages	0.0 (0.0 to 0.1)	3.0% (0.2 to 11.6%)	0.5 (0.0 to 1.7)
Lower respiratory infections	New Zealand	2015	All Ages	0.0 (0.0 to 0.1)	3.2% (0.2 to 12.2%)	0.5 (0.0 to 1.9)
Lower respiratory infections	New Zealand	1990	Age-standardized		4.2% (0.4 to 13.9%)	1.3 (0.1 to 4.2)
Lower respiratory infections	New Zealand	1995	Age-standardized		4.2% (0.4 to 13.8%)	0.9 (0.1 to 3.0)
Lower respiratory infections	New Zealand	2000	Age-standardized		4.1% (0.4 to 13.8%)	0.5 (0.0 to 1.8)
Lower respiratory infections	New Zealand	2005	Age-standardized		4.0% (0.3 to 13.6%)	0.4 (0.0 to 1.4)
Lower respiratory infections	New Zealand	2010	Age-standardized		3.0% (0.2 to 11.6%)	0.3 (0.0 to 1.2)

Cause name	Location	Year	Age group	Number (in thousands)	PAF	Rate (per 100,000)
Lower respiratory infections	New Zealand	2015	Age-standardized		3.2% (0.2 to 12.2%)	0.3 (0.0 to 1.2)
Tracheal, bronchus, and lung cancer	New Zealand	1990	All Ages	0.0 (0.0 to 0.1)	2.7% (0.7 to 6.1%)	1.0 (0.3 to 2.4)
Tracheal, bronchus, and lung cancer	New Zealand	1995	All Ages	0.0 (0.0 to 0.1)	2.7% (0.7 to 6.1%)	1.0 (0.3 to 2.3)
Tracheal, bronchus, and lung cancer	New Zealand	2000	All Ages	0.0 (0.0 to 0.1)	2.6% (0.7 to 6.0%)	1.0 (0.3 to 2.3)
Tracheal, bronchus, and lung cancer	New Zealand	2005	All Ages	0.0 (0.0 to 0.1)	2.5% (0.6 to 5.8%)	0.9 (0.2 to 2.1)
Tracheal, bronchus, and lung cancer	New Zealand	2010	All Ages	0.0 (0.0 to 0.1)	1.9% (0.4 to 4.9%)	0.7 (0.1 to 1.8)
Tracheal, bronchus, and lung cancer	New Zealand	2015	All Ages	0.0 (0.0 to 0.1)	2.0% (0.4 to 5.1%)	0.7 (0.2 to 1.9)
Tracheal, bronchus, and lung cancer	New Zealand	1990	Age-standardized		2.7% (0.7 to 6.1%)	1.0 (0.3 to 2.3)
Tracheal, bronchus, and lung cancer	New Zealand	1995	Age-standardized		2.7% (0.7 to 6.1%)	0.9 (0.3 to 2.2)
Tracheal, bronchus, and lung cancer	New Zealand	2000	Age-standardized		2.6% (0.7 to 6.0%)	0.9 (0.2 to 2.0)
Tracheal, bronchus, and lung cancer	New Zealand	2005	Age-standardized		2.5% (0.6 to 5.8%)	0.8 (0.2 to 1.8)
Tracheal, bronchus, and lung cancer	New Zealand	2010	Age-standardized		1.9% (0.4 to 4.9%)	0.5 (0.1 to 1.4)
Tracheal, bronchus, and lung cancer	New Zealand	2015	Age-standardized		2.0% (0.4 to 5.1%)	0.5 (0.1 to 1.4)
Cardiovascular diseases	New Zealand	1990	All Ages	0.6 (0.5 to 0.8)	5.7% (4.0 to 7.5%)	19.1 (13.2 to 25.2)
Cardiovascular diseases	New Zealand	1995	All Ages	0.6 (0.4 to 0.8)	5.5% (3.7 to 7.3%)	17.0 (11.5 to 23.0)
Cardiovascular diseases	New Zealand	2000	All Ages	0.6 (0.4 to 0.8)	5.2% (3.4 to 7.3%)	15.1 (9.8 to 20.9)
Cardiovascular diseases	New Zealand	2005	All Ages	0.5 (0.3 to 0.8)	4.9% (3.0 to 7.1%)	12.9 (8.0 to 18.7)
Cardiovascular diseases	New Zealand	2010	All Ages	0.4 (0.2 to 0.7)	3.9% (2.1 to 6.3%)	9.7 (5.3 to 15.6)
Cardiovascular diseases	New Zealand	2015	All Ages	0.5 (0.3 to 0.8)	4.1% (2.2 to 6.6%)	10.2 (5.5 to 16.5)
Cardiovascular diseases	New Zealand	1990	Age-standardized		5.7% (4.0 to 7.5%)	18.2 (12.6 to 24.0)
Cardiovascular diseases	New Zealand	1995	Age-standardized		5.5% (3.8 to 7.4%)	15.3 (10.5 to 20.7)
Cardiovascular diseases	New Zealand	2000	Age-standardized		5.3% (3.5 to 7.2%)	12.7 (8.5 to 17.3)
Cardiovascular diseases	New Zealand	2005	Age-standardized		5.0% (3.2 to 7.1%)	10.1 (6.4 to 14.3)
Cardiovascular diseases	New Zealand	2010	Age-standardized		4.0% (2.3 to 6.2%)	7.0 (4.0 to 10.8)
Cardiovascular diseases	New Zealand	2015	Age-standardized		4.1% (2.4 to 6.3%)	6.8 (3.9 to 10.4)
Ischemic heart disease	New Zealand	1990	All Ages	0.5 (0.3 to 0.7)	7.3% (4.8 to 10.1%)	15.6 (10.2 to 21.3)
Ischemic heart disease	New Zealand	1995	All Ages	0.5 (0.3 to 0.7)	7.2% (4.6 to 10.0%)	13.7 (8.8 to 19.3)
Ischemic heart disease	New Zealand	2000	All Ages	0.5 (0.3 to 0.7)	6.9% (4.3 to 10.0%)	12.0 (7.4 to 17.4)
Ischemic heart disease	New Zealand	2005	All Ages	0.4 (0.2 to 0.6)	6.6% (3.8 to 10.0%)	10.1 (5.8 to 15.3)
Ischemic heart disease	New Zealand	2010	All Ages	0.3 (0.2 to 0.6)	5.4% (2.6 to 9.0%)	7.6 (3.7 to 12.7)
Ischemic heart disease	New Zealand	2015	All Ages	0.4 (0.2 to 0.6)	5.6% (2.8 to 9.3%)	8.0 (4.0 to 13.7)
Ischemic heart disease	New Zealand	1990	Age-standardized		7.4% (4.8 to 10.1%)	14.9 (9.8 to 20.4)
Ischemic heart disease	New Zealand	1995	Age-standardized		7.2% (4.7 to 10.1%)	12.4 (8.1 to 17.4)
Ischemic heart disease	New Zealand	2000	Age-standardized		7.0% (4.4 to 10.0%)	10.1 (6.3 to 14.5)
Ischemic heart disease	New Zealand	2005	Age-standardized		6.7% (4.1 to 10.0%)	7.9 (4.8 to 11.8)
Ischemic heart disease	New Zealand	2010	Age-standardized		5.5% (2.9 to 8.8%)	5.5 (2.9 to 8.9)

Cause name	Location	Year	Age group	Number (in thousands)	PAF	Rate (per 100,000)
Ischemic heart disease	New Zealand	2015	Age-standardized		5.7% (3.0 to 9.2%)	5.4 (2.9 to 8.8)
Ischemic stroke	New Zealand	1990	All Ages	0.1 (0.0 to 0.1)	4.4% (1.9 to 7.9%)	1.6 (0.7 to 2.8)
Ischemic stroke	New Zealand	1995	All Ages	0.1 (0.0 to 0.1)	4.3% (1.8 to 8.3%)	1.5 (0.6 to 2.8)
Ischemic stroke	New Zealand	2000	All Ages	0.1 (0.0 to 0.1)	4.2% (1.6 to 8.4%)	1.4 (0.6 to 2.9)
Ischemic stroke	New Zealand	2005	All Ages	0.1 (0.0 to 0.1)	4.0% (1.4 to 8.5%)	1.3 (0.4 to 2.7)
Ischemic stroke	New Zealand	2010	All Ages	0.0 (0.0 to 0.1)	3.3% (0.9 to 7.8%)	1.0 (0.3 to 2.4)
Ischemic stroke	New Zealand	2015	All Ages	0.1 (0.0 to 0.1)	3.4% (0.9 to 8.1%)	1.0 (0.3 to 2.4)
Ischemic stroke	New Zealand	1990	Age-standardized		4.3% (1.9 to 8.0%)	1.5 (0.6 to 2.8)
Ischemic stroke	New Zealand	1995	Age-standardized		4.3% (1.8 to 8.3%)	1.3 (0.5 to 2.5)
Ischemic stroke	New Zealand	2000	Age-standardized		4.2% (1.7 to 8.3%)	1.2 (0.5 to 2.3)
Ischemic stroke	New Zealand	2005	Age-standardized		4.1% (1.5 to 8.4%)	1.0 (0.4 to 2.0)
Ischemic stroke	New Zealand	2010	Age-standardized		3.3% (1.0 to 7.7%)	0.7 (0.2 to 1.6)
Ischemic stroke	New Zealand	2015	Age-standardized		3.4% (1.1 to 7.9%)	0.6 (0.2 to 1.5)
Hemorrhagic stroke	New Zealand	1990	All Ages	0.1 (0.0 to 0.1)	4.8% (2.5 to 7.6%)	1.9 (1.0 to 3.1)
Hemorrhagic stroke	New Zealand	1995	All Ages	0.1 (0.0 to 0.1)	4.6% (2.4 to 7.8%)	1.8 (0.9 to 3.1)
Hemorrhagic stroke	New Zealand	2000	All Ages	0.1 (0.0 to 0.1)	4.5% (2.3 to 8.1%)	1.7 (0.8 to 2.9)
Hemorrhagic stroke	New Zealand	2005	All Ages	0.1 (0.0 to 0.1)	4.3% (2.0 to 8.1%)	1.5 (0.7 to 2.8)
Hemorrhagic stroke	New Zealand	2010	All Ages	0.1 (0.0 to 0.1)	3.5% (1.4 to 7.4%)	1.1 (0.4 to 2.4)
Hemorrhagic stroke	New Zealand	2015	All Ages	0.1 (0.0 to 0.1)	3.6% (1.4 to 7.7%)	1.1 (0.4 to 2.5)
Hemorrhagic stroke	New Zealand	1990	Age-standardized		4.8% (2.5 to 7.7%)	1.8 (1.0 to 3.0)
Hemorrhagic stroke	New Zealand	1995	Age-standardized		4.7% (2.5 to 7.8%)	1.6 (0.8 to 2.7)
Hemorrhagic stroke	New Zealand	2000	Age-standardized		4.6% (2.4 to 7.9%)	1.4 (0.7 to 2.4)
Hemorrhagic stroke	New Zealand	2005	Age-standardized		4.4% (2.1 to 8.0%)	1.2 (0.5 to 2.1)
Hemorrhagic stroke	New Zealand	2010	Age-standardized		3.6% (1.5 to 7.2%)	0.8 (0.3 to 1.7)
Hemorrhagic stroke	New Zealand	2015	Age-standardized		3.7% (1.6 to 7.4%)	0.8 (0.3 to 1.5)
Chronic obstructive pulmonary disease	New Zealand	1990	All Ages	0.1 (0.0 to 0.2)	5.9% (1.1 to 14.3%)	2.4 (0.4 to 5.8)
Chronic obstructive pulmonary disease	New Zealand	1995	All Ages	0.1 (0.0 to 0.2)	5.9% (1.1 to 14.2%)	2.6 (0.5 to 6.2)
Chronic obstructive pulmonary disease	New Zealand	2000	All Ages	0.1 (0.0 to 0.3)	5.9% (1.1 to 14.4%)	2.6 (0.5 to 6.4)
Chronic obstructive pulmonary disease	New Zealand	2005	All Ages	0.1 (0.0 to 0.3)	5.7% (1.0 to 13.9%)	2.4 (0.4 to 6.0)
Chronic obstructive pulmonary disease	New Zealand	2010	All Ages	0.1 (0.0 to 0.2)	4.4% (0.6 to 12.4%)	1.8 (0.2 to 5.1)
Chronic obstructive pulmonary disease	New Zealand	2015	All Ages	0.1 (0.0 to 0.2)	4.7% (0.7 to 12.6%)	2.0 (0.3 to 5.3)
Chronic obstructive pulmonary disease	New Zealand	1990	Age-standardized		5.9% (1.1 to 14.3%)	2.3 (0.4 to 5.5)
Chronic obstructive pulmonary disease	New Zealand	1995	Age-standardized		5.9% (1.1 to 14.2%)	2.3 (0.4 to 5.5)
Chronic obstructive pulmonary disease	New Zealand	2000	Age-standardized		5.9% (1.1 to 14.4%)	2.2 (0.4 to 5.3)
Chronic obstructive pulmonary disease	New Zealand	2005	Age-standardized		5.7% (1.0 to 13.9%)	1.9 (0.3 to 4.7)
Chronic obstructive pulmonary disease	New Zealand	2010	Age-standardized		4.4% (0.6 to 12.4%)	1.3 (0.2 to 3.7)

Cause name	Location	Year	Age group	Number (in thousands)	PAF	Rate (per 100,000)
Chronic obstructive pulmonary disease	New Zealand	2015	Age-standardized		4.7% (0.7 to 12.6%)	1.3 (0.2 to 3.5)
All causes	Brunei	1990	All Ages	0.0 (0.0 to 0.0)	2.0% (1.3 to 2.8%)	6.7 (4.3 to 9.4)
All causes	Brunei	1995	All Ages	0.0 (0.0 to 0.0)	2.1% (1.3 to 2.9%)	6.4 (4.1 to 8.8)
All causes	Brunei	2000	All Ages	0.0 (0.0 to 0.0)	1.9% (1.1 to 2.7%)	5.1 (3.1 to 7.3)
All causes	Brunei	2005	All Ages	0.0 (0.0 to 0.0)	2.7% (1.9 to 3.6%)	7.9 (5.4 to 10.4)
All causes	Brunei	2010	All Ages	0.0 (0.0 to 0.0)	2.3% (1.5 to 3.2%)	7.1 (4.6 to 9.9)
All causes	Brunei	2015	All Ages	0.0 (0.0 to 0.0)	1.6% (0.8 to 2.5%)	5.8 (2.9 to 9.1)
All causes	Brunei	1990	Age-standardized		2.4% (1.4 to 3.5%)	19.8 (12.2 to 28.7)
All causes	Brunei	1995	Age-standardized		2.4% (1.4 to 3.4%)	18.9 (11.4 to 27.2)
All causes	Brunei	2000	Age-standardized		2.2% (1.2 to 3.2%)	15.7 (8.7 to 23.4)
All causes	Brunei	2005	Age-standardized		3.0% (2.0 to 4.1%)	20.6 (13.7 to 28.5)
All causes	Brunei	2010	Age-standardized		2.4% (1.5 to 3.5%)	16.2 (10.1 to 23.3)
All causes	Brunei	2015	Age-standardized		1.6% (0.8 to 2.7%)	11.0 (5.1 to 17.7)
Lower respiratory infections	Brunei	1990	All Ages	0.0 (0.0 to 0.0)	3.7% (0.3 to 13.3%)	0.5 (0.0 to 1.7)
Lower respiratory infections	Brunei	1995	All Ages	0.0 (0.0 to 0.0)	3.7% (0.3 to 12.9%)	0.4 (0.0 to 1.5)
Lower respiratory infections	Brunei	2000	All Ages	0.0 (0.0 to 0.0)	3.4% (0.2 to 12.5%)	0.3 (0.0 to 1.2)
Lower respiratory infections	Brunei	2005	All Ages	0.0 (0.0 to 0.0)	5.6% (0.8 to 15.6%)	0.6 (0.1 to 1.8)
Lower respiratory infections	Brunei	2010	All Ages	0.0 (0.0 to 0.0)	4.3% (0.4 to 14.1%)	0.6 (0.1 to 1.8)
Lower respiratory infections	Brunei	2015	All Ages	0.0 (0.0 to 0.0)	2.7% (0.1 to 11.0%)	0.4 (0.0 to 1.7)
Lower respiratory infections	Brunei	1990	Age-standardized		3.7% (0.3 to 13.3%)	1.4 (0.1 to 4.6)
Lower respiratory infections	Brunei	1995	Age-standardized		3.7% (0.3 to 12.9%)	1.3 (0.1 to 4.3)
Lower respiratory infections	Brunei	2000	Age-standardized		3.4% (0.2 to 12.5%)	1.1 (0.1 to 3.9)
Lower respiratory infections	Brunei	2005	Age-standardized		5.6% (0.8 to 15.6%)	1.9 (0.3 to 5.2)
Lower respiratory infections	Brunei	2010	Age-standardized		4.3% (0.4 to 14.1%)	1.5 (0.1 to 5.0)
Lower respiratory infections	Brunei	2015	Age-standardized		2.7% (0.1 to 11.0%)	1.0 (0.0 to 3.9)
Tracheal, bronchus, and lung cancer	Brunei	1990	All Ages	0.0 (0.0 to 0.0)	2.3% (0.5 to 5.7%)	0.2 (0.1 to 0.5)
Tracheal, bronchus, and lung cancer	Brunei	1995	All Ages	0.0 (0.0 to 0.0)	2.3% (0.5 to 5.6%)	0.2 (0.0 to 0.5)
Tracheal, bronchus, and lung cancer	Brunei	2000	All Ages	0.0 (0.0 to 0.0)	2.1% (0.3 to 5.3%)	0.2 (0.0 to 0.5)
Tracheal, bronchus, and lung cancer	Brunei	2005	All Ages	0.0 (0.0 to 0.0)	3.6% (1.1 to 7.3%)	0.4 (0.1 to 0.8)
Tracheal, bronchus, and lung cancer	Brunei	2010	All Ages	0.0 (0.0 to 0.0)	2.7% (0.6 to 6.4%)	0.4 (0.1 to 0.8)
Tracheal, bronchus, and lung cancer	Brunei	2015	All Ages	0.0 (0.0 to 0.0)	1.7% (0.2 to 4.7%)	0.3 (0.0 to 0.8)
Tracheal, bronchus, and lung cancer	Brunei	1990	Age-standardized		2.3% (0.5 to 5.7%)	0.7 (0.2 to 1.7)
Tracheal, bronchus, and lung cancer	Brunei	1995	Age-standardized		2.3% (0.5 to 5.6%)	0.7 (0.1 to 1.6)
Tracheal, bronchus, and lung cancer	Brunei	2000	Age-standardized		2.2% (0.3 to 5.3%)	0.6 (0.1 to 1.5)
Tracheal, bronchus, and lung cancer	Brunei	2005	Age-standardized		3.6% (1.1 to 7.3%)	1.1 (0.3 to 2.1)
Tracheal, bronchus, and lung cancer	Brunei	2010	Age-standardized		2.7% (0.6 to 6.4%)	0.8 (0.2 to 1.9)

Cause name	Location	Year	Age group	Number (in thousands)	PAF	Rate (per 100,000)
Tracheal, bronchus, and lung cancer	Brunei	2015	Age-standardized		1.7% (0.2 to 4.7%)	0.5 (0.1 to 1.4)
Cardiovascular diseases	Brunei	1990	All Ages	0.0 (0.0 to 0.0)	5.5% (3.7 to 7.5%)	5.0 (3.3 to 6.8)
Cardiovascular diseases	Brunei	1995	All Ages	0.0 (0.0 to 0.0)	5.6% (3.8 to 7.5%)	4.9 (3.2 to 6.6)
Cardiovascular diseases	Brunei	2000	All Ages	0.0 (0.0 to 0.0)	5.3% (3.4 to 7.4%)	4.0 (2.5 to 5.5)
Cardiovascular diseases	Brunei	2005	All Ages	0.0 (0.0 to 0.0)	7.3% (5.2 to 9.3%)	5.9 (4.2 to 7.5)
Cardiovascular diseases	Brunei	2010	All Ages	0.0 (0.0 to 0.0)	6.1% (4.1 to 8.2%)	5.3 (3.6 to 7.1)
Cardiovascular diseases	Brunei	2015	All Ages	0.0 (0.0 to 0.0)	4.3% (2.4 to 6.5%)	4.5 (2.5 to 6.8)
Cardiovascular diseases	Brunei	1990	Age-standardized		5.0% (3.2 to 7.1%)	14.3 (9.0 to 20.4)
Cardiovascular diseases	Brunei	1995	Age-standardized		5.0% (3.1 to 6.9%)	13.8 (8.7 to 19.6)
Cardiovascular diseases	Brunei	2000	Age-standardized		4.6% (2.8 to 6.8%)	11.4 (6.8 to 16.7)
Cardiovascular diseases	Brunei	2005	Age-standardized		6.4% (4.4 to 8.5%)	14.3 (9.8 to 19.2)
Cardiovascular diseases	Brunei	2010	Age-standardized		5.4% (3.4 to 7.5%)	11.3 (7.2 to 15.8)
Cardiovascular diseases	Brunei	2015	Age-standardized		3.8% (1.9 to 6.1%)	7.9 (4.0 to 12.7)
Ischemic heart disease	Brunei	1990	All Ages	0.0 (0.0 to 0.0)	7.7% (4.9 to 10.7%)	3.5 (2.2 to 4.9)
Ischemic heart disease	Brunei	1995	All Ages	0.0 (0.0 to 0.0)	7.8% (4.9 to 10.7%)	3.5 (2.2 to 4.8)
Ischemic heart disease	Brunei	2000	All Ages	0.0 (0.0 to 0.0)	7.4% (4.3 to 10.6%)	2.8 (1.6 to 4.1)
Ischemic heart disease	Brunei	2005	All Ages	0.0 (0.0 to 0.0)	10.0% (6.9 to 13.0%)	4.2 (2.9 to 5.5)
Ischemic heart disease	Brunei	2010	All Ages	0.0 (0.0 to 0.0)	8.4% (5.5 to 11.5%)	3.9 (2.5 to 5.3)
Ischemic heart disease	Brunei	2015	All Ages	0.0 (0.0 to 0.0)	5.9% (2.9 to 9.2%)	3.3 (1.6 to 5.2)
Ischemic heart disease	Brunei	1990	Age-standardized		6.8% (3.9 to 9.8%)	9.8 (5.5 to 14.1)
Ischemic heart disease	Brunei	1995	Age-standardized		6.8% (3.8 to 9.9%)	9.5 (5.5 to 14.0)
Ischemic heart disease	Brunei	2000	Age-standardized		6.4% (3.4 to 9.6%)	7.9 (4.2 to 11.8)
Ischemic heart disease	Brunei	2005	Age-standardized		8.6% (5.7 to 11.8%)	10.1 (6.6 to 13.8)
Ischemic heart disease	Brunei	2010	Age-standardized		7.4% (4.4 to 10.6%)	8.1 (4.8 to 11.8)
Ischemic heart disease	Brunei	2015	Age-standardized		5.2% (2.3 to 8.7%)	5.7 (2.6 to 9.6)
Ischemic stroke	Brunei	1990	All Ages	0.0 (0.0 to 0.0)	4.2% (2.0 to 7.4%)	0.6 (0.3 to 1.0)
Ischemic stroke	Brunei	1995	All Ages	0.0 (0.0 to 0.0)	4.2% (1.9 to 7.4%)	0.5 (0.2 to 0.9)
Ischemic stroke	Brunei	2000	All Ages	0.0 (0.0 to 0.0)	3.9% (1.7 to 7.4%)	0.4 (0.2 to 0.8)
Ischemic stroke	Brunei	2005	All Ages	0.0 (0.0 to 0.0)	5.5% (2.8 to 9.2%)	0.6 (0.3 to 1.0)
Ischemic stroke	Brunei	2010	All Ages	0.0 (0.0 to 0.0)	4.6% (2.1 to 8.3%)	0.6 (0.2 to 1.0)
Ischemic stroke	Brunei	2015	All Ages	0.0 (0.0 to 0.0)	3.2% (1.0 to 6.6%)	0.5 (0.1 to 0.9)
Ischemic stroke	Brunei	1990	Age-standardized		4.0% (1.7 to 7.6%)	2.2 (0.9 to 4.2)
Ischemic stroke	Brunei	1995	Age-standardized		3.9% (1.6 to 7.5%)	2.1 (0.8 to 4.0)
Ischemic stroke	Brunei	2000	Age-standardized		3.7% (1.4 to 7.6%)	1.7 (0.6 to 3.7)
Ischemic stroke	Brunei	2005	Age-standardized		5.2% (2.4 to 9.4%)	2.1 (0.9 to 3.8)
Ischemic stroke	Brunei	2010	Age-standardized		4.4% (1.7 to 8.5%)	1.6 (0.6 to 3.1)

Cause name	Location	Year	Age group	Number (in thousands)	PAF	Rate (per 100,000)
Ischemic stroke	Brunei	2015	Age-standardized		3.0% (0.8 to 6.8%)	1.1 (0.3 to 2.5)
Hemorrhagic stroke	Brunei	1990	All Ages	0.0 (0.0 to 0.0)	5.3% (3.0 to 8.1%)	0.9 (0.5 to 1.4)
Hemorrhagic stroke	Brunei	1995	All Ages	0.0 (0.0 to 0.0)	5.4% (3.1 to 8.1%)	0.9 (0.5 to 1.4)
Hemorrhagic stroke	Brunei	2000	All Ages	0.0 (0.0 to 0.0)	5.1% (2.6 to 8.0%)	0.7 (0.4 to 1.1)
Hemorrhagic stroke	Brunei	2005	All Ages	0.0 (0.0 to 0.0)	7.1% (4.4 to 10.2%)	1.0 (0.6 to 1.4)
Hemorrhagic stroke	Brunei	2010	All Ages	0.0 (0.0 to 0.0)	5.8% (3.2 to 8.9%)	0.9 (0.5 to 1.3)
Hemorrhagic stroke	Brunei	2015	All Ages	0.0 (0.0 to 0.0)	4.0% (1.8 to 6.9%)	0.7 (0.3 to 1.2)
Hemorrhagic stroke	Brunei	1990	Age-standardized		4.8% (2.6 to 7.6%)	2.4 (1.2 to 3.9)
Hemorrhagic stroke	Brunei	1995	Age-standardized		4.8% (2.5 to 7.4%)	2.3 (1.2 to 3.6)
Hemorrhagic stroke	Brunei	2000	Age-standardized		4.4% (2.2 to 7.1%)	1.8 (0.9 to 2.9)
Hemorrhagic stroke	Brunei	2005	Age-standardized		6.2% (3.6 to 9.2%)	2.1 (1.2 to 3.1)
Hemorrhagic stroke	Brunei	2010	Age-standardized		5.2% (2.7 to 8.2%)	1.6 (0.8 to 2.5)
Hemorrhagic stroke	Brunei	2015	Age-standardized		3.6% (1.4 to 6.5%)	1.1 (0.4 to 1.9)
Chronic obstructive pulmonary disease	Brunei	1990	All Ages	0.0 (0.0 to 0.0)	5.1% (0.7 to 13.1%)	0.9 (0.1 to 2.3)
Chronic obstructive pulmonary disease	Brunei	1995	All Ages	0.0 (0.0 to 0.0)	5.1% (0.7 to 13.1%)	0.8 (0.1 to 2.1)
Chronic obstructive pulmonary disease	Brunei	2000	All Ages	0.0 (0.0 to 0.0)	4.8% (0.6 to 12.3%)	0.6 (0.1 to 1.6)
Chronic obstructive pulmonary disease	Brunei	2005	All Ages	0.0 (0.0 to 0.0)	7.4% (1.6 to 16.4%)	1.0 (0.2 to 2.2)
Chronic obstructive pulmonary disease	Brunei	2010	All Ages	0.0 (0.0 to 0.0)	5.9% (1.0 to 14.8%)	0.9 (0.2 to 2.2)
Chronic obstructive pulmonary disease	Brunei	2015	All Ages	0.0 (0.0 to 0.0)	3.9% (0.3 to 11.3%)	0.7 (0.1 to 2.0)
Chronic obstructive pulmonary disease	Brunei	1990	Age-standardized		5.3% (0.7 to 13.5%)	3.4 (0.5 to 8.4)
Chronic obstructive pulmonary disease	Brunei	1995	Age-standardized		5.2% (0.7 to 13.5%)	3.1 (0.4 to 7.9)
Chronic obstructive pulmonary disease	Brunei	2000	Age-standardized		4.9% (0.6 to 12.6%)	2.6 (0.3 to 6.6)
Chronic obstructive pulmonary disease	Brunei	2005	Age-standardized		7.5% (1.7 to 16.8%)	3.4 (0.8 to 7.4)
Chronic obstructive pulmonary disease	Brunei	2010	Age-standardized		6.0% (1.1 to 15.1%)	2.6 (0.5 to 6.3)
Chronic obstructive pulmonary disease	Brunei	2015	Age-standardized		3.9% (0.3 to 11.4%)	1.6 (0.1 to 4.8)
All causes	Japan	1990	All Ages	38.1 (29.9 to 48.3)	4.6% (3.6 to 5.8%)	31.0 (24.3 to 39.3)
All causes	Japan	1995	All Ages	40.7 (31.3 to 52.9)	4.4% (3.4 to 5.7%)	32.7 (25.1 to 42.5)
All causes	Japan	2000	All Ages	41.8 (31.7 to 54.4)	4.2% (3.2 to 5.5%)	33.3 (25.3 to 43.4)
All causes	Japan	2005	All Ages	48.2 (36.4 to 63.4)	4.4% (3.3 to 5.7%)	38.2 (28.9 to 50.3)
All causes	Japan	2010	All Ages	50.8 (37.2 to 68.4)	4.1% (3.0 to 5.5%)	40.2 (29.4 to 54.2)
All causes	Japan	2015	All Ages	60.6 (44.5 to 81.4)	4.3% (3.2 to 5.8%)	47.2 (34.7 to 63.4)
All causes	Japan	1990	Age-standardized		4.6% (3.5 to 5.9%)	26.7 (20.7 to 34.2)
All causes	Japan	1995	Age-standardized		4.3% (3.3 to 5.6%)	23.5 (18.0 to 30.5)
All causes	Japan	2000	Age-standardized		4.1% (3.1 to 5.3%)	20.1 (15.4 to 26.0)
All causes	Japan	2005	Age-standardized		4.2% (3.2 to 5.4%)	19.1 (14.7 to 24.8)
All causes	Japan	2010	Age-standardized		3.9% (3.0 to 5.1%)	16.7 (12.6 to 22.0)

Cause name	Location	Year	Age group	Number (in thousands)	PAF	Rate (per 100,000)
All causes	Japan	2015	Age-standardized		4.1% (3.1 to 5.3%)	16.8 (12.8 to 21.9)
Lower respiratory infections	Japan	1990	All Ages	7.7 (2.4 to 15.6)	11.0% (3.4 to 22.1%)	6.3 (2.0 to 12.7)
Lower respiratory infections	Japan	1995	All Ages	9.3 (2.8 to 18.7)	10.9% (3.3 to 21.8%)	7.5 (2.3 to 15.0)
Lower respiratory infections	Japan	2000	All Ages	10.2 (3.1 to 20.7)	10.8% (3.3 to 21.8%)	8.2 (2.5 to 16.5)
Lower respiratory infections	Japan	2005	All Ages	12.8 (4.2 to 25.1)	11.5% (3.8 to 22.4%)	10.2 (3.3 to 19.9)
Lower respiratory infections	Japan	2010	All Ages	14.2 (4.2 to 28.8)	10.7% (3.2 to 21.8%)	11.3 (3.4 to 22.8)
Lower respiratory infections	Japan	2015	All Ages	18.1 (5.9 to 35.2)	11.6% (3.7 to 22.5%)	14.1 (4.6 to 27.5)
Lower respiratory infections	Japan	1990	Age-standardized		11.0% (3.4 to 22.1%)	5.6 (1.7 to 11.3)
Lower respiratory infections	Japan	1995	Age-standardized		10.9% (3.3 to 21.8%)	5.4 (1.6 to 10.9)
Lower respiratory infections	Japan	2000	Age-standardized		10.8% (3.3 to 21.8%)	4.8 (1.5 to 9.7)
Lower respiratory infections	Japan	2005	Age-standardized		11.5% (3.8 to 22.4%)	4.8 (1.6 to 9.4)
Lower respiratory infections	Japan	2010	Age-standardized		10.7% (3.2 to 21.8%)	4.3 (1.3 to 8.6)
Lower respiratory infections	Japan	2015	Age-standardized		11.6% (3.7 to 22.5%)	4.5 (1.5 to 8.8)
Tracheal, bronchus, and lung cancer	Japan	1990	All Ages	2.9 (1.4 to 4.8)	7.6% (3.6 to 12.7%)	2.3 (1.1 to 3.9)
Tracheal, bronchus, and lung cancer	Japan	1995	All Ages	3.5 (1.7 to 5.9)	7.5% (3.6 to 12.5%)	2.8 (1.3 to 4.7)
Tracheal, bronchus, and lung cancer	Japan	2000	All Ages	4.1 (1.9 to 6.9)	7.4% (3.5 to 12.5%)	3.3 (1.5 to 5.5)
Tracheal, bronchus, and lung cancer	Japan	2005	All Ages	5.1 (2.4 to 8.4)	7.9% (3.8 to 13.1%)	4.0 (1.9 to 6.7)
Tracheal, bronchus, and lung cancer	Japan	2010	All Ages	5.2 (2.5 to 8.8)	7.4% (3.5 to 12.3%)	4.2 (2.0 to 7.0)
Tracheal, bronchus, and lung cancer	Japan	2015	All Ages	6.3 (3.0 to 10.4)	8.0% (3.9 to 13.2%)	4.9 (2.4 to 8.1)
Tracheal, bronchus, and lung cancer	Japan	1990	Age-standardized		7.6% (3.6 to 12.7%)	1.9 (0.9 to 3.2)
Tracheal, bronchus, and lung cancer	Japan	1995	Age-standardized		7.5% (3.6 to 12.4%)	2.0 (0.9 to 3.4)
Tracheal, bronchus, and lung cancer	Japan	2000	Age-standardized		7.4% (3.5 to 12.5%)	2.0 (0.9 to 3.3)
Tracheal, bronchus, and lung cancer	Japan	2005	Age-standardized		7.9% (3.8 to 13.1%)	2.1 (1.0 to 3.4)
Tracheal, bronchus, and lung cancer	Japan	2010	Age-standardized		7.4% (3.5 to 12.3%)	1.8 (0.9 to 3.1)
Tracheal, bronchus, and lung cancer	Japan	2015	Age-standardized		8.0% (3.9 to 13.2%)	1.9 (0.9 to 3.2)
Cardiovascular diseases	Japan	1990	All Ages	24.6 (19.1 to 30.5)	8.5% (6.6 to 10.5%)	20.0 (15.6 to 24.9)
Cardiovascular diseases	Japan	1995	All Ages	24.8 (19.1 to 31.2)	8.3% (6.4 to 10.5%)	19.9 (15.3 to 25.1)
Cardiovascular diseases	Japan	2000	All Ages	24.4 (18.7 to 30.9)	8.2% (6.2 to 10.4%)	19.4 (14.9 to 24.6)
Cardiovascular diseases	Japan	2005	All Ages	26.6 (19.9 to 33.7)	8.2% (6.2 to 10.4%)	21.1 (15.8 to 26.8)
Cardiovascular diseases	Japan	2010	All Ages	27.3 (19.7 to 35.7)	7.6% (5.6 to 10.0%)	21.6 (15.6 to 28.3)
Cardiovascular diseases	Japan	2015	All Ages	31.4 (22.3 to 41.8)	7.7% (5.5 to 10.2%)	24.5 (17.4 to 32.6)
Cardiovascular diseases	Japan	1990	Age-standardized		8.3% (6.4 to 10.5%)	17.1 (13.2 to 21.4)
Cardiovascular diseases	Japan	1995	Age-standardized		8.3% (6.4 to 10.5%)	14.3 (11.0 to 18.0)
Cardiovascular diseases	Japan	2000	Age-standardized		8.3% (6.4 to 10.5%)	11.9 (9.2 to 14.9)
Cardiovascular diseases	Japan	2005	Age-standardized		8.5% (6.6 to 10.6%)	10.8 (8.4 to 13.5)
Cardiovascular diseases	Japan	2010	Age-standardized		8.2% (6.3 to 10.2%)	9.4 (7.2 to 11.8)

Cause name	Location	Year	Age group	Number (in thousands)	PAF	Rate (per 100,000)
Cardiovascular diseases	Japan	2015	Age-standardized		8.4% (6.4 to 10.5%)	9.2 (7.0 to 11.5)
Ischemic heart disease	Japan	1990	All Ages	13.6 (9.8 to 17.2)	11.6% (8.4 to 14.6%)	11.0 (8.0 to 14.0)
Ischemic heart disease	Japan	1995	All Ages	13.5 (9.6 to 17.2)	11.5% (8.1 to 14.7%)	10.8 (7.7 to 13.8)
Ischemic heart disease	Japan	2000	All Ages	13.3 (9.5 to 17.2)	11.4% (8.1 to 14.7%)	10.6 (7.5 to 13.7)
Ischemic heart disease	Japan	2005	All Ages	14.8 (10.3 to 19.3)	11.5% (7.9 to 14.9%)	11.7 (8.2 to 15.3)
Ischemic heart disease	Japan	2010	All Ages	15.5 (10.1 to 21.0)	10.9% (7.1 to 14.6%)	12.3 (8.0 to 16.6)
Ischemic heart disease	Japan	2015	All Ages	18.0 (11.4 to 24.5)	11.0% (7.0 to 15.0%)	14.0 (8.9 to 19.1)
Ischemic heart disease	Japan	1990	Age-standardized		11.5% (8.1 to 14.7%)	9.4 (6.7 to 12.1)
Ischemic heart disease	Japan	1995	Age-standardized		11.5% (8.2 to 14.8%)	7.8 (5.6 to 9.9)
Ischemic heart disease	Japan	2000	Age-standardized		11.6% (8.4 to 14.8%)	6.5 (4.7 to 8.3)
Ischemic heart disease	Japan	2005	Age-standardized		12.0% (8.7 to 15.2%)	6.1 (4.4 to 7.7)
Ischemic heart disease	Japan	2010	Age-standardized		11.6% (8.3 to 14.8%)	5.4 (3.8 to 6.9)
Ischemic heart disease	Japan	2015	Age-standardized		11.9% (8.5 to 15.2%)	5.3 (3.8 to 6.8)
Ischemic stroke	Japan	1990	All Ages	6.4 (3.5 to 9.9)	7.7% (4.3 to 11.9%)	5.2 (2.9 to 8.1)
Ischemic stroke	Japan	1995	All Ages	6.7 (3.6 to 10.6)	7.6% (4.1 to 12.0%)	5.4 (2.9 to 8.5)
Ischemic stroke	Japan	2000	All Ages	6.5 (3.4 to 10.5)	7.5% (4.0 to 12.0%)	5.2 (2.7 to 8.3)
Ischemic stroke	Japan	2005	All Ages	6.8 (3.4 to 11.0)	7.6% (3.9 to 12.4%)	5.4 (2.7 to 8.8)
Ischemic stroke	Japan	2010	All Ages	6.7 (3.1 to 11.5)	7.2% (3.4 to 12.4%)	5.3 (2.5 to 9.1)
Ischemic stroke	Japan	2015	All Ages	7.7 (3.6 to 13.3)	7.5% (3.5 to 12.8%)	6.0 (2.8 to 10.4)
Ischemic stroke	Japan	1990	Age-standardized		7.6% (4.1 to 12.0%)	4.5 (2.5 to 7.2)
Ischemic stroke	Japan	1995	Age-standardized		7.5% (4.0 to 12.0%)	3.8 (2.1 to 6.1)
Ischemic stroke	Japan	2000	Age-standardized		7.5% (4.0 to 12.0%)	3.0 (1.6 to 4.9)
Ischemic stroke	Japan	2005	Age-standardized		7.7% (4.1 to 12.3%)	2.5 (1.3 to 4.0)
Ischemic stroke	Japan	2010	Age-standardized		7.4% (3.8 to 12.2%)	2.0 (1.0 to 3.3)
Ischemic stroke	Japan	2015	Age-standardized		7.7% (3.9 to 12.6%)	1.9 (1.0 to 3.1)
Hemorrhagic stroke	Japan	1990	All Ages	4.6 (3.3 to 6.0)	9.4% (6.7 to 12.2%)	3.8 (2.7 to 4.9)
Hemorrhagic stroke	Japan	1995	All Ages	4.6 (3.3 to 6.1)	9.1% (6.5 to 11.9%)	3.7 (2.6 to 4.9)
Hemorrhagic stroke	Japan	2000	All Ages	4.6 (3.2 to 6.2)	9.0% (6.3 to 11.9%)	3.7 (2.6 to 4.9)
Hemorrhagic stroke	Japan	2005	All Ages	5.0 (3.5 to 6.9)	9.0% (6.2 to 12.2%)	4.0 (2.8 to 5.5)
Hemorrhagic stroke	Japan	2010	All Ages	5.1 (3.3 to 7.3)	8.3% (5.4 to 11.8%)	4.0 (2.6 to 5.8)
Hemorrhagic stroke	Japan	2015	All Ages	5.7 (3.6 to 8.3)	8.4% (5.3 to 12.2%)	4.4 (2.8 to 6.4)
Hemorrhagic stroke	Japan	1990	Age-standardized		9.3% (6.6 to 12.1%)	3.1 (2.2 to 4.1)
Hemorrhagic stroke	Japan	1995	Age-standardized		9.2% (6.6 to 12.0%)	2.7 (1.9 to 3.5)
Hemorrhagic stroke	Japan	2000	Age-standardized		9.2% (6.6 to 12.0%)	2.4 (1.7 to 3.1)
Hemorrhagic stroke	Japan	2005	Age-standardized		9.5% (6.8 to 12.4%)	2.2 (1.6 to 2.9)
Hemorrhagic stroke	Japan	2010	Age-standardized		9.0% (6.4 to 12.0%)	2.0 (1.4 to 2.6)

Cause name	Location	Year	Age group	Number (in thousands)	PAF	Rate (per 100,000)
Hemorrhagic stroke	Japan	2015	Age-standardized		9.3% (6.6 to 12.4%)	1.9 (1.3 to 2.5)
Chronic obstructive pulmonary disease	Japan	1990	All Ages	2.9 (1.1 to 5.1)	13.2% (5.0 to 23.1%)	2.4 (0.9 to 4.1)
Chronic obstructive pulmonary disease	Japan	1995	All Ages	3.1 (1.2 to 5.5)	13.0% (4.9 to 23.1%)	2.5 (0.9 to 4.4)
Chronic obstructive pulmonary disease	Japan	2000	All Ages	3.1 (1.2 to 5.6)	13.0% (4.9 to 23.0%)	2.5 (1.0 to 4.5)
Chronic obstructive pulmonary disease	Japan	2005	All Ages	3.7 (1.5 to 6.5)	13.6% (5.3 to 23.6%)	2.9 (1.2 to 5.2)
Chronic obstructive pulmonary disease	Japan	2010	All Ages	4.0 (1.6 to 7.3)	12.9% (4.9 to 22.9%)	3.2 (1.2 to 5.8)
Chronic obstructive pulmonary disease	Japan	2015	All Ages	4.9 (1.9 to 8.7)	13.7% (5.4 to 23.8%)	3.8 (1.5 to 6.7)
Chronic obstructive pulmonary disease	Japan	1990	Age-standardized		13.2% (5.0 to 23.1%)	2.1 (0.8 to 3.6)
Chronic obstructive pulmonary disease	Japan	1995	Age-standardized		13.0% (4.9 to 23.0%)	1.8 (0.7 to 3.1)
Chronic obstructive pulmonary disease	Japan	2000	Age-standardized		12.9% (4.9 to 23.0%)	1.5 (0.6 to 2.6)
Chronic obstructive pulmonary disease	Japan	2005	Age-standardized		13.5% (5.2 to 23.6%)	1.4 (0.6 to 2.4)
Chronic obstructive pulmonary disease	Japan	2010	Age-standardized		12.9% (4.9 to 22.9%)	1.2 (0.5 to 2.2)
Chronic obstructive pulmonary disease	Japan	2015	Age-standardized		13.6% (5.4 to 23.8%)	1.2 (0.5 to 2.2)
All causes	Singapore	1990	All Ages	1.1 (0.8 to 1.4)	7.6% (5.5 to 9.8%)	38.9 (28.4 to 49.9)
All causes	Singapore	1995	All Ages	1.2 (0.9 to 1.5)	7.7% (5.6 to 9.8%)	39.9 (29.2 to 51.1)
All causes	Singapore	2000	All Ages	1.2 (0.9 to 1.5)	7.6% (5.6 to 9.7%)	36.8 (27.0 to 46.9)
All causes	Singapore	2005	All Ages	1.6 (1.2 to 2.0)	9.0% (6.7 to 11.4%)	44.1 (32.8 to 55.7)
All causes	Singapore	2010	All Ages	1.4 (1.0 to 2.0)	7.2% (4.8 to 9.8%)	38.0 (25.4 to 51.8)
All causes	Singapore	2015	All Ages	1.7 (1.1 to 2.3)	7.2% (4.9 to 9.8%)	42.4 (28.5 to 57.3)
All causes	Singapore	1990	Age-standardized		7.9% (5.7 to 10.2%)	62.3 (44.5 to 80.0)
All causes	Singapore	1995	Age-standardized		7.9% (5.7 to 10.1%)	58.4 (42.5 to 75.4)
All causes	Singapore	2000	Age-standardized		7.7% (5.6 to 9.9%)	48.1 (34.8 to 61.8)
All causes	Singapore	2005	Age-standardized		9.1% (6.7 to 11.5%)	48.0 (35.6 to 60.8)
All causes	Singapore	2010	Age-standardized		7.2% (4.8 to 9.8%)	34.8 (23.2 to 47.4)
All causes	Singapore	2015	Age-standardized		7.1% (4.8 to 9.6%)	32.5 (21.9 to 43.8)
Lower respiratory infections	Singapore	1990	All Ages	0.2 (0.1 to 0.4)	15.6% (6.3 to 26.7%)	8.4 (3.4 to 14.5)
Lower respiratory infections	Singapore	1995	All Ages	0.3 (0.1 to 0.5)	15.8% (6.2 to 26.7%)	9.8 (3.8 to 16.8)
Lower respiratory infections	Singapore	2000	All Ages	0.3 (0.1 to 0.6)	16.0% (6.4 to 26.9%)	10.0 (3.9 to 17.0)
Lower respiratory infections	Singapore	2005	All Ages	0.5 (0.3 to 0.8)	20.7% (10.4 to 31.2%)	15.1 (7.7 to 22.8)
Lower respiratory infections	Singapore	2010	All Ages	0.5 (0.2 to 0.9)	15.4% (5.5 to 26.8%)	13.8 (5.0 to 24.1)
Lower respiratory infections	Singapore	2015	All Ages	0.7 (0.3 to 1.1)	15.7% (6.2 to 26.9%)	17.0 (6.8 to 28.7)
Lower respiratory infections	Singapore	1990	Age-standardized		15.6% (6.3 to 26.7%)	14.7 (5.9 to 25.2)
Lower respiratory infections	Singapore	1995	Age-standardized		15.8% (6.2 to 26.7%)	15.2 (5.9 to 26.0)
Lower respiratory infections	Singapore	2000	Age-standardized		16.0% (6.4 to 26.9%)	13.7 (5.3 to 23.4)
Lower respiratory infections	Singapore	2005	Age-standardized		20.7% (10.4 to 31.2%)	17.0 (8.6 to 25.6)
Lower respiratory infections	Singapore	2010	Age-standardized		15.4% (5.5 to 26.8%)	12.8 (4.6 to 22.2)

Cause name	Location	Year	Age group	Number (in thousands)	PAF	Rate (per 100,000)
Lower respiratory infections	Singapore	2015	Age-standardized		15.7% (6.2 to 26.9%)	12.7 (5.1 to 21.4)
Tracheal, bronchus, and lung cancer	Singapore	1990	All Ages	0.1 (0.0 to 0.1)	10.9% (5.2 to 18.0%)	3.1 (1.5 to 5.1)
Tracheal, bronchus, and lung cancer	Singapore	1995	All Ages	0.1 (0.1 to 0.2)	11.0% (5.3 to 17.9%)	3.3 (1.6 to 5.3)
Tracheal, bronchus, and lung cancer	Singapore	2000	All Ages	0.1 (0.1 to 0.2)	11.2% (5.2 to 18.2%)	3.2 (1.5 to 5.2)
Tracheal, bronchus, and lung cancer	Singapore	2005	All Ages	0.1 (0.1 to 0.2)	14.7% (7.9 to 22.5%)	4.2 (2.2 to 6.5)
Tracheal, bronchus, and lung cancer	Singapore	2010	All Ages	0.1 (0.1 to 0.2)	10.8% (4.8 to 17.7%)	3.3 (1.5 to 5.4)
Tracheal, bronchus, and lung cancer	Singapore	2015	All Ages	0.1 (0.1 to 0.3)	11.0% (5.2 to 18.4%)	3.8 (1.8 to 6.5)
Tracheal, bronchus, and lung cancer	Singapore	1990	Age-standardized		10.9% (5.2 to 18.1%)	4.7 (2.3 to 7.8)
Tracheal, bronchus, and lung cancer	Singapore	1995	Age-standardized		11.0% (5.3 to 17.9%)	4.7 (2.3 to 7.6)
Tracheal, bronchus, and lung cancer	Singapore	2000	Age-standardized		11.2% (5.2 to 18.2%)	4.1 (1.9 to 6.6)
Tracheal, bronchus, and lung cancer	Singapore	2005	Age-standardized		14.7% (7.9 to 22.5%)	4.5 (2.4 to 7.0)
Tracheal, bronchus, and lung cancer	Singapore	2010	Age-standardized		10.8% (4.8 to 17.7%)	3.1 (1.4 to 5.1)
Tracheal, bronchus, and lung cancer	Singapore	2015	Age-standardized		11.0% (5.2 to 18.4%)	3.1 (1.4 to 5.3)
Cardiovascular diseases	Singapore	1990	All Ages	0.6 (0.5 to 0.8)	12.3% (9.6 to 14.8%)	22.8 (17.9 to 27.7)
Cardiovascular diseases	Singapore	1995	All Ages	0.7 (0.5 to 0.8)	11.7% (9.0 to 14.1%)	22.3 (17.3 to 26.9)
Cardiovascular diseases	Singapore	2000	All Ages	0.7 (0.5 to 0.8)	11.5% (9.0 to 14.1%)	20.2 (15.8 to 24.9)
Cardiovascular diseases	Singapore	2005	All Ages	0.8 (0.6 to 0.9)	12.8% (10.3 to 15.4%)	21.3 (17.0 to 25.9)
Cardiovascular diseases	Singapore	2010	All Ages	0.7 (0.5 to 0.9)	10.8% (8.2 to 13.4%)	18.2 (14.0 to 22.9)
Cardiovascular diseases	Singapore	2015	All Ages	0.7 (0.6 to 0.9)	10.3% (7.8 to 13.2%)	18.7 (14.1 to 23.7)
Cardiovascular diseases	Singapore	1990	Age-standardized		11.7% (9.1 to 14.3%)	34.8 (26.8 to 42.4)
Cardiovascular diseases	Singapore	1995	Age-standardized		11.3% (8.6 to 13.7%)	31.6 (24.3 to 38.5)
Cardiovascular diseases	Singapore	2000	Age-standardized		11.2% (8.6 to 13.8%)	25.7 (19.8 to 31.8)
Cardiovascular diseases	Singapore	2005	Age-standardized		12.5% (9.9 to 15.2%)	22.6 (17.9 to 27.7)
Cardiovascular diseases	Singapore	2010	Age-standardized		10.6% (8.2 to 13.2%)	16.5 (12.6 to 20.6)
Cardiovascular diseases	Singapore	2015	Age-standardized		10.4% (8.0 to 13.1%)	14.4 (11.0 to 18.1)
Ischemic heart disease	Singapore	1990	All Ages	0.4 (0.3 to 0.6)	15.3% (11.9 to 19.0%)	16.2 (12.5 to 20.2)
Ischemic heart disease	Singapore	1995	All Ages	0.5 (0.4 to 0.6)	14.7% (11.0 to 18.2%)	15.9 (12.0 to 19.7)
Ischemic heart disease	Singapore	2000	All Ages	0.5 (0.4 to 0.6)	14.6% (11.2 to 18.1%)	14.9 (11.4 to 18.6)
Ischemic heart disease	Singapore	2005	All Ages	0.6 (0.4 to 0.7)	16.1% (12.4 to 19.8%)	15.7 (12.1 to 19.5)
Ischemic heart disease	Singapore	2010	All Ages	0.5 (0.4 to 0.7)	13.9% (10.4 to 17.6%)	13.6 (10.1 to 17.5)
Ischemic heart disease	Singapore	2015	All Ages	0.5 (0.4 to 0.7)	13.6% (9.7 to 17.7%)	13.7 (9.8 to 17.6)
Ischemic heart disease	Singapore	1990	Age-standardized		14.6% (11.1 to 18.3%)	24.5 (18.5 to 30.7)
Ischemic heart disease	Singapore	1995	Age-standardized		14.1% (10.4 to 17.7%)	22.3 (16.4 to 28.1)
Ischemic heart disease	Singapore	2000	Age-standardized		14.0% (10.6 to 17.7%)	18.7 (14.1 to 23.6)
Ischemic heart disease	Singapore	2005	Age-standardized		15.7% (11.9 to 19.4%)	16.5 (12.5 to 20.7)
Ischemic heart disease	Singapore	2010	Age-standardized		13.7% (10.2 to 17.3%)	12.2 (9.1 to 15.6)

Cause name	Location	Year	Age group	Number (in thousands)	PAF	Rate (per 100,000)
Ischemic heart disease	Singapore	2015	Age-standardized		13.7% (10.1 to 17.5%)	10.6 (7.8 to 13.5)
Ischemic stroke	Singapore	1990	All Ages	0.1 (0.1 to 0.1)	10.2% (6.5 to 13.5%)	3.3 (2.1 to 4.4)
Ischemic stroke	Singapore	1995	All Ages	0.1 (0.1 to 0.1)	9.9% (6.5 to 13.5%)	3.4 (2.2 to 4.7)
Ischemic stroke	Singapore	2000	All Ages	0.1 (0.1 to 0.1)	9.8% (6.1 to 14.0%)	3.0 (1.8 to 4.2)
Ischemic stroke	Singapore	2005	All Ages	0.1 (0.1 to 0.2)	11.0% (7.1 to 15.5%)	3.2 (2.0 to 4.5)
Ischemic stroke	Singapore	2010	All Ages	0.1 (0.1 to 0.2)	9.2% (5.2 to 14.1%)	2.7 (1.5 to 4.2)
Ischemic stroke	Singapore	2015	All Ages	0.1 (0.1 to 0.2)	9.1% (4.9 to 14.1%)	3.0 (1.6 to 4.6)
Ischemic stroke	Singapore	1990	Age-standardized		9.8% (6.1 to 13.5%)	5.6 (3.5 to 7.8)
Ischemic stroke	Singapore	1995	Age-standardized		9.7% (6.2 to 13.5%)	5.2 (3.3 to 7.3)
Ischemic stroke	Singapore	2000	Age-standardized		9.6% (5.9 to 14.0%)	4.0 (2.4 to 5.8)
Ischemic stroke	Singapore	2005	Age-standardized		11.0% (7.0 to 15.5%)	3.6 (2.2 to 5.1)
Ischemic stroke	Singapore	2010	Age-standardized		9.2% (5.3 to 13.9%)	2.5 (1.4 to 3.8)
Ischemic stroke	Singapore	2015	Age-standardized		9.2% (5.3 to 13.9%)	2.2 (1.3 to 3.4)
Hemorrhagic stroke	Singapore	1990	All Ages	0.1 (0.1 to 0.1)	12.0% (8.5 to 15.2%)	3.3 (2.3 to 4.2)
Hemorrhagic stroke	Singapore	1995	All Ages	0.1 (0.1 to 0.1)	11.5% (8.3 to 14.9%)	3.0 (2.2 to 3.9)
Hemorrhagic stroke	Singapore	2000	All Ages	0.1 (0.1 to 0.1)	11.4% (8.2 to 14.7%)	2.4 (1.7 to 3.1)
Hemorrhagic stroke	Singapore	2005	All Ages	0.1 (0.1 to 0.1)	13.2% (9.6 to 16.7%)	2.4 (1.8 to 3.1)
Hemorrhagic stroke	Singapore	2010	All Ages	0.1 (0.1 to 0.1)	10.8% (7.6 to 14.7%)	2.0 (1.4 to 2.7)
Hemorrhagic stroke	Singapore	2015	All Ages	0.1 (0.1 to 0.1)	10.6% (7.2 to 14.2%)	2.0 (1.4 to 2.8)
Hemorrhagic stroke	Singapore	1990	Age-standardized		11.4% (7.9 to 14.6%)	4.7 (3.3 to 6.1)
Hemorrhagic stroke	Singapore	1995	Age-standardized		11.0% (7.8 to 14.3%)	4.1 (2.9 to 5.3)
Hemorrhagic stroke	Singapore	2000	Age-standardized		10.9% (7.7 to 14.3%)	2.9 (2.0 to 3.9)
Hemorrhagic stroke	Singapore	2005	Age-standardized		12.7% (9.1 to 16.2%)	2.5 (1.8 to 3.2)
Hemorrhagic stroke	Singapore	2010	Age-standardized		10.6% (7.4 to 14.3%)	1.7 (1.2 to 2.4)
Hemorrhagic stroke	Singapore	2015	Age-standardized		10.6% (7.4 to 14.0%)	1.6 (1.1 to 2.1)
Chronic obstructive pulmonary disease	Singapore	1990	All Ages	0.1 (0.1 to 0.2)	17.1% (7.4 to 28.1%)	4.6 (2.0 to 7.7)
Chronic obstructive pulmonary disease	Singapore	1995	All Ages	0.1 (0.1 to 0.2)	17.3% (7.1 to 28.4%)	4.5 (1.9 to 7.4)
Chronic obstructive pulmonary disease	Singapore	2000	All Ages	0.1 (0.1 to 0.2)	17.5% (7.8 to 28.9%)	3.3 (1.5 to 5.6)
Chronic obstructive pulmonary disease	Singapore	2005	All Ages	0.1 (0.1 to 0.2)	21.2% (10.7 to 32.9%)	3.5 (1.7 to 5.5)
Chronic obstructive pulmonary disease	Singapore	2010	All Ages	0.1 (0.0 to 0.2)	17.0% (7.0 to 28.0%)	2.6 (1.1 to 4.5)
Chronic obstructive pulmonary disease	Singapore	2015	All Ages	0.1 (0.1 to 0.2)	17.2% (7.2 to 28.4%)	2.8 (1.2 to 4.7)
Chronic obstructive pulmonary disease	Singapore	1990	Age-standardized		17.1% (7.5 to 28.2%)	8.0 (3.4 to 13.4)
Chronic obstructive pulmonary disease	Singapore	1995	Age-standardized		17.3% (7.1 to 28.5%)	7.0 (2.9 to 11.6)
Chronic obstructive pulmonary disease	Singapore	2000	Age-standardized		17.5% (7.8 to 28.9%)	4.6 (2.0 to 7.7)
Chronic obstructive pulmonary disease	Singapore	2005	Age-standardized		21.2% (10.7 to 32.9%)	3.9 (1.9 to 6.2)
Chronic obstructive pulmonary disease	Singapore	2010	Age-standardized		17.0% (7.0 to 28.0%)	2.5 (1.0 to 4.2)

Cause name	Location	Year	Age group	Number (in thousands)	PAF	Rate (per 100,000)
Chronic obstructive pulmonary disease	Singapore	2015	Age-standardized		17.2% (7.2 to 28.3%)	2.2 (0.9 to 3.6)
All causes	South Korea	1990	All Ages	15.1 (13.2 to 17.0)	6.3% (5.5 to 7.1%)	35.0 (30.6 to 39.5)
All causes	South Korea	1995	All Ages	13.7 (11.7 to 15.6)	5.6% (4.8 to 6.4%)	30.6 (26.2 to 35.0)
All causes	South Korea	2000	All Ages	13.1 (11.2 to 15.3)	5.4% (4.6 to 6.3%)	28.5 (24.2 to 33.2)
All causes	South Korea	2005	All Ages	13.1 (11.1 to 15.2)	5.3% (4.5 to 6.2%)	27.7 (23.5 to 32.1)
All causes	South Korea	2010	All Ages	13.8 (11.6 to 16.3)	5.2% (4.3 to 6.1%)	28.1 (23.6 to 33.1)
All causes	South Korea	2015	All Ages	18.2 (15.1 to 21.4)	5.7% (4.7 to 6.6%)	36.1 (30.0 to 42.6)
All causes	South Korea	1990	Age-standardized		6.8% (5.8 to 7.9%)	67.8 (57.1 to 78.6)
All causes	South Korea	1995	Age-standardized		6.1% (5.0 to 7.1%)	51.5 (42.7 to 60.1)
All causes	South Korea	2000	Age-standardized		5.7% (4.7 to 6.7%)	41.1 (34.1 to 48.5)
All causes	South Korea	2005	Age-standardized		5.5% (4.6 to 6.4%)	32.0 (26.8 to 37.6)
All causes	South Korea	2010	Age-standardized		5.2% (4.3 to 6.2%)	26.7 (22.1 to 31.6)
All causes	South Korea	2015	Age-standardized		5.6% (4.7 to 6.6%)	27.4 (22.7 to 32.3)
Lower respiratory infections	South Korea	1990	All Ages	1.0 (0.6 to 1.5)	20.2% (11.6 to 30.0%)	2.3 (1.3 to 3.4)
Lower respiratory infections	South Korea	1995	All Ages	0.9 (0.5 to 1.4)	20.3% (11.8 to 30.0%)	2.1 (1.2 to 3.1)
Lower respiratory infections	South Korea	2000	All Ages	1.1 (0.7 to 1.7)	20.4% (11.9 to 30.0%)	2.4 (1.4 to 3.7)
Lower respiratory infections	South Korea	2005	All Ages	1.4 (0.8 to 2.1)	20.5% (11.8 to 30.1%)	3.0 (1.7 to 4.5)
Lower respiratory infections	South Korea	2010	All Ages	2.0 (1.1 to 3.0)	20.0% (11.2 to 29.9%)	4.0 (2.3 to 6.1)
Lower respiratory infections	South Korea	2015	All Ages	3.0 (1.8 to 4.3)	22.0% (13.6 to 31.6%)	5.9 (3.6 to 8.5)
Lower respiratory infections	South Korea	1990	Age-standardized		20.2% (11.6 to 30.0%)	4.8 (2.8 to 7.1)
Lower respiratory infections	South Korea	1995	Age-standardized		20.3% (11.8 to 30.0%)	3.9 (2.3 to 5.8)
Lower respiratory infections	South Korea	2000	Age-standardized		20.4% (11.9 to 30.0%)	4.0 (2.3 to 6.0)
Lower respiratory infections	South Korea	2005	Age-standardized		20.5% (11.8 to 30.1%)	3.9 (2.2 to 5.8)
Lower respiratory infections	South Korea	2010	Age-standardized		20.0% (11.2 to 29.9%)	4.1 (2.3 to 6.2)
Lower respiratory infections	South Korea	2015	Age-standardized		22.0% (13.6 to 31.6%)	4.6 (2.8 to 6.6)
Tracheal, bronchus, and lung cancer	South Korea	1990	All Ages	0.9 (0.5 to 1.4)	14.3% (8.1 to 21.0%)	2.2 (1.2 to 3.2)
Tracheal, bronchus, and lung cancer	South Korea	1995	All Ages	1.4 (0.8 to 2.1)	14.3% (8.2 to 21.1%)	3.1 (1.7 to 4.6)
Tracheal, bronchus, and lung cancer	South Korea	2000	All Ages	1.8 (1.0 to 2.7)	14.4% (8.1 to 21.2%)	3.9 (2.2 to 5.8)
Tracheal, bronchus, and lung cancer	South Korea	2005	All Ages	2.2 (1.2 to 3.3)	14.5% (8.2 to 21.6%)	4.6 (2.6 to 6.9)
Tracheal, bronchus, and lung cancer	South Korea	2010	All Ages	2.4 (1.4 to 3.6)	14.1% (8.1 to 21.0%)	5.0 (2.8 to 7.4)
Tracheal, bronchus, and lung cancer	South Korea	2015	All Ages	3.3 (1.9 to 4.8)	15.6% (9.1 to 22.7%)	6.5 (3.8 to 9.6)
Tracheal, bronchus, and lung cancer	South Korea	1990	Age-standardized		14.3% (8.1 to 21.0%)	3.6 (2.0 to 5.2)
Tracheal, bronchus, and lung cancer	South Korea	1995	Age-standardized		14.3% (8.2 to 21.1%)	4.6 (2.6 to 6.9)
Tracheal, bronchus, and lung cancer	South Korea	2000	Age-standardized		14.4% (8.1 to 21.2%)	5.1 (2.9 to 7.5)
Tracheal, bronchus, and lung cancer	South Korea	2005	Age-standardized		14.5% (8.2 to 21.6%)	4.9 (2.8 to 7.3)
Tracheal, bronchus, and lung cancer	South Korea	2010	Age-standardized		14.1% (8.1 to 21.0%)	4.5 (2.5 to 6.7)

Cause name	Location	Year	Age group	Number (in thousands)	PAF	Rate (per 100,000)
Tracheal, bronchus, and lung cancer	South Korea	2015	Age-standardized		15.6% (9.1 to 22.7%)	4.9 (2.8 to 7.2)
Cardiovascular diseases	South Korea	1990	All Ages	12.2 (10.4 to 14.0)	13.3% (11.4 to 15.3%)	28.3 (24.1 to 32.4)
Cardiovascular diseases	South Korea	1995	All Ages	10.4 (8.7 to 12.1)	12.7% (10.6 to 14.8%)	23.3 (19.4 to 27.1)
Cardiovascular diseases	South Korea	2000	All Ages	9.1 (7.5 to 10.7)	12.2% (10.1 to 14.4%)	19.7 (16.2 to 23.1)
Cardiovascular diseases	South Korea	2005	All Ages	8.2 (6.8 to 9.7)	11.9% (9.7 to 13.9%)	17.4 (14.3 to 20.6)
Cardiovascular diseases	South Korea	2010	All Ages	7.9 (6.4 to 9.5)	11.3% (9.1 to 13.5%)	16.1 (13.0 to 19.2)
Cardiovascular diseases	South Korea	2015	All Ages	9.8 (7.8 to 11.9)	11.5% (9.2 to 13.8%)	19.5 (15.5 to 23.6)
Cardiovascular diseases	South Korea	1990	Age-standardized		12.2% (10.1 to 14.3%)	54.3 (44.8 to 64.1)
Cardiovascular diseases	South Korea	1995	Age-standardized		11.8% (9.5 to 14.1%)	39.0 (31.4 to 46.7)
Cardiovascular diseases	South Korea	2000	Age-standardized		11.4% (9.0 to 13.7%)	28.0 (22.3 to 34.0)
Cardiovascular diseases	South Korea	2005	Age-standardized		11.2% (8.9 to 13.6%)	19.8 (15.8 to 24.2)
Cardiovascular diseases	South Korea	2010	Age-standardized		11.0% (8.7 to 13.3%)	15.1 (11.9 to 18.4)
Cardiovascular diseases	South Korea	2015	Age-standardized		11.4% (9.1 to 13.8%)	14.7 (11.6 to 17.9)
Ischemic heart disease	South Korea	1990	All Ages	5.9 (4.9 to 6.9)	16.9% (14.0 to 19.9%)	13.7 (11.3 to 16.1)
Ischemic heart disease	South Korea	1995	All Ages	4.3 (3.4 to 5.1)	16.3% (13.2 to 19.5%)	9.5 (7.7 to 11.4)
Ischemic heart disease	South Korea	2000	All Ages	3.4 (2.8 to 4.2)	16.1% (13.0 to 19.4%)	7.4 (6.0 to 9.0)
Ischemic heart disease	South Korea	2005	All Ages	3.4 (2.7 to 4.2)	15.8% (12.3 to 19.2%)	7.2 (5.6 to 8.9)
Ischemic heart disease	South Korea	2010	All Ages	3.6 (2.7 to 4.5)	15.0% (11.4 to 18.7%)	7.4 (5.6 to 9.3)
Ischemic heart disease	South Korea	2015	All Ages	4.6 (3.3 to 5.9)	15.2% (11.1 to 19.3%)	9.1 (6.6 to 11.6)
Ischemic heart disease	South Korea	1990	Age-standardized		15.3% (11.7 to 18.8%)	26.0 (20.2 to 32.3)
Ischemic heart disease	South Korea	1995	Age-standardized		15.0% (11.3 to 18.7%)	15.9 (11.9 to 20.1)
Ischemic heart disease	South Korea	2000	Age-standardized		14.9% (11.2 to 18.7%)	10.5 (7.9 to 13.3)
Ischemic heart disease	South Korea	2005	Age-standardized		14.9% (11.1 to 18.8%)	8.2 (6.0 to 10.4)
Ischemic heart disease	South Korea	2010	Age-standardized		14.6% (10.6 to 18.5%)	7.0 (5.1 to 8.9)
Ischemic heart disease	South Korea	2015	Age-standardized		15.1% (10.9 to 19.3%)	6.9 (4.9 to 8.8)
Ischemic stroke	South Korea	1990	All Ages	3.1 (2.3 to 3.9)	11.9% (8.7 to 14.9%)	7.3 (5.3 to 9.1)
Ischemic stroke	South Korea	1995	All Ages	3.1 (2.2 to 3.9)	11.6% (8.4 to 14.9%)	6.9 (5.0 to 8.8)
Ischemic stroke	South Korea	2000	All Ages	2.9 (2.1 to 3.8)	11.4% (8.1 to 14.8%)	6.4 (4.5 to 8.3)
Ischemic stroke	South Korea	2005	All Ages	2.7 (1.9 to 3.6)	11.3% (7.9 to 14.8%)	5.7 (4.0 to 7.5)
Ischemic stroke	South Korea	2010	All Ages	2.4 (1.6 to 3.3)	10.8% (7.3 to 14.8%)	5.0 (3.3 to 6.8)
Ischemic stroke	South Korea	2015	All Ages	3.1 (2.0 to 4.3)	11.2% (7.4 to 15.5%)	6.1 (4.1 to 8.6)
Ischemic stroke	South Korea	1990	Age-standardized		11.2% (7.8 to 14.7%)	15.9 (11.0 to 20.8)
Ischemic stroke	South Korea	1995	Age-standardized		11.0% (7.6 to 14.8%)	12.9 (8.9 to 17.2)
Ischemic stroke	South Korea	2000	Age-standardized		10.9% (7.4 to 14.9%)	10.0 (6.7 to 13.5)
Ischemic stroke	South Korea	2005	Age-standardized		10.9% (7.4 to 15.0%)	6.9 (4.7 to 9.5)
Ischemic stroke	South Korea	2010	Age-standardized		10.6% (6.9 to 15.0%)	4.9 (3.2 to 6.9)

Cause name	Location	Year	Age group	Number (in thousands)	PAF	Rate (per 100,000)
Ischemic stroke	South Korea	2015	Age-standardized		11.1% (7.3 to 15.6%)	4.7 (3.1 to 6.5)
Hemorrhagic stroke	South Korea	1990	All Ages	3.2 (2.5 to 3.8)	14.0% (11.2 to 16.8%)	7.4 (5.9 to 8.9)
Hemorrhagic stroke	South Korea	1995	All Ages	3.1 (2.4 to 3.7)	13.6% (10.8 to 16.5%)	6.9 (5.4 to 8.3)
Hemorrhagic stroke	South Korea	2000	All Ages	2.7 (2.1 to 3.2)	13.4% (10.5 to 16.2%)	5.8 (4.6 to 7.0)
Hemorrhagic stroke	South Korea	2005	All Ages	2.1 (1.7 to 2.6)	13.3% (10.5 to 16.1%)	4.5 (3.5 to 5.4)
Hemorrhagic stroke	South Korea	2010	All Ages	1.8 (1.4 to 2.2)	12.7% (9.9 to 15.7%)	3.7 (2.9 to 4.5)
Hemorrhagic stroke	South Korea	2015	All Ages	2.1 (1.6 to 2.7)	13.0% (9.9 to 16.1%)	4.3 (3.3 to 5.4)
Hemorrhagic stroke	South Korea	1990	Age-standardized		12.8% (10.0 to 15.6%)	12.4 (9.6 to 15.2)
Hemorrhagic stroke	South Korea	1995	Age-standardized		12.6% (9.7 to 15.4%)	10.2 (7.8 to 12.6)
Hemorrhagic stroke	South Korea	2000	Age-standardized		12.5% (9.5 to 15.6%)	7.5 (5.7 to 9.3)
Hemorrhagic stroke	South Korea	2005	Age-standardized		12.5% (9.6 to 15.6%)	4.7 (3.6 to 5.8)
Hemorrhagic stroke	South Korea	2010	Age-standardized		12.3% (9.3 to 15.4%)	3.3 (2.5 to 4.1)
Hemorrhagic stroke	South Korea	2015	Age-standardized		12.9% (9.8 to 16.0%)	3.2 (2.4 to 4.0)
Chronic obstructive pulmonary disease	South Korea	1990	All Ages	0.9 (0.5 to 1.4)	20.7% (10.4 to 31.6%)	2.2 (1.1 to 3.3)
Chronic obstructive pulmonary disease	South Korea	1995	All Ages	1.0 (0.5 to 1.4)	20.8% (10.4 to 31.8%)	2.1 (1.1 to 3.2)
Chronic obstructive pulmonary disease	South Korea	2000	All Ages	1.1 (0.6 to 1.7)	21.0% (10.7 to 32.0%)	2.5 (1.2 to 3.7)
Chronic obstructive pulmonary disease	South Korea	2005	All Ages	1.3 (0.6 to 2.0)	21.1% (10.6 to 32.1%)	2.7 (1.3 to 4.2)
Chronic obstructive pulmonary disease	South Korea	2010	All Ages	1.5 (0.7 to 2.3)	20.7% (10.4 to 31.6%)	3.0 (1.5 to 4.6)
Chronic obstructive pulmonary disease	South Korea	2015	All Ages	2.1 (1.1 to 3.2)	22.3% (11.7 to 33.4%)	4.2 (2.2 to 6.3)
Chronic obstructive pulmonary disease	South Korea	1990	Age-standardized		20.8% (10.4 to 31.8%)	5.1 (2.5 to 7.9)
Chronic obstructive pulmonary disease	South Korea	1995	Age-standardized		20.9% (10.4 to 31.9%)	4.1 (2.0 to 6.2)
Chronic obstructive pulmonary disease	South Korea	2000	Age-standardized		21.0% (10.7 to 32.1%)	3.9 (2.0 to 5.9)
Chronic obstructive pulmonary disease	South Korea	2005	Age-standardized		21.1% (10.6 to 32.1%)	3.4 (1.6 to 5.1)
Chronic obstructive pulmonary disease	South Korea	2010	Age-standardized		20.7% (10.4 to 31.6%)	3.0 (1.4 to 4.5)
Chronic obstructive pulmonary disease	South Korea	2015	Age-standardized		22.3% (11.6 to 33.4%)	3.2 (1.6 to 4.8)
All causes	Andorra	1990	All Ages	0.0 (0.0 to 0.0)	4.3% (3.1 to 5.6%)	27.3 (19.4 to 35.8)
All causes	Andorra	1995	All Ages	0.0 (0.0 to 0.0)	4.1% (2.8 to 5.5%)	26.1 (17.6 to 34.8)
All causes	Andorra	2000	All Ages	0.0 (0.0 to 0.0)	3.9% (2.7 to 5.2%)	23.9 (16.6 to 31.7)
All causes	Andorra	2005	All Ages	0.0 (0.0 to 0.0)	3.8% (2.7 to 5.1%)	23.8 (16.6 to 32.1)
All causes	Andorra	2010	All Ages	0.0 (0.0 to 0.0)	3.8% (2.6 to 5.1%)	24.8 (17.1 to 33.4)
All causes	Andorra	2015	All Ages	0.0 (0.0 to 0.0)	3.6% (2.4 to 5.1%)	27.9 (18.6 to 39.1)
All causes	Andorra	1990	Age-standardized		4.2% (3.0 to 5.4%)	18.9 (13.5 to 24.6)
All causes	Andorra	1995	Age-standardized		4.0% (2.8 to 5.3%)	16.9 (11.6 to 22.4)
All causes	Andorra	2000	Age-standardized		3.8% (2.7 to 5.1%)	15.0 (10.3 to 19.8)
All causes	Andorra	2005	Age-standardized		3.7% (2.6 to 4.9%)	13.5 (9.5 to 18.1)
All causes	Andorra	2010	Age-standardized		3.7% (2.6 to 4.9%)	12.8 (9.1 to 17.1)

Cause name	Location	Year	Age group	Number (in thousands)	PAF	Rate (per 100,000)
All causes	Andorra	2015	Age-standardized		3.5% (2.4 to 4.8%)	12.5 (8.5 to 17.1)
Lower respiratory infections	Andorra	1990	All Ages	0.0 (0.0 to 0.0)	9.8% (2.3 to 21.3%)	3.2 (0.7 to 6.9)
Lower respiratory infections	Andorra	1995	All Ages	0.0 (0.0 to 0.0)	9.5% (2.3 to 21.1%)	3.2 (0.7 to 7.3)
Lower respiratory infections	Andorra	2000	All Ages	0.0 (0.0 to 0.0)	9.3% (2.2 to 20.6%)	3.0 (0.6 to 7.0)
Lower respiratory infections	Andorra	2005	All Ages	0.0 (0.0 to 0.0)	9.4% (2.1 to 20.8%)	3.2 (0.7 to 7.3)
Lower respiratory infections	Andorra	2010	All Ages	0.0 (0.0 to 0.0)	9.4% (2.3 to 21.3%)	3.4 (0.8 to 7.8)
Lower respiratory infections	Andorra	2015	All Ages	0.0 (0.0 to 0.0)	8.5% (1.9 to 19.2%)	3.6 (0.7 to 8.7)
Lower respiratory infections	Andorra	1990	Age-standardized		9.8% (2.3 to 21.3%)	2.1 (0.5 to 4.6)
Lower respiratory infections	Andorra	1995	Age-standardized		9.5% (2.3 to 21.1%)	2.0 (0.4 to 4.5)
Lower respiratory infections	Andorra	2000	Age-standardized		9.3% (2.2 to 20.6%)	1.8 (0.4 to 4.2)
Lower respiratory infections	Andorra	2005	Age-standardized		9.4% (2.1 to 20.8%)	1.7 (0.3 to 3.9)
Lower respiratory infections	Andorra	2010	Age-standardized		9.4% (2.3 to 21.3%)	1.6 (0.4 to 3.7)
Lower respiratory infections	Andorra	2015	Age-standardized		8.5% (1.9 to 19.2%)	1.4 (0.3 to 3.5)
Tracheal, bronchus, and lung cancer	Andorra	1990	All Ages	0.0 (0.0 to 0.0)	6.6% (2.8 to 11.7%)	2.2 (0.9 to 4.0)
Tracheal, bronchus, and lung cancer	Andorra	1995	All Ages	0.0 (0.0 to 0.0)	6.4% (2.6 to 11.7%)	2.1 (0.8 to 4.0)
Tracheal, bronchus, and lung cancer	Andorra	2000	All Ages	0.0 (0.0 to 0.0)	6.3% (2.7 to 11.1%)	2.2 (0.9 to 3.8)
Tracheal, bronchus, and lung cancer	Andorra	2005	All Ages	0.0 (0.0 to 0.0)	6.4% (2.7 to 11.6%)	2.3 (1.0 to 4.3)
Tracheal, bronchus, and lung cancer	Andorra	2010	All Ages	0.0 (0.0 to 0.0)	6.4% (2.5 to 11.6%)	2.4 (0.9 to 4.5)
Tracheal, bronchus, and lung cancer	Andorra	2015	All Ages	0.0 (0.0 to 0.0)	5.8% (2.1 to 10.7%)	2.5 (0.9 to 4.7)
Tracheal, bronchus, and lung cancer	Andorra	1990	Age-standardized		6.6% (2.8 to 11.7%)	1.6 (0.7 to 3.0)
Tracheal, bronchus, and lung cancer	Andorra	1995	Age-standardized		6.4% (2.6 to 11.7%)	1.5 (0.6 to 2.8)
Tracheal, bronchus, and lung cancer	Andorra	2000	Age-standardized		6.3% (2.7 to 11.1%)	1.4 (0.6 to 2.5)
Tracheal, bronchus, and lung cancer	Andorra	2005	Age-standardized		6.4% (2.7 to 11.6%)	1.4 (0.6 to 2.7)
Tracheal, bronchus, and lung cancer	Andorra	2010	Age-standardized		6.4% (2.5 to 11.6%)	1.4 (0.5 to 2.6)
Tracheal, bronchus, and lung cancer	Andorra	2015	Age-standardized		5.8% (2.1 to 10.7%)	1.3 (0.5 to 2.5)
Cardiovascular diseases	Andorra	1990	All Ages	0.0 (0.0 to 0.0)	7.9% (5.7 to 10.4%)	18.6 (12.9 to 24.8)
Cardiovascular diseases	Andorra	1995	All Ages	0.0 (0.0 to 0.0)	7.6% (5.2 to 10.1%)	17.4 (11.5 to 23.5)
Cardiovascular diseases	Andorra	2000	All Ages	0.0 (0.0 to 0.0)	7.4% (5.1 to 9.9%)	15.4 (10.3 to 20.8)
Cardiovascular diseases	Andorra	2005	All Ages	0.0 (0.0 to 0.0)	7.4% (5.1 to 9.9%)	14.6 (9.8 to 19.9)
Cardiovascular diseases	Andorra	2010	All Ages	0.0 (0.0 to 0.0)	7.2% (4.8 to 9.8%)	15.2 (10.2 to 20.9)
Cardiovascular diseases	Andorra	2015	All Ages	0.0 (0.0 to 0.0)	6.8% (4.3 to 9.5%)	17.5 (11.2 to 25.3)
Cardiovascular diseases	Andorra	1990	Age-standardized		8.1% (5.9 to 10.5%)	12.9 (9.1 to 17.0)
Cardiovascular diseases	Andorra	1995	Age-standardized		7.8% (5.5 to 10.3%)	11.4 (7.8 to 15.1)
Cardiovascular diseases	Andorra	2000	Age-standardized		7.6% (5.3 to 10.1%)	9.7 (6.7 to 13.0)
Cardiovascular diseases	Andorra	2005	Age-standardized		7.6% (5.4 to 10.1%)	8.4 (5.9 to 11.2)
Cardiovascular diseases	Andorra	2010	Age-standardized		7.5% (5.4 to 9.9%)	7.9 (5.5 to 10.6)

Cause name	Location	Year	Age group	Number (in thousands)	PAF	Rate (per 100,000)
Cardiovascular diseases	Andorra	2015	Age-standardized		7.2% (4.9 to 9.7%)	7.9 (5.4 to 10.9)
Ischemic heart disease	Andorra	1990	All Ages	0.0 (0.0 to 0.0)	10.7% (7.2 to 14.3%)	13.5 (9.0 to 18.3)
Ischemic heart disease	Andorra	1995	All Ages	0.0 (0.0 to 0.0)	10.4% (6.6 to 14.3%)	12.7 (7.9 to 17.7)
Ischemic heart disease	Andorra	2000	All Ages	0.0 (0.0 to 0.0)	10.3% (6.8 to 14.0%)	11.3 (7.3 to 15.8)
Ischemic heart disease	Andorra	2005	All Ages	0.0 (0.0 to 0.0)	10.2% (6.5 to 14.2%)	10.9 (6.9 to 15.2)
Ischemic heart disease	Andorra	2010	All Ages	0.0 (0.0 to 0.0)	10.1% (6.5 to 13.9%)	11.3 (7.1 to 16.2)
Ischemic heart disease	Andorra	2015	All Ages	0.0 (0.0 to 0.0)	9.5% (5.6 to 13.7%)	13.1 (7.7 to 19.4)
Ischemic heart disease	Andorra	1990	Age-standardized		11.0% (7.6 to 14.4%)	9.5 (6.5 to 12.7)
Ischemic heart disease	Andorra	1995	Age-standardized		10.8% (7.0 to 14.4%)	8.4 (5.4 to 11.4)
Ischemic heart disease	Andorra	2000	Age-standardized		10.6% (7.2 to 14.1%)	7.2 (4.8 to 9.9)
Ischemic heart disease	Andorra	2005	Age-standardized		10.6% (7.0 to 14.1%)	6.3 (4.2 to 8.7)
Ischemic heart disease	Andorra	2010	Age-standardized		10.5% (7.1 to 14.0%)	6.0 (4.0 to 8.2)
Ischemic heart disease	Andorra	2015	Age-standardized		10.0% (6.4 to 13.6%)	6.1 (3.8 to 8.5)
Ischemic stroke	Andorra	1990	All Ages	0.0 (0.0 to 0.0)	7.0% (3.4 to 11.6%)	2.8 (1.4 to 4.8)
Ischemic stroke	Andorra	1995	All Ages	0.0 (0.0 to 0.0)	6.8% (3.2 to 11.9%)	2.6 (1.2 to 4.7)
Ischemic stroke	Andorra	2000	All Ages	0.0 (0.0 to 0.0)	6.8% (3.2 to 11.7%)	2.3 (1.0 to 4.0)
Ischemic stroke	Andorra	2005	All Ages	0.0 (0.0 to 0.0)	6.8% (2.9 to 12.0%)	2.1 (0.9 to 3.8)
Ischemic stroke	Andorra	2010	All Ages	0.0 (0.0 to 0.0)	6.7% (2.8 to 12.0%)	2.1 (0.9 to 3.9)
Ischemic stroke	Andorra	2015	All Ages	0.0 (0.0 to 0.0)	6.3% (2.5 to 12.0%)	2.5 (0.9 to 4.8)
Ischemic stroke	Andorra	1990	Age-standardized		7.1% (3.6 to 11.5%)	1.9 (0.9 to 3.1)
Ischemic stroke	Andorra	1995	Age-standardized		6.9% (3.4 to 11.7%)	1.6 (0.8 to 2.8)
Ischemic stroke	Andorra	2000	Age-standardized		6.8% (3.3 to 11.7%)	1.4 (0.6 to 2.4)
Ischemic stroke	Andorra	2005	Age-standardized		6.9% (3.1 to 11.9%)	1.1 (0.5 to 2.0)
Ischemic stroke	Andorra	2010	Age-standardized		6.8% (3.0 to 11.9%)	1.0 (0.5 to 1.8)
Ischemic stroke	Andorra	2015	Age-standardized		6.5% (2.9 to 11.7%)	1.0 (0.4 to 1.9)
Hemorrhagic stroke	Andorra	1990	All Ages	0.0 (0.0 to 0.0)	7.6% (4.4 to 11.5%)	2.2 (1.2 to 3.6)
Hemorrhagic stroke	Andorra	1995	All Ages	0.0 (0.0 to 0.0)	7.4% (4.1 to 11.4%)	2.1 (1.1 to 3.5)
Hemorrhagic stroke	Andorra	2000	All Ages	0.0 (0.0 to 0.0)	7.2% (4.0 to 11.2%)	1.8 (0.9 to 3.0)
Hemorrhagic stroke	Andorra	2005	All Ages	0.0 (0.0 to 0.0)	7.2% (3.8 to 11.7%)	1.7 (0.9 to 2.8)
Hemorrhagic stroke	Andorra	2010	All Ages	0.0 (0.0 to 0.0)	7.1% (3.6 to 11.6%)	1.7 (0.8 to 3.0)
Hemorrhagic stroke	Andorra	2015	All Ages	0.0 (0.0 to 0.0)	6.6% (3.2 to 11.5%)	2.0 (0.9 to 3.5)
Hemorrhagic stroke	Andorra	1990	Age-standardized		7.7% (4.7 to 11.5%)	1.5 (0.9 to 2.5)
Hemorrhagic stroke	Andorra	1995	Age-standardized		7.5% (4.4 to 11.3%)	1.4 (0.8 to 2.2)
Hemorrhagic stroke	Andorra	2000	Age-standardized		7.4% (4.2 to 11.2%)	1.2 (0.6 to 1.9)
Hemorrhagic stroke	Andorra	2005	Age-standardized		7.4% (4.1 to 11.6%)	1.0 (0.5 to 1.6)
Hemorrhagic stroke	Andorra	2010	Age-standardized		7.4% (4.1 to 11.4%)	0.9 (0.5 to 1.5)

Cause name	Location	Year	Age group	Number (in thousands)	PAF	Rate (per 100,000)
Hemorrhagic stroke	Andorra	2015	Age-standardized		7.0% (3.7 to 11.2%)	0.9 (0.5 to 1.5)
Chronic obstructive pulmonary disease	Andorra	1990	All Ages	0.0 (0.0 to 0.0)	12.0% (3.7 to 22.0%)	3.3 (1.0 to 6.5)
Chronic obstructive pulmonary disease	Andorra	1995	All Ages	0.0 (0.0 to 0.0)	11.7% (4.0 to 21.2%)	3.3 (1.1 to 6.4)
Chronic obstructive pulmonary disease	Andorra	2000	All Ages	0.0 (0.0 to 0.0)	11.5% (3.7 to 21.7%)	3.4 (1.0 to 6.6)
Chronic obstructive pulmonary disease	Andorra	2005	All Ages	0.0 (0.0 to 0.0)	11.6% (3.8 to 21.8%)	3.7 (1.2 to 7.1)
Chronic obstructive pulmonary disease	Andorra	2010	All Ages	0.0 (0.0 to 0.0)	11.6% (3.9 to 21.7%)	3.8 (1.2 to 7.3)
Chronic obstructive pulmonary disease	Andorra	2015	All Ages	0.0 (0.0 to 0.0)	10.8% (3.2 to 20.4%)	4.3 (1.3 to 8.6)
Chronic obstructive pulmonary disease	Andorra	1990	Age-standardized		12.0% (3.7 to 22.0%)	2.2 (0.7 to 4.4)
Chronic obstructive pulmonary disease	Andorra	1995	Age-standardized		11.7% (4.0 to 21.1%)	2.1 (0.7 to 4.0)
Chronic obstructive pulmonary disease	Andorra	2000	Age-standardized		11.5% (3.7 to 21.7%)	2.1 (0.6 to 4.0)
Chronic obstructive pulmonary disease	Andorra	2005	Age-standardized		11.6% (3.8 to 21.8%)	2.0 (0.6 to 3.9)
Chronic obstructive pulmonary disease	Andorra	2010	Age-standardized		11.6% (3.9 to 21.7%)	1.9 (0.6 to 3.6)
Chronic obstructive pulmonary disease	Andorra	2015	Age-standardized		10.8% (3.2 to 20.4%)	1.8 (0.6 to 3.6)
All causes	Austria	1990	All Ages	4.9 (4.0 to 5.9)	5.9% (4.8 to 7.0%)	63.9 (52.6 to 76.3)
All causes	Austria	1995	All Ages	4.5 (3.6 to 5.5)	5.6% (4.5 to 6.8%)	57.4 (46.0 to 69.6)
All causes	Austria	2000	All Ages	4.2 (3.3 to 5.1)	5.4% (4.2 to 6.6%)	52.3 (41.2 to 64.2)
All causes	Austria	2005	All Ages	3.7 (2.9 to 4.6)	4.9% (3.8 to 6.1%)	44.9 (35.1 to 56.4)
All causes	Austria	2010	All Ages	3.6 (2.7 to 4.5)	4.6% (3.5 to 5.8%)	42.3 (32.3 to 53.3)
All causes	Austria	2015	All Ages	4.0 (3.1 to 5.0)	4.8% (3.6 to 6.0%)	45.8 (35.2 to 57.5)
All causes	Austria	1990	Age-standardized		5.7% (4.8 to 6.8%)	43.7 (36.4 to 51.6)
All causes	Austria	1995	Age-standardized		5.5% (4.5 to 6.6%)	38.6 (31.6 to 46.3)
All causes	Austria	2000	Age-standardized		5.2% (4.2 to 6.4%)	34.4 (27.5 to 42.0)
All causes	Austria	2005	Age-standardized		4.7% (3.8 to 5.8%)	26.6 (21.2 to 32.8)
All causes	Austria	2010	Age-standardized		4.4% (3.5 to 5.5%)	23.1 (18.3 to 28.5)
All causes	Austria	2015	Age-standardized		4.6% (3.6 to 5.7%)	22.9 (18.2 to 28.1)
Lower respiratory infections	Austria	1990	All Ages	0.3 (0.1 to 0.4)	16.1% (7.6 to 26.4%)	3.3 (1.6 to 5.5)
Lower respiratory infections	Austria	1995	All Ages	0.2 (0.1 to 0.4)	14.3% (6.0 to 24.7%)	2.6 (1.1 to 4.5)
Lower respiratory infections	Austria	2000	All Ages	0.2 (0.1 to 0.3)	13.2% (5.0 to 24.0%)	2.3 (0.9 to 4.3)
Lower respiratory infections	Austria	2005	All Ages	0.2 (0.1 to 0.3)	13.3% (5.1 to 24.2%)	2.2 (0.9 to 4.1)
Lower respiratory infections	Austria	2010	All Ages	0.2 (0.1 to 0.3)	13.1% (5.0 to 23.9%)	2.0 (0.7 to 3.7)
Lower respiratory infections	Austria	2015	All Ages	0.2 (0.1 to 0.3)	14.6% (6.3 to 25.1%)	2.2 (0.9 to 3.9)
Lower respiratory infections	Austria	1990	Age-standardized		16.1% (7.6 to 26.4%)	2.2 (1.1 to 3.7)
Lower respiratory infections	Austria	1995	Age-standardized		14.3% (6.0 to 24.7%)	1.6 (0.7 to 2.9)
Lower respiratory infections	Austria	2000	Age-standardized		13.2% (5.0 to 24.0%)	1.5 (0.6 to 2.8)
Lower respiratory infections	Austria	2005	Age-standardized		13.3% (5.1 to 24.2%)	1.3 (0.5 to 2.3)
Lower respiratory infections	Austria	2010	Age-standardized		13.1% (5.0 to 23.9%)	1.0 (0.4 to 1.9)

Cause name	Location	Year	Age group	Number (in thousands)	PAF	Rate (per 100,000)
Lower respiratory infections	Austria	2015	Age-standardized		14.6% (6.3 to 25.1%)	1.0 (0.4 to 1.8)
Tracheal, bronchus, and lung cancer	Austria	1990	All Ages	0.4 (0.2 to 0.6)	11.3% (6.1 to 17.5%)	4.7 (2.5 to 7.3)
Tracheal, bronchus, and lung cancer	Austria	1995	All Ages	0.3 (0.2 to 0.5)	9.9% (5.1 to 15.7%)	4.1 (2.1 to 6.5)
Tracheal, bronchus, and lung cancer	Austria	2000	All Ages	0.3 (0.2 to 0.5)	9.2% (4.6 to 14.9%)	3.9 (2.0 to 6.3)
Tracheal, bronchus, and lung cancer	Austria	2005	All Ages	0.3 (0.2 to 0.5)	9.2% (4.7 to 14.8%)	4.0 (2.0 to 6.3)
Tracheal, bronchus, and lung cancer	Austria	2010	All Ages	0.4 (0.2 to 0.6)	9.1% (4.6 to 14.5%)	4.1 (2.1 to 6.6)
Tracheal, bronchus, and lung cancer	Austria	2015	All Ages	0.4 (0.2 to 0.7)	10.2% (5.3 to 16.1%)	4.8 (2.5 to 7.6)
Tracheal, bronchus, and lung cancer	Austria	1990	Age-standardized		11.3% (6.1 to 17.5%)	3.5 (1.9 to 5.4)
Tracheal, bronchus, and lung cancer	Austria	1995	Age-standardized		9.9% (5.1 to 15.7%)	3.0 (1.6 to 4.8)
Tracheal, bronchus, and lung cancer	Austria	2000	Age-standardized		9.1% (4.6 to 14.9%)	2.7 (1.4 to 4.4)
Tracheal, bronchus, and lung cancer	Austria	2005	Age-standardized		9.2% (4.7 to 14.8%)	2.7 (1.3 to 4.3)
Tracheal, bronchus, and lung cancer	Austria	2010	Age-standardized		9.1% (4.6 to 14.5%)	2.6 (1.3 to 4.2)
Tracheal, bronchus, and lung cancer	Austria	2015	Age-standardized		10.2% (5.3 to 16.1%)	2.8 (1.5 to 4.4)
Cardiovascular diseases	Austria	1990	All Ages	3.9 (3.0 to 4.7)	9.5% (7.5 to 11.5%)	50.6 (39.7 to 61.3)
Cardiovascular diseases	Austria	1995	All Ages	3.6 (2.8 to 4.5)	9.1% (6.9 to 11.3%)	45.9 (35.0 to 57.0)
Cardiovascular diseases	Austria	2000	All Ages	3.3 (2.5 to 4.1)	8.9% (6.7 to 11.1%)	40.8 (30.8 to 51.2)
Cardiovascular diseases	Austria	2005	All Ages	2.7 (2.0 to 3.5)	8.5% (6.2 to 10.9%)	33.4 (24.5 to 43.0)
Cardiovascular diseases	Austria	2010	All Ages	2.6 (1.8 to 3.4)	8.2% (5.8 to 10.6%)	30.8 (22.0 to 40.1)
Cardiovascular diseases	Austria	2015	All Ages	2.8 (2.0 to 3.7)	8.4% (6.0 to 11.0%)	32.8 (23.2 to 42.9)
Cardiovascular diseases	Austria	1990	Age-standardized		9.8% (7.8 to 11.7%)	34.5 (27.6 to 41.5)
Cardiovascular diseases	Austria	1995	Age-standardized		9.5% (7.5 to 11.5%)	30.8 (24.2 to 37.4)
Cardiovascular diseases	Austria	2000	Age-standardized		9.1% (6.9 to 11.3%)	26.8 (20.5 to 33.3)
Cardiovascular diseases	Austria	2005	Age-standardized		8.8% (6.6 to 11.0%)	19.6 (14.8 to 24.7)
Cardiovascular diseases	Austria	2010	Age-standardized		8.6% (6.4 to 10.8%)	16.6 (12.3 to 21.0)
Cardiovascular diseases	Austria	2015	Age-standardized		8.8% (6.6 to 11.1%)	16.1 (12.0 to 20.4)
Ischemic heart disease	Austria	1990	All Ages	2.8 (2.1 to 3.5)	13.4% (9.9 to 17.0%)	36.4 (27.1 to 46.2)
Ischemic heart disease	Austria	1995	All Ages	2.8 (2.0 to 3.5)	12.6% (9.0 to 16.2%)	34.8 (24.8 to 44.5)
Ischemic heart disease	Austria	2000	All Ages	2.5 (1.8 to 3.3)	12.0% (8.5 to 15.5%)	31.8 (22.3 to 41.2)
Ischemic heart disease	Austria	2005	All Ages	2.2 (1.5 to 2.9)	11.8% (7.9 to 15.6%)	26.6 (17.8 to 35.3)
Ischemic heart disease	Austria	2010	All Ages	2.1 (1.4 to 2.8)	11.6% (7.6 to 15.6%)	24.9 (16.1 to 33.7)
Ischemic heart disease	Austria	2015	All Ages	2.3 (1.5 to 3.1)	12.0% (7.7 to 16.4%)	26.6 (17.0 to 35.7)
Ischemic heart disease	Austria	1990	Age-standardized		13.8% (10.6 to 17.2%)	25.2 (19.2 to 31.2)
Ischemic heart disease	Austria	1995	Age-standardized		13.0% (9.7 to 16.3%)	23.5 (17.5 to 29.4)
Ischemic heart disease	Austria	2000	Age-standardized		12.3% (8.8 to 15.8%)	21.0 (15.0 to 27.0)
Ischemic heart disease	Austria	2005	Age-standardized		12.2% (8.6 to 15.7%)	15.7 (11.0 to 20.3)
Ischemic heart disease	Austria	2010	Age-standardized		12.1% (8.4 to 15.8%)	13.5 (9.3 to 17.4)

Cause name	Location	Year	Age group	Number (in thousands)	PAF	Rate (per 100,000)
Ischemic heart disease	Austria	2015	Age-standardized		12.5% (8.7 to 16.3%)	13.1 (9.0 to 17.0)
Ischemic stroke	Austria	1990	All Ages	0.7 (0.4 to 1.0)	9.3% (5.7 to 13.8%)	8.8 (5.4 to 13.1)
Ischemic stroke	Austria	1995	All Ages	0.5 (0.3 to 0.8)	8.7% (4.8 to 13.3%)	6.6 (3.7 to 10.3)
Ischemic stroke	Austria	2000	All Ages	0.4 (0.2 to 0.7)	8.3% (4.6 to 12.9%)	5.3 (2.9 to 8.2)
Ischemic stroke	Austria	2005	All Ages	0.3 (0.2 to 0.5)	8.2% (4.3 to 13.1%)	3.9 (2.0 to 6.2)
Ischemic stroke	Austria	2010	All Ages	0.3 (0.1 to 0.5)	8.1% (4.2 to 13.2%)	3.3 (1.7 to 5.3)
Ischemic stroke	Austria	2015	All Ages	0.3 (0.1 to 0.5)	8.6% (4.3 to 13.8%)	3.5 (1.7 to 5.6)
Ischemic stroke	Austria	1990	Age-standardized		9.5% (5.9 to 13.7%)	5.7 (3.6 to 8.2)
Ischemic stroke	Austria	1995	Age-standardized		8.8% (5.2 to 13.1%)	4.2 (2.5 to 6.3)
Ischemic stroke	Austria	2000	Age-standardized		8.3% (4.6 to 12.9%)	3.3 (1.8 to 5.2)
Ischemic stroke	Austria	2005	Age-standardized		8.4% (4.6 to 13.0%)	2.1 (1.2 to 3.3)
Ischemic stroke	Austria	2010	Age-standardized		8.3% (4.5 to 13.0%)	1.7 (0.9 to 2.6)
Ischemic stroke	Austria	2015	Age-standardized		8.8% (4.9 to 13.5%)	1.6 (0.9 to 2.5)
Hemorrhagic stroke	Austria	1990	All Ages	0.4 (0.3 to 0.6)	10.2% (7.1 to 13.7%)	5.3 (3.7 to 7.2)
Hemorrhagic stroke	Austria	1995	All Ages	0.4 (0.2 to 0.5)	9.6% (6.4 to 13.2%)	4.5 (3.0 to 6.3)
Hemorrhagic stroke	Austria	2000	All Ages	0.3 (0.2 to 0.4)	9.1% (6.0 to 12.6%)	3.8 (2.5 to 5.4)
Hemorrhagic stroke	Austria	2005	All Ages	0.2 (0.1 to 0.4)	9.0% (5.8 to 12.8%)	2.9 (1.9 to 4.2)
Hemorrhagic stroke	Austria	2010	All Ages	0.2 (0.1 to 0.3)	8.8% (5.5 to 13.0%)	2.6 (1.6 to 3.8)
Hemorrhagic stroke	Austria	2015	All Ages	0.2 (0.1 to 0.4)	9.3% (5.8 to 13.5%)	2.8 (1.7 to 4.1)
Hemorrhagic stroke	Austria	1990	Age-standardized		10.6% (7.6 to 13.8%)	3.7 (2.6 to 4.9)
Hemorrhagic stroke	Austria	1995	Age-standardized		9.9% (6.9 to 13.1%)	3.1 (2.2 to 4.1)
Hemorrhagic stroke	Austria	2000	Age-standardized		9.3% (6.3 to 12.8%)	2.6 (1.8 to 3.6)
Hemorrhagic stroke	Austria	2005	Age-standardized		9.3% (6.3 to 12.8%)	1.8 (1.2 to 2.4)
Hemorrhagic stroke	Austria	2010	Age-standardized		9.2% (6.2 to 12.8%)	1.5 (1.0 to 2.0)
Hemorrhagic stroke	Austria	2015	Age-standardized		9.7% (6.5 to 13.3%)	1.4 (0.9 to 2.0)
Chronic obstructive pulmonary disease	Austria	1990	All Ages	0.4 (0.2 to 0.6)	17.6% (8.1 to 28.3%)	5.3 (2.5 to 8.4)
Chronic obstructive pulmonary disease	Austria	1995	All Ages	0.4 (0.2 to 0.6)	16.0% (7.1 to 26.6%)	4.9 (2.2 to 8.1)
Chronic obstructive pulmonary disease	Austria	2000	All Ages	0.4 (0.2 to 0.7)	15.1% (6.5 to 25.5%)	5.2 (2.2 to 8.7)
Chronic obstructive pulmonary disease	Austria	2005	All Ages	0.4 (0.2 to 0.7)	15.2% (6.5 to 25.6%)	5.4 (2.3 to 9.0)
Chronic obstructive pulmonary disease	Austria	2010	All Ages	0.5 (0.2 to 0.8)	15.1% (6.4 to 25.5%)	5.4 (2.2 to 9.0)
Chronic obstructive pulmonary disease	Austria	2015	All Ages	0.5 (0.2 to 0.9)	16.3% (7.3 to 26.9%)	6.1 (2.6 to 10.0)
Chronic obstructive pulmonary disease	Austria	1990	Age-standardized		17.5% (8.1 to 28.3%)	3.5 (1.6 to 5.5)
Chronic obstructive pulmonary disease	Austria	1995	Age-standardized		16.0% (7.1 to 26.6%)	3.2 (1.4 to 5.3)
Chronic obstructive pulmonary disease	Austria	2000	Age-standardized		15.1% (6.5 to 25.5%)	3.3 (1.4 to 5.6)
Chronic obstructive pulmonary disease	Austria	2005	Age-standardized		15.2% (6.5 to 25.6%)	3.1 (1.3 to 5.2)
Chronic obstructive pulmonary disease	Austria	2010	Age-standardized		15.0% (6.4 to 25.5%)	2.9 (1.2 to 4.8)

Cause name	Location	Year	Age group	Number (in thousands)	PAF	Rate (per 100,000)
Chronic obstructive pulmonary disease	Austria	2015	Age-standardized		16.3% (7.3 to 26.9%)	3.0 (1.3 to 4.9)
All causes	Belgium	1990	All Ages	6.8 (5.7 to 8.1)	6.5% (5.4 to 7.7%)	68.5 (56.8 to 81.3)
All causes	Belgium	1995	All Ages	6.3 (5.1 to 7.6)	6.0% (4.9 to 7.2%)	62.2 (50.8 to 74.7)
All causes	Belgium	2000	All Ages	6.0 (4.9 to 7.3)	5.7% (4.6 to 6.9%)	58.8 (47.4 to 71.7)
All causes	Belgium	2005	All Ages	5.5 (4.3 to 6.8)	5.3% (4.2 to 6.6%)	52.2 (41.3 to 64.5)
All causes	Belgium	2010	All Ages	5.3 (4.2 to 6.6)	5.0% (4.0 to 6.3%)	48.7 (38.5 to 60.8)
All causes	Belgium	2015	All Ages	5.6 (4.4 to 7.0)	4.9% (3.8 to 6.1%)	49.3 (38.6 to 61.9)
All causes	Belgium	1990	Age-standardized		6.3% (5.3 to 7.5%)	47.2 (39.3 to 55.6)
All causes	Belgium	1995	Age-standardized		5.8% (4.8 to 6.9%)	40.8 (33.6 to 48.5)
All causes	Belgium	2000	Age-standardized		5.5% (4.5 to 6.7%)	37.2 (30.3 to 45.3)
All causes	Belgium	2005	Age-standardized		5.2% (4.2 to 6.3%)	30.2 (24.2 to 36.8)
All causes	Belgium	2010	Age-standardized		4.9% (4.0 to 6.0%)	26.3 (21.2 to 32.4)
All causes	Belgium	2015	Age-standardized		4.8% (3.8 to 5.9%)	24.8 (19.7 to 30.7)
Lower respiratory infections	Belgium	1990	All Ages	0.6 (0.3 to 1.0)	16.6% (8.1 to 27.0%)	5.9 (2.8 to 9.6)
Lower respiratory infections	Belgium	1995	All Ages	0.6 (0.3 to 1.1)	15.4% (6.9 to 26.1%)	6.2 (2.8 to 10.6)
Lower respiratory infections	Belgium	2000	All Ages	0.8 (0.3 to 1.4)	14.5% (6.1 to 25.0%)	7.6 (3.2 to 13.4)
Lower respiratory infections	Belgium	2005	All Ages	0.8 (0.3 to 1.4)	14.1% (5.7 to 24.7%)	7.7 (3.0 to 13.5)
Lower respiratory infections	Belgium	2010	All Ages	0.8 (0.3 to 1.4)	14.1% (5.7 to 24.7%)	7.5 (3.1 to 13.2)
Lower respiratory infections	Belgium	2015	All Ages	0.9 (0.3 to 1.6)	13.6% (5.3 to 24.4%)	7.6 (3.0 to 14.1)
Lower respiratory infections	Belgium	1990	Age-standardized		16.6% (8.1 to 27.0%)	3.9 (1.9 to 6.4)
Lower respiratory infections	Belgium	1995	Age-standardized		15.4% (6.9 to 26.1%)	3.9 (1.7 to 6.7)
Lower respiratory infections	Belgium	2000	Age-standardized		14.5% (6.1 to 25.0%)	4.7 (2.0 to 8.3)
Lower respiratory infections	Belgium	2005	Age-standardized		14.1% (5.7 to 24.7%)	4.2 (1.7 to 7.3)
Lower respiratory infections	Belgium	2010	Age-standardized		14.1% (5.7 to 24.7%)	3.7 (1.5 to 6.5)
Lower respiratory infections	Belgium	2015	Age-standardized		13.6% (5.3 to 24.4%)	3.5 (1.4 to 6.4)
Tracheal, bronchus, and lung cancer	Belgium	1990	All Ages	0.8 (0.5 to 1.3)	11.6% (6.3 to 17.9%)	8.3 (4.5 to 12.7)
Tracheal, bronchus, and lung cancer	Belgium	1995	All Ages	0.8 (0.4 to 1.2)	10.8% (5.6 to 16.7%)	7.6 (4.0 to 11.8)
Tracheal, bronchus, and lung cancer	Belgium	2000	All Ages	0.7 (0.4 to 1.1)	10.1% (5.3 to 16.0%)	6.8 (3.5 to 10.7)
Tracheal, bronchus, and lung cancer	Belgium	2005	All Ages	0.7 (0.3 to 1.1)	9.8% (5.0 to 15.6%)	6.3 (3.2 to 10.1)
Tracheal, bronchus, and lung cancer	Belgium	2010	All Ages	0.7 (0.4 to 1.1)	9.8% (5.0 to 15.5%)	6.4 (3.3 to 10.2)
Tracheal, bronchus, and lung cancer	Belgium	2015	All Ages	0.7 (0.4 to 1.1)	9.4% (4.8 to 15.1%)	6.3 (3.2 to 9.9)
Tracheal, bronchus, and lung cancer	Belgium	1990	Age-standardized		11.6% (6.3 to 17.9%)	6.0 (3.3 to 9.2)
Tracheal, bronchus, and lung cancer	Belgium	1995	Age-standardized		10.8% (5.6 to 16.7%)	5.3 (2.8 to 8.2)
Tracheal, bronchus, and lung cancer	Belgium	2000	Age-standardized		10.1% (5.3 to 16.0%)	4.5 (2.3 to 7.1)
Tracheal, bronchus, and lung cancer	Belgium	2005	Age-standardized		9.8% (5.0 to 15.6%)	4.1 (2.1 to 6.5)
Tracheal, bronchus, and lung cancer	Belgium	2010	Age-standardized		9.8% (5.0 to 15.5%)	4.0 (2.0 to 6.4)

Cause name	Location	Year	Age group	Number (in thousands)	PAF	Rate (per 100,000)
Tracheal, bronchus, and lung cancer	Belgium	2015	Age-standardized		9.4% (4.8 to 15.1%)	3.7 (1.9 to 5.9)
Cardiovascular diseases	Belgium	1990	All Ages	4.5 (3.5 to 5.4)	10.8% (8.6 to 13.1%)	44.7 (35.4 to 54.2)
Cardiovascular diseases	Belgium	1995	All Ages	3.8 (3.0 to 4.7)	9.9% (7.8 to 12.2%)	38.0 (29.5 to 46.6)
Cardiovascular diseases	Belgium	2000	All Ages	3.5 (2.8 to 4.3)	9.4% (7.3 to 11.5%)	34.4 (26.8 to 42.3)
Cardiovascular diseases	Belgium	2005	All Ages	3.0 (2.3 to 3.8)	8.9% (6.8 to 11.1%)	29.0 (22.1 to 36.3)
Cardiovascular diseases	Belgium	2010	All Ages	2.8 (2.1 to 3.6)	8.6% (6.5 to 10.9%)	25.9 (19.5 to 32.9)
Cardiovascular diseases	Belgium	2015	All Ages	3.0 (2.2 to 3.9)	8.4% (6.1 to 10.7%)	26.3 (19.3 to 34.0)
Cardiovascular diseases	Belgium	1990	Age-standardized		11.1% (8.9 to 13.3%)	30.8 (24.7 to 37.0)
Cardiovascular diseases	Belgium	1995	Age-standardized		10.2% (8.1 to 12.3%)	25.0 (19.9 to 30.2)
Cardiovascular diseases	Belgium	2000	Age-standardized		9.6% (7.5 to 11.6%)	21.9 (17.2 to 26.8)
Cardiovascular diseases	Belgium	2005	Age-standardized		9.3% (7.3 to 11.3%)	16.8 (13.1 to 20.6)
Cardiovascular diseases	Belgium	2010	Age-standardized		9.1% (7.1 to 11.1%)	13.9 (10.9 to 17.2)
Cardiovascular diseases	Belgium	2015	Age-standardized		8.9% (6.9 to 11.0%)	13.2 (10.1 to 16.5)
Ischemic heart disease	Belgium	1990	All Ages	3.3 (2.5 to 4.1)	13.8% (10.4 to 17.3%)	32.8 (24.8 to 41.1)
Ischemic heart disease	Belgium	1995	All Ages	2.8 (2.1 to 3.6)	13.2% (9.8 to 16.8%)	27.9 (20.5 to 35.2)
Ischemic heart disease	Belgium	2000	All Ages	2.6 (1.9 to 3.3)	12.8% (9.3 to 16.2%)	25.3 (18.5 to 32.1)
Ischemic heart disease	Belgium	2005	All Ages	2.2 (1.6 to 2.9)	12.4% (8.8 to 16.0%)	21.1 (15.0 to 27.3)
Ischemic heart disease	Belgium	2010	All Ages	2.0 (1.4 to 2.7)	12.2% (8.3 to 16.1%)	18.7 (12.8 to 24.5)
Ischemic heart disease	Belgium	2015	All Ages	2.2 (1.4 to 2.9)	11.9% (7.9 to 15.8%)	19.2 (12.7 to 25.8)
Ischemic heart disease	Belgium	1990	Age-standardized		14.1% (10.9 to 17.4%)	22.8 (17.5 to 28.2)
Ischemic heart disease	Belgium	1995	Age-standardized		13.6% (10.3 to 16.8%)	18.5 (13.9 to 23.0)
Ischemic heart disease	Belgium	2000	Age-standardized		13.1% (9.7 to 16.4%)	16.2 (12.0 to 20.4)
Ischemic heart disease	Belgium	2005	Age-standardized		12.9% (9.5 to 16.2%)	12.3 (9.1 to 15.6)
Ischemic heart disease	Belgium	2010	Age-standardized		12.9% (9.4 to 16.2%)	10.2 (7.4 to 12.9)
Ischemic heart disease	Belgium	2015	Age-standardized		12.6% (9.1 to 16.0%)	9.7 (7.0 to 12.5)
Ischemic stroke	Belgium	1990	All Ages	0.7 (0.4 to 1.0)	9.5% (5.9 to 13.9%)	6.7 (4.1 to 9.9)
Ischemic stroke	Belgium	1995	All Ages	0.5 (0.3 to 0.8)	9.1% (5.3 to 13.6%)	5.1 (3.0 to 7.8)
Ischemic stroke	Belgium	2000	All Ages	0.5 (0.3 to 0.7)	8.8% (5.1 to 13.3%)	4.5 (2.6 to 6.9)
Ischemic stroke	Belgium	2005	All Ages	0.4 (0.2 to 0.6)	8.6% (4.7 to 13.3%)	3.9 (2.1 to 6.0)
Ischemic stroke	Belgium	2010	All Ages	0.4 (0.2 to 0.6)	8.5% (4.5 to 13.5%)	3.5 (1.9 to 5.6)
Ischemic stroke	Belgium	2015	All Ages	0.4 (0.2 to 0.7)	8.3% (4.2 to 13.5%)	3.5 (1.8 to 5.7)
Ischemic stroke	Belgium	1990	Age-standardized		9.6% (6.0 to 13.9%)	4.4 (2.8 to 6.4)
Ischemic stroke	Belgium	1995	Age-standardized		9.2% (5.6 to 13.6%)	3.2 (1.9 to 4.8)
Ischemic stroke	Belgium	2000	Age-standardized		8.9% (5.1 to 13.3%)	2.8 (1.6 to 4.2)
Ischemic stroke	Belgium	2005	Age-standardized		8.7% (5.0 to 13.2%)	2.1 (1.2 to 3.2)
Ischemic stroke	Belgium	2010	Age-standardized		8.7% (5.0 to 13.3%)	1.7 (1.0 to 2.7)

Cause name	Location	Year	Age group	Number (in thousands)	PAF	Rate (per 100,000)
Ischemic stroke	Belgium	2015	Age-standardized		8.5% (4.7 to 13.2%)	1.6 (0.9 to 2.5)
Hemorrhagic stroke	Belgium	1990	All Ages	0.5 (0.4 to 0.7)	10.5% (7.4 to 13.9%)	5.2 (3.6 to 6.8)
Hemorrhagic stroke	Belgium	1995	All Ages	0.5 (0.3 to 0.7)	10.0% (6.7 to 13.5%)	5.0 (3.3 to 6.8)
Hemorrhagic stroke	Belgium	2000	All Ages	0.5 (0.3 to 0.7)	9.6% (6.4 to 13.0%)	4.6 (3.1 to 6.4)
Hemorrhagic stroke	Belgium	2005	All Ages	0.4 (0.3 to 0.6)	9.3% (6.0 to 13.0%)	4.0 (2.6 to 5.8)
Hemorrhagic stroke	Belgium	2010	All Ages	0.4 (0.3 to 0.6)	9.1% (5.8 to 13.3%)	3.7 (2.4 to 5.4)
Hemorrhagic stroke	Belgium	2015	All Ages	0.4 (0.3 to 0.6)	8.8% (5.3 to 13.1%)	3.6 (2.2 to 5.5)
Hemorrhagic stroke	Belgium	1990	Age-standardized		10.8% (7.7 to 13.9%)	3.6 (2.6 to 4.8)
Hemorrhagic stroke	Belgium	1995	Age-standardized		10.2% (7.1 to 13.4%)	3.3 (2.3 to 4.4)
Hemorrhagic stroke	Belgium	2000	Age-standardized		9.8% (6.7 to 13.2%)	3.0 (2.0 to 4.0)
Hemorrhagic stroke	Belgium	2005	Age-standardized		9.6% (6.6 to 13.1%)	2.4 (1.6 to 3.3)
Hemorrhagic stroke	Belgium	2010	Age-standardized		9.6% (6.5 to 13.1%)	2.0 (1.4 to 2.8)
Hemorrhagic stroke	Belgium	2015	Age-standardized		9.3% (6.2 to 12.9%)	1.9 (1.2 to 2.6)
Chronic obstructive pulmonary disease	Belgium	1990	All Ages	1.0 (0.4 to 1.5)	18.0% (8.4 to 28.9%)	9.6 (4.4 to 15.2)
Chronic obstructive pulmonary disease	Belgium	1995	All Ages	1.1 (0.5 to 1.7)	17.1% (7.8 to 27.8%)	10.4 (4.7 to 16.8)
Chronic obstructive pulmonary disease	Belgium	2000	All Ages	1.0 (0.5 to 1.7)	16.3% (7.2 to 26.9%)	9.9 (4.4 to 16.3)
Chronic obstructive pulmonary disease	Belgium	2005	All Ages	1.0 (0.4 to 1.6)	15.9% (6.9 to 26.6%)	9.2 (4.0 to 15.1)
Chronic obstructive pulmonary disease	Belgium	2010	All Ages	1.0 (0.4 to 1.6)	15.9% (7.0 to 26.6%)	8.9 (3.9 to 14.8)
Chronic obstructive pulmonary disease	Belgium	2015	All Ages	1.0 (0.4 to 1.7)	15.5% (6.8 to 26.0%)	9.0 (3.8 to 14.9)
Chronic obstructive pulmonary disease	Belgium	1990	Age-standardized		18.0% (8.4 to 28.9%)	6.4 (3.0 to 10.2)
Chronic obstructive pulmonary disease	Belgium	1995	Age-standardized		17.0% (7.8 to 27.8%)	6.6 (3.0 to 10.8)
Chronic obstructive pulmonary disease	Belgium	2000	Age-standardized		16.3% (7.2 to 26.9%)	6.1 (2.7 to 10.0)
Chronic obstructive pulmonary disease	Belgium	2005	Age-standardized		15.9% (6.9 to 26.5%)	5.1 (2.2 to 8.4)
Chronic obstructive pulmonary disease	Belgium	2010	Age-standardized		15.9% (7.0 to 26.5%)	4.6 (2.0 to 7.7)
Chronic obstructive pulmonary disease	Belgium	2015	Age-standardized		15.5% (6.8 to 26.0%)	4.4 (1.8 to 7.2)
All causes	Cyprus	1990	All Ages	0.3 (0.3 to 0.4)	6.2% (4.9 to 7.5%)	48.1 (38.3 to 58.7)
All causes	Cyprus	1995	All Ages	0.3 (0.3 to 0.4)	6.1% (4.9 to 7.3%)	48.8 (39.1 to 58.9)
All causes	Cyprus	2000	All Ages	0.3 (0.3 to 0.4)	6.0% (4.9 to 7.2%)	44.5 (36.3 to 53.5)
All causes	Cyprus	2005	All Ages	0.3 (0.2 to 0.3)	5.0% (4.1 to 6.0%)	34.4 (28.4 to 41.3)
All causes	Cyprus	2010	All Ages	0.3 (0.2 to 0.3)	4.9% (3.9 to 5.9%)	31.6 (25.5 to 38.2)
All causes	Cyprus	2015	All Ages	0.3 (0.2 to 0.3)	5.2% (4.3 to 6.3%)	30.6 (25.3 to 37.0)
All causes	Cyprus	1990	Age-standardized		6.2% (5.0 to 7.4%)	47.2 (37.9 to 57.3)
All causes	Cyprus	1995	Age-standardized		6.1% (4.9 to 7.2%)	42.7 (34.6 to 51.0)
All causes	Cyprus	2000	Age-standardized		5.9% (4.9 to 7.0%)	36.5 (30.1 to 43.4)
All causes	Cyprus	2005	Age-standardized		5.0% (4.1 to 5.9%)	26.9 (22.3 to 32.1)
All causes	Cyprus	2010	Age-standardized		4.8% (3.9 to 5.8%)	23.3 (19.0 to 27.8)

Cause name	Location	Year	Age group	Number (in thousands)	PAF	Rate (per 100,000)
All causes	Cyprus	2015	Age-standardized		5.1% (4.3 to 6.1%)	21.4 (18.0 to 25.6)
Lower respiratory infections	Cyprus	1990	All Ages	0.0 (0.0 to 0.1)	13.6% (5.3 to 24.4%)	3.9 (1.5 to 7.2)
Lower respiratory infections	Cyprus	1995	All Ages	0.0 (0.0 to 0.1)	13.1% (4.8 to 23.9%)	3.7 (1.4 to 6.9)
Lower respiratory infections	Cyprus	2000	All Ages	0.0 (0.0 to 0.0)	13.2% (4.9 to 23.9%)	3.3 (1.2 to 6.0)
Lower respiratory infections	Cyprus	2005	All Ages	0.0 (0.0 to 0.0)	12.6% (4.5 to 23.4%)	2.6 (0.9 to 4.8)
Lower respiratory infections	Cyprus	2010	All Ages	0.0 (0.0 to 0.0)	13.4% (5.1 to 24.3%)	2.4 (0.8 to 4.6)
Lower respiratory infections	Cyprus	2015	All Ages	0.0 (0.0 to 0.0)	15.5% (6.9 to 25.9%)	2.6 (1.1 to 4.5)
Lower respiratory infections	Cyprus	1990	Age-standardized		13.6% (5.3 to 24.4%)	3.7 (1.4 to 7.0)
Lower respiratory infections	Cyprus	1995	Age-standardized		13.1% (4.8 to 23.9%)	3.1 (1.2 to 5.7)
Lower respiratory infections	Cyprus	2000	Age-standardized		13.2% (4.9 to 23.9%)	2.5 (0.9 to 4.6)
Lower respiratory infections	Cyprus	2005	Age-standardized		12.6% (4.5 to 23.4%)	1.9 (0.7 to 3.6)
Lower respiratory infections	Cyprus	2010	Age-standardized		13.4% (5.1 to 24.3%)	1.7 (0.6 to 3.2)
Lower respiratory infections	Cyprus	2015	Age-standardized		15.5% (6.9 to 25.9%)	1.7 (0.8 to 3.0)
Tracheal, bronchus, and lung cancer	Cyprus	1990	All Ages	0.0 (0.0 to 0.0)	9.4% (4.7 to 15.0%)	1.8 (0.9 to 2.8)
Tracheal, bronchus, and lung cancer	Cyprus	1995	All Ages	0.0 (0.0 to 0.0)	9.0% (4.6 to 14.5%)	1.9 (1.0 to 3.1)
Tracheal, bronchus, and lung cancer	Cyprus	2000	All Ages	0.0 (0.0 to 0.0)	9.1% (4.7 to 14.7%)	2.0 (1.0 to 3.2)
Tracheal, bronchus, and lung cancer	Cyprus	2005	All Ages	0.0 (0.0 to 0.0)	8.7% (4.3 to 14.4%)	2.4 (1.2 to 4.0)
Tracheal, bronchus, and lung cancer	Cyprus	2010	All Ages	0.0 (0.0 to 0.0)	9.3% (4.6 to 15.0%)	2.8 (1.4 to 4.6)
Tracheal, bronchus, and lung cancer	Cyprus	2015	All Ages	0.0 (0.0 to 0.0)	10.8% (5.7 to 17.1%)	3.0 (1.6 to 4.9)
Tracheal, bronchus, and lung cancer	Cyprus	1990	Age-standardized		9.4% (4.7 to 15.0%)	1.8 (0.9 to 2.8)
Tracheal, bronchus, and lung cancer	Cyprus	1995	Age-standardized		9.0% (4.6 to 14.5%)	1.8 (0.9 to 2.9)
Tracheal, bronchus, and lung cancer	Cyprus	2000	Age-standardized		9.1% (4.7 to 14.7%)	1.8 (0.9 to 2.9)
Tracheal, bronchus, and lung cancer	Cyprus	2005	Age-standardized		8.7% (4.3 to 14.4%)	2.0 (1.0 to 3.3)
Tracheal, bronchus, and lung cancer	Cyprus	2010	Age-standardized		9.3% (4.6 to 15.0%)	2.2 (1.1 to 3.5)
Tracheal, bronchus, and lung cancer	Cyprus	2015	Age-standardized		10.8% (5.7 to 17.1%)	2.2 (1.2 to 3.6)
Cardiovascular diseases	Cyprus	1990	All Ages	0.3 (0.2 to 0.3)	9.6% (7.3 to 11.9%)	36.9 (28.3 to 46.3)
Cardiovascular diseases	Cyprus	1995	All Ages	0.3 (0.2 to 0.3)	9.5% (7.4 to 11.7%)	37.7 (29.1 to 46.4)
Cardiovascular diseases	Cyprus	2000	All Ages	0.2 (0.2 to 0.3)	9.7% (7.6 to 11.9%)	34.2 (26.9 to 42.2)
Cardiovascular diseases	Cyprus	2005	All Ages	0.2 (0.1 to 0.2)	9.4% (7.4 to 11.5%)	25.4 (19.9 to 31.1)
Cardiovascular diseases	Cyprus	2010	All Ages	0.2 (0.1 to 0.2)	9.4% (7.4 to 11.6%)	22.3 (17.4 to 27.8)
Cardiovascular diseases	Cyprus	2015	All Ages	0.2 (0.1 to 0.2)	10.0% (7.9 to 12.2%)	21.0 (16.6 to 25.9)
Cardiovascular diseases	Cyprus	1990	Age-standardized		9.7% (7.5 to 11.9%)	36.4 (28.3 to 45.2)
Cardiovascular diseases	Cyprus	1995	Age-standardized		9.8% (7.8 to 11.8%)	33.2 (26.2 to 40.2)
Cardiovascular diseases	Cyprus	2000	Age-standardized		10.0% (8.0 to 12.0%)	28.3 (22.7 to 34.1)
Cardiovascular diseases	Cyprus	2005	Age-standardized		9.7% (7.7 to 11.7%)	20.0 (15.9 to 24.2)
Cardiovascular diseases	Cyprus	2010	Age-standardized		9.7% (7.7 to 11.7%)	16.5 (13.1 to 20.3)

Cause name	Location	Year	Age group	Number (in thousands)	PAF	Rate (per 100,000)
Cardiovascular diseases	Cyprus	2015	Age-standardized		10.3% (8.4 to 12.4%)	14.8 (11.9 to 18.0)
Ischemic heart disease	Cyprus	1990	All Ages	0.2 (0.1 to 0.2)	12.6% (9.1 to 16.2%)	27.9 (20.0 to 35.7)
Ischemic heart disease	Cyprus	1995	All Ages	0.2 (0.1 to 0.3)	12.6% (9.3 to 15.8%)	28.8 (21.2 to 36.5)
Ischemic heart disease	Cyprus	2000	All Ages	0.2 (0.1 to 0.2)	12.8% (9.5 to 16.1%)	26.4 (19.7 to 33.3)
Ischemic heart disease	Cyprus	2005	All Ages	0.1 (0.1 to 0.2)	12.7% (9.5 to 15.8%)	19.7 (14.8 to 24.9)
Ischemic heart disease	Cyprus	2010	All Ages	0.1 (0.1 to 0.2)	12.8% (9.5 to 16.0%)	17.4 (13.0 to 22.3)
Ischemic heart disease	Cyprus	2015	All Ages	0.1 (0.1 to 0.2)	13.6% (10.1 to 17.1%)	16.4 (12.3 to 20.7)
Ischemic heart disease	Cyprus	1990	Age-standardized		12.7% (9.4 to 16.1%)	27.5 (20.1 to 34.9)
Ischemic heart disease	Cyprus	1995	Age-standardized		12.9% (9.8 to 15.9%)	25.5 (19.3 to 31.7)
Ischemic heart disease	Cyprus	2000	Age-standardized		13.1% (10.1 to 16.1%)	22.0 (16.9 to 27.2)
Ischemic heart disease	Cyprus	2005	Age-standardized		13.0% (9.9 to 15.9%)	15.6 (12.0 to 19.2)
Ischemic heart disease	Cyprus	2010	Age-standardized		13.1% (10.0 to 16.2%)	13.0 (9.9 to 16.2)
Ischemic heart disease	Cyprus	2015	Age-standardized		14.0% (10.7 to 17.2%)	11.6 (9.0 to 14.4)
Ischemic stroke	Cyprus	1990	All Ages	0.0 (0.0 to 0.1)	8.5% (4.7 to 13.1%)	4.4 (2.4 to 7.1)
Ischemic stroke	Cyprus	1995	All Ages	0.0 (0.0 to 0.0)	8.4% (4.8 to 12.8%)	4.2 (2.4 to 6.6)
Ischemic stroke	Cyprus	2000	All Ages	0.0 (0.0 to 0.0)	8.4% (4.9 to 12.7%)	3.7 (2.1 to 5.7)
Ischemic stroke	Cyprus	2005	All Ages	0.0 (0.0 to 0.0)	8.2% (4.7 to 12.6%)	2.7 (1.5 to 4.2)
Ischemic stroke	Cyprus	2010	All Ages	0.0 (0.0 to 0.0)	8.4% (4.6 to 13.0%)	2.4 (1.3 to 3.7)
Ischemic stroke	Cyprus	2015	All Ages	0.0 (0.0 to 0.0)	9.1% (5.3 to 13.7%)	2.3 (1.3 to 3.5)
Ischemic stroke	Cyprus	1990	Age-standardized		8.5% (4.9 to 13.0%)	4.3 (2.4 to 6.7)
Ischemic stroke	Cyprus	1995	Age-standardized		8.5% (5.1 to 12.6%)	3.6 (2.1 to 5.4)
Ischemic stroke	Cyprus	2000	Age-standardized		8.6% (5.3 to 12.5%)	2.9 (1.7 to 4.3)
Ischemic stroke	Cyprus	2005	Age-standardized		8.4% (4.9 to 12.4%)	2.0 (1.2 to 3.1)
Ischemic stroke	Cyprus	2010	Age-standardized		8.5% (4.8 to 12.9%)	1.7 (1.0 to 2.6)
Ischemic stroke	Cyprus	2015	Age-standardized		9.2% (5.6 to 13.5%)	1.5 (0.9 to 2.3)
Hemorrhagic stroke	Cyprus	1990	All Ages	0.0 (0.0 to 0.0)	9.3% (6.2 to 12.8%)	4.7 (3.1 to 6.6)
Hemorrhagic stroke	Cyprus	1995	All Ages	0.0 (0.0 to 0.0)	9.2% (6.2 to 12.6%)	4.7 (3.2 to 6.5)
Hemorrhagic stroke	Cyprus	2000	All Ages	0.0 (0.0 to 0.0)	9.4% (6.4 to 12.7%)	4.1 (2.8 to 5.7)
Hemorrhagic stroke	Cyprus	2005	All Ages	0.0 (0.0 to 0.0)	9.1% (6.2 to 12.5%)	3.0 (2.0 to 4.2)
Hemorrhagic stroke	Cyprus	2010	All Ages	0.0 (0.0 to 0.0)	9.2% (6.0 to 12.8%)	2.5 (1.7 to 3.6)
Hemorrhagic stroke	Cyprus	2015	All Ages	0.0 (0.0 to 0.0)	10.0% (6.6 to 13.5%)	2.4 (1.6 to 3.3)
Hemorrhagic stroke	Cyprus	1990	Age-standardized		9.4% (6.3 to 12.7%)	4.6 (3.1 to 6.5)
Hemorrhagic stroke	Cyprus	1995	Age-standardized		9.4% (6.5 to 12.5%)	4.1 (2.8 to 5.6)
Hemorrhagic stroke	Cyprus	2000	Age-standardized		9.6% (6.7 to 12.7%)	3.4 (2.4 to 4.7)
Hemorrhagic stroke	Cyprus	2005	Age-standardized		9.3% (6.5 to 12.5%)	2.4 (1.6 to 3.2)
Hemorrhagic stroke	Cyprus	2010	Age-standardized		9.4% (6.3 to 12.8%)	1.9 (1.3 to 2.6)

Cause name	Location	Year	Age group	Number (in thousands)	PAF	Rate (per 100,000)
Hemorrhagic stroke	Cyprus	2015	Age-standardized		10.2% (7.1 to 13.4%)	1.7 (1.2 to 2.3)
Chronic obstructive pulmonary disease	Cyprus	1990	All Ages	0.0 (0.0 to 0.1)	15.4% (6.8 to 25.9%)	5.5 (2.3 to 9.4)
Chronic obstructive pulmonary disease	Cyprus	1995	All Ages	0.0 (0.0 to 0.1)	15.0% (6.4 to 25.3%)	5.5 (2.3 to 9.5)
Chronic obstructive pulmonary disease	Cyprus	2000	All Ages	0.0 (0.0 to 0.1)	15.1% (6.6 to 25.4%)	5.0 (2.1 to 8.4)
Chronic obstructive pulmonary disease	Cyprus	2005	All Ages	0.0 (0.0 to 0.1)	14.7% (6.2 to 25.1%)	4.0 (1.7 to 6.9)
Chronic obstructive pulmonary disease	Cyprus	2010	All Ages	0.0 (0.0 to 0.1)	15.3% (6.5 to 25.8%)	4.1 (1.7 to 7.0)
Chronic obstructive pulmonary disease	Cyprus	2015	All Ages	0.0 (0.0 to 0.1)	17.1% (7.8 to 27.7%)	4.0 (1.8 to 6.4)
Chronic obstructive pulmonary disease	Cyprus	1990	Age-standardized		15.4% (6.8 to 25.9%)	5.4 (2.3 to 9.1)
Chronic obstructive pulmonary disease	Cyprus	1995	Age-standardized		15.0% (6.4 to 25.3%)	4.6 (1.9 to 8.0)
Chronic obstructive pulmonary disease	Cyprus	2000	Age-standardized		15.1% (6.6 to 25.4%)	3.9 (1.6 to 6.6)
Chronic obstructive pulmonary disease	Cyprus	2005	Age-standardized		14.6% (6.2 to 25.1%)	3.0 (1.3 to 5.2)
Chronic obstructive pulmonary disease	Cyprus	2010	Age-standardized		15.3% (6.5 to 25.8%)	2.9 (1.2 to 4.9)
Chronic obstructive pulmonary disease	Cyprus	2015	Age-standardized		17.1% (7.8 to 27.6%)	2.6 (1.2 to 4.2)
All causes	Denmark	1990	All Ages	3.4 (2.7 to 4.1)	5.7% (4.5 to 6.9%)	65.9 (52.9 to 80.2)
All causes	Denmark	1995	All Ages	3.1 (2.5 to 3.9)	5.1% (4.0 to 6.4%)	60.0 (47.0 to 74.8)
All causes	Denmark	2000	All Ages	2.6 (2.0 to 3.3)	4.5% (3.5 to 5.8%)	48.3 (37.3 to 61.9)
All causes	Denmark	2005	All Ages	2.2 (1.7 to 2.9)	4.0% (3.0 to 5.3%)	40.7 (30.7 to 53.1)
All causes	Denmark	2010	All Ages	2.1 (1.6 to 2.7)	3.9% (2.9 to 5.1%)	37.9 (28.6 to 49.2)
All causes	Denmark	2015	All Ages	2.1 (1.6 to 2.8)	4.0% (3.0 to 5.2%)	37.3 (27.9 to 48.5)
All causes	Denmark	1990	Age-standardized		5.5% (4.5 to 6.6%)	44.1 (36.0 to 53.1)
All causes	Denmark	1995	Age-standardized		5.0% (4.0 to 6.1%)	39.2 (31.5 to 48.3)
All causes	Denmark	2000	Age-standardized		4.4% (3.5 to 5.6%)	31.1 (24.4 to 39.4)
All causes	Denmark	2005	Age-standardized		3.9% (3.0 to 5.0%)	25.3 (19.4 to 32.5)
All causes	Denmark	2010	Age-standardized		3.8% (2.9 to 4.9%)	22.6 (17.2 to 29.1)
All causes	Denmark	2015	Age-standardized		3.9% (2.9 to 5.0%)	21.4 (16.1 to 27.7)
Lower respiratory infections	Denmark	1990	All Ages	0.2 (0.1 to 0.4)	11.2% (3.6 to 22.2%)	4.0 (1.3 to 8.0)
Lower respiratory infections	Denmark	1995	All Ages	0.2 (0.1 to 0.5)	10.2% (2.8 to 21.3%)	4.0 (1.1 to 8.6)
Lower respiratory infections	Denmark	2000	All Ages	0.2 (0.1 to 0.4)	9.5% (2.5 to 20.5%)	3.5 (0.9 to 7.7)
Lower respiratory infections	Denmark	2005	All Ages	0.2 (0.1 to 0.4)	8.9% (2.2 to 20.0%)	3.7 (0.9 to 8.1)
Lower respiratory infections	Denmark	2010	All Ages	0.2 (0.1 to 0.5)	9.0% (2.2 to 20.0%)	4.0 (1.0 to 8.8)
Lower respiratory infections	Denmark	2015	All Ages	0.2 (0.1 to 0.5)	9.6% (2.5 to 20.6%)	4.0 (1.0 to 8.7)
Lower respiratory infections	Denmark	1990	Age-standardized		11.2% (3.6 to 22.2%)	2.5 (0.8 to 5.0)
Lower respiratory infections	Denmark	1995	Age-standardized		10.2% (2.8 to 21.3%)	2.4 (0.7 to 5.2)
Lower respiratory infections	Denmark	2000	Age-standardized		9.5% (2.5 to 20.5%)	2.1 (0.5 to 4.6)
Lower respiratory infections	Denmark	2005	Age-standardized		8.9% (2.2 to 20.0%)	2.1 (0.5 to 4.7)
Lower respiratory infections	Denmark	2010	Age-standardized		9.0% (2.2 to 20.0%)	2.3 (0.5 to 5.0)

Cause name	Location	Year	Age group	Number (in thousands)	PAF	Rate (per 100,000)
Lower respiratory infections	Denmark	2015	Age-standardized		9.6% (2.5 to 20.6%)	2.2 (0.6 to 4.8)
Tracheal, bronchus, and lung cancer	Denmark	1990	All Ages	0.3 (0.1 to 0.5)	7.7% (3.7 to 13.0%)	5.2 (2.5 to 8.7)
Tracheal, bronchus, and lung cancer	Denmark	1995	All Ages	0.3 (0.1 to 0.4)	6.9% (3.3 to 11.9%)	5.0 (2.3 to 8.5)
Tracheal, bronchus, and lung cancer	Denmark	2000	All Ages	0.2 (0.1 to 0.4)	6.4% (2.9 to 11.1%)	4.4 (2.0 to 7.6)
Tracheal, bronchus, and lung cancer	Denmark	2005	All Ages	0.2 (0.1 to 0.4)	6.1% (2.7 to 10.5%)	4.1 (1.8 to 7.2)
Tracheal, bronchus, and lung cancer	Denmark	2010	All Ages	0.2 (0.1 to 0.4)	6.1% (2.7 to 10.7%)	4.0 (1.8 to 6.9)
Tracheal, bronchus, and lung cancer	Denmark	2015	All Ages	0.2 (0.1 to 0.4)	6.5% (2.9 to 11.2%)	4.3 (1.9 to 7.3)
Tracheal, bronchus, and lung cancer	Denmark	1990	Age-standardized		7.7% (3.7 to 13.0%)	3.7 (1.8 to 6.3)
Tracheal, bronchus, and lung cancer	Denmark	1995	Age-standardized		6.9% (3.3 to 11.9%)	3.6 (1.7 to 6.1)
Tracheal, bronchus, and lung cancer	Denmark	2000	Age-standardized		6.4% (2.9 to 11.1%)	3.1 (1.4 to 5.4)
Tracheal, bronchus, and lung cancer	Denmark	2005	Age-standardized		6.1% (2.7 to 10.5%)	2.8 (1.2 to 4.8)
Tracheal, bronchus, and lung cancer	Denmark	2010	Age-standardized		6.1% (2.7 to 10.7%)	2.5 (1.1 to 4.4)
Tracheal, bronchus, and lung cancer	Denmark	2015	Age-standardized		6.5% (2.9 to 11.2%)	2.5 (1.1 to 4.3)
Cardiovascular diseases	Denmark	1990	All Ages	2.5 (1.9 to 3.1)	9.3% (7.1 to 11.5%)	49.3 (37.7 to 61.3)
Cardiovascular diseases	Denmark	1995	All Ages	2.2 (1.7 to 2.8)	8.7% (6.5 to 11.0%)	42.7 (32.0 to 54.2)
Cardiovascular diseases	Denmark	2000	All Ages	1.7 (1.2 to 2.2)	8.1% (5.9 to 10.5%)	31.8 (23.3 to 41.1)
Cardiovascular diseases	Denmark	2005	All Ages	1.4 (1.0 to 1.8)	7.6% (5.4 to 9.9%)	25.2 (18.0 to 32.8)
Cardiovascular diseases	Denmark	2010	All Ages	1.2 (0.9 to 1.6)	7.3% (5.3 to 9.5%)	21.9 (15.8 to 28.7)
Cardiovascular diseases	Denmark	2015	All Ages	1.2 (0.9 to 1.6)	7.5% (5.4 to 9.7%)	20.9 (15.1 to 27.4)
Cardiovascular diseases	Denmark	1990	Age-standardized		9.6% (7.5 to 11.8%)	32.9 (25.7 to 40.4)
Cardiovascular diseases	Denmark	1995	Age-standardized		9.0% (6.9 to 11.1%)	27.8 (21.4 to 34.6)
Cardiovascular diseases	Denmark	2000	Age-standardized		8.3% (6.3 to 10.5%)	20.4 (15.5 to 25.6)
Cardiovascular diseases	Denmark	2005	Age-standardized		7.8% (5.7 to 9.8%)	15.6 (11.6 to 19.8)
Cardiovascular diseases	Denmark	2010	Age-standardized		7.5% (5.5 to 9.5%)	13.1 (9.7 to 16.7)
Cardiovascular diseases	Denmark	2015	Age-standardized		7.6% (5.6 to 9.7%)	12.0 (8.8 to 15.5)
Ischemic heart disease	Denmark	1990	All Ages	2.0 (1.5 to 2.6)	11.5% (8.3 to 14.7%)	39.1 (28.4 to 50.4)
Ischemic heart disease	Denmark	1995	All Ages	1.7 (1.2 to 2.3)	10.9% (7.5 to 14.2%)	33.4 (23.2 to 43.6)
Ischemic heart disease	Denmark	2000	All Ages	1.3 (0.9 to 1.7)	10.4% (6.9 to 13.9%)	24.0 (16.3 to 32.4)
Ischemic heart disease	Denmark	2005	All Ages	1.0 (0.6 to 1.4)	10.1% (6.6 to 13.6%)	18.3 (11.9 to 25.0)
Ischemic heart disease	Denmark	2010	All Ages	0.9 (0.6 to 1.2)	10.1% (6.6 to 13.7%)	15.7 (10.3 to 21.4)
Ischemic heart disease	Denmark	2015	All Ages	0.9 (0.6 to 1.2)	10.3% (6.7 to 14.0%)	15.1 (9.9 to 20.6)
Ischemic heart disease	Denmark	1990	Age-standardized		11.9% (8.9 to 14.9%)	26.3 (19.6 to 33.1)
Ischemic heart disease	Denmark	1995	Age-standardized		11.2% (8.2 to 14.3%)	21.8 (15.9 to 27.8)
Ischemic heart disease	Denmark	2000	Age-standardized		10.7% (7.5 to 14.0%)	15.4 (10.8 to 20.2)
Ischemic heart disease	Denmark	2005	Age-standardized		10.3% (7.1 to 13.7%)	11.3 (7.7 to 15.1)
Ischemic heart disease	Denmark	2010	Age-standardized		10.3% (7.0 to 13.6%)	9.4 (6.3 to 12.5)

Cause name	Location	Year	Age group	Number (in thousands)	PAF	Rate (per 100,000)
Ischemic heart disease	Denmark	2015	Age-standardized		10.5% (7.0 to 13.9%)	8.7 (5.8 to 11.7)
Ischemic stroke	Denmark	1990	All Ages	0.3 (0.1 to 0.4)	7.8% (4.3 to 12.1%)	5.1 (2.8 to 7.9)
Ischemic stroke	Denmark	1995	All Ages	0.2 (0.1 to 0.4)	7.3% (3.8 to 11.7%)	4.6 (2.4 to 7.3)
Ischemic stroke	Denmark	2000	All Ages	0.2 (0.1 to 0.3)	7.0% (3.5 to 11.4%)	3.9 (2.0 to 6.3)
Ischemic stroke	Denmark	2005	All Ages	0.2 (0.1 to 0.3)	6.8% (3.3 to 11.4%)	3.4 (1.7 to 5.7)
Ischemic stroke	Denmark	2010	All Ages	0.2 (0.1 to 0.3)	6.8% (3.3 to 11.4%)	3.1 (1.5 to 5.2)
Ischemic stroke	Denmark	2015	All Ages	0.2 (0.1 to 0.3)	7.0% (3.4 to 11.7%)	2.9 (1.4 to 4.7)
Ischemic stroke	Denmark	1990	Age-standardized		7.9% (4.6 to 12.0%)	3.2 (1.8 to 4.9)
Ischemic stroke	Denmark	1995	Age-standardized		7.5% (4.1 to 11.5%)	2.8 (1.5 to 4.4)
Ischemic stroke	Denmark	2000	Age-standardized		7.1% (3.8 to 11.3%)	2.4 (1.3 to 3.7)
Ischemic stroke	Denmark	2005	Age-standardized		6.9% (3.5 to 11.2%)	2.0 (1.1 to 3.3)
Ischemic stroke	Denmark	2010	Age-standardized		6.8% (3.5 to 11.2%)	1.8 (0.9 to 2.9)
Ischemic stroke	Denmark	2015	Age-standardized		7.0% (3.6 to 11.6%)	1.6 (0.8 to 2.6)
Hemorrhagic stroke	Denmark	1990	All Ages	0.3 (0.2 to 0.4)	8.5% (5.5 to 11.7%)	5.1 (3.3 to 7.1)
Hemorrhagic stroke	Denmark	1995	All Ages	0.3 (0.1 to 0.4)	8.0% (5.1 to 11.5%)	4.7 (3.0 to 6.9)
Hemorrhagic stroke	Denmark	2000	All Ages	0.2 (0.1 to 0.3)	7.7% (4.7 to 11.2%)	3.9 (2.4 to 5.8)
Hemorrhagic stroke	Denmark	2005	All Ages	0.2 (0.1 to 0.3)	7.4% (4.4 to 11.1%)	3.5 (2.1 to 5.2)
Hemorrhagic stroke	Denmark	2010	All Ages	0.2 (0.1 to 0.3)	7.3% (4.3 to 11.1%)	3.1 (1.9 to 4.7)
Hemorrhagic stroke	Denmark	2015	All Ages	0.2 (0.1 to 0.3)	7.5% (4.5 to 11.2%)	2.9 (1.7 to 4.4)
Hemorrhagic stroke	Denmark	1990	Age-standardized		8.8% (6.0 to 11.8%)	3.5 (2.4 to 4.7)
Hemorrhagic stroke	Denmark	1995	Age-standardized		8.3% (5.5 to 11.5%)	3.1 (2.0 to 4.4)
Hemorrhagic stroke	Denmark	2000	Age-standardized		8.0% (5.1 to 11.1%)	2.6 (1.7 to 3.7)
Hemorrhagic stroke	Denmark	2005	Age-standardized		7.6% (4.8 to 11.0%)	2.2 (1.4 to 3.2)
Hemorrhagic stroke	Denmark	2010	Age-standardized		7.5% (4.7 to 11.0%)	1.9 (1.2 to 2.8)
Hemorrhagic stroke	Denmark	2015	Age-standardized		7.7% (4.8 to 11.3%)	1.7 (1.1 to 2.5)
Chronic obstructive pulmonary disease	Denmark	1990	All Ages	0.4 (0.1 to 0.7)	13.4% (5.1 to 23.3%)	7.4 (2.9 to 13.0)
Chronic obstructive pulmonary disease	Denmark	1995	All Ages	0.4 (0.2 to 0.8)	12.4% (4.6 to 22.4%)	8.3 (3.1 to 14.9)
Chronic obstructive pulmonary disease	Denmark	2000	All Ages	0.5 (0.2 to 0.8)	11.8% (4.1 to 21.5%)	8.5 (2.9 to 15.4)
Chronic obstructive pulmonary disease	Denmark	2005	All Ages	0.4 (0.1 to 0.8)	11.2% (3.8 to 20.8%)	7.7 (2.6 to 14.4)
Chronic obstructive pulmonary disease	Denmark	2010	All Ages	0.5 (0.1 to 0.8)	11.3% (3.7 to 20.8%)	8.0 (2.6 to 14.8)
Chronic obstructive pulmonary disease	Denmark	2015	All Ages	0.5 (0.1 to 0.8)	11.8% (4.0 to 21.6%)	8.2 (2.7 to 14.8)
Chronic obstructive pulmonary disease	Denmark	1990	Age-standardized		13.4% (5.1 to 23.3%)	4.9 (1.9 to 8.6)
Chronic obstructive pulmonary disease	Denmark	1995	Age-standardized		12.4% (4.6 to 22.4%)	5.5 (2.0 to 9.7)
Chronic obstructive pulmonary disease	Denmark	2000	Age-standardized		11.8% (4.0 to 21.4%)	5.5 (1.9 to 10.0)
Chronic obstructive pulmonary disease	Denmark	2005	Age-standardized		11.2% (3.8 to 20.8%)	4.8 (1.6 to 9.0)
Chronic obstructive pulmonary disease	Denmark	2010	Age-standardized		11.3% (3.7 to 20.8%)	4.8 (1.6 to 8.8)

Cause name	Location	Year	Age group	Number (in thousands)	PAF	Rate (per 100,000)
Chronic obstructive pulmonary disease	Denmark	2015	Age-standardized		11.8% (4.0 to 21.5%)	4.6 (1.5 to 8.4)
All causes	Finland	1990	All Ages	2.1 (1.6 to 2.7)	4.3% (3.3 to 5.4%)	43.0 (32.5 to 53.6)
All causes	Finland	1995	All Ages	1.9 (1.4 to 2.4)	3.8% (2.8 to 4.9%)	36.6 (26.7 to 47.1)
All causes	Finland	2000	All Ages	1.6 (1.2 to 2.2)	3.4% (2.4 to 4.4%)	31.7 (22.6 to 41.9)
All causes	Finland	2005	All Ages	1.4 (1.0 to 1.9)	2.9% (2.0 to 3.9%)	27.1 (18.9 to 36.3)
All causes	Finland	2010	All Ages	1.4 (1.0 to 2.0)	2.8% (1.9 to 3.8%)	26.8 (18.5 to 36.5)
All causes	Finland	2015	All Ages	1.6 (1.1 to 2.1)	2.9% (2.0 to 3.9%)	28.0 (19.1 to 38.2)
All causes	Finland	1990	Age-standardized		4.2% (3.2 to 5.3%)	33.5 (25.6 to 41.7)
All causes	Finland	1995	Age-standardized		3.7% (2.8 to 4.8%)	26.6 (19.7 to 34.0)
All causes	Finland	2000	Age-standardized		3.3% (2.4 to 4.3%)	21.6 (15.5 to 28.1)
All causes	Finland	2005	Age-standardized		2.9% (2.0 to 3.7%)	16.6 (11.8 to 21.7)
All causes	Finland	2010	Age-standardized		2.7% (1.9 to 3.6%)	14.6 (10.4 to 19.4)
All causes	Finland	2015	Age-standardized		2.8% (2.0 to 3.7%)	13.9 (10.0 to 18.4)
Lower respiratory infections	Finland	1990	All Ages	0.1 (0.0 to 0.4)	6.2% (1.0 to 16.7%)	3.1 (0.5 to 8.2)
Lower respiratory infections	Finland	1995	All Ages	0.1 (0.0 to 0.4)	5.6% (0.7 to 15.8%)	2.9 (0.4 to 8.2)
Lower respiratory infections	Finland	2000	All Ages	0.1 (0.0 to 0.3)	5.3% (0.7 to 15.3%)	2.3 (0.3 to 6.7)
Lower respiratory infections	Finland	2005	All Ages	0.1 (0.0 to 0.2)	5.1% (0.6 to 15.1%)	1.2 (0.1 to 3.6)
Lower respiratory infections	Finland	2010	All Ages	0.1 (0.0 to 0.1)	5.2% (0.7 to 15.4%)	0.9 (0.1 to 2.5)
Lower respiratory infections	Finland	2015	All Ages	0.1 (0.0 to 0.1)	5.6% (0.8 to 15.9%)	0.9 (0.1 to 2.5)
Lower respiratory infections	Finland	1990	Age-standardized		6.2% (1.0 to 16.7%)	2.4 (0.4 to 6.4)
Lower respiratory infections	Finland	1995	Age-standardized		5.6% (0.7 to 15.8%)	2.0 (0.3 to 5.8)
Lower respiratory infections	Finland	2000	Age-standardized		5.3% (0.7 to 15.3%)	1.5 (0.2 to 4.4)
Lower respiratory infections	Finland	2005	Age-standardized		5.1% (0.6 to 15.1%)	0.7 (0.1 to 2.1)
Lower respiratory infections	Finland	2010	Age-standardized		5.2% (0.7 to 15.4%)	0.5 (0.1 to 1.3)
Lower respiratory infections	Finland	2015	Age-standardized		5.6% (0.8 to 15.9%)	0.4 (0.1 to 1.2)
Tracheal, bronchus, and lung cancer	Finland	1990	All Ages	0.1 (0.0 to 0.1)	4.1% (1.5 to 7.8%)	1.6 (0.6 to 3.1)
Tracheal, bronchus, and lung cancer	Finland	1995	All Ages	0.1 (0.0 to 0.1)	3.6% (1.2 to 7.3%)	1.4 (0.5 to 2.7)
Tracheal, bronchus, and lung cancer	Finland	2000	All Ages	0.1 (0.0 to 0.1)	3.4% (1.2 to 6.9%)	1.3 (0.4 to 2.6)
Tracheal, bronchus, and lung cancer	Finland	2005	All Ages	0.1 (0.0 to 0.1)	3.3% (1.1 to 6.8%)	1.3 (0.4 to 2.7)
Tracheal, bronchus, and lung cancer	Finland	2010	All Ages	0.1 (0.0 to 0.1)	3.4% (1.1 to 6.9%)	1.4 (0.5 to 2.9)
Tracheal, bronchus, and lung cancer	Finland	2015	All Ages	0.1 (0.0 to 0.2)	3.7% (1.3 to 7.2%)	1.5 (0.5 to 3.0)
Tracheal, bronchus, and lung cancer	Finland	1990	Age-standardized		4.1% (1.5 to 7.8%)	1.3 (0.5 to 2.4)
Tracheal, bronchus, and lung cancer	Finland	1995	Age-standardized		3.6% (1.2 to 7.3%)	1.0 (0.4 to 2.0)
Tracheal, bronchus, and lung cancer	Finland	2000	Age-standardized		3.4% (1.2 to 6.9%)	0.9 (0.3 to 1.8)
Tracheal, bronchus, and lung cancer	Finland	2005	Age-standardized		3.3% (1.1 to 6.8%)	0.8 (0.3 to 1.7)
Tracheal, bronchus, and lung cancer	Finland	2010	Age-standardized		3.4% (1.1 to 6.9%)	0.8 (0.3 to 1.7)

Cause name	Location	Year	Age group	Number (in thousands)	PAF	Rate (per 100,000)
Tracheal, bronchus, and lung cancer	Finland	2015	Age-standardized		3.7% (1.3 to 7.2%)	0.8 (0.3 to 1.6)
Cardiovascular diseases	Finland	1990	All Ages	1.8 (1.4 to 2.3)	7.5% (5.7 to 9.4%)	36.5 (27.5 to 45.8)
Cardiovascular diseases	Finland	1995	All Ages	1.6 (1.1 to 2.0)	6.9% (5.0 to 8.9%)	30.7 (22.1 to 39.8)
Cardiovascular diseases	Finland	2000	All Ages	1.4 (1.0 to 1.8)	6.3% (4.4 to 8.3%)	26.4 (18.4 to 35.0)
Cardiovascular diseases	Finland	2005	All Ages	1.2 (0.8 to 1.7)	5.9% (4.0 to 8.0%)	22.9 (15.5 to 31.5)
Cardiovascular diseases	Finland	2010	All Ages	1.2 (0.8 to 1.7)	5.7% (3.8 to 7.9%)	22.7 (14.9 to 31.8)
Cardiovascular diseases	Finland	2015	All Ages	1.3 (0.8 to 1.8)	5.9% (3.8 to 8.2%)	23.6 (15.4 to 32.9)
Cardiovascular diseases	Finland	1990	Age-standardized		7.6% (5.8 to 9.5%)	28.5 (21.6 to 35.8)
Cardiovascular diseases	Finland	1995	Age-standardized		6.9% (5.1 to 8.9%)	22.4 (16.4 to 28.7)
Cardiovascular diseases	Finland	2000	Age-standardized		6.4% (4.5 to 8.4%)	18.0 (12.7 to 23.6)
Cardiovascular diseases	Finland	2005	Age-standardized		6.0% (4.2 to 8.0%)	14.0 (9.8 to 18.8)
Cardiovascular diseases	Finland	2010	Age-standardized		5.9% (4.1 to 7.9%)	12.4 (8.5 to 16.7)
Cardiovascular diseases	Finland	2015	Age-standardized		6.0% (4.2 to 8.1%)	11.7 (8.0 to 15.6)
Ischemic heart disease	Finland	1990	All Ages	1.5 (1.0 to 1.9)	9.2% (6.6 to 11.9%)	29.1 (20.7 to 37.8)
Ischemic heart disease	Finland	1995	All Ages	1.2 (0.8 to 1.7)	8.5% (5.8 to 11.4%)	24.4 (16.7 to 32.7)
Ischemic heart disease	Finland	2000	All Ages	1.1 (0.7 to 1.5)	8.1% (5.3 to 11.1%)	21.1 (14.0 to 29.0)
Ischemic heart disease	Finland	2005	All Ages	1.0 (0.6 to 1.4)	7.8% (4.9 to 11.0%)	18.3 (11.5 to 26.1)
Ischemic heart disease	Finland	2010	All Ages	1.0 (0.6 to 1.4)	7.8% (4.7 to 11.3%)	18.2 (11.0 to 26.2)
Ischemic heart disease	Finland	2015	All Ages	1.1 (0.6 to 1.5)	8.0% (4.7 to 11.8%)	18.9 (11.2 to 27.7)
Ischemic heart disease	Finland	1990	Age-standardized		9.3% (6.7 to 12.0%)	22.8 (16.2 to 29.6)
Ischemic heart disease	Finland	1995	Age-standardized		8.6% (6.0 to 11.4%)	17.9 (12.4 to 23.7)
Ischemic heart disease	Finland	2000	Age-standardized		8.2% (5.5 to 11.2%)	14.4 (9.6 to 19.6)
Ischemic heart disease	Finland	2005	Age-standardized		8.0% (5.2 to 11.1%)	11.2 (7.3 to 15.7)
Ischemic heart disease	Finland	2010	Age-standardized		8.0% (5.1 to 11.1%)	9.9 (6.4 to 14.0)
Ischemic heart disease	Finland	2015	Age-standardized		8.3% (5.3 to 11.6%)	9.4 (6.1 to 13.1)
Ischemic stroke	Finland	1990	All Ages	0.2 (0.1 to 0.4)	5.7% (2.9 to 9.4%)	4.8 (2.4 to 8.1)
Ischemic stroke	Finland	1995	All Ages	0.2 (0.1 to 0.4)	5.3% (2.5 to 9.3%)	4.1 (1.9 to 7.3)
Ischemic stroke	Finland	2000	All Ages	0.2 (0.1 to 0.3)	5.1% (2.2 to 9.1%)	3.5 (1.5 to 6.4)
Ischemic stroke	Finland	2005	All Ages	0.2 (0.1 to 0.3)	4.9% (2.0 to 9.1%)	3.0 (1.3 to 5.6)
Ischemic stroke	Finland	2010	All Ages	0.2 (0.1 to 0.3)	4.9% (1.9 to 9.4%)	3.0 (1.2 to 5.7)
Ischemic stroke	Finland	2015	All Ages	0.2 (0.1 to 0.3)	5.1% (1.9 to 9.9%)	3.1 (1.2 to 6.1)
Ischemic stroke	Finland	1990	Age-standardized		5.7% (2.9 to 9.5%)	3.7 (1.8 to 6.2)
Ischemic stroke	Finland	1995	Age-standardized		5.3% (2.6 to 9.2%)	2.9 (1.4 to 5.1)
Ischemic stroke	Finland	2000	Age-standardized		5.1% (2.3 to 9.1%)	2.3 (1.0 to 4.2)
Ischemic stroke	Finland	2005	Age-standardized		4.9% (2.2 to 9.1%)	1.8 (0.8 to 3.2)
Ischemic stroke	Finland	2010	Age-standardized		5.0% (2.2 to 9.2%)	1.5 (0.7 to 2.8)

Cause name	Location	Year	Age group	Number (in thousands)	PAF	Rate (per 100,000)
Ischemic stroke	Finland	2015	Age-standardized		5.2% (2.2 to 9.5%)	1.4 (0.6 to 2.7)
Hemorrhagic stroke	Finland	1990	All Ages	0.1 (0.1 to 0.2)	6.8% (4.4 to 9.3%)	2.5 (1.7 to 3.5)
Hemorrhagic stroke	Finland	1995	All Ages	0.1 (0.1 to 0.2)	6.2% (3.9 to 8.9%)	2.2 (1.3 to 3.1)
Hemorrhagic stroke	Finland	2000	All Ages	0.1 (0.1 to 0.1)	6.0% (3.6 to 8.8%)	1.8 (1.1 to 2.7)
Hemorrhagic stroke	Finland	2005	All Ages	0.1 (0.1 to 0.1)	5.7% (3.4 to 8.6%)	1.6 (1.0 to 2.4)
Hemorrhagic stroke	Finland	2010	All Ages	0.1 (0.1 to 0.1)	5.7% (3.2 to 8.8%)	1.6 (0.9 to 2.4)
Hemorrhagic stroke	Finland	2015	All Ages	0.1 (0.1 to 0.1)	5.8% (3.3 to 9.1%)	1.6 (0.9 to 2.5)
Hemorrhagic stroke	Finland	1990	Age-standardized		6.9% (4.5 to 9.5%)	2.0 (1.3 to 2.8)
Hemorrhagic stroke	Finland	1995	Age-standardized		6.3% (4.0 to 8.9%)	1.6 (1.0 to 2.3)
Hemorrhagic stroke	Finland	2000	Age-standardized		6.1% (3.8 to 8.8%)	1.3 (0.8 to 1.9)
Hemorrhagic stroke	Finland	2005	Age-standardized		5.9% (3.6 to 8.6%)	1.0 (0.6 to 1.5)
Hemorrhagic stroke	Finland	2010	Age-standardized		5.9% (3.6 to 8.7%)	0.9 (0.6 to 1.4)
Hemorrhagic stroke	Finland	2015	Age-standardized		6.1% (3.8 to 9.0%)	0.9 (0.5 to 1.3)
Chronic obstructive pulmonary disease	Finland	1990	All Ages	0.1 (0.0 to 0.2)	8.4% (2.1 to 17.2%)	1.8 (0.4 to 3.8)
Chronic obstructive pulmonary disease	Finland	1995	All Ages	0.1 (0.0 to 0.2)	7.7% (1.8 to 16.4%)	1.7 (0.4 to 3.6)
Chronic obstructive pulmonary disease	Finland	2000	All Ages	0.1 (0.0 to 0.2)	7.3% (1.6 to 16.0%)	1.8 (0.4 to 3.9)
Chronic obstructive pulmonary disease	Finland	2005	All Ages	0.1 (0.0 to 0.2)	7.1% (1.5 to 15.6%)	1.7 (0.4 to 3.7)
Chronic obstructive pulmonary disease	Finland	2010	All Ages	0.1 (0.0 to 0.2)	7.3% (1.6 to 15.8%)	1.8 (0.4 to 4.1)
Chronic obstructive pulmonary disease	Finland	2015	All Ages	0.1 (0.0 to 0.2)	7.8% (1.8 to 16.5%)	2.0 (0.5 to 4.2)
Chronic obstructive pulmonary disease	Finland	1990	Age-standardized		8.4% (2.1 to 17.2%)	1.4 (0.3 to 2.9)
Chronic obstructive pulmonary disease	Finland	1995	Age-standardized		7.7% (1.8 to 16.4%)	1.2 (0.3 to 2.6)
Chronic obstructive pulmonary disease	Finland	2000	Age-standardized		7.3% (1.6 to 16.0%)	1.2 (0.3 to 2.6)
Chronic obstructive pulmonary disease	Finland	2005	Age-standardized		7.1% (1.5 to 15.6%)	1.0 (0.2 to 2.3)
Chronic obstructive pulmonary disease	Finland	2010	Age-standardized		7.3% (1.6 to 15.8%)	1.0 (0.2 to 2.2)
Chronic obstructive pulmonary disease	Finland	2015	Age-standardized		7.8% (1.8 to 16.5%)	1.0 (0.2 to 2.1)
All causes	France	1990	All Ages	21.0 (16.6 to 25.9)	4.1% (3.2 to 5.0%)	37.0 (29.3 to 45.6)
All causes	France	1995	All Ages	20.3 (15.9 to 25.2)	3.9% (3.0 to 4.8%)	35.0 (27.4 to 43.6)
All causes	France	2000	All Ages	19.4 (15.0 to 24.3)	3.6% (2.8 to 4.6%)	32.8 (25.5 to 41.2)
All causes	France	2005	All Ages	17.9 (13.7 to 22.7)	3.4% (2.6 to 4.3%)	29.2 (22.4 to 37.0)
All causes	France	2010	All Ages	17.8 (13.6 to 22.7)	3.3% (2.5 to 4.2%)	28.3 (21.6 to 36.0)
All causes	France	2015	All Ages	20.3 (15.3 to 26.1)	3.3% (2.5 to 4.2%)	31.1 (23.5 to 40.0)
All causes	France	1990	Age-standardized		3.9% (3.2 to 4.8%)	25.9 (21.0 to 31.2)
All causes	France	1995	Age-standardized		3.7% (3.0 to 4.5%)	23.0 (18.4 to 28.1)
All causes	France	2000	Age-standardized		3.5% (2.8 to 4.4%)	21.2 (16.7 to 26.3)
All causes	France	2005	Age-standardized		3.3% (2.6 to 4.1%)	16.9 (13.4 to 21.0)
All causes	France	2010	Age-standardized		3.1% (2.5 to 3.9%)	14.9 (11.8 to 18.5)

Cause name	Location	Year	Age group	Number (in thousands)	PAF	Rate (per 100,000)
All causes	France	2015	Age-standardized		3.2% (2.5 to 3.9%)	14.8 (11.6 to 18.4)
Lower respiratory infections	France	1990	All Ages	2.1 (0.7 to 4.0)	12.3% (4.3 to 23.1%)	3.7 (1.3 to 7.0)
Lower respiratory infections	France	1995	All Ages	2.3 (0.8 to 4.6)	11.6% (3.8 to 22.5%)	4.0 (1.3 to 8.0)
Lower respiratory infections	France	2000	All Ages	2.4 (0.7 to 4.8)	11.0% (3.4 to 21.9%)	4.0 (1.2 to 8.1)
Lower respiratory infections	France	2005	All Ages	2.2 (0.7 to 4.6)	10.7% (3.2 to 21.7%)	3.6 (1.1 to 7.5)
Lower respiratory infections	France	2010	All Ages	2.3 (0.7 to 4.9)	10.7% (3.2 to 21.7%)	3.7 (1.1 to 7.7)
Lower respiratory infections	France	2015	All Ages	2.7 (0.8 to 5.6)	10.8% (3.3 to 21.8%)	4.1 (1.2 to 8.5)
Lower respiratory infections	France	1990	Age-standardized		12.3% (4.3 to 23.1%)	2.4 (0.8 to 4.6)
Lower respiratory infections	France	1995	Age-standardized		11.6% (3.8 to 22.5%)	2.4 (0.8 to 4.8)
Lower respiratory infections	France	2000	Age-standardized		11.0% (3.4 to 21.9%)	2.5 (0.7 to 5.0)
Lower respiratory infections	France	2005	Age-standardized		10.7% (3.2 to 21.7%)	1.9 (0.6 to 4.0)
Lower respiratory infections	France	2010	Age-standardized		10.7% (3.2 to 21.7%)	1.7 (0.5 to 3.6)
Lower respiratory infections	France	2015	Age-standardized		10.8% (3.3 to 21.8%)	1.7 (0.5 to 3.6)
Tracheal, bronchus, and lung cancer	France	1990	All Ages	1.9 (0.9 to 3.1)	8.5% (4.2 to 13.9%)	3.4 (1.7 to 5.5)
Tracheal, bronchus, and lung cancer	France	1995	All Ages	2.0 (1.0 to 3.3)	8.0% (3.9 to 13.2%)	3.5 (1.7 to 5.7)
Tracheal, bronchus, and lung cancer	France	2000	All Ages	2.1 (1.0 to 3.5)	7.5% (3.6 to 12.6%)	3.6 (1.7 to 6.0)
Tracheal, bronchus, and lung cancer	France	2005	All Ages	2.2 (1.0 to 3.7)	7.3% (3.5 to 12.4%)	3.6 (1.7 to 6.1)
Tracheal, bronchus, and lung cancer	France	2010	All Ages	2.4 (1.1 to 4.0)	7.3% (3.4 to 12.3%)	3.8 (1.8 to 6.4)
Tracheal, bronchus, and lung cancer	France	2015	All Ages	2.5 (1.2 to 4.3)	7.4% (3.5 to 12.5%)	3.9 (1.8 to 6.6)
Tracheal, bronchus, and lung cancer	France	1990	Age-standardized		8.5% (4.2 to 13.9%)	2.7 (1.3 to 4.4)
Tracheal, bronchus, and lung cancer	France	1995	Age-standardized		8.0% (3.9 to 13.2%)	2.6 (1.3 to 4.3)
Tracheal, bronchus, and lung cancer	France	2000	Age-standardized		7.5% (3.6 to 12.6%)	2.6 (1.2 to 4.3)
Tracheal, bronchus, and lung cancer	France	2005	Age-standardized		7.3% (3.5 to 12.4%)	2.5 (1.2 to 4.2)
Tracheal, bronchus, and lung cancer	France	2010	Age-standardized		7.3% (3.4 to 12.3%)	2.4 (1.1 to 4.1)
Tracheal, bronchus, and lung cancer	France	2015	Age-standardized		7.4% (3.5 to 12.5%)	2.4 (1.1 to 4.0)
Cardiovascular diseases	France	1990	All Ages	14.1 (10.7 to 17.9)	8.0% (6.1 to 10.0%)	25.0 (18.8 to 31.6)
Cardiovascular diseases	France	1995	All Ages	13.2 (9.8 to 16.8)	7.6% (5.7 to 9.7%)	22.8 (16.9 to 29.0)
Cardiovascular diseases	France	2000	All Ages	12.5 (9.3 to 16.0)	7.3% (5.4 to 9.2%)	21.2 (15.7 to 27.1)
Cardiovascular diseases	France	2005	All Ages	11.3 (8.2 to 14.8)	7.0% (5.1 to 9.1%)	18.5 (13.4 to 24.2)
Cardiovascular diseases	France	2010	All Ages	11.0 (7.8 to 14.6)	6.8% (4.8 to 9.0%)	17.5 (12.4 to 23.1)
Cardiovascular diseases	France	2015	All Ages	12.5 (8.6 to 16.7)	6.7% (4.7 to 9.0%)	19.2 (13.2 to 25.7)
Cardiovascular diseases	France	1990	Age-standardized		8.3% (6.6 to 10.1%)	17.5 (13.5 to 21.6)
Cardiovascular diseases	France	1995	Age-standardized		7.9% (6.2 to 9.8%)	15.0 (11.6 to 18.6)
Cardiovascular diseases	France	2000	Age-standardized		7.5% (5.7 to 9.4%)	13.7 (10.3 to 17.2)
Cardiovascular diseases	France	2005	Age-standardized		7.3% (5.6 to 9.2%)	10.7 (8.1 to 13.4)
Cardiovascular diseases	France	2010	Age-standardized		7.3% (5.5 to 9.1%)	9.1 (6.9 to 11.6)

Cause name	Location	Year	Age group	Number (in thousands)	PAF	Rate (per 100,000)
Cardiovascular diseases	France	2015	Age-standardized		7.3% (5.4 to 9.2%)	9.0 (6.8 to 11.4)
Ischemic heart disease	France	1990	All Ages	9.6 (6.7 to 12.5)	11.8% (8.3 to 15.2%)	17.0 (11.8 to 22.1)
Ischemic heart disease	France	1995	All Ages	9.2 (6.3 to 12.2)	11.4% (7.8 to 15.0%)	15.9 (10.8 to 21.0)
Ischemic heart disease	France	2000	All Ages	8.8 (6.0 to 11.8)	11.1% (7.5 to 14.6%)	15.0 (10.1 to 20.0)
Ischemic heart disease	France	2005	All Ages	8.0 (5.3 to 10.9)	10.9% (7.1 to 14.5%)	13.1 (8.7 to 17.8)
Ischemic heart disease	France	2010	All Ages	7.8 (5.0 to 10.6)	10.7% (6.8 to 14.6%)	12.4 (7.9 to 16.9)
Ischemic heart disease	France	2015	All Ages	9.0 (5.5 to 12.6)	10.6% (6.5 to 14.8%)	13.7 (8.4 to 19.3)
Ischemic heart disease	France	1990	Age-standardized		12.3% (9.1 to 15.4%)	12.0 (8.8 to 15.2)
Ischemic heart disease	France	1995	Age-standardized		11.9% (8.6 to 15.1%)	10.6 (7.6 to 13.4)
Ischemic heart disease	France	2000	Age-standardized		11.4% (8.0 to 14.8%)	9.7 (6.8 to 12.7)
Ischemic heart disease	France	2005	Age-standardized		11.4% (8.0 to 14.7%)	7.6 (5.3 to 9.9)
Ischemic heart disease	France	2010	Age-standardized		11.4% (8.0 to 14.7%)	6.5 (4.5 to 8.5)
Ischemic heart disease	France	2015	Age-standardized		11.4% (7.9 to 14.8%)	6.5 (4.5 to 8.5)
Ischemic stroke	France	1990	All Ages	2.3 (1.2 to 3.7)	8.0% (4.2 to 12.7%)	4.1 (2.2 to 6.5)
Ischemic stroke	France	1995	All Ages	2.0 (1.0 to 3.3)	7.7% (3.8 to 12.7%)	3.4 (1.7 to 5.6)
Ischemic stroke	France	2000	All Ages	1.9 (1.0 to 3.0)	7.5% (3.8 to 12.3%)	3.1 (1.6 to 5.1)
Ischemic stroke	France	2005	All Ages	1.7 (0.8 to 2.8)	7.3% (3.6 to 12.2%)	2.7 (1.4 to 4.5)
Ischemic stroke	France	2010	All Ages	1.6 (0.8 to 2.8)	7.3% (3.4 to 12.5%)	2.6 (1.2 to 4.4)
Ischemic stroke	France	2015	All Ages	1.8 (0.8 to 3.1)	7.2% (3.2 to 12.7%)	2.8 (1.2 to 4.8)
Ischemic stroke	France	1990	Age-standardized		8.1% (4.5 to 12.5%)	2.7 (1.5 to 4.1)
Ischemic stroke	France	1995	Age-standardized		7.8% (4.2 to 12.3%)	2.1 (1.2 to 3.3)
Ischemic stroke	France	2000	Age-standardized		7.6% (4.0 to 12.3%)	1.9 (1.0 to 3.1)
Ischemic stroke	France	2005	Age-standardized		7.5% (4.0 to 12.1%)	1.4 (0.8 to 2.3)
Ischemic stroke	France	2010	Age-standardized		7.5% (3.9 to 12.2%)	1.2 (0.6 to 2.0)
Ischemic stroke	France	2015	Age-standardized		7.5% (3.9 to 12.3%)	1.2 (0.6 to 1.9)
Hemorrhagic stroke	France	1990	All Ages	2.2 (1.4 to 3.1)	8.8% (5.7 to 12.4%)	3.9 (2.5 to 5.5)
Hemorrhagic stroke	France	1995	All Ages	2.0 (1.3 to 2.9)	8.5% (5.3 to 12.4%)	3.5 (2.2 to 5.0)
Hemorrhagic stroke	France	2000	All Ages	1.9 (1.2 to 2.7)	8.2% (5.1 to 11.9%)	3.1 (1.9 to 4.6)
Hemorrhagic stroke	France	2005	All Ages	1.6 (1.0 to 2.5)	8.0% (4.9 to 12.0%)	2.7 (1.6 to 4.1)
Hemorrhagic stroke	France	2010	All Ages	1.6 (0.9 to 2.5)	7.9% (4.6 to 12.1%)	2.5 (1.5 to 3.9)
Hemorrhagic stroke	France	2015	All Ages	1.8 (1.0 to 2.8)	7.8% (4.2 to 12.3%)	2.7 (1.5 to 4.4)
Hemorrhagic stroke	France	1990	Age-standardized		9.1% (6.2 to 12.3%)	2.8 (1.9 to 3.8)
Hemorrhagic stroke	France	1995	Age-standardized		8.8% (5.9 to 12.1%)	2.3 (1.6 to 3.2)
Hemorrhagic stroke	France	2000	Age-standardized		8.5% (5.5 to 12.0%)	2.1 (1.3 to 2.9)
Hemorrhagic stroke	France	2005	Age-standardized		8.4% (5.5 to 11.9%)	1.6 (1.0 to 2.3)
Hemorrhagic stroke	France	2010	Age-standardized		8.4% (5.5 to 11.9%)	1.4 (0.9 to 2.0)

Cause name	Location	Year	Age group	Number (in thousands)	PAF	Rate (per 100,000)
Hemorrhagic stroke	France	2015	Age-standardized		8.3% (5.4 to 12.0%)	1.3 (0.9 to 1.9)
Chronic obstructive pulmonary disease	France	1990	All Ages	2.8 (1.2 to 4.8)	14.3% (6.0 to 24.6%)	5.0 (2.1 to 8.5)
Chronic obstructive pulmonary disease	France	1995	All Ages	2.7 (1.1 to 4.7)	13.7% (5.4 to 23.8%)	4.7 (1.8 to 8.1)
Chronic obstructive pulmonary disease	France	2000	All Ages	2.4 (0.9 to 4.1)	13.2% (5.1 to 23.2%)	4.0 (1.5 to 7.0)
Chronic obstructive pulmonary disease	France	2005	All Ages	2.1 (0.8 to 3.7)	12.9% (4.9 to 22.8%)	3.4 (1.3 to 6.0)
Chronic obstructive pulmonary disease	France	2010	All Ages	2.1 (0.8 to 3.8)	12.9% (4.8 to 22.9%)	3.4 (1.2 to 6.0)
Chronic obstructive pulmonary disease	France	2015	All Ages	2.5 (1.0 to 4.5)	13.0% (4.9 to 23.0%)	3.9 (1.5 to 6.9)
Chronic obstructive pulmonary disease	France	1990	Age-standardized		14.3% (6.0 to 24.5%)	3.3 (1.4 to 5.6)
Chronic obstructive pulmonary disease	France	1995	Age-standardized		13.7% (5.4 to 23.8%)	2.9 (1.1 to 5.0)
Chronic obstructive pulmonary disease	France	2000	Age-standardized		13.2% (5.1 to 23.2%)	2.5 (0.9 to 4.3)
Chronic obstructive pulmonary disease	France	2005	Age-standardized		12.9% (4.9 to 22.8%)	1.8 (0.7 to 3.3)
Chronic obstructive pulmonary disease	France	2010	Age-standardized		12.9% (4.8 to 22.8%)	1.6 (0.6 to 2.9)
Chronic obstructive pulmonary disease	France	2015	Age-standardized		13.0% (4.9 to 22.9%)	1.7 (0.6 to 3.0)
All causes	Germany	1990	All Ages	58.2 (47.2 to 69.6)	6.4% (5.2 to 7.6%)	73.5 (59.7 to 87.9)
All causes	Germany	1995	All Ages	50.2 (40.3 to 61.2)	5.7% (4.5 to 6.9%)	61.6 (49.4 to 75.1)
All causes	Germany	2000	All Ages	44.2 (35.1 to 54.3)	5.2% (4.2 to 6.4%)	53.9 (42.8 to 66.2)
All causes	Germany	2005	All Ages	39.7 (31.4 to 49.3)	4.8% (3.8 to 5.9%)	48.2 (38.1 to 59.9)
All causes	Germany	2010	All Ages	39.4 (30.7 to 49.2)	4.6% (3.6 to 5.7%)	48.3 (37.6 to 60.3)
All causes	Germany	2015	All Ages	43.1 (33.3 to 54.2)	4.6% (3.5 to 5.7%)	51.6 (39.9 to 64.8)
All causes	Germany	1990	Age-standardized		6.2% (5.1 to 7.4%)	48.6 (40.0 to 57.6)
All causes	Germany	1995	Age-standardized		5.5% (4.5 to 6.6%)	39.6 (32.3 to 47.5)
All causes	Germany	2000	Age-standardized		5.1% (4.1 to 6.2%)	33.7 (27.0 to 41.3)
All causes	Germany	2005	Age-standardized		4.6% (3.7 to 5.7%)	26.9 (21.6 to 33.1)
All causes	Germany	2010	Age-standardized		4.5% (3.6 to 5.5%)	23.9 (19.1 to 29.4)
All causes	Germany	2015	Age-standardized		4.4% (3.5 to 5.5%)	22.9 (18.2 to 28.1)
Lower respiratory infections	Germany	1990	All Ages	3.1 (1.3 to 5.3)	14.9% (6.5 to 25.4%)	3.9 (1.7 to 6.6)
Lower respiratory infections	Germany	1995	All Ages	2.7 (1.0 to 4.9)	13.2% (5.0 to 24.1%)	3.3 (1.3 to 6.1)
Lower respiratory infections	Germany	2000	All Ages	2.8 (1.0 to 5.4)	12.3% (4.3 to 23.2%)	3.5 (1.2 to 6.6)
Lower respiratory infections	Germany	2005	All Ages	3.1 (1.0 to 6.1)	11.7% (3.9 to 22.6%)	3.7 (1.2 to 7.4)
Lower respiratory infections	Germany	2010	All Ages	3.4 (1.2 to 6.6)	12.0% (4.1 to 22.9%)	4.2 (1.4 to 8.1)
Lower respiratory infections	Germany	2015	All Ages	3.9 (1.3 to 7.3)	12.2% (4.3 to 23.1%)	4.6 (1.6 to 8.8)
Lower respiratory infections	Germany	1990	Age-standardized		14.9% (6.5 to 25.4%)	2.5 (1.1 to 4.2)
Lower respiratory infections	Germany	1995	Age-standardized		13.2% (5.0 to 24.1%)	2.1 (0.8 to 3.7)
Lower respiratory infections	Germany	2000	Age-standardized		12.3% (4.3 to 23.2%)	2.2 (0.8 to 4.1)
Lower respiratory infections	Germany	2005	Age-standardized		11.7% (3.9 to 22.6%)	2.0 (0.7 to 4.0)
Lower respiratory infections	Germany	2010	Age-standardized		12.0% (4.1 to 22.9%)	2.0 (0.7 to 3.8)

Cause name	Location	Year	Age group	Number (in thousands)	PAF	Rate (per 100,000)
Lower respiratory infections	Germany	2015	Age-standardized		12.2% (4.3 to 23.1%)	2.0 (0.7 to 3.7)
Tracheal, bronchus, and lung cancer	Germany	1990	All Ages	3.9 (2.0 to 6.1)	10.4% (5.5 to 16.4%)	5.0 (2.6 to 7.7)
Tracheal, bronchus, and lung cancer	Germany	1995	All Ages	3.7 (1.8 to 5.9)	9.1% (4.6 to 14.7%)	4.5 (2.2 to 7.2)
Tracheal, bronchus, and lung cancer	Germany	2000	All Ages	3.6 (1.8 to 5.9)	8.5% (4.2 to 13.9%)	4.4 (2.2 to 7.2)
Tracheal, bronchus, and lung cancer	Germany	2005	All Ages	3.5 (1.7 to 5.8)	8.1% (3.9 to 13.4%)	4.3 (2.1 to 7.0)
Tracheal, bronchus, and lung cancer	Germany	2010	All Ages	3.8 (1.9 to 6.2)	8.2% (4.0 to 13.6%)	4.7 (2.3 to 7.6)
Tracheal, bronchus, and lung cancer	Germany	2015	All Ages	4.2 (2.0 to 6.8)	8.4% (4.1 to 13.8%)	5.0 (2.5 to 8.1)
Tracheal, bronchus, and lung cancer	Germany	1990	Age-standardized		10.4% (5.5 to 16.4%)	3.5 (1.8 to 5.5)
Tracheal, bronchus, and lung cancer	Germany	1995	Age-standardized		9.1% (4.6 to 14.7%)	3.1 (1.6 to 5.0)
Tracheal, bronchus, and lung cancer	Germany	2000	Age-standardized		8.5% (4.2 to 13.9%)	2.8 (1.4 to 4.7)
Tracheal, bronchus, and lung cancer	Germany	2005	Age-standardized		8.1% (3.9 to 13.4%)	2.6 (1.3 to 4.2)
Tracheal, bronchus, and lung cancer	Germany	2010	Age-standardized		8.2% (4.0 to 13.6%)	2.6 (1.3 to 4.2)
Tracheal, bronchus, and lung cancer	Germany	2015	Age-standardized		8.4% (4.1 to 13.8%)	2.5 (1.2 to 4.1)
Cardiovascular diseases	Germany	1990	All Ages	45.7 (35.6 to 56.0)	10.3% (8.0 to 12.6%)	57.8 (44.9 to 70.7)
Cardiovascular diseases	Germany	1995	All Ages	38.9 (29.6 to 48.9)	9.6% (7.3 to 12.0%)	47.7 (36.4 to 60.0)
Cardiovascular diseases	Germany	2000	All Ages	33.1 (25.0 to 41.7)	8.9% (6.7 to 11.1%)	40.4 (30.5 to 50.8)
Cardiovascular diseases	Germany	2005	All Ages	28.6 (21.2 to 36.6)	8.2% (6.1 to 10.4%)	34.8 (25.7 to 44.4)
Cardiovascular diseases	Germany	2010	All Ages	27.1 (19.8 to 35.0)	7.9% (5.7 to 10.1%)	33.2 (24.3 to 42.9)
Cardiovascular diseases	Germany	2015	All Ages	29.3 (21.1 to 38.1)	7.9% (5.7 to 10.2%)	35.0 (25.2 to 45.6)
Cardiovascular diseases	Germany	1990	Age-standardized		10.6% (8.5 to 12.8%)	38.2 (30.5 to 46.1)
Cardiovascular diseases	Germany	1995	Age-standardized		9.8% (7.7 to 12.0%)	30.6 (24.1 to 37.5)
Cardiovascular diseases	Germany	2000	Age-standardized		9.0% (6.8 to 11.2%)	25.3 (19.3 to 31.6)
Cardiovascular diseases	Germany	2005	Age-standardized		8.4% (6.4 to 10.6%)	19.4 (14.7 to 24.3)
Cardiovascular diseases	Germany	2010	Age-standardized		8.2% (6.1 to 10.3%)	16.3 (12.3 to 20.5)
Cardiovascular diseases	Germany	2015	Age-standardized		8.2% (6.1 to 10.3%)	15.4 (11.6 to 19.5)
Ischemic heart disease	Germany	1990	All Ages	35.2 (25.9 to 44.6)	13.0% (9.5 to 16.4%)	44.5 (32.7 to 56.4)
Ischemic heart disease	Germany	1995	All Ages	30.1 (21.4 to 39.0)	12.2% (8.7 to 15.7%)	37.0 (26.2 to 47.8)
Ischemic heart disease	Germany	2000	All Ages	26.0 (18.3 to 33.6)	11.8% (8.3 to 15.1%)	31.7 (22.3 to 41.0)
Ischemic heart disease	Germany	2005	All Ages	22.6 (15.5 to 29.7)	11.4% (7.8 to 14.9%)	27.4 (18.8 to 36.1)
Ischemic heart disease	Germany	2010	All Ages	21.3 (14.3 to 28.7)	11.3% (7.5 to 15.0%)	26.1 (17.5 to 35.1)
Ischemic heart disease	Germany	2015	All Ages	23.1 (15.2 to 31.3)	11.3% (7.4 to 15.1%)	27.6 (18.1 to 37.5)
Ischemic heart disease	Germany	1990	Age-standardized		13.4% (10.1 to 16.6%)	29.6 (22.3 to 36.8)
Ischemic heart disease	Germany	1995	Age-standardized		12.6% (9.3 to 15.8%)	23.9 (17.7 to 30.0)
Ischemic heart disease	Germany	2000	Age-standardized		11.9% (8.5 to 15.3%)	19.8 (14.1 to 25.7)
Ischemic heart disease	Germany	2005	Age-standardized		11.7% (8.2 to 15.0%)	15.3 (10.9 to 19.8)
Ischemic heart disease	Germany	2010	Age-standardized		11.7% (8.2 to 15.1%)	12.9 (9.0 to 16.8)

Cause name	Location	Year	Age group	Number (in thousands)	PAF	Rate (per 100,000)
Ischemic heart disease	Germany	2015	Age-standardized		11.8% (8.2 to 15.2%)	12.2 (8.5 to 16.1)
Ischemic stroke	Germany	1990	All Ages	6.4 (3.8 to 9.6)	9.0% (5.3 to 13.4%)	8.1 (4.8 to 12.1)
Ischemic stroke	Germany	1995	All Ages	5.1 (2.8 to 8.0)	8.4% (4.6 to 13.1%)	6.3 (3.4 to 9.8)
Ischemic stroke	Germany	2000	All Ages	4.1 (2.3 to 6.4)	8.1% (4.4 to 12.5%)	5.0 (2.8 to 7.8)
Ischemic stroke	Germany	2005	All Ages	3.5 (1.9 to 5.6)	7.8% (4.2 to 12.3%)	4.3 (2.3 to 6.8)
Ischemic stroke	Germany	2010	All Ages	3.4 (1.8 to 5.5)	7.9% (4.1 to 12.7%)	4.2 (2.2 to 6.8)
Ischemic stroke	Germany	2015	All Ages	3.6 (1.9 to 5.9)	7.9% (4.1 to 12.8%)	4.3 (2.3 to 7.0)
Ischemic stroke	Germany	1990	Age-standardized		9.1% (5.6 to 13.3%)	5.1 (3.1 to 7.4)
Ischemic stroke	Germany	1995	Age-standardized		8.5% (4.9 to 12.8%)	3.9 (2.3 to 5.8)
Ischemic stroke	Germany	2000	Age-standardized		8.1% (4.5 to 12.5%)	3.0 (1.7 to 4.7)
Ischemic stroke	Germany	2005	Age-standardized		7.9% (4.4 to 12.3%)	2.3 (1.3 to 3.6)
Ischemic stroke	Germany	2010	Age-standardized		8.0% (4.5 to 12.5%)	2.0 (1.1 to 3.1)
Ischemic stroke	Germany	2015	Age-standardized		8.1% (4.5 to 12.7%)	1.8 (1.0 to 2.8)
Hemorrhagic stroke	Germany	1990	All Ages	4.1 (2.9 to 5.5)	9.9% (6.9 to 13.2%)	5.2 (3.6 to 7.0)
Hemorrhagic stroke	Germany	1995	All Ages	3.7 (2.4 to 5.1)	9.2% (6.1 to 12.8%)	4.5 (3.0 to 6.3)
Hemorrhagic stroke	Germany	2000	All Ages	3.1 (2.0 to 4.3)	8.8% (5.7 to 12.2%)	3.8 (2.4 to 5.2)
Hemorrhagic stroke	Germany	2005	All Ages	2.6 (1.6 to 3.6)	8.5% (5.5 to 12.1%)	3.1 (2.0 to 4.4)
Hemorrhagic stroke	Germany	2010	All Ages	2.4 (1.5 to 3.4)	8.5% (5.3 to 12.3%)	2.9 (1.8 to 4.2)
Hemorrhagic stroke	Germany	2015	All Ages	2.5 (1.5 to 3.7)	8.5% (5.2 to 12.5%)	3.0 (1.8 to 4.5)
Hemorrhagic stroke	Germany	1990	Age-standardized		10.2% (7.3 to 13.3%)	3.5 (2.5 to 4.7)
Hemorrhagic stroke	Germany	1995	Age-standardized		9.5% (6.5 to 12.6%)	2.9 (2.0 to 4.0)
Hemorrhagic stroke	Germany	2000	Age-standardized		8.9% (5.9 to 12.4%)	2.4 (1.6 to 3.3)
Hemorrhagic stroke	Germany	2005	Age-standardized		8.7% (5.8 to 12.2%)	1.8 (1.2 to 2.5)
Hemorrhagic stroke	Germany	2010	Age-standardized		8.8% (5.8 to 12.4%)	1.5 (1.0 to 2.0)
Hemorrhagic stroke	Germany	2015	Age-standardized		8.9% (5.8 to 12.4%)	1.4 (0.9 to 1.9)
Chronic obstructive pulmonary disease	Germany	1990	All Ages	5.4 (2.4 to 8.9)	16.6% (7.4 to 27.3%)	6.9 (3.1 to 11.3)
Chronic obstructive pulmonary disease	Germany	1995	All Ages	4.9 (2.1 to 8.2)	15.1% (6.6 to 25.6%)	6.0 (2.6 to 10.0)
Chronic obstructive pulmonary disease	Germany	2000	All Ages	4.6 (1.9 to 7.8)	14.4% (5.9 to 24.6%)	5.6 (2.3 to 9.5)
Chronic obstructive pulmonary disease	Germany	2005	All Ages	4.5 (1.8 to 7.8)	13.8% (5.5 to 24.0%)	5.4 (2.2 to 9.5)
Chronic obstructive pulmonary disease	Germany	2010	All Ages	5.1 (2.1 to 8.7)	14.1% (5.7 to 24.3%)	6.2 (2.5 to 10.7)
Chronic obstructive pulmonary disease	Germany	2015	All Ages	5.8 (2.3 to 10.0)	14.3% (5.8 to 24.5%)	6.9 (2.8 to 12.0)
Chronic obstructive pulmonary disease	Germany	1990	Age-standardized		16.5% (7.4 to 27.2%)	4.4 (2.0 to 7.2)
Chronic obstructive pulmonary disease	Germany	1995	Age-standardized		15.1% (6.6 to 25.5%)	3.8 (1.7 to 6.3)
Chronic obstructive pulmonary disease	Germany	2000	Age-standardized		14.3% (5.9 to 24.5%)	3.4 (1.4 to 5.8)
Chronic obstructive pulmonary disease	Germany	2005	Age-standardized		13.8% (5.5 to 24.0%)	3.0 (1.2 to 5.2)
Chronic obstructive pulmonary disease	Germany	2010	Age-standardized		14.1% (5.7 to 24.2%)	3.0 (1.2 to 5.1)

Cause name	Location	Year	Age group	Number (in thousands)	PAF	Rate (per 100,000)
Chronic obstructive pulmonary disease	Germany	2015	Age-standardized		14.3% (5.8 to 24.5%)	3.0 (1.2 to 5.1)
All causes	Greece	1990	All Ages	5.3 (4.3 to 6.5)	5.7% (4.6 to 7.0%)	52.7 (42.6 to 64.2)
All causes	Greece	1995	All Ages	5.3 (4.3 to 6.6)	5.4% (4.4 to 6.7%)	50.0 (40.0 to 61.8)
All causes	Greece	2000	All Ages	5.5 (4.4 to 6.8)	5.4% (4.2 to 6.6%)	50.3 (39.7 to 61.9)
All causes	Greece	2005	All Ages	5.1 (4.0 to 6.4)	4.9% (3.8 to 6.1%)	46.2 (36.1 to 57.4)
All causes	Greece	2010	All Ages	5.6 (4.3 to 7.0)	5.1% (3.9 to 6.4%)	49.9 (38.1 to 62.8)
All causes	Greece	2015	All Ages	6.8 (5.3 to 8.6)	5.5% (4.3 to 7.0%)	61.8 (48.1 to 78.6)
All causes	Greece	1990	Age-standardized		5.6% (4.5 to 6.8%)	38.7 (31.4 to 46.8)
All causes	Greece	1995	Age-standardized		5.3% (4.3 to 6.5%)	34.9 (28.1 to 42.9)
All causes	Greece	2000	Age-standardized		5.3% (4.2 to 6.5%)	33.2 (26.5 to 40.8)
All causes	Greece	2005	Age-standardized		4.8% (3.8 to 6.0%)	27.7 (21.9 to 34.2)
All causes	Greece	2010	Age-standardized		5.0% (4.0 to 6.2%)	26.0 (20.6 to 32.0)
All causes	Greece	2015	Age-standardized		5.5% (4.4 to 6.7%)	27.4 (22.2 to 33.6)
Lower respiratory infections	Greece	1990	All Ages	0.2 (0.1 to 0.4)	11.8% (3.9 to 22.9%)	2.2 (0.8 to 4.3)
Lower respiratory infections	Greece	1995	All Ages	0.2 (0.1 to 0.5)	10.9% (3.3 to 22.1%)	2.2 (0.7 to 4.6)
Lower respiratory infections	Greece	2000	All Ages	0.3 (0.1 to 0.6)	10.5% (3.0 to 21.5%)	2.7 (0.8 to 5.5)
Lower respiratory infections	Greece	2005	All Ages	0.3 (0.1 to 0.6)	9.2% (2.2 to 20.3%)	2.5 (0.6 to 5.4)
Lower respiratory infections	Greece	2010	All Ages	0.3 (0.1 to 0.6)	10.2% (2.8 to 21.4%)	2.6 (0.7 to 5.3)
Lower respiratory infections	Greece	2015	All Ages	0.4 (0.1 to 0.7)	11.8% (4.0 to 22.8%)	3.4 (1.1 to 6.7)
Lower respiratory infections	Greece	1990	Age-standardized		11.8% (3.9 to 22.9%)	1.7 (0.6 to 3.3)
Lower respiratory infections	Greece	1995	Age-standardized		10.9% (3.3 to 22.1%)	1.6 (0.5 to 3.3)
Lower respiratory infections	Greece	2000	Age-standardized		10.5% (3.0 to 21.5%)	1.8 (0.5 to 3.7)
Lower respiratory infections	Greece	2005	Age-standardized		9.2% (2.2 to 20.3%)	1.5 (0.3 to 3.2)
Lower respiratory infections	Greece	2010	Age-standardized		10.2% (2.8 to 21.4%)	1.3 (0.3 to 2.6)
Lower respiratory infections	Greece	2015	Age-standardized		11.8% (4.0 to 22.8%)	1.4 (0.5 to 2.7)
Tracheal, bronchus, and lung cancer	Greece	1990	All Ages	0.4 (0.2 to 0.7)	8.1% (3.9 to 13.4%)	4.1 (2.0 to 6.8)
Tracheal, bronchus, and lung cancer	Greece	1995	All Ages	0.5 (0.2 to 0.8)	7.5% (3.5 to 12.4%)	4.2 (2.0 to 7.1)
Tracheal, bronchus, and lung cancer	Greece	2000	All Ages	0.5 (0.2 to 0.8)	7.2% (3.4 to 12.2%)	4.4 (2.1 to 7.5)
Tracheal, bronchus, and lung cancer	Greece	2005	All Ages	0.4 (0.2 to 0.8)	6.2% (2.8 to 11.0%)	4.0 (1.8 to 7.0)
Tracheal, bronchus, and lung cancer	Greece	2010	All Ages	0.5 (0.2 to 0.8)	6.9% (3.2 to 11.8%)	4.3 (2.0 to 7.3)
Tracheal, bronchus, and lung cancer	Greece	2015	All Ages	0.6 (0.3 to 1.0)	8.1% (4.0 to 13.4%)	5.3 (2.5 to 8.8)
Tracheal, bronchus, and lung cancer	Greece	1990	Age-standardized		8.1% (3.9 to 13.4%)	3.0 (1.5 to 5.0)
Tracheal, bronchus, and lung cancer	Greece	1995	Age-standardized		7.5% (3.5 to 12.4%)	3.0 (1.4 to 5.0)
Tracheal, bronchus, and lung cancer	Greece	2000	Age-standardized		7.2% (3.4 to 12.1%)	2.9 (1.4 to 5.0)
Tracheal, bronchus, and lung cancer	Greece	2005	Age-standardized		6.2% (2.8 to 11.0%)	2.5 (1.1 to 4.3)
Tracheal, bronchus, and lung cancer	Greece	2010	Age-standardized		6.9% (3.2 to 11.7%)	2.5 (1.2 to 4.3)

Cause name	Location	Year	Age group	Number (in thousands)	PAF	Rate (per 100,000)
Tracheal, bronchus, and lung cancer	Greece	2015	Age-standardized		8.1% (4.0 to 13.4%)	2.8 (1.3 to 4.6)
Cardiovascular diseases	Greece	1990	All Ages	4.3 (3.3 to 5.3)	9.1% (7.1 to 11.2%)	42.4 (32.9 to 52.2)
Cardiovascular diseases	Greece	1995	All Ages	4.2 (3.3 to 5.3)	8.7% (6.8 to 10.9%)	39.9 (30.9 to 49.9)
Cardiovascular diseases	Greece	2000	All Ages	4.3 (3.3 to 5.4)	8.7% (6.6 to 10.8%)	39.6 (29.8 to 49.3)
Cardiovascular diseases	Greece	2005	All Ages	4.0 (3.0 to 5.0)	8.1% (6.1 to 10.2%)	36.0 (26.8 to 45.6)
Cardiovascular diseases	Greece	2010	All Ages	4.1 (3.0 to 5.4)	8.2% (6.1 to 10.5%)	36.9 (27.2 to 47.9)
Cardiovascular diseases	Greece	2015	All Ages	5.0 (3.6 to 6.5)	8.6% (6.3 to 11.2%)	45.3 (33.2 to 59.7)
Cardiovascular diseases	Greece	1990	Age-standardized		9.2% (7.2 to 11.3%)	31.1 (24.2 to 38.2)
Cardiovascular diseases	Greece	1995	Age-standardized		8.8% (6.9 to 11.0%)	27.9 (21.8 to 34.6)
Cardiovascular diseases	Greece	2000	Age-standardized		8.8% (6.7 to 11.0%)	26.2 (19.9 to 32.6)
Cardiovascular diseases	Greece	2005	Age-standardized		8.3% (6.4 to 10.5%)	21.6 (16.4 to 27.2)
Cardiovascular diseases	Greece	2010	Age-standardized		8.8% (6.7 to 10.9%)	19.3 (14.8 to 24.2)
Cardiovascular diseases	Greece	2015	Age-standardized		9.3% (7.2 to 11.5%)	20.2 (15.7 to 25.3)
Ischemic heart disease	Greece	1990	All Ages	2.7 (2.0 to 3.4)	12.2% (9.1 to 15.2%)	26.8 (20.2 to 33.7)
Ischemic heart disease	Greece	1995	All Ages	2.8 (2.0 to 3.5)	11.7% (8.6 to 14.8%)	25.8 (18.9 to 32.9)
Ischemic heart disease	Greece	2000	All Ages	2.9 (2.1 to 3.6)	11.4% (8.3 to 14.7%)	26.1 (18.9 to 33.4)
Ischemic heart disease	Greece	2005	All Ages	2.7 (1.9 to 3.5)	10.7% (7.6 to 13.8%)	24.1 (17.1 to 31.3)
Ischemic heart disease	Greece	2010	All Ages	2.9 (2.0 to 3.8)	10.9% (7.3 to 14.4%)	25.8 (17.4 to 34.1)
Ischemic heart disease	Greece	2015	All Ages	3.4 (2.3 to 4.6)	11.3% (7.5 to 15.1%)	31.5 (20.9 to 42.2)
Ischemic heart disease	Greece	1990	Age-standardized		12.3% (9.3 to 15.3%)	19.8 (14.9 to 24.8)
Ischemic heart disease	Greece	1995	Age-standardized		11.8% (8.8 to 14.9%)	18.2 (13.5 to 22.9)
Ischemic heart disease	Greece	2000	Age-standardized		11.6% (8.5 to 14.8%)	17.4 (12.8 to 22.3)
Ischemic heart disease	Greece	2005	Age-standardized		11.0% (8.0 to 14.0%)	14.8 (10.6 to 18.9)
Ischemic heart disease	Greece	2010	Age-standardized		11.6% (8.4 to 14.7%)	13.9 (10.1 to 17.7)
Ischemic heart disease	Greece	2015	Age-standardized		12.2% (8.9 to 15.4%)	14.5 (10.7 to 18.5)
Ischemic stroke	Greece	1990	All Ages	0.8 (0.5 to 1.3)	7.9% (4.5 to 12.1%)	8.1 (4.5 to 12.5)
Ischemic stroke	Greece	1995	All Ages	0.8 (0.4 to 1.3)	7.5% (4.0 to 11.9%)	7.3 (3.9 to 11.7)
Ischemic stroke	Greece	2000	All Ages	0.8 (0.4 to 1.2)	7.3% (3.8 to 11.7%)	6.9 (3.6 to 11.1)
Ischemic stroke	Greece	2005	All Ages	0.7 (0.3 to 1.1)	6.8% (3.4 to 11.4%)	6.0 (2.9 to 10.0)
Ischemic stroke	Greece	2010	All Ages	0.6 (0.3 to 1.1)	7.1% (3.3 to 11.9%)	5.7 (2.7 to 9.7)
Ischemic stroke	Greece	2015	All Ages	0.8 (0.4 to 1.3)	7.6% (3.6 to 12.7%)	7.2 (3.5 to 12.2)
Ischemic stroke	Greece	1990	Age-standardized		7.9% (4.5 to 12.1%)	5.8 (3.2 to 9.0)
Ischemic stroke	Greece	1995	Age-standardized		7.5% (4.1 to 11.9%)	5.0 (2.6 to 7.9)
Ischemic stroke	Greece	2000	Age-standardized		7.3% (3.7 to 11.8%)	4.4 (2.3 to 7.1)
Ischemic stroke	Greece	2005	Age-standardized		6.8% (3.3 to 11.4%)	3.4 (1.6 to 5.6)
Ischemic stroke	Greece	2010	Age-standardized		7.2% (3.6 to 11.8%)	2.7 (1.3 to 4.4)

Cause name	Location	Year	Age group	Number (in thousands)	PAF	Rate (per 100,000)
Ischemic stroke	Greece	2015	Age-standardized		7.8% (4.0 to 12.5%)	2.8 (1.4 to 4.5)
Hemorrhagic stroke	Greece	1990	All Ages	0.8 (0.5 to 1.1)	8.6% (5.5 to 12.0%)	7.5 (4.9 to 10.5)
Hemorrhagic stroke	Greece	1995	All Ages	0.7 (0.5 to 1.1)	8.2% (5.1 to 11.7%)	6.8 (4.3 to 9.8)
Hemorrhagic stroke	Greece	2000	All Ages	0.7 (0.5 to 1.1)	8.0% (4.9 to 11.5%)	6.6 (4.1 to 9.6)
Hemorrhagic stroke	Greece	2005	All Ages	0.6 (0.4 to 1.0)	7.4% (4.4 to 10.9%)	5.8 (3.4 to 8.7)
Hemorrhagic stroke	Greece	2010	All Ages	0.6 (0.4 to 0.9)	7.6% (4.4 to 11.6%)	5.4 (3.1 to 8.4)
Hemorrhagic stroke	Greece	2015	All Ages	0.7 (0.4 to 1.1)	8.1% (4.5 to 12.6%)	6.6 (3.8 to 10.3)
Hemorrhagic stroke	Greece	1990	Age-standardized		8.6% (5.6 to 12.0%)	5.5 (3.6 to 7.7)
Hemorrhagic stroke	Greece	1995	Age-standardized		8.3% (5.2 to 11.7%)	4.7 (3.1 to 6.8)
Hemorrhagic stroke	Greece	2000	Age-standardized		8.0% (5.0 to 11.6%)	4.4 (2.7 to 6.3)
Hemorrhagic stroke	Greece	2005	Age-standardized		7.5% (4.6 to 11.1%)	3.4 (2.1 to 5.1)
Hemorrhagic stroke	Greece	2010	Age-standardized		8.0% (4.9 to 11.6%)	2.8 (1.8 to 4.1)
Hemorrhagic stroke	Greece	2015	Age-standardized		8.6% (5.5 to 12.2%)	2.9 (1.9 to 4.2)
Chronic obstructive pulmonary disease	Greece	1990	All Ages	0.4 (0.1 to 0.7)	13.9% (5.4 to 24.1%)	3.9 (1.5 to 6.8)
Chronic obstructive pulmonary disease	Greece	1995	All Ages	0.4 (0.1 to 0.7)	13.1% (4.9 to 23.1%)	3.6 (1.3 to 6.3)
Chronic obstructive pulmonary disease	Greece	2000	All Ages	0.4 (0.1 to 0.7)	12.7% (4.8 to 22.9%)	3.6 (1.3 to 6.5)
Chronic obstructive pulmonary disease	Greece	2005	All Ages	0.4 (0.1 to 0.8)	11.5% (3.9 to 21.0%)	3.7 (1.3 to 6.8)
Chronic obstructive pulmonary disease	Greece	2010	All Ages	0.7 (0.2 to 1.2)	12.4% (4.4 to 22.4%)	6.1 (2.1 to 11.0)
Chronic obstructive pulmonary disease	Greece	2015	All Ages	0.9 (0.3 to 1.5)	13.9% (5.4 to 24.1%)	7.9 (3.1 to 13.7)
Chronic obstructive pulmonary disease	Greece	1990	Age-standardized		13.9% (5.4 to 24.1%)	2.8 (1.1 to 4.9)
Chronic obstructive pulmonary disease	Greece	1995	Age-standardized		13.1% (4.9 to 23.1%)	2.4 (0.9 to 4.3)
Chronic obstructive pulmonary disease	Greece	2000	Age-standardized		12.7% (4.8 to 22.9%)	2.3 (0.9 to 4.2)
Chronic obstructive pulmonary disease	Greece	2005	Age-standardized		11.5% (3.9 to 21.0%)	2.1 (0.7 to 3.9)
Chronic obstructive pulmonary disease	Greece	2010	Age-standardized		12.4% (4.4 to 22.3%)	2.8 (1.0 to 5.2)
Chronic obstructive pulmonary disease	Greece	2015	Age-standardized		13.9% (5.4 to 24.1%)	3.0 (1.2 to 5.3)
All causes	Iceland	1990	All Ages	0.1 (0.1 to 0.1)	4.2% (3.0 to 5.5%)	28.6 (20.7 to 37.3)
All causes	Iceland	1995	All Ages	0.1 (0.1 to 0.1)	3.9% (2.8 to 5.1%)	26.1 (18.9 to 34.2)
All causes	Iceland	2000	All Ages	0.1 (0.1 to 0.1)	3.6% (2.6 to 4.8%)	22.3 (16.2 to 29.8)
All causes	Iceland	2005	All Ages	0.1 (0.0 to 0.1)	3.3% (2.4 to 4.5%)	19.8 (14.0 to 26.7)
All causes	Iceland	2010	All Ages	0.1 (0.0 to 0.1)	3.2% (2.2 to 4.3%)	18.0 (12.0 to 24.4)
All causes	Iceland	2015	All Ages	0.1 (0.0 to 0.1)	3.1% (2.1 to 4.3%)	18.4 (12.3 to 25.3)
All causes	Iceland	1990	Age-standardized		4.2% (3.1 to 5.5%)	27.1 (20.0 to 35.1)
All causes	Iceland	1995	Age-standardized		3.8% (2.8 to 5.0%)	23.5 (17.1 to 30.4)
All causes	Iceland	2000	Age-standardized		3.6% (2.6 to 4.7%)	19.3 (14.1 to 25.4)
All causes	Iceland	2005	Age-standardized		3.3% (2.4 to 4.4%)	15.7 (11.3 to 20.9)
All causes	Iceland	2010	Age-standardized		3.1% (2.2 to 4.2%)	13.3 (9.2 to 17.9)

Cause name	Location	Year	Age group	Number (in thousands)	PAF	Rate (per 100,000)
All causes	Iceland	2015	Age-standardized		3.1% (2.1 to 4.1%)	12.4 (8.6 to 16.6)
Lower respiratory infections	Iceland	1990	All Ages	0.0 (0.0 to 0.0)	6.8% (1.2 to 17.4%)	3.3 (0.6 to 8.7)
Lower respiratory infections	Iceland	1995	All Ages	0.0 (0.0 to 0.0)	6.6% (1.1 to 17.1%)	2.9 (0.5 to 7.8)
Lower respiratory infections	Iceland	2000	All Ages	0.0 (0.0 to 0.0)	6.4% (1.1 to 17.2%)	2.1 (0.4 to 5.8)
Lower respiratory infections	Iceland	2005	All Ages	0.0 (0.0 to 0.0)	6.2% (1.0 to 16.4%)	1.8 (0.3 to 4.8)
Lower respiratory infections	Iceland	2010	All Ages	0.0 (0.0 to 0.0)	6.2% (1.0 to 16.4%)	1.7 (0.3 to 4.5)
Lower respiratory infections	Iceland	2015	All Ages	0.0 (0.0 to 0.0)	6.1% (0.9 to 16.7%)	1.7 (0.3 to 4.8)
Lower respiratory infections	Iceland	1990	Age-standardized		6.8% (1.2 to 17.4%)	3.0 (0.5 to 7.9)
Lower respiratory infections	Iceland	1995	Age-standardized		6.6% (1.1 to 17.1%)	2.5 (0.4 to 6.8)
Lower respiratory infections	Iceland	2000	Age-standardized		6.4% (1.1 to 17.2%)	1.8 (0.3 to 4.9)
Lower respiratory infections	Iceland	2005	Age-standardized		6.2% (1.0 to 16.4%)	1.4 (0.2 to 3.7)
Lower respiratory infections	Iceland	2010	Age-standardized		6.2% (1.0 to 16.4%)	1.2 (0.2 to 3.2)
Lower respiratory infections	Iceland	2015	Age-standardized		6.1% (0.9 to 16.7%)	1.1 (0.2 to 3.1)
Tracheal, bronchus, and lung cancer	Iceland	1990	All Ages	0.0 (0.0 to 0.0)	4.5% (1.8 to 8.4%)	1.4 (0.6 to 2.6)
Tracheal, bronchus, and lung cancer	Iceland	1995	All Ages	0.0 (0.0 to 0.0)	4.3% (1.7 to 8.3%)	1.5 (0.6 to 2.8)
Tracheal, bronchus, and lung cancer	Iceland	2000	All Ages	0.0 (0.0 to 0.0)	4.2% (1.7 to 8.1%)	1.5 (0.6 to 2.8)
Tracheal, bronchus, and lung cancer	Iceland	2005	All Ages	0.0 (0.0 to 0.0)	4.1% (1.5 to 8.0%)	1.5 (0.5 to 2.8)
Tracheal, bronchus, and lung cancer	Iceland	2010	All Ages	0.0 (0.0 to 0.0)	4.1% (1.4 to 7.9%)	1.4 (0.5 to 2.7)
Tracheal, bronchus, and lung cancer	Iceland	2015	All Ages	0.0 (0.0 to 0.0)	4.0% (1.4 to 7.8%)	1.4 (0.5 to 2.6)
Tracheal, bronchus, and lung cancer	Iceland	1990	Age-standardized		4.5% (1.8 to 8.4%)	1.4 (0.6 to 2.6)
Tracheal, bronchus, and lung cancer	Iceland	1995	Age-standardized		4.3% (1.7 to 8.3%)	1.4 (0.5 to 2.6)
Tracheal, bronchus, and lung cancer	Iceland	2000	Age-standardized		4.2% (1.7 to 8.1%)	1.3 (0.5 to 2.6)
Tracheal, bronchus, and lung cancer	Iceland	2005	Age-standardized		4.1% (1.5 to 8.0%)	1.3 (0.5 to 2.4)
Tracheal, bronchus, and lung cancer	Iceland	2010	Age-standardized		4.1% (1.4 to 7.9%)	1.1 (0.4 to 2.2)
Tracheal, bronchus, and lung cancer	Iceland	2015	Age-standardized		4.0% (1.4 to 7.8%)	1.0 (0.4 to 2.0)
Cardiovascular diseases	Iceland	1990	All Ages	0.1 (0.0 to 0.1)	7.5% (5.5 to 9.7%)	22.0 (15.9 to 28.7)
Cardiovascular diseases	Iceland	1995	All Ages	0.1 (0.0 to 0.1)	7.1% (5.0 to 9.4%)	19.7 (13.9 to 26.1)
Cardiovascular diseases	Iceland	2000	All Ages	0.1 (0.0 to 0.1)	6.8% (4.7 to 9.0%)	16.6 (11.7 to 22.3)
Cardiovascular diseases	Iceland	2005	All Ages	0.0 (0.0 to 0.1)	6.5% (4.3 to 8.9%)	14.5 (9.5 to 19.9)
Cardiovascular diseases	Iceland	2010	All Ages	0.0 (0.0 to 0.1)	6.3% (4.2 to 8.7%)	13.1 (8.5 to 18.2)
Cardiovascular diseases	Iceland	2015	All Ages	0.0 (0.0 to 0.1)	6.3% (4.0 to 8.7%)	13.3 (8.5 to 18.7)
Cardiovascular diseases	Iceland	1990	Age-standardized		7.6% (5.7 to 9.7%)	21.0 (15.6 to 27.1)
Cardiovascular diseases	Iceland	1995	Age-standardized		7.3% (5.2 to 9.5%)	17.8 (12.8 to 23.3)
Cardiovascular diseases	Iceland	2000	Age-standardized		6.9% (4.9 to 9.1%)	14.4 (10.3 to 19.1)
Cardiovascular diseases	Iceland	2005	Age-standardized		6.7% (4.6 to 8.9%)	11.5 (7.9 to 15.4)
Cardiovascular diseases	Iceland	2010	Age-standardized		6.5% (4.5 to 8.7%)	9.7 (6.6 to 13.1)

Cause name	Location	Year	Age group	Number (in thousands)	PAF	Rate (per 100,000)
Cardiovascular diseases	Iceland	2015	Age-standardized		6.5% (4.4 to 8.6%)	9.0 (6.0 to 12.2)
Ischemic heart disease	Iceland	1990	All Ages	0.1 (0.0 to 0.1)	9.2% (6.4 to 12.2%)	17.9 (12.4 to 24.1)
Ischemic heart disease	Iceland	1995	All Ages	0.0 (0.0 to 0.1)	8.9% (6.0 to 12.1%)	15.8 (10.7 to 21.5)
Ischemic heart disease	Iceland	2000	All Ages	0.0 (0.0 to 0.1)	8.7% (5.7 to 12.0%)	13.3 (8.7 to 18.2)
Ischemic heart disease	Iceland	2005	All Ages	0.0 (0.0 to 0.1)	8.5% (5.3 to 11.9%)	11.6 (7.3 to 16.4)
Ischemic heart disease	Iceland	2010	All Ages	0.0 (0.0 to 0.1)	8.3% (5.1 to 11.9%)	10.5 (6.4 to 15.1)
Ischemic heart disease	Iceland	2015	All Ages	0.0 (0.0 to 0.1)	8.2% (4.9 to 11.8%)	10.8 (6.5 to 15.6)
Ischemic heart disease	Iceland	1990	Age-standardized		9.4% (6.6 to 12.2%)	17.2 (12.1 to 22.5)
Ischemic heart disease	Iceland	1995	Age-standardized		9.1% (6.3 to 12.2%)	14.4 (10.0 to 19.2)
Ischemic heart disease	Iceland	2000	Age-standardized		8.9% (5.9 to 12.0%)	11.6 (7.7 to 15.6)
Ischemic heart disease	Iceland	2005	Age-standardized		8.7% (5.6 to 12.0%)	9.3 (6.1 to 12.9)
Ischemic heart disease	Iceland	2010	Age-standardized		8.6% (5.5 to 11.8%)	7.8 (5.1 to 10.8)
Ischemic heart disease	Iceland	2015	Age-standardized		8.5% (5.4 to 11.8%)	7.4 (4.7 to 10.2)
Ischemic stroke	Iceland	1990	All Ages	0.0 (0.0 to 0.0)	5.8% (2.7 to 10.1%)	2.2 (1.0 to 3.7)
Ischemic stroke	Iceland	1995	All Ages	0.0 (0.0 to 0.0)	5.6% (2.4 to 9.8%)	2.1 (0.9 to 3.8)
Ischemic stroke	Iceland	2000	All Ages	0.0 (0.0 to 0.0)	5.5% (2.3 to 9.9%)	1.8 (0.7 to 3.3)
Ischemic stroke	Iceland	2005	All Ages	0.0 (0.0 to 0.0)	5.4% (2.1 to 10.0%)	1.5 (0.6 to 2.9)
Ischemic stroke	Iceland	2010	All Ages	0.0 (0.0 to 0.0)	5.3% (2.0 to 10.0%)	1.4 (0.5 to 2.6)
Ischemic stroke	Iceland	2015	All Ages	0.0 (0.0 to 0.0)	5.2% (1.9 to 10.2%)	1.3 (0.5 to 2.6)
Ischemic stroke	Iceland	1990	Age-standardized		5.9% (2.8 to 10.0%)	2.0 (1.0 to 3.4)
Ischemic stroke	Iceland	1995	Age-standardized		5.6% (2.5 to 9.8%)	1.8 (0.8 to 3.2)
Ischemic stroke	Iceland	2000	Age-standardized		5.5% (2.4 to 9.9%)	1.5 (0.6 to 2.8)
Ischemic stroke	Iceland	2005	Age-standardized		5.4% (2.3 to 9.9%)	1.2 (0.5 to 2.2)
Ischemic stroke	Iceland	2010	Age-standardized		5.4% (2.2 to 9.8%)	1.0 (0.4 to 1.7)
Ischemic stroke	Iceland	2015	Age-standardized		5.3% (2.2 to 9.8%)	0.8 (0.3 to 1.6)
Hemorrhagic stroke	Iceland	1990	All Ages	0.0 (0.0 to 0.0)	6.3% (3.6 to 9.7%)	1.9 (1.1 to 2.9)
Hemorrhagic stroke	Iceland	1995	All Ages	0.0 (0.0 to 0.0)	6.1% (3.4 to 9.6%)	1.8 (1.0 to 2.8)
Hemorrhagic stroke	Iceland	2000	All Ages	0.0 (0.0 to 0.0)	5.9% (3.2 to 9.7%)	1.6 (0.9 to 2.6)
Hemorrhagic stroke	Iceland	2005	All Ages	0.0 (0.0 to 0.0)	5.8% (2.9 to 9.6%)	1.4 (0.7 to 2.3)
Hemorrhagic stroke	Iceland	2010	All Ages	0.0 (0.0 to 0.0)	5.7% (2.7 to 9.6%)	1.2 (0.6 to 2.0)
Hemorrhagic stroke	Iceland	2015	All Ages	0.0 (0.0 to 0.0)	5.6% (2.7 to 9.8%)	1.2 (0.6 to 2.1)
Hemorrhagic stroke	Iceland	1990	Age-standardized		6.4% (3.8 to 9.6%)	1.8 (1.1 to 2.7)
Hemorrhagic stroke	Iceland	1995	Age-standardized		6.2% (3.5 to 9.5%)	1.6 (0.9 to 2.5)
Hemorrhagic stroke	Iceland	2000	Age-standardized		6.0% (3.4 to 9.7%)	1.4 (0.8 to 2.2)
Hemorrhagic stroke	Iceland	2005	Age-standardized		5.9% (3.2 to 9.6%)	1.1 (0.6 to 1.8)
Hemorrhagic stroke	Iceland	2010	Age-standardized		5.8% (3.0 to 9.5%)	0.9 (0.5 to 1.4)

Cause name	Location	Year	Age group	Number (in thousands)	PAF	Rate (per 100,000)
Hemorrhagic stroke	Iceland	2015	Age-standardized		5.7% (3.0 to 9.5%)	0.8 (0.4 to 1.4)
Chronic obstructive pulmonary disease	Iceland	1990	All Ages	0.0 (0.0 to 0.0)	9.0% (2.4 to 17.9%)	1.8 (0.5 to 3.7)
Chronic obstructive pulmonary disease	Iceland	1995	All Ages	0.0 (0.0 to 0.0)	8.7% (2.4 to 17.7%)	2.0 (0.6 to 4.0)
Chronic obstructive pulmonary disease	Iceland	2000	All Ages	0.0 (0.0 to 0.0)	8.5% (2.3 to 17.4%)	2.0 (0.5 to 4.2)
Chronic obstructive pulmonary disease	Iceland	2005	All Ages	0.0 (0.0 to 0.0)	8.4% (2.1 to 17.3%)	2.0 (0.5 to 4.3)
Chronic obstructive pulmonary disease	Iceland	2010	All Ages	0.0 (0.0 to 0.0)	8.3% (2.2 to 17.4%)	1.9 (0.5 to 4.0)
Chronic obstructive pulmonary disease	Iceland	2015	All Ages	0.0 (0.0 to 0.0)	8.2% (2.1 to 16.7%)	1.9 (0.5 to 4.0)
Chronic obstructive pulmonary disease	Iceland	1990	Age-standardized		9.0% (2.4 to 17.9%)	1.7 (0.5 to 3.5)
Chronic obstructive pulmonary disease	Iceland	1995	Age-standardized		8.7% (2.4 to 17.7%)	1.7 (0.5 to 3.6)
Chronic obstructive pulmonary disease	Iceland	2000	Age-standardized		8.5% (2.3 to 17.4%)	1.7 (0.4 to 3.5)
Chronic obstructive pulmonary disease	Iceland	2005	Age-standardized		8.4% (2.1 to 17.3%)	1.6 (0.4 to 3.3)
Chronic obstructive pulmonary disease	Iceland	2010	Age-standardized		8.3% (2.2 to 17.4%)	1.4 (0.4 to 2.9)
Chronic obstructive pulmonary disease	Iceland	2015	Age-standardized		8.2% (2.1 to 16.7%)	1.3 (0.3 to 2.6)
All causes	Ireland	1990	All Ages	1.5 (1.2 to 1.8)	5.5% (4.3 to 6.8%)	41.5 (32.6 to 51.2)
All causes	Ireland	1995	All Ages	1.4 (1.1 to 1.8)	5.2% (4.1 to 6.5%)	38.8 (30.3 to 48.6)
All causes	Ireland	2000	All Ages	1.3 (1.0 to 1.6)	4.7% (3.6 to 6.0%)	34.0 (25.9 to 43.0)
All causes	Ireland	2005	All Ages	1.1 (0.8 to 1.4)	4.1% (3.1 to 5.3%)	26.2 (19.9 to 33.7)
All causes	Ireland	2010	All Ages	1.0 (0.8 to 1.3)	3.9% (2.9 to 5.0%)	22.8 (17.1 to 29.5)
All causes	Ireland	2015	All Ages	1.1 (0.8 to 1.5)	4.0% (3.0 to 5.2%)	23.8 (17.7 to 31.0)
All causes	Ireland	1990	Age-standardized		5.5% (4.3 to 6.7%)	39.4 (31.0 to 48.4)
All causes	Ireland	1995	Age-standardized		5.2% (4.1 to 6.4%)	35.0 (27.5 to 43.6)
All causes	Ireland	2000	Age-standardized		4.7% (3.6 to 5.9%)	30.2 (23.1 to 38.1)
All causes	Ireland	2005	Age-standardized		4.1% (3.1 to 5.2%)	22.9 (17.5 to 29.2)
All causes	Ireland	2010	Age-standardized		3.8% (2.9 to 4.9%)	19.3 (14.6 to 24.8)
All causes	Ireland	2015	Age-standardized		4.0% (3.0 to 5.1%)	19.3 (14.5 to 24.9)
Lower respiratory infections	Ireland	1990	All Ages	0.1 (0.0 to 0.3)	9.4% (2.3 to 20.4%)	4.4 (1.1 to 9.5)
Lower respiratory infections	Ireland	1995	All Ages	0.2 (0.0 to 0.4)	8.8% (2.1 to 19.9%)	4.4 (1.0 to 9.7)
Lower respiratory infections	Ireland	2000	All Ages	0.2 (0.0 to 0.4)	8.4% (1.9 to 19.6%)	4.2 (1.0 to 9.7)
Lower respiratory infections	Ireland	2005	All Ages	0.1 (0.0 to 0.3)	7.8% (1.6 to 18.7%)	3.2 (0.6 to 7.5)
Lower respiratory infections	Ireland	2010	All Ages	0.1 (0.0 to 0.3)	7.9% (1.6 to 18.8%)	2.7 (0.6 to 6.6)
Lower respiratory infections	Ireland	2015	All Ages	0.1 (0.0 to 0.3)	8.4% (1.8 to 19.4%)	2.9 (0.7 to 6.9)
Lower respiratory infections	Ireland	1990	Age-standardized		9.4% (2.3 to 20.4%)	4.1 (1.0 to 9.1)
Lower respiratory infections	Ireland	1995	Age-standardized		8.8% (2.1 to 19.9%)	3.8 (0.9 to 8.5)
Lower respiratory infections	Ireland	2000	Age-standardized		8.4% (1.9 to 19.6%)	3.6 (0.8 to 8.4)
Lower respiratory infections	Ireland	2005	Age-standardized		7.8% (1.6 to 18.7%)	2.7 (0.5 to 6.4)
Lower respiratory infections	Ireland	2010	Age-standardized		7.9% (1.6 to 18.8%)	2.2 (0.5 to 5.4)

Cause name	Location	Year	Age group	Number (in thousands)	PAF	Rate (per 100,000)
Lower respiratory infections	Ireland	2015	Age-standardized		8.4% (1.8 to 19.4%)	2.3 (0.5 to 5.4)
Tracheal, bronchus, and lung cancer	Ireland	1990	All Ages	0.1 (0.0 to 0.1)	6.3% (2.9 to 10.9%)	2.2 (1.0 to 3.9)
Tracheal, bronchus, and lung cancer	Ireland	1995	All Ages	0.1 (0.0 to 0.1)	6.0% (2.6 to 10.4%)	2.2 (1.0 to 3.8)
Tracheal, bronchus, and lung cancer	Ireland	2000	All Ages	0.1 (0.0 to 0.1)	5.7% (2.4 to 10.1%)	2.2 (0.9 to 3.8)
Tracheal, bronchus, and lung cancer	Ireland	2005	All Ages	0.1 (0.0 to 0.1)	5.2% (2.1 to 9.4%)	1.9 (0.8 to 3.5)
Tracheal, bronchus, and lung cancer	Ireland	2010	All Ages	0.1 (0.0 to 0.2)	5.3% (2.2 to 9.4%)	1.9 (0.8 to 3.4)
Tracheal, bronchus, and lung cancer	Ireland	2015	All Ages	0.1 (0.0 to 0.2)	5.6% (2.4 to 9.9%)	2.0 (0.9 to 3.6)
Tracheal, bronchus, and lung cancer	Ireland	1990	Age-standardized		6.3% (2.9 to 10.9%)	2.1 (0.9 to 3.7)
Tracheal, bronchus, and lung cancer	Ireland	1995	Age-standardized		6.0% (2.6 to 10.4%)	2.0 (0.9 to 3.6)
Tracheal, bronchus, and lung cancer	Ireland	2000	Age-standardized		5.7% (2.4 to 10.1%)	2.0 (0.8 to 3.4)
Tracheal, bronchus, and lung cancer	Ireland	2005	Age-standardized		5.2% (2.1 to 9.4%)	1.7 (0.7 to 3.2)
Tracheal, bronchus, and lung cancer	Ireland	2010	Age-standardized		5.3% (2.2 to 9.4%)	1.7 (0.7 to 3.0)
Tracheal, bronchus, and lung cancer	Ireland	2015	Age-standardized		5.6% (2.4 to 9.9%)	1.7 (0.7 to 3.0)
Cardiovascular diseases	Ireland	1990	All Ages	1.0 (0.8 to 1.3)	9.0% (7.1 to 11.0%)	29.3 (22.6 to 36.3)
Cardiovascular diseases	Ireland	1995	All Ages	1.0 (0.7 to 1.2)	8.6% (6.6 to 10.6%)	26.8 (20.7 to 33.4)
Cardiovascular diseases	Ireland	2000	All Ages	0.9 (0.7 to 1.1)	8.2% (6.2 to 10.2%)	22.7 (17.2 to 28.6)
Cardiovascular diseases	Ireland	2005	All Ages	0.7 (0.5 to 0.9)	7.7% (5.6 to 9.8%)	17.2 (12.7 to 22.2)
Cardiovascular diseases	Ireland	2010	All Ages	0.7 (0.5 to 0.9)	7.5% (5.4 to 9.6%)	14.7 (10.8 to 19.1)
Cardiovascular diseases	Ireland	2015	All Ages	0.7 (0.5 to 1.0)	7.6% (5.5 to 9.9%)	15.1 (10.8 to 20.0)
Cardiovascular diseases	Ireland	1990	Age-standardized		9.1% (7.2 to 11.1%)	28.0 (21.7 to 34.6)
Cardiovascular diseases	Ireland	1995	Age-standardized		8.7% (6.8 to 10.7%)	24.3 (18.9 to 30.2)
Cardiovascular diseases	Ireland	2000	Age-standardized		8.3% (6.4 to 10.3%)	20.3 (15.5 to 25.4)
Cardiovascular diseases	Ireland	2005	Age-standardized		7.8% (5.8 to 9.8%)	15.1 (11.3 to 19.3)
Cardiovascular diseases	Ireland	2010	Age-standardized		7.6% (5.5 to 9.6%)	12.5 (9.2 to 16.0)
Cardiovascular diseases	Ireland	2015	Age-standardized		7.7% (5.6 to 9.9%)	12.3 (8.9 to 16.0)
Ischemic heart disease	Ireland	1990	All Ages	0.8 (0.6 to 1.1)	11.3% (8.5 to 14.2%)	23.8 (17.9 to 29.9)
Ischemic heart disease	Ireland	1995	All Ages	0.8 (0.6 to 1.0)	10.8% (8.0 to 13.7%)	21.8 (15.9 to 27.9)
Ischemic heart disease	Ireland	2000	All Ages	0.7 (0.5 to 0.9)	10.5% (7.6 to 13.4%)	18.4 (13.1 to 23.6)
Ischemic heart disease	Ireland	2005	All Ages	0.6 (0.4 to 0.8)	10.0% (6.9 to 13.1%)	13.9 (9.7 to 18.3)
Ischemic heart disease	Ireland	2010	All Ages	0.5 (0.4 to 0.7)	9.9% (6.6 to 13.2%)	11.7 (8.0 to 15.6)
Ischemic heart disease	Ireland	2015	All Ages	0.6 (0.4 to 0.8)	10.1% (6.7 to 13.6%)	12.1 (8.0 to 16.4)
Ischemic heart disease	Ireland	1990	Age-standardized		11.4% (8.6 to 14.3%)	22.7 (17.2 to 28.4)
Ischemic heart disease	Ireland	1995	Age-standardized		11.0% (8.2 to 13.8%)	19.9 (14.7 to 25.2)
Ischemic heart disease	Ireland	2000	Age-standardized		10.6% (7.8 to 13.5%)	16.4 (11.9 to 21.0)
Ischemic heart disease	Ireland	2005	Age-standardized		10.1% (7.1 to 13.1%)	12.2 (8.7 to 15.9)
Ischemic heart disease	Ireland	2010	Age-standardized		10.0% (6.9 to 13.1%)	10.0 (7.0 to 13.2)

Cause name	Location	Year	Age group	Number (in thousands)	PAF	Rate (per 100,000)
Ischemic heart disease	Ireland	2015	Age-standardized		10.2% (6.9 to 13.5%)	9.9 (6.7 to 13.2)
Ischemic stroke	Ireland	1990	All Ages	0.1 (0.1 to 0.2)	7.1% (3.9 to 11.1%)	2.9 (1.6 to 4.6)
Ischemic stroke	Ireland	1995	All Ages	0.1 (0.1 to 0.1)	6.8% (3.6 to 11.1%)	2.6 (1.3 to 4.2)
Ischemic stroke	Ireland	2000	All Ages	0.1 (0.0 to 0.1)	6.6% (3.3 to 11.0%)	2.2 (1.1 to 3.7)
Ischemic stroke	Ireland	2005	All Ages	0.1 (0.0 to 0.1)	6.2% (2.9 to 11.0%)	1.7 (0.8 to 3.0)
Ischemic stroke	Ireland	2010	All Ages	0.1 (0.0 to 0.1)	6.2% (2.7 to 11.0%)	1.5 (0.6 to 2.6)
Ischemic stroke	Ireland	2015	All Ages	0.1 (0.0 to 0.1)	6.4% (2.8 to 11.3%)	1.5 (0.6 to 2.7)
Ischemic stroke	Ireland	1990	Age-standardized		7.1% (3.8 to 11.2%)	2.7 (1.5 to 4.4)
Ischemic stroke	Ireland	1995	Age-standardized		6.9% (3.6 to 11.0%)	2.3 (1.2 to 3.6)
Ischemic stroke	Ireland	2000	Age-standardized		6.6% (3.4 to 10.9%)	1.9 (1.0 to 3.2)
Ischemic stroke	Ireland	2005	Age-standardized		6.3% (3.0 to 10.9%)	1.4 (0.7 to 2.5)
Ischemic stroke	Ireland	2010	Age-standardized		6.3% (2.8 to 11.0%)	1.2 (0.5 to 2.2)
Ischemic stroke	Ireland	2015	Age-standardized		6.4% (2.9 to 11.2%)	1.2 (0.5 to 2.1)
Hemorrhagic stroke	Ireland	1990	All Ages	0.1 (0.1 to 0.1)	8.0% (5.3 to 10.9%)	2.6 (1.8 to 3.6)
Hemorrhagic stroke	Ireland	1995	All Ages	0.1 (0.1 to 0.1)	7.7% (4.9 to 10.8%)	2.4 (1.6 to 3.3)
Hemorrhagic stroke	Ireland	2000	All Ages	0.1 (0.1 to 0.1)	7.5% (4.7 to 10.7%)	2.2 (1.4 to 3.1)
Hemorrhagic stroke	Ireland	2005	All Ages	0.1 (0.0 to 0.1)	7.0% (4.2 to 10.4%)	1.7 (1.0 to 2.5)
Hemorrhagic stroke	Ireland	2010	All Ages	0.1 (0.0 to 0.1)	6.9% (4.0 to 10.6%)	1.5 (0.9 to 2.3)
Hemorrhagic stroke	Ireland	2015	All Ages	0.1 (0.0 to 0.1)	7.1% (4.2 to 11.0%)	1.5 (0.9 to 2.3)
Hemorrhagic stroke	Ireland	1990	Age-standardized		8.1% (5.4 to 11.0%)	2.5 (1.7 to 3.5)
Hemorrhagic stroke	Ireland	1995	Age-standardized		7.8% (5.1 to 10.7%)	2.2 (1.4 to 3.0)
Hemorrhagic stroke	Ireland	2000	Age-standardized		7.6% (4.9 to 10.7%)	1.9 (1.2 to 2.7)
Hemorrhagic stroke	Ireland	2005	Age-standardized		7.1% (4.4 to 10.4%)	1.5 (0.9 to 2.2)
Hemorrhagic stroke	Ireland	2010	Age-standardized		7.0% (4.2 to 10.5%)	1.3 (0.8 to 1.9)
Hemorrhagic stroke	Ireland	2015	Age-standardized		7.2% (4.3 to 10.9%)	1.2 (0.7 to 1.9)
Chronic obstructive pulmonary disease	Ireland	1990	All Ages	0.2 (0.1 to 0.4)	11.6% (4.0 to 21.3%)	5.5 (1.8 to 10.1)
Chronic obstructive pulmonary disease	Ireland	1995	All Ages	0.2 (0.1 to 0.4)	11.1% (3.7 to 20.8%)	5.5 (1.7 to 10.3)
Chronic obstructive pulmonary disease	Ireland	2000	All Ages	0.2 (0.1 to 0.4)	10.7% (3.5 to 20.1%)	4.9 (1.6 to 9.1)
Chronic obstructive pulmonary disease	Ireland	2005	All Ages	0.2 (0.1 to 0.3)	10.1% (3.2 to 19.4%)	3.9 (1.2 to 7.3)
Chronic obstructive pulmonary disease	Ireland	2010	All Ages	0.2 (0.1 to 0.3)	10.2% (3.1 to 19.6%)	3.5 (1.1 to 6.9)
Chronic obstructive pulmonary disease	Ireland	2015	All Ages	0.2 (0.1 to 0.4)	10.7% (3.3 to 20.0%)	3.8 (1.2 to 7.3)
Chronic obstructive pulmonary disease	Ireland	1990	Age-standardized		11.6% (4.0 to 21.3%)	5.1 (1.7 to 9.4)
Chronic obstructive pulmonary disease	Ireland	1995	Age-standardized		11.1% (3.7 to 20.8%)	4.8 (1.5 to 9.1)
Chronic obstructive pulmonary disease	Ireland	2000	Age-standardized		10.7% (3.5 to 20.1%)	4.3 (1.4 to 8.0)
Chronic obstructive pulmonary disease	Ireland	2005	Age-standardized		10.1% (3.2 to 19.4%)	3.3 (1.0 to 6.3)
Chronic obstructive pulmonary disease	Ireland	2010	Age-standardized		10.2% (3.1 to 19.6%)	3.0 (0.9 to 5.8)

Cause name	Location	Year	Age group	Number (in thousands)	PAF	Rate (per 100,000)
Chronic obstructive pulmonary disease	Ireland	2015	Age-standardized		10.7% (3.3 to 20.0%)	3.0 (1.0 to 5.8)
All causes	Israel	1990	All Ages	1.7 (1.4 to 2.0)	6.3% (5.2 to 7.4%)	37.4 (31.2 to 43.9)
All causes	Israel	1995	All Ages	1.9 (1.5 to 2.2)	5.6% (4.6 to 6.6%)	34.8 (28.7 to 41.2)
All causes	Israel	2000	All Ages	1.7 (1.4 to 2.1)	4.8% (3.9 to 5.8%)	29.0 (23.6 to 35.0)
All causes	Israel	2005	All Ages	1.7 (1.4 to 2.1)	4.4% (3.6 to 5.4%)	25.7 (20.7 to 31.4)
All causes	Israel	2010	All Ages	1.8 (1.4 to 2.2)	4.4% (3.5 to 5.4%)	23.7 (19.1 to 29.2)
All causes	Israel	2015	All Ages	2.1 (1.7 to 2.6)	4.8% (3.9 to 5.8%)	26.5 (21.4 to 32.3)
All causes	Israel	1990	Age-standardized		6.4% (5.3 to 7.5%)	43.9 (36.5 to 51.5)
All causes	Israel	1995	Age-standardized		5.6% (4.7 to 6.7%)	37.3 (30.8 to 44.2)
All causes	Israel	2000	Age-standardized		4.9% (4.0 to 5.8%)	29.5 (24.2 to 35.6)
All causes	Israel	2005	Age-standardized		4.4% (3.6 to 5.4%)	24.6 (20.1 to 29.8)
All causes	Israel	2010	Age-standardized		4.4% (3.6 to 5.3%)	20.7 (17.0 to 25.2)
All causes	Israel	2015	Age-standardized		4.8% (3.9 to 5.7%)	22.2 (18.2 to 26.7)
Lower respiratory infections	Israel	1990	All Ages	0.1 (0.1 to 0.2)	15.2% (6.9 to 25.9%)	2.7 (1.2 to 4.5)
Lower respiratory infections	Israel	1995	All Ages	0.1 (0.1 to 0.2)	14.8% (6.4 to 25.2%)	2.3 (1.0 to 3.9)
Lower respiratory infections	Israel	2000	All Ages	0.2 (0.1 to 0.3)	14.7% (6.2 to 25.3%)	2.7 (1.2 to 4.7)
Lower respiratory infections	Israel	2005	All Ages	0.2 (0.1 to 0.4)	14.4% (6.1 to 25.0%)	3.2 (1.3 to 5.7)
Lower respiratory infections	Israel	2010	All Ages	0.3 (0.1 to 0.5)	15.4% (6.8 to 25.9%)	3.8 (1.7 to 6.4)
Lower respiratory infections	Israel	2015	All Ages	0.4 (0.2 to 0.6)	17.4% (8.8 to 27.7%)	4.6 (2.3 to 7.5)
Lower respiratory infections	Israel	1990	Age-standardized		15.2% (6.9 to 25.9%)	3.1 (1.4 to 5.2)
Lower respiratory infections	Israel	1995	Age-standardized		14.8% (6.4 to 25.2%)	2.4 (1.0 to 4.1)
Lower respiratory infections	Israel	2000	Age-standardized		14.7% (6.2 to 25.3%)	2.7 (1.2 to 4.7)
Lower respiratory infections	Israel	2005	Age-standardized		14.4% (6.1 to 25.0%)	3.0 (1.2 to 5.2)
Lower respiratory infections	Israel	2010	Age-standardized		15.4% (6.8 to 25.9%)	3.2 (1.4 to 5.4)
Lower respiratory infections	Israel	2015	Age-standardized		17.4% (8.8 to 27.7%)	3.7 (1.8 to 6.0)
Tracheal, bronchus, and lung cancer	Israel	1990	All Ages	0.1 (0.1 to 0.2)	10.6% (5.5 to 16.5%)	2.3 (1.2 to 3.6)
Tracheal, bronchus, and lung cancer	Israel	1995	All Ages	0.1 (0.1 to 0.2)	10.3% (5.3 to 16.2%)	2.6 (1.3 to 4.1)
Tracheal, bronchus, and lung cancer	Israel	2000	All Ages	0.2 (0.1 to 0.2)	10.2% (5.3 to 16.2%)	2.6 (1.3 to 4.0)
Tracheal, bronchus, and lung cancer	Israel	2005	All Ages	0.2 (0.1 to 0.3)	10.0% (5.2 to 15.7%)	2.6 (1.3 to 4.0)
Tracheal, bronchus, and lung cancer	Israel	2010	All Ages	0.2 (0.1 to 0.3)	10.7% (5.6 to 16.6%)	2.7 (1.4 to 4.1)
Tracheal, bronchus, and lung cancer	Israel	2015	All Ages	0.3 (0.1 to 0.4)	12.2% (6.6 to 18.7%)	3.1 (1.7 to 4.6)
Tracheal, bronchus, and lung cancer	Israel	1990	Age-standardized		10.6% (5.5 to 16.5%)	2.7 (1.4 to 4.3)
Tracheal, bronchus, and lung cancer	Israel	1995	Age-standardized		10.3% (5.3 to 16.2%)	2.8 (1.4 to 4.5)
Tracheal, bronchus, and lung cancer	Israel	2000	Age-standardized		10.2% (5.3 to 16.2%)	2.7 (1.4 to 4.2)
Tracheal, bronchus, and lung cancer	Israel	2005	Age-standardized		10.0% (5.2 to 15.7%)	2.6 (1.4 to 4.0)
Tracheal, bronchus, and lung cancer	Israel	2010	Age-standardized		10.7% (5.6 to 16.6%)	2.5 (1.3 to 3.9)

Cause name	Location	Year	Age group	Number (in thousands)	PAF	Rate (per 100,000)
Tracheal, bronchus, and lung cancer	Israel	2015	Age-standardized		12.2% (6.6 to 18.7%)	2.8 (1.5 to 4.2)
Cardiovascular diseases	Israel	1990	All Ages	1.3 (1.1 to 1.6)	11.4% (9.3 to 13.7%)	29.0 (23.5 to 34.7)
Cardiovascular diseases	Israel	1995	All Ages	1.4 (1.1 to 1.7)	10.8% (8.7 to 13.1%)	26.8 (21.4 to 32.3)
Cardiovascular diseases	Israel	2000	All Ages	1.2 (1.0 to 1.5)	10.2% (7.9 to 12.4%)	20.4 (15.8 to 24.8)
Cardiovascular diseases	Israel	2005	All Ages	1.1 (0.8 to 1.4)	9.5% (7.3 to 11.8%)	16.7 (12.8 to 20.8)
Cardiovascular diseases	Israel	2010	All Ages	1.1 (0.8 to 1.3)	9.3% (7.1 to 11.7%)	14.2 (10.7 to 17.8)
Cardiovascular diseases	Israel	2015	All Ages	1.2 (0.9 to 1.6)	9.7% (7.3 to 12.2%)	15.2 (11.5 to 19.2)
Cardiovascular diseases	Israel	1990	Age-standardized		11.5% (9.3 to 13.7%)	34.1 (27.5 to 40.7)
Cardiovascular diseases	Israel	1995	Age-standardized		10.9% (8.8 to 13.1%)	28.8 (23.1 to 34.5)
Cardiovascular diseases	Israel	2000	Age-standardized		10.3% (8.1 to 12.5%)	20.7 (16.3 to 25.3)
Cardiovascular diseases	Israel	2005	Age-standardized		9.7% (7.6 to 11.9%)	16.0 (12.5 to 19.6)
Cardiovascular diseases	Israel	2010	Age-standardized		9.6% (7.5 to 11.7%)	12.3 (9.6 to 15.2)
Cardiovascular diseases	Israel	2015	Age-standardized		10.0% (7.8 to 12.3%)	12.7 (9.9 to 15.7)
Ischemic heart disease	Israel	1990	All Ages	1.0 (0.8 to 1.3)	13.7% (10.5 to 16.8%)	23.1 (17.9 to 28.2)
Ischemic heart disease	Israel	1995	All Ages	1.1 (0.8 to 1.4)	13.2% (10.0 to 16.4%)	20.9 (15.8 to 26.0)
Ischemic heart disease	Israel	2000	All Ages	0.9 (0.7 to 1.2)	13.0% (9.5 to 16.3%)	15.4 (11.4 to 19.6)
Ischemic heart disease	Israel	2005	All Ages	0.8 (0.6 to 1.1)	12.6% (8.9 to 16.2%)	12.4 (8.8 to 15.9)
Ischemic heart disease	Israel	2010	All Ages	0.8 (0.5 to 1.0)	12.8% (8.8 to 16.5%)	10.3 (7.2 to 13.4)
Ischemic heart disease	Israel	2015	All Ages	0.9 (0.6 to 1.2)	13.3% (9.0 to 17.5%)	11.0 (7.5 to 14.5)
Ischemic heart disease	Israel	1990	Age-standardized		13.8% (10.6 to 16.8%)	27.1 (20.9 to 33.1)
Ischemic heart disease	Israel	1995	Age-standardized		13.4% (10.2 to 16.5%)	22.4 (17.0 to 28.0)
Ischemic heart disease	Israel	2000	Age-standardized		13.1% (9.8 to 16.4%)	15.8 (11.7 to 19.8)
Ischemic heart disease	Israel	2005	Age-standardized		12.8% (9.3 to 16.3%)	11.9 (8.6 to 15.1)
Ischemic heart disease	Israel	2010	Age-standardized		13.1% (9.5 to 16.6%)	9.0 (6.6 to 11.4)
Ischemic heart disease	Israel	2015	Age-standardized		13.7% (9.8 to 17.5%)	9.3 (6.7 to 11.9)
Ischemic stroke	Israel	1990	All Ages	0.1 (0.1 to 0.2)	9.6% (6.4 to 13.1%)	3.1 (2.1 to 4.3)
Ischemic stroke	Israel	1995	All Ages	0.1 (0.1 to 0.2)	9.3% (5.8 to 13.0%)	2.9 (1.9 to 4.0)
Ischemic stroke	Israel	2000	All Ages	0.1 (0.1 to 0.2)	9.1% (5.5 to 13.1%)	2.4 (1.5 to 3.4)
Ischemic stroke	Israel	2005	All Ages	0.1 (0.1 to 0.2)	8.8% (5.2 to 13.2%)	2.1 (1.3 to 3.2)
Ischemic stroke	Israel	2010	All Ages	0.1 (0.1 to 0.2)	9.0% (5.2 to 13.7%)	1.9 (1.1 to 2.9)
Ischemic stroke	Israel	2015	All Ages	0.2 (0.1 to 0.3)	9.6% (5.5 to 14.4%)	2.1 (1.2 to 3.1)
Ischemic stroke	Israel	1990	Age-standardized		9.6% (6.3 to 13.1%)	3.7 (2.4 to 5.0)
Ischemic stroke	Israel	1995	Age-standardized		9.3% (5.9 to 13.1%)	3.1 (2.0 to 4.3)
Ischemic stroke	Israel	2000	Age-standardized		9.1% (5.5 to 13.2%)	2.4 (1.5 to 3.4)
Ischemic stroke	Israel	2005	Age-standardized		8.9% (5.4 to 13.1%)	2.0 (1.2 to 2.9)
Ischemic stroke	Israel	2010	Age-standardized		9.2% (5.5 to 13.6%)	1.6 (1.0 to 2.4)

Cause name	Location	Year	Age group	Number (in thousands)	PAF	Rate (per 100,000)
Ischemic stroke	Israel	2015	Age-standardized		9.8% (5.9 to 14.2%)	1.7 (1.0 to 2.5)
Hemorrhagic stroke	Israel	1990	All Ages	0.1 (0.1 to 0.2)	10.4% (7.6 to 13.3%)	2.8 (2.0 to 3.6)
Hemorrhagic stroke	Israel	1995	All Ages	0.2 (0.1 to 0.2)	10.0% (7.2 to 13.1%)	3.0 (2.2 to 4.0)
Hemorrhagic stroke	Israel	2000	All Ages	0.1 (0.1 to 0.2)	9.8% (6.8 to 13.1%)	2.5 (1.7 to 3.4)
Hemorrhagic stroke	Israel	2005	All Ages	0.1 (0.1 to 0.2)	9.6% (6.4 to 13.1%)	2.2 (1.5 to 3.0)
Hemorrhagic stroke	Israel	2010	All Ages	0.1 (0.1 to 0.2)	9.7% (6.4 to 13.5%)	1.9 (1.3 to 2.6)
Hemorrhagic stroke	Israel	2015	All Ages	0.2 (0.1 to 0.2)	10.3% (6.8 to 14.3%)	2.1 (1.4 to 2.9)
Hemorrhagic stroke	Israel	1990	Age-standardized		10.5% (7.7 to 13.4%)	3.3 (2.4 to 4.3)
Hemorrhagic stroke	Israel	1995	Age-standardized		10.1% (7.2 to 13.1%)	3.3 (2.4 to 4.3)
Hemorrhagic stroke	Israel	2000	Age-standardized		9.9% (6.9 to 13.1%)	2.6 (1.8 to 3.5)
Hemorrhagic stroke	Israel	2005	Age-standardized		9.7% (6.7 to 13.0%)	2.1 (1.5 to 2.9)
Hemorrhagic stroke	Israel	2010	Age-standardized		10.0% (6.9 to 13.3%)	1.7 (1.2 to 2.3)
Hemorrhagic stroke	Israel	2015	Age-standardized		10.6% (7.4 to 14.1%)	1.8 (1.2 to 2.4)
Chronic obstructive pulmonary disease	Israel	1990	All Ages	0.1 (0.1 to 0.3)	16.8% (7.7 to 27.3%)	3.4 (1.6 to 5.6)
Chronic obstructive pulmonary disease	Israel	1995	All Ages	0.2 (0.1 to 0.3)	16.4% (7.3 to 27.1%)	3.1 (1.4 to 5.1)
Chronic obstructive pulmonary disease	Israel	2000	All Ages	0.2 (0.1 to 0.3)	16.4% (7.5 to 26.9%)	3.3 (1.5 to 5.5)
Chronic obstructive pulmonary disease	Israel	2005	All Ages	0.2 (0.1 to 0.4)	16.1% (7.0 to 26.7%)	3.2 (1.4 to 5.3)
Chronic obstructive pulmonary disease	Israel	2010	All Ages	0.2 (0.1 to 0.4)	17.0% (7.7 to 27.5%)	3.1 (1.4 to 5.1)
Chronic obstructive pulmonary disease	Israel	2015	All Ages	0.3 (0.1 to 0.5)	18.7% (8.9 to 29.6%)	3.6 (1.7 to 5.7)
Chronic obstructive pulmonary disease	Israel	1990	Age-standardized		16.8% (7.7 to 27.4%)	4.0 (1.8 to 6.5)
Chronic obstructive pulmonary disease	Israel	1995	Age-standardized		16.4% (7.3 to 27.1%)	3.3 (1.4 to 5.4)
Chronic obstructive pulmonary disease	Israel	2000	Age-standardized		16.4% (7.5 to 26.9%)	3.4 (1.5 to 5.5)
Chronic obstructive pulmonary disease	Israel	2005	Age-standardized		16.1% (7.0 to 26.7%)	3.0 (1.3 to 5.0)
Chronic obstructive pulmonary disease	Israel	2010	Age-standardized		17.0% (7.7 to 27.5%)	2.7 (1.2 to 4.3)
Chronic obstructive pulmonary disease	Israel	2015	Age-standardized		18.7% (8.9 to 29.6%)	2.9 (1.4 to 4.7)
All causes	Italy	1990	All Ages	29.5 (24.4 to 35.1)	5.4% (4.5 to 6.4%)	51.8 (42.9 to 61.6)
All causes	Italy	1995	All Ages	27.9 (22.6 to 34.1)	5.0% (4.0 to 6.0%)	49.0 (39.7 to 59.8)
All causes	Italy	2000	All Ages	26.4 (21.3 to 32.5)	4.7% (3.8 to 5.7%)	46.3 (37.3 to 56.9)
All causes	Italy	2005	All Ages	24.6 (19.4 to 30.8)	4.4% (3.5 to 5.5%)	42.1 (33.2 to 52.6)
All causes	Italy	2010	All Ages	25.1 (19.6 to 31.9)	4.2% (3.3 to 5.3%)	41.5 (32.4 to 52.6)
All causes	Italy	2015	All Ages	33.7 (26.3 to 42.1)	4.9% (3.9 to 6.2%)	53.7 (41.9 to 67.1)
All causes	Italy	1990	Age-standardized		5.3% (4.4 to 6.2%)	35.9 (29.9 to 42.5)
All causes	Italy	1995	Age-standardized		4.8% (4.0 to 5.8%)	30.2 (24.9 to 36.2)
All causes	Italy	2000	Age-standardized		4.5% (3.7 to 5.5%)	26.3 (21.5 to 32.2)
All causes	Italy	2005	Age-standardized		4.3% (3.4 to 5.2%)	21.3 (17.1 to 26.1)
All causes	Italy	2010	Age-standardized		4.1% (3.3 to 5.0%)	18.9 (15.2 to 23.5)

Cause name	Location	Year	Age group	Number (in thousands)	PAF	Rate (per 100,000)
All causes	Italy	2015	Age-standardized		4.8% (3.9 to 5.8%)	22.2 (18.0 to 27.0)
Lower respiratory infections	Italy	1990	All Ages	1.5 (0.7 to 2.5)	15.6% (7.4 to 25.9%)	2.6 (1.2 to 4.4)
Lower respiratory infections	Italy	1995	All Ages	1.4 (0.6 to 2.3)	14.4% (6.2 to 24.9%)	2.4 (1.0 to 4.1)
Lower respiratory infections	Italy	2000	All Ages	1.4 (0.6 to 2.5)	13.3% (5.3 to 24.1%)	2.5 (1.0 to 4.4)
Lower respiratory infections	Italy	2005	All Ages	1.4 (0.5 to 2.7)	12.4% (4.5 to 23.3%)	2.4 (0.9 to 4.7)
Lower respiratory infections	Italy	2010	All Ages	1.6 (0.6 to 3.1)	12.7% (4.7 to 23.6%)	2.7 (1.0 to 5.1)
Lower respiratory infections	Italy	2015	All Ages	2.5 (1.2 to 4.2)	16.5% (8.2 to 26.7%)	4.0 (1.9 to 6.7)
Lower respiratory infections	Italy	1990	Age-standardized		15.6% (7.4 to 25.9%)	1.9 (0.9 to 3.1)
Lower respiratory infections	Italy	1995	Age-standardized		14.4% (6.2 to 24.9%)	1.4 (0.6 to 2.5)
Lower respiratory infections	Italy	2000	Age-standardized		13.3% (5.3 to 24.1%)	1.4 (0.5 to 2.5)
Lower respiratory infections	Italy	2005	Age-standardized		12.4% (4.5 to 23.3%)	1.2 (0.4 to 2.3)
Lower respiratory infections	Italy	2010	Age-standardized		12.7% (4.7 to 23.6%)	1.2 (0.4 to 2.2)
Lower respiratory infections	Italy	2015	Age-standardized		16.5% (8.2 to 26.7%)	1.5 (0.7 to 2.6)
Tracheal, bronchus, and lung cancer	Italy	1990	All Ages	3.4 (1.8 to 5.3)	10.9% (5.8 to 17.0%)	6.0 (3.2 to 9.3)
Tracheal, bronchus, and lung cancer	Italy	1995	All Ages	3.3 (1.7 to 5.2)	10.0% (5.2 to 15.9%)	5.8 (3.0 to 9.2)
Tracheal, bronchus, and lung cancer	Italy	2000	All Ages	3.1 (1.6 to 5.0)	9.2% (4.7 to 14.9%)	5.4 (2.8 to 8.7)
Tracheal, bronchus, and lung cancer	Italy	2005	All Ages	2.6 (1.3 to 4.2)	8.5% (4.2 to 14.0%)	4.4 (2.2 to 7.1)
Tracheal, bronchus, and lung cancer	Italy	2010	All Ages	3.1 (1.5 to 4.9)	8.8% (4.4 to 14.3%)	5.0 (2.5 to 8.1)
Tracheal, bronchus, and lung cancer	Italy	2015	All Ages	4.3 (2.3 to 6.6)	11.6% (6.3 to 17.8%)	6.9 (3.7 to 10.5)
Tracheal, bronchus, and lung cancer	Italy	1990	Age-standardized		10.9% (5.8 to 17.0%)	4.3 (2.3 to 6.7)
Tracheal, bronchus, and lung cancer	Italy	1995	Age-standardized		10.0% (5.2 to 15.9%)	3.8 (2.0 to 6.1)
Tracheal, bronchus, and lung cancer	Italy	2000	Age-standardized		9.2% (4.7 to 14.9%)	3.3 (1.7 to 5.2)
Tracheal, bronchus, and lung cancer	Italy	2005	Age-standardized		8.5% (4.2 to 14.0%)	2.5 (1.2 to 4.0)
Tracheal, bronchus, and lung cancer	Italy	2010	Age-standardized		8.8% (4.4 to 14.3%)	2.6 (1.3 to 4.2)
Tracheal, bronchus, and lung cancer	Italy	2015	Age-standardized		11.6% (6.3 to 17.8%)	3.4 (1.8 to 5.2)
Cardiovascular diseases	Italy	1990	All Ages	21.2 (16.7 to 25.7)	9.2% (7.3 to 11.2%)	37.3 (29.3 to 45.2)
Cardiovascular diseases	Italy	1995	All Ages	20.0 (15.3 to 24.9)	8.7% (6.7 to 10.8%)	35.2 (26.9 to 43.8)
Cardiovascular diseases	Italy	2000	All Ages	18.6 (14.0 to 23.4)	8.2% (6.2 to 10.3%)	32.6 (24.5 to 41.0)
Cardiovascular diseases	Italy	2005	All Ages	17.2 (12.6 to 22.0)	7.7% (5.7 to 10.0%)	29.3 (21.5 to 37.5)
Cardiovascular diseases	Italy	2010	All Ages	16.8 (11.9 to 21.9)	7.6% (5.4 to 9.9%)	27.7 (19.7 to 36.1)
Cardiovascular diseases	Italy	2015	All Ages	21.7 (15.6 to 28.0)	8.5% (6.1 to 10.9%)	34.6 (24.8 to 44.6)
Cardiovascular diseases	Italy	1990	Age-standardized		9.4% (7.5 to 11.3%)	25.8 (20.5 to 31.1)
Cardiovascular diseases	Italy	1995	Age-standardized		8.9% (7.0 to 10.9%)	21.6 (16.9 to 26.2)
Cardiovascular diseases	Italy	2000	Age-standardized		8.4% (6.5 to 10.5%)	18.6 (14.2 to 23.0)
Cardiovascular diseases	Italy	2005	Age-standardized		8.1% (6.2 to 10.2%)	14.8 (11.2 to 18.4)
Cardiovascular diseases	Italy	2010	Age-standardized		8.1% (6.1 to 10.1%)	12.6 (9.4 to 15.8)

Cause name	Location	Year	Age group	Number (in thousands)	PAF	Rate (per 100,000)
Cardiovascular diseases	Italy	2015	Age-standardized		9.0% (7.0 to 11.2%)	14.2 (10.8 to 17.7)
Ischemic heart disease	Italy	1990	All Ages	14.1 (10.5 to 17.7)	13.3% (10.0 to 16.7%)	24.7 (18.4 to 31.1)
Ischemic heart disease	Italy	1995	All Ages	13.5 (9.7 to 17.4)	12.7% (9.1 to 16.2%)	23.7 (17.0 to 30.5)
Ischemic heart disease	Italy	2000	All Ages	12.6 (8.8 to 16.5)	12.1% (8.5 to 15.6%)	22.2 (15.5 to 28.9)
Ischemic heart disease	Italy	2005	All Ages	11.7 (7.9 to 15.6)	11.5% (7.7 to 15.1%)	20.0 (13.5 to 26.7)
Ischemic heart disease	Italy	2010	All Ages	11.4 (7.4 to 15.7)	11.4% (7.3 to 15.4%)	18.9 (12.2 to 25.9)
Ischemic heart disease	Italy	2015	All Ages	14.8 (9.5 to 20.1)	12.5% (8.0 to 17.0%)	23.6 (15.1 to 32.0)
Ischemic heart disease	Italy	1990	Age-standardized		13.6% (10.3 to 16.8%)	17.2 (13.0 to 21.5)
Ischemic heart disease	Italy	1995	Age-standardized		13.0% (9.7 to 16.3%)	14.7 (11.0 to 18.4)
Ischemic heart disease	Italy	2000	Age-standardized		12.4% (8.9 to 15.8%)	12.7 (9.1 to 16.4)
Ischemic heart disease	Italy	2005	Age-standardized		12.0% (8.5 to 15.3%)	10.3 (7.4 to 13.3)
Ischemic heart disease	Italy	2010	Age-standardized		12.0% (8.5 to 15.6%)	8.7 (6.2 to 11.4)
Ischemic heart disease	Italy	2015	Age-standardized		13.3% (9.5 to 17.1%)	9.9 (7.0 to 12.7)
Ischemic stroke	Italy	1990	All Ages	4.2 (2.5 to 6.1)	9.2% (5.6 to 13.5%)	7.3 (4.5 to 10.7)
Ischemic stroke	Italy	1995	All Ages	3.7 (2.0 to 5.7)	8.6% (4.9 to 13.3%)	6.5 (3.6 to 10.0)
Ischemic stroke	Italy	2000	All Ages	3.3 (1.7 to 5.2)	8.2% (4.4 to 13.0%)	5.7 (3.0 to 9.0)
Ischemic stroke	Italy	2005	All Ages	2.8 (1.4 to 4.6)	7.8% (3.9 to 12.9%)	4.8 (2.4 to 7.8)
Ischemic stroke	Italy	2010	All Ages	2.7 (1.3 to 4.5)	7.8% (3.7 to 13.3%)	4.4 (2.1 to 7.5)
Ischemic stroke	Italy	2015	All Ages	3.5 (1.8 to 5.7)	8.9% (4.4 to 14.5%)	5.6 (2.8 to 9.1)
Ischemic stroke	Italy	1990	Age-standardized		9.2% (5.7 to 13.5%)	4.9 (3.0 to 7.1)
Ischemic stroke	Italy	1995	Age-standardized		8.8% (5.2 to 13.2%)	3.8 (2.2 to 5.6)
Ischemic stroke	Italy	2000	Age-standardized		8.3% (4.5 to 13.0%)	3.1 (1.7 to 4.8)
Ischemic stroke	Italy	2005	Age-standardized		7.9% (4.2 to 12.8%)	2.2 (1.2 to 3.6)
Ischemic stroke	Italy	2010	Age-standardized		8.0% (4.1 to 13.1%)	1.8 (0.9 to 3.0)
Ischemic stroke	Italy	2015	Age-standardized		9.1% (5.0 to 14.2%)	2.0 (1.1 to 3.2)
Hemorrhagic stroke	Italy	1990	All Ages	3.0 (2.1 to 4.0)	10.1% (7.1 to 13.5%)	5.3 (3.7 to 7.0)
Hemorrhagic stroke	Italy	1995	All Ages	2.9 (1.9 to 4.0)	9.5% (6.3 to 13.1%)	5.0 (3.3 to 7.0)
Hemorrhagic stroke	Italy	2000	All Ages	2.7 (1.7 to 3.8)	9.0% (5.8 to 12.6%)	4.7 (3.0 to 6.7)
Hemorrhagic stroke	Italy	2005	All Ages	2.6 (1.6 to 4.0)	8.4% (5.1 to 12.4%)	4.5 (2.8 to 6.7)
Hemorrhagic stroke	Italy	2010	All Ages	2.7 (1.6 to 4.1)	8.4% (4.9 to 12.8%)	4.4 (2.6 to 6.8)
Hemorrhagic stroke	Italy	2015	All Ages	3.4 (2.0 to 5.1)	9.5% (5.7 to 14.2%)	5.5 (3.3 to 8.2)
Hemorrhagic stroke	Italy	1990	Age-standardized		10.3% (7.3 to 13.5%)	3.7 (2.6 to 4.9)
Hemorrhagic stroke	Italy	1995	Age-standardized		9.8% (6.7 to 13.0%)	3.1 (2.2 to 4.2)
Hemorrhagic stroke	Italy	2000	Age-standardized		9.2% (6.2 to 12.7%)	2.7 (1.8 to 3.8)
Hemorrhagic stroke	Italy	2005	Age-standardized		8.8% (5.7 to 12.4%)	2.3 (1.5 to 3.3)
Hemorrhagic stroke	Italy	2010	Age-standardized		8.8% (5.7 to 12.6%)	2.0 (1.3 to 3.0)

Cause name	Location	Year	Age group	Number (in thousands)	PAF	Rate (per 100,000)
Hemorrhagic stroke	Italy	2015	Age-standardized		10.1% (6.7 to 14.1%)	2.3 (1.5 to 3.2)
Chronic obstructive pulmonary disease	Italy	1990	All Ages	3.3 (1.5 to 5.4)	17.1% (7.9 to 27.8%)	5.8 (2.6 to 9.5)
Chronic obstructive pulmonary disease	Italy	1995	All Ages	3.2 (1.4 to 5.3)	16.1% (7.2 to 26.8%)	5.7 (2.5 to 9.3)
Chronic obstructive pulmonary disease	Italy	2000	All Ages	3.3 (1.4 to 5.5)	15.2% (6.7 to 25.7%)	5.7 (2.5 to 9.6)
Chronic obstructive pulmonary disease	Italy	2005	All Ages	3.4 (1.4 to 5.9)	14.4% (6.1 to 24.7%)	5.9 (2.4 to 10.1)
Chronic obstructive pulmonary disease	Italy	2010	All Ages	3.7 (1.5 to 6.4)	14.7% (6.4 to 25.1%)	6.1 (2.5 to 10.5)
Chronic obstructive pulmonary disease	Italy	2015	All Ages	5.2 (2.5 to 8.4)	17.9% (8.3 to 28.7%)	8.3 (3.9 to 13.4)
Chronic obstructive pulmonary disease	Italy	1990	Age-standardized		17.1% (7.9 to 27.7%)	3.9 (1.8 to 6.4)
Chronic obstructive pulmonary disease	Italy	1995	Age-standardized		16.1% (7.2 to 26.8%)	3.3 (1.5 to 5.5)
Chronic obstructive pulmonary disease	Italy	2000	Age-standardized		15.2% (6.7 to 25.6%)	3.1 (1.4 to 5.2)
Chronic obstructive pulmonary disease	Italy	2005	Age-standardized		14.4% (6.1 to 24.6%)	2.8 (1.2 to 4.8)
Chronic obstructive pulmonary disease	Italy	2010	Age-standardized		14.7% (6.4 to 25.1%)	2.6 (1.1 to 4.4)
Chronic obstructive pulmonary disease	Italy	2015	Age-standardized		17.9% (8.3 to 28.7%)	3.1 (1.5 to 5.0)
All causes	Luxembourg	1990	All Ages	0.3 (0.2 to 0.3)	6.4% (5.3 to 7.6%)	65.2 (54.4 to 77.3)
All causes	Luxembourg	1995	All Ages	0.2 (0.2 to 0.3)	5.8% (4.8 to 6.9%)	53.2 (43.7 to 63.5)
All causes	Luxembourg	2000	All Ages	0.2 (0.2 to 0.2)	5.5% (4.5 to 6.5%)	44.3 (36.0 to 53.0)
All causes	Luxembourg	2005	All Ages	0.2 (0.1 to 0.2)	5.1% (4.1 to 6.2%)	38.7 (31.1 to 46.8)
All causes	Luxembourg	2010	All Ages	0.2 (0.1 to 0.2)	4.8% (3.8 to 5.9%)	33.2 (26.4 to 40.7)
All causes	Luxembourg	2015	All Ages	0.2 (0.1 to 0.2)	4.7% (3.7 to 5.8%)	31.5 (24.8 to 38.6)
All causes	Luxembourg	1990	Age-standardized		6.3% (5.2 to 7.4%)	49.4 (41.2 to 58.2)
All causes	Luxembourg	1995	Age-standardized		5.7% (4.7 to 6.8%)	39.9 (33.1 to 47.6)
All causes	Luxembourg	2000	Age-standardized		5.4% (4.4 to 6.4%)	33.8 (27.5 to 40.5)
All causes	Luxembourg	2005	Age-standardized		5.0% (4.1 to 6.1%)	27.4 (22.1 to 33.0)
All causes	Luxembourg	2010	Age-standardized		4.7% (3.8 to 5.7%)	22.4 (18.1 to 27.1)
All causes	Luxembourg	2015	Age-standardized		4.6% (3.7 to 5.6%)	20.1 (16.0 to 24.4)
Lower respiratory infections	Luxembourg	1990	All Ages	0.0 (0.0 to 0.0)	16.9% (8.4 to 27.3%)	3.8 (1.8 to 6.1)
Lower respiratory infections	Luxembourg	1995	All Ages	0.0 (0.0 to 0.0)	15.5% (6.9 to 26.1%)	3.4 (1.5 to 5.7)
Lower respiratory infections	Luxembourg	2000	All Ages	0.0 (0.0 to 0.0)	14.6% (6.2 to 25.2%)	3.5 (1.5 to 6.1)
Lower respiratory infections	Luxembourg	2005	All Ages	0.0 (0.0 to 0.0)	14.0% (5.4 to 25.1%)	3.6 (1.4 to 6.4)
Lower respiratory infections	Luxembourg	2010	All Ages	0.0 (0.0 to 0.0)	14.1% (5.8 to 24.7%)	3.4 (1.4 to 6.0)
Lower respiratory infections	Luxembourg	2015	All Ages	0.0 (0.0 to 0.0)	14.3% (5.9 to 25.0%)	3.4 (1.3 to 5.9)
Lower respiratory infections	Luxembourg	1990	Age-standardized		16.9% (8.4 to 27.3%)	2.9 (1.4 to 4.7)
Lower respiratory infections	Luxembourg	1995	Age-standardized		15.5% (6.9 to 26.1%)	2.5 (1.1 to 4.2)
Lower respiratory infections	Luxembourg	2000	Age-standardized		14.6% (6.2 to 25.2%)	2.7 (1.1 to 4.7)
Lower respiratory infections	Luxembourg	2005	Age-standardized		14.0% (5.4 to 25.1%)	2.5 (1.0 to 4.4)
Lower respiratory infections	Luxembourg	2010	Age-standardized		14.1% (5.8 to 24.7%)	2.2 (0.9 to 3.8)

Cause name	Location	Year	Age group	Number (in thousands)	PAF	Rate (per 100,000)
Lower respiratory infections	Luxembourg	2015	Age-standardized		14.3% (5.9 to 25.0%)	2.0 (0.8 to 3.4)
Tracheal, bronchus, and lung cancer	Luxembourg	1990	All Ages	0.0 (0.0 to 0.0)	11.9% (6.4 to 18.3%)	6.8 (3.7 to 10.5)
Tracheal, bronchus, and lung cancer	Luxembourg	1995	All Ages	0.0 (0.0 to 0.0)	10.8% (5.8 to 16.8%)	5.9 (3.1 to 9.1)
Tracheal, bronchus, and lung cancer	Luxembourg	2000	All Ages	0.0 (0.0 to 0.0)	10.1% (5.2 to 16.0%)	5.1 (2.6 to 8.0)
Tracheal, bronchus, and lung cancer	Luxembourg	2005	All Ages	0.0 (0.0 to 0.0)	9.7% (5.1 to 15.5%)	4.6 (2.4 to 7.4)
Tracheal, bronchus, and lung cancer	Luxembourg	2010	All Ages	0.0 (0.0 to 0.0)	9.8% (5.0 to 15.6%)	4.3 (2.2 to 7.0)
Tracheal, bronchus, and lung cancer	Luxembourg	2015	All Ages	0.0 (0.0 to 0.0)	9.9% (5.1 to 15.9%)	4.1 (2.1 to 6.6)
Tracheal, bronchus, and lung cancer	Luxembourg	1990	Age-standardized		11.9% (6.4 to 18.3%)	5.2 (2.8 to 8.1)
Tracheal, bronchus, and lung cancer	Luxembourg	1995	Age-standardized		10.8% (5.8 to 16.8%)	4.5 (2.4 to 7.0)
Tracheal, bronchus, and lung cancer	Luxembourg	2000	Age-standardized		10.1% (5.2 to 16.0%)	3.9 (2.0 to 6.2)
Tracheal, bronchus, and lung cancer	Luxembourg	2005	Age-standardized		9.7% (5.1 to 15.5%)	3.4 (1.7 to 5.4)
Tracheal, bronchus, and lung cancer	Luxembourg	2010	Age-standardized		9.8% (5.0 to 15.6%)	3.2 (1.6 to 5.1)
Tracheal, bronchus, and lung cancer	Luxembourg	2015	Age-standardized		9.9% (5.1 to 15.9%)	2.9 (1.5 to 4.7)
Cardiovascular diseases	Luxembourg	1990	All Ages	0.2 (0.1 to 0.2)	10.5% (8.6 to 12.6%)	47.4 (38.5 to 57.1)
Cardiovascular diseases	Luxembourg	1995	All Ages	0.1 (0.1 to 0.2)	9.8% (7.7 to 11.9%)	37.0 (29.1 to 45.4)
Cardiovascular diseases	Luxembourg	2000	All Ages	0.1 (0.1 to 0.2)	9.4% (7.3 to 11.6%)	29.8 (23.3 to 36.6)
Cardiovascular diseases	Luxembourg	2005	All Ages	0.1 (0.1 to 0.1)	8.9% (6.8 to 11.0%)	25.0 (19.2 to 31.1)
Cardiovascular diseases	Luxembourg	2010	All Ages	0.1 (0.1 to 0.1)	8.5% (6.4 to 10.6%)	20.1 (15.2 to 25.3)
Cardiovascular diseases	Luxembourg	2015	All Ages	0.1 (0.1 to 0.1)	8.3% (6.1 to 10.6%)	18.8 (13.8 to 24.2)
Cardiovascular diseases	Luxembourg	1990	Age-standardized		10.6% (8.7 to 12.7%)	35.9 (29.1 to 43.0)
Cardiovascular diseases	Luxembourg	1995	Age-standardized		9.9% (7.9 to 12.0%)	27.7 (22.0 to 33.9)
Cardiovascular diseases	Luxembourg	2000	Age-standardized		9.4% (7.4 to 11.6%)	22.7 (17.8 to 28.1)
Cardiovascular diseases	Luxembourg	2005	Age-standardized		9.0% (7.0 to 11.1%)	17.7 (13.6 to 21.9)
Cardiovascular diseases	Luxembourg	2010	Age-standardized		8.7% (6.8 to 10.7%)	13.5 (10.5 to 16.8)
Cardiovascular diseases	Luxembourg	2015	Age-standardized		8.7% (6.6 to 10.7%)	11.9 (9.0 to 14.9)
Ischemic heart disease	Luxembourg	1990	All Ages	0.1 (0.1 to 0.1)	14.2% (10.8 to 17.5%)	31.6 (24.2 to 39.2)
Ischemic heart disease	Luxembourg	1995	All Ages	0.1 (0.1 to 0.1)	13.5% (10.1 to 17.0%)	25.4 (19.0 to 32.1)
Ischemic heart disease	Luxembourg	2000	All Ages	0.1 (0.1 to 0.1)	13.0% (9.6 to 16.3%)	20.9 (15.3 to 26.6)
Ischemic heart disease	Luxembourg	2005	All Ages	0.1 (0.1 to 0.1)	12.6% (9.1 to 16.1%)	18.0 (12.9 to 23.0)
Ischemic heart disease	Luxembourg	2010	All Ages	0.1 (0.1 to 0.1)	12.4% (8.6 to 16.2%)	14.4 (9.9 to 18.7)
Ischemic heart disease	Luxembourg	2015	All Ages	0.1 (0.1 to 0.1)	12.3% (8.3 to 16.1%)	13.6 (9.1 to 18.1)
Ischemic heart disease	Luxembourg	1990	Age-standardized		14.3% (11.0 to 17.6%)	24.0 (18.5 to 29.7)
Ischemic heart disease	Luxembourg	1995	Age-standardized		13.7% (10.3 to 17.0%)	19.1 (14.6 to 23.9)
Ischemic heart disease	Luxembourg	2000	Age-standardized		13.0% (9.6 to 16.4%)	16.0 (11.7 to 20.3)
Ischemic heart disease	Luxembourg	2005	Age-standardized		12.8% (9.4 to 16.3%)	12.8 (9.3 to 16.2)
Ischemic heart disease	Luxembourg	2010	Age-standardized		12.7% (9.1 to 16.2%)	9.7 (6.9 to 12.4)

Cause name	Location	Year	Age group	Number (in thousands)	PAF	Rate (per 100,000)
Ischemic heart disease	Luxembourg	2015	Age-standardized		12.7% (9.2 to 16.3%)	8.7 (6.2 to 11.2)
Ischemic stroke	Luxembourg	1990	All Ages	0.0 (0.0 to 0.1)	9.7% (6.2 to 13.9%)	8.4 (5.4 to 12.1)
Ischemic stroke	Luxembourg	1995	All Ages	0.0 (0.0 to 0.0)	9.2% (5.5 to 13.6%)	5.9 (3.5 to 8.7)
Ischemic stroke	Luxembourg	2000	All Ages	0.0 (0.0 to 0.0)	8.9% (5.2 to 13.4%)	4.2 (2.4 to 6.4)
Ischemic stroke	Luxembourg	2005	All Ages	0.0 (0.0 to 0.0)	8.6% (4.8 to 13.2%)	3.2 (1.8 to 5.0)
Ischemic stroke	Luxembourg	2010	All Ages	0.0 (0.0 to 0.0)	8.6% (4.6 to 13.4%)	2.7 (1.5 to 4.2)
Ischemic stroke	Luxembourg	2015	All Ages	0.0 (0.0 to 0.0)	8.5% (4.4 to 13.6%)	2.5 (1.3 to 3.9)
Ischemic stroke	Luxembourg	1990	Age-standardized		9.8% (6.2 to 13.9%)	6.2 (4.0 to 8.9)
Ischemic stroke	Luxembourg	1995	Age-standardized		9.2% (5.7 to 13.6%)	4.3 (2.6 to 6.3)
Ischemic stroke	Luxembourg	2000	Age-standardized		8.8% (5.1 to 13.5%)	3.2 (1.8 to 4.9)
Ischemic stroke	Luxembourg	2005	Age-standardized		8.7% (4.9 to 13.2%)	2.2 (1.2 to 3.4)
Ischemic stroke	Luxembourg	2010	Age-standardized		8.7% (4.9 to 13.2%)	1.7 (1.0 to 2.6)
Ischemic stroke	Luxembourg	2015	Age-standardized		8.7% (4.9 to 13.4%)	1.5 (0.8 to 2.2)
Hemorrhagic stroke	Luxembourg	1990	All Ages	0.0 (0.0 to 0.0)	10.6% (7.5 to 13.9%)	7.4 (5.2 to 9.8)
Hemorrhagic stroke	Luxembourg	1995	All Ages	0.0 (0.0 to 0.0)	10.0% (6.9 to 13.6%)	5.7 (3.9 to 7.7)
Hemorrhagic stroke	Luxembourg	2000	All Ages	0.0 (0.0 to 0.0)	9.6% (6.4 to 13.2%)	4.7 (3.2 to 6.5)
Hemorrhagic stroke	Luxembourg	2005	All Ages	0.0 (0.0 to 0.0)	9.3% (6.1 to 13.0%)	3.8 (2.5 to 5.3)
Hemorrhagic stroke	Luxembourg	2010	All Ages	0.0 (0.0 to 0.0)	9.2% (5.9 to 13.1%)	3.1 (2.0 to 4.4)
Hemorrhagic stroke	Luxembourg	2015	All Ages	0.0 (0.0 to 0.0)	9.2% (5.7 to 13.3%)	2.7 (1.7 to 4.0)
Hemorrhagic stroke	Luxembourg	1990	Age-standardized		10.7% (7.6 to 14.0%)	5.6 (4.0 to 7.4)
Hemorrhagic stroke	Luxembourg	1995	Age-standardized		10.1% (7.0 to 13.5%)	4.3 (3.0 to 5.7)
Hemorrhagic stroke	Luxembourg	2000	Age-standardized		9.7% (6.5 to 13.3%)	3.6 (2.4 to 4.9)
Hemorrhagic stroke	Luxembourg	2005	Age-standardized		9.4% (6.3 to 13.0%)	2.7 (1.8 to 3.7)
Hemorrhagic stroke	Luxembourg	2010	Age-standardized		9.4% (6.3 to 13.0%)	2.1 (1.4 to 2.9)
Hemorrhagic stroke	Luxembourg	2015	Age-standardized		9.5% (6.2 to 13.2%)	1.7 (1.2 to 2.5)
Chronic obstructive pulmonary disease	Luxembourg	1990	All Ages	0.0 (0.0 to 0.0)	18.3% (8.8 to 29.1%)	7.3 (3.4 to 11.7)
Chronic obstructive pulmonary disease	Luxembourg	1995	All Ages	0.0 (0.0 to 0.1)	17.1% (8.0 to 27.8%)	6.9 (3.2 to 11.3)
Chronic obstructive pulmonary disease	Luxembourg	2000	All Ages	0.0 (0.0 to 0.0)	16.3% (7.6 to 26.9%)	5.9 (2.7 to 9.9)
Chronic obstructive pulmonary disease	Luxembourg	2005	All Ages	0.0 (0.0 to 0.0)	15.9% (7.1 to 26.3%)	5.5 (2.5 to 9.1)
Chronic obstructive pulmonary disease	Luxembourg	2010	All Ages	0.0 (0.0 to 0.0)	15.9% (7.0 to 26.4%)	5.3 (2.4 to 8.8)
Chronic obstructive pulmonary disease	Luxembourg	2015	All Ages	0.0 (0.0 to 0.1)	16.1% (7.1 to 26.5%)	5.3 (2.3 to 8.7)
Chronic obstructive pulmonary disease	Luxembourg	1990	Age-standardized		18.3% (8.8 to 29.1%)	5.4 (2.6 to 8.7)
Chronic obstructive pulmonary disease	Luxembourg	1995	Age-standardized		17.1% (8.0 to 27.8%)	5.1 (2.4 to 8.4)
Chronic obstructive pulmonary disease	Luxembourg	2000	Age-standardized		16.3% (7.5 to 26.9%)	4.5 (2.0 to 7.4)
Chronic obstructive pulmonary disease	Luxembourg	2005	Age-standardized		15.8% (7.1 to 26.3%)	3.8 (1.7 to 6.3)
Chronic obstructive pulmonary disease	Luxembourg	2010	Age-standardized		15.9% (7.0 to 26.3%)	3.5 (1.5 to 5.8)

Cause name	Location	Year	Age group	Number (in thousands)	PAF	Rate (per 100,000)
Chronic obstructive pulmonary disease	Luxembourg	2015	Age-standardized		16.1% (7.1 to 26.4%)	3.3 (1.4 to 5.4)
All causes	Malta	1990	All Ages	0.1 (0.1 to 0.2)	6.0% (4.8 to 7.4%)	38.8 (30.9 to 47.3)
All causes	Malta	1995	All Ages	0.1 (0.1 to 0.2)	5.6% (4.4 to 6.9%)	34.7 (27.0 to 42.7)
All causes	Malta	2000	All Ages	0.1 (0.1 to 0.2)	5.4% (4.2 to 6.7%)	34.6 (26.9 to 43.0)
All causes	Malta	2005	All Ages	0.1 (0.1 to 0.1)	4.8% (3.6 to 6.0%)	30.9 (23.4 to 38.9)
All causes	Malta	2010	All Ages	0.1 (0.1 to 0.2)	5.2% (4.1 to 6.5%)	36.6 (28.5 to 45.6)
All causes	Malta	2015	All Ages	0.2 (0.1 to 0.2)	5.3% (4.2 to 6.6%)	39.6 (30.5 to 49.4)
All causes	Malta	1990	Age-standardized		6.0% (4.8 to 7.4%)	40.3 (31.8 to 49.2)
All causes	Malta	1995	Age-standardized		5.5% (4.3 to 6.8%)	33.5 (25.9 to 41.3)
All causes	Malta	2000	Age-standardized		5.3% (4.1 to 6.7%)	30.7 (23.6 to 38.4)
All causes	Malta	2005	Age-standardized		4.7% (3.5 to 5.9%)	23.0 (17.4 to 29.0)
All causes	Malta	2010	Age-standardized		5.1% (4.0 to 6.3%)	23.5 (18.5 to 29.1)
All causes	Malta	2015	Age-standardized		5.2% (4.0 to 6.4%)	23.5 (18.0 to 29.2)
Lower respiratory infections	Malta	1990	All Ages	0.0 (0.0 to 0.0)	12.5% (4.3 to 23.8%)	2.4 (0.8 to 4.5)
Lower respiratory infections	Malta	1995	All Ages	0.0 (0.0 to 0.0)	12.1% (3.7 to 23.4%)	2.8 (0.9 to 5.5)
Lower respiratory infections	Malta	2000	All Ages	0.0 (0.0 to 0.0)	11.6% (3.6 to 23.1%)	3.3 (1.0 to 6.7)
Lower respiratory infections	Malta	2005	All Ages	0.0 (0.0 to 0.0)	10.3% (2.7 to 21.1%)	2.9 (0.8 to 6.0)
Lower respiratory infections	Malta	2010	All Ages	0.0 (0.0 to 0.0)	12.7% (4.4 to 23.8%)	3.7 (1.2 to 7.0)
Lower respiratory infections	Malta	2015	All Ages	0.0 (0.0 to 0.0)	13.6% (4.8 to 24.7%)	4.1 (1.4 to 7.3)
Lower respiratory infections	Malta	1990	Age-standardized		12.5% (4.3 to 23.8%)	2.6 (0.8 to 4.9)
Lower respiratory infections	Malta	1995	Age-standardized		12.1% (3.7 to 23.4%)	2.9 (0.9 to 5.5)
Lower respiratory infections	Malta	2000	Age-standardized		11.6% (3.6 to 23.1%)	3.1 (0.9 to 6.2)
Lower respiratory infections	Malta	2005	Age-standardized		10.3% (2.7 to 21.1%)	2.2 (0.6 to 4.6)
Lower respiratory infections	Malta	2010	Age-standardized		12.7% (4.4 to 23.8%)	2.4 (0.8 to 4.5)
Lower respiratory infections	Malta	2015	Age-standardized		13.6% (4.8 to 24.7%)	2.5 (0.9 to 4.5)
Tracheal, bronchus, and lung cancer	Malta	1990	All Ages	0.0 (0.0 to 0.0)	8.7% (4.2 to 14.5%)	2.3 (1.1 to 3.8)
Tracheal, bronchus, and lung cancer	Malta	1995	All Ages	0.0 (0.0 to 0.0)	8.3% (3.9 to 13.7%)	2.4 (1.1 to 4.0)
Tracheal, bronchus, and lung cancer	Malta	2000	All Ages	0.0 (0.0 to 0.0)	8.0% (3.5 to 13.4%)	2.5 (1.1 to 4.1)
Tracheal, bronchus, and lung cancer	Malta	2005	All Ages	0.0 (0.0 to 0.0)	7.0% (3.2 to 12.1%)	2.1 (1.0 to 3.6)
Tracheal, bronchus, and lung cancer	Malta	2010	All Ages	0.0 (0.0 to 0.0)	8.8% (4.3 to 14.7%)	3.2 (1.6 to 5.3)
Tracheal, bronchus, and lung cancer	Malta	2015	All Ages	0.0 (0.0 to 0.0)	9.4% (4.7 to 15.5%)	3.6 (1.8 to 5.9)
Tracheal, bronchus, and lung cancer	Malta	1990	Age-standardized		8.7% (4.2 to 14.4%)	2.3 (1.2 to 3.8)
Tracheal, bronchus, and lung cancer	Malta	1995	Age-standardized		8.3% (3.9 to 13.7%)	2.3 (1.0 to 3.8)
Tracheal, bronchus, and lung cancer	Malta	2000	Age-standardized		8.0% (3.5 to 13.4%)	2.1 (0.9 to 3.5)
Tracheal, bronchus, and lung cancer	Malta	2005	Age-standardized		7.0% (3.2 to 12.1%)	1.6 (0.7 to 2.7)
Tracheal, bronchus, and lung cancer	Malta	2010	Age-standardized		8.8% (4.3 to 14.7%)	2.1 (1.0 to 3.4)

Cause name	Location	Year	Age group	Number (in thousands)	PAF	Rate (per 100,000)
Tracheal, bronchus, and lung cancer	Malta	2015	Age-standardized		9.4% (4.7 to 15.4%)	2.1 (1.0 to 3.4)
Cardiovascular diseases	Malta	1990	All Ages	0.1 (0.1 to 0.1)	10.2% (8.1 to 12.4%)	31.0 (24.7 to 38.1)
Cardiovascular diseases	Malta	1995	All Ages	0.1 (0.1 to 0.1)	9.8% (7.7 to 12.1%)	26.2 (20.4 to 32.4)
Cardiovascular diseases	Malta	2000	All Ages	0.1 (0.1 to 0.1)	9.5% (7.4 to 12.0%)	25.5 (19.5 to 32.3)
Cardiovascular diseases	Malta	2005	All Ages	0.1 (0.1 to 0.1)	8.8% (6.6 to 11.2%)	22.9 (17.1 to 29.1)
Cardiovascular diseases	Malta	2010	All Ages	0.1 (0.1 to 0.1)	9.5% (7.1 to 12.1%)	26.1 (19.5 to 33.0)
Cardiovascular diseases	Malta	2015	All Ages	0.1 (0.1 to 0.1)	9.7% (7.3 to 12.2%)	28.0 (21.0 to 35.5)
Cardiovascular diseases	Malta	1990	Age-standardized		10.0% (7.9 to 12.3%)	32.0 (25.3 to 39.5)
Cardiovascular diseases	Malta	1995	Age-standardized		9.7% (7.5 to 12.0%)	25.2 (19.4 to 31.3)
Cardiovascular diseases	Malta	2000	Age-standardized		9.4% (7.2 to 12.0%)	22.6 (17.2 to 28.9)
Cardiovascular diseases	Malta	2005	Age-standardized		8.8% (6.6 to 11.2%)	17.0 (12.7 to 21.7)
Cardiovascular diseases	Malta	2010	Age-standardized		9.6% (7.3 to 12.1%)	16.8 (12.7 to 21.1)
Cardiovascular diseases	Malta	2015	Age-standardized		9.7% (7.3 to 12.3%)	16.6 (12.4 to 21.1)
Ischemic heart disease	Malta	1990	All Ages	0.1 (0.1 to 0.1)	12.8% (9.7 to 16.0%)	24.5 (18.6 to 30.9)
Ischemic heart disease	Malta	1995	All Ages	0.1 (0.1 to 0.1)	12.4% (9.3 to 15.7%)	20.6 (15.4 to 26.1)
Ischemic heart disease	Malta	2000	All Ages	0.1 (0.1 to 0.1)	12.1% (8.9 to 15.4%)	20.2 (14.8 to 25.7)
Ischemic heart disease	Malta	2005	All Ages	0.1 (0.1 to 0.1)	11.3% (8.2 to 14.8%)	18.0 (13.0 to 23.5)
Ischemic heart disease	Malta	2010	All Ages	0.1 (0.1 to 0.1)	12.2% (8.7 to 15.7%)	20.6 (14.7 to 26.5)
Ischemic heart disease	Malta	2015	All Ages	0.1 (0.1 to 0.1)	12.3% (8.7 to 16.0%)	22.4 (15.9 to 29.2)
Ischemic heart disease	Malta	1990	Age-standardized		12.6% (9.4 to 15.9%)	25.3 (18.9 to 32.0)
Ischemic heart disease	Malta	1995	Age-standardized		12.3% (9.1 to 15.6%)	19.8 (14.5 to 25.2)
Ischemic heart disease	Malta	2000	Age-standardized		12.0% (8.7 to 15.4%)	17.8 (12.9 to 23.0)
Ischemic heart disease	Malta	2005	Age-standardized		11.3% (8.2 to 14.8%)	13.3 (9.6 to 17.5)
Ischemic heart disease	Malta	2010	Age-standardized		12.3% (9.0 to 15.8%)	13.3 (9.6 to 17.0)
Ischemic heart disease	Malta	2015	Age-standardized		12.4% (8.7 to 16.2%)	13.3 (9.3 to 17.4)
Ischemic stroke	Malta	1990	All Ages	0.0 (0.0 to 0.0)	8.5% (5.1 to 12.3%)	3.3 (1.9 to 4.8)
Ischemic stroke	Malta	1995	All Ages	0.0 (0.0 to 0.0)	8.2% (4.8 to 12.3%)	2.8 (1.7 to 4.3)
Ischemic stroke	Malta	2000	All Ages	0.0 (0.0 to 0.0)	8.0% (4.4 to 12.1%)	2.7 (1.5 to 4.1)
Ischemic stroke	Malta	2005	All Ages	0.0 (0.0 to 0.0)	7.4% (3.8 to 11.8%)	2.5 (1.3 to 4.0)
Ischemic stroke	Malta	2010	All Ages	0.0 (0.0 to 0.0)	8.1% (4.3 to 13.0%)	2.8 (1.5 to 4.4)
Ischemic stroke	Malta	2015	All Ages	0.0 (0.0 to 0.0)	8.4% (4.6 to 13.1%)	2.8 (1.5 to 4.4)
Ischemic stroke	Malta	1990	Age-standardized		8.4% (4.9 to 12.4%)	3.4 (2.0 to 5.1)
Ischemic stroke	Malta	1995	Age-standardized		8.2% (4.7 to 12.4%)	2.8 (1.6 to 4.2)
Ischemic stroke	Malta	2000	Age-standardized		7.9% (4.3 to 12.2%)	2.4 (1.3 to 3.8)
Ischemic stroke	Malta	2005	Age-standardized		7.4% (3.8 to 11.9%)	1.8 (1.0 to 3.0)
Ischemic stroke	Malta	2010	Age-standardized		8.2% (4.4 to 12.9%)	1.8 (1.0 to 2.8)

Cause name	Location	Year	Age group	Number (in thousands)	PAF	Rate (per 100,000)
Ischemic stroke	Malta	2015	Age-standardized		8.4% (4.4 to 13.3%)	1.6 (0.9 to 2.6)
Hemorrhagic stroke	Malta	1990	All Ages	0.0 (0.0 to 0.0)	9.5% (6.4 to 12.6%)	3.3 (2.2 to 4.4)
Hemorrhagic stroke	Malta	1995	All Ages	0.0 (0.0 to 0.0)	9.1% (6.2 to 12.3%)	2.8 (1.9 to 3.8)
Hemorrhagic stroke	Malta	2000	All Ages	0.0 (0.0 to 0.0)	8.8% (5.8 to 12.0%)	2.6 (1.8 to 3.6)
Hemorrhagic stroke	Malta	2005	All Ages	0.0 (0.0 to 0.0)	8.1% (5.0 to 11.5%)	2.4 (1.5 to 3.5)
Hemorrhagic stroke	Malta	2010	All Ages	0.0 (0.0 to 0.0)	8.9% (5.6 to 12.7%)	2.7 (1.7 to 3.9)
Hemorrhagic stroke	Malta	2015	All Ages	0.0 (0.0 to 0.0)	9.0% (5.7 to 12.9%)	2.8 (1.8 to 4.1)
Hemorrhagic stroke	Malta	1990	Age-standardized		9.3% (6.3 to 12.5%)	3.3 (2.2 to 4.5)
Hemorrhagic stroke	Malta	1995	Age-standardized		9.0% (6.0 to 12.3%)	2.7 (1.8 to 3.7)
Hemorrhagic stroke	Malta	2000	Age-standardized		8.7% (5.7 to 12.1%)	2.3 (1.5 to 3.3)
Hemorrhagic stroke	Malta	2005	Age-standardized		8.1% (5.0 to 11.6%)	1.8 (1.1 to 2.6)
Hemorrhagic stroke	Malta	2010	Age-standardized		9.0% (5.8 to 12.6%)	1.8 (1.2 to 2.5)
Hemorrhagic stroke	Malta	2015	Age-standardized		9.1% (5.7 to 13.1%)	1.7 (1.1 to 2.4)
Chronic obstructive pulmonary disease	Malta	1990	All Ages	0.0 (0.0 to 0.0)	14.6% (5.7 to 24.9%)	3.2 (1.2 to 5.4)
Chronic obstructive pulmonary disease	Malta	1995	All Ages	0.0 (0.0 to 0.0)	14.1% (5.5 to 24.8%)	3.2 (1.3 to 5.7)
Chronic obstructive pulmonary disease	Malta	2000	All Ages	0.0 (0.0 to 0.0)	13.7% (5.3 to 24.1%)	3.3 (1.3 to 5.7)
Chronic obstructive pulmonary disease	Malta	2005	All Ages	0.0 (0.0 to 0.0)	12.6% (4.7 to 22.5%)	2.9 (1.1 to 5.4)
Chronic obstructive pulmonary disease	Malta	2010	All Ages	0.0 (0.0 to 0.0)	14.7% (6.0 to 25.4%)	3.5 (1.4 to 6.3)
Chronic obstructive pulmonary disease	Malta	2015	All Ages	0.0 (0.0 to 0.0)	15.5% (6.4 to 25.9%)	3.9 (1.7 to 6.7)
Chronic obstructive pulmonary disease	Malta	1990	Age-standardized		14.6% (5.7 to 24.9%)	3.4 (1.3 to 5.7)
Chronic obstructive pulmonary disease	Malta	1995	Age-standardized		14.1% (5.5 to 24.8%)	3.2 (1.2 to 5.6)
Chronic obstructive pulmonary disease	Malta	2000	Age-standardized		13.7% (5.3 to 24.1%)	2.9 (1.2 to 5.1)
Chronic obstructive pulmonary disease	Malta	2005	Age-standardized		12.5% (4.7 to 22.5%)	2.2 (0.8 to 4.0)
Chronic obstructive pulmonary disease	Malta	2010	Age-standardized		14.7% (6.0 to 25.3%)	2.2 (0.9 to 4.0)
Chronic obstructive pulmonary disease	Malta	2015	Age-standardized		15.4% (6.3 to 25.8%)	2.3 (1.0 to 3.9)
All causes	Netherlands	1990	All Ages	8.2 (6.8 to 9.6)	6.3% (5.3 to 7.5%)	54.6 (45.4 to 64.3)
All causes	Netherlands	1995	All Ages	8.1 (6.7 to 9.6)	6.0% (4.9 to 7.1%)	52.3 (43.1 to 62.4)
All causes	Netherlands	2000	All Ages	7.8 (6.4 to 9.5)	5.5% (4.5 to 6.7%)	49.2 (39.9 to 59.7)
All causes	Netherlands	2005	All Ages	6.9 (5.5 to 8.5)	5.1% (4.1 to 6.2%)	42.3 (33.9 to 52.1)
All causes	Netherlands	2010	All Ages	6.5 (5.2 to 8.1)	4.8% (3.8 to 6.0%)	39.3 (31.1 to 48.9)
All causes	Netherlands	2015	All Ages	6.8 (5.3 to 8.6)	4.7% (3.6 to 5.9%)	39.5 (30.7 to 50.0)
All causes	Netherlands	1990	Age-standardized		6.3% (5.3 to 7.4%)	44.0 (36.7 to 51.6)
All causes	Netherlands	1995	Age-standardized		5.9% (4.9 to 7.0%)	40.1 (33.3 to 47.6)
All causes	Netherlands	2000	Age-standardized		5.5% (4.5 to 6.6%)	36.0 (29.4 to 43.6)
All causes	Netherlands	2005	Age-standardized		5.0% (4.0 to 6.1%)	28.8 (23.3 to 35.2)
All causes	Netherlands	2010	Age-standardized		4.7% (3.7 to 5.8%)	24.4 (19.5 to 30.4)

Cause name	Location	Year	Age group	Number (in thousands)	PAF	Rate (per 100,000)
All causes	Netherlands	2015	Age-standardized		4.6% (3.6 to 5.7%)	22.8 (17.8 to 28.5)
Lower respiratory infections	Netherlands	1990	All Ages	0.7 (0.3 to 1.1)	15.8% (7.2 to 26.2%)	4.5 (2.0 to 7.6)
Lower respiratory infections	Netherlands	1995	All Ages	0.8 (0.4 to 1.5)	14.6% (6.1 to 25.2%)	5.4 (2.3 to 9.6)
Lower respiratory infections	Netherlands	2000	All Ages	1.0 (0.4 to 1.8)	13.7% (5.4 to 24.3%)	6.3 (2.5 to 11.4)
Lower respiratory infections	Netherlands	2005	All Ages	1.0 (0.4 to 1.8)	13.2% (5.0 to 24.2%)	6.0 (2.3 to 11.1)
Lower respiratory infections	Netherlands	2010	All Ages	1.0 (0.4 to 1.8)	13.3% (5.1 to 24.1%)	5.9 (2.2 to 10.9)
Lower respiratory infections	Netherlands	2015	All Ages	1.0 (0.4 to 1.9)	12.8% (4.6 to 23.7%)	5.8 (2.0 to 11.1)
Lower respiratory infections	Netherlands	1990	Age-standardized		15.8% (7.2 to 26.2%)	3.5 (1.6 to 5.9)
Lower respiratory infections	Netherlands	1995	Age-standardized		14.6% (6.1 to 25.2%)	4.0 (1.7 to 7.1)
Lower respiratory infections	Netherlands	2000	Age-standardized		13.7% (5.4 to 24.3%)	4.4 (1.8 to 8.1)
Lower respiratory infections	Netherlands	2005	Age-standardized		13.2% (5.0 to 24.2%)	3.9 (1.5 to 7.2)
Lower respiratory infections	Netherlands	2010	Age-standardized		13.3% (5.1 to 24.1%)	3.5 (1.3 to 6.5)
Lower respiratory infections	Netherlands	2015	Age-standardized		12.8% (4.6 to 23.7%)	3.2 (1.1 to 6.1)
Tracheal, bronchus, and lung cancer	Netherlands	1990	All Ages	1.0 (0.5 to 1.6)	11.0% (5.8 to 17.0%)	6.6 (3.5 to 10.4)
Tracheal, bronchus, and lung cancer	Netherlands	1995	All Ages	1.0 (0.5 to 1.5)	10.1% (5.3 to 16.1%)	6.2 (3.2 to 9.9)
Tracheal, bronchus, and lung cancer	Netherlands	2000	All Ages	1.0 (0.5 to 1.5)	9.5% (4.8 to 15.3%)	6.0 (3.1 to 9.5)
Tracheal, bronchus, and lung cancer	Netherlands	2005	All Ages	1.0 (0.5 to 1.5)	9.2% (4.6 to 14.8%)	5.8 (3.0 to 9.4)
Tracheal, bronchus, and lung cancer	Netherlands	2010	All Ages	1.0 (0.5 to 1.6)	9.2% (4.7 to 14.8%)	6.1 (3.1 to 9.8)
Tracheal, bronchus, and lung cancer	Netherlands	2015	All Ages	1.0 (0.5 to 1.7)	8.8% (4.4 to 14.4%)	5.9 (2.9 to 9.7)
Tracheal, bronchus, and lung cancer	Netherlands	1990	Age-standardized		11.0% (5.8 to 17.0%)	5.5 (2.9 to 8.6)
Tracheal, bronchus, and lung cancer	Netherlands	1995	Age-standardized		10.1% (5.3 to 16.1%)	5.0 (2.6 to 7.9)
Tracheal, bronchus, and lung cancer	Netherlands	2000	Age-standardized		9.5% (4.8 to 15.3%)	4.6 (2.3 to 7.3)
Tracheal, bronchus, and lung cancer	Netherlands	2005	Age-standardized		9.2% (4.6 to 14.8%)	4.2 (2.1 to 6.7)
Tracheal, bronchus, and lung cancer	Netherlands	2010	Age-standardized		9.2% (4.7 to 14.8%)	4.0 (2.0 to 6.5)
Tracheal, bronchus, and lung cancer	Netherlands	2015	Age-standardized		8.8% (4.4 to 14.4%)	3.6 (1.8 to 5.9)
Cardiovascular diseases	Netherlands	1990	All Ages	5.4 (4.3 to 6.5)	10.5% (8.5 to 12.6%)	36.1 (29.0 to 43.5)
Cardiovascular diseases	Netherlands	1995	All Ages	5.1 (4.0 to 6.1)	9.8% (7.8 to 11.8%)	32.8 (25.9 to 39.7)
Cardiovascular diseases	Netherlands	2000	All Ages	4.6 (3.6 to 5.6)	9.1% (7.2 to 11.1%)	28.9 (22.6 to 35.3)
Cardiovascular diseases	Netherlands	2005	All Ages	3.7 (2.8 to 4.7)	8.5% (6.5 to 10.5%)	22.9 (17.4 to 28.6)
Cardiovascular diseases	Netherlands	2010	All Ages	3.3 (2.5 to 4.1)	8.1% (6.1 to 10.1%)	19.7 (14.9 to 24.9)
Cardiovascular diseases	Netherlands	2015	All Ages	3.4 (2.5 to 4.3)	7.8% (5.8 to 9.8%)	19.6 (14.6 to 25.0)
Cardiovascular diseases	Netherlands	1990	Age-standardized		10.7% (8.7 to 12.8%)	29.2 (23.7 to 35.0)
Cardiovascular diseases	Netherlands	1995	Age-standardized		10.0% (8.0 to 12.0%)	25.3 (20.3 to 30.5)
Cardiovascular diseases	Netherlands	2000	Age-standardized		9.3% (7.4 to 11.2%)	21.2 (16.8 to 25.8)
Cardiovascular diseases	Netherlands	2005	Age-standardized		8.7% (6.8 to 10.6%)	15.6 (12.2 to 19.3)
Cardiovascular diseases	Netherlands	2010	Age-standardized		8.3% (6.5 to 10.2%)	12.3 (9.5 to 15.2)

Cause name	Location	Year	Age group	Number (in thousands)	PAF	Rate (per 100,000)
Cardiovascular diseases	Netherlands	2015	Age-standardized		8.0% (6.1 to 9.9%)	11.4 (8.7 to 14.2)
Ischemic heart disease	Netherlands	1990	All Ages	4.2 (3.2 to 5.1)	13.9% (10.7 to 17.1%)	27.8 (21.4 to 34.3)
Ischemic heart disease	Netherlands	1995	All Ages	3.8 (2.9 to 4.8)	13.3% (10.0 to 16.4%)	24.7 (18.6 to 31.0)
Ischemic heart disease	Netherlands	2000	All Ages	3.4 (2.5 to 4.2)	12.8% (9.5 to 16.1%)	21.1 (15.7 to 26.7)
Ischemic heart disease	Netherlands	2005	All Ages	2.7 (1.9 to 3.5)	12.4% (9.0 to 15.7%)	16.4 (11.8 to 21.2)
Ischemic heart disease	Netherlands	2010	All Ages	2.3 (1.7 to 3.1)	12.3% (8.6 to 15.8%)	14.1 (9.9 to 18.3)
Ischemic heart disease	Netherlands	2015	All Ages	2.4 (1.7 to 3.2)	11.9% (8.3 to 15.6%)	14.1 (9.8 to 18.5)
Ischemic heart disease	Netherlands	1990	Age-standardized		14.1% (11.0 to 17.2%)	22.6 (17.6 to 27.7)
Ischemic heart disease	Netherlands	1995	Age-standardized		13.5% (10.4 to 16.6%)	19.2 (14.8 to 23.8)
Ischemic heart disease	Netherlands	2000	Age-standardized		13.0% (9.8 to 16.2%)	15.6 (11.8 to 19.6)
Ischemic heart disease	Netherlands	2005	Age-standardized		12.7% (9.4 to 15.9%)	11.3 (8.4 to 14.4)
Ischemic heart disease	Netherlands	2010	Age-standardized		12.6% (9.1 to 15.9%)	8.8 (6.4 to 11.3)
Ischemic heart disease	Netherlands	2015	Age-standardized		12.3% (8.8 to 15.6%)	8.3 (5.9 to 10.7)
Ischemic stroke	Netherlands	1990	All Ages	0.7 (0.4 to 1.0)	9.3% (5.7 to 13.6%)	4.3 (2.6 to 6.3)
Ischemic stroke	Netherlands	1995	All Ages	0.6 (0.4 to 1.0)	8.9% (5.3 to 13.3%)	4.1 (2.5 to 6.3)
Ischemic stroke	Netherlands	2000	All Ages	0.6 (0.4 to 1.0)	8.6% (5.0 to 13.0%)	4.0 (2.3 to 6.1)
Ischemic stroke	Netherlands	2005	All Ages	0.5 (0.3 to 0.8)	8.4% (4.7 to 13.0%)	3.2 (1.8 to 5.1)
Ischemic stroke	Netherlands	2010	All Ages	0.5 (0.3 to 0.7)	8.4% (4.5 to 13.1%)	2.8 (1.5 to 4.4)
Ischemic stroke	Netherlands	2015	All Ages	0.5 (0.3 to 0.8)	8.1% (4.3 to 13.0%)	2.7 (1.4 to 4.5)
Ischemic stroke	Netherlands	1990	Age-standardized		9.4% (5.8 to 13.6%)	3.4 (2.1 to 4.9)
Ischemic stroke	Netherlands	1995	Age-standardized		9.0% (5.5 to 13.3%)	3.1 (1.8 to 4.6)
Ischemic stroke	Netherlands	2000	Age-standardized		8.7% (5.1 to 13.0%)	2.8 (1.7 to 4.3)
Ischemic stroke	Netherlands	2005	Age-standardized		8.5% (4.9 to 12.9%)	2.1 (1.2 to 3.2)
Ischemic stroke	Netherlands	2010	Age-standardized		8.5% (4.9 to 13.0%)	1.7 (1.0 to 2.6)
Ischemic stroke	Netherlands	2015	Age-standardized		8.3% (4.6 to 12.9%)	1.5 (0.8 to 2.4)
Hemorrhagic stroke	Netherlands	1990	All Ages	0.6 (0.4 to 0.8)	10.2% (7.1 to 13.5%)	4.0 (2.8 to 5.4)
Hemorrhagic stroke	Netherlands	1995	All Ages	0.6 (0.4 to 0.8)	9.8% (6.6 to 13.1%)	4.0 (2.7 to 5.4)
Hemorrhagic stroke	Netherlands	2000	All Ages	0.6 (0.4 to 0.8)	9.4% (6.3 to 12.9%)	3.8 (2.5 to 5.3)
Hemorrhagic stroke	Netherlands	2005	All Ages	0.5 (0.3 to 0.7)	9.1% (6.0 to 12.8%)	3.2 (2.1 to 4.5)
Hemorrhagic stroke	Netherlands	2010	All Ages	0.5 (0.3 to 0.7)	9.0% (5.9 to 13.0%)	2.8 (1.8 to 4.0)
Hemorrhagic stroke	Netherlands	2015	All Ages	0.5 (0.3 to 0.7)	8.8% (5.4 to 12.8%)	2.7 (1.7 to 4.0)
Hemorrhagic stroke	Netherlands	1990	Age-standardized		10.4% (7.4 to 13.6%)	3.3 (2.3 to 4.3)
Hemorrhagic stroke	Netherlands	1995	Age-standardized		9.9% (6.8 to 13.2%)	3.1 (2.1 to 4.1)
Hemorrhagic stroke	Netherlands	2000	Age-standardized		9.5% (6.5 to 12.9%)	2.8 (1.9 to 3.8)
Hemorrhagic stroke	Netherlands	2005	Age-standardized		9.3% (6.3 to 12.7%)	2.2 (1.5 to 3.0)
Hemorrhagic stroke	Netherlands	2010	Age-standardized		9.3% (6.2 to 12.8%)	1.8 (1.2 to 2.5)

Cause name	Location	Year	Age group	Number (in thousands)	PAF	Rate (per 100,000)
Hemorrhagic stroke	Netherlands	2015	Age-standardized		9.0% (5.9 to 12.7%)	1.6 (1.0 to 2.3)
Chronic obstructive pulmonary disease	Netherlands	1990	All Ages	1.1 (0.5 to 1.8)	17.4% (7.8 to 28.2%)	7.4 (3.4 to 12.1)
Chronic obstructive pulmonary disease	Netherlands	1995	All Ages	1.2 (0.5 to 2.0)	16.3% (7.3 to 27.1%)	7.8 (3.5 to 12.8)
Chronic obstructive pulmonary disease	Netherlands	2000	All Ages	1.3 (0.6 to 2.1)	15.6% (6.8 to 26.0%)	8.1 (3.5 to 13.4)
Chronic obstructive pulmonary disease	Netherlands	2005	All Ages	1.2 (0.5 to 2.1)	15.2% (6.6 to 25.6%)	7.6 (3.3 to 12.9)
Chronic obstructive pulmonary disease	Netherlands	2010	All Ages	1.3 (0.6 to 2.2)	15.3% (6.6 to 25.7%)	7.7 (3.4 to 13.1)
Chronic obstructive pulmonary disease	Netherlands	2015	All Ages	1.4 (0.6 to 2.4)	14.8% (6.2 to 25.1%)	8.1 (3.4 to 13.9)
Chronic obstructive pulmonary disease	Netherlands	1990	Age-standardized		17.4% (7.8 to 28.1%)	5.8 (2.6 to 9.5)
Chronic obstructive pulmonary disease	Netherlands	1995	Age-standardized		16.3% (7.3 to 27.1%)	5.8 (2.6 to 9.6)
Chronic obstructive pulmonary disease	Netherlands	2000	Age-standardized		15.5% (6.7 to 26.0%)	5.8 (2.5 to 9.6)
Chronic obstructive pulmonary disease	Netherlands	2005	Age-standardized		15.2% (6.5 to 25.6%)	5.1 (2.2 to 8.6)
Chronic obstructive pulmonary disease	Netherlands	2010	Age-standardized		15.2% (6.6 to 25.7%)	4.7 (2.0 to 8.0)
Chronic obstructive pulmonary disease	Netherlands	2015	Age-standardized		14.8% (6.2 to 25.1%)	4.6 (1.9 to 7.8)
All causes	Norway	1990	All Ages	2.3 (1.8 to 2.8)	5.0% (3.9 to 6.2%)	53.5 (41.3 to 66.4)
All causes	Norway	1995	All Ages	2.0 (1.6 to 2.6)	4.6% (3.5 to 5.9%)	47.2 (36.0 to 60.4)
All causes	Norway	2000	All Ages	1.8 (1.4 to 2.4)	4.1% (3.1 to 5.4%)	41.1 (30.9 to 53.8)
All causes	Norway	2005	All Ages	1.5 (1.1 to 2.0)	3.5% (2.6 to 4.7%)	31.9 (23.0 to 42.5)
All causes	Norway	2010	All Ages	1.4 (1.0 to 1.9)	3.4% (2.4 to 4.6%)	29.0 (20.9 to 38.9)
All causes	Norway	2015	All Ages	1.4 (1.0 to 1.9)	3.4% (2.4 to 4.6%)	27.1 (19.4 to 36.4)
All causes	Norway	1990	Age-standardized		4.9% (3.9 to 6.0%)	35.3 (27.7 to 43.2)
All causes	Norway	1995	Age-standardized		4.5% (3.5 to 5.6%)	30.0 (23.4 to 37.4)
All causes	Norway	2000	Age-standardized		4.0% (3.1 to 5.1%)	25.3 (19.5 to 32.0)
All causes	Norway	2005	Age-standardized		3.5% (2.6 to 4.5%)	18.9 (14.2 to 24.5)
All causes	Norway	2010	Age-standardized		3.3% (2.4 to 4.3%)	17.1 (12.7 to 22.5)
All causes	Norway	2015	Age-standardized		3.3% (2.4 to 4.3%)	16.0 (11.8 to 21.0)
Lower respiratory infections	Norway	1990	All Ages	0.3 (0.1 to 0.6)	9.1% (2.3 to 20.0%)	6.4 (1.6 to 14.2)
Lower respiratory infections	Norway	1995	All Ages	0.2 (0.1 to 0.6)	8.5% (2.0 to 19.5%)	5.6 (1.3 to 12.9)
Lower respiratory infections	Norway	2000	All Ages	0.2 (0.0 to 0.5)	8.1% (1.8 to 19.0%)	4.6 (1.0 to 10.7)
Lower respiratory infections	Norway	2005	All Ages	0.2 (0.0 to 0.4)	7.3% (1.4 to 18.1%)	3.6 (0.7 to 8.9)
Lower respiratory infections	Norway	2010	All Ages	0.2 (0.0 to 0.4)	7.4% (1.5 to 18.3%)	3.4 (0.7 to 8.1)
Lower respiratory infections	Norway	2015	All Ages	0.2 (0.0 to 0.4)	7.6% (1.5 to 18.4%)	3.2 (0.7 to 7.8)
Lower respiratory infections	Norway	1990	Age-standardized		9.1% (2.3 to 20.0%)	3.9 (1.0 to 8.6)
Lower respiratory infections	Norway	1995	Age-standardized		8.5% (2.0 to 19.5%)	3.2 (0.7 to 7.3)
Lower respiratory infections	Norway	2000	Age-standardized		8.1% (1.8 to 19.0%)	2.5 (0.5 to 5.8)
Lower respiratory infections	Norway	2005	Age-standardized		7.3% (1.4 to 18.1%)	1.9 (0.4 to 4.6)
Lower respiratory infections	Norway	2010	Age-standardized		7.4% (1.5 to 18.3%)	1.8 (0.4 to 4.4)

Cause name	Location	Year	Age group	Number (in thousands)	PAF	Rate (per 100,000)
Lower respiratory infections	Norway	2015	Age-standardized		7.6% (1.5 to 18.4%)	1.7 (0.4 to 4.2)
Tracheal, bronchus, and lung cancer	Norway	1990	All Ages	0.1 (0.0 to 0.2)	6.2% (2.8 to 10.7%)	2.2 (1.0 to 3.8)
Tracheal, bronchus, and lung cancer	Norway	1995	All Ages	0.1 (0.0 to 0.2)	5.8% (2.5 to 10.0%)	2.3 (1.0 to 3.9)
Tracheal, bronchus, and lung cancer	Norway	2000	All Ages	0.1 (0.1 to 0.2)	5.5% (2.3 to 9.7%)	2.4 (1.0 to 4.2)
Tracheal, bronchus, and lung cancer	Norway	2005	All Ages	0.1 (0.0 to 0.2)	4.9% (2.0 to 8.8%)	2.2 (0.9 to 3.9)
Tracheal, bronchus, and lung cancer	Norway	2010	All Ages	0.1 (0.1 to 0.2)	5.0% (2.0 to 9.0%)	2.3 (0.9 to 4.1)
Tracheal, bronchus, and lung cancer	Norway	2015	All Ages	0.1 (0.1 to 0.2)	5.1% (2.1 to 9.2%)	2.2 (0.9 to 4.0)
Tracheal, bronchus, and lung cancer	Norway	1990	Age-standardized		6.2% (2.8 to 10.7%)	1.6 (0.7 to 2.8)
Tracheal, bronchus, and lung cancer	Norway	1995	Age-standardized		5.8% (2.5 to 10.0%)	1.6 (0.7 to 2.9)
Tracheal, bronchus, and lung cancer	Norway	2000	Age-standardized		5.5% (2.3 to 9.7%)	1.7 (0.7 to 3.0)
Tracheal, bronchus, and lung cancer	Norway	2005	Age-standardized		4.9% (2.0 to 8.8%)	1.5 (0.6 to 2.8)
Tracheal, bronchus, and lung cancer	Norway	2010	Age-standardized		5.0% (2.0 to 9.0%)	1.6 (0.6 to 2.8)
Tracheal, bronchus, and lung cancer	Norway	2015	Age-standardized		5.1% (2.1 to 9.2%)	1.4 (0.6 to 2.6)
Cardiovascular diseases	Norway	1990	All Ages	1.8 (1.3 to 2.2)	8.5% (6.5 to 10.6%)	41.8 (31.7 to 52.4)
Cardiovascular diseases	Norway	1995	All Ages	1.6 (1.1 to 2.0)	7.9% (5.8 to 10.1%)	35.8 (26.3 to 45.7)
Cardiovascular diseases	Norway	2000	All Ages	1.3 (0.9 to 1.8)	7.3% (5.1 to 9.5%)	29.8 (21.0 to 39.0)
Cardiovascular diseases	Norway	2005	All Ages	1.0 (0.7 to 1.4)	6.6% (4.5 to 9.0%)	21.8 (14.8 to 29.7)
Cardiovascular diseases	Norway	2010	All Ages	0.9 (0.6 to 1.3)	6.5% (4.4 to 9.0%)	18.7 (12.6 to 26.2)
Cardiovascular diseases	Norway	2015	All Ages	0.9 (0.6 to 1.2)	6.5% (4.4 to 9.0%)	17.2 (11.5 to 24.0)
Cardiovascular diseases	Norway	1990	Age-standardized		8.8% (6.9 to 10.8%)	27.8 (21.7 to 34.1)
Cardiovascular diseases	Norway	1995	Age-standardized		8.2% (6.3 to 10.3%)	22.9 (17.5 to 28.6)
Cardiovascular diseases	Norway	2000	Age-standardized		7.6% (5.6 to 9.6%)	18.4 (13.7 to 23.3)
Cardiovascular diseases	Norway	2005	Age-standardized		6.9% (5.0 to 8.9%)	12.9 (9.4 to 16.7)
Cardiovascular diseases	Norway	2010	Age-standardized		6.8% (4.9 to 8.9%)	11.0 (7.8 to 14.5)
Cardiovascular diseases	Norway	2015	Age-standardized		6.7% (4.7 to 8.8%)	10.1 (7.1 to 13.4)
Ischemic heart disease	Norway	1990	All Ages	1.4 (1.0 to 1.8)	10.7% (7.8 to 13.7%)	32.5 (23.6 to 41.9)
Ischemic heart disease	Norway	1995	All Ages	1.2 (0.8 to 1.6)	10.1% (7.0 to 13.3%)	27.6 (19.1 to 36.6)
Ischemic heart disease	Norway	2000	All Ages	1.0 (0.7 to 1.4)	9.6% (6.3 to 13.1%)	22.7 (14.8 to 31.1)
Ischemic heart disease	Norway	2005	All Ages	0.8 (0.5 to 1.1)	9.0% (5.6 to 12.8%)	16.4 (10.1 to 23.7)
Ischemic heart disease	Norway	2010	All Ages	0.7 (0.4 to 1.0)	9.1% (5.5 to 13.0%)	14.0 (8.5 to 20.1)
Ischemic heart disease	Norway	2015	All Ages	0.7 (0.4 to 1.0)	9.2% (5.5 to 13.2%)	13.0 (7.8 to 18.7)
Ischemic heart disease	Norway	1990	Age-standardized		11.1% (8.3 to 13.9%)	21.9 (16.3 to 27.7)
Ischemic heart disease	Norway	1995	Age-standardized		10.5% (7.7 to 13.5%)	17.9 (13.1 to 23.0)
Ischemic heart disease	Norway	2000	Age-standardized		10.1% (7.1 to 13.1%)	14.2 (9.9 to 18.7)
Ischemic heart disease	Norway	2005	Age-standardized		9.5% (6.4 to 12.7%)	9.9 (6.6 to 13.4)
Ischemic heart disease	Norway	2010	Age-standardized		9.5% (6.3 to 12.9%)	8.3 (5.5 to 11.3)

Cause name	Location	Year	Age group	Number (in thousands)	PAF	Rate (per 100,000)
Ischemic heart disease	Norway	2015	Age-standardized		9.5% (6.2 to 12.9%)	7.7 (5.0 to 10.7)
Ischemic stroke	Norway	1990	All Ages	0.2 (0.1 to 0.3)	6.9% (3.5 to 11.1%)	5.0 (2.5 to 8.1)
Ischemic stroke	Norway	1995	All Ages	0.2 (0.1 to 0.3)	6.5% (3.1 to 11.0%)	4.3 (2.1 to 7.5)
Ischemic stroke	Norway	2000	All Ages	0.2 (0.1 to 0.3)	6.3% (2.9 to 11.0%)	3.7 (1.7 to 6.7)
Ischemic stroke	Norway	2005	All Ages	0.1 (0.1 to 0.2)	5.9% (2.4 to 10.8%)	2.8 (1.1 to 5.2)
Ischemic stroke	Norway	2010	All Ages	0.1 (0.1 to 0.2)	5.9% (2.4 to 10.9%)	2.4 (1.0 to 4.5)
Ischemic stroke	Norway	2015	All Ages	0.1 (0.0 to 0.2)	6.0% (2.4 to 11.1%)	2.1 (0.8 to 4.0)
Ischemic stroke	Norway	1990	Age-standardized		6.9% (3.6 to 11.1%)	3.0 (1.6 to 4.9)
Ischemic stroke	Norway	1995	Age-standardized		6.6% (3.4 to 10.9%)	2.5 (1.3 to 4.2)
Ischemic stroke	Norway	2000	Age-standardized		6.4% (3.2 to 10.9%)	2.1 (1.0 to 3.6)
Ischemic stroke	Norway	2005	Age-standardized		6.0% (2.8 to 10.6%)	1.5 (0.7 to 2.7)
Ischemic stroke	Norway	2010	Age-standardized		6.1% (2.7 to 10.8%)	1.3 (0.6 to 2.4)
Ischemic stroke	Norway	2015	Age-standardized		6.1% (2.7 to 11.0%)	1.2 (0.5 to 2.1)
Hemorrhagic stroke	Norway	1990	All Ages	0.2 (0.1 to 0.3)	7.4% (4.4 to 10.8%)	4.4 (2.6 to 6.5)
Hemorrhagic stroke	Norway	1995	All Ages	0.2 (0.1 to 0.3)	7.0% (4.0 to 10.7%)	3.9 (2.2 to 5.9)
Hemorrhagic stroke	Norway	2000	All Ages	0.1 (0.1 to 0.2)	6.7% (3.7 to 10.6%)	3.3 (1.8 to 5.3)
Hemorrhagic stroke	Norway	2005	All Ages	0.1 (0.1 to 0.2)	6.3% (3.3 to 10.4%)	2.5 (1.3 to 4.3)
Hemorrhagic stroke	Norway	2010	All Ages	0.1 (0.1 to 0.2)	6.3% (3.2 to 10.8%)	2.2 (1.1 to 3.8)
Hemorrhagic stroke	Norway	2015	All Ages	0.1 (0.1 to 0.2)	6.4% (3.2 to 10.9%)	2.0 (1.0 to 3.5)
Hemorrhagic stroke	Norway	1990	Age-standardized		7.6% (4.8 to 10.9%)	2.9 (1.8 to 4.2)
Hemorrhagic stroke	Norway	1995	Age-standardized		7.3% (4.5 to 10.6%)	2.4 (1.5 to 3.6)
Hemorrhagic stroke	Norway	2000	Age-standardized		7.0% (4.2 to 10.4%)	2.1 (1.2 to 3.1)
Hemorrhagic stroke	Norway	2005	Age-standardized		6.6% (3.8 to 10.1%)	1.5 (0.9 to 2.4)
Hemorrhagic stroke	Norway	2010	Age-standardized		6.6% (3.8 to 10.3%)	1.3 (0.8 to 2.1)
Hemorrhagic stroke	Norway	2015	Age-standardized		6.6% (3.7 to 10.5%)	1.2 (0.7 to 1.9)
Chronic obstructive pulmonary disease	Norway	1990	All Ages	0.1 (0.0 to 0.2)	11.4% (3.9 to 21.0%)	3.0 (1.0 to 5.6)
Chronic obstructive pulmonary disease	Norway	1995	All Ages	0.2 (0.1 to 0.3)	10.8% (3.5 to 20.3%)	3.6 (1.1 to 6.8)
Chronic obstructive pulmonary disease	Norway	2000	All Ages	0.2 (0.1 to 0.4)	10.4% (3.3 to 19.7%)	4.4 (1.4 to 8.5)
Chronic obstructive pulmonary disease	Norway	2005	All Ages	0.2 (0.1 to 0.4)	9.6% (2.9 to 18.7%)	4.4 (1.3 to 8.6)
Chronic obstructive pulmonary disease	Norway	2010	All Ages	0.2 (0.1 to 0.5)	9.7% (2.9 to 18.9%)	4.7 (1.4 to 9.2)
Chronic obstructive pulmonary disease	Norway	2015	All Ages	0.2 (0.1 to 0.5)	9.9% (3.0 to 19.2%)	4.6 (1.3 to 9.0)
Chronic obstructive pulmonary disease	Norway	1990	Age-standardized		11.4% (3.9 to 20.9%)	1.9 (0.6 to 3.5)
Chronic obstructive pulmonary disease	Norway	1995	Age-standardized		10.8% (3.5 to 20.3%)	2.2 (0.7 to 4.2)
Chronic obstructive pulmonary disease	Norway	2000	Age-standardized		10.4% (3.3 to 19.7%)	2.7 (0.8 to 5.2)
Chronic obstructive pulmonary disease	Norway	2005	Age-standardized		9.6% (2.9 to 18.7%)	2.6 (0.8 to 5.1)
Chronic obstructive pulmonary disease	Norway	2010	Age-standardized		9.7% (2.9 to 18.9%)	2.8 (0.8 to 5.4)

Cause name	Location	Year	Age group	Number (in thousands)	PAF	Rate (per 100,000)
Chronic obstructive pulmonary disease	Norway	2015	Age-standardized		9.9% (3.0 to 19.2%)	2.7 (0.8 to 5.3)
All causes	Portugal	1990	All Ages	4.5 (3.4 to 5.6)	4.3% (3.3 to 5.5%)	44.7 (34.2 to 56.4)
All causes	Portugal	1995	All Ages	4.4 (3.4 to 5.6)	4.2% (3.2 to 5.3%)	43.3 (33.3 to 55.0)
All causes	Portugal	2000	All Ages	4.3 (3.3 to 5.6)	4.1% (3.1 to 5.3%)	41.9 (31.7 to 54.4)
All causes	Portugal	2005	All Ages	4.0 (3.0 to 5.3)	3.9% (2.9 to 5.1%)	38.3 (28.3 to 49.7)
All causes	Portugal	2010	All Ages	3.6 (2.6 to 4.8)	3.5% (2.5 to 4.7%)	33.7 (24.2 to 45.2)
All causes	Portugal	2015	All Ages	3.7 (2.6 to 5.0)	3.5% (2.5 to 4.8%)	34.0 (24.5 to 46.1)
All causes	Portugal	1990	Age-standardized		4.2% (3.2 to 5.3%)	34.8 (26.6 to 43.8)
All causes	Portugal	1995	Age-standardized		4.1% (3.1 to 5.1%)	30.6 (23.7 to 38.7)
All causes	Portugal	2000	Age-standardized		3.9% (3.0 to 5.1%)	27.7 (21.1 to 35.8)
All causes	Portugal	2005	Age-standardized		3.8% (2.8 to 4.9%)	23.2 (17.4 to 29.9)
All causes	Portugal	2010	Age-standardized		3.4% (2.5 to 4.4%)	18.3 (13.5 to 24.2)
All causes	Portugal	2015	Age-standardized		3.4% (2.5 to 4.4%)	16.9 (12.5 to 22.3)
Lower respiratory infections	Portugal	1990	All Ages	0.3 (0.1 to 0.8)	8.4% (1.9 to 19.5%)	3.3 (0.8 to 7.6)
Lower respiratory infections	Portugal	1995	All Ages	0.4 (0.1 to 0.9)	8.3% (1.8 to 19.3%)	3.7 (0.8 to 8.8)
Lower respiratory infections	Portugal	2000	All Ages	0.5 (0.1 to 1.1)	8.2% (1.8 to 19.3%)	4.4 (0.9 to 10.6)
Lower respiratory infections	Portugal	2005	All Ages	0.5 (0.1 to 1.2)	8.5% (1.9 to 19.3%)	4.9 (1.1 to 11.4)
Lower respiratory infections	Portugal	2010	All Ages	0.6 (0.1 to 1.3)	8.0% (1.7 to 19.1%)	5.1 (1.1 to 12.2)
Lower respiratory infections	Portugal	2015	All Ages	0.6 (0.1 to 1.4)	8.3% (1.9 to 19.1%)	5.4 (1.2 to 12.8)
Lower respiratory infections	Portugal	1990	Age-standardized		8.4% (1.9 to 19.5%)	2.7 (0.6 to 6.2)
Lower respiratory infections	Portugal	1995	Age-standardized		8.3% (1.8 to 19.3%)	2.7 (0.6 to 6.3)
Lower respiratory infections	Portugal	2000	Age-standardized		8.2% (1.8 to 19.3%)	2.9 (0.6 to 7.1)
Lower respiratory infections	Portugal	2005	Age-standardized		8.5% (1.9 to 19.3%)	2.9 (0.6 to 6.9)
Lower respiratory infections	Portugal	2010	Age-standardized		8.0% (1.7 to 19.1%)	2.7 (0.6 to 6.4)
Lower respiratory infections	Portugal	2015	Age-standardized		8.3% (1.9 to 19.1%)	2.6 (0.6 to 6.0)
Tracheal, bronchus, and lung cancer	Portugal	1990	All Ages	0.1 (0.1 to 0.3)	5.7% (2.5 to 10.1%)	1.6 (0.7 to 2.7)
Tracheal, bronchus, and lung cancer	Portugal	1995	All Ages	0.2 (0.1 to 0.3)	5.6% (2.4 to 9.9%)	1.8 (0.8 to 3.2)
Tracheal, bronchus, and lung cancer	Portugal	2000	All Ages	0.2 (0.1 to 0.3)	5.5% (2.4 to 9.8%)	1.8 (0.8 to 3.2)
Tracheal, bronchus, and lung cancer	Portugal	2005	All Ages	0.2 (0.1 to 0.4)	5.7% (2.5 to 10.0%)	2.1 (0.9 to 3.7)
Tracheal, bronchus, and lung cancer	Portugal	2010	All Ages	0.2 (0.1 to 0.4)	5.4% (2.3 to 9.7%)	2.1 (0.9 to 3.7)
Tracheal, bronchus, and lung cancer	Portugal	2015	All Ages	0.2 (0.1 to 0.4)	5.6% (2.4 to 10.0%)	2.1 (0.9 to 3.8)
Tracheal, bronchus, and lung cancer	Portugal	1990	Age-standardized		5.7% (2.5 to 10.0%)	1.2 (0.5 to 2.1)
Tracheal, bronchus, and lung cancer	Portugal	1995	Age-standardized		5.6% (2.4 to 9.9%)	1.3 (0.6 to 2.3)
Tracheal, bronchus, and lung cancer	Portugal	2000	Age-standardized		5.5% (2.4 to 9.8%)	1.3 (0.5 to 2.3)
Tracheal, bronchus, and lung cancer	Portugal	2005	Age-standardized		5.7% (2.5 to 10.0%)	1.4 (0.6 to 2.4)
Tracheal, bronchus, and lung cancer	Portugal	2010	Age-standardized		5.4% (2.3 to 9.6%)	1.3 (0.6 to 2.3)

Cause name	Location	Year	Age group	Number (in thousands)	PAF	Rate (per 100,000)
Tracheal, bronchus, and lung cancer	Portugal	2015	Age-standardized		5.6% (2.4 to 10.0%)	1.2 (0.5 to 2.2)
Cardiovascular diseases	Portugal	1990	All Ages	3.6 (2.7 to 4.6)	7.6% (5.7 to 9.7%)	36.0 (26.9 to 46.0)
Cardiovascular diseases	Portugal	1995	All Ages	3.4 (2.5 to 4.4)	7.5% (5.5 to 9.6%)	33.6 (24.7 to 43.6)
Cardiovascular diseases	Portugal	2000	All Ages	3.2 (2.4 to 4.2)	7.2% (5.3 to 9.5%)	30.6 (22.7 to 40.1)
Cardiovascular diseases	Portugal	2005	All Ages	2.7 (2.0 to 3.6)	7.1% (5.1 to 9.2%)	25.8 (18.7 to 33.8)
Cardiovascular diseases	Portugal	2010	All Ages	2.3 (1.6 to 3.1)	6.5% (4.6 to 8.8%)	21.5 (15.1 to 28.9)
Cardiovascular diseases	Portugal	2015	All Ages	2.3 (1.6 to 3.1)	6.6% (4.6 to 8.9%)	21.4 (14.9 to 29.0)
Cardiovascular diseases	Portugal	1990	Age-standardized		7.7% (5.7 to 9.8%)	27.9 (21.0 to 35.6)
Cardiovascular diseases	Portugal	1995	Age-standardized		7.6% (5.7 to 9.6%)	23.7 (17.7 to 30.5)
Cardiovascular diseases	Portugal	2000	Age-standardized		7.4% (5.5 to 9.5%)	20.2 (15.1 to 26.3)
Cardiovascular diseases	Portugal	2005	Age-standardized		7.2% (5.3 to 9.3%)	15.7 (11.7 to 20.3)
Cardiovascular diseases	Portugal	2010	Age-standardized		6.8% (5.0 to 8.9%)	11.7 (8.6 to 15.4)
Cardiovascular diseases	Portugal	2015	Age-standardized		6.9% (5.1 to 9.0%)	10.7 (7.7 to 14.0)
Ischemic heart disease	Portugal	1990	All Ages	1.8 (1.3 to 2.3)	10.5% (7.5 to 13.3%)	17.9 (12.9 to 22.8)
Ischemic heart disease	Portugal	1995	All Ages	1.7 (1.3 to 2.2)	10.3% (7.4 to 13.2%)	17.1 (12.3 to 22.1)
Ischemic heart disease	Portugal	2000	All Ages	1.7 (1.2 to 2.2)	10.1% (7.0 to 13.2%)	16.0 (11.3 to 21.0)
Ischemic heart disease	Portugal	2005	All Ages	1.5 (1.0 to 2.0)	10.1% (6.8 to 13.4%)	14.0 (9.5 to 18.8)
Ischemic heart disease	Portugal	2010	All Ages	1.3 (0.8 to 1.7)	9.6% (6.2 to 13.1%)	11.8 (7.7 to 16.3)
Ischemic heart disease	Portugal	2015	All Ages	1.3 (0.8 to 1.8)	9.7% (6.1 to 13.4%)	11.9 (7.5 to 16.6)
Ischemic heart disease	Portugal	1990	Age-standardized		10.6% (7.7 to 13.4%)	13.9 (10.2 to 17.8)
Ischemic heart disease	Portugal	1995	Age-standardized		10.5% (7.7 to 13.4%)	12.2 (8.9 to 15.6)
Ischemic heart disease	Portugal	2000	Age-standardized		10.3% (7.3 to 13.3%)	10.7 (7.6 to 13.9)
Ischemic heart disease	Portugal	2005	Age-standardized		10.4% (7.2 to 13.5%)	8.6 (6.1 to 11.3)
Ischemic heart disease	Portugal	2010	Age-standardized		10.0% (6.9 to 13.2%)	6.5 (4.5 to 8.7)
Ischemic heart disease	Portugal	2015	Age-standardized		10.1% (6.9 to 13.5%)	6.0 (4.1 to 8.1)
Ischemic stroke	Portugal	1990	All Ages	1.0 (0.6 to 1.6)	6.8% (3.7 to 10.6%)	10.1 (5.5 to 15.7)
Ischemic stroke	Portugal	1995	All Ages	0.9 (0.5 to 1.4)	6.6% (3.4 to 10.6%)	8.9 (4.6 to 14.1)
Ischemic stroke	Portugal	2000	All Ages	0.8 (0.4 to 1.3)	6.5% (3.3 to 10.7%)	7.7 (4.0 to 12.7)
Ischemic stroke	Portugal	2005	All Ages	0.6 (0.3 to 1.1)	6.6% (3.3 to 10.9%)	6.0 (3.0 to 10.1)
Ischemic stroke	Portugal	2010	All Ages	0.5 (0.2 to 0.9)	6.3% (2.9 to 11.0%)	5.0 (2.3 to 8.6)
Ischemic stroke	Portugal	2015	All Ages	0.5 (0.2 to 0.9)	6.4% (2.9 to 11.1%)	4.9 (2.2 to 8.7)
Ischemic stroke	Portugal	1990	Age-standardized		6.8% (3.7 to 10.6%)	7.7 (4.2 to 12.0)
Ischemic stroke	Portugal	1995	Age-standardized		6.7% (3.5 to 10.6%)	6.1 (3.2 to 9.7)
Ischemic stroke	Portugal	2000	Age-standardized		6.6% (3.4 to 10.7%)	4.9 (2.5 to 8.1)
Ischemic stroke	Portugal	2005	Age-standardized		6.6% (3.3 to 10.9%)	3.5 (1.7 to 5.8)
Ischemic stroke	Portugal	2010	Age-standardized		6.4% (3.0 to 10.8%)	2.5 (1.2 to 4.3)

Cause name	Location	Year	Age group	Number (in thousands)	PAF	Rate (per 100,000)
Ischemic stroke	Portugal	2015	Age-standardized		6.5% (3.1 to 10.9%)	2.3 (1.0 to 3.9)
Hemorrhagic stroke	Portugal	1990	All Ages	0.8 (0.5 to 1.1)	7.4% (4.7 to 10.3%)	8.1 (5.1 to 11.5)
Hemorrhagic stroke	Portugal	1995	All Ages	0.8 (0.5 to 1.1)	7.2% (4.4 to 10.3%)	7.6 (4.6 to 10.9)
Hemorrhagic stroke	Portugal	2000	All Ages	0.7 (0.4 to 1.1)	7.1% (4.3 to 10.4%)	6.9 (4.2 to 10.2)
Hemorrhagic stroke	Portugal	2005	All Ages	0.6 (0.4 to 0.9)	7.2% (4.2 to 10.6%)	5.8 (3.4 to 8.7)
Hemorrhagic stroke	Portugal	2010	All Ages	0.5 (0.3 to 0.8)	6.8% (3.9 to 10.5%)	4.7 (2.7 to 7.3)
Hemorrhagic stroke	Portugal	2015	All Ages	0.5 (0.3 to 0.8)	6.9% (3.8 to 10.7%)	4.6 (2.6 to 7.2)
Hemorrhagic stroke	Portugal	1990	Age-standardized		7.4% (4.7 to 10.4%)	6.3 (4.0 to 8.9)
Hemorrhagic stroke	Portugal	1995	Age-standardized		7.3% (4.7 to 10.3%)	5.4 (3.4 to 7.7)
Hemorrhagic stroke	Portugal	2000	Age-standardized		7.3% (4.5 to 10.5%)	4.6 (2.9 to 6.8)
Hemorrhagic stroke	Portugal	2005	Age-standardized		7.3% (4.5 to 10.6%)	3.6 (2.2 to 5.2)
Hemorrhagic stroke	Portugal	2010	Age-standardized		7.1% (4.3 to 10.5%)	2.6 (1.6 to 3.9)
Hemorrhagic stroke	Portugal	2015	Age-standardized		7.2% (4.4 to 10.6%)	2.4 (1.5 to 3.5)
Chronic obstructive pulmonary disease	Portugal	1990	All Ages	0.4 (0.1 to 0.7)	10.7% (3.5 to 20.2%)	3.8 (1.2 to 7.1)
Chronic obstructive pulmonary disease	Portugal	1995	All Ages	0.4 (0.1 to 0.8)	10.6% (3.3 to 20.0%)	4.3 (1.4 to 8.0)
Chronic obstructive pulmonary disease	Portugal	2000	All Ages	0.5 (0.2 to 1.0)	10.5% (3.4 to 19.9%)	5.0 (1.6 to 9.5)
Chronic obstructive pulmonary disease	Portugal	2005	All Ages	0.6 (0.2 to 1.1)	10.8% (3.5 to 20.4%)	5.4 (1.8 to 10.3)
Chronic obstructive pulmonary disease	Portugal	2010	All Ages	0.5 (0.2 to 1.0)	10.3% (3.3 to 19.6%)	5.1 (1.6 to 9.6)
Chronic obstructive pulmonary disease	Portugal	2015	All Ages	0.6 (0.2 to 1.0)	10.6% (3.5 to 19.8%)	5.1 (1.7 to 9.5)
Chronic obstructive pulmonary disease	Portugal	1990	Age-standardized		10.7% (3.5 to 20.1%)	2.9 (0.9 to 5.4)
Chronic obstructive pulmonary disease	Portugal	1995	Age-standardized		10.5% (3.3 to 19.9%)	2.9 (0.9 to 5.5)
Chronic obstructive pulmonary disease	Portugal	2000	Age-standardized		10.5% (3.4 to 19.8%)	3.2 (1.0 to 6.1)
Chronic obstructive pulmonary disease	Portugal	2005	Age-standardized		10.7% (3.5 to 20.3%)	3.2 (1.0 to 6.0)
Chronic obstructive pulmonary disease	Portugal	2010	Age-standardized		10.3% (3.3 to 19.6%)	2.6 (0.8 to 5.0)
Chronic obstructive pulmonary disease	Portugal	2015	Age-standardized		10.6% (3.5 to 19.7%)	2.4 (0.8 to 4.4)
All causes	Spain	1990	All Ages	13.9 (10.9 to 17.4)	4.1% (3.3 to 5.2%)	35.7 (28.0 to 44.8)
All causes	Spain	1995	All Ages	14.1 (10.9 to 17.9)	4.0% (3.0 to 5.0%)	35.8 (27.5 to 45.5)
All causes	Spain	2000	All Ages	14.3 (10.8 to 18.5)	3.9% (2.9 to 5.0%)	35.5 (26.7 to 46.0)
All causes	Spain	2005	All Ages	14.0 (10.4 to 18.3)	3.6% (2.7 to 4.8%)	32.4 (24.0 to 42.1)
All causes	Spain	2010	All Ages	12.8 (9.4 to 16.9)	3.3% (2.4 to 4.4%)	27.6 (20.2 to 36.5)
All causes	Spain	2015	All Ages	13.4 (9.7 to 17.9)	3.3% (2.3 to 4.4%)	27.5 (19.9 to 36.7)
All causes	Spain	1990	Age-standardized		4.0% (3.2 to 5.0%)	27.4 (21.7 to 34.1)
All causes	Spain	1995	Age-standardized		3.8% (3.0 to 4.9%)	24.4 (19.1 to 30.8)
All causes	Spain	2000	Age-standardized		3.8% (2.9 to 4.8%)	22.2 (17.1 to 28.4)
All causes	Spain	2005	Age-standardized		3.6% (2.7 to 4.5%)	18.9 (14.4 to 24.2)
All causes	Spain	2010	Age-standardized		3.2% (2.4 to 4.2%)	15.0 (11.4 to 19.5)

Cause name	Location	Year	Age group	Number (in thousands)	PAF	Rate (per 100,000)
All causes	Spain	2015	Age-standardized		3.2% (2.4 to 4.1%)	13.9 (10.5 to 18.0)
Lower respiratory infections	Spain	1990	All Ages	0.8 (0.2 to 1.9)	8.9% (2.2 to 20.0%)	2.2 (0.5 to 4.9)
Lower respiratory infections	Spain	1995	All Ages	0.8 (0.2 to 2.0)	8.7% (2.0 to 19.7%)	2.2 (0.5 to 4.9)
Lower respiratory infections	Spain	2000	All Ages	1.0 (0.2 to 2.3)	8.6% (2.0 to 19.7%)	2.5 (0.6 to 5.6)
Lower respiratory infections	Spain	2005	All Ages	1.1 (0.3 to 2.6)	8.5% (1.9 to 19.6%)	2.5 (0.6 to 5.9)
Lower respiratory infections	Spain	2010	All Ages	1.1 (0.2 to 2.5)	8.2% (1.8 to 19.2%)	2.3 (0.5 to 5.4)
Lower respiratory infections	Spain	2015	All Ages	1.2 (0.3 to 2.8)	8.3% (1.8 to 19.3%)	2.4 (0.5 to 5.7)
Lower respiratory infections	Spain	1990	Age-standardized		8.9% (2.2 to 20.0%)	1.7 (0.4 to 3.8)
Lower respiratory infections	Spain	1995	Age-standardized		8.7% (2.0 to 19.7%)	1.4 (0.3 to 3.3)
Lower respiratory infections	Spain	2000	Age-standardized		8.6% (2.0 to 19.7%)	1.5 (0.3 to 3.4)
Lower respiratory infections	Spain	2005	Age-standardized		8.5% (1.9 to 19.6%)	1.4 (0.3 to 3.3)
Lower respiratory infections	Spain	2010	Age-standardized		8.2% (1.8 to 19.2%)	1.2 (0.3 to 2.7)
Lower respiratory infections	Spain	2015	Age-standardized		8.3% (1.8 to 19.3%)	1.1 (0.2 to 2.7)
Tracheal, bronchus, and lung cancer	Spain	1990	All Ages	0.9 (0.4 to 1.6)	6.0% (2.7 to 10.5%)	2.4 (1.0 to 4.1)
Tracheal, bronchus, and lung cancer	Spain	1995	All Ages	1.0 (0.4 to 1.8)	5.9% (2.6 to 10.2%)	2.6 (1.1 to 4.5)
Tracheal, bronchus, and lung cancer	Spain	2000	All Ages	1.1 (0.5 to 1.9)	5.8% (2.6 to 10.2%)	2.7 (1.2 to 4.7)
Tracheal, bronchus, and lung cancer	Spain	2005	All Ages	1.2 (0.5 to 2.1)	5.7% (2.5 to 10.1%)	2.7 (1.2 to 4.8)
Tracheal, bronchus, and lung cancer	Spain	2010	All Ages	1.2 (0.5 to 2.1)	5.6% (2.4 to 9.8%)	2.5 (1.1 to 4.4)
Tracheal, bronchus, and lung cancer	Spain	2015	All Ages	1.2 (0.5 to 2.1)	5.6% (2.4 to 9.9%)	2.4 (1.0 to 4.3)
Tracheal, bronchus, and lung cancer	Spain	1990	Age-standardized		6.0% (2.7 to 10.5%)	1.9 (0.8 to 3.2)
Tracheal, bronchus, and lung cancer	Spain	1995	Age-standardized		5.9% (2.6 to 10.2%)	1.9 (0.8 to 3.3)
Tracheal, bronchus, and lung cancer	Spain	2000	Age-standardized		5.8% (2.6 to 10.2%)	1.9 (0.8 to 3.3)
Tracheal, bronchus, and lung cancer	Spain	2005	Age-standardized		5.7% (2.5 to 10.1%)	1.8 (0.8 to 3.2)
Tracheal, bronchus, and lung cancer	Spain	2010	Age-standardized		5.6% (2.4 to 9.8%)	1.6 (0.7 to 2.9)
Tracheal, bronchus, and lung cancer	Spain	2015	Age-standardized		5.6% (2.4 to 9.9%)	1.5 (0.6 to 2.6)
Cardiovascular diseases	Spain	1990	All Ages	10.1 (7.6 to 12.7)	7.7% (5.8 to 9.6%)	25.9 (19.5 to 32.5)
Cardiovascular diseases	Spain	1995	All Ages	9.8 (7.3 to 12.6)	7.3% (5.5 to 9.3%)	24.8 (18.5 to 31.9)
Cardiovascular diseases	Spain	2000	All Ages	9.4 (6.9 to 12.1)	7.0% (5.2 to 9.0%)	23.3 (17.1 to 30.1)
Cardiovascular diseases	Spain	2005	All Ages	8.9 (6.4 to 11.6)	6.8% (4.9 to 8.9%)	20.4 (14.6 to 26.8)
Cardiovascular diseases	Spain	2010	All Ages	7.8 (5.5 to 10.6)	6.4% (4.5 to 8.5%)	16.9 (11.9 to 22.8)
Cardiovascular diseases	Spain	2015	All Ages	8.2 (5.6 to 11.2)	6.3% (4.3 to 8.5%)	16.8 (11.4 to 22.9)
Cardiovascular diseases	Spain	1990	Age-standardized		7.8% (5.9 to 9.6%)	19.8 (15.1 to 24.8)
Cardiovascular diseases	Spain	1995	Age-standardized		7.5% (5.7 to 9.4%)	17.0 (12.9 to 21.4)
Cardiovascular diseases	Spain	2000	Age-standardized		7.3% (5.5 to 9.2%)	14.7 (11.0 to 18.7)
Cardiovascular diseases	Spain	2005	Age-standardized		7.1% (5.3 to 9.0%)	12.0 (9.0 to 15.3)
Cardiovascular diseases	Spain	2010	Age-standardized		6.8% (5.0 to 8.6%)	9.3 (6.9 to 11.8)

Cause name	Location	Year	Age group	Number (in thousands)	PAF	Rate (per 100,000)
Cardiovascular diseases	Spain	2015	Age-standardized		6.6% (4.9 to 8.6%)	8.5 (6.2 to 11.0)
Ischemic heart disease	Spain	1990	All Ages	6.8 (4.9 to 8.8)	10.6% (7.6 to 13.7%)	17.4 (12.6 to 22.7)
Ischemic heart disease	Spain	1995	All Ages	6.8 (4.7 to 8.9)	10.3% (7.2 to 13.6%)	17.1 (11.9 to 22.5)
Ischemic heart disease	Spain	2000	All Ages	6.6 (4.5 to 8.8)	10.1% (6.8 to 13.5%)	16.3 (11.1 to 21.8)
Ischemic heart disease	Spain	2005	All Ages	6.2 (4.1 to 8.5)	10.0% (6.6 to 13.5%)	14.4 (9.4 to 19.5)
Ischemic heart disease	Spain	2010	All Ages	5.5 (3.5 to 7.7)	9.7% (6.2 to 13.5%)	12.0 (7.6 to 16.6)
Ischemic heart disease	Spain	2015	All Ages	5.8 (3.6 to 8.3)	9.6% (5.9 to 13.6%)	12.0 (7.3 to 16.9)
Ischemic heart disease	Spain	1990	Age-standardized		10.8% (7.8 to 13.8%)	13.4 (9.8 to 17.3)
Ischemic heart disease	Spain	1995	Age-standardized		10.6% (7.6 to 13.6%)	11.9 (8.5 to 15.3)
Ischemic heart disease	Spain	2000	Age-standardized		10.5% (7.4 to 13.7%)	10.4 (7.3 to 13.6)
Ischemic heart disease	Spain	2005	Age-standardized		10.4% (7.3 to 13.6%)	8.6 (6.0 to 11.3)
Ischemic heart disease	Spain	2010	Age-standardized		10.2% (7.1 to 13.5%)	6.7 (4.6 to 8.8)
Ischemic heart disease	Spain	2015	Age-standardized		10.2% (6.9 to 13.5%)	6.2 (4.2 to 8.2)
Ischemic stroke	Spain	1990	All Ages	2.0 (1.0 to 3.2)	6.8% (3.5 to 11.0%)	5.0 (2.6 to 8.2)
Ischemic stroke	Spain	1995	All Ages	1.7 (0.8 to 2.8)	6.7% (3.3 to 11.0%)	4.3 (2.1 to 7.2)
Ischemic stroke	Spain	2000	All Ages	1.5 (0.7 to 2.6)	6.6% (3.1 to 11.2%)	3.7 (1.8 to 6.4)
Ischemic stroke	Spain	2005	All Ages	1.3 (0.6 to 2.3)	6.5% (3.0 to 11.3%)	3.0 (1.4 to 5.3)
Ischemic stroke	Spain	2010	All Ages	1.1 (0.5 to 2.0)	6.3% (2.7 to 11.3%)	2.4 (1.0 to 4.4)
Ischemic stroke	Spain	2015	All Ages	1.2 (0.5 to 2.2)	6.3% (2.6 to 11.4%)	2.4 (1.0 to 4.4)
Ischemic stroke	Spain	1990	Age-standardized		6.9% (3.6 to 11.0%)	3.8 (2.0 to 6.1)
Ischemic stroke	Spain	1995	Age-standardized		6.7% (3.4 to 11.0%)	2.8 (1.4 to 4.6)
Ischemic stroke	Spain	2000	Age-standardized		6.6% (3.2 to 11.2%)	2.2 (1.1 to 3.7)
Ischemic stroke	Spain	2005	Age-standardized		6.6% (3.2 to 11.3%)	1.6 (0.8 to 2.8)
Ischemic stroke	Spain	2010	Age-standardized		6.4% (3.0 to 11.2%)	1.2 (0.5 to 2.1)
Ischemic stroke	Spain	2015	Age-standardized		6.4% (2.9 to 11.3%)	1.1 (0.5 to 1.9)
Hemorrhagic stroke	Spain	1990	All Ages	1.3 (0.9 to 1.9)	7.6% (4.8 to 10.7%)	3.4 (2.2 to 4.9)
Hemorrhagic stroke	Spain	1995	All Ages	1.3 (0.8 to 1.9)	7.3% (4.5 to 10.8%)	3.4 (2.1 to 4.9)
Hemorrhagic stroke	Spain	2000	All Ages	1.3 (0.8 to 2.0)	7.2% (4.3 to 10.7%)	3.3 (2.0 to 5.0)
Hemorrhagic stroke	Spain	2005	All Ages	1.3 (0.8 to 2.0)	7.0% (4.1 to 10.8%)	3.0 (1.8 to 4.6)
Hemorrhagic stroke	Spain	2010	All Ages	1.1 (0.6 to 1.8)	6.8% (3.7 to 10.9%)	2.5 (1.4 to 3.9)
Hemorrhagic stroke	Spain	2015	All Ages	1.2 (0.6 to 2.0)	6.7% (3.4 to 11.1%)	2.4 (1.3 to 4.0)
Hemorrhagic stroke	Spain	1990	Age-standardized		7.7% (5.0 to 10.8%)	2.7 (1.8 to 3.8)
Hemorrhagic stroke	Spain	1995	Age-standardized		7.5% (4.8 to 10.8%)	2.3 (1.5 to 3.4)
Hemorrhagic stroke	Spain	2000	Age-standardized		7.4% (4.6 to 10.8%)	2.1 (1.3 to 3.1)
Hemorrhagic stroke	Spain	2005	Age-standardized		7.3% (4.5 to 10.8%)	1.8 (1.1 to 2.6)
Hemorrhagic stroke	Spain	2010	Age-standardized		7.1% (4.3 to 10.6%)	1.4 (0.8 to 2.1)

Cause name	Location	Year	Age group	Number (in thousands)	PAF	Rate (per 100,000)
Hemorrhagic stroke	Spain	2015	Age-standardized		7.1% (4.1 to 10.7%)	1.2 (0.7 to 1.9)
Chronic obstructive pulmonary disease	Spain	1990	All Ages	2.1 (0.7 to 3.9)	11.2% (3.8 to 20.7%)	5.3 (1.8 to 9.9)
Chronic obstructive pulmonary disease	Spain	1995	All Ages	2.5 (0.8 to 4.6)	11.0% (3.6 to 20.4%)	6.3 (2.0 to 11.7)
Chronic obstructive pulmonary disease	Spain	2000	All Ages	2.8 (0.9 to 5.3)	10.9% (3.7 to 20.3%)	7.0 (2.3 to 13.1)
Chronic obstructive pulmonary disease	Spain	2005	All Ages	2.9 (0.9 to 5.5)	10.8% (3.5 to 20.2%)	6.7 (2.1 to 12.8)
Chronic obstructive pulmonary disease	Spain	2010	All Ages	2.7 (0.9 to 5.3)	10.5% (3.4 to 19.9%)	5.9 (1.9 to 11.4)
Chronic obstructive pulmonary disease	Spain	2015	All Ages	2.9 (0.9 to 5.6)	10.6% (3.4 to 20.0%)	6.0 (1.9 to 11.5)
Chronic obstructive pulmonary disease	Spain	1990	Age-standardized		11.2% (3.8 to 20.7%)	4.0 (1.3 to 7.4)
Chronic obstructive pulmonary disease	Spain	1995	Age-standardized		11.0% (3.6 to 20.4%)	4.1 (1.3 to 7.7)
Chronic obstructive pulmonary disease	Spain	2000	Age-standardized		10.9% (3.7 to 20.3%)	4.2 (1.4 to 7.8)
Chronic obstructive pulmonary disease	Spain	2005	Age-standardized		10.8% (3.5 to 20.2%)	3.7 (1.2 to 7.0)
Chronic obstructive pulmonary disease	Spain	2010	Age-standardized		10.5% (3.4 to 19.8%)	3.0 (1.0 to 5.7)
Chronic obstructive pulmonary disease	Spain	2015	Age-standardized		10.6% (3.4 to 20.0%)	2.8 (0.9 to 5.3)
All causes	Sweden	1990	All Ages	3.1 (2.1 to 4.2)	3.3% (2.2 to 4.4%)	36.1 (24.5 to 49.3)
All causes	Sweden	1995	All Ages	2.7 (1.7 to 3.8)	2.8% (1.8 to 4.0%)	30.2 (19.3 to 42.9)
All causes	Sweden	2000	All Ages	2.4 (1.4 to 3.5)	2.5% (1.5 to 3.7%)	26.5 (16.3 to 39.1)
All causes	Sweden	2005	All Ages	1.8 (1.0 to 2.9)	2.0% (1.1 to 3.1%)	20.3 (11.2 to 31.9)
All causes	Sweden	2010	All Ages	1.7 (0.9 to 2.7)	1.9% (1.0 to 3.0%)	18.3 (10.0 to 29.1)
All causes	Sweden	2015	All Ages	2.2 (1.3 to 3.3)	2.4% (1.4 to 3.5%)	22.4 (13.5 to 33.1)
All causes	Sweden	1990	Age-standardized		3.2% (2.2 to 4.2%)	21.1 (14.7 to 28.0)
All causes	Sweden	1995	Age-standardized		2.8% (1.8 to 3.8%)	16.9 (11.2 to 23.0)
All causes	Sweden	2000	Age-standardized		2.4% (1.6 to 3.4%)	14.1 (9.2 to 19.7)
All causes	Sweden	2005	Age-standardized		1.9% (1.1 to 2.8%)	10.3 (6.1 to 15.3)
All causes	Sweden	2010	Age-standardized		1.8% (1.1 to 2.8%)	9.1 (5.4 to 13.9)
All causes	Sweden	2015	Age-standardized		2.3% (1.5 to 3.3%)	11.0 (7.0 to 15.9)
Lower respiratory infections	Sweden	1990	All Ages	0.2 (0.0 to 0.6)	4.6% (0.5 to 14.4%)	2.3 (0.3 to 7.1)
Lower respiratory infections	Sweden	1995	All Ages	0.2 (0.0 to 0.6)	4.0% (0.4 to 13.8%)	2.0 (0.2 to 6.5)
Lower respiratory infections	Sweden	2000	All Ages	0.1 (0.0 to 0.5)	3.7% (0.3 to 13.2%)	1.6 (0.1 to 5.8)
Lower respiratory infections	Sweden	2005	All Ages	0.1 (0.0 to 0.4)	3.0% (0.2 to 11.8%)	1.1 (0.1 to 4.5)
Lower respiratory infections	Sweden	2010	All Ages	0.1 (0.0 to 0.4)	3.1% (0.2 to 11.9%)	1.0 (0.1 to 4.0)
Lower respiratory infections	Sweden	2015	All Ages	0.1 (0.0 to 0.5)	4.1% (0.4 to 13.8%)	1.4 (0.1 to 4.6)
Lower respiratory infections	Sweden	1990	Age-standardized		4.6% (0.5 to 14.4%)	1.2 (0.1 to 3.9)
Lower respiratory infections	Sweden	1995	Age-standardized		4.0% (0.4 to 13.8%)	1.0 (0.1 to 3.3)
Lower respiratory infections	Sweden	2000	Age-standardized		3.7% (0.3 to 13.2%)	0.8 (0.1 to 2.8)
Lower respiratory infections	Sweden	2005	Age-standardized		3.0% (0.2 to 11.8%)	0.5 (0.0 to 2.1)
Lower respiratory infections	Sweden	2010	Age-standardized		3.1% (0.2 to 11.9%)	0.5 (0.0 to 1.8)

Cause name	Location	Year	Age group	Number (in thousands)	PAF	Rate (per 100,000)
Lower respiratory infections	Sweden	2015	Age-standardized		4.1% (0.4 to 13.8%)	0.6 (0.1 to 2.2)
Tracheal, bronchus, and lung cancer	Sweden	1990	All Ages	0.1 (0.0 to 0.2)	2.9% (0.9 to 6.3%)	1.0 (0.3 to 2.2)
Tracheal, bronchus, and lung cancer	Sweden	1995	All Ages	0.1 (0.0 to 0.2)	2.5% (0.7 to 5.9%)	0.9 (0.2 to 2.1)
Tracheal, bronchus, and lung cancer	Sweden	2000	All Ages	0.1 (0.0 to 0.2)	2.3% (0.6 to 5.5%)	0.9 (0.2 to 2.1)
Tracheal, bronchus, and lung cancer	Sweden	2005	All Ages	0.1 (0.0 to 0.2)	1.8% (0.3 to 4.9%)	0.7 (0.1 to 1.9)
Tracheal, bronchus, and lung cancer	Sweden	2010	All Ages	0.1 (0.0 to 0.2)	1.9% (0.4 to 4.9%)	0.7 (0.1 to 1.9)
Tracheal, bronchus, and lung cancer	Sweden	2015	All Ages	0.1 (0.0 to 0.2)	2.6% (0.7 to 5.9%)	1.0 (0.3 to 2.3)
Tracheal, bronchus, and lung cancer	Sweden	1990	Age-standardized		2.9% (0.9 to 6.3%)	0.7 (0.2 to 1.4)
Tracheal, bronchus, and lung cancer	Sweden	1995	Age-standardized		2.5% (0.7 to 5.9%)	0.6 (0.2 to 1.4)
Tracheal, bronchus, and lung cancer	Sweden	2000	Age-standardized		2.3% (0.6 to 5.5%)	0.6 (0.1 to 1.3)
Tracheal, bronchus, and lung cancer	Sweden	2005	Age-standardized		1.8% (0.3 to 4.9%)	0.4 (0.1 to 1.2)
Tracheal, bronchus, and lung cancer	Sweden	2010	Age-standardized		1.9% (0.4 to 4.9%)	0.4 (0.1 to 1.1)
Tracheal, bronchus, and lung cancer	Sweden	2015	Age-standardized		2.6% (0.7 to 5.9%)	0.6 (0.2 to 1.3)
Cardiovascular diseases	Sweden	1990	All Ages	2.7 (1.8 to 3.7)	5.7% (3.8 to 7.8%)	31.2 (20.8 to 43.2)
Cardiovascular diseases	Sweden	1995	All Ages	2.3 (1.4 to 3.3)	5.1% (3.1 to 7.4%)	25.7 (15.7 to 37.6)
Cardiovascular diseases	Sweden	2000	All Ages	2.0 (1.1 to 3.0)	4.6% (2.6 to 7.0%)	22.2 (12.8 to 33.5)
Cardiovascular diseases	Sweden	2005	All Ages	1.5 (0.8 to 2.6)	3.8% (1.9 to 6.4%)	16.8 (8.5 to 28.5)
Cardiovascular diseases	Sweden	2010	All Ages	1.4 (0.7 to 2.4)	3.7% (1.9 to 6.4%)	14.9 (7.5 to 25.4)
Cardiovascular diseases	Sweden	2015	All Ages	1.8 (1.0 to 2.8)	4.6% (2.6 to 7.3%)	17.8 (10.0 to 28.2)
Cardiovascular diseases	Sweden	1990	Age-standardized		5.9% (4.0 to 7.9%)	18.2 (12.5 to 24.5)
Cardiovascular diseases	Sweden	1995	Age-standardized		5.3% (3.5 to 7.4%)	14.4 (9.5 to 20.2)
Cardiovascular diseases	Sweden	2000	Age-standardized		4.8% (3.0 to 6.8%)	11.8 (7.4 to 16.9)
Cardiovascular diseases	Sweden	2005	Age-standardized		4.0% (2.2 to 6.2%)	8.5 (4.8 to 13.3)
Cardiovascular diseases	Sweden	2010	Age-standardized		3.9% (2.2 to 6.2%)	7.4 (4.2 to 11.9)
Cardiovascular diseases	Sweden	2015	Age-standardized		4.8% (2.9 to 7.1%)	8.8 (5.3 to 13.1)
Ischemic heart disease	Sweden	1990	All Ages	2.2 (1.3 to 3.1)	7.1% (4.4 to 10.3%)	25.5 (15.7 to 36.5)
Ischemic heart disease	Sweden	1995	All Ages	1.8 (1.0 to 2.8)	6.5% (3.6 to 9.9%)	20.7 (11.6 to 31.3)
Ischemic heart disease	Sweden	2000	All Ages	1.6 (0.8 to 2.5)	6.1% (3.2 to 9.7%)	17.5 (9.2 to 28.0)
Ischemic heart disease	Sweden	2005	All Ages	1.2 (0.5 to 2.1)	5.1% (2.3 to 9.2%)	13.3 (6.0 to 23.9)
Ischemic heart disease	Sweden	2010	All Ages	1.1 (0.5 to 2.0)	5.2% (2.3 to 9.3%)	11.7 (5.3 to 21.1)
Ischemic heart disease	Sweden	2015	All Ages	1.4 (0.7 to 2.3)	6.4% (3.2 to 10.3%)	14.1 (7.0 to 23.2)
Ischemic heart disease	Sweden	1990	Age-standardized		7.4% (4.8 to 10.3%)	15.0 (9.7 to 20.9)
Ischemic heart disease	Sweden	1995	Age-standardized		6.8% (4.2 to 9.9%)	11.7 (7.2 to 17.0)
Ischemic heart disease	Sweden	2000	Age-standardized		6.4% (3.7 to 9.6%)	9.4 (5.5 to 14.2)
Ischemic heart disease	Sweden	2005	Age-standardized		5.4% (2.7 to 8.9%)	6.7 (3.4 to 11.3)
Ischemic heart disease	Sweden	2010	Age-standardized		5.4% (2.7 to 9.1%)	5.9 (3.0 to 9.9)

Cause name	Location	Year	Age group	Number (in thousands)	PAF	Rate (per 100,000)
Ischemic heart disease	Sweden	2015	Age-standardized		6.6% (3.7 to 10.3%)	7.0 (3.9 to 10.9)
Ischemic stroke	Sweden	1990	All Ages	0.3 (0.1 to 0.5)	4.5% (1.9 to 8.5%)	3.3 (1.4 to 6.2)
Ischemic stroke	Sweden	1995	All Ages	0.3 (0.1 to 0.5)	4.1% (1.5 to 8.3%)	2.9 (1.1 to 5.9)
Ischemic stroke	Sweden	2000	All Ages	0.3 (0.1 to 0.5)	3.8% (1.3 to 8.3%)	2.8 (0.9 to 6.0)
Ischemic stroke	Sweden	2005	All Ages	0.2 (0.1 to 0.5)	3.2% (0.9 to 7.8%)	2.1 (0.6 to 5.1)
Ischemic stroke	Sweden	2010	All Ages	0.2 (0.1 to 0.4)	3.3% (0.9 to 8.0%)	1.8 (0.5 to 4.5)
Ischemic stroke	Sweden	2015	All Ages	0.2 (0.1 to 0.5)	4.1% (1.3 to 8.9%)	2.1 (0.7 to 4.7)
Ischemic stroke	Sweden	1990	Age-standardized		4.6% (2.0 to 8.5%)	1.8 (0.8 to 3.3)
Ischemic stroke	Sweden	1995	Age-standardized		4.2% (1.7 to 8.1%)	1.5 (0.6 to 3.0)
Ischemic stroke	Sweden	2000	Age-standardized		3.9% (1.5 to 8.1%)	1.4 (0.5 to 2.8)
Ischemic stroke	Sweden	2005	Age-standardized		3.3% (1.1 to 7.5%)	1.0 (0.3 to 2.2)
Ischemic stroke	Sweden	2010	Age-standardized		3.3% (1.0 to 7.6%)	0.9 (0.3 to 2.0)
Ischemic stroke	Sweden	2015	Age-standardized		4.1% (1.5 to 8.6%)	1.0 (0.4 to 2.1)
Hemorrhagic stroke	Sweden	1990	All Ages	0.2 (0.1 to 0.3)	4.9% (2.5 to 8.0%)	2.4 (1.2 to 4.0)
Hemorrhagic stroke	Sweden	1995	All Ages	0.2 (0.1 to 0.3)	4.4% (2.1 to 7.9%)	2.0 (1.0 to 3.6)
Hemorrhagic stroke	Sweden	2000	All Ages	0.2 (0.1 to 0.3)	4.1% (1.8 to 7.9%)	1.9 (0.8 to 3.6)
Hemorrhagic stroke	Sweden	2005	All Ages	0.1 (0.1 to 0.3)	3.4% (1.3 to 7.3%)	1.5 (0.6 to 3.1)
Hemorrhagic stroke	Sweden	2010	All Ages	0.1 (0.1 to 0.3)	3.5% (1.3 to 7.5%)	1.3 (0.5 to 2.8)
Hemorrhagic stroke	Sweden	2015	All Ages	0.1 (0.1 to 0.3)	4.3% (1.8 to 8.4%)	1.6 (0.6 to 3.1)
Hemorrhagic stroke	Sweden	1990	Age-standardized		5.0% (2.7 to 8.0%)	1.4 (0.8 to 2.3)
Hemorrhagic stroke	Sweden	1995	Age-standardized		4.6% (2.4 to 7.7%)	1.2 (0.6 to 2.0)
Hemorrhagic stroke	Sweden	2000	Age-standardized		4.3% (2.2 to 7.6%)	1.0 (0.5 to 1.8)
Hemorrhagic stroke	Sweden	2005	Age-standardized		3.6% (1.6 to 6.8%)	0.8 (0.4 to 1.5)
Hemorrhagic stroke	Sweden	2010	Age-standardized		3.6% (1.6 to 7.1%)	0.7 (0.3 to 1.3)
Hemorrhagic stroke	Sweden	2015	Age-standardized		4.5% (2.1 to 8.1%)	0.8 (0.4 to 1.5)
Chronic obstructive pulmonary disease	Sweden	1990	All Ages	0.1 (0.0 to 0.3)	6.4% (1.3 to 14.9%)	1.6 (0.3 to 3.8)
Chronic obstructive pulmonary disease	Sweden	1995	All Ages	0.1 (0.0 to 0.4)	5.8% (1.0 to 14.0%)	1.7 (0.3 to 4.1)
Chronic obstructive pulmonary disease	Sweden	2000	All Ages	0.2 (0.0 to 0.4)	5.4% (0.9 to 13.5%)	1.8 (0.3 to 4.7)
Chronic obstructive pulmonary disease	Sweden	2005	All Ages	0.1 (0.0 to 0.4)	4.4% (0.5 to 12.2%)	1.7 (0.2 to 4.6)
Chronic obstructive pulmonary disease	Sweden	2010	All Ages	0.2 (0.0 to 0.4)	4.5% (0.6 to 12.3%)	1.7 (0.2 to 4.6)
Chronic obstructive pulmonary disease	Sweden	2015	All Ages	0.2 (0.0 to 0.5)	5.9% (1.1 to 14.2%)	2.2 (0.4 to 5.2)
Chronic obstructive pulmonary disease	Sweden	1990	Age-standardized		6.4% (1.3 to 14.9%)	0.9 (0.2 to 2.2)
Chronic obstructive pulmonary disease	Sweden	1995	Age-standardized		5.8% (1.0 to 14.0%)	0.9 (0.2 to 2.2)
Chronic obstructive pulmonary disease	Sweden	2000	Age-standardized		5.4% (0.9 to 13.5%)	1.0 (0.2 to 2.4)
Chronic obstructive pulmonary disease	Sweden	2005	Age-standardized		4.4% (0.5 to 12.2%)	0.8 (0.1 to 2.3)
Chronic obstructive pulmonary disease	Sweden	2010	Age-standardized		4.5% (0.6 to 12.3%)	0.8 (0.1 to 2.3)

Cause name	Location	Year	Age group	Number (in thousands)	PAF	Rate (per 100,000)
Chronic obstructive pulmonary disease	Sweden	2015	Age-standardized		5.9% (1.1 to 14.2%)	1.0 (0.2 to 2.5)
All causes	Switzerland	1990	All Ages	3.0 (2.3 to 3.6)	4.7% (3.7 to 5.8%)	44.0 (34.9 to 54.3)
All causes	Switzerland	1995	All Ages	2.8 (2.2 to 3.6)	4.4% (3.4 to 5.6%)	40.4 (31.1 to 51.2)
All causes	Switzerland	2000	All Ages	2.6 (2.0 to 3.3)	4.1% (3.1 to 5.2%)	35.6 (27.2 to 45.8)
All causes	Switzerland	2005	All Ages	2.3 (1.7 to 3.0)	3.7% (2.8 to 4.8%)	30.5 (22.9 to 39.8)
All causes	Switzerland	2010	All Ages	2.2 (1.6 to 2.8)	3.5% (2.6 to 4.5%)	27.9 (20.7 to 36.3)
All causes	Switzerland	2015	All Ages	2.5 (1.9 to 3.2)	3.7% (2.9 to 4.8%)	29.8 (22.3 to 38.9)
All causes	Switzerland	1990	Age-standardized		4.6% (3.7 to 5.6%)	29.9 (24.2 to 36.4)
All causes	Switzerland	1995	Age-standardized		4.3% (3.4 to 5.3%)	26.4 (20.8 to 32.7)
All causes	Switzerland	2000	Age-standardized		3.9% (3.1 to 5.0%)	22.2 (17.3 to 28.1)
All causes	Switzerland	2005	Age-standardized		3.6% (2.8 to 4.6%)	17.8 (13.7 to 22.7)
All causes	Switzerland	2010	Age-standardized		3.4% (2.6 to 4.3%)	15.3 (11.7 to 19.4)
All causes	Switzerland	2015	Age-standardized		3.6% (2.8 to 4.6%)	15.5 (12.0 to 19.9)
Lower respiratory infections	Switzerland	1990	All Ages	0.2 (0.1 to 0.5)	11.6% (3.8 to 22.5%)	3.5 (1.2 to 7.1)
Lower respiratory infections	Switzerland	1995	All Ages	0.2 (0.1 to 0.5)	10.6% (3.1 to 21.7%)	3.3 (1.0 to 6.9)
Lower respiratory infections	Switzerland	2000	All Ages	0.2 (0.1 to 0.4)	9.8% (2.7 to 20.8%)	2.8 (0.8 to 6.1)
Lower respiratory infections	Switzerland	2005	All Ages	0.2 (0.0 to 0.4)	9.5% (2.4 to 20.6%)	2.3 (0.6 to 5.1)
Lower respiratory infections	Switzerland	2010	All Ages	0.2 (0.0 to 0.4)	9.6% (2.5 to 20.7%)	2.2 (0.6 to 4.7)
Lower respiratory infections	Switzerland	2015	All Ages	0.2 (0.1 to 0.4)	11.3% (3.6 to 22.3%)	2.5 (0.8 to 5.1)
Lower respiratory infections	Switzerland	1990	Age-standardized		11.6% (3.8 to 22.5%)	2.3 (0.8 to 4.6)
Lower respiratory infections	Switzerland	1995	Age-standardized		10.6% (3.1 to 21.7%)	2.0 (0.6 to 4.2)
Lower respiratory infections	Switzerland	2000	Age-standardized		9.8% (2.7 to 20.8%)	1.7 (0.5 to 3.7)
Lower respiratory infections	Switzerland	2005	Age-standardized		9.5% (2.4 to 20.6%)	1.3 (0.3 to 2.8)
Lower respiratory infections	Switzerland	2010	Age-standardized		9.6% (2.5 to 20.7%)	1.1 (0.3 to 2.5)
Lower respiratory infections	Switzerland	2015	Age-standardized		11.3% (3.6 to 22.3%)	1.2 (0.4 to 2.5)
Tracheal, bronchus, and lung cancer	Switzerland	1990	All Ages	0.2 (0.1 to 0.4)	8.0% (3.8 to 13.2%)	3.6 (1.7 to 5.9)
Tracheal, bronchus, and lung cancer	Switzerland	1995	All Ages	0.2 (0.1 to 0.4)	7.2% (3.4 to 12.3%)	3.2 (1.5 to 5.4)
Tracheal, bronchus, and lung cancer	Switzerland	2000	All Ages	0.2 (0.1 to 0.4)	6.7% (3.1 to 11.4%)	3.0 (1.4 to 5.2)
Tracheal, bronchus, and lung cancer	Switzerland	2005	All Ages	0.2 (0.1 to 0.4)	6.5% (2.9 to 11.1%)	2.9 (1.3 to 5.0)
Tracheal, bronchus, and lung cancer	Switzerland	2010	All Ages	0.2 (0.1 to 0.4)	6.5% (3.0 to 11.3%)	2.9 (1.3 to 4.9)
Tracheal, bronchus, and lung cancer	Switzerland	2015	All Ages	0.3 (0.1 to 0.5)	7.7% (3.7 to 12.9%)	3.3 (1.6 to 5.5)
Tracheal, bronchus, and lung cancer	Switzerland	1990	Age-standardized		8.0% (3.8 to 13.2%)	2.7 (1.3 to 4.5)
Tracheal, bronchus, and lung cancer	Switzerland	1995	Age-standardized		7.2% (3.4 to 12.3%)	2.4 (1.1 to 4.0)
Tracheal, bronchus, and lung cancer	Switzerland	2000	Age-standardized		6.7% (3.1 to 11.4%)	2.1 (1.0 to 3.6)
Tracheal, bronchus, and lung cancer	Switzerland	2005	Age-standardized		6.5% (2.9 to 11.1%)	1.9 (0.9 to 3.3)
Tracheal, bronchus, and lung cancer	Switzerland	2010	Age-standardized		6.5% (3.0 to 11.3%)	1.8 (0.8 to 3.1)

Cause name	Location	Year	Age group	Number (in thousands)	PAF	Rate (per 100,000)
Tracheal, bronchus, and lung cancer	Switzerland	2015	Age-standardized		7.7% (3.7 to 12.9%)	2.0 (0.9 to 3.3)
Cardiovascular diseases	Switzerland	1990	All Ages	2.2 (1.6 to 2.8)	8.4% (6.3 to 10.6%)	32.6 (24.4 to 41.3)
Cardiovascular diseases	Switzerland	1995	All Ages	2.1 (1.5 to 2.7)	8.1% (5.9 to 10.5%)	29.7 (21.5 to 38.8)
Cardiovascular diseases	Switzerland	2000	All Ages	1.9 (1.3 to 2.5)	7.7% (5.4 to 10.1%)	25.7 (18.0 to 34.1)
Cardiovascular diseases	Switzerland	2005	All Ages	1.6 (1.1 to 2.2)	7.3% (5.1 to 9.7%)	21.6 (14.9 to 29.2)
Cardiovascular diseases	Switzerland	2010	All Ages	1.5 (1.0 to 2.0)	7.0% (4.8 to 9.4%)	19.3 (13.2 to 26.1)
Cardiovascular diseases	Switzerland	2015	All Ages	1.6 (1.1 to 2.2)	7.4% (5.1 to 10.0%)	19.8 (13.6 to 27.0)
Cardiovascular diseases	Switzerland	1990	Age-standardized		8.6% (6.7 to 10.7%)	22.1 (17.0 to 27.5)
Cardiovascular diseases	Switzerland	1995	Age-standardized		8.4% (6.3 to 10.6%)	19.3 (14.5 to 24.5)
Cardiovascular diseases	Switzerland	2000	Age-standardized		7.9% (5.8 to 10.2%)	16.0 (11.5 to 20.6)
Cardiovascular diseases	Switzerland	2005	Age-standardized		7.6% (5.4 to 9.8%)	12.5 (9.0 to 16.3)
Cardiovascular diseases	Switzerland	2010	Age-standardized		7.3% (5.2 to 9.5%)	10.4 (7.4 to 13.6)
Cardiovascular diseases	Switzerland	2015	Age-standardized		7.7% (5.6 to 10.0%)	10.2 (7.4 to 13.4)
Ischemic heart disease	Switzerland	1990	All Ages	1.7 (1.2 to 2.1)	11.5% (8.1 to 14.9%)	24.5 (17.2 to 31.7)
Ischemic heart disease	Switzerland	1995	All Ages	1.6 (1.1 to 2.2)	10.8% (7.3 to 14.5%)	23.2 (15.6 to 31.1)
Ischemic heart disease	Switzerland	2000	All Ages	1.5 (0.9 to 2.0)	10.3% (6.6 to 14.1%)	20.4 (13.1 to 28.1)
Ischemic heart disease	Switzerland	2005	All Ages	1.3 (0.8 to 1.8)	10.1% (6.3 to 14.1%)	17.1 (10.6 to 24.0)
Ischemic heart disease	Switzerland	2010	All Ages	1.2 (0.7 to 1.7)	10.1% (6.0 to 14.2%)	15.2 (9.2 to 21.6)
Ischemic heart disease	Switzerland	2015	All Ages	1.3 (0.8 to 1.8)	10.7% (6.5 to 15.1%)	15.6 (9.4 to 22.1)
Ischemic heart disease	Switzerland	1990	Age-standardized		11.8% (8.6 to 15.0%)	16.8 (12.2 to 21.5)
Ischemic heart disease	Switzerland	1995	Age-standardized		11.2% (7.9 to 14.5%)	15.2 (10.6 to 19.9)
Ischemic heart disease	Switzerland	2000	Age-standardized		10.6% (7.1 to 14.1%)	12.8 (8.5 to 17.1)
Ischemic heart disease	Switzerland	2005	Age-standardized		10.5% (6.9 to 14.1%)	10.0 (6.6 to 13.5)
Ischemic heart disease	Switzerland	2010	Age-standardized		10.4% (6.8 to 14.2%)	8.3 (5.4 to 11.3)
Ischemic heart disease	Switzerland	2015	Age-standardized		11.1% (7.4 to 14.9%)	8.1 (5.3 to 10.9)
Ischemic stroke	Switzerland	1990	All Ages	0.3 (0.1 to 0.5)	7.7% (3.9 to 12.4%)	4.4 (2.3 to 7.1)
Ischemic stroke	Switzerland	1995	All Ages	0.3 (0.1 to 0.4)	7.2% (3.5 to 12.2%)	3.6 (1.7 to 5.9)
Ischemic stroke	Switzerland	2000	All Ages	0.2 (0.1 to 0.3)	6.9% (3.1 to 11.9%)	2.8 (1.2 to 4.7)
Ischemic stroke	Switzerland	2005	All Ages	0.2 (0.1 to 0.3)	6.8% (3.0 to 12.0%)	2.4 (1.0 to 4.2)
Ischemic stroke	Switzerland	2010	All Ages	0.2 (0.1 to 0.3)	6.8% (3.0 to 12.1%)	2.2 (0.9 to 3.9)
Ischemic stroke	Switzerland	2015	All Ages	0.2 (0.1 to 0.3)	7.4% (3.3 to 12.8%)	2.3 (1.0 to 3.9)
Ischemic stroke	Switzerland	1990	Age-standardized		7.8% (4.2 to 12.3%)	2.8 (1.5 to 4.4)
Ischemic stroke	Switzerland	1995	Age-standardized		7.4% (3.7 to 12.0%)	2.2 (1.1 to 3.5)
Ischemic stroke	Switzerland	2000	Age-standardized		7.0% (3.3 to 11.8%)	1.6 (0.8 to 2.7)
Ischemic stroke	Switzerland	2005	Age-standardized		6.9% (3.3 to 11.7%)	1.3 (0.6 to 2.2)
Ischemic stroke	Switzerland	2010	Age-standardized		6.9% (3.2 to 11.8%)	1.1 (0.5 to 1.9)

Cause name	Location	Year	Age group	Number (in thousands)	PAF	Rate (per 100,000)
Ischemic stroke	Switzerland	2015	Age-standardized		7.5% (3.7 to 12.5%)	1.1 (0.5 to 1.9)
Hemorrhagic stroke	Switzerland	1990	All Ages	0.3 (0.2 to 0.4)	8.3% (5.2 to 12.1%)	3.8 (2.3 to 5.4)
Hemorrhagic stroke	Switzerland	1995	All Ages	0.2 (0.1 to 0.3)	7.9% (4.7 to 11.8%)	3.0 (1.8 to 4.5)
Hemorrhagic stroke	Switzerland	2000	All Ages	0.2 (0.1 to 0.3)	7.5% (4.3 to 11.6%)	2.5 (1.5 to 3.9)
Hemorrhagic stroke	Switzerland	2005	All Ages	0.1 (0.1 to 0.2)	7.3% (4.1 to 11.7%)	2.1 (1.1 to 3.3)
Hemorrhagic stroke	Switzerland	2010	All Ages	0.1 (0.1 to 0.2)	7.3% (3.9 to 11.8%)	1.9 (1.0 to 3.0)
Hemorrhagic stroke	Switzerland	2015	All Ages	0.2 (0.1 to 0.3)	7.8% (4.3 to 12.6%)	1.9 (1.1 to 3.1)
Hemorrhagic stroke	Switzerland	1990	Age-standardized		8.6% (5.6 to 12.0%)	2.6 (1.7 to 3.6)
Hemorrhagic stroke	Switzerland	1995	Age-standardized		8.1% (5.1 to 11.6%)	2.0 (1.3 to 2.8)
Hemorrhagic stroke	Switzerland	2000	Age-standardized		7.7% (4.7 to 11.4%)	1.6 (1.0 to 2.4)
Hemorrhagic stroke	Switzerland	2005	Age-standardized		7.6% (4.5 to 11.3%)	1.2 (0.7 to 1.8)
Hemorrhagic stroke	Switzerland	2010	Age-standardized		7.5% (4.5 to 11.5%)	1.0 (0.6 to 1.6)
Hemorrhagic stroke	Switzerland	2015	Age-standardized		8.1% (5.0 to 12.3%)	1.0 (0.6 to 1.5)
Chronic obstructive pulmonary disease	Switzerland	1990	All Ages	0.3 (0.1 to 0.5)	13.7% (5.4 to 23.7%)	4.2 (1.7 to 7.3)
Chronic obstructive pulmonary disease	Switzerland	1995	All Ages	0.3 (0.1 to 0.5)	12.8% (4.8 to 22.8%)	4.1 (1.6 to 7.4)
Chronic obstructive pulmonary disease	Switzerland	2000	All Ages	0.3 (0.1 to 0.5)	12.1% (4.2 to 22.0%)	4.0 (1.4 to 7.4)
Chronic obstructive pulmonary disease	Switzerland	2005	All Ages	0.3 (0.1 to 0.5)	11.8% (4.1 to 21.6%)	3.7 (1.3 to 6.8)
Chronic obstructive pulmonary disease	Switzerland	2010	All Ages	0.3 (0.1 to 0.5)	11.9% (4.2 to 21.8%)	3.6 (1.3 to 6.7)
Chronic obstructive pulmonary disease	Switzerland	2015	All Ages	0.4 (0.1 to 0.6)	13.4% (5.2 to 23.5%)	4.2 (1.6 to 7.3)
Chronic obstructive pulmonary disease	Switzerland	1990	Age-standardized		13.7% (5.4 to 23.7%)	2.8 (1.1 to 4.9)
Chronic obstructive pulmonary disease	Switzerland	1995	Age-standardized		12.8% (4.8 to 22.8%)	2.7 (1.0 to 4.8)
Chronic obstructive pulmonary disease	Switzerland	2000	Age-standardized		12.1% (4.2 to 22.0%)	2.4 (0.8 to 4.5)
Chronic obstructive pulmonary disease	Switzerland	2005	Age-standardized		11.8% (4.1 to 21.6%)	2.1 (0.7 to 3.9)
Chronic obstructive pulmonary disease	Switzerland	2010	Age-standardized		11.9% (4.2 to 21.7%)	1.9 (0.7 to 3.6)
Chronic obstructive pulmonary disease	Switzerland	2015	Age-standardized		13.4% (5.2 to 23.5%)	2.1 (0.8 to 3.7)
All causes	United Kingdom	1990	All Ages	42.8 (35.0 to 51.0)	6.6% (5.4 to 7.9%)	74.7 (61.1 to 89.2)
All causes	United Kingdom	1995	All Ages	39.6 (31.6 to 48.1)	6.2% (4.9 to 7.5%)	68.2 (54.4 to 82.9)
All causes	United Kingdom	2000	All Ages	34.5 (26.9 to 43.1)	5.6% (4.4 to 7.0%)	58.5 (45.6 to 73.2)
All causes	United Kingdom	2005	All Ages	29.1 (22.4 to 37.1)	4.9% (3.8 to 6.2%)	48.4 (37.1 to 61.5)
All causes	United Kingdom	2010	All Ages	26.0 (19.8 to 33.4)	4.5% (3.4 to 5.8%)	41.9 (31.9 to 53.8)
All causes	United Kingdom	2015	All Ages	27.2 (20.5 to 34.9)	4.5% (3.4 to 5.8%)	42.3 (31.9 to 54.4)
All causes	United Kingdom	1990	Age-standardized		6.5% (5.4 to 7.7%)	50.3 (41.6 to 59.5)
All causes	United Kingdom	1995	Age-standardized		6.1% (4.9 to 7.3%)	44.0 (35.6 to 53.1)
All causes	United Kingdom	2000	Age-standardized		5.5% (4.3 to 6.7%)	36.7 (29.1 to 45.1)
All causes	United Kingdom	2005	Age-standardized		4.8% (3.8 to 6.0%)	28.9 (22.7 to 35.8)
All causes	United Kingdom	2010	Age-standardized		4.4% (3.4 to 5.5%)	23.8 (18.4 to 29.9)

Cause name	Location	Year	Age group	Number (in thousands)	PAF	Rate (per 100,000)
All causes	United Kingdom	2015	Age-standardized		4.4% (3.4 to 5.6%)	22.8 (17.6 to 28.8)
Lower respiratory infections	United Kingdom	1990	All Ages	5.3 (2.0 to 9.7)	12.9% (4.9 to 23.8%)	9.2 (3.4 to 16.9)
Lower respiratory infections	United Kingdom	1995	All Ages	6.2 (2.2 to 11.8)	12.2% (4.3 to 23.0%)	10.6 (3.7 to 20.3)
Lower respiratory infections	United Kingdom	2000	All Ages	5.7 (1.8 to 11.1)	11.4% (3.7 to 22.3%)	9.6 (3.1 to 18.8)
Lower respiratory infections	United Kingdom	2005	All Ages	4.5 (1.3 to 9.1)	10.8% (3.2 to 21.7%)	7.4 (2.2 to 15.1)
Lower respiratory infections	United Kingdom	2010	All Ages	4.0 (1.2 to 8.3)	10.5% (3.1 to 21.6%)	6.5 (1.9 to 13.4)
Lower respiratory infections	United Kingdom	2015	All Ages	4.3 (1.3 to 8.8)	10.8% (3.3 to 21.8%)	6.7 (2.0 to 13.7)
Lower respiratory infections	United Kingdom	1990	Age-standardized		12.9% (4.9 to 23.8%)	5.8 (2.2 to 10.8)
Lower respiratory infections	United Kingdom	1995	Age-standardized		12.2% (4.3 to 23.0%)	6.3 (2.2 to 12.1)
Lower respiratory infections	United Kingdom	2000	Age-standardized		11.4% (3.7 to 22.3%)	5.6 (1.8 to 11.0)
Lower respiratory infections	United Kingdom	2005	Age-standardized		10.8% (3.2 to 21.7%)	4.1 (1.2 to 8.3)
Lower respiratory infections	United Kingdom	2010	Age-standardized		10.5% (3.1 to 21.6%)	3.4 (1.0 to 7.0)
Lower respiratory infections	United Kingdom	2015	Age-standardized		10.8% (3.3 to 21.8%)	3.3 (1.0 to 6.8)
Tracheal, bronchus, and lung cancer	United Kingdom	1990	All Ages	3.6 (1.8 to 5.9)	8.9% (4.4 to 14.5%)	6.3 (3.1 to 10.3)
Tracheal, bronchus, and lung cancer	United Kingdom	1995	All Ages	3.2 (1.6 to 5.3)	8.3% (4.1 to 13.6%)	5.6 (2.7 to 9.1)
Tracheal, bronchus, and lung cancer	United Kingdom	2000	All Ages	2.9 (1.4 to 4.8)	7.7% (3.7 to 12.9%)	4.9 (2.4 to 8.2)
Tracheal, bronchus, and lung cancer	United Kingdom	2005	All Ages	2.7 (1.3 to 4.6)	7.3% (3.5 to 12.3%)	4.5 (2.1 to 7.6)
Tracheal, bronchus, and lung cancer	United Kingdom	2010	All Ages	2.7 (1.3 to 4.5)	7.1% (3.4 to 12.1%)	4.3 (2.0 to 7.3)
Tracheal, bronchus, and lung cancer	United Kingdom	2015	All Ages	2.8 (1.3 to 4.7)	7.3% (3.5 to 12.3%)	4.4 (2.1 to 7.4)
Tracheal, bronchus, and lung cancer	United Kingdom	1990	Age-standardized		8.9% (4.4 to 14.5%)	4.4 (2.2 to 7.2)
Tracheal, bronchus, and lung cancer	United Kingdom	1995	Age-standardized		8.3% (4.1 to 13.6%)	3.8 (1.9 to 6.3)
Tracheal, bronchus, and lung cancer	United Kingdom	2000	Age-standardized		7.7% (3.7 to 12.9%)	3.3 (1.6 to 5.5)
Tracheal, bronchus, and lung cancer	United Kingdom	2005	Age-standardized		7.3% (3.5 to 12.3%)	2.9 (1.4 to 4.9)
Tracheal, bronchus, and lung cancer	United Kingdom	2010	Age-standardized		7.1% (3.4 to 12.1%)	2.7 (1.3 to 4.5)
Tracheal, bronchus, and lung cancer	United Kingdom	2015	Age-standardized		7.3% (3.5 to 12.3%)	2.6 (1.2 to 4.3)
Cardiovascular diseases	United Kingdom	1990	All Ages	28.9 (23.1 to 35.0)	10.0% (8.0 to 12.1%)	50.5 (40.3 to 61.1)
Cardiovascular diseases	United Kingdom	1995	All Ages	25.3 (19.7 to 31.1)	9.3% (7.3 to 11.4%)	43.6 (33.9 to 53.6)
Cardiovascular diseases	United Kingdom	2000	All Ages	21.1 (16.1 to 26.1)	8.7% (6.7 to 10.8%)	35.8 (27.4 to 44.4)
Cardiovascular diseases	United Kingdom	2005	All Ages	17.2 (12.8 to 21.8)	8.1% (6.0 to 10.2%)	28.5 (21.2 to 36.2)
Cardiovascular diseases	United Kingdom	2010	All Ages	14.6 (10.7 to 18.8)	7.7% (5.7 to 9.9%)	23.4 (17.2 to 30.2)
Cardiovascular diseases	United Kingdom	2015	All Ages	14.8 (10.8 to 19.2)	7.7% (5.6 to 10.0%)	23.1 (16.8 to 29.9)
Cardiovascular diseases	United Kingdom	1990	Age-standardized		10.4% (8.4 to 12.4%)	34.4 (27.9 to 41.3)
Cardiovascular diseases	United Kingdom	1995	Age-standardized		9.7% (7.7 to 11.7%)	28.6 (22.8 to 34.5)
Cardiovascular diseases	United Kingdom	2000	Age-standardized		9.0% (7.1 to 11.0%)	22.8 (17.9 to 27.9)
Cardiovascular diseases	United Kingdom	2005	Age-standardized		8.4% (6.5 to 10.4%)	17.3 (13.3 to 21.3)
Cardiovascular diseases	United Kingdom	2010	Age-standardized		8.1% (6.2 to 10.0%)	13.5 (10.3 to 16.8)

Cause name	Location	Year	Age group	Number (in thousands)	PAF	Rate (per 100,000)
Cardiovascular diseases	United Kingdom	2015	Age-standardized		8.1% (6.1 to 10.0%)	12.6 (9.6 to 15.8)
Ischemic heart disease	United Kingdom	1990	All Ages	22.4 (16.9 to 27.8)	12.7% (9.6 to 15.8%)	39.2 (29.5 to 48.6)
Ischemic heart disease	United Kingdom	1995	All Ages	19.6 (14.5 to 24.9)	12.1% (9.0 to 15.1%)	33.7 (25.1 to 42.9)
Ischemic heart disease	United Kingdom	2000	All Ages	15.9 (11.6 to 20.4)	11.6% (8.4 to 14.8%)	27.0 (19.7 to 34.6)
Ischemic heart disease	United Kingdom	2005	All Ages	12.6 (8.8 to 16.3)	11.2% (7.8 to 14.5%)	20.9 (14.6 to 27.1)
Ischemic heart disease	United Kingdom	2010	All Ages	10.6 (7.2 to 13.9)	11.0% (7.5 to 14.5%)	17.0 (11.6 to 22.4)
Ischemic heart disease	United Kingdom	2015	All Ages	10.8 (7.3 to 14.5)	11.0% (7.4 to 14.6%)	16.9 (11.3 to 22.6)
Ischemic heart disease	United Kingdom	1990	Age-standardized		13.1% (10.2 to 16.0%)	27.0 (20.9 to 32.9)
Ischemic heart disease	United Kingdom	1995	Age-standardized		12.5% (9.5 to 15.5%)	22.4 (17.1 to 27.7)
Ischemic heart disease	United Kingdom	2000	Age-standardized		12.0% (9.0 to 15.0%)	17.4 (13.1 to 21.8)
Ischemic heart disease	United Kingdom	2005	Age-standardized		11.7% (8.6 to 14.7%)	12.9 (9.5 to 16.3)
Ischemic heart disease	United Kingdom	2010	Age-standardized		11.5% (8.4 to 14.6%)	10.0 (7.3 to 12.7)
Ischemic heart disease	United Kingdom	2015	Age-standardized		11.5% (8.3 to 14.7%)	9.4 (6.8 to 12.0)
Ischemic stroke	United Kingdom	1990	All Ages	3.6 (2.1 to 5.4)	8.3% (4.9 to 12.5%)	6.3 (3.7 to 9.5)
Ischemic stroke	United Kingdom	1995	All Ages	3.1 (1.7 to 4.8)	7.9% (4.4 to 12.3%)	5.3 (2.9 to 8.3)
Ischemic stroke	United Kingdom	2000	All Ages	2.7 (1.4 to 4.4)	7.6% (3.9 to 12.2%)	4.6 (2.4 to 7.5)
Ischemic stroke	United Kingdom	2005	All Ages	2.4 (1.2 to 4.1)	7.3% (3.5 to 12.2%)	4.1 (2.0 to 6.8)
Ischemic stroke	United Kingdom	2010	All Ages	2.1 (1.0 to 3.6)	7.2% (3.3 to 12.3%)	3.4 (1.6 to 5.7)
Ischemic stroke	United Kingdom	2015	All Ages	2.1 (0.9 to 3.6)	7.2% (3.3 to 12.5%)	3.2 (1.5 to 5.6)
Ischemic stroke	United Kingdom	1990	Age-standardized		8.4% (5.0 to 12.5%)	4.0 (2.4 to 5.9)
Ischemic stroke	United Kingdom	1995	Age-standardized		8.1% (4.6 to 12.3%)	3.2 (1.8 to 4.8)
Ischemic stroke	United Kingdom	2000	Age-standardized		7.7% (4.2 to 12.1%)	2.7 (1.5 to 4.3)
Ischemic stroke	United Kingdom	2005	Age-standardized		7.4% (3.9 to 12.0%)	2.2 (1.2 to 3.6)
Ischemic stroke	United Kingdom	2010	Age-standardized		7.3% (3.7 to 12.1%)	1.8 (0.9 to 2.9)
Ischemic stroke	United Kingdom	2015	Age-standardized		7.4% (3.7 to 12.3%)	1.6 (0.8 to 2.6)
Hemorrhagic stroke	United Kingdom	1990	All Ages	2.9 (1.9 to 3.9)	9.2% (6.1 to 12.3%)	5.0 (3.4 to 6.8)
Hemorrhagic stroke	United Kingdom	1995	All Ages	2.6 (1.7 to 3.7)	8.7% (5.7 to 12.1%)	4.6 (3.0 to 6.4)
Hemorrhagic stroke	United Kingdom	2000	All Ages	2.4 (1.5 to 3.5)	8.4% (5.3 to 12.0%)	4.1 (2.6 to 5.9)
Hemorrhagic stroke	United Kingdom	2005	All Ages	2.1 (1.3 to 3.2)	8.0% (4.9 to 11.9%)	3.6 (2.2 to 5.3)
Hemorrhagic stroke	United Kingdom	2010	All Ages	1.9 (1.1 to 2.9)	7.8% (4.5 to 12.0%)	3.1 (1.8 to 4.7)
Hemorrhagic stroke	United Kingdom	2015	All Ages	1.9 (1.1 to 3.0)	7.8% (4.5 to 12.1%)	3.0 (1.7 to 4.7)
Hemorrhagic stroke	United Kingdom	1990	Age-standardized		9.5% (6.6 to 12.5%)	3.5 (2.4 to 4.6)
Hemorrhagic stroke	United Kingdom	1995	Age-standardized		9.1% (6.2 to 12.1%)	3.0 (2.1 to 4.0)
Hemorrhagic stroke	United Kingdom	2000	Age-standardized		8.7% (5.8 to 12.0%)	2.6 (1.8 to 3.7)
Hemorrhagic stroke	United Kingdom	2005	Age-standardized		8.3% (5.4 to 11.8%)	2.2 (1.4 to 3.1)
Hemorrhagic stroke	United Kingdom	2010	Age-standardized		8.1% (5.2 to 11.7%)	1.8 (1.1 to 2.6)

Cause name	Location	Year	Age group	Number (in thousands)	PAF	Rate (per 100,000)
Hemorrhagic stroke	United Kingdom	2015	Age-standardized		8.2% (5.2 to 12.0%)	1.6 (1.0 to 2.4)
Chronic obstructive pulmonary disease	United Kingdom	1990	All Ages	5.0 (2.2 to 8.3)	15.0% (6.5 to 25.4%)	8.7 (3.8 to 14.5)
Chronic obstructive pulmonary disease	United Kingdom	1995	All Ages	4.8 (2.0 to 8.4)	14.2% (5.7 to 24.4%)	8.3 (3.5 to 14.5)
Chronic obstructive pulmonary disease	United Kingdom	2000	All Ages	4.9 (1.9 to 8.6)	13.5% (5.2 to 23.6%)	8.2 (3.2 to 14.5)
Chronic obstructive pulmonary disease	United Kingdom	2005	All Ages	4.8 (1.8 to 8.6)	12.9% (4.8 to 22.8%)	8.0 (3.0 to 14.3)
Chronic obstructive pulmonary disease	United Kingdom	2010	All Ages	4.8 (1.8 to 8.6)	12.7% (4.7 to 22.7%)	7.7 (2.9 to 13.9)
Chronic obstructive pulmonary disease	United Kingdom	2015	All Ages	5.2 (2.0 to 9.4)	13.0% (4.9 to 23.0%)	8.1 (3.1 to 14.6)
Chronic obstructive pulmonary disease	United Kingdom	1990	Age-standardized		14.9% (6.5 to 25.3%)	5.6 (2.4 to 9.4)
Chronic obstructive pulmonary disease	United Kingdom	1995	Age-standardized		14.1% (5.7 to 24.4%)	5.2 (2.2 to 9.1)
Chronic obstructive pulmonary disease	United Kingdom	2000	Age-standardized		13.5% (5.2 to 23.5%)	5.0 (2.0 to 8.9)
Chronic obstructive pulmonary disease	United Kingdom	2005	Age-standardized		12.9% (4.8 to 22.7%)	4.6 (1.7 to 8.3)
Chronic obstructive pulmonary disease	United Kingdom	2010	Age-standardized		12.7% (4.7 to 22.7%)	4.2 (1.6 to 7.6)
Chronic obstructive pulmonary disease	United Kingdom	2015	Age-standardized		13.0% (4.9 to 23.0%)	4.2 (1.6 to 7.6)
All causes	England	1990	All Ages	35.8 (29.3 to 42.7)	6.7% (5.5 to 8.0%)	75.0 (61.5 to 89.4)
All causes	England	1995	All Ages	33.2 (26.5 to 40.5)	6.3% (5.0 to 7.6%)	68.7 (54.9 to 83.7)
All causes	England	2000	All Ages	29.0 (22.5 to 36.2)	5.7% (4.4 to 7.1%)	58.8 (45.8 to 73.5)
All causes	England	2005	All Ages	24.4 (18.7 to 31.0)	5.0% (3.8 to 6.3%)	48.3 (37.1 to 61.3)
All causes	England	2010	All Ages	21.8 (16.6 to 27.9)	4.6% (3.5 to 5.9%)	41.7 (31.8 to 53.5)
All causes	England	2015	All Ages	22.7 (17.2 to 29.3)	4.6% (3.5 to 5.9%)	42.0 (31.8 to 54.2)
All causes	England	1990	Age-standardized		6.6% (5.5 to 7.8%)	50.1 (41.5 to 59.2)
All causes	England	1995	Age-standardized		6.2% (5.0 to 7.4%)	43.9 (35.6 to 53.0)
All causes	England	2000	Age-standardized		5.6% (4.4 to 6.8%)	36.7 (29.0 to 45.1)
All causes	England	2005	Age-standardized		4.9% (3.8 to 6.0%)	28.7 (22.6 to 35.6)
All causes	England	2010	Age-standardized		4.4% (3.4 to 5.6%)	23.6 (18.3 to 29.7)
All causes	England	2015	Age-standardized		4.5% (3.4 to 5.7%)	22.6 (17.5 to 28.7)
Lower respiratory infections	England	1990	All Ages	4.5 (1.7 to 8.1)	13.4% (5.3 to 24.2%)	9.4 (3.6 to 16.9)
Lower respiratory infections	England	1995	All Ages	5.4 (1.9 to 10.1)	12.6% (4.6 to 23.5%)	11.1 (4.0 to 20.8)
Lower respiratory infections	England	2000	All Ages	5.0 (1.6 to 9.6)	11.8% (3.9 to 22.6%)	10.1 (3.3 to 19.4)
Lower respiratory infections	England	2005	All Ages	3.9 (1.2 to 7.7)	11.1% (3.4 to 22.1%)	7.6 (2.4 to 15.2)
Lower respiratory infections	England	2010	All Ages	3.5 (1.0 to 7.0)	10.9% (3.3 to 21.9%)	6.6 (2.0 to 13.5)
Lower respiratory infections	England	2015	All Ages	3.7 (1.1 to 7.4)	11.1% (3.5 to 22.2%)	6.9 (2.1 to 13.7)
Lower respiratory infections	England	1990	Age-standardized		13.4% (5.3 to 24.3%)	5.9 (2.3 to 10.6)
Lower respiratory infections	England	1995	Age-standardized		12.6% (4.6 to 23.5%)	6.5 (2.3 to 12.2)
Lower respiratory infections	England	2000	Age-standardized		11.8% (3.9 to 22.6%)	5.8 (1.9 to 11.3)
Lower respiratory infections	England	2005	Age-standardized		11.1% (3.4 to 22.1%)	4.2 (1.3 to 8.3)
Lower respiratory infections	England	2010	Age-standardized		10.9% (3.3 to 21.9%)	3.4 (1.0 to 7.0)

Cause name	Location	Year	Age group	Number (in thousands)	PAF	Rate (per 100,000)
Lower respiratory infections	England	2015	Age-standardized		11.1% (3.5 to 22.2%)	3.4 (1.0 to 6.8)
Tracheal, bronchus, and lung cancer	England	1990	All Ages	3.1 (1.6 to 5.0)	9.3% (4.7 to 14.9%)	6.5 (3.2 to 10.5)
Tracheal, bronchus, and lung cancer	England	1995	All Ages	2.7 (1.4 to 4.5)	8.6% (4.3 to 14.0%)	5.7 (2.8 to 9.2)
Tracheal, bronchus, and lung cancer	England	2000	All Ages	2.4 (1.2 to 4.0)	8.1% (3.9 to 13.4%)	5.0 (2.4 to 8.2)
Tracheal, bronchus, and lung cancer	England	2005	All Ages	2.3 (1.1 to 3.8)	7.6% (3.6 to 12.8%)	4.5 (2.1 to 7.5)
Tracheal, bronchus, and lung cancer	England	2010	All Ages	2.3 (1.1 to 3.8)	7.4% (3.5 to 12.5%)	4.3 (2.0 to 7.2)
Tracheal, bronchus, and lung cancer	England	2015	All Ages	2.4 (1.1 to 3.9)	7.6% (3.7 to 12.7%)	4.4 (2.1 to 7.3)
Tracheal, bronchus, and lung cancer	England	1990	Age-standardized		9.3% (4.7 to 14.9%)	4.5 (2.3 to 7.3)
Tracheal, bronchus, and lung cancer	England	1995	Age-standardized		8.6% (4.3 to 14.0%)	3.9 (1.9 to 6.3)
Tracheal, bronchus, and lung cancer	England	2000	Age-standardized		8.1% (3.9 to 13.4%)	3.3 (1.6 to 5.5)
Tracheal, bronchus, and lung cancer	England	2005	Age-standardized		7.6% (3.6 to 12.8%)	2.9 (1.4 to 4.9)
Tracheal, bronchus, and lung cancer	England	2010	Age-standardized		7.4% (3.5 to 12.5%)	2.7 (1.3 to 4.5)
Tracheal, bronchus, and lung cancer	England	2015	Age-standardized		7.6% (3.7 to 12.7%)	2.6 (1.2 to 4.3)
Cardiovascular diseases	England	1990	All Ages	24.0 (19.1 to 29.0)	10.1% (8.0 to 12.2%)	50.3 (40.1 to 60.8)
Cardiovascular diseases	England	1995	All Ages	21.0 (16.3 to 25.9)	9.4% (7.3 to 11.6%)	43.4 (33.7 to 53.4)
Cardiovascular diseases	England	2000	All Ages	17.5 (13.3 to 21.7)	8.8% (6.7 to 10.9%)	35.5 (27.0 to 43.9)
Cardiovascular diseases	England	2005	All Ages	14.2 (10.6 to 18.0)	8.1% (6.1 to 10.3%)	28.1 (20.9 to 35.7)
Cardiovascular diseases	England	2010	All Ages	12.0 (8.8 to 15.5)	7.8% (5.7 to 10.0%)	23.0 (16.9 to 29.7)
Cardiovascular diseases	England	2015	All Ages	12.2 (8.9 to 15.9)	7.7% (5.6 to 10.0%)	22.6 (16.5 to 29.3)
Cardiovascular diseases	England	1990	Age-standardized		10.5% (8.5 to 12.5%)	34.0 (27.5 to 40.7)
Cardiovascular diseases	England	1995	Age-standardized		9.8% (7.8 to 11.8%)	28.2 (22.5 to 34.0)
Cardiovascular diseases	England	2000	Age-standardized		9.1% (7.1 to 11.0%)	22.5 (17.6 to 27.5)
Cardiovascular diseases	England	2005	Age-standardized		8.5% (6.5 to 10.4%)	17.0 (13.1 to 20.9)
Cardiovascular diseases	England	2010	Age-standardized		8.1% (6.2 to 10.1%)	13.3 (10.1 to 16.5)
Cardiovascular diseases	England	2015	Age-standardized		8.1% (6.1 to 10.1%)	12.4 (9.4 to 15.6)
Ischemic heart disease	England	1990	All Ages	18.6 (14.0 to 23.0)	12.8% (9.8 to 15.9%)	38.9 (29.4 to 48.2)
Ischemic heart disease	England	1995	All Ages	16.2 (12.0 to 20.7)	12.2% (9.1 to 15.4%)	33.6 (24.9 to 42.7)
Ischemic heart disease	England	2000	All Ages	13.2 (9.6 to 16.9)	11.7% (8.5 to 14.9%)	26.8 (19.5 to 34.2)
Ischemic heart disease	England	2005	All Ages	10.4 (7.2 to 13.4)	11.3% (7.9 to 14.6%)	20.5 (14.3 to 26.6)
Ischemic heart disease	England	2010	All Ages	8.7 (5.9 to 11.4)	11.2% (7.6 to 14.7%)	16.7 (11.3 to 21.9)
Ischemic heart disease	England	2015	All Ages	8.9 (6.0 to 12.0)	11.1% (7.4 to 14.8%)	16.5 (11.0 to 22.1)
Ischemic heart disease	England	1990	Age-standardized		13.3% (10.3 to 16.2%)	26.6 (20.6 to 32.4)
Ischemic heart disease	England	1995	Age-standardized		12.7% (9.6 to 15.7%)	22.1 (16.8 to 27.4)
Ischemic heart disease	England	2000	Age-standardized		12.2% (9.2 to 15.2%)	17.2 (12.9 to 21.5)
Ischemic heart disease	England	2005	Age-standardized		11.8% (8.8 to 14.9%)	12.6 (9.3 to 15.9)
Ischemic heart disease	England	2010	Age-standardized		11.7% (8.5 to 14.9%)	9.8 (7.1 to 12.4)

Cause name	Location	Year	Age group	Number (in thousands)	PAF	Rate (per 100,000)
Ischemic heart disease	England	2015	Age-standardized		11.7% (8.4 to 14.9%)	9.2 (6.6 to 11.8)
Ischemic stroke	England	1990	All Ages	3.0 (1.8 to 4.5)	8.5% (4.9 to 12.7%)	6.4 (3.7 to 9.5)
Ischemic stroke	England	1995	All Ages	2.6 (1.4 to 4.0)	8.1% (4.5 to 12.5%)	5.3 (2.9 to 8.3)
Ischemic stroke	England	2000	All Ages	2.3 (1.2 to 3.7)	7.7% (4.0 to 12.4%)	4.6 (2.4 to 7.4)
Ischemic stroke	England	2005	All Ages	2.0 (1.0 to 3.4)	7.4% (3.6 to 12.4%)	4.0 (2.0 to 6.7)
Ischemic stroke	England	2010	All Ages	1.7 (0.8 to 2.9)	7.3% (3.4 to 12.5%)	3.3 (1.5 to 5.6)
Ischemic stroke	England	2015	All Ages	1.7 (0.8 to 2.9)	7.4% (3.3 to 12.7%)	3.2 (1.4 to 5.4)
Ischemic stroke	England	1990	Age-standardized		8.6% (5.1 to 12.7%)	4.0 (2.4 to 5.9)
Ischemic stroke	England	1995	Age-standardized		8.2% (4.7 to 12.4%)	3.2 (1.8 to 4.8)
Ischemic stroke	England	2000	Age-standardized		7.9% (4.3 to 12.3%)	2.7 (1.5 to 4.2)
Ischemic stroke	England	2005	Age-standardized		7.6% (3.9 to 12.2%)	2.2 (1.2 to 3.6)
Ischemic stroke	England	2010	Age-standardized		7.5% (3.8 to 12.3%)	1.7 (0.9 to 2.8)
Ischemic stroke	England	2015	Age-standardized		7.5% (3.8 to 12.5%)	1.6 (0.8 to 2.6)
Hemorrhagic stroke	England	1990	All Ages	2.4 (1.6 to 3.2)	9.3% (6.3 to 12.5%)	5.0 (3.4 to 6.8)
Hemorrhagic stroke	England	1995	All Ages	2.2 (1.4 to 3.1)	8.9% (5.8 to 12.3%)	4.5 (3.0 to 6.3)
Hemorrhagic stroke	England	2000	All Ages	2.0 (1.3 to 2.9)	8.5% (5.4 to 12.1%)	4.1 (2.6 to 5.8)
Hemorrhagic stroke	England	2005	All Ages	1.8 (1.1 to 2.7)	8.1% (4.9 to 12.1%)	3.5 (2.1 to 5.3)
Hemorrhagic stroke	England	2010	All Ages	1.6 (0.9 to 2.4)	7.9% (4.6 to 12.2%)	3.0 (1.8 to 4.7)
Hemorrhagic stroke	England	2015	All Ages	1.6 (0.9 to 2.5)	7.9% (4.5 to 12.3%)	2.9 (1.7 to 4.6)
Hemorrhagic stroke	England	1990	Age-standardized		9.6% (6.8 to 12.7%)	3.4 (2.4 to 4.5)
Hemorrhagic stroke	England	1995	Age-standardized		9.2% (6.3 to 12.3%)	3.0 (2.0 to 4.0)
Hemorrhagic stroke	England	2000	Age-standardized		8.8% (5.9 to 12.1%)	2.6 (1.8 to 3.6)
Hemorrhagic stroke	England	2005	Age-standardized		8.5% (5.5 to 12.0%)	2.2 (1.4 to 3.0)
Hemorrhagic stroke	England	2010	Age-standardized		8.3% (5.3 to 12.0%)	1.7 (1.1 to 2.5)
Hemorrhagic stroke	England	2015	Age-standardized		8.3% (5.3 to 12.2%)	1.6 (1.0 to 2.4)
Chronic obstructive pulmonary disease	England	1990	All Ages	4.3 (1.9 to 7.1)	15.3% (6.7 to 25.8%)	8.9 (3.9 to 15.0)
Chronic obstructive pulmonary disease	England	1995	All Ages	4.1 (1.7 to 7.1)	14.5% (6.0 to 24.8%)	8.5 (3.6 to 14.7)
Chronic obstructive pulmonary disease	England	2000	All Ages	4.1 (1.6 to 7.2)	13.8% (5.4 to 24.0%)	8.3 (3.3 to 14.6)
Chronic obstructive pulmonary disease	England	2005	All Ages	4.1 (1.6 to 7.3)	13.3% (5.0 to 23.3%)	8.0 (3.1 to 14.4)
Chronic obstructive pulmonary disease	England	2010	All Ages	4.0 (1.6 to 7.3)	13.1% (5.0 to 23.1%)	7.8 (3.0 to 13.9)
Chronic obstructive pulmonary disease	England	2015	All Ages	4.4 (1.7 to 7.9)	13.3% (5.1 to 23.4%)	8.2 (3.2 to 14.6)
Chronic obstructive pulmonary disease	England	1990	Age-standardized		15.3% (6.7 to 25.8%)	5.8 (2.5 to 9.6)
Chronic obstructive pulmonary disease	England	1995	Age-standardized		14.5% (6.0 to 24.8%)	5.3 (2.2 to 9.1)
Chronic obstructive pulmonary disease	England	2000	Age-standardized		13.8% (5.4 to 24.0%)	5.0 (2.0 to 8.8)
Chronic obstructive pulmonary disease	England	2005	Age-standardized		13.3% (5.0 to 23.2%)	4.6 (1.8 to 8.3)
Chronic obstructive pulmonary disease	England	2010	Age-standardized		13.1% (5.0 to 23.1%)	4.3 (1.6 to 7.6)

Cause name	Location	Year	Age group	Number (in thousands)	PAF	Rate (per 100,000)
Chronic obstructive pulmonary disease	England	2015	Age-standardized		13.3% (5.1 to 23.4%)	4.3 (1.7 to 7.6)
All causes	Northern Ireland	1990	All Ages	1.0 (0.8 to 1.2)	6.2% (4.8 to 7.6%)	61.0 (47.9 to 75.7)
All causes	Northern Ireland	1995	All Ages	0.9 (0.7 to 1.1)	5.7% (4.4 to 7.2%)	53.4 (41.0 to 67.6)
All causes	Northern Ireland	2000	All Ages	0.8 (0.6 to 1.0)	5.1% (3.9 to 6.6%)	44.9 (33.8 to 58.2)
All causes	Northern Ireland	2005	All Ages	0.7 (0.5 to 0.8)	4.4% (3.3 to 5.6%)	38.1 (28.5 to 49.1)
All causes	Northern Ireland	2010	All Ages	0.6 (0.5 to 0.8)	4.0% (2.9 to 5.2%)	34.3 (25.3 to 45.1)
All causes	Northern Ireland	2015	All Ages	0.7 (0.5 to 0.9)	4.2% (3.1 to 5.4%)	36.0 (26.4 to 47.2)
All causes	Northern Ireland	1990	Age-standardized		6.1% (4.9 to 7.5%)	50.2 (39.7 to 62.0)
All causes	Northern Ireland	1995	Age-standardized		5.7% (4.4 to 7.1%)	42.3 (32.9 to 53.0)
All causes	Northern Ireland	2000	Age-standardized		5.0% (3.9 to 6.5%)	34.5 (26.3 to 44.2)
All causes	Northern Ireland	2005	Age-standardized		4.3% (3.3 to 5.5%)	27.2 (20.6 to 34.7)
All causes	Northern Ireland	2010	Age-standardized		3.9% (2.9 to 5.0%)	22.9 (17.2 to 29.5)
All causes	Northern Ireland	2015	Age-standardized		4.0% (3.0 to 5.3%)	22.3 (16.7 to 28.8)
Lower respiratory infections	Northern Ireland	1990	All Ages	0.2 (0.0 to 0.3)	9.9% (2.7 to 20.9%)	9.8 (2.7 to 20.9)
Lower respiratory infections	Northern Ireland	1995	All Ages	0.1 (0.0 to 0.3)	9.3% (2.3 to 20.3%)	9.3 (2.4 to 20.6)
Lower respiratory infections	Northern Ireland	2000	All Ages	0.1 (0.0 to 0.3)	8.8% (2.1 to 19.8%)	7.6 (1.8 to 17.1)
Lower respiratory infections	Northern Ireland	2005	All Ages	0.1 (0.0 to 0.2)	8.2% (1.7 to 19.3%)	5.7 (1.2 to 13.4)
Lower respiratory infections	Northern Ireland	2010	All Ages	0.1 (0.0 to 0.2)	8.0% (1.6 to 18.8%)	5.0 (1.0 to 12.2)
Lower respiratory infections	Northern Ireland	2015	All Ages	0.1 (0.0 to 0.2)	8.6% (1.9 to 19.6%)	5.5 (1.2 to 13.0)
Lower respiratory infections	Northern Ireland	1990	Age-standardized		9.9% (2.7 to 20.9%)	7.8 (2.2 to 16.5)
Lower respiratory infections	Northern Ireland	1995	Age-standardized		9.3% (2.3 to 20.3%)	7.0 (1.8 to 15.4)
Lower respiratory infections	Northern Ireland	2000	Age-standardized		8.8% (2.1 to 19.8%)	5.6 (1.3 to 12.6)
Lower respiratory infections	Northern Ireland	2005	Age-standardized		8.2% (1.7 to 19.3%)	3.8 (0.8 to 9.1)
Lower respiratory infections	Northern Ireland	2010	Age-standardized		8.0% (1.6 to 18.8%)	3.1 (0.6 to 7.6)
Lower respiratory infections	Northern Ireland	2015	Age-standardized		8.6% (1.9 to 19.6%)	3.2 (0.7 to 7.5)
Tracheal, bronchus, and lung cancer	Northern Ireland	1990	All Ages	0.1 (0.0 to 0.1)	6.7% (3.1 to 11.4%)	3.5 (1.6 to 5.9)
Tracheal, bronchus, and lung cancer	Northern Ireland	1995	All Ages	0.1 (0.0 to 0.1)	6.3% (2.9 to 11.0%)	3.1 (1.4 to 5.5)
Tracheal, bronchus, and lung cancer	Northern Ireland	2000	All Ages	0.1 (0.0 to 0.1)	5.9% (2.7 to 10.4%)	3.0 (1.4 to 5.3)
Tracheal, bronchus, and lung cancer	Northern Ireland	2005	All Ages	0.1 (0.0 to 0.1)	5.5% (2.4 to 9.9%)	3.0 (1.3 to 5.3)
Tracheal, bronchus, and lung cancer	Northern Ireland	2010	All Ages	0.1 (0.0 to 0.1)	5.4% (2.2 to 9.6%)	3.0 (1.2 to 5.5)
Tracheal, bronchus, and lung cancer	Northern Ireland	2015	All Ages	0.1 (0.0 to 0.1)	5.8% (2.6 to 10.2%)	3.3 (1.5 to 5.8)
Tracheal, bronchus, and lung cancer	Northern Ireland	1990	Age-standardized		6.7% (3.1 to 11.4%)	2.9 (1.3 to 5.0)
Tracheal, bronchus, and lung cancer	Northern Ireland	1995	Age-standardized		6.3% (2.9 to 11.0%)	2.6 (1.2 to 4.5)
Tracheal, bronchus, and lung cancer	Northern Ireland	2000	Age-standardized		5.9% (2.7 to 10.4%)	2.4 (1.1 to 4.2)
Tracheal, bronchus, and lung cancer	Northern Ireland	2005	Age-standardized		5.5% (2.4 to 9.9%)	2.3 (1.0 to 4.0)
Tracheal, bronchus, and lung cancer	Northern Ireland	2010	Age-standardized		5.4% (2.2 to 9.6%)	2.2 (0.9 to 3.9)

Cause name	Location	Year	Age group	Number (in thousands)	PAF	Rate (per 100,000)
Tracheal, bronchus, and lung cancer	Northern Ireland	2015	Age-standardized		5.8% (2.6 to 10.2%)	2.2 (1.0 to 3.8)
Cardiovascular diseases	Northern Ireland	1990	All Ages	0.7 (0.5 to 0.8)	9.6% (7.6 to 11.7%)	42.3 (33.3 to 51.8)
Cardiovascular diseases	Northern Ireland	1995	All Ages	0.6 (0.5 to 0.7)	9.0% (7.0 to 11.0%)	35.8 (27.7 to 44.2)
Cardiovascular diseases	Northern Ireland	2000	All Ages	0.5 (0.4 to 0.6)	8.4% (6.3 to 10.5%)	29.2 (22.1 to 36.3)
Cardiovascular diseases	Northern Ireland	2005	All Ages	0.4 (0.3 to 0.5)	7.8% (5.8 to 10.0%)	24.2 (17.8 to 31.1)
Cardiovascular diseases	Northern Ireland	2010	All Ages	0.4 (0.3 to 0.5)	7.5% (5.4 to 9.6%)	20.8 (15.1 to 26.9)
Cardiovascular diseases	Northern Ireland	2015	All Ages	0.4 (0.3 to 0.5)	7.6% (5.5 to 9.8%)	21.1 (15.1 to 27.4)
Cardiovascular diseases	Northern Ireland	1990	Age-standardized		9.8% (7.8 to 11.9%)	35.2 (28.1 to 42.9)
Cardiovascular diseases	Northern Ireland	1995	Age-standardized		9.2% (7.3 to 11.3%)	28.7 (22.6 to 35.1)
Cardiovascular diseases	Northern Ireland	2000	Age-standardized		8.6% (6.6 to 10.6%)	22.6 (17.3 to 27.9)
Cardiovascular diseases	Northern Ireland	2005	Age-standardized		8.0% (6.1 to 10.1%)	17.4 (13.2 to 22.0)
Cardiovascular diseases	Northern Ireland	2010	Age-standardized		7.7% (5.8 to 9.7%)	14.0 (10.5 to 17.7)
Cardiovascular diseases	Northern Ireland	2015	Age-standardized		7.9% (5.9 to 9.9%)	13.2 (9.8 to 16.7)
Ischemic heart disease	Northern Ireland	1990	All Ages	0.5 (0.4 to 0.7)	11.6% (8.8 to 14.4%)	34.1 (26.2 to 42.7)
Ischemic heart disease	Northern Ireland	1995	All Ages	0.5 (0.4 to 0.6)	11.0% (8.2 to 13.9%)	28.5 (21.2 to 36.3)
Ischemic heart disease	Northern Ireland	2000	All Ages	0.4 (0.3 to 0.5)	10.6% (7.6 to 13.5%)	22.7 (16.3 to 29.2)
Ischemic heart disease	Northern Ireland	2005	All Ages	0.3 (0.2 to 0.4)	10.2% (7.1 to 13.2%)	18.4 (12.9 to 24.0)
Ischemic heart disease	Northern Ireland	2010	All Ages	0.3 (0.2 to 0.4)	10.0% (6.9 to 13.2%)	15.6 (10.7 to 20.6)
Ischemic heart disease	Northern Ireland	2015	All Ages	0.3 (0.2 to 0.4)	10.2% (6.9 to 13.5%)	15.8 (10.6 to 21.0)
Ischemic heart disease	Northern Ireland	1990	Age-standardized		11.8% (9.2 to 14.6%)	28.5 (22.3 to 35.3)
Ischemic heart disease	Northern Ireland	1995	Age-standardized		11.3% (8.5 to 14.1%)	23.0 (17.5 to 29.0)
Ischemic heart disease	Northern Ireland	2000	Age-standardized		10.9% (8.0 to 13.7%)	17.7 (13.1 to 22.6)
Ischemic heart disease	Northern Ireland	2005	Age-standardized		10.5% (7.6 to 13.3%)	13.4 (9.7 to 17.1)
Ischemic heart disease	Northern Ireland	2010	Age-standardized		10.3% (7.4 to 13.2%)	10.7 (7.7 to 13.8)
Ischemic heart disease	Northern Ireland	2015	Age-standardized		10.6% (7.6 to 13.5%)	10.0 (7.1 to 12.9)
Ischemic stroke	Northern Ireland	1990	All Ages	0.1 (0.0 to 0.1)	7.3% (4.0 to 11.2%)	4.4 (2.5 to 6.9)
Ischemic stroke	Northern Ireland	1995	All Ages	0.1 (0.0 to 0.1)	7.0% (3.7 to 11.2%)	3.9 (2.0 to 6.3)
Ischemic stroke	Northern Ireland	2000	All Ages	0.1 (0.0 to 0.1)	6.7% (3.4 to 11.1%)	3.5 (1.7 to 5.8)
Ischemic stroke	Northern Ireland	2005	All Ages	0.1 (0.0 to 0.1)	6.4% (3.0 to 11.1%)	3.1 (1.4 to 5.3)
Ischemic stroke	Northern Ireland	2010	All Ages	0.1 (0.0 to 0.1)	6.3% (2.8 to 11.0%)	2.7 (1.2 to 4.7)
Ischemic stroke	Northern Ireland	2015	All Ages	0.1 (0.0 to 0.1)	6.5% (2.9 to 11.5%)	2.7 (1.2 to 4.9)
Ischemic stroke	Northern Ireland	1990	Age-standardized		7.4% (4.1 to 11.2%)	3.5 (2.0 to 5.4)
Ischemic stroke	Northern Ireland	1995	Age-standardized		7.1% (3.8 to 11.2%)	2.9 (1.6 to 4.6)
Ischemic stroke	Northern Ireland	2000	Age-standardized		6.8% (3.5 to 11.1%)	2.5 (1.3 to 4.2)
Ischemic stroke	Northern Ireland	2005	Age-standardized		6.5% (3.2 to 10.9%)	2.1 (1.0 to 3.5)
Ischemic stroke	Northern Ireland	2010	Age-standardized		6.4% (3.0 to 10.8%)	1.7 (0.8 to 2.9)

Cause name	Location	Year	Age group	Number (in thousands)	PAF	Rate (per 100,000)
Ischemic stroke	Northern Ireland	2015	Age-standardized		6.6% (3.1 to 11.4%)	1.6 (0.7 to 2.8)
Hemorrhagic stroke	Northern Ireland	1990	All Ages	0.1 (0.0 to 0.1)	8.1% (5.2 to 11.2%)	3.8 (2.5 to 5.3)
Hemorrhagic stroke	Northern Ireland	1995	All Ages	0.1 (0.0 to 0.1)	7.7% (4.9 to 11.0%)	3.4 (2.2 to 4.9)
Hemorrhagic stroke	Northern Ireland	2000	All Ages	0.1 (0.0 to 0.1)	7.4% (4.6 to 10.8%)	3.0 (1.8 to 4.4)
Hemorrhagic stroke	Northern Ireland	2005	All Ages	0.1 (0.0 to 0.1)	7.0% (4.2 to 10.6%)	2.7 (1.6 to 4.1)
Hemorrhagic stroke	Northern Ireland	2010	All Ages	0.0 (0.0 to 0.1)	6.9% (3.9 to 10.6%)	2.5 (1.5 to 3.9)
Hemorrhagic stroke	Northern Ireland	2015	All Ages	0.1 (0.0 to 0.1)	7.1% (3.9 to 11.1%)	2.5 (1.5 to 4.0)
Hemorrhagic stroke	Northern Ireland	1990	Age-standardized		8.3% (5.5 to 11.2%)	3.2 (2.1 to 4.3)
Hemorrhagic stroke	Northern Ireland	1995	Age-standardized		7.9% (5.2 to 11.0%)	2.7 (1.8 to 3.8)
Hemorrhagic stroke	Northern Ireland	2000	Age-standardized		7.6% (4.9 to 10.8%)	2.3 (1.5 to 3.4)
Hemorrhagic stroke	Northern Ireland	2005	Age-standardized		7.2% (4.5 to 10.6%)	2.0 (1.2 to 2.9)
Hemorrhagic stroke	Northern Ireland	2010	Age-standardized		7.1% (4.3 to 10.4%)	1.7 (1.0 to 2.5)
Hemorrhagic stroke	Northern Ireland	2015	Age-standardized		7.3% (4.4 to 10.9%)	1.6 (1.0 to 2.4)
Chronic obstructive pulmonary disease	Northern Ireland	1990	All Ages	0.1 (0.0 to 0.1)	12.1% (4.1 to 21.9%)	5.3 (1.9 to 9.6)
Chronic obstructive pulmonary disease	Northern Ireland	1995	All Ages	0.1 (0.0 to 0.1)	11.6% (3.9 to 21.1%)	5.2 (1.8 to 9.4)
Chronic obstructive pulmonary disease	Northern Ireland	2000	All Ages	0.1 (0.0 to 0.2)	11.1% (3.6 to 20.7%)	5.2 (1.7 to 9.9)
Chronic obstructive pulmonary disease	Northern Ireland	2005	All Ages	0.1 (0.0 to 0.2)	10.5% (3.4 to 19.8%)	5.3 (1.7 to 10.1)
Chronic obstructive pulmonary disease	Northern Ireland	2010	All Ages	0.1 (0.0 to 0.2)	10.3% (3.2 to 19.9%)	5.5 (1.7 to 10.4)
Chronic obstructive pulmonary disease	Northern Ireland	2015	All Ages	0.1 (0.0 to 0.2)	10.9% (3.6 to 20.6%)	6.1 (2.0 to 11.6)
Chronic obstructive pulmonary disease	Northern Ireland	1990	Age-standardized		12.1% (4.1 to 21.9%)	4.3 (1.5 to 7.7)
Chronic obstructive pulmonary disease	Northern Ireland	1995	Age-standardized		11.6% (3.9 to 21.1%)	4.0 (1.4 to 7.3)
Chronic obstructive pulmonary disease	Northern Ireland	2000	Age-standardized		11.1% (3.6 to 20.7%)	3.9 (1.3 to 7.4)
Chronic obstructive pulmonary disease	Northern Ireland	2005	Age-standardized		10.5% (3.4 to 19.8%)	3.7 (1.2 to 7.0)
Chronic obstructive pulmonary disease	Northern Ireland	2010	Age-standardized		10.3% (3.2 to 19.9%)	3.6 (1.1 to 6.7)
Chronic obstructive pulmonary disease	Northern Ireland	2015	Age-standardized		10.9% (3.6 to 20.6%)	3.7 (1.2 to 7.0)
All causes	Scotland	1990	All Ages	3.9 (3.2 to 4.7)	6.3% (5.1 to 7.6%)	76.6 (62.2 to 92.0)
All causes	Scotland	1995	All Ages	3.5 (2.8 to 4.2)	5.7% (4.6 to 7.0%)	67.7 (53.8 to 82.7)
All causes	Scotland	2000	All Ages	3.0 (2.4 to 3.7)	5.1% (4.0 to 6.3%)	58.9 (46.4 to 72.7)
All causes	Scotland	2005	All Ages	2.6 (2.0 to 3.2)	4.5% (3.4 to 5.7%)	50.4 (38.5 to 63.7)
All causes	Scotland	2010	All Ages	2.3 (1.7 to 2.9)	4.1% (3.1 to 5.3%)	44.0 (33.3 to 56.7)
All causes	Scotland	2015	All Ages	2.3 (1.8 to 3.0)	4.2% (3.2 to 5.4%)	43.9 (33.3 to 56.9)
All causes	Scotland	1990	Age-standardized		6.2% (5.1 to 7.5%)	54.2 (44.3 to 64.9)
All causes	Scotland	1995	Age-standardized		5.6% (4.5 to 6.8%)	46.2 (37.2 to 55.9)
All causes	Scotland	2000	Age-standardized		5.0% (4.0 to 6.1%)	38.4 (30.5 to 47.0)
All causes	Scotland	2005	Age-standardized		4.4% (3.4 to 5.5%)	30.7 (23.9 to 38.4)
All causes	Scotland	2010	Age-standardized		4.0% (3.1 to 5.1%)	25.2 (19.3 to 32.2)

Cause name	Location	Year	Age group	Number (in thousands)	PAF	Rate (per 100,000)
All causes	Scotland	2015	Age-standardized		4.0% (3.1 to 5.2%)	23.8 (18.6 to 30.4)
Lower respiratory infections	Scotland	1990	All Ages	0.4 (0.1 to 0.9)	10.8% (3.3 to 21.7%)	8.3 (2.6 to 17.1)
Lower respiratory infections	Scotland	1995	All Ages	0.4 (0.1 to 0.8)	10.0% (2.7 to 21.0%)	7.6 (2.1 to 15.9)
Lower respiratory infections	Scotland	2000	All Ages	0.3 (0.1 to 0.7)	9.3% (2.4 to 20.3%)	6.3 (1.6 to 13.8)
Lower respiratory infections	Scotland	2005	All Ages	0.3 (0.1 to 0.7)	8.8% (2.2 to 19.9%)	5.6 (1.4 to 13.0)
Lower respiratory infections	Scotland	2010	All Ages	0.3 (0.1 to 0.6)	8.5% (1.9 to 19.5%)	5.2 (1.1 to 11.9)
Lower respiratory infections	Scotland	2015	All Ages	0.3 (0.1 to 0.7)	8.9% (2.1 to 19.8%)	5.3 (1.3 to 12.3)
Lower respiratory infections	Scotland	1990	Age-standardized		10.8% (3.3 to 21.7%)	5.7 (1.8 to 11.6)
Lower respiratory infections	Scotland	1995	Age-standardized		10.0% (2.7 to 21.0%)	4.9 (1.4 to 10.3)
Lower respiratory infections	Scotland	2000	Age-standardized		9.3% (2.4 to 20.3%)	3.9 (1.0 to 8.6)
Lower respiratory infections	Scotland	2005	Age-standardized		8.8% (2.2 to 19.9%)	3.2 (0.8 to 7.5)
Lower respiratory infections	Scotland	2010	Age-standardized		8.5% (1.9 to 19.5%)	2.8 (0.6 to 6.4)
Lower respiratory infections	Scotland	2015	Age-standardized		8.9% (2.1 to 19.8%)	2.7 (0.6 to 6.2)
Tracheal, bronchus, and lung cancer	Scotland	1990	All Ages	0.3 (0.1 to 0.5)	7.4% (3.4 to 12.3%)	6.4 (2.9 to 10.6)
Tracheal, bronchus, and lung cancer	Scotland	1995	All Ages	0.3 (0.1 to 0.5)	6.8% (3.1 to 11.7%)	5.8 (2.6 to 9.9)
Tracheal, bronchus, and lung cancer	Scotland	2000	All Ages	0.3 (0.1 to 0.5)	6.3% (2.8 to 10.9%)	5.3 (2.4 to 9.1)
Tracheal, bronchus, and lung cancer	Scotland	2005	All Ages	0.3 (0.1 to 0.4)	6.0% (2.7 to 10.3%)	5.0 (2.2 to 8.7)
Tracheal, bronchus, and lung cancer	Scotland	2010	All Ages	0.3 (0.1 to 0.4)	5.7% (2.5 to 10.2%)	4.8 (2.1 to 8.5)
Tracheal, bronchus, and lung cancer	Scotland	2015	All Ages	0.3 (0.1 to 0.5)	6.0% (2.7 to 10.4%)	4.8 (2.1 to 8.4)
Tracheal, bronchus, and lung cancer	Scotland	1990	Age-standardized		7.4% (3.4 to 12.3%)	4.6 (2.1 to 7.6)
Tracheal, bronchus, and lung cancer	Scotland	1995	Age-standardized		6.8% (3.1 to 11.7%)	4.1 (1.9 to 7.0)
Tracheal, bronchus, and lung cancer	Scotland	2000	Age-standardized		6.3% (2.8 to 10.9%)	3.6 (1.6 to 6.2)
Tracheal, bronchus, and lung cancer	Scotland	2005	Age-standardized		6.0% (2.7 to 10.3%)	3.2 (1.4 to 5.6)
Tracheal, bronchus, and lung cancer	Scotland	2010	Age-standardized		5.7% (2.5 to 10.2%)	2.9 (1.3 to 5.2)
Tracheal, bronchus, and lung cancer	Scotland	2015	Age-standardized		6.0% (2.7 to 10.4%)	2.8 (1.2 to 4.8)
Cardiovascular diseases	Scotland	1990	All Ages	2.8 (2.2 to 3.4)	9.8% (7.8 to 11.8%)	54.7 (43.7 to 66.6)
Cardiovascular diseases	Scotland	1995	All Ages	2.4 (1.9 to 2.9)	9.1% (7.2 to 11.1%)	46.9 (36.8 to 57.4)
Cardiovascular diseases	Scotland	2000	All Ages	2.0 (1.5 to 2.5)	8.4% (6.5 to 10.5%)	39.2 (30.1 to 48.5)
Cardiovascular diseases	Scotland	2005	All Ages	1.6 (1.2 to 2.1)	7.9% (6.0 to 10.0%)	31.9 (24.1 to 40.5)
Cardiovascular diseases	Scotland	2010	All Ages	1.4 (1.0 to 1.8)	7.5% (5.6 to 9.6%)	26.5 (19.6 to 34.0)
Cardiovascular diseases	Scotland	2015	All Ages	1.4 (1.0 to 1.8)	7.6% (5.5 to 9.7%)	25.9 (19.0 to 33.2)
Cardiovascular diseases	Scotland	1990	Age-standardized		10.0% (8.1 to 12.1%)	39.0 (31.5 to 47.1)
Cardiovascular diseases	Scotland	1995	Age-standardized		9.4% (7.5 to 11.3%)	32.3 (25.8 to 39.1)
Cardiovascular diseases	Scotland	2000	Age-standardized		8.7% (6.8 to 10.7%)	25.8 (20.1 to 31.7)
Cardiovascular diseases	Scotland	2005	Age-standardized		8.2% (6.4 to 10.2%)	19.6 (15.2 to 24.4)
Cardiovascular diseases	Scotland	2010	Age-standardized		7.9% (6.0 to 9.8%)	15.3 (11.7 to 19.1)

Cause name	Location	Year	Age group	Number (in thousands)	PAF	Rate (per 100,000)
Cardiovascular diseases	Scotland	2015	Age-standardized		7.9% (6.0 to 9.8%)	14.2 (10.7 to 17.7)
Ischemic heart disease	Scotland	1990	All Ages	2.1 (1.6 to 2.7)	12.1% (9.2 to 15.0%)	42.4 (32.3 to 52.8)
Ischemic heart disease	Scotland	1995	All Ages	1.8 (1.4 to 2.3)	11.5% (8.6 to 14.3%)	35.9 (27.1 to 45.3)
Ischemic heart disease	Scotland	2000	All Ages	1.5 (1.1 to 1.9)	10.9% (8.0 to 13.8%)	29.5 (21.5 to 37.5)
Ischemic heart disease	Scotland	2005	All Ages	1.2 (0.9 to 1.5)	10.6% (7.6 to 13.5%)	23.5 (16.8 to 30.3)
Ischemic heart disease	Scotland	2010	All Ages	1.0 (0.7 to 1.3)	10.3% (7.2 to 13.4%)	19.2 (13.4 to 25.3)
Ischemic heart disease	Scotland	2015	All Ages	1.0 (0.7 to 1.3)	10.4% (7.1 to 13.7%)	18.9 (12.9 to 24.7)
Ischemic heart disease	Scotland	1990	Age-standardized		12.4% (9.6 to 15.2%)	30.4 (23.5 to 37.5)
Ischemic heart disease	Scotland	1995	Age-standardized		11.8% (9.0 to 14.6%)	25.0 (19.2 to 31.2)
Ischemic heart disease	Scotland	2000	Age-standardized		11.2% (8.4 to 14.1%)	19.6 (14.7 to 24.5)
Ischemic heart disease	Scotland	2005	Age-standardized		10.9% (8.1 to 13.7%)	14.6 (10.8 to 18.6)
Ischemic heart disease	Scotland	2010	Age-standardized		10.7% (7.9 to 13.6%)	11.3 (8.3 to 14.5)
Ischemic heart disease	Scotland	2015	Age-standardized		10.8% (7.8 to 13.8%)	10.5 (7.6 to 13.4)
Ischemic stroke	Scotland	1990	All Ages	0.3 (0.2 to 0.5)	7.7% (4.4 to 11.6%)	6.5 (3.7 to 9.9)
Ischemic stroke	Scotland	1995	All Ages	0.3 (0.2 to 0.5)	7.3% (3.9 to 11.5%)	5.7 (3.1 to 9.1)
Ischemic stroke	Scotland	2000	All Ages	0.3 (0.1 to 0.4)	6.9% (3.6 to 11.4%)	5.1 (2.6 to 8.3)
Ischemic stroke	Scotland	2005	All Ages	0.2 (0.1 to 0.4)	6.7% (3.2 to 11.2%)	4.4 (2.2 to 7.6)
Ischemic stroke	Scotland	2010	All Ages	0.2 (0.1 to 0.4)	6.5% (3.0 to 11.3%)	3.8 (1.8 to 6.7)
Ischemic stroke	Scotland	2015	All Ages	0.2 (0.1 to 0.4)	6.6% (2.9 to 11.5%)	3.7 (1.7 to 6.5)
Ischemic stroke	Scotland	1990	Age-standardized		7.8% (4.5 to 11.7%)	4.4 (2.5 to 6.6)
Ischemic stroke	Scotland	1995	Age-standardized		7.4% (4.1 to 11.4%)	3.7 (2.0 to 5.7)
Ischemic stroke	Scotland	2000	Age-standardized		7.0% (3.7 to 11.3%)	3.1 (1.6 to 5.1)
Ischemic stroke	Scotland	2005	Age-standardized		6.8% (3.4 to 11.1%)	2.5 (1.3 to 4.2)
Ischemic stroke	Scotland	2010	Age-standardized		6.6% (3.2 to 11.1%)	2.0 (1.0 to 3.4)
Ischemic stroke	Scotland	2015	Age-standardized		6.7% (3.2 to 11.3%)	1.9 (0.9 to 3.2)
Hemorrhagic stroke	Scotland	1990	All Ages	0.3 (0.2 to 0.4)	8.5% (5.7 to 11.3%)	5.8 (3.8 to 7.9)
Hemorrhagic stroke	Scotland	1995	All Ages	0.3 (0.2 to 0.4)	8.0% (5.1 to 11.2%)	5.2 (3.3 to 7.4)
Hemorrhagic stroke	Scotland	2000	All Ages	0.2 (0.1 to 0.3)	7.6% (4.7 to 11.1%)	4.6 (2.8 to 6.8)
Hemorrhagic stroke	Scotland	2005	All Ages	0.2 (0.1 to 0.3)	7.3% (4.3 to 10.9%)	4.0 (2.4 to 6.1)
Hemorrhagic stroke	Scotland	2010	All Ages	0.2 (0.1 to 0.3)	7.1% (4.0 to 10.9%)	3.4 (2.0 to 5.3)
Hemorrhagic stroke	Scotland	2015	All Ages	0.2 (0.1 to 0.3)	7.1% (4.1 to 11.2%)	3.3 (1.9 to 5.2)
Hemorrhagic stroke	Scotland	1990	Age-standardized		8.7% (5.9 to 11.4%)	4.2 (2.8 to 5.5)
Hemorrhagic stroke	Scotland	1995	Age-standardized		8.2% (5.4 to 11.2%)	3.6 (2.4 to 4.9)
Hemorrhagic stroke	Scotland	2000	Age-standardized		7.8% (5.0 to 11.1%)	3.1 (2.0 to 4.4)
Hemorrhagic stroke	Scotland	2005	Age-standardized		7.5% (4.7 to 10.9%)	2.5 (1.5 to 3.6)
Hemorrhagic stroke	Scotland	2010	Age-standardized		7.4% (4.5 to 10.8%)	2.0 (1.2 to 3.0)

Cause name	Location	Year	Age group	Number (in thousands)	PAF	Rate (per 100,000)
Hemorrhagic stroke	Scotland	2015	Age-standardized		7.4% (4.5 to 11.0%)	1.8 (1.1 to 2.7)
Chronic obstructive pulmonary disease	Scotland	1990	All Ages	0.4 (0.1 to 0.7)	13.0% (5.0 to 22.9%)	7.3 (2.8 to 12.8)
Chronic obstructive pulmonary disease	Scotland	1995	All Ages	0.4 (0.1 to 0.7)	12.2% (4.4 to 22.1%)	7.5 (2.6 to 13.5)
Chronic obstructive pulmonary disease	Scotland	2000	All Ages	0.4 (0.1 to 0.8)	11.5% (4.0 to 21.3%)	8.1 (2.7 to 15.1)
Chronic obstructive pulmonary disease	Scotland	2005	All Ages	0.4 (0.1 to 0.8)	11.1% (3.5 to 20.6%)	7.9 (2.5 to 15.0)
Chronic obstructive pulmonary disease	Scotland	2010	All Ages	0.4 (0.1 to 0.8)	10.8% (3.4 to 20.3%)	7.5 (2.4 to 14.5)
Chronic obstructive pulmonary disease	Scotland	2015	All Ages	0.4 (0.1 to 0.8)	11.1% (3.8 to 20.6%)	7.9 (2.7 to 14.7)
Chronic obstructive pulmonary disease	Scotland	1990	Age-standardized		13.0% (5.0 to 22.9%)	5.0 (1.9 to 8.8)
Chronic obstructive pulmonary disease	Scotland	1995	Age-standardized		12.2% (4.3 to 22.1%)	5.0 (1.7 to 9.0)
Chronic obstructive pulmonary disease	Scotland	2000	Age-standardized		11.5% (4.0 to 21.3%)	5.1 (1.7 to 9.6)
Chronic obstructive pulmonary disease	Scotland	2005	Age-standardized		11.1% (3.5 to 20.6%)	4.7 (1.5 to 8.8)
Chronic obstructive pulmonary disease	Scotland	2010	Age-standardized		10.8% (3.4 to 20.3%)	4.2 (1.3 to 8.0)
Chronic obstructive pulmonary disease	Scotland	2015	Age-standardized		11.1% (3.8 to 20.6%)	4.1 (1.4 to 7.7)
All causes	Wales	1990	All Ages	2.1 (1.7 to 2.5)	6.1% (4.9 to 7.3%)	73.6 (59.3 to 88.2)
All causes	Wales	1995	All Ages	2.0 (1.6 to 2.4)	5.7% (4.5 to 7.0%)	68.5 (54.2 to 84.0)
All causes	Wales	2000	All Ages	1.8 (1.4 to 2.2)	5.2% (4.1 to 6.5%)	61.2 (47.5 to 76.7)
All causes	Wales	2005	All Ages	1.5 (1.2 to 2.0)	4.6% (3.5 to 5.9%)	52.2 (39.5 to 66.3)
All causes	Wales	2010	All Ages	1.4 (1.1 to 1.8)	4.3% (3.2 to 5.5%)	46.0 (34.6 to 59.0)
All causes	Wales	2015	All Ages	1.5 (1.1 to 1.9)	4.4% (3.3 to 5.7%)	47.9 (35.9 to 62.0)
All causes	Wales	1990	Age-standardized		6.0% (4.9 to 7.2%)	46.8 (38.3 to 55.8)
All causes	Wales	1995	Age-standardized		5.6% (4.5 to 6.8%)	41.2 (33.3 to 50.0)
All causes	Wales	2000	Age-standardized		5.1% (4.0 to 6.3%)	35.5 (28.0 to 43.8)
All causes	Wales	2005	Age-standardized		4.5% (3.5 to 5.6%)	28.6 (22.3 to 35.6)
All causes	Wales	2010	Age-standardized		4.2% (3.2 to 5.2%)	24.0 (18.6 to 30.3)
All causes	Wales	2015	Age-standardized		4.3% (3.3 to 5.4%)	23.6 (18.2 to 30.0)
Lower respiratory infections	Wales	1990	All Ages	0.2 (0.1 to 0.5)	11.3% (3.5 to 22.3%)	7.8 (2.4 to 15.6)
Lower respiratory infections	Wales	1995	All Ages	0.3 (0.1 to 0.6)	10.6% (3.2 to 21.7%)	9.3 (2.8 to 19.0)
Lower respiratory infections	Wales	2000	All Ages	0.3 (0.1 to 0.6)	10.2% (3.0 to 21.4%)	9.0 (2.6 to 18.9)
Lower respiratory infections	Wales	2005	All Ages	0.2 (0.1 to 0.5)	9.7% (2.6 to 20.7%)	7.3 (1.9 to 15.8)
Lower respiratory infections	Wales	2010	All Ages	0.2 (0.1 to 0.5)	9.5% (2.4 to 20.6%)	6.7 (1.8 to 14.9)
Lower respiratory infections	Wales	2015	All Ages	0.2 (0.1 to 0.5)	10.0% (2.7 to 21.2%)	7.3 (2.1 to 15.9)
Lower respiratory infections	Wales	1990	Age-standardized		11.3% (3.5 to 22.3%)	4.8 (1.5 to 9.5)
Lower respiratory infections	Wales	1995	Age-standardized		10.6% (3.2 to 21.7%)	5.2 (1.5 to 10.6)
Lower respiratory infections	Wales	2000	Age-standardized		10.2% (3.0 to 21.4%)	4.8 (1.4 to 10.2)
Lower respiratory infections	Wales	2005	Age-standardized		9.7% (2.6 to 20.7%)	3.7 (1.0 to 7.9)
Lower respiratory infections	Wales	2010	Age-standardized		9.5% (2.4 to 20.6%)	3.2 (0.8 to 7.1)

Cause name	Location	Year	Age group	Number (in thousands)	PAF	Rate (per 100,000)
Lower respiratory infections	Wales	2015	Age-standardized		10.0% (2.7 to 21.2%)	3.3 (0.9 to 7.1)
Tracheal, bronchus, and lung cancer	Wales	1990	All Ages	0.2 (0.1 to 0.3)	7.8% (3.7 to 13.0%)	5.4 (2.6 to 9.0)
Tracheal, bronchus, and lung cancer	Wales	1995	All Ages	0.1 (0.1 to 0.2)	7.3% (3.4 to 12.2%)	4.9 (2.3 to 8.2)
Tracheal, bronchus, and lung cancer	Wales	2000	All Ages	0.1 (0.1 to 0.2)	7.0% (3.2 to 12.0%)	4.6 (2.2 to 7.9)
Tracheal, bronchus, and lung cancer	Wales	2005	All Ages	0.1 (0.1 to 0.2)	6.6% (3.0 to 11.4%)	4.6 (2.0 to 7.8)
Tracheal, bronchus, and lung cancer	Wales	2010	All Ages	0.1 (0.1 to 0.2)	6.5% (2.9 to 11.1%)	4.4 (2.0 to 7.6)
Tracheal, bronchus, and lung cancer	Wales	2015	All Ages	0.1 (0.1 to 0.3)	6.8% (3.1 to 11.7%)	4.7 (2.1 to 8.1)
Tracheal, bronchus, and lung cancer	Wales	1990	Age-standardized		7.8% (3.7 to 13.0%)	3.6 (1.7 to 5.9)
Tracheal, bronchus, and lung cancer	Wales	1995	Age-standardized		7.3% (3.4 to 12.2%)	3.1 (1.5 to 5.2)
Tracheal, bronchus, and lung cancer	Wales	2000	Age-standardized		7.0% (3.2 to 12.0%)	2.9 (1.3 to 4.9)
Tracheal, bronchus, and lung cancer	Wales	2005	Age-standardized		6.6% (3.0 to 11.4%)	2.7 (1.2 to 4.7)
Tracheal, bronchus, and lung cancer	Wales	2010	Age-standardized		6.5% (2.9 to 11.1%)	2.5 (1.1 to 4.3)
Tracheal, bronchus, and lung cancer	Wales	2015	Age-standardized		6.8% (3.1 to 11.7%)	2.5 (1.1 to 4.3)
Cardiovascular diseases	Wales	1990	All Ages	1.5 (1.2 to 1.8)	9.6% (7.7 to 11.6%)	52.0 (41.5 to 63.2)
Cardiovascular diseases	Wales	1995	All Ages	1.3 (1.0 to 1.6)	8.9% (6.9 to 11.0%)	45.8 (35.4 to 56.9)
Cardiovascular diseases	Wales	2000	All Ages	1.1 (0.8 to 1.4)	8.3% (6.3 to 10.4%)	38.9 (29.1 to 48.7)
Cardiovascular diseases	Wales	2005	All Ages	1.0 (0.7 to 1.2)	7.8% (5.8 to 9.9%)	32.0 (23.7 to 41.2)
Cardiovascular diseases	Wales	2010	All Ages	0.8 (0.6 to 1.1)	7.5% (5.5 to 9.7%)	26.8 (19.6 to 34.6)
Cardiovascular diseases	Wales	2015	All Ages	0.8 (0.6 to 1.1)	7.6% (5.5 to 9.8%)	27.0 (19.6 to 35.0)
Cardiovascular diseases	Wales	1990	Age-standardized		9.9% (7.9 to 11.9%)	33.4 (26.9 to 40.1)
Cardiovascular diseases	Wales	1995	Age-standardized		9.2% (7.3 to 11.2%)	28.0 (22.2 to 34.2)
Cardiovascular diseases	Wales	2000	Age-standardized		8.7% (6.7 to 10.6%)	23.0 (17.8 to 28.2)
Cardiovascular diseases	Wales	2005	Age-standardized		8.2% (6.3 to 10.1%)	17.9 (13.7 to 22.3)
Cardiovascular diseases	Wales	2010	Age-standardized		7.9% (6.0 to 9.8%)	14.2 (10.9 to 17.8)
Cardiovascular diseases	Wales	2015	Age-standardized		7.9% (6.1 to 9.9%)	13.6 (10.4 to 17.0)
Ischemic heart disease	Wales	1990	All Ages	1.2 (0.9 to 1.5)	12.1% (9.1 to 15.0%)	40.7 (30.8 to 50.7)
Ischemic heart disease	Wales	1995	All Ages	1.0 (0.8 to 1.3)	11.5% (8.5 to 14.6%)	35.8 (26.4 to 45.6)
Ischemic heart disease	Wales	2000	All Ages	0.9 (0.6 to 1.1)	11.1% (8.0 to 14.2%)	29.8 (21.5 to 38.2)
Ischemic heart disease	Wales	2005	All Ages	0.7 (0.5 to 0.9)	10.8% (7.5 to 14.0%)	23.9 (16.4 to 31.5)
Ischemic heart disease	Wales	2010	All Ages	0.6 (0.4 to 0.8)	10.6% (7.3 to 14.0%)	19.8 (13.4 to 26.4)
Ischemic heart disease	Wales	2015	All Ages	0.6 (0.4 to 0.8)	10.8% (7.3 to 14.3%)	20.1 (13.6 to 26.9)
Ischemic heart disease	Wales	1990	Age-standardized		12.5% (9.6 to 15.3%)	26.4 (20.4 to 32.3)
Ischemic heart disease	Wales	1995	Age-standardized		12.0% (9.1 to 14.8%)	22.1 (16.9 to 27.5)
Ischemic heart disease	Wales	2000	Age-standardized		11.6% (8.7 to 14.5%)	17.8 (13.2 to 22.3)
Ischemic heart disease	Wales	2005	Age-standardized		11.3% (8.2 to 14.2%)	13.5 (9.9 to 17.1)
Ischemic heart disease	Wales	2010	Age-standardized		11.2% (8.2 to 14.2%)	10.7 (7.8 to 13.7)

Cause name	Location	Year	Age group	Number (in thousands)	PAF	Rate (per 100,000)
Ischemic heart disease	Wales	2015	Age-standardized		11.3% (8.2 to 14.4%)	10.3 (7.5 to 13.2)
Ischemic stroke	Wales	1990	All Ages	0.2 (0.1 to 0.3)	7.8% (4.5 to 11.9%)	6.2 (3.5 to 9.5)
Ischemic stroke	Wales	1995	All Ages	0.2 (0.1 to 0.3)	7.5% (4.0 to 11.9%)	5.4 (2.9 to 8.6)
Ischemic stroke	Wales	2000	All Ages	0.1 (0.1 to 0.2)	7.2% (3.7 to 11.8%)	4.9 (2.5 to 8.0)
Ischemic stroke	Wales	2005	All Ages	0.1 (0.1 to 0.2)	7.0% (3.3 to 11.9%)	4.4 (2.1 to 7.5)
Ischemic stroke	Wales	2010	All Ages	0.1 (0.1 to 0.2)	6.9% (3.2 to 11.8%)	3.7 (1.7 to 6.5)
Ischemic stroke	Wales	2015	All Ages	0.1 (0.1 to 0.2)	7.0% (3.2 to 12.0%)	3.6 (1.7 to 6.3)
Ischemic stroke	Wales	1990	Age-standardized		7.9% (4.6 to 12.0%)	3.7 (2.1 to 5.7)
Ischemic stroke	Wales	1995	Age-standardized		7.6% (4.2 to 11.8%)	3.0 (1.7 to 4.7)
Ischemic stroke	Wales	2000	Age-standardized		7.4% (3.9 to 11.6%)	2.6 (1.4 to 4.2)
Ischemic stroke	Wales	2005	Age-standardized		7.1% (3.7 to 11.6%)	2.2 (1.1 to 3.6)
Ischemic stroke	Wales	2010	Age-standardized		7.0% (3.6 to 11.6%)	1.8 (0.9 to 3.0)
Ischemic stroke	Wales	2015	Age-standardized		7.2% (3.6 to 11.8%)	1.6 (0.8 to 2.7)
Hemorrhagic stroke	Wales	1990	All Ages	0.1 (0.1 to 0.2)	8.6% (5.7 to 11.7%)	5.1 (3.3 to 6.9)
Hemorrhagic stroke	Wales	1995	All Ages	0.1 (0.1 to 0.2)	8.2% (5.2 to 11.5%)	4.6 (2.9 to 6.7)
Hemorrhagic stroke	Wales	2000	All Ages	0.1 (0.1 to 0.2)	7.9% (4.9 to 11.5%)	4.2 (2.6 to 6.2)
Hemorrhagic stroke	Wales	2005	All Ages	0.1 (0.1 to 0.2)	7.6% (4.5 to 11.5%)	3.7 (2.2 to 5.7)
Hemorrhagic stroke	Wales	2010	All Ages	0.1 (0.1 to 0.1)	7.5% (4.3 to 11.4%)	3.3 (1.9 to 5.0)
Hemorrhagic stroke	Wales	2015	All Ages	0.1 (0.1 to 0.1)	7.6% (4.3 to 11.7%)	3.3 (1.8 to 5.0)
Hemorrhagic stroke	Wales	1990	Age-standardized		8.9% (6.1 to 11.8%)	3.3 (2.2 to 4.4)
Hemorrhagic stroke	Wales	1995	Age-standardized		8.5% (5.7 to 11.6%)	2.8 (1.9 to 3.9)
Hemorrhagic stroke	Wales	2000	Age-standardized		8.3% (5.4 to 11.5%)	2.5 (1.7 to 3.5)
Hemorrhagic stroke	Wales	2005	Age-standardized		8.0% (5.1 to 11.4%)	2.1 (1.4 to 3.1)
Hemorrhagic stroke	Wales	2010	Age-standardized		7.9% (5.0 to 11.4%)	1.8 (1.1 to 2.6)
Hemorrhagic stroke	Wales	2015	Age-standardized		8.0% (5.1 to 11.7%)	1.7 (1.0 to 2.4)
Chronic obstructive pulmonary disease	Wales	1990	All Ages	0.2 (0.1 to 0.4)	13.5% (5.3 to 23.4%)	8.3 (3.3 to 14.4)
Chronic obstructive pulmonary disease	Wales	1995	All Ages	0.2 (0.1 to 0.4)	12.9% (4.8 to 22.9%)	8.5 (3.2 to 15.1)
Chronic obstructive pulmonary disease	Wales	2000	All Ages	0.3 (0.1 to 0.5)	12.5% (4.6 to 22.5%)	8.7 (3.2 to 15.6)
Chronic obstructive pulmonary disease	Wales	2005	All Ages	0.2 (0.1 to 0.5)	12.0% (4.2 to 21.8%)	8.2 (2.8 to 15.2)
Chronic obstructive pulmonary disease	Wales	2010	All Ages	0.2 (0.1 to 0.5)	11.8% (4.1 to 21.6%)	8.1 (2.8 to 15.0)
Chronic obstructive pulmonary disease	Wales	2015	All Ages	0.3 (0.1 to 0.5)	12.3% (4.5 to 22.2%)	8.8 (3.2 to 16.2)
Chronic obstructive pulmonary disease	Wales	1990	Age-standardized		13.5% (5.3 to 23.4%)	5.1 (2.0 to 8.8)
Chronic obstructive pulmonary disease	Wales	1995	Age-standardized		12.8% (4.8 to 22.8%)	4.9 (1.9 to 8.8)
Chronic obstructive pulmonary disease	Wales	2000	Age-standardized		12.4% (4.6 to 22.5%)	4.8 (1.8 to 8.7)
Chronic obstructive pulmonary disease	Wales	2005	Age-standardized		12.0% (4.2 to 21.8%)	4.4 (1.5 to 8.0)
Chronic obstructive pulmonary disease	Wales	2010	Age-standardized		11.8% (4.1 to 21.6%)	4.1 (1.4 to 7.6)

Cause name	Location	Year	Age group	Number (in thousands)	PAF	Rate (per 100,000)
Chronic obstructive pulmonary disease	Wales	2015	Age-standardized		12.3% (4.5 to 22.2%)	4.2 (1.5 to 7.7)
All causes	Argentina	1990	All Ages	13.5 (11.4 to 15.8)	5.4% (4.5 to 6.3%)	41.3 (34.8 to 48.3)
All causes	Argentina	1995	All Ages	13.8 (11.5 to 16.3)	5.3% (4.4 to 6.2%)	39.5 (32.9 to 46.5)
All causes	Argentina	2000	All Ages	15.0 (12.2 to 18.0)	5.3% (4.3 to 6.4%)	40.4 (32.9 to 48.6)
All causes	Argentina	2005	All Ages	15.4 (12.4 to 19.0)	5.2% (4.2 to 6.4%)	39.3 (31.7 to 48.5)
All causes	Argentina	2010	All Ages	16.2 (12.9 to 20.3)	5.1% (4.0 to 6.3%)	39.3 (31.3 to 49.1)
All causes	Argentina	2015	All Ages	16.6 (12.9 to 21.0)	4.9% (3.8 to 6.2%)	38.2 (29.7 to 48.4)
All causes	Argentina	1990	Age-standardized		5.5% (4.6 to 6.4%)	48.0 (40.0 to 56.6)
All causes	Argentina	1995	Age-standardized		5.4% (4.5 to 6.3%)	44.0 (36.5 to 52.1)
All causes	Argentina	2000	Age-standardized		5.4% (4.4 to 6.5%)	42.8 (34.9 to 51.5)
All causes	Argentina	2005	Age-standardized		5.2% (4.2 to 6.4%)	39.2 (31.6 to 48.2)
All causes	Argentina	2010	Age-standardized		5.1% (4.1 to 6.3%)	37.0 (29.7 to 46.1)
All causes	Argentina	2015	Age-standardized		4.8% (3.8 to 6.1%)	34.3 (26.9 to 43.2)
Lower respiratory infections	Argentina	1990	All Ages	1.4 (0.6 to 2.4)	13.7% (5.6 to 24.3%)	4.2 (1.7 to 7.3)
Lower respiratory infections	Argentina	1995	All Ages	1.7 (0.7 to 3.1)	13.9% (5.8 to 24.4%)	4.9 (2.0 to 8.8)
Lower respiratory infections	Argentina	2000	All Ages	2.5 (1.0 to 4.5)	13.5% (5.5 to 24.2%)	6.8 (2.7 to 12.2)
Lower respiratory infections	Argentina	2005	All Ages	3.2 (1.2 to 5.9)	12.9% (4.9 to 23.6%)	8.2 (3.1 to 15.1)
Lower respiratory infections	Argentina	2010	All Ages	3.6 (1.3 to 6.8)	12.4% (4.5 to 23.1%)	8.8 (3.2 to 16.4)
Lower respiratory infections	Argentina	2015	All Ages	3.6 (1.3 to 7.0)	11.7% (4.0 to 22.4%)	8.4 (2.9 to 16.1)
Lower respiratory infections	Argentina	1990	Age-standardized		13.7% (5.6 to 24.3%)	4.8 (2.0 to 8.4)
Lower respiratory infections	Argentina	1995	Age-standardized		13.9% (5.8 to 24.4%)	5.5 (2.3 to 9.8)
Lower respiratory infections	Argentina	2000	Age-standardized		13.5% (5.5 to 24.2%)	7.1 (2.9 to 12.8)
Lower respiratory infections	Argentina	2005	Age-standardized		12.9% (4.9 to 23.6%)	8.1 (3.0 to 14.8)
Lower respiratory infections	Argentina	2010	Age-standardized		12.4% (4.5 to 23.1%)	8.1 (3.0 to 15.2)
Lower respiratory infections	Argentina	2015	Age-standardized		11.7% (4.0 to 22.4%)	7.4 (2.5 to 14.1)
Tracheal, bronchus, and lung cancer	Argentina	1990	All Ages	0.8 (0.4 to 1.3)	9.5% (4.9 to 15.3%)	2.5 (1.3 to 4.0)
Tracheal, bronchus, and lung cancer	Argentina	1995	All Ages	0.9 (0.5 to 1.4)	9.6% (4.9 to 15.2%)	2.5 (1.3 to 4.0)
Tracheal, bronchus, and lung cancer	Argentina	2000	All Ages	0.9 (0.5 to 1.4)	9.3% (4.7 to 15.0%)	2.4 (1.2 to 3.9)
Tracheal, bronchus, and lung cancer	Argentina	2005	All Ages	0.9 (0.4 to 1.4)	8.9% (4.5 to 14.3%)	2.2 (1.1 to 3.6)
Tracheal, bronchus, and lung cancer	Argentina	2010	All Ages	0.9 (0.5 to 1.5)	8.5% (4.2 to 13.9%)	2.2 (1.1 to 3.6)
Tracheal, bronchus, and lung cancer	Argentina	2015	All Ages	0.9 (0.5 to 1.5)	8.0% (3.9 to 13.3%)	2.2 (1.1 to 3.5)
Tracheal, bronchus, and lung cancer	Argentina	1990	Age-standardized		9.5% (4.9 to 15.3%)	2.7 (1.4 to 4.4)
Tracheal, bronchus, and lung cancer	Argentina	1995	Age-standardized		9.6% (4.9 to 15.2%)	2.7 (1.4 to 4.3)
Tracheal, bronchus, and lung cancer	Argentina	2000	Age-standardized		9.3% (4.7 to 15.0%)	2.5 (1.3 to 4.1)
Tracheal, bronchus, and lung cancer	Argentina	2005	Age-standardized		8.9% (4.5 to 14.3%)	2.3 (1.1 to 3.7)
Tracheal, bronchus, and lung cancer	Argentina	2010	Age-standardized		8.5% (4.2 to 13.9%)	2.2 (1.1 to 3.5)

Cause name	Location	Year	Age group	Number (in thousands)	PAF	Rate (per 100,000)
Tracheal, bronchus, and lung cancer	Argentina	2015	Age-standardized		8.0% (3.9 to 13.3%)	2.0 (1.0 to 3.4)
Cardiovascular diseases	Argentina	1990	All Ages	10.0 (8.2 to 11.8)	9.7% (8.0 to 11.5%)	30.6 (25.1 to 36.2)
Cardiovascular diseases	Argentina	1995	All Ages	9.6 (7.8 to 11.4)	9.5% (7.8 to 11.3%)	27.3 (22.4 to 32.5)
Cardiovascular diseases	Argentina	2000	All Ages	9.5 (7.7 to 11.3)	9.4% (7.6 to 11.1%)	25.6 (20.7 to 30.5)
Cardiovascular diseases	Argentina	2005	All Ages	9.1 (7.3 to 11.1)	8.9% (7.1 to 10.8%)	23.3 (18.6 to 28.3)
Cardiovascular diseases	Argentina	2010	All Ages	9.3 (7.2 to 11.4)	8.5% (6.7 to 10.4%)	22.6 (17.6 to 27.7)
Cardiovascular diseases	Argentina	2015	All Ages	9.6 (7.4 to 12.0)	8.2% (6.3 to 10.2%)	22.1 (17.0 to 27.5)
Cardiovascular diseases	Argentina	1990	Age-standardized		9.5% (7.8 to 11.4%)	35.6 (29.0 to 42.5)
Cardiovascular diseases	Argentina	1995	Age-standardized		9.4% (7.7 to 11.2%)	30.4 (24.6 to 36.3)
Cardiovascular diseases	Argentina	2000	Age-standardized		9.4% (7.5 to 11.2%)	27.1 (21.8 to 32.5)
Cardiovascular diseases	Argentina	2005	Age-standardized		9.0% (7.2 to 10.9%)	23.4 (18.7 to 28.3)
Cardiovascular diseases	Argentina	2010	Age-standardized		8.7% (6.9 to 10.5%)	21.4 (16.9 to 26.1)
Cardiovascular diseases	Argentina	2015	Age-standardized		8.4% (6.6 to 10.3%)	20.0 (15.7 to 24.5)
Ischemic heart disease	Argentina	1990	All Ages	7.1 (5.5 to 8.7)	13.4% (10.4 to 16.4%)	21.8 (16.9 to 26.6)
Ischemic heart disease	Argentina	1995	All Ages	6.8 (5.2 to 8.3)	13.4% (10.3 to 16.3%)	19.4 (14.9 to 23.8)
Ischemic heart disease	Argentina	2000	All Ages	6.9 (5.3 to 8.6)	13.0% (9.9 to 16.0%)	18.7 (14.2 to 23.1)
Ischemic heart disease	Argentina	2005	All Ages	6.8 (5.0 to 8.5)	12.5% (9.4 to 15.7%)	17.3 (12.9 to 21.8)
Ischemic heart disease	Argentina	2010	All Ages	7.1 (5.2 to 9.1)	12.2% (8.9 to 15.4%)	17.2 (12.5 to 22.0)
Ischemic heart disease	Argentina	2015	All Ages	7.4 (5.3 to 9.5)	11.8% (8.4 to 15.0%)	17.0 (12.1 to 21.9)
Ischemic heart disease	Argentina	1990	Age-standardized		13.1% (10.0 to 16.1%)	25.5 (19.4 to 31.6)
Ischemic heart disease	Argentina	1995	Age-standardized		13.2% (10.0 to 16.3%)	21.7 (16.4 to 26.8)
Ischemic heart disease	Argentina	2000	Age-standardized		13.0% (9.9 to 16.1%)	19.8 (15.0 to 24.6)
Ischemic heart disease	Argentina	2005	Age-standardized		12.7% (9.6 to 15.8%)	17.3 (13.0 to 21.8)
Ischemic heart disease	Argentina	2010	Age-standardized		12.4% (9.3 to 15.4%)	16.3 (12.1 to 20.5)
Ischemic heart disease	Argentina	2015	Age-standardized		12.0% (8.8 to 15.1%)	15.4 (11.3 to 19.5)
Ischemic stroke	Argentina	1990	All Ages	1.8 (1.2 to 2.3)	9.7% (6.9 to 12.7%)	5.3 (3.8 to 7.0)
Ischemic stroke	Argentina	1995	All Ages	1.6 (1.1 to 2.1)	9.6% (6.6 to 12.6%)	4.6 (3.2 to 6.1)
Ischemic stroke	Argentina	2000	All Ages	1.3 (0.8 to 1.8)	9.0% (5.8 to 12.4%)	3.4 (2.2 to 4.7)
Ischemic stroke	Argentina	2005	All Ages	1.1 (0.7 to 1.7)	8.6% (5.3 to 12.5%)	2.9 (1.8 to 4.2)
Ischemic stroke	Argentina	2010	All Ages	1.1 (0.6 to 1.7)	8.3% (4.9 to 12.3%)	2.6 (1.5 to 4.0)
Ischemic stroke	Argentina	2015	All Ages	1.1 (0.6 to 1.7)	8.0% (4.6 to 12.3%)	2.5 (1.4 to 3.8)
Ischemic stroke	Argentina	1990	Age-standardized		9.5% (6.5 to 12.7%)	6.3 (4.3 to 8.4)
Ischemic stroke	Argentina	1995	Age-standardized		9.5% (6.5 to 12.7%)	5.1 (3.5 to 6.9)
Ischemic stroke	Argentina	2000	Age-standardized		9.0% (5.7 to 12.6%)	3.6 (2.3 to 5.0)
Ischemic stroke	Argentina	2005	Age-standardized		8.6% (5.3 to 12.5%)	2.9 (1.8 to 4.1)
Ischemic stroke	Argentina	2010	Age-standardized		8.4% (5.1 to 12.3%)	2.4 (1.5 to 3.6)

Cause name	Location	Year	Age group	Number (in thousands)	PAF	Rate (per 100,000)
Ischemic stroke	Argentina	2015	Age-standardized		8.1% (4.8 to 12.1%)	2.2 (1.3 to 3.3)
Hemorrhagic stroke	Argentina	1990	All Ages	1.1 (0.8 to 1.4)	11.1% (8.4 to 13.7%)	3.4 (2.6 to 4.3)
Hemorrhagic stroke	Argentina	1995	All Ages	1.2 (0.9 to 1.5)	11.1% (8.4 to 13.9%)	3.3 (2.5 to 4.2)
Hemorrhagic stroke	Argentina	2000	All Ages	1.3 (1.0 to 1.7)	10.8% (8.1 to 13.6%)	3.6 (2.7 to 4.5)
Hemorrhagic stroke	Argentina	2005	All Ages	1.2 (0.9 to 1.6)	10.3% (7.7 to 13.0%)	3.1 (2.3 to 4.0)
Hemorrhagic stroke	Argentina	2010	All Ages	1.1 (0.8 to 1.5)	9.9% (7.3 to 12.7%)	2.7 (2.0 to 3.6)
Hemorrhagic stroke	Argentina	2015	All Ages	1.1 (0.8 to 1.5)	9.5% (6.8 to 12.3%)	2.6 (1.8 to 3.4)
Hemorrhagic stroke	Argentina	1990	Age-standardized		10.9% (8.3 to 13.6%)	3.9 (2.9 to 4.8)
Hemorrhagic stroke	Argentina	1995	Age-standardized		11.0% (8.4 to 13.8%)	3.7 (2.8 to 4.6)
Hemorrhagic stroke	Argentina	2000	Age-standardized		10.8% (8.1 to 13.6%)	3.8 (2.8 to 4.8)
Hemorrhagic stroke	Argentina	2005	Age-standardized		10.4% (7.7 to 13.2%)	3.2 (2.4 to 4.1)
Hemorrhagic stroke	Argentina	2010	Age-standardized		10.1% (7.4 to 12.8%)	2.7 (2.0 to 3.5)
Hemorrhagic stroke	Argentina	2015	Age-standardized		9.7% (7.0 to 12.5%)	2.4 (1.7 to 3.2)
Chronic obstructive pulmonary disease	Argentina	1990	All Ages	1.3 (0.6 to 2.2)	15.4% (6.8 to 25.8%)	4.1 (1.8 to 6.8)
Chronic obstructive pulmonary disease	Argentina	1995	All Ages	1.7 (0.7 to 2.8)	15.5% (7.0 to 25.9%)	4.8 (2.1 to 7.9)
Chronic obstructive pulmonary disease	Argentina	2000	All Ages	2.1 (0.9 to 3.5)	15.3% (6.6 to 25.7%)	5.6 (2.4 to 9.5)
Chronic obstructive pulmonary disease	Argentina	2005	All Ages	2.2 (0.9 to 3.7)	14.8% (6.4 to 25.0%)	5.6 (2.4 to 9.6)
Chronic obstructive pulmonary disease	Argentina	2010	All Ages	2.4 (1.0 to 4.0)	14.3% (6.0 to 24.6%)	5.7 (2.4 to 9.8)
Chronic obstructive pulmonary disease	Argentina	2015	All Ages	2.4 (1.0 to 4.1)	13.7% (5.7 to 23.9%)	5.5 (2.2 to 9.5)
Chronic obstructive pulmonary disease	Argentina	1990	Age-standardized		15.4% (6.8 to 25.9%)	4.9 (2.1 to 8.1)
Chronic obstructive pulmonary disease	Argentina	1995	Age-standardized		15.6% (7.0 to 25.9%)	5.4 (2.4 to 9.0)
Chronic obstructive pulmonary disease	Argentina	2000	Age-standardized		15.3% (6.7 to 25.7%)	5.9 (2.6 to 10.0)
Chronic obstructive pulmonary disease	Argentina	2005	Age-standardized		14.8% (6.4 to 25.0%)	5.5 (2.3 to 9.4)
Chronic obstructive pulmonary disease	Argentina	2010	Age-standardized		14.3% (6.0 to 24.6%)	5.3 (2.2 to 9.0)
Chronic obstructive pulmonary disease	Argentina	2015	Age-standardized		13.7% (5.7 to 23.9%)	4.8 (2.0 to 8.3)
All causes	Chile	1990	All Ages	4.8 (4.0 to 5.8)	5.9% (4.8 to 7.0%)	36.7 (30.2 to 43.9)
All causes	Chile	1995	All Ages	4.5 (3.8 to 5.4)	5.7% (4.7 to 6.8%)	31.9 (26.4 to 38.0)
All causes	Chile	2000	All Ages	4.2 (3.5 to 5.0)	5.2% (4.4 to 6.2%)	27.8 (23.2 to 33.1)
All causes	Chile	2005	All Ages	4.6 (3.8 to 5.4)	5.1% (4.2 to 6.0%)	28.3 (23.6 to 33.5)
All causes	Chile	2010	All Ages	5.2 (4.3 to 6.3)	4.8% (3.9 to 5.8%)	30.5 (25.0 to 36.8)
All causes	Chile	2015	All Ages	5.9 (4.9 to 7.1)	4.8% (3.9 to 5.8%)	32.9 (27.0 to 39.5)
All causes	Chile	1990	Age-standardized		6.2% (5.1 to 7.5%)	55.7 (45.7 to 66.3)
All causes	Chile	1995	Age-standardized		5.9% (4.9 to 7.1%)	45.0 (37.1 to 54.0)
All causes	Chile	2000	Age-standardized		5.4% (4.5 to 6.4%)	35.5 (29.5 to 42.5)
All causes	Chile	2005	Age-standardized		5.1% (4.2 to 6.1%)	32.1 (26.6 to 38.0)
All causes	Chile	2010	Age-standardized		4.8% (3.9 to 5.8%)	30.1 (24.7 to 36.2)

Cause name	Location	Year	Age group	Number (in thousands)	PAF	Rate (per 100,000)
All causes	Chile	2015	Age-standardized		4.7% (3.9 to 5.7%)	28.2 (23.2 to 33.7)
Lower respiratory infections	Chile	1990	All Ages	1.2 (0.6 to 2.0)	17.1% (8.7 to 27.2%)	9.2 (4.6 to 14.9)
Lower respiratory infections	Chile	1995	All Ages	1.2 (0.6 to 1.9)	17.5% (9.1 to 27.5%)	8.4 (4.3 to 13.4)
Lower respiratory infections	Chile	2000	All Ages	1.0 (0.5 to 1.5)	17.9% (9.4 to 27.7%)	6.3 (3.3 to 10.0)
Lower respiratory infections	Chile	2005	All Ages	0.9 (0.5 to 1.3)	18.5% (10.2 to 28.4%)	5.3 (2.8 to 8.1)
Lower respiratory infections	Chile	2010	All Ages	0.9 (0.5 to 1.5)	17.5% (9.2 to 27.5%)	5.5 (2.9 to 8.6)
Lower respiratory infections	Chile	2015	All Ages	1.1 (0.6 to 1.8)	17.2% (8.9 to 27.2%)	6.1 (3.1 to 9.8)
Lower respiratory infections	Chile	1990	Age-standardized		17.1% (8.7 to 27.2%)	13.7 (6.8 to 22.1)
Lower respiratory infections	Chile	1995	Age-standardized		17.5% (9.1 to 27.5%)	11.9 (6.1 to 19.0)
Lower respiratory infections	Chile	2000	Age-standardized		17.9% (9.4 to 27.7%)	8.2 (4.3 to 12.9)
Lower respiratory infections	Chile	2005	Age-standardized		18.5% (10.2 to 28.4%)	6.1 (3.2 to 9.3)
Lower respiratory infections	Chile	2010	Age-standardized		17.5% (9.2 to 27.5%)	5.4 (2.8 to 8.5)
Lower respiratory infections	Chile	2015	Age-standardized		17.2% (8.9 to 27.2%)	5.1 (2.6 to 8.2)
Tracheal, bronchus, and lung cancer	Chile	1990	All Ages	0.2 (0.1 to 0.3)	11.9% (6.5 to 18.2%)	1.7 (0.9 to 2.6)
Tracheal, bronchus, and lung cancer	Chile	1995	All Ages	0.2 (0.1 to 0.4)	12.2% (6.8 to 18.6%)	1.7 (0.9 to 2.6)
Tracheal, bronchus, and lung cancer	Chile	2000	All Ages	0.3 (0.1 to 0.4)	12.5% (6.9 to 19.1%)	1.7 (0.9 to 2.6)
Tracheal, bronchus, and lung cancer	Chile	2005	All Ages	0.3 (0.2 to 0.5)	13.0% (7.2 to 19.4%)	2.0 (1.1 to 3.0)
Tracheal, bronchus, and lung cancer	Chile	2010	All Ages	0.4 (0.2 to 0.6)	12.3% (6.7 to 18.8%)	2.3 (1.3 to 3.4)
Tracheal, bronchus, and lung cancer	Chile	2015	All Ages	0.5 (0.3 to 0.7)	12.1% (6.6 to 18.5%)	2.5 (1.4 to 3.9)
Tracheal, bronchus, and lung cancer	Chile	1990	Age-standardized		11.9% (6.5 to 18.2%)	2.5 (1.3 to 3.8)
Tracheal, bronchus, and lung cancer	Chile	1995	Age-standardized		12.2% (6.8 to 18.6%)	2.3 (1.3 to 3.6)
Tracheal, bronchus, and lung cancer	Chile	2000	Age-standardized		12.5% (6.9 to 19.1%)	2.2 (1.2 to 3.3)
Tracheal, bronchus, and lung cancer	Chile	2005	Age-standardized		13.0% (7.2 to 19.5%)	2.3 (1.2 to 3.4)
Tracheal, bronchus, and lung cancer	Chile	2010	Age-standardized		12.3% (6.7 to 18.8%)	2.3 (1.3 to 3.4)
Tracheal, bronchus, and lung cancer	Chile	2015	Age-standardized		12.1% (6.6 to 18.4%)	2.2 (1.2 to 3.4)
Cardiovascular diseases	Chile	1990	All Ages	3.0 (2.4 to 3.5)	11.2% (9.1 to 13.2%)	22.5 (18.4 to 26.6)
Cardiovascular diseases	Chile	1995	All Ages	2.7 (2.2 to 3.2)	11.1% (9.1 to 13.1%)	18.9 (15.5 to 22.4)
Cardiovascular diseases	Chile	2000	All Ages	2.5 (2.0 to 2.9)	10.6% (8.7 to 12.5%)	16.2 (13.3 to 19.2)
Cardiovascular diseases	Chile	2005	All Ages	2.7 (2.2 to 3.2)	10.3% (8.4 to 12.2%)	16.7 (13.6 to 19.7)
Cardiovascular diseases	Chile	2010	All Ages	3.0 (2.4 to 3.6)	9.8% (7.8 to 11.8%)	17.8 (14.2 to 21.5)
Cardiovascular diseases	Chile	2015	All Ages	3.4 (2.7 to 4.2)	9.5% (7.5 to 11.6%)	19.0 (14.9 to 23.3)
Cardiovascular diseases	Chile	1990	Age-standardized		11.0% (8.8 to 13.1%)	34.4 (27.7 to 41.2)
Cardiovascular diseases	Chile	1995	Age-standardized		10.9% (8.8 to 12.9%)	26.6 (21.5 to 31.6)
Cardiovascular diseases	Chile	2000	Age-standardized		10.4% (8.4 to 12.3%)	20.5 (16.8 to 24.5)
Cardiovascular diseases	Chile	2005	Age-standardized		10.2% (8.3 to 12.1%)	18.7 (15.2 to 22.3)
Cardiovascular diseases	Chile	2010	Age-standardized		9.8% (7.8 to 11.7%)	17.5 (14.1 to 21.0)

Cause name	Location	Year	Age group	Number (in thousands)	PAF	Rate (per 100,000)
Cardiovascular diseases	Chile	2015	Age-standardized		9.6% (7.6 to 11.6%)	16.3 (12.9 to 19.9)
Ischemic heart disease	Chile	1990	All Ages	1.9 (1.5 to 2.4)	14.1% (10.8 to 17.4%)	14.7 (11.2 to 18.2)
Ischemic heart disease	Chile	1995	All Ages	1.7 (1.4 to 2.1)	14.4% (11.2 to 17.7%)	12.3 (9.5 to 15.1)
Ischemic heart disease	Chile	2000	All Ages	1.5 (1.2 to 1.9)	14.5% (11.3 to 17.9%)	10.1 (7.8 to 12.5)
Ischemic heart disease	Chile	2005	All Ages	1.7 (1.3 to 2.1)	14.7% (11.2 to 18.1%)	10.3 (7.9 to 12.8)
Ischemic heart disease	Chile	2010	All Ages	1.9 (1.4 to 2.3)	14.1% (10.5 to 17.8%)	10.9 (8.1 to 13.7)
Ischemic heart disease	Chile	2015	All Ages	2.1 (1.5 to 2.7)	13.8% (10.0 to 17.5%)	11.7 (8.5 to 15.0)
Ischemic heart disease	Chile	1990	Age-standardized		13.7% (10.3 to 17.3%)	22.6 (16.8 to 28.6)
Ischemic heart disease	Chile	1995	Age-standardized		14.1% (10.7 to 17.5%)	17.3 (13.1 to 21.6)
Ischemic heart disease	Chile	2000	Age-standardized		14.3% (11.0 to 17.8%)	12.9 (9.8 to 16.0)
Ischemic heart disease	Chile	2005	Age-standardized		14.5% (11.0 to 18.0%)	11.6 (8.8 to 14.4)
Ischemic heart disease	Chile	2010	Age-standardized		14.1% (10.5 to 17.7%)	10.7 (8.0 to 13.4)
Ischemic heart disease	Chile	2015	Age-standardized		13.9% (10.3 to 17.5%)	10.1 (7.4 to 12.8)
Ischemic stroke	Chile	1990	All Ages	0.6 (0.5 to 0.8)	10.6% (7.6 to 13.8%)	4.9 (3.5 to 6.3)
Ischemic stroke	Chile	1995	All Ages	0.6 (0.4 to 0.7)	10.6% (7.5 to 13.9%)	3.9 (2.8 to 5.2)
Ischemic stroke	Chile	2000	All Ages	0.5 (0.3 to 0.7)	10.3% (7.0 to 14.0%)	3.2 (2.1 to 4.3)
Ischemic stroke	Chile	2005	All Ages	0.5 (0.3 to 0.8)	10.2% (6.7 to 14.4%)	3.3 (2.1 to 4.7)
Ischemic stroke	Chile	2010	All Ages	0.6 (0.4 to 0.9)	9.7% (5.9 to 14.3%)	3.7 (2.2 to 5.4)
Ischemic stroke	Chile	2015	All Ages	0.7 (0.4 to 1.1)	9.6% (5.7 to 14.3%)	3.9 (2.3 to 5.9)
Ischemic stroke	Chile	1990	Age-standardized		10.4% (7.3 to 13.7%)	7.5 (5.2 to 9.9)
Ischemic stroke	Chile	1995	Age-standardized		10.5% (7.3 to 13.8%)	5.7 (3.9 to 7.6)
Ischemic stroke	Chile	2000	Age-standardized		10.2% (6.9 to 14.0%)	4.1 (2.8 to 5.7)
Ischemic stroke	Chile	2005	Age-standardized		10.2% (6.6 to 14.3%)	3.8 (2.4 to 5.4)
Ischemic stroke	Chile	2010	Age-standardized		9.8% (6.1 to 14.1%)	3.6 (2.2 to 5.3)
Ischemic stroke	Chile	2015	Age-standardized		9.6% (5.9 to 14.2%)	3.3 (2.1 to 4.9)
Hemorrhagic stroke	Chile	1990	All Ages	0.4 (0.3 to 0.5)	12.1% (9.3 to 14.9%)	2.9 (2.3 to 3.7)
Hemorrhagic stroke	Chile	1995	All Ages	0.4 (0.3 to 0.5)	12.3% (9.6 to 15.1%)	2.7 (2.1 to 3.3)
Hemorrhagic stroke	Chile	2000	All Ages	0.4 (0.3 to 0.6)	12.3% (9.5 to 15.1%)	2.9 (2.3 to 3.6)
Hemorrhagic stroke	Chile	2005	All Ages	0.5 (0.4 to 0.6)	12.2% (9.4 to 15.0%)	3.0 (2.3 to 3.8)
Hemorrhagic stroke	Chile	2010	All Ages	0.6 (0.4 to 0.7)	11.5% (8.7 to 14.5%)	3.2 (2.4 to 4.1)
Hemorrhagic stroke	Chile	2015	All Ages	0.6 (0.4 to 0.8)	11.2% (8.2 to 14.4%)	3.3 (2.5 to 4.3)
Hemorrhagic stroke	Chile	1990	Age-standardized		11.7% (8.9 to 14.6%)	4.2 (3.2 to 5.3)
Hemorrhagic stroke	Chile	1995	Age-standardized		11.9% (9.2 to 14.8%)	3.6 (2.7 to 4.5)
Hemorrhagic stroke	Chile	2000	Age-standardized		12.0% (9.2 to 14.9%)	3.5 (2.7 to 4.4)
Hemorrhagic stroke	Chile	2005	Age-standardized		12.0% (9.2 to 14.9%)	3.3 (2.5 to 4.2)
Hemorrhagic stroke	Chile	2010	Age-standardized		11.5% (8.6 to 14.4%)	3.1 (2.3 to 4.0)

Cause name	Location	Year	Age group	Number (in thousands)	PAF	Rate (per 100,000)
Hemorrhagic stroke	Chile	2015	Age-standardized		11.2% (8.3 to 14.3%)	2.9 (2.1 to 3.7)
Chronic obstructive pulmonary disease	Chile	1990	All Ages	0.4 (0.2 to 0.7)	18.0% (8.5 to 28.5%)	3.3 (1.5 to 5.2)
Chronic obstructive pulmonary disease	Chile	1995	All Ages	0.4 (0.2 to 0.7)	18.4% (8.9 to 29.1%)	2.9 (1.4 to 4.6)
Chronic obstructive pulmonary disease	Chile	2000	All Ages	0.5 (0.3 to 0.8)	18.8% (9.2 to 29.4%)	3.5 (1.7 to 5.5)
Chronic obstructive pulmonary disease	Chile	2005	All Ages	0.7 (0.3 to 1.1)	19.3% (9.5 to 30.0%)	4.3 (2.1 to 6.9)
Chronic obstructive pulmonary disease	Chile	2010	All Ages	0.8 (0.4 to 1.4)	18.6% (8.9 to 29.3%)	5.0 (2.4 to 8.0)
Chronic obstructive pulmonary disease	Chile	2015	All Ages	1.0 (0.5 to 1.5)	18.4% (8.8 to 29.0%)	5.3 (2.5 to 8.5)
Chronic obstructive pulmonary disease	Chile	1990	Age-standardized		18.1% (8.6 to 28.7%)	5.1 (2.4 to 8.2)
Chronic obstructive pulmonary disease	Chile	1995	Age-standardized		18.5% (8.9 to 29.2%)	4.1 (2.0 to 6.6)
Chronic obstructive pulmonary disease	Chile	2000	Age-standardized		18.8% (9.2 to 29.4%)	4.5 (2.2 to 7.2)
Chronic obstructive pulmonary disease	Chile	2005	Age-standardized		19.3% (9.5 to 30.0%)	5.0 (2.5 to 8.0)
Chronic obstructive pulmonary disease	Chile	2010	Age-standardized		18.6% (8.9 to 29.3%)	4.9 (2.4 to 7.9)
Chronic obstructive pulmonary disease	Chile	2015	Age-standardized		18.3% (8.8 to 29.0%)	4.5 (2.2 to 7.2)
All causes	Uruguay	1990	All Ages	1.5 (1.2 to 1.8)	5.0% (4.1 to 6.0%)	47.9 (39.1 to 57.3)
All causes	Uruguay	1995	All Ages	1.6 (1.3 to 1.9)	4.9% (3.9 to 5.9%)	48.1 (39.0 to 58.7)
All causes	Uruguay	2000	All Ages	1.5 (1.2 to 1.8)	4.6% (3.7 to 5.7%)	44.8 (35.5 to 54.7)
All causes	Uruguay	2005	All Ages	1.4 (1.1 to 1.7)	4.1% (3.2 to 5.2%)	40.6 (31.8 to 51.2)
All causes	Uruguay	2010	All Ages	1.4 (1.1 to 1.7)	4.0% (3.1 to 5.1%)	40.6 (31.3 to 51.6)
All causes	Uruguay	2015	All Ages	1.4 (1.1 to 1.8)	3.8% (2.9 to 4.9%)	40.7 (31.1 to 52.3)
All causes	Uruguay	1990	Age-standardized		4.9% (4.0 to 5.9%)	43.5 (35.4 to 52.2)
All causes	Uruguay	1995	Age-standardized		4.8% (3.9 to 5.8%)	41.3 (33.5 to 50.3)
All causes	Uruguay	2000	Age-standardized		4.6% (3.6 to 5.5%)	36.7 (29.4 to 44.5)
All causes	Uruguay	2005	Age-standardized		4.1% (3.2 to 5.1%)	31.0 (24.5 to 38.7)
All causes	Uruguay	2010	Age-standardized		3.9% (3.1 to 4.9%)	28.3 (22.3 to 35.5)
All causes	Uruguay	2015	Age-standardized		3.7% (2.9 to 4.7%)	26.6 (21.0 to 33.8)
Lower respiratory infections	Uruguay	1990	All Ages	0.1 (0.0 to 0.2)	11.4% (3.7 to 22.5%)	3.5 (1.1 to 7.0)
Lower respiratory infections	Uruguay	1995	All Ages	0.1 (0.0 to 0.3)	11.4% (3.6 to 22.5%)	4.2 (1.3 to 8.3)
Lower respiratory infections	Uruguay	2000	All Ages	0.2 (0.1 to 0.3)	11.7% (3.8 to 22.8%)	4.9 (1.6 to 9.7)
Lower respiratory infections	Uruguay	2005	All Ages	0.2 (0.1 to 0.3)	10.4% (2.9 to 21.5%)	4.8 (1.4 to 10.0)
Lower respiratory infections	Uruguay	2010	All Ages	0.2 (0.1 to 0.4)	10.3% (2.9 to 21.4%)	5.3 (1.5 to 10.9)
Lower respiratory infections	Uruguay	2015	All Ages	0.2 (0.1 to 0.4)	10.0% (2.7 to 21.1%)	5.4 (1.4 to 11.6)
Lower respiratory infections	Uruguay	1990	Age-standardized		11.4% (3.7 to 22.5%)	3.3 (1.1 to 6.6)
Lower respiratory infections	Uruguay	1995	Age-standardized		11.4% (3.6 to 22.5%)	3.6 (1.1 to 7.2)
Lower respiratory infections	Uruguay	2000	Age-standardized		11.7% (3.8 to 22.8%)	4.0 (1.3 to 7.9)
Lower respiratory infections	Uruguay	2005	Age-standardized		10.4% (2.9 to 21.5%)	3.6 (1.0 to 7.5)
Lower respiratory infections	Uruguay	2010	Age-standardized		10.3% (2.9 to 21.4%)	3.5 (1.0 to 7.3)

Cause name	Location	Year	Age group	Number (in thousands)	PAF	Rate (per 100,000)
Lower respiratory infections	Uruguay	2015	Age-standardized		10.0% (2.7 to 21.1%)	3.3 (0.9 to 7.1)
Tracheal, bronchus, and lung cancer	Uruguay	1990	All Ages	0.1 (0.1 to 0.2)	7.8% (3.8 to 13.0%)	3.6 (1.7 to 6.1)
Tracheal, bronchus, and lung cancer	Uruguay	1995	All Ages	0.1 (0.1 to 0.2)	7.9% (3.8 to 13.0%)	3.7 (1.8 to 6.1)
Tracheal, bronchus, and lung cancer	Uruguay	2000	All Ages	0.1 (0.1 to 0.2)	8.0% (3.8 to 13.2%)	3.5 (1.7 to 5.9)
Tracheal, bronchus, and lung cancer	Uruguay	2005	All Ages	0.1 (0.1 to 0.2)	7.1% (3.3 to 12.0%)	3.1 (1.4 to 5.2)
Tracheal, bronchus, and lung cancer	Uruguay	2010	All Ages	0.1 (0.1 to 0.2)	7.0% (3.3 to 12.0%)	3.1 (1.5 to 5.3)
Tracheal, bronchus, and lung cancer	Uruguay	2015	All Ages	0.1 (0.1 to 0.2)	6.8% (3.1 to 11.6%)	3.2 (1.5 to 5.4)
Tracheal, bronchus, and lung cancer	Uruguay	1990	Age-standardized		7.8% (3.8 to 13.0%)	3.3 (1.6 to 5.4)
Tracheal, bronchus, and lung cancer	Uruguay	1995	Age-standardized		7.9% (3.8 to 13.0%)	3.2 (1.6 to 5.3)
Tracheal, bronchus, and lung cancer	Uruguay	2000	Age-standardized		8.0% (3.8 to 13.2%)	3.0 (1.5 to 5.0)
Tracheal, bronchus, and lung cancer	Uruguay	2005	Age-standardized		7.1% (3.3 to 12.0%)	2.5 (1.2 to 4.3)
Tracheal, bronchus, and lung cancer	Uruguay	2010	Age-standardized		7.0% (3.3 to 12.0%)	2.5 (1.2 to 4.2)
Tracheal, bronchus, and lung cancer	Uruguay	2015	Age-standardized		6.8% (3.1 to 11.6%)	2.4 (1.1 to 4.1)
Cardiovascular diseases	Uruguay	1990	All Ages	1.1 (0.9 to 1.4)	9.3% (7.4 to 11.3%)	36.3 (28.7 to 44.0)
Cardiovascular diseases	Uruguay	1995	All Ages	1.1 (0.9 to 1.4)	8.9% (7.0 to 10.9%)	34.3 (26.8 to 42.0)
Cardiovascular diseases	Uruguay	2000	All Ages	1.0 (0.8 to 1.2)	8.5% (6.6 to 10.4%)	29.6 (23.1 to 36.2)
Cardiovascular diseases	Uruguay	2005	All Ages	0.9 (0.7 to 1.1)	7.7% (5.9 to 9.7%)	26.2 (20.0 to 33.1)
Cardiovascular diseases	Uruguay	2010	All Ages	0.8 (0.6 to 1.1)	7.4% (5.5 to 9.5%)	25.3 (18.8 to 32.4)
Cardiovascular diseases	Uruguay	2015	All Ages	0.9 (0.6 to 1.1)	7.2% (5.3 to 9.3%)	25.1 (18.2 to 32.7)
Cardiovascular diseases	Uruguay	1990	Age-standardized		9.3% (7.3 to 11.3%)	32.9 (26.0 to 40.1)
Cardiovascular diseases	Uruguay	1995	Age-standardized		9.0% (7.2 to 11.0%)	29.4 (23.2 to 35.7)
Cardiovascular diseases	Uruguay	2000	Age-standardized		8.6% (6.8 to 10.5%)	24.3 (19.0 to 29.6)
Cardiovascular diseases	Uruguay	2005	Age-standardized		7.9% (6.2 to 9.9%)	20.0 (15.6 to 24.9)
Cardiovascular diseases	Uruguay	2010	Age-standardized		7.7% (6.0 to 9.6%)	17.7 (13.7 to 22.0)
Cardiovascular diseases	Uruguay	2015	Age-standardized		7.5% (5.8 to 9.4%)	16.4 (12.6 to 20.6)
Ischemic heart disease	Uruguay	1990	All Ages	0.8 (0.6 to 1.0)	12.0% (9.0 to 14.9%)	25.2 (18.8 to 31.3)
Ischemic heart disease	Uruguay	1995	All Ages	0.7 (0.6 to 0.9)	11.9% (8.9 to 15.0%)	23.0 (17.0 to 29.0)
Ischemic heart disease	Uruguay	2000	All Ages	0.6 (0.5 to 0.8)	11.9% (8.8 to 15.2%)	18.6 (13.6 to 23.8)
Ischemic heart disease	Uruguay	2005	All Ages	0.5 (0.4 to 0.7)	11.2% (8.0 to 14.5%)	16.1 (11.4 to 20.9)
Ischemic heart disease	Uruguay	2010	All Ages	0.5 (0.4 to 0.7)	11.0% (7.6 to 14.4%)	15.8 (10.7 to 21.0)
Ischemic heart disease	Uruguay	2015	All Ages	0.6 (0.4 to 0.7)	10.7% (7.2 to 14.3%)	16.0 (10.7 to 21.6)
Ischemic heart disease	Uruguay	1990	Age-standardized		12.0% (9.0 to 14.9%)	22.8 (16.8 to 28.4)
Ischemic heart disease	Uruguay	1995	Age-standardized		12.1% (9.1 to 15.1%)	19.7 (14.8 to 24.8)
Ischemic heart disease	Uruguay	2000	Age-standardized		12.2% (9.1 to 15.3%)	15.3 (11.3 to 19.3)
Ischemic heart disease	Uruguay	2005	Age-standardized		11.6% (8.6 to 14.6%)	12.4 (9.1 to 15.7)
Ischemic heart disease	Uruguay	2010	Age-standardized		11.5% (8.4 to 14.6%)	11.2 (8.1 to 14.3)

Cause name	Location	Year	Age group	Number (in thousands)	PAF	Rate (per 100,000)
Ischemic heart disease	Uruguay	2015	Age-standardized		11.3% (8.1 to 14.4%)	10.6 (7.6 to 13.7)
Ischemic stroke	Uruguay	1990	All Ages	0.2 (0.1 to 0.3)	8.3% (5.2 to 11.9%)	7.3 (4.6 to 10.6)
Ischemic stroke	Uruguay	1995	All Ages	0.2 (0.1 to 0.3)	8.2% (5.0 to 12.0%)	7.3 (4.4 to 10.7)
Ischemic stroke	Uruguay	2000	All Ages	0.2 (0.1 to 0.3)	8.1% (4.7 to 12.1%)	6.6 (3.8 to 10.0)
Ischemic stroke	Uruguay	2005	All Ages	0.2 (0.1 to 0.3)	7.5% (4.1 to 11.7%)	6.1 (3.3 to 9.6)
Ischemic stroke	Uruguay	2010	All Ages	0.2 (0.1 to 0.3)	7.3% (3.8 to 11.9%)	5.8 (3.0 to 9.5)
Ischemic stroke	Uruguay	2015	All Ages	0.2 (0.1 to 0.3)	7.2% (3.6 to 12.0%)	5.7 (2.8 to 9.5)
Ischemic stroke	Uruguay	1990	Age-standardized		8.3% (5.1 to 12.0%)	6.7 (4.1 to 9.7)
Ischemic stroke	Uruguay	1995	Age-standardized		8.3% (5.1 to 12.1%)	6.2 (3.7 to 9.0)
Ischemic stroke	Uruguay	2000	Age-standardized		8.1% (4.8 to 12.2%)	5.2 (3.0 to 7.8)
Ischemic stroke	Uruguay	2005	Age-standardized		7.6% (4.4 to 11.6%)	4.4 (2.5 to 6.7)
Ischemic stroke	Uruguay	2010	Age-standardized		7.5% (4.3 to 11.7%)	3.8 (2.1 to 5.9)
Ischemic stroke	Uruguay	2015	Age-standardized		7.4% (4.1 to 11.7%)	3.4 (1.9 to 5.4)
Hemorrhagic stroke	Uruguay	1990	All Ages	0.1 (0.1 to 0.1)	9.4% (6.7 to 12.1%)	3.8 (2.7 to 4.8)
Hemorrhagic stroke	Uruguay	1995	All Ages	0.1 (0.1 to 0.2)	9.3% (6.7 to 12.2%)	4.0 (2.9 to 5.3)
Hemorrhagic stroke	Uruguay	2000	All Ages	0.1 (0.1 to 0.2)	9.4% (6.7 to 12.3%)	4.4 (3.1 to 5.7)
Hemorrhagic stroke	Uruguay	2005	All Ages	0.1 (0.1 to 0.2)	8.7% (5.9 to 11.6%)	4.0 (2.8 to 5.5)
Hemorrhagic stroke	Uruguay	2010	All Ages	0.1 (0.1 to 0.2)	8.3% (5.6 to 11.6%)	3.6 (2.4 to 5.1)
Hemorrhagic stroke	Uruguay	2015	All Ages	0.1 (0.1 to 0.2)	8.1% (5.2 to 11.6%)	3.5 (2.2 to 5.0)
Hemorrhagic stroke	Uruguay	1990	Age-standardized		9.4% (6.8 to 12.2%)	3.5 (2.5 to 4.5)
Hemorrhagic stroke	Uruguay	1995	Age-standardized		9.5% (6.9 to 12.4%)	3.6 (2.6 to 4.7)
Hemorrhagic stroke	Uruguay	2000	Age-standardized		9.7% (6.9 to 12.5%)	3.8 (2.7 to 4.9)
Hemorrhagic stroke	Uruguay	2005	Age-standardized		9.0% (6.3 to 11.8%)	3.3 (2.3 to 4.4)
Hemorrhagic stroke	Uruguay	2010	Age-standardized		8.8% (6.1 to 11.7%)	2.7 (1.9 to 3.6)
Hemorrhagic stroke	Uruguay	2015	Age-standardized		8.5% (5.9 to 11.7%)	2.4 (1.7 to 3.3)
Chronic obstructive pulmonary disease	Uruguay	1990	All Ages	0.1 (0.1 to 0.2)	13.5% (5.3 to 23.7%)	4.4 (1.8 to 7.6)
Chronic obstructive pulmonary disease	Uruguay	1995	All Ages	0.2 (0.1 to 0.3)	13.5% (5.3 to 23.6%)	5.9 (2.4 to 10.2)
Chronic obstructive pulmonary disease	Uruguay	2000	All Ages	0.2 (0.1 to 0.4)	13.8% (5.6 to 23.8%)	6.8 (2.8 to 11.7)
Chronic obstructive pulmonary disease	Uruguay	2005	All Ages	0.2 (0.1 to 0.4)	12.6% (4.4 to 22.6%)	6.5 (2.3 to 11.7)
Chronic obstructive pulmonary disease	Uruguay	2010	All Ages	0.2 (0.1 to 0.4)	12.5% (4.6 to 22.5%)	6.9 (2.5 to 12.7)
Chronic obstructive pulmonary disease	Uruguay	2015	All Ages	0.2 (0.1 to 0.4)	12.3% (4.5 to 22.4%)	7.0 (2.6 to 12.9)
Chronic obstructive pulmonary disease	Uruguay	1990	Age-standardized		13.5% (5.3 to 23.7%)	3.9 (1.6 to 6.8)
Chronic obstructive pulmonary disease	Uruguay	1995	Age-standardized		13.5% (5.3 to 23.6%)	5.0 (2.0 to 8.6)
Chronic obstructive pulmonary disease	Uruguay	2000	Age-standardized		13.8% (5.6 to 23.8%)	5.4 (2.2 to 9.3)
Chronic obstructive pulmonary disease	Uruguay	2005	Age-standardized		12.6% (4.4 to 22.5%)	4.8 (1.7 to 8.6)
Chronic obstructive pulmonary disease	Uruguay	2010	Age-standardized		12.5% (4.6 to 22.5%)	4.7 (1.7 to 8.6)

Cause name	Location	Year	Age group	Number (in thousands)	PAF	Rate (per 100,000)
Chronic obstructive pulmonary disease	Uruguay	2015	Age-standardized		12.2% (4.5 to 22.4%)	4.5 (1.6 to 8.1)
All causes	Belarus	1990	All Ages	11.7 (9.9 to 13.6)	10.4% (8.8 to 12.1%)	115.3 (97.5 to 134.2)
All causes	Belarus	1995	All Ages	12.4 (10.4 to 14.4)	9.2% (7.7 to 10.7%)	121.8 (102.4 to 141.7)
All causes	Belarus	2000	All Ages	12.2 (10.2 to 14.2)	8.7% (7.2 to 10.1%)	121.8 (101.8 to 142.2)
All causes	Belarus	2005	All Ages	11.8 (9.7 to 13.8)	8.3% (6.9 to 9.7%)	121.6 (100.5 to 142.6)
All causes	Belarus	2010	All Ages	11.5 (9.4 to 13.6)	8.4% (6.8 to 9.9%)	120.8 (99.0 to 142.8)
All causes	Belarus	2015	All Ages	12.2 (9.8 to 14.7)	8.3% (6.6 to 10.0%)	126.6 (101.8 to 153.0)
All causes	Belarus	1990	Age-standardized		10.2% (8.6 to 11.9%)	104.8 (88.5 to 122.4)
All causes	Belarus	1995	Age-standardized		9.0% (7.5 to 10.5%)	107.8 (90.5 to 125.6)
All causes	Belarus	2000	Age-standardized		8.4% (6.9 to 9.9%)	103.5 (85.4 to 122.0)
All causes	Belarus	2005	Age-standardized		8.1% (6.7 to 9.5%)	94.0 (77.4 to 111.1)
All causes	Belarus	2010	Age-standardized		8.1% (6.7 to 9.6%)	85.8 (71.0 to 101.3)
All causes	Belarus	2015	Age-standardized		8.0% (6.6 to 9.6%)	81.8 (67.1 to 98.0)
Lower respiratory infections	Belarus	1990	All Ages	0.3 (0.2 to 0.4)	23.8% (16.1 to 32.3%)	3.1 (2.1 to 4.3)
Lower respiratory infections	Belarus	1995	All Ages	0.3 (0.1 to 0.4)	18.7% (10.8 to 28.2%)	2.5 (1.4 to 3.8)
Lower respiratory infections	Belarus	2000	All Ages	0.2 (0.1 to 0.4)	16.5% (8.6 to 26.3%)	2.3 (1.2 to 3.8)
Lower respiratory infections	Belarus	2005	All Ages	0.2 (0.1 to 0.4)	15.5% (7.8 to 25.8%)	2.2 (1.1 to 3.7)
Lower respiratory infections	Belarus	2010	All Ages	0.2 (0.1 to 0.4)	16.5% (8.5 to 26.0%)	2.2 (1.1 to 3.7)
Lower respiratory infections	Belarus	2015	All Ages	0.2 (0.1 to 0.4)	16.6% (8.8 to 26.5%)	2.2 (1.1 to 3.6)
Lower respiratory infections	Belarus	1990	Age-standardized		23.8% (16.1 to 32.3%)	3.2 (2.2 to 4.4)
Lower respiratory infections	Belarus	1995	Age-standardized		18.7% (10.8 to 28.2%)	2.6 (1.5 to 4.0)
Lower respiratory infections	Belarus	2000	Age-standardized		16.5% (8.6 to 26.3%)	2.3 (1.2 to 3.8)
Lower respiratory infections	Belarus	2005	Age-standardized		15.5% (7.8 to 25.8%)	1.9 (1.0 to 3.3)
Lower respiratory infections	Belarus	2010	Age-standardized		16.5% (8.5 to 26.0%)	1.8 (0.9 to 2.9)
Lower respiratory infections	Belarus	2015	Age-standardized		16.6% (8.8 to 26.5%)	1.5 (0.8 to 2.6)
Tracheal, bronchus, and lung cancer	Belarus	1990	All Ages	0.6 (0.4 to 0.9)	17.1% (10.2 to 24.5%)	6.3 (3.8 to 9.1)
Tracheal, bronchus, and lung cancer	Belarus	1995	All Ages	0.6 (0.3 to 0.9)	13.2% (7.5 to 19.6%)	5.8 (3.2 to 8.6)
Tracheal, bronchus, and lung cancer	Belarus	2000	All Ages	0.5 (0.3 to 0.8)	11.5% (6.3 to 17.6%)	4.9 (2.7 to 7.6)
Tracheal, bronchus, and lung cancer	Belarus	2005	All Ages	0.4 (0.2 to 0.7)	10.8% (5.8 to 17.0%)	4.5 (2.4 to 7.0)
Tracheal, bronchus, and lung cancer	Belarus	2010	All Ages	0.5 (0.3 to 0.7)	11.5% (6.3 to 17.9%)	4.8 (2.6 to 7.5)
Tracheal, bronchus, and lung cancer	Belarus	2015	All Ages	0.5 (0.3 to 0.8)	11.6% (6.3 to 18.0%)	5.5 (2.9 to 8.6)
Tracheal, bronchus, and lung cancer	Belarus	1990	Age-standardized		17.1% (10.2 to 24.5%)	5.4 (3.2 to 7.8)
Tracheal, bronchus, and lung cancer	Belarus	1995	Age-standardized		13.2% (7.5 to 19.6%)	4.8 (2.7 to 7.1)
Tracheal, bronchus, and lung cancer	Belarus	2000	Age-standardized		11.5% (6.3 to 17.6%)	3.9 (2.1 to 6.0)
Tracheal, bronchus, and lung cancer	Belarus	2005	Age-standardized		10.8% (5.8 to 17.0%)	3.4 (1.9 to 5.3)
Tracheal, bronchus, and lung cancer	Belarus	2010	Age-standardized		11.5% (6.3 to 17.9%)	3.5 (1.9 to 5.5)

Cause name	Location	Year	Age group	Number (in thousands)	PAF	Rate (per 100,000)
Tracheal, bronchus, and lung cancer	Belarus	2015	Age-standardized		11.6% (6.3 to 18.0%)	3.8 (2.0 to 5.9)
Cardiovascular diseases	Belarus	1990	All Ages	9.3 (7.7 to 11.0)	14.6% (12.1 to 17.3%)	91.3 (75.8 to 108.0)
Cardiovascular diseases	Belarus	1995	All Ages	10.3 (8.5 to 12.2)	13.3% (10.9 to 15.7%)	101.3 (83.1 to 119.7)
Cardiovascular diseases	Belarus	2000	All Ages	10.5 (8.7 to 12.4)	12.6% (10.4 to 14.9%)	104.8 (86.7 to 124.1)
Cardiovascular diseases	Belarus	2005	All Ages	10.4 (8.5 to 12.3)	12.1% (9.9 to 14.3%)	107.5 (87.7 to 127.2)
Cardiovascular diseases	Belarus	2010	All Ages	10.2 (8.2 to 12.3)	12.2% (9.8 to 14.6%)	107.7 (86.8 to 129.4)
Cardiovascular diseases	Belarus	2015	All Ages	10.8 (8.5 to 13.2)	12.0% (9.3 to 14.7%)	112.3 (88.1 to 137.5)
Cardiovascular diseases	Belarus	1990	Age-standardized		14.5% (12.0 to 17.3%)	82.8 (68.9 to 98.5)
Cardiovascular diseases	Belarus	1995	Age-standardized		13.1% (10.7 to 15.6%)	89.6 (73.2 to 106.5)
Cardiovascular diseases	Belarus	2000	Age-standardized		12.3% (10.0 to 14.7%)	89.1 (72.3 to 106.5)
Cardiovascular diseases	Belarus	2005	Age-standardized		12.1% (9.8 to 14.3%)	83.0 (67.0 to 98.7)
Cardiovascular diseases	Belarus	2010	Age-standardized		12.3% (10.0 to 14.7%)	76.3 (62.0 to 91.2)
Cardiovascular diseases	Belarus	2015	Age-standardized		12.2% (9.8 to 14.8%)	72.2 (58.4 to 87.3)
Ischemic heart disease	Belarus	1990	All Ages	6.9 (5.4 to 8.5)	16.3% (12.8 to 20.0%)	68.2 (53.7 to 83.7)
Ischemic heart disease	Belarus	1995	All Ages	7.8 (6.0 to 9.5)	15.0% (11.8 to 18.2%)	76.4 (59.4 to 93.5)
Ischemic heart disease	Belarus	2000	All Ages	8.0 (6.2 to 9.7)	14.2% (11.1 to 17.3%)	80.0 (62.1 to 97.2)
Ischemic heart disease	Belarus	2005	All Ages	8.1 (6.4 to 9.9)	13.7% (10.8 to 16.7%)	83.8 (65.8 to 102.3)
Ischemic heart disease	Belarus	2010	All Ages	8.1 (6.2 to 10.1)	13.8% (10.5 to 17.1%)	85.4 (65.1 to 106.0)
Ischemic heart disease	Belarus	2015	All Ages	8.6 (6.5 to 10.9)	13.5% (10.0 to 17.0%)	89.8 (67.1 to 112.9)
Ischemic heart disease	Belarus	1990	Age-standardized		16.2% (12.7 to 19.9%)	62.0 (48.4 to 76.5)
Ischemic heart disease	Belarus	1995	Age-standardized		14.8% (11.4 to 18.1%)	67.7 (52.2 to 83.5)
Ischemic heart disease	Belarus	2000	Age-standardized		13.9% (10.7 to 17.1%)	68.3 (52.6 to 83.6)
Ischemic heart disease	Belarus	2005	Age-standardized		13.7% (10.7 to 16.7%)	64.8 (50.5 to 79.1)
Ischemic heart disease	Belarus	2010	Age-standardized		14.0% (10.7 to 17.3%)	60.5 (46.5 to 74.8)
Ischemic heart disease	Belarus	2015	Age-standardized		13.9% (10.6 to 17.3%)	57.7 (44.5 to 71.6)
Ischemic stroke	Belarus	1990	All Ages	1.6 (1.1 to 1.9)	12.6% (9.4 to 15.9%)	15.3 (11.2 to 19.1)
Ischemic stroke	Belarus	1995	All Ages	1.7 (1.2 to 2.1)	11.1% (7.9 to 14.3%)	16.4 (11.6 to 20.8)
Ischemic stroke	Belarus	2000	All Ages	1.7 (1.2 to 2.1)	10.4% (7.3 to 13.4%)	16.6 (11.7 to 21.2)
Ischemic stroke	Belarus	2005	All Ages	1.6 (1.1 to 2.1)	9.9% (6.8 to 13.0%)	16.1 (11.1 to 21.4)
Ischemic stroke	Belarus	2010	All Ages	1.5 (1.0 to 2.0)	10.0% (6.8 to 13.4%)	15.4 (10.6 to 20.6)
Ischemic stroke	Belarus	2015	All Ages	1.5 (1.1 to 2.1)	9.9% (6.6 to 13.5%)	16.0 (10.9 to 22.1)
Ischemic stroke	Belarus	1990	Age-standardized		12.5% (9.3 to 15.8%)	13.8 (10.1 to 17.5)
Ischemic stroke	Belarus	1995	Age-standardized		10.9% (7.6 to 14.2%)	14.5 (10.1 to 18.5)
Ischemic stroke	Belarus	2000	Age-standardized		10.1% (7.0 to 13.4%)	14.0 (9.6 to 18.5)
Ischemic stroke	Belarus	2005	Age-standardized		9.8% (6.8 to 13.0%)	12.3 (8.4 to 16.4)
Ischemic stroke	Belarus	2010	Age-standardized		10.1% (6.9 to 13.4%)	10.8 (7.5 to 14.4)

Cause name	Location	Year	Age group	Number (in thousands)	PAF	Rate (per 100,000)
Ischemic stroke	Belarus	2015	Age-standardized		10.1% (7.0 to 13.5%)	10.1 (7.1 to 13.6)
Hemorrhagic stroke	Belarus	1990	All Ages	0.8 (0.6 to 1.0)	14.8% (11.8 to 17.9%)	7.8 (6.0 to 9.7)
Hemorrhagic stroke	Belarus	1995	All Ages	0.9 (0.7 to 1.1)	12.9% (10.0 to 15.8%)	8.5 (6.5 to 10.7)
Hemorrhagic stroke	Belarus	2000	All Ages	0.8 (0.6 to 1.0)	11.9% (9.1 to 14.9%)	8.2 (6.2 to 10.4)
Hemorrhagic stroke	Belarus	2005	All Ages	0.7 (0.5 to 1.0)	11.5% (8.7 to 14.4%)	7.7 (5.6 to 9.8)
Hemorrhagic stroke	Belarus	2010	All Ages	0.7 (0.5 to 0.8)	11.7% (8.9 to 14.7%)	6.8 (5.0 to 8.7)
Hemorrhagic stroke	Belarus	2015	All Ages	0.6 (0.5 to 0.8)	11.6% (8.8 to 14.8%)	6.5 (4.7 to 8.4)
Hemorrhagic stroke	Belarus	1990	Age-standardized		14.7% (11.8 to 17.7%)	7.0 (5.4 to 8.7)
Hemorrhagic stroke	Belarus	1995	Age-standardized		12.9% (10.0 to 15.7%)	7.4 (5.6 to 9.2)
Hemorrhagic stroke	Belarus	2000	Age-standardized		11.9% (9.1 to 14.7%)	6.8 (5.1 to 8.8)
Hemorrhagic stroke	Belarus	2005	Age-standardized		11.6% (8.7 to 14.5%)	6.0 (4.4 to 7.5)
Hemorrhagic stroke	Belarus	2010	Age-standardized		11.9% (9.1 to 14.8%)	5.0 (3.7 to 6.3)
Hemorrhagic stroke	Belarus	2015	Age-standardized		11.9% (9.0 to 14.9%)	4.4 (3.2 to 5.7)
Chronic obstructive pulmonary disease	Belarus	1990	All Ages	1.5 (0.8 to 2.2)	23.5% (13.4 to 34.5%)	14.7 (8.1 to 21.9)
Chronic obstructive pulmonary disease	Belarus	1995	All Ages	1.3 (0.6 to 1.9)	19.4% (10.0 to 30.4%)	12.2 (6.2 to 19.1)
Chronic obstructive pulmonary disease	Belarus	2000	All Ages	1.0 (0.5 to 1.6)	17.6% (8.4 to 28.0%)	9.8 (4.6 to 15.6)
Chronic obstructive pulmonary disease	Belarus	2005	All Ages	0.7 (0.3 to 1.2)	16.8% (7.8 to 27.0%)	7.5 (3.4 to 12.1)
Chronic obstructive pulmonary disease	Belarus	2010	All Ages	0.6 (0.3 to 0.9)	17.6% (8.4 to 28.3%)	6.1 (2.9 to 9.7)
Chronic obstructive pulmonary disease	Belarus	2015	All Ages	0.6 (0.3 to 1.0)	17.7% (8.5 to 27.9%)	6.6 (3.1 to 10.6)
Chronic obstructive pulmonary disease	Belarus	1990	Age-standardized		23.5% (13.4 to 34.5%)	13.3 (7.4 to 19.8)
Chronic obstructive pulmonary disease	Belarus	1995	Age-standardized		19.4% (10.0 to 30.4%)	10.8 (5.5 to 16.8)
Chronic obstructive pulmonary disease	Belarus	2000	Age-standardized		17.6% (8.4 to 28.0%)	8.3 (3.9 to 13.2)
Chronic obstructive pulmonary disease	Belarus	2005	Age-standardized		16.8% (7.8 to 27.0%)	5.7 (2.6 to 9.2)
Chronic obstructive pulmonary disease	Belarus	2010	Age-standardized		17.6% (8.4 to 28.3%)	4.3 (2.0 to 6.9)
Chronic obstructive pulmonary disease	Belarus	2015	Age-standardized		17.7% (8.5 to 27.9%)	4.3 (2.0 to 6.8)
All causes	Estonia	1990	All Ages	1.2 (1.0 to 1.5)	6.3% (5.0 to 7.6%)	76.7 (61.1 to 93.2)
All causes	Estonia	1995	All Ages	1.1 (0.9 to 1.4)	5.3% (4.1 to 6.5%)	77.5 (60.4 to 95.6)
All causes	Estonia	2000	All Ages	0.9 (0.7 to 1.1)	4.6% (3.5 to 5.8%)	64.0 (48.3 to 80.5)
All causes	Estonia	2005	All Ages	0.8 (0.5 to 1.0)	4.2% (3.1 to 5.4%)	55.3 (40.4 to 71.7)
All causes	Estonia	2010	All Ages	0.6 (0.4 to 0.8)	3.8% (2.7 to 5.0%)	42.0 (29.9 to 55.9)
All causes	Estonia	2015	All Ages	0.7 (0.5 to 0.9)	4.3% (3.1 to 5.8%)	49.5 (35.1 to 66.3)
All causes	Estonia	1990	Age-standardized		6.1% (4.9 to 7.4%)	65.0 (51.7 to 79.1)
All causes	Estonia	1995	Age-standardized		5.1% (4.0 to 6.2%)	60.9 (47.9 to 74.9)
All causes	Estonia	2000	Age-standardized		4.4% (3.3 to 5.6%)	47.1 (35.1 to 59.3)
All causes	Estonia	2005	Age-standardized		4.0% (3.0 to 5.2%)	36.7 (26.9 to 47.3)
All causes	Estonia	2010	Age-standardized		3.6% (2.6 to 4.7%)	24.9 (18.1 to 32.2)

Cause name	Location	Year	Age group	Number (in thousands)	PAF	Rate (per 100,000)
All causes	Estonia	2015	Age-standardized		4.1% (3.0 to 5.2%)	25.9 (19.1 to 33.3)
Lower respiratory infections	Estonia	1990	All Ages	0.0 (0.0 to 0.1)	10.2% (3.0 to 21.3%)	1.5 (0.4 to 3.1)
Lower respiratory infections	Estonia	1995	All Ages	0.0 (0.0 to 0.1)	7.5% (1.5 to 18.1%)	1.6 (0.3 to 3.9)
Lower respiratory infections	Estonia	2000	All Ages	0.0 (0.0 to 0.1)	6.4% (1.0 to 17.1%)	1.4 (0.2 to 3.8)
Lower respiratory infections	Estonia	2005	All Ages	0.0 (0.0 to 0.0)	6.4% (1.0 to 16.9%)	1.1 (0.2 to 3.0)
Lower respiratory infections	Estonia	2010	All Ages	0.0 (0.0 to 0.0)	6.6% (1.1 to 17.2%)	0.9 (0.2 to 2.3)
Lower respiratory infections	Estonia	2015	All Ages	0.0 (0.0 to 0.0)	7.9% (1.6 to 18.9%)	1.0 (0.2 to 2.5)
Lower respiratory infections	Estonia	1990	Age-standardized		10.2% (3.0 to 21.3%)	1.4 (0.4 to 2.9)
Lower respiratory infections	Estonia	1995	Age-standardized		7.5% (1.5 to 18.1%)	1.4 (0.3 to 3.5)
Lower respiratory infections	Estonia	2000	Age-standardized		6.4% (1.0 to 17.1%)	1.2 (0.2 to 3.2)
Lower respiratory infections	Estonia	2005	Age-standardized		6.4% (1.0 to 16.9%)	0.9 (0.1 to 2.3)
Lower respiratory infections	Estonia	2010	Age-standardized		6.6% (1.1 to 17.2%)	0.6 (0.1 to 1.7)
Lower respiratory infections	Estonia	2015	Age-standardized		7.9% (1.6 to 18.9%)	0.7 (0.1 to 1.6)
Tracheal, bronchus, and lung cancer	Estonia	1990	All Ages	0.1 (0.0 to 0.1)	6.9% (3.2 to 11.8%)	3.1 (1.4 to 5.3)
Tracheal, bronchus, and lung cancer	Estonia	1995	All Ages	0.0 (0.0 to 0.1)	5.0% (2.1 to 9.2%)	2.7 (1.1 to 5.0)
Tracheal, bronchus, and lung cancer	Estonia	2000	All Ages	0.0 (0.0 to 0.1)	4.3% (1.7 to 8.0%)	2.2 (0.9 to 4.2)
Tracheal, bronchus, and lung cancer	Estonia	2005	All Ages	0.0 (0.0 to 0.1)	4.2% (1.6 to 8.1%)	2.2 (0.8 to 4.2)
Tracheal, bronchus, and lung cancer	Estonia	2010	All Ages	0.0 (0.0 to 0.1)	4.4% (1.7 to 8.4%)	2.0 (0.8 to 3.8)
Tracheal, bronchus, and lung cancer	Estonia	2015	All Ages	0.0 (0.0 to 0.1)	5.3% (2.2 to 9.6%)	2.3 (1.0 to 4.2)
Tracheal, bronchus, and lung cancer	Estonia	1990	Age-standardized		6.9% (3.2 to 11.8%)	2.6 (1.2 to 4.4)
Tracheal, bronchus, and lung cancer	Estonia	1995	Age-standardized		5.0% (2.1 to 9.2%)	2.1 (0.9 to 3.8)
Tracheal, bronchus, and lung cancer	Estonia	2000	Age-standardized		4.3% (1.7 to 8.0%)	1.6 (0.6 to 2.9)
Tracheal, bronchus, and lung cancer	Estonia	2005	Age-standardized		4.2% (1.6 to 8.1%)	1.5 (0.6 to 2.8)
Tracheal, bronchus, and lung cancer	Estonia	2010	Age-standardized		4.4% (1.7 to 8.4%)	1.3 (0.5 to 2.5)
Tracheal, bronchus, and lung cancer	Estonia	2015	Age-standardized		5.3% (2.2 to 9.5%)	1.4 (0.6 to 2.5)
Cardiovascular diseases	Estonia	1990	All Ages	1.1 (0.9 to 1.4)	9.6% (7.6 to 11.8%)	70.2 (55.2 to 86.1)
Cardiovascular diseases	Estonia	1995	All Ages	1.0 (0.8 to 1.3)	8.4% (6.5 to 10.5%)	71.5 (55.0 to 89.4)
Cardiovascular diseases	Estonia	2000	All Ages	0.8 (0.6 to 1.0)	7.5% (5.6 to 9.5%)	59.1 (44.3 to 74.8)
Cardiovascular diseases	Estonia	2005	All Ages	0.7 (0.5 to 0.9)	7.0% (5.0 to 9.1%)	50.7 (36.8 to 66.1)
Cardiovascular diseases	Estonia	2010	All Ages	0.5 (0.4 to 0.7)	6.3% (4.4 to 8.4%)	37.9 (26.4 to 51.0)
Cardiovascular diseases	Estonia	2015	All Ages	0.6 (0.4 to 0.8)	6.9% (4.7 to 9.3%)	44.6 (30.5 to 60.6)
Cardiovascular diseases	Estonia	1990	Age-standardized		9.7% (7.6 to 11.8%)	59.5 (46.8 to 73.2)
Cardiovascular diseases	Estonia	1995	Age-standardized		8.4% (6.5 to 10.5%)	56.1 (43.2 to 70.4)
Cardiovascular diseases	Estonia	2000	Age-standardized		7.5% (5.5 to 9.4%)	43.4 (32.0 to 55.1)
Cardiovascular diseases	Estonia	2005	Age-standardized		7.0% (5.1 to 9.1%)	33.4 (24.2 to 43.5)
Cardiovascular diseases	Estonia	2010	Age-standardized		6.4% (4.6 to 8.4%)	22.2 (16.0 to 29.3)

Cause name	Location	Year	Age group	Number (in thousands)	PAF	Rate (per 100,000)
Cardiovascular diseases	Estonia	2015	Age-standardized		7.1% (5.1 to 9.2%)	23.0 (16.6 to 30.3)
Ischemic heart disease	Estonia	1990	All Ages	0.8 (0.6 to 1.0)	11.3% (8.4 to 14.4%)	51.7 (37.9 to 65.8)
Ischemic heart disease	Estonia	1995	All Ages	0.8 (0.6 to 1.0)	10.1% (7.3 to 12.9%)	53.4 (38.8 to 68.7)
Ischemic heart disease	Estonia	2000	All Ages	0.6 (0.4 to 0.8)	9.2% (6.4 to 12.0%)	44.4 (31.5 to 58.5)
Ischemic heart disease	Estonia	2005	All Ages	0.5 (0.4 to 0.7)	8.9% (6.1 to 12.0%)	39.2 (27.0 to 53.0)
Ischemic heart disease	Estonia	2010	All Ages	0.4 (0.3 to 0.6)	8.7% (5.6 to 12.1%)	30.7 (19.4 to 42.7)
Ischemic heart disease	Estonia	2015	All Ages	0.5 (0.3 to 0.7)	9.3% (5.8 to 13.1%)	36.3 (22.4 to 51.0)
Ischemic heart disease	Estonia	1990	Age-standardized		11.4% (8.4 to 14.5%)	43.8 (32.3 to 56.0)
Ischemic heart disease	Estonia	1995	Age-standardized		10.1% (7.2 to 12.9%)	41.9 (30.3 to 53.6)
Ischemic heart disease	Estonia	2000	Age-standardized		9.1% (6.2 to 12.0%)	32.7 (22.5 to 43.2)
Ischemic heart disease	Estonia	2005	Age-standardized		9.0% (6.2 to 12.1%)	25.9 (17.9 to 35.0)
Ischemic heart disease	Estonia	2010	Age-standardized		9.0% (6.0 to 12.1%)	18.0 (12.0 to 24.5)
Ischemic heart disease	Estonia	2015	Age-standardized		9.7% (6.4 to 13.0%)	18.7 (12.4 to 25.3)
Ischemic stroke	Estonia	1990	All Ages	0.2 (0.1 to 0.3)	7.8% (4.8 to 11.2%)	14.4 (9.0 to 20.9)
Ischemic stroke	Estonia	1995	All Ages	0.2 (0.1 to 0.3)	6.7% (3.8 to 9.9%)	13.6 (7.9 to 20.4)
Ischemic stroke	Estonia	2000	All Ages	0.2 (0.1 to 0.2)	6.1% (3.3 to 9.1%)	11.4 (6.3 to 17.4)
Ischemic stroke	Estonia	2005	All Ages	0.1 (0.1 to 0.2)	5.9% (3.2 to 9.2%)	9.0 (4.9 to 14.1)
Ischemic stroke	Estonia	2010	All Ages	0.1 (0.0 to 0.1)	5.9% (3.1 to 9.7%)	5.6 (2.9 to 9.0)
Ischemic stroke	Estonia	2015	All Ages	0.1 (0.0 to 0.1)	6.4% (3.2 to 10.7%)	6.5 (3.3 to 10.8)
Ischemic stroke	Estonia	1990	Age-standardized		7.8% (4.8 to 11.2%)	12.2 (7.5 to 17.6)
Ischemic stroke	Estonia	1995	Age-standardized		6.6% (3.8 to 9.9%)	10.6 (6.2 to 16.1)
Ischemic stroke	Estonia	2000	Age-standardized		6.0% (3.3 to 9.3%)	8.3 (4.5 to 12.9)
Ischemic stroke	Estonia	2005	Age-standardized		5.9% (3.2 to 9.3%)	5.8 (3.2 to 9.2)
Ischemic stroke	Estonia	2010	Age-standardized		6.0% (3.2 to 9.6%)	3.2 (1.7 to 5.1)
Ischemic stroke	Estonia	2015	Age-standardized		6.6% (3.6 to 10.4%)	3.3 (1.8 to 5.2)
Hemorrhagic stroke	Estonia	1990	All Ages	0.1 (0.1 to 0.1)	9.1% (6.6 to 11.9%)	4.1 (2.9 to 5.5)
Hemorrhagic stroke	Estonia	1995	All Ages	0.1 (0.0 to 0.1)	7.9% (5.3 to 10.5%)	4.5 (3.0 to 6.1)
Hemorrhagic stroke	Estonia	2000	All Ages	0.0 (0.0 to 0.1)	7.1% (4.7 to 9.8%)	3.3 (2.2 to 4.5)
Hemorrhagic stroke	Estonia	2005	All Ages	0.0 (0.0 to 0.1)	6.9% (4.6 to 9.6%)	2.5 (1.6 to 3.4)
Hemorrhagic stroke	Estonia	2010	All Ages	0.0 (0.0 to 0.0)	6.8% (4.4 to 9.5%)	1.6 (1.0 to 2.3)
Hemorrhagic stroke	Estonia	2015	All Ages	0.0 (0.0 to 0.0)	7.3% (4.6 to 10.5%)	1.8 (1.1 to 2.6)
Hemorrhagic stroke	Estonia	1990	Age-standardized		9.2% (6.6 to 12.0%)	3.5 (2.5 to 4.7)
Hemorrhagic stroke	Estonia	1995	Age-standardized		7.9% (5.5 to 10.6%)	3.6 (2.5 to 4.8)
Hemorrhagic stroke	Estonia	2000	Age-standardized		7.2% (4.8 to 9.9%)	2.4 (1.6 to 3.4)
Hemorrhagic stroke	Estonia	2005	Age-standardized		7.1% (4.7 to 9.8%)	1.7 (1.2 to 2.4)
Hemorrhagic stroke	Estonia	2010	Age-standardized		7.1% (4.7 to 9.8%)	1.1 (0.7 to 1.5)

Cause name	Location	Year	Age group	Number (in thousands)	PAF	Rate (per 100,000)
Hemorrhagic stroke	Estonia	2015	Age-standardized		7.7% (5.1 to 10.5%)	1.0 (0.7 to 1.4)
Chronic obstructive pulmonary disease	Estonia	1990	All Ages	0.0 (0.0 to 0.1)	12.3% (4.6 to 22.1%)	1.9 (0.7 to 3.4)
Chronic obstructive pulmonary disease	Estonia	1995	All Ages	0.0 (0.0 to 0.1)	9.7% (3.0 to 18.9%)	1.6 (0.5 to 3.1)
Chronic obstructive pulmonary disease	Estonia	2000	All Ages	0.0 (0.0 to 0.0)	8.6% (2.5 to 17.3%)	1.3 (0.4 to 2.6)
Chronic obstructive pulmonary disease	Estonia	2005	All Ages	0.0 (0.0 to 0.0)	8.5% (2.2 to 17.5%)	1.4 (0.4 to 2.8)
Chronic obstructive pulmonary disease	Estonia	2010	All Ages	0.0 (0.0 to 0.0)	8.8% (2.4 to 17.7%)	1.3 (0.3 to 2.6)
Chronic obstructive pulmonary disease	Estonia	2015	All Ages	0.0 (0.0 to 0.0)	10.2% (3.2 to 19.5%)	1.5 (0.5 to 2.9)
Chronic obstructive pulmonary disease	Estonia	1990	Age-standardized		12.3% (4.6 to 22.1%)	1.6 (0.6 to 2.8)
Chronic obstructive pulmonary disease	Estonia	1995	Age-standardized		9.7% (3.0 to 18.9%)	1.3 (0.4 to 2.4)
Chronic obstructive pulmonary disease	Estonia	2000	Age-standardized		8.6% (2.5 to 17.2%)	0.9 (0.3 to 1.8)
Chronic obstructive pulmonary disease	Estonia	2005	Age-standardized		8.5% (2.2 to 17.5%)	0.9 (0.2 to 1.8)
Chronic obstructive pulmonary disease	Estonia	2010	Age-standardized		8.7% (2.4 to 17.7%)	0.7 (0.2 to 1.5)
Chronic obstructive pulmonary disease	Estonia	2015	Age-standardized		10.2% (3.2 to 19.5%)	0.8 (0.3 to 1.5)
All causes	Latvia	1990	All Ages	3.3 (2.8 to 3.9)	9.5% (8.0 to 11.1%)	124.7 (105.2 to 145.4)
All causes	Latvia	1995	All Ages	3.3 (2.8 to 3.9)	8.4% (7.0 to 9.8%)	133.1 (111.3 to 155.2)
All causes	Latvia	2000	All Ages	2.4 (2.0 to 2.9)	7.5% (6.2 to 8.9%)	102.0 (84.2 to 120.0)
All causes	Latvia	2005	All Ages	2.4 (2.0 to 2.8)	7.2% (5.9 to 8.5%)	103.1 (84.9 to 121.9)
All causes	Latvia	2010	All Ages	2.0 (1.6 to 2.4)	7.0% (5.7 to 8.4%)	89.7 (72.6 to 106.6)
All causes	Latvia	2015	All Ages	2.1 (1.7 to 2.5)	7.3% (5.9 to 8.9%)	94.5 (76.1 to 114.4)
All causes	Latvia	1990	Age-standardized		9.3% (7.9 to 10.7%)	101.0 (85.6 to 117.3)
All causes	Latvia	1995	Age-standardized		8.1% (6.8 to 9.4%)	103.5 (87.6 to 119.8)
All causes	Latvia	2000	Age-standardized		7.2% (5.9 to 8.5%)	75.2 (61.7 to 89.1)
All causes	Latvia	2005	Age-standardized		6.9% (5.7 to 8.2%)	69.2 (56.8 to 81.8)
All causes	Latvia	2010	Age-standardized		6.7% (5.5 to 7.9%)	54.8 (44.9 to 64.6)
All causes	Latvia	2015	Age-standardized		7.0% (5.8 to 8.3%)	52.3 (42.9 to 61.9)
Lower respiratory infections	Latvia	1990	All Ages	0.1 (0.1 to 0.1)	22.7% (14.4 to 32.1%)	3.4 (2.1 to 4.7)
Lower respiratory infections	Latvia	1995	All Ages	0.1 (0.1 to 0.1)	17.8% (9.3 to 28.0%)	3.6 (1.9 to 5.7)
Lower respiratory infections	Latvia	2000	All Ages	0.1 (0.0 to 0.1)	15.5% (7.2 to 26.0%)	2.9 (1.4 to 5.0)
Lower respiratory infections	Latvia	2005	All Ages	0.1 (0.0 to 0.1)	14.9% (6.5 to 25.3%)	3.0 (1.3 to 5.3)
Lower respiratory infections	Latvia	2010	All Ages	0.1 (0.0 to 0.1)	15.6% (7.0 to 25.9%)	2.7 (1.2 to 4.6)
Lower respiratory infections	Latvia	2015	All Ages	0.1 (0.0 to 0.1)	17.0% (8.3 to 27.2%)	2.8 (1.4 to 4.6)
Lower respiratory infections	Latvia	1990	Age-standardized		22.7% (14.4 to 32.1%)	3.0 (1.9 to 4.2)
Lower respiratory infections	Latvia	1995	Age-standardized		17.8% (9.3 to 28.0%)	3.3 (1.7 to 5.2)
Lower respiratory infections	Latvia	2000	Age-standardized		15.5% (7.2 to 26.0%)	2.5 (1.2 to 4.3)
Lower respiratory infections	Latvia	2005	Age-standardized		14.9% (6.5 to 25.3%)	2.4 (1.0 to 4.1)
Lower respiratory infections	Latvia	2010	Age-standardized		15.6% (7.0 to 25.9%)	2.0 (0.9 to 3.3)

Cause name	Location	Year	Age group	Number (in thousands)	PAF	Rate (per 100,000)
Lower respiratory infections	Latvia	2015	Age-standardized		17.0% (8.3 to 27.2%)	1.9 (0.9 to 3.0)
Tracheal, bronchus, and lung cancer	Latvia	1990	All Ages	0.2 (0.1 to 0.3)	16.2% (9.6 to 23.5%)	6.8 (4.1 to 9.8)
Tracheal, bronchus, and lung cancer	Latvia	1995	All Ages	0.2 (0.1 to 0.2)	12.5% (6.8 to 19.0%)	6.3 (3.4 to 9.6)
Tracheal, bronchus, and lung cancer	Latvia	2000	All Ages	0.1 (0.1 to 0.2)	10.8% (5.6 to 16.9%)	4.8 (2.5 to 7.5)
Tracheal, bronchus, and lung cancer	Latvia	2005	All Ages	0.1 (0.1 to 0.2)	10.4% (5.4 to 16.3%)	5.1 (2.7 to 8.0)
Tracheal, bronchus, and lung cancer	Latvia	2010	All Ages	0.1 (0.1 to 0.2)	10.9% (5.8 to 16.8%)	4.9 (2.6 to 7.5)
Tracheal, bronchus, and lung cancer	Latvia	2015	All Ages	0.1 (0.1 to 0.2)	11.9% (6.5 to 18.2%)	5.1 (2.8 to 7.9)
Tracheal, bronchus, and lung cancer	Latvia	1990	Age-standardized		16.2% (9.6 to 23.5%)	5.5 (3.3 to 8.0)
Tracheal, bronchus, and lung cancer	Latvia	1995	Age-standardized		12.5% (6.8 to 19.0%)	4.8 (2.6 to 7.3)
Tracheal, bronchus, and lung cancer	Latvia	2000	Age-standardized		10.8% (5.6 to 16.9%)	3.4 (1.8 to 5.2)
Tracheal, bronchus, and lung cancer	Latvia	2005	Age-standardized		10.3% (5.4 to 16.3%)	3.4 (1.8 to 5.4)
Tracheal, bronchus, and lung cancer	Latvia	2010	Age-standardized		10.8% (5.8 to 16.8%)	3.1 (1.6 to 4.9)
Tracheal, bronchus, and lung cancer	Latvia	2015	Age-standardized		11.9% (6.5 to 18.2%)	3.2 (1.7 to 4.8)
Cardiovascular diseases	Latvia	1990	All Ages	2.9 (2.4 to 3.4)	13.9% (11.5 to 16.4%)	109.6 (90.7 to 129.4)
Cardiovascular diseases	Latvia	1995	All Ages	3.0 (2.4 to 3.5)	12.7% (10.5 to 15.1%)	119.2 (97.8 to 140.9)
Cardiovascular diseases	Latvia	2000	All Ages	2.2 (1.8 to 2.6)	11.6% (9.4 to 13.7%)	92.0 (74.6 to 109.0)
Cardiovascular diseases	Latvia	2005	All Ages	2.1 (1.7 to 2.5)	11.0% (8.9 to 13.1%)	92.4 (74.7 to 110.6)
Cardiovascular diseases	Latvia	2010	All Ages	1.8 (1.4 to 2.2)	10.8% (8.6 to 13.1%)	79.6 (63.2 to 96.5)
Cardiovascular diseases	Latvia	2015	All Ages	1.9 (1.4 to 2.3)	11.1% (8.6 to 13.6%)	83.9 (65.2 to 103.5)
Cardiovascular diseases	Latvia	1990	Age-standardized		14.0% (11.7 to 16.5%)	88.5 (73.9 to 104.3)
Cardiovascular diseases	Latvia	1995	Age-standardized		12.7% (10.5 to 15.0%)	92.4 (76.5 to 108.5)
Cardiovascular diseases	Latvia	2000	Age-standardized		11.5% (9.3 to 13.7%)	67.7 (54.9 to 80.8)
Cardiovascular diseases	Latvia	2005	Age-standardized		11.0% (8.9 to 13.2%)	61.7 (49.8 to 74.0)
Cardiovascular diseases	Latvia	2010	Age-standardized		11.0% (8.9 to 13.1%)	48.2 (39.1 to 57.7)
Cardiovascular diseases	Latvia	2015	Age-standardized		11.3% (9.2 to 13.7%)	45.8 (36.9 to 55.3)
Ischemic heart disease	Latvia	1990	All Ages	2.0 (1.6 to 2.4)	16.2% (12.6 to 19.8%)	75.0 (58.5 to 92.1)
Ischemic heart disease	Latvia	1995	All Ages	2.1 (1.6 to 2.5)	15.2% (11.8 to 18.3%)	82.6 (64.2 to 100.0)
Ischemic heart disease	Latvia	2000	All Ages	1.5 (1.1 to 1.8)	13.9% (10.6 to 16.9%)	62.6 (47.7 to 76.8)
Ischemic heart disease	Latvia	2005	All Ages	1.5 (1.1 to 1.8)	13.5% (10.3 to 16.6%)	64.2 (48.6 to 79.5)
Ischemic heart disease	Latvia	2010	All Ages	1.3 (1.0 to 1.6)	13.4% (10.0 to 16.7%)	57.2 (42.6 to 71.5)
Ischemic heart disease	Latvia	2015	All Ages	1.3 (1.0 to 1.7)	13.6% (9.8 to 17.3%)	60.3 (43.8 to 77.7)
Ischemic heart disease	Latvia	1990	Age-standardized		16.4% (12.8 to 19.9%)	60.7 (47.6 to 74.2)
Ischemic heart disease	Latvia	1995	Age-standardized		15.2% (11.9 to 18.4%)	64.2 (50.4 to 77.8)
Ischemic heart disease	Latvia	2000	Age-standardized		13.8% (10.6 to 17.0%)	46.1 (34.9 to 56.8)
Ischemic heart disease	Latvia	2005	Age-standardized		13.7% (10.4 to 16.8%)	43.1 (32.7 to 53.2)
Ischemic heart disease	Latvia	2010	Age-standardized		13.8% (10.5 to 16.9%)	34.8 (26.5 to 42.9)

Cause name	Location	Year	Age group	Number (in thousands)	PAF	Rate (per 100,000)
Ischemic heart disease	Latvia	2015	Age-standardized		14.2% (10.7 to 17.6%)	33.1 (25.3 to 41.5)
Ischemic stroke	Latvia	1990	All Ages	0.6 (0.4 to 0.8)	11.9% (8.4 to 15.6%)	23.7 (16.7 to 30.9)
Ischemic stroke	Latvia	1995	All Ages	0.6 (0.4 to 0.8)	10.6% (7.2 to 14.1%)	24.1 (16.2 to 32.1)
Ischemic stroke	Latvia	2000	All Ages	0.5 (0.3 to 0.6)	9.7% (6.4 to 13.2%)	20.0 (13.2 to 27.0)
Ischemic stroke	Latvia	2005	All Ages	0.5 (0.3 to 0.6)	9.4% (6.1 to 13.0%)	20.3 (13.3 to 28.0)
Ischemic stroke	Latvia	2010	All Ages	0.4 (0.3 to 0.5)	9.6% (6.2 to 13.2%)	17.3 (11.3 to 24.1)
Ischemic stroke	Latvia	2015	All Ages	0.4 (0.3 to 0.6)	9.8% (6.2 to 13.9%)	18.4 (11.7 to 26.1)
Ischemic stroke	Latvia	1990	Age-standardized		12.0% (8.5 to 15.6%)	18.9 (13.4 to 24.4)
Ischemic stroke	Latvia	1995	Age-standardized		10.6% (7.2 to 14.0%)	18.5 (12.6 to 24.6)
Ischemic stroke	Latvia	2000	Age-standardized		9.6% (6.3 to 13.4%)	14.6 (9.4 to 20.3)
Ischemic stroke	Latvia	2005	Age-standardized		9.4% (6.1 to 13.1%)	13.2 (8.6 to 18.3)
Ischemic stroke	Latvia	2010	Age-standardized		9.7% (6.4 to 13.3%)	10.1 (6.7 to 13.8)
Ischemic stroke	Latvia	2015	Age-standardized		10.1% (6.8 to 13.9%)	9.5 (6.4 to 13.1)
Hemorrhagic stroke	Latvia	1990	All Ages	0.3 (0.2 to 0.4)	13.2% (10.3 to 16.4%)	10.9 (8.3 to 13.7)
Hemorrhagic stroke	Latvia	1995	All Ages	0.3 (0.2 to 0.4)	11.9% (8.9 to 14.9%)	12.5 (9.5 to 15.9)
Hemorrhagic stroke	Latvia	2000	All Ages	0.2 (0.2 to 0.3)	10.8% (8.0 to 13.8%)	9.5 (6.9 to 12.1)
Hemorrhagic stroke	Latvia	2005	All Ages	0.2 (0.1 to 0.2)	10.6% (7.8 to 13.5%)	7.9 (5.7 to 10.0)
Hemorrhagic stroke	Latvia	2010	All Ages	0.1 (0.1 to 0.1)	10.9% (8.1 to 13.8%)	5.1 (3.8 to 6.5)
Hemorrhagic stroke	Latvia	2015	All Ages	0.1 (0.1 to 0.1)	11.1% (8.0 to 14.1%)	5.3 (3.8 to 6.8)
Hemorrhagic stroke	Latvia	1990	Age-standardized		13.3% (10.4 to 16.2%)	8.9 (6.8 to 11.2)
Hemorrhagic stroke	Latvia	1995	Age-standardized		11.9% (9.1 to 14.8%)	9.7 (7.4 to 12.4)
Hemorrhagic stroke	Latvia	2000	Age-standardized		10.8% (8.0 to 13.9%)	7.0 (5.2 to 8.9)
Hemorrhagic stroke	Latvia	2005	Age-standardized		10.8% (8.1 to 13.7%)	5.4 (4.0 to 6.9)
Hemorrhagic stroke	Latvia	2010	Age-standardized		11.3% (8.5 to 14.2%)	3.3 (2.5 to 4.1)
Hemorrhagic stroke	Latvia	2015	Age-standardized		11.6% (8.8 to 14.6%)	3.1 (2.4 to 3.9)
Chronic obstructive pulmonary disease	Latvia	1990	All Ages	0.1 (0.1 to 0.2)	22.8% (12.2 to 33.8%)	4.9 (2.5 to 7.5)
Chronic obstructive pulmonary disease	Latvia	1995	All Ages	0.1 (0.1 to 0.2)	18.9% (9.3 to 29.8%)	4.0 (2.0 to 6.4)
Chronic obstructive pulmonary disease	Latvia	2000	All Ages	0.1 (0.0 to 0.1)	17.0% (7.8 to 27.7%)	2.3 (1.1 to 3.8)
Chronic obstructive pulmonary disease	Latvia	2005	All Ages	0.1 (0.0 to 0.1)	16.5% (7.4 to 27.1%)	2.5 (1.1 to 4.1)
Chronic obstructive pulmonary disease	Latvia	2010	All Ages	0.1 (0.0 to 0.1)	17.1% (7.9 to 27.6%)	2.4 (1.1 to 3.9)
Chronic obstructive pulmonary disease	Latvia	2015	All Ages	0.1 (0.0 to 0.1)	18.3% (8.6 to 29.1%)	2.6 (1.2 to 4.2)
Chronic obstructive pulmonary disease	Latvia	1990	Age-standardized		22.7% (12.2 to 33.8%)	4.0 (2.0 to 6.0)
Chronic obstructive pulmonary disease	Latvia	1995	Age-standardized		18.9% (9.3 to 29.7%)	3.1 (1.5 to 4.9)
Chronic obstructive pulmonary disease	Latvia	2000	Age-standardized		17.0% (7.8 to 27.7%)	1.7 (0.8 to 2.7)
Chronic obstructive pulmonary disease	Latvia	2005	Age-standardized		16.5% (7.4 to 27.1%)	1.6 (0.7 to 2.7)
Chronic obstructive pulmonary disease	Latvia	2010	Age-standardized		17.1% (7.9 to 27.5%)	1.5 (0.7 to 2.4)

Cause name	Location	Year	Age group	Number (in thousands)	PAF	Rate (per 100,000)
Chronic obstructive pulmonary disease	Latvia	2015	Age-standardized		18.3% (8.6 to 29.0%)	1.5 (0.7 to 2.3)
All causes	Lithuania	1990	All Ages	3.8 (3.1 to 4.5)	9.6% (8.0 to 11.4%)	102.4 (85.4 to 120.9)
All causes	Lithuania	1995	All Ages	3.8 (3.1 to 4.4)	8.2% (6.7 to 9.7%)	103.9 (85.6 to 122.3)
All causes	Lithuania	2000	All Ages	2.9 (2.3 to 3.4)	7.3% (5.9 to 8.6%)	81.6 (66.7 to 96.8)
All causes	Lithuania	2005	All Ages	2.9 (2.4 to 3.5)	6.9% (5.6 to 8.3%)	86.2 (70.0 to 102.9)
All causes	Lithuania	2010	All Ages	2.8 (2.2 to 3.3)	7.0% (5.6 to 8.4%)	84.8 (67.9 to 101.8)
All causes	Lithuania	2015	All Ages	2.7 (2.1 to 3.3)	7.1% (5.6 to 8.6%)	86.7 (68.3 to 105.5)
All causes	Lithuania	1990	Age-standardized		9.5% (7.9 to 11.2%)	90.9 (75.9 to 107.0)
All causes	Lithuania	1995	Age-standardized		8.0% (6.6 to 9.5%)	89.7 (73.8 to 105.8)
All causes	Lithuania	2000	Age-standardized		7.1% (5.7 to 8.4%)	66.6 (53.8 to 79.6)
All causes	Lithuania	2005	Age-standardized		6.7% (5.4 to 7.9%)	62.7 (50.9 to 74.8)
All causes	Lithuania	2010	Age-standardized		6.7% (5.5 to 8.0%)	54.5 (44.3 to 65.0)
All causes	Lithuania	2015	Age-standardized		6.7% (5.5 to 8.0%)	49.1 (40.0 to 58.6)
Lower respiratory infections	Lithuania	1990	All Ages	0.1 (0.1 to 0.1)	23.1% (14.7 to 32.5%)	2.2 (1.4 to 3.1)
Lower respiratory infections	Lithuania	1995	All Ages	0.1 (0.0 to 0.1)	18.2% (9.3 to 28.3%)	2.2 (1.1 to 3.4)
Lower respiratory infections	Lithuania	2000	All Ages	0.1 (0.0 to 0.1)	15.7% (7.1 to 26.0%)	2.2 (1.0 to 3.7)
Lower respiratory infections	Lithuania	2005	All Ages	0.1 (0.0 to 0.2)	14.9% (6.6 to 25.3%)	2.8 (1.2 to 4.8)
Lower respiratory infections	Lithuania	2010	All Ages	0.1 (0.1 to 0.2)	15.9% (7.4 to 26.3%)	3.0 (1.4 to 5.0)
Lower respiratory infections	Lithuania	2015	All Ages	0.1 (0.0 to 0.1)	16.1% (7.5 to 26.3%)	3.0 (1.4 to 4.9)
Lower respiratory infections	Lithuania	1990	Age-standardized		23.1% (14.7 to 32.5%)	2.1 (1.3 to 2.9)
Lower respiratory infections	Lithuania	1995	Age-standardized		18.2% (9.3 to 28.3%)	2.1 (1.1 to 3.3)
Lower respiratory infections	Lithuania	2000	Age-standardized		15.7% (7.1 to 26.0%)	2.0 (0.9 to 3.4)
Lower respiratory infections	Lithuania	2005	Age-standardized		14.9% (6.6 to 25.3%)	2.3 (1.0 to 3.9)
Lower respiratory infections	Lithuania	2010	Age-standardized		15.9% (7.4 to 26.3%)	2.2 (1.0 to 3.6)
Lower respiratory infections	Lithuania	2015	Age-standardized		16.1% (7.5 to 26.3%)	2.0 (0.9 to 3.2)
Tracheal, bronchus, and lung cancer	Lithuania	1990	All Ages	0.2 (0.1 to 0.3)	16.5% (9.7 to 23.8%)	6.3 (3.7 to 9.2)
Tracheal, bronchus, and lung cancer	Lithuania	1995	All Ages	0.2 (0.1 to 0.3)	12.8% (7.1 to 19.4%)	5.7 (3.1 to 8.6)
Tracheal, bronchus, and lung cancer	Lithuania	2000	All Ages	0.1 (0.1 to 0.2)	10.9% (5.8 to 17.0%)	4.4 (2.3 to 6.7)
Tracheal, bronchus, and lung cancer	Lithuania	2005	All Ages	0.1 (0.1 to 0.2)	10.4% (5.4 to 16.5%)	4.5 (2.3 to 7.1)
Tracheal, bronchus, and lung cancer	Lithuania	2010	All Ages	0.1 (0.1 to 0.2)	11.1% (5.8 to 17.1%)	4.5 (2.4 to 7.1)
Tracheal, bronchus, and lung cancer	Lithuania	2015	All Ages	0.1 (0.1 to 0.2)	11.2% (6.0 to 17.4%)	4.5 (2.4 to 7.1)
Tracheal, bronchus, and lung cancer	Lithuania	1990	Age-standardized		16.5% (9.7 to 23.8%)	5.6 (3.3 to 8.1)
Tracheal, bronchus, and lung cancer	Lithuania	1995	Age-standardized		12.8% (7.1 to 19.4%)	4.8 (2.6 to 7.2)
Tracheal, bronchus, and lung cancer	Lithuania	2000	Age-standardized		10.9% (5.8 to 17.0%)	3.4 (1.8 to 5.2)
Tracheal, bronchus, and lung cancer	Lithuania	2005	Age-standardized		10.4% (5.4 to 16.5%)	3.3 (1.7 to 5.2)
Tracheal, bronchus, and lung cancer	Lithuania	2010	Age-standardized		11.1% (5.8 to 17.1%)	3.1 (1.6 to 4.9)

Cause name	Location	Year	Age group	Number (in thousands)	PAF	Rate (per 100,000)
Tracheal, bronchus, and lung cancer	Lithuania	2015	Age-standardized		11.2% (6.0 to 17.4%)	2.9 (1.5 to 4.5)
Cardiovascular diseases	Lithuania	1990	All Ages	3.1 (2.5 to 3.8)	14.5% (11.8 to 17.5%)	85.1 (68.7 to 102.7)
Cardiovascular diseases	Lithuania	1995	All Ages	3.2 (2.6 to 3.9)	13.1% (10.5 to 15.7%)	88.9 (71.2 to 107.3)
Cardiovascular diseases	Lithuania	2000	All Ages	2.5 (2.0 to 3.0)	11.8% (9.4 to 14.1%)	70.1 (55.9 to 84.6)
Cardiovascular diseases	Lithuania	2005	All Ages	2.5 (2.0 to 3.1)	11.3% (9.1 to 13.7%)	74.3 (59.4 to 90.1)
Cardiovascular diseases	Lithuania	2010	All Ages	2.4 (1.9 to 2.9)	11.3% (8.9 to 13.9%)	73.1 (56.9 to 89.8)
Cardiovascular diseases	Lithuania	2015	All Ages	2.4 (1.8 to 2.9)	11.2% (8.6 to 13.9%)	74.9 (56.9 to 93.0)
Cardiovascular diseases	Lithuania	1990	Age-standardized		14.6% (11.9 to 17.5%)	75.5 (61.4 to 90.8)
Cardiovascular diseases	Lithuania	1995	Age-standardized		13.0% (10.5 to 15.6%)	76.8 (61.9 to 92.4)
Cardiovascular diseases	Lithuania	2000	Age-standardized		11.7% (9.1 to 14.2%)	57.3 (44.9 to 69.9)
Cardiovascular diseases	Lithuania	2005	Age-standardized		11.4% (9.1 to 13.8%)	53.9 (42.8 to 65.7)
Cardiovascular diseases	Lithuania	2010	Age-standardized		11.6% (9.2 to 14.0%)	46.6 (37.1 to 56.5)
Cardiovascular diseases	Lithuania	2015	Age-standardized		11.6% (9.1 to 14.0%)	41.9 (33.1 to 51.0)
Ischemic heart disease	Lithuania	1990	All Ages	2.5 (1.9 to 3.1)	16.0% (12.2 to 19.9%)	68.5 (52.3 to 85.3)
Ischemic heart disease	Lithuania	1995	All Ages	2.6 (2.0 to 3.2)	14.7% (11.2 to 18.2%)	71.1 (53.8 to 88.2)
Ischemic heart disease	Lithuania	2000	All Ages	1.9 (1.5 to 2.4)	13.6% (10.2 to 16.8%)	55.0 (41.5 to 68.4)
Ischemic heart disease	Lithuania	2005	All Ages	2.0 (1.5 to 2.5)	13.3% (10.0 to 16.6%)	58.3 (43.4 to 73.0)
Ischemic heart disease	Lithuania	2010	All Ages	1.9 (1.4 to 2.4)	13.3% (9.8 to 17.0%)	57.2 (41.9 to 72.8)
Ischemic heart disease	Lithuania	2015	All Ages	1.9 (1.3 to 2.4)	13.2% (9.4 to 16.9%)	59.1 (41.7 to 75.7)
Ischemic heart disease	Lithuania	1990	Age-standardized		16.1% (12.5 to 19.9%)	60.6 (47.0 to 75.1)
Ischemic heart disease	Lithuania	1995	Age-standardized		14.7% (11.1 to 18.2%)	61.4 (46.3 to 76.1)
Ischemic heart disease	Lithuania	2000	Age-standardized		13.4% (9.9 to 16.8%)	45.1 (33.2 to 56.9)
Ischemic heart disease	Lithuania	2005	Age-standardized		13.4% (10.0 to 16.7%)	42.4 (31.5 to 53.0)
Ischemic heart disease	Lithuania	2010	Age-standardized		13.7% (10.3 to 17.2%)	36.6 (27.2 to 45.8)
Ischemic heart disease	Lithuania	2015	Age-standardized		13.7% (10.3 to 17.1%)	33.1 (24.6 to 41.6)
Ischemic stroke	Lithuania	1990	All Ages	0.4 (0.3 to 0.6)	12.3% (8.9 to 15.8%)	12.0 (8.7 to 15.5)
Ischemic stroke	Lithuania	1995	All Ages	0.5 (0.3 to 0.6)	10.8% (7.5 to 14.3%)	12.5 (8.7 to 16.6)
Ischemic stroke	Lithuania	2000	All Ages	0.4 (0.3 to 0.5)	9.8% (6.6 to 13.3%)	11.2 (7.5 to 15.1)
Ischemic stroke	Lithuania	2005	All Ages	0.4 (0.3 to 0.6)	9.5% (6.3 to 13.0%)	12.1 (8.0 to 16.7)
Ischemic stroke	Lithuania	2010	All Ages	0.4 (0.3 to 0.6)	9.7% (6.3 to 13.5%)	12.2 (7.9 to 17.2)
Ischemic stroke	Lithuania	2015	All Ages	0.4 (0.2 to 0.6)	9.6% (6.0 to 13.7%)	12.2 (7.7 to 17.5)
Ischemic stroke	Lithuania	1990	Age-standardized		12.4% (9.0 to 15.8%)	10.7 (7.8 to 13.6)
Ischemic stroke	Lithuania	1995	Age-standardized		10.7% (7.5 to 14.2%)	10.8 (7.5 to 14.3)
Ischemic stroke	Lithuania	2000	Age-standardized		9.7% (6.3 to 13.4%)	9.0 (5.9 to 12.6)
Ischemic stroke	Lithuania	2005	Age-standardized		9.5% (6.3 to 13.1%)	8.6 (5.6 to 11.9)
Ischemic stroke	Lithuania	2010	Age-standardized		9.8% (6.6 to 13.5%)	7.5 (5.0 to 10.4)

Cause name	Location	Year	Age group	Number (in thousands)	PAF	Rate (per 100,000)
Ischemic stroke	Lithuania	2015	Age-standardized		9.9% (6.5 to 13.6%)	6.6 (4.3 to 9.1)
Hemorrhagic stroke	Lithuania	1990	All Ages	0.2 (0.1 to 0.2)	14.8% (11.8 to 17.7%)	4.6 (3.7 to 5.7)
Hemorrhagic stroke	Lithuania	1995	All Ages	0.2 (0.1 to 0.2)	12.9% (10.1 to 15.8%)	5.3 (4.2 to 6.6)
Hemorrhagic stroke	Lithuania	2000	All Ages	0.1 (0.1 to 0.2)	11.5% (8.8 to 14.2%)	3.9 (2.9 to 4.9)
Hemorrhagic stroke	Lithuania	2005	All Ages	0.1 (0.1 to 0.2)	11.4% (8.6 to 14.2%)	3.9 (2.9 to 4.8)
Hemorrhagic stroke	Lithuania	2010	All Ages	0.1 (0.1 to 0.1)	11.4% (8.7 to 14.3%)	3.6 (2.7 to 4.6)
Hemorrhagic stroke	Lithuania	2015	All Ages	0.1 (0.1 to 0.1)	11.3% (8.5 to 14.2%)	3.6 (2.7 to 4.6)
Hemorrhagic stroke	Lithuania	1990	Age-standardized		14.8% (11.9 to 17.7%)	4.2 (3.4 to 5.1)
Hemorrhagic stroke	Lithuania	1995	Age-standardized		12.9% (10.2 to 15.7%)	4.6 (3.6 to 5.7)
Hemorrhagic stroke	Lithuania	2000	Age-standardized		11.6% (8.8 to 14.4%)	3.2 (2.4 to 4.0)
Hemorrhagic stroke	Lithuania	2005	Age-standardized		11.6% (8.9 to 14.4%)	2.9 (2.2 to 3.6)
Hemorrhagic stroke	Lithuania	2010	Age-standardized		11.9% (9.1 to 14.7%)	2.5 (1.9 to 3.1)
Hemorrhagic stroke	Lithuania	2015	Age-standardized		11.9% (9.1 to 14.8%)	2.2 (1.7 to 2.8)
Chronic obstructive pulmonary disease	Lithuania	1990	All Ages	0.3 (0.2 to 0.5)	23.1% (12.4 to 34.2%)	8.7 (4.7 to 13.0)
Chronic obstructive pulmonary disease	Lithuania	1995	All Ages	0.3 (0.1 to 0.4)	19.3% (9.3 to 30.1%)	7.0 (3.4 to 11.0)
Chronic obstructive pulmonary disease	Lithuania	2000	All Ages	0.2 (0.1 to 0.3)	17.2% (7.8 to 28.0%)	5.0 (2.3 to 8.1)
Chronic obstructive pulmonary disease	Lithuania	2005	All Ages	0.2 (0.1 to 0.3)	16.6% (7.5 to 27.2%)	4.6 (2.0 to 7.5)
Chronic obstructive pulmonary disease	Lithuania	2010	All Ages	0.1 (0.1 to 0.2)	17.4% (7.9 to 28.1%)	4.2 (1.9 to 6.7)
Chronic obstructive pulmonary disease	Lithuania	2015	All Ages	0.1 (0.1 to 0.2)	17.6% (8.2 to 28.3%)	4.3 (2.0 to 6.9)
Chronic obstructive pulmonary disease	Lithuania	1990	Age-standardized		23.1% (12.3 to 34.1%)	7.7 (4.2 to 11.5)
Chronic obstructive pulmonary disease	Lithuania	1995	Age-standardized		19.2% (9.3 to 30.1%)	6.0 (2.9 to 9.4)
Chronic obstructive pulmonary disease	Lithuania	2000	Age-standardized		17.2% (7.8 to 28.0%)	4.0 (1.8 to 6.4)
Chronic obstructive pulmonary disease	Lithuania	2005	Age-standardized		16.6% (7.5 to 27.2%)	3.3 (1.5 to 5.3)
Chronic obstructive pulmonary disease	Lithuania	2010	Age-standardized		17.4% (7.9 to 28.1%)	2.6 (1.2 to 4.2)
Chronic obstructive pulmonary disease	Lithuania	2015	Age-standardized		17.6% (8.2 to 28.3%)	2.4 (1.1 to 3.9)
All causes	Moldova	1990	All Ages	4.1 (3.6 to 4.8)	9.7% (8.3 to 11.1%)	94.9 (81.3 to 109.1)
All causes	Moldova	1995	All Ages	4.6 (3.9 to 5.4)	8.9% (7.5 to 10.3%)	106.6 (90.2 to 123.6)
All causes	Moldova	2000	All Ages	3.5 (2.9 to 4.1)	8.3% (6.9 to 9.7%)	82.7 (68.9 to 96.7)
All causes	Moldova	2005	All Ages	3.4 (2.8 to 4.0)	7.6% (6.3 to 9.0%)	81.1 (66.8 to 96.2)
All causes	Moldova	2010	All Ages	3.5 (2.9 to 4.1)	7.8% (6.4 to 9.2%)	85.8 (70.7 to 101.2)
All causes	Moldova	2015	All Ages	2.9 (2.3 to 3.5)	7.6% (6.2 to 9.1%)	70.9 (57.7 to 84.9)
All causes	Moldova	1990	Age-standardized		10.0% (8.3 to 11.6%)	116.5 (97.8 to 136.5)
All causes	Moldova	1995	Age-standardized		9.0% (7.5 to 10.6%)	127.7 (106.4 to 150.0)
All causes	Moldova	2000	Age-standardized		8.4% (6.8 to 10.0%)	97.9 (79.7 to 116.7)
All causes	Moldova	2005	Age-standardized		7.6% (6.2 to 9.1%)	86.3 (70.0 to 103.2)
All causes	Moldova	2010	Age-standardized		7.8% (6.3 to 9.2%)	82.5 (67.1 to 97.5)

Cause name	Location	Year	Age group	Number (in thousands)	PAF	Rate (per 100,000)
All causes	Moldova	2015	Age-standardized		7.6% (6.2 to 9.2%)	64.1 (51.7 to 77.3)
Lower respiratory infections	Moldova	1990	All Ages	0.3 (0.2 to 0.4)	24.6% (17.0 to 32.9%)	6.3 (4.3 to 8.6)
Lower respiratory infections	Moldova	1995	All Ages	0.3 (0.1 to 0.4)	19.8% (12.4 to 28.7%)	5.7 (3.5 to 8.6)
Lower respiratory infections	Moldova	2000	All Ages	0.2 (0.1 to 0.3)	17.3% (9.6 to 26.7%)	4.7 (2.6 to 7.2)
Lower respiratory infections	Moldova	2005	All Ages	0.1 (0.1 to 0.3)	15.1% (7.8 to 24.8%)	3.5 (1.8 to 5.9)
Lower respiratory infections	Moldova	2010	All Ages	0.1 (0.1 to 0.3)	16.5% (8.9 to 26.2%)	3.8 (2.1 to 6.1)
Lower respiratory infections	Moldova	2015	All Ages	0.1 (0.1 to 0.2)	16.4% (9.3 to 26.0%)	2.9 (1.6 to 4.7)
Lower respiratory infections	Moldova	1990	Age-standardized		24.6% (17.0 to 32.9%)	6.6 (4.5 to 9.0)
Lower respiratory infections	Moldova	1995	Age-standardized		19.8% (12.4 to 28.7%)	6.5 (4.0 to 9.9)
Lower respiratory infections	Moldova	2000	Age-standardized		17.3% (9.6 to 26.7%)	5.7 (3.2 to 9.0)
Lower respiratory infections	Moldova	2005	Age-standardized		15.1% (7.8 to 24.8%)	3.8 (1.9 to 6.4)
Lower respiratory infections	Moldova	2010	Age-standardized		16.5% (8.9 to 26.2%)	3.7 (2.0 to 6.0)
Lower respiratory infections	Moldova	2015	Age-standardized		16.4% (9.3 to 26.0%)	2.7 (1.5 to 4.4)
Tracheal, bronchus, and lung cancer	Moldova	1990	All Ages	0.2 (0.1 to 0.3)	17.8% (10.7 to 25.3%)	4.7 (2.8 to 6.8)
Tracheal, bronchus, and lung cancer	Moldova	1995	All Ages	0.2 (0.1 to 0.3)	14.0% (8.1 to 20.6%)	3.8 (2.2 to 5.7)
Tracheal, bronchus, and lung cancer	Moldova	2000	All Ages	0.1 (0.1 to 0.1)	12.1% (6.6 to 18.6%)	2.3 (1.3 to 3.6)
Tracheal, bronchus, and lung cancer	Moldova	2005	All Ages	0.1 (0.1 to 0.1)	10.4% (5.6 to 15.8%)	2.3 (1.2 to 3.6)
Tracheal, bronchus, and lung cancer	Moldova	2010	All Ages	0.1 (0.1 to 0.2)	11.5% (6.3 to 17.8%)	2.8 (1.5 to 4.4)
Tracheal, bronchus, and lung cancer	Moldova	2015	All Ages	0.1 (0.1 to 0.2)	11.4% (6.3 to 17.7%)	2.5 (1.4 to 3.9)
Tracheal, bronchus, and lung cancer	Moldova	1990	Age-standardized		17.8% (10.7 to 25.2%)	5.0 (3.0 to 7.1)
Tracheal, bronchus, and lung cancer	Moldova	1995	Age-standardized		14.0% (8.1 to 20.6%)	3.9 (2.3 to 5.9)
Tracheal, bronchus, and lung cancer	Moldova	2000	Age-standardized		12.1% (6.6 to 18.6%)	2.4 (1.3 to 3.6)
Tracheal, bronchus, and lung cancer	Moldova	2005	Age-standardized		10.4% (5.6 to 15.8%)	2.2 (1.2 to 3.4)
Tracheal, bronchus, and lung cancer	Moldova	2010	Age-standardized		11.5% (6.3 to 17.8%)	2.5 (1.3 to 3.9)
Tracheal, bronchus, and lung cancer	Moldova	2015	Age-standardized		11.4% (6.3 to 17.7%)	2.0 (1.1 to 3.2)
Cardiovascular diseases	Moldova	1990	All Ages	3.2 (2.7 to 3.8)	15.1% (12.7 to 17.6%)	74.3 (62.3 to 86.6)
Cardiovascular diseases	Moldova	1995	All Ages	3.8 (3.2 to 4.5)	13.7% (11.3 to 16.2%)	88.1 (73.2 to 104.5)
Cardiovascular diseases	Moldova	2000	All Ages	2.9 (2.4 to 3.5)	12.6% (10.3 to 15.0%)	69.4 (56.7 to 82.8)
Cardiovascular diseases	Moldova	2005	All Ages	2.9 (2.4 to 3.5)	11.6% (9.4 to 13.9%)	69.4 (56.3 to 83.1)
Cardiovascular diseases	Moldova	2010	All Ages	3.0 (2.4 to 3.6)	11.8% (9.5 to 14.1%)	73.4 (58.9 to 87.7)
Cardiovascular diseases	Moldova	2015	All Ages	2.5 (2.0 to 3.0)	11.6% (9.1 to 14.0%)	60.9 (47.9 to 73.7)
Cardiovascular diseases	Moldova	1990	Age-standardized		14.5% (11.8 to 17.4%)	93.2 (76.3 to 111.7)
Cardiovascular diseases	Moldova	1995	Age-standardized		13.1% (10.6 to 15.7%)	106.6 (86.0 to 127.9)
Cardiovascular diseases	Moldova	2000	Age-standardized		12.1% (9.6 to 14.7%)	82.4 (65.3 to 100.0)
Cardiovascular diseases	Moldova	2005	Age-standardized		11.3% (9.0 to 13.7%)	74.0 (59.0 to 89.6)
Cardiovascular diseases	Moldova	2010	Age-standardized		11.6% (9.2 to 13.9%)	70.7 (55.9 to 85.1)

Cause name	Location	Year	Age group	Number (in thousands)	PAF	Rate (per 100,000)
Cardiovascular diseases	Moldova	2015	Age-standardized		11.4% (8.9 to 14.0%)	55.2 (43.1 to 67.6)
Ischemic heart disease	Moldova	1990	All Ages	2.3 (1.9 to 2.8)	16.5% (13.3 to 20.0%)	53.3 (42.8 to 64.4)
Ischemic heart disease	Moldova	1995	All Ages	2.8 (2.2 to 3.5)	15.0% (11.9 to 18.4%)	64.6 (50.8 to 79.3)
Ischemic heart disease	Moldova	2000	All Ages	2.2 (1.7 to 2.7)	14.0% (10.9 to 17.1%)	51.4 (39.9 to 63.0)
Ischemic heart disease	Moldova	2005	All Ages	2.1 (1.7 to 2.7)	13.0% (10.1 to 16.2%)	51.5 (39.8 to 64.1)
Ischemic heart disease	Moldova	2010	All Ages	2.3 (1.7 to 2.8)	13.4% (10.2 to 16.5%)	55.4 (41.9 to 68.4)
Ischemic heart disease	Moldova	2015	All Ages	1.9 (1.4 to 2.4)	13.2% (9.8 to 16.5%)	46.5 (34.7 to 57.8)
Ischemic heart disease	Moldova	1990	Age-standardized		15.8% (12.1 to 19.6%)	67.8 (52.1 to 84.1)
Ischemic heart disease	Moldova	1995	Age-standardized		14.3% (10.8 to 17.9%)	79.3 (59.5 to 99.0)
Ischemic heart disease	Moldova	2000	Age-standardized		13.2% (9.8 to 16.7%)	62.2 (45.9 to 78.6)
Ischemic heart disease	Moldova	2005	Age-standardized		12.6% (9.4 to 15.8%)	55.5 (41.5 to 70.1)
Ischemic heart disease	Moldova	2010	Age-standardized		13.1% (9.9 to 16.4%)	53.7 (40.0 to 67.0)
Ischemic heart disease	Moldova	2015	Age-standardized		12.9% (9.6 to 16.3%)	42.4 (31.2 to 53.3)
Ischemic stroke	Moldova	1990	All Ages	0.5 (0.4 to 0.6)	13.1% (9.8 to 16.2%)	11.2 (8.4 to 13.8)
Ischemic stroke	Moldova	1995	All Ages	0.5 (0.4 to 0.7)	11.6% (8.6 to 14.5%)	11.7 (8.6 to 14.8)
Ischemic stroke	Moldova	2000	All Ages	0.4 (0.3 to 0.5)	10.7% (7.8 to 13.7%)	8.5 (6.2 to 10.9)
Ischemic stroke	Moldova	2005	All Ages	0.4 (0.3 to 0.5)	9.8% (6.9 to 12.7%)	9.2 (6.5 to 11.9)
Ischemic stroke	Moldova	2010	All Ages	0.4 (0.3 to 0.5)	10.1% (7.2 to 13.0%)	10.0 (7.1 to 12.9)
Ischemic stroke	Moldova	2015	All Ages	0.3 (0.2 to 0.4)	10.0% (7.0 to 13.0%)	8.2 (5.7 to 10.8)
Ischemic stroke	Moldova	1990	Age-standardized		12.5% (9.2 to 15.9%)	14.2 (10.2 to 18.0)
Ischemic stroke	Moldova	1995	Age-standardized		11.1% (8.0 to 14.2%)	14.4 (10.3 to 18.6)
Ischemic stroke	Moldova	2000	Age-standardized		10.2% (7.2 to 13.4%)	10.0 (7.0 to 13.2)
Ischemic stroke	Moldova	2005	Age-standardized		9.5% (6.6 to 12.5%)	9.8 (6.8 to 12.8)
Ischemic stroke	Moldova	2010	Age-standardized		10.0% (7.0 to 13.1%)	9.7 (6.8 to 12.7)
Ischemic stroke	Moldova	2015	Age-standardized		9.8% (6.7 to 13.0%)	7.5 (5.1 to 10.1)
Hemorrhagic stroke	Moldova	1990	All Ages	0.4 (0.3 to 0.5)	15.0% (11.9 to 18.1%)	9.8 (7.9 to 11.9)
Hemorrhagic stroke	Moldova	1995	All Ages	0.5 (0.4 to 0.6)	13.3% (10.3 to 16.3%)	11.8 (9.2 to 14.5)
Hemorrhagic stroke	Moldova	2000	All Ages	0.4 (0.3 to 0.5)	12.1% (9.3 to 15.1%)	9.5 (7.3 to 12.0)
Hemorrhagic stroke	Moldova	2005	All Ages	0.4 (0.3 to 0.5)	11.1% (8.4 to 14.0%)	8.7 (6.5 to 10.9)
Hemorrhagic stroke	Moldova	2010	All Ages	0.3 (0.3 to 0.4)	11.6% (8.9 to 14.5%)	8.0 (6.2 to 10.1)
Hemorrhagic stroke	Moldova	2015	All Ages	0.3 (0.2 to 0.3)	11.3% (8.5 to 14.4%)	6.3 (4.6 to 8.1)
Hemorrhagic stroke	Moldova	1990	Age-standardized		14.4% (11.4 to 17.5%)	11.2 (9.0 to 13.6)
Hemorrhagic stroke	Moldova	1995	Age-standardized		12.8% (10.0 to 15.7%)	12.9 (10.0 to 15.8)
Hemorrhagic stroke	Moldova	2000	Age-standardized		11.7% (8.9 to 14.6%)	10.2 (7.8 to 12.7)
Hemorrhagic stroke	Moldova	2005	Age-standardized		10.8% (8.2 to 13.7%)	8.6 (6.4 to 10.9)
Hemorrhagic stroke	Moldova	2010	Age-standardized		11.3% (8.6 to 14.2%)	7.3 (5.5 to 9.2)

Cause name	Location	Year	Age group	Number (in thousands)	PAF	Rate (per 100,000)
Hemorrhagic stroke	Moldova	2015	Age-standardized		11.1% (8.3 to 14.0%)	5.4 (4.0 to 6.9)
Chronic obstructive pulmonary disease	Moldova	1990	All Ages	0.4 (0.2 to 0.6)	24.0% (13.5 to 35.0%)	9.6 (5.4 to 14.1)
Chronic obstructive pulmonary disease	Moldova	1995	All Ages	0.4 (0.2 to 0.6)	20.1% (10.4 to 30.5%)	9.0 (4.7 to 13.5)
Chronic obstructive pulmonary disease	Moldova	2000	All Ages	0.3 (0.1 to 0.4)	18.1% (8.9 to 28.4%)	6.3 (3.1 to 9.9)
Chronic obstructive pulmonary disease	Moldova	2005	All Ages	0.3 (0.1 to 0.4)	16.2% (7.6 to 26.5%)	5.9 (2.8 to 9.6)
Chronic obstructive pulmonary disease	Moldova	2010	All Ages	0.2 (0.1 to 0.4)	17.4% (8.5 to 27.9%)	5.8 (2.8 to 9.1)
Chronic obstructive pulmonary disease	Moldova	2015	All Ages	0.2 (0.1 to 0.3)	17.4% (8.2 to 27.6%)	4.6 (2.2 to 7.3)
Chronic obstructive pulmonary disease	Moldova	1990	Age-standardized		24.0% (13.5 to 35.0%)	11.8 (6.6 to 17.3)
Chronic obstructive pulmonary disease	Moldova	1995	Age-standardized		20.1% (10.4 to 30.6%)	10.6 (5.5 to 16.0)
Chronic obstructive pulmonary disease	Moldova	2000	Age-standardized		18.1% (8.9 to 28.4%)	7.4 (3.6 to 11.7)
Chronic obstructive pulmonary disease	Moldova	2005	Age-standardized		16.2% (7.6 to 26.5%)	6.4 (3.0 to 10.4)
Chronic obstructive pulmonary disease	Moldova	2010	Age-standardized		17.4% (8.5 to 27.9%)	5.6 (2.7 to 8.9)
Chronic obstructive pulmonary disease	Moldova	2015	Age-standardized		17.3% (8.2 to 27.6%)	4.2 (2.0 to 6.7)
All causes	Russia	1990	All Ages	133.2 (113.3 to 154.1)	8.1% (6.9 to 9.4%)	90.3 (76.8 to 104.4)
All causes	Russia	1995	All Ages	162.0 (136.4 to 188.8)	7.2% (6.1 to 8.4%)	109.9 (92.5 to 128.0)
All causes	Russia	2000	All Ages	157.3 (132.1 to 182.9)	7.0% (5.9 to 8.1%)	108.0 (90.6 to 125.5)
All causes	Russia	2005	All Ages	157.7 (132.7 to 184.0)	6.8% (5.7 to 7.9%)	110.2 (92.7 to 128.6)
All causes	Russia	2010	All Ages	138.1 (114.7 to 162.1)	6.9% (5.7 to 8.1%)	97.3 (80.9 to 114.2)
All causes	Russia	2015	All Ages	136.9 (111.3 to 161.1)	6.8% (5.5 to 8.0%)	92.5 (75.1 to 108.7)
All causes	Russia	1990	Age-standardized		8.0% (6.7 to 9.3%)	87.2 (73.2 to 101.3)
All causes	Russia	1995	Age-standardized		7.0% (5.9 to 8.2%)	100.2 (83.5 to 117.3)
All causes	Russia	2000	Age-standardized		6.8% (5.6 to 8.0%)	94.7 (78.3 to 112.0)
All causes	Russia	2005	Age-standardized		6.6% (5.5 to 7.7%)	90.6 (75.0 to 106.2)
All causes	Russia	2010	Age-standardized		6.7% (5.6 to 7.9%)	73.8 (61.3 to 86.4)
All causes	Russia	2015	Age-standardized		6.5% (5.4 to 7.6%)	62.6 (51.8 to 73.2)
Lower respiratory infections	Russia	1990	All Ages	4.3 (2.4 to 6.6)	17.8% (9.9 to 27.5%)	2.9 (1.6 to 4.5)
Lower respiratory infections	Russia	1995	All Ages	4.8 (2.1 to 8.6)	14.2% (6.4 to 24.4%)	3.3 (1.4 to 5.8)
Lower respiratory infections	Russia	2000	All Ages	5.6 (2.3 to 10.2)	13.1% (5.4 to 23.5%)	3.8 (1.6 to 7.0)
Lower respiratory infections	Russia	2005	All Ages	6.2 (2.4 to 11.5)	12.8% (5.3 to 23.3%)	4.3 (1.7 to 8.0)
Lower respiratory infections	Russia	2010	All Ages	5.9 (2.6 to 10.3)	14.0% (6.3 to 24.2%)	4.1 (1.9 to 7.2)
Lower respiratory infections	Russia	2015	All Ages	5.8 (2.6 to 10.1)	14.1% (6.4 to 24.2%)	3.9 (1.8 to 6.8)
Lower respiratory infections	Russia	1990	Age-standardized		17.8% (9.9 to 27.5%)	3.0 (1.7 to 4.7)
Lower respiratory infections	Russia	1995	Age-standardized		14.2% (6.4 to 24.4%)	3.3 (1.4 to 5.8)
Lower respiratory infections	Russia	2000	Age-standardized		13.1% (5.4 to 23.5%)	3.7 (1.5 to 6.7)
Lower respiratory infections	Russia	2005	Age-standardized		12.8% (5.3 to 23.3%)	3.8 (1.5 to 7.1)
Lower respiratory infections	Russia	2010	Age-standardized		14.0% (6.3 to 24.2%)	3.4 (1.5 to 6.0)

Cause name	Location	Year	Age group	Number (in thousands)	PAF	Rate (per 100,000)
Lower respiratory infections	Russia	2015	Age-standardized		14.1% (6.4 to 24.2%)	3.0 (1.4 to 5.2)
Tracheal, bronchus, and lung cancer	Russia	1990	All Ages	7.7 (4.3 to 11.8)	12.5% (6.9 to 18.9%)	5.2 (2.9 to 8.0)
Tracheal, bronchus, and lung cancer	Russia	1995	All Ages	7.3 (3.8 to 11.5)	9.8% (5.1 to 15.5%)	5.0 (2.6 to 7.8)
Tracheal, bronchus, and lung cancer	Russia	2000	All Ages	5.7 (2.9 to 9.1)	9.0% (4.6 to 14.6%)	3.9 (2.0 to 6.3)
Tracheal, bronchus, and lung cancer	Russia	2005	All Ages	5.3 (2.7 to 8.6)	8.8% (4.5 to 14.3%)	3.7 (1.9 to 6.0)
Tracheal, bronchus, and lung cancer	Russia	2010	All Ages	5.2 (2.7 to 8.2)	9.7% (5.1 to 15.4%)	3.7 (1.9 to 5.8)
Tracheal, bronchus, and lung cancer	Russia	2015	All Ages	5.5 (2.8 to 8.7)	9.7% (5.0 to 15.5%)	3.7 (1.9 to 5.9)
Tracheal, bronchus, and lung cancer	Russia	1990	Age-standardized		12.5% (6.9 to 18.9%)	4.6 (2.6 to 7.0)
Tracheal, bronchus, and lung cancer	Russia	1995	Age-standardized		9.8% (5.1 to 15.5%)	4.2 (2.2 to 6.6)
Tracheal, bronchus, and lung cancer	Russia	2000	Age-standardized		9.0% (4.6 to 14.6%)	3.2 (1.6 to 5.1)
Tracheal, bronchus, and lung cancer	Russia	2005	Age-standardized		8.8% (4.5 to 14.3%)	2.9 (1.5 to 4.7)
Tracheal, bronchus, and lung cancer	Russia	2010	Age-standardized		9.7% (5.1 to 15.4%)	2.8 (1.4 to 4.4)
Tracheal, bronchus, and lung cancer	Russia	2015	Age-standardized		9.7% (5.0 to 15.4%)	2.5 (1.3 to 4.0)
Cardiovascular diseases	Russia	1990	All Ages	112.8 (93.4 to 131.3)	12.3% (10.2 to 14.3%)	76.4 (63.3 to 89.0)
Cardiovascular diseases	Russia	1995	All Ages	141.6 (116.8 to 167.0)	11.4% (9.4 to 13.4%)	96.1 (79.2 to 113.2)
Cardiovascular diseases	Russia	2000	All Ages	138.5 (114.2 to 162.3)	10.8% (8.9 to 12.7%)	95.0 (78.3 to 111.3)
Cardiovascular diseases	Russia	2005	All Ages	139.9 (115.5 to 165.7)	10.5% (8.7 to 12.4%)	97.8 (80.7 to 115.8)
Cardiovascular diseases	Russia	2010	All Ages	121.7 (99.1 to 144.3)	10.5% (8.6 to 12.5%)	85.8 (69.9 to 101.8)
Cardiovascular diseases	Russia	2015	All Ages	120.1 (95.8 to 143.9)	10.4% (8.3 to 12.5%)	81.1 (64.6 to 97.2)
Cardiovascular diseases	Russia	1990	Age-standardized		12.1% (9.9 to 14.3%)	74.1 (60.8 to 87.3)
Cardiovascular diseases	Russia	1995	Age-standardized		11.1% (9.1 to 13.1%)	87.7 (71.7 to 103.5)
Cardiovascular diseases	Russia	2000	Age-standardized		10.5% (8.5 to 12.5%)	83.4 (67.4 to 99.0)
Cardiovascular diseases	Russia	2005	Age-standardized		10.4% (8.5 to 12.2%)	80.3 (65.4 to 95.1)
Cardiovascular diseases	Russia	2010	Age-standardized		10.5% (8.6 to 12.5%)	64.8 (52.8 to 76.8)
Cardiovascular diseases	Russia	2015	Age-standardized		10.4% (8.5 to 12.4%)	54.5 (44.6 to 64.7)
Ischemic heart disease	Russia	1990	All Ages	74.4 (58.7 to 90.3)	14.8% (11.6 to 18.0%)	50.4 (39.8 to 61.2)
Ischemic heart disease	Russia	1995	All Ages	94.9 (75.7 to 113.4)	14.0% (11.2 to 16.8%)	64.3 (51.4 to 76.9)
Ischemic heart disease	Russia	2000	All Ages	91.5 (73.3 to 110.1)	13.5% (10.7 to 16.2%)	62.8 (50.3 to 75.6)
Ischemic heart disease	Russia	2005	All Ages	94.9 (75.6 to 114.2)	13.3% (10.6 to 16.0%)	66.3 (52.8 to 79.8)
Ischemic heart disease	Russia	2010	All Ages	84.7 (65.6 to 103.2)	13.3% (10.3 to 16.2%)	59.7 (46.3 to 72.7)
Ischemic heart disease	Russia	2015	All Ages	84.6 (63.6 to 104.7)	13.0% (9.8 to 16.1%)	57.1 (42.9 to 70.7)
Ischemic heart disease	Russia	1990	Age-standardized		14.5% (11.2 to 17.7%)	48.7 (37.6 to 59.7)
Ischemic heart disease	Russia	1995	Age-standardized		13.7% (10.8 to 16.5%)	58.4 (45.7 to 70.3)
Ischemic heart disease	Russia	2000	Age-standardized		13.1% (10.2 to 16.0%)	54.9 (42.7 to 66.4)
Ischemic heart disease	Russia	2005	Age-standardized		13.1% (10.3 to 15.9%)	54.4 (42.7 to 65.8)
Ischemic heart disease	Russia	2010	Age-standardized		13.3% (10.4 to 16.2%)	45.2 (35.1 to 55.0)

Cause name	Location	Year	Age group	Number (in thousands)	PAF	Rate (per 100,000)
Ischemic heart disease	Russia	2015	Age-standardized		13.2% (10.2 to 16.1%)	38.5 (29.5 to 47.2)
Ischemic stroke	Russia	1990	All Ages	27.9 (19.4 to 36.5)	10.5% (7.4 to 13.7%)	18.9 (13.1 to 24.7)
Ischemic stroke	Russia	1995	All Ages	33.5 (22.7 to 45.1)	9.4% (6.4 to 12.7%)	22.7 (15.4 to 30.6)
Ischemic stroke	Russia	2000	All Ages	32.2 (21.3 to 43.4)	9.0% (6.0 to 12.1%)	22.1 (14.6 to 29.8)
Ischemic stroke	Russia	2005	All Ages	31.2 (20.8 to 42.6)	8.9% (5.9 to 12.1%)	21.8 (14.5 to 29.8)
Ischemic stroke	Russia	2010	All Ages	26.3 (17.2 to 36.1)	9.1% (6.0 to 12.5%)	18.5 (12.1 to 25.4)
Ischemic stroke	Russia	2015	All Ages	25.5 (16.2 to 36.4)	9.0% (5.8 to 12.6%)	17.2 (10.9 to 24.6)
Ischemic stroke	Russia	1990	Age-standardized		10.3% (7.1 to 13.7%)	18.7 (12.8 to 24.8)
Ischemic stroke	Russia	1995	Age-standardized		9.2% (6.1 to 12.5%)	21.2 (14.0 to 29.0)
Ischemic stroke	Russia	2000	Age-standardized		8.7% (5.6 to 12.2%)	19.9 (12.7 to 27.8)
Ischemic stroke	Russia	2005	Age-standardized		8.7% (5.7 to 12.0%)	18.1 (11.7 to 25.2)
Ischemic stroke	Russia	2010	Age-standardized		9.1% (6.1 to 12.4%)	13.9 (9.2 to 19.1)
Ischemic stroke	Russia	2015	Age-standardized		9.0% (5.9 to 12.4%)	11.4 (7.4 to 15.8)
Hemorrhagic stroke	Russia	1990	All Ages	10.5 (7.9 to 13.2)	11.8% (9.0 to 14.8%)	7.1 (5.4 to 8.9)
Hemorrhagic stroke	Russia	1995	All Ages	13.3 (9.7 to 16.8)	10.7% (8.0 to 13.6%)	9.0 (6.6 to 11.4)
Hemorrhagic stroke	Russia	2000	All Ages	14.8 (10.8 to 19.1)	10.2% (7.5 to 13.2%)	10.1 (7.4 to 13.1)
Hemorrhagic stroke	Russia	2005	All Ages	13.8 (10.3 to 17.6)	10.2% (7.6 to 12.9%)	9.7 (7.2 to 12.3)
Hemorrhagic stroke	Russia	2010	All Ages	10.7 (8.0 to 13.6)	10.5% (7.9 to 13.3%)	7.5 (5.6 to 9.6)
Hemorrhagic stroke	Russia	2015	All Ages	10.0 (7.2 to 12.8)	10.3% (7.5 to 13.4%)	6.8 (4.9 to 8.7)
Hemorrhagic stroke	Russia	1990	Age-standardized		11.6% (8.8 to 14.5%)	6.7 (5.1 to 8.5)
Hemorrhagic stroke	Russia	1995	Age-standardized		10.5% (7.9 to 13.4%)	8.0 (5.9 to 10.1)
Hemorrhagic stroke	Russia	2000	Age-standardized		10.0% (7.3 to 12.8%)	8.7 (6.4 to 11.1)
Hemorrhagic stroke	Russia	2005	Age-standardized		10.1% (7.5 to 12.9%)	7.8 (5.8 to 9.9)
Hemorrhagic stroke	Russia	2010	Age-standardized		10.6% (7.9 to 13.3%)	5.7 (4.3 to 7.2)
Hemorrhagic stroke	Russia	2015	Age-standardized		10.5% (7.9 to 13.3%)	4.7 (3.5 to 5.9)
Chronic obstructive pulmonary disease	Russia	1990	All Ages	8.5 (4.2 to 13.3)	18.7% (9.1 to 29.2%)	5.7 (2.8 to 9.0)
Chronic obstructive pulmonary disease	Russia	1995	All Ages	8.3 (3.8 to 13.6)	15.8% (7.1 to 26.2%)	5.6 (2.6 to 9.2)
Chronic obstructive pulmonary disease	Russia	2000	All Ages	7.6 (3.3 to 12.7)	14.8% (6.5 to 24.9%)	5.2 (2.3 to 8.7)
Chronic obstructive pulmonary disease	Russia	2005	All Ages	6.3 (2.7 to 10.6)	14.6% (6.4 to 24.8%)	4.4 (1.9 to 7.4)
Chronic obstructive pulmonary disease	Russia	2010	All Ages	5.3 (2.4 to 8.9)	15.6% (6.9 to 26.1%)	3.7 (1.7 to 6.3)
Chronic obstructive pulmonary disease	Russia	2015	All Ages	5.6 (2.5 to 9.3)	15.6% (7.0 to 26.0%)	3.8 (1.7 to 6.3)
Chronic obstructive pulmonary disease	Russia	1990	Age-standardized		18.7% (9.1 to 29.2%)	5.5 (2.7 to 8.7)
Chronic obstructive pulmonary disease	Russia	1995	Age-standardized		15.7% (7.1 to 26.2%)	5.1 (2.3 to 8.4)
Chronic obstructive pulmonary disease	Russia	2000	Age-standardized		14.8% (6.5 to 24.9%)	4.4 (1.9 to 7.5)
Chronic obstructive pulmonary disease	Russia	2005	Age-standardized		14.6% (6.4 to 24.8%)	3.5 (1.5 to 5.9)
Chronic obstructive pulmonary disease	Russia	2010	Age-standardized		15.6% (6.9 to 26.1%)	2.8 (1.2 to 4.7)

Cause name	Location	Year	Age group	Number (in thousands)	PAF	Rate (per 100,000)
Chronic obstructive pulmonary disease	Russia	2015	Age-standardized		15.6% (7.0 to 26.0%)	2.5 (1.1 to 4.2)
All causes	Ukraine	1990	All Ages	65.0 (55.7 to 75.3)	10.2% (8.8 to 11.9%)	126.7 (108.5 to 146.7)
All causes	Ukraine	1995	All Ages	74.0 (62.1 to 85.7)	9.2% (7.7 to 10.7%)	145.1 (121.8 to 168.1)
All causes	Ukraine	2000	All Ages	66.0 (55.2 to 77.0)	8.7% (7.3 to 10.2%)	134.9 (112.8 to 157.4)
All causes	Ukraine	2005	All Ages	64.8 (53.9 to 76.3)	8.3% (6.9 to 9.8%)	138.0 (114.9 to 162.4)
All causes	Ukraine	2010	All Ages	57.7 (46.5 to 68.7)	8.5% (6.8 to 10.1%)	125.8 (101.4 to 150.0)
All causes	Ukraine	2015	All Ages	59.0 (46.5 to 71.5)	8.3% (6.6 to 10.1%)	126.8 (99.9 to 153.8)
All causes	Ukraine	1990	Age-standardized		10.0% (8.4 to 11.5%)	105.5 (89.7 to 122.0)
All causes	Ukraine	1995	Age-standardized		8.9% (7.5 to 10.3%)	117.6 (98.6 to 136.4)
All causes	Ukraine	2000	Age-standardized		8.3% (6.9 to 9.8%)	105.7 (87.0 to 124.0)
All causes	Ukraine	2005	Age-standardized		7.9% (6.5 to 9.3%)	99.6 (82.1 to 117.3)
All causes	Ukraine	2010	Age-standardized		8.1% (6.6 to 9.6%)	83.6 (68.2 to 98.7)
All causes	Ukraine	2015	Age-standardized		7.9% (6.4 to 9.4%)	75.9 (62.0 to 90.3)
Lower respiratory infections	Ukraine	1990	All Ages	1.6 (1.1 to 2.1)	23.7% (16.1 to 32.3%)	3.1 (2.1 to 4.2)
Lower respiratory infections	Ukraine	1995	All Ages	1.5 (0.9 to 2.3)	18.7% (10.8 to 28.2%)	2.9 (1.7 to 4.5)
Lower respiratory infections	Ukraine	2000	All Ages	1.4 (0.7 to 2.3)	16.4% (8.5 to 26.1%)	2.9 (1.5 to 4.7)
Lower respiratory infections	Ukraine	2005	All Ages	1.3 (0.6 to 2.2)	14.6% (7.0 to 24.5%)	2.8 (1.3 to 4.7)
Lower respiratory infections	Ukraine	2010	All Ages	1.1 (0.6 to 1.9)	15.8% (8.0 to 25.7%)	2.5 (1.2 to 4.1)
Lower respiratory infections	Ukraine	2015	All Ages	1.1 (0.5 to 1.8)	15.6% (7.8 to 25.4%)	2.3 (1.1 to 3.8)
Lower respiratory infections	Ukraine	1990	Age-standardized		23.7% (16.1 to 32.3%)	3.2 (2.2 to 4.3)
Lower respiratory infections	Ukraine	1995	Age-standardized		18.7% (10.8 to 28.2%)	3.0 (1.7 to 4.6)
Lower respiratory infections	Ukraine	2000	Age-standardized		16.4% (8.5 to 26.1%)	2.9 (1.5 to 4.7)
Lower respiratory infections	Ukraine	2005	Age-standardized		14.6% (7.0 to 24.5%)	2.4 (1.2 to 4.1)
Lower respiratory infections	Ukraine	2010	Age-standardized		15.8% (8.0 to 25.7%)	2.0 (1.0 to 3.3)
Lower respiratory infections	Ukraine	2015	Age-standardized		15.6% (7.8 to 25.4%)	1.7 (0.8 to 2.8)
Tracheal, bronchus, and lung cancer	Ukraine	1990	All Ages	4.0 (2.4 to 5.6)	17.0% (10.3 to 24.5%)	7.8 (4.7 to 11.0)
Tracheal, bronchus, and lung cancer	Ukraine	1995	All Ages	3.4 (2.0 to 5.1)	13.1% (7.5 to 19.4%)	6.7 (3.8 to 9.9)
Tracheal, bronchus, and lung cancer	Ukraine	2000	All Ages	2.3 (1.2 to 3.5)	11.4% (6.2 to 17.7%)	4.6 (2.5 to 7.2)
Tracheal, bronchus, and lung cancer	Ukraine	2005	All Ages	1.8 (1.0 to 2.9)	10.1% (5.3 to 16.0%)	3.9 (2.0 to 6.1)
Tracheal, bronchus, and lung cancer	Ukraine	2010	All Ages	1.6 (0.9 to 2.5)	11.0% (5.9 to 16.9%)	3.5 (1.9 to 5.4)
Tracheal, bronchus, and lung cancer	Ukraine	2015	All Ages	1.7 (0.9 to 2.6)	10.8% (5.9 to 16.7%)	3.7 (2.0 to 5.7)
Tracheal, bronchus, and lung cancer	Ukraine	1990	Age-standardized		17.0% (10.3 to 24.5%)	6.1 (3.7 to 8.6)
Tracheal, bronchus, and lung cancer	Ukraine	1995	Age-standardized		13.1% (7.5 to 19.4%)	5.1 (2.9 to 7.6)
Tracheal, bronchus, and lung cancer	Ukraine	2000	Age-standardized		11.4% (6.2 to 17.7%)	3.4 (1.8 to 5.3)
Tracheal, bronchus, and lung cancer	Ukraine	2005	Age-standardized		10.1% (5.3 to 16.0%)	2.8 (1.4 to 4.4)
Tracheal, bronchus, and lung cancer	Ukraine	2010	Age-standardized		11.0% (5.9 to 16.9%)	2.4 (1.3 to 3.7)

Cause name	Location	Year	Age group	Number (in thousands)	PAF	Rate (per 100,000)
Tracheal, bronchus, and lung cancer	Ukraine	2015	Age-standardized		10.8% (5.8 to 16.7%)	2.3 (1.2 to 3.6)
Cardiovascular diseases	Ukraine	1990	All Ages	52.7 (44.0 to 61.9)	14.3% (11.9 to 16.8%)	102.7 (85.8 to 120.7)
Cardiovascular diseases	Ukraine	1995	All Ages	62.3 (51.5 to 73.8)	13.0% (10.8 to 15.4%)	122.1 (101.0 to 144.7)
Cardiovascular diseases	Ukraine	2000	All Ages	57.5 (47.0 to 68.2)	12.4% (10.1 to 14.7%)	117.6 (96.1 to 139.4)
Cardiovascular diseases	Ukraine	2005	All Ages	58.4 (47.6 to 69.9)	11.8% (9.6 to 14.0%)	124.3 (101.5 to 148.8)
Cardiovascular diseases	Ukraine	2010	All Ages	52.5 (41.6 to 63.7)	11.9% (9.4 to 14.4%)	114.6 (90.8 to 139.0)
Cardiovascular diseases	Ukraine	2015	All Ages	53.7 (41.5 to 65.9)	11.7% (9.0 to 14.3%)	115.4 (89.3 to 141.7)
Cardiovascular diseases	Ukraine	1990	Age-standardized		14.1% (11.7 to 16.8%)	85.3 (71.0 to 101.1)
Cardiovascular diseases	Ukraine	1995	Age-standardized		12.9% (10.7 to 15.2%)	98.8 (81.8 to 116.7)
Cardiovascular diseases	Ukraine	2000	Age-standardized		12.1% (9.8 to 14.5%)	91.9 (74.3 to 109.8)
Cardiovascular diseases	Ukraine	2005	Age-standardized		11.7% (9.5 to 14.0%)	89.4 (72.6 to 107.1)
Cardiovascular diseases	Ukraine	2010	Age-standardized		12.0% (9.7 to 14.4%)	75.7 (60.9 to 91.0)
Cardiovascular diseases	Ukraine	2015	Age-standardized		11.9% (9.4 to 14.3%)	68.6 (55.0 to 82.8)
Ischemic heart disease	Ukraine	1990	All Ages	37.1 (29.1 to 45.6)	16.1% (12.7 to 19.9%)	72.4 (56.8 to 89.0)
Ischemic heart disease	Ukraine	1995	All Ages	45.6 (35.9 to 55.4)	14.8% (11.7 to 18.1%)	89.4 (70.3 to 108.6)
Ischemic heart disease	Ukraine	2000	All Ages	44.3 (34.4 to 54.1)	14.0% (11.0 to 17.2%)	90.5 (70.3 to 110.5)
Ischemic heart disease	Ukraine	2005	All Ages	46.6 (36.3 to 57.1)	13.3% (10.3 to 16.3%)	99.3 (77.3 to 121.7)
Ischemic heart disease	Ukraine	2010	All Ages	42.4 (32.0 to 52.6)	13.3% (10.0 to 16.5%)	92.5 (69.9 to 114.8)
Ischemic heart disease	Ukraine	2015	All Ages	43.7 (32.1 to 55.1)	13.0% (9.6 to 16.4%)	94.0 (69.0 to 118.5)
Ischemic heart disease	Ukraine	1990	Age-standardized		16.0% (12.4 to 19.7%)	60.2 (46.7 to 74.2)
Ischemic heart disease	Ukraine	1995	Age-standardized		14.7% (11.5 to 18.0%)	72.4 (56.5 to 89.0)
Ischemic heart disease	Ukraine	2000	Age-standardized		13.8% (10.5 to 17.0%)	70.7 (54.2 to 87.1)
Ischemic heart disease	Ukraine	2005	Age-standardized		13.3% (10.2 to 16.3%)	71.5 (55.1 to 87.8)
Ischemic heart disease	Ukraine	2010	Age-standardized		13.5% (10.3 to 16.6%)	61.1 (46.8 to 75.6)
Ischemic heart disease	Ukraine	2015	Age-standardized		13.3% (10.1 to 16.5%)	55.9 (42.9 to 69.3)
Ischemic stroke	Ukraine	1990	All Ages	10.9 (8.0 to 14.0)	12.2% (9.0 to 15.7%)	21.2 (15.6 to 27.3)
Ischemic stroke	Ukraine	1995	All Ages	11.3 (8.0 to 15.0)	10.8% (7.7 to 14.2%)	22.2 (15.7 to 29.4)
Ischemic stroke	Ukraine	2000	All Ages	9.1 (6.3 to 11.9)	10.1% (7.0 to 13.2%)	18.6 (12.9 to 24.3)
Ischemic stroke	Ukraine	2005	All Ages	8.0 (5.5 to 10.7)	9.4% (6.4 to 12.7%)	17.0 (11.8 to 22.9)
Ischemic stroke	Ukraine	2010	All Ages	7.0 (4.6 to 9.5)	9.6% (6.3 to 13.0%)	15.3 (10.1 to 20.7)
Ischemic stroke	Ukraine	2015	All Ages	6.8 (4.3 to 9.6)	9.4% (6.1 to 13.1%)	14.7 (9.2 to 20.6)
Ischemic stroke	Ukraine	1990	Age-standardized		12.1% (8.8 to 15.6%)	17.6 (12.7 to 22.8)
Ischemic stroke	Ukraine	1995	Age-standardized		10.6% (7.4 to 14.0%)	18.0 (12.6 to 24.0)
Ischemic stroke	Ukraine	2000	Age-standardized		9.8% (6.7 to 13.3%)	14.6 (9.9 to 19.9)
Ischemic stroke	Ukraine	2005	Age-standardized		9.3% (6.3 to 12.6%)	12.1 (8.1 to 16.4)
Ischemic stroke	Ukraine	2010	Age-standardized		9.6% (6.5 to 12.9%)	9.9 (6.7 to 13.4)

Cause name	Location	Year	Age group	Number (in thousands)	PAF	Rate (per 100,000)
Ischemic stroke	Ukraine	2015	Age-standardized		9.5% (6.4 to 12.9%)	8.5 (5.7 to 11.7)
Hemorrhagic stroke	Ukraine	1990	All Ages	4.7 (3.7 to 5.8)	14.1% (11.3 to 17.0%)	9.1 (7.2 to 11.3)
Hemorrhagic stroke	Ukraine	1995	All Ages	5.4 (4.1 to 6.7)	12.6% (9.7 to 15.5%)	10.5 (8.1 to 13.2)
Hemorrhagic stroke	Ukraine	2000	All Ages	4.2 (3.1 to 5.2)	11.7% (8.8 to 14.5%)	8.5 (6.4 to 10.6)
Hemorrhagic stroke	Ukraine	2005	All Ages	3.7 (2.8 to 4.7)	11.0% (8.2 to 13.8%)	8.0 (6.0 to 10.1)
Hemorrhagic stroke	Ukraine	2010	All Ages	3.1 (2.4 to 4.0)	11.1% (8.4 to 14.1%)	6.9 (5.1 to 8.7)
Hemorrhagic stroke	Ukraine	2015	All Ages	3.1 (2.2 to 4.0)	10.9% (8.0 to 14.0%)	6.7 (4.7 to 8.7)
Hemorrhagic stroke	Ukraine	1990	Age-standardized		14.0% (11.3 to 16.9%)	7.5 (5.9 to 9.2)
Hemorrhagic stroke	Ukraine	1995	Age-standardized		12.5% (9.8 to 15.4%)	8.4 (6.5 to 10.4)
Hemorrhagic stroke	Ukraine	2000	Age-standardized		11.6% (8.9 to 14.5%)	6.6 (5.0 to 8.2)
Hemorrhagic stroke	Ukraine	2005	Age-standardized		11.1% (8.4 to 13.9%)	5.9 (4.4 to 7.3)
Hemorrhagic stroke	Ukraine	2010	Age-standardized		11.4% (8.7 to 14.3%)	4.7 (3.6 to 5.9)
Hemorrhagic stroke	Ukraine	2015	Age-standardized		11.2% (8.6 to 14.1%)	4.2 (3.1 to 5.3)
Chronic obstructive pulmonary disease	Ukraine	1990	All Ages	6.8 (3.8 to 10.1)	23.4% (12.9 to 34.5%)	13.2 (7.3 to 19.7)
Chronic obstructive pulmonary disease	Ukraine	1995	All Ages	6.8 (3.4 to 10.5)	19.4% (9.7 to 29.9%)	13.4 (6.7 to 20.6)
Chronic obstructive pulmonary disease	Ukraine	2000	All Ages	4.8 (2.3 to 7.6)	17.5% (8.4 to 27.9%)	9.8 (4.6 to 15.5)
Chronic obstructive pulmonary disease	Ukraine	2005	All Ages	3.3 (1.5 to 5.3)	16.0% (7.3 to 26.5%)	7.0 (3.2 to 11.4)
Chronic obstructive pulmonary disease	Ukraine	2010	All Ages	2.4 (1.1 to 3.8)	17.0% (8.1 to 27.5%)	5.2 (2.4 to 8.4)
Chronic obstructive pulmonary disease	Ukraine	2015	All Ages	2.5 (1.2 to 4.1)	16.8% (7.7 to 27.4%)	5.5 (2.5 to 8.8)
Chronic obstructive pulmonary disease	Ukraine	1990	Age-standardized		23.4% (12.9 to 34.5%)	10.9 (6.0 to 16.2)
Chronic obstructive pulmonary disease	Ukraine	1995	Age-standardized		19.3% (9.7 to 29.9%)	10.8 (5.4 to 16.6)
Chronic obstructive pulmonary disease	Ukraine	2000	Age-standardized		17.5% (8.4 to 27.9%)	7.5 (3.6 to 11.9)
Chronic obstructive pulmonary disease	Ukraine	2005	Age-standardized		16.0% (7.3 to 26.4%)	5.0 (2.3 to 8.1)
Chronic obstructive pulmonary disease	Ukraine	2010	Age-standardized		17.0% (8.0 to 27.5%)	3.4 (1.6 to 5.5)
Chronic obstructive pulmonary disease	Ukraine	2015	Age-standardized		16.8% (7.7 to 27.4%)	3.3 (1.5 to 5.2)
All causes	Albania	1990	All Ages	1.2 (1.0 to 1.5)	6.9% (5.6 to 8.6%)	36.1 (29.2 to 44.7)
All causes	Albania	1995	All Ages	1.1 (0.8 to 1.3)	6.3% (5.1 to 7.7%)	33.6 (27.3 to 41.5)
All causes	Albania	2000	All Ages	1.0 (0.8 to 1.2)	6.1% (5.0 to 7.2%)	32.6 (27.0 to 38.7)
All causes	Albania	2005	All Ages	1.0 (0.8 to 1.2)	5.7% (4.7 to 6.9%)	31.5 (25.8 to 37.8)
All causes	Albania	2010	All Ages	1.2 (1.0 to 1.5)	6.6% (5.5 to 7.9%)	42.3 (34.5 to 51.2)
All causes	Albania	2015	All Ages	1.5 (1.2 to 1.8)	7.1% (5.9 to 8.5%)	51.3 (41.3 to 62.7)
All causes	Albania	1990	Age-standardized		7.2% (5.9 to 8.7%)	58.1 (47.5 to 70.1)
All causes	Albania	1995	Age-standardized		6.5% (5.2 to 7.9%)	49.9 (40.2 to 61.0)
All causes	Albania	2000	Age-standardized		6.2% (5.1 to 7.5%)	46.8 (38.0 to 56.4)
All causes	Albania	2005	Age-standardized		5.7% (4.6 to 6.9%)	39.0 (31.1 to 47.6)
All causes	Albania	2010	Age-standardized		6.5% (5.3 to 7.9%)	41.4 (33.5 to 50.5)

Cause name	Location	Year	Age group	Number (in thousands)	PAF	Rate (per 100,000)
All causes	Albania	2015	Age-standardized		7.0% (5.7 to 8.4%)	42.9 (34.4 to 52.5)
Lower respiratory infections	Albania	1990	All Ages	0.3 (0.1 to 0.6)	15.1% (6.8 to 25.5%)	9.6 (4.1 to 17.1)
Lower respiratory infections	Albania	1995	All Ages	0.2 (0.1 to 0.4)	13.0% (5.1 to 23.7%)	7.1 (2.7 to 13.4)
Lower respiratory infections	Albania	2000	All Ages	0.1 (0.0 to 0.2)	12.4% (4.5 to 23.1%)	3.5 (1.3 to 6.6)
Lower respiratory infections	Albania	2005	All Ages	0.1 (0.0 to 0.1)	10.6% (3.2 to 21.5%)	1.8 (0.5 to 3.7)
Lower respiratory infections	Albania	2010	All Ages	0.1 (0.0 to 0.1)	13.2% (4.9 to 24.1%)	1.9 (0.7 to 3.6)
Lower respiratory infections	Albania	2015	All Ages	0.1 (0.0 to 0.1)	15.2% (6.7 to 25.8%)	2.2 (0.9 to 3.9)
Lower respiratory infections	Albania	1990	Age-standardized		15.1% (6.8 to 25.5%)	10.4 (4.2 to 19.0)
Lower respiratory infections	Albania	1995	Age-standardized		13.0% (5.1 to 23.7%)	7.8 (2.9 to 15.1)
Lower respiratory infections	Albania	2000	Age-standardized		12.4% (4.5 to 23.1%)	4.5 (1.6 to 8.5)
Lower respiratory infections	Albania	2005	Age-standardized		10.6% (3.2 to 21.5%)	2.4 (0.7 to 4.9)
Lower respiratory infections	Albania	2010	Age-standardized		13.2% (4.9 to 24.1%)	2.2 (0.8 to 4.1)
Lower respiratory infections	Albania	2015	Age-standardized		15.2% (6.7 to 25.8%)	2.1 (0.9 to 3.8)
Tracheal, bronchus, and lung cancer	Albania	1990	All Ages	0.1 (0.0 to 0.1)	10.4% (5.5 to 16.3%)	1.8 (0.9 to 2.9)
Tracheal, bronchus, and lung cancer	Albania	1995	All Ages	0.1 (0.0 to 0.1)	8.9% (4.5 to 14.3%)	1.8 (0.9 to 2.8)
Tracheal, bronchus, and lung cancer	Albania	2000	All Ages	0.1 (0.0 to 0.1)	8.5% (4.1 to 13.7%)	1.9 (1.0 to 3.2)
Tracheal, bronchus, and lung cancer	Albania	2005	All Ages	0.1 (0.0 to 0.1)	7.2% (3.4 to 12.1%)	1.8 (0.9 to 3.0)
Tracheal, bronchus, and lung cancer	Albania	2010	All Ages	0.1 (0.0 to 0.1)	9.1% (4.6 to 14.5%)	2.8 (1.4 to 4.4)
Tracheal, bronchus, and lung cancer	Albania	2015	All Ages	0.1 (0.1 to 0.2)	10.5% (5.5 to 16.6%)	3.7 (1.9 to 6.0)
Tracheal, bronchus, and lung cancer	Albania	1990	Age-standardized		10.5% (5.5 to 16.3%)	2.9 (1.5 to 4.6)
Tracheal, bronchus, and lung cancer	Albania	1995	Age-standardized		8.9% (4.5 to 14.3%)	2.5 (1.2 to 4.0)
Tracheal, bronchus, and lung cancer	Albania	2000	Age-standardized		8.5% (4.1 to 13.7%)	2.5 (1.2 to 4.1)
Tracheal, bronchus, and lung cancer	Albania	2005	Age-standardized		7.2% (3.4 to 12.1%)	2.0 (0.9 to 3.4)
Tracheal, bronchus, and lung cancer	Albania	2010	Age-standardized		9.1% (4.6 to 14.5%)	2.5 (1.3 to 3.9)
Tracheal, bronchus, and lung cancer	Albania	2015	Age-standardized		10.5% (5.5 to 16.6%)	3.0 (1.5 to 4.7)
Cardiovascular diseases	Albania	1990	All Ages	0.7 (0.6 to 0.8)	10.3% (8.5 to 12.1%)	21.8 (17.9 to 25.8)
Cardiovascular diseases	Albania	1995	All Ages	0.7 (0.6 to 0.8)	9.8% (8.0 to 11.6%)	22.3 (18.2 to 26.7)
Cardiovascular diseases	Albania	2000	All Ages	0.8 (0.6 to 1.0)	9.6% (7.8 to 11.5%)	25.2 (20.4 to 30.2)
Cardiovascular diseases	Albania	2005	All Ages	0.8 (0.7 to 1.0)	9.0% (7.2 to 10.8%)	26.4 (21.1 to 31.9)
Cardiovascular diseases	Albania	2010	All Ages	1.0 (0.8 to 1.3)	9.8% (8.1 to 11.8%)	35.4 (28.6 to 43.7)
Cardiovascular diseases	Albania	2015	All Ages	1.2 (1.0 to 1.5)	10.3% (8.2 to 12.3%)	42.4 (33.2 to 53.0)
Cardiovascular diseases	Albania	1990	Age-standardized		9.9% (8.0 to 11.9%)	39.1 (31.5 to 47.3)
Cardiovascular diseases	Albania	1995	Age-standardized		9.4% (7.6 to 11.4%)	35.6 (28.5 to 43.3)
Cardiovascular diseases	Albania	2000	Age-standardized		9.2% (7.3 to 11.2%)	36.6 (28.8 to 44.7)
Cardiovascular diseases	Albania	2005	Age-standardized		8.6% (6.8 to 10.6%)	32.5 (25.4 to 40.0)
Cardiovascular diseases	Albania	2010	Age-standardized		9.6% (7.7 to 11.6%)	34.5 (27.4 to 43.3)

Cause name	Location	Year	Age group	Number (in thousands)	PAF	Rate (per 100,000)
Cardiovascular diseases	Albania	2015	Age-standardized		10.1% (8.1 to 12.2%)	35.3 (27.5 to 44.4)
Ischemic heart disease	Albania	1990	All Ages	0.4 (0.3 to 0.5)	14.1% (11.1 to 17.0%)	13.4 (10.5 to 16.3)
Ischemic heart disease	Albania	1995	All Ages	0.4 (0.3 to 0.5)	13.3% (10.5 to 16.1%)	13.6 (10.7 to 16.8)
Ischemic heart disease	Albania	2000	All Ages	0.5 (0.4 to 0.6)	13.0% (10.2 to 15.9%)	15.5 (12.1 to 19.0)
Ischemic heart disease	Albania	2005	All Ages	0.5 (0.4 to 0.6)	12.2% (9.5 to 14.9%)	16.8 (13.1 to 20.6)
Ischemic heart disease	Albania	2010	All Ages	0.7 (0.5 to 0.8)	13.1% (10.3 to 16.0%)	22.6 (17.5 to 28.3)
Ischemic heart disease	Albania	2015	All Ages	0.8 (0.6 to 1.0)	13.6% (10.4 to 16.6%)	26.8 (20.0 to 34.1)
Ischemic heart disease	Albania	1990	Age-standardized		13.4% (10.3 to 16.6%)	23.7 (18.0 to 29.6)
Ischemic heart disease	Albania	1995	Age-standardized		12.7% (9.7 to 15.7%)	21.4 (16.2 to 26.7)
Ischemic heart disease	Albania	2000	Age-standardized		12.4% (9.3 to 15.4%)	22.1 (16.5 to 27.7)
Ischemic heart disease	Albania	2005	Age-standardized		11.7% (8.8 to 14.7%)	20.3 (15.2 to 25.6)
Ischemic heart disease	Albania	2010	Age-standardized		12.8% (9.8 to 16.0%)	21.9 (16.5 to 27.7)
Ischemic heart disease	Albania	2015	Age-standardized		13.5% (10.2 to 16.7%)	22.3 (16.4 to 28.8)
Ischemic stroke	Albania	1990	All Ages	0.1 (0.0 to 0.1)	9.8% (6.9 to 12.9%)	1.5 (1.0 to 2.5)
Ischemic stroke	Albania	1995	All Ages	0.0 (0.0 to 0.1)	9.1% (6.2 to 12.1%)	1.4 (0.9 to 2.3)
Ischemic stroke	Albania	2000	All Ages	0.1 (0.0 to 0.1)	8.8% (5.9 to 11.9%)	1.5 (1.0 to 2.4)
Ischemic stroke	Albania	2005	All Ages	0.1 (0.0 to 0.1)	8.1% (5.2 to 11.3%)	1.6 (1.0 to 2.5)
Ischemic stroke	Albania	2010	All Ages	0.1 (0.0 to 0.1)	9.0% (6.0 to 12.3%)	2.1 (1.3 to 3.3)
Ischemic stroke	Albania	2015	All Ages	0.1 (0.1 to 0.1)	9.5% (6.4 to 13.0%)	2.6 (1.7 to 4.1)
Ischemic stroke	Albania	1990	Age-standardized		9.5% (6.3 to 13.0%)	2.8 (1.8 to 4.8)
Ischemic stroke	Albania	1995	Age-standardized		8.8% (5.7 to 12.3%)	2.3 (1.4 to 3.9)
Ischemic stroke	Albania	2000	Age-standardized		8.5% (5.4 to 12.1%)	2.3 (1.4 to 3.8)
Ischemic stroke	Albania	2005	Age-standardized		7.8% (4.8 to 11.4%)	2.1 (1.2 to 3.2)
Ischemic stroke	Albania	2010	Age-standardized		8.8% (5.6 to 12.5%)	2.1 (1.3 to 3.4)
Ischemic stroke	Albania	2015	Age-standardized		9.5% (6.2 to 13.1%)	2.2 (1.4 to 3.4)
Hemorrhagic stroke	Albania	1990	All Ages	0.2 (0.2 to 0.3)	10.1% (7.3 to 13.1%)	6.9 (5.0 to 9.0)
Hemorrhagic stroke	Albania	1995	All Ages	0.2 (0.2 to 0.3)	9.3% (6.5 to 12.3%)	7.3 (5.2 to 9.6)
Hemorrhagic stroke	Albania	2000	All Ages	0.3 (0.2 to 0.3)	8.9% (6.0 to 11.9%)	8.2 (5.5 to 10.9)
Hemorrhagic stroke	Albania	2005	All Ages	0.3 (0.2 to 0.3)	8.2% (5.3 to 11.3%)	8.0 (5.2 to 11.0)
Hemorrhagic stroke	Albania	2010	All Ages	0.3 (0.2 to 0.4)	9.1% (6.2 to 12.3%)	10.7 (7.1 to 14.7)
Hemorrhagic stroke	Albania	2015	All Ages	0.4 (0.3 to 0.5)	9.6% (6.5 to 13.0%)	12.9 (8.6 to 18.2)
Hemorrhagic stroke	Albania	1990	Age-standardized		9.8% (6.8 to 13.1%)	12.6 (8.7 to 17.0)
Hemorrhagic stroke	Albania	1995	Age-standardized		9.0% (6.0 to 12.3%)	11.9 (7.9 to 16.2)
Hemorrhagic stroke	Albania	2000	Age-standardized		8.6% (5.5 to 12.0%)	12.2 (7.9 to 17.1)
Hemorrhagic stroke	Albania	2005	Age-standardized		7.9% (4.9 to 11.4%)	10.1 (6.3 to 14.9)
Hemorrhagic stroke	Albania	2010	Age-standardized		8.9% (5.8 to 12.5%)	10.6 (6.8 to 15.0)

Cause name	Location	Year	Age group	Number (in thousands)	PAF	Rate (per 100,000)
Hemorrhagic stroke	Albania	2015	Age-standardized		9.5% (6.3 to 13.2%)	10.8 (7.1 to 15.5)
Chronic obstructive pulmonary disease	Albania	1990	All Ages	0.1 (0.0 to 0.2)	16.1% (7.4 to 26.3%)	2.9 (1.3 to 4.8)
Chronic obstructive pulmonary disease	Albania	1995	All Ages	0.1 (0.0 to 0.1)	14.3% (6.1 to 24.2%)	2.4 (1.0 to 4.0)
Chronic obstructive pulmonary disease	Albania	2000	All Ages	0.1 (0.0 to 0.1)	14.0% (5.8 to 23.8%)	2.0 (0.8 to 3.5)
Chronic obstructive pulmonary disease	Albania	2005	All Ages	0.1 (0.0 to 0.1)	12.5% (4.9 to 22.2%)	1.6 (0.6 to 2.8)
Chronic obstructive pulmonary disease	Albania	2010	All Ages	0.1 (0.0 to 0.1)	14.9% (6.4 to 25.3%)	2.2 (0.9 to 3.6)
Chronic obstructive pulmonary disease	Albania	2015	All Ages	0.1 (0.0 to 0.1)	16.7% (7.6 to 27.2%)	3.0 (1.3 to 4.9)
Chronic obstructive pulmonary disease	Albania	1990	Age-standardized		16.5% (7.5 to 26.8%)	5.6 (2.5 to 9.3)
Chronic obstructive pulmonary disease	Albania	1995	Age-standardized		14.6% (6.2 to 24.8%)	4.0 (1.7 to 6.9)
Chronic obstructive pulmonary disease	Albania	2000	Age-standardized		14.1% (5.9 to 24.1%)	3.1 (1.3 to 5.4)
Chronic obstructive pulmonary disease	Albania	2005	Age-standardized		12.5% (4.9 to 22.2%)	2.0 (0.8 to 3.6)
Chronic obstructive pulmonary disease	Albania	2010	Age-standardized		14.9% (6.3 to 25.2%)	2.2 (0.9 to 3.6)
Chronic obstructive pulmonary disease	Albania	2015	Age-standardized		16.6% (7.5 to 27.1%)	2.5 (1.1 to 4.1)
All causes	Bosnia and Herzegovina	1990	All Ages	2.9 (2.6 to 3.3)	9.8% (8.7 to 11.1%)	64.7 (57.0 to 73.3)
All causes	Bosnia and Herzegovina	1995	All Ages	2.8 (2.4 to 3.1)	7.1% (6.2 to 8.2%)	70.3 (61.4 to 79.7)
All causes	Bosnia and Herzegovina	2000	All Ages	2.5 (2.2 to 2.9)	9.5% (8.3 to 10.8%)	66.4 (57.8 to 75.7)
All causes	Bosnia and Herzegovina	2005	All Ages	2.4 (2.1 to 2.7)	8.4% (7.3 to 9.7%)	61.8 (53.6 to 71.5)
All causes	Bosnia and Herzegovina	2010	All Ages	2.5 (2.1 to 2.9)	8.0% (7.0 to 9.3%)	64.5 (55.8 to 75.3)
All causes	Bosnia and Herzegovina	2015	All Ages	2.8 (2.4 to 3.3)	8.6% (7.4 to 10.1%)	74.3 (63.1 to 87.0)
All causes	Bosnia and Herzegovina	1990	Age-standardized		10.0% (8.6 to 11.5%)	89.1 (77.1 to 102.0)
All causes	Bosnia and Herzegovina	1995	Age-standardized		7.4% (6.3 to 8.6%)	83.9 (72.0 to 96.9)
All causes	Bosnia and Herzegovina	2000	Age-standardized		9.2% (7.8 to 10.7%)	70.5 (59.7 to 81.9)
All causes	Bosnia and Herzegovina	2005	Age-standardized		8.2% (7.0 to 9.5%)	54.6 (46.8 to 63.6)
All causes	Bosnia and Herzegovina	2010	Age-standardized		7.8% (6.7 to 9.1%)	48.0 (41.2 to 56.2)
All causes	Bosnia and Herzegovina	2015	Age-standardized		8.4% (7.3 to 9.9%)	48.5 (41.4 to 56.9)
Lower respiratory infections	Bosnia and Herzegovina	1990	All Ages	0.2 (0.1 to 0.2)	29.8% (22.1 to 37.4%)	3.4 (2.5 to 4.5)
Lower respiratory infections	Bosnia and Herzegovina	1995	All Ages	0.1 (0.1 to 0.2)	26.2% (18.3 to 34.3%)	3.0 (2.1 to 4.1)
Lower respiratory infections	Bosnia and Herzegovina	2000	All Ages	0.1 (0.1 to 0.1)	26.3% (18.4 to 34.3%)	2.5 (1.7 to 3.5)
Lower respiratory infections	Bosnia and Herzegovina	2005	All Ages	0.1 (0.1 to 0.1)	25.2% (17.3 to 33.4%)	2.2 (1.4 to 3.5)
Lower respiratory infections	Bosnia and Herzegovina	2010	All Ages	0.1 (0.1 to 0.2)	26.3% (18.5 to 34.4%)	2.4 (1.5 to 4.1)
Lower respiratory infections	Bosnia and Herzegovina	2015	All Ages	0.1 (0.1 to 0.2)	30.1% (22.3 to 37.7%)	3.0 (1.9 to 4.9)
Lower respiratory infections	Bosnia and Herzegovina	1990	Age-standardized		29.8% (22.1 to 37.4%)	4.8 (3.5 to 6.3)
Lower respiratory infections	Bosnia and Herzegovina	1995	Age-standardized		26.2% (18.3 to 34.3%)	4.0 (2.7 to 5.4)
Lower respiratory infections	Bosnia and Herzegovina	2000	Age-standardized		26.3% (18.4 to 34.3%)	3.0 (2.0 to 4.2)
Lower respiratory infections	Bosnia and Herzegovina	2005	Age-standardized		25.2% (17.3 to 33.4%)	2.3 (1.5 to 3.5)
Lower respiratory infections	Bosnia and Herzegovina	2010	Age-standardized		26.3% (18.5 to 34.4%)	2.0 (1.3 to 3.3)

Cause name	Location	Year	Age group	Number (in thousands)	PAF	Rate (per 100,000)
Lower respiratory infections	Bosnia and Herzegovina	2015	Age-standardized		30.1% (22.3 to 37.7%)	2.1 (1.3 to 3.3)
Tracheal, bronchus, and lung cancer	Bosnia and Herzegovina	1990	All Ages	0.3 (0.2 to 0.4)	21.8% (14.0 to 29.7%)	6.8 (4.4 to 9.4)
Tracheal, bronchus, and lung cancer	Bosnia and Herzegovina	1995	All Ages	0.3 (0.2 to 0.4)	18.9% (11.7 to 26.5%)	7.2 (4.4 to 10.2)
Tracheal, bronchus, and lung cancer	Bosnia and Herzegovina	2000	All Ages	0.3 (0.2 to 0.4)	19.0% (11.6 to 26.7%)	8.2 (5.0 to 11.7)
Tracheal, bronchus, and lung cancer	Bosnia and Herzegovina	2005	All Ages	0.3 (0.2 to 0.4)	18.0% (10.7 to 25.7%)	8.1 (4.8 to 11.6)
Tracheal, bronchus, and lung cancer	Bosnia and Herzegovina	2010	All Ages	0.4 (0.2 to 0.5)	19.0% (11.7 to 26.7%)	9.0 (5.4 to 12.9)
Tracheal, bronchus, and lung cancer	Bosnia and Herzegovina	2015	All Ages	0.4 (0.3 to 0.6)	22.1% (14.2 to 30.1%)	11.1 (7.0 to 15.4)
Tracheal, bronchus, and lung cancer	Bosnia and Herzegovina	1990	Age-standardized		21.8% (14.1 to 29.7%)	8.0 (5.1 to 10.9)
Tracheal, bronchus, and lung cancer	Bosnia and Herzegovina	1995	Age-standardized		18.9% (11.7 to 26.5%)	7.1 (4.3 to 10.1)
Tracheal, bronchus, and lung cancer	Bosnia and Herzegovina	2000	Age-standardized		18.9% (11.6 to 26.7%)	7.2 (4.3 to 10.3)
Tracheal, bronchus, and lung cancer	Bosnia and Herzegovina	2005	Age-standardized		18.0% (10.7 to 25.7%)	6.4 (3.8 to 9.1)
Tracheal, bronchus, and lung cancer	Bosnia and Herzegovina	2010	Age-standardized		19.0% (11.7 to 26.7%)	6.4 (3.9 to 9.2)
Tracheal, bronchus, and lung cancer	Bosnia and Herzegovina	2015	Age-standardized		22.0% (14.1 to 30.1%)	7.2 (4.5 to 10.1)
Cardiovascular diseases	Bosnia and Herzegovina	1990	All Ages	2.2 (1.9 to 2.5)	15.0% (12.9 to 17.3%)	48.3 (41.6 to 55.6)
Cardiovascular diseases	Bosnia and Herzegovina	1995	All Ages	2.1 (1.8 to 2.4)	14.0% (12.0 to 16.2%)	53.7 (45.7 to 61.8)
Cardiovascular diseases	Bosnia and Herzegovina	2000	All Ages	1.9 (1.6 to 2.2)	13.6% (11.6 to 15.8%)	49.7 (42.6 to 57.6)
Cardiovascular diseases	Bosnia and Herzegovina	2005	All Ages	1.8 (1.5 to 2.0)	12.3% (10.5 to 14.3%)	45.8 (39.0 to 53.4)
Cardiovascular diseases	Bosnia and Herzegovina	2010	All Ages	1.8 (1.5 to 2.1)	11.6% (9.7 to 13.8%)	46.7 (39.1 to 55.6)
Cardiovascular diseases	Bosnia and Herzegovina	2015	All Ages	2.0 (1.7 to 2.4)	12.0% (10.0 to 14.4%)	52.7 (43.7 to 63.1)
Cardiovascular diseases	Bosnia and Herzegovina	1990	Age-standardized		14.1% (11.9 to 16.6%)	67.0 (56.6 to 79.1)
Cardiovascular diseases	Bosnia and Herzegovina	1995	Age-standardized		13.1% (11.0 to 15.5%)	64.5 (53.8 to 76.4)
Cardiovascular diseases	Bosnia and Herzegovina	2000	Age-standardized		12.7% (10.5 to 15.1%)	53.5 (44.2 to 63.8)
Cardiovascular diseases	Bosnia and Herzegovina	2005	Age-standardized		11.7% (9.7 to 13.8%)	40.8 (33.7 to 48.3)
Cardiovascular diseases	Bosnia and Herzegovina	2010	Age-standardized		11.4% (9.4 to 13.6%)	34.9 (29.0 to 41.7)
Cardiovascular diseases	Bosnia and Herzegovina	2015	Age-standardized		12.1% (10.1 to 14.5%)	34.4 (28.5 to 41.2)
Ischemic heart disease	Bosnia and Herzegovina	1990	All Ages	1.5 (1.2 to 1.8)	19.5% (16.1 to 23.5%)	32.8 (26.8 to 39.6)
Ischemic heart disease	Bosnia and Herzegovina	1995	All Ages	1.4 (1.2 to 1.7)	18.4% (15.1 to 22.0%)	36.7 (29.8 to 44.4)
Ischemic heart disease	Bosnia and Herzegovina	2000	All Ages	1.3 (1.1 to 1.5)	18.1% (14.8 to 21.8%)	33.9 (27.7 to 40.7)
Ischemic heart disease	Bosnia and Herzegovina	2005	All Ages	1.1 (0.9 to 1.4)	17.5% (14.2 to 21.1%)	28.9 (23.4 to 35.2)
Ischemic heart disease	Bosnia and Herzegovina	2010	All Ages	1.1 (0.8 to 1.3)	17.3% (13.9 to 21.3%)	28.1 (22.2 to 34.9)
Ischemic heart disease	Bosnia and Herzegovina	2015	All Ages	1.2 (0.9 to 1.5)	18.0% (14.2 to 22.5%)	31.7 (24.5 to 40.3)
Ischemic heart disease	Bosnia and Herzegovina	1990	Age-standardized		18.3% (14.6 to 22.6%)	45.3 (36.0 to 56.2)
Ischemic heart disease	Bosnia and Herzegovina	1995	Age-standardized		17.1% (13.4 to 21.1%)	44.0 (34.3 to 54.3)
Ischemic heart disease	Bosnia and Herzegovina	2000	Age-standardized		16.9% (13.0 to 21.0%)	36.5 (28.1 to 45.4)
Ischemic heart disease	Bosnia and Herzegovina	2005	Age-standardized		16.7% (13.0 to 20.6%)	25.8 (19.9 to 32.3)
Ischemic heart disease	Bosnia and Herzegovina	2010	Age-standardized		17.2% (13.5 to 21.3%)	21.0 (16.4 to 26.4)

Cause name	Location	Year	Age group	Number (in thousands)	PAF	Rate (per 100,000)
Ischemic heart disease	Bosnia and Herzegovina	2015	Age-standardized		18.2% (14.4 to 22.7%)	20.7 (16.1 to 26.2)
Ischemic stroke	Bosnia and Herzegovina	1990	All Ages	0.3 (0.3 to 0.4)	14.6% (11.1 to 18.2%)	7.5 (5.6 to 9.5)
Ischemic stroke	Bosnia and Herzegovina	1995	All Ages	0.3 (0.2 to 0.4)	13.6% (10.2 to 16.9%)	8.2 (6.0 to 10.6)
Ischemic stroke	Bosnia and Herzegovina	2000	All Ages	0.3 (0.2 to 0.4)	13.6% (10.1 to 16.8%)	8.5 (6.2 to 10.6)
Ischemic stroke	Bosnia and Herzegovina	2005	All Ages	0.5 (0.3 to 0.6)	13.1% (9.8 to 16.3%)	11.6 (8.3 to 14.8)
Ischemic stroke	Bosnia and Herzegovina	2010	All Ages	0.6 (0.4 to 0.7)	13.2% (9.7 to 16.6%)	14.3 (10.2 to 18.4)
Ischemic stroke	Bosnia and Herzegovina	2015	All Ages	0.6 (0.5 to 0.8)	14.0% (10.3 to 18.0%)	16.3 (11.7 to 21.3)
Ischemic stroke	Bosnia and Herzegovina	1990	Age-standardized		14.0% (10.3 to 17.8%)	11.4 (8.4 to 14.7)
Ischemic stroke	Bosnia and Herzegovina	1995	Age-standardized		12.9% (9.2 to 16.6%)	10.9 (7.8 to 14.4)
Ischemic stroke	Bosnia and Herzegovina	2000	Age-standardized		12.8% (9.0 to 16.7%)	9.8 (6.7 to 13.1)
Ischemic stroke	Bosnia and Herzegovina	2005	Age-standardized		12.6% (9.0 to 16.3%)	10.6 (7.5 to 14.0)
Ischemic stroke	Bosnia and Herzegovina	2010	Age-standardized		13.0% (9.4 to 16.7%)	10.7 (7.5 to 13.9)
Ischemic stroke	Bosnia and Herzegovina	2015	Age-standardized		14.1% (10.4 to 18.0%)	10.5 (7.5 to 13.8)
Hemorrhagic stroke	Bosnia and Herzegovina	1990	All Ages	0.4 (0.3 to 0.4)	16.9% (13.9 to 19.9%)	8.0 (6.6 to 9.7)
Hemorrhagic stroke	Bosnia and Herzegovina	1995	All Ages	0.3 (0.3 to 0.4)	15.5% (12.5 to 18.5%)	8.7 (6.9 to 10.9)
Hemorrhagic stroke	Bosnia and Herzegovina	2000	All Ages	0.3 (0.2 to 0.4)	15.3% (12.3 to 18.3%)	7.3 (5.7 to 9.2)
Hemorrhagic stroke	Bosnia and Herzegovina	2005	All Ages	0.2 (0.1 to 0.3)	14.7% (11.7 to 17.6%)	5.2 (4.0 to 7.1)
Hemorrhagic stroke	Bosnia and Herzegovina	2010	All Ages	0.2 (0.1 to 0.3)	14.7% (11.7 to 17.5%)	4.4 (3.3 to 6.8)
Hemorrhagic stroke	Bosnia and Herzegovina	2015	All Ages	0.2 (0.1 to 0.3)	15.6% (12.5 to 18.8%)	4.7 (3.5 to 7.5)
Hemorrhagic stroke	Bosnia and Herzegovina	1990	Age-standardized		15.9% (12.9 to 18.7%)	10.3 (8.3 to 12.8)
Hemorrhagic stroke	Bosnia and Herzegovina	1995	Age-standardized		14.6% (11.6 to 17.4%)	9.7 (7.6 to 12.2)
Hemorrhagic stroke	Bosnia and Herzegovina	2000	Age-standardized		14.3% (11.2 to 17.2%)	7.2 (5.6 to 9.3)
Hemorrhagic stroke	Bosnia and Herzegovina	2005	Age-standardized		14.1% (11.0 to 16.9%)	4.4 (3.3 to 6.0)
Hemorrhagic stroke	Bosnia and Herzegovina	2010	Age-standardized		14.5% (11.5 to 17.3%)	3.2 (2.4 to 5.1)
Hemorrhagic stroke	Bosnia and Herzegovina	2015	Age-standardized		15.7% (12.6 to 18.8%)	3.1 (2.3 to 5.0)
Chronic obstructive pulmonary disease	Bosnia and Herzegovina	1990	All Ages	0.3 (0.2 to 0.4)	28.1% (17.2 to 40.0%)	6.1 (3.6 to 8.8)
Chronic obstructive pulmonary disease	Bosnia and Herzegovina	1995	All Ages	0.3 (0.1 to 0.4)	25.4% (14.5 to 36.6%)	6.4 (3.6 to 9.4)
Chronic obstructive pulmonary disease	Bosnia and Herzegovina	2000	All Ages	0.2 (0.1 to 0.3)	25.4% (14.6 to 36.7%)	6.0 (3.4 to 8.8)
Chronic obstructive pulmonary disease	Bosnia and Herzegovina	2005	All Ages	0.2 (0.1 to 0.3)	24.6% (13.6 to 35.7%)	5.7 (3.2 to 8.5)
Chronic obstructive pulmonary disease	Bosnia and Herzegovina	2010	All Ages	0.2 (0.1 to 0.4)	25.5% (14.7 to 37.2%)	6.3 (3.5 to 9.2)
Chronic obstructive pulmonary disease	Bosnia and Herzegovina	2015	All Ages	0.3 (0.2 to 0.4)	28.4% (17.5 to 40.4%)	7.6 (4.6 to 11.1)
Chronic obstructive pulmonary disease	Bosnia and Herzegovina	1990	Age-standardized		28.2% (17.2 to 40.1%)	9.3 (5.4 to 13.4)
Chronic obstructive pulmonary disease	Bosnia and Herzegovina	1995	Age-standardized		25.4% (14.5 to 36.6%)	8.3 (4.6 to 12.2)
Chronic obstructive pulmonary disease	Bosnia and Herzegovina	2000	Age-standardized		25.4% (14.6 to 36.7%)	6.7 (3.8 to 10.2)
Chronic obstructive pulmonary disease	Bosnia and Herzegovina	2005	Age-standardized		24.6% (13.6 to 35.7%)	5.2 (2.9 to 7.9)
Chronic obstructive pulmonary disease	Bosnia and Herzegovina	2010	Age-standardized		25.5% (14.7 to 37.1%)	4.7 (2.6 to 7.0)

Cause name	Location	Year	Age group	Number (in thousands)	PAF	Rate (per 100,000)
Chronic obstructive pulmonary disease	Bosnia and Herzegovina	2015	Age-standardized		28.4% (17.4 to 40.4%)	4.8 (3.0 to 7.2)
All causes	Bulgaria	1990	All Ages	12.8 (11.2 to 14.6)	11.8% (10.3 to 13.5%)	145.1 (126.9 to 165.8)
All causes	Bulgaria	1995	All Ages	12.2 (10.5 to 14.1)	10.5% (9.1 to 12.1%)	145.3 (125.3 to 167.7)
All causes	Bulgaria	2000	All Ages	10.9 (9.3 to 12.5)	9.5% (8.2 to 10.9%)	135.4 (116.3 to 155.9)
All causes	Bulgaria	2005	All Ages	9.4 (7.9 to 10.9)	8.3% (7.0 to 9.6%)	122.4 (102.9 to 142.1)
All causes	Bulgaria	2010	All Ages	9.3 (7.9 to 10.9)	8.6% (7.2 to 10.0%)	125.9 (106.5 to 146.8)
All causes	Bulgaria	2015	All Ages	9.5 (7.9 to 11.2)	8.7% (7.2 to 10.2%)	130.9 (108.9 to 154.4)
All causes	Bulgaria	1990	Age-standardized		11.6% (10.0 to 13.4%)	121.0 (104.6 to 139.8)
All causes	Bulgaria	1995	Age-standardized		10.3% (8.8 to 11.9%)	110.5 (95.1 to 128.1)
All causes	Bulgaria	2000	Age-standardized		9.3% (7.8 to 10.8%)	97.5 (82.6 to 113.6)
All causes	Bulgaria	2005	Age-standardized		8.1% (6.8 to 9.4%)	77.8 (65.3 to 90.3)
All causes	Bulgaria	2010	Age-standardized		8.4% (7.1 to 9.7%)	73.4 (62.2 to 85.2)
All causes	Bulgaria	2015	Age-standardized		8.5% (7.2 to 9.9%)	69.7 (58.7 to 81.5)
Lower respiratory infections	Bulgaria	1990	All Ages	1.2 (0.8 to 1.5)	29.4% (21.6 to 37.0%)	13.0 (9.6 to 16.5)
Lower respiratory infections	Bulgaria	1995	All Ages	0.8 (0.5 to 1.1)	24.8% (16.8 to 33.4%)	9.4 (6.3 to 12.7)
Lower respiratory infections	Bulgaria	2000	All Ages	0.5 (0.3 to 0.8)	22.2% (13.9 to 31.9%)	6.6 (4.1 to 9.3)
Lower respiratory infections	Bulgaria	2005	All Ages	0.4 (0.2 to 0.6)	18.7% (10.2 to 28.7%)	4.9 (2.6 to 7.4)
Lower respiratory infections	Bulgaria	2010	All Ages	0.4 (0.2 to 0.6)	20.9% (12.5 to 30.8%)	5.2 (3.0 to 7.6)
Lower respiratory infections	Bulgaria	2015	All Ages	0.4 (0.2 to 0.6)	21.7% (13.1 to 31.3%)	5.4 (3.2 to 7.9)
Lower respiratory infections	Bulgaria	1990	Age-standardized		29.4% (21.6 to 37.0%)	12.1 (9.0 to 15.4)
Lower respiratory infections	Bulgaria	1995	Age-standardized		24.8% (16.8 to 33.4%)	8.4 (5.6 to 11.2)
Lower respiratory infections	Bulgaria	2000	Age-standardized		22.2% (13.9 to 31.9%)	5.9 (3.7 to 8.4)
Lower respiratory infections	Bulgaria	2005	Age-standardized		18.7% (10.2 to 28.7%)	3.8 (2.1 to 5.8)
Lower respiratory infections	Bulgaria	2010	Age-standardized		20.9% (12.5 to 30.8%)	3.6 (2.1 to 5.4)
Lower respiratory infections	Bulgaria	2015	Age-standardized		21.7% (13.1 to 31.3%)	3.3 (1.9 to 4.8)
Tracheal, bronchus, and lung cancer	Bulgaria	1990	All Ages	0.7 (0.4 to 0.9)	21.4% (13.8 to 29.5%)	7.6 (4.9 to 10.6)
Tracheal, bronchus, and lung cancer	Bulgaria	1995	All Ages	0.6 (0.4 to 0.9)	17.8% (10.8 to 25.3%)	7.2 (4.3 to 10.3)
Tracheal, bronchus, and lung cancer	Bulgaria	2000	All Ages	0.5 (0.3 to 0.8)	15.8% (9.3 to 23.1%)	6.5 (3.8 to 9.5)
Tracheal, bronchus, and lung cancer	Bulgaria	2005	All Ages	0.5 (0.3 to 0.8)	13.2% (7.4 to 19.8%)	6.7 (3.8 to 10.2)
Tracheal, bronchus, and lung cancer	Bulgaria	2010	All Ages	0.6 (0.4 to 0.9)	14.8% (8.4 to 21.7%)	8.3 (4.7 to 12.3)
Tracheal, bronchus, and lung cancer	Bulgaria	2015	All Ages	0.6 (0.3 to 0.9)	15.4% (8.9 to 22.5%)	8.3 (4.7 to 12.2)
Tracheal, bronchus, and lung cancer	Bulgaria	1990	Age-standardized		21.4% (13.8 to 29.5%)	5.8 (3.7 to 8.1)
Tracheal, bronchus, and lung cancer	Bulgaria	1995	Age-standardized		17.8% (10.8 to 25.3%)	5.2 (3.1 to 7.4)
Tracheal, bronchus, and lung cancer	Bulgaria	2000	Age-standardized		15.8% (9.3 to 23.1%)	4.4 (2.6 to 6.5)
Tracheal, bronchus, and lung cancer	Bulgaria	2005	Age-standardized		13.2% (7.4 to 19.8%)	4.3 (2.4 to 6.6)
Tracheal, bronchus, and lung cancer	Bulgaria	2010	Age-standardized		14.8% (8.4 to 21.7%)	5.1 (2.9 to 7.5)

Cause name	Location	Year	Age group	Number (in thousands)	PAF	Rate (per 100,000)
Tracheal, bronchus, and lung cancer	Bulgaria	2015	Age-standardized		15.4% (8.9 to 22.5%)	4.8 (2.7 to 7.1)
Cardiovascular diseases	Bulgaria	1990	All Ages	10.2 (8.7 to 11.8)	15.1% (12.8 to 17.6%)	115.1 (98.2 to 133.8)
Cardiovascular diseases	Bulgaria	1995	All Ages	10.1 (8.4 to 11.9)	13.6% (11.5 to 16.0%)	120.4 (100.8 to 141.3)
Cardiovascular diseases	Bulgaria	2000	All Ages	9.1 (7.7 to 10.7)	12.3% (10.3 to 14.5%)	114.1 (95.7 to 134.0)
Cardiovascular diseases	Bulgaria	2005	All Ages	7.9 (6.5 to 9.4)	11.0% (9.1 to 13.0%)	102.9 (84.3 to 121.8)
Cardiovascular diseases	Bulgaria	2010	All Ages	7.7 (6.3 to 9.1)	11.3% (9.3 to 13.5%)	103.2 (84.4 to 123.0)
Cardiovascular diseases	Bulgaria	2015	All Ages	7.8 (6.3 to 9.4)	11.4% (9.2 to 13.6%)	107.5 (86.4 to 128.7)
Cardiovascular diseases	Bulgaria	1990	Age-standardized		14.8% (12.5 to 17.6%)	95.4 (80.4 to 113.3)
Cardiovascular diseases	Bulgaria	1995	Age-standardized		13.5% (11.2 to 16.0%)	90.8 (75.9 to 107.9)
Cardiovascular diseases	Bulgaria	2000	Age-standardized		12.1% (10.0 to 14.4%)	81.4 (67.0 to 96.6)
Cardiovascular diseases	Bulgaria	2005	Age-standardized		11.1% (9.0 to 13.1%)	64.8 (53.0 to 76.2)
Cardiovascular diseases	Bulgaria	2010	Age-standardized		11.5% (9.5 to 13.7%)	59.5 (49.0 to 70.6)
Cardiovascular diseases	Bulgaria	2015	Age-standardized		11.7% (9.6 to 13.8%)	56.6 (46.3 to 66.9)
Ischemic heart disease	Bulgaria	1990	All Ages	6.5 (5.2 to 8.0)	18.1% (14.5 to 22.3%)	73.1 (58.5 to 90.4)
Ischemic heart disease	Bulgaria	1995	All Ages	6.7 (5.2 to 8.2)	16.8% (13.3 to 20.6%)	79.6 (62.4 to 97.9)
Ischemic heart disease	Bulgaria	2000	All Ages	6.1 (4.8 to 7.5)	16.0% (12.5 to 19.5%)	76.5 (60.2 to 93.5)
Ischemic heart disease	Bulgaria	2005	All Ages	5.3 (4.0 to 6.5)	14.7% (11.3 to 18.1%)	68.5 (52.4 to 84.3)
Ischemic heart disease	Bulgaria	2010	All Ages	5.1 (3.8 to 6.3)	15.2% (11.5 to 18.9%)	68.4 (51.5 to 85.0)
Ischemic heart disease	Bulgaria	2015	All Ages	5.2 (3.9 to 6.6)	15.2% (11.3 to 19.1%)	72.0 (53.8 to 90.9)
Ischemic heart disease	Bulgaria	1990	Age-standardized		17.8% (13.9 to 22.2%)	61.1 (47.6 to 76.6)
Ischemic heart disease	Bulgaria	1995	Age-standardized		16.6% (12.8 to 20.5%)	60.4 (46.9 to 75.0)
Ischemic heart disease	Bulgaria	2000	Age-standardized		15.7% (12.1 to 19.5%)	55.1 (42.4 to 68.5)
Ischemic heart disease	Bulgaria	2005	Age-standardized		14.8% (11.4 to 18.3%)	43.4 (33.2 to 53.4)
Ischemic heart disease	Bulgaria	2010	Age-standardized		15.5% (11.9 to 19.1%)	39.7 (30.3 to 49.0)
Ischemic heart disease	Bulgaria	2015	Age-standardized		15.7% (12.1 to 19.5%)	38.1 (29.2 to 47.6)
Ischemic stroke	Bulgaria	1990	All Ages	1.7 (1.3 to 2.1)	14.3% (10.8 to 17.9%)	19.0 (14.3 to 23.8)
Ischemic stroke	Bulgaria	1995	All Ages	1.5 (1.1 to 1.9)	12.9% (9.4 to 16.3%)	17.8 (13.0 to 22.8)
Ischemic stroke	Bulgaria	2000	All Ages	1.3 (1.0 to 1.7)	12.0% (8.6 to 15.4%)	16.5 (11.9 to 21.4)
Ischemic stroke	Bulgaria	2005	All Ages	1.4 (1.0 to 1.9)	10.7% (7.4 to 14.3%)	18.1 (12.5 to 24.1)
Ischemic stroke	Bulgaria	2010	All Ages	1.5 (1.0 to 2.0)	11.3% (7.9 to 14.9%)	20.2 (14.0 to 27.1)
Ischemic stroke	Bulgaria	2015	All Ages	1.5 (1.0 to 2.0)	11.3% (7.8 to 15.3%)	20.5 (14.1 to 27.7)
Ischemic stroke	Bulgaria	1990	Age-standardized		14.0% (10.4 to 17.8%)	15.8 (11.7 to 20.0)
Ischemic stroke	Bulgaria	1995	Age-standardized		12.7% (9.2 to 16.2%)	13.4 (9.7 to 17.3)
Ischemic stroke	Bulgaria	2000	Age-standardized		11.8% (8.3 to 15.4%)	11.6 (8.2 to 15.3)
Ischemic stroke	Bulgaria	2005	Age-standardized		10.7% (7.4 to 14.3%)	11.1 (7.6 to 14.9)
Ischemic stroke	Bulgaria	2010	Age-standardized		11.4% (7.9 to 15.1%)	11.2 (7.8 to 15.0)

Cause name	Location	Year	Age group	Number (in thousands)	PAF	Rate (per 100,000)
Ischemic stroke	Bulgaria	2015	Age-standardized		11.5% (8.0 to 15.3%)	10.3 (7.2 to 13.7)
Hemorrhagic stroke	Bulgaria	1990	All Ages	2.0 (1.6 to 2.4)	15.8% (12.6 to 18.8%)	23.0 (18.3 to 27.7)
Hemorrhagic stroke	Bulgaria	1995	All Ages	1.9 (1.5 to 2.4)	14.2% (11.2 to 17.3%)	23.0 (17.9 to 28.0)
Hemorrhagic stroke	Bulgaria	2000	All Ages	1.7 (1.3 to 2.1)	13.1% (10.1 to 16.1%)	21.1 (16.2 to 26.0)
Hemorrhagic stroke	Bulgaria	2005	All Ages	1.3 (0.9 to 1.6)	11.7% (8.7 to 14.6%)	16.3 (12.1 to 20.5)
Hemorrhagic stroke	Bulgaria	2010	All Ages	1.1 (0.8 to 1.4)	12.2% (9.0 to 15.4%)	14.7 (10.8 to 18.5)
Hemorrhagic stroke	Bulgaria	2015	All Ages	1.1 (0.8 to 1.4)	12.3% (9.0 to 15.5%)	15.1 (11.2 to 19.3)
Hemorrhagic stroke	Bulgaria	1990	Age-standardized		15.5% (12.4 to 18.4%)	18.6 (14.8 to 22.4)
Hemorrhagic stroke	Bulgaria	1995	Age-standardized		14.1% (11.1 to 17.0%)	16.9 (13.3 to 20.6)
Hemorrhagic stroke	Bulgaria	2000	Age-standardized		13.0% (10.0 to 16.0%)	14.6 (11.2 to 18.0)
Hemorrhagic stroke	Bulgaria	2005	Age-standardized		11.9% (8.9 to 14.9%)	10.3 (7.8 to 12.9)
Hemorrhagic stroke	Bulgaria	2010	Age-standardized		12.5% (9.4 to 15.7%)	8.6 (6.5 to 10.7)
Hemorrhagic stroke	Bulgaria	2015	Age-standardized		12.7% (9.6 to 15.8%)	8.2 (6.2 to 10.3)
Chronic obstructive pulmonary disease	Bulgaria	1990	All Ages	0.8 (0.5 to 1.2)	27.9% (16.7 to 39.8%)	9.3 (5.7 to 13.4)
Chronic obstructive pulmonary disease	Bulgaria	1995	All Ages	0.7 (0.4 to 1.0)	24.4% (13.6 to 35.8%)	8.3 (4.6 to 12.2)
Chronic obstructive pulmonary disease	Bulgaria	2000	All Ages	0.7 (0.4 to 1.0)	22.4% (11.9 to 33.5%)	8.2 (4.4 to 12.2)
Chronic obstructive pulmonary disease	Bulgaria	2005	All Ages	0.6 (0.3 to 0.9)	19.7% (9.6 to 30.5%)	7.9 (3.9 to 12.3)
Chronic obstructive pulmonary disease	Bulgaria	2010	All Ages	0.7 (0.4 to 1.0)	21.5% (10.7 to 32.4%)	9.2 (4.8 to 14.0)
Chronic obstructive pulmonary disease	Bulgaria	2015	All Ages	0.7 (0.4 to 1.1)	22.0% (11.5 to 33.2%)	9.8 (5.2 to 14.8)
Chronic obstructive pulmonary disease	Bulgaria	1990	Age-standardized		27.9% (16.7 to 39.7%)	7.6 (4.6 to 11.0)
Chronic obstructive pulmonary disease	Bulgaria	1995	Age-standardized		24.4% (13.6 to 35.8%)	6.2 (3.5 to 9.1)
Chronic obstructive pulmonary disease	Bulgaria	2000	Age-standardized		22.4% (11.9 to 33.5%)	5.8 (3.1 to 8.6)
Chronic obstructive pulmonary disease	Bulgaria	2005	Age-standardized		19.7% (9.6 to 30.4%)	4.9 (2.4 to 7.6)
Chronic obstructive pulmonary disease	Bulgaria	2010	Age-standardized		21.4% (10.7 to 32.4%)	5.2 (2.7 to 7.9)
Chronic obstructive pulmonary disease	Bulgaria	2015	Age-standardized		22.0% (11.5 to 33.1%)	5.0 (2.6 to 7.6)
All causes	Croatia	1990	All Ages	3.8 (3.3 to 4.5)	7.5% (6.4 to 8.7%)	80.4 (68.5 to 93.5)
All causes	Croatia	1995	All Ages	3.6 (3.0 to 4.3)	7.0% (5.9 to 8.2%)	78.3 (65.5 to 92.2)
All causes	Croatia	2000	All Ages	3.5 (2.9 to 4.0)	6.7% (5.6 to 7.9%)	78.2 (65.4 to 91.1)
All causes	Croatia	2005	All Ages	3.2 (2.7 to 3.8)	6.4% (5.3 to 7.6%)	73.2 (60.8 to 86.7)
All causes	Croatia	2010	All Ages	3.2 (2.6 to 3.8)	6.2% (5.1 to 7.4%)	73.6 (60.2 to 87.9)
All causes	Croatia	2015	All Ages	3.5 (2.8 to 4.3)	6.7% (5.5 to 8.1%)	82.9 (67.0 to 100.2)
All causes	Croatia	1990	Age-standardized		7.3% (6.2 to 8.5%)	69.3 (58.7 to 80.9)
All causes	Croatia	1995	Age-standardized		6.8% (5.6 to 8.1%)	62.1 (51.6 to 73.4)
All causes	Croatia	2000	Age-standardized		6.5% (5.4 to 7.7%)	59.0 (48.8 to 69.5)
All causes	Croatia	2005	Age-standardized		6.2% (5.1 to 7.4%)	48.4 (39.9 to 57.8)
All causes	Croatia	2010	Age-standardized		6.0% (5.0 to 7.2%)	42.8 (35.3 to 50.8)

Cause name	Location	Year	Age group	Number (in thousands)	PAF	Rate (per 100,000)
All causes	Croatia	2015	Age-standardized		6.6% (5.4 to 7.8%)	43.2 (35.5 to 51.7)
Lower respiratory infections	Croatia	1990	All Ages	0.2 (0.1 to 0.3)	19.7% (11.1 to 29.3%)	4.2 (2.4 to 6.3)
Lower respiratory infections	Croatia	1995	All Ages	0.2 (0.1 to 0.3)	16.7% (8.3 to 27.0%)	4.1 (2.0 to 6.8)
Lower respiratory infections	Croatia	2000	All Ages	0.2 (0.1 to 0.3)	15.3% (6.9 to 25.8%)	4.3 (1.9 to 7.3)
Lower respiratory infections	Croatia	2005	All Ages	0.2 (0.1 to 0.3)	14.9% (6.5 to 25.5%)	3.7 (1.6 to 6.3)
Lower respiratory infections	Croatia	2010	All Ages	0.1 (0.1 to 0.2)	14.9% (6.6 to 25.4%)	2.8 (1.2 to 4.8)
Lower respiratory infections	Croatia	2015	All Ages	0.1 (0.1 to 0.2)	17.8% (9.4 to 28.0%)	3.2 (1.7 to 5.1)
Lower respiratory infections	Croatia	1990	Age-standardized		19.7% (11.1 to 29.3%)	3.9 (2.2 to 5.8)
Lower respiratory infections	Croatia	1995	Age-standardized		16.7% (8.3 to 27.0%)	3.5 (1.7 to 5.8)
Lower respiratory infections	Croatia	2000	Age-standardized		15.3% (6.9 to 25.8%)	3.5 (1.6 to 5.9)
Lower respiratory infections	Croatia	2005	Age-standardized		14.9% (6.5 to 25.5%)	2.5 (1.1 to 4.3)
Lower respiratory infections	Croatia	2010	Age-standardized		14.9% (6.6 to 25.4%)	1.6 (0.7 to 2.8)
Lower respiratory infections	Croatia	2015	Age-standardized		17.8% (9.4 to 28.0%)	1.7 (0.9 to 2.7)
Tracheal, bronchus, and lung cancer	Croatia	1990	All Ages	0.3 (0.2 to 0.5)	13.9% (8.0 to 20.7%)	7.1 (4.0 to 10.6)
Tracheal, bronchus, and lung cancer	Croatia	1995	All Ages	0.3 (0.2 to 0.5)	11.7% (6.3 to 18.1%)	6.3 (3.4 to 9.9)
Tracheal, bronchus, and lung cancer	Croatia	2000	All Ages	0.3 (0.1 to 0.4)	10.6% (5.7 to 16.6%)	6.5 (3.4 to 10.0)
Tracheal, bronchus, and lung cancer	Croatia	2005	All Ages	0.3 (0.1 to 0.4)	10.4% (5.5 to 16.3%)	6.5 (3.3 to 10.0)
Tracheal, bronchus, and lung cancer	Croatia	2010	All Ages	0.3 (0.1 to 0.5)	10.4% (5.5 to 16.2%)	6.6 (3.5 to 10.3)
Tracheal, bronchus, and lung cancer	Croatia	2015	All Ages	0.3 (0.2 to 0.5)	12.5% (6.9 to 19.1%)	7.8 (4.3 to 11.9)
Tracheal, bronchus, and lung cancer	Croatia	1990	Age-standardized		13.9% (8.0 to 20.7%)	5.7 (3.3 to 8.5)
Tracheal, bronchus, and lung cancer	Croatia	1995	Age-standardized		11.6% (6.3 to 18.1%)	4.7 (2.5 to 7.3)
Tracheal, bronchus, and lung cancer	Croatia	2000	Age-standardized		10.6% (5.7 to 16.6%)	4.5 (2.4 to 6.9)
Tracheal, bronchus, and lung cancer	Croatia	2005	Age-standardized		10.4% (5.5 to 16.3%)	4.2 (2.2 to 6.5)
Tracheal, bronchus, and lung cancer	Croatia	2010	Age-standardized		10.4% (5.5 to 16.2%)	4.1 (2.1 to 6.3)
Tracheal, bronchus, and lung cancer	Croatia	2015	Age-standardized		12.5% (6.9 to 19.1%)	4.5 (2.5 to 6.9)
Cardiovascular diseases	Croatia	1990	All Ages	3.1 (2.6 to 3.6)	11.5% (9.5 to 13.5%)	64.6 (53.5 to 76.4)
Cardiovascular diseases	Croatia	1995	All Ages	2.9 (2.4 to 3.5)	11.2% (9.1 to 13.2%)	63.2 (51.3 to 74.8)
Cardiovascular diseases	Croatia	2000	All Ages	2.8 (2.2 to 3.3)	10.4% (8.4 to 12.4%)	62.2 (50.6 to 74.3)
Cardiovascular diseases	Croatia	2005	All Ages	2.5 (2.0 to 3.0)	10.1% (8.1 to 12.1%)	57.3 (45.9 to 68.5)
Cardiovascular diseases	Croatia	2010	All Ages	2.5 (1.9 to 3.0)	10.0% (7.8 to 12.1%)	57.7 (45.0 to 70.6)
Cardiovascular diseases	Croatia	2015	All Ages	2.7 (2.1 to 3.4)	10.5% (8.2 to 13.0%)	64.2 (49.5 to 80.1)
Cardiovascular diseases	Croatia	1990	Age-standardized		11.3% (9.3 to 13.4%)	55.9 (46.0 to 66.4)
Cardiovascular diseases	Croatia	1995	Age-standardized		11.0% (8.9 to 13.1%)	50.3 (40.4 to 60.1)
Cardiovascular diseases	Croatia	2000	Age-standardized		10.1% (8.0 to 12.2%)	47.1 (37.3 to 56.9)
Cardiovascular diseases	Croatia	2005	Age-standardized		10.1% (8.0 to 12.2%)	38.0 (30.0 to 45.9)
Cardiovascular diseases	Croatia	2010	Age-standardized		10.1% (8.0 to 12.3%)	33.5 (26.4 to 40.8)

Cause name	Location	Year	Age group	Number (in thousands)	PAF	Rate (per 100,000)
Cardiovascular diseases	Croatia	2015	Age-standardized		10.9% (8.6 to 13.3%)	33.2 (26.0 to 40.5)
Ischemic heart disease	Croatia	1990	All Ages	2.0 (1.6 to 2.5)	15.2% (11.9 to 18.7%)	42.0 (32.5 to 51.7)
Ischemic heart disease	Croatia	1995	All Ages	1.9 (1.5 to 2.4)	14.1% (10.7 to 17.3%)	41.9 (31.7 to 51.7)
Ischemic heart disease	Croatia	2000	All Ages	1.8 (1.4 to 2.3)	13.5% (10.3 to 16.6%)	41.4 (31.6 to 51.2)
Ischemic heart disease	Croatia	2005	All Ages	1.7 (1.3 to 2.1)	13.1% (9.7 to 16.3%)	38.9 (28.9 to 48.7)
Ischemic heart disease	Croatia	2010	All Ages	1.7 (1.2 to 2.2)	12.7% (9.2 to 16.2%)	40.0 (28.8 to 51.1)
Ischemic heart disease	Croatia	2015	All Ages	1.9 (1.3 to 2.5)	13.4% (9.4 to 17.4%)	44.8 (31.5 to 58.6)
Ischemic heart disease	Croatia	1990	Age-standardized		15.0% (11.5 to 18.7%)	36.3 (27.8 to 45.1)
Ischemic heart disease	Croatia	1995	Age-standardized		13.8% (10.4 to 17.3%)	33.4 (25.1 to 41.8)
Ischemic heart disease	Croatia	2000	Age-standardized		13.2% (9.7 to 16.6%)	31.6 (23.3 to 40.2)
Ischemic heart disease	Croatia	2005	Age-standardized		13.1% (9.7 to 16.4%)	26.0 (19.1 to 32.8)
Ischemic heart disease	Croatia	2010	Age-standardized		13.0% (9.5 to 16.4%)	23.3 (17.0 to 29.7)
Ischemic heart disease	Croatia	2015	Age-standardized		13.9% (10.1 to 17.7%)	23.2 (16.9 to 29.6)
Ischemic stroke	Croatia	1990	All Ages	0.5 (0.4 to 0.7)	11.1% (7.8 to 14.5%)	11.3 (7.8 to 14.9)
Ischemic stroke	Croatia	1995	All Ages	0.5 (0.3 to 0.7)	10.1% (6.7 to 13.6%)	10.4 (7.0 to 14.3)
Ischemic stroke	Croatia	2000	All Ages	0.5 (0.3 to 0.6)	9.6% (6.3 to 12.9%)	10.6 (7.0 to 14.4)
Ischemic stroke	Croatia	2005	All Ages	0.4 (0.3 to 0.6)	9.3% (6.0 to 12.9%)	9.8 (6.3 to 13.6)
Ischemic stroke	Croatia	2010	All Ages	0.4 (0.3 to 0.6)	9.1% (5.7 to 12.9%)	9.9 (6.2 to 14.0)
Ischemic stroke	Croatia	2015	All Ages	0.5 (0.3 to 0.7)	9.9% (6.2 to 14.2%)	11.1 (7.0 to 16.1)
Ischemic stroke	Croatia	1990	Age-standardized		10.9% (7.5 to 14.5%)	9.9 (6.8 to 13.3)
Ischemic stroke	Croatia	1995	Age-standardized		9.9% (6.6 to 13.5%)	8.4 (5.6 to 11.6)
Ischemic stroke	Croatia	2000	Age-standardized		9.3% (5.9 to 13.2%)	8.0 (5.1 to 11.3)
Ischemic stroke	Croatia	2005	Age-standardized		9.2% (5.9 to 13.0%)	6.4 (4.0 to 9.0)
Ischemic stroke	Croatia	2010	Age-standardized		9.2% (5.8 to 13.1%)	5.5 (3.5 to 7.8)
Ischemic stroke	Croatia	2015	Age-standardized		10.1% (6.5 to 14.1%)	5.5 (3.5 to 7.7)
Hemorrhagic stroke	Croatia	1990	All Ages	0.5 (0.4 to 0.7)	12.6% (9.7 to 15.5%)	11.4 (8.8 to 14.1)
Hemorrhagic stroke	Croatia	1995	All Ages	0.5 (0.4 to 0.6)	11.4% (8.6 to 14.5%)	10.9 (8.2 to 13.9)
Hemorrhagic stroke	Croatia	2000	All Ages	0.5 (0.3 to 0.6)	10.6% (7.8 to 13.5%)	10.2 (7.3 to 13.0)
Hemorrhagic stroke	Croatia	2005	All Ages	0.4 (0.3 to 0.5)	10.3% (7.4 to 13.3%)	8.6 (6.2 to 11.3)
Hemorrhagic stroke	Croatia	2010	All Ages	0.3 (0.2 to 0.5)	10.0% (7.0 to 13.2%)	7.8 (5.5 to 10.4)
Hemorrhagic stroke	Croatia	2015	All Ages	0.4 (0.3 to 0.5)	10.8% (7.6 to 14.2%)	8.3 (5.8 to 11.0)
Hemorrhagic stroke	Croatia	1990	Age-standardized		12.4% (9.5 to 15.3%)	9.7 (7.5 to 12.0)
Hemorrhagic stroke	Croatia	1995	Age-standardized		11.3% (8.5 to 14.2%)	8.4 (6.4 to 10.7)
Hemorrhagic stroke	Croatia	2000	Age-standardized		10.5% (7.7 to 13.5%)	7.4 (5.4 to 9.6)
Hemorrhagic stroke	Croatia	2005	Age-standardized		10.3% (7.5 to 13.2%)	5.7 (4.1 to 7.4)
Hemorrhagic stroke	Croatia	2010	Age-standardized		10.2% (7.4 to 13.3%)	4.6 (3.3 to 6.1)

Cause name	Location	Year	Age group	Number (in thousands)	PAF	Rate (per 100,000)
Hemorrhagic stroke	Croatia	2015	Age-standardized		11.2% (8.1 to 14.3%)	4.5 (3.3 to 5.8)
Chronic obstructive pulmonary disease	Croatia	1990	All Ages	0.2 (0.1 to 0.3)	20.4% (10.4 to 31.2%)	4.4 (2.2 to 6.8)
Chronic obstructive pulmonary disease	Croatia	1995	All Ages	0.2 (0.1 to 0.3)	18.0% (8.3 to 28.8%)	4.6 (2.1 to 7.4)
Chronic obstructive pulmonary disease	Croatia	2000	All Ages	0.2 (0.1 to 0.4)	16.8% (7.6 to 27.5%)	5.3 (2.4 to 8.6)
Chronic obstructive pulmonary disease	Croatia	2005	All Ages	0.3 (0.1 to 0.4)	16.6% (7.3 to 27.2%)	5.7 (2.6 to 9.4)
Chronic obstructive pulmonary disease	Croatia	2010	All Ages	0.3 (0.1 to 0.5)	16.6% (7.5 to 27.3%)	6.4 (2.9 to 10.6)
Chronic obstructive pulmonary disease	Croatia	2015	All Ages	0.3 (0.2 to 0.5)	19.0% (9.2 to 29.9%)	7.8 (3.7 to 12.3)
Chronic obstructive pulmonary disease	Croatia	1990	Age-standardized		20.4% (10.4 to 31.2%)	3.8 (1.9 to 5.9)
Chronic obstructive pulmonary disease	Croatia	1995	Age-standardized		18.0% (8.3 to 28.8%)	3.7 (1.7 to 5.9)
Chronic obstructive pulmonary disease	Croatia	2000	Age-standardized		16.8% (7.6 to 27.5%)	3.9 (1.8 to 6.4)
Chronic obstructive pulmonary disease	Croatia	2005	Age-standardized		16.5% (7.3 to 27.1%)	3.7 (1.7 to 6.1)
Chronic obstructive pulmonary disease	Croatia	2010	Age-standardized		16.5% (7.5 to 27.3%)	3.6 (1.7 to 5.9)
Chronic obstructive pulmonary disease	Croatia	2015	Age-standardized		19.0% (9.2 to 29.9%)	3.9 (1.9 to 6.2)
All causes	Czech Republic	1990	All Ages	12.8 (11.0 to 14.7)	10.1% (8.7 to 11.7%)	124.1 (106.8 to 143.1)
All causes	Czech Republic	1995	All Ages	10.0 (8.5 to 11.7)	8.7% (7.3 to 10.1%)	97.5 (82.3 to 113.7)
All causes	Czech Republic	2000	All Ages	8.2 (6.9 to 9.6)	7.5% (6.3 to 8.8%)	79.7 (67.0 to 93.7)
All causes	Czech Republic	2005	All Ages	7.4 (6.1 to 8.8)	6.9% (5.7 to 8.2%)	72.3 (59.4 to 85.8)
All causes	Czech Republic	2010	All Ages	7.5 (6.0 to 9.0)	6.9% (5.6 to 8.3%)	71.6 (57.9 to 85.9)
All causes	Czech Republic	2015	All Ages	7.8 (6.2 to 9.4)	7.1% (5.7 to 8.6%)	72.6 (58.1 to 87.9)
All causes	Czech Republic	1990	Age-standardized		10.0% (8.6 to 11.5%)	104.4 (89.7 to 120.7)
All causes	Czech Republic	1995	Age-standardized		8.5% (7.2 to 9.9%)	79.4 (67.4 to 92.7)
All causes	Czech Republic	2000	Age-standardized		7.4% (6.1 to 8.7%)	63.3 (52.4 to 74.9)
All causes	Czech Republic	2005	Age-standardized		6.8% (5.6 to 8.1%)	51.0 (41.8 to 60.6)
All causes	Czech Republic	2010	Age-standardized		6.8% (5.5 to 8.1%)	45.7 (37.3 to 54.5)
All causes	Czech Republic	2015	Age-standardized		6.9% (5.6 to 8.3%)	42.4 (34.3 to 50.8)
Lower respiratory infections	Czech Republic	1990	All Ages	0.7 (0.5 to 0.9)	26.1% (18.2 to 34.2%)	6.5 (4.5 to 8.7)
Lower respiratory infections	Czech Republic	1995	All Ages	0.6 (0.3 to 0.8)	20.8% (12.3 to 30.7%)	5.3 (3.1 to 7.7)
Lower respiratory infections	Czech Republic	2000	All Ages	0.5 (0.2 to 0.8)	17.4% (8.8 to 27.7%)	4.6 (2.3 to 7.4)
Lower respiratory infections	Czech Republic	2005	All Ages	0.5 (0.2 to 0.8)	16.2% (7.7 to 26.6%)	4.9 (2.3 to 8.1)
Lower respiratory infections	Czech Republic	2010	All Ages	0.6 (0.3 to 0.9)	16.8% (8.3 to 27.1%)	5.4 (2.7 to 9.0)
Lower respiratory infections	Czech Republic	2015	All Ages	0.6 (0.3 to 1.0)	17.7% (9.1 to 27.8%)	5.7 (2.8 to 9.1)
Lower respiratory infections	Czech Republic	1990	Age-standardized		26.1% (18.2 to 34.2%)	5.7 (4.0 to 7.6)
Lower respiratory infections	Czech Republic	1995	Age-standardized		20.8% (12.3 to 30.7%)	4.5 (2.6 to 6.5)
Lower respiratory infections	Czech Republic	2000	Age-standardized		17.4% (8.8 to 27.7%)	3.9 (1.9 to 6.1)
Lower respiratory infections	Czech Republic	2005	Age-standardized		16.2% (7.7 to 26.6%)	3.5 (1.7 to 5.8)
Lower respiratory infections	Czech Republic	2010	Age-standardized		16.8% (8.3 to 27.1%)	3.5 (1.7 to 5.8)

Cause name	Location	Year	Age group	Number (in thousands)	PAF	Rate (per 100,000)
Lower respiratory infections	Czech Republic	2015	Age-standardized		17.7% (9.1 to 27.8%)	3.4 (1.7 to 5.4)
Tracheal, bronchus, and lung cancer	Czech Republic	1990	All Ages	1.1 (0.7 to 1.6)	18.8% (11.7 to 26.5%)	10.9 (6.7 to 15.5)
Tracheal, bronchus, and lung cancer	Czech Republic	1995	All Ages	0.8 (0.5 to 1.2)	14.7% (8.5 to 21.6%)	8.2 (4.7 to 12.1)
Tracheal, bronchus, and lung cancer	Czech Republic	2000	All Ages	0.7 (0.4 to 1.1)	12.2% (6.6 to 18.7%)	6.7 (3.7 to 10.2)
Tracheal, bronchus, and lung cancer	Czech Republic	2005	All Ages	0.6 (0.4 to 1.0)	11.3% (6.1 to 17.5%)	6.3 (3.4 to 9.7)
Tracheal, bronchus, and lung cancer	Czech Republic	2010	All Ages	0.7 (0.4 to 1.1)	11.8% (6.4 to 18.1%)	6.6 (3.5 to 10.1)
Tracheal, bronchus, and lung cancer	Czech Republic	2015	All Ages	0.7 (0.4 to 1.1)	12.4% (6.8 to 19.0%)	6.6 (3.6 to 10.1)
Tracheal, bronchus, and lung cancer	Czech Republic	1990	Age-standardized		18.8% (11.6 to 26.5%)	9.1 (5.6 to 12.9)
Tracheal, bronchus, and lung cancer	Czech Republic	1995	Age-standardized		14.7% (8.5 to 21.6%)	6.6 (3.8 to 9.8)
Tracheal, bronchus, and lung cancer	Czech Republic	2000	Age-standardized		12.2% (6.6 to 18.7%)	5.1 (2.8 to 7.7)
Tracheal, bronchus, and lung cancer	Czech Republic	2005	Age-standardized		11.3% (6.1 to 17.5%)	4.4 (2.4 to 6.8)
Tracheal, bronchus, and lung cancer	Czech Republic	2010	Age-standardized		11.8% (6.4 to 18.1%)	4.3 (2.3 to 6.6)
Tracheal, bronchus, and lung cancer	Czech Republic	2015	Age-standardized		12.4% (6.8 to 19.0%)	4.0 (2.2 to 6.1)
Cardiovascular diseases	Czech Republic	1990	All Ages	10.3 (8.7 to 12.2)	14.8% (12.4 to 17.4%)	100.3 (84.5 to 118.1)
Cardiovascular diseases	Czech Republic	1995	All Ages	8.3 (6.7 to 9.9)	13.1% (10.6 to 15.6%)	80.2 (65.3 to 96.0)
Cardiovascular diseases	Czech Republic	2000	All Ages	6.7 (5.4 to 7.9)	11.6% (9.4 to 13.9%)	64.9 (52.4 to 77.5)
Cardiovascular diseases	Czech Republic	2005	All Ages	5.9 (4.6 to 7.2)	10.9% (8.6 to 13.2%)	57.3 (45.0 to 69.8)
Cardiovascular diseases	Czech Republic	2010	All Ages	5.7 (4.4 to 7.1)	10.9% (8.4 to 13.5%)	55.0 (42.4 to 68.3)
Cardiovascular diseases	Czech Republic	2015	All Ages	5.9 (4.5 to 7.4)	11.0% (8.4 to 13.8%)	55.3 (41.9 to 69.4)
Cardiovascular diseases	Czech Republic	1990	Age-standardized		14.7% (12.4 to 17.4%)	84.2 (70.7 to 99.7)
Cardiovascular diseases	Czech Republic	1995	Age-standardized		13.0% (10.6 to 15.5%)	65.3 (53.4 to 77.7)
Cardiovascular diseases	Czech Republic	2000	Age-standardized		11.5% (9.1 to 13.9%)	51.7 (41.0 to 62.5)
Cardiovascular diseases	Czech Republic	2005	Age-standardized		10.9% (8.6 to 13.3%)	40.3 (31.6 to 49.2)
Cardiovascular diseases	Czech Republic	2010	Age-standardized		11.0% (8.5 to 13.5%)	35.0 (27.2 to 43.1)
Cardiovascular diseases	Czech Republic	2015	Age-standardized		11.2% (8.6 to 13.8%)	32.1 (24.8 to 39.7)
Ischemic heart disease	Czech Republic	1990	All Ages	7.2 (5.8 to 8.9)	17.3% (13.8 to 21.2%)	70.3 (56.0 to 86.3)
Ischemic heart disease	Czech Republic	1995	All Ages	6.0 (4.7 to 7.5)	15.5% (12.0 to 19.1%)	58.2 (45.2 to 72.6)
Ischemic heart disease	Czech Republic	2000	All Ages	4.8 (3.7 to 6.0)	14.2% (10.8 to 17.6%)	46.9 (35.7 to 58.1)
Ischemic heart disease	Czech Republic	2005	All Ages	4.4 (3.2 to 5.5)	13.4% (9.9 to 17.0%)	42.5 (31.3 to 53.9)
Ischemic heart disease	Czech Republic	2010	All Ages	4.4 (3.2 to 5.7)	13.4% (9.6 to 17.2%)	42.3 (30.3 to 54.4)
Ischemic heart disease	Czech Republic	2015	All Ages	4.6 (3.2 to 6.0)	13.5% (9.4 to 17.5%)	42.9 (29.8 to 56.1)
Ischemic heart disease	Czech Republic	1990	Age-standardized		17.3% (13.7 to 21.1%)	59.1 (46.8 to 72.7)
Ischemic heart disease	Czech Republic	1995	Age-standardized		15.4% (11.9 to 19.1%)	47.3 (36.7 to 58.8)
Ischemic heart disease	Czech Republic	2000	Age-standardized		14.0% (10.4 to 17.5%)	37.3 (27.9 to 46.9)
Ischemic heart disease	Czech Republic	2005	Age-standardized		13.5% (10.0 to 17.0%)	30.0 (22.0 to 37.8)
Ischemic heart disease	Czech Republic	2010	Age-standardized		13.6% (9.9 to 17.3%)	26.9 (19.5 to 34.4)

Cause name	Location	Year	Age group	Number (in thousands)	PAF	Rate (per 100,000)
Ischemic heart disease	Czech Republic	2015	Age-standardized		13.7% (9.9 to 17.6%)	24.9 (17.9 to 32.1)
Ischemic stroke	Czech Republic	1990	All Ages	2.0 (1.4 to 2.6)	12.8% (9.2 to 16.6%)	19.2 (13.9 to 25.2)
Ischemic stroke	Czech Republic	1995	All Ages	1.5 (1.0 to 2.0)	11.1% (7.5 to 15.1%)	14.3 (9.6 to 19.6)
Ischemic stroke	Czech Republic	2000	All Ages	1.2 (0.8 to 1.7)	10.0% (6.5 to 13.8%)	12.1 (7.9 to 16.8)
Ischemic stroke	Czech Republic	2005	All Ages	1.0 (0.6 to 1.5)	9.5% (5.9 to 13.7%)	10.1 (6.2 to 14.6)
Ischemic stroke	Czech Republic	2010	All Ages	0.9 (0.6 to 1.3)	9.6% (5.8 to 14.0%)	8.7 (5.3 to 12.8)
Ischemic stroke	Czech Republic	2015	All Ages	0.9 (0.5 to 1.3)	9.8% (5.8 to 14.4%)	8.5 (5.0 to 12.5)
Ischemic stroke	Czech Republic	1990	Age-standardized		12.7% (9.1 to 16.6%)	16.0 (11.5 to 21.0)
Ischemic stroke	Czech Republic	1995	Age-standardized		11.1% (7.5 to 15.1%)	11.7 (7.9 to 16.2)
Ischemic stroke	Czech Republic	2000	Age-standardized		9.8% (6.2 to 14.2%)	9.8 (6.1 to 14.1)
Ischemic stroke	Czech Republic	2005	Age-standardized		9.5% (5.9 to 13.8%)	7.0 (4.4 to 10.3)
Ischemic stroke	Czech Republic	2010	Age-standardized		9.7% (5.9 to 14.0%)	5.4 (3.4 to 7.9)
Ischemic stroke	Czech Republic	2015	Age-standardized		9.9% (6.1 to 14.3%)	4.8 (2.9 to 7.0)
Hemorrhagic stroke	Czech Republic	1990	All Ages	1.1 (0.9 to 1.4)	14.2% (11.1 to 17.3%)	10.8 (8.4 to 13.2)
Hemorrhagic stroke	Czech Republic	1995	All Ages	0.8 (0.6 to 1.0)	12.4% (9.2 to 15.5%)	7.7 (5.8 to 9.7)
Hemorrhagic stroke	Czech Republic	2000	All Ages	0.6 (0.4 to 0.8)	11.0% (8.1 to 14.2%)	5.9 (4.3 to 7.6)
Hemorrhagic stroke	Czech Republic	2005	All Ages	0.5 (0.3 to 0.6)	10.5% (7.4 to 13.7%)	4.7 (3.3 to 6.2)
Hemorrhagic stroke	Czech Republic	2010	All Ages	0.4 (0.3 to 0.6)	10.6% (7.4 to 14.0%)	4.0 (2.8 to 5.4)
Hemorrhagic stroke	Czech Republic	2015	All Ages	0.4 (0.3 to 0.6)	10.7% (7.4 to 14.3%)	3.9 (2.7 to 5.2)
Hemorrhagic stroke	Czech Republic	1990	Age-standardized		14.2% (11.1 to 17.3%)	9.1 (7.1 to 11.1)
Hemorrhagic stroke	Czech Republic	1995	Age-standardized		12.3% (9.3 to 15.5%)	6.2 (4.7 to 7.9)
Hemorrhagic stroke	Czech Republic	2000	Age-standardized		10.9% (7.8 to 14.1%)	4.6 (3.3 to 6.0)
Hemorrhagic stroke	Czech Republic	2005	Age-standardized		10.6% (7.5 to 13.8%)	3.3 (2.4 to 4.4)
Hemorrhagic stroke	Czech Republic	2010	Age-standardized		10.8% (7.8 to 14.0%)	2.6 (1.9 to 3.4)
Hemorrhagic stroke	Czech Republic	2015	Age-standardized		11.0% (7.8 to 14.3%)	2.3 (1.7 to 3.0)
Chronic obstructive pulmonary disease	Czech Republic	1990	All Ages	0.7 (0.4 to 1.0)	25.4% (14.5 to 36.8%)	6.4 (3.7 to 9.4)
Chronic obstructive pulmonary disease	Czech Republic	1995	All Ages	0.4 (0.2 to 0.6)	21.3% (10.9 to 32.1%)	3.8 (2.0 to 5.8)
Chronic obstructive pulmonary disease	Czech Republic	2000	All Ages	0.4 (0.2 to 0.6)	18.6% (8.9 to 29.4%)	3.5 (1.7 to 5.5)
Chronic obstructive pulmonary disease	Czech Republic	2005	All Ages	0.4 (0.2 to 0.7)	17.7% (8.1 to 28.4%)	3.9 (1.8 to 6.4)
Chronic obstructive pulmonary disease	Czech Republic	2010	All Ages	0.5 (0.2 to 0.8)	18.2% (8.6 to 29.0%)	4.6 (2.2 to 7.5)
Chronic obstructive pulmonary disease	Czech Republic	2015	All Ages	0.5 (0.3 to 0.9)	18.9% (9.0 to 29.7%)	5.0 (2.4 to 8.0)
Chronic obstructive pulmonary disease	Czech Republic	1990	Age-standardized		25.3% (14.5 to 36.8%)	5.3 (3.1 to 7.8)
Chronic obstructive pulmonary disease	Czech Republic	1995	Age-standardized		21.3% (10.9 to 32.1%)	3.1 (1.6 to 4.7)
Chronic obstructive pulmonary disease	Czech Republic	2000	Age-standardized		18.6% (8.9 to 29.4%)	2.7 (1.3 to 4.3)
Chronic obstructive pulmonary disease	Czech Republic	2005	Age-standardized		17.6% (8.1 to 28.4%)	2.7 (1.3 to 4.5)
Chronic obstructive pulmonary disease	Czech Republic	2010	Age-standardized		18.2% (8.6 to 28.9%)	2.9 (1.4 to 4.8)

Cause name	Location	Year	Age group	Number (in thousands)	PAF	Rate (per 100,000)
Chronic obstructive pulmonary disease	Czech Republic	2015	Age-standardized		18.9% (9.0 to 29.7%)	2.9 (1.4 to 4.7)
All causes	Hungary	1990	All Ages	13.2 (11.5 to 15.1)	9.1% (7.9 to 10.5%)	127.3 (110.6 to 145.8)
All causes	Hungary	1995	All Ages	11.8 (10.1 to 13.6)	8.1% (6.9 to 9.4%)	114.2 (97.3 to 131.8)
All causes	Hungary	2000	All Ages	10.1 (8.6 to 11.8)	7.4% (6.3 to 8.6%)	99.2 (84.4 to 115.6)
All causes	Hungary	2005	All Ages	9.4 (7.8 to 11.0)	7.0% (5.8 to 8.2%)	93.1 (77.6 to 109.5)
All causes	Hungary	2010	All Ages	9.3 (7.7 to 11.0)	7.1% (5.8 to 8.4%)	92.6 (76.4 to 109.9)
All causes	Hungary	2015	All Ages	8.6 (7.0 to 10.4)	7.0% (5.7 to 8.4%)	84.3 (68.4 to 101.9)
All causes	Hungary	1990	Age-standardized		8.9% (7.7 to 10.2%)	101.4 (87.8 to 116.0)
All causes	Hungary	1995	Age-standardized		7.9% (6.8 to 9.2%)	87.0 (74.3 to 100.5)
All causes	Hungary	2000	Age-standardized		7.2% (6.1 to 8.5%)	72.7 (61.3 to 84.9)
All causes	Hungary	2005	Age-standardized		6.8% (5.7 to 8.0%)	61.9 (51.8 to 72.7)
All causes	Hungary	2010	Age-standardized		6.9% (5.8 to 8.2%)	57.0 (47.5 to 67.2)
All causes	Hungary	2015	Age-standardized		6.8% (5.6 to 8.1%)	48.2 (39.8 to 57.6)
Lower respiratory infections	Hungary	1990	All Ages	0.4 (0.3 to 0.5)	26.6% (18.7 to 34.8%)	3.5 (2.5 to 4.6)
Lower respiratory infections	Hungary	1995	All Ages	0.3 (0.2 to 0.4)	21.9% (13.4 to 31.5%)	2.8 (1.7 to 4.0)
Lower respiratory infections	Hungary	2000	All Ages	0.2 (0.1 to 0.4)	19.5% (10.9 to 29.2%)	2.2 (1.2 to 3.4)
Lower respiratory infections	Hungary	2005	All Ages	0.2 (0.1 to 0.3)	17.9% (9.4 to 27.9%)	1.9 (1.0 to 3.1)
Lower respiratory infections	Hungary	2010	All Ages	0.2 (0.1 to 0.3)	18.6% (10.0 to 28.6%)	2.0 (1.0 to 3.1)
Lower respiratory infections	Hungary	2015	All Ages	0.2 (0.1 to 0.3)	18.7% (10.1 to 28.9%)	1.9 (1.0 to 3.0)
Lower respiratory infections	Hungary	1990	Age-standardized		26.6% (18.7 to 34.8%)	3.2 (2.2 to 4.2)
Lower respiratory infections	Hungary	1995	Age-standardized		21.9% (13.4 to 31.5%)	2.3 (1.4 to 3.4)
Lower respiratory infections	Hungary	2000	Age-standardized		19.5% (10.9 to 29.2%)	1.8 (1.0 to 2.8)
Lower respiratory infections	Hungary	2005	Age-standardized		17.9% (9.4 to 27.9%)	1.4 (0.7 to 2.2)
Lower respiratory infections	Hungary	2010	Age-standardized		18.6% (10.0 to 28.6%)	1.3 (0.7 to 2.0)
Lower respiratory infections	Hungary	2015	Age-standardized		18.7% (10.1 to 28.9%)	1.1 (0.6 to 1.8)
Tracheal, bronchus, and lung cancer	Hungary	1990	All Ages	1.3 (0.8 to 1.8)	19.2% (11.9 to 27.0%)	12.5 (7.7 to 17.6)
Tracheal, bronchus, and lung cancer	Hungary	1995	All Ages	1.2 (0.7 to 1.8)	15.6% (9.1 to 22.7%)	11.7 (6.8 to 17.1)
Tracheal, bronchus, and lung cancer	Hungary	2000	All Ages	1.1 (0.6 to 1.7)	13.7% (7.8 to 20.4%)	10.9 (6.1 to 16.4)
Tracheal, bronchus, and lung cancer	Hungary	2005	All Ages	1.0 (0.6 to 1.6)	12.6% (7.0 to 19.1%)	10.3 (5.7 to 15.7)
Tracheal, bronchus, and lung cancer	Hungary	2010	All Ages	1.1 (0.6 to 1.6)	13.1% (7.3 to 19.8%)	10.8 (6.0 to 16.3)
Tracheal, bronchus, and lung cancer	Hungary	2015	All Ages	1.0 (0.5 to 1.5)	13.2% (7.3 to 19.8%)	9.5 (5.3 to 14.3)
Tracheal, bronchus, and lung cancer	Hungary	1990	Age-standardized		19.2% (11.9 to 27.0%)	9.8 (6.1 to 13.8)
Tracheal, bronchus, and lung cancer	Hungary	1995	Age-standardized		15.6% (9.1 to 22.7%)	8.9 (5.2 to 13.0)
Tracheal, bronchus, and lung cancer	Hungary	2000	Age-standardized		13.7% (7.8 to 20.4%)	7.9 (4.4 to 11.9)
Tracheal, bronchus, and lung cancer	Hungary	2005	Age-standardized		12.6% (7.0 to 19.1%)	7.1 (3.9 to 10.7)
Tracheal, bronchus, and lung cancer	Hungary	2010	Age-standardized		13.1% (7.3 to 19.8%)	7.0 (3.9 to 10.6)

Cause name	Location	Year	Age group	Number (in thousands)	PAF	Rate (per 100,000)
Tracheal, bronchus, and lung cancer	Hungary	2015	Age-standardized		13.2% (7.3 to 19.8%)	5.9 (3.3 to 9.0)
Cardiovascular diseases	Hungary	1990	All Ages	10.3 (8.8 to 12.0)	13.9% (11.9 to 16.2%)	99.3 (84.5 to 115.4)
Cardiovascular diseases	Hungary	1995	All Ages	9.3 (7.7 to 10.9)	12.8% (10.7 to 15.1%)	89.9 (74.8 to 105.8)
Cardiovascular diseases	Hungary	2000	All Ages	8.0 (6.6 to 9.4)	11.9% (9.9 to 14.0%)	78.3 (64.8 to 92.4)
Cardiovascular diseases	Hungary	2005	All Ages	7.3 (5.9 to 8.8)	11.2% (9.1 to 13.4%)	72.4 (58.7 to 86.9)
Cardiovascular diseases	Hungary	2010	All Ages	7.0 (5.6 to 8.6)	11.1% (8.7 to 13.4%)	70.1 (55.4 to 85.4)
Cardiovascular diseases	Hungary	2015	All Ages	6.5 (5.1 to 8.1)	10.9% (8.4 to 13.4%)	63.9 (49.9 to 79.4)
Cardiovascular diseases	Hungary	1990	Age-standardized		13.9% (11.8 to 16.3%)	78.9 (66.8 to 91.8)
Cardiovascular diseases	Hungary	1995	Age-standardized		12.8% (10.8 to 15.1%)	68.4 (57.3 to 80.1)
Cardiovascular diseases	Hungary	2000	Age-standardized		11.9% (9.8 to 14.0%)	57.3 (47.2 to 67.7)
Cardiovascular diseases	Hungary	2005	Age-standardized		11.4% (9.3 to 13.6%)	47.9 (39.0 to 57.2)
Cardiovascular diseases	Hungary	2010	Age-standardized		11.3% (9.1 to 13.5%)	42.8 (34.2 to 51.5)
Cardiovascular diseases	Hungary	2015	Age-standardized		11.2% (8.8 to 13.6%)	36.0 (28.7 to 44.0)
Ischemic heart disease	Hungary	1990	All Ages	7.0 (5.7 to 8.5)	17.9% (14.5 to 21.6%)	67.7 (54.6 to 82.1)
Ischemic heart disease	Hungary	1995	All Ages	6.5 (5.1 to 7.9)	16.3% (12.8 to 19.8%)	63.0 (49.6 to 76.8)
Ischemic heart disease	Hungary	2000	All Ages	5.7 (4.4 to 7.0)	15.2% (11.8 to 18.6%)	55.5 (43.2 to 68.1)
Ischemic heart disease	Hungary	2005	All Ages	5.4 (4.1 to 6.7)	14.4% (10.9 to 17.9%)	53.7 (40.6 to 66.7)
Ischemic heart disease	Hungary	2010	All Ages	5.3 (4.0 to 6.7)	14.3% (10.6 to 18.0%)	53.3 (39.5 to 67.2)
Ischemic heart disease	Hungary	2015	All Ages	5.0 (3.6 to 6.4)	14.0% (10.0 to 17.9%)	49.0 (35.2 to 63.1)
Ischemic heart disease	Hungary	1990	Age-standardized		17.9% (14.3 to 21.6%)	53.8 (43.3 to 65.4)
Ischemic heart disease	Hungary	1995	Age-standardized		16.3% (12.9 to 19.8%)	47.9 (37.7 to 58.2)
Ischemic heart disease	Hungary	2000	Age-standardized		15.2% (11.6 to 18.7%)	40.7 (31.1 to 50.3)
Ischemic heart disease	Hungary	2005	Age-standardized		14.6% (11.2 to 18.1%)	35.6 (27.3 to 43.9)
Ischemic heart disease	Hungary	2010	Age-standardized		14.6% (11.1 to 18.2%)	32.5 (24.7 to 40.3)
Ischemic heart disease	Hungary	2015	Age-standardized		14.5% (10.8 to 18.2%)	27.7 (20.7 to 34.9)
Ischemic stroke	Hungary	1990	All Ages	2.1 (1.6 to 2.7)	13.5% (10.0 to 17.1%)	20.1 (15.0 to 25.6)
Ischemic stroke	Hungary	1995	All Ages	1.8 (1.3 to 2.4)	12.0% (8.6 to 15.5%)	17.7 (12.7 to 23.0)
Ischemic stroke	Hungary	2000	All Ages	1.6 (1.1 to 2.1)	11.1% (7.8 to 14.5%)	15.6 (10.9 to 20.6)
Ischemic stroke	Hungary	2005	All Ages	1.3 (0.9 to 1.8)	10.5% (7.2 to 14.0%)	13.1 (9.0 to 17.8)
Ischemic stroke	Hungary	2010	All Ages	1.2 (0.8 to 1.7)	10.5% (7.1 to 14.3%)	12.0 (8.1 to 16.5)
Ischemic stroke	Hungary	2015	All Ages	1.1 (0.7 to 1.5)	10.4% (6.7 to 14.6%)	10.7 (6.9 to 15.0)
Ischemic stroke	Hungary	1990	Age-standardized		13.5% (9.9 to 17.0%)	15.8 (11.8 to 20.2)
Ischemic stroke	Hungary	1995	Age-standardized		12.0% (8.6 to 15.4%)	13.3 (9.6 to 17.3)
Ischemic stroke	Hungary	2000	Age-standardized		11.0% (7.7 to 14.6%)	11.3 (7.8 to 15.0)
Ischemic stroke	Hungary	2005	Age-standardized		10.6% (7.3 to 14.1%)	8.5 (5.8 to 11.4)
Ischemic stroke	Hungary	2010	Age-standardized		10.7% (7.4 to 14.4%)	7.1 (4.9 to 9.5)

Cause name	Location	Year	Age group	Number (in thousands)	PAF	Rate (per 100,000)
Ischemic stroke	Hungary	2015	Age-standardized		10.6% (7.2 to 14.4%)	5.8 (3.9 to 8.0)
Hemorrhagic stroke	Hungary	1990	All Ages	1.2 (1.0 to 1.4)	15.3% (12.4 to 18.2%)	11.5 (9.3 to 13.7)
Hemorrhagic stroke	Hungary	1995	All Ages	1.0 (0.8 to 1.2)	13.8% (10.9 to 16.8%)	9.3 (7.3 to 11.4)
Hemorrhagic stroke	Hungary	2000	All Ages	0.7 (0.6 to 0.9)	12.8% (10.0 to 15.7%)	7.2 (5.6 to 8.8)
Hemorrhagic stroke	Hungary	2005	All Ages	0.6 (0.4 to 0.7)	12.1% (9.3 to 15.0%)	5.5 (4.2 to 6.9)
Hemorrhagic stroke	Hungary	2010	All Ages	0.5 (0.4 to 0.6)	12.0% (9.2 to 15.1%)	4.8 (3.7 to 6.1)
Hemorrhagic stroke	Hungary	2015	All Ages	0.4 (0.3 to 0.6)	11.8% (8.8 to 14.9%)	4.2 (3.1 to 5.4)
Hemorrhagic stroke	Hungary	1990	Age-standardized		15.4% (12.4 to 18.2%)	9.2 (7.4 to 11.0)
Hemorrhagic stroke	Hungary	1995	Age-standardized		14.0% (11.1 to 16.8%)	7.2 (5.7 to 8.7)
Hemorrhagic stroke	Hungary	2000	Age-standardized		12.9% (10.1 to 15.8%)	5.3 (4.1 to 6.5)
Hemorrhagic stroke	Hungary	2005	Age-standardized		12.4% (9.6 to 15.2%)	3.8 (3.0 to 4.7)
Hemorrhagic stroke	Hungary	2010	Age-standardized		12.4% (9.7 to 15.4%)	3.1 (2.4 to 3.9)
Hemorrhagic stroke	Hungary	2015	Age-standardized		12.3% (9.4 to 15.2%)	2.5 (1.9 to 3.2)
Chronic obstructive pulmonary disease	Hungary	1990	All Ages	1.3 (0.7 to 1.8)	25.8% (15.0 to 37.1%)	12.0 (7.0 to 17.4)
Chronic obstructive pulmonary disease	Hungary	1995	All Ages	1.0 (0.5 to 1.5)	22.2% (11.7 to 33.3%)	9.8 (5.1 to 14.7)
Chronic obstructive pulmonary disease	Hungary	2000	All Ages	0.8 (0.4 to 1.2)	20.3% (10.1 to 31.3%)	7.8 (3.9 to 12.1)
Chronic obstructive pulmonary disease	Hungary	2005	All Ages	0.9 (0.4 to 1.3)	19.1% (9.1 to 30.0%)	8.5 (4.1 to 13.3)
Chronic obstructive pulmonary disease	Hungary	2010	All Ages	1.0 (0.5 to 1.5)	19.6% (9.5 to 30.5%)	9.8 (4.8 to 15.2)
Chronic obstructive pulmonary disease	Hungary	2015	All Ages	0.9 (0.5 to 1.4)	19.7% (9.6 to 30.7%)	9.0 (4.4 to 13.9)
Chronic obstructive pulmonary disease	Hungary	1990	Age-standardized		25.7% (14.9 to 37.0%)	9.5 (5.6 to 13.8)
Chronic obstructive pulmonary disease	Hungary	1995	Age-standardized		22.1% (11.7 to 33.2%)	7.4 (3.9 to 11.1)
Chronic obstructive pulmonary disease	Hungary	2000	Age-standardized		20.2% (10.1 to 31.2%)	5.7 (2.8 to 8.8)
Chronic obstructive pulmonary disease	Hungary	2005	Age-standardized		19.0% (9.1 to 30.0%)	5.6 (2.7 to 8.7)
Chronic obstructive pulmonary disease	Hungary	2010	Age-standardized		19.5% (9.5 to 30.5%)	6.0 (2.9 to 9.3)
Chronic obstructive pulmonary disease	Hungary	2015	Age-standardized		19.7% (9.6 to 30.6%)	5.1 (2.5 to 7.9)
All causes	Macedonia	1990	All Ages	1.5 (1.3 to 1.6)	10.2% (9.0 to 11.4%)	72.8 (64.5 to 81.4)
All causes	Macedonia	1995	All Ages	1.5 (1.3 to 1.7)	9.8% (8.6 to 11.1%)	76.5 (67.2 to 86.7)
All causes	Macedonia	2000	All Ages	1.5 (1.3 to 1.7)	9.3% (8.2 to 10.6%)	75.2 (65.8 to 85.3)
All causes	Macedonia	2005	All Ages	1.5 (1.3 to 1.7)	8.6% (7.4 to 9.8%)	72.6 (62.9 to 83.5)
All causes	Macedonia	2010	All Ages	1.6 (1.4 to 1.9)	9.0% (7.7 to 10.4%)	77.4 (66.7 to 89.6)
All causes	Macedonia	2015	All Ages	1.6 (1.4 to 1.9)	9.3% (8.0 to 10.6%)	77.8 (66.6 to 90.2)
All causes	Macedonia	1990	Age-standardized		10.1% (8.8 to 11.5%)	97.7 (85.1 to 111.0)
All causes	Macedonia	1995	Age-standardized		9.6% (8.3 to 11.0%)	98.6 (85.3 to 113.1)
All causes	Macedonia	2000	Age-standardized		9.0% (7.7 to 10.4%)	89.6 (76.6 to 103.6)
All causes	Macedonia	2005	Age-standardized		8.3% (7.0 to 9.6%)	74.6 (63.3 to 86.9)
All causes	Macedonia	2010	Age-standardized		8.7% (7.4 to 10.1%)	71.1 (60.4 to 82.8)

Cause name	Location	Year	Age group	Number (in thousands)	PAF	Rate (per 100,000)
All causes	Macedonia	2015	Age-standardized		9.0% (7.7 to 10.5%)	64.4 (54.6 to 75.2)
Lower respiratory infections	Macedonia	1990	All Ages	0.1 (0.1 to 0.1)	31.7% (23.9 to 39.2%)	4.3 (3.2 to 5.6)
Lower respiratory infections	Macedonia	1995	All Ages	0.1 (0.0 to 0.1)	28.1% (20.0 to 36.0%)	2.8 (2.0 to 3.8)
Lower respiratory infections	Macedonia	2000	All Ages	0.0 (0.0 to 0.1)	25.5% (17.7 to 33.8%)	1.9 (1.2 to 2.8)
Lower respiratory infections	Macedonia	2005	All Ages	0.0 (0.0 to 0.1)	22.1% (13.8 to 31.6%)	1.6 (1.0 to 2.6)
Lower respiratory infections	Macedonia	2010	All Ages	0.0 (0.0 to 0.1)	25.1% (16.9 to 33.6%)	1.8 (1.1 to 2.7)
Lower respiratory infections	Macedonia	2015	All Ages	0.0 (0.0 to 0.1)	27.5% (19.8 to 35.5%)	1.9 (1.3 to 2.9)
Lower respiratory infections	Macedonia	1990	Age-standardized		31.7% (23.9 to 39.2%)	5.3 (3.9 to 6.8)
Lower respiratory infections	Macedonia	1995	Age-standardized		28.1% (20.0 to 36.0%)	3.7 (2.7 to 4.9)
Lower respiratory infections	Macedonia	2000	Age-standardized		25.5% (17.7 to 33.8%)	2.5 (1.6 to 3.8)
Lower respiratory infections	Macedonia	2005	Age-standardized		22.1% (13.8 to 31.6%)	1.9 (1.2 to 3.1)
Lower respiratory infections	Macedonia	2010	Age-standardized		25.1% (16.9 to 33.6%)	1.9 (1.2 to 2.8)
Lower respiratory infections	Macedonia	2015	Age-standardized		27.5% (19.8 to 35.5%)	1.9 (1.3 to 2.8)
Tracheal, bronchus, and lung cancer	Macedonia	1990	All Ages	0.1 (0.1 to 0.1)	23.4% (15.4 to 31.6%)	5.5 (3.6 to 7.6)
Tracheal, bronchus, and lung cancer	Macedonia	1995	All Ages	0.1 (0.1 to 0.2)	20.3% (12.8 to 28.3%)	5.6 (3.6 to 8.0)
Tracheal, bronchus, and lung cancer	Macedonia	2000	All Ages	0.1 (0.1 to 0.2)	18.3% (11.2 to 25.9%)	5.9 (3.6 to 8.5)
Tracheal, bronchus, and lung cancer	Macedonia	2005	All Ages	0.1 (0.1 to 0.2)	15.7% (9.2 to 23.0%)	5.8 (3.4 to 8.6)
Tracheal, bronchus, and lung cancer	Macedonia	2010	All Ages	0.1 (0.1 to 0.2)	18.0% (10.8 to 25.6%)	7.1 (4.3 to 10.2)
Tracheal, bronchus, and lung cancer	Macedonia	2015	All Ages	0.2 (0.1 to 0.2)	19.9% (12.3 to 27.7%)	7.7 (4.7 to 11.0)
Tracheal, bronchus, and lung cancer	Macedonia	1990	Age-standardized		23.4% (15.4 to 31.6%)	6.3 (4.1 to 8.7)
Tracheal, bronchus, and lung cancer	Macedonia	1995	Age-standardized		20.3% (12.8 to 28.3%)	6.1 (3.9 to 8.7)
Tracheal, bronchus, and lung cancer	Macedonia	2000	Age-standardized		18.3% (11.2 to 25.9%)	5.9 (3.6 to 8.4)
Tracheal, bronchus, and lung cancer	Macedonia	2005	Age-standardized		15.7% (9.2 to 23.0%)	5.3 (3.1 to 7.8)
Tracheal, bronchus, and lung cancer	Macedonia	2010	Age-standardized		18.0% (10.8 to 25.6%)	5.9 (3.6 to 8.5)
Tracheal, bronchus, and lung cancer	Macedonia	2015	Age-standardized		19.9% (12.3 to 27.6%)	5.9 (3.6 to 8.5)
Cardiovascular diseases	Macedonia	1990	All Ages	1.1 (1.0 to 1.3)	14.6% (12.7 to 16.8%)	57.1 (49.5 to 65.1)
Cardiovascular diseases	Macedonia	1995	All Ages	1.2 (1.1 to 1.4)	13.8% (11.8 to 15.8%)	62.5 (53.6 to 71.9)
Cardiovascular diseases	Macedonia	2000	All Ages	1.3 (1.1 to 1.5)	13.2% (11.3 to 15.1%)	62.7 (53.8 to 72.2)
Cardiovascular diseases	Macedonia	2005	All Ages	1.2 (1.1 to 1.4)	12.1% (10.2 to 14.0%)	60.6 (51.5 to 70.3)
Cardiovascular diseases	Macedonia	2010	All Ages	1.3 (1.1 to 1.5)	12.5% (10.6 to 14.6%)	63.7 (53.9 to 74.2)
Cardiovascular diseases	Macedonia	2015	All Ages	1.3 (1.1 to 1.5)	13.0% (11.0 to 15.1%)	63.1 (52.6 to 74.3)
Cardiovascular diseases	Macedonia	1990	Age-standardized		13.6% (11.6 to 15.9%)	77.5 (66.0 to 90.2)
Cardiovascular diseases	Macedonia	1995	Age-standardized		12.7% (10.8 to 14.9%)	81.0 (68.9 to 94.8)
Cardiovascular diseases	Macedonia	2000	Age-standardized		12.1% (10.1 to 14.3%)	75.1 (62.9 to 88.4)
Cardiovascular diseases	Macedonia	2005	Age-standardized		11.4% (9.4 to 13.5%)	62.4 (51.9 to 73.6)
Cardiovascular diseases	Macedonia	2010	Age-standardized		12.0% (10.0 to 14.2%)	58.7 (48.7 to 69.1)

Cause name	Location	Year	Age group	Number (in thousands)	PAF	Rate (per 100,000)
Cardiovascular diseases	Macedonia	2015	Age-standardized		12.6% (10.5 to 14.8%)	52.3 (43.0 to 62.0)
Ischemic heart disease	Macedonia	1990	All Ages	0.6 (0.5 to 0.7)	20.7% (17.4 to 24.3%)	30.1 (25.2 to 35.9)
Ischemic heart disease	Macedonia	1995	All Ages	0.7 (0.5 to 0.8)	19.4% (16.1 to 23.1%)	33.2 (27.4 to 39.7)
Ischemic heart disease	Macedonia	2000	All Ages	0.7 (0.6 to 0.8)	18.5% (15.3 to 21.9%)	33.2 (27.5 to 39.5)
Ischemic heart disease	Macedonia	2005	All Ages	0.7 (0.6 to 0.8)	17.0% (13.9 to 20.3%)	32.7 (26.7 to 39.5)
Ischemic heart disease	Macedonia	2010	All Ages	0.7 (0.6 to 0.8)	17.6% (14.2 to 21.1%)	33.3 (26.7 to 40.3)
Ischemic heart disease	Macedonia	2015	All Ages	0.7 (0.6 to 0.8)	18.2% (14.7 to 22.0%)	33.5 (26.7 to 41.0)
Ischemic heart disease	Macedonia	1990	Age-standardized		19.5% (15.9 to 23.7%)	39.6 (32.2 to 48.3)
Ischemic heart disease	Macedonia	1995	Age-standardized		18.2% (14.8 to 22.4%)	41.6 (33.7 to 51.6)
Ischemic heart disease	Macedonia	2000	Age-standardized		17.3% (13.8 to 21.2%)	38.8 (31.0 to 47.8)
Ischemic heart disease	Macedonia	2005	Age-standardized		16.2% (12.9 to 19.7%)	33.3 (26.2 to 41.1)
Ischemic heart disease	Macedonia	2010	Age-standardized		17.0% (13.5 to 20.7%)	30.4 (23.9 to 37.2)
Ischemic heart disease	Macedonia	2015	Age-standardized		17.8% (14.2 to 21.8%)	27.6 (21.7 to 34.1)
Ischemic stroke	Macedonia	1990	All Ages	0.3 (0.2 to 0.4)	15.2% (11.8 to 18.8%)	13.8 (10.7 to 17.4)
Ischemic stroke	Macedonia	1995	All Ages	0.3 (0.2 to 0.4)	14.0% (10.6 to 17.5%)	14.8 (11.0 to 18.6)
Ischemic stroke	Macedonia	2000	All Ages	0.3 (0.2 to 0.4)	13.3% (9.9 to 16.6%)	15.1 (11.2 to 19.0)
Ischemic stroke	Macedonia	2005	All Ages	0.3 (0.2 to 0.4)	12.1% (8.7 to 15.4%)	14.7 (10.7 to 19.1)
Ischemic stroke	Macedonia	2010	All Ages	0.3 (0.3 to 0.5)	12.8% (9.2 to 16.2%)	16.7 (12.2 to 21.6)
Ischemic stroke	Macedonia	2015	All Ages	0.3 (0.3 to 0.4)	13.4% (9.7 to 17.0%)	16.3 (12.0 to 21.1)
Ischemic stroke	Macedonia	1990	Age-standardized		14.6% (10.9 to 18.4%)	20.5 (15.3 to 26.3)
Ischemic stroke	Macedonia	1995	Age-standardized		13.4% (9.8 to 17.2%)	21.3 (15.5 to 27.6)
Ischemic stroke	Macedonia	2000	Age-standardized		12.6% (9.0 to 16.4%)	19.7 (14.1 to 26.2)
Ischemic stroke	Macedonia	2005	Age-standardized		11.7% (8.3 to 15.2%)	16.0 (11.3 to 21.4)
Ischemic stroke	Macedonia	2010	Age-standardized		12.5% (9.0 to 16.1%)	16.0 (11.4 to 21.1)
Ischemic stroke	Macedonia	2015	Age-standardized		13.2% (9.4 to 17.1%)	13.9 (9.9 to 18.1)
Hemorrhagic stroke	Macedonia	1990	All Ages	0.3 (0.2 to 0.3)	16.9% (13.9 to 19.8%)	13.2 (10.8 to 15.6)
Hemorrhagic stroke	Macedonia	1995	All Ages	0.3 (0.2 to 0.3)	15.6% (12.5 to 18.6%)	14.5 (11.6 to 17.4)
Hemorrhagic stroke	Macedonia	2000	All Ages	0.3 (0.2 to 0.4)	14.7% (11.6 to 17.7%)	14.4 (11.4 to 17.4)
Hemorrhagic stroke	Macedonia	2005	All Ages	0.3 (0.2 to 0.3)	13.4% (10.4 to 16.6%)	13.1 (10.2 to 16.3)
Hemorrhagic stroke	Macedonia	2010	All Ages	0.3 (0.2 to 0.4)	14.1% (11.1 to 17.2%)	13.8 (10.7 to 17.0)
Hemorrhagic stroke	Macedonia	2015	All Ages	0.3 (0.2 to 0.3)	14.8% (11.7 to 17.8%)	13.3 (10.4 to 16.4)
Hemorrhagic stroke	Macedonia	1990	Age-standardized		16.0% (12.9 to 19.1%)	17.5 (13.9 to 21.0)
Hemorrhagic stroke	Macedonia	1995	Age-standardized		14.7% (11.6 to 17.8%)	18.1 (14.1 to 22.0)
Hemorrhagic stroke	Macedonia	2000	Age-standardized		13.9% (10.7 to 16.9%)	16.5 (12.8 to 20.4)
Hemorrhagic stroke	Macedonia	2005	Age-standardized		12.8% (9.9 to 15.8%)	13.1 (10.0 to 16.4)
Hemorrhagic stroke	Macedonia	2010	Age-standardized		13.7% (10.6 to 16.8%)	12.4 (9.5 to 15.5)

Cause name	Location	Year	Age group	Number (in thousands)	PAF	Rate (per 100,000)
Hemorrhagic stroke	Macedonia	2015	Age-standardized		14.5% (11.3 to 17.5%)	10.9 (8.4 to 13.6)
Chronic obstructive pulmonary disease	Macedonia	1990	All Ages	0.1 (0.1 to 0.2)	29.5% (17.9 to 41.8%)	5.8 (3.6 to 8.3)
Chronic obstructive pulmonary disease	Macedonia	1995	All Ages	0.1 (0.1 to 0.2)	26.7% (15.9 to 38.1%)	5.5 (3.3 to 7.9)
Chronic obstructive pulmonary disease	Macedonia	2000	All Ages	0.1 (0.1 to 0.1)	24.9% (14.1 to 36.2%)	4.7 (2.7 to 6.8)
Chronic obstructive pulmonary disease	Macedonia	2005	All Ages	0.1 (0.1 to 0.1)	22.3% (11.7 to 33.2%)	4.6 (2.4 to 6.9)
Chronic obstructive pulmonary disease	Macedonia	2010	All Ages	0.1 (0.1 to 0.1)	24.6% (13.8 to 35.8%)	4.9 (2.7 to 7.1)
Chronic obstructive pulmonary disease	Macedonia	2015	All Ages	0.1 (0.1 to 0.1)	26.5% (15.5 to 38.0%)	5.1 (2.9 to 7.4)
Chronic obstructive pulmonary disease	Macedonia	1990	Age-standardized		29.6% (18.0 to 42.0%)	8.6 (5.3 to 12.3)
Chronic obstructive pulmonary disease	Macedonia	1995	Age-standardized		26.8% (15.9 to 38.2%)	7.7 (4.6 to 11.2)
Chronic obstructive pulmonary disease	Macedonia	2000	Age-standardized		24.9% (14.1 to 36.2%)	6.1 (3.5 to 9.0)
Chronic obstructive pulmonary disease	Macedonia	2005	Age-standardized		22.3% (11.7 to 33.2%)	5.0 (2.6 to 7.6)
Chronic obstructive pulmonary disease	Macedonia	2010	Age-standardized		24.5% (13.8 to 35.8%)	4.6 (2.6 to 6.8)
Chronic obstructive pulmonary disease	Macedonia	2015	Age-standardized		26.4% (15.5 to 38.0%)	4.3 (2.5 to 6.3)
All causes	Montenegro	1990	All Ages	0.3 (0.3 to 0.4)	7.8% (6.6 to 9.2%)	55.9 (46.4 to 65.3)
All causes	Montenegro	1995	All Ages	0.4 (0.3 to 0.5)	7.5% (6.2 to 8.9%)	64.2 (52.7 to 76.0)
All causes	Montenegro	2000	All Ages	0.4 (0.3 to 0.5)	7.4% (6.2 to 8.7%)	67.3 (56.0 to 79.2)
All causes	Montenegro	2005	All Ages	0.4 (0.3 to 0.5)	7.2% (6.0 to 8.4%)	63.4 (53.0 to 74.8)
All causes	Montenegro	2010	All Ages	0.4 (0.3 to 0.5)	7.7% (6.4 to 9.0%)	64.1 (53.4 to 75.3)
All causes	Montenegro	2015	All Ages	0.4 (0.4 to 0.5)	8.0% (6.7 to 9.4%)	70.1 (58.0 to 82.6)
All causes	Montenegro	1990	Age-standardized		7.9% (6.6 to 9.2%)	61.3 (50.7 to 71.7)
All causes	Montenegro	1995	Age-standardized		7.4% (6.1 to 8.8%)	65.3 (53.6 to 77.4)
All causes	Montenegro	2000	Age-standardized		7.2% (5.9 to 8.6%)	65.5 (53.5 to 78.2)
All causes	Montenegro	2005	Age-standardized		7.0% (5.8 to 8.3%)	55.1 (45.2 to 65.8)
All causes	Montenegro	2010	Age-standardized		7.5% (6.2 to 8.9%)	51.6 (42.7 to 60.8)
All causes	Montenegro	2015	Age-standardized		7.8% (6.5 to 9.2%)	51.8 (42.6 to 61.0)
Lower respiratory infections	Montenegro	1990	All Ages	0.0 (0.0 to 0.0)	19.2% (10.7 to 29.1%)	2.1 (1.1 to 3.4)
Lower respiratory infections	Montenegro	1995	All Ages	0.0 (0.0 to 0.0)	16.5% (7.9 to 26.9%)	1.9 (0.9 to 3.2)
Lower respiratory infections	Montenegro	2000	All Ages	0.0 (0.0 to 0.0)	15.6% (7.2 to 25.9%)	1.9 (0.8 to 3.2)
Lower respiratory infections	Montenegro	2005	All Ages	0.0 (0.0 to 0.0)	14.7% (6.2 to 25.2%)	1.5 (0.6 to 2.6)
Lower respiratory infections	Montenegro	2010	All Ages	0.0 (0.0 to 0.0)	17.4% (8.9 to 27.5%)	1.6 (0.8 to 2.6)
Lower respiratory infections	Montenegro	2015	All Ages	0.0 (0.0 to 0.0)	18.9% (10.2 to 28.8%)	1.8 (1.0 to 2.8)
Lower respiratory infections	Montenegro	1990	Age-standardized		19.2% (10.7 to 29.1%)	2.3 (1.3 to 3.7)
Lower respiratory infections	Montenegro	1995	Age-standardized		16.5% (7.9 to 26.9%)	2.0 (1.0 to 3.4)
Lower respiratory infections	Montenegro	2000	Age-standardized		15.6% (7.2 to 25.9%)	2.1 (0.9 to 3.5)
Lower respiratory infections	Montenegro	2005	Age-standardized		14.7% (6.2 to 25.2%)	1.4 (0.6 to 2.5)
Lower respiratory infections	Montenegro	2010	Age-standardized		17.4% (8.9 to 27.5%)	1.4 (0.7 to 2.3)

Cause name	Location	Year	Age group	Number (in thousands)	PAF	Rate (per 100,000)
Lower respiratory infections	Montenegro	2015	Age-standardized		18.9% (10.2 to 28.8%)	1.4 (0.8 to 2.2)
Tracheal, bronchus, and lung cancer	Montenegro	1990	All Ages	0.0 (0.0 to 0.1)	13.5% (7.6 to 20.3%)	5.3 (3.0 to 8.0)
Tracheal, bronchus, and lung cancer	Montenegro	1995	All Ages	0.0 (0.0 to 0.1)	11.5% (6.2 to 17.9%)	5.5 (2.9 to 8.4)
Tracheal, bronchus, and lung cancer	Montenegro	2000	All Ages	0.0 (0.0 to 0.1)	10.9% (5.7 to 16.9%)	5.9 (3.1 to 9.3)
Tracheal, bronchus, and lung cancer	Montenegro	2005	All Ages	0.0 (0.0 to 0.1)	10.2% (5.3 to 16.0%)	5.7 (3.0 to 9.1)
Tracheal, bronchus, and lung cancer	Montenegro	2010	All Ages	0.0 (0.0 to 0.1)	12.2% (6.7 to 18.6%)	6.4 (3.5 to 10.1)
Tracheal, bronchus, and lung cancer	Montenegro	2015	All Ages	0.1 (0.0 to 0.1)	13.3% (7.4 to 19.8%)	7.4 (4.1 to 11.3)
Tracheal, bronchus, and lung cancer	Montenegro	1990	Age-standardized		13.5% (7.6 to 20.3%)	5.7 (3.2 to 8.6)
Tracheal, bronchus, and lung cancer	Montenegro	1995	Age-standardized		11.5% (6.2 to 17.9%)	5.3 (2.8 to 8.2)
Tracheal, bronchus, and lung cancer	Montenegro	2000	Age-standardized		10.9% (5.7 to 16.9%)	5.2 (2.7 to 8.1)
Tracheal, bronchus, and lung cancer	Montenegro	2005	Age-standardized		10.2% (5.3 to 16.0%)	4.7 (2.4 to 7.5)
Tracheal, bronchus, and lung cancer	Montenegro	2010	Age-standardized		12.2% (6.7 to 18.5%)	5.0 (2.7 to 7.8)
Tracheal, bronchus, and lung cancer	Montenegro	2015	Age-standardized		13.3% (7.4 to 19.8%)	5.3 (3.0 to 8.2)
Cardiovascular diseases	Montenegro	1990	All Ages	0.3 (0.2 to 0.4)	11.7% (9.7 to 13.9%)	47.2 (38.7 to 56.1)
Cardiovascular diseases	Montenegro	1995	All Ages	0.3 (0.3 to 0.4)	10.9% (8.9 to 13.0%)	55.5 (44.6 to 65.9)
Cardiovascular diseases	Montenegro	2000	All Ages	0.4 (0.3 to 0.4)	10.9% (8.9 to 12.8%)	58.1 (47.5 to 68.8)
Cardiovascular diseases	Montenegro	2005	All Ages	0.3 (0.3 to 0.4)	10.4% (8.5 to 12.3%)	54.8 (44.9 to 65.2)
Cardiovascular diseases	Montenegro	2010	All Ages	0.3 (0.3 to 0.4)	11.1% (9.1 to 13.1%)	54.6 (44.8 to 64.4)
Cardiovascular diseases	Montenegro	2015	All Ages	0.4 (0.3 to 0.4)	11.3% (9.3 to 13.4%)	59.2 (48.1 to 70.0)
Cardiovascular diseases	Montenegro	1990	Age-standardized		11.7% (9.6 to 13.8%)	51.9 (42.2 to 61.6)
Cardiovascular diseases	Montenegro	1995	Age-standardized		10.8% (8.7 to 12.8%)	56.6 (45.6 to 67.4)
Cardiovascular diseases	Montenegro	2000	Age-standardized		10.4% (8.4 to 12.6%)	56.8 (45.4 to 68.6)
Cardiovascular diseases	Montenegro	2005	Age-standardized		10.2% (8.2 to 12.2%)	47.7 (38.5 to 57.6)
Cardiovascular diseases	Montenegro	2010	Age-standardized		10.9% (8.9 to 13.1%)	44.0 (35.6 to 52.4)
Cardiovascular diseases	Montenegro	2015	Age-standardized		11.3% (9.2 to 13.4%)	43.7 (35.7 to 51.9)
Ischemic heart disease	Montenegro	1990	All Ages	0.1 (0.1 to 0.2)	15.6% (12.2 to 19.1%)	24.9 (19.5 to 30.2)
Ischemic heart disease	Montenegro	1995	All Ages	0.2 (0.1 to 0.2)	14.6% (11.3 to 17.7%)	29.2 (22.6 to 35.4)
Ischemic heart disease	Montenegro	2000	All Ages	0.2 (0.1 to 0.2)	14.5% (11.6 to 17.5%)	30.8 (24.4 to 37.6)
Ischemic heart disease	Montenegro	2005	All Ages	0.2 (0.1 to 0.2)	14.1% (11.2 to 17.0%)	29.7 (23.4 to 36.1)
Ischemic heart disease	Montenegro	2010	All Ages	0.2 (0.1 to 0.2)	14.9% (11.7 to 18.0%)	30.6 (24.1 to 37.1)
Ischemic heart disease	Montenegro	2015	All Ages	0.2 (0.2 to 0.3)	15.1% (11.7 to 18.4%)	33.4 (25.7 to 41.2)
Ischemic heart disease	Montenegro	1990	Age-standardized		15.5% (12.1 to 18.9%)	27.2 (21.3 to 33.0)
Ischemic heart disease	Montenegro	1995	Age-standardized		14.4% (11.1 to 17.6%)	29.5 (22.9 to 36.0)
Ischemic heart disease	Montenegro	2000	Age-standardized		14.0% (10.8 to 17.1%)	29.7 (22.8 to 36.7)
Ischemic heart disease	Montenegro	2005	Age-standardized		13.8% (10.7 to 16.8%)	25.7 (19.9 to 31.4)
Ischemic heart disease	Montenegro	2010	Age-standardized		14.8% (11.5 to 17.9%)	24.6 (19.1 to 30.1)

Cause name	Location	Year	Age group	Number (in thousands)	PAF	Rate (per 100,000)
Ischemic heart disease	Montenegro	2015	Age-standardized		15.1% (11.7 to 18.5%)	24.7 (19.0 to 30.7)
Ischemic stroke	Montenegro	1990	All Ages	0.0 (0.0 to 0.0)	10.6% (6.9 to 14.8%)	2.9 (1.8 to 4.3)
Ischemic stroke	Montenegro	1995	All Ages	0.0 (0.0 to 0.0)	9.8% (6.1 to 14.0%)	3.4 (2.0 to 5.0)
Ischemic stroke	Montenegro	2000	All Ages	0.0 (0.0 to 0.0)	9.7% (6.4 to 13.3%)	3.4 (2.2 to 4.9)
Ischemic stroke	Montenegro	2005	All Ages	0.0 (0.0 to 0.0)	9.4% (6.1 to 12.9%)	3.6 (2.4 to 5.1)
Ischemic stroke	Montenegro	2010	All Ages	0.0 (0.0 to 0.0)	10.2% (6.7 to 14.0%)	4.1 (2.7 to 5.9)
Ischemic stroke	Montenegro	2015	All Ages	0.0 (0.0 to 0.0)	10.5% (7.0 to 14.4%)	4.5 (2.9 to 6.6)
Ischemic stroke	Montenegro	1990	Age-standardized		10.6% (6.9 to 14.7%)	3.2 (2.0 to 4.8)
Ischemic stroke	Montenegro	1995	Age-standardized		9.7% (6.0 to 13.9%)	3.5 (2.1 to 5.3)
Ischemic stroke	Montenegro	2000	Age-standardized		9.4% (5.8 to 13.6%)	3.6 (2.1 to 5.5)
Ischemic stroke	Montenegro	2005	Age-standardized		9.2% (5.8 to 13.1%)	3.3 (2.1 to 4.9)
Ischemic stroke	Montenegro	2010	Age-standardized		10.1% (6.5 to 14.1%)	3.3 (2.1 to 4.9)
Ischemic stroke	Montenegro	2015	Age-standardized		10.5% (6.8 to 14.5%)	3.3 (2.1 to 4.8)
Hemorrhagic stroke	Montenegro	1990	All Ages	0.1 (0.1 to 0.2)	11.6% (8.6 to 14.9%)	19.5 (14.2 to 25.5)
Hemorrhagic stroke	Montenegro	1995	All Ages	0.1 (0.1 to 0.2)	10.7% (7.5 to 13.9%)	22.9 (16.3 to 30.3)
Hemorrhagic stroke	Montenegro	2000	All Ages	0.1 (0.1 to 0.2)	10.5% (7.5 to 13.5%)	23.9 (17.3 to 31.0)
Hemorrhagic stroke	Montenegro	2005	All Ages	0.1 (0.1 to 0.2)	10.0% (7.1 to 13.1%)	21.5 (15.2 to 28.2)
Hemorrhagic stroke	Montenegro	2010	All Ages	0.1 (0.1 to 0.2)	10.8% (7.7 to 14.0%)	20.0 (14.1 to 26.3)
Hemorrhagic stroke	Montenegro	2015	All Ages	0.1 (0.1 to 0.2)	11.1% (8.0 to 14.5%)	21.4 (15.1 to 28.3)
Hemorrhagic stroke	Montenegro	1990	Age-standardized		11.5% (8.5 to 14.8%)	21.5 (15.6 to 28.1)
Hemorrhagic stroke	Montenegro	1995	Age-standardized		10.5% (7.4 to 13.8%)	23.6 (16.7 to 31.4)
Hemorrhagic stroke	Montenegro	2000	Age-standardized		10.1% (7.0 to 13.4%)	23.5 (16.5 to 31.4)
Hemorrhagic stroke	Montenegro	2005	Age-standardized		9.8% (6.7 to 13.1%)	18.8 (12.9 to 25.2)
Hemorrhagic stroke	Montenegro	2010	Age-standardized		10.7% (7.6 to 14.1%)	16.1 (11.2 to 21.3)
Hemorrhagic stroke	Montenegro	2015	Age-standardized		11.2% (7.9 to 14.6%)	15.8 (11.1 to 20.9)
Chronic obstructive pulmonary disease	Montenegro	1990	All Ages	0.0 (0.0 to 0.0)	20.1% (9.9 to 31.1%)	1.2 (0.6 to 2.0)
Chronic obstructive pulmonary disease	Montenegro	1995	All Ages	0.0 (0.0 to 0.0)	17.9% (8.3 to 28.7%)	1.4 (0.6 to 2.3)
Chronic obstructive pulmonary disease	Montenegro	2000	All Ages	0.0 (0.0 to 0.0)	17.2% (8.1 to 28.0%)	1.4 (0.6 to 2.3)
Chronic obstructive pulmonary disease	Montenegro	2005	All Ages	0.0 (0.0 to 0.0)	16.4% (7.4 to 27.0%)	1.4 (0.6 to 2.2)
Chronic obstructive pulmonary disease	Montenegro	2010	All Ages	0.0 (0.0 to 0.0)	18.7% (8.9 to 29.5%)	1.5 (0.7 to 2.4)
Chronic obstructive pulmonary disease	Montenegro	2015	All Ages	0.0 (0.0 to 0.0)	19.8% (9.6 to 30.5%)	1.7 (0.8 to 2.8)
Chronic obstructive pulmonary disease	Montenegro	1990	Age-standardized		20.1% (9.9 to 31.1%)	1.4 (0.7 to 2.2)
Chronic obstructive pulmonary disease	Montenegro	1995	Age-standardized		17.9% (8.3 to 28.7%)	1.4 (0.6 to 2.4)
Chronic obstructive pulmonary disease	Montenegro	2000	Age-standardized		17.2% (8.1 to 28.0%)	1.5 (0.7 to 2.4)
Chronic obstructive pulmonary disease	Montenegro	2005	Age-standardized		16.4% (7.3 to 27.0%)	1.2 (0.5 to 2.0)
Chronic obstructive pulmonary disease	Montenegro	2010	Age-standardized		18.7% (8.9 to 29.5%)	1.2 (0.6 to 2.0)

Cause name	Location	Year	Age group	Number (in thousands)	PAF	Rate (per 100,000)
Chronic obstructive pulmonary disease	Montenegro	2015	Age-standardized		19.8% (9.6 to 30.5%)	1.3 (0.6 to 2.0)
All causes	Poland	1990	All Ages	41.9 (36.2 to 48.5)	10.7% (9.3 to 12.4%)	109.7 (94.8 to 126.9)
All causes	Poland	1995	All Ages	36.2 (30.8 to 42.1)	9.3% (7.9 to 10.8%)	94.1 (80.0 to 109.5)
All causes	Poland	2000	All Ages	29.8 (25.3 to 34.5)	8.1% (6.8 to 9.4%)	77.8 (65.9 to 90.0)
All causes	Poland	2005	All Ages	28.1 (23.7 to 32.7)	7.6% (6.4 to 8.9%)	73.6 (62.1 to 85.6)
All causes	Poland	2010	All Ages	29.4 (24.7 to 34.5)	7.7% (6.5 to 9.1%)	76.9 (64.6 to 90.2)
All causes	Poland	2015	All Ages	27.9 (22.9 to 33.3)	7.2% (6.0 to 8.6%)	71.6 (58.8 to 85.7)
All causes	Poland	1990	Age-standardized		10.6% (9.1 to 12.3%)	110.0 (94.5 to 127.6)
All causes	Poland	1995	Age-standardized		9.2% (7.8 to 10.8%)	90.2 (76.4 to 105.5)
All causes	Poland	2000	Age-standardized		8.0% (6.7 to 9.3%)	70.4 (59.0 to 81.9)
All causes	Poland	2005	Age-standardized		7.5% (6.3 to 8.7%)	58.2 (49.0 to 67.9)
All causes	Poland	2010	Age-standardized		7.6% (6.4 to 8.8%)	53.7 (45.4 to 63.0)
All causes	Poland	2015	Age-standardized		7.0% (5.8 to 8.2%)	44.9 (37.4 to 53.1)
Lower respiratory infections	Poland	1990	All Ages	2.5 (1.8 to 3.1)	29.5% (21.9 to 37.1%)	6.5 (4.7 to 8.2)
Lower respiratory infections	Poland	1995	All Ages	2.0 (1.3 to 2.7)	24.1% (15.9 to 33.1%)	5.2 (3.4 to 7.0)
Lower respiratory infections	Poland	2000	All Ages	1.8 (1.1 to 2.6)	20.6% (12.2 to 30.4%)	4.8 (2.8 to 6.9)
Lower respiratory infections	Poland	2005	All Ages	2.0 (1.2 to 3.0)	19.9% (11.5 to 29.7%)	5.2 (3.0 to 7.8)
Lower respiratory infections	Poland	2010	All Ages	2.4 (1.5 to 3.5)	21.3% (12.8 to 31.0%)	6.3 (3.8 to 9.2)
Lower respiratory infections	Poland	2015	All Ages	2.3 (1.3 to 3.5)	19.4% (11.0 to 29.3%)	5.9 (3.3 to 9.0)
Lower respiratory infections	Poland	1990	Age-standardized		29.5% (21.9 to 37.1%)	6.7 (4.9 to 8.5)
Lower respiratory infections	Poland	1995	Age-standardized		24.1% (15.9 to 33.1%)	5.2 (3.4 to 7.0)
Lower respiratory infections	Poland	2000	Age-standardized		20.6% (12.2 to 30.4%)	4.6 (2.7 to 6.7)
Lower respiratory infections	Poland	2005	Age-standardized		19.9% (11.5 to 29.7%)	4.2 (2.4 to 6.4)
Lower respiratory infections	Poland	2010	Age-standardized		21.3% (12.8 to 31.0%)	4.4 (2.7 to 6.5)
Lower respiratory infections	Poland	2015	Age-standardized		19.4% (11.0 to 29.3%)	3.7 (2.1 to 5.6)
Tracheal, bronchus, and lung cancer	Poland	1990	All Ages	3.8 (2.4 to 5.3)	21.6% (13.9 to 29.7%)	9.9 (6.2 to 13.7)
Tracheal, bronchus, and lung cancer	Poland	1995	All Ages	3.4 (2.0 to 4.9)	17.3% (10.3 to 24.7%)	8.8 (5.2 to 12.8)
Tracheal, bronchus, and lung cancer	Poland	2000	All Ages	3.2 (1.8 to 4.7)	14.6% (8.3 to 21.5%)	8.3 (4.8 to 12.3)
Tracheal, bronchus, and lung cancer	Poland	2005	All Ages	3.3 (1.9 to 4.9)	14.1% (8.0 to 20.9%)	8.6 (4.9 to 12.8)
Tracheal, bronchus, and lung cancer	Poland	2010	All Ages	3.6 (2.1 to 5.4)	15.1% (8.8 to 22.1%)	9.5 (5.5 to 14.0)
Tracheal, bronchus, and lung cancer	Poland	2015	All Ages	3.3 (1.8 to 4.9)	13.7% (7.7 to 20.5%)	8.5 (4.7 to 12.6)
Tracheal, bronchus, and lung cancer	Poland	1990	Age-standardized		21.6% (13.8 to 29.7%)	9.5 (6.0 to 13.2)
Tracheal, bronchus, and lung cancer	Poland	1995	Age-standardized		17.3% (10.3 to 24.7%)	8.0 (4.8 to 11.6)
Tracheal, bronchus, and lung cancer	Poland	2000	Age-standardized		14.6% (8.3 to 21.5%)	7.1 (4.1 to 10.5)
Tracheal, bronchus, and lung cancer	Poland	2005	Age-standardized		14.1% (8.0 to 20.9%)	6.7 (3.9 to 10.1)
Tracheal, bronchus, and lung cancer	Poland	2010	Age-standardized		15.1% (8.8 to 22.1%)	6.8 (4.0 to 10.1)

Cause name	Location	Year	Age group	Number (in thousands)	PAF	Rate (per 100,000)
Tracheal, bronchus, and lung cancer	Poland	2015	Age-standardized		13.7% (7.7 to 20.5%)	5.6 (3.2 to 8.5)
Cardiovascular diseases	Poland	1990	All Ages	32.6 (27.5 to 38.6)	15.4% (13.0 to 18.2%)	85.3 (71.9 to 101.0)
Cardiovascular diseases	Poland	1995	All Ages	28.5 (23.5 to 33.8)	13.8% (11.4 to 16.3%)	74.1 (61.2 to 87.8)
Cardiovascular diseases	Poland	2000	All Ages	22.8 (18.9 to 27.1)	12.4% (10.3 to 14.7%)	59.4 (49.3 to 70.7)
Cardiovascular diseases	Poland	2005	All Ages	20.8 (17.1 to 24.7)	11.9% (9.7 to 14.1%)	54.3 (44.7 to 64.7)
Cardiovascular diseases	Poland	2010	All Ages	21.0 (16.9 to 25.3)	11.9% (9.5 to 14.3%)	54.8 (44.0 to 66.1)
Cardiovascular diseases	Poland	2015	All Ages	20.0 (15.6 to 24.5)	11.1% (8.7 to 13.6%)	51.3 (40.0 to 63.0)
Cardiovascular diseases	Poland	1990	Age-standardized		15.2% (12.7 to 18.1%)	85.7 (71.7 to 102.3)
Cardiovascular diseases	Poland	1995	Age-standardized		13.6% (11.2 to 16.2%)	71.2 (58.5 to 85.1)
Cardiovascular diseases	Poland	2000	Age-standardized		12.2% (9.9 to 14.5%)	54.0 (44.0 to 64.7)
Cardiovascular diseases	Poland	2005	Age-standardized		11.8% (9.6 to 14.1%)	43.0 (35.0 to 51.3)
Cardiovascular diseases	Poland	2010	Age-standardized		12.0% (9.8 to 14.4%)	38.1 (30.8 to 45.8)
Cardiovascular diseases	Poland	2015	Age-standardized		11.4% (9.2 to 13.8%)	31.9 (25.4 to 38.5)
Ischemic heart disease	Poland	1990	All Ages	25.6 (20.7 to 31.4)	18.6% (15.0 to 22.8%)	67.0 (54.2 to 82.1)
Ischemic heart disease	Poland	1995	All Ages	22.1 (17.3 to 26.9)	16.8% (13.3 to 20.6%)	57.3 (44.9 to 69.8)
Ischemic heart disease	Poland	2000	All Ages	16.7 (13.0 to 20.4)	15.6% (12.2 to 19.1%)	43.5 (34.0 to 53.1)
Ischemic heart disease	Poland	2005	All Ages	15.1 (11.6 to 18.8)	15.0% (11.6 to 18.7%)	39.6 (30.4 to 49.2)
Ischemic heart disease	Poland	2010	All Ages	15.5 (11.6 to 19.6)	15.1% (11.3 to 19.0%)	40.6 (30.4 to 51.3)
Ischemic heart disease	Poland	2015	All Ages	15.1 (10.8 to 19.3)	14.3% (10.3 to 18.3%)	38.7 (27.8 to 49.7)
Ischemic heart disease	Poland	1990	Age-standardized		18.4% (14.7 to 22.8%)	67.3 (53.7 to 82.9)
Ischemic heart disease	Poland	1995	Age-standardized		16.6% (12.9 to 20.4%)	55.0 (42.9 to 67.5)
Ischemic heart disease	Poland	2000	Age-standardized		15.3% (11.6 to 19.0%)	39.6 (30.1 to 49.2)
Ischemic heart disease	Poland	2005	Age-standardized		15.0% (11.4 to 18.7%)	31.4 (23.9 to 39.0)
Ischemic heart disease	Poland	2010	Age-standardized		15.4% (11.6 to 19.2%)	28.2 (21.4 to 35.3)
Ischemic heart disease	Poland	2015	Age-standardized		14.7% (10.9 to 18.5%)	24.0 (17.8 to 30.4)
Ischemic stroke	Poland	1990	All Ages	4.3 (3.3 to 5.5)	14.3% (10.7 to 17.9%)	11.3 (8.5 to 14.3)
Ischemic stroke	Poland	1995	All Ages	3.9 (2.8 to 5.1)	12.5% (8.9 to 16.1%)	10.2 (7.3 to 13.2)
Ischemic stroke	Poland	2000	All Ages	3.6 (2.6 to 4.7)	11.4% (8.0 to 14.8%)	9.5 (6.6 to 12.4)
Ischemic stroke	Poland	2005	All Ages	3.4 (2.4 to 4.5)	11.0% (7.6 to 14.7%)	8.9 (6.2 to 11.8)
Ischemic stroke	Poland	2010	All Ages	3.4 (2.3 to 4.6)	11.3% (7.7 to 15.3%)	8.9 (6.1 to 12.0)
Ischemic stroke	Poland	2015	All Ages	3.1 (2.0 to 4.5)	10.5% (6.7 to 14.9%)	8.1 (5.2 to 11.4)
Ischemic stroke	Poland	1990	Age-standardized		14.1% (10.5 to 18.0%)	11.5 (8.6 to 14.6)
Ischemic stroke	Poland	1995	Age-standardized		12.3% (8.7 to 16.1%)	10.0 (7.1 to 13.1)
Ischemic stroke	Poland	2000	Age-standardized		11.2% (7.7 to 15.0%)	8.7 (6.0 to 11.7)
Ischemic stroke	Poland	2005	Age-standardized		11.0% (7.5 to 14.9%)	7.0 (4.8 to 9.4)
Ischemic stroke	Poland	2010	Age-standardized		11.4% (7.9 to 15.3%)	6.1 (4.2 to 8.2)

Cause name	Location	Year	Age group	Number<br>(in thousands)	PAF	Rate<br>(per 100,000)
Ischemic stroke	Poland	2015	Age-standardized		10.8%<br>(7.2 to 14.8%)	4.9<br>(3.3 to 6.7)
Hemorrhagic stroke	Poland	1990	All Ages	2.7<br>(2.2 to 3.1)	17.0%<br>(14.1 to 19.8%)	7.0<br>(5.8 to 8.1)
Hemorrhagic stroke	Poland	1995	All Ages	2.5<br>(2.0 to 3.0)	14.9%<br>(12.0 to 17.8%)	6.5<br>(5.2 to 7.8)
Hemorrhagic stroke	Poland	2000	All Ages	2.5<br>(1.9 to 3.0)	13.4%<br>(10.5 to 16.2%)	6.4<br>(5.0 to 7.9)
Hemorrhagic stroke	Poland	2005	All Ages	2.2<br>(1.7 to 2.7)	13.0%<br>(10.2 to 15.9%)	5.8<br>(4.6 to 7.1)
Hemorrhagic stroke	Poland	2010	All Ages	2.0<br>(1.6 to 2.5)	13.2%<br>(10.4 to 16.4%)	5.2<br>(4.1 to 6.5)
Hemorrhagic stroke	Poland	2015	All Ages	1.8<br>(1.3 to 2.2)	12.3%<br>(9.3 to 15.4%)	4.5<br>(3.4 to 5.7)
Hemorrhagic stroke	Poland	1990	Age-standardized		16.8%<br>(13.9 to 19.6%)	6.9<br>(5.7 to 8.0)
Hemorrhagic stroke	Poland	1995	Age-standardized		14.7%<br>(11.9 to 17.5%)	6.1<br>(4.9 to 7.4)
Hemorrhagic stroke	Poland	2000	Age-standardized		13.2%<br>(10.3 to 16.1%)	5.6<br>(4.4 to 6.9)
Hemorrhagic stroke	Poland	2005	Age-standardized		13.0%<br>(10.2 to 15.9%)	4.6<br>(3.6 to 5.7)
Hemorrhagic stroke	Poland	2010	Age-standardized		13.5%<br>(10.7 to 16.4%)	3.8<br>(3.0 to 4.6)
Hemorrhagic stroke	Poland	2015	Age-standardized		12.7%<br>(9.9 to 15.6%)	3.0<br>(2.3 to 3.7)
Chronic obstructive pulmonary disease	Poland	1990	All Ages	3.1<br>(1.9 to 4.4)	28.0%<br>(16.9 to 39.8%)	8.0<br>(4.9 to 11.5)
Chronic obstructive pulmonary disease	Poland	1995	All Ages	2.4<br>(1.3 to 3.5)	23.9%<br>(13.0 to 35.2%)	6.1<br>(3.3 to 9.0)
Chronic obstructive pulmonary disease	Poland	2000	All Ages	2.0<br>(1.0 to 3.1)	21.2%<br>(10.7 to 32.2%)	5.3<br>(2.7 to 8.1)
Chronic obstructive pulmonary disease	Poland	2005	All Ages	2.1<br>(1.0 to 3.3)	20.6%<br>(10.3 to 31.7%)	5.5<br>(2.7 to 8.5)
Chronic obstructive pulmonary disease	Poland	2010	All Ages	2.4<br>(1.2 to 3.7)	21.7%<br>(11.2 to 32.8%)	6.3<br>(3.2 to 9.6)
Chronic obstructive pulmonary disease	Poland	2015	All Ages	2.3<br>(1.1 to 3.6)	20.3%<br>(10.0 to 31.1%)	5.9<br>(2.9 to 9.1)
Chronic obstructive pulmonary disease	Poland	1990	Age-standardized		28.0%<br>(16.9 to 39.8%)	8.1<br>(4.9 to 11.5)
Chronic obstructive pulmonary disease	Poland	1995	Age-standardized		23.9%<br>(13.0 to 35.2%)	5.8<br>(3.2 to 8.5)
Chronic obstructive pulmonary disease	Poland	2000	Age-standardized		21.2%<br>(10.7 to 32.2%)	4.7<br>(2.4 to 7.2)
Chronic obstructive pulmonary disease	Poland	2005	Age-standardized		20.6%<br>(10.2 to 31.6%)	4.3<br>(2.1 to 6.6)
Chronic obstructive pulmonary disease	Poland	2010	Age-standardized		21.7%<br>(11.2 to 32.8%)	4.3<br>(2.2 to 6.6)
Chronic obstructive pulmonary disease	Poland	2015	Age-standardized		20.3%<br>(10.0 to 31.1%)	3.7<br>(1.8 to 5.7)
All causes	Romania	1990	All Ages	24.3<br>(21.1 to 27.6)	10.0%<br>(8.7 to 11.3%)	103.6<br>(90.0 to 117.9)
All causes	Romania	1995	All Ages	24.3<br>(20.7 to 27.9)	8.9%<br>(7.6 to 10.2%)	105.6<br>(89.9 to 121.2)
All causes	Romania	2000	All Ages	20.7<br>(17.7 to 23.8)	8.2%<br>(7.0 to 9.4%)	93.6<br>(79.9 to 107.7)
All causes	Romania	2005	All Ages	18.4<br>(15.4 to 21.6)	7.2%<br>(6.0 to 8.4%)	85.9<br>(71.7 to 100.6)
All causes	Romania	2010	All Ages	19.1<br>(15.9 to 22.4)	7.4%<br>(6.2 to 8.6%)	94.4<br>(78.4 to 110.2)
All causes	Romania	2015	All Ages	18.1<br>(14.8 to 21.5)	7.2%<br>(5.9 to 8.5%)	92.9<br>(75.6 to 110.2)
All causes	Romania	1990	Age-standardized		9.9%<br>(8.5 to 11.3%)	102.8<br>(88.4 to 117.6)
All causes	Romania	1995	Age-standardized		8.8%<br>(7.5 to 10.1%)	97.3<br>(82.9 to 112.3)
All causes	Romania	2000	Age-standardized		8.0%<br>(6.7 to 9.3%)	81.1<br>(68.2 to 94.4)
All causes	Romania	2005	Age-standardized		7.0%<br>(5.8 to 8.2%)	64.7<br>(53.8 to 76.4)
All causes	Romania	2010	Age-standardized		7.2%<br>(6.1 to 8.4%)	62.0<br>(51.8 to 72.2)

Cause name	Location	Year	Age group	Number (in thousands)	PAF	Rate (per 100,000)
All causes	Romania	2015	Age-standardized		7.1% (5.8 to 8.3%)	54.9 (45.2 to 64.5)
Lower respiratory infections	Romania	1990	All Ages	2.3 (1.5 to 3.1)	24.2% (15.9 to 33.0%)	9.6 (6.3 to 13.3)
Lower respiratory infections	Romania	1995	All Ages	1.6 (0.9 to 2.4)	19.6% (11.0 to 29.3%)	6.9 (3.9 to 10.3)
Lower respiratory infections	Romania	2000	All Ages	1.3 (0.6 to 2.0)	17.5% (8.9 to 27.7%)	5.7 (2.9 to 9.1)
Lower respiratory infections	Romania	2005	All Ages	1.0 (0.4 to 1.6)	14.8% (6.4 to 25.4%)	4.5 (1.9 to 7.6)
Lower respiratory infections	Romania	2010	All Ages	1.0 (0.5 to 1.6)	16.9% (8.4 to 27.1%)	4.8 (2.4 to 7.7)
Lower respiratory infections	Romania	2015	All Ages	0.8 (0.4 to 1.4)	16.4% (7.9 to 26.7%)	4.4 (2.1 to 7.1)
Lower respiratory infections	Romania	1990	Age-standardized		24.2% (15.9 to 33.0%)	10.8 (7.1 to 14.8)
Lower respiratory infections	Romania	1995	Age-standardized		19.6% (11.0 to 29.3%)	7.7 (4.3 to 11.6)
Lower respiratory infections	Romania	2000	Age-standardized		17.5% (8.9 to 27.7%)	6.3 (3.2 to 10.0)
Lower respiratory infections	Romania	2005	Age-standardized		14.8% (6.4 to 25.4%)	4.4 (1.8 to 7.5)
Lower respiratory infections	Romania	2010	Age-standardized		16.9% (8.4 to 27.1%)	4.0 (2.0 to 6.5)
Lower respiratory infections	Romania	2015	Age-standardized		16.4% (7.9 to 26.7%)	3.2 (1.6 to 5.3)
Tracheal, bronchus, and lung cancer	Romania	1990	All Ages	1.1 (0.7 to 1.6)	17.3% (10.4 to 24.6%)	4.9 (2.9 to 7.0)
Tracheal, bronchus, and lung cancer	Romania	1995	All Ages	1.2 (0.6 to 1.7)	13.8% (7.7 to 20.6%)	5.0 (2.8 to 7.4)
Tracheal, bronchus, and lung cancer	Romania	2000	All Ages	1.0 (0.6 to 1.5)	12.2% (6.7 to 18.7%)	4.5 (2.5 to 6.9)
Tracheal, bronchus, and lung cancer	Romania	2005	All Ages	0.9 (0.5 to 1.4)	10.3% (5.4 to 16.2%)	4.2 (2.2 to 6.5)
Tracheal, bronchus, and lung cancer	Romania	2010	All Ages	1.1 (0.6 to 1.7)	11.8% (6.4 to 18.1%)	5.4 (2.9 to 8.3)
Tracheal, bronchus, and lung cancer	Romania	2015	All Ages	1.0 (0.5 to 1.5)	11.5% (6.2 to 17.7%)	5.2 (2.8 to 7.9)
Tracheal, bronchus, and lung cancer	Romania	1990	Age-standardized		17.3% (10.4 to 24.6%)	4.3 (2.6 to 6.2)
Tracheal, bronchus, and lung cancer	Romania	1995	Age-standardized		13.8% (7.7 to 20.6%)	4.2 (2.3 to 6.2)
Tracheal, bronchus, and lung cancer	Romania	2000	Age-standardized		12.2% (6.7 to 18.7%)	3.5 (1.9 to 5.4)
Tracheal, bronchus, and lung cancer	Romania	2005	Age-standardized		10.3% (5.4 to 16.2%)	3.1 (1.6 to 4.8)
Tracheal, bronchus, and lung cancer	Romania	2010	Age-standardized		11.8% (6.4 to 18.1%)	3.7 (2.0 to 5.7)
Tracheal, bronchus, and lung cancer	Romania	2015	Age-standardized		11.5% (6.2 to 17.7%)	3.3 (1.8 to 5.0)
Cardiovascular diseases	Romania	1990	All Ages	18.1 (15.4 to 20.9)	13.3% (11.3 to 15.4%)	77.3 (65.6 to 89.4)
Cardiovascular diseases	Romania	1995	All Ages	19.7 (16.5 to 22.9)	12.3% (10.3 to 14.4%)	85.4 (71.5 to 99.5)
Cardiovascular diseases	Romania	2000	All Ages	17.0 (14.2 to 19.9)	11.4% (9.5 to 13.3%)	76.9 (64.2 to 89.7)
Cardiovascular diseases	Romania	2005	All Ages	15.4 (12.5 to 18.1)	10.1% (8.2 to 11.9%)	71.8 (58.1 to 84.6)
Cardiovascular diseases	Romania	2010	All Ages	15.8 (12.7 to 18.8)	10.3% (8.3 to 12.3%)	77.9 (62.4 to 92.8)
Cardiovascular diseases	Romania	2015	All Ages	15.1 (11.9 to 18.4)	10.0% (8.0 to 12.2%)	77.1 (60.9 to 94.3)
Cardiovascular diseases	Romania	1990	Age-standardized		13.0% (10.9 to 15.3%)	76.0 (63.8 to 89.2)
Cardiovascular diseases	Romania	1995	Age-standardized		12.1% (10.0 to 14.1%)	77.8 (64.6 to 91.3)
Cardiovascular diseases	Romania	2000	Age-standardized		11.1% (9.0 to 13.2%)	65.6 (53.2 to 77.9)
Cardiovascular diseases	Romania	2005	Age-standardized		10.1% (8.1 to 11.9%)	53.2 (42.7 to 63.1)
Cardiovascular diseases	Romania	2010	Age-standardized		10.5% (8.5 to 12.5%)	50.3 (40.6 to 59.8)

Cause name	Location	Year	Age group	Number (in thousands)	PAF	Rate (per 100,000)
Cardiovascular diseases	Romania	2015	Age-standardized		10.3% (8.3 to 12.3%)	44.8 (35.9 to 53.9)
Ischemic heart disease	Romania	1990	All Ages	11.8 (9.5 to 14.4)	16.9% (13.5 to 20.6%)	50.5 (40.6 to 61.4)
Ischemic heart disease	Romania	1995	All Ages	12.7 (10.0 to 15.4)	15.6% (12.3 to 18.9%)	55.0 (43.4 to 66.8)
Ischemic heart disease	Romania	2000	All Ages	10.7 (8.4 to 13.0)	14.8% (11.6 to 17.9%)	48.3 (37.9 to 58.7)
Ischemic heart disease	Romania	2005	All Ages	9.6 (7.3 to 11.8)	13.5% (10.3 to 16.5%)	44.7 (33.9 to 55.1)
Ischemic heart disease	Romania	2010	All Ages	9.9 (7.5 to 12.4)	13.9% (10.5 to 17.4%)	48.7 (36.8 to 61.0)
Ischemic heart disease	Romania	2015	All Ages	9.6 (6.9 to 12.3)	13.5% (9.8 to 17.1%)	49.0 (35.3 to 62.9)
Ischemic heart disease	Romania	1990	Age-standardized		16.5% (12.9 to 20.3%)	49.8 (38.9 to 60.9)
Ischemic heart disease	Romania	1995	Age-standardized		15.3% (11.9 to 18.8%)	50.3 (39.3 to 61.8)
Ischemic heart disease	Romania	2000	Age-standardized		14.3% (10.9 to 17.8%)	41.6 (31.7 to 51.5)
Ischemic heart disease	Romania	2005	Age-standardized		13.4% (10.2 to 16.6%)	33.4 (25.2 to 41.6)
Ischemic heart disease	Romania	2010	Age-standardized		14.2% (10.8 to 17.5%)	31.6 (24.0 to 39.2)
Ischemic heart disease	Romania	2015	Age-standardized		13.8% (10.4 to 17.3%)	28.6 (21.3 to 36.2)
Ischemic stroke	Romania	1990	All Ages	3.3 (2.5 to 4.2)	12.8% (9.4 to 16.3%)	14.2 (10.5 to 18.0)
Ischemic stroke	Romania	1995	All Ages	3.6 (2.5 to 4.6)	11.4% (8.1 to 14.7%)	15.4 (11.0 to 19.9)
Ischemic stroke	Romania	2000	All Ages	3.2 (2.2 to 4.1)	10.6% (7.4 to 13.9%)	14.3 (10.0 to 18.6)
Ischemic stroke	Romania	2005	All Ages	3.0 (2.0 to 4.0)	9.5% (6.3 to 12.9%)	13.8 (9.1 to 18.6)
Ischemic stroke	Romania	2010	All Ages	3.1 (2.1 to 4.3)	10.0% (6.7 to 13.6%)	15.3 (10.1 to 20.9)
Ischemic stroke	Romania	2015	All Ages	2.9 (1.9 to 4.1)	9.7% (6.2 to 13.6%)	14.9 (9.5 to 21.2)
Ischemic stroke	Romania	1990	Age-standardized		12.5% (9.1 to 16.0%)	14.2 (10.2 to 18.2)
Ischemic stroke	Romania	1995	Age-standardized		11.1% (7.8 to 14.5%)	14.3 (10.1 to 18.7)
Ischemic stroke	Romania	2000	Age-standardized		10.3% (7.0 to 13.8%)	12.3 (8.5 to 16.5)
Ischemic stroke	Romania	2005	Age-standardized		9.4% (6.2 to 12.9%)	10.1 (6.6 to 13.9)
Ischemic stroke	Romania	2010	Age-standardized		10.1% (6.7 to 13.8%)	9.6 (6.4 to 13.2)
Ischemic stroke	Romania	2015	Age-standardized		9.8% (6.4 to 13.7%)	8.3 (5.5 to 11.6)
Hemorrhagic stroke	Romania	1990	All Ages	3.0 (2.4 to 3.6)	14.5% (11.6 to 17.5%)	12.7 (10.0 to 15.3)
Hemorrhagic stroke	Romania	1995	All Ages	3.5 (2.7 to 4.3)	12.9% (9.9 to 16.0%)	15.1 (11.7 to 18.7)
Hemorrhagic stroke	Romania	2000	All Ages	3.1 (2.4 to 4.0)	11.8% (8.9 to 14.8%)	14.2 (10.6 to 17.9)
Hemorrhagic stroke	Romania	2005	All Ages	2.8 (2.1 to 3.6)	10.5% (7.7 to 13.5%)	13.3 (9.6 to 17.0)
Hemorrhagic stroke	Romania	2010	All Ages	2.8 (2.0 to 3.6)	11.0% (8.0 to 14.0%)	13.9 (10.0 to 17.6)
Hemorrhagic stroke	Romania	2015	All Ages	2.6 (1.8 to 3.4)	10.6% (7.6 to 13.8%)	13.2 (9.3 to 17.2)
Hemorrhagic stroke	Romania	1990	Age-standardized		14.2% (11.3 to 17.2%)	12.0 (9.5 to 14.6)
Hemorrhagic stroke	Romania	1995	Age-standardized		12.7% (9.9 to 15.6%)	13.2 (10.2 to 16.3)
Hemorrhagic stroke	Romania	2000	Age-standardized		11.6% (8.8 to 14.6%)	11.7 (8.8 to 14.6)
Hemorrhagic stroke	Romania	2005	Age-standardized		10.6% (7.7 to 13.5%)	9.7 (7.1 to 12.4)
Hemorrhagic stroke	Romania	2010	Age-standardized		11.2% (8.3 to 14.1%)	9.1 (6.7 to 11.5)

Cause name	Location	Year	Age group	Number (in thousands)	PAF	Rate (per 100,000)
Hemorrhagic stroke	Romania	2015	Age-standardized		10.9% (8.0 to 13.9%)	7.9 (5.8 to 10.0)
Chronic obstructive pulmonary disease	Romania	1990	All Ages	2.7 (1.5 to 4.0)	23.9% (13.1 to 35.1%)	11.7 (6.5 to 17.2)
Chronic obstructive pulmonary disease	Romania	1995	All Ages	1.9 (1.0 to 2.9)	20.4% (10.1 to 31.3%)	8.3 (4.2 to 12.8)
Chronic obstructive pulmonary disease	Romania	2000	All Ages	1.4 (0.7 to 2.3)	18.7% (8.9 to 29.6%)	6.5 (3.1 to 10.2)
Chronic obstructive pulmonary disease	Romania	2005	All Ages	1.2 (0.5 to 1.9)	16.5% (7.4 to 27.2%)	5.5 (2.5 to 8.9)
Chronic obstructive pulmonary disease	Romania	2010	All Ages	1.3 (0.6 to 2.0)	18.2% (8.6 to 29.1%)	6.2 (3.0 to 9.7)
Chronic obstructive pulmonary disease	Romania	2015	All Ages	1.2 (0.6 to 1.9)	17.9% (8.4 to 28.7%)	6.2 (3.0 to 9.9)
Chronic obstructive pulmonary disease	Romania	1990	Age-standardized		23.9% (13.1 to 35.1%)	11.7 (6.4 to 17.1)
Chronic obstructive pulmonary disease	Romania	1995	Age-standardized		20.4% (10.1 to 31.3%)	7.7 (3.9 to 11.7)
Chronic obstructive pulmonary disease	Romania	2000	Age-standardized		18.7% (8.9 to 29.6%)	5.6 (2.7 to 8.8)
Chronic obstructive pulmonary disease	Romania	2005	Age-standardized		16.5% (7.4 to 27.2%)	4.0 (1.8 to 6.5)
Chronic obstructive pulmonary disease	Romania	2010	Age-standardized		18.2% (8.6 to 29.1%)	4.0 (1.9 to 6.2)
Chronic obstructive pulmonary disease	Romania	2015	Age-standardized		17.8% (8.4 to 28.7%)	3.6 (1.7 to 5.6)
All causes	Serbia	1990	All Ages	6.8 (5.8 to 7.9)	8.2% (7.0 to 9.4%)	71.7 (60.9 to 83.2)
All causes	Serbia	1995	All Ages	7.8 (6.6 to 9.0)	7.8% (6.5 to 9.0%)	79.0 (66.6 to 91.4)
All causes	Serbia	2000	All Ages	8.4 (7.2 to 9.7)	7.7% (6.5 to 8.9%)	88.8 (75.5 to 102.8)
All causes	Serbia	2005	All Ages	7.7 (6.4 to 9.0)	6.9% (5.8 to 8.1%)	83.1 (69.6 to 97.9)
All causes	Serbia	2010	All Ages	7.6 (6.3 to 9.0)	7.1% (5.9 to 8.4%)	83.9 (69.9 to 99.0)
All causes	Serbia	2015	All Ages	7.2 (5.9 to 8.5)	7.1% (5.9 to 8.4%)	80.9 (66.6 to 96.0)
All causes	Serbia	1990	Age-standardized		8.1% (6.8 to 9.4%)	74.2 (62.2 to 86.9)
All causes	Serbia	1995	Age-standardized		7.6% (6.3 to 8.9%)	77.6 (64.0 to 90.9)
All causes	Serbia	2000	Age-standardized		7.5% (6.2 to 8.8%)	76.9 (64.1 to 90.2)
All causes	Serbia	2005	Age-standardized		6.7% (5.6 to 8.0%)	62.7 (51.9 to 74.4)
All causes	Serbia	2010	Age-standardized		6.9% (5.8 to 8.2%)	56.7 (47.3 to 67.0)
All causes	Serbia	2015	Age-standardized		6.9% (5.7 to 8.2%)	50.1 (41.5 to 59.3)
Lower respiratory infections	Serbia	1990	All Ages	0.3 (0.1 to 0.4)	20.0% (11.7 to 29.7%)	2.6 (1.5 to 4.0)
Lower respiratory infections	Serbia	1995	All Ages	0.2 (0.1 to 0.3)	16.7% (8.3 to 27.0%)	2.1 (1.0 to 3.4)
Lower respiratory infections	Serbia	2000	All Ages	0.2 (0.1 to 0.3)	16.4% (8.0 to 26.8%)	1.9 (0.9 to 3.3)
Lower respiratory infections	Serbia	2005	All Ages	0.2 (0.1 to 0.3)	14.6% (6.3 to 25.2%)	1.7 (0.7 to 3.1)
Lower respiratory infections	Serbia	2010	All Ages	0.2 (0.1 to 0.3)	16.7% (8.3 to 26.9%)	2.1 (1.0 to 3.5)
Lower respiratory infections	Serbia	2015	All Ages	0.2 (0.1 to 0.3)	17.6% (9.1 to 27.8%)	2.3 (1.2 to 3.7)
Lower respiratory infections	Serbia	1990	Age-standardized		20.0% (11.7 to 29.7%)	2.9 (1.6 to 4.4)
Lower respiratory infections	Serbia	1995	Age-standardized		16.7% (8.3 to 27.0%)	2.2 (1.1 to 3.7)
Lower respiratory infections	Serbia	2000	Age-standardized		16.4% (8.0 to 26.8%)	1.9 (0.9 to 3.1)
Lower respiratory infections	Serbia	2005	Age-standardized		14.6% (6.3 to 25.2%)	1.4 (0.6 to 2.5)
Lower respiratory infections	Serbia	2010	Age-standardized		16.7% (8.3 to 26.9%)	1.5 (0.7 to 2.5)

Cause name	Location	Year	Age group	Number (in thousands)	PAF	Rate (per 100,000)
Lower respiratory infections	Serbia	2015	Age-standardized		17.6% (9.1 to 27.8%)	1.5 (0.8 to 2.5)
Tracheal, bronchus, and lung cancer	Serbia	1990	All Ages	0.5 (0.3 to 0.8)	14.1% (8.0 to 21.0%)	5.5 (3.1 to 8.3)
Tracheal, bronchus, and lung cancer	Serbia	1995	All Ages	0.5 (0.3 to 0.8)	11.7% (6.3 to 18.0%)	5.3 (2.9 to 8.3)
Tracheal, bronchus, and lung cancer	Serbia	2000	All Ages	0.6 (0.3 to 0.9)	11.5% (6.3 to 17.7%)	6.1 (3.3 to 9.4)
Tracheal, bronchus, and lung cancer	Serbia	2005	All Ages	0.6 (0.3 to 0.9)	10.1% (5.2 to 16.0%)	6.1 (3.1 to 9.8)
Tracheal, bronchus, and lung cancer	Serbia	2010	All Ages	0.7 (0.4 to 1.0)	11.7% (6.3 to 18.1%)	7.5 (4.0 to 11.5)
Tracheal, bronchus, and lung cancer	Serbia	2015	All Ages	0.7 (0.4 to 1.0)	12.4% (6.8 to 18.7%)	7.4 (4.0 to 11.3)
Tracheal, bronchus, and lung cancer	Serbia	1990	Age-standardized		14.1% (8.0 to 21.0%)	5.0 (2.8 to 7.6)
Tracheal, bronchus, and lung cancer	Serbia	1995	Age-standardized		11.7% (6.3 to 18.0%)	4.5 (2.5 to 7.0)
Tracheal, bronchus, and lung cancer	Serbia	2000	Age-standardized		11.5% (6.3 to 17.7%)	4.7 (2.5 to 7.3)
Tracheal, bronchus, and lung cancer	Serbia	2005	Age-standardized		10.1% (5.2 to 16.0%)	4.5 (2.3 to 7.2)
Tracheal, bronchus, and lung cancer	Serbia	2010	Age-standardized		11.7% (6.3 to 18.1%)	5.0 (2.7 to 7.8)
Tracheal, bronchus, and lung cancer	Serbia	2015	Age-standardized		12.4% (6.8 to 18.7%)	4.7 (2.6 to 7.3)
Cardiovascular diseases	Serbia	1990	All Ages	5.6 (4.6 to 6.6)	12.4% (10.3 to 14.4%)	58.7 (48.8 to 69.1)
Cardiovascular diseases	Serbia	1995	All Ages	6.6 (5.4 to 7.7)	11.5% (9.4 to 13.5%)	66.4 (54.1 to 78.1)
Cardiovascular diseases	Serbia	2000	All Ages	7.1 (5.9 to 8.3)	11.3% (9.3 to 13.3%)	74.9 (61.9 to 87.8)
Cardiovascular diseases	Serbia	2005	All Ages	6.4 (5.2 to 7.6)	10.3% (8.3 to 12.2%)	69.5 (56.4 to 82.8)
Cardiovascular diseases	Serbia	2010	All Ages	6.2 (5.0 to 7.4)	10.6% (8.5 to 12.6%)	68.0 (54.9 to 81.1)
Cardiovascular diseases	Serbia	2015	All Ages	5.8 (4.6 to 7.0)	10.6% (8.5 to 12.8%)	65.0 (51.8 to 78.6)
Cardiovascular diseases	Serbia	1990	Age-standardized		11.9% (9.8 to 14.1%)	61.2 (50.2 to 72.8)
Cardiovascular diseases	Serbia	1995	Age-standardized		11.0% (8.9 to 13.1%)	65.7 (52.8 to 78.2)
Cardiovascular diseases	Serbia	2000	Age-standardized		10.9% (8.8 to 13.0%)	65.4 (53.2 to 77.9)
Cardiovascular diseases	Serbia	2005	Age-standardized		10.1% (8.1 to 12.0%)	52.5 (42.3 to 62.9)
Cardiovascular diseases	Serbia	2010	Age-standardized		10.6% (8.6 to 12.6%)	45.9 (37.0 to 55.0)
Cardiovascular diseases	Serbia	2015	Age-standardized		10.8% (8.6 to 12.9%)	40.0 (32.1 to 48.2)
Ischemic heart disease	Serbia	1990	All Ages	3.3 (2.6 to 4.0)	15.6% (12.3 to 19.0%)	34.6 (27.0 to 42.3)
Ischemic heart disease	Serbia	1995	All Ages	3.9 (3.0 to 4.7)	14.6% (11.3 to 17.7%)	39.2 (30.5 to 47.7)
Ischemic heart disease	Serbia	2000	All Ages	4.1 (3.3 to 5.0)	14.5% (11.4 to 17.5%)	43.6 (34.3 to 53.0)
Ischemic heart disease	Serbia	2005	All Ages	3.8 (2.9 to 4.7)	13.4% (10.3 to 16.5%)	41.3 (31.8 to 50.9)
Ischemic heart disease	Serbia	2010	All Ages	3.7 (2.8 to 4.6)	13.9% (10.5 to 17.2%)	40.8 (30.9 to 50.5)
Ischemic heart disease	Serbia	2015	All Ages	3.5 (2.6 to 4.4)	14.0% (10.3 to 17.7%)	39.3 (29.1 to 49.7)
Ischemic heart disease	Serbia	1990	Age-standardized		15.1% (11.5 to 18.6%)	35.8 (27.4 to 44.4)
Ischemic heart disease	Serbia	1995	Age-standardized		13.9% (10.5 to 17.3%)	38.7 (28.9 to 48.2)
Ischemic heart disease	Serbia	2000	Age-standardized		13.9% (10.5 to 17.3%)	38.3 (28.9 to 47.8)
Ischemic heart disease	Serbia	2005	Age-standardized		13.3% (10.0 to 16.4%)	31.4 (23.9 to 38.7)
Ischemic heart disease	Serbia	2010	Age-standardized		13.9% (10.5 to 17.4%)	27.6 (20.7 to 34.4)

Cause name	Location	Year	Age group	Number (in thousands)	PAF	Rate (per 100,000)
Ischemic heart disease	Serbia	2015	Age-standardized		14.2% (10.7 to 17.8%)	24.3 (18.4 to 30.4)
Ischemic stroke	Serbia	1990	All Ages	1.3 (0.9 to 1.7)	11.3% (8.1 to 14.7%)	13.3 (9.2 to 17.6)
Ischemic stroke	Serbia	1995	All Ages	1.5 (1.0 to 2.0)	10.3% (7.1 to 13.7%)	15.0 (10.4 to 19.8)
Ischemic stroke	Serbia	2000	All Ages	1.7 (1.1 to 2.2)	10.3% (7.0 to 13.5%)	17.7 (12.1 to 23.1)
Ischemic stroke	Serbia	2005	All Ages	1.6 (1.0 to 2.2)	9.4% (6.2 to 12.7%)	17.5 (11.3 to 23.7)
Ischemic stroke	Serbia	2010	All Ages	1.7 (1.1 to 2.3)	9.9% (6.6 to 13.4%)	18.2 (12.0 to 24.8)
Ischemic stroke	Serbia	2015	All Ages	1.5 (1.0 to 2.1)	10.1% (6.6 to 14.0%)	17.4 (11.1 to 24.3)
Ischemic stroke	Serbia	1990	Age-standardized		11.0% (7.7 to 14.5%)	14.4 (9.7 to 19.4)
Ischemic stroke	Serbia	1995	Age-standardized		9.9% (6.6 to 13.5%)	15.5 (10.2 to 21.2)
Ischemic stroke	Serbia	2000	Age-standardized		9.9% (6.5 to 13.4%)	15.8 (10.3 to 21.6)
Ischemic stroke	Serbia	2005	Age-standardized		9.3% (6.0 to 12.9%)	13.1 (8.3 to 18.6)
Ischemic stroke	Serbia	2010	Age-standardized		9.9% (6.5 to 13.5%)	12.0 (7.8 to 16.7)
Ischemic stroke	Serbia	2015	Age-standardized		10.2% (6.6 to 14.0%)	10.5 (6.8 to 14.6)
Hemorrhagic stroke	Serbia	1990	All Ages	1.0 (0.8 to 1.3)	12.7% (9.9 to 15.7%)	10.8 (8.3 to 13.4)
Hemorrhagic stroke	Serbia	1995	All Ages	1.2 (0.9 to 1.5)	11.5% (8.6 to 14.6%)	12.3 (9.2 to 15.6)
Hemorrhagic stroke	Serbia	2000	All Ages	1.3 (1.0 to 1.7)	11.3% (8.4 to 14.5%)	13.5 (10.0 to 17.4)
Hemorrhagic stroke	Serbia	2005	All Ages	1.0 (0.7 to 1.3)	10.4% (7.5 to 13.5%)	10.8 (7.7 to 14.2)
Hemorrhagic stroke	Serbia	2010	All Ages	0.8 (0.6 to 1.1)	10.9% (8.0 to 13.9%)	9.1 (6.6 to 11.9)
Hemorrhagic stroke	Serbia	2015	All Ages	0.7 (0.5 to 1.0)	11.1% (8.0 to 14.3%)	8.3 (5.9 to 10.8)
Hemorrhagic stroke	Serbia	1990	Age-standardized		12.3% (9.4 to 15.2%)	10.9 (8.2 to 13.6)
Hemorrhagic stroke	Serbia	1995	Age-standardized		11.1% (8.2 to 14.0%)	11.5 (8.5 to 14.7)
Hemorrhagic stroke	Serbia	2000	Age-standardized		11.0% (8.1 to 13.9%)	11.3 (8.3 to 14.3)
Hemorrhagic stroke	Serbia	2005	Age-standardized		10.3% (7.5 to 13.2%)	8.0 (5.7 to 10.4)
Hemorrhagic stroke	Serbia	2010	Age-standardized		11.0% (8.1 to 14.0%)	6.2 (4.5 to 8.0)
Hemorrhagic stroke	Serbia	2015	Age-standardized		11.3% (8.4 to 14.4%)	5.2 (3.8 to 6.8)
Chronic obstructive pulmonary disease	Serbia	1990	All Ages	0.5 (0.2 to 0.7)	20.6% (10.3 to 31.6%)	4.9 (2.4 to 7.4)
Chronic obstructive pulmonary disease	Serbia	1995	All Ages	0.5 (0.2 to 0.8)	18.0% (8.4 to 28.7%)	5.2 (2.4 to 8.3)
Chronic obstructive pulmonary disease	Serbia	2000	All Ages	0.6 (0.3 to 0.9)	17.8% (8.2 to 28.5%)	5.9 (2.7 to 9.4)
Chronic obstructive pulmonary disease	Serbia	2005	All Ages	0.5 (0.2 to 0.9)	16.3% (7.2 to 27.1%)	5.7 (2.5 to 9.5)
Chronic obstructive pulmonary disease	Serbia	2010	All Ages	0.6 (0.3 to 0.9)	18.1% (8.5 to 28.9%)	6.3 (2.9 to 10.0)
Chronic obstructive pulmonary disease	Serbia	2015	All Ages	0.6 (0.3 to 0.9)	18.8% (9.0 to 29.6%)	6.2 (2.9 to 9.8)
Chronic obstructive pulmonary disease	Serbia	1990	Age-standardized		20.6% (10.3 to 31.6%)	5.1 (2.6 to 7.9)
Chronic obstructive pulmonary disease	Serbia	1995	Age-standardized		18.0% (8.4 to 28.7%)	5.1 (2.4 to 8.1)
Chronic obstructive pulmonary disease	Serbia	2000	Age-standardized		17.8% (8.2 to 28.5%)	5.0 (2.3 to 8.0)
Chronic obstructive pulmonary disease	Serbia	2005	Age-standardized		16.3% (7.2 to 27.0%)	4.2 (1.9 to 7.0)
Chronic obstructive pulmonary disease	Serbia	2010	Age-standardized		18.1% (8.5 to 28.8%)	4.2 (1.9 to 6.7)

Cause name	Location	Year	Age group	Number (in thousands)	PAF	Rate (per 100,000)
Chronic obstructive pulmonary disease	Serbia	2015	Age-standardized		18.8% (9.0 to 29.6%)	3.8 (1.8 to 6.0)
All causes	Slovakia	1990	All Ages	5.6 (4.9 to 6.5)	10.3% (8.9 to 11.9%)	107.0 (92.2 to 123.4)
All causes	Slovakia	1995	All Ages	4.7 (3.9 to 5.5)	9.0% (7.5 to 10.6%)	87.9 (73.5 to 103.0)
All causes	Slovakia	2000	All Ages	4.3 (3.6 to 5.0)	8.1% (6.8 to 9.5%)	79.2 (65.9 to 92.4)
All causes	Slovakia	2005	All Ages	4.1 (3.3 to 4.8)	7.6% (6.2 to 9.1%)	75.2 (61.3 to 89.5)
All causes	Slovakia	2010	All Ages	3.9 (3.1 to 4.6)	7.4% (6.0 to 8.8%)	70.9 (57.5 to 84.7)
All causes	Slovakia	2015	All Ages	3.5 (2.8 to 4.2)	7.1% (5.7 to 8.5%)	63.2 (51.1 to 76.1)
All causes	Slovakia	1990	Age-standardized		10.3% (8.9 to 12.0%)	110.3 (94.2 to 127.8)
All causes	Slovakia	1995	Age-standardized		9.0% (7.5 to 10.6%)	87.5 (73.4 to 103.1)
All causes	Slovakia	2000	Age-standardized		8.1% (6.6 to 9.5%)	76.4 (62.7 to 90.3)
All causes	Slovakia	2005	Age-standardized		7.6% (6.1 to 9.0%)	64.7 (52.7 to 77.1)
All causes	Slovakia	2010	Age-standardized		7.3% (5.9 to 8.7%)	56.1 (45.5 to 67.2)
All causes	Slovakia	2015	Age-standardized		7.0% (5.6 to 8.4%)	46.3 (37.4 to 55.8)
Lower respiratory infections	Slovakia	1990	All Ages	0.7 (0.4 to 0.9)	25.4% (17.5 to 33.7%)	12.5 (7.9 to 17.4)
Lower respiratory infections	Slovakia	1995	All Ages	0.5 (0.3 to 0.8)	20.5% (11.9 to 30.2%)	9.1 (5.1 to 14.1)
Lower respiratory infections	Slovakia	2000	All Ages	0.4 (0.2 to 0.6)	17.8% (9.2 to 27.9%)	6.5 (3.3 to 10.5)
Lower respiratory infections	Slovakia	2005	All Ages	0.3 (0.2 to 0.6)	16.8% (8.2 to 27.1%)	6.2 (3.0 to 10.4)
Lower respiratory infections	Slovakia	2010	All Ages	0.3 (0.2 to 0.5)	16.9% (8.4 to 27.1%)	6.1 (2.9 to 10.0)
Lower respiratory infections	Slovakia	2015	All Ages	0.3 (0.1 to 0.5)	17.1% (8.4 to 27.3%)	5.7 (2.7 to 9.4)
Lower respiratory infections	Slovakia	1990	Age-standardized		25.4% (17.5 to 33.7%)	13.2 (8.4 to 18.5)
Lower respiratory infections	Slovakia	1995	Age-standardized		20.5% (11.9 to 30.2%)	9.5 (5.2 to 14.6)
Lower respiratory infections	Slovakia	2000	Age-standardized		17.8% (9.2 to 27.9%)	6.7 (3.4 to 10.8)
Lower respiratory infections	Slovakia	2005	Age-standardized		16.8% (8.2 to 27.1%)	5.5 (2.7 to 9.2)
Lower respiratory infections	Slovakia	2010	Age-standardized		16.9% (8.4 to 27.1%)	5.0 (2.4 to 8.1)
Lower respiratory infections	Slovakia	2015	Age-standardized		17.1% (8.4 to 27.3%)	4.3 (2.0 to 7.1)
Tracheal, bronchus, and lung cancer	Slovakia	1990	All Ages	0.4 (0.3 to 0.6)	18.2% (11.1 to 25.7%)	8.0 (4.8 to 11.3)
Tracheal, bronchus, and lung cancer	Slovakia	1995	All Ages	0.3 (0.2 to 0.5)	14.5% (8.3 to 21.5%)	5.9 (3.4 to 8.8)
Tracheal, bronchus, and lung cancer	Slovakia	2000	All Ages	0.3 (0.1 to 0.4)	12.5% (6.8 to 19.0%)	5.1 (2.8 to 7.8)
Tracheal, bronchus, and lung cancer	Slovakia	2005	All Ages	0.3 (0.1 to 0.4)	11.8% (6.4 to 18.1%)	4.7 (2.6 to 7.3)
Tracheal, bronchus, and lung cancer	Slovakia	2010	All Ages	0.3 (0.1 to 0.4)	11.9% (6.4 to 18.2%)	4.9 (2.6 to 7.5)
Tracheal, bronchus, and lung cancer	Slovakia	2015	All Ages	0.3 (0.1 to 0.4)	12.0% (6.6 to 18.3%)	4.7 (2.5 to 7.2)
Tracheal, bronchus, and lung cancer	Slovakia	1990	Age-standardized		18.2% (11.1 to 25.7%)	7.9 (4.8 to 11.2)
Tracheal, bronchus, and lung cancer	Slovakia	1995	Age-standardized		14.5% (8.3 to 21.5%)	5.7 (3.2 to 8.4)
Tracheal, bronchus, and lung cancer	Slovakia	2000	Age-standardized		12.5% (6.8 to 19.0%)	4.6 (2.5 to 7.1)
Tracheal, bronchus, and lung cancer	Slovakia	2005	Age-standardized		11.8% (6.4 to 18.1%)	4.0 (2.2 to 6.2)
Tracheal, bronchus, and lung cancer	Slovakia	2010	Age-standardized		11.9% (6.4 to 18.2%)	3.8 (2.0 to 5.8)

Cause name	Location	Year	Age group	Number (in thousands)	PAF	Rate (per 100,000)
Tracheal, bronchus, and lung cancer	Slovakia	2015	Age-standardized		12.0% (6.6 to 18.3%)	3.4 (1.8 to 5.3)
Cardiovascular diseases	Slovakia	1990	All Ages	4.4 (3.7 to 5.2)	14.9% (12.5 to 17.6%)	83.0 (69.4 to 98.1)
Cardiovascular diseases	Slovakia	1995	All Ages	3.8 (3.1 to 4.5)	13.3% (10.9 to 16.0%)	70.3 (57.4 to 84.0)
Cardiovascular diseases	Slovakia	2000	All Ages	3.5 (2.9 to 4.2)	12.3% (10.1 to 14.7%)	64.8 (52.9 to 77.6)
Cardiovascular diseases	Slovakia	2005	All Ages	3.3 (2.6 to 4.0)	11.7% (9.3 to 14.2%)	61.4 (48.9 to 74.2)
Cardiovascular diseases	Slovakia	2010	All Ages	3.1 (2.4 to 3.8)	11.5% (9.0 to 14.0%)	56.9 (44.5 to 69.8)
Cardiovascular diseases	Slovakia	2015	All Ages	2.8 (2.1 to 3.5)	11.3% (8.6 to 13.9%)	49.9 (38.4 to 62.1)
Cardiovascular diseases	Slovakia	1990	Age-standardized		14.7% (12.2 to 17.6%)	85.5 (70.6 to 102.0)
Cardiovascular diseases	Slovakia	1995	Age-standardized		13.2% (10.7 to 15.8%)	69.9 (56.5 to 84.0)
Cardiovascular diseases	Slovakia	2000	Age-standardized		12.1% (9.6 to 14.6%)	62.4 (49.7 to 75.6)
Cardiovascular diseases	Slovakia	2005	Age-standardized		11.7% (9.2 to 14.1%)	52.7 (41.6 to 64.2)
Cardiovascular diseases	Slovakia	2010	Age-standardized		11.5% (8.9 to 14.0%)	44.9 (35.2 to 55.2)
Cardiovascular diseases	Slovakia	2015	Age-standardized		11.3% (8.7 to 13.9%)	36.4 (28.3 to 45.1)
Ischemic heart disease	Slovakia	1990	All Ages	3.4 (2.7 to 4.2)	17.2% (13.8 to 21.0%)	65.0 (51.7 to 79.0)
Ischemic heart disease	Slovakia	1995	All Ages	3.0 (2.3 to 3.7)	15.6% (12.1 to 19.1%)	55.6 (43.1 to 68.3)
Ischemic heart disease	Slovakia	2000	All Ages	2.8 (2.1 to 3.4)	14.5% (11.2 to 17.8%)	51.4 (39.8 to 63.1)
Ischemic heart disease	Slovakia	2005	All Ages	2.6 (2.0 to 3.3)	13.9% (10.6 to 17.4%)	48.9 (37.0 to 61.2)
Ischemic heart disease	Slovakia	2010	All Ages	2.5 (1.8 to 3.1)	13.7% (10.1 to 17.3%)	45.3 (33.7 to 57.2)
Ischemic heart disease	Slovakia	2015	All Ages	2.2 (1.6 to 2.8)	13.6% (9.7 to 17.4%)	39.7 (28.4 to 51.1)
Ischemic heart disease	Slovakia	1990	Age-standardized		17.0% (13.4 to 20.8%)	67.0 (52.5 to 82.1)
Ischemic heart disease	Slovakia	1995	Age-standardized		15.3% (11.9 to 19.0%)	55.2 (42.6 to 68.3)
Ischemic heart disease	Slovakia	2000	Age-standardized		14.2% (10.7 to 17.7%)	49.7 (37.4 to 62.0)
Ischemic heart disease	Slovakia	2005	Age-standardized		13.8% (10.4 to 17.4%)	42.0 (31.5 to 52.9)
Ischemic heart disease	Slovakia	2010	Age-standardized		13.7% (10.1 to 17.3%)	35.8 (26.6 to 45.1)
Ischemic heart disease	Slovakia	2015	Age-standardized		13.6% (9.8 to 17.4%)	29.0 (20.9 to 37.0)
Ischemic stroke	Slovakia	1990	All Ages	0.5 (0.4 to 0.7)	13.1% (9.6 to 16.5%)	9.9 (7.4 to 12.7)
Ischemic stroke	Slovakia	1995	All Ages	0.5 (0.3 to 0.6)	11.5% (8.0 to 15.0%)	8.4 (5.9 to 11.0)
Ischemic stroke	Slovakia	2000	All Ages	0.4 (0.3 to 0.6)	10.6% (7.3 to 14.0%)	8.0 (5.5 to 10.7)
Ischemic stroke	Slovakia	2005	All Ages	0.4 (0.3 to 0.6)	10.2% (6.9 to 13.7%)	7.8 (5.3 to 10.5)
Ischemic stroke	Slovakia	2010	All Ages	0.4 (0.3 to 0.6)	10.1% (6.7 to 13.8%)	7.6 (5.1 to 10.3)
Ischemic stroke	Slovakia	2015	All Ages	0.4 (0.2 to 0.5)	10.0% (6.6 to 14.0%)	6.7 (4.4 to 9.5)
Ischemic stroke	Slovakia	1990	Age-standardized		12.9% (9.4 to 16.5%)	10.3 (7.5 to 13.4)
Ischemic stroke	Slovakia	1995	Age-standardized		11.3% (7.9 to 14.9%)	8.5 (5.9 to 11.2)
Ischemic stroke	Slovakia	2000	Age-standardized		10.4% (7.0 to 14.1%)	7.7 (5.2 to 10.5)
Ischemic stroke	Slovakia	2005	Age-standardized		10.1% (6.8 to 13.7%)	6.7 (4.5 to 9.2)
Ischemic stroke	Slovakia	2010	Age-standardized		10.1% (6.7 to 13.8%)	6.0 (4.0 to 8.2)

Cause name	Location	Year	Age group	Number (in thousands)	PAF	Rate (per 100,000)
Ischemic stroke	Slovakia	2015	Age-standardized		10.1% (6.6 to 14.0%)	4.9 (3.3 to 6.9)
Hemorrhagic stroke	Slovakia	1990	All Ages	0.4 (0.3 to 0.5)	14.6% (11.6 to 17.6%)	8.0 (6.3 to 9.8)
Hemorrhagic stroke	Slovakia	1995	All Ages	0.3 (0.3 to 0.4)	12.8% (9.8 to 15.9%)	6.3 (4.8 to 8.0)
Hemorrhagic stroke	Slovakia	2000	All Ages	0.3 (0.2 to 0.4)	11.8% (8.8 to 14.6%)	5.4 (4.0 to 6.8)
Hemorrhagic stroke	Slovakia	2005	All Ages	0.3 (0.2 to 0.3)	11.3% (8.5 to 14.1%)	4.7 (3.5 to 5.9)
Hemorrhagic stroke	Slovakia	2010	All Ages	0.2 (0.2 to 0.3)	11.3% (8.5 to 14.4%)	4.0 (3.0 to 5.1)
Hemorrhagic stroke	Slovakia	2015	All Ages	0.2 (0.1 to 0.3)	11.3% (8.3 to 14.3%)	3.4 (2.5 to 4.5)
Hemorrhagic stroke	Slovakia	1990	Age-standardized		14.4% (11.4 to 17.4%)	8.2 (6.4 to 10.1)
Hemorrhagic stroke	Slovakia	1995	Age-standardized		12.6% (9.7 to 15.6%)	6.2 (4.7 to 7.7)
Hemorrhagic stroke	Slovakia	2000	Age-standardized		11.5% (8.5 to 14.5%)	5.0 (3.7 to 6.4)
Hemorrhagic stroke	Slovakia	2005	Age-standardized		11.3% (8.4 to 14.2%)	4.0 (3.0 to 5.0)
Hemorrhagic stroke	Slovakia	2010	Age-standardized		11.3% (8.5 to 14.3%)	3.2 (2.3 to 4.1)
Hemorrhagic stroke	Slovakia	2015	Age-standardized		11.3% (8.4 to 14.2%)	2.5 (1.9 to 3.2)
Chronic obstructive pulmonary disease	Slovakia	1990	All Ages	0.2 (0.1 to 0.3)	24.8% (13.9 to 36.1%)	3.6 (2.0 to 5.2)
Chronic obstructive pulmonary disease	Slovakia	1995	All Ages	0.1 (0.1 to 0.2)	21.1% (10.7 to 32.0%)	2.6 (1.3 to 3.9)
Chronic obstructive pulmonary disease	Slovakia	2000	All Ages	0.1 (0.1 to 0.2)	18.9% (9.1 to 29.9%)	2.8 (1.3 to 4.3)
Chronic obstructive pulmonary disease	Slovakia	2005	All Ages	0.2 (0.1 to 0.2)	18.1% (8.4 to 28.9%)	2.9 (1.4 to 4.5)
Chronic obstructive pulmonary disease	Slovakia	2010	All Ages	0.2 (0.1 to 0.3)	18.2% (8.6 to 29.0%)	3.1 (1.5 to 5.0)
Chronic obstructive pulmonary disease	Slovakia	2015	All Ages	0.2 (0.1 to 0.3)	18.4% (8.7 to 29.2%)	2.9 (1.4 to 4.7)
Chronic obstructive pulmonary disease	Slovakia	1990	Age-standardized		24.8% (13.9 to 36.0%)	3.7 (2.0 to 5.3)
Chronic obstructive pulmonary disease	Slovakia	1995	Age-standardized		21.1% (10.7 to 32.0%)	2.6 (1.3 to 3.9)
Chronic obstructive pulmonary disease	Slovakia	2000	Age-standardized		18.9% (9.0 to 29.9%)	2.7 (1.3 to 4.2)
Chronic obstructive pulmonary disease	Slovakia	2005	Age-standardized		18.1% (8.4 to 28.8%)	2.5 (1.2 to 3.9)
Chronic obstructive pulmonary disease	Slovakia	2010	Age-standardized		18.2% (8.6 to 29.0%)	2.5 (1.2 to 3.9)
Chronic obstructive pulmonary disease	Slovakia	2015	Age-standardized		18.3% (8.6 to 29.2%)	2.2 (1.0 to 3.5)
All causes	Slovenia	1990	All Ages	1.2 (1.0 to 1.4)	6.4% (5.5 to 7.4%)	59.8 (51.2 to 69.6)
All causes	Slovenia	1995	All Ages	1.1 (0.9 to 1.3)	5.9% (4.9 to 7.0%)	56.6 (47.2 to 66.5)
All causes	Slovenia	2000	All Ages	1.0 (0.8 to 1.2)	5.3% (4.4 to 6.3%)	49.6 (41.2 to 59.2)
All causes	Slovenia	2005	All Ages	0.9 (0.8 to 1.1)	5.0% (4.0 to 5.9%)	46.6 (38.2 to 55.7)
All causes	Slovenia	2010	All Ages	0.9 (0.7 to 1.1)	4.7% (3.8 to 5.7%)	43.0 (34.6 to 52.6)
All causes	Slovenia	2015	All Ages	1.0 (0.8 to 1.3)	5.4% (4.3 to 6.5%)	50.4 (40.6 to 61.5)
All causes	Slovenia	1990	Age-standardized		6.3% (5.4 to 7.4%)	55.5 (47.5 to 64.8)
All causes	Slovenia	1995	Age-standardized		5.8% (4.9 to 6.8%)	47.9 (40.1 to 56.2)
All causes	Slovenia	2000	Age-standardized		5.2% (4.2 to 6.2%)	39.9 (32.8 to 48.0)
All causes	Slovenia	2005	Age-standardized		4.8% (3.9 to 5.8%)	31.8 (25.9 to 38.1)
All causes	Slovenia	2010	Age-standardized		4.5% (3.7 to 5.5%)	25.3 (20.5 to 30.6)

Cause name	Location	Year	Age group	Number (in thousands)	PAF	Rate (per 100,000)
All causes	Slovenia	2015	Age-standardized		5.2% (4.2 to 6.2%)	25.9 (21.2 to 31.3)
Lower respiratory infections	Slovenia	1990	All Ages	0.1 (0.1 to 0.2)	19.6% (11.1 to 29.6%)	5.6 (3.2 to 8.6)
Lower respiratory infections	Slovenia	1995	All Ages	0.1 (0.1 to 0.2)	16.5% (8.0 to 26.8%)	5.4 (2.7 to 8.9)
Lower respiratory infections	Slovenia	2000	All Ages	0.1 (0.1 to 0.2)	14.4% (6.2 to 25.0%)	5.4 (2.3 to 9.2)
Lower respiratory infections	Slovenia	2005	All Ages	0.1 (0.0 to 0.2)	14.3% (6.0 to 24.9%)	5.4 (2.2 to 9.4)
Lower respiratory infections	Slovenia	2010	All Ages	0.1 (0.0 to 0.2)	13.7% (5.4 to 24.4%)	5.0 (2.0 to 9.1)
Lower respiratory infections	Slovenia	2015	All Ages	0.1 (0.1 to 0.2)	17.0% (8.4 to 27.2%)	6.7 (3.2 to 10.9)
Lower respiratory infections	Slovenia	1990	Age-standardized		19.6% (11.1 to 29.6%)	5.3 (3.0 to 8.2)
Lower respiratory infections	Slovenia	1995	Age-standardized		16.5% (8.0 to 26.8%)	4.7 (2.3 to 7.7)
Lower respiratory infections	Slovenia	2000	Age-standardized		14.4% (6.2 to 25.0%)	4.6 (1.9 to 8.0)
Lower respiratory infections	Slovenia	2005	Age-standardized		14.3% (6.0 to 24.9%)	3.7 (1.5 to 6.5)
Lower respiratory infections	Slovenia	2010	Age-standardized		13.7% (5.4 to 24.4%)	2.9 (1.1 to 5.2)
Lower respiratory infections	Slovenia	2015	Age-standardized		17.0% (8.4 to 27.2%)	3.2 (1.6 to 5.3)
Tracheal, bronchus, and lung cancer	Slovenia	1990	All Ages	0.1 (0.1 to 0.2)	13.9% (7.8 to 20.7%)	5.8 (3.2 to 8.6)
Tracheal, bronchus, and lung cancer	Slovenia	1995	All Ages	0.1 (0.1 to 0.2)	11.6% (6.2 to 17.9%)	5.4 (2.9 to 8.4)
Tracheal, bronchus, and lung cancer	Slovenia	2000	All Ages	0.1 (0.1 to 0.2)	10.0% (5.1 to 16.0%)	5.1 (2.6 to 8.1)
Tracheal, bronchus, and lung cancer	Slovenia	2005	All Ages	0.1 (0.1 to 0.2)	9.9% (5.1 to 15.7%)	5.3 (2.8 to 8.5)
Tracheal, bronchus, and lung cancer	Slovenia	2010	All Ages	0.1 (0.1 to 0.2)	9.5% (4.7 to 15.3%)	4.8 (2.4 to 7.8)
Tracheal, bronchus, and lung cancer	Slovenia	2015	All Ages	0.1 (0.1 to 0.2)	11.9% (6.3 to 18.3%)	5.5 (3.0 to 8.6)
Tracheal, bronchus, and lung cancer	Slovenia	1990	Age-standardized		13.9% (7.8 to 20.7%)	5.1 (2.9 to 7.6)
Tracheal, bronchus, and lung cancer	Slovenia	1995	Age-standardized		11.6% (6.2 to 17.9%)	4.4 (2.4 to 6.9)
Tracheal, bronchus, and lung cancer	Slovenia	2000	Age-standardized		10.0% (5.1 to 15.9%)	3.8 (2.0 to 6.0)
Tracheal, bronchus, and lung cancer	Slovenia	2005	Age-standardized		9.9% (5.1 to 15.7%)	3.7 (1.9 to 5.8)
Tracheal, bronchus, and lung cancer	Slovenia	2010	Age-standardized		9.5% (4.7 to 15.3%)	3.1 (1.5 to 5.0)
Tracheal, bronchus, and lung cancer	Slovenia	2015	Age-standardized		11.9% (6.3 to 18.2%)	3.2 (1.7 to 5.0)
Cardiovascular diseases	Slovenia	1990	All Ages	0.8 (0.7 to 1.0)	9.7% (8.0 to 11.5%)	42.5 (35.1 to 50.2)
Cardiovascular diseases	Slovenia	1995	All Ages	0.8 (0.6 to 1.0)	9.5% (7.6 to 11.5%)	39.8 (31.7 to 47.8)
Cardiovascular diseases	Slovenia	2000	All Ages	0.7 (0.5 to 0.8)	8.8% (7.0 to 10.5%)	33.8 (27.0 to 40.8)
Cardiovascular diseases	Slovenia	2005	All Ages	0.6 (0.5 to 0.8)	8.3% (6.5 to 10.1%)	31.0 (24.0 to 37.8)
Cardiovascular diseases	Slovenia	2010	All Ages	0.6 (0.4 to 0.7)	7.9% (6.0 to 9.9%)	28.7 (21.6 to 36.0)
Cardiovascular diseases	Slovenia	2015	All Ages	0.7 (0.5 to 0.8)	8.5% (6.4 to 10.7%)	32.5 (24.4 to 41.2)
Cardiovascular diseases	Slovenia	1990	Age-standardized		9.6% (7.9 to 11.4%)	39.5 (32.3 to 46.8)
Cardiovascular diseases	Slovenia	1995	Age-standardized		9.4% (7.5 to 11.4%)	33.6 (26.9 to 40.5)
Cardiovascular diseases	Slovenia	2000	Age-standardized		8.6% (6.7 to 10.5%)	27.2 (21.3 to 33.4)
Cardiovascular diseases	Slovenia	2005	Age-standardized		8.3% (6.5 to 10.2%)	21.1 (16.4 to 25.8)
Cardiovascular diseases	Slovenia	2010	Age-standardized		8.1% (6.2 to 10.0%)	16.9 (12.9 to 20.9)

Cause name	Location	Year	Age group	Number (in thousands)	PAF	Rate (per 100,000)
Cardiovascular diseases	Slovenia	2015	Age-standardized		8.8% (6.8 to 10.8%)	16.6 (12.8 to 20.6)
Ischemic heart disease	Slovenia	1990	All Ages	0.5 (0.4 to 0.7)	15.2% (11.8 to 18.8%)	26.8 (20.6 to 33.2)
Ischemic heart disease	Slovenia	1995	All Ages	0.5 (0.4 to 0.7)	13.9% (10.4 to 17.4%)	26.4 (19.6 to 33.3)
Ischemic heart disease	Slovenia	2000	All Ages	0.5 (0.3 to 0.6)	13.1% (9.9 to 16.3%)	22.9 (17.1 to 28.7)
Ischemic heart disease	Slovenia	2005	All Ages	0.4 (0.3 to 0.5)	12.7% (9.2 to 16.2%)	21.3 (15.3 to 27.2)
Ischemic heart disease	Slovenia	2010	All Ages	0.4 (0.3 to 0.5)	12.1% (8.3 to 15.9%)	19.7 (13.4 to 25.7)
Ischemic heart disease	Slovenia	2015	All Ages	0.5 (0.3 to 0.6)	12.9% (8.6 to 17.2%)	22.3 (14.7 to 29.9)
Ischemic heart disease	Slovenia	1990	Age-standardized		15.0% (11.6 to 18.6%)	24.8 (19.1 to 30.8)
Ischemic heart disease	Slovenia	1995	Age-standardized		13.8% (10.3 to 17.3%)	22.3 (16.7 to 28.1)
Ischemic heart disease	Slovenia	2000	Age-standardized		12.9% (9.3 to 16.3%)	18.5 (13.4 to 23.7)
Ischemic heart disease	Slovenia	2005	Age-standardized		12.8% (9.2 to 16.2%)	14.5 (10.4 to 18.6)
Ischemic heart disease	Slovenia	2010	Age-standardized		12.4% (8.8 to 16.0%)	11.6 (8.1 to 15.0)
Ischemic heart disease	Slovenia	2015	Age-standardized		13.4% (9.5 to 17.4%)	11.4 (8.0 to 14.9)
Ischemic stroke	Slovenia	1990	All Ages	0.2 (0.1 to 0.3)	10.9% (7.7 to 14.5%)	10.4 (7.3 to 14.1)
Ischemic stroke	Slovenia	1995	All Ages	0.1 (0.1 to 0.2)	9.9% (6.3 to 13.9%)	7.8 (4.9 to 11.0)
Ischemic stroke	Slovenia	2000	All Ages	0.1 (0.1 to 0.2)	9.1% (5.9 to 12.9%)	5.8 (3.7 to 8.1)
Ischemic stroke	Slovenia	2005	All Ages	0.1 (0.1 to 0.2)	8.9% (5.6 to 13.0%)	5.4 (3.3 to 7.8)
Ischemic stroke	Slovenia	2010	All Ages	0.1 (0.1 to 0.2)	8.5% (4.8 to 13.0%)	5.3 (3.0 to 7.9)
Ischemic stroke	Slovenia	2015	All Ages	0.1 (0.1 to 0.2)	9.4% (5.4 to 14.2%)	5.9 (3.4 to 8.9)
Ischemic stroke	Slovenia	1990	Age-standardized		10.9% (7.5 to 14.6%)	9.8 (6.8 to 13.3)
Ischemic stroke	Slovenia	1995	Age-standardized		9.8% (6.4 to 13.7%)	6.7 (4.3 to 9.3)
Ischemic stroke	Slovenia	2000	Age-standardized		9.0% (5.4 to 13.1%)	4.7 (2.8 to 6.8)
Ischemic stroke	Slovenia	2005	Age-standardized		8.9% (5.5 to 13.1%)	3.6 (2.2 to 5.3)
Ischemic stroke	Slovenia	2010	Age-standardized		8.6% (5.0 to 13.0%)	3.0 (1.7 to 4.4)
Ischemic stroke	Slovenia	2015	Age-standardized		9.6% (5.8 to 14.1%)	2.9 (1.8 to 4.2)
Hemorrhagic stroke	Slovenia	1990	All Ages	0.1 (0.1 to 0.1)	12.8% (10.0 to 15.7%)	5.3 (4.1 to 6.5)
Hemorrhagic stroke	Slovenia	1995	All Ages	0.1 (0.1 to 0.1)	11.3% (8.4 to 14.4%)	5.6 (4.1 to 7.1)
Hemorrhagic stroke	Slovenia	2000	All Ages	0.1 (0.1 to 0.1)	10.3% (7.4 to 13.2%)	5.2 (3.7 to 6.6)
Hemorrhagic stroke	Slovenia	2005	All Ages	0.1 (0.1 to 0.1)	10.0% (7.1 to 13.1%)	4.3 (3.1 to 5.6)
Hemorrhagic stroke	Slovenia	2010	All Ages	0.1 (0.1 to 0.1)	9.4% (6.3 to 12.9%)	3.8 (2.6 to 5.3)
Hemorrhagic stroke	Slovenia	2015	All Ages	0.1 (0.1 to 0.1)	10.3% (7.0 to 14.1%)	4.2 (2.8 to 5.8)
Hemorrhagic stroke	Slovenia	1990	Age-standardized		12.7% (9.8 to 15.6%)	4.9 (3.8 to 6.0)
Hemorrhagic stroke	Slovenia	1995	Age-standardized		11.3% (8.4 to 14.2%)	4.7 (3.5 to 5.9)
Hemorrhagic stroke	Slovenia	2000	Age-standardized		10.2% (7.3 to 13.2%)	4.0 (2.9 to 5.3)
Hemorrhagic stroke	Slovenia	2005	Age-standardized		10.1% (7.2 to 13.2%)	2.9 (2.1 to 3.9)
Hemorrhagic stroke	Slovenia	2010	Age-standardized		9.6% (6.6 to 13.0%)	2.3 (1.6 to 3.1)

Cause name	Location	Year	Age group	Number (in thousands)	PAF	Rate (per 100,000)
Hemorrhagic stroke	Slovenia	2015	Age-standardized		10.7% (7.5 to 14.1%)	2.3 (1.6 to 3.0)
Chronic obstructive pulmonary disease	Slovenia	1990	All Ages	0.1 (0.1 to 0.2)	20.4% (10.1 to 31.3%)	6.0 (3.0 to 9.1)
Chronic obstructive pulmonary disease	Slovenia	1995	All Ages	0.1 (0.1 to 0.2)	17.9% (8.4 to 28.7%)	6.0 (2.9 to 9.5)
Chronic obstructive pulmonary disease	Slovenia	2000	All Ages	0.1 (0.1 to 0.2)	16.2% (7.2 to 26.9%)	5.4 (2.4 to 8.9)
Chronic obstructive pulmonary disease	Slovenia	2005	All Ages	0.1 (0.0 to 0.2)	16.1% (7.2 to 26.8%)	4.9 (2.2 to 8.3)
Chronic obstructive pulmonary disease	Slovenia	2010	All Ages	0.1 (0.0 to 0.1)	15.6% (6.7 to 26.0%)	4.4 (1.9 to 7.5)
Chronic obstructive pulmonary disease	Slovenia	2015	All Ages	0.1 (0.1 to 0.2)	18.3% (8.7 to 29.0%)	5.7 (2.7 to 9.2)
Chronic obstructive pulmonary disease	Slovenia	1990	Age-standardized		20.4% (10.1 to 31.3%)	5.6 (2.8 to 8.6)
Chronic obstructive pulmonary disease	Slovenia	1995	Age-standardized		17.9% (8.4 to 28.7%)	5.1 (2.4 to 8.1)
Chronic obstructive pulmonary disease	Slovenia	2000	Age-standardized		16.2% (7.2 to 26.8%)	4.3 (1.9 to 7.1)
Chronic obstructive pulmonary disease	Slovenia	2005	Age-standardized		16.1% (7.2 to 26.8%)	3.3 (1.5 to 5.5)
Chronic obstructive pulmonary disease	Slovenia	2010	Age-standardized		15.6% (6.7 to 26.0%)	2.5 (1.1 to 4.3)
Chronic obstructive pulmonary disease	Slovenia	2015	Age-standardized		18.3% (8.7 to 29.0%)	2.8 (1.3 to 4.5)
All causes	Armenia	1990	All Ages	1.9 (1.5 to 2.3)	7.6% (6.0 to 9.1%)	54.3 (43.5 to 65.4)
All causes	Armenia	1995	All Ages	2.0 (1.6 to 2.5)	7.3% (5.8 to 8.9%)	62.9 (49.9 to 76.3)
All causes	Armenia	2000	All Ages	2.0 (1.6 to 2.5)	7.6% (6.0 to 9.3%)	65.9 (52.5 to 80.0)
All causes	Armenia	2005	All Ages	2.3 (1.8 to 2.7)	7.8% (6.3 to 9.5%)	74.6 (60.5 to 90.9)
All causes	Armenia	2010	All Ages	2.3 (1.8 to 2.7)	8.1% (6.5 to 9.9%)	76.3 (61.1 to 92.5)
All causes	Armenia	2015	All Ages	2.3 (1.8 to 2.8)	8.0% (6.3 to 9.8%)	76.1 (59.8 to 93.3)
All causes	Armenia	1990	Age-standardized		8.0% (6.3 to 9.7%)	79.9 (63.4 to 97.6)
All causes	Armenia	1995	Age-standardized		7.4% (5.8 to 9.2%)	80.7 (63.4 to 99.2)
All causes	Armenia	2000	Age-standardized		7.6% (5.9 to 9.2%)	77.0 (59.5 to 94.4)
All causes	Armenia	2005	Age-standardized		7.8% (6.2 to 9.5%)	75.7 (60.7 to 92.7)
All causes	Armenia	2010	Age-standardized		8.1% (6.4 to 9.8%)	71.5 (57.2 to 86.7)
All causes	Armenia	2015	Age-standardized		7.9% (6.2 to 9.6%)	62.1 (48.9 to 75.9)
Lower respiratory infections	Armenia	1990	All Ages	0.2 (0.1 to 0.4)	16.5% (8.1 to 26.7%)	6.1 (3.0 to 10.0)
Lower respiratory infections	Armenia	1995	All Ages	0.1 (0.1 to 0.2)	14.1% (6.2 to 24.5%)	4.1 (1.8 to 7.2)
Lower respiratory infections	Armenia	2000	All Ages	0.1 (0.0 to 0.1)	15.1% (6.8 to 25.5%)	2.6 (1.1 to 4.4)
Lower respiratory infections	Armenia	2005	All Ages	0.1 (0.0 to 0.1)	16.7% (8.2 to 26.9%)	2.9 (1.4 to 4.7)
Lower respiratory infections	Armenia	2010	All Ages	0.1 (0.1 to 0.1)	19.6% (11.3 to 29.6%)	3.3 (1.7 to 5.2)
Lower respiratory infections	Armenia	2015	All Ages	0.1 (0.0 to 0.1)	19.3% (10.5 to 29.4%)	2.8 (1.5 to 4.4)
Lower respiratory infections	Armenia	1990	Age-standardized		16.5% (8.1 to 26.7%)	5.8 (2.8 to 9.6)
Lower respiratory infections	Armenia	1995	Age-standardized		14.1% (6.2 to 24.5%)	4.5 (2.0 to 7.9)
Lower respiratory infections	Armenia	2000	Age-standardized		15.1% (6.8 to 25.5%)	3.3 (1.4 to 5.7)
Lower respiratory infections	Armenia	2005	Age-standardized		16.7% (8.2 to 26.9%)	3.3 (1.6 to 5.5)
Lower respiratory infections	Armenia	2010	Age-standardized		19.6% (11.3 to 29.6%)	3.4 (1.9 to 5.4)

Cause name	Location	Year	Age group	Number (in thousands)	PAF	Rate (per 100,000)
Lower respiratory infections	Armenia	2015	Age-standardized		19.3% (10.5 to 29.4%)	2.7 (1.4 to 4.2)
Tracheal, bronchus, and lung cancer	Armenia	1990	All Ages	0.1 (0.1 to 0.1)	11.5% (5.9 to 18.0%)	2.7 (1.4 to 4.3)
Tracheal, bronchus, and lung cancer	Armenia	1995	All Ages	0.1 (0.1 to 0.1)	9.7% (4.8 to 16.1%)	2.8 (1.4 to 4.7)
Tracheal, bronchus, and lung cancer	Armenia	2000	All Ages	0.1 (0.1 to 0.2)	10.5% (4.9 to 17.3%)	3.3 (1.6 to 5.5)
Tracheal, bronchus, and lung cancer	Armenia	2005	All Ages	0.1 (0.1 to 0.2)	11.7% (5.8 to 19.0%)	4.4 (2.2 to 7.3)
Tracheal, bronchus, and lung cancer	Armenia	2010	All Ages	0.2 (0.1 to 0.3)	13.8% (7.4 to 21.5%)	5.5 (2.9 to 8.6)
Tracheal, bronchus, and lung cancer	Armenia	2015	All Ages	0.2 (0.1 to 0.3)	13.6% (7.4 to 21.3%)	5.6 (3.0 to 8.8)
Tracheal, bronchus, and lung cancer	Armenia	1990	Age-standardized		11.5% (5.9 to 18.0%)	3.4 (1.8 to 5.4)
Tracheal, bronchus, and lung cancer	Armenia	1995	Age-standardized		9.7% (4.8 to 16.1%)	3.1 (1.5 to 5.0)
Tracheal, bronchus, and lung cancer	Armenia	2000	Age-standardized		10.5% (5.0 to 17.3%)	3.4 (1.6 to 5.6)
Tracheal, bronchus, and lung cancer	Armenia	2005	Age-standardized		11.7% (5.8 to 19.0%)	4.3 (2.1 to 7.1)
Tracheal, bronchus, and lung cancer	Armenia	2010	Age-standardized		13.8% (7.4 to 21.5%)	5.2 (2.8 to 8.2)
Tracheal, bronchus, and lung cancer	Armenia	2015	Age-standardized		13.6% (7.4 to 21.3%)	4.6 (2.5 to 7.3)
Cardiovascular diseases	Armenia	1990	All Ages	1.4 (1.1 to 1.7)	12.0% (9.5 to 14.5%)	39.8 (31.4 to 48.2)
Cardiovascular diseases	Armenia	1995	All Ages	1.6 (1.3 to 2.0)	11.3% (9.0 to 13.8%)	49.6 (39.2 to 60.2)
Cardiovascular diseases	Armenia	2000	All Ages	1.7 (1.3 to 2.0)	11.5% (9.1 to 13.9%)	53.5 (42.2 to 65.2)
Cardiovascular diseases	Armenia	2005	All Ages	1.8 (1.4 to 2.2)	11.8% (9.5 to 14.3%)	60.0 (47.6 to 73.4)
Cardiovascular diseases	Armenia	2010	All Ages	1.8 (1.4 to 2.2)	12.5% (9.8 to 15.1%)	60.0 (47.0 to 73.8)
Cardiovascular diseases	Armenia	2015	All Ages	1.8 (1.4 to 2.3)	12.1% (9.3 to 14.9%)	60.4 (45.9 to 75.0)
Cardiovascular diseases	Armenia	1990	Age-standardized		11.5% (8.9 to 14.0%)	61.5 (47.5 to 75.6)
Cardiovascular diseases	Armenia	1995	Age-standardized		10.8% (8.3 to 13.4%)	64.9 (50.1 to 80.5)
Cardiovascular diseases	Armenia	2000	Age-standardized		11.0% (8.4 to 13.4%)	62.8 (47.9 to 77.6)
Cardiovascular diseases	Armenia	2005	Age-standardized		11.6% (9.1 to 14.2%)	60.7 (47.5 to 75.6)
Cardiovascular diseases	Armenia	2010	Age-standardized		12.5% (9.8 to 15.1%)	55.9 (43.6 to 68.6)
Cardiovascular diseases	Armenia	2015	Age-standardized		12.3% (9.5 to 15.1%)	48.8 (37.9 to 60.2)
Ischemic heart disease	Armenia	1990	All Ages	1.1 (0.8 to 1.3)	14.2% (10.8 to 17.5%)	30.7 (23.2 to 38.0)
Ischemic heart disease	Armenia	1995	All Ages	1.2 (1.0 to 1.5)	13.3% (10.2 to 16.6%)	38.4 (29.3 to 47.6)
Ischemic heart disease	Armenia	2000	All Ages	1.3 (1.0 to 1.6)	13.5% (10.1 to 16.6%)	40.8 (30.8 to 50.5)
Ischemic heart disease	Armenia	2005	All Ages	1.4 (1.0 to 1.7)	13.9% (10.5 to 17.2%)	45.4 (34.3 to 57.2)
Ischemic heart disease	Armenia	2010	All Ages	1.4 (1.0 to 1.7)	14.7% (11.1 to 18.3%)	46.0 (34.7 to 58.3)
Ischemic heart disease	Armenia	2015	All Ages	1.4 (1.0 to 1.8)	14.3% (10.4 to 18.2%)	46.8 (33.9 to 60.3)
Ischemic heart disease	Armenia	1990	Age-standardized		13.4% (9.8 to 16.8%)	47.3 (34.6 to 59.4)
Ischemic heart disease	Armenia	1995	Age-standardized		12.6% (9.2 to 16.1%)	50.2 (37.0 to 64.2)
Ischemic heart disease	Armenia	2000	Age-standardized		12.8% (9.1 to 16.2%)	48.2 (34.4 to 61.5)
Ischemic heart disease	Armenia	2005	Age-standardized		13.6% (10.2 to 17.1%)	46.2 (34.3 to 59.1)
Ischemic heart disease	Armenia	2010	Age-standardized		14.7% (11.2 to 18.5%)	42.9 (32.5 to 54.4)

Cause name	Location	Year	Age group	Number (in thousands)	PAF	Rate (per 100,000)
Ischemic heart disease	Armenia	2015	Age-standardized		14.5% (10.8 to 18.2%)	37.9 (27.9 to 48.1)
Ischemic stroke	Armenia	1990	All Ages	0.2 (0.1 to 0.2)	10.0% (6.9 to 13.3%)	4.9 (3.2 to 6.7)
Ischemic stroke	Armenia	1995	All Ages	0.2 (0.1 to 0.3)	9.3% (6.0 to 12.6%)	6.1 (3.9 to 8.3)
Ischemic stroke	Armenia	2000	All Ages	0.2 (0.1 to 0.3)	9.6% (6.5 to 12.8%)	7.2 (4.8 to 9.7)
Ischemic stroke	Armenia	2005	All Ages	0.3 (0.2 to 0.3)	10.0% (6.7 to 13.3%)	8.3 (5.7 to 11.3)
Ischemic stroke	Armenia	2010	All Ages	0.2 (0.2 to 0.3)	10.7% (7.4 to 14.5%)	8.0 (5.4 to 10.8)
Ischemic stroke	Armenia	2015	All Ages	0.2 (0.1 to 0.3)	10.4% (6.8 to 14.5%)	7.8 (5.1 to 11.1)
Ischemic stroke	Armenia	1990	Age-standardized		9.6% (6.3 to 13.2%)	8.1 (5.3 to 11.3)
Ischemic stroke	Armenia	1995	Age-standardized		8.9% (5.6 to 12.4%)	8.3 (5.2 to 11.7)
Ischemic stroke	Armenia	2000	Age-standardized		9.2% (6.0 to 12.6%)	8.5 (5.5 to 11.9)
Ischemic stroke	Armenia	2005	Age-standardized		9.8% (6.6 to 13.3%)	8.3 (5.6 to 11.5)
Ischemic stroke	Armenia	2010	Age-standardized		10.7% (7.4 to 14.6%)	7.4 (5.1 to 10.0)
Ischemic stroke	Armenia	2015	Age-standardized		10.6% (7.1 to 14.5%)	6.3 (4.2 to 8.8)
Hemorrhagic stroke	Armenia	1990	All Ages	0.1 (0.1 to 0.2)	11.4% (8.4 to 14.5%)	4.2 (3.0 to 5.5)
Hemorrhagic stroke	Armenia	1995	All Ages	0.2 (0.1 to 0.2)	10.4% (7.4 to 13.4%)	5.2 (3.5 to 6.9)
Hemorrhagic stroke	Armenia	2000	All Ages	0.2 (0.1 to 0.2)	10.6% (7.6 to 13.8%)	5.5 (3.9 to 7.5)
Hemorrhagic stroke	Armenia	2005	All Ages	0.2 (0.1 to 0.3)	11.1% (8.0 to 14.2%)	6.3 (4.5 to 8.3)
Hemorrhagic stroke	Armenia	2010	All Ages	0.2 (0.1 to 0.2)	12.0% (9.1 to 15.3%)	6.0 (4.3 to 8.0)
Hemorrhagic stroke	Armenia	2015	All Ages	0.2 (0.1 to 0.2)	11.8% (8.4 to 15.2%)	5.7 (4.0 to 7.6)
Hemorrhagic stroke	Armenia	1990	Age-standardized		10.8% (7.8 to 13.7%)	6.1 (4.2 to 8.1)
Hemorrhagic stroke	Armenia	1995	Age-standardized		10.0% (7.0 to 12.9%)	6.3 (4.3 to 8.5)
Hemorrhagic stroke	Armenia	2000	Age-standardized		10.2% (7.4 to 13.3%)	6.1 (4.3 to 8.4)
Hemorrhagic stroke	Armenia	2005	Age-standardized		11.0% (8.0 to 13.9%)	6.2 (4.4 to 8.2)
Hemorrhagic stroke	Armenia	2010	Age-standardized		12.1% (9.0 to 15.3%)	5.6 (4.0 to 7.4)
Hemorrhagic stroke	Armenia	2015	Age-standardized		11.9% (8.7 to 15.2%)	4.7 (3.3 to 6.3)
Chronic obstructive pulmonary disease	Armenia	1990	All Ages	0.2 (0.1 to 0.3)	17.4% (8.5 to 28.1%)	5.7 (2.7 to 9.2)
Chronic obstructive pulmonary disease	Armenia	1995	All Ages	0.2 (0.1 to 0.4)	15.5% (6.6 to 25.8%)	6.3 (2.7 to 10.7)
Chronic obstructive pulmonary disease	Armenia	2000	All Ages	0.2 (0.1 to 0.3)	16.3% (7.5 to 26.4%)	6.4 (2.9 to 10.6)
Chronic obstructive pulmonary disease	Armenia	2005	All Ages	0.2 (0.1 to 0.4)	17.7% (8.3 to 28.3%)	7.3 (3.4 to 12.0)
Chronic obstructive pulmonary disease	Armenia	2010	All Ages	0.2 (0.1 to 0.4)	20.0% (10.1 to 31.4%)	7.5 (3.7 to 11.9)
Chronic obstructive pulmonary disease	Armenia	2015	All Ages	0.2 (0.1 to 0.3)	19.8% (10.0 to 30.0%)	7.4 (3.7 to 11.4)
Chronic obstructive pulmonary disease	Armenia	1990	Age-standardized		17.5% (8.5 to 28.2%)	9.2 (4.4 to 14.9)
Chronic obstructive pulmonary disease	Armenia	1995	Age-standardized		15.5% (6.6 to 25.8%)	8.3 (3.5 to 14.1)
Chronic obstructive pulmonary disease	Armenia	2000	Age-standardized		16.3% (7.5 to 26.4%)	7.5 (3.4 to 12.3)
Chronic obstructive pulmonary disease	Armenia	2005	Age-standardized		17.7% (8.3 to 28.3%)	7.4 (3.5 to 12.1)
Chronic obstructive pulmonary disease	Armenia	2010	Age-standardized		20.0% (10.1 to 31.4%)	7.0 (3.4 to 11.1)

Cause name	Location	Year	Age group	Number (in thousands)	PAF	Rate (per 100,000)
Chronic obstructive pulmonary disease	Armenia	2015	Age-standardized		19.8% (10.0 to 30.0%)	5.9 (3.0 to 9.1)
All causes	Azerbaijan	1990	All Ages	5.6 (4.7 to 6.5)	9.8% (8.3 to 11.4%)	77.7 (65.8 to 91.1)
All causes	Azerbaijan	1995	All Ages	5.5 (4.6 to 6.5)	9.1% (7.6 to 10.7%)	71.1 (59.6 to 84.2)
All causes	Azerbaijan	2000	All Ages	5.3 (4.5 to 6.2)	9.2% (7.8 to 10.7%)	65.1 (54.8 to 75.9)
All causes	Azerbaijan	2005	All Ages	5.1 (4.3 to 6.0)	8.5% (7.1 to 9.8%)	59.2 (49.7 to 68.7)
All causes	Azerbaijan	2010	All Ages	5.9 (5.0 to 6.8)	9.2% (7.9 to 10.5%)	64.1 (54.2 to 73.8)
All causes	Azerbaijan	2015	All Ages	6.1 (5.2 to 7.1)	9.6% (8.3 to 11.1%)	62.2 (53.0 to 72.6)
All causes	Azerbaijan	1990	Age-standardized		10.1% (8.4 to 11.8%)	134.8 (112.6 to 157.1)
All causes	Azerbaijan	1995	Age-standardized		9.5% (7.9 to 11.0%)	121.3 (102.1 to 141.8)
All causes	Azerbaijan	2000	Age-standardized		9.5% (7.9 to 11.1%)	116.2 (96.3 to 135.6)
All causes	Azerbaijan	2005	Age-standardized		8.7% (7.2 to 10.2%)	96.9 (80.3 to 113.0)
All causes	Azerbaijan	2010	Age-standardized		9.5% (8.0 to 11.0%)	97.4 (81.6 to 113.8)
All causes	Azerbaijan	2015	Age-standardized		9.8% (8.2 to 11.4%)	81.2 (67.7 to 96.0)
Lower respiratory infections	Azerbaijan	1990	All Ages	1.7 (1.1 to 2.6)	20.3% (12.6 to 29.5%)	24.3 (15.1 to 35.8)
Lower respiratory infections	Azerbaijan	1995	All Ages	1.5 (0.9 to 2.3)	18.1% (10.4 to 27.5%)	19.3 (11.0 to 30.0)
Lower respiratory infections	Azerbaijan	2000	All Ages	1.0 (0.6 to 1.6)	18.5% (10.3 to 28.2%)	12.3 (6.9 to 19.1)
Lower respiratory infections	Azerbaijan	2005	All Ages	0.7 (0.4 to 1.2)	17.0% (9.3 to 26.7%)	8.5 (4.6 to 13.4)
Lower respiratory infections	Azerbaijan	2010	All Ages	0.7 (0.4 to 1.1)	19.9% (12.2 to 29.2%)	7.8 (4.4 to 12.1)
Lower respiratory infections	Azerbaijan	2015	All Ages	0.6 (0.4 to 0.9)	22.1% (14.0 to 30.9%)	6.0 (3.7 to 8.7)
Lower respiratory infections	Azerbaijan	1990	Age-standardized		20.3% (12.6 to 29.5%)	18.9 (11.7 to 27.7)
Lower respiratory infections	Azerbaijan	1995	Age-standardized		18.1% (10.4 to 27.5%)	17.2 (9.9 to 26.7)
Lower respiratory infections	Azerbaijan	2000	Age-standardized		18.5% (10.3 to 28.2%)	14.0 (7.8 to 21.7)
Lower respiratory infections	Azerbaijan	2005	Age-standardized		17.0% (9.3 to 26.7%)	9.8 (5.2 to 15.6)
Lower respiratory infections	Azerbaijan	2010	Age-standardized		19.9% (12.2 to 29.2%)	8.3 (4.8 to 12.8)
Lower respiratory infections	Azerbaijan	2015	Age-standardized		22.1% (14.0 to 30.9%)	6.4 (4.0 to 9.4)
Tracheal, bronchus, and lung cancer	Azerbaijan	1990	All Ages	0.1 (0.1 to 0.2)	14.2% (8.3 to 20.9%)	2.1 (1.2 to 3.1)
Tracheal, bronchus, and lung cancer	Azerbaijan	1995	All Ages	0.1 (0.1 to 0.2)	12.6% (7.1 to 19.0%)	1.6 (0.9 to 2.4)
Tracheal, bronchus, and lung cancer	Azerbaijan	2000	All Ages	0.1 (0.1 to 0.2)	12.9% (7.3 to 19.5%)	1.5 (0.9 to 2.3)
Tracheal, bronchus, and lung cancer	Azerbaijan	2005	All Ages	0.1 (0.1 to 0.2)	11.8% (6.4 to 17.8%)	1.5 (0.8 to 2.2)
Tracheal, bronchus, and lung cancer	Azerbaijan	2010	All Ages	0.2 (0.1 to 0.3)	14.0% (8.0 to 21.1%)	2.0 (1.1 to 3.1)
Tracheal, bronchus, and lung cancer	Azerbaijan	2015	All Ages	0.3 (0.1 to 0.4)	15.7% (9.2 to 22.8%)	2.6 (1.5 to 3.9)
Tracheal, bronchus, and lung cancer	Azerbaijan	1990	Age-standardized		14.3% (8.3 to 21.0%)	3.3 (1.9 to 5.0)
Tracheal, bronchus, and lung cancer	Azerbaijan	1995	Age-standardized		12.6% (7.1 to 19.1%)	2.5 (1.4 to 3.8)
Tracheal, bronchus, and lung cancer	Azerbaijan	2000	Age-standardized		12.9% (7.3 to 19.5%)	2.3 (1.3 to 3.5)
Tracheal, bronchus, and lung cancer	Azerbaijan	2005	Age-standardized		11.8% (6.4 to 17.9%)	2.1 (1.1 to 3.2)
Tracheal, bronchus, and lung cancer	Azerbaijan	2010	Age-standardized		14.1% (8.1 to 21.2%)	2.8 (1.6 to 4.1)

Cause name	Location	Year	Age group	Number (in thousands)	PAF	Rate (per 100,000)
Tracheal, bronchus, and lung cancer	Azerbaijan	2015	Age-standardized		15.7% (9.2 to 22.8%)	2.9 (1.7 to 4.3)
Cardiovascular diseases	Azerbaijan	1990	All Ages	3.4 (2.8 to 3.9)	14.7% (12.5 to 17.0%)	46.8 (39.5 to 54.1)
Cardiovascular diseases	Azerbaijan	1995	All Ages	3.6 (3.0 to 4.2)	14.5% (12.3 to 16.7%)	46.1 (39.1 to 53.5)
Cardiovascular diseases	Azerbaijan	2000	All Ages	3.9 (3.3 to 4.6)	14.4% (12.2 to 16.8%)	47.7 (40.1 to 56.2)
Cardiovascular diseases	Azerbaijan	2005	All Ages	4.0 (3.4 to 4.7)	13.4% (11.4 to 15.6%)	46.3 (38.8 to 54.0)
Cardiovascular diseases	Azerbaijan	2010	All Ages	4.7 (3.9 to 5.5)	14.1% (11.8 to 16.5%)	50.7 (42.5 to 59.6)
Cardiovascular diseases	Azerbaijan	2015	All Ages	4.9 (4.1 to 5.8)	14.8% (12.4 to 17.5%)	50.0 (41.9 to 59.7)
Cardiovascular diseases	Azerbaijan	1990	Age-standardized		13.3% (10.7 to 15.9%)	101.8 (82.0 to 121.5)
Cardiovascular diseases	Azerbaijan	1995	Age-standardized		13.1% (10.8 to 15.6%)	92.4 (75.7 to 109.7)
Cardiovascular diseases	Azerbaijan	2000	Age-standardized		13.0% (10.7 to 15.7%)	92.3 (75.0 to 110.7)
Cardiovascular diseases	Azerbaijan	2005	Age-standardized		12.5% (10.2 to 14.8%)	79.6 (65.0 to 94.7)
Cardiovascular diseases	Azerbaijan	2010	Age-standardized		13.4% (11.0 to 15.8%)	80.3 (65.5 to 95.7)
Cardiovascular diseases	Azerbaijan	2015	Age-standardized		14.1% (11.5 to 16.8%)	66.8 (55.0 to 80.1)
Ischemic heart disease	Azerbaijan	1990	All Ages	2.6 (2.1 to 3.1)	16.5% (13.5 to 19.6%)	36.5 (29.9 to 43.5)
Ischemic heart disease	Azerbaijan	1995	All Ages	2.8 (2.3 to 3.4)	16.3% (13.4 to 19.2%)	36.6 (30.0 to 43.5)
Ischemic heart disease	Azerbaijan	2000	All Ages	3.1 (2.5 to 3.7)	16.2% (13.2 to 19.5%)	37.7 (30.7 to 45.4)
Ischemic heart disease	Azerbaijan	2005	All Ages	3.1 (2.5 to 3.7)	15.3% (12.6 to 18.3%)	36.0 (29.4 to 43.0)
Ischemic heart disease	Azerbaijan	2010	All Ages	3.6 (2.9 to 4.4)	15.9% (12.9 to 19.1%)	39.3 (31.5 to 47.2)
Ischemic heart disease	Azerbaijan	2015	All Ages	3.9 (3.1 to 4.7)	16.7% (13.5 to 20.0%)	39.4 (31.6 to 48.0)
Ischemic heart disease	Azerbaijan	1990	Age-standardized		14.8% (11.2 to 18.3%)	79.2 (60.0 to 98.3)
Ischemic heart disease	Azerbaijan	1995	Age-standardized		14.6% (11.4 to 17.7%)	73.3 (57.5 to 89.4)
Ischemic heart disease	Azerbaijan	2000	Age-standardized		14.5% (11.3 to 17.9%)	73.0 (56.7 to 90.1)
Ischemic heart disease	Azerbaijan	2005	Age-standardized		14.1% (11.1 to 17.2%)	61.7 (48.1 to 75.3)
Ischemic heart disease	Azerbaijan	2010	Age-standardized		15.0% (11.7 to 18.3%)	62.1 (48.1 to 76.1)
Ischemic heart disease	Azerbaijan	2015	Age-standardized		15.7% (12.4 to 19.4%)	52.4 (41.1 to 64.5)
Ischemic stroke	Azerbaijan	1990	All Ages	0.2 (0.1 to 0.3)	12.0% (9.1 to 14.8%)	2.8 (2.0 to 3.7)
Ischemic stroke	Azerbaijan	1995	All Ages	0.2 (0.1 to 0.3)	11.6% (8.6 to 14.5%)	2.5 (1.8 to 3.2)
Ischemic stroke	Azerbaijan	2000	All Ages	0.2 (0.2 to 0.3)	11.7% (8.6 to 14.7%)	2.8 (2.0 to 3.6)
Ischemic stroke	Azerbaijan	2005	All Ages	0.3 (0.2 to 0.3)	10.9% (7.8 to 14.0%)	2.9 (2.0 to 3.8)
Ischemic stroke	Azerbaijan	2010	All Ages	0.3 (0.2 to 0.4)	11.6% (8.4 to 14.7%)	3.2 (2.3 to 4.2)
Ischemic stroke	Azerbaijan	2015	All Ages	0.3 (0.2 to 0.4)	12.2% (9.0 to 15.3%)	3.0 (2.1 to 3.9)
Ischemic stroke	Azerbaijan	1990	Age-standardized		10.9% (7.5 to 14.7%)	6.7 (4.4 to 9.4)
Ischemic stroke	Azerbaijan	1995	Age-standardized		10.6% (7.5 to 13.8%)	5.6 (3.8 to 7.6)
Ischemic stroke	Azerbaijan	2000	Age-standardized		10.7% (7.5 to 13.9%)	5.9 (3.9 to 8.0)
Ischemic stroke	Azerbaijan	2005	Age-standardized		10.2% (7.1 to 13.4%)	5.4 (3.7 to 7.4)
Ischemic stroke	Azerbaijan	2010	Age-standardized		11.1% (7.8 to 14.5%)	5.4 (3.7 to 7.3)

Cause name	Location	Year	Age group	Number (in thousands)	PAF	Rate (per 100,000)
Ischemic stroke	Azerbaijan	2015	Age-standardized		11.8% (8.5 to 15.0%)	4.2 (2.9 to 5.6)
Hemorrhagic stroke	Azerbaijan	1990	All Ages	0.5 (0.4 to 0.7)	12.9% (10.2 to 15.9%)	7.6 (5.8 to 9.4)
Hemorrhagic stroke	Azerbaijan	1995	All Ages	0.5 (0.4 to 0.7)	12.5% (9.6 to 15.4%)	7.0 (5.3 to 8.7)
Hemorrhagic stroke	Azerbaijan	2000	All Ages	0.6 (0.4 to 0.8)	12.4% (9.4 to 15.5%)	7.2 (5.4 to 9.2)
Hemorrhagic stroke	Azerbaijan	2005	All Ages	0.6 (0.5 to 0.8)	11.6% (8.7 to 14.7%)	7.4 (5.6 to 9.5)
Hemorrhagic stroke	Azerbaijan	2010	All Ages	0.8 (0.6 to 1.0)	12.4% (9.5 to 15.5%)	8.2 (6.2 to 10.3)
Hemorrhagic stroke	Azerbaijan	2015	All Ages	0.8 (0.6 to 1.0)	13.2% (10.4 to 16.2%)	7.7 (5.9 to 9.7)
Hemorrhagic stroke	Azerbaijan	1990	Age-standardized		11.7% (8.7 to 14.9%)	15.9 (11.5 to 20.7)
Hemorrhagic stroke	Azerbaijan	1995	Age-standardized		11.4% (8.6 to 14.3%)	13.5 (10.0 to 17.0)
Hemorrhagic stroke	Azerbaijan	2000	Age-standardized		11.3% (8.4 to 14.2%)	13.4 (9.9 to 17.1)
Hemorrhagic stroke	Azerbaijan	2005	Age-standardized		10.8% (7.9 to 13.7%)	12.5 (9.1 to 16.1)
Hemorrhagic stroke	Azerbaijan	2010	Age-standardized		11.8% (8.9 to 14.8%)	12.8 (9.4 to 16.3)
Hemorrhagic stroke	Azerbaijan	2015	Age-standardized		12.5% (9.5 to 15.6%)	10.2 (7.6 to 13.0)
Chronic obstructive pulmonary disease	Azerbaijan	1990	All Ages	0.3 (0.2 to 0.5)	20.2% (10.3 to 30.6%)	4.5 (2.3 to 6.8)
Chronic obstructive pulmonary disease	Azerbaijan	1995	All Ages	0.3 (0.2 to 0.5)	18.4% (9.0 to 28.6%)	4.2 (2.0 to 6.5)
Chronic obstructive pulmonary disease	Azerbaijan	2000	All Ages	0.3 (0.1 to 0.5)	18.7% (9.4 to 29.0%)	3.6 (1.8 to 5.7)
Chronic obstructive pulmonary disease	Azerbaijan	2005	All Ages	0.3 (0.1 to 0.4)	17.6% (8.5 to 28.1%)	3.0 (1.4 to 4.8)
Chronic obstructive pulmonary disease	Azerbaijan	2010	All Ages	0.3 (0.2 to 0.5)	20.1% (10.6 to 30.4%)	3.6 (1.9 to 5.5)
Chronic obstructive pulmonary disease	Azerbaijan	2015	All Ages	0.4 (0.2 to 0.5)	21.8% (11.8 to 32.6%)	3.6 (1.9 to 5.4)
Chronic obstructive pulmonary disease	Azerbaijan	1990	Age-standardized		20.5% (10.4 to 31.1%)	10.8 (5.4 to 16.4)
Chronic obstructive pulmonary disease	Azerbaijan	1995	Age-standardized		18.7% (9.2 to 29.1%)	9.2 (4.4 to 14.5)
Chronic obstructive pulmonary disease	Azerbaijan	2000	Age-standardized		19.0% (9.5 to 29.5%)	7.5 (3.8 to 11.9)
Chronic obstructive pulmonary disease	Azerbaijan	2005	Age-standardized		17.8% (8.6 to 28.4%)	5.5 (2.6 to 8.8)
Chronic obstructive pulmonary disease	Azerbaijan	2010	Age-standardized		20.3% (10.6 to 30.6%)	6.0 (3.1 to 9.3)
Chronic obstructive pulmonary disease	Azerbaijan	2015	Age-standardized		21.9% (11.8 to 32.7%)	5.1 (2.7 to 7.8)
All causes	Georgia	1990	All Ages	4.7 (4.0 to 5.5)	8.8% (7.4 to 10.3%)	85.8 (72.3 to 100.5)
All causes	Georgia	1995	All Ages	4.0 (3.3 to 4.7)	8.2% (6.7 to 9.6%)	78.6 (64.1 to 93.3)
All causes	Georgia	2000	All Ages	4.0 (3.3 to 4.8)	8.3% (6.8 to 9.8%)	84.6 (68.6 to 99.9)
All causes	Georgia	2005	All Ages	4.1 (3.4 to 4.8)	8.1% (6.7 to 9.5%)	91.0 (75.0 to 107.2)
All causes	Georgia	2010	All Ages	4.2 (3.5 to 5.0)	8.1% (6.8 to 9.6%)	98.5 (81.7 to 118.2)
All causes	Georgia	2015	All Ages	3.9 (3.2 to 4.7)	7.8% (6.4 to 9.2%)	97.7 (79.8 to 116.2)
All causes	Georgia	1990	Age-standardized		8.7% (7.3 to 10.3%)	88.8 (73.7 to 105.4)
All causes	Georgia	1995	Age-standardized		8.0% (6.5 to 9.4%)	77.3 (62.5 to 92.7)
All causes	Georgia	2000	Age-standardized		8.1% (6.5 to 9.6%)	78.1 (62.9 to 93.5)
All causes	Georgia	2005	Age-standardized		7.9% (6.5 to 9.2%)	74.4 (60.5 to 87.7)
All causes	Georgia	2010	Age-standardized		7.9% (6.7 to 9.3%)	71.5 (59.9 to 85.2)

Cause name	Location	Year	Age group	Number (in thousands)	PAF	Rate (per 100,000)
All causes	Georgia	2015	Age-standardized		7.6% (6.3 to 8.8%)	67.6 (56.0 to 79.4)
Lower respiratory infections	Georgia	1990	All Ages	0.3 (0.1 to 0.5)	15.5% (7.4 to 25.8%)	5.7 (2.7 to 9.7)
Lower respiratory infections	Georgia	1995	All Ages	0.2 (0.1 to 0.4)	13.0% (5.2 to 23.8%)	3.8 (1.5 to 7.0)
Lower respiratory infections	Georgia	2000	All Ages	0.1 (0.1 to 0.3)	13.5% (5.4 to 24.1%)	3.0 (1.2 to 5.5)
Lower respiratory infections	Georgia	2005	All Ages	0.1 (0.1 to 0.2)	14.1% (6.3 to 24.7%)	2.4 (1.1 to 4.4)
Lower respiratory infections	Georgia	2010	All Ages	0.1 (0.1 to 0.2)	16.8% (8.4 to 27.0%)	2.8 (1.4 to 4.7)
Lower respiratory infections	Georgia	2015	All Ages	0.1 (0.1 to 0.2)	16.9% (8.6 to 27.1%)	2.7 (1.4 to 4.6)
Lower respiratory infections	Georgia	1990	Age-standardized		15.5% (7.4 to 25.8%)	6.1 (2.9 to 10.4)
Lower respiratory infections	Georgia	1995	Age-standardized		13.0% (5.2 to 23.8%)	4.4 (1.7 to 8.1)
Lower respiratory infections	Georgia	2000	Age-standardized		13.5% (5.4 to 24.1%)	3.7 (1.5 to 6.8)
Lower respiratory infections	Georgia	2005	Age-standardized		14.1% (6.3 to 24.7%)	2.6 (1.1 to 4.7)
Lower respiratory infections	Georgia	2010	Age-standardized		16.8% (8.4 to 27.0%)	2.4 (1.2 to 4.0)
Lower respiratory infections	Georgia	2015	Age-standardized		16.9% (8.6 to 27.1%)	2.2 (1.1 to 3.6)
Tracheal, bronchus, and lung cancer	Georgia	1990	All Ages	0.1 (0.1 to 0.2)	10.8% (5.8 to 16.8%)	2.5 (1.3 to 3.9)
Tracheal, bronchus, and lung cancer	Georgia	1995	All Ages	0.1 (0.0 to 0.1)	9.0% (4.5 to 14.4%)	1.7 (0.8 to 2.8)
Tracheal, bronchus, and lung cancer	Georgia	2000	All Ages	0.1 (0.0 to 0.1)	9.3% (4.7 to 15.2%)	1.9 (0.9 to 3.1)
Tracheal, bronchus, and lung cancer	Georgia	2005	All Ages	0.1 (0.1 to 0.2)	9.8% (5.0 to 15.6%)	2.4 (1.2 to 3.8)
Tracheal, bronchus, and lung cancer	Georgia	2010	All Ages	0.1 (0.1 to 0.2)	11.7% (6.3 to 18.2%)	3.5 (1.8 to 5.5)
Tracheal, bronchus, and lung cancer	Georgia	2015	All Ages	0.2 (0.1 to 0.2)	11.8% (6.6 to 18.4%)	3.9 (2.2 to 6.0)
Tracheal, bronchus, and lung cancer	Georgia	1990	Age-standardized		10.8% (5.8 to 16.8%)	2.3 (1.2 to 3.6)
Tracheal, bronchus, and lung cancer	Georgia	1995	Age-standardized		9.0% (4.5 to 14.4%)	1.5 (0.7 to 2.4)
Tracheal, bronchus, and lung cancer	Georgia	2000	Age-standardized		9.3% (4.7 to 15.2%)	1.6 (0.8 to 2.5)
Tracheal, bronchus, and lung cancer	Georgia	2005	Age-standardized		9.8% (5.0 to 15.5%)	1.9 (1.0 to 3.0)
Tracheal, bronchus, and lung cancer	Georgia	2010	Age-standardized		11.7% (6.3 to 18.1%)	2.7 (1.4 to 4.3)
Tracheal, bronchus, and lung cancer	Georgia	2015	Age-standardized		11.8% (6.6 to 18.4%)	2.9 (1.6 to 4.5)
Cardiovascular diseases	Georgia	1990	All Ages	4.1 (3.4 to 4.9)	12.4% (10.3 to 14.6%)	74.8 (62.0 to 88.7)
Cardiovascular diseases	Georgia	1995	All Ages	3.6 (2.9 to 4.3)	11.4% (9.3 to 13.6%)	71.1 (57.2 to 85.3)
Cardiovascular diseases	Georgia	2000	All Ages	3.7 (2.9 to 4.4)	11.4% (9.3 to 13.5%)	76.7 (61.4 to 91.4)
Cardiovascular diseases	Georgia	2005	All Ages	3.6 (2.9 to 4.3)	11.1% (9.0 to 13.0%)	79.5 (64.6 to 94.8)
Cardiovascular diseases	Georgia	2010	All Ages	3.6 (2.9 to 4.4)	11.3% (9.3 to 13.6%)	83.9 (68.0 to 102.0)
Cardiovascular diseases	Georgia	2015	All Ages	3.4 (2.7 to 4.0)	10.8% (8.7 to 13.0%)	83.5 (66.7 to 101.2)
Cardiovascular diseases	Georgia	1990	Age-standardized		12.1% (9.9 to 14.4%)	77.3 (63.3 to 92.5)
Cardiovascular diseases	Georgia	1995	Age-standardized		11.1% (8.9 to 13.3%)	69.3 (55.5 to 83.9)
Cardiovascular diseases	Georgia	2000	Age-standardized		11.1% (8.9 to 13.3%)	70.0 (55.7 to 84.3)
Cardiovascular diseases	Georgia	2005	Age-standardized		11.0% (8.9 to 13.0%)	64.6 (51.7 to 77.2)
Cardiovascular diseases	Georgia	2010	Age-standardized		11.6% (9.6 to 13.8%)	60.6 (49.5 to 72.7)

Cause name	Location	Year	Age group	Number (in thousands)	PAF	Rate (per 100,000)
Cardiovascular diseases	Georgia	2015	Age-standardized		11.2% (9.1 to 13.2%)	57.4 (46.4 to 68.3)
Ischemic heart disease	Georgia	1990	All Ages	2.9 (2.3 to 3.6)	14.3% (11.2 to 17.4%)	53.2 (41.6 to 64.9)
Ischemic heart disease	Georgia	1995	All Ages	2.6 (2.0 to 3.2)	13.2% (10.2 to 16.0%)	51.3 (39.4 to 63.0)
Ischemic heart disease	Georgia	2000	All Ages	2.6 (2.0 to 3.2)	13.3% (10.3 to 16.3%)	54.0 (41.3 to 66.5)
Ischemic heart disease	Georgia	2005	All Ages	2.4 (1.9 to 3.0)	13.4% (10.4 to 16.4%)	54.0 (42.0 to 66.3)
Ischemic heart disease	Georgia	2010	All Ages	2.4 (1.8 to 3.0)	14.1% (10.9 to 17.5%)	56.6 (42.7 to 70.7)
Ischemic heart disease	Georgia	2015	All Ages	2.3 (1.7 to 2.9)	13.9% (10.4 to 17.4%)	56.8 (42.5 to 71.3)
Ischemic heart disease	Georgia	1990	Age-standardized		13.9% (10.7 to 17.1%)	55.0 (42.5 to 67.9)
Ischemic heart disease	Georgia	1995	Age-standardized		12.9% (9.8 to 15.8%)	50.1 (37.6 to 62.1)
Ischemic heart disease	Georgia	2000	Age-standardized		12.9% (9.7 to 16.0%)	49.4 (37.0 to 61.7)
Ischemic heart disease	Georgia	2005	Age-standardized		13.3% (10.2 to 16.4%)	44.1 (33.8 to 54.1)
Ischemic heart disease	Georgia	2010	Age-standardized		14.5% (11.3 to 17.7%)	41.0 (31.7 to 50.9)
Ischemic heart disease	Georgia	2015	Age-standardized		14.3% (11.1 to 17.6%)	39.2 (30.0 to 48.3)
Ischemic stroke	Georgia	1990	All Ages	0.4 (0.3 to 0.6)	10.0% (7.0 to 13.2%)	7.8 (5.3 to 10.5)
Ischemic stroke	Georgia	1995	All Ages	0.4 (0.3 to 0.5)	9.1% (6.0 to 12.3%)	7.4 (4.9 to 10.1)
Ischemic stroke	Georgia	2000	All Ages	0.4 (0.3 to 0.6)	9.3% (6.2 to 12.5%)	8.9 (5.9 to 12.1)
Ischemic stroke	Georgia	2005	All Ages	0.4 (0.3 to 0.6)	9.4% (6.3 to 12.6%)	9.0 (5.9 to 12.2)
Ischemic stroke	Georgia	2010	All Ages	0.4 (0.3 to 0.6)	10.1% (6.8 to 13.6%)	9.6 (6.3 to 13.4)
Ischemic stroke	Georgia	2015	All Ages	0.4 (0.3 to 0.6)	10.0% (6.7 to 13.6%)	10.8 (7.1 to 15.3)
Ischemic stroke	Georgia	1990	Age-standardized		9.8% (6.6 to 13.2%)	8.3 (5.4 to 11.3)
Ischemic stroke	Georgia	1995	Age-standardized		8.9% (5.7 to 12.2%)	7.3 (4.7 to 10.3)
Ischemic stroke	Georgia	2000	Age-standardized		9.0% (5.8 to 12.4%)	8.1 (5.2 to 11.2)
Ischemic stroke	Georgia	2005	Age-standardized		9.3% (6.2 to 12.7%)	7.1 (4.7 to 9.8)
Ischemic stroke	Georgia	2010	Age-standardized		10.3% (7.2 to 13.5%)	6.8 (4.6 to 9.2)
Ischemic stroke	Georgia	2015	Age-standardized		10.2% (7.0 to 13.6%)	7.2 (4.8 to 9.9)
Hemorrhagic stroke	Georgia	1990	All Ages	0.8 (0.6 to 1.0)	10.9% (8.1 to 14.0%)	13.8 (10.2 to 17.6)
Hemorrhagic stroke	Georgia	1995	All Ages	0.6 (0.5 to 0.8)	9.9% (7.0 to 12.8%)	12.4 (8.8 to 16.4)
Hemorrhagic stroke	Georgia	2000	All Ages	0.7 (0.5 to 0.9)	10.0% (7.1 to 13.0%)	13.9 (9.8 to 18.2)
Hemorrhagic stroke	Georgia	2005	All Ages	0.8 (0.5 to 1.0)	10.0% (7.1 to 13.0%)	16.5 (11.9 to 21.5)
Hemorrhagic stroke	Georgia	2010	All Ages	0.8 (0.6 to 1.0)	10.8% (7.7 to 14.0%)	17.7 (12.8 to 23.0)
Hemorrhagic stroke	Georgia	2015	All Ages	0.6 (0.5 to 0.8)	10.7% (7.7 to 13.9%)	15.9 (11.3 to 21.1)
Hemorrhagic stroke	Georgia	1990	Age-standardized		10.6% (7.8 to 13.7%)	14.1 (10.3 to 18.4)
Hemorrhagic stroke	Georgia	1995	Age-standardized		9.6% (6.9 to 12.6%)	11.9 (8.4 to 15.7)
Hemorrhagic stroke	Georgia	2000	Age-standardized		9.7% (6.9 to 12.8%)	12.4 (8.7 to 16.4)
Hemorrhagic stroke	Georgia	2005	Age-standardized		10.0% (7.1 to 13.0%)	13.3 (9.7 to 17.5)
Hemorrhagic stroke	Georgia	2010	Age-standardized		11.1% (8.2 to 14.0%)	12.8 (9.4 to 16.5)

Cause name	Location	Year	Age group	Number (in thousands)	PAF	Rate (per 100,000)
Hemorrhagic stroke	Georgia	2015	Age-standardized		11.0% (8.1 to 14.2%)	11.0 (8.0 to 14.3)
Chronic obstructive pulmonary disease	Georgia	1990	All Ages	0.2 (0.1 to 0.3)	16.8% (7.8 to 27.3%)	2.9 (1.3 to 4.8)
Chronic obstructive pulmonary disease	Georgia	1995	All Ages	0.1 (0.1 to 0.2)	14.7% (6.4 to 24.7%)	2.1 (0.9 to 3.6)
Chronic obstructive pulmonary disease	Georgia	2000	All Ages	0.1 (0.1 to 0.2)	15.1% (6.5 to 25.4%)	3.0 (1.3 to 5.1)
Chronic obstructive pulmonary disease	Georgia	2005	All Ages	0.3 (0.1 to 0.5)	15.7% (6.9 to 26.0%)	6.7 (2.9 to 11.3)
Chronic obstructive pulmonary disease	Georgia	2010	All Ages	0.4 (0.2 to 0.6)	17.9% (8.5 to 28.5%)	8.3 (4.0 to 13.2)
Chronic obstructive pulmonary disease	Georgia	2015	All Ages	0.3 (0.1 to 0.5)	18.0% (8.5 to 28.8%)	7.5 (3.5 to 12.1)
Chronic obstructive pulmonary disease	Georgia	1990	Age-standardized		16.8% (7.7 to 27.3%)	3.1 (1.4 to 5.0)
Chronic obstructive pulmonary disease	Georgia	1995	Age-standardized		14.6% (6.4 to 24.7%)	2.1 (0.9 to 3.6)
Chronic obstructive pulmonary disease	Georgia	2000	Age-standardized		15.0% (6.5 to 25.3%)	2.8 (1.2 to 4.7)
Chronic obstructive pulmonary disease	Georgia	2005	Age-standardized		15.6% (6.8 to 25.9%)	5.4 (2.3 to 9.1)
Chronic obstructive pulmonary disease	Georgia	2010	Age-standardized		17.8% (8.5 to 28.3%)	5.8 (2.8 to 9.3)
Chronic obstructive pulmonary disease	Georgia	2015	Age-standardized		17.9% (8.5 to 28.6%)	5.0 (2.4 to 8.1)
All causes	Kazakhstan	1990	All Ages	9.8 (8.4 to 11.4)	7.2% (6.1 to 8.4%)	59.2 (50.5 to 68.6)
All causes	Kazakhstan	1995	All Ages	11.5 (9.7 to 13.4)	6.6% (5.5 to 7.6%)	71.4 (60.1 to 83.1)
All causes	Kazakhstan	2000	All Ages	10.4 (8.8 to 12.0)	6.7% (5.7 to 7.8%)	68.9 (58.4 to 79.8)
All causes	Kazakhstan	2005	All Ages	10.5 (8.9 to 12.2)	6.4% (5.5 to 7.5%)	67.7 (57.7 to 78.6)
All causes	Kazakhstan	2010	All Ages	9.4 (7.9 to 10.9)	6.5% (5.5 to 7.5%)	57.4 (48.3 to 66.3)
All causes	Kazakhstan	2015	All Ages	10.1 (8.5 to 11.8)	7.0% (5.9 to 8.0%)	57.7 (48.5 to 67.2)
All causes	Kazakhstan	1990	Age-standardized		7.7% (6.4 to 9.0%)	88.1 (73.5 to 103.1)
All causes	Kazakhstan	1995	Age-standardized		6.9% (5.7 to 8.0%)	99.1 (82.4 to 116.4)
All causes	Kazakhstan	2000	Age-standardized		7.0% (5.8 to 8.2%)	96.6 (80.4 to 113.5)
All causes	Kazakhstan	2005	Age-standardized		6.7% (5.5 to 7.9%)	91.2 (75.4 to 107.4)
All causes	Kazakhstan	2010	Age-standardized		6.8% (5.6 to 8.0%)	77.7 (64.1 to 91.3)
All causes	Kazakhstan	2015	Age-standardized		7.2% (6.0 to 8.5%)	74.9 (61.3 to 88.4)
Lower respiratory infections	Kazakhstan	1990	All Ages	1.0 (0.6 to 1.7)	16.2% (8.9 to 25.8%)	6.3 (3.5 to 9.9)
Lower respiratory infections	Kazakhstan	1995	All Ages	0.8 (0.4 to 1.4)	13.4% (6.5 to 23.1%)	4.9 (2.4 to 8.4)
Lower respiratory infections	Kazakhstan	2000	All Ages	0.6 (0.3 to 1.0)	13.5% (6.6 to 23.0%)	3.9 (1.9 to 6.8)
Lower respiratory infections	Kazakhstan	2005	All Ages	0.5 (0.2 to 0.9)	12.4% (5.7 to 22.2%)	3.3 (1.5 to 6.0)
Lower respiratory infections	Kazakhstan	2010	All Ages	0.5 (0.2 to 0.8)	13.8% (6.9 to 23.3%)	2.9 (1.5 to 5.0)
Lower respiratory infections	Kazakhstan	2015	All Ages	0.5 (0.3 to 0.8)	15.7% (8.5 to 24.9%)	2.8 (1.5 to 4.6)
Lower respiratory infections	Kazakhstan	1990	Age-standardized		16.2% (8.9 to 25.8%)	5.9 (3.3 to 9.3)
Lower respiratory infections	Kazakhstan	1995	Age-standardized		13.4% (6.5 to 23.1%)	5.2 (2.5 to 9.0)
Lower respiratory infections	Kazakhstan	2000	Age-standardized		13.5% (6.6 to 23.0%)	4.6 (2.3 to 8.0)
Lower respiratory infections	Kazakhstan	2005	Age-standardized		12.4% (5.7 to 22.2%)	3.7 (1.6 to 6.7)
Lower respiratory infections	Kazakhstan	2010	Age-standardized		13.8% (6.9 to 23.3%)	3.1 (1.6 to 5.3)

Cause name	Location	Year	Age group	Number (in thousands)	PAF	Rate (per 100,000)
Lower respiratory infections	Kazakhstan	2015	Age-standardized		15.7% (8.5 to 24.9%)	3.0 (1.6 to 4.9)
Tracheal, bronchus, and lung cancer	Kazakhstan	1990	All Ages	0.5 (0.3 to 0.8)	11.3% (6.2 to 17.4%)	3.3 (1.8 to 5.1)
Tracheal, bronchus, and lung cancer	Kazakhstan	1995	All Ages	0.5 (0.2 to 0.8)	9.2% (4.7 to 14.6%)	3.0 (1.5 to 4.7)
Tracheal, bronchus, and lung cancer	Kazakhstan	2000	All Ages	0.4 (0.2 to 0.6)	9.3% (4.9 to 14.7%)	2.5 (1.3 to 3.9)
Tracheal, bronchus, and lung cancer	Kazakhstan	2005	All Ages	0.3 (0.2 to 0.6)	8.5% (4.3 to 13.8%)	2.2 (1.1 to 3.6)
Tracheal, bronchus, and lung cancer	Kazakhstan	2010	All Ages	0.3 (0.2 to 0.5)	9.5% (5.0 to 14.9%)	2.1 (1.1 to 3.3)
Tracheal, bronchus, and lung cancer	Kazakhstan	2015	All Ages	0.4 (0.2 to 0.7)	10.9% (6.1 to 16.9%)	2.5 (1.3 to 3.8)
Tracheal, bronchus, and lung cancer	Kazakhstan	1990	Age-standardized		11.3% (6.2 to 17.4%)	4.4 (2.4 to 6.8)
Tracheal, bronchus, and lung cancer	Kazakhstan	1995	Age-standardized		9.2% (4.7 to 14.6%)	3.8 (1.9 to 6.0)
Tracheal, bronchus, and lung cancer	Kazakhstan	2000	Age-standardized		9.3% (4.9 to 14.7%)	3.1 (1.6 to 4.9)
Tracheal, bronchus, and lung cancer	Kazakhstan	2005	Age-standardized		8.5% (4.3 to 13.8%)	2.7 (1.4 to 4.4)
Tracheal, bronchus, and lung cancer	Kazakhstan	2010	Age-standardized		9.5% (5.0 to 14.9%)	2.7 (1.4 to 4.2)
Tracheal, bronchus, and lung cancer	Kazakhstan	2015	Age-standardized		10.9% (6.1 to 16.9%)	3.0 (1.6 to 4.5)
Cardiovascular diseases	Kazakhstan	1990	All Ages	7.2 (6.0 to 8.5)	11.9% (9.8 to 14.0%)	43.6 (36.1 to 51.4)
Cardiovascular diseases	Kazakhstan	1995	All Ages	9.2 (7.6 to 10.8)	10.9% (9.1 to 12.8%)	57.1 (47.4 to 66.9)
Cardiovascular diseases	Kazakhstan	2000	All Ages	8.6 (7.2 to 10.1)	11.0% (9.2 to 12.8%)	57.1 (47.7 to 66.7)
Cardiovascular diseases	Kazakhstan	2005	All Ages	8.8 (7.5 to 10.4)	10.7% (9.0 to 12.5%)	57.2 (48.2 to 67.1)
Cardiovascular diseases	Kazakhstan	2010	All Ages	7.8 (6.4 to 9.1)	10.9% (9.0 to 12.7%)	47.4 (39.4 to 55.6)
Cardiovascular diseases	Kazakhstan	2015	All Ages	8.2 (6.8 to 9.6)	11.6% (9.7 to 13.6%)	46.8 (38.7 to 54.9)
Cardiovascular diseases	Kazakhstan	1990	Age-standardized		11.4% (9.3 to 13.6%)	68.0 (54.8 to 81.2)
Cardiovascular diseases	Kazakhstan	1995	Age-standardized		10.5% (8.6 to 12.4%)	80.8 (65.6 to 95.3)
Cardiovascular diseases	Kazakhstan	2000	Age-standardized		10.4% (8.5 to 12.4%)	81.2 (66.6 to 96.3)
Cardiovascular diseases	Kazakhstan	2005	Age-standardized		10.2% (8.3 to 12.1%)	77.9 (63.3 to 93.0)
Cardiovascular diseases	Kazakhstan	2010	Age-standardized		10.4% (8.4 to 12.4%)	65.1 (52.7 to 77.4)
Cardiovascular diseases	Kazakhstan	2015	Age-standardized		11.0% (9.0 to 13.1%)	61.6 (49.4 to 73.4)
Ischemic heart disease	Kazakhstan	1990	All Ages	5.0 (4.0 to 6.1)	14.3% (11.2 to 17.3%)	30.2 (23.8 to 36.6)
Ischemic heart disease	Kazakhstan	1995	All Ages	6.6 (5.3 to 7.9)	13.3% (10.6 to 16.0%)	41.0 (32.7 to 49.1)
Ischemic heart disease	Kazakhstan	2000	All Ages	6.3 (4.9 to 7.5)	13.4% (10.6 to 16.1%)	41.4 (32.6 to 49.9)
Ischemic heart disease	Kazakhstan	2005	All Ages	6.5 (5.3 to 7.9)	13.1% (10.6 to 15.7%)	42.4 (34.2 to 51.3)
Ischemic heart disease	Kazakhstan	2010	All Ages	5.6 (4.4 to 6.7)	13.4% (10.5 to 16.0%)	34.2 (27.0 to 41.2)
Ischemic heart disease	Kazakhstan	2015	All Ages	6.0 (4.7 to 7.3)	14.1% (11.2 to 17.1%)	33.9 (26.7 to 41.3)
Ischemic heart disease	Kazakhstan	1990	Age-standardized		13.6% (10.3 to 16.7%)	46.9 (35.8 to 58.2)
Ischemic heart disease	Kazakhstan	1995	Age-standardized		12.7% (9.8 to 15.4%)	57.8 (44.7 to 70.1)
Ischemic heart disease	Kazakhstan	2000	Age-standardized		12.6% (9.6 to 15.3%)	58.9 (45.1 to 72.1)
Ischemic heart disease	Kazakhstan	2005	Age-standardized		12.3% (9.3 to 15.1%)	57.8 (44.3 to 71.3)
Ischemic heart disease	Kazakhstan	2010	Age-standardized		12.6% (9.6 to 15.5%)	46.9 (35.8 to 58.0)

Cause name	Location	Year	Age group	Number (in thousands)	PAF	Rate (per 100,000)
Ischemic heart disease	Kazakhstan	2015	Age-standardized		13.3% (10.2 to 16.4%)	44.7 (33.8 to 55.7)
Ischemic stroke	Kazakhstan	1990	All Ages	1.3 (0.9 to 1.7)	10.0% (7.1 to 13.2%)	7.7 (5.5 to 10.2)
Ischemic stroke	Kazakhstan	1995	All Ages	1.4 (0.9 to 1.8)	9.0% (6.2 to 12.1%)	8.4 (5.8 to 11.3)
Ischemic stroke	Kazakhstan	2000	All Ages	1.4 (1.0 to 1.8)	9.3% (6.5 to 12.0%)	9.3 (6.5 to 12.1)
Ischemic stroke	Kazakhstan	2005	All Ages	1.4 (1.0 to 1.8)	9.1% (6.4 to 11.8%)	8.9 (6.4 to 11.7)
Ischemic stroke	Kazakhstan	2010	All Ages	1.3 (0.9 to 1.7)	9.4% (6.7 to 12.2%)	7.8 (5.5 to 10.2)
Ischemic stroke	Kazakhstan	2015	All Ages	1.3 (1.0 to 1.7)	10.1% (7.3 to 13.0%)	7.5 (5.4 to 9.8)
Ischemic stroke	Kazakhstan	1990	Age-standardized		9.7% (6.6 to 13.2%)	12.7 (8.7 to 17.4)
Ischemic stroke	Kazakhstan	1995	Age-standardized		8.7% (5.9 to 11.9%)	12.6 (8.5 to 17.6)
Ischemic stroke	Kazakhstan	2000	Age-standardized		8.8% (6.0 to 12.0%)	13.9 (9.4 to 19.0)
Ischemic stroke	Kazakhstan	2005	Age-standardized		8.6% (5.9 to 11.6%)	12.7 (8.7 to 17.1)
Ischemic stroke	Kazakhstan	2010	Age-standardized		9.0% (6.1 to 12.0%)	11.3 (7.7 to 15.2)
Ischemic stroke	Kazakhstan	2015	Age-standardized		9.6% (6.7 to 12.8%)	10.3 (7.2 to 14.1)
Hemorrhagic stroke	Kazakhstan	1990	All Ages	0.9 (0.7 to 1.2)	11.4% (8.7 to 14.1%)	5.6 (4.3 to 7.1)
Hemorrhagic stroke	Kazakhstan	1995	All Ages	1.2 (0.9 to 1.6)	10.3% (7.7 to 13.0%)	7.7 (5.7 to 9.8)
Hemorrhagic stroke	Kazakhstan	2000	All Ages	1.0 (0.7 to 1.2)	10.5% (7.9 to 13.4%)	6.3 (4.7 to 8.0)
Hemorrhagic stroke	Kazakhstan	2005	All Ages	0.9 (0.7 to 1.2)	10.3% (7.8 to 13.2%)	5.9 (4.5 to 7.6)
Hemorrhagic stroke	Kazakhstan	2010	All Ages	0.9 (0.7 to 1.1)	10.7% (8.2 to 13.5%)	5.5 (4.1 to 7.0)
Hemorrhagic stroke	Kazakhstan	2015	All Ages	1.0 (0.7 to 1.2)	11.5% (8.9 to 14.2%)	5.4 (4.1 to 7.0)
Hemorrhagic stroke	Kazakhstan	1990	Age-standardized		10.9% (8.2 to 13.8%)	8.4 (6.3 to 10.6)
Hemorrhagic stroke	Kazakhstan	1995	Age-standardized		9.9% (7.4 to 12.6%)	10.4 (7.7 to 13.3)
Hemorrhagic stroke	Kazakhstan	2000	Age-standardized		10.0% (7.5 to 12.7%)	8.4 (6.2 to 10.7)
Hemorrhagic stroke	Kazakhstan	2005	Age-standardized		9.8% (7.2 to 12.5%)	7.5 (5.5 to 9.6)
Hemorrhagic stroke	Kazakhstan	2010	Age-standardized		10.2% (7.6 to 12.9%)	6.9 (5.1 to 8.9)
Hemorrhagic stroke	Kazakhstan	2015	Age-standardized		10.9% (8.3 to 13.5%)	6.6 (4.8 to 8.4)
Chronic obstructive pulmonary disease	Kazakhstan	1990	All Ages	1.0 (0.5 to 1.6)	17.2% (8.3 to 27.5%)	6.1 (2.9 to 9.9)
Chronic obstructive pulmonary disease	Kazakhstan	1995	All Ages	1.0 (0.5 to 1.8)	14.7% (6.7 to 24.7%)	6.4 (3.0 to 11.0)
Chronic obstructive pulmonary disease	Kazakhstan	2000	All Ages	0.8 (0.4 to 1.4)	14.7% (6.8 to 24.6%)	5.4 (2.5 to 9.1)
Chronic obstructive pulmonary disease	Kazakhstan	2005	All Ages	0.8 (0.4 to 1.3)	13.9% (6.2 to 23.7%)	5.0 (2.2 to 8.5)
Chronic obstructive pulmonary disease	Kazakhstan	2010	All Ages	0.8 (0.4 to 1.3)	15.0% (7.0 to 25.1%)	4.9 (2.3 to 8.2)
Chronic obstructive pulmonary disease	Kazakhstan	2015	All Ages	1.0 (0.5 to 1.6)	16.7% (8.0 to 27.1%)	5.5 (2.6 to 8.9)
Chronic obstructive pulmonary disease	Kazakhstan	1990	Age-standardized		17.2% (8.3 to 27.6%)	9.7 (4.7 to 15.9)
Chronic obstructive pulmonary disease	Kazakhstan	1995	Age-standardized		14.7% (6.8 to 24.8%)	9.3 (4.3 to 15.8)
Chronic obstructive pulmonary disease	Kazakhstan	2000	Age-standardized		14.7% (6.8 to 24.7%)	7.7 (3.6 to 13.0)
Chronic obstructive pulmonary disease	Kazakhstan	2005	Age-standardized		13.9% (6.2 to 23.8%)	6.9 (3.1 to 11.7)
Chronic obstructive pulmonary disease	Kazakhstan	2010	Age-standardized		15.1% (7.0 to 25.1%)	6.9 (3.2 to 11.6)

Cause name	Location	Year	Age group	Number (in thousands)	PAF	Rate (per 100,000)
Chronic obstructive pulmonary disease	Kazakhstan	2015	Age-standardized		16.7% (8.0 to 27.1%)	7.4 (3.6 to 12.0)
All causes	Kyrgyzstan	1990	All Ages	2.7 (2.2 to 3.3)	7.2% (5.9 to 8.8%)	61.0 (50.0 to 74.5)
All causes	Kyrgyzstan	1995	All Ages	2.7 (2.2 to 3.2)	6.8% (5.6 to 8.2%)	57.5 (47.2 to 69.5)
All causes	Kyrgyzstan	2000	All Ages	2.6 (2.1 to 3.1)	6.8% (5.6 to 8.0%)	51.7 (42.6 to 61.5)
All causes	Kyrgyzstan	2005	All Ages	2.7 (2.3 to 3.2)	6.8% (5.7 to 7.9%)	52.5 (44.0 to 61.7)
All causes	Kyrgyzstan	2010	All Ages	2.7 (2.2 to 3.1)	6.7% (5.6 to 7.9%)	48.6 (40.2 to 56.9)
All causes	Kyrgyzstan	2015	All Ages	2.7 (2.3 to 3.2)	7.0% (5.9 to 8.2%)	45.7 (38.1 to 53.7)
All causes	Kyrgyzstan	1990	Age-standardized		7.8% (6.4 to 9.4%)	97.3 (79.8 to 116.7)
All causes	Kyrgyzstan	1995	Age-standardized		7.5% (6.2 to 9.0%)	101.6 (82.6 to 122.2)
All causes	Kyrgyzstan	2000	Age-standardized		7.4% (6.1 to 8.9%)	94.9 (77.7 to 114.9)
All causes	Kyrgyzstan	2005	Age-standardized		7.5% (6.2 to 8.8%)	93.4 (77.3 to 110.6)
All causes	Kyrgyzstan	2010	Age-standardized		7.6% (6.2 to 9.0%)	90.3 (73.4 to 106.7)
All causes	Kyrgyzstan	2015	Age-standardized		7.8% (6.4 to 9.3%)	81.9 (66.8 to 97.1)
Lower respiratory infections	Kyrgyzstan	1990	All Ages	0.6 (0.3 to 1.0)	14.2% (6.6 to 24.5%)	13.6 (6.4 to 23.5)
Lower respiratory infections	Kyrgyzstan	1995	All Ages	0.4 (0.2 to 0.8)	12.6% (5.3 to 23.1%)	9.0 (3.7 to 16.4)
Lower respiratory infections	Kyrgyzstan	2000	All Ages	0.3 (0.1 to 0.6)	12.7% (5.2 to 23.1%)	6.2 (2.5 to 11.2)
Lower respiratory infections	Kyrgyzstan	2005	All Ages	0.2 (0.1 to 0.4)	12.8% (5.1 to 23.3%)	4.6 (1.9 to 8.4)
Lower respiratory infections	Kyrgyzstan	2010	All Ages	0.2 (0.1 to 0.4)	13.5% (5.9 to 23.9%)	4.2 (1.8 to 7.5)
Lower respiratory infections	Kyrgyzstan	2015	All Ages	0.2 (0.1 to 0.4)	14.3% (6.4 to 24.4%)	3.5 (1.5 to 6.2)
Lower respiratory infections	Kyrgyzstan	1990	Age-standardized		14.2% (6.6 to 24.5%)	9.7 (4.5 to 16.8)
Lower respiratory infections	Kyrgyzstan	1995	Age-standardized		12.6% (5.3 to 23.1%)	7.3 (3.1 to 13.5)
Lower respiratory infections	Kyrgyzstan	2000	Age-standardized		12.7% (5.2 to 23.1%)	6.1 (2.5 to 11.2)
Lower respiratory infections	Kyrgyzstan	2005	Age-standardized		12.8% (5.1 to 23.3%)	4.9 (2.0 to 8.9)
Lower respiratory infections	Kyrgyzstan	2010	Age-standardized		13.5% (5.9 to 23.9%)	4.1 (1.8 to 7.3)
Lower respiratory infections	Kyrgyzstan	2015	Age-standardized		14.3% (6.4 to 24.4%)	3.4 (1.5 to 6.0)
Tracheal, bronchus, and lung cancer	Kyrgyzstan	1990	All Ages	0.1 (0.0 to 0.1)	9.8% (5.1 to 15.4%)	1.4 (0.7 to 2.3)
Tracheal, bronchus, and lung cancer	Kyrgyzstan	1995	All Ages	0.1 (0.0 to 0.1)	8.7% (4.4 to 14.0%)	1.1 (0.5 to 1.7)
Tracheal, bronchus, and lung cancer	Kyrgyzstan	2000	All Ages	0.0 (0.0 to 0.1)	8.7% (4.4 to 14.1%)	0.9 (0.4 to 1.4)
Tracheal, bronchus, and lung cancer	Kyrgyzstan	2005	All Ages	0.0 (0.0 to 0.1)	8.8% (4.4 to 14.1%)	0.8 (0.4 to 1.3)
Tracheal, bronchus, and lung cancer	Kyrgyzstan	2010	All Ages	0.0 (0.0 to 0.1)	9.3% (4.7 to 14.8%)	0.8 (0.4 to 1.3)
Tracheal, bronchus, and lung cancer	Kyrgyzstan	2015	All Ages	0.1 (0.0 to 0.1)	9.8% (5.1 to 15.5%)	0.9 (0.5 to 1.4)
Tracheal, bronchus, and lung cancer	Kyrgyzstan	1990	Age-standardized		9.8% (5.1 to 15.4%)	2.3 (1.2 to 3.6)
Tracheal, bronchus, and lung cancer	Kyrgyzstan	1995	Age-standardized		8.7% (4.4 to 14.1%)	1.9 (0.9 to 3.0)
Tracheal, bronchus, and lung cancer	Kyrgyzstan	2000	Age-standardized		8.7% (4.4 to 14.2%)	1.5 (0.7 to 2.4)
Tracheal, bronchus, and lung cancer	Kyrgyzstan	2005	Age-standardized		8.8% (4.4 to 14.1%)	1.4 (0.7 to 2.3)
Tracheal, bronchus, and lung cancer	Kyrgyzstan	2010	Age-standardized		9.3% (4.7 to 14.8%)	1.4 (0.7 to 2.3)

Cause name	Location	Year	Age group	Number (in thousands)	PAF	Rate (per 100,000)
Tracheal, bronchus, and lung cancer	Kyrgyzstan	2015	Age-standardized		9.9% (5.1 to 15.5%)	1.5 (0.7 to 2.3)
Cardiovascular diseases	Kyrgyzstan	1990	All Ages	1.6 (1.3 to 1.9)	11.2% (9.2 to 13.1%)	36.3 (29.6 to 42.5)
Cardiovascular diseases	Kyrgyzstan	1995	All Ages	1.8 (1.5 to 2.1)	10.9% (9.0 to 12.9%)	38.4 (31.3 to 45.3)
Cardiovascular diseases	Kyrgyzstan	2000	All Ages	1.8 (1.5 to 2.2)	11.1% (9.1 to 13.0%)	37.1 (30.4 to 43.6)
Cardiovascular diseases	Kyrgyzstan	2005	All Ages	2.1 (1.7 to 2.5)	11.1% (9.2 to 13.0%)	40.1 (33.1 to 47.7)
Cardiovascular diseases	Kyrgyzstan	2010	All Ages	2.1 (1.7 to 2.5)	11.4% (9.3 to 13.5%)	38.3 (31.1 to 45.4)
Cardiovascular diseases	Kyrgyzstan	2015	All Ages	2.1 (1.8 to 2.6)	11.8% (9.8 to 14.0%)	36.4 (29.9 to 43.5)
Cardiovascular diseases	Kyrgyzstan	1990	Age-standardized		10.9% (8.7 to 12.9%)	66.9 (53.5 to 80.1)
Cardiovascular diseases	Kyrgyzstan	1995	Age-standardized		10.4% (8.4 to 12.5%)	74.2 (59.7 to 89.0)
Cardiovascular diseases	Kyrgyzstan	2000	Age-standardized		10.6% (8.5 to 12.6%)	72.1 (57.9 to 85.8)
Cardiovascular diseases	Kyrgyzstan	2005	Age-standardized		10.8% (8.7 to 12.9%)	73.9 (59.8 to 89.1)
Cardiovascular diseases	Kyrgyzstan	2010	Age-standardized		11.0% (8.8 to 13.2%)	73.8 (58.8 to 88.2)
Cardiovascular diseases	Kyrgyzstan	2015	Age-standardized		11.3% (9.1 to 13.6%)	67.5 (54.3 to 81.7)
Ischemic heart disease	Kyrgyzstan	1990	All Ages	1.0 (0.8 to 1.3)	13.5% (10.4 to 16.4%)	23.2 (17.8 to 28.4)
Ischemic heart disease	Kyrgyzstan	1995	All Ages	1.1 (0.9 to 1.4)	13.2% (10.3 to 16.0%)	24.6 (19.1 to 30.1)
Ischemic heart disease	Kyrgyzstan	2000	All Ages	1.2 (0.9 to 1.4)	13.4% (10.7 to 16.2%)	23.9 (18.9 to 29.0)
Ischemic heart disease	Kyrgyzstan	2005	All Ages	1.4 (1.1 to 1.7)	13.1% (10.3 to 15.9%)	27.0 (21.1 to 33.1)
Ischemic heart disease	Kyrgyzstan	2010	All Ages	1.5 (1.1 to 1.8)	13.2% (10.2 to 16.2%)	27.0 (20.8 to 33.2)
Ischemic heart disease	Kyrgyzstan	2015	All Ages	1.5 (1.2 to 1.9)	13.7% (10.7 to 16.7%)	25.8 (20.0 to 32.0)
Ischemic heart disease	Kyrgyzstan	1990	Age-standardized		12.9% (9.7 to 16.0%)	42.9 (31.7 to 53.6)
Ischemic heart disease	Kyrgyzstan	1995	Age-standardized		12.4% (9.3 to 15.5%)	47.7 (35.9 to 59.7)
Ischemic heart disease	Kyrgyzstan	2000	Age-standardized		12.5% (9.4 to 15.4%)	47.1 (35.3 to 58.6)
Ischemic heart disease	Kyrgyzstan	2005	Age-standardized		12.6% (9.6 to 15.6%)	50.2 (37.8 to 62.5)
Ischemic heart disease	Kyrgyzstan	2010	Age-standardized		12.6% (9.5 to 15.8%)	52.8 (39.5 to 66.1)
Ischemic heart disease	Kyrgyzstan	2015	Age-standardized		13.0% (9.8 to 16.1%)	48.7 (36.7 to 60.9)
Ischemic stroke	Kyrgyzstan	1990	All Ages	0.3 (0.2 to 0.4)	9.4% (6.5 to 12.7%)	6.5 (4.4 to 8.8)
Ischemic stroke	Kyrgyzstan	1995	All Ages	0.3 (0.2 to 0.4)	9.1% (6.2 to 12.2%)	6.6 (4.5 to 9.0)
Ischemic stroke	Kyrgyzstan	2000	All Ages	0.3 (0.2 to 0.4)	9.4% (6.6 to 12.3%)	6.3 (4.3 to 8.3)
Ischemic stroke	Kyrgyzstan	2005	All Ages	0.3 (0.2 to 0.4)	9.4% (6.5 to 12.2%)	6.1 (4.3 to 8.1)
Ischemic stroke	Kyrgyzstan	2010	All Ages	0.3 (0.2 to 0.4)	9.6% (6.8 to 12.5%)	5.2 (3.6 to 6.8)
Ischemic stroke	Kyrgyzstan	2015	All Ages	0.3 (0.2 to 0.4)	10.0% (7.2 to 13.0%)	4.8 (3.4 to 6.4)
Ischemic stroke	Kyrgyzstan	1990	Age-standardized		9.1% (6.1 to 12.7%)	12.4 (8.1 to 17.4)
Ischemic stroke	Kyrgyzstan	1995	Age-standardized		8.7% (5.7 to 12.1%)	13.6 (8.6 to 18.9)
Ischemic stroke	Kyrgyzstan	2000	Age-standardized		8.9% (6.1 to 12.1%)	12.7 (8.4 to 17.2)
Ischemic stroke	Kyrgyzstan	2005	Age-standardized		9.1% (6.3 to 12.1%)	11.7 (8.1 to 15.7)
Ischemic stroke	Kyrgyzstan	2010	Age-standardized		9.3% (6.2 to 12.3%)	10.5 (7.0 to 14.0)

Cause name	Location	Year	Age group	Number (in thousands)	PAF	Rate (per 100,000)
Ischemic stroke	Kyrgyzstan	2015	Age-standardized		9.6% (6.6 to 12.6%)	9.4 (6.4 to 12.5)
Hemorrhagic stroke	Kyrgyzstan	1990	All Ages	0.3 (0.2 to 0.4)	10.9% (8.2 to 13.7%)	6.7 (5.0 to 8.6)
Hemorrhagic stroke	Kyrgyzstan	1995	All Ages	0.3 (0.2 to 0.4)	10.5% (7.7 to 13.3%)	7.2 (5.2 to 9.2)
Hemorrhagic stroke	Kyrgyzstan	2000	All Ages	0.3 (0.3 to 0.5)	10.7% (7.9 to 13.6%)	6.9 (5.1 to 9.0)
Hemorrhagic stroke	Kyrgyzstan	2005	All Ages	0.4 (0.3 to 0.5)	10.7% (8.0 to 13.6%)	7.0 (5.3 to 8.9)
Hemorrhagic stroke	Kyrgyzstan	2010	All Ages	0.3 (0.3 to 0.4)	11.1% (8.4 to 14.0%)	6.1 (4.5 to 7.9)
Hemorrhagic stroke	Kyrgyzstan	2015	All Ages	0.3 (0.3 to 0.4)	11.6% (8.7 to 14.5%)	5.8 (4.3 to 7.4)
Hemorrhagic stroke	Kyrgyzstan	1990	Age-standardized		10.5% (7.8 to 13.2%)	11.6 (8.5 to 15.0)
Hemorrhagic stroke	Kyrgyzstan	1995	Age-standardized		10.0% (7.4 to 12.8%)	12.9 (9.4 to 16.7)
Hemorrhagic stroke	Kyrgyzstan	2000	Age-standardized		10.2% (7.5 to 13.1%)	12.3 (8.9 to 16.2)
Hemorrhagic stroke	Kyrgyzstan	2005	Age-standardized		10.3% (7.6 to 13.1%)	12.0 (8.8 to 15.5)
Hemorrhagic stroke	Kyrgyzstan	2010	Age-standardized		10.5% (7.8 to 13.3%)	10.6 (7.8 to 13.6)
Hemorrhagic stroke	Kyrgyzstan	2015	Age-standardized		10.8% (8.1 to 13.7%)	9.4 (6.9 to 12.2)
Chronic obstructive pulmonary disease	Kyrgyzstan	1990	All Ages	0.4 (0.2 to 0.7)	15.6% (7.1 to 26.0%)	9.7 (4.4 to 16.0)
Chronic obstructive pulmonary disease	Kyrgyzstan	1995	All Ages	0.4 (0.2 to 0.7)	14.3% (6.3 to 24.3%)	9.1 (4.0 to 15.3)
Chronic obstructive pulmonary disease	Kyrgyzstan	2000	All Ages	0.4 (0.2 to 0.6)	14.4% (6.3 to 24.7%)	7.5 (3.2 to 12.6)
Chronic obstructive pulmonary disease	Kyrgyzstan	2005	All Ages	0.4 (0.1 to 0.6)	14.5% (6.1 to 24.7%)	7.0 (3.0 to 11.7)
Chronic obstructive pulmonary disease	Kyrgyzstan	2010	All Ages	0.3 (0.1 to 0.5)	15.1% (6.6 to 25.2%)	5.4 (2.3 to 9.0)
Chronic obstructive pulmonary disease	Kyrgyzstan	2015	All Ages	0.3 (0.1 to 0.5)	15.8% (7.3 to 26.5%)	4.8 (2.2 to 7.9)
Chronic obstructive pulmonary disease	Kyrgyzstan	1990	Age-standardized		15.7% (7.1 to 26.2%)	18.5 (8.4 to 30.4)
Chronic obstructive pulmonary disease	Kyrgyzstan	1995	Age-standardized		14.4% (6.3 to 24.4%)	18.2 (8.0 to 30.9)
Chronic obstructive pulmonary disease	Kyrgyzstan	2000	Age-standardized		14.5% (6.3 to 24.9%)	15.2 (6.4 to 25.4)
Chronic obstructive pulmonary disease	Kyrgyzstan	2005	Age-standardized		14.5% (6.2 to 24.8%)	13.2 (5.6 to 22.4)
Chronic obstructive pulmonary disease	Kyrgyzstan	2010	Age-standardized		15.2% (6.6 to 25.3%)	11.0 (4.6 to 18.3)
Chronic obstructive pulmonary disease	Kyrgyzstan	2015	Age-standardized		15.9% (7.3 to 26.5%)	9.6 (4.3 to 15.7)
All causes	Mongolia	1990	All Ages	1.2 (0.9 to 1.5)	6.2% (4.7 to 8.1%)	53.4 (40.2 to 70.6)
All causes	Mongolia	1995	All Ages	1.1 (0.8 to 1.4)	5.8% (4.6 to 7.2%)	46.8 (37.0 to 58.8)
All causes	Mongolia	2000	All Ages	1.2 (1.0 to 1.4)	6.4% (5.4 to 7.5%)	48.5 (40.6 to 56.9)
All causes	Mongolia	2005	All Ages	1.2 (1.0 to 1.4)	6.2% (5.3 to 7.2%)	46.5 (39.5 to 54.1)
All causes	Mongolia	2010	All Ages	1.3 (1.1 to 1.5)	6.2% (5.3 to 7.2%)	46.3 (39.4 to 54.1)
All causes	Mongolia	2015	All Ages	1.5 (1.3 to 1.7)	6.7% (5.8 to 7.8%)	49.6 (42.4 to 57.5)
All causes	Mongolia	1990	Age-standardized		6.4% (5.2 to 7.6%)	88.9 (72.8 to 106.9)
All causes	Mongolia	1995	Age-standardized		6.2% (5.1 to 7.3%)	95.4 (78.5 to 112.9)
All causes	Mongolia	2000	Age-standardized		6.9% (5.8 to 8.0%)	105.9 (87.7 to 124.1)
All causes	Mongolia	2005	Age-standardized		6.8% (5.7 to 7.9%)	99.7 (83.2 to 117.6)
All causes	Mongolia	2010	Age-standardized		6.8% (5.7 to 8.0%)	91.3 (76.5 to 107.7)

Cause name	Location	Year	Age group	Number (in thousands)	PAF	Rate (per 100,000)
All causes	Mongolia	2015	Age-standardized		7.3% (6.1 to 8.6%)	90.0 (75.2 to 106.1)
Lower respiratory infections	Mongolia	1990	All Ages	0.5 (0.2 to 0.8)	15.1% (7.0 to 25.3%)	22.6 (10.4 to 38.6)
Lower respiratory infections	Mongolia	1995	All Ages	0.3 (0.1 to 0.6)	13.7% (5.9 to 24.2%)	13.5 (5.8 to 24.3)
Lower respiratory infections	Mongolia	2000	All Ages	0.2 (0.1 to 0.4)	15.8% (8.1 to 25.6%)	9.7 (4.9 to 16.2)
Lower respiratory infections	Mongolia	2005	All Ages	0.2 (0.1 to 0.3)	15.3% (7.7 to 25.1%)	6.5 (3.3 to 10.9)
Lower respiratory infections	Mongolia	2010	All Ages	0.1 (0.1 to 0.3)	16.5% (8.9 to 26.2%)	5.5 (2.8 to 9.0)
Lower respiratory infections	Mongolia	2015	All Ages	0.1 (0.1 to 0.2)	18.6% (10.6 to 28.2%)	4.4 (2.4 to 6.8)
Lower respiratory infections	Mongolia	1990	Age-standardized		15.1% (7.0 to 25.3%)	16.2 (7.4 to 27.6)
Lower respiratory infections	Mongolia	1995	Age-standardized		13.7% (5.9 to 24.2%)	12.6 (5.4 to 22.5)
Lower respiratory infections	Mongolia	2000	Age-standardized		15.8% (8.1 to 25.6%)	11.0 (5.6 to 18.1)
Lower respiratory infections	Mongolia	2005	Age-standardized		15.3% (7.7 to 25.1%)	7.4 (3.7 to 12.3)
Lower respiratory infections	Mongolia	2010	Age-standardized		16.5% (8.9 to 26.2%)	5.6 (2.8 to 9.1)
Lower respiratory infections	Mongolia	2015	Age-standardized		18.6% (10.6 to 28.2%)	4.6 (2.5 to 7.2)
Tracheal, bronchus, and lung cancer	Mongolia	1990	All Ages	0.0 (0.0 to 0.1)	10.4% (5.5 to 16.4%)	1.6 (0.8 to 2.6)
Tracheal, bronchus, and lung cancer	Mongolia	1995	All Ages	0.0 (0.0 to 0.1)	9.4% (4.9 to 15.1%)	1.6 (0.8 to 2.6)
Tracheal, bronchus, and lung cancer	Mongolia	2000	All Ages	0.0 (0.0 to 0.1)	11.0% (5.8 to 16.8%)	1.5 (0.8 to 2.4)
Tracheal, bronchus, and lung cancer	Mongolia	2005	All Ages	0.0 (0.0 to 0.1)	10.6% (5.7 to 16.5%)	1.4 (0.7 to 2.2)
Tracheal, bronchus, and lung cancer	Mongolia	2010	All Ages	0.1 (0.0 to 0.1)	11.5% (6.2 to 17.5%)	1.7 (0.9 to 2.6)
Tracheal, bronchus, and lung cancer	Mongolia	2015	All Ages	0.1 (0.0 to 0.1)	13.1% (7.3 to 19.5%)	2.1 (1.2 to 3.3)
Tracheal, bronchus, and lung cancer	Mongolia	1990	Age-standardized		10.5% (5.5 to 16.4%)	3.5 (1.8 to 5.7)
Tracheal, bronchus, and lung cancer	Mongolia	1995	Age-standardized		9.5% (4.9 to 15.2%)	3.8 (1.9 to 6.1)
Tracheal, bronchus, and lung cancer	Mongolia	2000	Age-standardized		11.0% (5.8 to 16.9%)	3.7 (2.0 to 5.8)
Tracheal, bronchus, and lung cancer	Mongolia	2005	Age-standardized		10.6% (5.7 to 16.6%)	3.3 (1.7 to 5.1)
Tracheal, bronchus, and lung cancer	Mongolia	2010	Age-standardized		11.5% (6.3 to 17.5%)	3.6 (2.0 to 5.6)
Tracheal, bronchus, and lung cancer	Mongolia	2015	Age-standardized		13.1% (7.3 to 19.6%)	4.2 (2.3 to 6.4)
Cardiovascular diseases	Mongolia	1990	All Ages	0.6 (0.5 to 0.7)	11.2% (9.4 to 13.0%)	27.2 (22.6 to 32.2)
Cardiovascular diseases	Mongolia	1995	All Ages	0.7 (0.6 to 0.8)	11.0% (9.3 to 12.8%)	29.7 (24.8 to 34.7)
Cardiovascular diseases	Mongolia	2000	All Ages	0.8 (0.7 to 1.0)	12.3% (10.5 to 14.2%)	35.2 (29.9 to 41.0)
Cardiovascular diseases	Mongolia	2005	All Ages	0.9 (0.8 to 1.1)	12.8% (10.9 to 14.6%)	37.1 (31.3 to 43.1)
Cardiovascular diseases	Mongolia	2010	All Ages	1.0 (0.9 to 1.2)	13.5% (11.6 to 15.4%)	37.8 (32.1 to 44.1)
Cardiovascular diseases	Mongolia	2015	All Ages	1.2 (1.0 to 1.4)	14.0% (12.1 to 16.0%)	41.5 (35.0 to 48.1)
Cardiovascular diseases	Mongolia	1990	Age-standardized		10.6% (8.7 to 12.6%)	63.7 (52.0 to 77.0)
Cardiovascular diseases	Mongolia	1995	Age-standardized		10.4% (8.5 to 12.3%)	73.2 (59.3 to 87.8)
Cardiovascular diseases	Mongolia	2000	Age-standardized		11.4% (9.4 to 13.3%)	85.5 (69.9 to 100.8)
Cardiovascular diseases	Mongolia	2005	Age-standardized		11.6% (9.6 to 13.6%)	85.0 (69.7 to 100.6)
Cardiovascular diseases	Mongolia	2010	Age-standardized		12.1% (10.1 to 14.1%)	78.8 (65.4 to 94.0)

Cause name	Location	Year	Age group	Number (in thousands)	PAF	Rate (per 100,000)
Cardiovascular diseases	Mongolia	2015	Age-standardized		12.7% (10.6 to 14.8%)	77.8 (64.3 to 92.7)
Ischemic heart disease	Mongolia	1990	All Ages	0.4 (0.3 to 0.5)	14.7% (11.9 to 17.5%)	19.6 (15.7 to 23.9)
Ischemic heart disease	Mongolia	1995	All Ages	0.5 (0.4 to 0.6)	14.4% (11.8 to 17.1%)	21.5 (17.3 to 25.7)
Ischemic heart disease	Mongolia	2000	All Ages	0.6 (0.5 to 0.7)	15.5% (12.8 to 18.3%)	24.1 (19.6 to 28.8)
Ischemic heart disease	Mongolia	2005	All Ages	0.6 (0.5 to 0.7)	15.5% (12.8 to 18.2%)	22.7 (18.6 to 27.1)
Ischemic heart disease	Mongolia	2010	All Ages	0.6 (0.5 to 0.7)	16.1% (13.3 to 18.9%)	22.4 (18.4 to 26.7)
Ischemic heart disease	Mongolia	2015	All Ages	0.7 (0.6 to 0.9)	16.5% (13.7 to 19.6%)	24.8 (20.2 to 29.7)
Ischemic heart disease	Mongolia	1990	Age-standardized		13.6% (10.5 to 16.8%)	47.2 (36.1 to 58.9)
Ischemic heart disease	Mongolia	1995	Age-standardized		13.2% (10.2 to 16.2%)	54.3 (41.4 to 67.2)
Ischemic heart disease	Mongolia	2000	Age-standardized		14.0% (11.0 to 17.0%)	60.1 (46.7 to 73.6)
Ischemic heart disease	Mongolia	2005	Age-standardized		13.7% (10.7 to 16.6%)	53.9 (41.5 to 66.8)
Ischemic heart disease	Mongolia	2010	Age-standardized		14.2% (11.2 to 17.2%)	48.6 (38.2 to 61.0)
Ischemic heart disease	Mongolia	2015	Age-standardized		14.8% (11.6 to 18.0%)	48.6 (37.7 to 60.8)
Ischemic stroke	Mongolia	1990	All Ages	0.0 (0.0 to 0.0)	10.4% (7.5 to 13.4%)	1.0 (0.7 to 1.5)
Ischemic stroke	Mongolia	1995	All Ages	0.0 (0.0 to 0.0)	10.0% (7.2 to 12.9%)	1.1 (0.7 to 1.6)
Ischemic stroke	Mongolia	2000	All Ages	0.0 (0.0 to 0.0)	10.9% (8.1 to 13.8%)	1.3 (1.0 to 1.9)
Ischemic stroke	Mongolia	2005	All Ages	0.0 (0.0 to 0.1)	11.0% (8.2 to 13.7%)	1.5 (1.1 to 2.0)
Ischemic stroke	Mongolia	2010	All Ages	0.0 (0.0 to 0.1)	11.7% (9.0 to 14.5%)	1.5 (1.1 to 2.0)
Ischemic stroke	Mongolia	2015	All Ages	0.1 (0.0 to 0.1)	12.2% (9.5 to 15.2%)	1.6 (1.2 to 2.1)
Ischemic stroke	Mongolia	1990	Age-standardized		9.8% (6.7 to 13.1%)	2.6 (1.7 to 4.0)
Ischemic stroke	Mongolia	1995	Age-standardized		9.3% (6.4 to 12.6%)	2.9 (1.9 to 4.3)
Ischemic stroke	Mongolia	2000	Age-standardized		10.1% (7.2 to 13.4%)	3.6 (2.4 to 5.2)
Ischemic stroke	Mongolia	2005	Age-standardized		10.0% (7.1 to 13.1%)	3.7 (2.5 to 5.3)
Ischemic stroke	Mongolia	2010	Age-standardized		10.6% (7.7 to 13.6%)	3.4 (2.4 to 4.8)
Ischemic stroke	Mongolia	2015	Age-standardized		11.2% (8.2 to 14.4%)	3.3 (2.4 to 4.6)
Hemorrhagic stroke	Mongolia	1990	All Ages	0.1 (0.1 to 0.2)	11.5% (8.8 to 14.5%)	6.6 (4.8 to 9.3)
Hemorrhagic stroke	Mongolia	1995	All Ages	0.2 (0.1 to 0.2)	11.2% (8.5 to 14.1%)	7.1 (5.3 to 9.8)
Hemorrhagic stroke	Mongolia	2000	All Ages	0.2 (0.2 to 0.3)	12.4% (9.6 to 15.3%)	9.8 (7.5 to 12.9)
Hemorrhagic stroke	Mongolia	2005	All Ages	0.3 (0.3 to 0.4)	12.2% (9.4 to 15.0%)	12.9 (9.9 to 16.2)
Hemorrhagic stroke	Mongolia	2010	All Ages	0.4 (0.3 to 0.5)	12.8% (10.0 to 15.6%)	14.0 (10.7 to 17.4)
Hemorrhagic stroke	Mongolia	2015	All Ages	0.4 (0.3 to 0.6)	13.5% (10.6 to 16.5%)	15.0 (11.6 to 18.6)
Hemorrhagic stroke	Mongolia	1990	Age-standardized		11.2% (8.4 to 14.0%)	14.0 (10.1 to 20.0)
Hemorrhagic stroke	Mongolia	1995	Age-standardized		10.7% (8.0 to 13.5%)	15.9 (11.8 to 22.0)
Hemorrhagic stroke	Mongolia	2000	Age-standardized		11.5% (8.7 to 14.4%)	21.7 (16.3 to 29.2)
Hemorrhagic stroke	Mongolia	2005	Age-standardized		11.0% (8.3 to 13.9%)	27.4 (20.5 to 34.7)
Hemorrhagic stroke	Mongolia	2010	Age-standardized		11.5% (8.8 to 14.3%)	26.8 (20.4 to 33.7)

Cause name	Location	Year	Age group	Number (in thousands)	PAF	Rate (per 100,000)
Hemorrhagic stroke	Mongolia	2015	Age-standardized		12.1% (9.3 to 15.1%)	25.8 (19.7 to 32.8)
Chronic obstructive pulmonary disease	Mongolia	1990	All Ages	0.0 (0.0 to 0.1)	13.7% (6.2 to 22.4%)	2.1 (0.9 to 3.5)
Chronic obstructive pulmonary disease	Mongolia	1995	All Ages	0.1 (0.0 to 0.1)	13.6% (6.0 to 22.5%)	2.1 (0.9 to 3.5)
Chronic obstructive pulmonary disease	Mongolia	2000	All Ages	0.1 (0.0 to 0.1)	15.7% (7.3 to 25.2%)	2.0 (0.9 to 3.3)
Chronic obstructive pulmonary disease	Mongolia	2005	All Ages	0.0 (0.0 to 0.1)	15.7% (7.4 to 25.4%)	1.5 (0.7 to 2.4)
Chronic obstructive pulmonary disease	Mongolia	2010	All Ages	0.0 (0.0 to 0.1)	16.8% (7.9 to 26.7%)	1.3 (0.6 to 2.2)
Chronic obstructive pulmonary disease	Mongolia	2015	All Ages	0.1 (0.0 to 0.1)	18.7% (9.4 to 29.0%)	1.6 (0.7 to 2.4)
Chronic obstructive pulmonary disease	Mongolia	1990	Age-standardized		15.8% (7.2 to 25.6%)	5.4 (2.5 to 9.2)
Chronic obstructive pulmonary disease	Mongolia	1995	Age-standardized		14.9% (6.7 to 24.9%)	5.9 (2.6 to 9.8)
Chronic obstructive pulmonary disease	Mongolia	2000	Age-standardized		16.7% (7.7 to 26.9%)	5.7 (2.6 to 9.5)
Chronic obstructive pulmonary disease	Mongolia	2005	Age-standardized		16.3% (7.6 to 26.5%)	4.0 (1.8 to 6.6)
Chronic obstructive pulmonary disease	Mongolia	2010	Age-standardized		17.3% (8.2 to 27.7%)	3.3 (1.6 to 5.3)
Chronic obstructive pulmonary disease	Mongolia	2015	Age-standardized		19.1% (9.5 to 29.5%)	3.5 (1.7 to 5.4)
All causes	Tajikistan	1990	All Ages	6.0 (5.2 to 6.9)	13.4% (11.6 to 15.3%)	113.6 (98.6 to 130.2)
All causes	Tajikistan	1995	All Ages	5.7 (4.9 to 6.5)	12.1% (10.5 to 13.9%)	98.3 (84.5 to 112.5)
All causes	Tajikistan	2000	All Ages	4.9 (4.3 to 5.6)	11.8% (10.3 to 13.4%)	79.5 (68.8 to 90.7)
All causes	Tajikistan	2005	All Ages	4.0 (3.5 to 4.7)	10.2% (8.8 to 11.7%)	59.0 (50.8 to 67.9)
All causes	Tajikistan	2010	All Ages	4.1 (3.5 to 4.8)	10.3% (9.0 to 11.8%)	53.9 (46.4 to 62.2)
All causes	Tajikistan	2015	All Ages	4.0 (3.4 to 4.6)	10.3% (8.9 to 11.8%)	47.1 (40.5 to 54.2)
All causes	Tajikistan	1990	Age-standardized		14.1% (12.5 to 16.0%)	170.3 (149.5 to 192.3)
All causes	Tajikistan	1995	Age-standardized		13.2% (11.6 to 15.0%)	171.3 (149.2 to 195.3)
All causes	Tajikistan	2000	Age-standardized		13.2% (11.4 to 15.0%)	169.9 (146.8 to 195.0)
All causes	Tajikistan	2005	Age-standardized		11.6% (9.9 to 13.3%)	135.0 (115.8 to 155.7)
All causes	Tajikistan	2010	Age-standardized		11.9% (10.3 to 13.6%)	121.1 (103.7 to 139.9)
All causes	Tajikistan	2015	Age-standardized		11.8% (10.1 to 13.7%)	105.3 (89.7 to 122.7)
Lower respiratory infections	Tajikistan	1990	All Ages	3.1 (2.3 to 3.8)	36.1% (28.3 to 43.3%)	57.7 (44.2 to 71.7)
Lower respiratory infections	Tajikistan	1995	All Ages	2.8 (2.1 to 3.5)	33.3% (25.7 to 40.1%)	48.0 (36.2 to 60.4)
Lower respiratory infections	Tajikistan	2000	All Ages	2.1 (1.5 to 2.6)	33.3% (25.3 to 40.7%)	33.4 (24.7 to 41.5)
Lower respiratory infections	Tajikistan	2005	All Ages	1.3 (0.9 to 1.6)	28.0% (20.6 to 35.9%)	18.3 (13.1 to 23.8)
Lower respiratory infections	Tajikistan	2010	All Ages	1.2 (0.8 to 1.5)	29.5% (21.9 to 37.3%)	15.1 (10.9 to 19.7)
Lower respiratory infections	Tajikistan	2015	All Ages	1.1 (0.7 to 1.4)	29.8% (22.1 to 37.4%)	12.4 (8.7 to 16.5)
Lower respiratory infections	Tajikistan	1990	Age-standardized		36.1% (28.3 to 43.3%)	38.5 (29.7 to 47.4)
Lower respiratory infections	Tajikistan	1995	Age-standardized		33.3% (25.7 to 40.1%)	37.6 (27.7 to 47.0)
Lower respiratory infections	Tajikistan	2000	Age-standardized		33.3% (25.3 to 40.7%)	32.7 (24.1 to 40.8)
Lower respiratory infections	Tajikistan	2005	Age-standardized		28.0% (20.6 to 35.9%)	20.4 (14.6 to 26.4)
Lower respiratory infections	Tajikistan	2010	Age-standardized		29.5% (21.9 to 37.3%)	17.3 (12.6 to 22.3)

Cause name	Location	Year	Age group	Number (in thousands)	PAF	Rate (per 100,000)
Lower respiratory infections	Tajikistan	2015	Age-standardized		29.8% (22.1 to 37.4%)	14.8 (10.5 to 19.1)
Tracheal, bronchus, and lung cancer	Tajikistan	1990	All Ages	0.1 (0.1 to 0.1)	28.0% (19.4 to 36.5%)	2.0 (1.4 to 2.7)
Tracheal, bronchus, and lung cancer	Tajikistan	1995	All Ages	0.1 (0.0 to 0.1)	25.1% (16.9 to 33.5%)	1.1 (0.7 to 1.5)
Tracheal, bronchus, and lung cancer	Tajikistan	2000	All Ages	0.1 (0.0 to 0.1)	25.2% (16.8 to 34.0%)	0.8 (0.6 to 1.1)
Tracheal, bronchus, and lung cancer	Tajikistan	2005	All Ages	0.1 (0.0 to 0.1)	20.4% (12.7 to 28.8%)	0.7 (0.4 to 1.0)
Tracheal, bronchus, and lung cancer	Tajikistan	2010	All Ages	0.1 (0.0 to 0.1)	21.7% (13.7 to 29.7%)	0.7 (0.5 to 1.0)
Tracheal, bronchus, and lung cancer	Tajikistan	2015	All Ages	0.1 (0.0 to 0.1)	22.0% (13.9 to 30.6%)	0.8 (0.5 to 1.1)
Tracheal, bronchus, and lung cancer	Tajikistan	1990	Age-standardized		28.2% (19.6 to 36.9%)	4.1 (2.9 to 5.5)
Tracheal, bronchus, and lung cancer	Tajikistan	1995	Age-standardized		25.4% (17.1 to 33.8%)	2.5 (1.6 to 3.3)
Tracheal, bronchus, and lung cancer	Tajikistan	2000	Age-standardized		25.5% (16.9 to 34.3%)	2.0 (1.4 to 2.7)
Tracheal, bronchus, and lung cancer	Tajikistan	2005	Age-standardized		20.6% (12.8 to 29.0%)	1.6 (1.0 to 2.4)
Tracheal, bronchus, and lung cancer	Tajikistan	2010	Age-standardized		21.9% (13.9 to 30.1%)	1.7 (1.1 to 2.3)
Tracheal, bronchus, and lung cancer	Tajikistan	2015	Age-standardized		22.2% (14.0 to 30.9%)	1.6 (1.0 to 2.3)
Cardiovascular diseases	Tajikistan	1990	All Ages	2.3 (2.0 to 2.7)	18.2% (15.7 to 21.0%)	43.5 (37.5 to 50.4)
Cardiovascular diseases	Tajikistan	1995	All Ages	2.4 (2.1 to 2.8)	17.7% (15.4 to 20.4%)	41.2 (35.5 to 47.5)
Cardiovascular diseases	Tajikistan	2000	All Ages	2.4 (2.1 to 2.8)	17.8% (15.4 to 20.4%)	39.1 (33.5 to 45.1)
Cardiovascular diseases	Tajikistan	2005	All Ages	2.4 (2.1 to 2.8)	16.0% (13.7 to 18.4%)	35.4 (30.2 to 41.2)
Cardiovascular diseases	Tajikistan	2010	All Ages	2.6 (2.2 to 3.0)	16.3% (14.1 to 18.8%)	33.6 (28.7 to 38.8)
Cardiovascular diseases	Tajikistan	2015	All Ages	2.6 (2.2 to 3.0)	16.7% (14.3 to 19.4%)	30.0 (25.4 to 35.4)
Cardiovascular diseases	Tajikistan	1990	Age-standardized		17.6% (15.0 to 20.9%)	102.6 (87.3 to 121.0)
Cardiovascular diseases	Tajikistan	1995	Age-standardized		16.9% (14.3 to 19.9%)	109.3 (92.4 to 128.9)
Cardiovascular diseases	Tajikistan	2000	Age-standardized		16.7% (14.0 to 19.7%)	115.9 (96.7 to 137.7)
Cardiovascular diseases	Tajikistan	2005	Age-standardized		15.2% (12.7 to 17.9%)	99.3 (82.3 to 117.9)
Cardiovascular diseases	Tajikistan	2010	Age-standardized		15.6% (13.1 to 18.4%)	89.7 (75.3 to 106.6)
Cardiovascular diseases	Tajikistan	2015	Age-standardized		15.6% (13.1 to 18.7%)	78.1 (65.1 to 94.5)
Ischemic heart disease	Tajikistan	1990	All Ages	1.6 (1.3 to 1.9)	20.7% (17.0 to 25.2%)	29.7 (24.2 to 36.6)
Ischemic heart disease	Tajikistan	1995	All Ages	1.7 (1.4 to 2.0)	20.4% (17.0 to 24.5%)	28.3 (23.3 to 33.9)
Ischemic heart disease	Tajikistan	2000	All Ages	1.7 (1.4 to 2.0)	20.5% (17.1 to 24.6%)	26.8 (22.0 to 32.2)
Ischemic heart disease	Tajikistan	2005	All Ages	1.7 (1.3 to 2.0)	18.5% (15.3 to 22.3%)	24.2 (19.6 to 29.4)
Ischemic heart disease	Tajikistan	2010	All Ages	1.8 (1.5 to 2.2)	18.9% (15.5 to 22.8%)	23.4 (19.1 to 28.6)
Ischemic heart disease	Tajikistan	2015	All Ages	1.8 (1.5 to 2.2)	19.3% (16.0 to 23.3%)	21.1 (17.1 to 25.9)
Ischemic heart disease	Tajikistan	1990	Age-standardized		19.8% (16.0 to 24.6%)	70.2 (56.5 to 87.7)
Ischemic heart disease	Tajikistan	1995	Age-standardized		19.0% (15.3 to 23.6%)	75.1 (59.8 to 93.1)
Ischemic heart disease	Tajikistan	2000	Age-standardized		18.9% (15.1 to 23.5%)	79.6 (62.6 to 99.6)
Ischemic heart disease	Tajikistan	2005	Age-standardized		17.4% (13.7 to 21.4%)	67.9 (53.0 to 84.4)
Ischemic heart disease	Tajikistan	2010	Age-standardized		17.8% (14.0 to 22.0%)	62.2 (49.5 to 78.0)

Cause name	Location	Year	Age group	Number (in thousands)	PAF	Rate (per 100,000)
Ischemic heart disease	Tajikistan	2015	Age-standardized		17.8% (14.0 to 22.2%)	54.7 (43.0 to 68.8)
Ischemic stroke	Tajikistan	1990	All Ages	0.3 (0.2 to 0.4)	16.6% (13.0 to 20.4%)	5.8 (4.3 to 7.5)
Ischemic stroke	Tajikistan	1995	All Ages	0.3 (0.2 to 0.4)	15.9% (12.4 to 19.3%)	5.4 (3.9 to 6.9)
Ischemic stroke	Tajikistan	2000	All Ages	0.3 (0.3 to 0.4)	16.2% (12.7 to 19.8%)	5.4 (3.9 to 6.8)
Ischemic stroke	Tajikistan	2005	All Ages	0.3 (0.2 to 0.4)	14.2% (10.9 to 17.4%)	4.9 (3.5 to 6.3)
Ischemic stroke	Tajikistan	2010	All Ages	0.3 (0.2 to 0.4)	14.5% (11.1 to 17.8%)	4.5 (3.2 to 5.7)
Ischemic stroke	Tajikistan	2015	All Ages	0.3 (0.2 to 0.4)	14.7% (11.3 to 18.0%)	3.8 (2.7 to 4.8)
Ischemic stroke	Tajikistan	1990	Age-standardized		16.1% (12.4 to 20.1%)	14.5 (10.5 to 18.8)
Ischemic stroke	Tajikistan	1995	Age-standardized		15.2% (11.4 to 19.0%)	15.7 (11.0 to 20.5)
Ischemic stroke	Tajikistan	2000	Age-standardized		15.2% (11.3 to 19.2%)	17.3 (12.1 to 22.7)
Ischemic stroke	Tajikistan	2005	Age-standardized		13.6% (10.1 to 17.1%)	14.8 (10.4 to 19.2)
Ischemic stroke	Tajikistan	2010	Age-standardized		14.0% (10.5 to 17.7%)	12.9 (9.0 to 16.8)
Ischemic stroke	Tajikistan	2015	Age-standardized		14.0% (10.2 to 17.6%)	11.0 (7.5 to 14.4)
Hemorrhagic stroke	Tajikistan	1990	All Ages	0.4 (0.3 to 0.5)	18.9% (15.8 to 22.2%)	8.0 (6.1 to 9.5)
Hemorrhagic stroke	Tajikistan	1995	All Ages	0.4 (0.4 to 0.5)	18.0% (14.8 to 21.1%)	7.4 (6.0 to 8.9)
Hemorrhagic stroke	Tajikistan	2000	All Ages	0.4 (0.3 to 0.5)	18.0% (14.8 to 21.2%)	6.9 (5.4 to 8.4)
Hemorrhagic stroke	Tajikistan	2005	All Ages	0.4 (0.3 to 0.5)	15.7% (12.7 to 18.7%)	6.2 (4.8 to 7.6)
Hemorrhagic stroke	Tajikistan	2010	All Ages	0.4 (0.3 to 0.5)	16.3% (13.3 to 19.3%)	5.8 (4.5 to 7.1)
Hemorrhagic stroke	Tajikistan	2015	All Ages	0.4 (0.3 to 0.5)	16.8% (13.8 to 19.8%)	5.1 (4.0 to 6.3)
Hemorrhagic stroke	Tajikistan	1990	Age-standardized		18.1% (14.9 to 21.3%)	17.9 (13.7 to 21.5)
Hemorrhagic stroke	Tajikistan	1995	Age-standardized		16.9% (13.7 to 20.0%)	18.5 (14.6 to 22.8)
Hemorrhagic stroke	Tajikistan	2000	Age-standardized		16.7% (13.3 to 20.0%)	19.0 (14.5 to 23.9)
Hemorrhagic stroke	Tajikistan	2005	Age-standardized		14.8% (11.7 to 17.9%)	16.6 (12.6 to 20.8)
Hemorrhagic stroke	Tajikistan	2010	Age-standardized		15.3% (12.1 to 18.4%)	14.6 (11.1 to 18.2)
Hemorrhagic stroke	Tajikistan	2015	Age-standardized		15.4% (12.2 to 18.5%)	12.4 (9.5 to 15.6)
Chronic obstructive pulmonary disease	Tajikistan	1990	All Ages	0.6 (0.4 to 0.8)	33.0% (21.9 to 47.4%)	10.4 (6.9 to 14.9)
Chronic obstructive pulmonary disease	Tajikistan	1995	All Ages	0.5 (0.3 to 0.7)	30.0% (19.0 to 42.8%)	8.1 (5.2 to 11.7)
Chronic obstructive pulmonary disease	Tajikistan	2000	All Ages	0.4 (0.3 to 0.6)	30.0% (19.0 to 42.3%)	6.2 (4.0 to 8.9)
Chronic obstructive pulmonary disease	Tajikistan	2005	All Ages	0.3 (0.2 to 0.5)	26.0% (15.5 to 37.6%)	4.7 (2.8 to 7.0)
Chronic obstructive pulmonary disease	Tajikistan	2010	All Ages	0.3 (0.2 to 0.5)	27.3% (16.8 to 39.1%)	4.5 (2.7 to 6.8)
Chronic obstructive pulmonary disease	Tajikistan	2015	All Ages	0.3 (0.2 to 0.5)	27.6% (16.6 to 39.9%)	3.9 (2.4 to 5.8)
Chronic obstructive pulmonary disease	Tajikistan	1990	Age-standardized		33.5% (22.2 to 48.2%)	25.1 (16.8 to 36.0)
Chronic obstructive pulmonary disease	Tajikistan	1995	Age-standardized		30.9% (19.5 to 44.0%)	22.1 (14.0 to 32.2)
Chronic obstructive pulmonary disease	Tajikistan	2000	Age-standardized		31.0% (19.6 to 43.7%)	19.3 (12.3 to 27.6)
Chronic obstructive pulmonary disease	Tajikistan	2005	Age-standardized		26.7% (15.8 to 38.5%)	13.7 (8.1 to 20.4)
Chronic obstructive pulmonary disease	Tajikistan	2010	Age-standardized		27.9% (17.2 to 39.9%)	12.4 (7.4 to 18.6)

Cause name	Location	Year	Age group	Number (in thousands)	PAF	Rate (per 100,000)
Chronic obstructive pulmonary disease	Tajikistan	2015	Age-standardized		28.1% (17.0 to 40.6%)	10.8 (6.5 to 15.8)
All causes	Turkmenistan	1990	All Ages	3.5 (2.9 to 4.1)	11.3% (9.7 to 13.2%)	95.2 (79.5 to 112.0)
All causes	Turkmenistan	1995	All Ages	3.8 (3.2 to 4.4)	11.1% (9.6 to 12.8%)	89.7 (76.6 to 104.8)
All causes	Turkmenistan	2000	All Ages	3.8 (3.3 to 4.5)	11.1% (9.5 to 12.7%)	84.8 (72.5 to 98.3)
All causes	Turkmenistan	2005	All Ages	2.9 (2.4 to 3.4)	8.5% (7.1 to 10.0%)	60.4 (50.9 to 71.4)
All causes	Turkmenistan	2010	All Ages	3.1 (2.7 to 3.6)	9.6% (8.2 to 10.9%)	62.0 (53.0 to 71.8)
All causes	Turkmenistan	2015	All Ages	3.1 (2.6 to 3.5)	9.5% (8.2 to 10.9%)	57.1 (48.9 to 65.6)
All causes	Turkmenistan	1990	Age-standardized		11.7% (10.1 to 13.3%)	148.8 (128.8 to 169.9)
All causes	Turkmenistan	1995	Age-standardized		11.6% (10.0 to 13.2%)	156.1 (134.3 to 178.3)
All causes	Turkmenistan	2000	Age-standardized		11.6% (10.0 to 13.3%)	154.9 (132.8 to 178.2)
All causes	Turkmenistan	2005	Age-standardized		9.1% (7.6 to 10.7%)	111.7 (92.8 to 131.8)
All causes	Turkmenistan	2010	Age-standardized		10.2% (8.6 to 11.9%)	111.0 (93.0 to 130.1)
All causes	Turkmenistan	2015	Age-standardized		10.1% (8.5 to 11.8%)	100.4 (84.3 to 116.9)
Lower respiratory infections	Turkmenistan	1990	All Ages	1.6 (1.1 to 2.1)	25.7% (18.2 to 33.5%)	43.1 (29.8 to 56.8)
Lower respiratory infections	Turkmenistan	1995	All Ages	1.4 (0.9 to 1.9)	24.3% (16.9 to 32.7%)	32.7 (22.1 to 45.8)
Lower respiratory infections	Turkmenistan	2000	All Ages	1.1 (0.7 to 1.6)	24.5% (17.1 to 32.9%)	24.9 (16.4 to 35.6)
Lower respiratory infections	Turkmenistan	2005	All Ages	0.6 (0.3 to 1.0)	16.7% (8.8 to 26.1%)	12.4 (6.2 to 20.9)
Lower respiratory infections	Turkmenistan	2010	All Ages	0.6 (0.3 to 0.9)	21.9% (14.2 to 31.0%)	11.2 (6.6 to 17.3)
Lower respiratory infections	Turkmenistan	2015	All Ages	0.4 (0.3 to 0.7)	22.6% (15.0 to 31.4%)	8.1 (4.9 to 12.5)
Lower respiratory infections	Turkmenistan	1990	Age-standardized		25.7% (18.2 to 33.5%)	27.4 (19.1 to 36.0)
Lower respiratory infections	Turkmenistan	1995	Age-standardized		24.3% (16.9 to 32.7%)	24.5 (16.5 to 33.9)
Lower respiratory infections	Turkmenistan	2000	Age-standardized		24.5% (17.1 to 32.9%)	22.5 (15.0 to 31.9)
Lower respiratory infections	Turkmenistan	2005	Age-standardized		16.7% (8.8 to 26.1%)	11.9 (5.9 to 19.9)
Lower respiratory infections	Turkmenistan	2010	Age-standardized		21.9% (14.2 to 31.0%)	11.1 (6.7 to 17.0)
Lower respiratory infections	Turkmenistan	2015	Age-standardized		22.6% (15.0 to 31.4%)	8.5 (5.2 to 12.9)
Tracheal, bronchus, and lung cancer	Turkmenistan	1990	All Ages	0.1 (0.0 to 0.1)	18.4% (11.3 to 25.9%)	1.3 (0.8 to 1.9)
Tracheal, bronchus, and lung cancer	Turkmenistan	1995	All Ages	0.1 (0.0 to 0.1)	17.3% (10.6 to 24.5%)	1.2 (0.7 to 1.7)
Tracheal, bronchus, and lung cancer	Turkmenistan	2000	All Ages	0.1 (0.0 to 0.1)	17.4% (10.5 to 24.8%)	1.2 (0.7 to 1.7)
Tracheal, bronchus, and lung cancer	Turkmenistan	2005	All Ages	0.0 (0.0 to 0.1)	11.5% (6.2 to 17.9%)	0.8 (0.4 to 1.2)
Tracheal, bronchus, and lung cancer	Turkmenistan	2010	All Ages	0.1 (0.0 to 0.1)	15.3% (8.8 to 22.3%)	0.9 (0.5 to 1.4)
Tracheal, bronchus, and lung cancer	Turkmenistan	2015	All Ages	0.1 (0.0 to 0.1)	16.0% (9.3 to 23.0%)	1.0 (0.6 to 1.4)
Tracheal, bronchus, and lung cancer	Turkmenistan	1990	Age-standardized		18.5% (11.4 to 26.1%)	2.7 (1.6 to 3.7)
Tracheal, bronchus, and lung cancer	Turkmenistan	1995	Age-standardized		17.4% (10.7 to 24.6%)	2.4 (1.5 to 3.4)
Tracheal, bronchus, and lung cancer	Turkmenistan	2000	Age-standardized		17.5% (10.6 to 25.1%)	2.3 (1.3 to 3.2)
Tracheal, bronchus, and lung cancer	Turkmenistan	2005	Age-standardized		11.6% (6.3 to 18.1%)	1.4 (0.8 to 2.2)
Tracheal, bronchus, and lung cancer	Turkmenistan	2010	Age-standardized		15.5% (8.9 to 22.5%)	1.6 (0.9 to 2.3)

Cause name	Location	Year	Age group	Number (in thousands)	PAF	Rate (per 100,000)
Tracheal, bronchus, and lung cancer	Turkmenistan	2015	Age-standardized		16.1% (9.4 to 23.2%)	1.5 (0.9 to 2.2)
Cardiovascular diseases	Turkmenistan	1990	All Ages	1.7 (1.4 to 1.9)	16.2% (13.9 to 18.6%)	45.4 (38.8 to 52.3)
Cardiovascular diseases	Turkmenistan	1995	All Ages	2.1 (1.8 to 2.4)	16.1% (13.9 to 18.5%)	50.9 (44.0 to 58.3)
Cardiovascular diseases	Turkmenistan	2000	All Ages	2.4 (2.1 to 2.8)	16.4% (14.2 to 18.8%)	53.5 (46.3 to 61.4)
Cardiovascular diseases	Turkmenistan	2005	All Ages	2.1 (1.8 to 2.4)	13.5% (11.5 to 15.7%)	44.0 (36.9 to 51.1)
Cardiovascular diseases	Turkmenistan	2010	All Ages	2.4 (2.0 to 2.8)	15.1% (12.8 to 17.5%)	47.5 (40.2 to 54.9)
Cardiovascular diseases	Turkmenistan	2015	All Ages	2.5 (2.1 to 2.9)	15.3% (13.1 to 17.6%)	46.1 (39.4 to 53.4)
Cardiovascular diseases	Turkmenistan	1990	Age-standardized		15.5% (13.0 to 18.3%)	105.5 (88.6 to 124.3)
Cardiovascular diseases	Turkmenistan	1995	Age-standardized		15.3% (12.9 to 17.9%)	117.1 (98.7 to 137.7)
Cardiovascular diseases	Turkmenistan	2000	Age-standardized		15.3% (12.9 to 17.9%)	118.0 (99.3 to 138.6)
Cardiovascular diseases	Turkmenistan	2005	Age-standardized		12.7% (10.3 to 15.1%)	91.4 (74.7 to 109.4)
Cardiovascular diseases	Turkmenistan	2010	Age-standardized		14.2% (11.7 to 16.8%)	93.4 (77.1 to 110.8)
Cardiovascular diseases	Turkmenistan	2015	Age-standardized		14.2% (11.8 to 16.9%)	86.9 (71.6 to 103.4)
Ischemic heart disease	Turkmenistan	1990	All Ages	1.3 (1.0 to 1.5)	18.3% (15.2 to 21.7%)	34.1 (28.2 to 40.4)
Ischemic heart disease	Turkmenistan	1995	All Ages	1.6 (1.4 to 1.9)	18.3% (15.3 to 21.5%)	38.9 (32.6 to 45.9)
Ischemic heart disease	Turkmenistan	2000	All Ages	1.9 (1.6 to 2.2)	18.6% (15.5 to 21.7%)	41.3 (34.4 to 48.7)
Ischemic heart disease	Turkmenistan	2005	All Ages	1.6 (1.3 to 2.0)	15.5% (12.7 to 18.3%)	34.2 (27.9 to 41.0)
Ischemic heart disease	Turkmenistan	2010	All Ages	1.8 (1.5 to 2.1)	17.1% (14.0 to 20.3%)	35.6 (29.2 to 42.6)
Ischemic heart disease	Turkmenistan	2015	All Ages	1.8 (1.5 to 2.2)	17.3% (14.2 to 20.5%)	34.2 (28.2 to 41.0)
Ischemic heart disease	Turkmenistan	1990	Age-standardized		17.2% (13.7 to 20.9%)	80.0 (63.9 to 97.2)
Ischemic heart disease	Turkmenistan	1995	Age-standardized		16.9% (13.5 to 20.5%)	90.2 (72.2 to 109.2)
Ischemic heart disease	Turkmenistan	2000	Age-standardized		17.0% (13.7 to 20.5%)	91.4 (73.6 to 110.4)
Ischemic heart disease	Turkmenistan	2005	Age-standardized		14.2% (11.1 to 17.4%)	71.3 (55.8 to 87.8)
Ischemic heart disease	Turkmenistan	2010	Age-standardized		15.9% (12.5 to 19.3%)	70.9 (55.6 to 87.0)
Ischemic heart disease	Turkmenistan	2015	Age-standardized		15.8% (12.3 to 19.4%)	65.5 (51.3 to 80.4)
Ischemic stroke	Turkmenistan	1990	All Ages	0.0 (0.0 to 0.1)	12.7% (9.3 to 16.4%)	1.0 (0.7 to 1.3)
Ischemic stroke	Turkmenistan	1995	All Ages	0.0 (0.0 to 0.1)	12.4% (9.0 to 15.9%)	0.9 (0.7 to 1.3)
Ischemic stroke	Turkmenistan	2000	All Ages	0.0 (0.0 to 0.1)	12.6% (9.2 to 16.1%)	1.0 (0.7 to 1.3)
Ischemic stroke	Turkmenistan	2005	All Ages	0.0 (0.0 to 0.1)	10.0% (6.8 to 13.4%)	0.8 (0.5 to 1.1)
Ischemic stroke	Turkmenistan	2010	All Ages	0.1 (0.0 to 0.1)	11.5% (8.2 to 15.2%)	0.9 (0.6 to 1.3)
Ischemic stroke	Turkmenistan	2015	All Ages	0.1 (0.0 to 0.1)	11.7% (8.3 to 15.5%)	0.9 (0.6 to 1.3)
Ischemic stroke	Turkmenistan	1990	Age-standardized		12.2% (8.6 to 16.4%)	2.6 (1.8 to 3.6)
Ischemic stroke	Turkmenistan	1995	Age-standardized		11.9% (8.1 to 15.9%)	2.7 (1.8 to 3.7)
Ischemic stroke	Turkmenistan	2000	Age-standardized		11.9% (8.1 to 16.0%)	2.7 (1.8 to 3.9)
Ischemic stroke	Turkmenistan	2005	Age-standardized		9.5% (6.0 to 13.6%)	2.0 (1.2 to 3.0)
Ischemic stroke	Turkmenistan	2010	Age-standardized		11.1% (7.5 to 15.2%)	2.2 (1.4 to 3.2)

Cause name	Location	Year	Age group	Number (in thousands)	PAF	Rate (per 100,000)
Ischemic stroke	Turkmenistan	2015	Age-standardized		11.2% (7.6 to 15.4%)	2.2 (1.4 to 3.2)
Hemorrhagic stroke	Turkmenistan	1990	All Ages	0.4 (0.3 to 0.5)	15.4% (12.4 to 18.3%)	10.4 (8.4 to 12.5)
Hemorrhagic stroke	Turkmenistan	1995	All Ages	0.5 (0.4 to 0.6)	15.0% (12.0 to 17.7%)	11.0 (8.9 to 13.4)
Hemorrhagic stroke	Turkmenistan	2000	All Ages	0.5 (0.4 to 0.6)	15.1% (12.3 to 18.0%)	11.3 (9.0 to 13.6)
Hemorrhagic stroke	Turkmenistan	2005	All Ages	0.4 (0.3 to 0.5)	12.1% (9.5 to 14.8%)	9.1 (7.0 to 11.4)
Hemorrhagic stroke	Turkmenistan	2010	All Ages	0.6 (0.4 to 0.7)	14.1% (11.4 to 16.9%)	10.9 (8.7 to 13.4)
Hemorrhagic stroke	Turkmenistan	2015	All Ages	0.6 (0.5 to 0.7)	14.5% (11.7 to 17.4%)	11.0 (8.8 to 13.4)
Hemorrhagic stroke	Turkmenistan	1990	Age-standardized		14.6% (11.7 to 17.5%)	22.9 (18.2 to 27.9)
Hemorrhagic stroke	Turkmenistan	1995	Age-standardized		14.2% (11.3 to 16.9%)	24.3 (19.3 to 29.5)
Hemorrhagic stroke	Turkmenistan	2000	Age-standardized		14.2% (11.3 to 17.3%)	23.8 (18.9 to 29.3)
Hemorrhagic stroke	Turkmenistan	2005	Age-standardized		11.3% (8.6 to 14.1%)	18.1 (13.5 to 23.1)
Hemorrhagic stroke	Turkmenistan	2010	Age-standardized		13.2% (10.3 to 16.0%)	20.3 (15.8 to 25.2)
Hemorrhagic stroke	Turkmenistan	2015	Age-standardized		13.3% (10.5 to 16.2%)	19.2 (15.0 to 23.7)
Chronic obstructive pulmonary disease	Turkmenistan	1990	All Ages	0.2 (0.1 to 0.3)	23.9% (13.9 to 34.7%)	5.4 (3.0 to 7.9)
Chronic obstructive pulmonary disease	Turkmenistan	1995	All Ages	0.2 (0.1 to 0.3)	22.9% (13.1 to 33.4%)	4.9 (2.8 to 7.4)
Chronic obstructive pulmonary disease	Turkmenistan	2000	All Ages	0.2 (0.1 to 0.4)	23.0% (12.9 to 33.5%)	5.2 (2.8 to 7.8)
Chronic obstructive pulmonary disease	Turkmenistan	2005	All Ages	0.1 (0.1 to 0.2)	17.0% (8.2 to 26.7%)	3.2 (1.5 to 5.1)
Chronic obstructive pulmonary disease	Turkmenistan	2010	All Ages	0.1 (0.1 to 0.2)	21.0% (11.2 to 31.4%)	2.4 (1.3 to 3.6)
Chronic obstructive pulmonary disease	Turkmenistan	2015	All Ages	0.1 (0.1 to 0.1)	21.6% (11.7 to 32.0%)	1.9 (1.0 to 2.8)
Chronic obstructive pulmonary disease	Turkmenistan	1990	Age-standardized		24.7% (14.3 to 35.9%)	13.2 (7.4 to 19.6)
Chronic obstructive pulmonary disease	Turkmenistan	1995	Age-standardized		23.6% (13.4 to 34.4%)	12.0 (6.7 to 18.0)
Chronic obstructive pulmonary disease	Turkmenistan	2000	Age-standardized		23.6% (13.3 to 34.6%)	12.2 (6.7 to 18.2)
Chronic obstructive pulmonary disease	Turkmenistan	2005	Age-standardized		17.5% (8.4 to 27.5%)	7.0 (3.3 to 11.1)
Chronic obstructive pulmonary disease	Turkmenistan	2010	Age-standardized		21.6% (11.6 to 32.3%)	4.9 (2.6 to 7.4)
Chronic obstructive pulmonary disease	Turkmenistan	2015	Age-standardized		22.1% (11.9 to 32.6%)	3.6 (1.9 to 5.4)
All causes	Uzbekistan	1990	All Ages	15.9 (13.8 to 18.1)	11.1% (9.7 to 12.6%)	77.7 (67.2 to 88.1)
All causes	Uzbekistan	1995	All Ages	17.4 (15.0 to 19.8)	10.7% (9.4 to 12.1%)	76.3 (65.9 to 87.2)
All causes	Uzbekistan	2000	All Ages	16.9 (14.6 to 19.3)	10.3% (9.0 to 11.7%)	68.6 (59.1 to 78.4)
All causes	Uzbekistan	2005	All Ages	16.1 (14.0 to 18.6)	9.4% (8.1 to 10.6%)	61.9 (53.6 to 71.4)
All causes	Uzbekistan	2010	All Ages	16.9 (14.4 to 19.6)	9.6% (8.3 to 11.0%)	60.8 (51.7 to 70.4)
All causes	Uzbekistan	2015	All Ages	17.5 (15.1 to 20.3)	9.8% (8.4 to 11.2%)	58.6 (50.5 to 67.7)
All causes	Uzbekistan	1990	Age-standardized		11.4% (9.8 to 13.1%)	133.5 (114.4 to 155.1)
All causes	Uzbekistan	1995	Age-standardized		11.0% (9.4 to 12.7%)	138.3 (117.7 to 161.0)
All causes	Uzbekistan	2000	Age-standardized		10.7% (9.1 to 12.4%)	130.7 (111.9 to 151.0)
All causes	Uzbekistan	2005	Age-standardized		9.8% (8.3 to 11.3%)	114.0 (96.7 to 132.0)
All causes	Uzbekistan	2010	Age-standardized		10.1% (8.6 to 11.8%)	104.7 (88.2 to 122.4)

Cause name	Location	Year	Age group	Number (in thousands)	PAF	Rate (per 100,000)
All causes	Uzbekistan	2015	Age-standardized		10.3% (8.7 to 12.0%)	98.3 (83.0 to 116.2)
Lower respiratory infections	Uzbekistan	1990	All Ages	5.2 (3.8 to 6.6)	30.1% (22.6 to 37.2%)	25.1 (18.5 to 32.1)
Lower respiratory infections	Uzbekistan	1995	All Ages	5.2 (3.7 to 6.7)	27.4% (20.2 to 34.9%)	22.6 (16.3 to 29.4)
Lower respiratory infections	Uzbekistan	2000	All Ages	3.8 (2.7 to 5.2)	26.4% (19.5 to 34.4%)	15.6 (11.0 to 21.0)
Lower respiratory infections	Uzbekistan	2005	All Ages	3.0 (2.0 to 4.3)	23.2% (16.1 to 31.5%)	11.5 (7.6 to 16.6)
Lower respiratory infections	Uzbekistan	2010	All Ages	2.9 (1.9 to 4.0)	25.2% (17.7 to 33.2%)	10.2 (6.8 to 14.3)
Lower respiratory infections	Uzbekistan	2015	All Ages	2.4 (1.7 to 3.3)	26.2% (19.1 to 33.8%)	8.1 (5.5 to 11.2)
Lower respiratory infections	Uzbekistan	1990	Age-standardized		30.1% (22.6 to 37.2%)	17.4 (12.9 to 22.0)
Lower respiratory infections	Uzbekistan	1995	Age-standardized		27.4% (20.2 to 34.9%)	17.9 (12.9 to 23.2)
Lower respiratory infections	Uzbekistan	2000	Age-standardized		26.4% (19.5 to 34.4%)	15.0 (10.6 to 20.1)
Lower respiratory infections	Uzbekistan	2005	Age-standardized		23.2% (16.1 to 31.5%)	11.9 (7.9 to 16.8)
Lower respiratory infections	Uzbekistan	2010	Age-standardized		25.2% (17.7 to 33.2%)	10.3 (6.9 to 14.1)
Lower respiratory infections	Uzbekistan	2015	Age-standardized		26.2% (19.1 to 33.8%)	8.4 (5.8 to 11.5)
Tracheal, bronchus, and lung cancer	Uzbekistan	1990	All Ages	0.4 (0.3 to 0.5)	22.3% (14.4 to 30.4%)	1.9 (1.2 to 2.6)
Tracheal, bronchus, and lung cancer	Uzbekistan	1995	All Ages	0.3 (0.2 to 0.5)	19.9% (12.6 to 27.6%)	1.4 (0.9 to 2.0)
Tracheal, bronchus, and lung cancer	Uzbekistan	2000	All Ages	0.3 (0.2 to 0.4)	19.0% (11.8 to 26.3%)	1.1 (0.7 to 1.6)
Tracheal, bronchus, and lung cancer	Uzbekistan	2005	All Ages	0.3 (0.1 to 0.4)	16.4% (9.8 to 23.5%)	1.0 (0.6 to 1.4)
Tracheal, bronchus, and lung cancer	Uzbekistan	2010	All Ages	0.3 (0.2 to 0.5)	18.0% (11.1 to 25.2%)	1.2 (0.7 to 1.7)
Tracheal, bronchus, and lung cancer	Uzbekistan	2015	All Ages	0.4 (0.2 to 0.6)	18.9% (11.7 to 26.3%)	1.3 (0.8 to 1.9)
Tracheal, bronchus, and lung cancer	Uzbekistan	1990	Age-standardized		22.4% (14.5 to 30.6%)	3.7 (2.4 to 5.1)
Tracheal, bronchus, and lung cancer	Uzbekistan	1995	Age-standardized		20.1% (12.7 to 27.8%)	2.8 (1.8 to 3.9)
Tracheal, bronchus, and lung cancer	Uzbekistan	2000	Age-standardized		19.1% (11.9 to 26.5%)	2.2 (1.4 to 3.0)
Tracheal, bronchus, and lung cancer	Uzbekistan	2005	Age-standardized		16.6% (9.9 to 23.7%)	1.7 (1.0 to 2.5)
Tracheal, bronchus, and lung cancer	Uzbekistan	2010	Age-standardized		18.1% (11.2 to 25.4%)	1.9 (1.2 to 2.8)
Tracheal, bronchus, and lung cancer	Uzbekistan	2015	Age-standardized		19.0% (11.8 to 26.4%)	2.0 (1.2 to 2.9)
Cardiovascular diseases	Uzbekistan	1990	All Ages	9.2 (7.7 to 10.9)	15.0% (12.6 to 17.6%)	44.6 (37.5 to 53.2)
Cardiovascular diseases	Uzbekistan	1995	All Ages	10.7 (9.0 to 12.5)	14.6% (12.3 to 17.0%)	46.8 (39.6 to 54.9)
Cardiovascular diseases	Uzbekistan	2000	All Ages	11.6 (9.9 to 13.4)	14.4% (12.4 to 16.7%)	47.1 (40.2 to 54.5)
Cardiovascular diseases	Uzbekistan	2005	All Ages	11.9 (10.1 to 13.9)	13.4% (11.4 to 15.6%)	45.8 (38.8 to 53.3)
Cardiovascular diseases	Uzbekistan	2010	All Ages	12.7 (10.6 to 15.1)	14.0% (11.8 to 16.5%)	45.8 (38.1 to 54.1)
Cardiovascular diseases	Uzbekistan	2015	All Ages	13.6 (11.4 to 16.1)	14.2% (12.0 to 16.8%)	45.5 (38.2 to 53.9)
Cardiovascular diseases	Uzbekistan	1990	Age-standardized		14.7% (12.2 to 17.5%)	98.7 (82.0 to 119.2)
Cardiovascular diseases	Uzbekistan	1995	Age-standardized		14.1% (11.7 to 16.8%)	105.3 (87.4 to 125.4)
Cardiovascular diseases	Uzbekistan	2000	Age-standardized		13.8% (11.5 to 16.4%)	103.3 (85.9 to 122.2)
Cardiovascular diseases	Uzbekistan	2005	Age-standardized		12.9% (10.7 to 15.3%)	93.2 (76.4 to 110.9)
Cardiovascular diseases	Uzbekistan	2010	Age-standardized		13.5% (11.1 to 16.1%)	85.5 (70.1 to 102.7)

Cause name	Location	Year	Age group	Number (in thousands)	PAF	Rate (per 100,000)
Cardiovascular diseases	Uzbekistan	2015	Age-standardized		13.7% (11.2 to 16.4%)	81.2 (66.7 to 98.2)
Ischemic heart disease	Uzbekistan	1990	All Ages	6.6 (5.3 to 8.2)	18.4% (14.7 to 22.8%)	32.1 (25.6 to 39.8)
Ischemic heart disease	Uzbekistan	1995	All Ages	7.7 (6.2 to 9.3)	18.0% (14.6 to 21.8%)	33.7 (27.4 to 40.9)
Ischemic heart disease	Uzbekistan	2000	All Ages	8.4 (6.9 to 10.2)	17.9% (14.6 to 21.5%)	34.4 (28.0 to 41.4)
Ischemic heart disease	Uzbekistan	2005	All Ages	8.9 (7.2 to 10.8)	16.7% (13.6 to 19.9%)	34.2 (27.7 to 41.3)
Ischemic heart disease	Uzbekistan	2010	All Ages	9.6 (7.6 to 11.6)	17.2% (13.9 to 20.9%)	34.3 (27.4 to 41.8)
Ischemic heart disease	Uzbekistan	2015	All Ages	10.3 (8.2 to 12.6)	17.5% (14.0 to 21.3%)	34.3 (27.4 to 42.0)
Ischemic heart disease	Uzbekistan	1990	Age-standardized		17.8% (14.0 to 22.4%)	71.4 (56.1 to 89.8)
Ischemic heart disease	Uzbekistan	1995	Age-standardized		17.1% (13.6 to 21.4%)	76.3 (60.0 to 94.2)
Ischemic heart disease	Uzbekistan	2000	Age-standardized		16.8% (13.2 to 20.8%)	75.4 (59.1 to 92.6)
Ischemic heart disease	Uzbekistan	2005	Age-standardized		15.8% (12.4 to 19.4%)	69.5 (54.1 to 85.3)
Ischemic heart disease	Uzbekistan	2010	Age-standardized		16.5% (13.0 to 20.3%)	64.3 (49.9 to 79.7)
Ischemic heart disease	Uzbekistan	2015	Age-standardized		16.6% (12.9 to 20.6%)	61.4 (47.1 to 76.9)
Ischemic stroke	Uzbekistan	1990	All Ages	1.2 (0.9 to 1.5)	15.2% (12.0 to 18.5%)	5.6 (4.3 to 7.1)
Ischemic stroke	Uzbekistan	1995	All Ages	1.4 (1.0 to 1.7)	14.5% (11.2 to 17.6%)	5.9 (4.5 to 7.3)
Ischemic stroke	Uzbekistan	2000	All Ages	1.5 (1.1 to 1.8)	14.1% (11.0 to 17.1%)	6.0 (4.6 to 7.4)
Ischemic stroke	Uzbekistan	2005	All Ages	1.4 (1.0 to 1.8)	12.9% (9.8 to 15.8%)	5.4 (4.0 to 6.8)
Ischemic stroke	Uzbekistan	2010	All Ages	1.5 (1.1 to 1.9)	13.5% (10.5 to 16.6%)	5.2 (4.0 to 6.7)
Ischemic stroke	Uzbekistan	2015	All Ages	1.6 (1.2 to 2.0)	13.8% (10.7 to 17.1%)	5.2 (3.9 to 6.7)
Ischemic stroke	Uzbekistan	1990	Age-standardized		14.8% (11.5 to 18.2%)	12.6 (9.4 to 15.9)
Ischemic stroke	Uzbekistan	1995	Age-standardized		13.9% (10.6 to 17.2%)	13.6 (10.0 to 17.1)
Ischemic stroke	Uzbekistan	2000	Age-standardized		13.5% (10.1 to 16.8%)	13.4 (9.9 to 17.0)
Ischemic stroke	Uzbekistan	2005	Age-standardized		12.4% (9.1 to 15.6%)	11.3 (8.2 to 14.5)
Ischemic stroke	Uzbekistan	2010	Age-standardized		13.0% (9.7 to 16.3%)	10.1 (7.6 to 13.1)
Ischemic stroke	Uzbekistan	2015	Age-standardized		13.3% (10.0 to 16.6%)	9.6 (7.0 to 12.5)
Hemorrhagic stroke	Uzbekistan	1990	All Ages	1.4 (1.1 to 1.8)	16.4% (13.4 to 19.4%)	6.9 (5.3 to 8.5)
Hemorrhagic stroke	Uzbekistan	1995	All Ages	1.6 (1.3 to 2.0)	15.6% (12.5 to 18.6%)	7.2 (5.6 to 8.8)
Hemorrhagic stroke	Uzbekistan	2000	All Ages	1.7 (1.3 to 2.1)	15.1% (12.0 to 18.1%)	6.8 (5.3 to 8.4)
Hemorrhagic stroke	Uzbekistan	2005	All Ages	1.6 (1.2 to 2.0)	13.7% (10.8 to 16.7%)	6.2 (4.8 to 7.7)
Hemorrhagic stroke	Uzbekistan	2010	All Ages	1.7 (1.3 to 2.1)	14.5% (11.7 to 17.4%)	6.2 (4.8 to 7.7)
Hemorrhagic stroke	Uzbekistan	2015	All Ages	1.8 (1.4 to 2.3)	14.9% (12.0 to 18.0%)	6.0 (4.7 to 7.5)
Hemorrhagic stroke	Uzbekistan	1990	Age-standardized		15.8% (12.8 to 18.8%)	14.7 (11.1 to 18.4)
Hemorrhagic stroke	Uzbekistan	1995	Age-standardized		14.9% (11.7 to 17.9%)	15.4 (11.7 to 19.1)
Hemorrhagic stroke	Uzbekistan	2000	Age-standardized		14.3% (11.0 to 17.3%)	14.5 (10.9 to 18.2)
Hemorrhagic stroke	Uzbekistan	2005	Age-standardized		13.0% (10.0 to 16.1%)	12.4 (9.1 to 15.7)
Hemorrhagic stroke	Uzbekistan	2010	Age-standardized		13.8% (10.8 to 16.9%)	11.1 (8.4 to 14.0)

Cause name	Location	Year	Age group	Number (in thousands)	PAF	Rate (per 100,000)
Hemorrhagic stroke	Uzbekistan	2015	Age-standardized		14.0% (11.0 to 17.2%)	10.3 (7.7 to 13.0)
Chronic obstructive pulmonary disease	Uzbekistan	1990	All Ages	1.2 (0.8 to 1.8)	28.2% (17.6 to 39.9%)	6.1 (3.7 to 8.7)
Chronic obstructive pulmonary disease	Uzbekistan	1995	All Ages	1.2 (0.7 to 1.8)	26.0% (15.6 to 37.6%)	5.4 (3.2 to 8.0)
Chronic obstructive pulmonary disease	Uzbekistan	2000	All Ages	1.2 (0.7 to 1.7)	25.1% (14.5 to 36.4%)	4.7 (2.7 to 6.9)
Chronic obstructive pulmonary disease	Uzbekistan	2005	All Ages	0.9 (0.5 to 1.4)	22.6% (12.9 to 33.5%)	3.6 (2.0 to 5.2)
Chronic obstructive pulmonary disease	Uzbekistan	2010	All Ages	1.0 (0.6 to 1.5)	24.2% (13.9 to 35.5%)	3.7 (2.1 to 5.3)
Chronic obstructive pulmonary disease	Uzbekistan	2015	All Ages	1.1 (0.6 to 1.6)	25.0% (14.6 to 36.5%)	3.6 (2.1 to 5.3)
Chronic obstructive pulmonary disease	Uzbekistan	1990	Age-standardized		28.4% (17.8 to 40.2%)	13.7 (8.3 to 19.7)
Chronic obstructive pulmonary disease	Uzbekistan	1995	Age-standardized		26.2% (15.8 to 38.0%)	12.3 (7.3 to 18.0)
Chronic obstructive pulmonary disease	Uzbekistan	2000	Age-standardized		25.4% (14.7 to 36.7%)	10.2 (5.9 to 15.0)
Chronic obstructive pulmonary disease	Uzbekistan	2005	Age-standardized		22.8% (13.0 to 33.8%)	7.2 (4.0 to 10.7)
Chronic obstructive pulmonary disease	Uzbekistan	2010	Age-standardized		24.4% (14.1 to 35.9%)	6.9 (4.0 to 10.0)
Chronic obstructive pulmonary disease	Uzbekistan	2015	Age-standardized		25.2% (14.7 to 36.7%)	6.6 (3.8 to 9.6)
All causes	Colombia	1990	All Ages	7.8 (6.6 to 9.1)	4.5% (3.9 to 5.3%)	22.6 (19.3 to 26.3)
All causes	Colombia	1995	All Ages	8.5 (7.2 to 10.0)	4.6% (3.9 to 5.3%)	22.8 (19.2 to 26.6)
All causes	Colombia	2000	All Ages	9.0 (7.6 to 10.5)	4.6% (3.9 to 5.4%)	22.3 (18.7 to 25.9)
All causes	Colombia	2005	All Ages	10.1 (8.4 to 11.8)	5.1% (4.2 to 6.0%)	23.2 (19.4 to 27.2)
All causes	Colombia	2010	All Ages	9.8 (8.0 to 11.7)	4.6% (3.8 to 5.6%)	21.3 (17.3 to 25.5)
All causes	Colombia	2015	All Ages	10.9 (8.9 to 13.1)	4.9% (4.0 to 5.9%)	22.6 (18.5 to 27.1)
All causes	Colombia	1990	Age-standardized		5.7% (4.8 to 6.6%)	49.3 (41.6 to 57.7)
All causes	Colombia	1995	Age-standardized		5.6% (4.7 to 6.6%)	47.4 (39.7 to 55.6)
All causes	Colombia	2000	Age-standardized		5.5% (4.6 to 6.5%)	43.8 (36.4 to 51.7)
All causes	Colombia	2005	Age-standardized		5.8% (4.8 to 6.9%)	42.0 (34.6 to 50.2)
All causes	Colombia	2010	Age-standardized		5.1% (4.1 to 6.2%)	34.3 (27.3 to 41.7)
All causes	Colombia	2015	Age-standardized		5.2% (4.2 to 6.4%)	31.7 (25.4 to 38.6)
Lower respiratory infections	Colombia	1990	All Ages	1.4 (0.7 to 2.3)	17.5% (9.1 to 27.7%)	4.2 (2.1 to 6.6)
Lower respiratory infections	Colombia	1995	All Ages	1.4 (0.7 to 2.3)	17.0% (8.5 to 27.2%)	3.8 (1.9 to 6.0)
Lower respiratory infections	Colombia	2000	All Ages	1.3 (0.6 to 2.1)	17.0% (8.6 to 27.4%)	3.2 (1.6 to 5.1)
Lower respiratory infections	Colombia	2005	All Ages	1.4 (0.7 to 2.2)	17.7% (9.2 to 27.8%)	3.3 (1.7 to 5.2)
Lower respiratory infections	Colombia	2010	All Ages	1.2 (0.5 to 2.1)	14.3% (6.1 to 24.8%)	2.6 (1.1 to 4.5)
Lower respiratory infections	Colombia	2015	All Ages	1.3 (0.6 to 2.1)	15.2% (6.9 to 25.4%)	2.6 (1.2 to 4.4)
Lower respiratory infections	Colombia	1990	Age-standardized		17.5% (9.1 to 27.7%)	6.2 (3.2 to 9.9)
Lower respiratory infections	Colombia	1995	Age-standardized		17.0% (8.5 to 27.2%)	5.8 (2.9 to 9.4)
Lower respiratory infections	Colombia	2000	Age-standardized		17.0% (8.6 to 27.4%)	5.2 (2.6 to 8.3)
Lower respiratory infections	Colombia	2005	Age-standardized		17.7% (9.2 to 27.8%)	5.2 (2.8 to 8.2)
Lower respiratory infections	Colombia	2010	Age-standardized		14.3% (6.1 to 24.8%)	3.9 (1.7 to 6.8)

Cause name	Location	Year	Age group	Number (in thousands)	PAF	Rate (per 100,000)
Lower respiratory infections	Colombia	2015	Age-standardized		15.2% (6.9 to 25.4%)	3.6 (1.6 to 6.1)
Tracheal, bronchus, and lung cancer	Colombia	1990	All Ages	0.3 (0.2 to 0.5)	12.2% (6.7 to 18.7%)	0.9 (0.5 to 1.3)
Tracheal, bronchus, and lung cancer	Colombia	1995	All Ages	0.4 (0.2 to 0.5)	11.9% (6.5 to 18.1%)	0.9 (0.5 to 1.4)
Tracheal, bronchus, and lung cancer	Colombia	2000	All Ages	0.4 (0.2 to 0.6)	11.8% (6.5 to 18.1%)	1.0 (0.5 to 1.5)
Tracheal, bronchus, and lung cancer	Colombia	2005	All Ages	0.5 (0.3 to 0.7)	12.4% (6.8 to 18.8%)	1.1 (0.6 to 1.7)
Tracheal, bronchus, and lung cancer	Colombia	2010	All Ages	0.5 (0.2 to 0.7)	9.9% (5.1 to 15.7%)	1.0 (0.5 to 1.5)
Tracheal, bronchus, and lung cancer	Colombia	2015	All Ages	0.6 (0.3 to 0.8)	10.6% (5.6 to 16.6%)	1.1 (0.6 to 1.8)
Tracheal, bronchus, and lung cancer	Colombia	1990	Age-standardized		12.3% (6.7 to 18.7%)	2.0 (1.1 to 3.0)
Tracheal, bronchus, and lung cancer	Colombia	1995	Age-standardized		11.9% (6.5 to 18.1%)	2.0 (1.1 to 3.0)
Tracheal, bronchus, and lung cancer	Colombia	2000	Age-standardized		11.9% (6.5 to 18.2%)	1.9 (1.0 to 3.0)
Tracheal, bronchus, and lung cancer	Colombia	2005	Age-standardized		12.4% (6.8 to 18.8%)	1.9 (1.1 to 2.9)
Tracheal, bronchus, and lung cancer	Colombia	2010	Age-standardized		9.9% (5.1 to 15.8%)	1.5 (0.8 to 2.4)
Tracheal, bronchus, and lung cancer	Colombia	2015	Age-standardized		10.6% (5.6 to 16.6%)	1.5 (0.8 to 2.4)
Cardiovascular diseases	Colombia	1990	All Ages	5.1 (4.3 to 5.9)	11.6% (9.8 to 13.4%)	14.9 (12.6 to 17.3)
Cardiovascular diseases	Colombia	1995	All Ages	5.5 (4.7 to 6.5)	11.3% (9.5 to 13.2%)	14.8 (12.4 to 17.3)
Cardiovascular diseases	Colombia	2000	All Ages	5.7 (4.8 to 6.7)	11.2% (9.4 to 13.1%)	14.2 (11.9 to 16.5)
Cardiovascular diseases	Colombia	2005	All Ages	6.2 (5.2 to 7.3)	11.3% (9.4 to 13.2%)	14.3 (12.0 to 16.8)
Cardiovascular diseases	Colombia	2010	All Ages	6.2 (5.0 to 7.4)	10.2% (8.3 to 12.2%)	13.4 (10.9 to 16.0)
Cardiovascular diseases	Colombia	2015	All Ages	6.8 (5.5 to 8.3)	10.4% (8.4 to 12.4%)	14.2 (11.4 to 17.1)
Cardiovascular diseases	Colombia	1990	Age-standardized		11.0% (9.1 to 13.0%)	34.2 (28.2 to 40.6)
Cardiovascular diseases	Colombia	1995	Age-standardized		10.8% (8.9 to 12.8%)	31.8 (26.1 to 37.7)
Cardiovascular diseases	Colombia	2000	Age-standardized		10.7% (8.7 to 12.7%)	28.0 (22.8 to 33.5)
Cardiovascular diseases	Colombia	2005	Age-standardized		10.7% (8.7 to 12.8%)	25.7 (20.8 to 30.9)
Cardiovascular diseases	Colombia	2010	Age-standardized		9.8% (7.7 to 11.9%)	21.3 (16.7 to 25.9)
Cardiovascular diseases	Colombia	2015	Age-standardized		10.0% (7.8 to 12.1%)	19.5 (15.3 to 23.9)
Ischemic heart disease	Colombia	1990	All Ages	3.7 (3.0 to 4.4)	15.6% (12.8 to 18.6%)	10.8 (8.7 to 12.9)
Ischemic heart disease	Colombia	1995	All Ages	4.0 (3.2 to 4.8)	15.3% (12.4 to 18.2%)	10.7 (8.6 to 12.8)
Ischemic heart disease	Colombia	2000	All Ages	4.1 (3.3 to 4.9)	15.1% (12.1 to 18.1%)	10.2 (8.2 to 12.2)
Ischemic heart disease	Colombia	2005	All Ages	4.5 (3.6 to 5.5)	15.0% (11.8 to 18.1%)	10.5 (8.2 to 12.6)
Ischemic heart disease	Colombia	2010	All Ages	4.6 (3.6 to 5.7)	13.5% (10.3 to 16.5%)	10.1 (7.7 to 12.5)
Ischemic heart disease	Colombia	2015	All Ages	5.2 (4.0 to 6.5)	13.7% (10.5 to 16.8%)	10.8 (8.2 to 13.4)
Ischemic heart disease	Colombia	1990	Age-standardized		14.7% (11.4 to 17.9%)	25.0 (19.5 to 30.6)
Ischemic heart disease	Colombia	1995	Age-standardized		14.4% (11.2 to 17.6%)	23.0 (17.9 to 28.1)
Ischemic heart disease	Colombia	2000	Age-standardized		14.2% (11.0 to 17.5%)	20.2 (15.7 to 25.0)
Ischemic heart disease	Colombia	2005	Age-standardized		14.2% (10.8 to 17.7%)	18.8 (14.3 to 23.5)
Ischemic heart disease	Colombia	2010	Age-standardized		12.8% (9.5 to 16.2%)	16.0 (11.8 to 20.4)

Cause name	Location	Year	Age group	Number (in thousands)	PAF	Rate (per 100,000)
Ischemic heart disease	Colombia	2015	Age-standardized		13.1% (9.6 to 16.5%)	14.8 (10.9 to 18.9)
Ischemic stroke	Colombia	1990	All Ages	0.6 (0.4 to 0.7)	10.8% (7.8 to 13.9%)	1.6 (1.1 to 2.1)
Ischemic stroke	Colombia	1995	All Ages	0.6 (0.4 to 0.8)	10.5% (7.4 to 13.7%)	1.5 (1.1 to 2.0)
Ischemic stroke	Colombia	2000	All Ages	0.6 (0.4 to 0.7)	10.3% (7.0 to 13.7%)	1.4 (0.9 to 1.8)
Ischemic stroke	Colombia	2005	All Ages	0.6 (0.4 to 0.8)	10.3% (7.0 to 14.0%)	1.3 (0.9 to 1.8)
Ischemic stroke	Colombia	2010	All Ages	0.5 (0.3 to 0.8)	9.1% (5.7 to 13.0%)	1.2 (0.7 to 1.7)
Ischemic stroke	Colombia	2015	All Ages	0.6 (0.4 to 0.8)	9.3% (6.0 to 13.3%)	1.2 (0.8 to 1.7)
Ischemic stroke	Colombia	1990	Age-standardized		10.4% (7.0 to 13.9%)	4.1 (2.7 to 5.5)
Ischemic stroke	Colombia	1995	Age-standardized		10.1% (6.8 to 13.7%)	3.6 (2.4 to 5.0)
Ischemic stroke	Colombia	2000	Age-standardized		10.0% (6.5 to 13.8%)	3.0 (2.0 to 4.2)
Ischemic stroke	Colombia	2005	Age-standardized		10.0% (6.5 to 14.1%)	2.7 (1.7 to 3.7)
Ischemic stroke	Colombia	2010	Age-standardized		8.9% (5.5 to 13.1%)	2.1 (1.2 to 3.0)
Ischemic stroke	Colombia	2015	Age-standardized		9.1% (5.6 to 13.4%)	1.8 (1.1 to 2.7)
Hemorrhagic stroke	Colombia	1990	All Ages	0.8 (0.7 to 1.0)	12.0% (9.6 to 14.6%)	2.5 (1.9 to 3.0)
Hemorrhagic stroke	Colombia	1995	All Ages	1.0 (0.8 to 1.2)	11.7% (9.2 to 14.3%)	2.6 (2.0 to 3.2)
Hemorrhagic stroke	Colombia	2000	All Ages	1.1 (0.8 to 1.3)	11.8% (9.2 to 14.4%)	2.6 (2.0 to 3.2)
Hemorrhagic stroke	Colombia	2005	All Ages	1.1 (0.8 to 1.3)	11.9% (9.3 to 14.5%)	2.5 (1.9 to 3.1)
Hemorrhagic stroke	Colombia	2010	All Ages	1.0 (0.7 to 1.3)	10.5% (7.8 to 13.2%)	2.2 (1.6 to 2.7)
Hemorrhagic stroke	Colombia	2015	All Ages	1.1 (0.8 to 1.4)	10.7% (8.0 to 13.5%)	2.2 (1.6 to 2.8)
Hemorrhagic stroke	Colombia	1990	Age-standardized		11.8% (9.1 to 14.6%)	5.1 (4.0 to 6.4)
Hemorrhagic stroke	Colombia	1995	Age-standardized		11.4% (8.7 to 14.2%)	5.1 (3.9 to 6.4)
Hemorrhagic stroke	Colombia	2000	Age-standardized		11.3% (8.5 to 14.1%)	4.8 (3.6 to 6.0)
Hemorrhagic stroke	Colombia	2005	Age-standardized		11.3% (8.5 to 14.3%)	4.2 (3.1 to 5.3)
Hemorrhagic stroke	Colombia	2010	Age-standardized		10.0% (7.2 to 13.1%)	3.2 (2.3 to 4.2)
Hemorrhagic stroke	Colombia	2015	Age-standardized		10.2% (7.3 to 13.3%)	2.9 (2.1 to 3.8)
Chronic obstructive pulmonary disease	Colombia	1990	All Ages	0.9 (0.4 to 1.5)	17.1% (8.2 to 27.1%)	2.7 (1.3 to 4.3)
Chronic obstructive pulmonary disease	Colombia	1995	All Ages	1.2 (0.6 to 1.9)	17.7% (8.3 to 28.1%)	3.3 (1.5 to 5.2)
Chronic obstructive pulmonary disease	Colombia	2000	All Ages	1.6 (0.8 to 2.6)	18.1% (8.5 to 28.7%)	3.9 (1.9 to 6.3)
Chronic obstructive pulmonary disease	Colombia	2005	All Ages	2.0 (1.0 to 3.1)	18.7% (8.9 to 29.5%)	4.6 (2.2 to 7.3)
Chronic obstructive pulmonary disease	Colombia	2010	All Ages	2.0 (0.9 to 3.3)	16.0% (7.0 to 26.6%)	4.3 (1.9 to 7.1)
Chronic obstructive pulmonary disease	Colombia	2015	All Ages	2.3 (1.0 to 3.6)	16.7% (7.6 to 27.4%)	4.7 (2.1 to 7.6)
Chronic obstructive pulmonary disease	Colombia	1990	Age-standardized		18.2% (8.8 to 28.9%)	6.9 (3.3 to 11.0)
Chronic obstructive pulmonary disease	Colombia	1995	Age-standardized		18.1% (8.5 to 28.7%)	7.8 (3.7 to 12.4)
Chronic obstructive pulmonary disease	Colombia	2000	Age-standardized		18.2% (8.6 to 28.9%)	8.7 (4.2 to 14.0)
Chronic obstructive pulmonary disease	Colombia	2005	Age-standardized		18.8% (9.0 to 29.6%)	9.2 (4.5 to 14.5)
Chronic obstructive pulmonary disease	Colombia	2010	Age-standardized		16.0% (7.1 to 26.7%)	7.5 (3.3 to 12.5)

Cause name	Location	Year	Age group	Number (in thousands)	PAF	Rate (per 100,000)
Chronic obstructive pulmonary disease	Colombia	2015	Age-standardized		16.8% (7.6 to 27.4%)	7.1 (3.3 to 11.4)
All causes	Costa Rica	1990	All Ages	0.6 (0.5 to 0.7)	5.2% (4.4 to 6.0%)	19.5 (16.4 to 22.7)
All causes	Costa Rica	1995	All Ages	0.7 (0.6 to 0.8)	5.0% (4.2 to 5.9%)	20.0 (16.8 to 23.4)
All causes	Costa Rica	2000	All Ages	0.8 (0.7 to 0.9)	5.0% (4.2 to 5.9%)	19.9 (16.6 to 23.5)
All causes	Costa Rica	2005	All Ages	0.8 (0.6 to 0.9)	4.6% (3.8 to 5.5%)	18.3 (15.1 to 21.8)
All causes	Costa Rica	2010	All Ages	0.7 (0.6 to 0.9)	3.8% (3.0 to 4.6%)	15.9 (12.6 to 19.4)
All causes	Costa Rica	2015	All Ages	1.0 (0.8 to 1.2)	4.6% (3.7 to 5.5%)	21.4 (17.5 to 25.8)
All causes	Costa Rica	1990	Age-standardized		6.0% (4.9 to 7.1%)	39.9 (32.9 to 47.3)
All causes	Costa Rica	1995	Age-standardized		5.6% (4.7 to 6.7%)	37.6 (31.1 to 44.7)
All causes	Costa Rica	2000	Age-standardized		5.5% (4.5 to 6.5%)	34.3 (28.1 to 40.9)
All causes	Costa Rica	2005	Age-standardized		4.9% (4.0 to 5.9%)	27.8 (22.6 to 33.4)
All causes	Costa Rica	2010	Age-standardized		3.9% (3.0 to 4.8%)	20.6 (16.2 to 25.4)
All causes	Costa Rica	2015	Age-standardized		4.6% (3.8 to 5.6%)	24.0 (19.4 to 29.0)
Lower respiratory infections	Costa Rica	1990	All Ages	0.1 (0.0 to 0.1)	17.6% (9.0 to 27.7%)	2.8 (1.4 to 4.5)
Lower respiratory infections	Costa Rica	1995	All Ages	0.1 (0.1 to 0.1)	17.4% (8.8 to 27.7%)	2.7 (1.4 to 4.4)
Lower respiratory infections	Costa Rica	2000	All Ages	0.1 (0.1 to 0.1)	18.0% (9.4 to 28.1%)	2.4 (1.2 to 3.8)
Lower respiratory infections	Costa Rica	2005	All Ages	0.1 (0.0 to 0.1)	17.2% (8.3 to 27.3%)	2.0 (1.0 to 3.2)
Lower respiratory infections	Costa Rica	2010	All Ages	0.1 (0.0 to 0.1)	12.6% (4.6 to 23.5%)	1.4 (0.5 to 2.7)
Lower respiratory infections	Costa Rica	2015	All Ages	0.1 (0.1 to 0.2)	16.8% (8.2 to 27.2%)	2.1 (1.0 to 3.5)
Lower respiratory infections	Costa Rica	1990	Age-standardized		17.6% (9.0 to 27.7%)	5.0 (2.5 to 7.9)
Lower respiratory infections	Costa Rica	1995	Age-standardized		17.4% (8.8 to 27.7%)	4.8 (2.4 to 7.6)
Lower respiratory infections	Costa Rica	2000	Age-standardized		18.0% (9.4 to 28.1%)	4.0 (2.1 to 6.3)
Lower respiratory infections	Costa Rica	2005	Age-standardized		17.2% (8.3 to 27.3%)	3.0 (1.5 to 4.8)
Lower respiratory infections	Costa Rica	2010	Age-standardized		12.6% (4.6 to 23.5%)	1.9 (0.7 to 3.6)
Lower respiratory infections	Costa Rica	2015	Age-standardized		16.8% (8.2 to 27.2%)	2.4 (1.1 to 4.0)
Tracheal, bronchus, and lung cancer	Costa Rica	1990	All Ages	0.0 (0.0 to 0.0)	12.3% (6.8 to 18.8%)	0.7 (0.4 to 1.1)
Tracheal, bronchus, and lung cancer	Costa Rica	1995	All Ages	0.0 (0.0 to 0.0)	12.2% (6.7 to 18.5%)	0.8 (0.4 to 1.2)
Tracheal, bronchus, and lung cancer	Costa Rica	2000	All Ages	0.0 (0.0 to 0.1)	12.6% (7.0 to 19.2%)	0.8 (0.5 to 1.3)
Tracheal, bronchus, and lung cancer	Costa Rica	2005	All Ages	0.0 (0.0 to 0.1)	12.0% (6.5 to 18.5%)	0.8 (0.4 to 1.2)
Tracheal, bronchus, and lung cancer	Costa Rica	2010	All Ages	0.0 (0.0 to 0.1)	8.7% (4.3 to 14.2%)	0.6 (0.3 to 1.0)
Tracheal, bronchus, and lung cancer	Costa Rica	2015	All Ages	0.1 (0.0 to 0.1)	11.7% (6.3 to 18.0%)	1.0 (0.5 to 1.6)
Tracheal, bronchus, and lung cancer	Costa Rica	1990	Age-standardized		12.3% (6.8 to 18.8%)	1.4 (0.8 to 2.1)
Tracheal, bronchus, and lung cancer	Costa Rica	1995	Age-standardized		12.2% (6.7 to 18.6%)	1.4 (0.8 to 2.1)
Tracheal, bronchus, and lung cancer	Costa Rica	2000	Age-standardized		12.7% (7.0 to 19.2%)	1.4 (0.8 to 2.2)
Tracheal, bronchus, and lung cancer	Costa Rica	2005	Age-standardized		12.0% (6.5 to 18.6%)	1.2 (0.7 to 1.8)
Tracheal, bronchus, and lung cancer	Costa Rica	2010	Age-standardized		8.7% (4.3 to 14.2%)	0.8 (0.4 to 1.3)

Cause name	Location	Year	Age group	Number (in thousands)	PAF	Rate (per 100,000)
Tracheal, bronchus, and lung cancer	Costa Rica	2015	Age-standardized		11.7% (6.3 to 18.0%)	1.1 (0.6 to 1.8)
Cardiovascular diseases	Costa Rica	1990	All Ages	0.4 (0.4 to 0.5)	12.1% (10.0 to 14.3%)	13.6 (11.3 to 16.1)
Cardiovascular diseases	Costa Rica	1995	All Ages	0.5 (0.4 to 0.6)	11.7% (9.7 to 13.8%)	13.9 (11.4 to 16.4)
Cardiovascular diseases	Costa Rica	2000	All Ages	0.5 (0.4 to 0.6)	11.2% (9.2 to 13.2%)	12.9 (10.6 to 15.4)
Cardiovascular diseases	Costa Rica	2005	All Ages	0.5 (0.4 to 0.6)	10.5% (8.5 to 12.6%)	11.9 (9.6 to 14.2)
Cardiovascular diseases	Costa Rica	2010	All Ages	0.5 (0.4 to 0.6)	9.0% (7.2 to 10.9%)	10.8 (8.5 to 13.1)
Cardiovascular diseases	Costa Rica	2015	All Ages	0.7 (0.5 to 0.8)	10.2% (8.2 to 12.3%)	14.0 (11.1 to 17.0)
Cardiovascular diseases	Costa Rica	1990	Age-standardized		11.6% (9.3 to 14.0%)	28.2 (22.6 to 34.0)
Cardiovascular diseases	Costa Rica	1995	Age-standardized		11.2% (9.0 to 13.4%)	25.9 (20.7 to 31.3)
Cardiovascular diseases	Costa Rica	2000	Age-standardized		10.7% (8.6 to 12.9%)	21.9 (17.6 to 26.6)
Cardiovascular diseases	Costa Rica	2005	Age-standardized		10.2% (8.0 to 12.3%)	17.6 (13.9 to 21.5)
Cardiovascular diseases	Costa Rica	2010	Age-standardized		8.8% (6.8 to 10.8%)	13.8 (10.7 to 17.1)
Cardiovascular diseases	Costa Rica	2015	Age-standardized		10.0% (7.9 to 12.1%)	15.4 (12.1 to 18.8)
Ischemic heart disease	Costa Rica	1990	All Ages	0.3 (0.3 to 0.4)	15.0% (11.8 to 18.1%)	10.6 (8.3 to 12.9)
Ischemic heart disease	Costa Rica	1995	All Ages	0.4 (0.3 to 0.5)	15.0% (11.9 to 18.1%)	10.8 (8.5 to 13.1)
Ischemic heart disease	Costa Rica	2000	All Ages	0.4 (0.3 to 0.5)	15.1% (11.7 to 18.3%)	9.9 (7.7 to 12.1)
Ischemic heart disease	Costa Rica	2005	All Ages	0.4 (0.3 to 0.5)	14.6% (11.4 to 18.0%)	9.2 (7.1 to 11.4)
Ischemic heart disease	Costa Rica	2010	All Ages	0.4 (0.3 to 0.5)	12.8% (9.6 to 15.9%)	8.4 (6.3 to 10.5)
Ischemic heart disease	Costa Rica	2015	All Ages	0.5 (0.4 to 0.7)	14.2% (10.7 to 17.6%)	10.9 (8.1 to 13.5)
Ischemic heart disease	Costa Rica	1990	Age-standardized		14.1% (10.7 to 17.6%)	22.0 (16.5 to 27.5)
Ischemic heart disease	Costa Rica	1995	Age-standardized		14.2% (10.9 to 17.6%)	20.1 (15.2 to 25.1)
Ischemic heart disease	Costa Rica	2000	Age-standardized		14.4% (10.8 to 18.0%)	16.7 (12.6 to 20.9)
Ischemic heart disease	Costa Rica	2005	Age-standardized		14.0% (10.6 to 17.6%)	13.6 (10.2 to 17.1)
Ischemic heart disease	Costa Rica	2010	Age-standardized		12.4% (9.1 to 15.6%)	10.7 (7.8 to 13.7)
Ischemic heart disease	Costa Rica	2015	Age-standardized		13.9% (10.2 to 17.5%)	11.9 (8.7 to 15.0)
Ischemic stroke	Costa Rica	1990	All Ages	0.1 (0.0 to 0.1)	10.5% (7.3 to 14.0%)	1.6 (1.1 to 2.2)
Ischemic stroke	Costa Rica	1995	All Ages	0.1 (0.0 to 0.1)	10.3% (7.0 to 13.9%)	1.6 (1.1 to 2.2)
Ischemic stroke	Costa Rica	2000	All Ages	0.1 (0.0 to 0.1)	10.3% (6.8 to 14.4%)	1.4 (0.9 to 1.9)
Ischemic stroke	Costa Rica	2005	All Ages	0.1 (0.0 to 0.1)	9.9% (6.2 to 14.1%)	1.2 (0.7 to 1.7)
Ischemic stroke	Costa Rica	2010	All Ages	0.1 (0.0 to 0.1)	8.3% (4.7 to 12.7%)	1.0 (0.6 to 1.6)
Ischemic stroke	Costa Rica	2015	All Ages	0.1 (0.0 to 0.1)	9.6% (5.8 to 14.1%)	1.4 (0.8 to 2.1)
Ischemic stroke	Costa Rica	1990	Age-standardized		10.1% (6.7 to 14.1%)	3.7 (2.4 to 5.2)
Ischemic stroke	Costa Rica	1995	Age-standardized		10.0% (6.5 to 14.0%)	3.3 (2.2 to 4.7)
Ischemic stroke	Costa Rica	2000	Age-standardized		10.1% (6.4 to 14.4%)	2.6 (1.6 to 3.7)
Ischemic stroke	Costa Rica	2005	Age-standardized		9.7% (5.9 to 14.2%)	1.9 (1.2 to 2.8)
Ischemic stroke	Costa Rica	2010	Age-standardized		8.2% (4.6 to 12.7%)	1.4 (0.8 to 2.2)

Cause name	Location	Year	Age group	Number (in thousands)	PAF	Rate (per 100,000)
Ischemic stroke	Costa Rica	2015	Age-standardized		9.5% (5.7 to 14.1%)	1.6 (0.9 to 2.4)
Hemorrhagic stroke	Costa Rica	1990	All Ages	0.0 (0.0 to 0.1)	12.2% (9.5 to 14.8%)	1.3 (1.0 to 1.7)
Hemorrhagic stroke	Costa Rica	1995	All Ages	0.1 (0.0 to 0.1)	12.0% (9.3 to 14.8%)	1.4 (1.1 to 1.8)
Hemorrhagic stroke	Costa Rica	2000	All Ages	0.1 (0.1 to 0.1)	12.0% (9.1 to 14.8%)	1.6 (1.2 to 2.0)
Hemorrhagic stroke	Costa Rica	2005	All Ages	0.1 (0.1 to 0.1)	11.5% (8.6 to 14.4%)	1.5 (1.1 to 1.9)
Hemorrhagic stroke	Costa Rica	2010	All Ages	0.1 (0.0 to 0.1)	9.6% (6.8 to 12.8%)	1.3 (0.9 to 1.8)
Hemorrhagic stroke	Costa Rica	2015	All Ages	0.1 (0.1 to 0.1)	11.0% (8.1 to 14.3%)	1.7 (1.3 to 2.3)
Hemorrhagic stroke	Costa Rica	1990	Age-standardized		11.6% (8.8 to 14.5%)	2.5 (1.9 to 3.1)
Hemorrhagic stroke	Costa Rica	1995	Age-standardized		11.4% (8.6 to 14.4%)	2.5 (1.8 to 3.1)
Hemorrhagic stroke	Costa Rica	2000	Age-standardized		11.4% (8.4 to 14.4%)	2.6 (1.9 to 3.4)
Hemorrhagic stroke	Costa Rica	2005	Age-standardized		11.0% (8.0 to 14.3%)	2.1 (1.5 to 2.8)
Hemorrhagic stroke	Costa Rica	2010	Age-standardized		9.3% (6.4 to 12.6%)	1.6 (1.1 to 2.3)
Hemorrhagic stroke	Costa Rica	2015	Age-standardized		10.8% (7.7 to 14.2%)	1.9 (1.4 to 2.5)
Chronic obstructive pulmonary disease	Costa Rica	1990	All Ages	0.1 (0.0 to 0.1)	18.0% (8.6 to 28.4%)	2.3 (1.1 to 3.6)
Chronic obstructive pulmonary disease	Costa Rica	1995	All Ages	0.1 (0.0 to 0.1)	18.2% (8.7 to 28.6%)	2.6 (1.2 to 4.1)
Chronic obstructive pulmonary disease	Costa Rica	2000	All Ages	0.1 (0.1 to 0.2)	18.9% (9.1 to 29.6%)	3.7 (1.8 to 5.9)
Chronic obstructive pulmonary disease	Costa Rica	2005	All Ages	0.1 (0.1 to 0.3)	18.3% (8.7 to 28.9%)	3.6 (1.7 to 5.8)
Chronic obstructive pulmonary disease	Costa Rica	2010	All Ages	0.1 (0.1 to 0.2)	14.5% (6.1 to 24.9%)	3.0 (1.3 to 5.2)
Chronic obstructive pulmonary disease	Costa Rica	2015	All Ages	0.2 (0.1 to 0.3)	18.1% (8.4 to 29.0%)	4.3 (2.1 to 7.0)
Chronic obstructive pulmonary disease	Costa Rica	1990	Age-standardized		18.6% (8.8 to 29.4%)	5.3 (2.5 to 8.4)
Chronic obstructive pulmonary disease	Costa Rica	1995	Age-standardized		18.5% (8.8 to 29.0%)	5.5 (2.6 to 8.7)
Chronic obstructive pulmonary disease	Costa Rica	2000	Age-standardized		19.0% (9.2 to 29.8%)	7.1 (3.4 to 11.2)
Chronic obstructive pulmonary disease	Costa Rica	2005	Age-standardized		18.3% (8.7 to 29.1%)	5.9 (2.8 to 9.5)
Chronic obstructive pulmonary disease	Costa Rica	2010	Age-standardized		14.5% (6.1 to 24.9%)	4.1 (1.7 to 7.2)
Chronic obstructive pulmonary disease	Costa Rica	2015	Age-standardized		18.1% (8.4 to 29.0%)	5.0 (2.4 to 8.1)
All causes	El Salvador	1990	All Ages	1.8 (1.5 to 2.0)	5.1% (4.4 to 5.8%)	33.1 (28.6 to 37.8)
All causes	El Salvador	1995	All Ages	1.9 (1.6 to 2.1)	5.7% (4.9 to 6.5%)	33.2 (28.6 to 38.1)
All causes	El Salvador	2000	All Ages	1.9 (1.6 to 2.2)	6.0% (5.1 to 6.9%)	32.6 (27.8 to 37.6)
All causes	El Salvador	2005	All Ages	1.9 (1.6 to 2.2)	5.8% (4.9 to 6.7%)	31.8 (26.9 to 36.9)
All causes	El Salvador	2010	All Ages	1.8 (1.6 to 2.2)	5.3% (4.5 to 6.2%)	30.4 (25.6 to 35.8)
All causes	El Salvador	2015	All Ages	2.2 (1.9 to 2.6)	5.9% (5.0 to 6.9%)	36.4 (30.7 to 43.1)
All causes	El Salvador	1990	Age-standardized		6.7% (5.7 to 7.7%)	67.6 (57.8 to 77.4)
All causes	El Salvador	1995	Age-standardized		6.9% (5.9 to 7.9%)	63.1 (54.2 to 73.0)
All causes	El Salvador	2000	Age-standardized		6.9% (5.9 to 8.0%)	56.2 (47.7 to 65.2)
All causes	El Salvador	2005	Age-standardized		6.4% (5.4 to 7.5%)	50.0 (42.0 to 58.3)
All causes	El Salvador	2010	Age-standardized		5.7% (4.8 to 6.7%)	42.1 (35.0 to 49.8)

Cause name	Location	Year	Age group	Number (in thousands)	PAF	Rate (per 100,000)
All causes	El Salvador	2015	Age-standardized		6.2% (5.2 to 7.2%)	43.8 (36.8 to 52.0)
Lower respiratory infections	El Salvador	1990	All Ages	0.6 (0.4 to 0.8)	27.0% (19.1 to 35.1%)	10.7 (7.5 to 14.4)
Lower respiratory infections	El Salvador	1995	All Ages	0.6 (0.4 to 0.8)	27.3% (19.6 to 35.4%)	11.2 (7.6 to 14.8)
Lower respiratory infections	El Salvador	2000	All Ages	0.7 (0.5 to 0.9)	28.4% (20.7 to 36.2%)	11.9 (7.8 to 16.0)
Lower respiratory infections	El Salvador	2005	All Ages	0.6 (0.4 to 0.8)	26.7% (18.7 to 34.6%)	10.4 (6.5 to 14.3)
Lower respiratory infections	El Salvador	2010	All Ages	0.5 (0.3 to 0.8)	23.3% (15.3 to 32.4%)	8.8 (5.3 to 12.7)
Lower respiratory infections	El Salvador	2015	All Ages	0.6 (0.4 to 0.9)	26.0% (18.2 to 34.2%)	10.5 (6.6 to 14.7)
Lower respiratory infections	El Salvador	1990	Age-standardized		27.0% (19.1 to 35.1%)	17.3 (11.9 to 23.1)
Lower respiratory infections	El Salvador	1995	Age-standardized		27.3% (19.6 to 35.4%)	18.0 (11.8 to 24.3)
Lower respiratory infections	El Salvador	2000	Age-standardized		28.4% (20.7 to 36.2%)	18.5 (11.5 to 25.3)
Lower respiratory infections	El Salvador	2005	Age-standardized		26.7% (18.7 to 34.6%)	15.7 (9.3 to 21.6)
Lower respiratory infections	El Salvador	2010	Age-standardized		23.3% (15.3 to 32.4%)	12.0 (7.1 to 17.3)
Lower respiratory infections	El Salvador	2015	Age-standardized		26.0% (18.2 to 34.2%)	12.5 (7.8 to 17.6)
Tracheal, bronchus, and lung cancer	El Salvador	1990	All Ages	0.0 (0.0 to 0.1)	19.3% (11.9 to 27.2%)	0.7 (0.4 to 1.0)
Tracheal, bronchus, and lung cancer	El Salvador	1995	All Ages	0.0 (0.0 to 0.1)	19.6% (12.0 to 27.7%)	0.8 (0.5 to 1.1)
Tracheal, bronchus, and lung cancer	El Salvador	2000	All Ages	0.1 (0.0 to 0.1)	20.5% (12.8 to 28.3%)	0.9 (0.6 to 1.3)
Tracheal, bronchus, and lung cancer	El Salvador	2005	All Ages	0.1 (0.0 to 0.1)	19.1% (11.8 to 27.0%)	1.0 (0.6 to 1.5)
Tracheal, bronchus, and lung cancer	El Salvador	2010	All Ages	0.1 (0.0 to 0.1)	16.5% (9.8 to 23.7%)	1.0 (0.6 to 1.4)
Tracheal, bronchus, and lung cancer	El Salvador	2015	All Ages	0.1 (0.1 to 0.1)	18.6% (11.3 to 26.3%)	1.3 (0.8 to 1.8)
Tracheal, bronchus, and lung cancer	El Salvador	1990	Age-standardized		19.5% (12.0 to 27.4%)	1.4 (0.9 to 2.0)
Tracheal, bronchus, and lung cancer	El Salvador	1995	Age-standardized		19.7% (12.1 to 27.9%)	1.5 (0.9 to 2.1)
Tracheal, bronchus, and lung cancer	El Salvador	2000	Age-standardized		20.6% (12.9 to 28.5%)	1.6 (1.0 to 2.2)
Tracheal, bronchus, and lung cancer	El Salvador	2005	Age-standardized		19.2% (11.9 to 27.1%)	1.6 (1.0 to 2.2)
Tracheal, bronchus, and lung cancer	El Salvador	2010	Age-standardized		16.5% (9.8 to 23.8%)	1.4 (0.8 to 2.0)
Tracheal, bronchus, and lung cancer	El Salvador	2015	Age-standardized		18.6% (11.3 to 26.4%)	1.5 (0.9 to 2.2)
Cardiovascular diseases	El Salvador	1990	All Ages	1.0 (0.9 to 1.2)	15.0% (13.0 to 17.2%)	19.1 (16.4 to 21.9)
Cardiovascular diseases	El Salvador	1995	All Ages	1.0 (0.9 to 1.2)	15.3% (13.1 to 17.7%)	18.6 (15.9 to 21.6)
Cardiovascular diseases	El Salvador	2000	All Ages	1.0 (0.8 to 1.2)	15.5% (13.4 to 17.9%)	17.3 (14.7 to 20.2)
Cardiovascular diseases	El Salvador	2005	All Ages	1.1 (0.9 to 1.3)	14.8% (12.5 to 17.5%)	17.7 (14.7 to 21.0)
Cardiovascular diseases	El Salvador	2010	All Ages	1.1 (0.9 to 1.3)	13.7% (11.3 to 16.3%)	17.9 (14.7 to 21.4)
Cardiovascular diseases	El Salvador	2015	All Ages	1.3 (1.0 to 1.6)	14.1% (11.5 to 17.0%)	21.2 (16.9 to 26.0)
Cardiovascular diseases	El Salvador	1990	Age-standardized		14.5% (12.1 to 17.2%)	42.0 (34.9 to 49.8)
Cardiovascular diseases	El Salvador	1995	Age-standardized		14.6% (12.2 to 17.4%)	37.4 (31.0 to 44.6)
Cardiovascular diseases	El Salvador	2000	Age-standardized		14.8% (12.4 to 17.7%)	31.0 (25.5 to 37.2)
Cardiovascular diseases	El Salvador	2005	Age-standardized		14.3% (11.7 to 17.3%)	28.2 (22.7 to 34.2)
Cardiovascular diseases	El Salvador	2010	Age-standardized		13.5% (10.9 to 16.2%)	24.8 (19.9 to 30.0)

Cause name	Location	Year	Age group	Number (in thousands)	PAF	Rate (per 100,000)
Cardiovascular diseases	El Salvador	2015	Age-standardized		14.1% (11.4 to 17.0%)	25.5 (20.3 to 31.4)
Ischemic heart disease	El Salvador	1990	All Ages	0.7 (0.6 to 0.8)	18.8% (15.7 to 22.3%)	13.5 (11.1 to 16.0)
Ischemic heart disease	El Salvador	1995	All Ages	0.8 (0.6 to 0.9)	18.8% (15.6 to 22.4%)	13.7 (11.2 to 16.3)
Ischemic heart disease	El Salvador	2000	All Ages	0.8 (0.6 to 0.9)	19.0% (15.7 to 22.8%)	13.4 (10.9 to 16.0)
Ischemic heart disease	El Salvador	2005	All Ages	0.8 (0.7 to 1.0)	18.1% (14.6 to 21.9%)	14.2 (11.4 to 17.3)
Ischemic heart disease	El Salvador	2010	All Ages	0.9 (0.7 to 1.1)	16.7% (13.3 to 20.3%)	14.5 (11.4 to 17.8)
Ischemic heart disease	El Salvador	2015	All Ages	1.1 (0.8 to 1.3)	17.1% (13.4 to 21.2%)	17.1 (13.2 to 21.5)
Ischemic heart disease	El Salvador	1990	Age-standardized		17.5% (13.8 to 21.6%)	30.2 (23.5 to 37.4)
Ischemic heart disease	El Salvador	1995	Age-standardized		17.5% (13.7 to 21.8%)	27.8 (21.6 to 34.9)
Ischemic heart disease	El Salvador	2000	Age-standardized		17.9% (14.2 to 22.1%)	24.2 (18.8 to 30.0)
Ischemic heart disease	El Salvador	2005	Age-standardized		17.3% (13.5 to 21.5%)	22.6 (17.5 to 28.3)
Ischemic heart disease	El Salvador	2010	Age-standardized		16.4% (12.7 to 20.1%)	20.2 (15.4 to 25.2)
Ischemic heart disease	El Salvador	2015	Age-standardized		17.0% (13.2 to 21.1%)	20.7 (15.8 to 26.0)
Ischemic stroke	El Salvador	1990	All Ages	0.1 (0.1 to 0.2)	14.2% (11.0 to 17.1%)	2.6 (2.1 to 3.2)
Ischemic stroke	El Salvador	1995	All Ages	0.1 (0.1 to 0.1)	14.1% (11.0 to 17.2%)	2.2 (1.7 to 2.7)
Ischemic stroke	El Salvador	2000	All Ages	0.1 (0.1 to 0.1)	14.1% (10.8 to 17.5%)	1.6 (1.2 to 2.1)
Ischemic stroke	El Salvador	2005	All Ages	0.1 (0.1 to 0.1)	13.3% (9.8 to 16.9%)	1.5 (1.1 to 2.0)
Ischemic stroke	El Salvador	2010	All Ages	0.1 (0.1 to 0.1)	12.1% (8.7 to 15.8%)	1.4 (1.0 to 2.0)
Ischemic stroke	El Salvador	2015	All Ages	0.1 (0.1 to 0.1)	12.7% (9.0 to 16.8%)	1.8 (1.2 to 2.4)
Ischemic stroke	El Salvador	1990	Age-standardized		13.5% (10.0 to 17.0%)	6.3 (4.7 to 8.0)
Ischemic stroke	El Salvador	1995	Age-standardized		13.4% (9.9 to 17.1%)	4.9 (3.6 to 6.2)
Ischemic stroke	El Salvador	2000	Age-standardized		13.6% (9.9 to 17.4%)	3.1 (2.3 to 4.2)
Ischemic stroke	El Salvador	2005	Age-standardized		12.9% (9.2 to 17.0%)	2.5 (1.7 to 3.4)
Ischemic stroke	El Salvador	2010	Age-standardized		11.9% (8.4 to 15.9%)	2.0 (1.4 to 2.9)
Ischemic stroke	El Salvador	2015	Age-standardized		12.7% (8.9 to 16.8%)	2.1 (1.5 to 3.0)
Hemorrhagic stroke	El Salvador	1990	All Ages	0.2 (0.1 to 0.2)	14.7% (12.1 to 17.1%)	3.0 (2.5 to 3.5)
Hemorrhagic stroke	El Salvador	1995	All Ages	0.1 (0.1 to 0.2)	15.5% (12.9 to 18.0%)	2.7 (2.2 to 3.2)
Hemorrhagic stroke	El Salvador	2000	All Ages	0.1 (0.1 to 0.2)	15.8% (13.2 to 18.5%)	2.3 (1.9 to 2.7)
Hemorrhagic stroke	El Salvador	2005	All Ages	0.1 (0.1 to 0.1)	15.1% (12.5 to 17.8%)	2.1 (1.7 to 2.5)
Hemorrhagic stroke	El Salvador	2010	All Ages	0.1 (0.1 to 0.1)	13.9% (11.2 to 16.5%)	2.0 (1.6 to 2.4)
Hemorrhagic stroke	El Salvador	2015	All Ages	0.1 (0.1 to 0.2)	14.6% (11.7 to 17.5%)	2.3 (1.8 to 2.8)
Hemorrhagic stroke	El Salvador	1990	Age-standardized		15.3% (12.4 to 18.1%)	5.5 (4.5 to 6.5)
Hemorrhagic stroke	El Salvador	1995	Age-standardized		15.4% (12.5 to 18.2%)	4.7 (3.8 to 5.6)
Hemorrhagic stroke	El Salvador	2000	Age-standardized		15.5% (12.6 to 18.4%)	3.8 (3.0 to 4.5)
Hemorrhagic stroke	El Salvador	2005	Age-standardized		14.8% (12.0 to 17.7%)	3.1 (2.5 to 3.7)
Hemorrhagic stroke	El Salvador	2010	Age-standardized		13.7% (10.9 to 16.6%)	2.6 (2.1 to 3.3)

Cause name	Location	Year	Age group	Number (in thousands)	PAF	Rate (per 100,000)
Hemorrhagic stroke	El Salvador	2015	Age-standardized		14.6% (11.7 to 17.5%)	2.7 (2.1 to 3.3)
Chronic obstructive pulmonary disease	El Salvador	1990	All Ages	0.1 (0.1 to 0.2)	17.3% (9.6 to 26.5%)	2.6 (1.5 to 3.8)
Chronic obstructive pulmonary disease	El Salvador	1995	All Ages	0.1 (0.1 to 0.2)	22.7% (13.2 to 33.2%)	2.6 (1.5 to 3.8)
Chronic obstructive pulmonary disease	El Salvador	2000	All Ages	0.1 (0.1 to 0.2)	26.2% (15.7 to 37.4%)	2.5 (1.5 to 3.6)
Chronic obstructive pulmonary disease	El Salvador	2005	All Ages	0.2 (0.1 to 0.2)	25.4% (14.7 to 36.6%)	2.6 (1.5 to 3.8)
Chronic obstructive pulmonary disease	El Salvador	2010	All Ages	0.2 (0.1 to 0.2)	22.9% (12.4 to 33.9%)	2.7 (1.5 to 4.0)
Chronic obstructive pulmonary disease	El Salvador	2015	All Ages	0.2 (0.1 to 0.3)	25.0% (14.3 to 36.2%)	3.5 (2.0 to 5.2)
Chronic obstructive pulmonary disease	El Salvador	1990	Age-standardized		23.3% (13.6 to 33.8%)	6.9 (3.9 to 10.1)
Chronic obstructive pulmonary disease	El Salvador	1995	Age-standardized		25.2% (14.8 to 36.3%)	6.2 (3.6 to 9.0)
Chronic obstructive pulmonary disease	El Salvador	2000	Age-standardized		26.8% (16.0 to 38.2%)	5.0 (3.0 to 7.3)
Chronic obstructive pulmonary disease	El Salvador	2005	Age-standardized		25.6% (14.9 to 36.9%)	4.6 (2.6 to 6.6)
Chronic obstructive pulmonary disease	El Salvador	2010	Age-standardized		23.0% (12.5 to 34.1%)	3.9 (2.2 to 5.8)
Chronic obstructive pulmonary disease	El Salvador	2015	Age-standardized		25.1% (14.3 to 36.3%)	4.3 (2.5 to 6.3)
All causes	Guatemala	1990	All Ages	4.0 (3.1 to 5.0)	5.7% (4.4 to 7.1%)	43.3 (33.5 to 53.9)
All causes	Guatemala	1995	All Ages	4.4 (3.5 to 5.5)	6.5% (5.0 to 8.0%)	42.6 (33.2 to 52.5)
All causes	Guatemala	2000	All Ages	5.0 (4.0 to 6.1)	7.2% (5.7 to 8.8%)	42.7 (33.8 to 52.1)
All causes	Guatemala	2005	All Ages	4.8 (3.9 to 5.9)	6.5% (5.2 to 7.9%)	36.6 (29.2 to 44.9)
All causes	Guatemala	2010	All Ages	4.2 (3.3 to 5.3)	5.4% (4.1 to 6.7%)	28.6 (22.1 to 35.8)
All causes	Guatemala	2015	All Ages	4.9 (3.9 to 5.9)	5.8% (4.8 to 6.9%)	29.8 (24.0 to 36.2)
All causes	Guatemala	1990	Age-standardized		6.7% (5.5 to 8.1%)	80.6 (65.8 to 97.7)
All causes	Guatemala	1995	Age-standardized		7.2% (5.9 to 8.6%)	77.7 (63.5 to 93.4)
All causes	Guatemala	2000	Age-standardized		7.6% (6.3 to 9.1%)	74.3 (61.2 to 88.9)
All causes	Guatemala	2005	Age-standardized		6.9% (5.7 to 8.2%)	63.5 (52.4 to 76.1)
All causes	Guatemala	2010	Age-standardized		5.8% (4.7 to 7.1%)	50.9 (41.1 to 62.2)
All causes	Guatemala	2015	Age-standardized		6.3% (5.3 to 7.4%)	52.2 (42.3 to 63.7)
Lower respiratory infections	Guatemala	1990	All Ages	2.8 (1.9 to 3.7)	25.3% (17.5 to 33.7%)	30.0 (20.5 to 40.4)
Lower respiratory infections	Guatemala	1995	All Ages	3.0 (2.0 to 4.1)	25.6% (17.6 to 33.9%)	29.1 (19.8 to 39.1)
Lower respiratory infections	Guatemala	2000	All Ages	3.4 (2.4 to 4.5)	26.8% (18.9 to 34.9%)	29.3 (20.4 to 38.4)
Lower respiratory infections	Guatemala	2005	All Ages	3.1 (2.2 to 4.2)	26.2% (18.3 to 34.4%)	23.8 (16.5 to 31.8)
Lower respiratory infections	Guatemala	2010	All Ages	2.3 (1.4 to 3.4)	22.0% (13.5 to 31.5%)	15.9 (9.7 to 22.7)
Lower respiratory infections	Guatemala	2015	All Ages	2.5 (1.7 to 3.4)	25.0% (16.9 to 33.6%)	15.2 (10.2 to 20.7)
Lower respiratory infections	Guatemala	1990	Age-standardized		25.3% (17.5 to 33.7%)	43.6 (30.0 to 58.4)
Lower respiratory infections	Guatemala	1995	Age-standardized		25.6% (17.6 to 33.9%)	41.9 (28.4 to 56.4)
Lower respiratory infections	Guatemala	2000	Age-standardized		26.8% (18.9 to 34.9%)	41.6 (29.1 to 54.9)
Lower respiratory infections	Guatemala	2005	Age-standardized		26.2% (18.3 to 34.4%)	34.3 (24.1 to 46.0)
Lower respiratory infections	Guatemala	2010	Age-standardized		22.0% (13.5 to 31.5%)	24.0 (14.6 to 34.5)

Cause name	Location	Year	Age group	Number (in thousands)	PAF	Rate (per 100,000)
Lower respiratory infections	Guatemala	2015	Age-standardized		25.0% (16.9 to 33.6%)	23.6 (15.9 to 32.0)
Tracheal, bronchus, and lung cancer	Guatemala	1990	All Ages	0.1 (0.0 to 0.1)	17.8% (10.8 to 25.3%)	0.5 (0.3 to 0.7)
Tracheal, bronchus, and lung cancer	Guatemala	1995	All Ages	0.1 (0.0 to 0.1)	18.0% (11.0 to 25.4%)	0.6 (0.3 to 0.8)
Tracheal, bronchus, and lung cancer	Guatemala	2000	All Ages	0.1 (0.1 to 0.1)	18.9% (11.6 to 26.4%)	0.7 (0.4 to 0.9)
Tracheal, bronchus, and lung cancer	Guatemala	2005	All Ages	0.1 (0.1 to 0.1)	18.5% (11.3 to 25.9%)	0.7 (0.4 to 1.0)
Tracheal, bronchus, and lung cancer	Guatemala	2010	All Ages	0.1 (0.1 to 0.1)	15.4% (9.0 to 22.4%)	0.6 (0.3 to 0.8)
Tracheal, bronchus, and lung cancer	Guatemala	2015	All Ages	0.1 (0.1 to 0.2)	17.7% (10.8 to 25.2%)	0.7 (0.4 to 1.0)
Tracheal, bronchus, and lung cancer	Guatemala	1990	Age-standardized		18.1% (11.0 to 25.6%)	1.4 (0.8 to 1.9)
Tracheal, bronchus, and lung cancer	Guatemala	1995	Age-standardized		18.3% (11.1 to 25.8%)	1.4 (0.9 to 2.0)
Tracheal, bronchus, and lung cancer	Guatemala	2000	Age-standardized		19.2% (11.8 to 26.9%)	1.6 (1.0 to 2.3)
Tracheal, bronchus, and lung cancer	Guatemala	2005	Age-standardized		18.7% (11.4 to 26.3%)	1.5 (0.9 to 2.2)
Tracheal, bronchus, and lung cancer	Guatemala	2010	Age-standardized		15.6% (9.1 to 22.6%)	1.2 (0.7 to 1.7)
Tracheal, bronchus, and lung cancer	Guatemala	2015	Age-standardized		17.9% (10.9 to 25.4%)	1.3 (0.8 to 1.9)
Cardiovascular diseases	Guatemala	1990	All Ages	1.0 (0.9 to 1.2)	14.4% (12.5 to 16.4%)	11.2 (9.7 to 12.8)
Cardiovascular diseases	Guatemala	1995	All Ages	1.2 (1.0 to 1.3)	14.4% (12.5 to 16.3%)	11.3 (9.8 to 12.9)
Cardiovascular diseases	Guatemala	2000	All Ages	1.3 (1.1 to 1.5)	15.0% (13.1 to 17.0%)	11.1 (9.8 to 12.6)
Cardiovascular diseases	Guatemala	2005	All Ages	1.4 (1.2 to 1.6)	14.6% (12.7 to 16.8%)	10.5 (9.1 to 12.0)
Cardiovascular diseases	Guatemala	2010	All Ages	1.6 (1.3 to 1.8)	13.2% (11.2 to 15.4%)	10.5 (8.9 to 12.3)
Cardiovascular diseases	Guatemala	2015	All Ages	2.0 (1.6 to 2.4)	14.0% (11.8 to 16.4%)	11.9 (9.7 to 14.4)
Cardiovascular diseases	Guatemala	1990	Age-standardized		13.9% (11.6 to 16.5%)	30.5 (25.3 to 36.3)
Cardiovascular diseases	Guatemala	1995	Age-standardized		14.0% (11.8 to 16.5%)	29.4 (24.6 to 34.7)
Cardiovascular diseases	Guatemala	2000	Age-standardized		14.6% (12.5 to 17.2%)	26.7 (22.6 to 31.3)
Cardiovascular diseases	Guatemala	2005	Age-standardized		14.3% (12.1 to 16.9%)	23.5 (19.8 to 27.7)
Cardiovascular diseases	Guatemala	2010	Age-standardized		13.0% (10.8 to 15.3%)	22.1 (18.3 to 26.2)
Cardiovascular diseases	Guatemala	2015	Age-standardized		13.7% (11.3 to 16.3%)	23.2 (18.6 to 28.4)
Ischemic heart disease	Guatemala	1990	All Ages	0.8 (0.7 to 0.9)	18.7% (15.7 to 21.8%)	8.5 (7.1 to 10.0)
Ischemic heart disease	Guatemala	1995	All Ages	0.8 (0.7 to 1.0)	18.7% (15.8 to 21.9%)	8.2 (6.9 to 9.6)
Ischemic heart disease	Guatemala	2000	All Ages	0.9 (0.8 to 1.1)	19.0% (16.0 to 22.4%)	7.6 (6.4 to 9.0)
Ischemic heart disease	Guatemala	2005	All Ages	1.0 (0.8 to 1.2)	18.4% (15.2 to 21.9%)	7.4 (6.1 to 8.8)
Ischemic heart disease	Guatemala	2010	All Ages	1.1 (0.9 to 1.3)	16.6% (13.4 to 20.0%)	7.5 (6.0 to 9.1)
Ischemic heart disease	Guatemala	2015	All Ages	1.4 (1.1 to 1.8)	17.4% (13.8 to 21.1%)	8.6 (6.6 to 10.7)
Ischemic heart disease	Guatemala	1990	Age-standardized		17.1% (13.6 to 20.9%)	23.5 (18.5 to 28.8)
Ischemic heart disease	Guatemala	1995	Age-standardized		17.2% (13.7 to 21.2%)	21.6 (17.2 to 26.4)
Ischemic heart disease	Guatemala	2000	Age-standardized		17.8% (14.1 to 21.8%)	18.7 (14.9 to 22.8)
Ischemic heart disease	Guatemala	2005	Age-standardized		17.4% (13.8 to 21.5%)	16.9 (13.4 to 20.6)
Ischemic heart disease	Guatemala	2010	Age-standardized		16.0% (12.4 to 19.7%)	15.9 (12.2 to 19.7)

Cause name	Location	Year	Age group	Number (in thousands)	PAF	Rate (per 100,000)
Ischemic heart disease	Guatemala	2015	Age-standardized		16.8% (13.0 to 20.8%)	16.8 (12.6 to 21.4)
Ischemic stroke	Guatemala	1990	All Ages	0.1 (0.1 to 0.1)	13.9% (10.9 to 16.8%)	1.2 (0.9 to 1.5)
Ischemic stroke	Guatemala	1995	All Ages	0.1 (0.1 to 0.2)	13.9% (10.9 to 16.7%)	1.4 (1.1 to 1.8)
Ischemic stroke	Guatemala	2000	All Ages	0.2 (0.1 to 0.2)	14.0% (10.9 to 17.1%)	1.4 (1.1 to 1.7)
Ischemic stroke	Guatemala	2005	All Ages	0.2 (0.1 to 0.2)	13.4% (10.1 to 16.7%)	1.3 (0.9 to 1.6)
Ischemic stroke	Guatemala	2010	All Ages	0.2 (0.1 to 0.3)	11.9% (8.6 to 15.3%)	1.3 (0.9 to 1.7)
Ischemic stroke	Guatemala	2015	All Ages	0.2 (0.2 to 0.3)	12.6% (9.1 to 16.3%)	1.5 (1.0 to 2.0)
Ischemic stroke	Guatemala	1990	Age-standardized		13.0% (9.6 to 16.5%)	3.7 (2.7 to 4.8)
Ischemic stroke	Guatemala	1995	Age-standardized		13.1% (9.7 to 16.6%)	4.2 (3.1 to 5.3)
Ischemic stroke	Guatemala	2000	Age-standardized		13.4% (9.8 to 17.0%)	3.8 (2.8 to 4.9)
Ischemic stroke	Guatemala	2005	Age-standardized		13.0% (9.4 to 16.7%)	3.1 (2.3 to 4.1)
Ischemic stroke	Guatemala	2010	Age-standardized		11.6% (8.1 to 15.4%)	2.9 (2.0 to 3.9)
Ischemic stroke	Guatemala	2015	Age-standardized		12.5% (8.8 to 16.4%)	3.0 (2.1 to 4.1)
Hemorrhagic stroke	Guatemala	1990	All Ages	0.1 (0.1 to 0.2)	13.6% (11.4 to 15.8%)	1.4 (1.2 to 1.7)
Hemorrhagic stroke	Guatemala	1995	All Ages	0.2 (0.1 to 0.2)	13.6% (11.4 to 15.8%)	1.7 (1.4 to 2.0)
Hemorrhagic stroke	Guatemala	2000	All Ages	0.2 (0.2 to 0.3)	14.4% (12.1 to 16.6%)	2.1 (1.7 to 2.4)
Hemorrhagic stroke	Guatemala	2005	All Ages	0.2 (0.2 to 0.3)	14.5% (12.2 to 16.8%)	1.8 (1.5 to 2.1)
Hemorrhagic stroke	Guatemala	2010	All Ages	0.3 (0.2 to 0.3)	13.2% (10.8 to 15.6%)	1.7 (1.4 to 2.0)
Hemorrhagic stroke	Guatemala	2015	All Ages	0.3 (0.2 to 0.4)	14.4% (11.8 to 16.9%)	1.9 (1.5 to 2.4)
Hemorrhagic stroke	Guatemala	1990	Age-standardized		14.8% (12.2 to 17.5%)	3.2 (2.6 to 3.8)
Hemorrhagic stroke	Guatemala	1995	Age-standardized		15.1% (12.4 to 17.6%)	3.7 (3.0 to 4.3)
Hemorrhagic stroke	Guatemala	2000	Age-standardized		15.6% (13.0 to 18.1%)	4.2 (3.5 to 4.9)
Hemorrhagic stroke	Guatemala	2005	Age-standardized		15.2% (12.5 to 17.8%)	3.6 (2.9 to 4.2)
Hemorrhagic stroke	Guatemala	2010	Age-standardized		13.5% (10.7 to 16.3%)	3.3 (2.6 to 4.0)
Hemorrhagic stroke	Guatemala	2015	Age-standardized		14.4% (11.6 to 17.2%)	3.5 (2.7 to 4.3)
Chronic obstructive pulmonary disease	Guatemala	1990	All Ages	0.1 (0.1 to 0.2)	9.3% (5.1 to 14.0%)	1.6 (0.9 to 2.4)
Chronic obstructive pulmonary disease	Guatemala	1995	All Ages	0.2 (0.1 to 0.3)	10.2% (5.9 to 15.2%)	1.6 (0.9 to 2.4)
Chronic obstructive pulmonary disease	Guatemala	2000	All Ages	0.2 (0.1 to 0.3)	16.5% (9.8 to 24.2%)	1.6 (0.9 to 2.3)
Chronic obstructive pulmonary disease	Guatemala	2005	All Ages	0.2 (0.1 to 0.3)	22.6% (12.9 to 32.7%)	1.6 (0.9 to 2.4)
Chronic obstructive pulmonary disease	Guatemala	2010	All Ages	0.2 (0.1 to 0.4)	21.1% (11.2 to 31.6%)	1.6 (0.9 to 2.4)
Chronic obstructive pulmonary disease	Guatemala	2015	All Ages	0.3 (0.2 to 0.5)	23.7% (13.1 to 34.7%)	1.9 (1.1 to 2.8)
Chronic obstructive pulmonary disease	Guatemala	1990	Age-standardized		19.7% (11.0 to 28.9%)	5.2 (2.8 to 7.8)
Chronic obstructive pulmonary disease	Guatemala	1995	Age-standardized		20.0% (11.4 to 29.0%)	5.0 (2.7 to 7.2)
Chronic obstructive pulmonary disease	Guatemala	2000	Age-standardized		23.3% (13.7 to 33.5%)	4.4 (2.6 to 6.4)
Chronic obstructive pulmonary disease	Guatemala	2005	Age-standardized		24.7% (14.0 to 35.9%)	4.1 (2.4 to 5.9)
Chronic obstructive pulmonary disease	Guatemala	2010	Age-standardized		21.9% (11.6 to 32.8%)	3.7 (2.0 to 5.6)

Cause name	Location	Year	Age group	Number (in thousands)	PAF	Rate (per 100,000)
Chronic obstructive pulmonary disease	Guatemala	2015	Age-standardized		24.3% (13.5 to 35.6%)	4.1 (2.2 to 5.9)
All causes	Honduras	1990	All Ages	1.4 (1.2 to 1.6)	5.3% (4.6 to 6.0%)	29.1 (25.3 to 33.1)
All causes	Honduras	1995	All Ages	1.9 (1.6 to 2.2)	6.3% (5.5 to 7.1%)	33.7 (28.6 to 39.4)
All causes	Honduras	2000	All Ages	2.2 (1.8 to 2.7)	6.8% (5.9 to 7.7%)	35.5 (28.9 to 42.9)
All causes	Honduras	2005	All Ages	2.6 (2.0 to 3.2)	7.2% (6.3 to 8.2%)	37.1 (29.2 to 46.1)
All causes	Honduras	2010	All Ages	2.6 (2.0 to 3.3)	6.6% (5.7 to 7.7%)	34.3 (26.7 to 43.4)
All causes	Honduras	2015	All Ages	3.1 (2.5 to 3.9)	7.4% (6.3 to 8.5%)	38.9 (30.3 to 48.5)
All causes	Honduras	1990	Age-standardized		7.7% (6.6 to 8.7%)	65.6 (56.4 to 75.2)
All causes	Honduras	1995	Age-standardized		8.3% (7.1 to 9.5%)	79.5 (66.4 to 94.7)
All causes	Honduras	2000	Age-standardized		8.4% (7.3 to 9.7%)	82.4 (65.6 to 99.9)
All causes	Honduras	2005	Age-standardized		8.6% (7.4 to 9.9%)	81.5 (63.3 to 101.8)
All causes	Honduras	2010	Age-standardized		7.6% (6.4 to 8.8%)	69.7 (54.0 to 88.7)
All causes	Honduras	2015	Age-standardized		8.2% (7.0 to 9.5%)	72.0 (56.0 to 90.5)
Lower respiratory infections	Honduras	1990	All Ages	0.4 (0.2 to 0.5)	25.3% (17.5 to 33.8%)	7.1 (4.8 to 9.7)
Lower respiratory infections	Honduras	1995	All Ages	0.4 (0.2 to 0.5)	25.4% (17.8 to 33.7%)	6.2 (4.1 to 8.7)
Lower respiratory infections	Honduras	2000	All Ages	0.4 (0.2 to 0.5)	26.1% (18.2 to 34.1%)	5.6 (3.8 to 7.8)
Lower respiratory infections	Honduras	2005	All Ages	0.4 (0.3 to 0.5)	27.3% (19.6 to 35.4%)	5.6 (3.9 to 7.6)
Lower respiratory infections	Honduras	2010	All Ages	0.4 (0.2 to 0.5)	23.6% (15.5 to 32.4%)	4.7 (3.0 to 6.7)
Lower respiratory infections	Honduras	2015	All Ages	0.4 (0.2 to 0.5)	26.1% (18.4 to 34.4%)	4.6 (2.9 to 6.5)
Lower respiratory infections	Honduras	1990	Age-standardized		25.3% (17.5 to 33.8%)	7.9 (5.3 to 10.9)
Lower respiratory infections	Honduras	1995	Age-standardized		25.4% (17.8 to 33.7%)	8.3 (5.4 to 11.8)
Lower respiratory infections	Honduras	2000	Age-standardized		26.1% (18.2 to 34.1%)	8.5 (5.6 to 12.2)
Lower respiratory infections	Honduras	2005	Age-standardized		27.3% (19.6 to 35.4%)	9.0 (6.0 to 12.7)
Lower respiratory infections	Honduras	2010	Age-standardized		23.6% (15.5 to 32.4%)	7.8 (4.7 to 11.3)
Lower respiratory infections	Honduras	2015	Age-standardized		26.1% (18.4 to 34.4%)	7.5 (4.6 to 10.8)
Tracheal, bronchus, and lung cancer	Honduras	1990	All Ages	0.0 (0.0 to 0.0)	18.0% (11.0 to 25.5%)	0.5 (0.3 to 0.7)
Tracheal, bronchus, and lung cancer	Honduras	1995	All Ages	0.0 (0.0 to 0.1)	18.2% (11.1 to 25.9%)	0.6 (0.3 to 0.8)
Tracheal, bronchus, and lung cancer	Honduras	2000	All Ages	0.0 (0.0 to 0.1)	18.7% (11.5 to 26.6%)	0.7 (0.4 to 1.0)
Tracheal, bronchus, and lung cancer	Honduras	2005	All Ages	0.1 (0.0 to 0.1)	19.7% (12.4 to 27.7%)	0.8 (0.4 to 1.2)
Tracheal, bronchus, and lung cancer	Honduras	2010	All Ages	0.1 (0.0 to 0.1)	16.8% (10.0 to 24.1%)	0.8 (0.4 to 1.2)
Tracheal, bronchus, and lung cancer	Honduras	2015	All Ages	0.1 (0.0 to 0.1)	18.8% (11.5 to 26.4%)	0.9 (0.5 to 1.5)
Tracheal, bronchus, and lung cancer	Honduras	1990	Age-standardized		18.1% (11.1 to 25.7%)	1.2 (0.7 to 1.8)
Tracheal, bronchus, and lung cancer	Honduras	1995	Age-standardized		18.2% (11.1 to 26.0%)	1.5 (0.9 to 2.1)
Tracheal, bronchus, and lung cancer	Honduras	2000	Age-standardized		18.8% (11.5 to 26.7%)	1.6 (0.9 to 2.3)
Tracheal, bronchus, and lung cancer	Honduras	2005	Age-standardized		19.8% (12.4 to 27.8%)	1.8 (1.0 to 2.6)
Tracheal, bronchus, and lung cancer	Honduras	2010	Age-standardized		16.8% (10.1 to 24.1%)	1.5 (0.8 to 2.4)

Cause name	Location	Year	Age group	Number (in thousands)	PAF	Rate (per 100,000)
Tracheal, bronchus, and lung cancer	Honduras	2015	Age-standardized		18.8% (11.6 to 26.5%)	1.7 (0.9 to 2.8)
Cardiovascular diseases	Honduras	1990	All Ages	0.9 (0.8 to 1.1)	12.8% (11.2 to 14.5%)	18.8 (16.2 to 21.4)
Cardiovascular diseases	Honduras	1995	All Ages	1.3 (1.1 to 1.5)	13.9% (12.0 to 15.9%)	23.2 (19.0 to 27.5)
Cardiovascular diseases	Honduras	2000	All Ages	1.6 (1.2 to 1.9)	14.4% (12.3 to 16.6%)	25.0 (19.7 to 31.1)
Cardiovascular diseases	Honduras	2005	All Ages	1.8 (1.4 to 2.2)	14.6% (12.5 to 17.0%)	25.6 (19.9 to 32.3)
Cardiovascular diseases	Honduras	2010	All Ages	1.8 (1.4 to 2.3)	13.4% (11.2 to 15.7%)	23.6 (17.9 to 30.8)
Cardiovascular diseases	Honduras	2015	All Ages	2.2 (1.7 to 2.9)	14.2% (11.9 to 16.8%)	27.6 (21.0 to 35.6)
Cardiovascular diseases	Honduras	1990	Age-standardized		14.2% (12.1 to 16.6%)	47.8 (40.4 to 55.7)
Cardiovascular diseases	Honduras	1995	Age-standardized		14.4% (12.1 to 16.8%)	58.9 (47.4 to 71.4)
Cardiovascular diseases	Honduras	2000	Age-standardized		14.5% (12.1 to 17.0%)	60.6 (46.8 to 75.4)
Cardiovascular diseases	Honduras	2005	Age-standardized		14.6% (12.2 to 17.2%)	58.0 (44.5 to 73.6)
Cardiovascular diseases	Honduras	2010	Age-standardized		13.3% (10.9 to 15.7%)	49.0 (37.1 to 63.7)
Cardiovascular diseases	Honduras	2015	Age-standardized		14.0% (11.6 to 16.7%)	51.4 (39.3 to 66.2)
Ischemic heart disease	Honduras	1990	All Ages	0.7 (0.6 to 0.8)	19.1% (16.1 to 22.2%)	13.7 (11.5 to 16.2)
Ischemic heart disease	Honduras	1995	All Ages	1.0 (0.8 to 1.2)	18.5% (15.5 to 21.9%)	17.2 (13.7 to 21.1)
Ischemic heart disease	Honduras	2000	All Ages	1.2 (0.9 to 1.5)	18.4% (15.3 to 21.8%)	18.5 (14.3 to 23.5)
Ischemic heart disease	Honduras	2005	All Ages	1.3 (1.0 to 1.7)	18.4% (15.0 to 22.1%)	18.7 (14.0 to 24.1)
Ischemic heart disease	Honduras	2010	All Ages	1.3 (1.0 to 1.7)	16.9% (13.5 to 20.4%)	17.1 (12.7 to 22.6)
Ischemic heart disease	Honduras	2015	All Ages	1.6 (1.2 to 2.1)	17.6% (14.1 to 21.4%)	20.2 (15.1 to 26.3)
Ischemic heart disease	Honduras	1990	Age-standardized		17.9% (14.5 to 21.4%)	35.3 (28.3 to 42.5)
Ischemic heart disease	Honduras	1995	Age-standardized		17.5% (14.1 to 21.2%)	44.0 (33.9 to 54.8)
Ischemic heart disease	Honduras	2000	Age-standardized		17.5% (14.1 to 21.2%)	45.2 (33.9 to 57.8)
Ischemic heart disease	Honduras	2005	Age-standardized		17.8% (14.2 to 21.8%)	42.4 (31.1 to 55.1)
Ischemic heart disease	Honduras	2010	Age-standardized		16.4% (12.8 to 20.0%)	35.5 (26.0 to 46.8)
Ischemic heart disease	Honduras	2015	Age-standardized		17.1% (13.4 to 21.1%)	37.6 (27.8 to 48.8)
Ischemic stroke	Honduras	1990	All Ages	0.1 (0.1 to 0.1)	13.6% (10.7 to 16.4%)	2.4 (1.9 to 2.9)
Ischemic stroke	Honduras	1995	All Ages	0.2 (0.1 to 0.2)	13.5% (10.4 to 16.6%)	3.0 (2.2 to 3.8)
Ischemic stroke	Honduras	2000	All Ages	0.2 (0.1 to 0.3)	13.6% (10.3 to 16.7%)	3.2 (2.3 to 4.3)
Ischemic stroke	Honduras	2005	All Ages	0.3 (0.2 to 0.3)	13.7% (10.4 to 17.2%)	3.6 (2.5 to 4.9)
Ischemic stroke	Honduras	2010	All Ages	0.3 (0.2 to 0.4)	12.4% (9.1 to 15.9%)	3.4 (2.3 to 4.8)
Ischemic stroke	Honduras	2015	All Ages	0.3 (0.2 to 0.5)	13.1% (9.6 to 16.7%)	3.9 (2.7 to 5.5)
Ischemic stroke	Honduras	1990	Age-standardized		13.2% (9.9 to 16.6%)	6.8 (5.0 to 8.8)
Ischemic stroke	Honduras	1995	Age-standardized		13.1% (9.5 to 16.6%)	8.2 (5.9 to 10.9)
Ischemic stroke	Honduras	2000	Age-standardized		13.2% (9.7 to 16.8%)	8.5 (5.9 to 11.6)
Ischemic stroke	Honduras	2005	Age-standardized		13.5% (9.9 to 17.1%)	8.5 (5.9 to 12.0)
Ischemic stroke	Honduras	2010	Age-standardized		12.2% (8.8 to 15.9%)	7.4 (5.0 to 10.6)

Cause name	Location	Year	Age group	Number (in thousands)	PAF	Rate (per 100,000)
Ischemic stroke	Honduras	2015	Age-standardized		12.9% (9.3 to 16.7%)	7.7 (5.2 to 10.9)
Hemorrhagic stroke	Honduras	1990	All Ages	0.1 (0.1 to 0.2)	6.5% (5.3 to 8.6%)	2.7 (2.2 to 3.3)
Hemorrhagic stroke	Honduras	1995	All Ages	0.2 (0.1 to 0.2)	8.7% (7.1 to 11.1%)	3.0 (2.3 to 3.9)
Hemorrhagic stroke	Honduras	2000	All Ages	0.2 (0.1 to 0.3)	10.4% (8.5 to 12.7%)	3.2 (2.3 to 4.4)
Hemorrhagic stroke	Honduras	2005	All Ages	0.2 (0.2 to 0.3)	12.0% (9.8 to 14.5%)	3.3 (2.5 to 4.6)
Hemorrhagic stroke	Honduras	2010	All Ages	0.2 (0.2 to 0.3)	12.0% (9.6 to 14.4%)	3.1 (2.2 to 4.4)
Hemorrhagic stroke	Honduras	2015	All Ages	0.3 (0.2 to 0.4)	13.2% (10.6 to 15.8%)	3.4 (2.5 to 4.8)
Hemorrhagic stroke	Honduras	1990	Age-standardized		11.6% (9.6 to 13.9%)	5.8 (4.7 to 7.5)
Hemorrhagic stroke	Honduras	1995	Age-standardized		12.9% (10.4 to 15.5%)	6.7 (5.2 to 8.9)
Hemorrhagic stroke	Honduras	2000	Age-standardized		13.5% (11.0 to 16.2%)	7.0 (5.1 to 9.6)
Hemorrhagic stroke	Honduras	2005	Age-standardized		14.1% (11.4 to 16.8%)	7.1 (5.1 to 9.9)
Hemorrhagic stroke	Honduras	2010	Age-standardized		12.9% (10.2 to 15.6%)	6.1 (4.3 to 8.6)
Hemorrhagic stroke	Honduras	2015	Age-standardized		13.8% (11.0 to 16.6%)	6.1 (4.3 to 8.5)
Chronic obstructive pulmonary disease	Honduras	1990	All Ages	0.1 (0.1 to 0.2)	19.3% (10.2 to 29.6%)	2.8 (1.5 to 4.3)
Chronic obstructive pulmonary disease	Honduras	1995	All Ages	0.2 (0.1 to 0.3)	21.5% (11.5 to 31.9%)	3.7 (2.0 to 5.7)
Chronic obstructive pulmonary disease	Honduras	2000	All Ages	0.3 (0.1 to 0.4)	23.4% (12.9 to 34.1%)	4.3 (2.3 to 6.7)
Chronic obstructive pulmonary disease	Honduras	2005	All Ages	0.4 (0.2 to 0.6)	25.5% (15.0 to 36.5%)	5.2 (2.7 to 8.1)
Chronic obstructive pulmonary disease	Honduras	2010	All Ages	0.4 (0.2 to 0.6)	23.1% (12.5 to 34.3%)	5.2 (2.6 to 8.2)
Chronic obstructive pulmonary disease	Honduras	2015	All Ages	0.5 (0.3 to 0.7)	25.1% (14.3 to 36.4%)	5.7 (3.1 to 8.9)
Chronic obstructive pulmonary disease	Honduras	1990	Age-standardized		23.6% (13.2 to 34.7%)	8.7 (4.8 to 13.1)
Chronic obstructive pulmonary disease	Honduras	1995	Age-standardized		24.1% (13.4 to 35.2%)	10.8 (5.8 to 16.7)
Chronic obstructive pulmonary disease	Honduras	2000	Age-standardized		24.9% (14.3 to 36.2%)	11.7 (6.1 to 18.0)
Chronic obstructive pulmonary disease	Honduras	2005	Age-standardized		26.1% (15.2 to 37.4%)	12.8 (6.7 to 20.1)
Chronic obstructive pulmonary disease	Honduras	2010	Age-standardized		23.4% (12.6 to 34.5%)	11.5 (5.7 to 18.3)
Chronic obstructive pulmonary disease	Honduras	2015	Age-standardized		25.3% (14.4 to 36.6%)	11.3 (6.1 to 17.7)
All causes	Mexico	1990	All Ages	18.1 (15.0 to 21.7)	4.1% (3.4 to 4.9%)	21.2 (17.5 to 25.3)
All causes	Mexico	1995	All Ages	20.1 (17.0 to 23.7)	4.4% (3.7 to 5.2%)	21.3 (18.0 to 25.1)
All causes	Mexico	2000	All Ages	21.3 (18.1 to 24.9)	4.6% (3.9 to 5.4%)	20.8 (17.6 to 24.2)
All causes	Mexico	2005	All Ages	22.6 (19.1 to 26.3)	4.5% (3.8 to 5.2%)	20.6 (17.4 to 24.0)
All causes	Mexico	2010	All Ages	23.6 (19.6 to 27.8)	4.0% (3.3 to 4.7%)	19.9 (16.5 to 23.4)
All causes	Mexico	2015	All Ages	29.0 (23.9 to 34.6)	4.2% (3.5 to 5.0%)	22.8 (18.8 to 27.2)
All causes	Mexico	1990	Age-standardized		4.8% (4.0 to 5.6%)	41.3 (34.6 to 48.7)
All causes	Mexico	1995	Age-standardized		5.1% (4.3 to 5.9%)	41.0 (34.7 to 48.0)
All causes	Mexico	2000	Age-standardized		5.2% (4.4 to 6.1%)	38.7 (32.9 to 45.0)
All causes	Mexico	2005	Age-standardized		4.9% (4.1 to 5.8%)	35.5 (29.9 to 41.7)
All causes	Mexico	2010	Age-standardized		4.3% (3.6 to 5.1%)	30.9 (25.6 to 36.6)

Cause name	Location	Year	Age group	Number (in thousands)	PAF	Rate (per 100,000)
All causes	Mexico	2015	Age-standardized		4.4% (3.6 to 5.3%)	32.2 (26.5 to 38.6)
Lower respiratory infections	Mexico	1990	All Ages	6.0 (3.5 to 8.9)	19.7% (11.4 to 29.5%)	7.0 (4.0 to 10.4)
Lower respiratory infections	Mexico	1995	All Ages	5.4 (3.3 to 7.9)	20.8% (12.5 to 30.4%)	5.7 (3.4 to 8.4)
Lower respiratory infections	Mexico	2000	All Ages	4.7 (3.0 to 6.8)	21.9% (13.7 to 31.3%)	4.6 (2.9 to 6.6)
Lower respiratory infections	Mexico	2005	All Ages	4.2 (2.5 to 6.2)	20.4% (12.1 to 30.1%)	3.8 (2.3 to 5.7)
Lower respiratory infections	Mexico	2010	All Ages	3.7 (1.8 to 5.9)	16.8% (8.3 to 27.0%)	3.1 (1.5 to 5.0)
Lower respiratory infections	Mexico	2015	All Ages	4.2 (2.1 to 6.8)	16.8% (8.3 to 27.0%)	3.3 (1.6 to 5.4)
Lower respiratory infections	Mexico	1990	Age-standardized		19.7% (11.4 to 29.5%)	9.2 (5.3 to 13.7)
Lower respiratory infections	Mexico	1995	Age-standardized		20.8% (12.5 to 30.4%)	8.0 (4.8 to 11.7)
Lower respiratory infections	Mexico	2000	Age-standardized		21.8% (13.8 to 31.3%)	6.8 (4.2 to 9.8)
Lower respiratory infections	Mexico	2005	Age-standardized		20.4% (12.1 to 30.1%)	5.6 (3.3 to 8.4)
Lower respiratory infections	Mexico	2010	Age-standardized		16.8% (8.3 to 27.0%)	4.4 (2.1 to 7.0)
Lower respiratory infections	Mexico	2015	Age-standardized		16.8% (8.3 to 27.0%)	4.4 (2.2 to 7.2)
Tracheal, bronchus, and lung cancer	Mexico	1990	All Ages	0.7 (0.4 to 1.1)	13.0% (7.2 to 19.6%)	0.8 (0.5 to 1.2)
Tracheal, bronchus, and lung cancer	Mexico	1995	All Ages	0.9 (0.5 to 1.3)	13.7% (7.8 to 20.4%)	0.9 (0.5 to 1.3)
Tracheal, bronchus, and lung cancer	Mexico	2000	All Ages	1.0 (0.6 to 1.4)	14.6% (8.5 to 21.4%)	1.0 (0.5 to 1.4)
Tracheal, bronchus, and lung cancer	Mexico	2005	All Ages	1.0 (0.6 to 1.5)	13.7% (7.9 to 20.5%)	0.9 (0.5 to 1.4)
Tracheal, bronchus, and lung cancer	Mexico	2010	All Ages	0.9 (0.5 to 1.4)	11.2% (6.0 to 17.3%)	0.7 (0.4 to 1.2)
Tracheal, bronchus, and lung cancer	Mexico	2015	All Ages	1.1 (0.6 to 1.6)	11.3% (6.1 to 17.4%)	0.8 (0.4 to 1.3)
Tracheal, bronchus, and lung cancer	Mexico	1990	Age-standardized		13.0% (7.3 to 19.7%)	1.8 (1.0 to 2.7)
Tracheal, bronchus, and lung cancer	Mexico	1995	Age-standardized		13.8% (7.8 to 20.5%)	1.9 (1.1 to 2.8)
Tracheal, bronchus, and lung cancer	Mexico	2000	Age-standardized		14.7% (8.5 to 21.5%)	1.8 (1.1 to 2.7)
Tracheal, bronchus, and lung cancer	Mexico	2005	Age-standardized		13.8% (7.9 to 20.6%)	1.6 (0.9 to 2.4)
Tracheal, bronchus, and lung cancer	Mexico	2010	Age-standardized		11.3% (6.0 to 17.4%)	1.2 (0.6 to 1.8)
Tracheal, bronchus, and lung cancer	Mexico	2015	Age-standardized		11.3% (6.1 to 17.4%)	1.2 (0.6 to 1.8)
Cardiovascular diseases	Mexico	1990	All Ages	9.0 (7.6 to 10.5)	12.0% (10.2 to 14.0%)	10.5 (8.9 to 12.3)
Cardiovascular diseases	Mexico	1995	All Ages	10.8 (9.1 to 12.7)	12.5% (10.5 to 14.7%)	11.4 (9.6 to 13.4)
Cardiovascular diseases	Mexico	2000	All Ages	11.9 (10.0 to 13.9)	13.0% (11.0 to 15.1%)	11.6 (9.8 to 13.5)
Cardiovascular diseases	Mexico	2005	All Ages	13.2 (11.1 to 15.6)	12.6% (10.6 to 14.8%)	12.0 (10.1 to 14.2)
Cardiovascular diseases	Mexico	2010	All Ages	14.7 (12.1 to 17.4)	11.6% (9.5 to 13.7%)	12.4 (10.2 to 14.7)
Cardiovascular diseases	Mexico	2015	All Ages	18.3 (14.6 to 22.0)	11.3% (9.1 to 13.6%)	14.4 (11.5 to 17.3)
Cardiovascular diseases	Mexico	1990	Age-standardized		11.7% (9.7 to 13.8%)	23.3 (19.1 to 27.6)
Cardiovascular diseases	Mexico	1995	Age-standardized		12.2% (10.1 to 14.5%)	23.7 (19.6 to 28.1)
Cardiovascular diseases	Mexico	2000	Age-standardized		12.6% (10.4 to 14.9%)	22.4 (18.4 to 26.5)
Cardiovascular diseases	Mexico	2005	Age-standardized		12.3% (10.1 to 14.6%)	21.0 (17.3 to 25.1)
Cardiovascular diseases	Mexico	2010	Age-standardized		11.3% (9.1 to 13.5%)	19.3 (15.7 to 23.1)

Cause name	Location	Year	Age group	Number (in thousands)	PAF	Rate (per 100,000)
Cardiovascular diseases	Mexico	2015	Age-standardized		11.1% (8.8 to 13.4%)	20.2 (16.1 to 24.4)
Ischemic heart disease	Mexico	1990	All Ages	6.4 (5.2 to 7.7)	15.8% (12.6 to 18.9%)	7.5 (6.0 to 9.0)
Ischemic heart disease	Mexico	1995	All Ages	7.8 (6.3 to 9.4)	16.1% (12.9 to 19.4%)	8.3 (6.6 to 10.0)
Ischemic heart disease	Mexico	2000	All Ages	8.7 (7.0 to 10.4)	16.5% (13.3 to 19.7%)	8.4 (6.8 to 10.1)
Ischemic heart disease	Mexico	2005	All Ages	9.8 (7.7 to 11.8)	15.9% (12.6 to 19.2%)	8.9 (7.0 to 10.8)
Ischemic heart disease	Mexico	2010	All Ages	11.1 (8.7 to 13.6)	14.5% (11.3 to 17.7%)	9.4 (7.3 to 11.4)
Ischemic heart disease	Mexico	2015	All Ages	14.0 (10.5 to 17.5)	14.1% (10.6 to 17.6%)	11.0 (8.3 to 13.8)
Ischemic heart disease	Mexico	1990	Age-standardized		15.1% (11.6 to 18.5%)	16.7 (12.9 to 20.5)
Ischemic heart disease	Mexico	1995	Age-standardized		15.5% (12.0 to 18.9%)	17.3 (13.5 to 21.2)
Ischemic heart disease	Mexico	2000	Age-standardized		15.8% (12.3 to 19.4%)	16.3 (12.7 to 20.1)
Ischemic heart disease	Mexico	2005	Age-standardized		15.4% (12.0 to 18.8%)	15.6 (12.1 to 19.2)
Ischemic heart disease	Mexico	2010	Age-standardized		14.1% (10.8 to 17.4%)	14.6 (11.1 to 18.1)
Ischemic heart disease	Mexico	2015	Age-standardized		13.8% (10.3 to 17.3%)	15.5 (11.5 to 19.5)
Ischemic stroke	Mexico	1990	All Ages	1.2 (0.8 to 1.5)	11.1% (7.8 to 14.6%)	1.4 (1.0 to 1.8)
Ischemic stroke	Mexico	1995	All Ages	1.3 (0.9 to 1.7)	11.4% (8.0 to 14.9%)	1.4 (1.0 to 1.8)
Ischemic stroke	Mexico	2000	All Ages	1.4 (1.0 to 1.8)	11.7% (8.4 to 15.2%)	1.3 (1.0 to 1.7)
Ischemic stroke	Mexico	2005	All Ages	1.4 (1.0 to 1.9)	11.2% (7.8 to 14.8%)	1.3 (0.9 to 1.7)
Ischemic stroke	Mexico	2010	All Ages	1.5 (1.0 to 2.1)	10.0% (6.7 to 13.8%)	1.2 (0.8 to 1.7)
Ischemic stroke	Mexico	2015	All Ages	1.8 (1.2 to 2.6)	9.8% (6.2 to 14.0%)	1.4 (0.9 to 2.0)
Ischemic stroke	Mexico	1990	Age-standardized		10.8% (7.4 to 14.6%)	3.3 (2.2 to 4.4)
Ischemic stroke	Mexico	1995	Age-standardized		11.1% (7.7 to 14.9%)	3.0 (2.1 to 4.1)
Ischemic stroke	Mexico	2000	Age-standardized		11.5% (8.0 to 15.3%)	2.8 (1.9 to 3.7)
Ischemic stroke	Mexico	2005	Age-standardized		11.0% (7.5 to 14.8%)	2.4 (1.7 to 3.3)
Ischemic stroke	Mexico	2010	Age-standardized		9.9% (6.5 to 13.7%)	2.1 (1.4 to 2.9)
Ischemic stroke	Mexico	2015	Age-standardized		9.7% (6.2 to 13.9%)	2.1 (1.3 to 3.0)
Hemorrhagic stroke	Mexico	1990	All Ages	1.4 (1.1 to 1.7)	12.6% (10.1 to 15.1%)	1.6 (1.3 to 2.0)
Hemorrhagic stroke	Mexico	1995	All Ages	1.7 (1.4 to 2.0)	13.1% (10.5 to 15.7%)	1.8 (1.4 to 2.2)
Hemorrhagic stroke	Mexico	2000	All Ages	1.9 (1.5 to 2.2)	13.5% (10.9 to 16.2%)	1.8 (1.5 to 2.2)
Hemorrhagic stroke	Mexico	2005	All Ages	2.0 (1.6 to 2.4)	13.0% (10.4 to 15.7%)	1.8 (1.5 to 2.2)
Hemorrhagic stroke	Mexico	2010	All Ages	2.1 (1.6 to 2.6)	11.6% (9.0 to 14.3%)	1.7 (1.4 to 2.2)
Hemorrhagic stroke	Mexico	2015	All Ages	2.4 (1.9 to 3.1)	11.4% (8.7 to 14.3%)	1.9 (1.5 to 2.4)
Hemorrhagic stroke	Mexico	1990	Age-standardized		12.5% (9.8 to 15.4%)	3.4 (2.6 to 4.1)
Hemorrhagic stroke	Mexico	1995	Age-standardized		12.9% (10.1 to 15.8%)	3.5 (2.7 to 4.2)
Hemorrhagic stroke	Mexico	2000	Age-standardized		13.2% (10.4 to 16.2%)	3.3 (2.6 to 4.0)
Hemorrhagic stroke	Mexico	2005	Age-standardized		12.7% (9.8 to 15.5%)	3.0 (2.4 to 3.8)
Hemorrhagic stroke	Mexico	2010	Age-standardized		11.3% (8.6 to 14.1%)	2.6 (2.0 to 3.3)

Cause name	Location	Year	Age group	Number (in thousands)	PAF	Rate (per 100,000)
Hemorrhagic stroke	Mexico	2015	Age-standardized		11.1% (8.3 to 14.1%)	2.6 (1.9 to 3.3)
Chronic obstructive pulmonary disease	Mexico	1990	All Ages	2.5 (1.2 to 3.8)	18.3% (9.1 to 28.1%)	2.9 (1.4 to 4.4)
Chronic obstructive pulmonary disease	Mexico	1995	All Ages	3.1 (1.6 to 4.6)	20.0% (10.4 to 30.2%)	3.2 (1.7 to 4.9)
Chronic obstructive pulmonary disease	Mexico	2000	All Ages	3.7 (2.0 to 5.6)	21.6% (11.4 to 32.4%)	3.6 (1.9 to 5.5)
Chronic obstructive pulmonary disease	Mexico	2005	All Ages	4.2 (2.1 to 6.5)	20.7% (10.6 to 31.5%)	3.8 (2.0 to 5.9)
Chronic obstructive pulmonary disease	Mexico	2010	All Ages	4.3 (2.1 to 7.0)	17.9% (8.5 to 28.5%)	3.6 (1.7 to 5.9)
Chronic obstructive pulmonary disease	Mexico	2015	All Ages	5.5 (2.6 to 8.9)	17.9% (8.5 to 28.7%)	4.3 (2.1 to 7.0)
Chronic obstructive pulmonary disease	Mexico	1990	Age-standardized		19.7% (9.8 to 30.3%)	7.0 (3.5 to 10.7)
Chronic obstructive pulmonary disease	Mexico	1995	Age-standardized		20.7% (10.7 to 31.3%)	7.4 (3.8 to 11.2)
Chronic obstructive pulmonary disease	Mexico	2000	Age-standardized		21.9% (11.5 to 32.8%)	7.7 (4.0 to 11.5)
Chronic obstructive pulmonary disease	Mexico	2005	Age-standardized		20.8% (10.6 to 31.7%)	7.2 (3.7 to 11.1)
Chronic obstructive pulmonary disease	Mexico	2010	Age-standardized		18.0% (8.5 to 28.6%)	6.0 (2.9 to 9.7)
Chronic obstructive pulmonary disease	Mexico	2015	Age-standardized		17.9% (8.5 to 28.8%)	6.4 (3.1 to 10.3)
All causes	Nicaragua	1990	All Ages	0.8 (0.7 to 1.0)	4.2% (3.4 to 5.1%)	19.2 (15.7 to 23.6)
All causes	Nicaragua	1995	All Ages	0.8 (0.7 to 1.0)	4.4% (3.7 to 5.2%)	17.4 (14.5 to 20.6)
All causes	Nicaragua	2000	All Ages	0.8 (0.7 to 1.0)	4.8% (4.0 to 5.6%)	16.8 (14.1 to 19.8)
All causes	Nicaragua	2005	All Ages	1.0 (0.8 to 1.1)	5.1% (4.3 to 5.9%)	18.0 (15.1 to 21.2)
All causes	Nicaragua	2010	All Ages	1.0 (0.8 to 1.1)	4.5% (3.7 to 5.4%)	16.7 (13.8 to 19.9)
All causes	Nicaragua	2015	All Ages	1.2 (1.0 to 1.5)	5.1% (4.2 to 6.0%)	19.9 (16.4 to 24.1)
All causes	Nicaragua	1990	Age-standardized		5.7% (4.8 to 6.6%)	39.2 (33.1 to 45.8)
All causes	Nicaragua	1995	Age-standardized		5.7% (4.8 to 6.5%)	37.8 (31.8 to 44.2)
All causes	Nicaragua	2000	Age-standardized		5.8% (4.9 to 6.7%)	36.0 (30.0 to 42.3)
All causes	Nicaragua	2005	Age-standardized		5.9% (4.9 to 6.9%)	36.5 (30.6 to 43.2)
All causes	Nicaragua	2010	Age-standardized		5.0% (4.1 to 6.0%)	31.3 (25.7 to 37.6)
All causes	Nicaragua	2015	Age-standardized		5.5% (4.5 to 6.6%)	33.4 (27.2 to 40.5)
Lower respiratory infections	Nicaragua	1990	All Ages	0.4 (0.3 to 0.6)	23.6% (15.7 to 32.1%)	9.6 (6.5 to 13.5)
Lower respiratory infections	Nicaragua	1995	All Ages	0.3 (0.2 to 0.5)	23.5% (15.8 to 31.9%)	7.2 (4.7 to 10.1)
Lower respiratory infections	Nicaragua	2000	All Ages	0.3 (0.2 to 0.4)	24.0% (16.5 to 32.4%)	5.7 (3.8 to 8.0)
Lower respiratory infections	Nicaragua	2005	All Ages	0.3 (0.2 to 0.4)	23.0% (15.4 to 31.8%)	4.9 (3.2 to 6.9)
Lower respiratory infections	Nicaragua	2010	All Ages	0.2 (0.1 to 0.3)	18.0% (10.0 to 27.5%)	3.5 (1.9 to 5.5)
Lower respiratory infections	Nicaragua	2015	All Ages	0.2 (0.1 to 0.3)	20.5% (12.6 to 29.7%)	3.6 (2.1 to 5.4)
Lower respiratory infections	Nicaragua	1990	Age-standardized		23.6% (15.7 to 32.1%)	9.7 (6.4 to 13.5)
Lower respiratory infections	Nicaragua	1995	Age-standardized		23.5% (15.8 to 31.9%)	8.3 (5.6 to 11.6)
Lower respiratory infections	Nicaragua	2000	Age-standardized		24.0% (16.5 to 32.4%)	7.2 (4.9 to 10.2)
Lower respiratory infections	Nicaragua	2005	Age-standardized		23.0% (15.4 to 31.8%)	6.8 (4.4 to 9.6)
Lower respiratory infections	Nicaragua	2010	Age-standardized		18.0% (10.0 to 27.5%)	5.0 (2.7 to 7.7)

Cause name	Location	Year	Age group	Number (in thousands)	PAF	Rate (per 100,000)
Lower respiratory infections	Nicaragua	2015	Age-standardized		20.5% (12.6 to 29.7%)	5.1 (3.0 to 7.7)
Tracheal, bronchus, and lung cancer	Nicaragua	1990	All Ages	0.0 (0.0 to 0.0)	16.7% (10.1 to 23.9%)	0.4 (0.2 to 0.5)
Tracheal, bronchus, and lung cancer	Nicaragua	1995	All Ages	0.0 (0.0 to 0.0)	16.6% (10.0 to 23.7%)	0.4 (0.2 to 0.6)
Tracheal, bronchus, and lung cancer	Nicaragua	2000	All Ages	0.0 (0.0 to 0.0)	17.0% (10.2 to 24.5%)	0.4 (0.3 to 0.6)
Tracheal, bronchus, and lung cancer	Nicaragua	2005	All Ages	0.0 (0.0 to 0.0)	16.3% (9.6 to 23.3%)	0.5 (0.3 to 0.7)
Tracheal, bronchus, and lung cancer	Nicaragua	2010	All Ages	0.0 (0.0 to 0.0)	12.5% (6.8 to 19.0%)	0.4 (0.2 to 0.7)
Tracheal, bronchus, and lung cancer	Nicaragua	2015	All Ages	0.0 (0.0 to 0.1)	14.5% (8.3 to 21.7%)	0.6 (0.3 to 0.9)
Tracheal, bronchus, and lung cancer	Nicaragua	1990	Age-standardized		16.9% (10.2 to 24.2%)	1.0 (0.6 to 1.4)
Tracheal, bronchus, and lung cancer	Nicaragua	1995	Age-standardized		16.8% (10.1 to 23.9%)	1.0 (0.6 to 1.5)
Tracheal, bronchus, and lung cancer	Nicaragua	2000	Age-standardized		17.2% (10.3 to 24.8%)	1.1 (0.6 to 1.6)
Tracheal, bronchus, and lung cancer	Nicaragua	2005	Age-standardized		16.4% (9.6 to 23.5%)	1.1 (0.6 to 1.6)
Tracheal, bronchus, and lung cancer	Nicaragua	2010	Age-standardized		12.6% (6.9 to 19.1%)	0.8 (0.5 to 1.3)
Tracheal, bronchus, and lung cancer	Nicaragua	2015	Age-standardized		14.5% (8.3 to 21.8%)	1.0 (0.6 to 1.5)
Cardiovascular diseases	Nicaragua	1990	All Ages	0.4 (0.3 to 0.4)	13.4% (11.5 to 15.3%)	8.3 (7.1 to 9.6)
Cardiovascular diseases	Nicaragua	1995	All Ages	0.4 (0.4 to 0.5)	13.1% (11.2 to 15.0%)	8.8 (7.5 to 10.1)
Cardiovascular diseases	Nicaragua	2000	All Ages	0.5 (0.4 to 0.5)	13.3% (11.3 to 15.4%)	9.2 (7.8 to 10.7)
Cardiovascular diseases	Nicaragua	2005	All Ages	0.6 (0.5 to 0.7)	12.9% (10.8 to 15.2%)	10.5 (8.7 to 12.4)
Cardiovascular diseases	Nicaragua	2010	All Ages	0.6 (0.5 to 0.7)	11.2% (9.0 to 13.4%)	10.5 (8.5 to 12.7)
Cardiovascular diseases	Nicaragua	2015	All Ages	0.8 (0.6 to 1.0)	11.9% (9.6 to 14.3%)	12.8 (10.1 to 15.7)
Cardiovascular diseases	Nicaragua	1990	Age-standardized		12.7% (10.6 to 15.1%)	25.1 (20.6 to 29.8)
Cardiovascular diseases	Nicaragua	1995	Age-standardized		12.5% (10.2 to 14.7%)	24.8 (20.3 to 29.8)
Cardiovascular diseases	Nicaragua	2000	Age-standardized		12.7% (10.4 to 15.1%)	23.5 (19.1 to 28.2)
Cardiovascular diseases	Nicaragua	2005	Age-standardized		12.4% (10.1 to 15.0%)	23.5 (18.9 to 28.3)
Cardiovascular diseases	Nicaragua	2010	Age-standardized		10.9% (8.6 to 13.3%)	20.6 (16.3 to 25.1)
Cardiovascular diseases	Nicaragua	2015	Age-standardized		11.6% (9.2 to 14.1%)	21.9 (17.1 to 27.1)
Ischemic heart disease	Nicaragua	1990	All Ages	0.2 (0.2 to 0.3)	17.6% (14.5 to 20.9%)	5.4 (4.4 to 6.4)
Ischemic heart disease	Nicaragua	1995	All Ages	0.3 (0.2 to 0.3)	17.3% (14.2 to 20.4%)	5.8 (4.7 to 7.0)
Ischemic heart disease	Nicaragua	2000	All Ages	0.3 (0.3 to 0.4)	17.1% (13.8 to 20.4%)	6.3 (5.1 to 7.6)
Ischemic heart disease	Nicaragua	2005	All Ages	0.4 (0.3 to 0.5)	16.3% (12.8 to 20.0%)	7.3 (5.7 to 9.1)
Ischemic heart disease	Nicaragua	2010	All Ages	0.4 (0.3 to 0.5)	14.3% (10.8 to 17.8%)	7.5 (5.7 to 9.4)
Ischemic heart disease	Nicaragua	2015	All Ages	0.6 (0.4 to 0.7)	15.0% (11.4 to 18.8%)	9.3 (6.9 to 11.8)
Ischemic heart disease	Nicaragua	1990	Age-standardized		16.0% (12.3 to 19.9%)	16.4 (12.6 to 20.4)
Ischemic heart disease	Nicaragua	1995	Age-standardized		15.9% (12.3 to 19.7%)	16.6 (12.8 to 20.8)
Ischemic heart disease	Nicaragua	2000	Age-standardized		16.0% (12.2 to 19.9%)	16.3 (12.3 to 20.4)
Ischemic heart disease	Nicaragua	2005	Age-standardized		15.6% (11.8 to 19.6%)	16.5 (12.4 to 21.1)
Ischemic heart disease	Nicaragua	2010	Age-standardized		13.8% (10.1 to 17.5%)	14.8 (10.8 to 19.0)

Cause name	Location	Year	Age group	Number (in thousands)	PAF	Rate (per 100,000)
Ischemic heart disease	Nicaragua	2015	Age-standardized		14.6% (10.8 to 18.4%)	15.9 (11.5 to 20.4)
Ischemic stroke	Nicaragua	1990	All Ages	0.1 (0.0 to 0.1)	12.9% (9.8 to 15.9%)	1.4 (1.0 to 1.7)
Ischemic stroke	Nicaragua	1995	All Ages	0.1 (0.0 to 0.1)	12.7% (9.6 to 15.7%)	1.3 (1.0 to 1.7)
Ischemic stroke	Nicaragua	2000	All Ages	0.1 (0.0 to 0.1)	12.5% (9.2 to 16.0%)	1.2 (0.9 to 1.5)
Ischemic stroke	Nicaragua	2005	All Ages	0.1 (0.1 to 0.1)	11.9% (8.4 to 15.7%)	1.2 (0.8 to 1.6)
Ischemic stroke	Nicaragua	2010	All Ages	0.1 (0.0 to 0.1)	10.0% (6.6 to 13.9%)	1.2 (0.8 to 1.6)
Ischemic stroke	Nicaragua	2015	All Ages	0.1 (0.1 to 0.1)	10.8% (7.2 to 15.1%)	1.4 (0.9 to 2.0)
Ischemic stroke	Nicaragua	1990	Age-standardized		12.0% (8.6 to 15.7%)	4.7 (3.2 to 6.3)
Ischemic stroke	Nicaragua	1995	Age-standardized		12.0% (8.4 to 15.7%)	4.2 (2.9 to 5.6)
Ischemic stroke	Nicaragua	2000	Age-standardized		12.0% (8.4 to 15.9%)	3.3 (2.3 to 4.4)
Ischemic stroke	Nicaragua	2005	Age-standardized		11.5% (7.9 to 15.7%)	2.9 (2.0 to 4.0)
Ischemic stroke	Nicaragua	2010	Age-standardized		9.9% (6.4 to 13.9%)	2.4 (1.6 to 3.5)
Ischemic stroke	Nicaragua	2015	Age-standardized		10.7% (7.1 to 15.0%)	2.6 (1.7 to 3.7)
Hemorrhagic stroke	Nicaragua	1990	All Ages	0.1 (0.1 to 0.1)	14.3% (11.7 to 17.0%)	1.6 (1.3 to 1.9)
Hemorrhagic stroke	Nicaragua	1995	All Ages	0.1 (0.1 to 0.1)	14.4% (11.7 to 17.1%)	1.6 (1.3 to 2.0)
Hemorrhagic stroke	Nicaragua	2000	All Ages	0.1 (0.1 to 0.1)	14.4% (11.5 to 17.0%)	1.7 (1.4 to 2.1)
Hemorrhagic stroke	Nicaragua	2005	All Ages	0.1 (0.1 to 0.1)	13.8% (10.9 to 16.6%)	1.9 (1.5 to 2.3)
Hemorrhagic stroke	Nicaragua	2010	All Ages	0.1 (0.1 to 0.1)	11.7% (9.0 to 14.6%)	1.8 (1.4 to 2.2)
Hemorrhagic stroke	Nicaragua	2015	All Ages	0.1 (0.1 to 0.2)	12.6% (9.7 to 15.7%)	2.1 (1.6 to 2.7)
Hemorrhagic stroke	Nicaragua	1990	Age-standardized		14.1% (11.2 to 17.1%)	4.0 (3.1 to 5.1)
Hemorrhagic stroke	Nicaragua	1995	Age-standardized		13.9% (11.1 to 16.8%)	4.0 (3.2 to 4.9)
Hemorrhagic stroke	Nicaragua	2000	Age-standardized		13.8% (10.8 to 16.8%)	4.0 (3.1 to 4.9)
Hemorrhagic stroke	Nicaragua	2005	Age-standardized		13.3% (10.3 to 16.3%)	4.0 (3.1 to 5.0)
Hemorrhagic stroke	Nicaragua	2010	Age-standardized		11.3% (8.5 to 14.4%)	3.3 (2.4 to 4.3)
Hemorrhagic stroke	Nicaragua	2015	Age-standardized		12.2% (9.1 to 15.3%)	3.5 (2.5 to 4.5)
Chronic obstructive pulmonary disease	Nicaragua	1990	All Ages	0.0 (0.0 to 0.1)	17.7% (9.7 to 27.0%)	0.9 (0.5 to 1.4)
Chronic obstructive pulmonary disease	Nicaragua	1995	All Ages	0.1 (0.0 to 0.1)	20.8% (11.5 to 30.8%)	1.1 (0.6 to 1.6)
Chronic obstructive pulmonary disease	Nicaragua	2000	All Ages	0.1 (0.0 to 0.1)	22.7% (12.8 to 33.4%)	1.4 (0.8 to 2.1)
Chronic obstructive pulmonary disease	Nicaragua	2005	All Ages	0.1 (0.1 to 0.2)	22.4% (12.1 to 32.9%)	2.1 (1.1 to 3.1)
Chronic obstructive pulmonary disease	Nicaragua	2010	All Ages	0.1 (0.1 to 0.2)	18.5% (9.2 to 29.0%)	2.3 (1.1 to 3.5)
Chronic obstructive pulmonary disease	Nicaragua	2015	All Ages	0.2 (0.1 to 0.3)	20.6% (10.7 to 31.4%)	2.9 (1.5 to 4.5)
Chronic obstructive pulmonary disease	Nicaragua	1990	Age-standardized		22.2% (12.6 to 32.9%)	3.4 (1.9 to 5.1)
Chronic obstructive pulmonary disease	Nicaragua	1995	Age-standardized		22.7% (12.7 to 33.4%)	3.6 (1.9 to 5.4)
Chronic obstructive pulmonary disease	Nicaragua	2000	Age-standardized		23.4% (13.1 to 34.4%)	4.2 (2.3 to 6.2)
Chronic obstructive pulmonary disease	Nicaragua	2005	Age-standardized		22.7% (12.3 to 33.3%)	5.1 (2.7 to 7.6)
Chronic obstructive pulmonary disease	Nicaragua	2010	Age-standardized		18.7% (9.3 to 29.3%)	4.8 (2.4 to 7.5)

Cause name	Location	Year	Age group	Number (in thousands)	PAF	Rate (per 100,000)
Chronic obstructive pulmonary disease	Nicaragua	2015	Age-standardized		20.7% (10.7 to 31.5%)	5.3 (2.8 to 8.3)
All causes	Panama	1990	All Ages	0.5 (0.4 to 0.6)	4.3% (3.6 to 5.1%)	18.8 (15.4 to 22.5)
All causes	Panama	1995	All Ages	0.5 (0.4 to 0.6)	4.1% (3.4 to 5.0%)	18.4 (15.0 to 22.3)
All causes	Panama	2000	All Ages	0.5 (0.4 to 0.7)	4.0% (3.3 to 5.0%)	17.8 (14.3 to 22.0)
All causes	Panama	2005	All Ages	0.6 (0.5 to 0.7)	3.8% (3.0 to 4.7%)	17.1 (13.4 to 21.4)
All causes	Panama	2010	All Ages	0.6 (0.4 to 0.7)	3.1% (2.3 to 4.0%)	15.2 (11.4 to 19.8)
All causes	Panama	2015	All Ages	0.8 (0.6 to 1.0)	3.8% (2.9 to 4.7%)	19.2 (14.3 to 24.5)
All causes	Panama	1990	Age-standardized		5.2% (4.2 to 6.1%)	37.8 (30.6 to 45.4)
All causes	Panama	1995	Age-standardized		4.8% (3.9 to 5.8%)	34.2 (27.3 to 41.8)
All causes	Panama	2000	Age-standardized		4.6% (3.7 to 5.7%)	30.3 (24.3 to 37.7)
All causes	Panama	2005	Age-standardized		4.1% (3.3 to 5.1%)	26.4 (20.5 to 33.0)
All causes	Panama	2010	Age-standardized		3.3% (2.5 to 4.3%)	21.0 (15.8 to 27.2)
All causes	Panama	2015	Age-standardized		3.9% (3.0 to 4.9%)	23.6 (17.5 to 30.1)
Lower respiratory infections	Panama	1990	All Ages	0.1 (0.0 to 0.1)	13.6% (5.6 to 24.0%)	2.6 (1.1 to 4.9)
Lower respiratory infections	Panama	1995	All Ages	0.1 (0.0 to 0.1)	13.3% (5.3 to 23.9%)	2.6 (1.0 to 4.7)
Lower respiratory infections	Panama	2000	All Ages	0.1 (0.0 to 0.1)	13.9% (5.9 to 24.7%)	2.7 (1.1 to 4.8)
Lower respiratory infections	Panama	2005	All Ages	0.1 (0.0 to 0.2)	12.2% (4.4 to 22.9%)	2.7 (1.0 to 5.1)
Lower respiratory infections	Panama	2010	All Ages	0.1 (0.0 to 0.2)	8.7% (2.0 to 19.4%)	2.3 (0.5 to 5.2)
Lower respiratory infections	Panama	2015	All Ages	0.1 (0.0 to 0.2)	11.4% (3.7 to 22.6%)	3.0 (1.0 to 6.1)
Lower respiratory infections	Panama	1990	Age-standardized		13.6% (5.6 to 24.0%)	4.3 (1.8 to 7.9)
Lower respiratory infections	Panama	1995	Age-standardized		13.3% (5.3 to 23.9%)	3.9 (1.6 to 7.4)
Lower respiratory infections	Panama	2000	Age-standardized		13.9% (5.9 to 24.7%)	3.9 (1.6 to 7.0)
Lower respiratory infections	Panama	2005	Age-standardized		12.2% (4.4 to 22.9%)	3.8 (1.4 to 7.2)
Lower respiratory infections	Panama	2010	Age-standardized		8.7% (2.0 to 19.4%)	2.9 (0.7 to 6.7)
Lower respiratory infections	Panama	2015	Age-standardized		11.4% (3.7 to 22.6%)	3.6 (1.2 to 7.2)
Tracheal, bronchus, and lung cancer	Panama	1990	All Ages	0.0 (0.0 to 0.0)	9.4% (4.8 to 15.2%)	0.8 (0.4 to 1.3)
Tracheal, bronchus, and lung cancer	Panama	1995	All Ages	0.0 (0.0 to 0.0)	9.2% (4.6 to 14.7%)	0.8 (0.4 to 1.3)
Tracheal, bronchus, and lung cancer	Panama	2000	All Ages	0.0 (0.0 to 0.0)	9.6% (4.9 to 15.6%)	0.8 (0.4 to 1.4)
Tracheal, bronchus, and lung cancer	Panama	2005	All Ages	0.0 (0.0 to 0.0)	8.4% (4.1 to 13.8%)	0.7 (0.3 to 1.2)
Tracheal, bronchus, and lung cancer	Panama	2010	All Ages	0.0 (0.0 to 0.0)	5.8% (2.4 to 10.2%)	0.5 (0.2 to 0.9)
Tracheal, bronchus, and lung cancer	Panama	2015	All Ages	0.0 (0.0 to 0.1)	7.8% (3.8 to 12.9%)	0.8 (0.4 to 1.3)
Tracheal, bronchus, and lung cancer	Panama	1990	Age-standardized		9.4% (4.8 to 15.2%)	1.6 (0.8 to 2.6)
Tracheal, bronchus, and lung cancer	Panama	1995	Age-standardized		9.2% (4.6 to 14.7%)	1.5 (0.7 to 2.4)
Tracheal, bronchus, and lung cancer	Panama	2000	Age-standardized		9.7% (4.9 to 15.6%)	1.4 (0.7 to 2.3)
Tracheal, bronchus, and lung cancer	Panama	2005	Age-standardized		8.4% (4.1 to 13.8%)	1.1 (0.5 to 1.8)
Tracheal, bronchus, and lung cancer	Panama	2010	Age-standardized		5.8% (2.4 to 10.2%)	0.7 (0.3 to 1.3)

Cause name	Location	Year	Age group	Number (in thousands)	PAF	Rate (per 100,000)
Tracheal, bronchus, and lung cancer	Panama	2015	Age-standardized		7.8% (3.8 to 12.9%)	1.0 (0.5 to 1.6)
Cardiovascular diseases	Panama	1990	All Ages	0.3 (0.3 to 0.4)	10.5% (8.6 to 12.4%)	13.6 (11.1 to 16.3)
Cardiovascular diseases	Panama	1995	All Ages	0.4 (0.3 to 0.4)	10.1% (8.1 to 12.0%)	12.9 (10.5 to 15.5)
Cardiovascular diseases	Panama	2000	All Ages	0.4 (0.3 to 0.4)	9.9% (7.9 to 12.0%)	11.8 (9.4 to 14.4)
Cardiovascular diseases	Panama	2005	All Ages	0.4 (0.3 to 0.5)	9.3% (7.3 to 11.3%)	11.4 (8.8 to 14.0)
Cardiovascular diseases	Panama	2010	All Ages	0.4 (0.3 to 0.5)	7.8% (5.8 to 9.8%)	10.6 (7.9 to 13.5)
Cardiovascular diseases	Panama	2015	All Ages	0.5 (0.4 to 0.7)	8.8% (6.8 to 11.1%)	12.9 (9.6 to 16.6)
Cardiovascular diseases	Panama	1990	Age-standardized		10.3% (8.2 to 12.4%)	28.1 (22.4 to 33.9)
Cardiovascular diseases	Panama	1995	Age-standardized		9.9% (7.8 to 11.9%)	24.5 (19.3 to 29.8)
Cardiovascular diseases	Panama	2000	Age-standardized		9.7% (7.6 to 12.0%)	20.5 (16.0 to 25.3)
Cardiovascular diseases	Panama	2005	Age-standardized		9.1% (7.1 to 11.3%)	17.8 (13.6 to 22.2)
Cardiovascular diseases	Panama	2010	Age-standardized		7.7% (5.7 to 9.8%)	14.7 (10.8 to 18.9)
Cardiovascular diseases	Panama	2015	Age-standardized		8.7% (6.7 to 11.0%)	15.9 (11.8 to 20.4)
Ischemic heart disease	Panama	1990	All Ages	0.2 (0.2 to 0.3)	13.2% (10.2 to 16.2%)	9.3 (7.1 to 11.5)
Ischemic heart disease	Panama	1995	All Ages	0.2 (0.2 to 0.3)	13.0% (9.8 to 16.1%)	8.4 (6.3 to 10.6)
Ischemic heart disease	Panama	2000	All Ages	0.2 (0.2 to 0.3)	13.1% (9.8 to 16.5%)	7.4 (5.4 to 9.3)
Ischemic heart disease	Panama	2005	All Ages	0.2 (0.2 to 0.3)	12.3% (9.0 to 15.6%)	7.3 (5.3 to 9.4)
Ischemic heart disease	Panama	2010	All Ages	0.3 (0.2 to 0.4)	10.5% (7.3 to 13.7%)	7.2 (5.1 to 9.5)
Ischemic heart disease	Panama	2015	All Ages	0.4 (0.2 to 0.5)	11.7% (8.2 to 15.1%)	8.8 (6.1 to 11.7)
Ischemic heart disease	Panama	1990	Age-standardized		12.6% (9.3 to 15.9%)	19.2 (14.2 to 24.5)
Ischemic heart disease	Panama	1995	Age-standardized		12.5% (9.1 to 15.8%)	15.9 (11.7 to 20.2)
Ischemic heart disease	Panama	2000	Age-standardized		12.7% (9.2 to 16.2%)	12.8 (9.2 to 16.4)
Ischemic heart disease	Panama	2005	Age-standardized		12.0% (8.6 to 15.4%)	11.4 (8.0 to 14.9)
Ischemic heart disease	Panama	2010	Age-standardized		10.2% (7.1 to 13.6%)	10.0 (6.9 to 13.3)
Ischemic heart disease	Panama	2015	Age-standardized		11.6% (8.1 to 15.0%)	10.8 (7.4 to 14.5)
Ischemic stroke	Panama	1990	All Ages	0.1 (0.0 to 0.1)	9.1% (5.9 to 12.5%)	2.3 (1.5 to 3.3)
Ischemic stroke	Panama	1995	All Ages	0.1 (0.0 to 0.1)	8.8% (5.4 to 12.5%)	2.3 (1.4 to 3.4)
Ischemic stroke	Panama	2000	All Ages	0.1 (0.0 to 0.1)	8.8% (5.3 to 13.1%)	2.1 (1.2 to 3.1)
Ischemic stroke	Panama	2005	All Ages	0.1 (0.0 to 0.1)	8.1% (4.7 to 12.4%)	1.8 (1.0 to 2.8)
Ischemic stroke	Panama	2010	All Ages	0.1 (0.0 to 0.1)	6.7% (3.4 to 10.9%)	1.5 (0.8 to 2.5)
Ischemic stroke	Panama	2015	All Ages	0.1 (0.0 to 0.1)	7.7% (4.2 to 12.5%)	1.9 (1.0 to 3.1)
Ischemic stroke	Panama	1990	Age-standardized		8.9% (5.6 to 12.6%)	5.1 (3.1 to 7.3)
Ischemic stroke	Panama	1995	Age-standardized		8.6% (5.1 to 12.5%)	4.7 (2.8 to 6.9)
Ischemic stroke	Panama	2000	Age-standardized		8.7% (5.1 to 13.1%)	3.8 (2.2 to 5.7)
Ischemic stroke	Panama	2005	Age-standardized		8.1% (4.6 to 12.4%)	3.0 (1.7 to 4.6)
Ischemic stroke	Panama	2010	Age-standardized		6.6% (3.3 to 10.9%)	2.2 (1.1 to 3.6)

Cause name	Location	Year	Age group	Number (in thousands)	PAF	Rate (per 100,000)
Ischemic stroke	Panama	2015	Age-standardized		7.7% (4.2 to 12.4%)	2.4 (1.3 to 3.8)
Hemorrhagic stroke	Panama	1990	All Ages	0.1 (0.0 to 0.1)	10.3% (7.8 to 12.9%)	2.0 (1.5 to 2.5)
Hemorrhagic stroke	Panama	1995	All Ages	0.1 (0.0 to 0.1)	10.0% (7.4 to 12.7%)	2.2 (1.6 to 2.8)
Hemorrhagic stroke	Panama	2000	All Ages	0.1 (0.1 to 0.1)	10.1% (7.3 to 13.0%)	2.4 (1.7 to 3.1)
Hemorrhagic stroke	Panama	2005	All Ages	0.1 (0.1 to 0.1)	9.3% (6.6 to 12.3%)	2.3 (1.6 to 3.0)
Hemorrhagic stroke	Panama	2010	All Ages	0.1 (0.0 to 0.1)	7.6% (5.0 to 10.7%)	1.9 (1.2 to 2.7)
Hemorrhagic stroke	Panama	2015	All Ages	0.1 (0.1 to 0.1)	8.8% (6.0 to 12.1%)	2.2 (1.5 to 3.2)
Hemorrhagic stroke	Panama	1990	Age-standardized		10.1% (7.4 to 12.9%)	3.8 (2.7 to 4.9)
Hemorrhagic stroke	Panama	1995	Age-standardized		9.7% (7.0 to 12.7%)	3.9 (2.8 to 5.2)
Hemorrhagic stroke	Panama	2000	Age-standardized		9.8% (6.9 to 12.9%)	3.9 (2.7 to 5.3)
Hemorrhagic stroke	Panama	2005	Age-standardized		9.0% (6.2 to 12.3%)	3.4 (2.3 to 4.6)
Hemorrhagic stroke	Panama	2010	Age-standardized		7.4% (4.8 to 10.6%)	2.6 (1.6 to 3.7)
Hemorrhagic stroke	Panama	2015	Age-standardized		8.7% (5.8 to 12.0%)	2.7 (1.8 to 3.8)
Chronic obstructive pulmonary disease	Panama	1990	All Ages	0.0 (0.0 to 0.1)	13.5% (5.9 to 22.5%)	1.7 (0.8 to 2.9)
Chronic obstructive pulmonary disease	Panama	1995	All Ages	0.1 (0.0 to 0.1)	14.4% (6.1 to 24.2%)	2.1 (0.9 to 3.6)
Chronic obstructive pulmonary disease	Panama	2000	All Ages	0.1 (0.0 to 0.1)	15.3% (6.6 to 25.8%)	2.5 (1.1 to 4.1)
Chronic obstructive pulmonary disease	Panama	2005	All Ages	0.1 (0.0 to 0.1)	13.9% (5.6 to 23.5%)	2.3 (0.9 to 3.8)
Chronic obstructive pulmonary disease	Panama	2010	All Ages	0.1 (0.0 to 0.1)	10.6% (3.6 to 19.8%)	1.8 (0.6 to 3.3)
Chronic obstructive pulmonary disease	Panama	2015	All Ages	0.1 (0.0 to 0.2)	13.2% (5.4 to 22.8%)	2.4 (1.0 to 4.3)
Chronic obstructive pulmonary disease	Panama	1990	Age-standardized		14.7% (6.5 to 24.6%)	3.8 (1.7 to 6.4)
Chronic obstructive pulmonary disease	Panama	1995	Age-standardized		14.9% (6.4 to 25.2%)	4.3 (1.8 to 7.2)
Chronic obstructive pulmonary disease	Panama	2000	Age-standardized		15.5% (6.7 to 26.2%)	4.6 (1.9 to 7.6)
Chronic obstructive pulmonary disease	Panama	2005	Age-standardized		14.0% (5.7 to 23.8%)	3.7 (1.5 to 6.3)
Chronic obstructive pulmonary disease	Panama	2010	Age-standardized		10.7% (3.6 to 20.0%)	2.6 (0.9 to 4.8)
Chronic obstructive pulmonary disease	Panama	2015	Age-standardized		13.3% (5.4 to 22.9%)	3.1 (1.2 to 5.4)
All causes	Venezuela	1990	All Ages	5.6 (4.9 to 6.3)	6.3% (5.5 to 7.1%)	28.0 (24.5 to 31.6)
All causes	Venezuela	1995	All Ages	6.1 (5.3 to 6.9)	5.9% (5.2 to 6.7%)	27.3 (23.8 to 30.9)
All causes	Venezuela	2000	All Ages	6.8 (6.0 to 7.7)	6.1% (5.3 to 6.9%)	27.6 (24.3 to 31.5)
All causes	Venezuela	2005	All Ages	7.1 (6.2 to 8.1)	5.7% (4.9 to 6.5%)	26.6 (23.0 to 30.4)
All causes	Venezuela	2010	All Ages	7.5 (6.4 to 8.6)	5.2% (4.4 to 6.0%)	25.7 (22.0 to 29.6)
All causes	Venezuela	2015	All Ages	9.3 (7.8 to 11.1)	5.5% (4.7 to 6.3%)	30.0 (25.0 to 35.6)
All causes	Venezuela	1990	Age-standardized		7.5% (6.4 to 8.6%)	66.7 (57.1 to 76.3)
All causes	Venezuela	1995	Age-standardized		7.0% (5.9 to 8.0%)	61.8 (52.6 to 71.3)
All causes	Venezuela	2000	Age-standardized		7.0% (6.0 to 8.1%)	57.8 (49.5 to 66.7)
All causes	Venezuela	2005	Age-standardized		6.5% (5.5 to 7.5%)	50.4 (42.7 to 58.4)
All causes	Venezuela	2010	Age-standardized		5.7% (4.8 to 6.7%)	43.5 (36.6 to 51.3)

Cause name	Location	Year	Age group	Number (in thousands)	PAF	Rate (per 100,000)
All causes	Venezuela	2015	Age-standardized		5.9% (5.0 to 6.9%)	45.1 (37.0 to 54.1)
Lower respiratory infections	Venezuela	1990	All Ages	1.1 (0.7 to 1.4)	23.8% (15.6 to 32.7%)	5.3 (3.5 to 7.2)
Lower respiratory infections	Venezuela	1995	All Ages	1.0 (0.6 to 1.4)	22.1% (13.7 to 31.6%)	4.4 (2.7 to 6.3)
Lower respiratory infections	Venezuela	2000	All Ages	1.0 (0.7 to 1.4)	23.9% (15.7 to 33.0%)	4.0 (2.7 to 5.5)
Lower respiratory infections	Venezuela	2005	All Ages	1.0 (0.6 to 1.5)	21.9% (13.7 to 31.5%)	3.8 (2.4 to 5.4)
Lower respiratory infections	Venezuela	2010	All Ages	1.0 (0.5 to 1.5)	18.4% (9.9 to 28.4%)	3.4 (1.8 to 5.2)
Lower respiratory infections	Venezuela	2015	All Ages	1.2 (0.6 to 1.8)	19.0% (10.7 to 28.9%)	3.7 (2.1 to 5.7)
Lower respiratory infections	Venezuela	1990	Age-standardized		23.8% (15.6 to 32.7%)	9.8 (6.4 to 13.4)
Lower respiratory infections	Venezuela	1995	Age-standardized		22.1% (13.7 to 31.6%)	8.3 (5.1 to 11.9)
Lower respiratory infections	Venezuela	2000	Age-standardized		23.9% (15.7 to 33.0%)	7.6 (5.0 to 10.3)
Lower respiratory infections	Venezuela	2005	Age-standardized		21.9% (13.7 to 31.5%)	6.7 (4.1 to 9.5)
Lower respiratory infections	Venezuela	2010	Age-standardized		18.4% (9.9 to 28.4%)	5.5 (3.0 to 8.6)
Lower respiratory infections	Venezuela	2015	Age-standardized		19.0% (10.7 to 28.9%)	5.6 (3.1 to 8.5)
Tracheal, bronchus, and lung cancer	Venezuela	1990	All Ages	0.3 (0.2 to 0.4)	17.0% (10.1 to 24.2%)	1.4 (0.8 to 2.1)
Tracheal, bronchus, and lung cancer	Venezuela	1995	All Ages	0.3 (0.2 to 0.5)	15.7% (9.1 to 22.9%)	1.4 (0.8 to 2.1)
Tracheal, bronchus, and lung cancer	Venezuela	2000	All Ages	0.4 (0.3 to 0.6)	17.0% (10.1 to 24.1%)	1.7 (1.0 to 2.4)
Tracheal, bronchus, and lung cancer	Venezuela	2005	All Ages	0.5 (0.3 to 0.7)	15.5% (9.1 to 22.8%)	1.7 (1.0 to 2.5)
Tracheal, bronchus, and lung cancer	Venezuela	2010	All Ages	0.5 (0.3 to 0.7)	12.9% (7.1 to 19.4%)	1.6 (0.9 to 2.4)
Tracheal, bronchus, and lung cancer	Venezuela	2015	All Ages	0.6 (0.3 to 1.0)	13.4% (7.5 to 20.0%)	2.0 (1.1 to 3.0)
Tracheal, bronchus, and lung cancer	Venezuela	1990	Age-standardized		17.0% (10.1 to 24.3%)	3.3 (1.9 to 4.8)
Tracheal, bronchus, and lung cancer	Venezuela	1995	Age-standardized		15.7% (9.1 to 22.9%)	3.1 (1.8 to 4.6)
Tracheal, bronchus, and lung cancer	Venezuela	2000	Age-standardized		17.0% (10.2 to 24.2%)	3.3 (2.0 to 4.8)
Tracheal, bronchus, and lung cancer	Venezuela	2005	Age-standardized		15.6% (9.1 to 22.8%)	3.0 (1.8 to 4.4)
Tracheal, bronchus, and lung cancer	Venezuela	2010	Age-standardized		12.9% (7.1 to 19.4%)	2.6 (1.5 to 3.9)
Tracheal, bronchus, and lung cancer	Venezuela	2015	Age-standardized		13.4% (7.5 to 20.0%)	2.8 (1.6 to 4.4)
Cardiovascular diseases	Venezuela	1990	All Ages	3.9 (3.3 to 4.4)	14.3% (12.3 to 16.4%)	19.5 (16.8 to 22.3)
Cardiovascular diseases	Venezuela	1995	All Ages	4.3 (3.7 to 4.9)	13.7% (11.7 to 15.8%)	19.3 (16.5 to 22.3)
Cardiovascular diseases	Venezuela	2000	All Ages	4.7 (4.0 to 5.4)	14.0% (12.0 to 16.2%)	19.1 (16.4 to 22.1)
Cardiovascular diseases	Venezuela	2005	All Ages	4.9 (4.1 to 5.7)	13.3% (11.3 to 15.5%)	18.3 (15.5 to 21.4)
Cardiovascular diseases	Venezuela	2010	All Ages	5.2 (4.4 to 6.2)	12.3% (10.4 to 14.4%)	18.1 (15.2 to 21.2)
Cardiovascular diseases	Venezuela	2015	All Ages	6.5 (5.3 to 7.9)	12.4% (10.4 to 14.6%)	21.1 (17.1 to 25.3)
Cardiovascular diseases	Venezuela	1990	Age-standardized		13.2% (11.0 to 15.6%)	48.3 (40.1 to 57.0)
Cardiovascular diseases	Venezuela	1995	Age-standardized		12.6% (10.4 to 15.0%)	44.6 (36.8 to 53.0)
Cardiovascular diseases	Venezuela	2000	Age-standardized		13.0% (10.8 to 15.4%)	40.1 (33.3 to 47.7)
Cardiovascular diseases	Venezuela	2005	Age-standardized		12.3% (10.1 to 14.7%)	34.5 (28.2 to 41.3)
Cardiovascular diseases	Venezuela	2010	Age-standardized		11.5% (9.3 to 13.7%)	30.3 (24.7 to 36.3)

Cause name	Location	Year	Age group	Number (in thousands)	PAF	Rate (per 100,000)
Cardiovascular diseases	Venezuela	2015	Age-standardized		11.7% (9.5 to 13.9%)	31.2 (24.9 to 37.9)
Ischemic heart disease	Venezuela	1990	All Ages	2.9 (2.4 to 3.4)	18.2% (15.1 to 21.4%)	14.4 (11.9 to 17.1)
Ischemic heart disease	Venezuela	1995	All Ages	3.2 (2.6 to 3.8)	17.4% (14.4 to 20.6%)	14.3 (11.7 to 17.0)
Ischemic heart disease	Venezuela	2000	All Ages	3.5 (2.8 to 4.1)	17.9% (14.7 to 21.3%)	14.1 (11.6 to 16.9)
Ischemic heart disease	Venezuela	2005	All Ages	3.6 (3.0 to 4.4)	17.0% (13.9 to 20.4%)	13.6 (11.0 to 16.4)
Ischemic heart disease	Venezuela	2010	All Ages	3.9 (3.2 to 4.8)	15.8% (12.7 to 18.9%)	13.6 (10.9 to 16.4)
Ischemic heart disease	Venezuela	2015	All Ages	5.0 (3.9 to 6.1)	15.9% (12.7 to 19.0%)	16.0 (12.5 to 19.7)
Ischemic heart disease	Venezuela	1990	Age-standardized		16.6% (13.1 to 20.3%)	35.9 (28.2 to 43.7)
Ischemic heart disease	Venezuela	1995	Age-standardized		16.1% (12.5 to 19.6%)	33.2 (25.9 to 40.6)
Ischemic heart disease	Venezuela	2000	Age-standardized		16.6% (13.1 to 20.4%)	29.5 (23.0 to 36.4)
Ischemic heart disease	Venezuela	2005	Age-standardized		15.9% (12.2 to 19.5%)	25.6 (19.8 to 31.5)
Ischemic heart disease	Venezuela	2010	Age-standardized		14.8% (11.3 to 18.2%)	22.6 (17.3 to 28.0)
Ischemic heart disease	Venezuela	2015	Age-standardized		15.0% (11.4 to 18.5%)	23.5 (17.8 to 29.4)
Ischemic stroke	Venezuela	1990	All Ages	0.5 (0.4 to 0.6)	13.0% (9.8 to 16.1%)	2.4 (1.8 to 3.0)
Ischemic stroke	Venezuela	1995	All Ages	0.5 (0.3 to 0.6)	12.1% (8.9 to 15.4%)	2.0 (1.5 to 2.6)
Ischemic stroke	Venezuela	2000	All Ages	0.4 (0.3 to 0.5)	12.3% (8.8 to 16.1%)	1.7 (1.2 to 2.2)
Ischemic stroke	Venezuela	2005	All Ages	0.4 (0.3 to 0.6)	11.5% (7.9 to 15.4%)	1.6 (1.1 to 2.2)
Ischemic stroke	Venezuela	2010	All Ages	0.5 (0.3 to 0.6)	10.4% (6.9 to 14.4%)	1.6 (1.0 to 2.2)
Ischemic stroke	Venezuela	2015	All Ages	0.6 (0.4 to 0.8)	10.5% (7.0 to 14.6%)	1.8 (1.2 to 2.5)
Ischemic stroke	Venezuela	1990	Age-standardized		12.2% (8.7 to 16.0%)	6.9 (4.9 to 9.1)
Ischemic stroke	Venezuela	1995	Age-standardized		11.5% (8.0 to 15.4%)	5.4 (3.7 to 7.3)
Ischemic stroke	Venezuela	2000	Age-standardized		11.8% (8.1 to 16.1%)	4.1 (2.8 to 5.6)
Ischemic stroke	Venezuela	2005	Age-standardized		11.2% (7.3 to 15.5%)	3.5 (2.3 to 4.9)
Ischemic stroke	Venezuela	2010	Age-standardized		10.1% (6.4 to 14.5%)	3.0 (1.9 to 4.3)
Ischemic stroke	Venezuela	2015	Age-standardized		10.3% (6.6 to 14.6%)	3.0 (1.9 to 4.4)
Hemorrhagic stroke	Venezuela	1990	All Ages	0.5 (0.4 to 0.6)	15.5% (12.8 to 18.3%)	2.6 (2.2 to 3.1)
Hemorrhagic stroke	Venezuela	1995	All Ages	0.7 (0.5 to 0.8)	14.5% (11.8 to 17.3%)	2.9 (2.4 to 3.5)
Hemorrhagic stroke	Venezuela	2000	All Ages	0.8 (0.7 to 1.0)	14.9% (12.1 to 17.5%)	3.3 (2.7 to 3.9)
Hemorrhagic stroke	Venezuela	2005	All Ages	0.8 (0.7 to 1.0)	13.9% (11.1 to 16.6%)	3.1 (2.4 to 3.7)
Hemorrhagic stroke	Venezuela	2010	All Ages	0.8 (0.7 to 1.0)	12.5% (9.6 to 15.2%)	2.9 (2.3 to 3.5)
Hemorrhagic stroke	Venezuela	2015	All Ages	1.0 (0.8 to 1.3)	12.6% (9.8 to 15.5%)	3.3 (2.5 to 4.2)
Hemorrhagic stroke	Venezuela	1990	Age-standardized		14.4% (11.6 to 17.3%)	5.5 (4.4 to 6.7)
Hemorrhagic stroke	Venezuela	1995	Age-standardized		13.5% (10.7 to 16.3%)	6.0 (4.7 to 7.4)
Hemorrhagic stroke	Venezuela	2000	Age-standardized		13.8% (10.8 to 16.9%)	6.4 (5.0 to 7.8)
Hemorrhagic stroke	Venezuela	2005	Age-standardized		13.0% (10.0 to 16.1%)	5.4 (4.1 to 6.8)
Hemorrhagic stroke	Venezuela	2010	Age-standardized		11.7% (8.7 to 14.9%)	4.6 (3.5 to 5.8)

Cause name	Location	Year	Age group	Number (in thousands)	PAF	Rate (per 100,000)
Hemorrhagic stroke	Venezuela	2015	Age-standardized		11.9% (8.8 to 15.1%)	4.7 (3.4 to 6.1)
Chronic obstructive pulmonary disease	Venezuela	1990	All Ages	0.4 (0.2 to 0.5)	23.1% (12.6 to 34.1%)	1.8 (1.0 to 2.7)
Chronic obstructive pulmonary disease	Venezuela	1995	All Ages	0.5 (0.3 to 0.7)	22.0% (11.5 to 33.0%)	2.1 (1.1 to 3.2)
Chronic obstructive pulmonary disease	Venezuela	2000	All Ages	0.7 (0.4 to 1.0)	23.5% (12.9 to 34.4%)	2.8 (1.5 to 4.1)
Chronic obstructive pulmonary disease	Venezuela	2005	All Ages	0.8 (0.4 to 1.1)	22.0% (11.7 to 33.0%)	2.8 (1.5 to 4.3)
Chronic obstructive pulmonary disease	Venezuela	2010	All Ages	0.8 (0.4 to 1.2)	19.3% (9.3 to 30.2%)	2.7 (1.3 to 4.2)
Chronic obstructive pulmonary disease	Venezuela	2015	All Ages	1.0 (0.5 to 1.6)	19.8% (9.8 to 30.6%)	3.2 (1.6 to 5.1)
Chronic obstructive pulmonary disease	Venezuela	1990	Age-standardized		23.5% (12.8 to 34.6%)	5.3 (2.9 to 7.8)
Chronic obstructive pulmonary disease	Venezuela	1995	Age-standardized		22.2% (11.6 to 33.3%)	5.7 (3.0 to 8.7)
Chronic obstructive pulmonary disease	Venezuela	2000	Age-standardized		23.6% (13.0 to 34.6%)	6.8 (3.6 to 10.0)
Chronic obstructive pulmonary disease	Venezuela	2005	Age-standardized		22.1% (11.8 to 33.1%)	6.2 (3.3 to 9.3)
Chronic obstructive pulmonary disease	Venezuela	2010	Age-standardized		19.3% (9.4 to 30.3%)	5.1 (2.6 to 8.2)
Chronic obstructive pulmonary disease	Venezuela	2015	Age-standardized		19.8% (9.9 to 30.6%)	5.4 (2.7 to 8.5)
All causes	Bolivia	1990	All Ages	3.4 (2.6 to 4.4)	5.2% (4.0 to 6.7%)	49.5 (37.6 to 64.1)
All causes	Bolivia	1995	All Ages	3.4 (2.7 to 4.3)	5.4% (4.3 to 6.8%)	45.0 (35.0 to 56.6)
All causes	Bolivia	2000	All Ages	3.4 (2.7 to 4.1)	5.7% (4.6 to 6.9%)	40.2 (32.3 to 49.2)
All causes	Bolivia	2005	All Ages	4.0 (3.3 to 4.7)	6.8% (5.8 to 8.0%)	43.0 (35.8 to 50.7)
All causes	Bolivia	2010	All Ages	3.6 (2.9 to 4.5)	5.9% (4.8 to 7.0%)	36.5 (29.3 to 44.6)
All causes	Bolivia	2015	All Ages	4.1 (3.2 to 5.0)	6.0% (5.0 to 7.2%)	37.7 (29.6 to 46.4)
All causes	Bolivia	1990	Age-standardized		6.2% (5.1 to 7.6%)	82.0 (66.0 to 101.5)
All causes	Bolivia	1995	Age-standardized		6.4% (5.2 to 7.6%)	76.3 (61.6 to 92.6)
All causes	Bolivia	2000	Age-standardized		6.5% (5.5 to 7.8%)	68.7 (56.1 to 82.6)
All causes	Bolivia	2005	Age-standardized		7.6% (6.5 to 8.9%)	71.4 (59.5 to 84.1)
All causes	Bolivia	2010	Age-standardized		6.4% (5.3 to 7.6%)	57.1 (46.1 to 69.4)
All causes	Bolivia	2015	Age-standardized		6.4% (5.3 to 7.6%)	54.4 (42.7 to 66.8)
Lower respiratory infections	Bolivia	1990	All Ages	1.9 (1.1 to 2.9)	21.4% (13.2 to 31.1%)	27.8 (16.5 to 41.3)
Lower respiratory infections	Bolivia	1995	All Ages	1.8 (1.1 to 2.6)	21.9% (13.6 to 31.4%)	23.5 (14.1 to 34.6)
Lower respiratory infections	Bolivia	2000	All Ages	1.6 (1.0 to 2.4)	22.6% (14.5 to 32.0%)	19.5 (12.2 to 28.2)
Lower respiratory infections	Bolivia	2005	All Ages	1.8 (1.3 to 2.5)	27.0% (19.3 to 35.0%)	19.9 (14.0 to 26.9)
Lower respiratory infections	Bolivia	2010	All Ages	1.5 (0.9 to 2.1)	21.7% (13.8 to 31.1%)	14.7 (8.8 to 21.4)
Lower respiratory infections	Bolivia	2015	All Ages	1.5 (0.9 to 2.3)	21.5% (13.2 to 31.1%)	14.0 (8.2 to 20.9)
Lower respiratory infections	Bolivia	1990	Age-standardized		21.4% (13.2 to 31.1%)	34.0 (20.4 to 51.3)
Lower respiratory infections	Bolivia	1995	Age-standardized		21.9% (13.6 to 31.4%)	31.0 (18.7 to 45.4)
Lower respiratory infections	Bolivia	2000	Age-standardized		22.6% (14.5 to 32.0%)	27.6 (17.0 to 40.0)
Lower respiratory infections	Bolivia	2005	Age-standardized		27.1% (19.3 to 35.0%)	29.1 (20.1 to 39.4)
Lower respiratory infections	Bolivia	2010	Age-standardized		21.7% (13.8 to 31.1%)	21.3 (12.5 to 31.0)

Cause name	Location	Year	Age group	Number (in thousands)	PAF	Rate (per 100,000)
Lower respiratory infections	Bolivia	2015	Age-standardized		21.5% (13.2 to 31.1%)	19.3 (11.2 to 28.8)
Tracheal, bronchus, and lung cancer	Bolivia	1990	All Ages	0.1 (0.0 to 0.1)	15.1% (8.7 to 22.1%)	0.8 (0.5 to 1.2)
Tracheal, bronchus, and lung cancer	Bolivia	1995	All Ages	0.1 (0.0 to 0.1)	15.5% (9.2 to 22.5%)	0.9 (0.5 to 1.3)
Tracheal, bronchus, and lung cancer	Bolivia	2000	All Ages	0.1 (0.1 to 0.1)	16.1% (9.5 to 23.4%)	0.9 (0.5 to 1.4)
Tracheal, bronchus, and lung cancer	Bolivia	2005	All Ages	0.1 (0.1 to 0.2)	19.5% (12.3 to 27.3%)	1.3 (0.8 to 1.8)
Tracheal, bronchus, and lung cancer	Bolivia	2010	All Ages	0.1 (0.1 to 0.2)	15.4% (9.0 to 22.4%)	1.1 (0.7 to 1.7)
Tracheal, bronchus, and lung cancer	Bolivia	2015	All Ages	0.1 (0.1 to 0.2)	15.2% (8.7 to 22.3%)	1.3 (0.7 to 1.9)
Tracheal, bronchus, and lung cancer	Bolivia	1990	Age-standardized		15.2% (8.8 to 22.1%)	1.8 (1.0 to 2.7)
Tracheal, bronchus, and lung cancer	Bolivia	1995	Age-standardized		15.6% (9.2 to 22.6%)	1.8 (1.1 to 2.7)
Tracheal, bronchus, and lung cancer	Bolivia	2000	Age-standardized		16.1% (9.5 to 23.4%)	1.9 (1.1 to 2.7)
Tracheal, bronchus, and lung cancer	Bolivia	2005	Age-standardized		19.6% (12.3 to 27.3%)	2.3 (1.5 to 3.4)
Tracheal, bronchus, and lung cancer	Bolivia	2010	Age-standardized		15.4% (9.0 to 22.4%)	1.9 (1.1 to 2.8)
Tracheal, bronchus, and lung cancer	Bolivia	2015	Age-standardized		15.3% (8.7 to 22.3%)	1.9 (1.1 to 2.9)
Cardiovascular diseases	Bolivia	1990	All Ages	1.2 (1.0 to 1.4)	12.5% (10.8 to 14.1%)	17.4 (14.6 to 20.6)
Cardiovascular diseases	Bolivia	1995	All Ages	1.3 (1.1 to 1.5)	12.4% (10.8 to 14.1%)	17.2 (14.4 to 20.2)
Cardiovascular diseases	Bolivia	2000	All Ages	1.4 (1.2 to 1.6)	12.5% (10.7 to 14.3%)	16.3 (13.7 to 19.1)
Cardiovascular diseases	Bolivia	2005	All Ages	1.7 (1.4 to 2.0)	13.8% (11.8 to 15.8%)	18.2 (15.2 to 21.5)
Cardiovascular diseases	Bolivia	2010	All Ages	1.7 (1.4 to 2.1)	12.2% (10.3 to 14.2%)	17.4 (14.0 to 21.4)
Cardiovascular diseases	Bolivia	2015	All Ages	2.0 (1.6 to 2.5)	12.1% (10.1 to 14.3%)	18.9 (14.9 to 23.3)
Cardiovascular diseases	Bolivia	1990	Age-standardized		12.6% (10.7 to 14.5%)	37.6 (31.1 to 45.2)
Cardiovascular diseases	Bolivia	1995	Age-standardized		12.5% (10.6 to 14.6%)	35.4 (29.1 to 42.2)
Cardiovascular diseases	Bolivia	2000	Age-standardized		12.6% (10.6 to 14.6%)	32.0 (26.2 to 38.0)
Cardiovascular diseases	Bolivia	2005	Age-standardized		13.7% (11.6 to 15.9%)	32.8 (27.0 to 38.9)
Cardiovascular diseases	Bolivia	2010	Age-standardized		12.2% (10.2 to 14.3%)	28.4 (22.7 to 35.0)
Cardiovascular diseases	Bolivia	2015	Age-standardized		12.1% (10.0 to 14.3%)	27.8 (22.0 to 34.3)
Ischemic heart disease	Bolivia	1990	All Ages	0.8 (0.6 to 0.9)	17.5% (14.7 to 20.5%)	11.0 (9.0 to 13.5)
Ischemic heart disease	Bolivia	1995	All Ages	0.8 (0.7 to 1.0)	17.2% (14.3 to 20.3%)	10.8 (8.7 to 13.2)
Ischemic heart disease	Bolivia	2000	All Ages	0.9 (0.7 to 1.1)	17.1% (14.1 to 20.3%)	10.4 (8.3 to 12.7)
Ischemic heart disease	Bolivia	2005	All Ages	1.1 (0.8 to 1.3)	18.0% (14.8 to 21.7%)	11.5 (9.2 to 14.2)
Ischemic heart disease	Bolivia	2010	All Ages	1.1 (0.8 to 1.4)	16.0% (12.6 to 19.5%)	11.2 (8.6 to 14.2)
Ischemic heart disease	Bolivia	2015	All Ages	1.3 (1.0 to 1.7)	15.7% (12.0 to 19.3%)	12.3 (9.2 to 16.1)
Ischemic heart disease	Bolivia	1990	Age-standardized		16.3% (13.0 to 19.5%)	24.4 (19.2 to 30.2)
Ischemic heart disease	Bolivia	1995	Age-standardized		16.2% (12.9 to 19.6%)	22.8 (17.5 to 28.3)
Ischemic heart disease	Bolivia	2000	Age-standardized		16.3% (12.8 to 19.8%)	20.8 (16.0 to 25.7)
Ischemic heart disease	Bolivia	2005	Age-standardized		17.4% (13.9 to 21.4%)	21.1 (16.4 to 26.4)
Ischemic heart disease	Bolivia	2010	Age-standardized		15.7% (12.1 to 19.3%)	18.4 (14.0 to 23.7)

Cause name	Location	Year	Age group	Number (in thousands)	PAF	Rate (per 100,000)
Ischemic heart disease	Bolivia	2015	Age-standardized		15.5% (11.9 to 19.2%)	18.3 (13.6 to 23.9)
Ischemic stroke	Bolivia	1990	All Ages	0.2 (0.1 to 0.3)	13.2% (10.3 to 16.0%)	2.9 (2.2 to 3.7)
Ischemic stroke	Bolivia	1995	All Ages	0.2 (0.2 to 0.3)	13.1% (10.1 to 15.9%)	2.9 (2.2 to 3.7)
Ischemic stroke	Bolivia	2000	All Ages	0.2 (0.2 to 0.3)	13.0% (9.9 to 16.0%)	2.8 (2.1 to 3.6)
Ischemic stroke	Bolivia	2005	All Ages	0.3 (0.2 to 0.4)	14.1% (10.8 to 17.3%)	3.1 (2.3 to 3.9)
Ischemic stroke	Bolivia	2010	All Ages	0.3 (0.2 to 0.4)	12.1% (8.9 to 15.5%)	3.0 (2.0 to 4.0)
Ischemic stroke	Bolivia	2015	All Ages	0.4 (0.2 to 0.5)	11.8% (8.5 to 15.5%)	3.2 (2.2 to 4.5)
Ischemic stroke	Bolivia	1990	Age-standardized		12.3% (9.2 to 15.4%)	6.7 (4.8 to 8.8)
Ischemic stroke	Bolivia	1995	Age-standardized		12.4% (9.2 to 15.5%)	6.4 (4.6 to 8.3)
Ischemic stroke	Bolivia	2000	Age-standardized		12.5% (9.2 to 15.8%)	5.7 (4.1 to 7.4)
Ischemic stroke	Bolivia	2005	Age-standardized		13.7% (10.1 to 17.2%)	5.7 (4.2 to 7.5)
Ischemic stroke	Bolivia	2010	Age-standardized		11.9% (8.6 to 15.4%)	4.9 (3.4 to 6.7)
Ischemic stroke	Bolivia	2015	Age-standardized		11.8% (8.4 to 15.4%)	4.8 (3.3 to 6.7)
Hemorrhagic stroke	Bolivia	1990	All Ages	0.2 (0.2 to 0.3)	13.0% (10.5 to 15.4%)	3.5 (2.7 to 4.4)
Hemorrhagic stroke	Bolivia	1995	All Ages	0.3 (0.2 to 0.3)	13.3% (10.9 to 15.8%)	3.4 (2.7 to 4.3)
Hemorrhagic stroke	Bolivia	2000	All Ages	0.3 (0.2 to 0.3)	13.6% (11.2 to 16.2%)	3.1 (2.5 to 3.9)
Hemorrhagic stroke	Bolivia	2005	All Ages	0.3 (0.3 to 0.4)	15.6% (13.0 to 18.0%)	3.6 (2.9 to 4.5)
Hemorrhagic stroke	Bolivia	2010	All Ages	0.3 (0.3 to 0.4)	13.7% (11.1 to 16.4%)	3.3 (2.5 to 4.3)
Hemorrhagic stroke	Bolivia	2015	All Ages	0.4 (0.3 to 0.5)	13.6% (10.9 to 16.3%)	3.3 (2.4 to 4.4)
Hemorrhagic stroke	Bolivia	1990	Age-standardized		13.7% (11.1 to 16.4%)	6.6 (5.0 to 8.3)
Hemorrhagic stroke	Bolivia	1995	Age-standardized		13.8% (11.2 to 16.5%)	6.2 (4.8 to 7.9)
Hemorrhagic stroke	Bolivia	2000	Age-standardized		14.0% (11.3 to 16.8%)	5.5 (4.3 to 7.0)
Hemorrhagic stroke	Bolivia	2005	Age-standardized		15.5% (12.8 to 18.3%)	6.0 (4.8 to 7.6)
Hemorrhagic stroke	Bolivia	2010	Age-standardized		13.6% (10.9 to 16.4%)	5.1 (3.8 to 6.7)
Hemorrhagic stroke	Bolivia	2015	Age-standardized		13.5% (10.7 to 16.4%)	4.7 (3.5 to 6.3)
Chronic obstructive pulmonary disease	Bolivia	1990	All Ages	0.2 (0.1 to 0.4)	20.7% (10.9 to 31.0%)	3.5 (1.8 to 5.3)
Chronic obstructive pulmonary disease	Bolivia	1995	All Ages	0.3 (0.1 to 0.4)	21.3% (11.1 to 31.8%)	3.5 (1.8 to 5.4)
Chronic obstructive pulmonary disease	Bolivia	2000	All Ages	0.3 (0.1 to 0.4)	22.0% (11.6 to 32.7%)	3.4 (1.7 to 5.2)
Chronic obstructive pulmonary disease	Bolivia	2005	All Ages	0.3 (0.2 to 0.5)	25.5% (14.8 to 36.9%)	3.6 (2.1 to 5.6)
Chronic obstructive pulmonary disease	Bolivia	2010	All Ages	0.3 (0.2 to 0.5)	21.7% (11.2 to 32.5%)	3.3 (1.7 to 5.3)
Chronic obstructive pulmonary disease	Bolivia	2015	All Ages	0.4 (0.2 to 0.6)	21.7% (11.3 to 32.5%)	3.6 (1.8 to 5.7)
Chronic obstructive pulmonary disease	Bolivia	1990	Age-standardized		21.5% (11.3 to 32.3%)	8.6 (4.4 to 13.2)
Chronic obstructive pulmonary disease	Bolivia	1995	Age-standardized		21.9% (11.5 to 32.8%)	8.1 (4.1 to 12.5)
Chronic obstructive pulmonary disease	Bolivia	2000	Age-standardized		22.5% (11.9 to 33.4%)	7.3 (3.7 to 11.4)
Chronic obstructive pulmonary disease	Bolivia	2005	Age-standardized		25.9% (15.0 to 37.4%)	7.1 (4.0 to 10.8)
Chronic obstructive pulmonary disease	Bolivia	2010	Age-standardized		21.9% (11.3 to 32.7%)	5.6 (2.9 to 9.0)

Cause name	Location	Year	Age group	Number (in thousands)	PAF	Rate (per 100,000)
Chronic obstructive pulmonary disease	Bolivia	2015	Age-standardized		21.8% (11.3 to 32.6%)	5.3 (2.8 to 8.5)
All causes	Ecuador	1990	All Ages	2.3 (1.7 to 2.9)	4.1% (3.2 to 5.3%)	22.0 (16.9 to 28.4)
All causes	Ecuador	1995	All Ages	2.5 (2.0 to 3.2)	4.3% (3.4 to 5.4%)	22.0 (17.2 to 27.8)
All causes	Ecuador	2000	All Ages	2.9 (2.3 to 3.7)	4.5% (3.6 to 5.6%)	23.2 (18.4 to 29.0)
All causes	Ecuador	2005	All Ages	3.4 (2.7 to 4.2)	4.7% (3.8 to 5.7%)	24.7 (19.8 to 30.4)
All causes	Ecuador	2010	All Ages	2.9 (2.2 to 3.7)	3.7% (2.9 to 4.7%)	19.5 (15.0 to 24.9)
All causes	Ecuador	2015	All Ages	3.0 (2.3 to 3.9)	3.5% (2.7 to 4.5%)	18.7 (14.3 to 23.9)
All causes	Ecuador	1990	Age-standardized		4.9% (3.9 to 6.0%)	42.1 (33.6 to 52.0)
All causes	Ecuador	1995	Age-standardized		4.9% (3.9 to 6.0%)	40.2 (32.3 to 49.9)
All causes	Ecuador	2000	Age-standardized		4.9% (3.9 to 6.0%)	40.3 (32.2 to 49.5)
All causes	Ecuador	2005	Age-standardized		5.0% (4.0 to 6.1%)	39.6 (31.8 to 48.5)
All causes	Ecuador	2010	Age-standardized		3.9% (3.0 to 5.0%)	29.5 (22.6 to 37.3)
All causes	Ecuador	2015	Age-standardized		3.6% (2.8 to 4.6%)	26.0 (19.8 to 33.3)
Lower respiratory infections	Ecuador	1990	All Ages	0.8 (0.4 to 1.4)	14.9% (6.6 to 25.3%)	8.2 (3.7 to 14.1)
Lower respiratory infections	Ecuador	1995	All Ages	0.9 (0.4 to 1.5)	15.2% (6.9 to 25.5%)	7.6 (3.4 to 12.9)
Lower respiratory infections	Ecuador	2000	All Ages	0.9 (0.4 to 1.5)	15.2% (6.9 to 25.7%)	7.1 (3.2 to 12.0)
Lower respiratory infections	Ecuador	2005	All Ages	1.0 (0.5 to 1.7)	16.3% (7.8 to 26.6%)	7.4 (3.5 to 12.2)
Lower respiratory infections	Ecuador	2010	All Ages	0.8 (0.3 to 1.5)	12.4% (4.4 to 23.2%)	5.2 (1.8 to 10.0)
Lower respiratory infections	Ecuador	2015	All Ages	0.7 (0.2 to 1.5)	11.2% (3.6 to 22.2%)	4.6 (1.4 to 9.3)
Lower respiratory infections	Ecuador	1990	Age-standardized		14.9% (6.6 to 25.3%)	11.4 (5.1 to 19.7)
Lower respiratory infections	Ecuador	1995	Age-standardized		15.2% (6.9 to 25.5%)	10.7 (4.8 to 18.1)
Lower respiratory infections	Ecuador	2000	Age-standardized		15.2% (6.9 to 25.7%)	10.1 (4.5 to 17.3)
Lower respiratory infections	Ecuador	2005	Age-standardized		16.3% (7.8 to 26.6%)	10.5 (5.0 to 17.2)
Lower respiratory infections	Ecuador	2010	Age-standardized		12.4% (4.4 to 23.2%)	7.3 (2.6 to 14.1)
Lower respiratory infections	Ecuador	2015	Age-standardized		11.2% (3.6 to 22.2%)	6.1 (1.9 to 12.5)
Tracheal, bronchus, and lung cancer	Ecuador	1990	All Ages	0.0 (0.0 to 0.1)	10.3% (5.4 to 16.2%)	0.4 (0.2 to 0.7)
Tracheal, bronchus, and lung cancer	Ecuador	1995	All Ages	0.1 (0.0 to 0.1)	10.5% (5.6 to 16.6%)	0.5 (0.3 to 0.8)
Tracheal, bronchus, and lung cancer	Ecuador	2000	All Ages	0.1 (0.0 to 0.1)	10.6% (5.6 to 16.5%)	0.6 (0.3 to 1.0)
Tracheal, bronchus, and lung cancer	Ecuador	2005	All Ages	0.1 (0.1 to 0.2)	11.3% (6.1 to 17.5%)	0.8 (0.4 to 1.2)
Tracheal, bronchus, and lung cancer	Ecuador	2010	All Ages	0.1 (0.1 to 0.2)	8.5% (4.2 to 13.9%)	0.6 (0.3 to 1.1)
Tracheal, bronchus, and lung cancer	Ecuador	2015	All Ages	0.1 (0.1 to 0.2)	7.6% (3.7 to 12.8%)	0.6 (0.3 to 1.1)
Tracheal, bronchus, and lung cancer	Ecuador	1990	Age-standardized		10.4% (5.5 to 16.3%)	0.9 (0.5 to 1.5)
Tracheal, bronchus, and lung cancer	Ecuador	1995	Age-standardized		10.6% (5.6 to 16.6%)	1.1 (0.6 to 1.7)
Tracheal, bronchus, and lung cancer	Ecuador	2000	Age-standardized		10.6% (5.6 to 16.6%)	1.2 (0.6 to 1.8)
Tracheal, bronchus, and lung cancer	Ecuador	2005	Age-standardized		11.4% (6.1 to 17.5%)	1.3 (0.7 to 2.1)
Tracheal, bronchus, and lung cancer	Ecuador	2010	Age-standardized		8.5% (4.2 to 13.9%)	1.0 (0.5 to 1.6)

Cause name	Location	Year	Age group	Number (in thousands)	PAF	Rate (per 100,000)
Tracheal, bronchus, and lung cancer	Ecuador	2015	Age-standardized		7.7% (3.7 to 12.8%)	0.9 (0.4 to 1.5)
Cardiovascular diseases	Ecuador	1990	All Ages	1.2 (1.0 to 1.4)	10.8% (9.1 to 12.5%)	11.4 (9.6 to 13.3)
Cardiovascular diseases	Ecuador	1995	All Ages	1.4 (1.1 to 1.6)	10.8% (9.0 to 12.6%)	11.9 (10.0 to 13.9)
Cardiovascular diseases	Ecuador	2000	All Ages	1.7 (1.4 to 2.0)	10.9% (9.0 to 12.8%)	13.1 (10.7 to 15.6)
Cardiovascular diseases	Ecuador	2005	All Ages	1.9 (1.5 to 2.3)	10.8% (8.9 to 12.8%)	13.8 (11.2 to 16.4)
Cardiovascular diseases	Ecuador	2010	All Ages	1.7 (1.4 to 2.1)	9.1% (7.4 to 10.9%)	11.4 (9.1 to 13.8)
Cardiovascular diseases	Ecuador	2015	All Ages	1.8 (1.4 to 2.3)	8.6% (6.9 to 10.4%)	11.4 (8.8 to 14.0)
Cardiovascular diseases	Ecuador	1990	Age-standardized		10.4% (8.4 to 12.3%)	24.7 (20.1 to 29.6)
Cardiovascular diseases	Ecuador	1995	Age-standardized		10.4% (8.5 to 12.4%)	23.9 (19.4 to 28.7)
Cardiovascular diseases	Ecuador	2000	Age-standardized		10.6% (8.5 to 12.7%)	24.2 (19.3 to 29.2)
Cardiovascular diseases	Ecuador	2005	Age-standardized		10.6% (8.5 to 12.7%)	23.0 (18.3 to 27.5)
Cardiovascular diseases	Ecuador	2010	Age-standardized		8.9% (7.1 to 10.8%)	17.5 (13.7 to 21.3)
Cardiovascular diseases	Ecuador	2015	Age-standardized		8.4% (6.6 to 10.3%)	15.8 (12.1 to 19.7)
Ischemic heart disease	Ecuador	1990	All Ages	0.8 (0.6 to 1.0)	14.3% (11.4 to 17.2%)	7.7 (6.1 to 9.3)
Ischemic heart disease	Ecuador	1995	All Ages	0.9 (0.7 to 1.1)	14.3% (11.3 to 17.2%)	8.2 (6.4 to 9.9)
Ischemic heart disease	Ecuador	2000	All Ages	1.2 (0.9 to 1.5)	14.1% (10.9 to 17.1%)	9.4 (7.3 to 11.7)
Ischemic heart disease	Ecuador	2005	All Ages	1.4 (1.0 to 1.7)	14.3% (10.9 to 17.6%)	9.9 (7.6 to 12.1)
Ischemic heart disease	Ecuador	2010	All Ages	1.2 (0.9 to 1.5)	12.8% (9.5 to 15.8%)	8.1 (6.0 to 10.1)
Ischemic heart disease	Ecuador	2015	All Ages	1.3 (1.0 to 1.7)	12.1% (8.9 to 15.2%)	8.1 (5.9 to 10.3)
Ischemic heart disease	Ecuador	1990	Age-standardized		13.5% (10.2 to 16.7%)	17.1 (12.9 to 21.3)
Ischemic heart disease	Ecuador	1995	Age-standardized		13.6% (10.3 to 16.8%)	16.7 (12.6 to 20.9)
Ischemic heart disease	Ecuador	2000	Age-standardized		13.5% (10.2 to 16.9%)	17.5 (13.2 to 22.1)
Ischemic heart disease	Ecuador	2005	Age-standardized		13.9% (10.4 to 17.3%)	16.5 (12.3 to 20.5)
Ischemic heart disease	Ecuador	2010	Age-standardized		12.4% (9.1 to 15.6%)	12.4 (9.1 to 15.8)
Ischemic heart disease	Ecuador	2015	Age-standardized		11.8% (8.6 to 15.0%)	11.3 (8.1 to 14.7)
Ischemic stroke	Ecuador	1990	All Ages	0.2 (0.1 to 0.2)	10.3% (7.6 to 13.2%)	1.8 (1.3 to 2.4)
Ischemic stroke	Ecuador	1995	All Ages	0.2 (0.1 to 0.3)	10.2% (7.2 to 13.2%)	1.8 (1.3 to 2.3)
Ischemic stroke	Ecuador	2000	All Ages	0.2 (0.1 to 0.3)	9.9% (6.8 to 13.4%)	1.7 (1.2 to 2.3)
Ischemic stroke	Ecuador	2005	All Ages	0.3 (0.2 to 0.4)	10.1% (6.9 to 13.9%)	1.9 (1.3 to 2.6)
Ischemic stroke	Ecuador	2010	All Ages	0.2 (0.2 to 0.4)	8.7% (5.6 to 12.4%)	1.6 (1.0 to 2.3)
Ischemic stroke	Ecuador	2015	All Ages	0.3 (0.2 to 0.4)	8.2% (5.1 to 11.9%)	1.6 (1.0 to 2.4)
Ischemic stroke	Ecuador	1990	Age-standardized		9.9% (6.9 to 13.1%)	4.2 (2.9 to 5.6)
Ischemic stroke	Ecuador	1995	Age-standardized		9.8% (6.7 to 13.2%)	3.8 (2.6 to 5.1)
Ischemic stroke	Ecuador	2000	Age-standardized		9.7% (6.5 to 13.3%)	3.3 (2.2 to 4.6)
Ischemic stroke	Ecuador	2005	Age-standardized		10.0% (6.7 to 13.8%)	3.3 (2.1 to 4.6)
Ischemic stroke	Ecuador	2010	Age-standardized		8.6% (5.3 to 12.4%)	2.6 (1.6 to 3.8)

Cause name	Location	Year	Age group	Number (in thousands)	PAF	Rate (per 100,000)
Ischemic stroke	Ecuador	2015	Age-standardized		8.1% (4.8 to 12.0%)	2.3 (1.4 to 3.4)
Hemorrhagic stroke	Ecuador	1990	All Ages	0.2 (0.1 to 0.2)	11.8% (9.2 to 14.3%)	1.8 (1.4 to 2.3)
Hemorrhagic stroke	Ecuador	1995	All Ages	0.2 (0.2 to 0.3)	11.7% (9.2 to 14.2%)	1.9 (1.5 to 2.3)
Hemorrhagic stroke	Ecuador	2000	All Ages	0.3 (0.2 to 0.3)	11.5% (9.0 to 14.1%)	2.0 (1.5 to 2.4)
Hemorrhagic stroke	Ecuador	2005	All Ages	0.3 (0.2 to 0.4)	11.8% (9.2 to 14.4%)	2.1 (1.6 to 2.6)
Hemorrhagic stroke	Ecuador	2010	All Ages	0.3 (0.2 to 0.3)	10.1% (7.5 to 12.8%)	1.7 (1.3 to 2.2)
Hemorrhagic stroke	Ecuador	2015	All Ages	0.3 (0.2 to 0.4)	9.5% (6.9 to 12.2%)	1.7 (1.2 to 2.2)
Hemorrhagic stroke	Ecuador	1990	Age-standardized		11.6% (8.8 to 14.3%)	3.4 (2.6 to 4.3)
Hemorrhagic stroke	Ecuador	1995	Age-standardized		11.5% (8.8 to 14.1%)	3.4 (2.5 to 4.2)
Hemorrhagic stroke	Ecuador	2000	Age-standardized		11.2% (8.5 to 13.9%)	3.3 (2.5 to 4.1)
Hemorrhagic stroke	Ecuador	2005	Age-standardized		11.5% (8.8 to 14.1%)	3.2 (2.4 to 4.0)
Hemorrhagic stroke	Ecuador	2010	Age-standardized		9.8% (7.2 to 12.5%)	2.5 (1.8 to 3.3)
Hemorrhagic stroke	Ecuador	2015	Age-standardized		9.2% (6.7 to 12.0%)	2.2 (1.5 to 2.9)
Chronic obstructive pulmonary disease	Ecuador	1990	All Ages	0.2 (0.1 to 0.3)	11.9% (5.2 to 19.6%)	2.0 (0.9 to 3.3)
Chronic obstructive pulmonary disease	Ecuador	1995	All Ages	0.2 (0.1 to 0.4)	14.4% (6.7 to 23.6%)	2.0 (0.9 to 3.2)
Chronic obstructive pulmonary disease	Ecuador	2000	All Ages	0.3 (0.1 to 0.5)	16.4% (7.5 to 26.7%)	2.4 (1.1 to 3.9)
Chronic obstructive pulmonary disease	Ecuador	2005	All Ages	0.4 (0.2 to 0.6)	17.4% (8.0 to 28.0%)	2.7 (1.3 to 4.3)
Chronic obstructive pulmonary disease	Ecuador	2010	All Ages	0.3 (0.1 to 0.6)	14.2% (5.9 to 24.2%)	2.2 (0.9 to 3.8)
Chronic obstructive pulmonary disease	Ecuador	2015	All Ages	0.3 (0.1 to 0.6)	13.2% (5.1 to 23.2%)	2.1 (0.8 to 3.7)
Chronic obstructive pulmonary disease	Ecuador	1990	Age-standardized		15.1% (6.8 to 24.8%)	5.1 (2.3 to 8.3)
Chronic obstructive pulmonary disease	Ecuador	1995	Age-standardized		16.0% (7.3 to 26.2%)	4.6 (2.0 to 7.4)
Chronic obstructive pulmonary disease	Ecuador	2000	Age-standardized		16.7% (7.6 to 27.3%)	4.8 (2.2 to 7.8)
Chronic obstructive pulmonary disease	Ecuador	2005	Age-standardized		17.6% (8.1 to 28.2%)	4.9 (2.2 to 7.8)
Chronic obstructive pulmonary disease	Ecuador	2010	Age-standardized		14.3% (5.9 to 24.4%)	3.6 (1.5 to 6.2)
Chronic obstructive pulmonary disease	Ecuador	2015	Age-standardized		13.3% (5.1 to 23.3%)	3.1 (1.2 to 5.5)
All causes	Peru	1990	All Ages	8.3 (6.4 to 10.4)	6.2% (4.8 to 7.8%)	37.8 (29.1 to 47.6)
All causes	Peru	1995	All Ages	8.3 (6.8 to 10.1)	6.5% (5.3 to 7.9%)	34.5 (28.2 to 41.9)
All causes	Peru	2000	All Ages	7.7 (6.4 to 9.2)	6.8% (5.6 to 8.0%)	29.8 (24.6 to 35.4)
All causes	Peru	2005	All Ages	8.6 (7.2 to 10.2)	7.9% (6.5 to 9.3%)	31.2 (25.9 to 36.7)
All causes	Peru	2010	All Ages	7.7 (6.0 to 9.7)	6.6% (5.2 to 8.2%)	26.2 (20.3 to 32.9)
All causes	Peru	2015	All Ages	8.4 (6.6 to 10.7)	6.7% (5.3 to 8.4%)	26.8 (21.0 to 34.1)
All causes	Peru	1990	Age-standardized		7.0% (5.8 to 8.5%)	65.9 (53.8 to 79.6)
All causes	Peru	1995	Age-standardized		7.4% (6.1 to 8.8%)	63.9 (53.2 to 76.2)
All causes	Peru	2000	Age-standardized		7.7% (6.3 to 9.1%)	55.8 (46.0 to 66.1)
All causes	Peru	2005	Age-standardized		8.7% (7.2 to 10.3%)	53.5 (44.1 to 63.1)
All causes	Peru	2010	Age-standardized		7.0% (5.6 to 8.8%)	39.9 (31.0 to 50.3)

Cause name	Location	Year	Age group	Number (in thousands)	PAF	Rate (per 100,000)
All causes	Peru	2015	Age-standardized		7.0% (5.5 to 8.8%)	37.5 (29.3 to 47.9)
Lower respiratory infections	Peru	1990	All Ages	4.8 (2.9 to 6.9)	21.2% (13.0 to 30.6%)	21.8 (13.4 to 31.7)
Lower respiratory infections	Peru	1995	All Ages	4.2 (2.8 to 6.0)	23.3% (15.6 to 32.4%)	17.6 (11.5 to 24.8)
Lower respiratory infections	Peru	2000	All Ages	3.9 (2.7 to 5.3)	25.0% (17.4 to 33.2%)	15.1 (10.5 to 20.4)
Lower respiratory infections	Peru	2005	All Ages	4.5 (3.1 to 5.9)	27.5% (19.9 to 35.3%)	16.1 (11.1 to 21.2)
Lower respiratory infections	Peru	2010	All Ages	3.8 (2.3 to 5.6)	21.3% (13.1 to 30.8%)	13.0 (7.8 to 19.0)
Lower respiratory infections	Peru	2015	All Ages	4.1 (2.4 to 6.1)	21.2% (13.0 to 30.7%)	13.1 (7.7 to 19.4)
Lower respiratory infections	Peru	1990	Age-standardized		21.2% (13.0 to 30.6%)	27.7 (16.7 to 39.9)
Lower respiratory infections	Peru	1995	Age-standardized		23.3% (15.6 to 32.4%)	26.6 (17.3 to 37.8)
Lower respiratory infections	Peru	2000	Age-standardized		25.0% (17.4 to 33.2%)	26.4 (17.9 to 35.7)
Lower respiratory infections	Peru	2005	Age-standardized		27.5% (19.9 to 35.3%)	27.1 (18.4 to 36.1)
Lower respiratory infections	Peru	2010	Age-standardized		21.3% (13.1 to 30.8%)	19.7 (11.7 to 29.1)
Lower respiratory infections	Peru	2015	Age-standardized		21.2% (13.0 to 30.7%)	18.3 (10.8 to 27.3)
Tracheal, bronchus, and lung cancer	Peru	1990	All Ages	0.2 (0.1 to 0.4)	14.9% (8.6 to 21.9%)	1.1 (0.6 to 1.6)
Tracheal, bronchus, and lung cancer	Peru	1995	All Ages	0.3 (0.2 to 0.5)	16.5% (10.0 to 23.7%)	1.3 (0.8 to 1.9)
Tracheal, bronchus, and lung cancer	Peru	2000	All Ages	0.3 (0.2 to 0.4)	17.9% (10.9 to 25.5%)	1.2 (0.7 to 1.7)
Tracheal, bronchus, and lung cancer	Peru	2005	All Ages	0.3 (0.2 to 0.5)	19.9% (12.4 to 27.6%)	1.2 (0.7 to 1.6)
Tracheal, bronchus, and lung cancer	Peru	2010	All Ages	0.3 (0.2 to 0.5)	15.0% (8.7 to 21.9%)	1.0 (0.6 to 1.5)
Tracheal, bronchus, and lung cancer	Peru	2015	All Ages	0.4 (0.2 to 0.5)	15.0% (8.7 to 22.0%)	1.1 (0.6 to 1.7)
Tracheal, bronchus, and lung cancer	Peru	1990	Age-standardized		15.0% (8.7 to 22.0%)	2.5 (1.5 to 3.7)
Tracheal, bronchus, and lung cancer	Peru	1995	Age-standardized		16.6% (10.1 to 23.8%)	2.8 (1.7 to 4.1)
Tracheal, bronchus, and lung cancer	Peru	2000	Age-standardized		18.0% (10.9 to 25.6%)	2.3 (1.4 to 3.3)
Tracheal, bronchus, and lung cancer	Peru	2005	Age-standardized		19.9% (12.5 to 27.7%)	2.0 (1.3 to 2.8)
Tracheal, bronchus, and lung cancer	Peru	2010	Age-standardized		15.0% (8.7 to 22.0%)	1.6 (0.9 to 2.3)
Tracheal, bronchus, and lung cancer	Peru	2015	Age-standardized		15.0% (8.8 to 22.0%)	1.6 (0.9 to 2.4)
Cardiovascular diseases	Peru	1990	All Ages	2.9 (2.5 to 3.4)	12.9% (11.1 to 14.7%)	13.4 (11.5 to 15.4)
Cardiovascular diseases	Peru	1995	All Ages	3.3 (2.8 to 3.9)	13.7% (11.7 to 15.7%)	13.8 (11.6 to 16.0)
Cardiovascular diseases	Peru	2000	All Ages	3.0 (2.5 to 3.5)	14.0% (12.0 to 16.3%)	11.4 (9.6 to 13.3)
Cardiovascular diseases	Peru	2005	All Ages	3.2 (2.7 to 3.7)	14.6% (12.5 to 17.0%)	11.4 (9.8 to 13.5)
Cardiovascular diseases	Peru	2010	All Ages	2.9 (2.4 to 3.5)	12.6% (10.5 to 14.7%)	9.8 (8.1 to 11.8)
Cardiovascular diseases	Peru	2015	All Ages	3.2 (2.5 to 3.9)	12.3% (10.1 to 14.7%)	10.1 (8.0 to 12.4)
Cardiovascular diseases	Peru	1990	Age-standardized		12.9% (10.6 to 15.2%)	31.9 (26.2 to 38.0)
Cardiovascular diseases	Peru	1995	Age-standardized		13.4% (11.1 to 15.9%)	30.1 (24.7 to 35.5)
Cardiovascular diseases	Peru	2000	Age-standardized		13.6% (11.3 to 16.2%)	22.6 (18.5 to 27.1)
Cardiovascular diseases	Peru	2005	Age-standardized		14.1% (11.8 to 16.9%)	19.6 (16.2 to 23.7)
Cardiovascular diseases	Peru	2010	Age-standardized		12.3% (10.1 to 14.6%)	14.8 (11.9 to 18.0)

Cause name	Location	Year	Age group	Number (in thousands)	PAF	Rate (per 100,000)
Cardiovascular diseases	Peru	2015	Age-standardized		12.1% (9.7 to 14.5%)	14.0 (11.0 to 17.2)
Ischemic heart disease	Peru	1990	All Ages	2.0 (1.7 to 2.4)	16.6% (13.7 to 19.7%)	9.3 (7.6 to 11.1)
Ischemic heart disease	Peru	1995	All Ages	2.3 (1.8 to 2.7)	17.1% (13.9 to 20.5%)	9.4 (7.5 to 11.3)
Ischemic heart disease	Peru	2000	All Ages	2.0 (1.6 to 2.5)	17.4% (14.1 to 21.0%)	7.8 (6.2 to 9.5)
Ischemic heart disease	Peru	2005	All Ages	2.3 (1.8 to 2.8)	18.2% (14.9 to 22.1%)	8.2 (6.6 to 10.0)
Ischemic heart disease	Peru	2010	All Ages	2.1 (1.6 to 2.6)	15.9% (12.5 to 19.4%)	7.0 (5.4 to 8.7)
Ischemic heart disease	Peru	2015	All Ages	2.3 (1.7 to 2.9)	15.4% (11.8 to 19.2%)	7.2 (5.4 to 9.3)
Ischemic heart disease	Peru	1990	Age-standardized		15.4% (11.9 to 19.0%)	22.6 (17.3 to 28.2)
Ischemic heart disease	Peru	1995	Age-standardized		16.0% (12.3 to 19.8%)	20.8 (15.9 to 25.9)
Ischemic heart disease	Peru	2000	Age-standardized		16.4% (12.6 to 20.5%)	15.8 (11.9 to 19.7)
Ischemic heart disease	Peru	2005	Age-standardized		17.3% (13.5 to 21.5%)	14.2 (11.0 to 18.0)
Ischemic heart disease	Peru	2010	Age-standardized		15.3% (11.6 to 19.0%)	10.7 (8.0 to 13.6)
Ischemic heart disease	Peru	2015	Age-standardized		15.0% (11.2 to 19.0%)	10.1 (7.4 to 13.1)
Ischemic stroke	Peru	1990	All Ages	0.5 (0.4 to 0.6)	12.4% (9.5 to 15.3%)	2.2 (1.6 to 2.7)
Ischemic stroke	Peru	1995	All Ages	0.6 (0.4 to 0.7)	13.0% (9.9 to 16.1%)	2.3 (1.7 to 2.9)
Ischemic stroke	Peru	2000	All Ages	0.4 (0.3 to 0.6)	13.2% (10.0 to 16.5%)	1.7 (1.3 to 2.1)
Ischemic stroke	Peru	2005	All Ages	0.4 (0.3 to 0.5)	13.8% (10.5 to 17.3%)	1.4 (1.1 to 1.8)
Ischemic stroke	Peru	2010	All Ages	0.4 (0.3 to 0.5)	11.7% (8.4 to 15.2%)	1.2 (0.9 to 1.7)
Ischemic stroke	Peru	2015	All Ages	0.4 (0.3 to 0.6)	11.4% (8.1 to 15.2%)	1.3 (0.9 to 1.8)
Ischemic stroke	Peru	1990	Age-standardized		11.7% (8.4 to 15.0%)	5.6 (4.0 to 7.2)
Ischemic stroke	Peru	1995	Age-standardized		12.4% (9.0 to 15.8%)	5.4 (3.9 to 7.1)
Ischemic stroke	Peru	2000	Age-standardized		12.7% (9.2 to 16.4%)	3.6 (2.6 to 4.7)
Ischemic stroke	Peru	2005	Age-standardized		13.4% (9.7 to 17.2%)	2.6 (1.9 to 3.4)
Ischemic stroke	Peru	2010	Age-standardized		11.4% (8.0 to 15.2%)	2.0 (1.4 to 2.7)
Ischemic stroke	Peru	2015	Age-standardized		11.3% (7.8 to 15.2%)	1.9 (1.3 to 2.6)
Hemorrhagic stroke	Peru	1990	All Ages	0.4 (0.3 to 0.5)	9.7% (7.7 to 11.7%)	1.9 (1.5 to 2.3)
Hemorrhagic stroke	Peru	1995	All Ages	0.5 (0.4 to 0.6)	12.0% (9.9 to 14.2%)	2.1 (1.7 to 2.5)
Hemorrhagic stroke	Peru	2000	All Ages	0.5 (0.4 to 0.6)	14.2% (11.8 to 16.5%)	1.9 (1.5 to 2.3)
Hemorrhagic stroke	Peru	2005	All Ages	0.5 (0.4 to 0.6)	15.4% (12.8 to 17.8%)	1.8 (1.5 to 2.2)
Hemorrhagic stroke	Peru	2010	All Ages	0.5 (0.4 to 0.6)	13.3% (10.8 to 15.8%)	1.6 (1.3 to 2.0)
Hemorrhagic stroke	Peru	2015	All Ages	0.5 (0.4 to 0.6)	13.2% (10.6 to 15.8%)	1.5 (1.2 to 1.9)
Hemorrhagic stroke	Peru	1990	Age-standardized		11.9% (9.5 to 14.4%)	3.7 (2.9 to 4.6)
Hemorrhagic stroke	Peru	1995	Age-standardized		13.2% (10.7 to 15.8%)	3.8 (3.1 to 4.6)
Hemorrhagic stroke	Peru	2000	Age-standardized		14.3% (11.5 to 16.8%)	3.2 (2.5 to 3.9)
Hemorrhagic stroke	Peru	2005	Age-standardized		15.2% (12.5 to 17.9%)	2.8 (2.3 to 3.4)
Hemorrhagic stroke	Peru	2010	Age-standardized		13.1% (10.4 to 15.7%)	2.2 (1.7 to 2.7)

Cause name	Location	Year	Age group	Number (in thousands)	PAF	Rate (per 100,000)
Hemorrhagic stroke	Peru	2015	Age-standardized		12.9% (10.2 to 15.6%)	2.0 (1.5 to 2.5)
Chronic obstructive pulmonary disease	Peru	1990	All Ages	0.3 (0.2 to 0.5)	14.7% (7.7 to 22.6%)	1.5 (0.8 to 2.2)
Chronic obstructive pulmonary disease	Peru	1995	All Ages	0.4 (0.2 to 0.7)	19.7% (10.5 to 28.9%)	1.8 (1.0 to 2.7)
Chronic obstructive pulmonary disease	Peru	2000	All Ages	0.5 (0.3 to 0.8)	23.4% (13.5 to 33.9%)	2.1 (1.1 to 3.0)
Chronic obstructive pulmonary disease	Peru	2005	All Ages	0.7 (0.4 to 1.0)	25.7% (15.0 to 37.1%)	2.5 (1.4 to 3.7)
Chronic obstructive pulmonary disease	Peru	2010	All Ages	0.7 (0.4 to 1.1)	21.2% (10.9 to 31.9%)	2.4 (1.2 to 3.7)
Chronic obstructive pulmonary disease	Peru	2015	All Ages	0.8 (0.4 to 1.2)	21.3% (11.2 to 32.2%)	2.5 (1.3 to 3.9)
Chronic obstructive pulmonary disease	Peru	1990	Age-standardized		19.3% (10.1 to 29.1%)	3.9 (2.0 to 5.9)
Chronic obstructive pulmonary disease	Peru	1995	Age-standardized		22.0% (11.7 to 32.5%)	4.4 (2.4 to 6.6)
Chronic obstructive pulmonary disease	Peru	2000	Age-standardized		24.1% (14.0 to 35.0%)	4.5 (2.5 to 6.6)
Chronic obstructive pulmonary disease	Peru	2005	Age-standardized		26.2% (15.3 to 37.7%)	4.8 (2.7 to 7.0)
Chronic obstructive pulmonary disease	Peru	2010	Age-standardized		21.4% (11.1 to 32.2%)	3.9 (2.0 to 6.0)
Chronic obstructive pulmonary disease	Peru	2015	Age-standardized		21.4% (11.3 to 32.4%)	3.6 (1.9 to 5.7)
All causes	Antigua and Barbuda	1990	All Ages	0.0 (0.0 to 0.0)	4.9% (3.2 to 6.8%)	32.6 (21.2 to 45.1)
All causes	Antigua and Barbuda	1995	All Ages	0.0 (0.0 to 0.0)	4.8% (3.1 to 6.5%)	32.9 (21.3 to 45.2)
All causes	Antigua and Barbuda	2000	All Ages	0.0 (0.0 to 0.0)	4.4% (2.8 to 5.9%)	28.5 (18.1 to 39.2)
All causes	Antigua and Barbuda	2005	All Ages	0.0 (0.0 to 0.0)	4.3% (2.7 to 5.8%)	27.0 (17.2 to 37.4)
All causes	Antigua and Barbuda	2010	All Ages	0.0 (0.0 to 0.0)	3.7% (2.2 to 5.3%)	22.1 (13.1 to 31.6)
All causes	Antigua and Barbuda	2015	All Ages	0.0 (0.0 to 0.0)	3.8% (2.4 to 5.3%)	22.7 (13.8 to 32.4)
All causes	Antigua and Barbuda	1990	Age-standardized		5.1% (3.4 to 7.0%)	42.1 (27.6 to 58.3)
All causes	Antigua and Barbuda	1995	Age-standardized		4.9% (3.2 to 6.7%)	40.5 (26.2 to 55.6)
All causes	Antigua and Barbuda	2000	Age-standardized		4.5% (2.9 to 6.1%)	37.5 (24.2 to 51.0)
All causes	Antigua and Barbuda	2005	Age-standardized		4.4% (2.8 to 5.9%)	34.2 (22.0 to 46.8)
All causes	Antigua and Barbuda	2010	Age-standardized		3.8% (2.3 to 5.4%)	28.4 (16.7 to 40.5)
All causes	Antigua and Barbuda	2015	Age-standardized		3.9% (2.4 to 5.4%)	28.3 (17.2 to 40.5)
Lower respiratory infections	Antigua and Barbuda	1990	All Ages	0.0 (0.0 to 0.0)	14.3% (3.9 to 27.1%)	5.4 (1.4 to 10.1)
Lower respiratory infections	Antigua and Barbuda	1995	All Ages	0.0 (0.0 to 0.0)	14.3% (3.7 to 27.2%)	5.6 (1.4 to 10.6)
Lower respiratory infections	Antigua and Barbuda	2000	All Ages	0.0 (0.0 to 0.0)	14.4% (3.9 to 27.2%)	5.3 (1.4 to 10.2)
Lower respiratory infections	Antigua and Barbuda	2005	All Ages	0.0 (0.0 to 0.0)	13.8% (3.9 to 26.4%)	5.0 (1.4 to 9.7)
Lower respiratory infections	Antigua and Barbuda	2010	All Ages	0.0 (0.0 to 0.0)	11.3% (2.2 to 23.6%)	3.9 (0.7 to 8.0)
Lower respiratory infections	Antigua and Barbuda	2015	All Ages	0.0 (0.0 to 0.0)	11.8% (2.6 to 24.9%)	3.9 (0.9 to 8.2)
Lower respiratory infections	Antigua and Barbuda	1990	Age-standardized		14.3% (3.9 to 27.1%)	6.7 (1.8 to 12.7)
Lower respiratory infections	Antigua and Barbuda	1995	Age-standardized		14.3% (3.7 to 27.2%)	6.5 (1.6 to 12.4)
Lower respiratory infections	Antigua and Barbuda	2000	Age-standardized		14.4% (3.9 to 27.2%)	6.5 (1.7 to 12.5)
Lower respiratory infections	Antigua and Barbuda	2005	Age-standardized		13.8% (3.9 to 26.4%)	6.0 (1.7 to 11.5)
Lower respiratory infections	Antigua and Barbuda	2010	Age-standardized		11.3% (2.2 to 23.6%)	4.9 (0.9 to 10.1)

Cause name	Location	Year	Age group	Number (in thousands)	PAF	Rate (per 100,000)
Lower respiratory infections	Antigua and Barbuda	2015	Age-standardized		11.8% (2.6 to 24.9%)	5.0 (1.1 to 10.5)
Tracheal, bronchus, and lung cancer	Antigua and Barbuda	1990	All Ages	0.0 (0.0 to 0.0)	9.9% (3.6 to 18.4%)	0.8 (0.3 to 1.5)
Tracheal, bronchus, and lung cancer	Antigua and Barbuda	1995	All Ages	0.0 (0.0 to 0.0)	9.9% (3.7 to 18.4%)	0.8 (0.3 to 1.5)
Tracheal, bronchus, and lung cancer	Antigua and Barbuda	2000	All Ages	0.0 (0.0 to 0.0)	10.0% (3.5 to 18.2%)	0.8 (0.3 to 1.4)
Tracheal, bronchus, and lung cancer	Antigua and Barbuda	2005	All Ages	0.0 (0.0 to 0.0)	9.5% (3.5 to 17.6%)	0.7 (0.3 to 1.4)
Tracheal, bronchus, and lung cancer	Antigua and Barbuda	2010	All Ages	0.0 (0.0 to 0.0)	7.8% (2.3 to 15.2%)	0.6 (0.2 to 1.2)
Tracheal, bronchus, and lung cancer	Antigua and Barbuda	2015	All Ages	0.0 (0.0 to 0.0)	8.1% (2.6 to 15.8%)	0.7 (0.2 to 1.3)
Tracheal, bronchus, and lung cancer	Antigua and Barbuda	1990	Age-standardized		9.9% (3.6 to 18.5%)	1.0 (0.4 to 1.9)
Tracheal, bronchus, and lung cancer	Antigua and Barbuda	1995	Age-standardized		9.9% (3.7 to 18.5%)	1.0 (0.4 to 1.9)
Tracheal, bronchus, and lung cancer	Antigua and Barbuda	2000	Age-standardized		10.0% (3.5 to 18.3%)	1.1 (0.4 to 2.0)
Tracheal, bronchus, and lung cancer	Antigua and Barbuda	2005	Age-standardized		9.5% (3.6 to 17.6%)	1.0 (0.4 to 1.9)
Tracheal, bronchus, and lung cancer	Antigua and Barbuda	2010	Age-standardized		7.8% (2.3 to 15.2%)	0.8 (0.2 to 1.6)
Tracheal, bronchus, and lung cancer	Antigua and Barbuda	2015	Age-standardized		8.1% (2.6 to 15.8%)	0.8 (0.3 to 1.6)
Cardiovascular diseases	Antigua and Barbuda	1990	All Ages	0.0 (0.0 to 0.0)	9.5% (6.5 to 12.5%)	25.3 (17.2 to 33.6)
Cardiovascular diseases	Antigua and Barbuda	1995	All Ages	0.0 (0.0 to 0.0)	9.4% (6.5 to 12.3%)	25.3 (17.4 to 33.4)
Cardiovascular diseases	Antigua and Barbuda	2000	All Ages	0.0 (0.0 to 0.0)	9.2% (6.2 to 12.3%)	21.3 (14.4 to 28.2)
Cardiovascular diseases	Antigua and Barbuda	2005	All Ages	0.0 (0.0 to 0.0)	8.9% (6.1 to 11.7%)	20.2 (13.6 to 26.6)
Cardiovascular diseases	Antigua and Barbuda	2010	All Ages	0.0 (0.0 to 0.0)	8.1% (5.2 to 11.0%)	16.8 (10.6 to 22.8)
Cardiovascular diseases	Antigua and Barbuda	2015	All Ages	0.0 (0.0 to 0.0)	8.2% (5.5 to 11.0%)	17.3 (11.2 to 23.7)
Cardiovascular diseases	Antigua and Barbuda	1990	Age-standardized		9.5% (6.6 to 12.6%)	32.9 (22.4 to 44.2)
Cardiovascular diseases	Antigua and Barbuda	1995	Age-standardized		9.5% (6.6 to 12.4%)	31.6 (21.8 to 41.2)
Cardiovascular diseases	Antigua and Barbuda	2000	Age-standardized		9.3% (6.3 to 12.4%)	28.4 (19.2 to 37.4)
Cardiovascular diseases	Antigua and Barbuda	2005	Age-standardized		9.0% (6.3 to 11.7%)	25.9 (17.7 to 34.1)
Cardiovascular diseases	Antigua and Barbuda	2010	Age-standardized		8.0% (5.1 to 10.8%)	21.6 (13.6 to 29.2)
Cardiovascular diseases	Antigua and Barbuda	2015	Age-standardized		8.0% (5.3 to 10.7%)	21.4 (13.9 to 29.3)
Ischemic heart disease	Antigua and Barbuda	1990	All Ages	0.0 (0.0 to 0.0)	13.5% (9.2 to 17.8%)	15.0 (10.1 to 19.9)
Ischemic heart disease	Antigua and Barbuda	1995	All Ages	0.0 (0.0 to 0.0)	13.4% (9.0 to 17.7%)	15.2 (10.2 to 20.4)
Ischemic heart disease	Antigua and Barbuda	2000	All Ages	0.0 (0.0 to 0.0)	13.2% (8.9 to 17.6%)	13.3 (8.9 to 17.7)
Ischemic heart disease	Antigua and Barbuda	2005	All Ages	0.0 (0.0 to 0.0)	12.8% (8.6 to 17.2%)	13.1 (8.8 to 17.9)
Ischemic heart disease	Antigua and Barbuda	2010	All Ages	0.0 (0.0 to 0.0)	11.7% (7.1 to 16.3%)	11.1 (6.6 to 15.5)
Ischemic heart disease	Antigua and Barbuda	2015	All Ages	0.0 (0.0 to 0.0)	12.1% (7.7 to 16.5%)	11.3 (7.3 to 15.6)
Ischemic heart disease	Antigua and Barbuda	1990	Age-standardized		13.5% (9.1 to 17.9%)	19.6 (13.1 to 26.3)
Ischemic heart disease	Antigua and Barbuda	1995	Age-standardized		13.5% (9.1 to 17.8%)	19.0 (12.9 to 25.2)
Ischemic heart disease	Antigua and Barbuda	2000	Age-standardized		13.3% (9.0 to 17.6%)	17.7 (11.9 to 23.3)
Ischemic heart disease	Antigua and Barbuda	2005	Age-standardized		12.8% (8.8 to 17.3%)	16.8 (11.4 to 22.7)
Ischemic heart disease	Antigua and Barbuda	2010	Age-standardized		11.6% (7.0 to 16.0%)	14.3 (8.5 to 19.9)

Cause name	Location	Year	Age group	Number (in thousands)	PAF	Rate (per 100,000)
Ischemic heart disease	Antigua and Barbuda	2015	Age-standardized		11.7% (7.4 to 16.2%)	14.0 (8.8 to 19.5)
Ischemic stroke	Antigua and Barbuda	1990	All Ages	0.0 (0.0 to 0.0)	9.4% (5.4 to 13.5%)	5.5 (3.1 to 8.0)
Ischemic stroke	Antigua and Barbuda	1995	All Ages	0.0 (0.0 to 0.0)	9.2% (5.3 to 13.7%)	5.2 (3.0 to 7.9)
Ischemic stroke	Antigua and Barbuda	2000	All Ages	0.0 (0.0 to 0.0)	9.2% (5.1 to 13.5%)	3.7 (2.1 to 5.5)
Ischemic stroke	Antigua and Barbuda	2005	All Ages	0.0 (0.0 to 0.0)	9.0% (4.9 to 13.2%)	3.1 (1.7 to 4.6)
Ischemic stroke	Antigua and Barbuda	2010	All Ages	0.0 (0.0 to 0.0)	8.1% (4.3 to 12.8%)	2.4 (1.2 to 3.8)
Ischemic stroke	Antigua and Barbuda	2015	All Ages	0.0 (0.0 to 0.0)	8.3% (4.5 to 13.1%)	2.5 (1.3 to 3.9)
Ischemic stroke	Antigua and Barbuda	1990	Age-standardized		9.3% (5.3 to 13.7%)	7.0 (4.1 to 10.4)
Ischemic stroke	Antigua and Barbuda	1995	Age-standardized		9.3% (5.4 to 13.8%)	6.4 (3.7 to 9.6)
Ischemic stroke	Antigua and Barbuda	2000	Age-standardized		9.3% (5.3 to 13.5%)	5.0 (2.8 to 7.2)
Ischemic stroke	Antigua and Barbuda	2005	Age-standardized		9.1% (5.2 to 13.3%)	4.0 (2.2 to 5.9)
Ischemic stroke	Antigua and Barbuda	2010	Age-standardized		8.1% (4.3 to 12.7%)	3.1 (1.6 to 5.0)
Ischemic stroke	Antigua and Barbuda	2015	Age-standardized		8.2% (4.3 to 13.0%)	3.2 (1.6 to 5.1)
Hemorrhagic stroke	Antigua and Barbuda	1990	All Ages	0.0 (0.0 to 0.0)	10.3% (6.2 to 14.3%)	4.8 (2.9 to 6.8)
Hemorrhagic stroke	Antigua and Barbuda	1995	All Ages	0.0 (0.0 to 0.0)	10.2% (6.3 to 14.4%)	4.9 (3.0 to 7.0)
Hemorrhagic stroke	Antigua and Barbuda	2000	All Ages	0.0 (0.0 to 0.0)	10.2% (6.4 to 14.2%)	4.4 (2.7 to 6.1)
Hemorrhagic stroke	Antigua and Barbuda	2005	All Ages	0.0 (0.0 to 0.0)	10.0% (6.1 to 13.9%)	4.0 (2.4 to 5.6)
Hemorrhagic stroke	Antigua and Barbuda	2010	All Ages	0.0 (0.0 to 0.0)	9.0% (5.3 to 13.1%)	3.3 (2.0 to 4.8)
Hemorrhagic stroke	Antigua and Barbuda	2015	All Ages	0.0 (0.0 to 0.0)	9.3% (5.5 to 13.4%)	3.5 (2.0 to 5.2)
Hemorrhagic stroke	Antigua and Barbuda	1990	Age-standardized		10.4% (6.2 to 14.7%)	6.3 (3.8 to 8.9)
Hemorrhagic stroke	Antigua and Barbuda	1995	Age-standardized		10.3% (6.4 to 14.6%)	6.2 (3.8 to 8.7)
Hemorrhagic stroke	Antigua and Barbuda	2000	Age-standardized		10.2% (6.3 to 14.1%)	5.8 (3.5 to 8.0)
Hemorrhagic stroke	Antigua and Barbuda	2005	Age-standardized		10.0% (6.2 to 13.9%)	5.1 (3.2 to 7.2)
Hemorrhagic stroke	Antigua and Barbuda	2010	Age-standardized		8.8% (5.2 to 13.0%)	4.2 (2.4 to 6.1)
Hemorrhagic stroke	Antigua and Barbuda	2015	Age-standardized		9.0% (5.3 to 13.2%)	4.2 (2.3 to 6.2)
Chronic obstructive pulmonary disease	Antigua and Barbuda	1990	All Ages	0.0 (0.0 to 0.0)	15.7% (5.4 to 27.9%)	1.2 (0.4 to 2.1)
Chronic obstructive pulmonary disease	Antigua and Barbuda	1995	All Ages	0.0 (0.0 to 0.0)	15.7% (5.8 to 28.2%)	1.2 (0.4 to 2.1)
Chronic obstructive pulmonary disease	Antigua and Barbuda	2000	All Ages	0.0 (0.0 to 0.0)	15.8% (5.5 to 28.8%)	1.1 (0.4 to 2.0)
Chronic obstructive pulmonary disease	Antigua and Barbuda	2005	All Ages	0.0 (0.0 to 0.0)	15.3% (4.9 to 27.6%)	1.1 (0.3 to 1.9)
Chronic obstructive pulmonary disease	Antigua and Barbuda	2010	All Ages	0.0 (0.0 to 0.0)	13.1% (4.1 to 24.8%)	0.8 (0.3 to 1.6)
Chronic obstructive pulmonary disease	Antigua and Barbuda	2015	All Ages	0.0 (0.0 to 0.0)	13.6% (3.9 to 26.0%)	0.9 (0.2 to 1.6)
Chronic obstructive pulmonary disease	Antigua and Barbuda	1990	Age-standardized		15.8% (5.4 to 28.1%)	1.5 (0.5 to 2.6)
Chronic obstructive pulmonary disease	Antigua and Barbuda	1995	Age-standardized		15.7% (5.8 to 28.3%)	1.4 (0.5 to 2.6)
Chronic obstructive pulmonary disease	Antigua and Barbuda	2000	Age-standardized		15.9% (5.5 to 28.9%)	1.5 (0.5 to 2.7)
Chronic obstructive pulmonary disease	Antigua and Barbuda	2005	Age-standardized		15.4% (4.9 to 27.7%)	1.4 (0.4 to 2.5)
Chronic obstructive pulmonary disease	Antigua and Barbuda	2010	Age-standardized		13.2% (4.1 to 24.9%)	1.1 (0.3 to 2.1)

Cause name	Location	Year	Age group	Number (in thousands)	PAF	Rate (per 100,000)
Chronic obstructive pulmonary disease	Antigua and Barbuda	2015	Age-standardized		13.7% (3.9 to 26.0%)	1.1 (0.3 to 2.1)
All causes	The Bahamas	1990	All Ages	0.1 (0.1 to 0.1)	4.4% (3.2 to 5.7%)	27.7 (20.5 to 35.7)
All causes	The Bahamas	1995	All Ages	0.1 (0.1 to 0.1)	4.1% (3.0 to 5.2%)	25.5 (18.7 to 32.7)
All causes	The Bahamas	2000	All Ages	0.1 (0.1 to 0.1)	4.0% (2.8 to 5.2%)	26.4 (18.6 to 35.0)
All causes	The Bahamas	2005	All Ages	0.1 (0.1 to 0.1)	3.8% (2.7 to 5.0%)	25.5 (17.9 to 33.4)
All causes	The Bahamas	2010	All Ages	0.1 (0.1 to 0.1)	3.0% (1.9 to 4.1%)	20.8 (13.2 to 28.0)
All causes	The Bahamas	2015	All Ages	0.1 (0.1 to 0.1)	3.5% (2.4 to 4.7%)	25.7 (17.0 to 34.6)
All causes	The Bahamas	1990	Age-standardized		5.0% (3.7 to 6.5%)	56.1 (41.6 to 73.0)
All causes	The Bahamas	1995	Age-standardized		4.6% (3.3 to 5.9%)	47.2 (34.0 to 60.8)
All causes	The Bahamas	2000	Age-standardized		4.4% (3.1 to 5.7%)	43.9 (30.8 to 58.6)
All causes	The Bahamas	2005	Age-standardized		4.0% (2.9 to 5.3%)	37.4 (26.0 to 49.6)
All causes	The Bahamas	2010	Age-standardized		3.2% (2.0 to 4.3%)	27.7 (17.3 to 37.9)
All causes	The Bahamas	2015	Age-standardized		3.6% (2.3 to 4.8%)	29.6 (19.4 to 40.2)
Lower respiratory infections	The Bahamas	1990	All Ages	0.0 (0.0 to 0.0)	16.0% (5.9 to 26.7%)	5.5 (2.1 to 9.6)
Lower respiratory infections	The Bahamas	1995	All Ages	0.0 (0.0 to 0.0)	15.4% (6.1 to 26.8%)	4.6 (1.8 to 8.1)
Lower respiratory infections	The Bahamas	2000	All Ages	0.0 (0.0 to 0.0)	15.5% (5.5 to 27.5%)	4.6 (1.6 to 8.3)
Lower respiratory infections	The Bahamas	2005	All Ages	0.0 (0.0 to 0.0)	14.6% (5.4 to 26.1%)	4.5 (1.7 to 8.4)
Lower respiratory infections	The Bahamas	2010	All Ages	0.0 (0.0 to 0.0)	10.4% (2.5 to 22.1%)	3.3 (0.8 to 7.1)
Lower respiratory infections	The Bahamas	2015	All Ages	0.0 (0.0 to 0.0)	11.8% (2.9 to 23.4%)	4.1 (1.0 to 8.3)
Lower respiratory infections	The Bahamas	1990	Age-standardized		16.0% (5.9 to 26.7%)	10.1 (3.9 to 17.9)
Lower respiratory infections	The Bahamas	1995	Age-standardized		15.4% (6.1 to 26.8%)	8.2 (3.1 to 14.7)
Lower respiratory infections	The Bahamas	2000	Age-standardized		15.5% (5.5 to 27.5%)	7.7 (2.8 to 13.9)
Lower respiratory infections	The Bahamas	2005	Age-standardized		14.6% (5.4 to 26.1%)	6.9 (2.5 to 12.9)
Lower respiratory infections	The Bahamas	2010	Age-standardized		10.4% (2.5 to 22.1%)	4.7 (1.1 to 10.1)
Lower respiratory infections	The Bahamas	2015	Age-standardized		11.8% (2.9 to 23.4%)	5.1 (1.3 to 10.3)
Tracheal, bronchus, and lung cancer	The Bahamas	1990	All Ages	0.0 (0.0 to 0.0)	11.0% (5.2 to 18.9%)	1.2 (0.5 to 2.0)
Tracheal, bronchus, and lung cancer	The Bahamas	1995	All Ages	0.0 (0.0 to 0.0)	10.6% (4.8 to 18.5%)	1.1 (0.5 to 1.9)
Tracheal, bronchus, and lung cancer	The Bahamas	2000	All Ages	0.0 (0.0 to 0.0)	10.7% (4.6 to 19.1%)	1.2 (0.5 to 2.3)
Tracheal, bronchus, and lung cancer	The Bahamas	2005	All Ages	0.0 (0.0 to 0.0)	10.1% (4.4 to 17.4%)	1.2 (0.5 to 2.2)
Tracheal, bronchus, and lung cancer	The Bahamas	2010	All Ages	0.0 (0.0 to 0.0)	7.0% (2.6 to 13.3%)	0.9 (0.3 to 1.7)
Tracheal, bronchus, and lung cancer	The Bahamas	2015	All Ages	0.0 (0.0 to 0.0)	8.0% (3.4 to 14.3%)	1.2 (0.4 to 2.1)
Tracheal, bronchus, and lung cancer	The Bahamas	1990	Age-standardized		11.1% (5.2 to 19.0%)	2.2 (1.0 to 3.8)
Tracheal, bronchus, and lung cancer	The Bahamas	1995	Age-standardized		10.7% (4.8 to 18.6%)	2.0 (0.8 to 3.3)
Tracheal, bronchus, and lung cancer	The Bahamas	2000	Age-standardized		10.8% (4.6 to 19.1%)	1.9 (0.8 to 3.6)
Tracheal, bronchus, and lung cancer	The Bahamas	2005	Age-standardized		10.1% (4.4 to 17.5%)	1.7 (0.7 to 3.0)
Tracheal, bronchus, and lung cancer	The Bahamas	2010	Age-standardized		7.0% (2.6 to 13.3%)	1.1 (0.4 to 2.0)

Cause name	Location	Year	Age group	Number (in thousands)	PAF	Rate (per 100,000)
Tracheal, bronchus, and lung cancer	The Bahamas	2015	Age-standardized		8.0% (3.4 to 14.3%)	1.3 (0.5 to 2.3)
Cardiovascular diseases	The Bahamas	1990	All Ages	0.1 (0.0 to 0.1)	10.1% (7.9 to 12.6%)	19.6 (15.3 to 24.5)
Cardiovascular diseases	The Bahamas	1995	All Ages	0.1 (0.0 to 0.1)	10.0% (7.7 to 12.3%)	18.5 (14.2 to 23.0)
Cardiovascular diseases	The Bahamas	2000	All Ages	0.1 (0.0 to 0.1)	9.5% (7.0 to 12.0%)	19.3 (14.2 to 24.5)
Cardiovascular diseases	The Bahamas	2005	All Ages	0.1 (0.0 to 0.1)	8.7% (6.5 to 10.9%)	18.5 (13.7 to 23.5)
Cardiovascular diseases	The Bahamas	2010	All Ages	0.1 (0.0 to 0.1)	7.2% (5.0 to 9.4%)	15.7 (10.7 to 20.5)
Cardiovascular diseases	The Bahamas	2015	All Ages	0.1 (0.1 to 0.1)	7.6% (5.3 to 9.7%)	19.2 (13.1 to 25.1)
Cardiovascular diseases	The Bahamas	1990	Age-standardized		9.6% (7.4 to 12.1%)	40.6 (31.2 to 51.7)
Cardiovascular diseases	The Bahamas	1995	Age-standardized		9.3% (7.0 to 11.6%)	34.5 (26.1 to 43.7)
Cardiovascular diseases	The Bahamas	2000	Age-standardized		9.0% (6.5 to 11.4%)	32.0 (23.1 to 41.4)
Cardiovascular diseases	The Bahamas	2005	Age-standardized		8.2% (6.0 to 10.3%)	26.8 (19.6 to 34.2)
Cardiovascular diseases	The Bahamas	2010	Age-standardized		6.8% (4.6 to 8.9%)	20.6 (14.2 to 27.3)
Cardiovascular diseases	The Bahamas	2015	Age-standardized		7.2% (4.9 to 9.4%)	21.8 (14.5 to 28.9)
Ischemic heart disease	The Bahamas	1990	All Ages	0.0 (0.0 to 0.0)	15.3% (11.8 to 19.4%)	13.5 (10.3 to 17.0)
Ischemic heart disease	The Bahamas	1995	All Ages	0.0 (0.0 to 0.1)	15.4% (11.7 to 19.3%)	12.7 (9.6 to 16.1)
Ischemic heart disease	The Bahamas	2000	All Ages	0.0 (0.0 to 0.1)	15.1% (11.0 to 19.0%)	13.4 (9.8 to 17.2)
Ischemic heart disease	The Bahamas	2005	All Ages	0.0 (0.0 to 0.1)	14.5% (10.6 to 18.2%)	12.5 (9.1 to 16.0)
Ischemic heart disease	The Bahamas	2010	All Ages	0.0 (0.0 to 0.1)	12.3% (8.3 to 16.3%)	10.6 (7.2 to 14.2)
Ischemic heart disease	The Bahamas	2015	All Ages	0.1 (0.0 to 0.1)	12.8% (8.9 to 16.5%)	13.1 (9.0 to 17.1)
Ischemic heart disease	The Bahamas	1990	Age-standardized		14.2% (10.7 to 18.2%)	27.7 (20.8 to 35.8)
Ischemic heart disease	The Bahamas	1995	Age-standardized		14.1% (10.4 to 18.0%)	23.6 (17.3 to 30.6)
Ischemic heart disease	The Bahamas	2000	Age-standardized		14.0% (9.7 to 18.1%)	22.1 (15.7 to 28.8)
Ischemic heart disease	The Bahamas	2005	Age-standardized		13.5% (9.7 to 17.3%)	18.0 (12.7 to 23.1)
Ischemic heart disease	The Bahamas	2010	Age-standardized		11.5% (7.7 to 15.6%)	13.9 (9.2 to 18.9)
Ischemic heart disease	The Bahamas	2015	Age-standardized		12.2% (8.3 to 15.9%)	14.8 (10.0 to 19.7)
Ischemic stroke	The Bahamas	1990	All Ages	0.0 (0.0 to 0.0)	10.5% (7.1 to 14.3%)	3.1 (2.0 to 4.2)
Ischemic stroke	The Bahamas	1995	All Ages	0.0 (0.0 to 0.0)	10.3% (7.0 to 13.8%)	2.7 (1.8 to 3.7)
Ischemic stroke	The Bahamas	2000	All Ages	0.0 (0.0 to 0.0)	10.2% (6.5 to 14.2%)	2.6 (1.6 to 3.6)
Ischemic stroke	The Bahamas	2005	All Ages	0.0 (0.0 to 0.0)	9.7% (6.1 to 13.6%)	2.6 (1.6 to 3.7)
Ischemic stroke	The Bahamas	2010	All Ages	0.0 (0.0 to 0.0)	8.0% (4.6 to 12.1%)	2.1 (1.2 to 3.3)
Ischemic stroke	The Bahamas	2015	All Ages	0.0 (0.0 to 0.0)	8.5% (4.6 to 12.4%)	2.7 (1.4 to 4.1)
Ischemic stroke	The Bahamas	1990	Age-standardized		9.9% (6.3 to 13.9%)	7.0 (4.3 to 9.9)
Ischemic stroke	The Bahamas	1995	Age-standardized		9.7% (6.2 to 13.6%)	5.7 (3.6 to 8.1)
Ischemic stroke	The Bahamas	2000	Age-standardized		9.7% (5.9 to 13.9%)	4.7 (2.9 to 6.8)
Ischemic stroke	The Bahamas	2005	Age-standardized		9.3% (5.7 to 13.4%)	4.1 (2.5 to 6.1)
Ischemic stroke	The Bahamas	2010	Age-standardized		7.6% (4.1 to 11.9%)	3.1 (1.7 to 5.0)

Cause name	Location	Year	Age group	Number (in thousands)	PAF	Rate (per 100,000)
Ischemic stroke	The Bahamas	2015	Age-standardized		8.2% (4.3 to 12.4%)	3.2 (1.6 to 5.1)
Hemorrhagic stroke	The Bahamas	1990	All Ages	0.0 (0.0 to 0.0)	11.6% (8.2 to 15.2%)	3.1 (2.2 to 4.1)
Hemorrhagic stroke	The Bahamas	1995	All Ages	0.0 (0.0 to 0.0)	11.8% (8.4 to 15.4%)	3.1 (2.2 to 4.1)
Hemorrhagic stroke	The Bahamas	2000	All Ages	0.0 (0.0 to 0.0)	11.6% (8.0 to 15.3%)	3.3 (2.3 to 4.5)
Hemorrhagic stroke	The Bahamas	2005	All Ages	0.0 (0.0 to 0.0)	11.0% (7.5 to 14.7%)	3.4 (2.4 to 4.7)
Hemorrhagic stroke	The Bahamas	2010	All Ages	0.0 (0.0 to 0.0)	9.0% (5.5 to 12.8%)	2.9 (1.8 to 4.2)
Hemorrhagic stroke	The Bahamas	2015	All Ages	0.0 (0.0 to 0.0)	9.5% (5.7 to 13.1%)	3.4 (2.0 to 4.8)
Hemorrhagic stroke	The Bahamas	1990	Age-standardized		11.0% (7.7 to 14.8%)	5.9 (4.1 to 8.0)
Hemorrhagic stroke	The Bahamas	1995	Age-standardized		11.0% (7.6 to 14.4%)	5.3 (3.6 to 6.9)
Hemorrhagic stroke	The Bahamas	2000	Age-standardized		10.9% (7.3 to 14.7%)	5.2 (3.5 to 7.1)
Hemorrhagic stroke	The Bahamas	2005	Age-standardized		10.3% (6.9 to 14.1%)	4.8 (3.2 to 6.5)
Hemorrhagic stroke	The Bahamas	2010	Age-standardized		8.5% (5.1 to 12.2%)	3.7 (2.2 to 5.3)
Hemorrhagic stroke	The Bahamas	2015	Age-standardized		9.1% (5.3 to 12.7%)	3.8 (2.2 to 5.4)
Chronic obstructive pulmonary disease	The Bahamas	1990	All Ages	0.0 (0.0 to 0.0)	16.7% (6.6 to 27.6%)	1.4 (0.6 to 2.3)
Chronic obstructive pulmonary disease	The Bahamas	1995	All Ages	0.0 (0.0 to 0.0)	16.4% (6.6 to 27.9%)	1.2 (0.5 to 2.1)
Chronic obstructive pulmonary disease	The Bahamas	2000	All Ages	0.0 (0.0 to 0.0)	16.6% (7.0 to 27.9%)	1.3 (0.5 to 2.2)
Chronic obstructive pulmonary disease	The Bahamas	2005	All Ages	0.0 (0.0 to 0.0)	15.9% (6.5 to 27.3%)	1.2 (0.5 to 2.1)
Chronic obstructive pulmonary disease	The Bahamas	2010	All Ages	0.0 (0.0 to 0.0)	12.2% (3.8 to 22.3%)	0.9 (0.3 to 1.7)
Chronic obstructive pulmonary disease	The Bahamas	2015	All Ages	0.0 (0.0 to 0.0)	13.5% (4.3 to 24.8%)	1.2 (0.4 to 2.2)
Chronic obstructive pulmonary disease	The Bahamas	1990	Age-standardized		17.1% (6.8 to 28.2%)	3.2 (1.3 to 5.2)
Chronic obstructive pulmonary disease	The Bahamas	1995	Age-standardized		16.6% (6.7 to 28.3%)	2.5 (1.0 to 4.3)
Chronic obstructive pulmonary disease	The Bahamas	2000	Age-standardized		16.7% (7.0 to 28.1%)	2.3 (1.0 to 3.9)
Chronic obstructive pulmonary disease	The Bahamas	2005	Age-standardized		16.0% (6.5 to 27.4%)	1.9 (0.8 to 3.3)
Chronic obstructive pulmonary disease	The Bahamas	2010	Age-standardized		12.3% (3.8 to 22.4%)	1.3 (0.4 to 2.4)
Chronic obstructive pulmonary disease	The Bahamas	2015	Age-standardized		13.6% (4.3 to 24.8%)	1.4 (0.5 to 2.7)
All causes	Barbados	1990	All Ages	0.1 (0.1 to 0.1)	5.1% (3.4 to 6.8%)	41.5 (27.9 to 56.2)
All causes	Barbados	1995	All Ages	0.1 (0.1 to 0.2)	5.0% (3.3 to 7.1%)	44.4 (28.6 to 62.4)
All causes	Barbados	2000	All Ages	0.1 (0.1 to 0.2)	4.6% (2.9 to 6.5%)	41.4 (25.3 to 58.9)
All causes	Barbados	2005	All Ages	0.1 (0.1 to 0.1)	4.1% (2.4 to 5.8%)	35.9 (21.2 to 51.2)
All causes	Barbados	2010	All Ages	0.1 (0.1 to 0.1)	3.7% (2.0 to 5.4%)	34.5 (19.0 to 50.3)
All causes	Barbados	2015	All Ages	0.1 (0.1 to 0.2)	3.8% (2.2 to 5.5%)	37.8 (21.9 to 55.1)
All causes	Barbados	1990	Age-standardized		5.1% (3.5 to 6.8%)	43.0 (29.1 to 58.2)
All causes	Barbados	1995	Age-standardized		5.0% (3.3 to 7.0%)	42.1 (27.6 to 58.9)
All causes	Barbados	2000	Age-standardized		4.5% (2.8 to 6.4%)	36.1 (22.0 to 51.0)
All causes	Barbados	2005	Age-standardized		4.0% (2.4 to 5.7%)	29.6 (17.6 to 42.1)
All causes	Barbados	2010	Age-standardized		3.6% (2.0 to 5.2%)	26.4 (14.7 to 38.3)

Cause name	Location	Year	Age group	Number (in thousands)	PAF	Rate (per 100,000)
All causes	Barbados	2015	Age-standardized		3.7% (2.2 to 5.3%)	26.6 (15.5 to 38.7)
Lower respiratory infections	Barbados	1990	All Ages	0.0 (0.0 to 0.0)	16.6% (5.3 to 29.4%)	6.1 (2.0 to 11.1)
Lower respiratory infections	Barbados	1995	All Ages	0.0 (0.0 to 0.0)	16.8% (5.4 to 30.6%)	7.0 (2.2 to 12.8)
Lower respiratory infections	Barbados	2000	All Ages	0.0 (0.0 to 0.1)	16.4% (4.6 to 30.5%)	9.1 (2.6 to 17.2)
Lower respiratory infections	Barbados	2005	All Ages	0.0 (0.0 to 0.1)	14.4% (3.1 to 27.8%)	9.3 (2.0 to 18.3)
Lower respiratory infections	Barbados	2010	All Ages	0.0 (0.0 to 0.1)	12.5% (2.5 to 25.4%)	8.6 (1.7 to 17.4)
Lower respiratory infections	Barbados	2015	All Ages	0.0 (0.0 to 0.1)	13.0% (3.1 to 26.0%)	9.1 (2.2 to 18.5)
Lower respiratory infections	Barbados	1990	Age-standardized		16.6% (5.3 to 29.4%)	6.2 (2.1 to 11.2)
Lower respiratory infections	Barbados	1995	Age-standardized		16.8% (5.4 to 30.6%)	6.5 (2.0 to 11.8)
Lower respiratory infections	Barbados	2000	Age-standardized		16.4% (4.6 to 30.5%)	7.8 (2.2 to 14.6)
Lower respiratory infections	Barbados	2005	Age-standardized		14.4% (3.1 to 27.8%)	7.5 (1.6 to 14.8)
Lower respiratory infections	Barbados	2010	Age-standardized		12.5% (2.5 to 25.4%)	6.5 (1.3 to 13.2)
Lower respiratory infections	Barbados	2015	Age-standardized		13.0% (3.1 to 26.0%)	6.5 (1.6 to 13.0)
Tracheal, bronchus, and lung cancer	Barbados	1990	All Ages	0.0 (0.0 to 0.0)	11.6% (4.3 to 20.8%)	1.2 (0.4 to 2.2)
Tracheal, bronchus, and lung cancer	Barbados	1995	All Ages	0.0 (0.0 to 0.0)	11.8% (4.6 to 22.3%)	1.4 (0.5 to 2.6)
Tracheal, bronchus, and lung cancer	Barbados	2000	All Ages	0.0 (0.0 to 0.0)	11.5% (4.2 to 21.3%)	1.4 (0.5 to 2.6)
Tracheal, bronchus, and lung cancer	Barbados	2005	All Ages	0.0 (0.0 to 0.0)	10.0% (3.5 to 19.0%)	1.2 (0.4 to 2.3)
Tracheal, bronchus, and lung cancer	Barbados	2010	All Ages	0.0 (0.0 to 0.0)	8.6% (2.7 to 16.9%)	1.2 (0.4 to 2.3)
Tracheal, bronchus, and lung cancer	Barbados	2015	All Ages	0.0 (0.0 to 0.0)	9.0% (2.7 to 17.1%)	1.4 (0.4 to 2.6)
Tracheal, bronchus, and lung cancer	Barbados	1990	Age-standardized		11.7% (4.3 to 20.8%)	1.3 (0.5 to 2.4)
Tracheal, bronchus, and lung cancer	Barbados	1995	Age-standardized		11.8% (4.6 to 22.4%)	1.4 (0.5 to 2.7)
Tracheal, bronchus, and lung cancer	Barbados	2000	Age-standardized		11.5% (4.1 to 21.3%)	1.3 (0.4 to 2.4)
Tracheal, bronchus, and lung cancer	Barbados	2005	Age-standardized		10.0% (3.5 to 19.0%)	1.1 (0.4 to 2.0)
Tracheal, bronchus, and lung cancer	Barbados	2010	Age-standardized		8.6% (2.7 to 16.8%)	0.9 (0.3 to 1.8)
Tracheal, bronchus, and lung cancer	Barbados	2015	Age-standardized		9.0% (2.7 to 17.1%)	1.0 (0.3 to 1.9)
Cardiovascular diseases	Barbados	1990	All Ages	0.1 (0.1 to 0.1)	10.3% (7.1 to 13.3%)	32.2 (22.6 to 42.5)
Cardiovascular diseases	Barbados	1995	All Ages	0.1 (0.1 to 0.1)	10.2% (6.9 to 13.9%)	33.9 (22.9 to 46.4)
Cardiovascular diseases	Barbados	2000	All Ages	0.1 (0.1 to 0.1)	9.7% (6.4 to 12.9%)	28.9 (19.2 to 39.1)
Cardiovascular diseases	Barbados	2005	All Ages	0.1 (0.0 to 0.1)	8.9% (5.9 to 11.9%)	23.8 (15.9 to 31.9)
Cardiovascular diseases	Barbados	2010	All Ages	0.1 (0.0 to 0.1)	8.1% (4.8 to 11.0%)	23.3 (13.8 to 31.8)
Cardiovascular diseases	Barbados	2015	All Ages	0.1 (0.1 to 0.1)	8.3% (5.6 to 11.2%)	25.5 (16.7 to 35.2)
Cardiovascular diseases	Barbados	1990	Age-standardized		10.5% (7.3 to 13.7%)	33.5 (23.4 to 44.0)
Cardiovascular diseases	Barbados	1995	Age-standardized		10.6% (7.2 to 14.2%)	32.3 (22.1 to 43.8)
Cardiovascular diseases	Barbados	2000	Age-standardized		10.0% (6.7 to 13.2%)	25.4 (17.0 to 34.1)
Cardiovascular diseases	Barbados	2005	Age-standardized		9.0% (6.0 to 12.1%)	19.7 (13.3 to 26.2)
Cardiovascular diseases	Barbados	2010	Age-standardized		8.3% (4.9 to 11.1%)	17.9 (10.8 to 24.2)

Cause name	Location	Year	Age group	Number (in thousands)	PAF	Rate (per 100,000)
Cardiovascular diseases	Barbados	2015	Age-standardized		8.4% (5.6 to 11.3%)	18.0 (11.9 to 24.6)
Ischemic heart disease	Barbados	1990	All Ages	0.1 (0.0 to 0.1)	13.9% (9.5 to 18.7%)	20.4 (14.0 to 27.6)
Ischemic heart disease	Barbados	1995	All Ages	0.1 (0.0 to 0.1)	13.9% (9.3 to 18.7%)	21.0 (13.8 to 28.9)
Ischemic heart disease	Barbados	2000	All Ages	0.1 (0.0 to 0.1)	13.8% (9.3 to 18.7%)	18.4 (12.3 to 25.0)
Ischemic heart disease	Barbados	2005	All Ages	0.0 (0.0 to 0.1)	13.1% (8.7 to 17.8%)	14.8 (9.4 to 20.2)
Ischemic heart disease	Barbados	2010	All Ages	0.0 (0.0 to 0.1)	12.3% (7.4 to 16.8%)	14.1 (8.5 to 19.6)
Ischemic heart disease	Barbados	2015	All Ages	0.0 (0.0 to 0.1)	12.6% (8.2 to 17.1%)	15.8 (10.0 to 22.3)
Ischemic heart disease	Barbados	1990	Age-standardized		14.2% (9.8 to 19.0%)	21.3 (14.7 to 28.4)
Ischemic heart disease	Barbados	1995	Age-standardized		14.3% (9.7 to 19.4%)	20.2 (13.5 to 27.3)
Ischemic heart disease	Barbados	2000	Age-standardized		14.2% (9.6 to 19.1%)	16.2 (11.0 to 22.0)
Ischemic heart disease	Barbados	2005	Age-standardized		13.4% (8.9 to 17.9%)	12.4 (8.1 to 16.6)
Ischemic heart disease	Barbados	2010	Age-standardized		12.6% (7.7 to 16.9%)	10.9 (6.6 to 15.0)
Ischemic heart disease	Barbados	2015	Age-standardized		12.7% (8.5 to 17.2%)	11.1 (7.2 to 15.6)
Ischemic stroke	Barbados	1990	All Ages	0.0 (0.0 to 0.0)	9.9% (5.9 to 14.5%)	7.1 (4.2 to 10.7)
Ischemic stroke	Barbados	1995	All Ages	0.0 (0.0 to 0.0)	9.8% (5.3 to 14.9%)	7.7 (4.2 to 11.7)
Ischemic stroke	Barbados	2000	All Ages	0.0 (0.0 to 0.0)	9.8% (5.3 to 14.7%)	5.4 (2.9 to 8.3)
Ischemic stroke	Barbados	2005	All Ages	0.0 (0.0 to 0.0)	9.1% (4.9 to 13.8%)	4.3 (2.4 to 6.7)
Ischemic stroke	Barbados	2010	All Ages	0.0 (0.0 to 0.0)	8.4% (3.8 to 13.2%)	4.3 (2.0 to 6.9)
Ischemic stroke	Barbados	2015	All Ages	0.0 (0.0 to 0.0)	8.6% (4.7 to 13.1%)	4.6 (2.5 to 7.4)
Ischemic stroke	Barbados	1990	Age-standardized		10.0% (6.0 to 14.6%)	7.1 (4.3 to 10.5)
Ischemic stroke	Barbados	1995	Age-standardized		10.1% (5.6 to 15.1%)	7.0 (3.9 to 10.4)
Ischemic stroke	Barbados	2000	Age-standardized		9.9% (5.5 to 14.7%)	4.6 (2.5 to 7.0)
Ischemic stroke	Barbados	2005	Age-standardized		9.3% (5.1 to 13.8%)	3.5 (1.9 to 5.3)
Ischemic stroke	Barbados	2010	Age-standardized		8.5% (4.0 to 13.2%)	3.3 (1.5 to 5.1)
Ischemic stroke	Barbados	2015	Age-standardized		8.7% (4.9 to 13.1%)	3.2 (1.8 to 5.0)
Hemorrhagic stroke	Barbados	1990	All Ages	0.0 (0.0 to 0.0)	10.9% (6.9 to 15.2%)	4.7 (3.0 to 6.7)
Hemorrhagic stroke	Barbados	1995	All Ages	0.0 (0.0 to 0.0)	10.9% (6.5 to 15.7%)	5.2 (3.1 to 7.7)
Hemorrhagic stroke	Barbados	2000	All Ages	0.0 (0.0 to 0.0)	10.7% (6.4 to 15.6%)	5.1 (3.0 to 7.6)
Hemorrhagic stroke	Barbados	2005	All Ages	0.0 (0.0 to 0.0)	10.0% (5.8 to 14.2%)	4.6 (2.6 to 6.7)
Hemorrhagic stroke	Barbados	2010	All Ages	0.0 (0.0 to 0.0)	9.2% (4.7 to 13.6%)	4.8 (2.5 to 7.1)
Hemorrhagic stroke	Barbados	2015	All Ages	0.0 (0.0 to 0.0)	9.4% (5.5 to 13.4%)	5.1 (3.0 to 7.7)
Hemorrhagic stroke	Barbados	1990	Age-standardized		11.2% (7.2 to 15.5%)	5.0 (3.2 to 7.1)
Hemorrhagic stroke	Barbados	1995	Age-standardized		11.2% (6.9 to 16.0%)	5.1 (3.1 to 7.4)
Hemorrhagic stroke	Barbados	2000	Age-standardized		10.9% (6.7 to 15.8%)	4.5 (2.7 to 6.7)
Hemorrhagic stroke	Barbados	2005	Age-standardized		10.1% (5.8 to 14.4%)	3.9 (2.2 to 5.5)
Hemorrhagic stroke	Barbados	2010	Age-standardized		9.3% (4.8 to 13.6%)	3.7 (2.0 to 5.4)

Cause name	Location	Year	Age group	Number (in thousands)	PAF	Rate (per 100,000)
Hemorrhagic stroke	Barbados	2015	Age-standardized		9.5% (5.5 to 13.4%)	3.6 (2.1 to 5.4)
Chronic obstructive pulmonary disease	Barbados	1990	All Ages	0.0 (0.0 to 0.0)	17.6% (6.5 to 30.2%)	1.9 (0.7 to 3.3)
Chronic obstructive pulmonary disease	Barbados	1995	All Ages	0.0 (0.0 to 0.0)	17.9% (6.7 to 31.9%)	2.1 (0.8 to 3.6)
Chronic obstructive pulmonary disease	Barbados	2000	All Ages	0.0 (0.0 to 0.0)	17.6% (6.3 to 30.8%)	2.0 (0.7 to 3.4)
Chronic obstructive pulmonary disease	Barbados	2005	All Ages	0.0 (0.0 to 0.0)	15.9% (5.4 to 29.0%)	1.7 (0.6 to 3.0)
Chronic obstructive pulmonary disease	Barbados	2010	All Ages	0.0 (0.0 to 0.0)	14.3% (4.2 to 26.3%)	1.5 (0.4 to 2.8)
Chronic obstructive pulmonary disease	Barbados	2015	All Ages	0.0 (0.0 to 0.0)	14.8% (4.8 to 27.3%)	1.7 (0.5 to 3.3)
Chronic obstructive pulmonary disease	Barbados	1990	Age-standardized		17.6% (6.5 to 30.1%)	1.9 (0.7 to 3.3)
Chronic obstructive pulmonary disease	Barbados	1995	Age-standardized		17.8% (6.7 to 31.8%)	1.9 (0.7 to 3.3)
Chronic obstructive pulmonary disease	Barbados	2000	Age-standardized		17.5% (6.3 to 30.7%)	1.7 (0.6 to 2.9)
Chronic obstructive pulmonary disease	Barbados	2005	Age-standardized		15.9% (5.3 to 28.9%)	1.3 (0.5 to 2.4)
Chronic obstructive pulmonary disease	Barbados	2010	Age-standardized		14.2% (4.2 to 26.2%)	1.1 (0.3 to 2.1)
Chronic obstructive pulmonary disease	Barbados	2015	Age-standardized		14.7% (4.8 to 27.2%)	1.2 (0.4 to 2.3)
All causes	Belize	1990	All Ages	0.1 (0.1 to 0.1)	6.0% (5.0 to 7.0%)	32.0 (26.7 to 37.9)
All causes	Belize	1995	All Ages	0.1 (0.1 to 0.1)	6.3% (5.4 to 7.4%)	35.8 (30.3 to 41.9)
All causes	Belize	2000	All Ages	0.1 (0.1 to 0.1)	5.8% (4.9 to 6.7%)	32.7 (27.7 to 37.8)
All causes	Belize	2005	All Ages	0.1 (0.1 to 0.1)	4.8% (4.1 to 5.6%)	23.2 (19.6 to 27.2)
All causes	Belize	2010	All Ages	0.1 (0.1 to 0.1)	4.3% (3.6 to 5.1%)	21.3 (17.6 to 25.3)
All causes	Belize	2015	All Ages	0.1 (0.1 to 0.1)	4.9% (4.1 to 5.7%)	23.7 (19.9 to 28.4)
All causes	Belize	1990	Age-standardized		7.2% (6.1 to 8.4%)	65.8 (55.1 to 76.7)
All causes	Belize	1995	Age-standardized		7.3% (6.3 to 8.5%)	73.6 (62.4 to 85.4)
All causes	Belize	2000	Age-standardized		6.9% (5.9 to 8.0%)	76.3 (64.5 to 88.5)
All causes	Belize	2005	Age-standardized		6.0% (5.0 to 7.0%)	62.5 (52.5 to 73.4)
All causes	Belize	2010	Age-standardized		5.1% (4.2 to 6.1%)	47.7 (39.2 to 57.1)
All causes	Belize	2015	Age-standardized		5.8% (4.8 to 6.7%)	53.4 (44.3 to 64.0)
Lower respiratory infections	Belize	1990	All Ages	0.0 (0.0 to 0.0)	21.9% (14.2 to 30.7%)	10.6 (6.7 to 15.3)
Lower respiratory infections	Belize	1995	All Ages	0.0 (0.0 to 0.0)	21.5% (13.8 to 30.5%)	9.9 (6.2 to 14.4)
Lower respiratory infections	Belize	2000	All Ages	0.0 (0.0 to 0.0)	21.8% (14.0 to 30.9%)	8.8 (5.5 to 12.7)
Lower respiratory infections	Belize	2005	All Ages	0.0 (0.0 to 0.0)	19.8% (11.7 to 29.2%)	6.3 (3.7 to 9.4)
Lower respiratory infections	Belize	2010	All Ages	0.0 (0.0 to 0.0)	16.9% (9.1 to 26.5%)	5.4 (2.9 to 8.7)
Lower respiratory infections	Belize	2015	All Ages	0.0 (0.0 to 0.0)	20.5% (12.8 to 29.5%)	6.1 (3.7 to 9.1)
Lower respiratory infections	Belize	1990	Age-standardized		21.9% (14.2 to 30.7%)	16.0 (10.1 to 22.7)
Lower respiratory infections	Belize	1995	Age-standardized		21.5% (13.8 to 30.5%)	16.6 (10.4 to 24.2)
Lower respiratory infections	Belize	2000	Age-standardized		21.8% (14.0 to 30.9%)	17.5 (10.9 to 25.2)
Lower respiratory infections	Belize	2005	Age-standardized		19.8% (11.7 to 29.2%)	14.9 (8.8 to 22.3)
Lower respiratory infections	Belize	2010	Age-standardized		16.9% (9.1 to 26.5%)	11.4 (6.2 to 18.3)

Cause name	Location	Year	Age group	Number (in thousands)	PAF	Rate (per 100,000)
Lower respiratory infections	Belize	2015	Age-standardized		20.5% (12.8 to 29.5%)	13.5 (8.3 to 20.1)
Tracheal, bronchus, and lung cancer	Belize	1990	All Ages	0.0 (0.0 to 0.0)	15.4% (8.9 to 22.3%)	0.9 (0.5 to 1.3)
Tracheal, bronchus, and lung cancer	Belize	1995	All Ages	0.0 (0.0 to 0.0)	15.2% (8.8 to 22.1%)	1.2 (0.7 to 1.7)
Tracheal, bronchus, and lung cancer	Belize	2000	All Ages	0.0 (0.0 to 0.0)	15.4% (9.0 to 22.3%)	1.3 (0.7 to 1.9)
Tracheal, bronchus, and lung cancer	Belize	2005	All Ages	0.0 (0.0 to 0.0)	13.9% (7.9 to 20.8%)	1.0 (0.6 to 1.5)
Tracheal, bronchus, and lung cancer	Belize	2010	All Ages	0.0 (0.0 to 0.0)	11.7% (6.4 to 17.9%)	1.0 (0.5 to 1.5)
Tracheal, bronchus, and lung cancer	Belize	2015	All Ages	0.0 (0.0 to 0.0)	14.4% (8.2 to 21.3%)	1.3 (0.7 to 1.9)
Tracheal, bronchus, and lung cancer	Belize	1990	Age-standardized		15.5% (9.0 to 22.5%)	2.1 (1.2 to 3.1)
Tracheal, bronchus, and lung cancer	Belize	1995	Age-standardized		15.3% (8.8 to 22.3%)	2.6 (1.5 to 3.8)
Tracheal, bronchus, and lung cancer	Belize	2000	Age-standardized		15.5% (9.1 to 22.5%)	3.0 (1.7 to 4.4)
Tracheal, bronchus, and lung cancer	Belize	2005	Age-standardized		14.0% (7.9 to 21.0%)	2.6 (1.5 to 4.0)
Tracheal, bronchus, and lung cancer	Belize	2010	Age-standardized		11.8% (6.4 to 18.0%)	2.1 (1.1 to 3.2)
Tracheal, bronchus, and lung cancer	Belize	2015	Age-standardized		14.5% (8.3 to 21.4%)	2.6 (1.4 to 3.9)
Cardiovascular diseases	Belize	1990	All Ages	0.0 (0.0 to 0.0)	12.1% (10.2 to 14.1%)	18.2 (15.2 to 21.4)
Cardiovascular diseases	Belize	1995	All Ages	0.1 (0.0 to 0.1)	12.4% (10.6 to 14.5%)	21.9 (18.4 to 25.8)
Cardiovascular diseases	Belize	2000	All Ages	0.1 (0.0 to 0.1)	12.6% (10.6 to 14.5%)	19.9 (16.6 to 23.3)
Cardiovascular diseases	Belize	2005	All Ages	0.0 (0.0 to 0.1)	11.8% (10.0 to 13.6%)	13.9 (11.8 to 16.2)
Cardiovascular diseases	Belize	2010	All Ages	0.0 (0.0 to 0.1)	10.7% (9.0 to 12.4%)	13.0 (10.8 to 15.2)
Cardiovascular diseases	Belize	2015	All Ages	0.1 (0.0 to 0.1)	11.8% (10.1 to 13.5%)	14.3 (11.9 to 16.9)
Cardiovascular diseases	Belize	1990	Age-standardized		12.2% (10.2 to 14.4%)	42.2 (35.0 to 50.3)
Cardiovascular diseases	Belize	1995	Age-standardized		12.5% (10.5 to 14.7%)	48.1 (40.2 to 56.8)
Cardiovascular diseases	Belize	2000	Age-standardized		12.3% (10.2 to 14.5%)	48.7 (40.2 to 57.9)
Cardiovascular diseases	Belize	2005	Age-standardized		11.3% (9.4 to 13.4%)	38.9 (32.1 to 46.4)
Cardiovascular diseases	Belize	2010	Age-standardized		10.2% (8.3 to 12.1%)	29.5 (23.8 to 35.2)
Cardiovascular diseases	Belize	2015	Age-standardized		11.0% (9.2 to 12.9%)	32.1 (26.3 to 38.5)
Ischemic heart disease	Belize	1990	All Ages	0.0 (0.0 to 0.0)	16.3% (13.1 to 19.6%)	12.8 (10.1 to 15.6)
Ischemic heart disease	Belize	1995	All Ages	0.0 (0.0 to 0.0)	16.2% (13.0 to 19.5%)	15.6 (12.5 to 19.0)
Ischemic heart disease	Belize	2000	All Ages	0.0 (0.0 to 0.0)	16.7% (13.6 to 19.9%)	14.2 (11.3 to 17.1)
Ischemic heart disease	Belize	2005	All Ages	0.0 (0.0 to 0.0)	16.3% (13.4 to 19.3%)	9.9 (8.1 to 11.9)
Ischemic heart disease	Belize	2010	All Ages	0.0 (0.0 to 0.0)	15.2% (12.4 to 18.1%)	9.1 (7.3 to 11.0)
Ischemic heart disease	Belize	2015	All Ages	0.0 (0.0 to 0.0)	16.8% (13.8 to 19.9%)	9.8 (7.9 to 11.8)
Ischemic heart disease	Belize	1990	Age-standardized		16.0% (12.7 to 19.3%)	29.8 (23.2 to 36.7)
Ischemic heart disease	Belize	1995	Age-standardized		16.0% (12.7 to 19.3%)	34.2 (27.2 to 42.1)
Ischemic heart disease	Belize	2000	Age-standardized		16.0% (12.6 to 19.4%)	34.8 (27.1 to 42.8)
Ischemic heart disease	Belize	2005	Age-standardized		15.2% (11.9 to 18.5%)	27.6 (21.5 to 34.0)
Ischemic heart disease	Belize	2010	Age-standardized		14.2% (11.1 to 17.3%)	20.6 (16.0 to 25.4)

Cause name	Location	Year	Age group	Number (in thousands)	PAF	Rate (per 100,000)
Ischemic heart disease	Belize	2015	Age-standardized		15.3% (12.0 to 18.7%)	22.0 (16.9 to 27.2)
Ischemic stroke	Belize	1990	All Ages	0.0 (0.0 to 0.0)	11.9% (8.7 to 15.2%)	2.8 (2.0 to 3.6)
Ischemic stroke	Belize	1995	All Ages	0.0 (0.0 to 0.0)	11.8% (8.6 to 15.1%)	3.2 (2.3 to 4.1)
Ischemic stroke	Belize	2000	All Ages	0.0 (0.0 to 0.0)	12.0% (8.9 to 15.2%)	2.5 (1.9 to 3.2)
Ischemic stroke	Belize	2005	All Ages	0.0 (0.0 to 0.0)	11.5% (8.5 to 14.5%)	1.7 (1.2 to 2.2)
Ischemic stroke	Belize	2010	All Ages	0.0 (0.0 to 0.0)	10.6% (7.7 to 13.4%)	1.6 (1.2 to 2.2)
Ischemic stroke	Belize	2015	All Ages	0.0 (0.0 to 0.0)	11.9% (9.0 to 15.0%)	1.8 (1.4 to 2.4)
Ischemic stroke	Belize	1990	Age-standardized		11.8% (8.4 to 15.1%)	6.6 (4.7 to 8.6)
Ischemic stroke	Belize	1995	Age-standardized		11.7% (8.5 to 15.0%)	7.1 (5.0 to 9.3)
Ischemic stroke	Belize	2000	Age-standardized		11.7% (8.5 to 15.1%)	6.6 (4.7 to 8.7)
Ischemic stroke	Belize	2005	Age-standardized		11.0% (7.6 to 14.4%)	5.2 (3.6 to 7.0)
Ischemic stroke	Belize	2010	Age-standardized		10.1% (7.1 to 13.4%)	4.1 (2.8 to 5.5)
Ischemic stroke	Belize	2015	Age-standardized		11.2% (7.9 to 14.6%)	4.6 (3.3 to 6.3)
Hemorrhagic stroke	Belize	1990	All Ages	0.0 (0.0 to 0.0)	12.4% (9.9 to 15.1%)	2.6 (2.0 to 3.1)
Hemorrhagic stroke	Belize	1995	All Ages	0.0 (0.0 to 0.0)	12.8% (10.1 to 15.5%)	3.2 (2.5 to 3.9)
Hemorrhagic stroke	Belize	2000	All Ages	0.0 (0.0 to 0.0)	13.2% (10.6 to 15.9%)	3.1 (2.5 to 3.8)
Hemorrhagic stroke	Belize	2005	All Ages	0.0 (0.0 to 0.0)	12.8% (10.2 to 15.4%)	2.3 (1.8 to 2.9)
Hemorrhagic stroke	Belize	2010	All Ages	0.0 (0.0 to 0.0)	11.7% (9.2 to 14.4%)	2.3 (1.8 to 2.9)
Hemorrhagic stroke	Belize	2015	All Ages	0.0 (0.0 to 0.0)	13.4% (10.6 to 16.2%)	2.6 (2.1 to 3.3)
Hemorrhagic stroke	Belize	1990	Age-standardized		13.0% (10.2 to 15.9%)	5.8 (4.5 to 7.1)
Hemorrhagic stroke	Belize	1995	Age-standardized		13.0% (10.3 to 15.8%)	6.8 (5.3 to 8.4)
Hemorrhagic stroke	Belize	2000	Age-standardized		13.0% (10.2 to 15.9%)	7.3 (5.7 to 9.1)
Hemorrhagic stroke	Belize	2005	Age-standardized		12.2% (9.3 to 15.1%)	6.0 (4.6 to 7.6)
Hemorrhagic stroke	Belize	2010	Age-standardized		11.1% (8.4 to 14.0%)	4.9 (3.7 to 6.3)
Hemorrhagic stroke	Belize	2015	Age-standardized		12.4% (9.5 to 15.4%)	5.5 (4.2 to 6.9)
Chronic obstructive pulmonary disease	Belize	1990	All Ages	0.0 (0.0 to 0.0)	19.4% (10.5 to 29.1%)	2.3 (1.2 to 3.5)
Chronic obstructive pulmonary disease	Belize	1995	All Ages	0.0 (0.0 to 0.0)	20.4% (11.0 to 30.7%)	2.8 (1.5 to 4.2)
Chronic obstructive pulmonary disease	Belize	2000	All Ages	0.0 (0.0 to 0.0)	20.7% (11.2 to 31.1%)	2.8 (1.5 to 4.1)
Chronic obstructive pulmonary disease	Belize	2005	All Ages	0.0 (0.0 to 0.0)	19.3% (10.0 to 29.6%)	2.0 (1.0 to 3.1)
Chronic obstructive pulmonary disease	Belize	2010	All Ages	0.0 (0.0 to 0.0)	17.2% (8.3 to 27.5%)	1.9 (0.9 to 3.0)
Chronic obstructive pulmonary disease	Belize	2015	All Ages	0.0 (0.0 to 0.0)	20.1% (10.3 to 30.6%)	2.1 (1.1 to 3.2)
Chronic obstructive pulmonary disease	Belize	1990	Age-standardized		21.3% (11.5 to 32.0%)	5.5 (2.9 to 8.4)
Chronic obstructive pulmonary disease	Belize	1995	Age-standardized		21.3% (11.5 to 32.2%)	6.2 (3.3 to 9.4)
Chronic obstructive pulmonary disease	Belize	2000	Age-standardized		21.6% (11.6 to 32.4%)	7.1 (3.8 to 10.6)
Chronic obstructive pulmonary disease	Belize	2005	Age-standardized		20.1% (10.3 to 30.8%)	6.1 (3.1 to 9.2)
Chronic obstructive pulmonary disease	Belize	2010	Age-standardized		17.7% (8.5 to 28.3%)	4.6 (2.2 to 7.4)

Cause name	Location	Year	Age group	Number (in thousands)	PAF	Rate (per 100,000)
Chronic obstructive pulmonary disease	Belize	2015	Age-standardized		20.6% (10.5 to 31.4%)	5.2 (2.7 to 8.1)
All causes	Bermuda	1990	All Ages	0.0 (0.0 to 0.0)	5.0% (3.5 to 6.5%)	35.8 (25.0 to 46.8)
All causes	Bermuda	1995	All Ages	0.0 (0.0 to 0.0)	4.5% (3.1 to 5.9%)	31.5 (21.8 to 41.1)
All causes	Bermuda	2000	All Ages	0.0 (0.0 to 0.0)	4.0% (2.8 to 5.3%)	26.9 (18.6 to 35.9)
All causes	Bermuda	2005	All Ages	0.0 (0.0 to 0.0)	3.9% (2.6 to 5.3%)	23.3 (15.7 to 31.8)
All causes	Bermuda	2010	All Ages	0.0 (0.0 to 0.0)	3.2% (2.0 to 4.5%)	19.0 (11.6 to 26.9)
All causes	Bermuda	2015	All Ages	0.0 (0.0 to 0.0)	3.0% (1.6 to 4.4%)	18.6 (9.8 to 26.9)
All causes	Bermuda	1990	Age-standardized		5.1% (3.6 to 6.8%)	52.1 (36.1 to 68.3)
All causes	Bermuda	1995	Age-standardized		4.7% (3.2 to 6.1%)	43.7 (30.3 to 57.1)
All causes	Bermuda	2000	Age-standardized		4.1% (2.8 to 5.4%)	35.5 (24.5 to 47.5)
All causes	Bermuda	2005	Age-standardized		3.9% (2.7 to 5.4%)	28.9 (19.5 to 39.5)
All causes	Bermuda	2010	Age-standardized		3.2% (2.0 to 4.5%)	21.7 (13.3 to 30.8)
All causes	Bermuda	2015	Age-standardized		3.0% (1.6 to 4.4%)	19.4 (10.1 to 27.9)
Lower respiratory infections	Bermuda	1990	All Ages	0.0 (0.0 to 0.0)	10.3% (1.8 to 22.6%)	2.4 (0.4 to 5.3)
Lower respiratory infections	Bermuda	1995	All Ages	0.0 (0.0 to 0.0)	9.8% (1.7 to 21.8%)	2.3 (0.4 to 5.1)
Lower respiratory infections	Bermuda	2000	All Ages	0.0 (0.0 to 0.0)	9.7% (1.6 to 21.7%)	2.2 (0.4 to 4.9)
Lower respiratory infections	Bermuda	2005	All Ages	0.0 (0.0 to 0.0)	9.9% (1.6 to 21.6%)	2.2 (0.4 to 4.8)
Lower respiratory infections	Bermuda	2010	All Ages	0.0 (0.0 to 0.0)	7.8% (0.9 to 19.7%)	1.8 (0.2 to 4.6)
Lower respiratory infections	Bermuda	2015	All Ages	0.0 (0.0 to 0.0)	7.1% (0.7 to 20.2%)	1.7 (0.2 to 4.7)
Lower respiratory infections	Bermuda	1990	Age-standardized		10.3% (1.8 to 22.6%)	3.5 (0.6 to 7.8)
Lower respiratory infections	Bermuda	1995	Age-standardized		9.8% (1.7 to 21.8%)	3.2 (0.6 to 7.1)
Lower respiratory infections	Bermuda	2000	Age-standardized		9.7% (1.6 to 21.7%)	3.0 (0.5 to 6.5)
Lower respiratory infections	Bermuda	2005	Age-standardized		9.9% (1.6 to 21.6%)	2.8 (0.4 to 6.0)
Lower respiratory infections	Bermuda	2010	Age-standardized		7.8% (0.9 to 19.7%)	2.1 (0.3 to 5.3)
Lower respiratory infections	Bermuda	2015	Age-standardized		7.1% (0.7 to 20.2%)	1.8 (0.2 to 4.9)
Tracheal, bronchus, and lung cancer	Bermuda	1990	All Ages	0.0 (0.0 to 0.0)	7.0% (2.5 to 13.8%)	2.1 (0.8 to 4.2)
Tracheal, bronchus, and lung cancer	Bermuda	1995	All Ages	0.0 (0.0 to 0.0)	6.6% (2.3 to 12.6%)	2.0 (0.7 to 3.9)
Tracheal, bronchus, and lung cancer	Bermuda	2000	All Ages	0.0 (0.0 to 0.0)	6.6% (2.3 to 12.6%)	2.0 (0.7 to 3.8)
Tracheal, bronchus, and lung cancer	Bermuda	2005	All Ages	0.0 (0.0 to 0.0)	6.8% (2.2 to 12.8%)	1.9 (0.6 to 3.5)
Tracheal, bronchus, and lung cancer	Bermuda	2010	All Ages	0.0 (0.0 to 0.0)	5.2% (1.3 to 10.7%)	1.5 (0.4 to 3.0)
Tracheal, bronchus, and lung cancer	Bermuda	2015	All Ages	0.0 (0.0 to 0.0)	4.8% (0.8 to 10.2%)	1.4 (0.2 to 2.9)
Tracheal, bronchus, and lung cancer	Bermuda	1990	Age-standardized		7.0% (2.5 to 13.8%)	3.0 (1.1 to 6.0)
Tracheal, bronchus, and lung cancer	Bermuda	1995	Age-standardized		6.7% (2.3 to 12.7%)	2.8 (0.9 to 5.3)
Tracheal, bronchus, and lung cancer	Bermuda	2000	Age-standardized		6.6% (2.3 to 12.6%)	2.6 (0.9 to 5.0)
Tracheal, bronchus, and lung cancer	Bermuda	2005	Age-standardized		6.8% (2.2 to 12.8%)	2.3 (0.7 to 4.3)
Tracheal, bronchus, and lung cancer	Bermuda	2010	Age-standardized		5.2% (1.3 to 10.7%)	1.7 (0.4 to 3.4)

Cause name	Location	Year	Age group	Number (in thousands)	PAF	Rate (per 100,000)
Tracheal, bronchus, and lung cancer	Bermuda	2015	Age-standardized		4.8% (0.8 to 10.2%)	1.4 (0.2 to 3.0)
Cardiovascular diseases	Bermuda	1990	All Ages	0.0 (0.0 to 0.0)	9.9% (7.1 to 12.7%)	29.9 (21.3 to 38.5)
Cardiovascular diseases	Bermuda	1995	All Ages	0.0 (0.0 to 0.0)	9.4% (6.6 to 12.1%)	25.9 (18.5 to 33.3)
Cardiovascular diseases	Bermuda	2000	All Ages	0.0 (0.0 to 0.0)	8.8% (6.2 to 11.4%)	21.4 (15.2 to 27.9)
Cardiovascular diseases	Bermuda	2005	All Ages	0.0 (0.0 to 0.0)	8.4% (5.8 to 11.2%)	18.0 (12.3 to 24.1)
Cardiovascular diseases	Bermuda	2010	All Ages	0.0 (0.0 to 0.0)	7.3% (4.7 to 10.0%)	14.8 (9.6 to 20.3)
Cardiovascular diseases	Bermuda	2015	All Ages	0.0 (0.0 to 0.0)	6.9% (3.9 to 9.6%)	14.7 (8.2 to 21.0)
Cardiovascular diseases	Bermuda	1990	Age-standardized		9.7% (7.0 to 12.7%)	43.4 (31.0 to 56.6)
Cardiovascular diseases	Bermuda	1995	Age-standardized		9.2% (6.5 to 12.0%)	35.9 (25.2 to 46.4)
Cardiovascular diseases	Bermuda	2000	Age-standardized		8.7% (6.1 to 11.2%)	28.2 (19.8 to 36.9)
Cardiovascular diseases	Bermuda	2005	Age-standardized		8.3% (5.7 to 11.2%)	22.3 (15.0 to 30.0)
Cardiovascular diseases	Bermuda	2010	Age-standardized		7.2% (4.7 to 9.9%)	16.9 (10.9 to 23.2)
Cardiovascular diseases	Bermuda	2015	Age-standardized		6.9% (3.9 to 9.6%)	15.2 (8.5 to 21.8)
Ischemic heart disease	Bermuda	1990	All Ages	0.0 (0.0 to 0.0)	12.4% (8.8 to 15.9%)	24.5 (17.3 to 31.8)
Ischemic heart disease	Bermuda	1995	All Ages	0.0 (0.0 to 0.0)	11.9% (8.2 to 15.7%)	20.8 (14.3 to 27.4)
Ischemic heart disease	Bermuda	2000	All Ages	0.0 (0.0 to 0.0)	11.5% (7.9 to 15.1%)	17.0 (11.7 to 22.6)
Ischemic heart disease	Bermuda	2005	All Ages	0.0 (0.0 to 0.0)	11.5% (7.7 to 15.4%)	13.9 (9.3 to 18.8)
Ischemic heart disease	Bermuda	2010	All Ages	0.0 (0.0 to 0.0)	10.2% (6.3 to 14.0%)	11.4 (7.1 to 16.0)
Ischemic heart disease	Bermuda	2015	All Ages	0.0 (0.0 to 0.0)	9.6% (5.3 to 13.6%)	11.5 (6.4 to 16.5)
Ischemic heart disease	Bermuda	1990	Age-standardized		12.1% (8.5 to 15.8%)	35.6 (24.7 to 46.4)
Ischemic heart disease	Bermuda	1995	Age-standardized		11.7% (8.0 to 15.5%)	28.8 (19.5 to 38.2)
Ischemic heart disease	Bermuda	2000	Age-standardized		11.4% (7.7 to 15.0%)	22.4 (15.2 to 29.9)
Ischemic heart disease	Bermuda	2005	Age-standardized		11.3% (7.5 to 15.3%)	17.2 (11.3 to 23.4)
Ischemic heart disease	Bermuda	2010	Age-standardized		10.1% (6.1 to 13.9%)	13.0 (8.0 to 18.3)
Ischemic heart disease	Bermuda	2015	Age-standardized		9.6% (5.3 to 13.5%)	11.9 (6.5 to 17.1)
Ischemic stroke	Bermuda	1990	All Ages	0.0 (0.0 to 0.0)	8.1% (4.9 to 12.0%)	2.9 (1.7 to 4.3)
Ischemic stroke	Bermuda	1995	All Ages	0.0 (0.0 to 0.0)	7.7% (4.3 to 11.6%)	2.7 (1.5 to 4.0)
Ischemic stroke	Bermuda	2000	All Ages	0.0 (0.0 to 0.0)	7.5% (3.9 to 11.7%)	2.2 (1.1 to 3.4)
Ischemic stroke	Bermuda	2005	All Ages	0.0 (0.0 to 0.0)	7.5% (3.9 to 11.8%)	2.0 (1.0 to 3.2)
Ischemic stroke	Bermuda	2010	All Ages	0.0 (0.0 to 0.0)	6.5% (2.8 to 10.8%)	1.7 (0.7 to 2.8)
Ischemic stroke	Bermuda	2015	All Ages	0.0 (0.0 to 0.0)	6.1% (2.4 to 10.9%)	1.6 (0.6 to 2.8)
Ischemic stroke	Bermuda	1990	Age-standardized		7.9% (4.7 to 12.0%)	4.3 (2.5 to 6.6)
Ischemic stroke	Bermuda	1995	Age-standardized		7.6% (4.3 to 11.7%)	3.8 (2.1 to 5.8)
Ischemic stroke	Bermuda	2000	Age-standardized		7.4% (3.9 to 11.7%)	2.9 (1.5 to 4.6)
Ischemic stroke	Bermuda	2005	Age-standardized		7.5% (3.9 to 11.8%)	2.5 (1.3 to 4.1)
Ischemic stroke	Bermuda	2010	Age-standardized		6.5% (2.8 to 10.8%)	1.9 (0.8 to 3.3)

Cause name	Location	Year	Age group	Number (in thousands)	PAF	Rate (per 100,000)
Ischemic stroke	Bermuda	2015	Age-standardized		6.1% (2.4 to 10.8%)	1.7 (0.6 to 2.9)
Hemorrhagic stroke	Bermuda	1990	All Ages	0.0 (0.0 to 0.0)	9.0% (5.9 to 12.7%)	2.5 (1.6 to 3.5)
Hemorrhagic stroke	Bermuda	1995	All Ages	0.0 (0.0 to 0.0)	8.6% (5.3 to 11.9%)	2.4 (1.5 to 3.3)
Hemorrhagic stroke	Bermuda	2000	All Ages	0.0 (0.0 to 0.0)	8.3% (4.9 to 11.9%)	2.2 (1.3 to 3.2)
Hemorrhagic stroke	Bermuda	2005	All Ages	0.0 (0.0 to 0.0)	8.2% (4.8 to 11.7%)	2.1 (1.2 to 3.0)
Hemorrhagic stroke	Bermuda	2010	All Ages	0.0 (0.0 to 0.0)	7.1% (3.7 to 10.9%)	1.7 (0.9 to 2.6)
Hemorrhagic stroke	Bermuda	2015	All Ages	0.0 (0.0 to 0.0)	6.7% (3.2 to 10.5%)	1.6 (0.8 to 2.6)
Hemorrhagic stroke	Bermuda	1990	Age-standardized		8.9% (5.7 to 12.6%)	3.5 (2.3 to 5.0)
Hemorrhagic stroke	Bermuda	1995	Age-standardized		8.4% (5.2 to 11.8%)	3.2 (2.0 to 4.6)
Hemorrhagic stroke	Bermuda	2000	Age-standardized		8.2% (4.8 to 11.8%)	2.9 (1.7 to 4.2)
Hemorrhagic stroke	Bermuda	2005	Age-standardized		8.1% (4.7 to 11.7%)	2.6 (1.5 to 3.7)
Hemorrhagic stroke	Bermuda	2010	Age-standardized		7.1% (3.6 to 10.9%)	2.0 (1.0 to 3.0)
Hemorrhagic stroke	Bermuda	2015	Age-standardized		6.7% (3.1 to 10.5%)	1.7 (0.8 to 2.6)
Chronic obstructive pulmonary disease	Bermuda	1990	All Ages	0.0 (0.0 to 0.0)	12.3% (3.7 to 22.9%)	1.4 (0.4 to 2.7)
Chronic obstructive pulmonary disease	Bermuda	1995	All Ages	0.0 (0.0 to 0.0)	11.9% (3.6 to 22.4%)	1.3 (0.4 to 2.5)
Chronic obstructive pulmonary disease	Bermuda	2000	All Ages	0.0 (0.0 to 0.0)	11.9% (3.2 to 22.9%)	1.3 (0.3 to 2.5)
Chronic obstructive pulmonary disease	Bermuda	2005	All Ages	0.0 (0.0 to 0.0)	12.1% (3.7 to 22.5%)	1.2 (0.4 to 2.3)
Chronic obstructive pulmonary disease	Bermuda	2010	All Ages	0.0 (0.0 to 0.0)	10.1% (2.3 to 20.6%)	0.9 (0.2 to 1.9)
Chronic obstructive pulmonary disease	Bermuda	2015	All Ages	0.0 (0.0 to 0.0)	9.4% (1.3 to 20.0%)	0.9 (0.1 to 1.9)
Chronic obstructive pulmonary disease	Bermuda	1990	Age-standardized		12.4% (3.7 to 23.1%)	2.1 (0.6 to 4.0)
Chronic obstructive pulmonary disease	Bermuda	1995	Age-standardized		12.0% (3.6 to 22.6%)	1.9 (0.6 to 3.5)
Chronic obstructive pulmonary disease	Bermuda	2000	Age-standardized		11.9% (3.2 to 23.0%)	1.7 (0.5 to 3.3)
Chronic obstructive pulmonary disease	Bermuda	2005	Age-standardized		12.1% (3.8 to 22.6%)	1.5 (0.5 to 2.8)
Chronic obstructive pulmonary disease	Bermuda	2010	Age-standardized		10.1% (2.3 to 20.6%)	1.1 (0.2 to 2.2)
Chronic obstructive pulmonary disease	Bermuda	2015	Age-standardized		9.4% (1.3 to 20.0%)	0.9 (0.1 to 2.0)
All causes	Cuba	1990	All Ages	5.4 (4.5 to 6.2)	7.3% (6.1 to 8.5%)	50.8 (42.7 to 59.0)
All causes	Cuba	1995	All Ages	5.8 (4.8 to 6.9)	7.2% (5.9 to 8.5%)	53.4 (44.2 to 63.0)
All causes	Cuba	2000	All Ages	6.1 (5.0 to 7.2)	7.2% (6.0 to 8.6%)	54.6 (44.9 to 64.5)
All causes	Cuba	2005	All Ages	6.0 (5.0 to 7.2)	6.9% (5.7 to 8.2%)	53.5 (44.1 to 64.0)
All causes	Cuba	2010	All Ages	5.4 (4.4 to 6.7)	5.6% (4.5 to 6.9%)	48.0 (38.5 to 59.1)
All causes	Cuba	2015	All Ages	6.6 (5.3 to 8.0)	6.1% (4.9 to 7.3%)	57.6 (46.3 to 69.8)
All causes	Cuba	1990	Age-standardized		7.4% (6.3 to 8.6%)	57.4 (48.4 to 66.4)
All causes	Cuba	1995	Age-standardized		7.3% (6.0 to 8.5%)	56.7 (47.0 to 66.5)
All causes	Cuba	2000	Age-standardized		7.3% (6.0 to 8.5%)	53.7 (44.5 to 63.2)
All causes	Cuba	2005	Age-standardized		6.9% (5.7 to 8.2%)	48.3 (40.0 to 57.8)
All causes	Cuba	2010	Age-standardized		5.6% (4.5 to 6.8%)	37.6 (30.4 to 46.0)

Cause name	Location	Year	Age group	Number (in thousands)	PAF	Rate (per 100,000)
All causes	Cuba	2015	Age-standardized		6.0% (4.8 to 7.1%)	39.4 (31.9 to 47.5)
Lower respiratory infections	Cuba	1990	All Ages	0.7 (0.4 to 1.1)	18.3% (10.5 to 28.1%)	6.9 (4.0 to 10.7)
Lower respiratory infections	Cuba	1995	All Ages	0.8 (0.5 to 1.3)	17.7% (9.5 to 27.4%)	7.7 (4.1 to 12.1)
Lower respiratory infections	Cuba	2000	All Ages	1.0 (0.5 to 1.6)	17.6% (9.3 to 27.6%)	9.2 (4.9 to 14.3)
Lower respiratory infections	Cuba	2005	All Ages	1.1 (0.5 to 1.7)	16.9% (8.8 to 27.1%)	9.3 (4.8 to 14.9)
Lower respiratory infections	Cuba	2010	All Ages	0.8 (0.4 to 1.5)	13.2% (5.5 to 23.7%)	7.4 (3.1 to 13.4)
Lower respiratory infections	Cuba	2015	All Ages	1.1 (0.5 to 1.8)	15.1% (7.1 to 25.2%)	9.3 (4.3 to 15.6)
Lower respiratory infections	Cuba	1990	Age-standardized		18.3% (10.5 to 28.1%)	7.7 (4.4 to 11.9)
Lower respiratory infections	Cuba	1995	Age-standardized		17.7% (9.5 to 27.4%)	8.0 (4.3 to 12.6)
Lower respiratory infections	Cuba	2000	Age-standardized		17.6% (9.3 to 27.6%)	9.0 (4.7 to 13.9)
Lower respiratory infections	Cuba	2005	Age-standardized		16.9% (8.8 to 27.1%)	8.4 (4.3 to 13.4)
Lower respiratory infections	Cuba	2010	Age-standardized		13.2% (5.5 to 23.7%)	5.8 (2.4 to 10.4)
Lower respiratory infections	Cuba	2015	Age-standardized		15.1% (7.1 to 25.2%)	6.2 (2.9 to 10.4)
Tracheal, bronchus, and lung cancer	Cuba	1990	All Ages	0.4 (0.2 to 0.6)	12.9% (7.1 to 19.5%)	4.0 (2.2 to 6.0)
Tracheal, bronchus, and lung cancer	Cuba	1995	All Ages	0.4 (0.2 to 0.7)	12.4% (6.8 to 18.7%)	3.9 (2.1 to 5.9)
Tracheal, bronchus, and lung cancer	Cuba	2000	All Ages	0.5 (0.3 to 0.7)	12.3% (6.8 to 18.8%)	4.4 (2.4 to 6.7)
Tracheal, bronchus, and lung cancer	Cuba	2005	All Ages	0.6 (0.3 to 0.8)	11.8% (6.6 to 18.3%)	4.8 (2.7 to 7.4)
Tracheal, bronchus, and lung cancer	Cuba	2010	All Ages	0.5 (0.3 to 0.8)	9.1% (4.6 to 14.6%)	4.3 (2.2 to 6.9)
Tracheal, bronchus, and lung cancer	Cuba	2015	All Ages	0.7 (0.3 to 1.0)	10.5% (5.5 to 16.5%)	5.7 (3.0 to 8.8)
Tracheal, bronchus, and lung cancer	Cuba	1990	Age-standardized		12.9% (7.2 to 19.5%)	4.5 (2.5 to 6.8)
Tracheal, bronchus, and lung cancer	Cuba	1995	Age-standardized		12.4% (6.8 to 18.7%)	4.3 (2.3 to 6.5)
Tracheal, bronchus, and lung cancer	Cuba	2000	Age-standardized		12.3% (6.8 to 18.8%)	4.4 (2.4 to 6.7)
Tracheal, bronchus, and lung cancer	Cuba	2005	Age-standardized		11.8% (6.6 to 18.3%)	4.4 (2.5 to 6.8)
Tracheal, bronchus, and lung cancer	Cuba	2010	Age-standardized		9.1% (4.6 to 14.6%)	3.5 (1.8 to 5.5)
Tracheal, bronchus, and lung cancer	Cuba	2015	Age-standardized		10.5% (5.5 to 16.5%)	4.0 (2.1 to 6.2)
Cardiovascular diseases	Cuba	1990	All Ages	3.9 (3.2 to 4.7)	12.7% (10.4 to 15.2%)	37.0 (30.1 to 44.4)
Cardiovascular diseases	Cuba	1995	All Ages	4.2 (3.3 to 5.0)	12.2% (9.8 to 14.7%)	38.2 (30.5 to 46.0)
Cardiovascular diseases	Cuba	2000	All Ages	4.1 (3.3 to 4.9)	11.7% (9.3 to 14.1%)	36.5 (29.2 to 44.3)
Cardiovascular diseases	Cuba	2005	All Ages	3.9 (3.1 to 4.7)	11.0% (8.7 to 13.3%)	34.3 (27.1 to 41.8)
Cardiovascular diseases	Cuba	2010	All Ages	3.6 (2.7 to 4.5)	9.4% (7.3 to 11.7%)	31.5 (24.2 to 39.3)
Cardiovascular diseases	Cuba	2015	All Ages	4.2 (3.2 to 5.2)	9.9% (7.6 to 12.2%)	36.7 (28.1 to 45.6)
Cardiovascular diseases	Cuba	1990	Age-standardized		12.8% (10.5 to 15.3%)	41.9 (34.2 to 50.0)
Cardiovascular diseases	Cuba	1995	Age-standardized		12.3% (9.9 to 14.7%)	40.6 (32.7 to 48.8)
Cardiovascular diseases	Cuba	2000	Age-standardized		11.7% (9.4 to 14.1%)	35.9 (28.9 to 43.3)
Cardiovascular diseases	Cuba	2005	Age-standardized		11.0% (8.8 to 13.3%)	30.9 (24.6 to 37.5)
Cardiovascular diseases	Cuba	2010	Age-standardized		9.5% (7.5 to 11.7%)	24.6 (19.2 to 30.3)

Cause name	Location	Year	Age group	Number (in thousands)	PAF	Rate (per 100,000)
Cardiovascular diseases	Cuba	2015	Age-standardized		10.0% (7.9 to 12.2%)	25.1 (19.6 to 30.9)
Ischemic heart disease	Cuba	1990	All Ages	3.0 (2.3 to 3.8)	14.6% (11.3 to 18.0%)	28.8 (22.1 to 35.6)
Ischemic heart disease	Cuba	1995	All Ages	3.2 (2.4 to 4.0)	14.2% (10.7 to 17.7%)	29.5 (22.3 to 37.0)
Ischemic heart disease	Cuba	2000	All Ages	3.0 (2.3 to 3.8)	14.1% (10.6 to 17.6%)	27.2 (20.2 to 34.2)
Ischemic heart disease	Cuba	2005	All Ages	2.8 (2.1 to 3.6)	13.7% (10.1 to 17.4%)	25.2 (18.6 to 31.8)
Ischemic heart disease	Cuba	2010	All Ages	2.6 (1.9 to 3.4)	12.2% (8.7 to 15.7%)	23.1 (16.4 to 29.7)
Ischemic heart disease	Cuba	2015	All Ages	3.1 (2.2 to 4.0)	12.8% (9.1 to 16.4%)	27.1 (19.1 to 34.9)
Ischemic heart disease	Cuba	1990	Age-standardized		14.7% (11.4 to 18.1%)	32.6 (25.2 to 40.2)
Ischemic heart disease	Cuba	1995	Age-standardized		14.3% (10.9 to 17.7%)	31.3 (23.8 to 39.0)
Ischemic heart disease	Cuba	2000	Age-standardized		14.2% (10.8 to 17.7%)	26.8 (20.4 to 33.5)
Ischemic heart disease	Cuba	2005	Age-standardized		13.8% (10.2 to 17.3%)	22.7 (16.8 to 28.6)
Ischemic heart disease	Cuba	2010	Age-standardized		12.3% (9.0 to 15.7%)	18.1 (13.1 to 23.0)
Ischemic heart disease	Cuba	2015	Age-standardized		13.0% (9.6 to 16.4%)	18.5 (13.4 to 23.5)
Ischemic stroke	Cuba	1990	All Ages	0.5 (0.3 to 0.6)	10.7% (7.5 to 14.0%)	4.5 (3.2 to 6.0)
Ischemic stroke	Cuba	1995	All Ages	0.5 (0.4 to 0.7)	10.3% (7.1 to 14.0%)	4.8 (3.3 to 6.5)
Ischemic stroke	Cuba	2000	All Ages	0.5 (0.4 to 0.8)	10.0% (6.6 to 14.1%)	4.8 (3.1 to 6.7)
Ischemic stroke	Cuba	2005	All Ages	0.6 (0.4 to 0.8)	9.8% (6.3 to 13.9%)	5.1 (3.3 to 7.3)
Ischemic stroke	Cuba	2010	All Ages	0.6 (0.3 to 0.8)	8.5% (5.1 to 12.6%)	4.9 (2.9 to 7.4)
Ischemic stroke	Cuba	2015	All Ages	0.6 (0.4 to 0.9)	9.0% (5.5 to 13.3%)	5.6 (3.3 to 8.3)
Ischemic stroke	Cuba	1990	Age-standardized		10.8% (7.6 to 14.0%)	5.1 (3.6 to 6.7)
Ischemic stroke	Cuba	1995	Age-standardized		10.4% (7.3 to 13.9%)	5.1 (3.5 to 6.8)
Ischemic stroke	Cuba	2000	Age-standardized		10.1% (6.7 to 14.0%)	4.7 (3.1 to 6.5)
Ischemic stroke	Cuba	2005	Age-standardized		9.8% (6.4 to 13.8%)	4.6 (3.0 to 6.5)
Ischemic stroke	Cuba	2010	Age-standardized		8.5% (5.2 to 12.6%)	3.8 (2.3 to 5.7)
Ischemic stroke	Cuba	2015	Age-standardized		9.1% (5.6 to 13.2%)	3.8 (2.3 to 5.4)
Hemorrhagic stroke	Cuba	1990	All Ages	0.4 (0.3 to 0.5)	12.5% (9.8 to 15.2%)	3.7 (2.9 to 4.4)
Hemorrhagic stroke	Cuba	1995	All Ages	0.4 (0.3 to 0.5)	12.1% (9.3 to 14.9%)	3.9 (3.0 to 4.9)
Hemorrhagic stroke	Cuba	2000	All Ages	0.5 (0.4 to 0.6)	11.9% (9.2 to 14.7%)	4.5 (3.4 to 5.6)
Hemorrhagic stroke	Cuba	2005	All Ages	0.4 (0.3 to 0.6)	11.5% (8.7 to 14.5%)	3.9 (3.0 to 5.0)
Hemorrhagic stroke	Cuba	2010	All Ages	0.4 (0.3 to 0.5)	9.8% (7.1 to 12.7%)	3.4 (2.4 to 4.5)
Hemorrhagic stroke	Cuba	2015	All Ages	0.5 (0.3 to 0.6)	10.4% (7.5 to 13.5%)	4.0 (2.9 to 5.2)
Hemorrhagic stroke	Cuba	1990	Age-standardized		12.6% (9.8 to 15.3%)	4.2 (3.3 to 5.1)
Hemorrhagic stroke	Cuba	1995	Age-standardized		12.1% (9.3 to 14.9%)	4.2 (3.3 to 5.2)
Hemorrhagic stroke	Cuba	2000	Age-standardized		11.9% (9.2 to 14.7%)	4.4 (3.4 to 5.5)
Hemorrhagic stroke	Cuba	2005	Age-standardized		11.5% (8.7 to 14.5%)	3.6 (2.7 to 4.5)
Hemorrhagic stroke	Cuba	2010	Age-standardized		9.9% (7.1 to 12.7%)	2.7 (1.9 to 3.6)

Cause name	Location	Year	Age group	Number (in thousands)	PAF	Rate (per 100,000)
Hemorrhagic stroke	Cuba	2015	Age-standardized		10.5% (7.7 to 13.4%)	2.8 (2.0 to 3.6)
Chronic obstructive pulmonary disease	Cuba	1990	All Ages	0.3 (0.1 to 0.5)	19.1% (9.3 to 29.9%)	3.0 (1.5 to 4.7)
Chronic obstructive pulmonary disease	Cuba	1995	All Ages	0.4 (0.2 to 0.6)	18.6% (8.8 to 29.4%)	3.6 (1.7 to 5.6)
Chronic obstructive pulmonary disease	Cuba	2000	All Ages	0.5 (0.2 to 0.8)	18.6% (9.1 to 29.4%)	4.5 (2.2 to 7.1)
Chronic obstructive pulmonary disease	Cuba	2005	All Ages	0.6 (0.3 to 0.9)	18.1% (8.5 to 29.2%)	5.1 (2.4 to 8.1)
Chronic obstructive pulmonary disease	Cuba	2010	All Ages	0.5 (0.2 to 0.9)	15.0% (6.7 to 25.3%)	4.8 (2.1 to 8.1)
Chronic obstructive pulmonary disease	Cuba	2015	All Ages	0.7 (0.3 to 1.1)	16.5% (7.7 to 27.0%)	6.0 (2.8 to 9.8)
Chronic obstructive pulmonary disease	Cuba	1990	Age-standardized		19.1% (9.3 to 29.9%)	3.4 (1.6 to 5.3)
Chronic obstructive pulmonary disease	Cuba	1995	Age-standardized		18.6% (8.8 to 29.4%)	3.8 (1.8 to 5.9)
Chronic obstructive pulmonary disease	Cuba	2000	Age-standardized		18.6% (9.1 to 29.4%)	4.4 (2.1 to 7.0)
Chronic obstructive pulmonary disease	Cuba	2005	Age-standardized		18.1% (8.5 to 29.1%)	4.6 (2.2 to 7.3)
Chronic obstructive pulmonary disease	Cuba	2010	Age-standardized		15.0% (6.7 to 25.3%)	3.7 (1.7 to 6.3)
Chronic obstructive pulmonary disease	Cuba	2015	Age-standardized		16.5% (7.7 to 27.0%)	4.1 (1.9 to 6.6)
All causes	Dominica	1990	All Ages	0.0 (0.0 to 0.0)	4.3% (3.1 to 5.4%)	26.5 (19.2 to 34.1)
All causes	Dominica	1995	All Ages	0.0 (0.0 to 0.0)	4.2% (3.1 to 5.5%)	27.3 (19.6 to 35.5)
All causes	Dominica	2000	All Ages	0.0 (0.0 to 0.0)	4.0% (2.8 to 5.3%)	25.5 (17.8 to 34.0)
All causes	Dominica	2005	All Ages	0.0 (0.0 to 0.0)	3.8% (2.6 to 5.1%)	24.8 (17.0 to 33.3)
All causes	Dominica	2010	All Ages	0.0 (0.0 to 0.0)	3.4% (2.4 to 4.6%)	24.6 (16.9 to 32.9)
All causes	Dominica	2015	All Ages	0.0 (0.0 to 0.0)	3.3% (2.2 to 4.5%)	27.2 (18.2 to 37.9)
All causes	Dominica	1990	Age-standardized		4.6% (3.4 to 5.8%)	40.1 (29.1 to 51.3)
All causes	Dominica	1995	Age-standardized		4.5% (3.3 to 5.8%)	39.2 (28.2 to 51.1)
All causes	Dominica	2000	Age-standardized		4.2% (2.9 to 5.5%)	34.1 (23.8 to 45.8)
All causes	Dominica	2005	Age-standardized		3.9% (2.7 to 5.2%)	31.0 (21.3 to 41.8)
All causes	Dominica	2010	Age-standardized		3.4% (2.4 to 4.6%)	28.5 (19.5 to 38.3)
All causes	Dominica	2015	Age-standardized		3.3% (2.2 to 4.5%)	28.9 (19.3 to 40.3)
Lower respiratory infections	Dominica	1990	All Ages	0.0 (0.0 to 0.0)	15.0% (5.5 to 26.6%)	4.4 (1.6 to 8.0)
Lower respiratory infections	Dominica	1995	All Ages	0.0 (0.0 to 0.0)	14.9% (5.2 to 26.7%)	4.7 (1.6 to 8.7)
Lower respiratory infections	Dominica	2000	All Ages	0.0 (0.0 to 0.0)	15.0% (5.4 to 26.8%)	4.8 (1.7 to 8.8)
Lower respiratory infections	Dominica	2005	All Ages	0.0 (0.0 to 0.0)	14.1% (4.8 to 25.8%)	4.7 (1.6 to 8.8)
Lower respiratory infections	Dominica	2010	All Ages	0.0 (0.0 to 0.0)	12.1% (3.5 to 23.5%)	4.3 (1.2 to 8.6)
Lower respiratory infections	Dominica	2015	All Ages	0.0 (0.0 to 0.0)	11.6% (3.4 to 23.0%)	4.6 (1.3 to 9.3)
Lower respiratory infections	Dominica	1990	Age-standardized		15.0% (5.5 to 26.6%)	6.1 (2.2 to 11.2)
Lower respiratory infections	Dominica	1995	Age-standardized		14.9% (5.2 to 26.7%)	6.3 (2.2 to 11.7)
Lower respiratory infections	Dominica	2000	Age-standardized		15.0% (5.4 to 26.8%)	6.2 (2.2 to 11.5)
Lower respiratory infections	Dominica	2005	Age-standardized		14.1% (4.8 to 25.8%)	5.8 (1.9 to 10.9)
Lower respiratory infections	Dominica	2010	Age-standardized		12.1% (3.5 to 23.5%)	5.0 (1.4 to 10.0)

Cause name	Location	Year	Age group	Number (in thousands)	PAF	Rate (per 100,000)
Lower respiratory infections	Dominica	2015	Age-standardized		11.6% (3.4 to 23.0%)	4.9 (1.4 to 9.9)
Tracheal, bronchus, and lung cancer	Dominica	1990	All Ages	0.0 (0.0 to 0.0)	10.4% (4.3 to 17.6%)	1.0 (0.4 to 1.6)
Tracheal, bronchus, and lung cancer	Dominica	1995	All Ages	0.0 (0.0 to 0.0)	10.3% (4.6 to 18.0%)	1.1 (0.5 to 1.9)
Tracheal, bronchus, and lung cancer	Dominica	2000	All Ages	0.0 (0.0 to 0.0)	10.4% (4.6 to 18.1%)	1.1 (0.5 to 2.0)
Tracheal, bronchus, and lung cancer	Dominica	2005	All Ages	0.0 (0.0 to 0.0)	9.7% (4.1 to 16.8%)	1.1 (0.5 to 1.9)
Tracheal, bronchus, and lung cancer	Dominica	2010	All Ages	0.0 (0.0 to 0.0)	8.3% (3.3 to 15.0%)	1.1 (0.4 to 2.0)
Tracheal, bronchus, and lung cancer	Dominica	2015	All Ages	0.0 (0.0 to 0.0)	8.0% (3.2 to 14.7%)	1.2 (0.5 to 2.3)
Tracheal, bronchus, and lung cancer	Dominica	1990	Age-standardized		10.4% (4.3 to 17.6%)	1.4 (0.6 to 2.4)
Tracheal, bronchus, and lung cancer	Dominica	1995	Age-standardized		10.4% (4.6 to 18.1%)	1.5 (0.7 to 2.7)
Tracheal, bronchus, and lung cancer	Dominica	2000	Age-standardized		10.4% (4.6 to 18.1%)	1.5 (0.7 to 2.6)
Tracheal, bronchus, and lung cancer	Dominica	2005	Age-standardized		9.7% (4.1 to 16.8%)	1.4 (0.6 to 2.4)
Tracheal, bronchus, and lung cancer	Dominica	2010	Age-standardized		8.3% (3.3 to 15.1%)	1.2 (0.5 to 2.3)
Tracheal, bronchus, and lung cancer	Dominica	2015	Age-standardized		8.0% (3.2 to 14.7%)	1.3 (0.5 to 2.4)
Cardiovascular diseases	Dominica	1990	All Ages	0.0 (0.0 to 0.0)	8.9% (6.8 to 11.0%)	19.3 (14.5 to 24.1)
Cardiovascular diseases	Dominica	1995	All Ages	0.0 (0.0 to 0.0)	9.0% (6.7 to 11.2%)	19.6 (14.6 to 24.6)
Cardiovascular diseases	Dominica	2000	All Ages	0.0 (0.0 to 0.0)	8.8% (6.4 to 11.2%)	17.6 (12.7 to 22.6)
Cardiovascular diseases	Dominica	2005	All Ages	0.0 (0.0 to 0.0)	8.3% (6.1 to 10.7%)	17.0 (12.3 to 21.7)
Cardiovascular diseases	Dominica	2010	All Ages	0.0 (0.0 to 0.0)	7.6% (5.4 to 9.9%)	17.2 (12.3 to 22.9)
Cardiovascular diseases	Dominica	2015	All Ages	0.0 (0.0 to 0.0)	7.3% (5.0 to 9.5%)	19.2 (13.0 to 25.2)
Cardiovascular diseases	Dominica	1990	Age-standardized		8.8% (6.7 to 11.0%)	29.6 (22.2 to 37.2)
Cardiovascular diseases	Dominica	1995	Age-standardized		8.9% (6.7 to 11.1%)	28.5 (21.1 to 36.0)
Cardiovascular diseases	Dominica	2000	Age-standardized		8.7% (6.3 to 11.1%)	23.7 (17.0 to 30.8)
Cardiovascular diseases	Dominica	2005	Age-standardized		8.3% (6.0 to 10.6%)	21.4 (15.3 to 27.4)
Cardiovascular diseases	Dominica	2010	Age-standardized		7.5% (5.3 to 9.8%)	20.0 (14.2 to 26.5)
Cardiovascular diseases	Dominica	2015	Age-standardized		7.2% (5.0 to 9.4%)	20.4 (13.8 to 26.8)
Ischemic heart disease	Dominica	1990	All Ages	0.0 (0.0 to 0.0)	13.7% (10.1 to 17.4%)	13.5 (9.6 to 17.2)
Ischemic heart disease	Dominica	1995	All Ages	0.0 (0.0 to 0.0)	13.6% (10.0 to 17.2%)	13.9 (10.1 to 17.7)
Ischemic heart disease	Dominica	2000	All Ages	0.0 (0.0 to 0.0)	13.6% (9.7 to 17.6%)	12.4 (8.5 to 16.3)
Ischemic heart disease	Dominica	2005	All Ages	0.0 (0.0 to 0.0)	13.2% (9.3 to 17.2%)	11.4 (8.0 to 15.0)
Ischemic heart disease	Dominica	2010	All Ages	0.0 (0.0 to 0.0)	12.4% (8.7 to 16.1%)	11.4 (8.0 to 15.1)
Ischemic heart disease	Dominica	2015	All Ages	0.0 (0.0 to 0.0)	12.1% (8.1 to 16.0%)	12.7 (8.4 to 17.0)
Ischemic heart disease	Dominica	1990	Age-standardized		13.5% (9.8 to 17.1%)	20.7 (14.5 to 26.7)
Ischemic heart disease	Dominica	1995	Age-standardized		13.4% (9.7 to 17.0%)	20.2 (14.6 to 26.1)
Ischemic heart disease	Dominica	2000	Age-standardized		13.3% (9.4 to 17.4%)	16.7 (11.4 to 22.1)
Ischemic heart disease	Dominica	2005	Age-standardized		13.0% (9.1 to 17.1%)	14.4 (10.0 to 18.9)
Ischemic heart disease	Dominica	2010	Age-standardized		12.2% (8.6 to 15.9%)	13.2 (9.2 to 17.6)

Cause name	Location	Year	Age group	Number (in thousands)	PAF	Rate (per 100,000)
Ischemic heart disease	Dominica	2015	Age-standardized		12.0% (8.0 to 15.9%)	13.4 (8.9 to 18.0)
Ischemic stroke	Dominica	1990	All Ages	0.0 (0.0 to 0.0)	9.7% (6.2 to 13.5%)	3.2 (2.0 to 4.5)
Ischemic stroke	Dominica	1995	All Ages	0.0 (0.0 to 0.0)	9.6% (5.8 to 13.5%)	3.0 (1.9 to 4.3)
Ischemic stroke	Dominica	2000	All Ages	0.0 (0.0 to 0.0)	9.5% (6.0 to 13.9%)	2.6 (1.6 to 3.8)
Ischemic stroke	Dominica	2005	All Ages	0.0 (0.0 to 0.0)	9.1% (5.3 to 13.3%)	2.7 (1.5 to 3.9)
Ischemic stroke	Dominica	2010	All Ages	0.0 (0.0 to 0.0)	8.3% (4.9 to 12.5%)	2.8 (1.6 to 4.1)
Ischemic stroke	Dominica	2015	All Ages	0.0 (0.0 to 0.0)	8.1% (4.3 to 12.5%)	3.1 (1.6 to 4.8)
Ischemic stroke	Dominica	1990	Age-standardized		9.6% (6.0 to 13.5%)	4.9 (3.1 to 7.1)
Ischemic stroke	Dominica	1995	Age-standardized		9.5% (5.7 to 13.5%)	4.5 (2.8 to 6.4)
Ischemic stroke	Dominica	2000	Age-standardized		9.4% (5.9 to 13.9%)	3.6 (2.2 to 5.2)
Ischemic stroke	Dominica	2005	Age-standardized		9.0% (5.2 to 13.2%)	3.4 (2.0 to 5.1)
Ischemic stroke	Dominica	2010	Age-standardized		8.3% (4.9 to 12.5%)	3.3 (1.9 to 4.9)
Ischemic stroke	Dominica	2015	Age-standardized		8.1% (4.3 to 12.4%)	3.3 (1.7 to 5.1)
Hemorrhagic stroke	Dominica	1990	All Ages	0.0 (0.0 to 0.0)	10.8% (7.6 to 14.2%)	2.6 (1.8 to 3.5)
Hemorrhagic stroke	Dominica	1995	All Ages	0.0 (0.0 to 0.0)	10.7% (7.3 to 14.2%)	2.7 (1.8 to 3.6)
Hemorrhagic stroke	Dominica	2000	All Ages	0.0 (0.0 to 0.0)	10.7% (7.4 to 14.7%)	2.6 (1.8 to 3.7)
Hemorrhagic stroke	Dominica	2005	All Ages	0.0 (0.0 to 0.0)	10.1% (6.5 to 13.5%)	2.9 (1.8 to 3.9)
Hemorrhagic stroke	Dominica	2010	All Ages	0.0 (0.0 to 0.0)	9.2% (5.9 to 13.0%)	3.1 (2.0 to 4.3)
Hemorrhagic stroke	Dominica	2015	All Ages	0.0 (0.0 to 0.0)	9.0% (5.6 to 12.7%)	3.4 (2.1 to 4.9)
Hemorrhagic stroke	Dominica	1990	Age-standardized		10.7% (7.4 to 14.2%)	3.9 (2.7 to 5.2)
Hemorrhagic stroke	Dominica	1995	Age-standardized		10.6% (7.1 to 14.2%)	3.8 (2.5 to 5.1)
Hemorrhagic stroke	Dominica	2000	Age-standardized		10.6% (7.2 to 14.6%)	3.5 (2.4 to 4.9)
Hemorrhagic stroke	Dominica	2005	Age-standardized		10.0% (6.4 to 13.5%)	3.6 (2.3 to 4.8)
Hemorrhagic stroke	Dominica	2010	Age-standardized		9.1% (5.8 to 12.9%)	3.5 (2.2 to 5.0)
Hemorrhagic stroke	Dominica	2015	Age-standardized		8.9% (5.4 to 12.6%)	3.6 (2.2 to 5.2)
Chronic obstructive pulmonary disease	Dominica	1990	All Ages	0.0 (0.0 to 0.0)	16.1% (6.2 to 27.2%)	1.9 (0.7 to 3.3)
Chronic obstructive pulmonary disease	Dominica	1995	All Ages	0.0 (0.0 to 0.0)	16.1% (6.6 to 27.0%)	2.0 (0.8 to 3.3)
Chronic obstructive pulmonary disease	Dominica	2000	All Ages	0.0 (0.0 to 0.0)	16.2% (6.7 to 27.5%)	2.0 (0.8 to 3.3)
Chronic obstructive pulmonary disease	Dominica	2005	All Ages	0.0 (0.0 to 0.0)	15.5% (5.8 to 27.0%)	2.0 (0.7 to 3.5)
Chronic obstructive pulmonary disease	Dominica	2010	All Ages	0.0 (0.0 to 0.0)	13.8% (5.3 to 24.3%)	2.0 (0.7 to 3.5)
Chronic obstructive pulmonary disease	Dominica	2015	All Ages	0.0 (0.0 to 0.0)	13.4% (4.8 to 23.7%)	2.2 (0.8 to 4.0)
Chronic obstructive pulmonary disease	Dominica	1990	Age-standardized		16.3% (6.3 to 27.6%)	3.0 (1.2 to 5.1)
Chronic obstructive pulmonary disease	Dominica	1995	Age-standardized		16.2% (6.7 to 27.3%)	2.9 (1.2 to 5.0)
Chronic obstructive pulmonary disease	Dominica	2000	Age-standardized		16.3% (6.7 to 27.7%)	2.7 (1.1 to 4.6)
Chronic obstructive pulmonary disease	Dominica	2005	Age-standardized		15.5% (5.8 to 27.1%)	2.5 (0.9 to 4.5)
Chronic obstructive pulmonary disease	Dominica	2010	Age-standardized		13.8% (5.3 to 24.3%)	2.3 (0.8 to 4.1)

Cause name	Location	Year	Age group	Number (in thousands)	PAF	Rate (per 100,000)
Chronic obstructive pulmonary disease	Dominica	2015	Age-standardized		13.4% (4.8 to 23.7%)	2.3 (0.8 to 4.3)
All causes	Dominican Republic	1990	All Ages	1.4 (1.2 to 1.6)	3.9% (3.3 to 4.5%)	19.3 (16.2 to 22.7)
All causes	Dominican Republic	1995	All Ages	1.7 (1.4 to 2.0)	4.4% (3.6 to 5.1%)	21.7 (18.2 to 25.6)
All causes	Dominican Republic	2000	All Ages	2.3 (1.9 to 2.7)	5.2% (4.4 to 6.1%)	26.6 (22.3 to 31.3)
All causes	Dominican Republic	2005	All Ages	2.4 (2.0 to 2.9)	4.8% (3.9 to 5.6%)	26.3 (21.7 to 31.1)
All causes	Dominican Republic	2010	All Ages	2.9 (2.4 to 3.4)	5.2% (4.3 to 6.2%)	29.0 (23.9 to 34.7)
All causes	Dominican Republic	2015	All Ages	3.3 (2.7 to 4.0)	5.5% (4.5 to 6.5%)	31.6 (26.0 to 37.7)
All causes	Dominican Republic	1990	Age-standardized		5.2% (4.3 to 6.0%)	41.2 (34.4 to 48.2)
All causes	Dominican Republic	1995	Age-standardized		5.3% (4.4 to 6.2%)	43.1 (35.5 to 50.8)
All causes	Dominican Republic	2000	Age-standardized		6.1% (5.0 to 7.1%)	48.4 (40.2 to 57.2)
All causes	Dominican Republic	2005	Age-standardized		5.4% (4.4 to 6.4%)	43.5 (35.4 to 51.7)
All causes	Dominican Republic	2010	Age-standardized		5.6% (4.6 to 6.8%)	43.8 (35.7 to 52.5)
All causes	Dominican Republic	2015	Age-standardized		5.8% (4.8 to 6.9%)	43.3 (35.3 to 51.7)
Lower respiratory infections	Dominican Republic	1990	All Ages	0.3 (0.1 to 0.5)	17.0% (8.8 to 26.9%)	4.0 (2.1 to 6.5)
Lower respiratory infections	Dominican Republic	1995	All Ages	0.3 (0.2 to 0.5)	17.1% (9.0 to 27.0%)	3.8 (2.0 to 6.0)
Lower respiratory infections	Dominican Republic	2000	All Ages	0.4 (0.3 to 0.6)	21.9% (13.3 to 31.2%)	4.8 (2.9 to 7.0)
Lower respiratory infections	Dominican Republic	2005	All Ages	0.4 (0.2 to 0.6)	17.3% (9.3 to 27.2%)	4.2 (2.2 to 6.8)
Lower respiratory infections	Dominican Republic	2010	All Ages	0.4 (0.2 to 0.7)	17.0% (8.7 to 26.8%)	4.2 (2.2 to 6.9)
Lower respiratory infections	Dominican Republic	2015	All Ages	0.4 (0.2 to 0.7)	16.6% (8.3 to 26.6%)	4.0 (2.0 to 6.8)
Lower respiratory infections	Dominican Republic	1990	Age-standardized		17.0% (8.8 to 26.9%)	4.9 (2.5 to 8.0)
Lower respiratory infections	Dominican Republic	1995	Age-standardized		17.1% (9.0 to 27.0%)	5.1 (2.7 to 8.1)
Lower respiratory infections	Dominican Republic	2000	Age-standardized		21.9% (13.3 to 31.2%)	6.9 (4.0 to 9.9)
Lower respiratory infections	Dominican Republic	2005	Age-standardized		17.3% (9.3 to 27.2%)	5.7 (3.0 to 9.2)
Lower respiratory infections	Dominican Republic	2010	Age-standardized		17.0% (8.7 to 26.8%)	5.6 (2.9 to 9.2)
Lower respiratory infections	Dominican Republic	2015	Age-standardized		16.6% (8.3 to 26.6%)	5.1 (2.5 to 8.6)
Tracheal, bronchus, and lung cancer	Dominican Republic	1990	All Ages	0.0 (0.0 to 0.1)	11.8% (6.4 to 17.9%)	0.6 (0.3 to 0.9)
Tracheal, bronchus, and lung cancer	Dominican Republic	1995	All Ages	0.1 (0.0 to 0.1)	11.9% (6.4 to 18.1%)	0.8 (0.4 to 1.2)
Tracheal, bronchus, and lung cancer	Dominican Republic	2000	All Ages	0.1 (0.1 to 0.1)	15.5% (8.9 to 23.2%)	1.1 (0.6 to 1.7)
Tracheal, bronchus, and lung cancer	Dominican Republic	2005	All Ages	0.1 (0.1 to 0.1)	12.1% (6.6 to 18.6%)	1.0 (0.5 to 1.5)
Tracheal, bronchus, and lung cancer	Dominican Republic	2010	All Ages	0.1 (0.1 to 0.2)	11.9% (6.4 to 18.3%)	1.1 (0.6 to 1.6)
Tracheal, bronchus, and lung cancer	Dominican Republic	2015	All Ages	0.1 (0.1 to 0.2)	11.6% (6.2 to 18.1%)	1.2 (0.6 to 1.8)
Tracheal, bronchus, and lung cancer	Dominican Republic	1990	Age-standardized		11.8% (6.4 to 18.0%)	1.4 (0.8 to 2.1)
Tracheal, bronchus, and lung cancer	Dominican Republic	1995	Age-standardized		11.9% (6.5 to 18.1%)	1.6 (0.9 to 2.4)
Tracheal, bronchus, and lung cancer	Dominican Republic	2000	Age-standardized		15.6% (9.0 to 23.2%)	2.1 (1.2 to 3.1)
Tracheal, bronchus, and lung cancer	Dominican Republic	2005	Age-standardized		12.1% (6.6 to 18.6%)	1.6 (0.9 to 2.5)
Tracheal, bronchus, and lung cancer	Dominican Republic	2010	Age-standardized		11.9% (6.4 to 18.3%)	1.6 (0.9 to 2.6)

Cause name	Location	Year	Age group	Number (in thousands)	PAF	Rate (per 100,000)
Tracheal, bronchus, and lung cancer	Dominican Republic	2015	Age-standardized		11.6% (6.2 to 18.2%)	1.6 (0.9 to 2.6)
Cardiovascular diseases	Dominican Republic	1990	All Ages	1.0 (0.8 to 1.1)	11.6% (9.8 to 13.3%)	13.7 (11.6 to 15.9)
Cardiovascular diseases	Dominican Republic	1995	All Ages	1.3 (1.0 to 1.5)	11.4% (9.7 to 13.3%)	16.0 (13.2 to 18.7)
Cardiovascular diseases	Dominican Republic	2000	All Ages	1.6 (1.3 to 1.9)	12.6% (10.5 to 14.8%)	19.0 (15.6 to 22.4)
Cardiovascular diseases	Dominican Republic	2005	All Ages	1.8 (1.5 to 2.1)	11.3% (9.3 to 13.5%)	19.4 (15.9 to 23.2)
Cardiovascular diseases	Dominican Republic	2010	All Ages	2.2 (1.7 to 2.6)	11.1% (9.0 to 13.2%)	21.9 (17.6 to 26.3)
Cardiovascular diseases	Dominican Republic	2015	All Ages	2.6 (2.1 to 3.1)	11.0% (8.8 to 13.2%)	24.4 (19.6 to 29.5)
Cardiovascular diseases	Dominican Republic	1990	Age-standardized		11.0% (9.0 to 12.9%)	32.0 (26.2 to 38.0)
Cardiovascular diseases	Dominican Republic	1995	Age-standardized		10.9% (8.9 to 13.0%)	33.5 (27.2 to 40.1)
Cardiovascular diseases	Dominican Republic	2000	Age-standardized		12.1% (9.9 to 14.5%)	36.1 (28.8 to 43.5)
Cardiovascular diseases	Dominican Republic	2005	Age-standardized		11.0% (8.9 to 13.3%)	33.1 (26.7 to 40.0)
Cardiovascular diseases	Dominican Republic	2010	Age-standardized		10.8% (8.6 to 13.1%)	33.6 (26.9 to 40.6)
Cardiovascular diseases	Dominican Republic	2015	Age-standardized		10.8% (8.7 to 13.1%)	33.7 (26.9 to 40.9)
Ischemic heart disease	Dominican Republic	1990	All Ages	0.7 (0.6 to 0.8)	15.6% (12.8 to 18.5%)	9.5 (7.7 to 11.3)
Ischemic heart disease	Dominican Republic	1995	All Ages	0.9 (0.7 to 1.1)	15.2% (12.1 to 18.3%)	11.1 (8.8 to 13.4)
Ischemic heart disease	Dominican Republic	2000	All Ages	1.1 (0.9 to 1.4)	16.4% (13.0 to 19.7%)	12.9 (10.1 to 15.9)
Ischemic heart disease	Dominican Republic	2005	All Ages	1.2 (1.0 to 1.5)	14.6% (11.4 to 17.9%)	13.4 (10.5 to 16.7)
Ischemic heart disease	Dominican Republic	2010	All Ages	1.5 (1.2 to 1.9)	14.2% (10.8 to 17.7%)	15.5 (11.8 to 19.3)
Ischemic heart disease	Dominican Republic	2015	All Ages	1.8 (1.4 to 2.3)	14.0% (10.7 to 17.4%)	17.4 (13.2 to 21.8)
Ischemic heart disease	Dominican Republic	1990	Age-standardized		14.4% (11.0 to 17.6%)	22.4 (17.2 to 27.4)
Ischemic heart disease	Dominican Republic	1995	Age-standardized		14.3% (10.9 to 17.5%)	23.2 (17.6 to 28.9)
Ischemic heart disease	Dominican Republic	2000	Age-standardized		15.6% (12.0 to 19.1%)	24.5 (18.4 to 30.6)
Ischemic heart disease	Dominican Republic	2005	Age-standardized		14.2% (10.8 to 17.7%)	22.8 (17.2 to 28.7)
Ischemic heart disease	Dominican Republic	2010	Age-standardized		13.8% (10.4 to 17.4%)	23.6 (17.7 to 30.0)
Ischemic heart disease	Dominican Republic	2015	Age-standardized		13.8% (10.5 to 17.2%)	24.0 (18.0 to 30.2)
Ischemic stroke	Dominican Republic	1990	All Ages	0.1 (0.1 to 0.2)	11.1% (8.3 to 14.1%)	2.1 (1.6 to 2.7)
Ischemic stroke	Dominican Republic	1995	All Ages	0.2 (0.1 to 0.2)	10.8% (7.8 to 13.8%)	2.4 (1.7 to 3.0)
Ischemic stroke	Dominican Republic	2000	All Ages	0.2 (0.2 to 0.3)	11.9% (8.6 to 15.4%)	2.8 (2.0 to 3.7)
Ischemic stroke	Dominican Republic	2005	All Ages	0.3 (0.2 to 0.3)	10.3% (6.9 to 13.9%)	2.7 (1.8 to 3.6)
Ischemic stroke	Dominican Republic	2010	All Ages	0.3 (0.2 to 0.4)	9.9% (6.7 to 13.5%)	2.9 (1.9 to 4.0)
Ischemic stroke	Dominican Republic	2015	All Ages	0.3 (0.2 to 0.5)	9.8% (6.4 to 13.6%)	3.1 (2.0 to 4.5)
Ischemic stroke	Dominican Republic	1990	Age-standardized		10.4% (7.3 to 13.8%)	5.4 (3.7 to 7.1)
Ischemic stroke	Dominican Republic	1995	Age-standardized		10.3% (7.1 to 13.8%)	5.3 (3.7 to 7.2)
Ischemic stroke	Dominican Republic	2000	Age-standardized		11.5% (8.1 to 15.4%)	5.6 (3.8 to 7.6)
Ischemic stroke	Dominican Republic	2005	Age-standardized		10.1% (6.6 to 13.9%)	4.8 (3.1 to 6.7)
Ischemic stroke	Dominican Republic	2010	Age-standardized		9.8% (6.5 to 13.5%)	4.5 (3.0 to 6.3)

Cause name	Location	Year	Age group	Number (in thousands)	PAF	Rate (per 100,000)
Ischemic stroke	Dominican Republic	2015	Age-standardized		9.8% (6.3 to 13.6%)	4.4 (2.9 to 6.3)
Hemorrhagic stroke	Dominican Republic	1990	All Ages	0.1 (0.1 to 0.2)	11.8% (9.3 to 14.3%)	2.1 (1.6 to 2.6)
Hemorrhagic stroke	Dominican Republic	1995	All Ages	0.2 (0.1 to 0.3)	11.8% (9.2 to 14.4%)	2.5 (2.0 to 3.1)
Hemorrhagic stroke	Dominican Republic	2000	All Ages	0.3 (0.2 to 0.4)	13.2% (10.3 to 16.2%)	3.3 (2.5 to 4.1)
Hemorrhagic stroke	Dominican Republic	2005	All Ages	0.3 (0.2 to 0.4)	11.4% (8.6 to 14.2%)	3.3 (2.5 to 4.2)
Hemorrhagic stroke	Dominican Republic	2010	All Ages	0.4 (0.3 to 0.5)	11.0% (8.2 to 13.9%)	3.6 (2.7 to 4.6)
Hemorrhagic stroke	Dominican Republic	2015	All Ages	0.4 (0.3 to 0.5)	10.9% (8.0 to 14.1%)	3.9 (2.7 to 5.1)
Hemorrhagic stroke	Dominican Republic	1990	Age-standardized		11.6% (8.9 to 14.3%)	4.3 (3.3 to 5.5)
Hemorrhagic stroke	Dominican Republic	1995	Age-standardized		11.4% (8.7 to 14.2%)	4.9 (3.7 to 6.2)
Hemorrhagic stroke	Dominican Republic	2000	Age-standardized		12.8% (9.8 to 16.0%)	6.0 (4.5 to 7.6)
Hemorrhagic stroke	Dominican Republic	2005	Age-standardized		11.1% (8.2 to 14.1%)	5.5 (4.0 to 6.9)
Hemorrhagic stroke	Dominican Republic	2010	Age-standardized		10.8% (7.9 to 13.8%)	5.4 (3.9 to 7.0)
Hemorrhagic stroke	Dominican Republic	2015	Age-standardized		10.7% (7.8 to 14.0%)	5.3 (3.7 to 6.9)
Chronic obstructive pulmonary disease	Dominican Republic	1990	All Ages	0.1 (0.0 to 0.1)	15.7% (7.5 to 24.4%)	1.0 (0.5 to 1.6)
Chronic obstructive pulmonary disease	Dominican Republic	1995	All Ages	0.1 (0.1 to 0.1)	17.0% (8.2 to 26.7%)	1.2 (0.6 to 1.9)
Chronic obstructive pulmonary disease	Dominican Republic	2000	All Ages	0.1 (0.1 to 0.2)	21.3% (11.2 to 32.0%)	1.6 (0.8 to 2.5)
Chronic obstructive pulmonary disease	Dominican Republic	2005	All Ages	0.1 (0.1 to 0.2)	17.9% (8.4 to 28.5%)	1.7 (0.8 to 2.6)
Chronic obstructive pulmonary disease	Dominican Republic	2010	All Ages	0.2 (0.1 to 0.3)	17.7% (8.9 to 28.3%)	1.9 (0.9 to 2.9)
Chronic obstructive pulmonary disease	Dominican Republic	2015	All Ages	0.2 (0.1 to 0.3)	17.5% (8.6 to 27.9%)	2.0 (1.0 to 3.2)
Chronic obstructive pulmonary disease	Dominican Republic	1990	Age-standardized		17.5% (8.3 to 27.5%)	2.9 (1.4 to 4.6)
Chronic obstructive pulmonary disease	Dominican Republic	1995	Age-standardized		17.8% (8.6 to 28.2%)	2.9 (1.4 to 4.7)
Chronic obstructive pulmonary disease	Dominican Republic	2000	Age-standardized		21.7% (11.4 to 32.7%)	3.4 (1.8 to 5.2)
Chronic obstructive pulmonary disease	Dominican Republic	2005	Age-standardized		18.2% (8.6 to 28.9%)	3.0 (1.4 to 4.8)
Chronic obstructive pulmonary disease	Dominican Republic	2010	Age-standardized		17.8% (9.0 to 28.6%)	3.0 (1.5 to 4.7)
Chronic obstructive pulmonary disease	Dominican Republic	2015	Age-standardized		17.7% (8.6 to 28.0%)	2.9 (1.4 to 4.6)
All causes	Grenada	1990	All Ages	0.1 (0.0 to 0.1)	6.0% (4.2 to 8.0%)	48.1 (34.0 to 63.7)
All causes	Grenada	1995	All Ages	0.0 (0.0 to 0.1)	5.9% (4.0 to 7.7%)	43.8 (29.8 to 56.7)
All causes	Grenada	2000	All Ages	0.0 (0.0 to 0.1)	5.4% (3.7 to 7.4%)	42.0 (27.9 to 57.5)
All causes	Grenada	2005	All Ages	0.0 (0.0 to 0.1)	4.7% (3.1 to 6.4%)	38.2 (25.4 to 51.5)
All causes	Grenada	2010	All Ages	0.0 (0.0 to 0.1)	4.1% (2.6 to 5.6%)	32.9 (21.5 to 44.6)
All causes	Grenada	2015	All Ages	0.0 (0.0 to 0.1)	4.2% (2.8 to 5.8%)	33.9 (22.3 to 47.0)
All causes	Grenada	1990	Age-standardized		6.4% (4.5 to 8.4%)	67.2 (47.9 to 88.7)
All causes	Grenada	1995	Age-standardized		6.1% (4.2 to 8.0%)	62.8 (43.2 to 81.4)
All causes	Grenada	2000	Age-standardized		5.6% (3.8 to 7.6%)	55.7 (37.3 to 75.7)
All causes	Grenada	2005	Age-standardized		4.9% (3.3 to 6.6%)	50.4 (33.7 to 67.7)
All causes	Grenada	2010	Age-standardized		4.2% (2.8 to 5.7%)	42.2 (28.0 to 57.0)

Cause name	Location	Year	Age group	Number (in thousands)	PAF	Rate (per 100,000)
All causes	Grenada	2015	Age-standardized		4.3% (2.9 to 6.0%)	43.0 (28.3 to 59.6)
Lower respiratory infections	Grenada	1990	All Ages	0.0 (0.0 to 0.0)	16.6% (5.7 to 28.2%)	10.3 (3.6 to 17.7)
Lower respiratory infections	Grenada	1995	All Ages	0.0 (0.0 to 0.0)	16.6% (5.9 to 28.6%)	9.3 (3.3 to 16.2)
Lower respiratory infections	Grenada	2000	All Ages	0.0 (0.0 to 0.0)	16.6% (5.6 to 29.2%)	10.3 (3.4 to 17.9)
Lower respiratory infections	Grenada	2005	All Ages	0.0 (0.0 to 0.0)	14.4% (4.9 to 26.7%)	9.0 (3.0 to 17.1)
Lower respiratory infections	Grenada	2010	All Ages	0.0 (0.0 to 0.0)	12.1% (3.3 to 24.3%)	7.4 (2.0 to 14.9)
Lower respiratory infections	Grenada	2015	All Ages	0.0 (0.0 to 0.0)	12.9% (3.7 to 25.0%)	7.7 (2.1 to 15.0)
Lower respiratory infections	Grenada	1990	Age-standardized		16.6% (5.7 to 28.2%)	13.4 (4.8 to 23.2)
Lower respiratory infections	Grenada	1995	Age-standardized		16.6% (5.9 to 28.6%)	12.9 (4.6 to 22.5)
Lower respiratory infections	Grenada	2000	Age-standardized		16.6% (5.6 to 29.2%)	12.9 (4.3 to 22.2)
Lower respiratory infections	Grenada	2005	Age-standardized		14.4% (4.9 to 26.7%)	11.1 (3.8 to 21.2)
Lower respiratory infections	Grenada	2010	Age-standardized		12.1% (3.3 to 24.3%)	8.9 (2.4 to 17.8)
Lower respiratory infections	Grenada	2015	Age-standardized		12.9% (3.7 to 25.0%)	9.4 (2.6 to 18.3)
Tracheal, bronchus, and lung cancer	Grenada	1990	All Ages	0.0 (0.0 to 0.0)	11.5% (5.2 to 20.1%)	1.1 (0.5 to 1.9)
Tracheal, bronchus, and lung cancer	Grenada	1995	All Ages	0.0 (0.0 to 0.0)	11.5% (5.0 to 19.6%)	1.1 (0.5 to 1.9)
Tracheal, bronchus, and lung cancer	Grenada	2000	All Ages	0.0 (0.0 to 0.0)	11.5% (4.8 to 20.5%)	1.2 (0.5 to 2.1)
Tracheal, bronchus, and lung cancer	Grenada	2005	All Ages	0.0 (0.0 to 0.0)	9.9% (4.0 to 17.7%)	1.1 (0.4 to 2.0)
Tracheal, bronchus, and lung cancer	Grenada	2010	All Ages	0.0 (0.0 to 0.0)	8.3% (3.1 to 15.5%)	0.9 (0.4 to 1.7)
Tracheal, bronchus, and lung cancer	Grenada	2015	All Ages	0.0 (0.0 to 0.0)	8.9% (3.4 to 16.1%)	1.1 (0.4 to 2.0)
Tracheal, bronchus, and lung cancer	Grenada	1990	Age-standardized		11.6% (5.2 to 20.1%)	1.5 (0.7 to 2.6)
Tracheal, bronchus, and lung cancer	Grenada	1995	Age-standardized		11.6% (5.0 to 19.7%)	1.6 (0.7 to 2.7)
Tracheal, bronchus, and lung cancer	Grenada	2000	Age-standardized		11.6% (4.8 to 20.5%)	1.7 (0.7 to 2.9)
Tracheal, bronchus, and lung cancer	Grenada	2005	Age-standardized		10.0% (4.0 to 17.7%)	1.5 (0.6 to 2.8)
Tracheal, bronchus, and lung cancer	Grenada	2010	Age-standardized		8.3% (3.2 to 15.6%)	1.3 (0.5 to 2.4)
Tracheal, bronchus, and lung cancer	Grenada	2015	Age-standardized		8.9% (3.4 to 16.2%)	1.4 (0.5 to 2.6)
Cardiovascular diseases	Grenada	1990	All Ages	0.0 (0.0 to 0.0)	10.8% (8.1 to 13.6%)	33.7 (25.0 to 42.9)
Cardiovascular diseases	Grenada	1995	All Ages	0.0 (0.0 to 0.0)	10.6% (7.8 to 13.3%)	30.7 (22.6 to 38.8)
Cardiovascular diseases	Grenada	2000	All Ages	0.0 (0.0 to 0.0)	10.1% (7.4 to 13.0%)	27.7 (20.2 to 35.8)
Cardiovascular diseases	Grenada	2005	All Ages	0.0 (0.0 to 0.0)	9.3% (6.7 to 11.9%)	25.7 (18.5 to 33.0)
Cardiovascular diseases	Grenada	2010	All Ages	0.0 (0.0 to 0.0)	8.4% (5.7 to 11.1%)	22.6 (15.4 to 29.5)
Cardiovascular diseases	Grenada	2015	All Ages	0.0 (0.0 to 0.0)	8.7% (6.2 to 11.3%)	23.2 (16.6 to 30.5)
Cardiovascular diseases	Grenada	1990	Age-standardized		10.7% (7.9 to 13.5%)	47.9 (35.6 to 61.2)
Cardiovascular diseases	Grenada	1995	Age-standardized		10.6% (7.8 to 13.4%)	44.4 (32.8 to 55.9)
Cardiovascular diseases	Grenada	2000	Age-standardized		10.4% (7.6 to 13.4%)	37.5 (27.6 to 48.6)
Cardiovascular diseases	Grenada	2005	Age-standardized		9.5% (6.9 to 12.1%)	34.5 (24.8 to 44.3)
Cardiovascular diseases	Grenada	2010	Age-standardized		8.6% (6.0 to 11.3%)	29.6 (20.5 to 38.9)

Cause name	Location	Year	Age group	Number (in thousands)	PAF	Rate (per 100,000)
Cardiovascular diseases	Grenada	2015	Age-standardized		8.7% (6.2 to 11.3%)	29.8 (21.2 to 39.2)
Ischemic heart disease	Grenada	1990	All Ages	0.0 (0.0 to 0.0)	14.7% (11.0 to 18.8%)	20.8 (15.4 to 26.8)
Ischemic heart disease	Grenada	1995	All Ages	0.0 (0.0 to 0.0)	14.4% (10.8 to 18.3%)	18.8 (13.9 to 23.8)
Ischemic heart disease	Grenada	2000	All Ages	0.0 (0.0 to 0.0)	14.3% (10.3 to 18.6%)	16.9 (12.0 to 21.9)
Ischemic heart disease	Grenada	2005	All Ages	0.0 (0.0 to 0.0)	13.4% (9.5 to 17.5%)	16.0 (11.3 to 21.0)
Ischemic heart disease	Grenada	2010	All Ages	0.0 (0.0 to 0.0)	12.5% (8.4 to 16.5%)	14.1 (9.5 to 18.8)
Ischemic heart disease	Grenada	2015	All Ages	0.0 (0.0 to 0.0)	12.9% (9.2 to 17.1%)	14.4 (10.2 to 19.2)
Ischemic heart disease	Grenada	1990	Age-standardized		14.5% (10.7 to 18.5%)	29.7 (21.9 to 38.2)
Ischemic heart disease	Grenada	1995	Age-standardized		14.4% (10.5 to 18.5%)	27.4 (20.0 to 34.9)
Ischemic heart disease	Grenada	2000	Age-standardized		14.6% (10.6 to 18.8%)	23.0 (16.3 to 29.6)
Ischemic heart disease	Grenada	2005	Age-standardized		13.7% (9.7 to 17.8%)	21.6 (15.1 to 28.2)
Ischemic heart disease	Grenada	2010	Age-standardized		12.7% (8.7 to 16.7%)	18.5 (12.6 to 24.6)
Ischemic heart disease	Grenada	2015	Age-standardized		12.9% (9.2 to 17.1%)	18.5 (13.1 to 24.7)
Ischemic stroke	Grenada	1990	All Ages	0.0 (0.0 to 0.0)	10.5% (6.9 to 14.5%)	7.0 (4.6 to 9.7)
Ischemic stroke	Grenada	1995	All Ages	0.0 (0.0 to 0.0)	10.3% (6.3 to 14.3%)	6.5 (4.0 to 9.1)
Ischemic stroke	Grenada	2000	All Ages	0.0 (0.0 to 0.0)	10.1% (6.1 to 14.5%)	5.4 (3.3 to 7.8)
Ischemic stroke	Grenada	2005	All Ages	0.0 (0.0 to 0.0)	9.3% (5.5 to 13.4%)	4.6 (2.8 to 6.8)
Ischemic stroke	Grenada	2010	All Ages	0.0 (0.0 to 0.0)	8.4% (4.8 to 12.6%)	3.9 (2.2 to 6.0)
Ischemic stroke	Grenada	2015	All Ages	0.0 (0.0 to 0.0)	8.7% (5.0 to 12.9%)	4.1 (2.3 to 6.2)
Ischemic stroke	Grenada	1990	Age-standardized		10.3% (6.7 to 14.4%)	10.0 (6.4 to 14.0)
Ischemic stroke	Grenada	1995	Age-standardized		10.2% (6.3 to 14.3%)	9.3 (5.7 to 13.0)
Ischemic stroke	Grenada	2000	Age-standardized		10.3% (6.3 to 14.6%)	7.2 (4.3 to 10.3)
Ischemic stroke	Grenada	2005	Age-standardized		9.4% (5.7 to 13.3%)	6.0 (3.7 to 8.6)
Ischemic stroke	Grenada	2010	Age-standardized		8.6% (5.0 to 12.5%)	5.1 (3.0 to 7.6)
Ischemic stroke	Grenada	2015	Age-standardized		8.8% (5.0 to 12.9%)	5.3 (3.0 to 7.9)
Hemorrhagic stroke	Grenada	1990	All Ages	0.0 (0.0 to 0.0)	11.4% (7.9 to 15.3%)	5.9 (4.1 to 8.0)
Hemorrhagic stroke	Grenada	1995	All Ages	0.0 (0.0 to 0.0)	11.3% (7.5 to 14.9%)	5.4 (3.5 to 7.3)
Hemorrhagic stroke	Grenada	2000	All Ages	0.0 (0.0 to 0.0)	11.1% (7.2 to 15.2%)	5.4 (3.6 to 7.5)
Hemorrhagic stroke	Grenada	2005	All Ages	0.0 (0.0 to 0.0)	10.3% (6.6 to 14.0%)	5.1 (3.3 to 7.0)
Hemorrhagic stroke	Grenada	2010	All Ages	0.0 (0.0 to 0.0)	9.4% (5.9 to 13.2%)	4.5 (2.8 to 6.5)
Hemorrhagic stroke	Grenada	2015	All Ages	0.0 (0.0 to 0.0)	9.8% (6.1 to 13.6%)	4.7 (2.9 to 6.5)
Hemorrhagic stroke	Grenada	1990	Age-standardized		11.5% (8.0 to 15.5%)	8.2 (5.8 to 11.1)
Hemorrhagic stroke	Grenada	1995	Age-standardized		11.4% (7.5 to 15.3%)	7.8 (5.1 to 10.6)
Hemorrhagic stroke	Grenada	2000	Age-standardized		11.4% (7.5 to 15.7%)	7.4 (4.9 to 10.2)
Hemorrhagic stroke	Grenada	2005	Age-standardized		10.5% (6.9 to 14.3%)	6.9 (4.5 to 9.5)
Hemorrhagic stroke	Grenada	2010	Age-standardized		9.5% (6.1 to 13.3%)	5.9 (3.8 to 8.3)

Cause name	Location	Year	Age group	Number (in thousands)	PAF	Rate (per 100,000)
Hemorrhagic stroke	Grenada	2015	Age-standardized		9.7% (6.1 to 13.5%)	6.0 (3.7 to 8.3)
Chronic obstructive pulmonary disease	Grenada	1990	All Ages	0.0 (0.0 to 0.0)	17.2% (6.9 to 28.7%)	3.0 (1.2 to 4.9)
Chronic obstructive pulmonary disease	Grenada	1995	All Ages	0.0 (0.0 to 0.0)	17.4% (7.2 to 29.0%)	2.7 (1.1 to 4.5)
Chronic obstructive pulmonary disease	Grenada	2000	All Ages	0.0 (0.0 to 0.0)	17.4% (7.2 to 29.4%)	2.8 (1.1 to 4.7)
Chronic obstructive pulmonary disease	Grenada	2005	All Ages	0.0 (0.0 to 0.0)	15.7% (5.8 to 27.6%)	2.5 (0.9 to 4.4)
Chronic obstructive pulmonary disease	Grenada	2010	All Ages	0.0 (0.0 to 0.0)	13.8% (4.8 to 24.3%)	1.9 (0.7 to 3.5)
Chronic obstructive pulmonary disease	Grenada	2015	All Ages	0.0 (0.0 to 0.0)	14.4% (4.7 to 25.4%)	1.9 (0.6 to 3.5)
Chronic obstructive pulmonary disease	Grenada	1990	Age-standardized		17.5% (7.0 to 29.1%)	4.3 (1.7 to 7.1)
Chronic obstructive pulmonary disease	Grenada	1995	Age-standardized		17.6% (7.2 to 29.3%)	3.9 (1.5 to 6.5)
Chronic obstructive pulmonary disease	Grenada	2000	Age-standardized		17.5% (7.2 to 29.6%)	3.6 (1.5 to 6.1)
Chronic obstructive pulmonary disease	Grenada	2005	Age-standardized		15.8% (5.9 to 27.8%)	3.2 (1.2 to 5.8)
Chronic obstructive pulmonary disease	Grenada	2010	Age-standardized		13.9% (4.8 to 24.5%)	2.5 (0.9 to 4.5)
Chronic obstructive pulmonary disease	Grenada	2015	Age-standardized		14.5% (4.7 to 25.6%)	2.5 (0.8 to 4.5)
All causes	Guyana	1990	All Ages	0.3 (0.3 to 0.4)	5.6% (4.8 to 6.5%)	44.1 (37.3 to 51.0)
All causes	Guyana	1995	All Ages	0.3 (0.3 to 0.4)	5.8% (5.0 to 6.7%)	45.5 (38.9 to 52.6)
All causes	Guyana	2000	All Ages	0.3 (0.3 to 0.4)	5.2% (4.2 to 6.3%)	41.2 (33.0 to 50.6)
All causes	Guyana	2005	All Ages	0.3 (0.2 to 0.3)	4.5% (3.5 to 5.5%)	36.6 (28.6 to 45.2)
All causes	Guyana	2010	All Ages	0.3 (0.2 to 0.3)	4.1% (3.1 to 5.1%)	32.9 (24.8 to 41.6)
All causes	Guyana	2015	All Ages	0.3 (0.2 to 0.4)	4.6% (3.5 to 5.7%)	36.7 (27.7 to 46.2)
All causes	Guyana	1990	Age-standardized		6.9% (5.8 to 7.9%)	94.5 (80.1 to 109.6)
All causes	Guyana	1995	Age-standardized		6.8% (5.7 to 8.0%)	104.7 (87.0 to 123.4)
All causes	Guyana	2000	Age-standardized		6.1% (5.0 to 7.5%)	81.9 (65.8 to 100.6)
All causes	Guyana	2005	Age-standardized		5.2% (4.1 to 6.4%)	71.7 (55.7 to 89.4)
All causes	Guyana	2010	Age-standardized		4.6% (3.5 to 5.8%)	61.0 (45.9 to 78.1)
All causes	Guyana	2015	Age-standardized		4.9% (3.8 to 6.1%)	61.9 (46.1 to 78.0)
Lower respiratory infections	Guyana	1990	All Ages	0.1 (0.0 to 0.1)	20.1% (12.1 to 29.3%)	9.1 (5.5 to 13.5)
Lower respiratory infections	Guyana	1995	All Ages	0.1 (0.0 to 0.1)	20.1% (12.1 to 29.2%)	8.5 (5.0 to 12.5)
Lower respiratory infections	Guyana	2000	All Ages	0.1 (0.0 to 0.1)	20.0% (11.3 to 30.5%)	8.4 (4.7 to 13.2)
Lower respiratory infections	Guyana	2005	All Ages	0.0 (0.0 to 0.1)	15.0% (6.6 to 25.4%)	5.9 (2.6 to 10.2)
Lower respiratory infections	Guyana	2010	All Ages	0.0 (0.0 to 0.1)	12.6% (4.6 to 23.4%)	4.5 (1.7 to 8.5)
Lower respiratory infections	Guyana	2015	All Ages	0.0 (0.0 to 0.1)	14.3% (6.0 to 24.8%)	4.6 (1.9 to 8.2)
Lower respiratory infections	Guyana	1990	Age-standardized		20.1% (12.1 to 29.3%)	14.7 (8.8 to 21.9)
Lower respiratory infections	Guyana	1995	Age-standardized		20.1% (12.1 to 29.2%)	17.1 (10.0 to 25.9)
Lower respiratory infections	Guyana	2000	Age-standardized		20.0% (11.3 to 30.5%)	14.5 (7.9 to 22.9)
Lower respiratory infections	Guyana	2005	Age-standardized		15.0% (6.6 to 25.4%)	10.7 (4.8 to 18.8)
Lower respiratory infections	Guyana	2010	Age-standardized		12.6% (4.6 to 23.4%)	8.6 (3.2 to 16.2)

Cause name	Location	Year	Age group	Number (in thousands)	PAF	Rate (per 100,000)
Lower respiratory infections	Guyana	2015	Age-standardized		14.3% (6.0 to 24.8%)	8.8 (3.5 to 15.6)
Tracheal, bronchus, and lung cancer	Guyana	1990	All Ages	0.0 (0.0 to 0.0)	14.0% (8.1 to 20.9%)	0.5 (0.3 to 0.8)
Tracheal, bronchus, and lung cancer	Guyana	1995	All Ages	0.0 (0.0 to 0.0)	14.1% (7.9 to 20.7%)	0.6 (0.3 to 0.9)
Tracheal, bronchus, and lung cancer	Guyana	2000	All Ages	0.0 (0.0 to 0.0)	14.1% (7.8 to 22.0%)	0.6 (0.3 to 1.0)
Tracheal, bronchus, and lung cancer	Guyana	2005	All Ages	0.0 (0.0 to 0.0)	10.3% (5.0 to 16.8%)	0.5 (0.2 to 0.8)
Tracheal, bronchus, and lung cancer	Guyana	2010	All Ages	0.0 (0.0 to 0.0)	8.6% (4.0 to 14.5%)	0.5 (0.2 to 0.8)
Tracheal, bronchus, and lung cancer	Guyana	2015	All Ages	0.0 (0.0 to 0.0)	9.8% (4.8 to 16.2%)	0.6 (0.3 to 1.0)
Tracheal, bronchus, and lung cancer	Guyana	1990	Age-standardized		14.1% (8.2 to 21.1%)	1.2 (0.7 to 1.8)
Tracheal, bronchus, and lung cancer	Guyana	1995	Age-standardized		14.2% (8.0 to 20.8%)	1.2 (0.7 to 1.8)
Tracheal, bronchus, and lung cancer	Guyana	2000	Age-standardized		14.2% (7.8 to 22.1%)	1.3 (0.7 to 2.0)
Tracheal, bronchus, and lung cancer	Guyana	2005	Age-standardized		10.4% (5.0 to 16.8%)	0.9 (0.4 to 1.5)
Tracheal, bronchus, and lung cancer	Guyana	2010	Age-standardized		8.6% (4.0 to 14.6%)	0.7 (0.3 to 1.3)
Tracheal, bronchus, and lung cancer	Guyana	2015	Age-standardized		9.9% (4.8 to 16.3%)	0.9 (0.4 to 1.4)
Cardiovascular diseases	Guyana	1990	All Ages	0.3 (0.2 to 0.3)	12.0% (10.3 to 13.7%)	32.7 (27.7 to 37.5)
Cardiovascular diseases	Guyana	1995	All Ages	0.3 (0.2 to 0.3)	12.4% (10.7 to 14.2%)	34.8 (29.7 to 40.0)
Cardiovascular diseases	Guyana	2000	All Ages	0.2 (0.2 to 0.3)	12.4% (10.2 to 14.7%)	30.5 (24.9 to 36.4)
Cardiovascular diseases	Guyana	2005	All Ages	0.2 (0.2 to 0.3)	10.8% (8.8 to 13.1%)	28.9 (22.9 to 35.0)
Cardiovascular diseases	Guyana	2010	All Ages	0.2 (0.1 to 0.3)	10.0% (7.6 to 12.1%)	26.7 (20.2 to 33.3)
Cardiovascular diseases	Guyana	2015	All Ages	0.2 (0.2 to 0.3)	10.8% (8.6 to 13.3%)	30.1 (23.2 to 37.3)
Cardiovascular diseases	Guyana	1990	Age-standardized		11.6% (9.8 to 13.4%)	74.3 (62.3 to 86.2)
Cardiovascular diseases	Guyana	1995	Age-standardized		11.3% (9.3 to 13.4%)	81.6 (66.6 to 96.5)
Cardiovascular diseases	Guyana	2000	Age-standardized		11.9% (9.8 to 14.2%)	62.6 (50.4 to 75.0)
Cardiovascular diseases	Guyana	2005	Age-standardized		10.1% (8.0 to 12.3%)	56.8 (44.8 to 69.5)
Cardiovascular diseases	Guyana	2010	Age-standardized		9.1% (6.9 to 11.2%)	49.0 (37.3 to 61.4)
Cardiovascular diseases	Guyana	2015	Age-standardized		9.7% (7.5 to 11.9%)	49.5 (38.0 to 62.2)
Ischemic heart disease	Guyana	1990	All Ages	0.1 (0.1 to 0.2)	17.1% (13.9 to 20.1%)	18.6 (15.1 to 22.2)
Ischemic heart disease	Guyana	1995	All Ages	0.1 (0.1 to 0.2)	17.2% (14.2 to 20.3%)	20.5 (16.9 to 24.4)
Ischemic heart disease	Guyana	2000	All Ages	0.1 (0.1 to 0.2)	17.3% (14.0 to 20.8%)	18.8 (15.1 to 22.7)
Ischemic heart disease	Guyana	2005	All Ages	0.1 (0.1 to 0.2)	15.1% (11.9 to 18.5%)	18.5 (14.3 to 22.7)
Ischemic heart disease	Guyana	2010	All Ages	0.1 (0.1 to 0.2)	14.1% (10.6 to 17.3%)	17.5 (13.1 to 21.9)
Ischemic heart disease	Guyana	2015	All Ages	0.1 (0.1 to 0.2)	15.0% (11.6 to 18.6%)	20.0 (14.9 to 25.4)
Ischemic heart disease	Guyana	1990	Age-standardized		16.1% (13.0 to 19.2%)	42.1 (33.8 to 50.7)
Ischemic heart disease	Guyana	1995	Age-standardized		15.4% (11.9 to 19.0%)	48.6 (37.6 to 60.5)
Ischemic heart disease	Guyana	2000	Age-standardized		16.3% (13.0 to 19.6%)	38.3 (30.4 to 46.7)
Ischemic heart disease	Guyana	2005	Age-standardized		13.8% (10.4 to 17.2%)	36.0 (27.0 to 45.1)
Ischemic heart disease	Guyana	2010	Age-standardized		12.6% (9.3 to 15.9%)	31.5 (23.0 to 40.3)

Cause name	Location	Year	Age group	Number (in thousands)	PAF	Rate (per 100,000)
Ischemic heart disease	Guyana	2015	Age-standardized		13.3% (10.0 to 16.8%)	32.2 (24.2 to 41.4)
Ischemic stroke	Guyana	1990	All Ages	0.1 (0.0 to 0.1)	12.2% (9.3 to 15.3%)	6.4 (4.8 to 8.0)
Ischemic stroke	Guyana	1995	All Ages	0.1 (0.0 to 0.1)	12.2% (9.3 to 15.2%)	6.3 (4.7 to 7.9)
Ischemic stroke	Guyana	2000	All Ages	0.0 (0.0 to 0.1)	12.1% (8.9 to 15.4%)	4.9 (3.5 to 6.2)
Ischemic stroke	Guyana	2005	All Ages	0.0 (0.0 to 0.0)	10.2% (7.2 to 13.3%)	4.1 (2.9 to 5.5)
Ischemic stroke	Guyana	2010	All Ages	0.0 (0.0 to 0.0)	9.3% (6.3 to 12.4%)	3.5 (2.4 to 4.8)
Ischemic stroke	Guyana	2015	All Ages	0.0 (0.0 to 0.0)	10.1% (6.9 to 13.6%)	3.9 (2.7 to 5.5)
Ischemic stroke	Guyana	1990	Age-standardized		11.8% (8.8 to 14.9%)	15.6 (11.7 to 19.7)
Ischemic stroke	Guyana	1995	Age-standardized		11.2% (7.8 to 14.8%)	16.3 (11.3 to 21.8)
Ischemic stroke	Guyana	2000	Age-standardized		11.7% (8.3 to 15.0%)	10.9 (7.7 to 14.1)
Ischemic stroke	Guyana	2005	Age-standardized		9.6% (6.4 to 12.9%)	9.2 (6.1 to 12.4)
Ischemic stroke	Guyana	2010	Age-standardized		8.6% (5.5 to 11.9%)	7.7 (4.9 to 10.9)
Ischemic stroke	Guyana	2015	Age-standardized		9.2% (5.9 to 12.7%)	7.6 (4.9 to 10.9)
Hemorrhagic stroke	Guyana	1990	All Ages	0.1 (0.1 to 0.1)	13.4% (10.7 to 16.4%)	7.7 (6.1 to 9.6)
Hemorrhagic stroke	Guyana	1995	All Ages	0.1 (0.1 to 0.1)	13.5% (10.8 to 16.3%)	7.9 (6.3 to 9.8)
Hemorrhagic stroke	Guyana	2000	All Ages	0.1 (0.0 to 0.1)	13.3% (10.3 to 16.5%)	6.8 (5.1 to 8.6)
Hemorrhagic stroke	Guyana	2005	All Ages	0.1 (0.0 to 0.1)	11.3% (8.3 to 14.6%)	6.2 (4.6 to 8.1)
Hemorrhagic stroke	Guyana	2010	All Ages	0.0 (0.0 to 0.1)	10.3% (7.2 to 13.5%)	5.7 (4.0 to 7.6)
Hemorrhagic stroke	Guyana	2015	All Ages	0.1 (0.0 to 0.1)	11.1% (8.0 to 14.7%)	6.3 (4.4 to 8.4)
Hemorrhagic stroke	Guyana	1990	Age-standardized		13.1% (10.4 to 16.2%)	16.6 (13.0 to 20.9)
Hemorrhagic stroke	Guyana	1995	Age-standardized		12.6% (9.6 to 15.7%)	16.8 (12.8 to 21.1)
Hemorrhagic stroke	Guyana	2000	Age-standardized		12.9% (9.8 to 16.0%)	13.5 (10.0 to 17.0)
Hemorrhagic stroke	Guyana	2005	Age-standardized		10.5% (7.6 to 13.7%)	11.6 (8.2 to 15.3)
Hemorrhagic stroke	Guyana	2010	Age-standardized		9.4% (6.4 to 12.4%)	9.8 (6.7 to 13.2)
Hemorrhagic stroke	Guyana	2015	Age-standardized		10.1% (6.9 to 13.5%)	9.6 (6.5 to 13.1)
Chronic obstructive pulmonary disease	Guyana	1990	All Ages	0.0 (0.0 to 0.0)	18.9% (10.1 to 28.8%)	1.7 (0.9 to 2.6)
Chronic obstructive pulmonary disease	Guyana	1995	All Ages	0.0 (0.0 to 0.0)	19.4% (9.9 to 29.4%)	1.7 (0.9 to 2.7)
Chronic obstructive pulmonary disease	Guyana	2000	All Ages	0.0 (0.0 to 0.0)	19.3% (9.2 to 30.6%)	1.6 (0.8 to 2.5)
Chronic obstructive pulmonary disease	Guyana	2005	All Ages	0.0 (0.0 to 0.0)	15.7% (7.3 to 25.5%)	1.4 (0.7 to 2.3)
Chronic obstructive pulmonary disease	Guyana	2010	All Ages	0.0 (0.0 to 0.0)	13.7% (5.6 to 23.3%)	1.2 (0.5 to 2.0)
Chronic obstructive pulmonary disease	Guyana	2015	All Ages	0.0 (0.0 to 0.0)	15.2% (6.4 to 25.2%)	1.3 (0.6 to 2.2)
Chronic obstructive pulmonary disease	Guyana	1990	Age-standardized		20.1% (10.7 to 30.9%)	4.3 (2.3 to 6.6)
Chronic obstructive pulmonary disease	Guyana	1995	Age-standardized		20.2% (10.4 to 31.0%)	4.7 (2.4 to 7.5)
Chronic obstructive pulmonary disease	Guyana	2000	Age-standardized		20.1% (9.6 to 31.7%)	3.5 (1.7 to 5.6)
Chronic obstructive pulmonary disease	Guyana	2005	Age-standardized		16.1% (7.4 to 26.2%)	3.2 (1.5 to 5.3)
Chronic obstructive pulmonary disease	Guyana	2010	Age-standardized		14.0% (5.7 to 23.7%)	2.6 (1.0 to 4.5)

Cause name	Location	Year	Age group	Number (in thousands)	PAF	Rate (per 100,000)
Chronic obstructive pulmonary disease	Guyana	2015	Age-standardized		15.5% (6.5 to 25.7%)	2.7 (1.1 to 4.5)
All causes	Haiti	1990	All Ages	4.9 (4.0 to 5.9)	5.2% (4.3 to 6.2%)	69.3 (56.3 to 83.7)
All causes	Haiti	1995	All Ages	4.6 (3.8 to 5.6)	5.0% (4.2 to 6.0%)	59.1 (48.5 to 71.4)
All causes	Haiti	2000	All Ages	4.4 (3.5 to 5.3)	5.0% (4.1 to 6.0%)	50.9 (40.4 to 62.3)
All causes	Haiti	2005	All Ages	4.4 (3.5 to 5.5)	4.9% (4.1 to 5.9%)	47.6 (37.8 to 59.4)
All causes	Haiti	2010	All Ages	4.1 (3.2 to 5.1)	1.5% (0.7 to 3.3%)	41.2 (32.0 to 51.4)
All causes	Haiti	2015	All Ages	4.7 (3.7 to 5.8)	5.3% (4.4 to 6.2%)	43.6 (34.1 to 54.4)
All causes	Haiti	1990	Age-standardized		6.7% (5.7 to 7.7%)	124.7 (104.3 to 146.0)
All causes	Haiti	1995	Age-standardized		6.3% (5.4 to 7.5%)	110.6 (91.2 to 132.1)
All causes	Haiti	2000	Age-standardized		6.2% (5.2 to 7.3%)	98.7 (79.3 to 119.0)
All causes	Haiti	2005	Age-standardized		6.1% (5.1 to 7.1%)	92.6 (73.5 to 113.4)
All causes	Haiti	2010	Age-standardized		2.2% (1.2 to 4.4%)	79.4 (62.0 to 98.5)
All causes	Haiti	2015	Age-standardized		6.1% (5.1 to 7.2%)	81.3 (63.3 to 101.9)
Lower respiratory infections	Haiti	1990	All Ages	1.9 (1.2 to 2.7)	22.5% (14.4 to 31.4%)	27.1 (16.9 to 38.4)
Lower respiratory infections	Haiti	1995	All Ages	1.7 (1.1 to 2.5)	22.5% (14.8 to 31.3%)	22.3 (14.5 to 32.1)
Lower respiratory infections	Haiti	2000	All Ages	1.6 (1.0 to 2.3)	22.6% (14.8 to 31.5%)	18.3 (11.9 to 26.6)
Lower respiratory infections	Haiti	2005	All Ages	1.5 (0.9 to 2.2)	22.1% (14.2 to 30.7%)	16.3 (10.1 to 23.7)
Lower respiratory infections	Haiti	2010	All Ages	1.2 (0.7 to 1.9)	17.9% (10.1 to 27.3%)	12.0 (6.5 to 19.1)
Lower respiratory infections	Haiti	2015	All Ages	1.2 (0.7 to 1.8)	19.8% (11.7 to 29.3%)	11.1 (6.2 to 17.1)
Lower respiratory infections	Haiti	1990	Age-standardized		22.5% (14.4 to 31.4%)	29.4 (18.5 to 42.6)
Lower respiratory infections	Haiti	1995	Age-standardized		22.5% (14.8 to 31.3%)	26.6 (16.9 to 39.0)
Lower respiratory infections	Haiti	2000	Age-standardized		22.6% (14.8 to 31.5%)	24.2 (15.0 to 36.8)
Lower respiratory infections	Haiti	2005	Age-standardized		22.1% (14.2 to 30.7%)	22.8 (13.8 to 34.8)
Lower respiratory infections	Haiti	2010	Age-standardized		17.9% (10.1 to 27.3%)	17.7 (9.2 to 29.0)
Lower respiratory infections	Haiti	2015	Age-standardized		19.8% (11.7 to 29.3%)	17.5 (9.8 to 27.7)
Tracheal, bronchus, and lung cancer	Haiti	1990	All Ages	0.1 (0.0 to 0.1)	15.9% (9.3 to 23.0%)	1.0 (0.6 to 1.6)
Tracheal, bronchus, and lung cancer	Haiti	1995	All Ages	0.1 (0.0 to 0.1)	15.9% (9.3 to 23.1%)	0.9 (0.5 to 1.5)
Tracheal, bronchus, and lung cancer	Haiti	2000	All Ages	0.1 (0.0 to 0.1)	16.0% (9.5 to 23.4%)	0.9 (0.5 to 1.4)
Tracheal, bronchus, and lung cancer	Haiti	2005	All Ages	0.1 (0.0 to 0.1)	15.6% (9.3 to 22.7%)	0.9 (0.5 to 1.4)
Tracheal, bronchus, and lung cancer	Haiti	2010	All Ages	0.1 (0.0 to 0.1)	12.5% (7.0 to 19.2%)	0.8 (0.4 to 1.3)
Tracheal, bronchus, and lung cancer	Haiti	2015	All Ages	0.1 (0.1 to 0.2)	13.9% (8.0 to 20.8%)	0.9 (0.5 to 1.5)
Tracheal, bronchus, and lung cancer	Haiti	1990	Age-standardized		16.0% (9.4 to 23.1%)	2.2 (1.2 to 3.4)
Tracheal, bronchus, and lung cancer	Haiti	1995	Age-standardized		16.0% (9.4 to 23.2%)	2.0 (1.1 to 3.2)
Tracheal, bronchus, and lung cancer	Haiti	2000	Age-standardized		16.1% (9.5 to 23.5%)	1.9 (1.1 to 3.1)
Tracheal, bronchus, and lung cancer	Haiti	2005	Age-standardized		15.7% (9.3 to 22.8%)	1.9 (1.0 to 3.0)
Tracheal, bronchus, and lung cancer	Haiti	2010	Age-standardized		12.5% (7.0 to 19.3%)	1.5 (0.8 to 2.5)

Cause name	Location	Year	Age group	Number (in thousands)	PAF	Rate (per 100,000)
Tracheal, bronchus, and lung cancer	Haiti	2015	Age-standardized		14.0% (8.1 to 20.8%)	1.7 (0.9 to 2.8)
Cardiovascular diseases	Haiti	1990	All Ages	2.7 (2.3 to 3.2)	13.0% (11.1 to 14.9%)	38.0 (31.7 to 44.8)
Cardiovascular diseases	Haiti	1995	All Ages	2.6 (2.1 to 3.1)	12.9% (10.9 to 14.8%)	32.8 (26.9 to 39.2)
Cardiovascular diseases	Haiti	2000	All Ages	2.5 (1.9 to 3.0)	12.7% (10.8 to 14.7%)	28.7 (22.7 to 35.6)
Cardiovascular diseases	Haiti	2005	All Ages	2.5 (2.0 to 3.3)	12.5% (10.5 to 14.4%)	27.4 (21.3 to 35.0)
Cardiovascular diseases	Haiti	2010	All Ages	2.6 (2.0 to 3.2)	11.2% (9.4 to 13.1%)	25.7 (19.7 to 32.4)
Cardiovascular diseases	Haiti	2015	All Ages	3.1 (2.3 to 3.9)	11.9% (10.0 to 13.7%)	28.4 (21.5 to 36.2)
Cardiovascular diseases	Haiti	1990	Age-standardized		12.6% (10.6 to 14.6%)	85.0 (69.3 to 100.2)
Cardiovascular diseases	Haiti	1995	Age-standardized		12.4% (10.4 to 14.5%)	74.2 (60.1 to 89.9)
Cardiovascular diseases	Haiti	2000	Age-standardized		12.2% (10.1 to 14.4%)	65.0 (50.3 to 81.5)
Cardiovascular diseases	Haiti	2005	Age-standardized		12.0% (9.8 to 14.0%)	60.6 (46.2 to 78.4)
Cardiovascular diseases	Haiti	2010	Age-standardized		10.7% (8.7 to 12.6%)	53.9 (40.3 to 68.7)
Cardiovascular diseases	Haiti	2015	Age-standardized		11.3% (9.4 to 13.3%)	55.4 (41.5 to 71.4)
Ischemic heart disease	Haiti	1990	All Ages	1.7 (1.4 to 2.0)	18.1% (15.1 to 21.4%)	23.5 (19.1 to 28.7)
Ischemic heart disease	Haiti	1995	All Ages	1.6 (1.3 to 2.0)	17.9% (14.9 to 21.1%)	20.1 (16.1 to 24.9)
Ischemic heart disease	Haiti	2000	All Ages	1.5 (1.1 to 1.9)	17.8% (14.8 to 21.0%)	17.3 (13.4 to 21.8)
Ischemic heart disease	Haiti	2005	All Ages	1.5 (1.2 to 2.0)	17.6% (14.5 to 20.8%)	16.5 (12.4 to 21.2)
Ischemic heart disease	Haiti	2010	All Ages	1.5 (1.2 to 2.0)	16.0% (13.2 to 19.0%)	15.4 (11.6 to 19.9)
Ischemic heart disease	Haiti	2015	All Ages	1.8 (1.4 to 2.4)	16.7% (13.8 to 19.8%)	17.0 (12.7 to 22.1)
Ischemic heart disease	Haiti	1990	Age-standardized		16.8% (13.6 to 20.1%)	52.9 (41.7 to 64.1)
Ischemic heart disease	Haiti	1995	Age-standardized		16.7% (13.4 to 19.9%)	45.6 (35.2 to 56.9)
Ischemic heart disease	Haiti	2000	Age-standardized		16.6% (13.1 to 20.0%)	39.2 (29.4 to 49.8)
Ischemic heart disease	Haiti	2005	Age-standardized		16.3% (13.1 to 19.7%)	36.4 (26.9 to 47.4)
Ischemic heart disease	Haiti	2010	Age-standardized		14.9% (11.7 to 18.1%)	32.4 (23.7 to 42.0)
Ischemic heart disease	Haiti	2015	Age-standardized		15.6% (12.4 to 18.8%)	33.2 (24.4 to 43.3)
Ischemic stroke	Haiti	1990	All Ages	0.5 (0.4 to 0.6)	13.3% (10.4 to 16.2%)	7.0 (5.0 to 9.1)
Ischemic stroke	Haiti	1995	All Ages	0.5 (0.3 to 0.6)	13.1% (10.1 to 16.1%)	6.0 (4.2 to 8.0)
Ischemic stroke	Haiti	2000	All Ages	0.4 (0.3 to 0.6)	13.0% (9.9 to 15.8%)	5.2 (3.5 to 7.1)
Ischemic stroke	Haiti	2005	All Ages	0.5 (0.3 to 0.6)	12.7% (9.7 to 15.7%)	4.9 (3.2 to 6.8)
Ischemic stroke	Haiti	2010	All Ages	0.5 (0.3 to 0.6)	11.2% (8.3 to 14.1%)	4.5 (3.0 to 6.3)
Ischemic stroke	Haiti	2015	All Ages	0.5 (0.4 to 0.8)	11.8% (8.9 to 14.8%)	5.0 (3.3 to 7.1)
Ischemic stroke	Haiti	1990	Age-standardized		12.5% (9.4 to 15.7%)	17.0 (11.9 to 22.5)
Ischemic stroke	Haiti	1995	Age-standardized		12.4% (9.2 to 15.5%)	14.8 (10.1 to 20.0)
Ischemic stroke	Haiti	2000	Age-standardized		12.3% (9.1 to 15.5%)	12.8 (8.5 to 18.0)
Ischemic stroke	Haiti	2005	Age-standardized		12.0% (8.8 to 15.3%)	11.8 (7.6 to 16.8)
Ischemic stroke	Haiti	2010	Age-standardized		10.6% (7.5 to 13.7%)	10.4 (6.6 to 15.0)

Cause name	Location	Year	Age group	Number (in thousands)	PAF	Rate (per 100,000)
Ischemic stroke	Haiti	2015	Age-standardized		11.2% (8.0 to 14.6%)	10.7 (6.9 to 15.5)
Hemorrhagic stroke	Haiti	1990	All Ages	0.5 (0.4 to 0.7)	13.7% (11.0 to 16.4%)	7.5 (5.6 to 9.7)
Hemorrhagic stroke	Haiti	1995	All Ages	0.5 (0.4 to 0.7)	13.7% (11.0 to 16.4%)	6.7 (4.9 to 8.7)
Hemorrhagic stroke	Haiti	2000	All Ages	0.5 (0.4 to 0.7)	13.8% (11.1 to 16.5%)	6.2 (4.4 to 8.3)
Hemorrhagic stroke	Haiti	2005	All Ages	0.6 (0.4 to 0.8)	13.6% (10.9 to 16.4%)	6.1 (4.2 to 8.2)
Hemorrhagic stroke	Haiti	2010	All Ages	0.6 (0.4 to 0.8)	12.2% (9.6 to 14.9%)	5.7 (4.0 to 7.7)
Hemorrhagic stroke	Haiti	2015	All Ages	0.7 (0.5 to 0.9)	13.1% (10.3 to 16.0%)	6.4 (4.3 to 8.8)
Hemorrhagic stroke	Haiti	1990	Age-standardized		13.9% (11.2 to 16.7%)	15.1 (11.0 to 19.6)
Hemorrhagic stroke	Haiti	1995	Age-standardized		13.7% (10.7 to 16.5%)	13.8 (9.8 to 18.1)
Hemorrhagic stroke	Haiti	2000	Age-standardized		13.6% (10.7 to 16.5%)	13.0 (8.9 to 17.3)
Hemorrhagic stroke	Haiti	2005	Age-standardized		13.3% (10.5 to 16.3%)	12.4 (8.2 to 16.7)
Hemorrhagic stroke	Haiti	2010	Age-standardized		11.8% (9.0 to 14.7%)	11.1 (7.2 to 15.2)
Hemorrhagic stroke	Haiti	2015	Age-standardized		12.5% (9.7 to 15.4%)	11.6 (7.7 to 16.0)
Chronic obstructive pulmonary disease	Haiti	1990	All Ages	0.2 (0.1 to 0.4)	17.1% (8.6 to 26.6%)	3.2 (1.5 to 5.1)
Chronic obstructive pulmonary disease	Haiti	1995	All Ages	0.2 (0.1 to 0.4)	18.1% (9.2 to 27.5%)	3.0 (1.4 to 4.9)
Chronic obstructive pulmonary disease	Haiti	2000	All Ages	0.3 (0.1 to 0.4)	19.1% (10.1 to 28.9%)	3.0 (1.4 to 4.8)
Chronic obstructive pulmonary disease	Haiti	2005	All Ages	0.3 (0.1 to 0.5)	19.2% (10.3 to 29.1%)	2.9 (1.5 to 4.8)
Chronic obstructive pulmonary disease	Haiti	2010	All Ages	0.3 (0.1 to 0.5)	16.8% (8.5 to 26.4%)	2.7 (1.3 to 4.7)
Chronic obstructive pulmonary disease	Haiti	2015	All Ages	0.3 (0.2 to 0.6)	18.9% (9.9 to 28.8%)	3.1 (1.5 to 5.2)
Chronic obstructive pulmonary disease	Haiti	1990	Age-standardized		21.0% (10.9 to 31.6%)	8.1 (3.7 to 13.1)
Chronic obstructive pulmonary disease	Haiti	1995	Age-standardized		21.2% (11.6 to 31.9%)	7.8 (3.7 to 12.6)
Chronic obstructive pulmonary disease	Haiti	2000	Age-standardized		21.6% (11.7 to 31.9%)	7.6 (3.6 to 12.4)
Chronic obstructive pulmonary disease	Haiti	2005	Age-standardized		21.3% (11.5 to 31.7%)	7.3 (3.6 to 11.9)
Chronic obstructive pulmonary disease	Haiti	2010	Age-standardized		18.1% (9.2 to 28.3%)	6.3 (2.9 to 10.9)
Chronic obstructive pulmonary disease	Haiti	2015	Age-standardized		19.8% (10.3 to 30.5%)	6.6 (3.1 to 11.1)
All causes	Jamaica	1990	All Ages	0.7 (0.6 to 0.9)	5.1% (4.2 to 6.0%)	30.1 (24.8 to 36.0)
All causes	Jamaica	1995	All Ages	0.8 (0.6 to 0.9)	5.0% (4.1 to 6.0%)	31.3 (25.4 to 37.7)
All causes	Jamaica	2000	All Ages	0.8 (0.6 to 0.9)	4.6% (3.7 to 5.6%)	30.2 (24.4 to 36.3)
All causes	Jamaica	2005	All Ages	0.7 (0.6 to 0.9)	4.3% (3.5 to 5.3%)	27.5 (22.0 to 33.4)
All causes	Jamaica	2010	All Ages	0.7 (0.5 to 0.9)	3.6% (2.8 to 4.5%)	25.3 (19.6 to 31.9)
All causes	Jamaica	2015	All Ages	0.9 (0.7 to 1.1)	4.1% (3.3 to 5.1%)	31.7 (25.0 to 39.4)
All causes	Jamaica	1990	Age-standardized		5.4% (4.5 to 6.3%)	40.2 (33.4 to 47.6)
All causes	Jamaica	1995	Age-standardized		5.3% (4.3 to 6.2%)	40.5 (33.2 to 48.3)
All causes	Jamaica	2000	Age-standardized		4.8% (4.0 to 5.7%)	37.3 (30.6 to 44.4)
All causes	Jamaica	2005	Age-standardized		4.4% (3.6 to 5.4%)	32.2 (26.1 to 38.6)
All causes	Jamaica	2010	Age-standardized		3.6% (2.9 to 4.5%)	28.0 (22.2 to 34.8)

Cause name	Location	Year	Age group	Number (in thousands)	PAF	Rate (per 100,000)
All causes	Jamaica	2015	Age-standardized		4.2% (3.4 to 5.0%)	32.4 (25.8 to 40.0)
Lower respiratory infections	Jamaica	1990	All Ages	0.1 (0.1 to 0.2)	17.2% (8.6 to 27.4%)	4.5 (2.3 to 7.3)
Lower respiratory infections	Jamaica	1995	All Ages	0.1 (0.1 to 0.2)	17.1% (8.3 to 27.4%)	4.6 (2.3 to 7.6)
Lower respiratory infections	Jamaica	2000	All Ages	0.1 (0.1 to 0.2)	17.1% (8.4 to 27.2%)	4.8 (2.3 to 7.9)
Lower respiratory infections	Jamaica	2005	All Ages	0.1 (0.1 to 0.2)	16.7% (8.0 to 26.9%)	4.2 (2.0 to 7.0)
Lower respiratory infections	Jamaica	2010	All Ages	0.1 (0.0 to 0.2)	11.9% (3.9 to 22.9%)	3.2 (1.1 to 6.1)
Lower respiratory infections	Jamaica	2015	All Ages	0.1 (0.1 to 0.2)	14.6% (6.1 to 25.1%)	4.1 (1.7 to 7.3)
Lower respiratory infections	Jamaica	1990	Age-standardized		17.2% (8.6 to 27.4%)	5.1 (2.6 to 8.3)
Lower respiratory infections	Jamaica	1995	Age-standardized		17.1% (8.3 to 27.4%)	5.3 (2.6 to 8.6)
Lower respiratory infections	Jamaica	2000	Age-standardized		17.1% (8.4 to 27.2%)	5.4 (2.6 to 8.8)
Lower respiratory infections	Jamaica	2005	Age-standardized		16.7% (8.0 to 26.9%)	4.6 (2.2 to 7.7)
Lower respiratory infections	Jamaica	2010	Age-standardized		11.9% (3.9 to 22.9%)	3.3 (1.1 to 6.4)
Lower respiratory infections	Jamaica	2015	Age-standardized		14.6% (6.1 to 25.1%)	4.0 (1.7 to 7.2)
Tracheal, bronchus, and lung cancer	Jamaica	1990	All Ages	0.0 (0.0 to 0.1)	12.0% (6.5 to 18.5%)	1.3 (0.7 to 2.1)
Tracheal, bronchus, and lung cancer	Jamaica	1995	All Ages	0.0 (0.0 to 0.1)	11.9% (6.6 to 18.2%)	1.6 (0.9 to 2.4)
Tracheal, bronchus, and lung cancer	Jamaica	2000	All Ages	0.1 (0.0 to 0.1)	11.9% (6.5 to 18.2%)	1.8 (1.0 to 2.8)
Tracheal, bronchus, and lung cancer	Jamaica	2005	All Ages	0.0 (0.0 to 0.1)	11.6% (6.3 to 17.8%)	1.7 (0.9 to 2.6)
Tracheal, bronchus, and lung cancer	Jamaica	2010	All Ages	0.0 (0.0 to 0.1)	8.2% (4.0 to 13.6%)	1.4 (0.7 to 2.4)
Tracheal, bronchus, and lung cancer	Jamaica	2015	All Ages	0.1 (0.0 to 0.1)	10.1% (5.3 to 15.9%)	2.0 (1.0 to 3.2)
Tracheal, bronchus, and lung cancer	Jamaica	1990	Age-standardized		12.0% (6.5 to 18.5%)	2.0 (1.1 to 3.1)
Tracheal, bronchus, and lung cancer	Jamaica	1995	Age-standardized		11.9% (6.6 to 18.2%)	2.3 (1.3 to 3.5)
Tracheal, bronchus, and lung cancer	Jamaica	2000	Age-standardized		11.9% (6.5 to 18.2%)	2.5 (1.3 to 3.7)
Tracheal, bronchus, and lung cancer	Jamaica	2005	Age-standardized		11.6% (6.3 to 17.9%)	2.1 (1.1 to 3.3)
Tracheal, bronchus, and lung cancer	Jamaica	2010	Age-standardized		8.2% (4.0 to 13.6%)	1.7 (0.8 to 2.8)
Tracheal, bronchus, and lung cancer	Jamaica	2015	Age-standardized		10.1% (5.3 to 15.9%)	2.1 (1.1 to 3.4)
Cardiovascular diseases	Jamaica	1990	All Ages	0.5 (0.4 to 0.6)	9.3% (7.5 to 11.1%)	22.0 (17.6 to 26.5)
Cardiovascular diseases	Jamaica	1995	All Ages	0.6 (0.4 to 0.7)	9.6% (7.6 to 11.6%)	22.4 (17.7 to 27.3)
Cardiovascular diseases	Jamaica	2000	All Ages	0.5 (0.4 to 0.6)	9.9% (7.9 to 12.0%)	20.3 (16.0 to 24.6)
Cardiovascular diseases	Jamaica	2005	All Ages	0.5 (0.4 to 0.6)	9.4% (7.3 to 11.4%)	18.4 (14.3 to 22.7)
Cardiovascular diseases	Jamaica	2010	All Ages	0.5 (0.4 to 0.6)	7.9% (6.0 to 10.0%)	18.1 (13.6 to 23.1)
Cardiovascular diseases	Jamaica	2015	All Ages	0.6 (0.5 to 0.8)	8.7% (6.7 to 10.8%)	22.2 (16.6 to 28.4)
Cardiovascular diseases	Jamaica	1990	Age-standardized		9.7% (7.9 to 11.5%)	30.1 (24.3 to 36.0)
Cardiovascular diseases	Jamaica	1995	Age-standardized		9.9% (7.9 to 11.9%)	29.5 (23.5 to 35.5)
Cardiovascular diseases	Jamaica	2000	Age-standardized		10.2% (8.2 to 12.1%)	25.3 (20.2 to 30.5)
Cardiovascular diseases	Jamaica	2005	Age-standardized		9.6% (7.6 to 11.5%)	21.7 (17.1 to 26.6)
Cardiovascular diseases	Jamaica	2010	Age-standardized		8.0% (6.2 to 10.0%)	20.1 (15.4 to 25.4)

Cause name	Location	Year	Age group	Number (in thousands)	PAF	Rate (per 100,000)
Cardiovascular diseases	Jamaica	2015	Age-standardized		8.9% (7.0 to 10.8%)	22.8 (17.3 to 28.7)
Ischemic heart disease	Jamaica	1990	All Ages	0.3 (0.2 to 0.3)	14.1% (10.6 to 17.6%)	11.1 (8.3 to 13.8)
Ischemic heart disease	Jamaica	1995	All Ages	0.3 (0.2 to 0.4)	13.9% (10.2 to 17.7%)	11.2 (8.2 to 14.3)
Ischemic heart disease	Jamaica	2000	All Ages	0.3 (0.2 to 0.3)	14.1% (10.5 to 17.8%)	10.0 (7.4 to 12.6)
Ischemic heart disease	Jamaica	2005	All Ages	0.2 (0.2 to 0.3)	13.7% (10.0 to 17.6%)	9.0 (6.5 to 11.5)
Ischemic heart disease	Jamaica	2010	All Ages	0.3 (0.2 to 0.3)	11.9% (8.3 to 15.4%)	9.0 (6.4 to 11.9)
Ischemic heart disease	Jamaica	2015	All Ages	0.3 (0.2 to 0.4)	12.9% (9.3 to 16.6%)	11.1 (7.9 to 14.9)
Ischemic heart disease	Jamaica	1990	Age-standardized		14.4% (11.1 to 17.8%)	15.2 (11.5 to 18.6)
Ischemic heart disease	Jamaica	1995	Age-standardized		14.3% (10.8 to 17.7%)	14.8 (11.2 to 18.6)
Ischemic heart disease	Jamaica	2000	Age-standardized		14.4% (11.1 to 17.9%)	12.5 (9.5 to 15.5)
Ischemic heart disease	Jamaica	2005	Age-standardized		14.0% (10.4 to 17.6%)	10.6 (7.8 to 13.6)
Ischemic heart disease	Jamaica	2010	Age-standardized		12.1% (8.9 to 15.4%)	10.0 (7.3 to 13.1)
Ischemic heart disease	Jamaica	2015	Age-standardized		13.2% (9.7 to 16.5%)	11.4 (8.3 to 14.9)
Ischemic stroke	Jamaica	1990	All Ages	0.1 (0.1 to 0.2)	10.2% (6.9 to 13.9%)	6.2 (4.2 to 8.6)
Ischemic stroke	Jamaica	1995	All Ages	0.1 (0.1 to 0.2)	10.0% (6.4 to 14.0%)	6.0 (3.9 to 8.6)
Ischemic stroke	Jamaica	2000	All Ages	0.1 (0.1 to 0.2)	10.0% (6.4 to 14.1%)	5.0 (3.3 to 7.2)
Ischemic stroke	Jamaica	2005	All Ages	0.1 (0.1 to 0.2)	9.7% (6.2 to 14.0%)	4.5 (2.8 to 6.6)
Ischemic stroke	Jamaica	2010	All Ages	0.1 (0.1 to 0.2)	8.1% (4.7 to 12.5%)	4.3 (2.4 to 6.6)
Ischemic stroke	Jamaica	2015	All Ages	0.1 (0.1 to 0.2)	9.1% (5.5 to 13.3%)	5.3 (3.2 to 8.0)
Ischemic stroke	Jamaica	1990	Age-standardized		10.4% (7.2 to 13.9%)	8.3 (5.7 to 11.3)
Ischemic stroke	Jamaica	1995	Age-standardized		10.2% (6.7 to 14.0%)	7.8 (5.2 to 10.7)
Ischemic stroke	Jamaica	2000	Age-standardized		10.2% (6.7 to 14.0%)	6.1 (4.1 to 8.5)
Ischemic stroke	Jamaica	2005	Age-standardized		9.9% (6.4 to 13.9%)	5.2 (3.4 to 7.5)
Ischemic stroke	Jamaica	2010	Age-standardized		8.3% (4.9 to 12.3%)	4.7 (2.8 to 7.1)
Ischemic stroke	Jamaica	2015	Age-standardized		9.2% (5.7 to 13.2%)	5.4 (3.3 to 7.9)
Hemorrhagic stroke	Jamaica	1990	All Ages	0.1 (0.1 to 0.1)	11.0% (8.3 to 13.7%)	4.7 (3.6 to 6.1)
Hemorrhagic stroke	Jamaica	1995	All Ages	0.1 (0.1 to 0.2)	10.9% (8.0 to 14.0%)	5.1 (3.7 to 6.7)
Hemorrhagic stroke	Jamaica	2000	All Ages	0.1 (0.1 to 0.2)	10.9% (7.9 to 14.1%)	5.3 (3.8 to 7.0)
Hemorrhagic stroke	Jamaica	2005	All Ages	0.1 (0.1 to 0.2)	10.6% (7.7 to 14.0%)	4.9 (3.5 to 6.6)
Hemorrhagic stroke	Jamaica	2010	All Ages	0.1 (0.1 to 0.2)	8.9% (5.9 to 12.4%)	4.7 (3.1 to 6.7)
Hemorrhagic stroke	Jamaica	2015	All Ages	0.2 (0.1 to 0.2)	9.9% (6.8 to 13.2%)	5.8 (4.0 to 8.0)
Hemorrhagic stroke	Jamaica	1990	Age-standardized		11.5% (8.8 to 14.3%)	6.7 (5.0 to 8.5)
Hemorrhagic stroke	Jamaica	1995	Age-standardized		11.3% (8.4 to 14.2%)	6.9 (5.1 to 8.9)
Hemorrhagic stroke	Jamaica	2000	Age-standardized		11.2% (8.2 to 14.3%)	6.7 (4.9 to 8.8)
Hemorrhagic stroke	Jamaica	2005	Age-standardized		10.8% (7.9 to 14.0%)	5.9 (4.2 to 7.8)
Hemorrhagic stroke	Jamaica	2010	Age-standardized		9.0% (6.2 to 12.2%)	5.3 (3.6 to 7.4)

Cause name	Location	Year	Age group	Number (in thousands)	PAF	Rate (per 100,000)
Hemorrhagic stroke	Jamaica	2015	Age-standardized		10.1% (7.1 to 13.2%)	6.0 (4.2 to 8.2)
Chronic obstructive pulmonary disease	Jamaica	1990	All Ages	0.1 (0.0 to 0.1)	18.0% (8.5 to 28.5%)	2.3 (1.0 to 3.7)
Chronic obstructive pulmonary disease	Jamaica	1995	All Ages	0.1 (0.0 to 0.1)	18.0% (8.6 to 28.6%)	2.7 (1.3 to 4.4)
Chronic obstructive pulmonary disease	Jamaica	2000	All Ages	0.1 (0.0 to 0.1)	18.1% (8.7 to 29.0%)	3.3 (1.6 to 5.3)
Chronic obstructive pulmonary disease	Jamaica	2005	All Ages	0.1 (0.0 to 0.1)	17.8% (8.3 to 28.3%)	3.1 (1.5 to 5.1)
Chronic obstructive pulmonary disease	Jamaica	2010	All Ages	0.1 (0.0 to 0.1)	13.9% (5.5 to 24.0%)	2.6 (1.1 to 4.6)
Chronic obstructive pulmonary disease	Jamaica	2015	All Ages	0.1 (0.0 to 0.2)	16.2% (7.3 to 26.8%)	3.4 (1.5 to 5.7)
Chronic obstructive pulmonary disease	Jamaica	1990	Age-standardized		18.2% (8.6 to 28.8%)	3.1 (1.4 to 4.9)
Chronic obstructive pulmonary disease	Jamaica	1995	Age-standardized		18.2% (8.7 to 28.8%)	3.5 (1.6 to 5.7)
Chronic obstructive pulmonary disease	Jamaica	2000	Age-standardized		18.2% (8.7 to 29.1%)	4.1 (2.0 to 6.6)
Chronic obstructive pulmonary disease	Jamaica	2005	Age-standardized		17.9% (8.3 to 28.4%)	3.7 (1.7 to 5.9)
Chronic obstructive pulmonary disease	Jamaica	2010	Age-standardized		13.9% (5.5 to 24.1%)	2.9 (1.2 to 5.1)
Chronic obstructive pulmonary disease	Jamaica	2015	Age-standardized		16.2% (7.3 to 26.8%)	3.5 (1.6 to 5.8)
All causes	Puerto Rico	1990	All Ages	1.5 (0.8 to 2.2)	5.4% (3.0 to 8.1%)	41.3 (22.7 to 62.6)
All causes	Puerto Rico	1995	All Ages	1.6 (0.8 to 2.4)	5.0% (2.7 to 7.5%)	43.3 (23.0 to 64.6)
All causes	Puerto Rico	2000	All Ages	1.8 (1.0 to 2.8)	5.8% (3.0 to 9.0%)	48.0 (25.1 to 74.6)
All causes	Puerto Rico	2005	All Ages	1.6 (0.9 to 2.5)	5.0% (2.8 to 7.6%)	43.5 (23.7 to 65.2)
All causes	Puerto Rico	2010	All Ages	1.5 (0.7 to 2.5)	4.5% (2.0 to 7.3%)	41.0 (18.1 to 66.6)
All causes	Puerto Rico	2015	All Ages	1.5 (0.8 to 2.4)	4.6% (2.3 to 7.2%)	41.5 (20.9 to 64.4)
All causes	Puerto Rico	1990	Age-standardized		5.4% (3.0 to 8.2%)	43.6 (24.0 to 66.0)
All causes	Puerto Rico	1995	Age-standardized		5.0% (2.7 to 7.4%)	41.1 (22.0 to 61.2)
All causes	Puerto Rico	2000	Age-standardized		5.6% (3.0 to 8.8%)	41.5 (21.7 to 64.3)
All causes	Puerto Rico	2005	Age-standardized		4.9% (2.7 to 7.3%)	34.5 (18.8 to 51.8)
All causes	Puerto Rico	2010	Age-standardized		4.4% (1.9 to 7.0%)	30.2 (13.4 to 48.6)
All causes	Puerto Rico	2015	Age-standardized		4.4% (2.2 to 6.9%)	28.0 (13.8 to 43.2)
Lower respiratory infections	Puerto Rico	1990	All Ages	0.3 (0.0 to 0.5)	15.6% (2.7 to 30.8%)	7.0 (1.2 to 13.9)
Lower respiratory infections	Puerto Rico	1995	All Ages	0.3 (0.1 to 0.6)	15.5% (2.8 to 31.1%)	7.4 (1.3 to 14.9)
Lower respiratory infections	Puerto Rico	2000	All Ages	0.4 (0.1 to 0.7)	18.9% (3.5 to 37.0%)	9.4 (1.8 to 18.4)
Lower respiratory infections	Puerto Rico	2005	All Ages	0.3 (0.1 to 0.6)	15.5% (3.0 to 30.9%)	8.4 (1.5 to 16.7)
Lower respiratory infections	Puerto Rico	2010	All Ages	0.3 (0.0 to 0.6)	14.0% (1.7 to 29.8%)	7.8 (0.9 to 16.6)
Lower respiratory infections	Puerto Rico	2015	All Ages	0.3 (0.1 to 0.6)	14.6% (2.4 to 29.7%)	8.1 (1.4 to 16.2)
Lower respiratory infections	Puerto Rico	1990	Age-standardized		15.6% (2.7 to 30.8%)	7.3 (1.3 to 14.5)
Lower respiratory infections	Puerto Rico	1995	Age-standardized		15.5% (2.8 to 31.1%)	6.9 (1.2 to 14.0)
Lower respiratory infections	Puerto Rico	2000	Age-standardized		18.9% (3.5 to 37.0%)	8.0 (1.6 to 15.7)
Lower respiratory infections	Puerto Rico	2005	Age-standardized		15.5% (3.0 to 30.9%)	6.6 (1.2 to 13.1)
Lower respiratory infections	Puerto Rico	2010	Age-standardized		14.0% (1.7 to 29.8%)	5.6 (0.7 to 12.0)

Cause name	Location	Year	Age group	Number (in thousands)	PAF	Rate (per 100,000)
Lower respiratory infections	Puerto Rico	2015	Age-standardized		14.6% (2.4 to 29.7%)	5.4 (0.9 to 10.8)
Tracheal, bronchus, and lung cancer	Puerto Rico	1990	All Ages	0.1 (0.0 to 0.1)	11.0% (2.8 to 22.9%)	1.7 (0.4 to 3.6)
Tracheal, bronchus, and lung cancer	Puerto Rico	1995	All Ages	0.1 (0.0 to 0.1)	10.8% (2.8 to 22.8%)	2.0 (0.5 to 4.2)
Tracheal, bronchus, and lung cancer	Puerto Rico	2000	All Ages	0.1 (0.0 to 0.2)	13.4% (3.4 to 27.8%)	2.4 (0.6 to 5.1)
Tracheal, bronchus, and lung cancer	Puerto Rico	2005	All Ages	0.1 (0.0 to 0.2)	10.9% (2.8 to 22.1%)	2.1 (0.5 to 4.2)
Tracheal, bronchus, and lung cancer	Puerto Rico	2010	All Ages	0.1 (0.0 to 0.2)	9.8% (2.1 to 21.4%)	2.1 (0.4 to 4.6)
Tracheal, bronchus, and lung cancer	Puerto Rico	2015	All Ages	0.1 (0.0 to 0.2)	10.2% (2.5 to 21.8%)	2.2 (0.5 to 4.5)
Tracheal, bronchus, and lung cancer	Puerto Rico	1990	Age-standardized		11.0% (2.8 to 22.9%)	1.9 (0.5 to 3.9)
Tracheal, bronchus, and lung cancer	Puerto Rico	1995	Age-standardized		10.8% (2.8 to 22.8%)	1.9 (0.5 to 4.1)
Tracheal, bronchus, and lung cancer	Puerto Rico	2000	Age-standardized		13.4% (3.4 to 27.8%)	2.2 (0.6 to 4.6)
Tracheal, bronchus, and lung cancer	Puerto Rico	2005	Age-standardized		10.8% (2.8 to 22.1%)	1.7 (0.4 to 3.4)
Tracheal, bronchus, and lung cancer	Puerto Rico	2010	Age-standardized		9.8% (2.1 to 21.4%)	1.6 (0.3 to 3.5)
Tracheal, bronchus, and lung cancer	Puerto Rico	2015	Age-standardized		10.2% (2.5 to 21.8%)	1.5 (0.4 to 3.2)
Cardiovascular diseases	Puerto Rico	1990	All Ages	1.0 (0.6 to 1.4)	11.2% (6.9 to 15.6%)	28.7 (17.7 to 40.1)
Cardiovascular diseases	Puerto Rico	1995	All Ages	1.1 (0.6 to 1.4)	10.6% (6.5 to 14.7%)	28.4 (17.2 to 39.1)
Cardiovascular diseases	Puerto Rico	2000	All Ages	1.1 (0.7 to 1.6)	11.2% (6.8 to 16.0%)	29.1 (17.5 to 41.6)
Cardiovascular diseases	Puerto Rico	2005	All Ages	1.0 (0.6 to 1.4)	10.0% (6.1 to 14.1%)	26.4 (16.0 to 37.4)
Cardiovascular diseases	Puerto Rico	2010	All Ages	0.9 (0.5 to 1.4)	9.2% (5.0 to 13.5%)	24.5 (13.4 to 36.3)
Cardiovascular diseases	Puerto Rico	2015	All Ages	0.9 (0.5 to 1.3)	9.3% (5.2 to 13.3%)	24.6 (13.7 to 35.9)
Cardiovascular diseases	Puerto Rico	1990	Age-standardized		11.3% (7.0 to 15.6%)	30.4 (18.8 to 42.3)
Cardiovascular diseases	Puerto Rico	1995	Age-standardized		10.7% (6.6 to 14.8%)	27.2 (16.5 to 37.2)
Cardiovascular diseases	Puerto Rico	2000	Age-standardized		11.4% (6.9 to 16.1%)	25.3 (15.4 to 35.8)
Cardiovascular diseases	Puerto Rico	2005	Age-standardized		10.2% (6.2 to 14.2%)	21.2 (12.9 to 29.6)
Cardiovascular diseases	Puerto Rico	2010	Age-standardized		9.4% (5.2 to 13.7%)	18.2 (9.8 to 26.6)
Cardiovascular diseases	Puerto Rico	2015	Age-standardized		9.5% (5.5 to 13.6%)	16.8 (9.6 to 23.8)
Ischemic heart disease	Puerto Rico	1990	All Ages	0.8 (0.5 to 1.2)	13.8% (8.5 to 19.5%)	23.8 (14.6 to 33.3)
Ischemic heart disease	Puerto Rico	1995	All Ages	0.8 (0.5 to 1.2)	13.6% (8.3 to 19.0%)	22.6 (13.8 to 31.9)
Ischemic heart disease	Puerto Rico	2000	All Ages	0.9 (0.5 to 1.2)	14.5% (8.8 to 20.9%)	22.6 (13.8 to 32.6)
Ischemic heart disease	Puerto Rico	2005	All Ages	0.8 (0.5 to 1.1)	13.2% (7.9 to 18.6%)	20.5 (12.3 to 29.1)
Ischemic heart disease	Puerto Rico	2010	All Ages	0.7 (0.4 to 1.0)	12.4% (6.5 to 18.4%)	18.9 (9.8 to 27.9)
Ischemic heart disease	Puerto Rico	2015	All Ages	0.7 (0.4 to 1.0)	12.5% (6.8 to 18.3%)	19.2 (10.4 to 27.7)
Ischemic heart disease	Puerto Rico	1990	Age-standardized		13.9% (8.6 to 19.5%)	25.3 (15.5 to 35.2)
Ischemic heart disease	Puerto Rico	1995	Age-standardized		13.8% (8.5 to 19.3%)	21.7 (13.3 to 30.5)
Ischemic heart disease	Puerto Rico	2000	Age-standardized		14.8% (9.1 to 21.1%)	19.7 (12.1 to 28.1)
Ischemic heart disease	Puerto Rico	2005	Age-standardized		13.5% (8.2 to 18.7%)	16.5 (10.1 to 23.1)
Ischemic heart disease	Puerto Rico	2010	Age-standardized		12.8% (6.9 to 18.7%)	14.1 (7.4 to 20.7)

Cause name	Location	Year	Age group	Number (in thousands)	PAF	Rate (per 100,000)
Ischemic heart disease	Puerto Rico	2015	Age-standardized		12.9% (7.2 to 18.4%)	13.1 (7.1 to 18.8)
Ischemic stroke	Puerto Rico	1990	All Ages	0.1 (0.0 to 0.2)	9.5% (4.5 to 14.9%)	2.8 (1.3 to 4.4)
Ischemic stroke	Puerto Rico	1995	All Ages	0.1 (0.1 to 0.2)	9.3% (4.2 to 14.8%)	2.9 (1.3 to 4.7)
Ischemic stroke	Puerto Rico	2000	All Ages	0.1 (0.1 to 0.2)	10.3% (4.6 to 16.7%)	3.0 (1.4 to 5.0)
Ischemic stroke	Puerto Rico	2005	All Ages	0.1 (0.1 to 0.2)	9.1% (4.0 to 14.9%)	2.7 (1.2 to 4.5)
Ischemic stroke	Puerto Rico	2010	All Ages	0.1 (0.0 to 0.2)	8.5% (3.3 to 14.9%)	2.6 (1.0 to 4.5)
Ischemic stroke	Puerto Rico	2015	All Ages	0.1 (0.0 to 0.2)	8.7% (3.4 to 14.5%)	2.5 (1.0 to 4.3)
Ischemic stroke	Puerto Rico	1990	Age-standardized		9.6% (4.5 to 15.0%)	2.9 (1.3 to 4.6)
Ischemic stroke	Puerto Rico	1995	Age-standardized		9.4% (4.4 to 14.9%)	2.7 (1.2 to 4.3)
Ischemic stroke	Puerto Rico	2000	Age-standardized		10.4% (4.8 to 16.8%)	2.6 (1.2 to 4.1)
Ischemic stroke	Puerto Rico	2005	Age-standardized		9.2% (4.2 to 15.0%)	2.1 (1.0 to 3.4)
Ischemic stroke	Puerto Rico	2010	Age-standardized		8.7% (3.4 to 14.8%)	1.8 (0.7 to 3.1)
Ischemic stroke	Puerto Rico	2015	Age-standardized		8.8% (3.6 to 14.6%)	1.6 (0.7 to 2.7)
Hemorrhagic stroke	Puerto Rico	1990	All Ages	0.1 (0.0 to 0.1)	10.8% (5.8 to 15.8%)	2.0 (1.1 to 3.0)
Hemorrhagic stroke	Puerto Rico	1995	All Ages	0.1 (0.1 to 0.2)	10.5% (5.2 to 15.8%)	2.9 (1.4 to 4.4)
Hemorrhagic stroke	Puerto Rico	2000	All Ages	0.1 (0.1 to 0.2)	11.5% (5.7 to 17.9%)	3.5 (1.7 to 5.4)
Hemorrhagic stroke	Puerto Rico	2005	All Ages	0.1 (0.1 to 0.2)	10.1% (5.2 to 15.4%)	3.2 (1.6 to 4.9)
Hemorrhagic stroke	Puerto Rico	2010	All Ages	0.1 (0.1 to 0.2)	9.4% (4.0 to 15.3%)	3.1 (1.3 to 5.0)
Hemorrhagic stroke	Puerto Rico	2015	All Ages	0.1 (0.1 to 0.2)	9.5% (4.3 to 15.1%)	3.0 (1.3 to 4.7)
Hemorrhagic stroke	Puerto Rico	1990	Age-standardized		10.9% (5.8 to 15.9%)	2.2 (1.1 to 3.2)
Hemorrhagic stroke	Puerto Rico	1995	Age-standardized		10.6% (5.4 to 15.9%)	2.8 (1.4 to 4.2)
Hemorrhagic stroke	Puerto Rico	2000	Age-standardized		11.7% (6.0 to 18.1%)	3.0 (1.5 to 4.7)
Hemorrhagic stroke	Puerto Rico	2005	Age-standardized		10.3% (5.5 to 15.5%)	2.6 (1.3 to 3.9)
Hemorrhagic stroke	Puerto Rico	2010	Age-standardized		9.6% (4.3 to 15.4%)	2.3 (1.0 to 3.7)
Hemorrhagic stroke	Puerto Rico	2015	Age-standardized		9.8% (4.7 to 15.3%)	2.1 (1.0 to 3.2)
Chronic obstructive pulmonary disease	Puerto Rico	1990	All Ages	0.1 (0.0 to 0.3)	17.0% (5.0 to 31.5%)	4.0 (1.1 to 7.5)
Chronic obstructive pulmonary disease	Puerto Rico	1995	All Ages	0.2 (0.1 to 0.4)	16.9% (5.2 to 31.7%)	5.5 (1.7 to 10.4)
Chronic obstructive pulmonary disease	Puerto Rico	2000	All Ages	0.3 (0.1 to 0.5)	19.6% (5.5 to 36.1%)	7.1 (2.0 to 13.3)
Chronic obstructive pulmonary disease	Puerto Rico	2005	All Ages	0.3 (0.1 to 0.5)	16.9% (4.8 to 31.4%)	6.7 (1.9 to 12.5)
Chronic obstructive pulmonary disease	Puerto Rico	2010	All Ages	0.3 (0.1 to 0.5)	15.7% (3.2 to 30.5%)	6.7 (1.4 to 13.2)
Chronic obstructive pulmonary disease	Puerto Rico	2015	All Ages	0.2 (0.1 to 0.5)	16.1% (4.1 to 30.3%)	6.6 (1.7 to 12.6)
Chronic obstructive pulmonary disease	Puerto Rico	1990	Age-standardized		17.0% (5.0 to 31.5%)	4.1 (1.2 to 7.8)
Chronic obstructive pulmonary disease	Puerto Rico	1995	Age-standardized		16.9% (5.2 to 31.7%)	5.1 (1.6 to 9.7)
Chronic obstructive pulmonary disease	Puerto Rico	2000	Age-standardized		19.6% (5.5 to 36.1%)	6.0 (1.7 to 11.2)
Chronic obstructive pulmonary disease	Puerto Rico	2005	Age-standardized		16.9% (4.8 to 31.4%)	5.1 (1.4 to 9.5)
Chronic obstructive pulmonary disease	Puerto Rico	2010	Age-standardized		15.6% (3.2 to 30.5%)	4.7 (1.0 to 9.3)

Cause name	Location	Year	Age group	Number (in thousands)	PAF	Rate (per 100,000)
Chronic obstructive pulmonary disease	Puerto Rico	2015	Age-standardized		16.1% (4.1 to 30.2%)	4.3 (1.1 to 8.1)
All causes	Saint Lucia	1990	All Ages	0.1 (0.0 to 0.1)	4.9% (3.7 to 6.2%)	36.5 (27.6 to 46.2)
All causes	Saint Lucia	1995	All Ages	0.1 (0.0 to 0.1)	4.8% (3.6 to 6.1%)	37.5 (28.2 to 47.0)
All causes	Saint Lucia	2000	All Ages	0.1 (0.0 to 0.1)	4.6% (3.3 to 5.9%)	35.1 (24.9 to 45.7)
All causes	Saint Lucia	2005	All Ages	0.1 (0.0 to 0.1)	4.2% (3.0 to 5.5%)	30.7 (21.5 to 40.5)
All causes	Saint Lucia	2010	All Ages	0.1 (0.0 to 0.1)	3.7% (2.4 to 5.0%)	27.3 (17.6 to 37.1)
All causes	Saint Lucia	2015	All Ages	0.1 (0.0 to 0.1)	3.7% (2.6 to 5.0%)	29.7 (20.0 to 39.7)
All causes	Saint Lucia	1990	Age-standardized		5.3% (4.0 to 6.7%)	53.6 (39.9 to 67.9)
All causes	Saint Lucia	1995	Age-standardized		5.0% (3.8 to 6.3%)	49.8 (37.8 to 62.2)
All causes	Saint Lucia	2000	Age-standardized		4.7% (3.4 to 6.1%)	44.2 (31.8 to 57.4)
All causes	Saint Lucia	2005	Age-standardized		4.4% (3.1 to 5.7%)	38.4 (27.5 to 50.2)
All causes	Saint Lucia	2010	Age-standardized		3.7% (2.5 to 5.0%)	29.7 (19.3 to 40.2)
All causes	Saint Lucia	2015	Age-standardized		3.8% (2.6 to 5.1%)	30.6 (20.8 to 40.8)
Lower respiratory infections	Saint Lucia	1990	All Ages	0.0 (0.0 to 0.0)	15.8% (6.2 to 26.5%)	6.7 (2.6 to 11.5)
Lower respiratory infections	Saint Lucia	1995	All Ages	0.0 (0.0 to 0.0)	15.8% (5.6 to 27.0%)	6.5 (2.3 to 10.9)
Lower respiratory infections	Saint Lucia	2000	All Ages	0.0 (0.0 to 0.0)	16.0% (6.2 to 27.2%)	6.8 (2.6 to 11.6)
Lower respiratory infections	Saint Lucia	2005	All Ages	0.0 (0.0 to 0.0)	14.3% (4.8 to 25.3%)	6.0 (2.1 to 11.1)
Lower respiratory infections	Saint Lucia	2010	All Ages	0.0 (0.0 to 0.0)	12.2% (3.5 to 23.7%)	5.1 (1.5 to 10.0)
Lower respiratory infections	Saint Lucia	2015	All Ages	0.0 (0.0 to 0.0)	12.0% (3.6 to 23.7%)	5.1 (1.6 to 10.4)
Lower respiratory infections	Saint Lucia	1990	Age-standardized		15.8% (6.2 to 26.5%)	8.7 (3.4 to 14.8)
Lower respiratory infections	Saint Lucia	1995	Age-standardized		15.8% (5.6 to 27.0%)	7.9 (2.8 to 13.3)
Lower respiratory infections	Saint Lucia	2000	Age-standardized		16.0% (6.2 to 27.2%)	7.9 (3.0 to 13.4)
Lower respiratory infections	Saint Lucia	2005	Age-standardized		14.3% (4.8 to 25.3%)	6.9 (2.4 to 12.7)
Lower respiratory infections	Saint Lucia	2010	Age-standardized		12.2% (3.5 to 23.7%)	5.4 (1.6 to 10.5)
Lower respiratory infections	Saint Lucia	2015	Age-standardized		12.0% (3.6 to 23.7%)	5.2 (1.6 to 10.6)
Tracheal, bronchus, and lung cancer	Saint Lucia	1990	All Ages	0.0 (0.0 to 0.0)	11.0% (5.2 to 18.1%)	1.0 (0.5 to 1.7)
Tracheal, bronchus, and lung cancer	Saint Lucia	1995	All Ages	0.0 (0.0 to 0.0)	11.0% (5.1 to 18.3%)	1.1 (0.5 to 1.8)
Tracheal, bronchus, and lung cancer	Saint Lucia	2000	All Ages	0.0 (0.0 to 0.0)	11.1% (5.0 to 18.9%)	1.1 (0.5 to 1.9)
Tracheal, bronchus, and lung cancer	Saint Lucia	2005	All Ages	0.0 (0.0 to 0.0)	9.8% (4.2 to 17.1%)	0.9 (0.4 to 1.6)
Tracheal, bronchus, and lung cancer	Saint Lucia	2010	All Ages	0.0 (0.0 to 0.0)	8.4% (3.4 to 15.0%)	0.8 (0.3 to 1.5)
Tracheal, bronchus, and lung cancer	Saint Lucia	2015	All Ages	0.0 (0.0 to 0.0)	8.2% (3.5 to 14.7%)	1.0 (0.4 to 1.7)
Tracheal, bronchus, and lung cancer	Saint Lucia	1990	Age-standardized		11.0% (5.2 to 18.2%)	1.4 (0.7 to 2.4)
Tracheal, bronchus, and lung cancer	Saint Lucia	1995	Age-standardized		11.0% (5.2 to 18.4%)	1.5 (0.7 to 2.5)
Tracheal, bronchus, and lung cancer	Saint Lucia	2000	Age-standardized		11.1% (5.0 to 18.9%)	1.5 (0.7 to 2.6)
Tracheal, bronchus, and lung cancer	Saint Lucia	2005	Age-standardized		9.9% (4.3 to 17.2%)	1.2 (0.5 to 2.2)
Tracheal, bronchus, and lung cancer	Saint Lucia	2010	Age-standardized		8.4% (3.4 to 15.0%)	0.9 (0.4 to 1.7)

Cause name	Location	Year	Age group	Number (in thousands)	PAF	Rate (per 100,000)
Tracheal, bronchus, and lung cancer	Saint Lucia	2015	Age-standardized		8.2% (3.5 to 14.7%)	1.0 (0.4 to 1.8)
Cardiovascular diseases	Saint Lucia	1990	All Ages	0.0 (0.0 to 0.0)	9.5% (7.4 to 11.8%)	25.7 (19.5 to 32.0)
Cardiovascular diseases	Saint Lucia	1995	All Ages	0.0 (0.0 to 0.1)	9.4% (7.3 to 11.6%)	26.4 (20.4 to 32.5)
Cardiovascular diseases	Saint Lucia	2000	All Ages	0.0 (0.0 to 0.1)	9.0% (6.6 to 11.6%)	23.3 (17.1 to 30.1)
Cardiovascular diseases	Saint Lucia	2005	All Ages	0.0 (0.0 to 0.0)	8.4% (6.2 to 10.9%)	20.5 (14.7 to 26.7)
Cardiovascular diseases	Saint Lucia	2010	All Ages	0.0 (0.0 to 0.0)	7.5% (5.1 to 9.9%)	18.8 (12.7 to 25.2)
Cardiovascular diseases	Saint Lucia	2015	All Ages	0.0 (0.0 to 0.1)	7.5% (5.4 to 9.7%)	20.7 (14.6 to 26.9)
Cardiovascular diseases	Saint Lucia	1990	Age-standardized		9.5% (7.2 to 11.9%)	38.6 (29.2 to 48.6)
Cardiovascular diseases	Saint Lucia	1995	Age-standardized		9.6% (7.5 to 11.8%)	35.7 (27.8 to 43.8)
Cardiovascular diseases	Saint Lucia	2000	Age-standardized		9.3% (7.0 to 11.9%)	30.0 (22.1 to 38.4)
Cardiovascular diseases	Saint Lucia	2005	Age-standardized		8.6% (6.4 to 11.0%)	26.0 (18.9 to 33.5)
Cardiovascular diseases	Saint Lucia	2010	Age-standardized		7.6% (5.3 to 9.9%)	20.5 (14.1 to 27.3)
Cardiovascular diseases	Saint Lucia	2015	Age-standardized		7.5% (5.4 to 9.7%)	21.4 (15.1 to 27.7)
Ischemic heart disease	Saint Lucia	1990	All Ages	0.0 (0.0 to 0.0)	14.1% (10.7 to 18.0%)	14.7 (11.0 to 18.8)
Ischemic heart disease	Saint Lucia	1995	All Ages	0.0 (0.0 to 0.0)	13.7% (10.3 to 17.3%)	15.1 (11.4 to 19.2)
Ischemic heart disease	Saint Lucia	2000	All Ages	0.0 (0.0 to 0.0)	13.5% (9.6 to 17.7%)	12.9 (9.1 to 17.0)
Ischemic heart disease	Saint Lucia	2005	All Ages	0.0 (0.0 to 0.0)	12.8% (8.9 to 16.7%)	11.8 (8.2 to 15.7)
Ischemic heart disease	Saint Lucia	2010	All Ages	0.0 (0.0 to 0.0)	12.1% (8.1 to 16.0%)	10.5 (7.0 to 14.2)
Ischemic heart disease	Saint Lucia	2015	All Ages	0.0 (0.0 to 0.0)	12.0% (8.2 to 15.8%)	11.7 (7.9 to 15.4)
Ischemic heart disease	Saint Lucia	1990	Age-standardized		13.9% (10.4 to 17.8%)	22.4 (16.5 to 28.8)
Ischemic heart disease	Saint Lucia	1995	Age-standardized		13.9% (10.5 to 17.6%)	20.5 (15.5 to 26.0)
Ischemic heart disease	Saint Lucia	2000	Age-standardized		13.9% (10.1 to 17.9%)	16.6 (12.1 to 21.5)
Ischemic heart disease	Saint Lucia	2005	Age-standardized		13.1% (9.4 to 16.8%)	15.0 (10.7 to 19.6)
Ischemic heart disease	Saint Lucia	2010	Age-standardized		12.2% (8.2 to 16.0%)	11.5 (7.7 to 15.2)
Ischemic heart disease	Saint Lucia	2015	Age-standardized		12.0% (8.3 to 15.6%)	12.0 (8.2 to 15.8)
Ischemic stroke	Saint Lucia	1990	All Ages	0.0 (0.0 to 0.0)	10.1% (6.6 to 13.7%)	5.9 (3.8 to 8.0)
Ischemic stroke	Saint Lucia	1995	All Ages	0.0 (0.0 to 0.0)	9.8% (6.3 to 13.6%)	6.0 (3.9 to 8.5)
Ischemic stroke	Saint Lucia	2000	All Ages	0.0 (0.0 to 0.0)	9.5% (5.7 to 14.1%)	5.1 (3.0 to 7.6)
Ischemic stroke	Saint Lucia	2005	All Ages	0.0 (0.0 to 0.0)	8.9% (5.0 to 13.2%)	4.1 (2.4 to 6.3)
Ischemic stroke	Saint Lucia	2010	All Ages	0.0 (0.0 to 0.0)	8.2% (4.5 to 12.5%)	3.9 (2.2 to 6.0)
Ischemic stroke	Saint Lucia	2015	All Ages	0.0 (0.0 to 0.0)	8.1% (4.4 to 12.4%)	4.2 (2.3 to 6.5)
Ischemic stroke	Saint Lucia	1990	Age-standardized		9.9% (6.4 to 13.9%)	8.8 (5.7 to 12.3)
Ischemic stroke	Saint Lucia	1995	Age-standardized		9.9% (6.4 to 13.7%)	8.0 (5.2 to 11.1)
Ischemic stroke	Saint Lucia	2000	Age-standardized		9.7% (5.9 to 14.0%)	6.3 (3.9 to 9.1)
Ischemic stroke	Saint Lucia	2005	Age-standardized		9.1% (5.4 to 13.2%)	5.2 (3.1 to 7.6)
Ischemic stroke	Saint Lucia	2010	Age-standardized		8.3% (4.7 to 12.4%)	4.2 (2.4 to 6.5)

Cause name	Location	Year	Age group	Number (in thousands)	PAF	Rate (per 100,000)
Ischemic stroke	Saint Lucia	2015	Age-standardized		8.2% (4.6 to 12.3%)	4.3 (2.4 to 6.6)
Hemorrhagic stroke	Saint Lucia	1990	All Ages	0.0 (0.0 to 0.0)	10.9% (7.6 to 14.2%)	5.0 (3.5 to 6.6)
Hemorrhagic stroke	Saint Lucia	1995	All Ages	0.0 (0.0 to 0.0)	10.7% (7.5 to 14.1%)	5.2 (3.6 to 7.0)
Hemorrhagic stroke	Saint Lucia	2000	All Ages	0.0 (0.0 to 0.0)	10.4% (6.8 to 14.3%)	5.4 (3.5 to 7.6)
Hemorrhagic stroke	Saint Lucia	2005	All Ages	0.0 (0.0 to 0.0)	9.7% (6.3 to 13.7%)	4.5 (2.9 to 6.4)
Hemorrhagic stroke	Saint Lucia	2010	All Ages	0.0 (0.0 to 0.0)	9.0% (5.4 to 12.9%)	4.4 (2.6 to 6.4)
Hemorrhagic stroke	Saint Lucia	2015	All Ages	0.0 (0.0 to 0.0)	8.9% (5.6 to 12.5%)	4.9 (3.0 to 6.9)
Hemorrhagic stroke	Saint Lucia	1990	Age-standardized		11.0% (7.7 to 14.3%)	7.4 (5.2 to 9.8)
Hemorrhagic stroke	Saint Lucia	1995	Age-standardized		10.9% (7.7 to 14.3%)	7.1 (5.0 to 9.5)
Hemorrhagic stroke	Saint Lucia	2000	Age-standardized		10.7% (7.1 to 14.5%)	7.1 (4.8 to 9.9)
Hemorrhagic stroke	Saint Lucia	2005	Age-standardized		10.0% (6.5 to 13.7%)	5.8 (3.8 to 8.1)
Hemorrhagic stroke	Saint Lucia	2010	Age-standardized		9.0% (5.5 to 12.8%)	4.8 (2.9 to 7.0)
Hemorrhagic stroke	Saint Lucia	2015	Age-standardized		8.9% (5.5 to 12.4%)	5.0 (3.1 to 7.2)
Chronic obstructive pulmonary disease	Saint Lucia	1990	All Ages	0.0 (0.0 to 0.0)	16.3% (7.1 to 27.4%)	3.2 (1.4 to 5.3)
Chronic obstructive pulmonary disease	Saint Lucia	1995	All Ages	0.0 (0.0 to 0.0)	16.8% (7.5 to 27.5%)	3.6 (1.6 to 5.9)
Chronic obstructive pulmonary disease	Saint Lucia	2000	All Ages	0.0 (0.0 to 0.0)	17.0% (6.7 to 29.0%)	3.8 (1.5 to 6.5)
Chronic obstructive pulmonary disease	Saint Lucia	2005	All Ages	0.0 (0.0 to 0.0)	15.6% (6.1 to 26.3%)	3.3 (1.3 to 5.7)
Chronic obstructive pulmonary disease	Saint Lucia	2010	All Ages	0.0 (0.0 to 0.0)	13.9% (4.8 to 24.7%)	2.6 (0.9 to 4.7)
Chronic obstructive pulmonary disease	Saint Lucia	2015	All Ages	0.0 (0.0 to 0.0)	13.8% (5.2 to 24.3%)	2.8 (1.1 to 5.1)
Chronic obstructive pulmonary disease	Saint Lucia	1990	Age-standardized		16.8% (7.2 to 28.0%)	4.8 (2.1 to 7.9)
Chronic obstructive pulmonary disease	Saint Lucia	1995	Age-standardized		17.0% (7.5 to 27.8%)	4.8 (2.1 to 7.8)
Chronic obstructive pulmonary disease	Saint Lucia	2000	Age-standardized		17.1% (6.8 to 29.1%)	4.8 (1.9 to 8.1)
Chronic obstructive pulmonary disease	Saint Lucia	2005	Age-standardized		15.6% (6.1 to 26.5%)	4.1 (1.6 to 7.1)
Chronic obstructive pulmonary disease	Saint Lucia	2010	Age-standardized		13.9% (4.8 to 24.7%)	2.9 (1.0 to 5.2)
Chronic obstructive pulmonary disease	Saint Lucia	2015	Age-standardized		13.8% (5.2 to 24.3%)	2.9 (1.1 to 5.3)
All causes	Saint Vincent and the Grenadines	1990	All Ages	0.0 (0.0 to 0.1)	4.7% (3.2 to 6.1%)	32.2 (22.3 to 42.7)
All causes	Saint Vincent and the Grenadines	1995	All Ages	0.0 (0.0 to 0.1)	4.7% (3.2 to 6.1%)	35.1 (23.9 to 46.2)
All causes	Saint Vincent and the Grenadines	2000	All Ages	0.0 (0.0 to 0.1)	4.4% (3.2 to 5.8%)	36.4 (26.1 to 47.7)
All causes	Saint Vincent and the Grenadines	2005	All Ages	0.0 (0.0 to 0.1)	4.2% (3.0 to 5.5%)	33.0 (23.4 to 43.7)
All causes	Saint Vincent and the Grenadines	2010	All Ages	0.0 (0.0 to 0.0)	3.8% (2.5 to 5.1%)	29.5 (19.2 to 39.9)
All causes	Saint Vincent and the Grenadines	2015	All Ages	0.0 (0.0 to 0.1)	3.9% (2.6 to 5.3%)	32.7 (22.1 to 44.6)
All causes	Saint Vincent and the Grenadines	1990	Age-standardized		5.1% (3.6 to 6.7%)	52.5 (36.8 to 69.5)
All causes	Saint Vincent and the Grenadines	1995	Age-standardized		5.0% (3.5 to 6.5%)	54.3 (37.4 to 71.1)
All causes	Saint Vincent and the Grenadines	2000	Age-standardized		4.7% (3.4 to 6.1%)	52.2 (37.6 to 68.3)
All causes	Saint Vincent and the Grenadines	2005	Age-standardized		4.4% (3.1 to 5.8%)	46.3 (33.0 to 61.2)
All causes	Saint Vincent and the Grenadines	2010	Age-standardized		4.0% (2.6 to 5.4%)	41.5 (26.7 to 56.2)

Cause name	Location	Year	Age group	Number (in thousands)	PAF	Rate (per 100,000)
All causes	Saint Vincent and the Grenadines	2015	Age-standardized		4.0% (2.7 to 5.5%)	40.7 (27.2 to 55.4)
Lower respiratory infections	Saint Vincent and the Grenadines	1990	All Ages	0.0 (0.0 to 0.0)	15.7% (5.7 to 27.7%)	5.9 (2.1 to 10.5)
Lower respiratory infections	Saint Vincent and the Grenadines	1995	All Ages	0.0 (0.0 to 0.0)	15.9% (6.0 to 28.0%)	6.2 (2.3 to 10.9)
Lower respiratory infections	Saint Vincent and the Grenadines	2000	All Ages	0.0 (0.0 to 0.0)	15.9% (5.4 to 27.2%)	6.4 (2.1 to 11.3)
Lower respiratory infections	Saint Vincent and the Grenadines	2005	All Ages	0.0 (0.0 to 0.0)	13.9% (4.4 to 25.9%)	5.3 (1.6 to 9.9)
Lower respiratory infections	Saint Vincent and the Grenadines	2010	All Ages	0.0 (0.0 to 0.0)	11.7% (2.9 to 23.3%)	4.3 (1.0 to 8.8)
Lower respiratory infections	Saint Vincent and the Grenadines	2015	All Ages	0.0 (0.0 to 0.0)	12.2% (3.1 to 24.4%)	4.6 (1.2 to 9.3)
Lower respiratory infections	Saint Vincent and the Grenadines	1990	Age-standardized		15.7% (5.7 to 27.7%)	8.1 (2.9 to 14.3)
Lower respiratory infections	Saint Vincent and the Grenadines	1995	Age-standardized		15.9% (6.0 to 28.0%)	8.6 (3.1 to 15.1)
Lower respiratory infections	Saint Vincent and the Grenadines	2000	Age-standardized		15.9% (5.4 to 27.2%)	8.5 (2.9 to 14.9)
Lower respiratory infections	Saint Vincent and the Grenadines	2005	Age-standardized		13.9% (4.4 to 25.9%)	7.2 (2.2 to 13.3)
Lower respiratory infections	Saint Vincent and the Grenadines	2010	Age-standardized		11.7% (2.9 to 23.3%)	6.0 (1.5 to 12.3)
Lower respiratory infections	Saint Vincent and the Grenadines	2015	Age-standardized		12.2% (3.1 to 24.4%)	5.8 (1.5 to 11.9)
Tracheal, bronchus, and lung cancer	Saint Vincent and the Grenadines	1990	All Ages	0.0 (0.0 to 0.0)	10.9% (4.5 to 19.3%)	0.7 (0.3 to 1.3)
Tracheal, bronchus, and lung cancer	Saint Vincent and the Grenadines	1995	All Ages	0.0 (0.0 to 0.0)	11.0% (4.8 to 19.2%)	0.8 (0.3 to 1.5)
Tracheal, bronchus, and lung cancer	Saint Vincent and the Grenadines	2000	All Ages	0.0 (0.0 to 0.0)	11.0% (4.9 to 19.2%)	1.0 (0.4 to 1.7)
Tracheal, bronchus, and lung cancer	Saint Vincent and the Grenadines	2005	All Ages	0.0 (0.0 to 0.0)	9.6% (3.9 to 18.0%)	0.9 (0.4 to 1.6)
Tracheal, bronchus, and lung cancer	Saint Vincent and the Grenadines	2010	All Ages	0.0 (0.0 to 0.0)	8.0% (3.0 to 14.9%)	0.8 (0.3 to 1.4)
Tracheal, bronchus, and lung cancer	Saint Vincent and the Grenadines	2015	All Ages	0.0 (0.0 to 0.0)	8.3% (3.1 to 16.3%)	0.9 (0.3 to 1.8)
Tracheal, bronchus, and lung cancer	Saint Vincent and the Grenadines	1990	Age-standardized		11.0% (4.5 to 19.3%)	1.2 (0.5 to 2.1)
Tracheal, bronchus, and lung cancer	Saint Vincent and the Grenadines	1995	Age-standardized		11.1% (4.8 to 19.3%)	1.3 (0.5 to 2.4)
Tracheal, bronchus, and lung cancer	Saint Vincent and the Grenadines	2000	Age-standardized		11.0% (4.9 to 19.3%)	1.5 (0.6 to 2.6)
Tracheal, bronchus, and lung cancer	Saint Vincent and the Grenadines	2005	Age-standardized		9.6% (3.9 to 18.0%)	1.2 (0.5 to 2.3)
Tracheal, bronchus, and lung cancer	Saint Vincent and the Grenadines	2010	Age-standardized		8.0% (3.0 to 14.9%)	1.0 (0.4 to 1.9)
Tracheal, bronchus, and lung cancer	Saint Vincent and the Grenadines	2015	Age-standardized		8.4% (3.1 to 16.3%)	1.1 (0.4 to 2.1)
Cardiovascular diseases	Saint Vincent and the Grenadines	1990	All Ages	0.0 (0.0 to 0.0)	9.9% (7.3 to 12.7%)	24.2 (17.6 to 31.3)
Cardiovascular diseases	Saint Vincent and the Grenadines	1995	All Ages	0.0 (0.0 to 0.0)	10.0% (7.3 to 12.8%)	26.5 (19.1 to 34.0)
Cardiovascular diseases	Saint Vincent and the Grenadines	2000	All Ages	0.0 (0.0 to 0.0)	9.9% (7.2 to 12.6%)	27.2 (20.1 to 34.8)
Cardiovascular diseases	Saint Vincent and the Grenadines	2005	All Ages	0.0 (0.0 to 0.0)	9.2% (6.8 to 11.9%)	25.2 (18.4 to 32.8)
Cardiovascular diseases	Saint Vincent and the Grenadines	2010	All Ages	0.0 (0.0 to 0.0)	8.6% (6.0 to 11.2%)	23.2 (16.1 to 30.3)
Cardiovascular diseases	Saint Vincent and the Grenadines	2015	All Ages	0.0 (0.0 to 0.0)	8.7% (6.2 to 11.4%)	25.8 (18.1 to 34.2)
Cardiovascular diseases	Saint Vincent and the Grenadines	1990	Age-standardized		10.0% (7.3 to 12.9%)	40.7 (29.7 to 52.9)
Cardiovascular diseases	Saint Vincent and the Grenadines	1995	Age-standardized		10.1% (7.3 to 12.8%)	41.8 (30.0 to 53.6)
Cardiovascular diseases	Saint Vincent and the Grenadines	2000	Age-standardized		10.0% (7.3 to 12.7%)	39.6 (29.2 to 50.9)
Cardiovascular diseases	Saint Vincent and the Grenadines	2005	Age-standardized		9.2% (6.7 to 11.9%)	35.5 (25.8 to 46.5)
Cardiovascular diseases	Saint Vincent and the Grenadines	2010	Age-standardized		8.4% (5.8 to 11.0%)	32.7 (22.3 to 43.2)

Cause name	Location	Year	Age group	Number (in thousands)	PAF	Rate (per 100,000)
Cardiovascular diseases	Saint Vincent and the Grenadines	2015	Age-standardized		8.5% (5.9 to 11.2%)	32.0 (22.5 to 42.6)
Ischemic heart disease	Saint Vincent and the Grenadines	1990	All Ages	0.0 (0.0 to 0.0)	13.9% (10.0 to 18.1%)	16.3 (11.5 to 21.3)
Ischemic heart disease	Saint Vincent and the Grenadines	1995	All Ages	0.0 (0.0 to 0.0)	13.8% (9.7 to 17.6%)	18.0 (12.5 to 23.0)
Ischemic heart disease	Saint Vincent and the Grenadines	2000	All Ages	0.0 (0.0 to 0.0)	13.7% (9.7 to 18.1%)	18.5 (13.1 to 24.5)
Ischemic heart disease	Saint Vincent and the Grenadines	2005	All Ages	0.0 (0.0 to 0.0)	13.0% (9.3 to 16.9%)	17.0 (12.1 to 22.4)
Ischemic heart disease	Saint Vincent and the Grenadines	2010	All Ages	0.0 (0.0 to 0.0)	12.2% (8.0 to 16.0%)	15.9 (10.6 to 21.0)
Ischemic heart disease	Saint Vincent and the Grenadines	2015	All Ages	0.0 (0.0 to 0.0)	12.5% (8.5 to 16.4%)	17.8 (12.1 to 23.9)
Ischemic heart disease	Saint Vincent and the Grenadines	1990	Age-standardized		13.9% (9.8 to 18.0%)	27.5 (19.3 to 35.9)
Ischemic heart disease	Saint Vincent and the Grenadines	1995	Age-standardized		13.8% (9.5 to 17.6%)	28.5 (19.6 to 36.7)
Ischemic heart disease	Saint Vincent and the Grenadines	2000	Age-standardized		13.7% (9.7 to 18.2%)	27.0 (19.0 to 35.9)
Ischemic heart disease	Saint Vincent and the Grenadines	2005	Age-standardized		12.8% (9.1 to 16.8%)	24.0 (17.0 to 31.8)
Ischemic heart disease	Saint Vincent and the Grenadines	2010	Age-standardized		11.8% (7.6 to 15.7%)	22.3 (14.3 to 29.8)
Ischemic heart disease	Saint Vincent and the Grenadines	2015	Age-standardized		12.1% (8.1 to 16.2%)	22.0 (14.7 to 29.8)
Ischemic stroke	Saint Vincent and the Grenadines	1990	All Ages	0.0 (0.0 to 0.0)	9.9% (5.9 to 13.8%)	4.3 (2.6 to 6.0)
Ischemic stroke	Saint Vincent and the Grenadines	1995	All Ages	0.0 (0.0 to 0.0)	9.9% (6.1 to 13.7%)	4.5 (2.8 to 6.4)
Ischemic stroke	Saint Vincent and the Grenadines	2000	All Ages	0.0 (0.0 to 0.0)	9.8% (6.1 to 14.1%)	4.3 (2.6 to 6.1)
Ischemic stroke	Saint Vincent and the Grenadines	2005	All Ages	0.0 (0.0 to 0.0)	9.1% (5.4 to 13.3%)	3.8 (2.2 to 5.7)
Ischemic stroke	Saint Vincent and the Grenadines	2010	All Ages	0.0 (0.0 to 0.0)	8.3% (4.6 to 12.4%)	3.3 (1.8 to 5.0)
Ischemic stroke	Saint Vincent and the Grenadines	2015	All Ages	0.0 (0.0 to 0.0)	8.5% (4.9 to 12.8%)	3.5 (2.0 to 5.3)
Ischemic stroke	Saint Vincent and the Grenadines	1990	Age-standardized		9.8% (5.8 to 13.9%)	7.2 (4.3 to 10.3)
Ischemic stroke	Saint Vincent and the Grenadines	1995	Age-standardized		9.9% (6.0 to 13.8%)	7.1 (4.4 to 10.1)
Ischemic stroke	Saint Vincent and the Grenadines	2000	Age-standardized		9.8% (6.1 to 14.1%)	6.2 (3.8 to 8.9)
Ischemic stroke	Saint Vincent and the Grenadines	2005	Age-standardized		9.1% (5.3 to 13.2%)	5.5 (3.1 to 8.2)
Ischemic stroke	Saint Vincent and the Grenadines	2010	Age-standardized		8.2% (4.4 to 12.4%)	4.8 (2.6 to 7.5)
Ischemic stroke	Saint Vincent and the Grenadines	2015	Age-standardized		8.4% (4.7 to 12.8%)	4.5 (2.5 to 7.0)
Hemorrhagic stroke	Saint Vincent and the Grenadines	1990	All Ages	0.0 (0.0 to 0.0)	10.5% (6.8 to 14.3%)	3.6 (2.3 to 4.9)
Hemorrhagic stroke	Saint Vincent and the Grenadines	1995	All Ages	0.0 (0.0 to 0.0)	10.8% (7.1 to 14.5%)	4.0 (2.6 to 5.3)
Hemorrhagic stroke	Saint Vincent and the Grenadines	2000	All Ages	0.0 (0.0 to 0.0)	10.7% (7.2 to 14.5%)	4.4 (3.0 to 6.1)
Hemorrhagic stroke	Saint Vincent and the Grenadines	2005	All Ages	0.0 (0.0 to 0.0)	10.1% (6.4 to 14.0%)	4.4 (2.8 to 6.1)
Hemorrhagic stroke	Saint Vincent and the Grenadines	2010	All Ages	0.0 (0.0 to 0.0)	9.2% (5.5 to 13.0%)	4.0 (2.4 to 5.8)
Hemorrhagic stroke	Saint Vincent and the Grenadines	2015	All Ages	0.0 (0.0 to 0.0)	9.5% (5.9 to 13.5%)	4.5 (2.8 to 6.3)
Hemorrhagic stroke	Saint Vincent and the Grenadines	1990	Age-standardized		10.8% (6.9 to 14.6%)	6.0 (3.8 to 8.1)
Hemorrhagic stroke	Saint Vincent and the Grenadines	1995	Age-standardized		10.9% (7.2 to 14.8%)	6.2 (4.1 to 8.3)
Hemorrhagic stroke	Saint Vincent and the Grenadines	2000	Age-standardized		10.8% (7.3 to 14.7%)	6.4 (4.3 to 8.9)
Hemorrhagic stroke	Saint Vincent and the Grenadines	2005	Age-standardized		10.0% (6.4 to 13.8%)	6.1 (3.9 to 8.5)
Hemorrhagic stroke	Saint Vincent and the Grenadines	2010	Age-standardized		9.0% (5.3 to 12.7%)	5.5 (3.2 to 7.9)

Cause name	Location	Year	Age group	Number (in thousands)	PAF	Rate (per 100,000)
Hemorrhagic stroke	Saint Vincent and the Grenadines	2015	Age-standardized		9.2% (5.6 to 13.3%)	5.5 (3.3 to 7.8)
Chronic obstructive pulmonary disease	Saint Vincent and the Grenadines	1990	All Ages	0.0 (0.0 to 0.0)	16.0% (6.1 to 27.7%)	1.5 (0.6 to 2.6)
Chronic obstructive pulmonary disease	Saint Vincent and the Grenadines	1995	All Ages	0.0 (0.0 to 0.0)	16.6% (6.4 to 28.2%)	1.6 (0.6 to 2.8)
Chronic obstructive pulmonary disease	Saint Vincent and the Grenadines	2000	All Ages	0.0 (0.0 to 0.0)	16.7% (6.9 to 29.6%)	1.8 (0.7 to 3.2)
Chronic obstructive pulmonary disease	Saint Vincent and the Grenadines	2005	All Ages	0.0 (0.0 to 0.0)	15.2% (5.5 to 26.2%)	1.6 (0.6 to 2.8)
Chronic obstructive pulmonary disease	Saint Vincent and the Grenadines	2010	All Ages	0.0 (0.0 to 0.0)	13.4% (4.3 to 24.0%)	1.3 (0.4 to 2.3)
Chronic obstructive pulmonary disease	Saint Vincent and the Grenadines	2015	All Ages	0.0 (0.0 to 0.0)	13.8% (4.6 to 24.7%)	1.5 (0.5 to 2.6)
Chronic obstructive pulmonary disease	Saint Vincent and the Grenadines	1990	Age-standardized		16.6% (6.4 to 28.8%)	2.5 (1.0 to 4.3)
Chronic obstructive pulmonary disease	Saint Vincent and the Grenadines	1995	Age-standardized		16.9% (6.6 to 28.9%)	2.6 (1.0 to 4.4)
Chronic obstructive pulmonary disease	Saint Vincent and the Grenadines	2000	Age-standardized		16.9% (7.0 to 30.0%)	2.6 (1.1 to 4.6)
Chronic obstructive pulmonary disease	Saint Vincent and the Grenadines	2005	Age-standardized		15.4% (5.5 to 26.4%)	2.3 (0.8 to 4.0)
Chronic obstructive pulmonary disease	Saint Vincent and the Grenadines	2010	Age-standardized		13.5% (4.3 to 24.2%)	1.8 (0.6 to 3.4)
Chronic obstructive pulmonary disease	Saint Vincent and the Grenadines	2015	Age-standardized		13.8% (4.6 to 24.8%)	1.8 (0.6 to 3.4)
All causes	Suriname	1990	All Ages	0.1 (0.1 to 0.2)	5.8% (4.5 to 7.4%)	36.2 (27.8 to 46.2)
All causes	Suriname	1995	All Ages	0.2 (0.1 to 0.2)	5.7% (4.3 to 7.3%)	35.3 (26.7 to 45.2)
All causes	Suriname	2000	All Ages	0.2 (0.1 to 0.2)	5.6% (4.2 to 7.1%)	36.0 (27.1 to 45.8)
All causes	Suriname	2005	All Ages	0.1 (0.1 to 0.2)	4.4% (3.0 to 5.9%)	30.0 (20.8 to 40.6)
All causes	Suriname	2010	All Ages	0.1 (0.1 to 0.2)	3.9% (2.6 to 5.2%)	27.0 (18.0 to 36.4)
All causes	Suriname	2015	All Ages	0.2 (0.1 to 0.2)	4.5% (3.2 to 5.9%)	30.7 (21.7 to 41.4)
All causes	Suriname	1990	Age-standardized		6.8% (5.2 to 8.6%)	66.1 (50.5 to 84.1)
All causes	Suriname	1995	Age-standardized		6.6% (5.0 to 8.3%)	63.5 (47.6 to 81.6)
All causes	Suriname	2000	Age-standardized		6.3% (4.7 to 8.0%)	62.6 (46.7 to 79.9)
All causes	Suriname	2005	Age-standardized		4.8% (3.3 to 6.5%)	47.2 (32.5 to 64.0)
All causes	Suriname	2010	Age-standardized		4.2% (2.8 to 5.6%)	39.2 (25.6 to 53.1)
All causes	Suriname	2015	Age-standardized		4.7% (3.3 to 6.2%)	40.9 (28.8 to 55.5)
Lower respiratory infections	Suriname	1990	All Ages	0.0 (0.0 to 0.1)	20.6% (10.9 to 31.4%)	7.3 (3.8 to 11.4)
Lower respiratory infections	Suriname	1995	All Ages	0.0 (0.0 to 0.1)	20.8% (11.4 to 31.4%)	7.1 (3.8 to 10.9)
Lower respiratory infections	Suriname	2000	All Ages	0.0 (0.0 to 0.1)	20.8% (10.8 to 31.9%)	6.9 (3.6 to 10.8)
Lower respiratory infections	Suriname	2005	All Ages	0.0 (0.0 to 0.0)	14.1% (5.3 to 25.0%)	4.8 (1.8 to 8.6)
Lower respiratory infections	Suriname	2010	All Ages	0.0 (0.0 to 0.0)	12.3% (4.2 to 24.0%)	4.1 (1.4 to 8.2)
Lower respiratory infections	Suriname	2015	All Ages	0.0 (0.0 to 0.1)	14.9% (5.8 to 26.5%)	4.8 (1.8 to 8.6)
Lower respiratory infections	Suriname	1990	Age-standardized		20.6% (10.9 to 31.4%)	10.5 (5.5 to 16.5)
Lower respiratory infections	Suriname	1995	Age-standardized		20.8% (11.4 to 31.4%)	10.7 (5.8 to 16.6)
Lower respiratory infections	Suriname	2000	Age-standardized		20.8% (10.8 to 31.9%)	11.0 (5.6 to 17.0)
Lower respiratory infections	Suriname	2005	Age-standardized		14.1% (5.3 to 25.0%)	7.2 (2.7 to 13.0)
Lower respiratory infections	Suriname	2010	Age-standardized		12.3% (4.2 to 24.0%)	6.0 (2.0 to 11.9)

Cause name	Location	Year	Age group	Number (in thousands)	PAF	Rate (per 100,000)
Lower respiratory infections	Suriname	2015	Age-standardized		14.9% (5.8 to 26.5%)	6.6 (2.5 to 11.7)
Tracheal, bronchus, and lung cancer	Suriname	1990	All Ages	0.0 (0.0 to 0.0)	14.6% (7.7 to 23.8%)	1.1 (0.5 to 1.7)
Tracheal, bronchus, and lung cancer	Suriname	1995	All Ages	0.0 (0.0 to 0.0)	14.8% (7.4 to 24.6%)	1.1 (0.6 to 1.9)
Tracheal, bronchus, and lung cancer	Suriname	2000	All Ages	0.0 (0.0 to 0.0)	14.7% (7.4 to 24.5%)	1.3 (0.6 to 2.1)
Tracheal, bronchus, and lung cancer	Suriname	2005	All Ages	0.0 (0.0 to 0.0)	9.8% (4.2 to 18.0%)	1.0 (0.4 to 1.8)
Tracheal, bronchus, and lung cancer	Suriname	2010	All Ages	0.0 (0.0 to 0.0)	8.4% (3.2 to 15.9%)	0.9 (0.4 to 1.7)
Tracheal, bronchus, and lung cancer	Suriname	2015	All Ages	0.0 (0.0 to 0.0)	10.3% (4.7 to 17.6%)	1.2 (0.6 to 2.0)
Tracheal, bronchus, and lung cancer	Suriname	1990	Age-standardized		14.7% (7.7 to 23.9%)	1.9 (1.0 to 3.2)
Tracheal, bronchus, and lung cancer	Suriname	1995	Age-standardized		14.8% (7.4 to 24.7%)	2.0 (1.0 to 3.3)
Tracheal, bronchus, and lung cancer	Suriname	2000	Age-standardized		14.8% (7.4 to 24.6%)	2.1 (1.0 to 3.5)
Tracheal, bronchus, and lung cancer	Suriname	2005	Age-standardized		9.8% (4.3 to 18.0%)	1.4 (0.6 to 2.7)
Tracheal, bronchus, and lung cancer	Suriname	2010	Age-standardized		8.4% (3.2 to 15.9%)	1.3 (0.5 to 2.4)
Tracheal, bronchus, and lung cancer	Suriname	2015	Age-standardized		10.3% (4.7 to 17.6%)	1.5 (0.7 to 2.6)
Cardiovascular diseases	Suriname	1990	All Ages	0.1 (0.1 to 0.1)	12.5% (9.9 to 15.5%)	25.6 (20.0 to 31.7)
Cardiovascular diseases	Suriname	1995	All Ages	0.1 (0.1 to 0.1)	12.5% (9.8 to 15.2%)	25.0 (19.4 to 30.9)
Cardiovascular diseases	Suriname	2000	All Ages	0.1 (0.1 to 0.1)	12.4% (9.5 to 15.3%)	25.6 (19.8 to 32.1)
Cardiovascular diseases	Suriname	2005	All Ages	0.1 (0.1 to 0.1)	10.0% (7.2 to 12.8%)	22.6 (16.4 to 29.6)
Cardiovascular diseases	Suriname	2010	All Ages	0.1 (0.1 to 0.1)	9.1% (6.3 to 11.9%)	20.6 (14.1 to 27.0)
Cardiovascular diseases	Suriname	2015	All Ages	0.1 (0.1 to 0.2)	10.1% (7.5 to 12.7%)	23.0 (16.7 to 29.8)
Cardiovascular diseases	Suriname	1990	Age-standardized		12.0% (9.3 to 15.1%)	48.8 (37.7 to 61.5)
Cardiovascular diseases	Suriname	1995	Age-standardized		11.8% (9.2 to 14.6%)	46.5 (35.3 to 57.9)
Cardiovascular diseases	Suriname	2000	Age-standardized		11.6% (8.8 to 14.6%)	45.3 (34.1 to 57.2)
Cardiovascular diseases	Suriname	2005	Age-standardized		9.4% (6.6 to 12.3%)	35.7 (25.0 to 47.3)
Cardiovascular diseases	Suriname	2010	Age-standardized		8.7% (5.9 to 11.4%)	29.8 (20.2 to 39.5)
Cardiovascular diseases	Suriname	2015	Age-standardized		9.6% (7.1 to 12.3%)	30.4 (22.1 to 39.8)
Ischemic heart disease	Suriname	1990	All Ages	0.1 (0.1 to 0.1)	16.6% (12.9 to 20.7%)	17.1 (13.2 to 21.3)
Ischemic heart disease	Suriname	1995	All Ages	0.1 (0.1 to 0.1)	16.8% (12.9 to 21.0%)	15.6 (11.8 to 19.7)
Ischemic heart disease	Suriname	2000	All Ages	0.1 (0.1 to 0.1)	17.0% (13.1 to 21.1%)	14.5 (11.1 to 18.2)
Ischemic heart disease	Suriname	2005	All Ages	0.1 (0.0 to 0.1)	14.1% (10.2 to 18.0%)	12.7 (9.0 to 16.5)
Ischemic heart disease	Suriname	2010	All Ages	0.1 (0.0 to 0.1)	13.0% (9.1 to 17.1%)	11.9 (8.2 to 15.8)
Ischemic heart disease	Suriname	2015	All Ages	0.1 (0.1 to 0.1)	14.1% (10.2 to 18.0%)	13.5 (9.6 to 17.7)
Ischemic heart disease	Suriname	1990	Age-standardized		15.7% (11.9 to 19.8%)	32.5 (24.4 to 41.3)
Ischemic heart disease	Suriname	1995	Age-standardized		15.7% (11.7 to 19.9%)	28.8 (21.2 to 36.7)
Ischemic heart disease	Suriname	2000	Age-standardized		15.8% (11.8 to 19.9%)	25.1 (18.7 to 32.1)
Ischemic heart disease	Suriname	2005	Age-standardized		13.2% (9.4 to 17.1%)	19.5 (13.7 to 25.6)
Ischemic heart disease	Suriname	2010	Age-standardized		12.2% (8.4 to 16.1%)	16.9 (11.4 to 22.6)

Cause name	Location	Year	Age group	Number (in thousands)	PAF	Rate (per 100,000)
Ischemic heart disease	Suriname	2015	Age-standardized		13.4% (9.6 to 17.3%)	17.5 (12.2 to 23.3)
Ischemic stroke	Suriname	1990	All Ages	0.0 (0.0 to 0.0)	11.9% (8.4 to 16.1%)	4.1 (2.8 to 5.6)
Ischemic stroke	Suriname	1995	All Ages	0.0 (0.0 to 0.0)	11.9% (8.2 to 15.7%)	4.3 (2.9 to 5.7)
Ischemic stroke	Suriname	2000	All Ages	0.0 (0.0 to 0.0)	11.8% (8.1 to 15.8%)	4.7 (3.2 to 6.3)
Ischemic stroke	Suriname	2005	All Ages	0.0 (0.0 to 0.0)	9.2% (5.6 to 13.1%)	4.1 (2.5 to 5.8)
Ischemic stroke	Suriname	2010	All Ages	0.0 (0.0 to 0.0)	8.4% (5.0 to 12.2%)	3.5 (2.0 to 5.1)
Ischemic stroke	Suriname	2015	All Ages	0.0 (0.0 to 0.0)	9.3% (5.9 to 13.2%)	3.9 (2.4 to 5.6)
Ischemic stroke	Suriname	1990	Age-standardized		11.4% (7.8 to 15.7%)	8.5 (5.8 to 11.8)
Ischemic stroke	Suriname	1995	Age-standardized		11.3% (7.4 to 15.2%)	8.7 (5.7 to 11.8)
Ischemic stroke	Suriname	2000	Age-standardized		11.2% (7.3 to 15.3%)	9.2 (6.0 to 12.8)
Ischemic stroke	Suriname	2005	Age-standardized		8.8% (5.0 to 12.9%)	7.1 (4.0 to 10.5)
Ischemic stroke	Suriname	2010	Age-standardized		8.1% (4.6 to 12.1%)	5.5 (3.0 to 8.4)
Ischemic stroke	Suriname	2015	Age-standardized		9.1% (5.5 to 13.1%)	5.6 (3.3 to 8.3)
Hemorrhagic stroke	Suriname	1990	All Ages	0.0 (0.0 to 0.0)	12.7% (9.2 to 16.6%)	4.3 (3.2 to 5.7)
Hemorrhagic stroke	Suriname	1995	All Ages	0.0 (0.0 to 0.0)	12.8% (9.3 to 16.4%)	5.1 (3.7 to 6.7)
Hemorrhagic stroke	Suriname	2000	All Ages	0.0 (0.0 to 0.0)	12.9% (9.3 to 16.6%)	6.4 (4.6 to 8.4)
Hemorrhagic stroke	Suriname	2005	All Ages	0.0 (0.0 to 0.0)	10.1% (6.6 to 14.1%)	5.9 (3.7 to 8.3)
Hemorrhagic stroke	Suriname	2010	All Ages	0.0 (0.0 to 0.0)	9.3% (5.9 to 12.8%)	5.2 (3.2 to 7.3)
Hemorrhagic stroke	Suriname	2015	All Ages	0.0 (0.0 to 0.0)	10.4% (7.1 to 14.0%)	5.7 (3.8 to 7.8)
Hemorrhagic stroke	Suriname	1990	Age-standardized		12.5% (9.0 to 16.6%)	7.7 (5.6 to 10.3)
Hemorrhagic stroke	Suriname	1995	Age-standardized		12.4% (8.8 to 16.0%)	9.0 (6.5 to 11.9)
Hemorrhagic stroke	Suriname	2000	Age-standardized		12.2% (8.6 to 16.1%)	11.0 (7.7 to 14.6)
Hemorrhagic stroke	Suriname	2005	Age-standardized		9.7% (6.1 to 13.7%)	9.1 (5.7 to 12.9)
Hemorrhagic stroke	Suriname	2010	Age-standardized		8.8% (5.3 to 12.5%)	7.4 (4.5 to 10.5)
Hemorrhagic stroke	Suriname	2015	Age-standardized		9.9% (6.7 to 13.7%)	7.4 (4.8 to 10.5)
Chronic obstructive pulmonary disease	Suriname	1990	All Ages	0.0 (0.0 to 0.0)	19.4% (9.0 to 30.2%)	2.3 (1.0 to 3.6)
Chronic obstructive pulmonary disease	Suriname	1995	All Ages	0.0 (0.0 to 0.0)	19.7% (9.6 to 32.0%)	2.1 (1.0 to 3.4)
Chronic obstructive pulmonary disease	Suriname	2000	All Ages	0.0 (0.0 to 0.0)	19.8% (10.0 to 32.0%)	2.2 (1.1 to 3.6)
Chronic obstructive pulmonary disease	Suriname	2005	All Ages	0.0 (0.0 to 0.0)	14.8% (5.9 to 25.4%)	1.7 (0.7 to 3.0)
Chronic obstructive pulmonary disease	Suriname	2010	All Ages	0.0 (0.0 to 0.0)	13.3% (5.3 to 24.0%)	1.4 (0.6 to 2.6)
Chronic obstructive pulmonary disease	Suriname	2015	All Ages	0.0 (0.0 to 0.0)	15.7% (6.7 to 27.0%)	1.7 (0.7 to 3.0)
Chronic obstructive pulmonary disease	Suriname	1990	Age-standardized		20.4% (9.6 to 31.9%)	4.8 (2.2 to 7.6)
Chronic obstructive pulmonary disease	Suriname	1995	Age-standardized		20.6% (10.0 to 33.2%)	4.3 (2.1 to 7.0)
Chronic obstructive pulmonary disease	Suriname	2000	Age-standardized		20.5% (10.3 to 33.1%)	4.2 (2.0 to 6.9)
Chronic obstructive pulmonary disease	Suriname	2005	Age-standardized		15.1% (6.1 to 26.1%)	2.9 (1.1 to 5.0)
Chronic obstructive pulmonary disease	Suriname	2010	Age-standardized		13.5% (5.3 to 24.3%)	2.2 (0.9 to 4.0)

Cause name	Location	Year	Age group	Number (in thousands)	PAF	Rate (per 100,000)
Chronic obstructive pulmonary disease	Suriname	2015	Age-standardized		15.8% (6.7 to 27.2%)	2.4 (1.0 to 4.2)
All causes	Trinidad and Tobago	1990	All Ages	0.5 (0.4 to 0.6)	5.6% (4.6 to 6.6%)	39.1 (32.5 to 46.0)
All causes	Trinidad and Tobago	1995	All Ages	0.5 (0.4 to 0.6)	5.6% (4.6 to 6.5%)	40.5 (33.6 to 47.8)
All causes	Trinidad and Tobago	2000	All Ages	0.5 (0.4 to 0.6)	5.2% (4.3 to 6.1%)	40.6 (33.7 to 47.7)
All causes	Trinidad and Tobago	2005	All Ages	0.4 (0.4 to 0.5)	4.3% (3.5 to 5.1%)	32.9 (26.8 to 39.3)
All causes	Trinidad and Tobago	2010	All Ages	0.4 (0.3 to 0.5)	3.8% (3.0 to 4.6%)	30.8 (24.7 to 37.4)
All causes	Trinidad and Tobago	2015	All Ages	0.5 (0.4 to 0.6)	4.2% (3.4 to 5.1%)	35.9 (28.7 to 43.4)
All causes	Trinidad and Tobago	1990	Age-standardized		5.9% (4.9 to 7.0%)	65.9 (54.6 to 78.2)
All causes	Trinidad and Tobago	1995	Age-standardized		5.8% (4.8 to 6.9%)	63.6 (52.5 to 75.3)
All causes	Trinidad and Tobago	2000	Age-standardized		5.4% (4.4 to 6.3%)	57.9 (47.5 to 68.5)
All causes	Trinidad and Tobago	2005	Age-standardized		4.4% (3.5 to 5.3%)	42.2 (33.9 to 50.9)
All causes	Trinidad and Tobago	2010	Age-standardized		3.8% (3.0 to 4.7%)	35.4 (27.9 to 43.5)
All causes	Trinidad and Tobago	2015	Age-standardized		4.2% (3.3 to 5.1%)	37.7 (29.7 to 46.2)
Lower respiratory infections	Trinidad and Tobago	1990	All Ages	0.1 (0.0 to 0.1)	16.1% (7.6 to 26.7%)	5.0 (2.4 to 8.3)
Lower respiratory infections	Trinidad and Tobago	1995	All Ages	0.1 (0.0 to 0.1)	16.0% (7.8 to 26.1%)	4.7 (2.2 to 7.7)
Lower respiratory infections	Trinidad and Tobago	2000	All Ages	0.1 (0.0 to 0.1)	16.0% (7.5 to 26.4%)	4.3 (2.0 to 7.0)
Lower respiratory infections	Trinidad and Tobago	2005	All Ages	0.0 (0.0 to 0.1)	12.8% (4.9 to 23.8%)	3.2 (1.2 to 5.9)
Lower respiratory infections	Trinidad and Tobago	2010	All Ages	0.0 (0.0 to 0.1)	10.5% (3.4 to 21.3%)	2.7 (0.9 to 5.4)
Lower respiratory infections	Trinidad and Tobago	2015	All Ages	0.0 (0.0 to 0.1)	12.2% (4.2 to 22.7%)	3.2 (1.1 to 6.1)
Lower respiratory infections	Trinidad and Tobago	1990	Age-standardized		16.1% (7.6 to 26.7%)	8.0 (3.8 to 13.3)
Lower respiratory infections	Trinidad and Tobago	1995	Age-standardized		16.0% (7.8 to 26.1%)	7.3 (3.5 to 11.9)
Lower respiratory infections	Trinidad and Tobago	2000	Age-standardized		16.0% (7.5 to 26.4%)	6.2 (2.9 to 10.3)
Lower respiratory infections	Trinidad and Tobago	2005	Age-standardized		12.8% (4.9 to 23.8%)	4.3 (1.6 to 7.9)
Lower respiratory infections	Trinidad and Tobago	2010	Age-standardized		10.5% (3.4 to 21.3%)	3.3 (1.1 to 6.7)
Lower respiratory infections	Trinidad and Tobago	2015	Age-standardized		12.2% (4.2 to 22.7%)	3.7 (1.3 to 7.1)
Tracheal, bronchus, and lung cancer	Trinidad and Tobago	1990	All Ages	0.0 (0.0 to 0.0)	11.2% (6.0 to 17.4%)	0.9 (0.5 to 1.3)
Tracheal, bronchus, and lung cancer	Trinidad and Tobago	1995	All Ages	0.0 (0.0 to 0.0)	11.1% (5.9 to 17.5%)	0.9 (0.5 to 1.4)
Tracheal, bronchus, and lung cancer	Trinidad and Tobago	2000	All Ages	0.0 (0.0 to 0.0)	11.1% (5.9 to 17.7%)	1.0 (0.5 to 1.6)
Tracheal, bronchus, and lung cancer	Trinidad and Tobago	2005	All Ages	0.0 (0.0 to 0.0)	8.8% (4.2 to 14.4%)	0.9 (0.4 to 1.4)
Tracheal, bronchus, and lung cancer	Trinidad and Tobago	2010	All Ages	0.0 (0.0 to 0.0)	7.2% (3.4 to 12.2%)	0.8 (0.4 to 1.4)
Tracheal, bronchus, and lung cancer	Trinidad and Tobago	2015	All Ages	0.0 (0.0 to 0.0)	8.4% (4.0 to 13.9%)	1.1 (0.5 to 1.8)
Tracheal, bronchus, and lung cancer	Trinidad and Tobago	1990	Age-standardized		11.2% (6.0 to 17.4%)	1.4 (0.8 to 2.2)
Tracheal, bronchus, and lung cancer	Trinidad and Tobago	1995	Age-standardized		11.1% (6.0 to 17.6%)	1.4 (0.7 to 2.2)
Tracheal, bronchus, and lung cancer	Trinidad and Tobago	2000	Age-standardized		11.2% (5.9 to 17.8%)	1.4 (0.7 to 2.2)
Tracheal, bronchus, and lung cancer	Trinidad and Tobago	2005	Age-standardized		8.8% (4.2 to 14.4%)	1.1 (0.5 to 1.7)
Tracheal, bronchus, and lung cancer	Trinidad and Tobago	2010	Age-standardized		7.2% (3.4 to 12.2%)	0.9 (0.4 to 1.5)

Cause name	Location	Year	Age group	Number (in thousands)	PAF	Rate (per 100,000)
Tracheal, bronchus, and lung cancer	Trinidad and Tobago	2015	Age-standardized		8.4% (4.0 to 13.8%)	1.0 (0.5 to 1.8)
Cardiovascular diseases	Trinidad and Tobago	1990	All Ages	0.4 (0.3 to 0.5)	11.7% (9.7 to 13.7%)	31.5 (26.3 to 37.0)
Cardiovascular diseases	Trinidad and Tobago	1995	All Ages	0.4 (0.4 to 0.5)	11.7% (9.7 to 13.7%)	33.2 (27.6 to 39.1)
Cardiovascular diseases	Trinidad and Tobago	2000	All Ages	0.4 (0.4 to 0.5)	11.5% (9.5 to 13.4%)	33.5 (27.7 to 39.3)
Cardiovascular diseases	Trinidad and Tobago	2005	All Ages	0.4 (0.3 to 0.4)	10.2% (8.3 to 12.1%)	27.4 (22.2 to 32.5)
Cardiovascular diseases	Trinidad and Tobago	2010	All Ages	0.3 (0.3 to 0.4)	9.2% (7.3 to 11.1%)	25.9 (20.7 to 31.5)
Cardiovascular diseases	Trinidad and Tobago	2015	All Ages	0.4 (0.3 to 0.5)	9.9% (8.0 to 11.9%)	30.0 (23.8 to 36.5)
Cardiovascular diseases	Trinidad and Tobago	1990	Age-standardized		11.2% (9.2 to 13.2%)	53.3 (43.6 to 63.6)
Cardiovascular diseases	Trinidad and Tobago	1995	Age-standardized		11.2% (9.2 to 13.3%)	52.0 (42.4 to 61.6)
Cardiovascular diseases	Trinidad and Tobago	2000	Age-standardized		11.0% (9.0 to 13.0%)	47.5 (38.8 to 56.2)
Cardiovascular diseases	Trinidad and Tobago	2005	Age-standardized		9.7% (7.8 to 11.7%)	34.9 (27.8 to 42.5)
Cardiovascular diseases	Trinidad and Tobago	2010	Age-standardized		8.8% (6.9 to 10.7%)	29.6 (23.3 to 36.4)
Cardiovascular diseases	Trinidad and Tobago	2015	Age-standardized		9.4% (7.4 to 11.5%)	31.1 (24.1 to 38.5)
Ischemic heart disease	Trinidad and Tobago	1990	All Ages	0.3 (0.2 to 0.3)	15.2% (12.3 to 18.3%)	22.5 (18.1 to 26.9)
Ischemic heart disease	Trinidad and Tobago	1995	All Ages	0.3 (0.2 to 0.4)	15.1% (12.1 to 18.0%)	23.9 (19.0 to 28.5)
Ischemic heart disease	Trinidad and Tobago	2000	All Ages	0.3 (0.3 to 0.4)	15.0% (12.1 to 18.1%)	24.2 (19.3 to 28.9)
Ischemic heart disease	Trinidad and Tobago	2005	All Ages	0.3 (0.2 to 0.3)	13.6% (10.7 to 16.6%)	19.9 (15.7 to 24.4)
Ischemic heart disease	Trinidad and Tobago	2010	All Ages	0.3 (0.2 to 0.3)	12.3% (9.7 to 15.1%)	19.0 (14.8 to 23.6)
Ischemic heart disease	Trinidad and Tobago	2015	All Ages	0.3 (0.2 to 0.4)	13.2% (10.3 to 16.0%)	22.2 (17.0 to 27.7)
Ischemic heart disease	Trinidad and Tobago	1990	Age-standardized		14.6% (11.6 to 17.6%)	37.8 (30.0 to 45.9)
Ischemic heart disease	Trinidad and Tobago	1995	Age-standardized		14.4% (11.3 to 17.5%)	37.2 (29.1 to 44.9)
Ischemic heart disease	Trinidad and Tobago	2000	Age-standardized		14.3% (11.2 to 17.5%)	34.1 (26.5 to 41.9)
Ischemic heart disease	Trinidad and Tobago	2005	Age-standardized		12.9% (10.0 to 16.0%)	25.2 (19.3 to 31.4)
Ischemic heart disease	Trinidad and Tobago	2010	Age-standardized		11.7% (8.9 to 14.7%)	21.5 (16.3 to 27.1)
Ischemic heart disease	Trinidad and Tobago	2015	Age-standardized		12.6% (9.5 to 15.7%)	22.8 (16.9 to 28.8)
Ischemic stroke	Trinidad and Tobago	1990	All Ages	0.1 (0.0 to 0.1)	10.4% (7.4 to 13.6%)	4.8 (3.4 to 6.3)
Ischemic stroke	Trinidad and Tobago	1995	All Ages	0.1 (0.0 to 0.1)	10.3% (7.2 to 13.4%)	4.9 (3.4 to 6.4)
Ischemic stroke	Trinidad and Tobago	2000	All Ages	0.1 (0.0 to 0.1)	10.2% (7.2 to 13.4%)	4.4 (3.1 to 5.9)
Ischemic stroke	Trinidad and Tobago	2005	All Ages	0.0 (0.0 to 0.1)	9.0% (5.8 to 12.3%)	3.4 (2.2 to 4.6)
Ischemic stroke	Trinidad and Tobago	2010	All Ages	0.0 (0.0 to 0.1)	8.0% (5.2 to 11.4%)	3.0 (2.0 to 4.4)
Ischemic stroke	Trinidad and Tobago	2015	All Ages	0.1 (0.0 to 0.1)	8.7% (5.8 to 12.0%)	3.4 (2.3 to 4.8)
Ischemic stroke	Trinidad and Tobago	1990	Age-standardized		10.1% (7.0 to 13.4%)	8.7 (6.0 to 11.7)
Ischemic stroke	Trinidad and Tobago	1995	Age-standardized		10.0% (6.8 to 13.4%)	8.2 (5.4 to 11.0)
Ischemic stroke	Trinidad and Tobago	2000	Age-standardized		9.9% (6.7 to 13.4%)	6.7 (4.5 to 9.2)
Ischemic stroke	Trinidad and Tobago	2005	Age-standardized		8.7% (5.4 to 12.2%)	4.7 (2.9 to 6.6)
Ischemic stroke	Trinidad and Tobago	2010	Age-standardized		7.7% (4.7 to 11.5%)	3.8 (2.3 to 5.6)

Cause name	Location	Year	Age group	Number (in thousands)	PAF	Rate (per 100,000)
Ischemic stroke	Trinidad and Tobago	2015	Age-standardized		8.4% (5.3 to 12.0%)	3.9 (2.4 to 5.5)
Hemorrhagic stroke	Trinidad and Tobago	1990	All Ages	0.1 (0.0 to 0.1)	11.6% (8.8 to 14.4%)	4.2 (3.2 to 5.3)
Hemorrhagic stroke	Trinidad and Tobago	1995	All Ages	0.1 (0.0 to 0.1)	11.6% (8.7 to 14.5%)	4.4 (3.4 to 5.6)
Hemorrhagic stroke	Trinidad and Tobago	2000	All Ages	0.1 (0.1 to 0.1)	11.4% (8.7 to 14.2%)	4.9 (3.7 to 6.2)
Hemorrhagic stroke	Trinidad and Tobago	2005	All Ages	0.1 (0.0 to 0.1)	10.0% (7.4 to 12.9%)	4.1 (3.0 to 5.3)
Hemorrhagic stroke	Trinidad and Tobago	2010	All Ages	0.1 (0.0 to 0.1)	8.9% (6.3 to 11.6%)	3.8 (2.7 to 5.1)
Hemorrhagic stroke	Trinidad and Tobago	2015	All Ages	0.1 (0.0 to 0.1)	9.6% (6.8 to 12.5%)	4.4 (3.0 to 5.8)
Hemorrhagic stroke	Trinidad and Tobago	1990	Age-standardized		11.2% (8.4 to 14.1%)	6.9 (5.1 to 8.7)
Hemorrhagic stroke	Trinidad and Tobago	1995	Age-standardized		11.1% (8.2 to 14.1%)	6.7 (5.0 to 8.5)
Hemorrhagic stroke	Trinidad and Tobago	2000	Age-standardized		10.9% (8.1 to 13.9%)	6.7 (5.0 to 8.5)
Hemorrhagic stroke	Trinidad and Tobago	2005	Age-standardized		9.6% (6.8 to 12.6%)	5.1 (3.6 to 6.7)
Hemorrhagic stroke	Trinidad and Tobago	2010	Age-standardized		8.5% (5.8 to 11.3%)	4.3 (2.9 to 5.9)
Hemorrhagic stroke	Trinidad and Tobago	2015	Age-standardized		9.2% (6.4 to 12.1%)	4.5 (3.1 to 6.0)
Chronic obstructive pulmonary disease	Trinidad and Tobago	1990	All Ages	0.0 (0.0 to 0.0)	16.6% (7.8 to 27.0%)	1.7 (0.8 to 2.8)
Chronic obstructive pulmonary disease	Trinidad and Tobago	1995	All Ages	0.0 (0.0 to 0.0)	16.7% (7.8 to 27.0%)	1.7 (0.8 to 2.8)
Chronic obstructive pulmonary disease	Trinidad and Tobago	2000	All Ages	0.0 (0.0 to 0.0)	16.9% (8.0 to 27.5%)	1.8 (0.8 to 3.0)
Chronic obstructive pulmonary disease	Trinidad and Tobago	2005	All Ages	0.0 (0.0 to 0.0)	14.3% (6.2 to 24.1%)	1.4 (0.6 to 2.4)
Chronic obstructive pulmonary disease	Trinidad and Tobago	2010	All Ages	0.0 (0.0 to 0.0)	12.4% (4.8 to 21.8%)	1.3 (0.5 to 2.3)
Chronic obstructive pulmonary disease	Trinidad and Tobago	2015	All Ages	0.0 (0.0 to 0.0)	13.9% (5.9 to 24.2%)	1.6 (0.7 to 2.8)
Chronic obstructive pulmonary disease	Trinidad and Tobago	1990	Age-standardized		17.0% (8.0 to 27.5%)	3.1 (1.4 to 5.0)
Chronic obstructive pulmonary disease	Trinidad and Tobago	1995	Age-standardized		16.9% (7.9 to 27.3%)	2.9 (1.4 to 4.7)
Chronic obstructive pulmonary disease	Trinidad and Tobago	2000	Age-standardized		17.0% (8.1 to 27.7%)	2.7 (1.3 to 4.5)
Chronic obstructive pulmonary disease	Trinidad and Tobago	2005	Age-standardized		14.4% (6.2 to 24.3%)	1.9 (0.8 to 3.3)
Chronic obstructive pulmonary disease	Trinidad and Tobago	2010	Age-standardized		12.4% (4.8 to 21.9%)	1.6 (0.6 to 2.9)
Chronic obstructive pulmonary disease	Trinidad and Tobago	2015	Age-standardized		13.9% (5.8 to 24.2%)	1.8 (0.8 to 3.2)
All causes	Virgin Islands, U.S.	1990	All Ages	0.0 (0.0 to 0.1)	5.3% (3.1 to 7.6%)	29.9 (18.0 to 43.4)
All causes	Virgin Islands, U.S.	1995	All Ages	0.0 (0.0 to 0.1)	5.2% (3.1 to 7.4%)	34.1 (20.3 to 48.8)
All causes	Virgin Islands, U.S.	2000	All Ages	0.0 (0.0 to 0.1)	5.3% (3.1 to 7.9%)	39.7 (23.3 to 59.0)
All causes	Virgin Islands, U.S.	2005	All Ages	0.1 (0.0 to 0.1)	4.9% (2.8 to 7.1%)	42.3 (23.8 to 61.8)
All causes	Virgin Islands, U.S.	2010	All Ages	0.1 (0.0 to 0.1)	4.4% (1.9 to 6.8%)	45.8 (19.9 to 70.5)
All causes	Virgin Islands, U.S.	2015	All Ages	0.1 (0.0 to 0.1)	4.7% (2.7 to 6.8%)	56.9 (33.3 to 83.8)
All causes	Virgin Islands, U.S.	1990	Age-standardized		5.5% (3.2 to 8.1%)	50.0 (29.3 to 73.2)
All causes	Virgin Islands, U.S.	1995	Age-standardized		5.3% (3.1 to 7.7%)	44.8 (26.3 to 65.1)
All causes	Virgin Islands, U.S.	2000	Age-standardized		5.3% (3.1 to 8.0%)	43.6 (25.4 to 65.5)
All causes	Virgin Islands, U.S.	2005	Age-standardized		4.8% (2.7 to 7.1%)	39.1 (21.9 to 57.9)
All causes	Virgin Islands, U.S.	2010	Age-standardized		4.3% (1.8 to 6.6%)	36.1 (15.4 to 55.9)

Cause name	Location	Year	Age group	Number (in thousands)	PAF	Rate (per 100,000)
All causes	Virgin Islands, U.S.	2015	Age-standardized		4.4% (2.6 to 6.5%)	37.1 (21.5 to 54.8)
Lower respiratory infections	Virgin Islands, U.S.	1990	All Ages	0.0 (0.0 to 0.0)	15.2% (2.8 to 29.1%)	2.6 (0.5 to 5.1)
Lower respiratory infections	Virgin Islands, U.S.	1995	All Ages	0.0 (0.0 to 0.0)	15.2% (2.7 to 30.1%)	3.0 (0.5 to 5.9)
Lower respiratory infections	Virgin Islands, U.S.	2000	All Ages	0.0 (0.0 to 0.0)	16.3% (3.2 to 32.4%)	3.6 (0.7 to 7.3)
Lower respiratory infections	Virgin Islands, U.S.	2005	All Ages	0.0 (0.0 to 0.0)	14.5% (2.1 to 29.1%)	3.7 (0.5 to 7.6)
Lower respiratory infections	Virgin Islands, U.S.	2010	All Ages	0.0 (0.0 to 0.0)	12.7% (1.1 to 28.0%)	3.8 (0.3 to 8.7)
Lower respiratory infections	Virgin Islands, U.S.	2015	All Ages	0.0 (0.0 to 0.0)	13.6% (2.3 to 28.3%)	4.9 (0.8 to 10.7)
Lower respiratory infections	Virgin Islands, U.S.	1990	Age-standardized		15.2% (2.8 to 29.1%)	4.7 (0.8 to 9.2)
Lower respiratory infections	Virgin Islands, U.S.	1995	Age-standardized		15.2% (2.7 to 30.1%)	4.2 (0.7 to 8.4)
Lower respiratory infections	Virgin Islands, U.S.	2000	Age-standardized		16.3% (3.2 to 32.4%)	4.3 (0.8 to 8.7)
Lower respiratory infections	Virgin Islands, U.S.	2005	Age-standardized		14.5% (2.1 to 29.1%)	3.7 (0.5 to 7.6)
Lower respiratory infections	Virgin Islands, U.S.	2010	Age-standardized		12.7% (1.1 to 28.0%)	3.3 (0.3 to 7.5)
Lower respiratory infections	Virgin Islands, U.S.	2015	Age-standardized		13.6% (2.3 to 28.3%)	3.4 (0.6 to 7.5)
Tracheal, bronchus, and lung cancer	Virgin Islands, U.S.	1990	All Ages	0.0 (0.0 to 0.0)	10.6% (2.8 to 22.7%)	1.5 (0.4 to 3.2)
Tracheal, bronchus, and lung cancer	Virgin Islands, U.S.	1995	All Ages	0.0 (0.0 to 0.0)	10.6% (3.0 to 22.2%)	1.9 (0.6 to 3.9)
Tracheal, bronchus, and lung cancer	Virgin Islands, U.S.	2000	All Ages	0.0 (0.0 to 0.0)	11.4% (3.4 to 24.1%)	2.4 (0.7 to 5.1)
Tracheal, bronchus, and lung cancer	Virgin Islands, U.S.	2005	All Ages	0.0 (0.0 to 0.0)	10.1% (2.5 to 20.1%)	2.5 (0.6 to 5.0)
Tracheal, bronchus, and lung cancer	Virgin Islands, U.S.	2010	All Ages	0.0 (0.0 to 0.0)	8.8% (1.1 to 19.0%)	2.7 (0.3 to 5.9)
Tracheal, bronchus, and lung cancer	Virgin Islands, U.S.	2015	All Ages	0.0 (0.0 to 0.0)	9.4% (2.4 to 21.2%)	3.4 (0.9 to 7.5)
Tracheal, bronchus, and lung cancer	Virgin Islands, U.S.	1990	Age-standardized		10.6% (2.8 to 22.8%)	2.2 (0.6 to 4.6)
Tracheal, bronchus, and lung cancer	Virgin Islands, U.S.	1995	Age-standardized		10.6% (3.0 to 22.2%)	2.2 (0.7 to 4.7)
Tracheal, bronchus, and lung cancer	Virgin Islands, U.S.	2000	Age-standardized		11.4% (3.4 to 24.1%)	2.4 (0.7 to 5.1)
Tracheal, bronchus, and lung cancer	Virgin Islands, U.S.	2005	Age-standardized		10.1% (2.5 to 20.1%)	2.1 (0.5 to 4.3)
Tracheal, bronchus, and lung cancer	Virgin Islands, U.S.	2010	Age-standardized		8.8% (1.1 to 19.0%)	2.0 (0.2 to 4.3)
Tracheal, bronchus, and lung cancer	Virgin Islands, U.S.	2015	Age-standardized		9.4% (2.4 to 21.1%)	2.2 (0.5 to 4.8)
Cardiovascular diseases	Virgin Islands, U.S.	1990	All Ages	0.0 (0.0 to 0.0)	11.4% (7.3 to 15.8%)	24.4 (15.6 to 33.6)
Cardiovascular diseases	Virgin Islands, U.S.	1995	All Ages	0.0 (0.0 to 0.0)	11.2% (7.3 to 15.3%)	27.7 (17.9 to 38.2)
Cardiovascular diseases	Virgin Islands, U.S.	2000	All Ages	0.0 (0.0 to 0.1)	11.1% (6.9 to 15.5%)	31.8 (19.9 to 44.5)
Cardiovascular diseases	Virgin Islands, U.S.	2005	All Ages	0.0 (0.0 to 0.1)	10.2% (6.3 to 14.3%)	34.0 (21.0 to 47.7)
Cardiovascular diseases	Virgin Islands, U.S.	2010	All Ages	0.0 (0.0 to 0.1)	9.3% (4.4 to 13.5%)	37.1 (17.5 to 54.1)
Cardiovascular diseases	Virgin Islands, U.S.	2015	All Ages	0.1 (0.0 to 0.1)	9.4% (5.7 to 13.1%)	45.6 (28.0 to 63.6)
Cardiovascular diseases	Virgin Islands, U.S.	1990	Age-standardized		10.5% (6.4 to 14.9%)	40.5 (25.0 to 57.1)
Cardiovascular diseases	Virgin Islands, U.S.	1995	Age-standardized		10.5% (6.7 to 14.5%)	36.2 (22.8 to 50.0)
Cardiovascular diseases	Virgin Islands, U.S.	2000	Age-standardized		10.6% (6.6 to 15.0%)	34.7 (21.5 to 49.2)
Cardiovascular diseases	Virgin Islands, U.S.	2005	Age-standardized		9.8% (6.0 to 14.0%)	31.3 (19.1 to 44.3)
Cardiovascular diseases	Virgin Islands, U.S.	2010	Age-standardized		9.0% (4.3 to 13.2%)	29.0 (13.7 to 42.5)

Cause name	Location	Year	Age group	Number (in thousands)	PAF	Rate (per 100,000)
Cardiovascular diseases	Virgin Islands, U.S.	2015	Age-standardized		9.3% (5.7 to 12.9%)	29.6 (17.9 to 41.6)
Ischemic heart disease	Virgin Islands, U.S.	1990	All Ages	0.0 (0.0 to 0.0)	14.8% (9.4 to 20.4%)	19.6 (12.5 to 26.8)
Ischemic heart disease	Virgin Islands, U.S.	1995	All Ages	0.0 (0.0 to 0.0)	14.6% (9.4 to 20.1%)	22.2 (14.5 to 30.4)
Ischemic heart disease	Virgin Islands, U.S.	2000	All Ages	0.0 (0.0 to 0.0)	14.7% (9.2 to 20.4%)	25.6 (16.0 to 35.5)
Ischemic heart disease	Virgin Islands, U.S.	2005	All Ages	0.0 (0.0 to 0.0)	13.6% (8.2 to 19.0%)	27.6 (16.8 to 39.0)
Ischemic heart disease	Virgin Islands, U.S.	2010	All Ages	0.0 (0.0 to 0.1)	12.7% (6.4 to 18.7%)	30.0 (15.4 to 44.0)
Ischemic heart disease	Virgin Islands, U.S.	2015	All Ages	0.0 (0.0 to 0.1)	12.9% (7.8 to 17.8%)	36.8 (22.9 to 50.9)
Ischemic heart disease	Virgin Islands, U.S.	1990	Age-standardized		13.5% (8.2 to 19.2%)	32.6 (19.8 to 46.3)
Ischemic heart disease	Virgin Islands, U.S.	1995	Age-standardized		13.7% (8.7 to 18.9%)	29.0 (18.2 to 40.4)
Ischemic heart disease	Virgin Islands, U.S.	2000	Age-standardized		14.0% (8.6 to 19.7%)	27.8 (17.1 to 39.3)
Ischemic heart disease	Virgin Islands, U.S.	2005	Age-standardized		13.2% (7.9 to 18.8%)	25.3 (15.3 to 36.4)
Ischemic heart disease	Virgin Islands, U.S.	2010	Age-standardized		12.3% (6.2 to 18.2%)	23.4 (11.8 to 34.4)
Ischemic heart disease	Virgin Islands, U.S.	2015	Age-standardized		12.8% (7.7 to 17.9%)	23.9 (14.6 to 33.4)
Ischemic stroke	Virgin Islands, U.S.	1990	All Ages	0.0 (0.0 to 0.0)	10.3% (5.5 to 15.4%)	2.1 (1.1 to 3.2)
Ischemic stroke	Virgin Islands, U.S.	1995	All Ages	0.0 (0.0 to 0.0)	10.2% (5.4 to 15.4%)	2.4 (1.2 to 3.7)
Ischemic stroke	Virgin Islands, U.S.	2000	All Ages	0.0 (0.0 to 0.0)	10.3% (5.2 to 16.1%)	2.6 (1.3 to 4.2)
Ischemic stroke	Virgin Islands, U.S.	2005	All Ages	0.0 (0.0 to 0.0)	9.5% (4.5 to 14.9%)	2.7 (1.3 to 4.3)
Ischemic stroke	Virgin Islands, U.S.	2010	All Ages	0.0 (0.0 to 0.0)	8.7% (3.2 to 14.3%)	3.0 (1.1 to 5.0)
Ischemic stroke	Virgin Islands, U.S.	2015	All Ages	0.0 (0.0 to 0.0)	9.0% (4.2 to 14.4%)	3.9 (1.8 to 6.2)
Ischemic stroke	Virgin Islands, U.S.	1990	Age-standardized		9.6% (4.8 to 15.0%)	3.8 (1.9 to 6.1)
Ischemic stroke	Virgin Islands, U.S.	1995	Age-standardized		9.7% (5.0 to 14.9%)	3.4 (1.7 to 5.3)
Ischemic stroke	Virgin Islands, U.S.	2000	Age-standardized		10.0% (4.8 to 15.7%)	3.1 (1.5 to 4.9)
Ischemic stroke	Virgin Islands, U.S.	2005	Age-standardized		9.2% (4.2 to 14.7%)	2.6 (1.2 to 4.3)
Ischemic stroke	Virgin Islands, U.S.	2010	Age-standardized		8.5% (3.0 to 14.2%)	2.5 (0.9 to 4.2)
Ischemic stroke	Virgin Islands, U.S.	2015	Age-standardized		8.9% (4.1 to 14.5%)	2.5 (1.2 to 4.1)
Hemorrhagic stroke	Virgin Islands, U.S.	1990	All Ages	0.0 (0.0 to 0.0)	11.4% (6.2 to 17.0%)	2.7 (1.5 to 4.1)
Hemorrhagic stroke	Virgin Islands, U.S.	1995	All Ages	0.0 (0.0 to 0.0)	11.4% (6.2 to 17.2%)	3.1 (1.7 to 4.6)
Hemorrhagic stroke	Virgin Islands, U.S.	2000	All Ages	0.0 (0.0 to 0.0)	11.6% (6.3 to 17.7%)	3.6 (1.9 to 5.6)
Hemorrhagic stroke	Virgin Islands, U.S.	2005	All Ages	0.0 (0.0 to 0.0)	10.6% (5.3 to 16.3%)	3.7 (1.9 to 5.6)
Hemorrhagic stroke	Virgin Islands, U.S.	2010	All Ages	0.0 (0.0 to 0.0)	9.6% (3.6 to 15.1%)	4.1 (1.5 to 6.3)
Hemorrhagic stroke	Virgin Islands, U.S.	2015	All Ages	0.0 (0.0 to 0.0)	9.8% (5.1 to 14.9%)	5.0 (2.5 to 7.8)
Hemorrhagic stroke	Virgin Islands, U.S.	1990	Age-standardized		10.6% (5.7 to 16.0%)	4.1 (2.2 to 6.3)
Hemorrhagic stroke	Virgin Islands, U.S.	1995	Age-standardized		10.7% (5.9 to 16.1%)	3.8 (2.1 to 5.7)
Hemorrhagic stroke	Virgin Islands, U.S.	2000	Age-standardized		11.0% (5.9 to 16.9%)	3.8 (2.0 to 5.9)
Hemorrhagic stroke	Virgin Islands, U.S.	2005	Age-standardized		10.2% (5.0 to 15.6%)	3.3 (1.7 to 5.1)
Hemorrhagic stroke	Virgin Islands, U.S.	2010	Age-standardized		9.4% (3.5 to 15.1%)	3.1 (1.1 to 5.0)

Cause name	Location	Year	Age group	Number (in thousands)	PAF	Rate (per 100,000)
Hemorrhagic stroke	Virgin Islands, U.S.	2015	Age-standardized		9.8% (5.0 to 14.9%)	3.2 (1.7 to 5.1)
Chronic obstructive pulmonary disease	Virgin Islands, U.S.	1990	All Ages	0.0 (0.0 to 0.0)	16.3% (4.2 to 30.8%)	1.4 (0.4 to 2.7)
Chronic obstructive pulmonary disease	Virgin Islands, U.S.	1995	All Ages	0.0 (0.0 to 0.0)	16.4% (5.0 to 30.0%)	1.6 (0.5 to 3.0)
Chronic obstructive pulmonary disease	Virgin Islands, U.S.	2000	All Ages	0.0 (0.0 to 0.0)	17.5% (4.9 to 31.8%)	1.9 (0.5 to 3.6)
Chronic obstructive pulmonary disease	Virgin Islands, U.S.	2005	All Ages	0.0 (0.0 to 0.0)	16.0% (4.6 to 29.2%)	2.1 (0.6 to 3.8)
Chronic obstructive pulmonary disease	Virgin Islands, U.S.	2010	All Ages	0.0 (0.0 to 0.0)	14.5% (1.7 to 29.1%)	2.2 (0.3 to 4.6)
Chronic obstructive pulmonary disease	Virgin Islands, U.S.	2015	All Ages	0.0 (0.0 to 0.0)	15.3% (4.1 to 30.1%)	2.9 (0.8 to 5.7)
Chronic obstructive pulmonary disease	Virgin Islands, U.S.	1990	Age-standardized		16.5% (4.3 to 31.1%)	2.6 (0.7 to 5.0)
Chronic obstructive pulmonary disease	Virgin Islands, U.S.	1995	Age-standardized		16.5% (5.1 to 30.1%)	2.3 (0.7 to 4.3)
Chronic obstructive pulmonary disease	Virgin Islands, U.S.	2000	Age-standardized		17.5% (4.9 to 31.8%)	2.3 (0.6 to 4.2)
Chronic obstructive pulmonary disease	Virgin Islands, U.S.	2005	Age-standardized		16.0% (4.6 to 29.2%)	2.0 (0.6 to 3.7)
Chronic obstructive pulmonary disease	Virgin Islands, U.S.	2010	Age-standardized		14.4% (1.7 to 29.0%)	1.8 (0.2 to 3.7)
Chronic obstructive pulmonary disease	Virgin Islands, U.S.	2015	Age-standardized		15.2% (4.1 to 30.0%)	1.9 (0.5 to 3.7)
All causes	Brazil	1990	All Ages	42.2 (35.0 to 50.2)	4.6% (3.9 to 5.5%)	28.0 (23.2 to 33.4)
All causes	Brazil	1995	All Ages	45.9 (38.5 to 54.6)	4.7% (4.0 to 5.6%)	28.1 (23.6 to 33.5)
All causes	Brazil	2000	All Ages	49.3 (41.1 to 58.5)	4.8% (4.0 to 5.7%)	28.0 (23.4 to 33.3)
All causes	Brazil	2005	All Ages	46.9 (38.6 to 56.4)	4.3% (3.6 to 5.2%)	24.9 (20.5 to 29.9)
All causes	Brazil	2010	All Ages	43.1 (34.7 to 53.4)	3.6% (2.9 to 4.5%)	21.7 (17.4 to 26.8)
All causes	Brazil	2015	All Ages	52.3 (41.9 to 65.1)	3.9% (3.1 to 4.8%)	25.2 (20.2 to 31.3)
All causes	Brazil	1990	Age-standardized		5.5% (4.5 to 6.6%)	60.8 (49.8 to 72.7)
All causes	Brazil	1995	Age-standardized		5.5% (4.5 to 6.5%)	57.8 (47.4 to 69.5)
All causes	Brazil	2000	Age-standardized		5.4% (4.4 to 6.5%)	52.6 (43.1 to 63.6)
All causes	Brazil	2005	Age-standardized		4.7% (3.7 to 5.7%)	40.7 (32.6 to 49.9)
All causes	Brazil	2010	Age-standardized		3.8% (3.0 to 4.8%)	30.6 (24.2 to 38.7)
All causes	Brazil	2015	Age-standardized		3.9% (3.1 to 5.0%)	30.9 (24.2 to 39.0)
Lower respiratory infections	Brazil	1990	All Ages	7.2 (2.9 to 13.7)	12.1% (4.8 to 22.3%)	4.8 (1.9 to 9.1)
Lower respiratory infections	Brazil	1995	All Ages	7.2 (3.0 to 13.2)	12.8% (5.4 to 23.0%)	4.4 (1.8 to 8.1)
Lower respiratory infections	Brazil	2000	All Ages	7.3 (3.2 to 13.2)	13.5% (5.9 to 23.7%)	4.2 (1.8 to 7.5)
Lower respiratory infections	Brazil	2005	All Ages	6.7 (2.4 to 13.1)	11.7% (4.3 to 22.3%)	3.6 (1.3 to 7.0)
Lower respiratory infections	Brazil	2010	All Ages	6.2 (1.8 to 13.6)	9.5% (2.8 to 20.0%)	3.1 (0.9 to 6.8)
Lower respiratory infections	Brazil	2015	All Ages	7.5 (2.3 to 16.1)	9.9% (3.0 to 20.5%)	3.6 (1.1 to 7.8)
Lower respiratory infections	Brazil	1990	Age-standardized		12.6% (5.1 to 22.8%)	8.0 (3.3 to 15.1)
Lower respiratory infections	Brazil	1995	Age-standardized		13.0% (5.6 to 23.2%)	7.8 (3.3 to 14.3)
Lower respiratory infections	Brazil	2000	Age-standardized		13.7% (6.0 to 23.9%)	7.4 (3.3 to 13.4)
Lower respiratory infections	Brazil	2005	Age-standardized		11.8% (4.3 to 22.4%)	5.9 (2.1 to 11.6)
Lower respiratory infections	Brazil	2010	Age-standardized		9.5% (2.8 to 20.0%)	4.6 (1.3 to 10.1)

Cause name	Location	Year	Age group	Number (in thousands)	PAF	Rate (per 100,000)
Lower respiratory infections	Brazil	2015	Age-standardized		9.9% (3.0 to 20.5%)	4.7 (1.4 to 10.0)
Tracheal, bronchus, and lung cancer	Brazil	1990	All Ages	1.2 (0.6 to 2.0)	9.1% (4.7 to 14.6%)	0.8 (0.4 to 1.3)
Tracheal, bronchus, and lung cancer	Brazil	1995	All Ages	1.6 (0.8 to 2.5)	9.4% (4.9 to 15.0%)	1.0 (0.5 to 1.5)
Tracheal, bronchus, and lung cancer	Brazil	2000	All Ages	2.0 (1.0 to 3.1)	9.8% (5.1 to 15.5%)	1.1 (0.6 to 1.8)
Tracheal, bronchus, and lung cancer	Brazil	2005	All Ages	1.9 (1.0 to 3.2)	8.3% (4.1 to 13.6%)	1.0 (0.5 to 1.7)
Tracheal, bronchus, and lung cancer	Brazil	2010	All Ages	1.7 (0.8 to 3.0)	6.5% (3.0 to 11.1%)	0.9 (0.4 to 1.5)
Tracheal, bronchus, and lung cancer	Brazil	2015	All Ages	2.2 (1.0 to 3.6)	6.7% (3.1 to 11.3%)	1.0 (0.5 to 1.8)
Tracheal, bronchus, and lung cancer	Brazil	1990	Age-standardized		9.1% (4.7 to 14.6%)	1.7 (0.9 to 2.7)
Tracheal, bronchus, and lung cancer	Brazil	1995	Age-standardized		9.4% (4.9 to 15.0%)	1.9 (1.0 to 3.0)
Tracheal, bronchus, and lung cancer	Brazil	2000	Age-standardized		9.8% (5.1 to 15.5%)	2.0 (1.0 to 3.1)
Tracheal, bronchus, and lung cancer	Brazil	2005	Age-standardized		8.3% (4.1 to 13.6%)	1.6 (0.8 to 2.6)
Tracheal, bronchus, and lung cancer	Brazil	2010	Age-standardized		6.5% (3.0 to 11.1%)	1.2 (0.5 to 2.0)
Tracheal, bronchus, and lung cancer	Brazil	2015	Age-standardized		6.7% (3.1 to 11.3%)	1.2 (0.6 to 2.1)
Cardiovascular diseases	Brazil	1990	All Ages	28.6 (24.2 to 33.2)	10.7% (9.0 to 12.4%)	19.0 (16.1 to 22.0)
Cardiovascular diseases	Brazil	1995	All Ages	30.4 (25.6 to 35.2)	10.5% (8.8 to 12.1%)	18.6 (15.7 to 21.6)
Cardiovascular diseases	Brazil	2000	All Ages	31.8 (26.6 to 37.0)	10.2% (8.6 to 11.9%)	18.1 (15.2 to 21.0)
Cardiovascular diseases	Brazil	2005	All Ages	30.7 (25.4 to 36.2)	9.4% (7.8 to 11.0%)	16.3 (13.5 to 19.2)
Cardiovascular diseases	Brazil	2010	All Ages	28.7 (23.2 to 34.4)	8.1% (6.6 to 9.6%)	14.4 (11.7 to 17.3)
Cardiovascular diseases	Brazil	2015	All Ages	34.4 (27.6 to 41.7)	8.1% (6.6 to 9.7%)	16.5 (13.3 to 20.1)
Cardiovascular diseases	Brazil	1990	Age-standardized		9.8% (8.0 to 11.5%)	42.0 (34.3 to 49.8)
Cardiovascular diseases	Brazil	1995	Age-standardized		9.6% (7.8 to 11.3%)	38.0 (31.1 to 45.2)
Cardiovascular diseases	Brazil	2000	Age-standardized		9.4% (7.7 to 11.2%)	33.2 (27.0 to 39.6)
Cardiovascular diseases	Brazil	2005	Age-standardized		8.8% (7.1 to 10.4%)	25.9 (20.8 to 31.2)
Cardiovascular diseases	Brazil	2010	Age-standardized		7.6% (6.1 to 9.2%)	19.8 (15.6 to 24.2)
Cardiovascular diseases	Brazil	2015	Age-standardized		7.8% (6.1 to 9.4%)	19.9 (15.7 to 24.4)
Ischemic heart disease	Brazil	1990	All Ages	18.2 (15.0 to 21.8)	14.3% (11.7 to 16.9%)	12.1 (9.9 to 14.5)
Ischemic heart disease	Brazil	1995	All Ages	19.3 (15.9 to 22.9)	14.4% (11.8 to 17.1%)	11.9 (9.7 to 14.1)
Ischemic heart disease	Brazil	2000	All Ages	20.4 (16.5 to 24.2)	14.4% (11.8 to 17.0%)	11.6 (9.4 to 13.8)
Ischemic heart disease	Brazil	2005	All Ages	20.0 (16.1 to 24.0)	13.4% (10.8 to 16.0%)	10.6 (8.6 to 12.7)
Ischemic heart disease	Brazil	2010	All Ages	19.1 (14.9 to 23.4)	11.7% (9.2 to 14.3%)	9.6 (7.5 to 11.7)
Ischemic heart disease	Brazil	2015	All Ages	22.9 (17.7 to 28.4)	11.7% (9.1 to 14.4%)	11.0 (8.5 to 13.7)
Ischemic heart disease	Brazil	1990	Age-standardized		12.9% (10.0 to 15.7%)	26.9 (20.9 to 32.9)
Ischemic heart disease	Brazil	1995	Age-standardized		12.9% (10.0 to 15.8%)	24.2 (18.7 to 29.6)
Ischemic heart disease	Brazil	2000	Age-standardized		13.1% (10.1 to 16.1%)	21.2 (16.3 to 26.0)
Ischemic heart disease	Brazil	2005	Age-standardized		12.4% (9.6 to 15.3%)	16.7 (12.8 to 20.7)
Ischemic heart disease	Brazil	2010	Age-standardized		11.0% (8.4 to 13.8%)	13.1 (9.8 to 16.4)

Cause name	Location	Year	Age group	Number (in thousands)	PAF	Rate (per 100,000)
Ischemic heart disease	Brazil	2015	Age-standardized		11.2% (8.4 to 14.0%)	13.2 (9.8 to 16.7)
Ischemic stroke	Brazil	1990	All Ages	2.8 (1.9 to 3.7)	8.9% (6.1 to 11.8%)	1.9 (1.3 to 2.5)
Ischemic stroke	Brazil	1995	All Ages	2.9 (2.0 to 3.9)	8.9% (6.0 to 11.9%)	1.8 (1.2 to 2.4)
Ischemic stroke	Brazil	2000	All Ages	2.9 (1.9 to 3.9)	8.9% (5.8 to 12.1%)	1.6 (1.1 to 2.2)
Ischemic stroke	Brazil	2005	All Ages	2.8 (1.7 to 3.9)	8.1% (5.1 to 11.6%)	1.5 (0.9 to 2.1)
Ischemic stroke	Brazil	2010	All Ages	2.7 (1.6 to 4.0)	7.0% (4.1 to 10.4%)	1.3 (0.8 to 2.0)
Ischemic stroke	Brazil	2015	All Ages	3.4 (1.9 to 5.2)	7.1% (4.0 to 10.9%)	1.6 (0.9 to 2.5)
Ischemic stroke	Brazil	1990	Age-standardized		8.3% (5.1 to 11.8%)	5.1 (3.2 to 7.4)
Ischemic stroke	Brazil	1995	Age-standardized		8.3% (5.1 to 12.1%)	4.5 (2.8 to 6.6)
Ischemic stroke	Brazil	2000	Age-standardized		8.4% (5.1 to 12.4%)	3.7 (2.2 to 5.5)
Ischemic stroke	Brazil	2005	Age-standardized		7.8% (4.5 to 11.7%)	2.8 (1.6 to 4.2)
Ischemic stroke	Brazil	2010	Age-standardized		6.8% (3.7 to 10.6%)	2.1 (1.2 to 3.3)
Ischemic stroke	Brazil	2015	Age-standardized		6.9% (3.8 to 11.0%)	2.2 (1.2 to 3.4)
Hemorrhagic stroke	Brazil	1990	All Ages	7.5 (5.7 to 9.5)	10.8% (8.2 to 13.5%)	5.0 (3.8 to 6.3)
Hemorrhagic stroke	Brazil	1995	All Ages	8.2 (6.2 to 10.2)	10.8% (8.2 to 13.6%)	5.0 (3.8 to 6.3)
Hemorrhagic stroke	Brazil	2000	All Ages	8.6 (6.5 to 10.7)	10.8% (8.2 to 13.4%)	4.9 (3.7 to 6.1)
Hemorrhagic stroke	Brazil	2005	All Ages	7.9 (5.8 to 10.1)	9.8% (7.2 to 12.5%)	4.2 (3.1 to 5.4)
Hemorrhagic stroke	Brazil	2010	All Ages	6.9 (4.8 to 9.1)	8.3% (5.8 to 10.8%)	3.5 (2.4 to 4.6)
Hemorrhagic stroke	Brazil	2015	All Ages	8.1 (5.6 to 10.7)	8.3% (5.8 to 11.0%)	3.9 (2.7 to 5.1)
Hemorrhagic stroke	Brazil	1990	Age-standardized		9.8% (7.2 to 12.5%)	9.9 (7.3 to 12.7)
Hemorrhagic stroke	Brazil	1995	Age-standardized		9.8% (7.3 to 12.6%)	9.3 (6.9 to 11.9)
Hemorrhagic stroke	Brazil	2000	Age-standardized		9.9% (7.3 to 12.7%)	8.3 (6.1 to 10.7)
Hemorrhagic stroke	Brazil	2005	Age-standardized		9.1% (6.5 to 12.0%)	6.4 (4.5 to 8.4)
Hemorrhagic stroke	Brazil	2010	Age-standardized		7.8% (5.3 to 10.5%)	4.6 (3.2 to 6.2)
Hemorrhagic stroke	Brazil	2015	Age-standardized		8.0% (5.4 to 10.8%)	4.5 (3.1 to 6.2)
Chronic obstructive pulmonary disease	Brazil	1990	All Ages	5.2 (2.3 to 8.8)	14.2% (6.2 to 24.1%)	3.5 (1.5 to 5.9)
Chronic obstructive pulmonary disease	Brazil	1995	All Ages	6.7 (2.9 to 11.3)	14.6% (6.5 to 24.6%)	4.1 (1.8 to 6.9)
Chronic obstructive pulmonary disease	Brazil	2000	All Ages	8.2 (3.6 to 13.6)	15.1% (6.8 to 25.3%)	4.6 (2.1 to 7.7)
Chronic obstructive pulmonary disease	Brazil	2005	All Ages	7.5 (3.1 to 13.0)	13.6% (5.7 to 23.5%)	4.0 (1.7 to 6.9)
Chronic obstructive pulmonary disease	Brazil	2010	All Ages	6.6 (2.4 to 12.0)	11.3% (4.0 to 20.5%)	3.3 (1.2 to 6.1)
Chronic obstructive pulmonary disease	Brazil	2015	All Ages	8.2 (3.1 to 14.9)	11.6% (4.2 to 21.1%)	4.0 (1.5 to 7.2)
Chronic obstructive pulmonary disease	Brazil	1990	Age-standardized		14.2% (6.2 to 24.3%)	9.2 (4.0 to 15.6)
Chronic obstructive pulmonary disease	Brazil	1995	Age-standardized		14.6% (6.5 to 24.6%)	10.1 (4.4 to 17.0)
Chronic obstructive pulmonary disease	Brazil	2000	Age-standardized		15.1% (6.8 to 25.4%)	10.0 (4.5 to 16.8)
Chronic obstructive pulmonary disease	Brazil	2005	Age-standardized		13.6% (5.7 to 23.5%)	7.3 (3.0 to 12.6)
Chronic obstructive pulmonary disease	Brazil	2010	Age-standardized		11.3% (4.0 to 20.5%)	5.1 (1.8 to 9.3)

Cause name	Location	Year	Age group	Number (in thousands)	PAF	Rate (per 100,000)
Chronic obstructive pulmonary disease	Brazil	2015	Age-standardized		11.6% (4.2 to 21.1%)	5.2 (1.9 to 9.4)
All causes	Paraguay	1990	All Ages	1.0 (0.8 to 1.2)	5.7% (4.7 to 6.6%)	23.5 (19.4 to 27.9)
All causes	Paraguay	1995	All Ages	1.2 (1.0 to 1.4)	5.9% (4.9 to 6.9%)	24.7 (20.5 to 29.5)
All causes	Paraguay	2000	All Ages	1.3 (1.1 to 1.5)	5.5% (4.6 to 6.4%)	23.6 (19.7 to 28.0)
All causes	Paraguay	2005	All Ages	1.5 (1.2 to 1.7)	5.4% (4.6 to 6.2%)	24.9 (20.7 to 29.2)
All causes	Paraguay	2010	All Ages	1.5 (1.2 to 1.8)	4.7% (3.9 to 5.6%)	23.8 (19.5 to 28.7)
All causes	Paraguay	2015	All Ages	1.6 (1.2 to 1.9)	4.4% (3.6 to 5.2%)	23.5 (18.7 to 28.4)
All causes	Paraguay	1990	Age-standardized		6.8% (5.8 to 8.0%)	51.1 (41.9 to 60.2)
All causes	Paraguay	1995	Age-standardized		6.9% (5.8 to 8.1%)	52.5 (43.4 to 62.5)
All causes	Paraguay	2000	Age-standardized		6.3% (5.2 to 7.4%)	47.6 (39.6 to 56.4)
All causes	Paraguay	2005	Age-standardized		6.0% (5.1 to 7.0%)	46.9 (38.9 to 55.1)
All causes	Paraguay	2010	Age-standardized		5.1% (4.2 to 6.1%)	41.2 (33.7 to 49.8)
All causes	Paraguay	2015	Age-standardized		4.7% (3.8 to 5.6%)	37.5 (29.5 to 45.9)
Lower respiratory infections	Paraguay	1990	All Ages	0.3 (0.1 to 0.4)	18.2% (9.5 to 28.3%)	5.8 (3.0 to 9.3)
Lower respiratory infections	Paraguay	1995	All Ages	0.3 (0.1 to 0.4)	18.6% (9.9 to 28.6%)	5.7 (3.1 to 8.8)
Lower respiratory infections	Paraguay	2000	All Ages	0.3 (0.1 to 0.5)	18.6% (10.0 to 28.4%)	5.5 (2.9 to 8.8)
Lower respiratory infections	Paraguay	2005	All Ages	0.3 (0.2 to 0.5)	18.8% (10.3 to 28.9%)	5.0 (2.7 to 8.0)
Lower respiratory infections	Paraguay	2010	All Ages	0.3 (0.1 to 0.4)	14.9% (6.4 to 25.4%)	4.0 (1.7 to 7.1)
Lower respiratory infections	Paraguay	2015	All Ages	0.2 (0.1 to 0.4)	12.8% (4.8 to 23.6%)	3.3 (1.2 to 6.5)
Lower respiratory infections	Paraguay	1990	Age-standardized		18.2% (9.5 to 28.3%)	8.3 (4.3 to 13.3)
Lower respiratory infections	Paraguay	1995	Age-standardized		18.6% (9.9 to 28.6%)	8.9 (4.8 to 14.2)
Lower respiratory infections	Paraguay	2000	Age-standardized		18.6% (10.0 to 28.4%)	9.0 (4.8 to 14.6)
Lower respiratory infections	Paraguay	2005	Age-standardized		18.8% (10.3 to 28.9%)	8.4 (4.5 to 13.5)
Lower respiratory infections	Paraguay	2010	Age-standardized		14.9% (6.4 to 25.4%)	6.5 (2.7 to 11.7)
Lower respiratory infections	Paraguay	2015	Age-standardized		12.8% (4.8 to 23.6%)	5.2 (1.8 to 10.1)
Tracheal, bronchus, and lung cancer	Paraguay	1990	All Ages	0.0 (0.0 to 0.1)	12.8% (7.1 to 19.3%)	0.7 (0.4 to 1.1)
Tracheal, bronchus, and lung cancer	Paraguay	1995	All Ages	0.0 (0.0 to 0.1)	13.0% (7.3 to 19.6%)	0.8 (0.5 to 1.3)
Tracheal, bronchus, and lung cancer	Paraguay	2000	All Ages	0.1 (0.0 to 0.1)	13.0% (7.2 to 19.7%)	1.0 (0.6 to 1.6)
Tracheal, bronchus, and lung cancer	Paraguay	2005	All Ages	0.1 (0.0 to 0.1)	13.2% (7.3 to 19.7%)	1.2 (0.7 to 1.9)
Tracheal, bronchus, and lung cancer	Paraguay	2010	All Ages	0.1 (0.0 to 0.1)	10.3% (5.3 to 16.4%)	1.1 (0.6 to 1.8)
Tracheal, bronchus, and lung cancer	Paraguay	2015	All Ages	0.1 (0.0 to 0.1)	8.8% (4.4 to 14.2%)	1.1 (0.6 to 1.8)
Tracheal, bronchus, and lung cancer	Paraguay	1990	Age-standardized		12.8% (7.1 to 19.4%)	1.6 (0.9 to 2.5)
Tracheal, bronchus, and lung cancer	Paraguay	1995	Age-standardized		13.1% (7.3 to 19.7%)	1.8 (1.0 to 2.7)
Tracheal, bronchus, and lung cancer	Paraguay	2000	Age-standardized		13.1% (7.2 to 19.8%)	2.1 (1.2 to 3.2)
Tracheal, bronchus, and lung cancer	Paraguay	2005	Age-standardized		13.2% (7.3 to 19.8%)	2.3 (1.3 to 3.5)
Tracheal, bronchus, and lung cancer	Paraguay	2010	Age-standardized		10.3% (5.3 to 16.4%)	1.9 (1.0 to 3.1)

Cause name	Location	Year	Age group	Number (in thousands)	PAF	Rate (per 100,000)
Tracheal, bronchus, and lung cancer	Paraguay	2015	Age-standardized		8.9% (4.4 to 14.2%)	1.7 (0.9 to 2.8)
Cardiovascular diseases	Paraguay	1990	All Ages	0.6 (0.5 to 0.8)	12.7% (10.7 to 14.6%)	15.2 (12.7 to 17.7)
Cardiovascular diseases	Paraguay	1995	All Ages	0.8 (0.7 to 0.9)	12.7% (10.8 to 14.7%)	16.3 (13.5 to 19.2)
Cardiovascular diseases	Paraguay	2000	All Ages	0.8 (0.7 to 0.9)	12.3% (10.4 to 14.1%)	15.0 (12.6 to 17.6)
Cardiovascular diseases	Paraguay	2005	All Ages	1.0 (0.8 to 1.1)	12.1% (10.2 to 14.0%)	16.3 (13.5 to 19.4)
Cardiovascular diseases	Paraguay	2010	All Ages	1.0 (0.8 to 1.2)	10.7% (8.9 to 12.5%)	16.5 (13.5 to 19.8)
Cardiovascular diseases	Paraguay	2015	All Ages	1.1 (0.9 to 1.4)	10.0% (8.2 to 11.8%)	16.8 (13.2 to 20.7)
Cardiovascular diseases	Paraguay	1990	Age-standardized		12.0% (9.9 to 14.1%)	36.4 (29.6 to 43.6)
Cardiovascular diseases	Paraguay	1995	Age-standardized		12.1% (9.9 to 14.2%)	36.9 (29.9 to 44.1)
Cardiovascular diseases	Paraguay	2000	Age-standardized		11.7% (9.7 to 13.7%)	31.5 (25.9 to 37.7)
Cardiovascular diseases	Paraguay	2005	Age-standardized		11.6% (9.6 to 13.6%)	31.2 (25.2 to 37.8)
Cardiovascular diseases	Paraguay	2010	Age-standardized		10.3% (8.4 to 12.2%)	28.7 (23.0 to 34.7)
Cardiovascular diseases	Paraguay	2015	Age-standardized		9.6% (7.8 to 11.5%)	26.7 (20.8 to 33.2)
Ischemic heart disease	Paraguay	1990	All Ages	0.4 (0.3 to 0.5)	15.8% (12.8 to 18.9%)	9.4 (7.5 to 11.4)
Ischemic heart disease	Paraguay	1995	All Ages	0.5 (0.4 to 0.6)	15.9% (12.9 to 19.0%)	10.0 (8.0 to 12.1)
Ischemic heart disease	Paraguay	2000	All Ages	0.5 (0.4 to 0.6)	15.8% (12.8 to 18.9%)	9.1 (7.2 to 10.9)
Ischemic heart disease	Paraguay	2005	All Ages	0.6 (0.5 to 0.7)	15.8% (12.8 to 19.0%)	9.9 (7.8 to 12.1)
Ischemic heart disease	Paraguay	2010	All Ages	0.7 (0.5 to 0.8)	14.3% (11.3 to 17.2%)	10.5 (8.2 to 13.0)
Ischemic heart disease	Paraguay	2015	All Ages	0.7 (0.5 to 0.9)	13.3% (10.3 to 16.1%)	10.9 (8.1 to 13.7)
Ischemic heart disease	Paraguay	1990	Age-standardized		14.7% (11.4 to 18.2%)	22.7 (17.4 to 28.3)
Ischemic heart disease	Paraguay	1995	Age-standardized		14.9% (11.6 to 18.3%)	22.8 (17.4 to 28.5)
Ischemic heart disease	Paraguay	2000	Age-standardized		14.9% (11.6 to 18.3%)	19.2 (14.7 to 23.6)
Ischemic heart disease	Paraguay	2005	Age-standardized		15.1% (11.8 to 18.5%)	19.0 (14.5 to 23.6)
Ischemic heart disease	Paraguay	2010	Age-standardized		13.7% (10.5 to 16.8%)	18.1 (13.8 to 22.9)
Ischemic heart disease	Paraguay	2015	Age-standardized		12.8% (9.7 to 15.8%)	17.3 (12.6 to 22.1)
Ischemic stroke	Paraguay	1990	All Ages	0.1 (0.1 to 0.1)	10.7% (7.5 to 14.1%)	1.8 (1.2 to 2.4)
Ischemic stroke	Paraguay	1995	All Ages	0.1 (0.1 to 0.1)	10.8% (7.5 to 14.1%)	1.8 (1.2 to 2.4)
Ischemic stroke	Paraguay	2000	All Ages	0.1 (0.1 to 0.1)	10.6% (7.2 to 14.3%)	1.4 (0.9 to 1.9)
Ischemic stroke	Paraguay	2005	All Ages	0.1 (0.1 to 0.1)	10.6% (7.2 to 14.3%)	1.5 (1.0 to 2.1)
Ischemic stroke	Paraguay	2010	All Ages	0.1 (0.1 to 0.1)	9.2% (5.8 to 13.3%)	1.4 (0.9 to 2.1)
Ischemic stroke	Paraguay	2015	All Ages	0.1 (0.1 to 0.1)	8.5% (5.1 to 12.5%)	1.5 (0.9 to 2.3)
Ischemic stroke	Paraguay	1990	Age-standardized		10.3% (6.8 to 14.2%)	4.9 (3.1 to 6.8)
Ischemic stroke	Paraguay	1995	Age-standardized		10.4% (6.9 to 14.4%)	4.7 (3.1 to 6.7)
Ischemic stroke	Paraguay	2000	Age-standardized		10.3% (6.6 to 14.4%)	3.3 (2.1 to 4.8)
Ischemic stroke	Paraguay	2005	Age-standardized		10.3% (6.7 to 14.3%)	3.1 (2.0 to 4.5)
Ischemic stroke	Paraguay	2010	Age-standardized		9.1% (5.6 to 13.4%)	2.8 (1.7 to 4.2)

Cause name	Location	Year	Age group	Number (in thousands)	PAF	Rate (per 100,000)
Ischemic stroke	Paraguay	2015	Age-standardized		8.3% (4.8 to 12.5%)	2.6 (1.5 to 4.0)
Hemorrhagic stroke	Paraguay	1990	All Ages	0.2 (0.1 to 0.2)	12.6% (9.9 to 15.4%)	4.0 (3.1 to 4.9)
Hemorrhagic stroke	Paraguay	1995	All Ages	0.2 (0.2 to 0.3)	12.8% (10.0 to 15.6%)	4.4 (3.4 to 5.5)
Hemorrhagic stroke	Paraguay	2000	All Ages	0.2 (0.2 to 0.3)	12.6% (9.8 to 15.4%)	4.6 (3.5 to 5.7)
Hemorrhagic stroke	Paraguay	2005	All Ages	0.3 (0.2 to 0.4)	12.7% (9.9 to 15.4%)	5.0 (3.8 to 6.3)
Hemorrhagic stroke	Paraguay	2010	All Ages	0.3 (0.2 to 0.4)	11.0% (8.2 to 13.8%)	4.6 (3.4 to 6.0)
Hemorrhagic stroke	Paraguay	2015	All Ages	0.3 (0.2 to 0.4)	10.1% (7.3 to 12.9%)	4.4 (3.1 to 5.8)
Hemorrhagic stroke	Paraguay	1990	Age-standardized		12.1% (9.3 to 15.0%)	8.8 (6.6 to 11.1)
Hemorrhagic stroke	Paraguay	1995	Age-standardized		12.2% (9.4 to 15.0%)	9.4 (7.0 to 11.8)
Hemorrhagic stroke	Paraguay	2000	Age-standardized		12.1% (9.3 to 15.0%)	9.1 (6.8 to 11.6)
Hemorrhagic stroke	Paraguay	2005	Age-standardized		12.1% (9.3 to 15.1%)	9.1 (6.8 to 11.6)
Hemorrhagic stroke	Paraguay	2010	Age-standardized		10.6% (7.7 to 13.5%)	7.7 (5.5 to 10.2)
Hemorrhagic stroke	Paraguay	2015	Age-standardized		9.7% (6.9 to 12.6%)	6.8 (4.8 to 9.2)
Chronic obstructive pulmonary disease	Paraguay	1990	All Ages	0.1 (0.0 to 0.1)	17.5% (8.3 to 27.4%)	1.8 (0.9 to 2.8)
Chronic obstructive pulmonary disease	Paraguay	1995	All Ages	0.1 (0.1 to 0.1)	18.6% (9.2 to 28.9%)	2.0 (1.0 to 3.0)
Chronic obstructive pulmonary disease	Paraguay	2000	All Ages	0.1 (0.1 to 0.2)	18.8% (9.0 to 29.0%)	2.1 (1.0 to 3.3)
Chronic obstructive pulmonary disease	Paraguay	2005	All Ages	0.1 (0.1 to 0.2)	19.1% (9.3 to 29.4%)	2.3 (1.2 to 3.7)
Chronic obstructive pulmonary disease	Paraguay	2010	All Ages	0.1 (0.1 to 0.2)	16.1% (7.2 to 26.5%)	2.2 (1.0 to 3.5)
Chronic obstructive pulmonary disease	Paraguay	2015	All Ages	0.1 (0.1 to 0.3)	14.5% (6.0 to 24.5%)	2.3 (0.9 to 3.8)
Chronic obstructive pulmonary disease	Paraguay	1990	Age-standardized		18.8% (9.0 to 29.4%)	4.8 (2.3 to 7.6)
Chronic obstructive pulmonary disease	Paraguay	1995	Age-standardized		19.3% (9.5 to 30.0%)	4.9 (2.5 to 7.8)
Chronic obstructive pulmonary disease	Paraguay	2000	Age-standardized		19.3% (9.3 to 30.0%)	5.0 (2.4 to 7.8)
Chronic obstructive pulmonary disease	Paraguay	2005	Age-standardized		19.5% (9.5 to 30.2%)	4.9 (2.4 to 7.7)
Chronic obstructive pulmonary disease	Paraguay	2010	Age-standardized		16.3% (7.2 to 26.8%)	4.1 (1.8 to 6.7)
Chronic obstructive pulmonary disease	Paraguay	2015	Age-standardized		14.6% (6.1 to 24.7%)	3.9 (1.6 to 6.4)
All causes	China	1990	All Ages	945.4 (773.2 to 1,127.3)	10.7% (8.8 to 12.7%)	81.9 (67.0 to 97.6)
All causes	China	1995	All Ages	981.6 (805.6 to 1,167.3)	11.1% (9.1 to 13.3%)	79.5 (65.2 to 94.5)
All causes	China	2000	All Ages	1,039.3 (863.0 to 1,226.8)	11.4% (9.5 to 13.5%)	81.2 (67.4 to 95.9)
All causes	China	2005	All Ages	1,140.1 (972.0 to 1,319.3)	12.0% (10.2 to 13.9%)	86.8 (74.0 to 100.5)
All causes	China	2010	All Ages	1,098.8 (953.0 to 1,258.7)	11.7% (10.2 to 13.4%)	81.5 (70.6 to 93.3)
All causes	China	2015	All Ages	1,108.1 (948.7 to 1,272.8)	11.8% (10.2 to 13.4%)	80.1 (68.6 to 92.0)
All causes	China	1990	Age-standardized		12.1% (9.5 to 14.7%)	147.3 (116.4 to 179.3)
All causes	China	1995	Age-standardized		12.2% (9.7 to 14.8%)	137.2 (109.5 to 166.4)
All causes	China	2000	Age-standardized		12.2% (10.0 to 14.5%)	127.7 (104.8 to 152.5)
All causes	China	2005	Age-standardized		12.5% (10.5 to 14.6%)	120.6 (101.8 to 140.8)
All causes	China	2010	Age-standardized		12.0% (10.3 to 13.8%)	98.8 (84.6 to 113.6)

Cause name	Location	Year	Age group	Number (in thousands)	PAF	Rate (per 100,000)
All causes	China	2015	Age-standardized		11.9% (10.2 to 13.6%)	84.3 (71.5 to 96.7)
Lower respiratory infections	China	1990	All Ages	155.0 (115.2 to 196.6)	29.7% (22.1 to 37.1%)	13.4 (10.0 to 17.0)
Lower respiratory infections	China	1995	All Ages	118.1 (87.8 to 150.4)	29.8% (22.4 to 37.3%)	9.6 (7.1 to 12.2)
Lower respiratory infections	China	2000	All Ages	90.2 (67.6 to 114.5)	30.4% (22.8 to 37.9%)	7.1 (5.3 to 8.9)
Lower respiratory infections	China	2005	All Ages	73.4 (55.3 to 92.6)	32.3% (24.8 to 39.4%)	5.6 (4.2 to 7.1)
Lower respiratory infections	China	2010	All Ages	68.0 (51.1 to 85.9)	32.4% (24.9 to 39.4%)	5.0 (3.8 to 6.4)
Lower respiratory infections	China	2015	All Ages	66.3 (49.4 to 84.1)	32.3% (24.8 to 39.3%)	4.8 (3.6 to 6.1)
Lower respiratory infections	China	1990	Age-standardized		29.7% (22.1 to 37.2%)	15.4 (11.5 to 19.5)
Lower respiratory infections	China	1995	Age-standardized		29.9% (22.4 to 37.4%)	13.6 (10.1 to 17.2)
Lower respiratory infections	China	2000	Age-standardized		30.4% (22.9 to 37.9%)	11.4 (8.5 to 14.4)
Lower respiratory infections	China	2005	Age-standardized		32.3% (24.8 to 39.4%)	8.7 (6.6 to 10.9)
Lower respiratory infections	China	2010	Age-standardized		32.4% (24.9 to 39.4%)	7.0 (5.2 to 8.8)
Lower respiratory infections	China	2015	Age-standardized		32.3% (24.8 to 39.3%)	5.8 (4.3 to 7.3)
Tracheal, bronchus, and lung cancer	China	1990	All Ages	59.4 (37.9 to 82.3)	22.2% (14.4 to 30.2%)	5.1 (3.3 to 7.1)
Tracheal, bronchus, and lung cancer	China	1995	All Ages	70.2 (44.8 to 96.4)	22.6% (14.7 to 30.7%)	5.7 (3.6 to 7.8)
Tracheal, bronchus, and lung cancer	China	2000	All Ages	91.5 (60.0 to 123.8)	23.2% (15.2 to 31.3%)	7.2 (4.7 to 9.7)
Tracheal, bronchus, and lung cancer	China	2005	All Ages	118.4 (78.8 to 158.1)	24.8% (16.6 to 33.0%)	9.0 (6.0 to 12.0)
Tracheal, bronchus, and lung cancer	China	2010	All Ages	134.8 (89.6 to 179.7)	25.1% (16.9 to 33.3%)	10.0 (6.6 to 13.3)
Tracheal, bronchus, and lung cancer	China	2015	All Ages	146.0 (96.6 to 193.7)	25.2% (16.9 to 33.4%)	10.6 (7.0 to 14.0)
Tracheal, bronchus, and lung cancer	China	1990	Age-standardized		22.3% (14.5 to 30.3%)	8.3 (5.3 to 11.5)
Tracheal, bronchus, and lung cancer	China	1995	Age-standardized		22.6% (14.7 to 30.7%)	8.6 (5.5 to 11.8)
Tracheal, bronchus, and lung cancer	China	2000	Age-standardized		23.3% (15.3 to 31.4%)	9.8 (6.5 to 13.3)
Tracheal, bronchus, and lung cancer	China	2005	Age-standardized		24.8% (16.6 to 33.1%)	11.0 (7.4 to 14.8)
Tracheal, bronchus, and lung cancer	China	2010	Age-standardized		25.2% (16.9 to 33.4%)	10.9 (7.2 to 14.5)
Tracheal, bronchus, and lung cancer	China	2015	Age-standardized		25.2% (16.9 to 33.4%)	10.0 (6.6 to 13.3)
Cardiovascular diseases	China	1990	All Ages	347.2 (298.8 to 396.3)	14.0% (12.2 to 15.8%)	30.1 (25.9 to 34.3)
Cardiovascular diseases	China	1995	All Ages	382.6 (329.8 to 434.3)	14.3% (12.4 to 16.1%)	31.0 (26.7 to 35.2)
Cardiovascular diseases	China	2000	All Ages	463.4 (399.9 to 523.7)	15.0% (13.1 to 16.9%)	36.2 (31.3 to 40.9)
Cardiovascular diseases	China	2005	All Ages	580.1 (505.0 to 653.2)	16.4% (14.3 to 18.4%)	44.2 (38.5 to 49.8)
Cardiovascular diseases	China	2010	All Ages	609.0 (530.1 to 691.5)	16.3% (14.1 to 18.3%)	45.1 (39.3 to 51.3)
Cardiovascular diseases	China	2015	All Ages	614.0 (529.0 to 701.0)	16.0% (13.9 to 18.2%)	44.4 (38.2 to 50.7)
Cardiovascular diseases	China	1990	Age-standardized		13.1% (11.1 to 15.0%)	53.4 (45.1 to 61.7)
Cardiovascular diseases	China	1995	Age-standardized		13.3% (11.2 to 15.3%)	50.7 (42.7 to 58.7)
Cardiovascular diseases	China	2000	Age-standardized		14.1% (11.9 to 16.2%)	53.4 (45.0 to 61.7)
Cardiovascular diseases	China	2005	Age-standardized		15.4% (13.0 to 17.7%)	58.3 (49.5 to 67.1)
Cardiovascular diseases	China	2010	Age-standardized		15.4% (13.0 to 17.8%)	52.8 (44.8 to 61.5)

Cause name	Location	Year	Age group	Number (in thousands)	PAF	Rate (per 100,000)
Cardiovascular diseases	China	2015	Age-standardized		15.2% (12.8 to 17.8%)	45.3 (38.3 to 52.9)
Ischemic heart disease	China	1990	All Ages	124.5 (104.7 to 146.8)	20.6% (17.5 to 24.0%)	10.8 (9.1 to 12.7)
Ischemic heart disease	China	1995	All Ages	131.6 (111.2 to 155.7)	20.6% (17.5 to 24.2%)	10.7 (9.0 to 12.6)
Ischemic heart disease	China	2000	All Ages	165.3 (139.1 to 196.4)	20.6% (17.4 to 24.4%)	12.9 (10.9 to 15.3)
Ischemic heart disease	China	2005	All Ages	239.5 (199.4 to 285.9)	20.5% (17.2 to 24.7%)	18.2 (15.2 to 21.8)
Ischemic heart disease	China	2010	All Ages	280.6 (231.2 to 341.6)	20.2% (16.7 to 24.4%)	20.8 (17.1 to 25.3)
Ischemic heart disease	China	2015	All Ages	291.8 (237.2 to 356.8)	20.0% (16.4 to 24.5%)	21.1 (17.1 to 25.8)
Ischemic heart disease	China	1990	Age-standardized		19.0% (15.4 to 23.1%)	19.2 (15.5 to 23.5)
Ischemic heart disease	China	1995	Age-standardized		19.0% (15.4 to 23.3%)	17.4 (14.1 to 21.2)
Ischemic heart disease	China	2000	Age-standardized		19.1% (15.4 to 23.4%)	19.0 (15.4 to 23.2)
Ischemic heart disease	China	2005	Age-standardized		19.2% (15.4 to 23.8%)	24.2 (19.4 to 30.0)
Ischemic heart disease	China	2010	Age-standardized		19.0% (15.1 to 23.9%)	24.6 (19.4 to 31.0)
Ischemic heart disease	China	2015	Age-standardized		19.0% (15.0 to 23.9%)	21.7 (17.0 to 27.4)
Ischemic stroke	China	1990	All Ages	63.7 (50.1 to 80.2)	16.1% (12.9 to 19.1%)	5.5 (4.3 to 6.9)
Ischemic stroke	China	1995	All Ages	69.8 (55.4 to 84.5)	15.9% (12.7 to 18.9%)	5.6 (4.5 to 6.8)
Ischemic stroke	China	2000	All Ages	87.1 (68.6 to 104.6)	15.9% (12.6 to 19.0%)	6.8 (5.4 to 8.2)
Ischemic stroke	China	2005	All Ages	114.0 (89.9 to 137.1)	16.3% (12.9 to 19.5%)	8.7 (6.8 to 10.4)
Ischemic stroke	China	2010	All Ages	121.0 (93.2 to 147.1)	16.2% (12.6 to 19.5%)	9.0 (6.9 to 10.9)
Ischemic stroke	China	2015	All Ages	122.6 (93.0 to 149.9)	16.1% (12.6 to 19.5%)	8.9 (6.7 to 10.8)
Ischemic stroke	China	1990	Age-standardized		15.1% (11.6 to 18.4%)	10.5 (8.1 to 13.4)
Ischemic stroke	China	1995	Age-standardized		15.1% (11.6 to 18.4%)	10.0 (7.6 to 12.2)
Ischemic stroke	China	2000	Age-standardized		15.1% (11.5 to 18.6%)	10.8 (8.2 to 13.4)
Ischemic stroke	China	2005	Age-standardized		15.5% (11.8 to 19.2%)	12.2 (9.2 to 15.1)
Ischemic stroke	China	2010	Age-standardized		15.5% (11.7 to 19.3%)	11.1 (8.3 to 13.9)
Ischemic stroke	China	2015	Age-standardized		15.5% (11.7 to 19.3%)	9.5 (7.0 to 11.9)
Hemorrhagic stroke	China	1990	All Ages	159.0 (131.6 to 188.5)	17.0% (14.1 to 20.1%)	13.8 (11.4 to 16.3)
Hemorrhagic stroke	China	1995	All Ages	181.2 (150.3 to 212.8)	17.1% (14.2 to 20.1%)	14.7 (12.2 to 17.2)
Hemorrhagic stroke	China	2000	All Ages	211.0 (173.0 to 247.3)	17.3% (14.3 to 20.2%)	16.5 (13.5 to 19.3)
Hemorrhagic stroke	China	2005	All Ages	226.7 (188.1 to 266.5)	17.8% (14.9 to 20.7%)	17.3 (14.3 to 20.3)
Hemorrhagic stroke	China	2010	All Ages	207.3 (172.7 to 244.1)	17.8% (14.8 to 20.6%)	15.4 (12.8 to 18.1)
Hemorrhagic stroke	China	2015	All Ages	199.7 (163.8 to 236.6)	17.6% (14.5 to 20.5%)	14.4 (11.8 to 17.1)
Hemorrhagic stroke	China	1990	Age-standardized		16.0% (12.9 to 19.0%)	23.6 (19.2 to 28.2)
Hemorrhagic stroke	China	1995	Age-standardized		16.1% (13.0 to 19.0%)	23.3 (18.9 to 27.6)
Hemorrhagic stroke	China	2000	Age-standardized		16.2% (13.1 to 19.2%)	23.6 (18.9 to 28.1)
Hemorrhagic stroke	China	2005	Age-standardized		16.7% (13.6 to 19.8%)	21.9 (17.6 to 26.0)
Hemorrhagic stroke	China	2010	Age-standardized		16.8% (13.6 to 19.9%)	17.1 (13.8 to 20.5)

Cause name	Location	Year	Age group	Number (in thousands)	PAF	Rate (per 100,000)
Hemorrhagic stroke	China	2015	Age-standardized		16.7% (13.4 to 19.8%)	14.1 (11.3 to 16.9)
Chronic obstructive pulmonary disease	China	1990	All Ages	383.9 (237.5 to 544.6)	28.6% (17.6 to 40.3%)	33.2 (20.6 to 47.2)
Chronic obstructive pulmonary disease	China	1995	All Ages	410.7 (257.4 to 583.9)	29.0% (17.9 to 41.0%)	33.2 (20.8 to 47.3)
Chronic obstructive pulmonary disease	China	2000	All Ages	394.1 (244.9 to 563.2)	29.6% (18.4 to 41.8%)	30.8 (19.1 to 44.0)
Chronic obstructive pulmonary disease	China	2005	All Ages	368.2 (233.7 to 521.3)	31.0% (19.6 to 43.9%)	28.0 (17.8 to 39.7)
Chronic obstructive pulmonary disease	China	2010	All Ages	287.0 (181.7 to 405.9)	31.1% (19.7 to 43.9%)	21.3 (13.5 to 30.1)
Chronic obstructive pulmonary disease	China	2015	All Ages	281.7 (176.6 to 400.6)	30.9% (19.5 to 43.6%)	20.4 (12.8 to 29.0)
Chronic obstructive pulmonary disease	China	1990	Age-standardized		28.8% (17.8 to 40.7%)	70.2 (43.4 to 99.6)
Chronic obstructive pulmonary disease	China	1995	Age-standardized		29.1% (18.0 to 41.2%)	64.4 (40.4 to 91.5)
Chronic obstructive pulmonary disease	China	2000	Age-standardized		29.6% (18.4 to 41.9%)	53.2 (33.0 to 75.9)
Chronic obstructive pulmonary disease	China	2005	Age-standardized		31.1% (19.6 to 43.9%)	42.5 (27.0 to 60.2)
Chronic obstructive pulmonary disease	China	2010	Age-standardized		31.2% (19.8 to 43.9%)	28.1 (17.8 to 39.8)
Chronic obstructive pulmonary disease	China	2015	Age-standardized		30.9% (19.5 to 43.6%)	23.2 (14.5 to 33.0)
All causes	North Korea	1990	All Ages	9.2 (6.7 to 12.4)	7.6% (6.4 to 9.0%)	45.8 (33.2 to 61.2)
All causes	North Korea	1995	All Ages	11.0 (8.1 to 14.4)	5.2% (3.5 to 7.6%)	50.5 (37.3 to 66.2)
All causes	North Korea	2000	All Ages	13.1 (10.0 to 17.1)	5.6% (4.0 to 7.9%)	57.7 (43.8 to 75.3)
All causes	North Korea	2005	All Ages	15.1 (11.8 to 18.9)	8.6% (7.2 to 10.3%)	63.6 (49.6 to 79.6)
All causes	North Korea	2010	All Ages	16.5 (12.8 to 20.3)	8.8% (7.3 to 10.4%)	67.2 (52.2 to 82.8)
All causes	North Korea	2015	All Ages	18.8 (14.6 to 23.9)	9.3% (7.7 to 11.1%)	74.9 (58.1 to 95.0)
All causes	North Korea	1990	Age-standardized		8.9% (7.1 to 10.8%)	88.9 (64.1 to 117.9)
All causes	North Korea	1995	Age-standardized		6.0% (4.1 to 8.6%)	88.0 (64.7 to 116.2)
All causes	North Korea	2000	Age-standardized		6.2% (4.3 to 8.8%)	91.7 (68.0 to 118.7)
All causes	North Korea	2005	Age-standardized		9.2% (7.5 to 11.1%)	90.8 (70.5 to 114.6)
All causes	North Korea	2010	Age-standardized		9.1% (7.4 to 11.0%)	85.8 (66.0 to 105.6)
All causes	North Korea	2015	Age-standardized		9.5% (7.8 to 11.5%)	85.0 (65.2 to 107.5)
Lower respiratory infections	North Korea	1990	All Ages	1.7 (0.8 to 3.3)	22.1% (14.4 to 31.2%)	8.4 (3.7 to 16.4)
Lower respiratory infections	North Korea	1995	All Ages	1.5 (0.8 to 2.9)	22.1% (14.6 to 31.0%)	7.0 (3.4 to 13.2)
Lower respiratory infections	North Korea	2000	All Ages	1.4 (0.7 to 2.6)	22.2% (15.1 to 31.0%)	6.1 (3.3 to 11.5)
Lower respiratory infections	North Korea	2005	All Ages	1.2 (0.7 to 2.0)	22.7% (15.2 to 31.2%)	5.1 (2.9 to 8.3)
Lower respiratory infections	North Korea	2010	All Ages	1.1 (0.7 to 1.7)	22.5% (15.1 to 31.3%)	4.5 (2.7 to 7.1)
Lower respiratory infections	North Korea	2015	All Ages	1.1 (0.7 to 1.7)	23.7% (16.6 to 32.1%)	4.5 (2.8 to 6.7)
Lower respiratory infections	North Korea	1990	Age-standardized		22.1% (14.4 to 31.2%)	10.7 (5.6 to 19.0)
Lower respiratory infections	North Korea	1995	Age-standardized		22.1% (14.6 to 31.0%)	9.2 (5.0 to 16.4)
Lower respiratory infections	North Korea	2000	Age-standardized		22.2% (15.1 to 31.0%)	8.7 (4.8 to 15.0)
Lower respiratory infections	North Korea	2005	Age-standardized		22.7% (15.2 to 31.2%)	7.5 (4.4 to 12.0)
Lower respiratory infections	North Korea	2010	Age-standardized		22.5% (15.1 to 31.3%)	6.4 (3.9 to 10.4)

Cause name	Location	Year	Age group	Number (in thousands)	PAF	Rate (per 100,000)
Lower respiratory infections	North Korea	2015	Age-standardized		23.7% (16.6 to 32.1%)	5.8 (3.6 to 9.0)
Tracheal, bronchus, and lung cancer	North Korea	1990	All Ages	0.7 (0.4 to 1.1)	15.8% (9.3 to 22.8%)	3.5 (1.9 to 5.5)
Tracheal, bronchus, and lung cancer	North Korea	1995	All Ages	0.9 (0.5 to 1.4)	15.7% (9.5 to 22.9%)	4.1 (2.3 to 6.3)
Tracheal, bronchus, and lung cancer	North Korea	2000	All Ages	1.1 (0.6 to 1.6)	15.9% (9.3 to 22.9%)	4.6 (2.6 to 7.1)
Tracheal, bronchus, and lung cancer	North Korea	2005	All Ages	1.2 (0.7 to 1.8)	16.3% (9.8 to 23.2%)	5.2 (3.0 to 7.6)
Tracheal, bronchus, and lung cancer	North Korea	2010	All Ages	1.3 (0.8 to 2.0)	16.1% (9.6 to 23.2%)	5.5 (3.1 to 8.2)
Tracheal, bronchus, and lung cancer	North Korea	2015	All Ages	1.6 (0.9 to 2.4)	17.1% (10.2 to 24.4%)	6.2 (3.6 to 9.5)
Tracheal, bronchus, and lung cancer	North Korea	1990	Age-standardized		15.8% (9.3 to 22.9%)	5.7 (3.2 to 9.0)
Tracheal, bronchus, and lung cancer	North Korea	1995	Age-standardized		15.8% (9.5 to 22.9%)	5.9 (3.4 to 9.1)
Tracheal, bronchus, and lung cancer	North Korea	2000	Age-standardized		15.9% (9.3 to 22.9%)	6.0 (3.4 to 9.1)
Tracheal, bronchus, and lung cancer	North Korea	2005	Age-standardized		16.3% (9.8 to 23.2%)	6.1 (3.5 to 8.9)
Tracheal, bronchus, and lung cancer	North Korea	2010	Age-standardized		16.1% (9.6 to 23.2%)	5.9 (3.3 to 8.9)
Tracheal, bronchus, and lung cancer	North Korea	2015	Age-standardized		17.1% (10.2 to 24.4%)	6.2 (3.6 to 9.5)
Cardiovascular diseases	North Korea	1990	All Ages	4.4 (3.3 to 5.8)	12.5% (10.7 to 14.3%)	21.9 (16.1 to 28.5)
Cardiovascular diseases	North Korea	1995	All Ages	5.5 (4.1 to 7.2)	12.5% (10.7 to 14.5%)	25.2 (18.8 to 33.3)
Cardiovascular diseases	North Korea	2000	All Ages	7.0 (5.3 to 9.2)	13.0% (11.1 to 14.9%)	30.6 (23.3 to 40.5)
Cardiovascular diseases	North Korea	2005	All Ages	8.4 (6.6 to 10.5)	13.2% (11.3 to 15.3%)	35.3 (27.7 to 44.1)
Cardiovascular diseases	North Korea	2010	All Ages	9.4 (7.5 to 11.5)	13.0% (11.1 to 14.9%)	38.1 (30.4 to 47.0)
Cardiovascular diseases	North Korea	2015	All Ages	10.7 (8.4 to 13.5)	13.4% (11.5 to 15.3%)	42.5 (33.5 to 53.8)
Cardiovascular diseases	North Korea	1990	Age-standardized		11.3% (9.3 to 13.2%)	41.9 (30.5 to 54.8)
Cardiovascular diseases	North Korea	1995	Age-standardized		11.3% (9.3 to 13.3%)	42.1 (31.1 to 55.9)
Cardiovascular diseases	North Korea	2000	Age-standardized		11.7% (9.6 to 13.6%)	45.8 (34.8 to 60.6)
Cardiovascular diseases	North Korea	2005	Age-standardized		12.0% (10.1 to 14.0%)	47.5 (37.3 to 59.5)
Cardiovascular diseases	North Korea	2010	Age-standardized		12.0% (10.0 to 14.1%)	46.1 (36.5 to 57.0)
Cardiovascular diseases	North Korea	2015	Age-standardized		12.5% (10.5 to 14.6%)	46.2 (36.1 to 58.3)
Ischemic heart disease	North Korea	1990	All Ages	1.9 (1.3 to 2.5)	18.1% (15.0 to 21.1%)	9.2 (6.6 to 12.2)
Ischemic heart disease	North Korea	1995	All Ages	2.3 (1.7 to 3.0)	17.9% (15.0 to 20.9%)	10.4 (7.6 to 13.8)
Ischemic heart disease	North Korea	2000	All Ages	2.9 (2.1 to 3.8)	18.0% (15.0 to 21.2%)	12.6 (9.3 to 16.5)
Ischemic heart disease	North Korea	2005	All Ages	3.5 (2.7 to 4.5)	17.9% (14.7 to 21.1%)	14.9 (11.3 to 18.8)
Ischemic heart disease	North Korea	2010	All Ages	4.0 (3.1 to 5.1)	17.5% (14.5 to 20.7%)	16.5 (12.6 to 20.7)
Ischemic heart disease	North Korea	2015	All Ages	4.7 (3.5 to 6.1)	17.6% (14.8 to 20.9%)	18.6 (13.9 to 24.3)
Ischemic heart disease	North Korea	1990	Age-standardized		15.9% (12.3 to 19.4%)	17.6 (12.4 to 23.5)
Ischemic heart disease	North Korea	1995	Age-standardized		15.8% (12.4 to 19.2%)	17.6 (12.2 to 23.5)
Ischemic heart disease	North Korea	2000	Age-standardized		15.9% (12.6 to 19.3%)	19.2 (13.7 to 25.6)
Ischemic heart disease	North Korea	2005	Age-standardized		16.1% (12.6 to 19.8%)	20.5 (15.0 to 26.9)
Ischemic heart disease	North Korea	2010	Age-standardized		16.0% (12.6 to 19.5%)	20.5 (15.4 to 26.3)

Cause name	Location	Year	Age group	Number (in thousands)	PAF	Rate (per 100,000)
Ischemic heart disease	North Korea	2015	Age-standardized		16.5% (13.1 to 20.1%)	20.7 (14.9 to 27.7)
Ischemic stroke	North Korea	1990	All Ages	0.7 (0.5 to 1.1)	13.1% (10.3 to 16.2%)	3.6 (2.3 to 5.2)
Ischemic stroke	North Korea	1995	All Ages	0.9 (0.6 to 1.3)	13.1% (10.1 to 16.1%)	4.3 (2.8 to 6.2)
Ischemic stroke	North Korea	2000	All Ages	1.2 (0.8 to 1.8)	13.2% (10.3 to 16.2%)	5.5 (3.6 to 7.9)
Ischemic stroke	North Korea	2005	All Ages	1.6 (1.1 to 2.2)	13.2% (10.2 to 16.4%)	6.6 (4.4 to 9.4)
Ischemic stroke	North Korea	2010	All Ages	1.8 (1.2 to 2.5)	13.0% (9.9 to 16.1%)	7.2 (4.7 to 10.0)
Ischemic stroke	North Korea	2015	All Ages	2.0 (1.3 to 3.0)	13.2% (10.1 to 16.3%)	8.1 (5.3 to 11.8)
Ischemic stroke	North Korea	1990	Age-standardized		11.9% (8.7 to 15.2%)	7.8 (4.9 to 11.4)
Ischemic stroke	North Korea	1995	Age-standardized		11.9% (8.7 to 15.3%)	8.0 (5.0 to 12.0)
Ischemic stroke	North Korea	2000	Age-standardized		12.0% (8.8 to 15.3%)	9.1 (5.7 to 13.3)
Ischemic stroke	North Korea	2005	Age-standardized		12.2% (9.0 to 15.4%)	9.5 (6.1 to 13.8)
Ischemic stroke	North Korea	2010	Age-standardized		12.1% (8.9 to 15.4%)	9.2 (6.0 to 13.2)
Ischemic stroke	North Korea	2015	Age-standardized		12.6% (9.3 to 15.7%)	9.3 (5.9 to 13.6)
Hemorrhagic stroke	North Korea	1990	All Ages	1.9 (1.3 to 2.5)	14.2% (11.5 to 17.0%)	9.2 (6.5 to 12.5)
Hemorrhagic stroke	North Korea	1995	All Ages	2.3 (1.6 to 3.1)	14.2% (11.4 to 17.1%)	10.5 (7.3 to 14.4)
Hemorrhagic stroke	North Korea	2000	All Ages	2.9 (2.0 to 4.0)	14.4% (11.5 to 17.4%)	12.5 (8.8 to 17.4)
Hemorrhagic stroke	North Korea	2005	All Ages	3.3 (2.4 to 4.3)	14.4% (11.5 to 17.4%)	13.9 (10.0 to 18.1)
Hemorrhagic stroke	North Korea	2010	All Ages	3.5 (2.6 to 4.6)	14.2% (11.3 to 17.1%)	14.4 (10.5 to 18.9)
Hemorrhagic stroke	North Korea	2015	All Ages	4.0 (2.8 to 5.3)	14.6% (11.9 to 17.6%)	15.8 (11.2 to 21.1)
Hemorrhagic stroke	North Korea	1990	Age-standardized		12.9% (10.0 to 15.7%)	16.4 (11.5 to 22.3)
Hemorrhagic stroke	North Korea	1995	Age-standardized		12.9% (10.1 to 15.9%)	16.5 (11.4 to 22.8)
Hemorrhagic stroke	North Korea	2000	Age-standardized		13.1% (10.2 to 16.0%)	17.5 (12.2 to 24.1)
Hemorrhagic stroke	North Korea	2005	Age-standardized		13.3% (10.5 to 16.1%)	17.5 (12.6 to 23.2)
Hemorrhagic stroke	North Korea	2010	Age-standardized		13.2% (10.4 to 16.2%)	16.4 (11.9 to 21.4)
Hemorrhagic stroke	North Korea	2015	Age-standardized		13.8% (10.9 to 16.6%)	16.2 (11.7 to 21.5)
Chronic obstructive pulmonary disease	North Korea	1990	All Ages	2.4 (1.2 to 4.1)	21.9% (11.9 to 32.4%)	12.0 (6.0 to 20.2)
Chronic obstructive pulmonary disease	North Korea	1995	All Ages	3.1 (1.6 to 5.2)	21.9% (11.9 to 32.7%)	14.2 (7.1 to 24.1)
Chronic obstructive pulmonary disease	North Korea	2000	All Ages	3.7 (1.9 to 6.3)	22.1% (12.2 to 32.8%)	16.4 (8.3 to 27.5)
Chronic obstructive pulmonary disease	North Korea	2005	All Ages	4.3 (2.2 to 7.0)	22.5% (12.7 to 33.2%)	18.0 (9.2 to 29.4)
Chronic obstructive pulmonary disease	North Korea	2010	All Ages	4.7 (2.4 to 7.5)	22.3% (12.2 to 33.2%)	19.1 (9.6 to 30.7)
Chronic obstructive pulmonary disease	North Korea	2015	All Ages	5.5 (2.8 to 8.7)	23.3% (13.1 to 34.3%)	21.7 (11.1 to 34.5)
Chronic obstructive pulmonary disease	North Korea	1990	Age-standardized		22.1% (12.0 to 32.7%)	30.6 (15.6 to 50.7)
Chronic obstructive pulmonary disease	North Korea	1995	Age-standardized		22.0% (11.9 to 32.8%)	30.8 (15.8 to 52.1)
Chronic obstructive pulmonary disease	North Korea	2000	Age-standardized		22.1% (12.3 to 32.9%)	31.3 (15.8 to 52.3)
Chronic obstructive pulmonary disease	North Korea	2005	Age-standardized		22.5% (12.7 to 33.3%)	29.8 (15.2 to 49.5)
Chronic obstructive pulmonary disease	North Korea	2010	Age-standardized		22.3% (12.2 to 33.2%)	27.3 (13.9 to 43.8)

Cause name	Location	Year	Age group	Number (in thousands)	PAF	Rate (per 100,000)
Chronic obstructive pulmonary disease	North Korea	2015	Age-standardized		23.3% (13.1 to 34.3%)	26.8 (13.7 to 42.6)
All causes	Taiwan	1990	All Ages	6.1 (5.3 to 6.9)	5.7% (4.9 to 6.5%)	29.9 (26.1 to 34.0)
All causes	Taiwan	1995	All Ages	6.6 (5.7 to 7.5)	5.5% (4.8 to 6.3%)	31.1 (26.7 to 35.7)
All causes	Taiwan	2000	All Ages	7.1 (6.1 to 8.3)	5.6% (4.8 to 6.5%)	32.3 (27.7 to 37.7)
All causes	Taiwan	2005	All Ages	8.4 (7.2 to 9.6)	6.0% (5.2 to 6.9%)	36.8 (31.7 to 42.3)
All causes	Taiwan	2010	All Ages	9.6 (8.0 to 11.2)	6.4% (5.3 to 7.5%)	41.5 (34.7 to 48.4)
All causes	Taiwan	2015	All Ages	11.0 (8.8 to 13.2)	6.4% (5.3 to 7.6%)	45.2 (36.2 to 54.5)
All causes	Taiwan	1990	Age-standardized		6.1% (5.2 to 7.1%)	52.5 (44.9 to 60.9)
All causes	Taiwan	1995	Age-standardized		5.8% (4.9 to 6.8%)	46.5 (39.2 to 54.1)
All causes	Taiwan	2000	Age-standardized		5.8% (4.9 to 6.7%)	41.4 (35.0 to 48.2)
All causes	Taiwan	2005	Age-standardized		6.1% (5.2 to 7.0%)	38.8 (33.1 to 44.7)
All causes	Taiwan	2010	Age-standardized		6.4% (5.3 to 7.4%)	36.3 (30.3 to 42.3)
All causes	Taiwan	2015	Age-standardized		6.2% (5.2 to 7.4%)	34.3 (27.5 to 41.4)
Lower respiratory infections	Taiwan	1990	All Ages	0.8 (0.5 to 1.2)	21.1% (12.7 to 31.0%)	3.9 (2.3 to 5.8)
Lower respiratory infections	Taiwan	1995	All Ages	0.9 (0.5 to 1.3)	21.1% (12.5 to 30.9%)	4.2 (2.5 to 6.2)
Lower respiratory infections	Taiwan	2000	All Ages	1.1 (0.7 to 1.6)	22.6% (14.4 to 32.0%)	5.0 (3.0 to 7.4)
Lower respiratory infections	Taiwan	2005	All Ages	1.6 (0.8 to 2.4)	23.7% (15.4 to 32.9%)	6.9 (3.7 to 10.4)
Lower respiratory infections	Taiwan	2010	All Ages	2.2 (0.9 to 3.4)	23.1% (14.7 to 32.4%)	9.4 (4.0 to 14.9)
Lower respiratory infections	Taiwan	2015	All Ages	2.6 (1.2 to 4.1)	22.9% (14.3 to 32.0%)	10.6 (4.7 to 16.8)
Lower respiratory infections	Taiwan	1990	Age-standardized		21.1% (12.7 to 31.0%)	8.2 (4.7 to 12.2)
Lower respiratory infections	Taiwan	1995	Age-standardized		21.1% (12.5 to 30.9%)	7.2 (4.2 to 10.7)
Lower respiratory infections	Taiwan	2000	Age-standardized		22.6% (14.4 to 32.0%)	7.2 (4.3 to 10.5)
Lower respiratory infections	Taiwan	2005	Age-standardized		23.7% (15.4 to 32.9%)	7.7 (4.1 to 11.6)
Lower respiratory infections	Taiwan	2010	Age-standardized		23.1% (14.7 to 32.4%)	8.3 (3.5 to 13.1)
Lower respiratory infections	Taiwan	2015	Age-standardized		22.9% (14.3 to 32.0%)	8.0 (3.5 to 12.6)
Tracheal, bronchus, and lung cancer	Taiwan	1990	All Ages	0.6 (0.4 to 0.9)	14.9% (8.6 to 22.0%)	3.0 (1.7 to 4.4)
Tracheal, bronchus, and lung cancer	Taiwan	1995	All Ages	0.8 (0.5 to 1.2)	15.0% (8.6 to 21.8%)	3.9 (2.2 to 5.7)
Tracheal, bronchus, and lung cancer	Taiwan	2000	All Ages	1.1 (0.7 to 1.6)	16.1% (9.6 to 23.4%)	5.1 (3.0 to 7.5)
Tracheal, bronchus, and lung cancer	Taiwan	2005	All Ages	1.4 (0.8 to 2.0)	16.9% (10.1 to 24.3%)	6.0 (3.6 to 8.9)
Tracheal, bronchus, and lung cancer	Taiwan	2010	All Ages	1.5 (0.9 to 2.2)	16.5% (9.7 to 23.6%)	6.3 (3.7 to 9.3)
Tracheal, bronchus, and lung cancer	Taiwan	2015	All Ages	1.6 (0.9 to 2.5)	16.3% (9.6 to 23.4%)	6.7 (3.8 to 10.2)
Tracheal, bronchus, and lung cancer	Taiwan	1990	Age-standardized		14.9% (8.6 to 22.0%)	4.4 (2.5 to 6.4)
Tracheal, bronchus, and lung cancer	Taiwan	1995	Age-standardized		15.0% (8.6 to 21.8%)	5.1 (2.9 to 7.5)
Tracheal, bronchus, and lung cancer	Taiwan	2000	Age-standardized		16.1% (9.6 to 23.4%)	6.0 (3.5 to 8.9)
Tracheal, bronchus, and lung cancer	Taiwan	2005	Age-standardized		16.9% (10.1 to 24.3%)	6.2 (3.7 to 9.1)
Tracheal, bronchus, and lung cancer	Taiwan	2010	Age-standardized		16.4% (9.7 to 23.6%)	5.5 (3.3 to 8.2)

Cause name	Location	Year	Age group	Number (in thousands)	PAF	Rate (per 100,000)
Tracheal, bronchus, and lung cancer	Taiwan	2015	Age-standardized		16.3% (9.6 to 23.4%)	5.2 (3.0 to 7.9)
Cardiovascular diseases	Taiwan	1990	All Ages	4.2 (3.6 to 4.9)	12.7% (10.8 to 14.6%)	20.9 (17.8 to 24.2)
Cardiovascular diseases	Taiwan	1995	All Ages	4.4 (3.7 to 5.2)	12.4% (10.6 to 14.3%)	20.8 (17.5 to 24.3)
Cardiovascular diseases	Taiwan	2000	All Ages	4.3 (3.6 to 5.2)	12.4% (10.5 to 14.3%)	19.8 (16.5 to 23.7)
Cardiovascular diseases	Taiwan	2005	All Ages	4.6 (3.8 to 5.5)	12.5% (10.5 to 14.5%)	20.1 (16.7 to 24.2)
Cardiovascular diseases	Taiwan	2010	All Ages	4.6 (3.7 to 5.6)	12.1% (10.0 to 14.2%)	19.8 (16.2 to 24.3)
Cardiovascular diseases	Taiwan	2015	All Ages	5.1 (3.9 to 6.4)	11.7% (9.4 to 14.2%)	21.0 (16.3 to 26.5)
Cardiovascular diseases	Taiwan	1990	Age-standardized		11.7% (9.7 to 13.7%)	35.7 (29.1 to 42.4)
Cardiovascular diseases	Taiwan	1995	Age-standardized		11.6% (9.6 to 13.7%)	30.5 (24.8 to 36.5)
Cardiovascular diseases	Taiwan	2000	Age-standardized		11.8% (9.7 to 13.9%)	24.8 (19.9 to 30.1)
Cardiovascular diseases	Taiwan	2005	Age-standardized		12.2% (10.1 to 14.3%)	20.8 (17.0 to 25.4)
Cardiovascular diseases	Taiwan	2010	Age-standardized		12.0% (9.9 to 14.2%)	17.2 (13.9 to 21.0)
Cardiovascular diseases	Taiwan	2015	Age-standardized		11.7% (9.5 to 14.1%)	16.0 (12.5 to 20.0)
Ischemic heart disease	Taiwan	1990	All Ages	2.2 (1.8 to 2.7)	16.8% (13.8 to 19.8%)	11.1 (9.0 to 13.4)
Ischemic heart disease	Taiwan	1995	All Ages	2.4 (1.9 to 2.9)	16.5% (13.3 to 19.8%)	11.2 (8.9 to 13.7)
Ischemic heart disease	Taiwan	2000	All Ages	2.3 (1.8 to 2.9)	16.7% (13.3 to 20.3%)	10.7 (8.4 to 13.4)
Ischemic heart disease	Taiwan	2005	All Ages	2.6 (2.0 to 3.3)	16.8% (13.2 to 20.5%)	11.3 (8.8 to 14.4)
Ischemic heart disease	Taiwan	2010	All Ages	2.8 (2.1 to 3.5)	16.1% (12.3 to 20.0%)	11.9 (9.0 to 15.2)
Ischemic heart disease	Taiwan	2015	All Ages	3.1 (2.2 to 4.1)	15.6% (11.5 to 19.8%)	12.8 (9.2 to 17.1)
Ischemic heart disease	Taiwan	1990	Age-standardized		15.4% (11.8 to 19.1%)	19.2 (14.3 to 24.1)
Ischemic heart disease	Taiwan	1995	Age-standardized		15.3% (11.6 to 19.1%)	16.5 (12.4 to 21.1)
Ischemic heart disease	Taiwan	2000	Age-standardized		15.8% (12.0 to 19.7%)	13.5 (10.1 to 17.4)
Ischemic heart disease	Taiwan	2005	Age-standardized		16.3% (12.5 to 20.2%)	11.8 (8.9 to 15.2)
Ischemic heart disease	Taiwan	2010	Age-standardized		16.0% (12.2 to 19.8%)	10.3 (7.8 to 13.2)
Ischemic heart disease	Taiwan	2015	Age-standardized		15.8% (11.8 to 19.7%)	9.7 (7.1 to 12.8)
Ischemic stroke	Taiwan	1990	All Ages	0.8 (0.6 to 1.1)	12.3% (9.2 to 15.3%)	4.0 (3.0 to 5.2)
Ischemic stroke	Taiwan	1995	All Ages	0.8 (0.6 to 1.1)	12.1% (8.9 to 15.2%)	4.0 (2.9 to 5.2)
Ischemic stroke	Taiwan	2000	All Ages	0.8 (0.6 to 1.1)	12.3% (9.0 to 15.6%)	3.8 (2.8 to 5.0)
Ischemic stroke	Taiwan	2005	All Ages	0.8 (0.6 to 1.1)	12.5% (9.0 to 15.9%)	3.8 (2.7 to 5.0)
Ischemic stroke	Taiwan	2010	All Ages	0.8 (0.6 to 1.1)	12.0% (8.4 to 15.8%)	3.5 (2.4 to 4.9)
Ischemic stroke	Taiwan	2015	All Ages	0.9 (0.6 to 1.3)	11.7% (7.9 to 16.0%)	3.8 (2.4 to 5.3)
Ischemic stroke	Taiwan	1990	Age-standardized		11.6% (8.2 to 15.0%)	7.5 (5.1 to 10.0)
Ischemic stroke	Taiwan	1995	Age-standardized		11.5% (8.1 to 15.0%)	6.2 (4.3 to 8.5)
Ischemic stroke	Taiwan	2000	Age-standardized		11.9% (8.3 to 15.7%)	5.1 (3.5 to 6.9)
Ischemic stroke	Taiwan	2005	Age-standardized		12.3% (8.6 to 16.0%)	4.0 (2.8 to 5.5)
Ischemic stroke	Taiwan	2010	Age-standardized		12.0% (8.4 to 15.7%)	3.1 (2.1 to 4.3)

Cause name	Location	Year	Age group	Number (in thousands)	PAF	Rate (per 100,000)
Ischemic stroke	Taiwan	2015	Age-standardized		11.9% (8.2 to 15.8%)	2.9 (1.9 to 4.0)
Hemorrhagic stroke	Taiwan	1990	All Ages	1.2 (0.9 to 1.5)	13.8% (11.0 to 16.7%)	5.8 (4.6 to 7.2)
Hemorrhagic stroke	Taiwan	1995	All Ages	1.2 (1.0 to 1.5)	13.7% (10.8 to 16.5%)	5.6 (4.5 to 6.9)
Hemorrhagic stroke	Taiwan	2000	All Ages	1.2 (0.9 to 1.4)	14.0% (11.1 to 16.8%)	5.2 (4.1 to 6.5)
Hemorrhagic stroke	Taiwan	2005	All Ages	1.1 (0.9 to 1.4)	14.3% (11.4 to 17.2%)	5.0 (3.9 to 6.2)
Hemorrhagic stroke	Taiwan	2010	All Ages	1.0 (0.8 to 1.3)	13.8% (10.8 to 16.9%)	4.4 (3.4 to 5.7)
Hemorrhagic stroke	Taiwan	2015	All Ages	1.1 (0.8 to 1.4)	13.4% (10.1 to 16.6%)	4.5 (3.2 to 5.9)
Hemorrhagic stroke	Taiwan	1990	Age-standardized		12.8% (9.9 to 15.8%)	9.1 (6.9 to 11.5)
Hemorrhagic stroke	Taiwan	1995	Age-standardized		12.8% (9.8 to 15.9%)	7.7 (5.9 to 9.8)
Hemorrhagic stroke	Taiwan	2000	Age-standardized		13.3% (10.2 to 16.4%)	6.2 (4.8 to 7.8)
Hemorrhagic stroke	Taiwan	2005	Age-standardized		13.8% (10.8 to 16.9%)	5.0 (3.9 to 6.3)
Hemorrhagic stroke	Taiwan	2010	Age-standardized		13.6% (10.6 to 16.6%)	3.8 (2.9 to 4.9)
Hemorrhagic stroke	Taiwan	2015	Age-standardized		13.4% (10.3 to 16.6%)	3.4 (2.5 to 4.5)
Chronic obstructive pulmonary disease	Taiwan	1990	All Ages	0.4 (0.2 to 0.6)	21.5% (11.1 to 32.5%)	2.1 (1.1 to 3.2)
Chronic obstructive pulmonary disease	Taiwan	1995	All Ages	0.5 (0.2 to 0.7)	21.5% (11.2 to 32.6%)	2.2 (1.1 to 3.3)
Chronic obstructive pulmonary disease	Taiwan	2000	All Ages	0.5 (0.3 to 0.8)	22.7% (12.0 to 34.0%)	2.4 (1.3 to 3.6)
Chronic obstructive pulmonary disease	Taiwan	2005	All Ages	0.8 (0.5 to 1.3)	23.6% (12.8 to 34.9%)	3.7 (2.0 to 5.6)
Chronic obstructive pulmonary disease	Taiwan	2010	All Ages	1.4 (0.7 to 2.1)	23.1% (12.2 to 34.3%)	6.0 (3.2 to 9.2)
Chronic obstructive pulmonary disease	Taiwan	2015	All Ages	1.7 (0.9 to 2.6)	23.0% (12.4 to 34.1%)	6.8 (3.6 to 10.8)
Chronic obstructive pulmonary disease	Taiwan	1990	Age-standardized		21.5% (11.2 to 32.6%)	4.3 (2.2 to 6.4)
Chronic obstructive pulmonary disease	Taiwan	1995	Age-standardized		21.6% (11.2 to 32.7%)	3.7 (1.8 to 5.6)
Chronic obstructive pulmonary disease	Taiwan	2000	Age-standardized		22.7% (12.0 to 34.1%)	3.4 (1.7 to 5.0)
Chronic obstructive pulmonary disease	Taiwan	2005	Age-standardized		23.6% (12.8 to 34.9%)	4.1 (2.2 to 6.2)
Chronic obstructive pulmonary disease	Taiwan	2010	Age-standardized		23.1% (12.2 to 34.3%)	5.3 (2.8 to 8.1)
Chronic obstructive pulmonary disease	Taiwan	2015	Age-standardized		23.0% (12.4 to 34.1%)	5.1 (2.7 to 8.0)
All causes	Cambodia	1990	All Ages	5.5 (4.2 to 7.1)	5.6% (4.3 to 7.1%)	61.5 (46.3 to 78.8)
All causes	Cambodia	1995	All Ages	6.0 (4.6 to 7.7)	5.7% (4.4 to 7.2%)	56.5 (43.8 to 72.8)
All causes	Cambodia	2000	All Ages	5.9 (4.7 to 7.3)	5.7% (4.6 to 7.1%)	48.4 (38.7 to 60.0)
All causes	Cambodia	2005	All Ages	6.0 (5.0 to 7.2)	6.4% (5.4 to 7.5%)	44.8 (37.4 to 53.8)
All causes	Cambodia	2010	All Ages	5.6 (4.6 to 6.7)	6.1% (5.2 to 7.2%)	38.7 (32.2 to 46.5)
All causes	Cambodia	2015	All Ages	6.1 (4.9 to 7.4)	6.8% (5.9 to 7.9%)	39.2 (31.4 to 47.2)
All causes	Cambodia	1990	Age-standardized		6.7% (5.5 to 8.0%)	112.8 (91.6 to 137.5)
All causes	Cambodia	1995	Age-standardized		6.7% (5.6 to 8.0%)	111.5 (91.8 to 133.9)
All causes	Cambodia	2000	Age-standardized		6.8% (5.7 to 8.0%)	106.3 (88.3 to 127.1)
All causes	Cambodia	2005	Age-standardized		7.5% (6.3 to 8.7%)	101.0 (84.8 to 118.6)
All causes	Cambodia	2010	Age-standardized		7.1% (6.0 to 8.4%)	84.7 (70.4 to 101.1)

Cause name	Location	Year	Age group	Number (in thousands)	PAF	Rate (per 100,000)
All causes	Cambodia	2015	Age-standardized		7.7% (6.6 to 9.0%)	81.7 (66.4 to 98.7)
Lower respiratory infections	Cambodia	1990	All Ages	3.0 (1.7 to 4.5)	19.2% (11.5 to 28.4%)	33.5 (18.6 to 49.9)
Lower respiratory infections	Cambodia	1995	All Ages	3.0 (1.7 to 4.5)	19.3% (11.5 to 28.8%)	27.8 (16.0 to 42.8)
Lower respiratory infections	Cambodia	2000	All Ages	2.5 (1.5 to 3.9)	19.4% (11.6 to 28.8%)	20.4 (12.1 to 31.5)
Lower respiratory infections	Cambodia	2005	All Ages	2.0 (1.3 to 3.0)	21.0% (13.5 to 30.2%)	15.2 (9.6 to 22.3)
Lower respiratory infections	Cambodia	2010	All Ages	1.5 (0.8 to 2.2)	19.1% (11.2 to 28.5%)	10.1 (5.9 to 15.3)
Lower respiratory infections	Cambodia	2015	All Ages	1.4 (0.8 to 2.0)	21.4% (13.4 to 30.5%)	8.8 (5.5 to 13.0)
Lower respiratory infections	Cambodia	1990	Age-standardized		19.2% (11.5 to 28.4%)	33.7 (18.5 to 52.8)
Lower respiratory infections	Cambodia	1995	Age-standardized		19.3% (11.5 to 28.8%)	32.2 (18.3 to 50.8)
Lower respiratory infections	Cambodia	2000	Age-standardized		19.4% (11.6 to 28.8%)	28.5 (16.6 to 44.4)
Lower respiratory infections	Cambodia	2005	Age-standardized		21.0% (13.5 to 30.2%)	24.6 (15.3 to 36.7)
Lower respiratory infections	Cambodia	2010	Age-standardized		19.1% (11.2 to 28.5%)	18.1 (10.2 to 28.6)
Lower respiratory infections	Cambodia	2015	Age-standardized		21.4% (13.4 to 30.5%)	16.9 (10.1 to 25.4)
Tracheal, bronchus, and lung cancer	Cambodia	1990	All Ages	0.1 (0.0 to 0.1)	13.4% (7.6 to 19.9%)	0.9 (0.5 to 1.4)
Tracheal, bronchus, and lung cancer	Cambodia	1995	All Ages	0.1 (0.1 to 0.2)	13.6% (7.7 to 20.2%)	1.0 (0.5 to 1.6)
Tracheal, bronchus, and lung cancer	Cambodia	2000	All Ages	0.1 (0.1 to 0.2)	13.6% (7.7 to 20.1%)	1.0 (0.5 to 1.6)
Tracheal, bronchus, and lung cancer	Cambodia	2005	All Ages	0.2 (0.1 to 0.2)	14.8% (8.6 to 21.3%)	1.2 (0.7 to 1.8)
Tracheal, bronchus, and lung cancer	Cambodia	2010	All Ages	0.2 (0.1 to 0.3)	13.4% (7.5 to 19.8%)	1.2 (0.6 to 1.8)
Tracheal, bronchus, and lung cancer	Cambodia	2015	All Ages	0.2 (0.1 to 0.4)	15.1% (8.9 to 22.2%)	1.4 (0.8 to 2.3)
Tracheal, bronchus, and lung cancer	Cambodia	1990	Age-standardized		13.5% (7.7 to 20.0%)	2.3 (1.2 to 3.6)
Tracheal, bronchus, and lung cancer	Cambodia	1995	Age-standardized		13.6% (7.7 to 20.2%)	2.5 (1.3 to 4.0)
Tracheal, bronchus, and lung cancer	Cambodia	2000	Age-standardized		13.6% (7.7 to 20.2%)	2.6 (1.3 to 4.2)
Tracheal, bronchus, and lung cancer	Cambodia	2005	Age-standardized		14.9% (8.7 to 21.4%)	2.8 (1.5 to 4.4)
Tracheal, bronchus, and lung cancer	Cambodia	2010	Age-standardized		13.5% (7.5 to 19.8%)	2.5 (1.3 to 4.0)
Tracheal, bronchus, and lung cancer	Cambodia	2015	Age-standardized		15.2% (8.9 to 22.2%)	2.8 (1.5 to 4.4)
Cardiovascular diseases	Cambodia	1990	All Ages	2.2 (1.8 to 2.6)	13.0% (11.3 to 14.8%)	24.4 (20.2 to 28.8)
Cardiovascular diseases	Cambodia	1995	All Ages	2.6 (2.2 to 3.1)	13.0% (11.3 to 14.8%)	24.7 (20.6 to 29.0)
Cardiovascular diseases	Cambodia	2000	All Ages	2.9 (2.4 to 3.4)	13.2% (11.3 to 15.0%)	23.8 (19.9 to 28.0)
Cardiovascular diseases	Cambodia	2005	All Ages	3.3 (2.8 to 3.9)	13.8% (11.9 to 15.7%)	24.8 (21.0 to 28.8)
Cardiovascular diseases	Cambodia	2010	All Ages	3.4 (2.8 to 4.0)	13.1% (11.2 to 14.9%)	23.7 (19.7 to 28.1)
Cardiovascular diseases	Cambodia	2015	All Ages	3.9 (3.1 to 4.7)	13.8% (11.9 to 15.7%)	24.8 (19.8 to 30.4)
Cardiovascular diseases	Cambodia	1990	Age-standardized		12.2% (10.3 to 14.1%)	66.9 (55.4 to 79.9)
Cardiovascular diseases	Cambodia	1995	Age-standardized		12.2% (10.4 to 14.0%)	66.3 (54.4 to 78.5)
Cardiovascular diseases	Cambodia	2000	Age-standardized		12.1% (10.2 to 14.0%)	64.1 (52.9 to 76.3)
Cardiovascular diseases	Cambodia	2005	Age-standardized		12.6% (10.7 to 14.5%)	61.9 (51.5 to 73.3)
Cardiovascular diseases	Cambodia	2010	Age-standardized		12.0% (10.0 to 13.9%)	53.8 (43.9 to 64.5)

Cause name	Location	Year	Age group	Number (in thousands)	PAF	Rate (per 100,000)
Cardiovascular diseases	Cambodia	2015	Age-standardized		12.5% (10.5 to 14.6%)	51.7 (41.1 to 63.4)
Ischemic heart disease	Cambodia	1990	All Ages	1.4 (1.1 to 1.7)	17.8% (14.8 to 20.9%)	15.3 (12.2 to 18.6)
Ischemic heart disease	Cambodia	1995	All Ages	1.6 (1.3 to 2.0)	17.7% (14.8 to 20.8%)	15.5 (12.5 to 18.6)
Ischemic heart disease	Cambodia	2000	All Ages	1.8 (1.4 to 2.2)	17.7% (14.8 to 20.9%)	14.6 (11.8 to 17.7)
Ischemic heart disease	Cambodia	2005	All Ages	2.0 (1.7 to 2.4)	18.1% (15.1 to 21.2%)	15.1 (12.4 to 18.2)
Ischemic heart disease	Cambodia	2010	All Ages	2.1 (1.7 to 2.6)	17.1% (14.3 to 20.1%)	14.7 (11.8 to 17.8)
Ischemic heart disease	Cambodia	2015	All Ages	2.4 (1.9 to 3.0)	17.7% (14.6 to 20.8%)	15.4 (11.9 to 19.2)
Ischemic heart disease	Cambodia	1990	Age-standardized		15.8% (12.8 to 18.8%)	41.6 (32.6 to 51.9)
Ischemic heart disease	Cambodia	1995	Age-standardized		15.7% (12.7 to 18.8%)	41.3 (32.6 to 51.0)
Ischemic heart disease	Cambodia	2000	Age-standardized		15.6% (12.6 to 18.7%)	39.2 (30.7 to 48.5)
Ischemic heart disease	Cambodia	2005	Age-standardized		16.1% (12.9 to 19.3%)	37.8 (29.5 to 45.8)
Ischemic heart disease	Cambodia	2010	Age-standardized		15.2% (12.1 to 18.4%)	33.2 (25.7 to 41.1)
Ischemic heart disease	Cambodia	2015	Age-standardized		15.8% (12.7 to 19.1%)	31.8 (24.1 to 40.3)
Ischemic stroke	Cambodia	1990	All Ages	0.2 (0.1 to 0.3)	12.0% (9.0 to 15.0%)	2.2 (1.5 to 3.1)
Ischemic stroke	Cambodia	1995	All Ages	0.2 (0.2 to 0.3)	12.0% (9.0 to 15.0%)	2.3 (1.5 to 3.2)
Ischemic stroke	Cambodia	2000	All Ages	0.3 (0.2 to 0.4)	12.0% (9.0 to 15.0%)	2.4 (1.6 to 3.2)
Ischemic stroke	Cambodia	2005	All Ages	0.3 (0.3 to 0.5)	12.4% (9.4 to 15.3%)	2.6 (1.8 to 3.3)
Ischemic stroke	Cambodia	2010	All Ages	0.4 (0.3 to 0.5)	11.6% (8.7 to 14.5%)	2.5 (1.8 to 3.3)
Ischemic stroke	Cambodia	2015	All Ages	0.4 (0.3 to 0.6)	12.2% (9.2 to 15.2%)	2.7 (1.9 to 3.6)
Ischemic stroke	Cambodia	1990	Age-standardized		10.9% (7.7 to 14.3%)	8.0 (5.1 to 11.7)
Ischemic stroke	Cambodia	1995	Age-standardized		10.9% (7.7 to 14.3%)	7.9 (5.1 to 11.4)
Ischemic stroke	Cambodia	2000	Age-standardized		10.9% (7.7 to 14.3%)	8.2 (5.3 to 11.4)
Ischemic stroke	Cambodia	2005	Age-standardized		11.4% (8.2 to 14.9%)	8.2 (5.5 to 11.2)
Ischemic stroke	Cambodia	2010	Age-standardized		10.7% (7.5 to 14.1%)	7.2 (4.8 to 9.8)
Ischemic stroke	Cambodia	2015	Age-standardized		11.3% (8.1 to 14.7%)	7.0 (4.6 to 9.6)
Hemorrhagic stroke	Cambodia	1990	All Ages	0.6 (0.5 to 0.8)	13.2% (10.4 to 16.1%)	6.9 (5.3 to 8.7)
Hemorrhagic stroke	Cambodia	1995	All Ages	0.7 (0.6 to 0.9)	13.3% (10.5 to 16.0%)	6.9 (5.4 to 8.8)
Hemorrhagic stroke	Cambodia	2000	All Ages	0.8 (0.7 to 1.1)	13.5% (10.8 to 16.2%)	6.8 (5.3 to 8.6)
Hemorrhagic stroke	Cambodia	2005	All Ages	0.9 (0.7 to 1.2)	14.2% (11.4 to 17.1%)	7.1 (5.5 to 8.8)
Hemorrhagic stroke	Cambodia	2010	All Ages	0.9 (0.7 to 1.2)	13.4% (10.7 to 16.2%)	6.5 (5.0 to 8.3)
Hemorrhagic stroke	Cambodia	2015	All Ages	1.1 (0.8 to 1.3)	14.1% (11.4 to 17.0%)	6.7 (5.1 to 8.6)
Hemorrhagic stroke	Cambodia	1990	Age-standardized		12.5% (9.8 to 15.3%)	17.3 (13.0 to 22.4)
Hemorrhagic stroke	Cambodia	1995	Age-standardized		12.5% (9.8 to 15.4%)	17.0 (12.8 to 22.1)
Hemorrhagic stroke	Cambodia	2000	Age-standardized		12.5% (9.8 to 15.3%)	16.7 (12.6 to 21.3)
Hemorrhagic stroke	Cambodia	2005	Age-standardized		13.0% (10.3 to 15.9%)	16.0 (12.3 to 20.5)
Hemorrhagic stroke	Cambodia	2010	Age-standardized		12.2% (9.3 to 14.9%)	13.4 (10.0 to 17.3)

Cause name	Location	Year	Age group	Number (in thousands)	PAF	Rate (per 100,000)
Hemorrhagic stroke	Cambodia	2015	Age-standardized		12.9% (10.1 to 15.8%)	12.9 (9.5 to 16.7)
Chronic obstructive pulmonary disease	Cambodia	1990	All Ages	0.3 (0.1 to 0.5)	14.0% (6.1 to 23.9%)	2.8 (1.1 to 5.2)
Chronic obstructive pulmonary disease	Cambodia	1995	All Ages	0.3 (0.1 to 0.6)	15.4% (7.1 to 25.2%)	3.1 (1.2 to 5.4)
Chronic obstructive pulmonary disease	Cambodia	2000	All Ages	0.4 (0.2 to 0.7)	16.8% (8.2 to 26.5%)	3.2 (1.4 to 5.5)
Chronic obstructive pulmonary disease	Cambodia	2005	All Ages	0.5 (0.3 to 0.8)	19.1% (10.1 to 29.1%)	3.7 (1.9 to 5.9)
Chronic obstructive pulmonary disease	Cambodia	2010	All Ages	0.5 (0.3 to 0.9)	18.5% (9.6 to 28.7%)	3.6 (1.8 to 6.0)
Chronic obstructive pulmonary disease	Cambodia	2015	All Ages	0.6 (0.3 to 1.0)	20.7% (11.0 to 31.6%)	4.1 (2.1 to 6.6)
Chronic obstructive pulmonary disease	Cambodia	1990	Age-standardized		18.7% (9.3 to 29.0%)	10.0 (3.7 to 18.9)
Chronic obstructive pulmonary disease	Cambodia	1995	Age-standardized		18.9% (9.6 to 29.2%)	10.5 (4.0 to 19.0)
Chronic obstructive pulmonary disease	Cambodia	2000	Age-standardized		19.2% (9.9 to 29.7%)	11.1 (4.9 to 19.2)
Chronic obstructive pulmonary disease	Cambodia	2005	Age-standardized		20.7% (11.1 to 30.9%)	11.7 (5.9 to 19.0)
Chronic obstructive pulmonary disease	Cambodia	2010	Age-standardized		19.4% (10.0 to 29.9%)	10.3 (5.2 to 16.9)
Chronic obstructive pulmonary disease	Cambodia	2015	Age-standardized		21.2% (11.2 to 32.2%)	10.3 (5.1 to 16.9)
All causes	Indonesia	1990	All Ages	53.2 (41.7 to 66.9)	3.9% (3.1 to 4.9%)	29.3 (23.0 to 36.9)
All causes	Indonesia	1995	All Ages	57.3 (46.6 to 70.1)	4.3% (3.5 to 5.2%)	29.1 (23.7 to 35.7)
All causes	Indonesia	2000	All Ages	64.3 (53.6 to 76.1)	4.6% (3.9 to 5.4%)	30.4 (25.3 to 36.0)
All causes	Indonesia	2005	All Ages	71.1 (59.7 to 82.9)	4.9% (4.2 to 5.7%)	31.4 (26.3 to 36.6)
All causes	Indonesia	2010	All Ages	71.4 (58.5 to 85.5)	4.7% (4.0 to 5.5%)	29.4 (24.1 to 35.2)
All causes	Indonesia	2015	All Ages	78.6 (62.0 to 96.7)	5.0% (4.3 to 5.8%)	30.5 (24.1 to 37.5)
All causes	Indonesia	1990	Age-standardized		4.6% (3.7 to 5.5%)	57.9 (46.6 to 69.7)
All causes	Indonesia	1995	Age-standardized		4.9% (4.0 to 5.8%)	58.0 (47.4 to 69.7)
All causes	Indonesia	2000	Age-standardized		5.0% (4.2 to 6.0%)	57.4 (47.4 to 68.2)
All causes	Indonesia	2005	Age-standardized		5.3% (4.4 to 6.2%)	57.3 (47.5 to 68.6)
All causes	Indonesia	2010	Age-standardized		5.0% (4.1 to 5.9%)	51.4 (41.4 to 62.3)
All causes	Indonesia	2015	Age-standardized		5.1% (4.3 to 6.1%)	49.9 (38.5 to 61.6)
Lower respiratory infections	Indonesia	1990	All Ages	13.4 (4.8 to 25.1)	14.2% (6.1 to 24.6%)	7.4 (2.7 to 13.8)
Lower respiratory infections	Indonesia	1995	All Ages	10.5 (4.1 to 19.9)	14.2% (6.1 to 24.7%)	5.3 (2.1 to 10.1)
Lower respiratory infections	Indonesia	2000	All Ages	8.6 (3.3 to 16.2)	14.1% (6.0 to 24.6%)	4.1 (1.6 to 7.7)
Lower respiratory infections	Indonesia	2005	All Ages	7.9 (3.3 to 14.1)	14.6% (6.3 to 25.0%)	3.5 (1.5 to 6.2)
Lower respiratory infections	Indonesia	2010	All Ages	6.2 (2.3 to 11.7)	12.6% (4.7 to 23.2%)	2.5 (0.9 to 4.8)
Lower respiratory infections	Indonesia	2015	All Ages	5.5 (2.2 to 10.1)	13.2% (5.3 to 23.8%)	2.1 (0.9 to 3.9)
Lower respiratory infections	Indonesia	1990	Age-standardized		14.2% (6.1 to 24.6%)	7.5 (3.0 to 13.6)
Lower respiratory infections	Indonesia	1995	Age-standardized		14.2% (6.1 to 24.7%)	6.3 (2.5 to 11.5)
Lower respiratory infections	Indonesia	2000	Age-standardized		14.1% (6.0 to 24.6%)	5.3 (2.2 to 9.9)
Lower respiratory infections	Indonesia	2005	Age-standardized		14.6% (6.3 to 25.0%)	4.8 (2.0 to 8.5)
Lower respiratory infections	Indonesia	2010	Age-standardized		12.6% (4.7 to 23.2%)	3.7 (1.3 to 6.9)

Cause name	Location	Year	Age group	Number (in thousands)	PAF	Rate (per 100,000)
Lower respiratory infections	Indonesia	2015	Age-standardized		13.2% (5.3 to 23.8%)	3.4 (1.3 to 6.3)
Tracheal, bronchus, and lung cancer	Indonesia	1990	All Ages	0.4 (0.2 to 0.6)	9.7% (5.0 to 15.4%)	0.2 (0.1 to 0.3)
Tracheal, bronchus, and lung cancer	Indonesia	1995	All Ages	0.5 (0.3 to 0.8)	9.7% (5.0 to 15.5%)	0.3 (0.1 to 0.4)
Tracheal, bronchus, and lung cancer	Indonesia	2000	All Ages	0.6 (0.3 to 1.0)	9.7% (5.0 to 15.4%)	0.3 (0.1 to 0.5)
Tracheal, bronchus, and lung cancer	Indonesia	2005	All Ages	0.7 (0.4 to 1.1)	10.0% (5.2 to 15.8%)	0.3 (0.2 to 0.5)
Tracheal, bronchus, and lung cancer	Indonesia	2010	All Ages	0.7 (0.4 to 1.2)	8.6% (4.3 to 14.0%)	0.3 (0.1 to 0.5)
Tracheal, bronchus, and lung cancer	Indonesia	2015	All Ages	0.9 (0.5 to 1.5)	9.1% (4.6 to 14.6%)	0.4 (0.2 to 0.6)
Tracheal, bronchus, and lung cancer	Indonesia	1990	Age-standardized		9.8% (5.1 to 15.6%)	0.5 (0.2 to 0.7)
Tracheal, bronchus, and lung cancer	Indonesia	1995	Age-standardized		9.8% (5.1 to 15.6%)	0.5 (0.2 to 0.8)
Tracheal, bronchus, and lung cancer	Indonesia	2000	Age-standardized		9.7% (5.0 to 15.5%)	0.5 (0.3 to 0.8)
Tracheal, bronchus, and lung cancer	Indonesia	2005	Age-standardized		10.1% (5.2 to 15.9%)	0.5 (0.3 to 0.9)
Tracheal, bronchus, and lung cancer	Indonesia	2010	Age-standardized		8.6% (4.3 to 14.0%)	0.5 (0.2 to 0.8)
Tracheal, bronchus, and lung cancer	Indonesia	2015	Age-standardized		9.1% (4.6 to 14.7%)	0.5 (0.3 to 0.9)
Cardiovascular diseases	Indonesia	1990	All Ages	33.8 (27.7 to 39.9)	11.7% (9.9 to 13.4%)	18.7 (15.3 to 22.0)
Cardiovascular diseases	Indonesia	1995	All Ages	39.7 (33.0 to 46.8)	11.7% (9.9 to 13.4%)	20.2 (16.8 to 23.8)
Cardiovascular diseases	Indonesia	2000	All Ages	47.8 (39.6 to 55.9)	11.7% (9.9 to 13.4%)	22.6 (18.7 to 26.4)
Cardiovascular diseases	Indonesia	2005	All Ages	54.5 (45.2 to 63.9)	12.0% (10.2 to 13.7%)	24.0 (20.0 to 28.2)
Cardiovascular diseases	Indonesia	2010	All Ages	56.7 (46.2 to 67.4)	11.2% (9.4 to 12.9%)	23.3 (19.0 to 27.7)
Cardiovascular diseases	Indonesia	2015	All Ages	64.6 (50.5 to 79.8)	11.5% (9.7 to 13.3%)	25.1 (19.6 to 31.0)
Cardiovascular diseases	Indonesia	1990	Age-standardized		10.7% (8.8 to 12.5%)	41.3 (33.3 to 49.8)
Cardiovascular diseases	Indonesia	1995	Age-standardized		10.7% (8.7 to 12.5%)	42.4 (34.7 to 50.3)
Cardiovascular diseases	Indonesia	2000	Age-standardized		10.6% (8.7 to 12.5%)	43.4 (35.5 to 51.3)
Cardiovascular diseases	Indonesia	2005	Age-standardized		10.9% (8.9 to 12.7%)	44.0 (35.4 to 52.8)
Cardiovascular diseases	Indonesia	2010	Age-standardized		10.1% (8.2 to 12.0%)	40.3 (31.8 to 49.2)
Cardiovascular diseases	Indonesia	2015	Age-standardized		10.3% (8.3 to 12.1%)	40.0 (30.6 to 49.6)
Ischemic heart disease	Indonesia	1990	All Ages	18.5 (15.0 to 22.3)	15.6% (13.0 to 18.4%)	10.2 (8.3 to 12.3)
Ischemic heart disease	Indonesia	1995	All Ages	21.8 (17.9 to 26.3)	15.5% (12.9 to 18.2%)	11.1 (9.1 to 13.4)
Ischemic heart disease	Indonesia	2000	All Ages	25.8 (21.2 to 31.0)	15.5% (12.8 to 18.2%)	12.2 (10.0 to 14.6)
Ischemic heart disease	Indonesia	2005	All Ages	29.3 (23.7 to 35.5)	15.8% (13.1 to 18.5%)	12.9 (10.5 to 15.6)
Ischemic heart disease	Indonesia	2010	All Ages	31.2 (24.6 to 38.2)	14.8% (12.1 to 17.5%)	12.9 (10.1 to 15.7)
Ischemic heart disease	Indonesia	2015	All Ages	34.5 (26.0 to 43.6)	15.3% (12.6 to 18.1%)	13.4 (10.1 to 16.9)
Ischemic heart disease	Indonesia	1990	Age-standardized		14.0% (11.1 to 16.8%)	22.5 (17.6 to 27.7)
Ischemic heart disease	Indonesia	1995	Age-standardized		13.9% (10.9 to 16.7%)	23.2 (18.2 to 28.4)
Ischemic heart disease	Indonesia	2000	Age-standardized		13.9% (11.0 to 16.8%)	23.1 (18.4 to 28.4)
Ischemic heart disease	Indonesia	2005	Age-standardized		14.2% (11.3 to 17.0%)	23.2 (18.3 to 28.4)
Ischemic heart disease	Indonesia	2010	Age-standardized		13.3% (10.4 to 15.9%)	21.7 (16.6 to 27.2)

Cause name	Location	Year	Age group	Number (in thousands)	PAF	Rate (per 100,000)
Ischemic heart disease	Indonesia	2015	Age-standardized		13.6% (10.8 to 16.4%)	20.6 (15.2 to 26.2)
Ischemic stroke	Indonesia	1990	All Ages	4.2 (2.8 to 5.9)	10.2% (7.2 to 12.9%)	2.3 (1.5 to 3.2)
Ischemic stroke	Indonesia	1995	All Ages	5.0 (3.3 to 6.8)	10.1% (7.1 to 12.9%)	2.5 (1.7 to 3.4)
Ischemic stroke	Indonesia	2000	All Ages	6.3 (4.3 to 8.4)	10.0% (7.0 to 12.8%)	3.0 (2.0 to 4.0)
Ischemic stroke	Indonesia	2005	All Ages	7.3 (5.0 to 9.8)	10.1% (7.1 to 13.0%)	3.2 (2.2 to 4.3)
Ischemic stroke	Indonesia	2010	All Ages	7.5 (5.0 to 10.2)	9.3% (6.4 to 12.1%)	3.1 (2.0 to 4.2)
Ischemic stroke	Indonesia	2015	All Ages	8.9 (5.8 to 12.2)	9.5% (6.6 to 12.3%)	3.4 (2.2 to 4.7)
Ischemic stroke	Indonesia	1990	Age-standardized		9.4% (6.3 to 12.7%)	6.3 (4.0 to 9.0)
Ischemic stroke	Indonesia	1995	Age-standardized		9.4% (6.2 to 12.7%)	6.4 (4.1 to 9.2)
Ischemic stroke	Indonesia	2000	Age-standardized		9.3% (6.1 to 12.6%)	6.9 (4.4 to 9.6)
Ischemic stroke	Indonesia	2005	Age-standardized		9.4% (6.3 to 12.7%)	7.2 (4.7 to 10.2)
Ischemic stroke	Indonesia	2010	Age-standardized		8.7% (5.6 to 12.1%)	6.5 (4.1 to 9.7)
Ischemic stroke	Indonesia	2015	Age-standardized		8.9% (5.8 to 12.4%)	6.9 (4.2 to 10.1)
Hemorrhagic stroke	Indonesia	1990	All Ages	11.1 (8.3 to 14.2)	11.6% (8.9 to 14.5%)	6.1 (4.6 to 7.8)
Hemorrhagic stroke	Indonesia	1995	All Ages	12.9 (9.6 to 16.6)	11.6% (8.8 to 14.5%)	6.6 (4.9 to 8.4)
Hemorrhagic stroke	Indonesia	2000	All Ages	15.8 (11.9 to 20.0)	11.6% (8.8 to 14.6%)	7.5 (5.6 to 9.5)
Hemorrhagic stroke	Indonesia	2005	All Ages	17.9 (13.3 to 22.7)	11.8% (9.1 to 14.6%)	7.9 (5.9 to 10.0)
Hemorrhagic stroke	Indonesia	2010	All Ages	18.0 (13.1 to 23.1)	10.9% (8.1 to 13.6%)	7.4 (5.4 to 9.5)
Hemorrhagic stroke	Indonesia	2015	All Ages	21.3 (15.2 to 27.9)	11.2% (8.5 to 14.0%)	8.3 (5.9 to 10.8)
Hemorrhagic stroke	Indonesia	1990	Age-standardized		10.7% (8.0 to 13.5%)	12.5 (9.2 to 16.3)
Hemorrhagic stroke	Indonesia	1995	Age-standardized		10.7% (8.0 to 13.4%)	12.8 (9.4 to 16.6)
Hemorrhagic stroke	Indonesia	2000	Age-standardized		10.6% (7.9 to 13.4%)	13.4 (9.9 to 17.5)
Hemorrhagic stroke	Indonesia	2005	Age-standardized		10.8% (8.1 to 13.5%)	13.6 (9.9 to 17.8)
Hemorrhagic stroke	Indonesia	2010	Age-standardized		9.9% (7.2 to 12.7%)	12.1 (8.6 to 15.6)
Hemorrhagic stroke	Indonesia	2015	Age-standardized		10.1% (7.5 to 13.0%)	12.5 (8.7 to 16.6)
Chronic obstructive pulmonary disease	Indonesia	1990	All Ages	5.5 (2.4 to 9.4)	14.6% (6.5 to 24.4%)	3.1 (1.3 to 5.2)
Chronic obstructive pulmonary disease	Indonesia	1995	All Ages	6.6 (2.9 to 11.1)	15.1% (6.6 to 25.2%)	3.4 (1.5 to 5.7)
Chronic obstructive pulmonary disease	Indonesia	2000	All Ages	7.3 (3.1 to 12.3)	15.3% (6.8 to 25.4%)	3.4 (1.5 to 5.8)
Chronic obstructive pulmonary disease	Indonesia	2005	All Ages	7.9 (3.4 to 13.3)	15.8% (7.2 to 26.2%)	3.5 (1.5 to 5.9)
Chronic obstructive pulmonary disease	Indonesia	2010	All Ages	7.9 (3.2 to 13.7)	14.3% (6.1 to 24.3%)	3.2 (1.3 to 5.7)
Chronic obstructive pulmonary disease	Indonesia	2015	All Ages	7.6 (3.1 to 13.2)	14.9% (6.6 to 25.1%)	2.9 (1.2 to 5.1)
Chronic obstructive pulmonary disease	Indonesia	1990	Age-standardized		15.6% (6.9 to 25.9%)	8.6 (3.7 to 14.7)
Chronic obstructive pulmonary disease	Indonesia	1995	Age-standardized		15.7% (6.9 to 26.1%)	8.9 (3.8 to 14.9)
Chronic obstructive pulmonary disease	Indonesia	2000	Age-standardized		15.6% (6.9 to 26.1%)	8.1 (3.6 to 13.8)
Chronic obstructive pulmonary disease	Indonesia	2005	Age-standardized		16.1% (7.3 to 26.6%)	7.9 (3.4 to 13.3)
Chronic obstructive pulmonary disease	Indonesia	2010	Age-standardized		14.4% (6.2 to 24.6%)	6.9 (2.8 to 11.9)

Cause name	Location	Year	Age group	Number (in thousands)	PAF	Rate (per 100,000)
Chronic obstructive pulmonary disease	Indonesia	2015	Age-standardized		15.0% (6.6 to 25.2%)	5.9 (2.4 to 10.1)
All causes	Laos	1990	All Ages	3.7 (2.7 to 5.0)	5.7% (4.3 to 7.8%)	87.3 (64.3 to 119.6)
All causes	Laos	1995	All Ages	3.8 (2.8 to 5.0)	5.8% (4.4 to 7.6%)	78.0 (58.2 to 103.0)
All causes	Laos	2000	All Ages	3.6 (2.8 to 4.6)	6.0% (4.8 to 7.7%)	66.9 (52.6 to 86.5)
All causes	Laos	2005	All Ages	3.4 (2.7 to 4.3)	6.5% (5.4 to 8.1%)	59.3 (47.8 to 74.5)
All causes	Laos	2010	All Ages	3.0 (2.4 to 3.7)	6.5% (5.4 to 7.8%)	48.1 (38.6 to 58.9)
All causes	Laos	2015	All Ages	3.0 (2.5 to 3.7)	7.0% (6.0 to 8.2%)	44.5 (36.0 to 54.4)
All causes	Laos	1990	Age-standardized		6.9% (5.7 to 8.3%)	138.6 (109.0 to 173.4)
All causes	Laos	1995	Age-standardized		7.1% (5.9 to 8.5%)	134.6 (108.3 to 164.9)
All causes	Laos	2000	Age-standardized		7.2% (6.0 to 8.5%)	124.2 (103.1 to 147.9)
All causes	Laos	2005	Age-standardized		7.7% (6.6 to 9.1%)	113.4 (95.5 to 134.7)
All causes	Laos	2010	Age-standardized		7.6% (6.5 to 8.9%)	95.9 (79.5 to 114.6)
All causes	Laos	2015	Age-standardized		8.2% (7.0 to 9.4%)	91.1 (74.6 to 111.1)
Lower respiratory infections	Laos	1990	All Ages	2.1 (1.2 to 3.4)	20.4% (12.7 to 29.8%)	49.2 (28.1 to 79.6)
Lower respiratory infections	Laos	1995	All Ages	2.0 (1.1 to 3.1)	20.6% (12.7 to 30.0%)	40.7 (22.2 to 64.3)
Lower respiratory infections	Laos	2000	All Ages	1.7 (1.0 to 2.7)	20.7% (12.8 to 29.8%)	31.8 (18.8 to 50.2)
Lower respiratory infections	Laos	2005	All Ages	1.5 (1.0 to 2.3)	22.2% (14.6 to 31.2%)	26.1 (16.7 to 39.6)
Lower respiratory infections	Laos	2010	All Ages	1.1 (0.7 to 1.8)	21.2% (13.5 to 30.3%)	18.2 (11.0 to 28.0)
Lower respiratory infections	Laos	2015	All Ages	1.0 (0.6 to 1.5)	23.3% (16.0 to 31.9%)	14.5 (9.0 to 21.7)
Lower respiratory infections	Laos	1990	Age-standardized		20.4% (12.7 to 29.8%)	43.9 (25.1 to 68.5)
Lower respiratory infections	Laos	1995	Age-standardized		20.6% (12.7 to 30.0%)	39.8 (23.1 to 62.1)
Lower respiratory infections	Laos	2000	Age-standardized		20.7% (12.8 to 29.8%)	35.5 (21.4 to 54.4)
Lower respiratory infections	Laos	2005	Age-standardized		22.2% (14.6 to 31.2%)	32.0 (20.5 to 47.5)
Lower respiratory infections	Laos	2010	Age-standardized		21.2% (13.5 to 30.3%)	24.6 (14.8 to 37.0)
Lower respiratory infections	Laos	2015	Age-standardized		23.3% (16.0 to 31.9%)	22.1 (13.8 to 31.8)
Tracheal, bronchus, and lung cancer	Laos	1990	All Ages	0.1 (0.0 to 0.1)	14.3% (8.4 to 21.1%)	1.2 (0.6 to 2.0)
Tracheal, bronchus, and lung cancer	Laos	1995	All Ages	0.1 (0.0 to 0.1)	14.4% (8.3 to 21.2%)	1.3 (0.7 to 2.0)
Tracheal, bronchus, and lung cancer	Laos	2000	All Ages	0.1 (0.0 to 0.1)	14.5% (8.5 to 21.4%)	1.3 (0.7 to 2.1)
Tracheal, bronchus, and lung cancer	Laos	2005	All Ages	0.1 (0.0 to 0.1)	15.7% (9.3 to 22.5%)	1.4 (0.7 to 2.1)
Tracheal, bronchus, and lung cancer	Laos	2010	All Ages	0.1 (0.0 to 0.1)	14.9% (8.8 to 21.5%)	1.3 (0.7 to 2.0)
Tracheal, bronchus, and lung cancer	Laos	2015	All Ages	0.1 (0.1 to 0.2)	16.6% (10.1 to 23.7%)	1.5 (0.8 to 2.4)
Tracheal, bronchus, and lung cancer	Laos	1990	Age-standardized		14.4% (8.4 to 21.2%)	2.8 (1.5 to 4.5)
Tracheal, bronchus, and lung cancer	Laos	1995	Age-standardized		14.5% (8.4 to 21.3%)	3.0 (1.6 to 4.8)
Tracheal, bronchus, and lung cancer	Laos	2000	Age-standardized		14.6% (8.6 to 21.5%)	3.1 (1.7 to 4.9)
Tracheal, bronchus, and lung cancer	Laos	2005	Age-standardized		15.8% (9.4 to 22.6%)	3.2 (1.7 to 4.9)
Tracheal, bronchus, and lung cancer	Laos	2010	Age-standardized		15.0% (8.8 to 21.6%)	3.0 (1.6 to 4.6)

Cause name	Location	Year	Age group	Number (in thousands)	PAF	Rate (per 100,000)
Tracheal, bronchus, and lung cancer	Laos	2015	Age-standardized		16.7% (10.1 to 23.8%)	3.3 (1.8 to 5.1)
Cardiovascular diseases	Laos	1990	All Ages	1.4 (1.1 to 1.8)	13.2% (11.5 to 15.0%)	33.0 (25.8 to 41.9)
Cardiovascular diseases	Laos	1995	All Ages	1.5 (1.3 to 1.9)	13.3% (11.5 to 15.1%)	32.0 (25.9 to 39.4)
Cardiovascular diseases	Laos	2000	All Ages	1.6 (1.3 to 1.9)	13.4% (11.6 to 15.1%)	30.0 (25.1 to 35.6)
Cardiovascular diseases	Laos	2005	All Ages	1.6 (1.3 to 1.9)	13.9% (12.1 to 15.8%)	27.9 (23.4 to 32.7)
Cardiovascular diseases	Laos	2010	All Ages	1.6 (1.3 to 1.8)	13.6% (11.8 to 15.4%)	24.8 (20.8 to 29.1)
Cardiovascular diseases	Laos	2015	All Ages	1.7 (1.3 to 2.1)	14.3% (12.5 to 16.2%)	24.5 (19.6 to 30.7)
Cardiovascular diseases	Laos	1990	Age-standardized		12.8% (10.9 to 14.7%)	80.4 (63.0 to 101.7)
Cardiovascular diseases	Laos	1995	Age-standardized		12.9% (10.9 to 14.8%)	79.7 (63.9 to 97.6)
Cardiovascular diseases	Laos	2000	Age-standardized		12.9% (11.0 to 14.8%)	74.0 (61.2 to 89.0)
Cardiovascular diseases	Laos	2005	Age-standardized		13.3% (11.4 to 15.3%)	66.8 (55.5 to 79.8)
Cardiovascular diseases	Laos	2010	Age-standardized		12.9% (11.0 to 14.9%)	57.9 (47.7 to 69.4)
Cardiovascular diseases	Laos	2015	Age-standardized		13.4% (11.4 to 15.5%)	55.2 (44.0 to 69.1)
Ischemic heart disease	Laos	1990	All Ages	0.9 (0.7 to 1.2)	18.0% (15.0 to 21.0%)	21.0 (15.9 to 27.2)
Ischemic heart disease	Laos	1995	All Ages	1.0 (0.8 to 1.3)	18.0% (15.1 to 21.0%)	20.5 (16.3 to 25.9)
Ischemic heart disease	Laos	2000	All Ages	1.0 (0.8 to 1.2)	17.9% (14.9 to 20.8%)	19.0 (15.2 to 23.3)
Ischemic heart disease	Laos	2005	All Ages	1.0 (0.8 to 1.2)	18.3% (15.5 to 21.4%)	17.6 (14.4 to 21.6)
Ischemic heart disease	Laos	2010	All Ages	1.0 (0.8 to 1.2)	17.8% (14.9 to 20.7%)	15.8 (12.7 to 19.1)
Ischemic heart disease	Laos	2015	All Ages	1.1 (0.8 to 1.4)	18.5% (15.5 to 21.5%)	15.7 (12.2 to 19.9)
Ischemic heart disease	Laos	1990	Age-standardized		16.5% (13.5 to 19.5%)	51.0 (38.2 to 66.2)
Ischemic heart disease	Laos	1995	Age-standardized		16.5% (13.5 to 19.7%)	51.0 (39.6 to 63.9)
Ischemic heart disease	Laos	2000	Age-standardized		16.4% (13.3 to 19.6%)	47.0 (37.1 to 58.4)
Ischemic heart disease	Laos	2005	Age-standardized		16.8% (13.7 to 20.1%)	42.2 (33.3 to 51.9)
Ischemic heart disease	Laos	2010	Age-standardized		16.3% (13.1 to 19.5%)	36.9 (29.1 to 45.4)
Ischemic heart disease	Laos	2015	Age-standardized		16.8% (13.5 to 20.2%)	35.3 (27.1 to 45.1)
Ischemic stroke	Laos	1990	All Ages	0.1 (0.1 to 0.2)	12.4% (9.4 to 15.3%)	3.2 (2.2 to 4.8)
Ischemic stroke	Laos	1995	All Ages	0.1 (0.1 to 0.2)	12.3% (9.3 to 15.2%)	3.1 (2.1 to 4.5)
Ischemic stroke	Laos	2000	All Ages	0.2 (0.1 to 0.2)	12.3% (9.3 to 15.2%)	3.0 (2.1 to 4.1)
Ischemic stroke	Laos	2005	All Ages	0.2 (0.1 to 0.2)	12.7% (9.6 to 15.5%)	2.9 (2.0 to 3.8)
Ischemic stroke	Laos	2010	All Ages	0.2 (0.1 to 0.2)	12.2% (9.2 to 15.2%)	2.6 (1.8 to 3.4)
Ischemic stroke	Laos	2015	All Ages	0.2 (0.1 to 0.2)	12.8% (9.7 to 15.8%)	2.6 (1.8 to 3.4)
Ischemic stroke	Laos	1990	Age-standardized		11.5% (8.3 to 14.7%)	9.4 (6.1 to 14.3)
Ischemic stroke	Laos	1995	Age-standardized		11.5% (8.3 to 14.6%)	9.2 (6.0 to 13.4)
Ischemic stroke	Laos	2000	Age-standardized		11.5% (8.2 to 14.6%)	8.8 (5.9 to 12.5)
Ischemic stroke	Laos	2005	Age-standardized		11.9% (8.5 to 15.3%)	8.3 (5.6 to 11.2)
Ischemic stroke	Laos	2010	Age-standardized		11.5% (8.2 to 14.8%)	7.2 (4.9 to 9.9)

Cause name	Location	Year	Age group	Number (in thousands)	PAF	Rate (per 100,000)
Ischemic stroke	Laos	2015	Age-standardized		12.0% (8.6 to 15.5%)	7.0 (4.6 to 9.6)
Hemorrhagic stroke	Laos	1990	All Ages	0.4 (0.3 to 0.5)	13.4% (10.8 to 16.0%)	8.8 (6.5 to 11.8)
Hemorrhagic stroke	Laos	1995	All Ages	0.4 (0.3 to 0.5)	13.5% (10.9 to 16.2%)	8.4 (6.5 to 11.0)
Hemorrhagic stroke	Laos	2000	All Ages	0.4 (0.3 to 0.5)	13.6% (11.1 to 16.2%)	8.0 (6.2 to 10.1)
Hemorrhagic stroke	Laos	2005	All Ages	0.4 (0.3 to 0.5)	14.2% (11.6 to 16.8%)	7.4 (5.9 to 9.1)
Hemorrhagic stroke	Laos	2010	All Ages	0.4 (0.3 to 0.5)	13.8% (11.2 to 16.5%)	6.4 (5.0 to 8.0)
Hemorrhagic stroke	Laos	2015	All Ages	0.4 (0.3 to 0.6)	14.7% (12.0 to 17.4%)	6.2 (4.7 to 8.2)
Hemorrhagic stroke	Laos	1990	Age-standardized		13.1% (10.5 to 15.9%)	20.1 (14.4 to 27.1)
Hemorrhagic stroke	Laos	1995	Age-standardized		13.2% (10.5 to 16.0%)	19.5 (14.8 to 25.3)
Hemorrhagic stroke	Laos	2000	Age-standardized		13.2% (10.4 to 16.0%)	18.2 (14.1 to 23.4)
Hemorrhagic stroke	Laos	2005	Age-standardized		13.6% (10.9 to 16.4%)	16.3 (12.8 to 20.4)
Hemorrhagic stroke	Laos	2010	Age-standardized		13.1% (10.4 to 15.9%)	13.8 (10.5 to 17.5)
Hemorrhagic stroke	Laos	2015	Age-standardized		13.7% (10.9 to 16.5%)	12.9 (9.5 to 16.8)
Chronic obstructive pulmonary disease	Laos	1990	All Ages	0.2 (0.1 to 0.3)	13.9% (5.9 to 23.6%)	3.8 (1.4 to 7.2)
Chronic obstructive pulmonary disease	Laos	1995	All Ages	0.2 (0.1 to 0.3)	15.2% (7.2 to 24.9%)	4.0 (1.6 to 7.0)
Chronic obstructive pulmonary disease	Laos	2000	All Ages	0.2 (0.1 to 0.4)	16.5% (8.1 to 26.0%)	3.9 (1.5 to 6.6)
Chronic obstructive pulmonary disease	Laos	2005	All Ages	0.2 (0.1 to 0.4)	18.3% (9.6 to 28.0%)	3.9 (1.9 to 6.4)
Chronic obstructive pulmonary disease	Laos	2010	All Ages	0.2 (0.1 to 0.4)	18.5% (9.3 to 28.5%)	3.7 (1.9 to 6.0)
Chronic obstructive pulmonary disease	Laos	2015	All Ages	0.3 (0.1 to 0.4)	20.7% (11.1 to 31.0%)	3.9 (2.0 to 6.2)
Chronic obstructive pulmonary disease	Laos	1990	Age-standardized		19.2% (9.9 to 29.3%)	11.4 (4.1 to 21.6)
Chronic obstructive pulmonary disease	Laos	1995	Age-standardized		19.6% (10.0 to 29.7%)	12.1 (5.0 to 20.9)
Chronic obstructive pulmonary disease	Laos	2000	Age-standardized		19.8% (10.1 to 29.9%)	11.6 (4.8 to 19.5)
Chronic obstructive pulmonary disease	Laos	2005	Age-standardized		21.1% (11.4 to 31.4%)	11.3 (5.4 to 18.8)
Chronic obstructive pulmonary disease	Laos	2010	Age-standardized		20.6% (10.6 to 31.2%)	10.5 (5.2 to 16.8)
Chronic obstructive pulmonary disease	Laos	2015	Age-standardized		22.4% (12.2 to 33.1%)	10.6 (5.5 to 16.9)
All causes	Malaysia	1990	All Ages	4.8 (4.0 to 5.8)	6.0% (4.9 to 7.2%)	26.4 (21.7 to 31.9)
All causes	Malaysia	1995	All Ages	5.5 (4.5 to 6.6)	6.1% (5.0 to 7.4%)	26.6 (21.8 to 32.0)
All causes	Malaysia	2000	All Ages	6.2 (5.1 to 7.5)	6.3% (5.2 to 7.7%)	26.5 (21.6 to 32.2)
All causes	Malaysia	2005	All Ages	8.0 (6.6 to 9.6)	7.3% (6.1 to 8.8%)	31.0 (25.7 to 37.3)
All causes	Malaysia	2010	All Ages	8.2 (6.6 to 10.1)	6.4% (5.2 to 7.9%)	29.1 (23.6 to 35.9)
All causes	Malaysia	2015	All Ages	9.5 (7.6 to 12.0)	6.5% (5.3 to 8.0%)	31.4 (25.0 to 39.7)
All causes	Malaysia	1990	Age-standardized		6.8% (5.5 to 8.3%)	65.5 (53.1 to 79.8)
All causes	Malaysia	1995	Age-standardized		6.9% (5.6 to 8.4%)	65.3 (53.0 to 79.9)
All causes	Malaysia	2000	Age-standardized		6.9% (5.6 to 8.5%)	61.9 (50.0 to 76.2)
All causes	Malaysia	2005	Age-standardized		7.9% (6.4 to 9.5%)	65.6 (53.6 to 79.8)
All causes	Malaysia	2010	Age-standardized		6.8% (5.4 to 8.6%)	55.4 (43.7 to 69.6)

Cause name	Location	Year	Age group	Number (in thousands)	PAF	Rate (per 100,000)
All causes	Malaysia	2015	Age-standardized		6.8% (5.4 to 8.4%)	51.7 (40.1 to 66.3)
Lower respiratory infections	Malaysia	1990	All Ages	1.0 (0.4 to 1.8)	13.4% (5.3 to 24.1%)	5.5 (2.1 to 10.1)
Lower respiratory infections	Malaysia	1995	All Ages	1.1 (0.5 to 2.1)	13.5% (5.5 to 24.2%)	5.5 (2.2 to 10.2)
Lower respiratory infections	Malaysia	2000	All Ages	1.3 (0.5 to 2.5)	13.7% (5.6 to 24.3%)	5.7 (2.3 to 10.5)
Lower respiratory infections	Malaysia	2005	All Ages	2.0 (0.9 to 3.5)	16.5% (8.1 to 26.7%)	7.8 (3.5 to 13.5)
Lower respiratory infections	Malaysia	2010	All Ages	1.9 (0.7 to 3.6)	13.2% (5.1 to 24.0%)	6.8 (2.4 to 13.0)
Lower respiratory infections	Malaysia	2015	All Ages	2.2 (0.8 to 4.2)	13.4% (5.4 to 23.9%)	7.2 (2.7 to 13.9)
Lower respiratory infections	Malaysia	1990	Age-standardized		13.4% (5.3 to 24.1%)	13.6 (5.3 to 25.6)
Lower respiratory infections	Malaysia	1995	Age-standardized		13.5% (5.5 to 24.2%)	13.7 (5.5 to 25.6)
Lower respiratory infections	Malaysia	2000	Age-standardized		13.7% (5.6 to 24.3%)	13.9 (5.5 to 25.6)
Lower respiratory infections	Malaysia	2005	Age-standardized		16.5% (8.1 to 26.7%)	17.8 (8.2 to 30.5)
Lower respiratory infections	Malaysia	2010	Age-standardized		13.2% (5.1 to 24.0%)	14.2 (5.2 to 27.1)
Lower respiratory infections	Malaysia	2015	Age-standardized		13.4% (5.4 to 23.9%)	13.0 (5.0 to 24.3)
Tracheal, bronchus, and lung cancer	Malaysia	1990	All Ages	0.2 (0.1 to 0.3)	9.2% (4.7 to 14.8%)	0.9 (0.5 to 1.5)
Tracheal, bronchus, and lung cancer	Malaysia	1995	All Ages	0.2 (0.1 to 0.3)	9.3% (4.7 to 14.9%)	1.0 (0.5 to 1.5)
Tracheal, bronchus, and lung cancer	Malaysia	2000	All Ages	0.2 (0.1 to 0.4)	9.5% (4.8 to 15.1%)	1.0 (0.5 to 1.6)
Tracheal, bronchus, and lung cancer	Malaysia	2005	All Ages	0.4 (0.2 to 0.5)	11.5% (6.2 to 17.7%)	1.4 (0.7 to 2.1)
Tracheal, bronchus, and lung cancer	Malaysia	2010	All Ages	0.4 (0.2 to 0.6)	9.1% (4.6 to 14.7%)	1.2 (0.6 to 2.0)
Tracheal, bronchus, and lung cancer	Malaysia	2015	All Ages	0.4 (0.2 to 0.7)	9.2% (4.6 to 14.8%)	1.4 (0.7 to 2.4)
Tracheal, bronchus, and lung cancer	Malaysia	1990	Age-standardized		9.3% (4.7 to 14.9%)	2.3 (1.1 to 3.6)
Tracheal, bronchus, and lung cancer	Malaysia	1995	Age-standardized		9.4% (4.7 to 15.0%)	2.3 (1.2 to 3.7)
Tracheal, bronchus, and lung cancer	Malaysia	2000	Age-standardized		9.5% (4.8 to 15.2%)	2.3 (1.2 to 3.6)
Tracheal, bronchus, and lung cancer	Malaysia	2005	Age-standardized		11.5% (6.2 to 17.7%)	2.7 (1.5 to 4.2)
Tracheal, bronchus, and lung cancer	Malaysia	2010	Age-standardized		9.1% (4.6 to 14.7%)	2.2 (1.1 to 3.7)
Tracheal, bronchus, and lung cancer	Malaysia	2015	Age-standardized		9.2% (4.6 to 14.8%)	2.3 (1.1 to 3.7)
Cardiovascular diseases	Malaysia	1990	All Ages	3.2 (2.7 to 3.8)	11.4% (9.6 to 13.2%)	17.8 (14.9 to 20.7)
Cardiovascular diseases	Malaysia	1995	All Ages	3.7 (3.1 to 4.3)	11.4% (9.7 to 13.2%)	17.7 (14.9 to 20.5)
Cardiovascular diseases	Malaysia	2000	All Ages	4.1 (3.4 to 4.8)	11.8% (9.9 to 13.6%)	17.5 (14.7 to 20.3)
Cardiovascular diseases	Malaysia	2005	All Ages	5.0 (4.3 to 5.8)	12.9% (11.0 to 14.7%)	19.5 (16.6 to 22.7)
Cardiovascular diseases	Malaysia	2010	All Ages	5.3 (4.4 to 6.3)	11.7% (9.8 to 13.5%)	18.9 (15.8 to 22.3)
Cardiovascular diseases	Malaysia	2015	All Ages	6.2 (4.9 to 7.4)	11.7% (9.8 to 13.6%)	20.3 (16.3 to 24.6)
Cardiovascular diseases	Malaysia	1990	Age-standardized		10.6% (8.8 to 12.5%)	43.4 (35.3 to 51.4)
Cardiovascular diseases	Malaysia	1995	Age-standardized		10.6% (8.7 to 12.6%)	42.2 (34.2 to 50.1)
Cardiovascular diseases	Malaysia	2000	Age-standardized		10.8% (8.8 to 12.8%)	39.3 (31.9 to 47.0)
Cardiovascular diseases	Malaysia	2005	Age-standardized		11.7% (9.7 to 13.7%)	39.2 (32.2 to 46.9)
Cardiovascular diseases	Malaysia	2010	Age-standardized		10.7% (8.7 to 12.6%)	34.3 (27.6 to 41.4)

Cause name	Location	Year	Age group	Number (in thousands)	PAF	Rate (per 100,000)
Cardiovascular diseases	Malaysia	2015	Age-standardized		10.7% (8.7 to 12.7%)	31.7 (25.0 to 38.9)
Ischemic heart disease	Malaysia	1990	All Ages	2.3 (1.8 to 2.7)	14.6% (11.9 to 17.3%)	12.5 (10.1 to 14.9)
Ischemic heart disease	Malaysia	1995	All Ages	2.6 (2.1 to 3.1)	14.6% (12.0 to 17.4%)	12.6 (10.2 to 15.0)
Ischemic heart disease	Malaysia	2000	All Ages	3.0 (2.4 to 3.6)	14.8% (12.1 to 17.5%)	12.7 (10.3 to 15.2)
Ischemic heart disease	Malaysia	2005	All Ages	3.6 (3.0 to 4.3)	16.0% (13.2 to 18.9%)	14.1 (11.5 to 16.8)
Ischemic heart disease	Malaysia	2010	All Ages	3.9 (3.2 to 4.7)	14.6% (11.9 to 17.4%)	13.9 (11.3 to 16.6)
Ischemic heart disease	Malaysia	2015	All Ages	4.6 (3.6 to 5.6)	14.6% (11.8 to 17.3%)	15.0 (11.7 to 18.6)
Ischemic heart disease	Malaysia	1990	Age-standardized		13.4% (10.4 to 16.3%)	30.4 (23.6 to 37.3)
Ischemic heart disease	Malaysia	1995	Age-standardized		13.3% (10.2 to 16.2%)	30.1 (23.2 to 36.9)
Ischemic heart disease	Malaysia	2000	Age-standardized		13.4% (10.3 to 16.4%)	28.4 (21.8 to 34.6)
Ischemic heart disease	Malaysia	2005	Age-standardized		14.4% (11.3 to 17.5%)	27.8 (21.7 to 34.4)
Ischemic heart disease	Malaysia	2010	Age-standardized		13.2% (10.3 to 16.1%)	24.7 (18.9 to 30.8)
Ischemic heart disease	Malaysia	2015	Age-standardized		13.3% (10.3 to 16.2%)	23.2 (17.6 to 29.3)
Ischemic stroke	Malaysia	1990	All Ages	0.3 (0.2 to 0.4)	9.6% (6.6 to 12.4%)	1.6 (1.1 to 2.1)
Ischemic stroke	Malaysia	1995	All Ages	0.3 (0.2 to 0.4)	9.6% (6.6 to 12.4%)	1.4 (1.0 to 1.9)
Ischemic stroke	Malaysia	2000	All Ages	0.3 (0.2 to 0.4)	9.7% (6.7 to 12.5%)	1.4 (1.0 to 1.9)
Ischemic stroke	Malaysia	2005	All Ages	0.4 (0.3 to 0.6)	10.6% (7.6 to 13.5%)	1.7 (1.2 to 2.2)
Ischemic stroke	Malaysia	2010	All Ages	0.5 (0.3 to 0.6)	9.4% (6.5 to 12.3%)	1.6 (1.1 to 2.1)
Ischemic stroke	Malaysia	2015	All Ages	0.5 (0.4 to 0.7)	9.4% (6.4 to 12.3%)	1.7 (1.1 to 2.3)
Ischemic stroke	Malaysia	1990	Age-standardized		9.0% (5.9 to 12.4%)	4.5 (2.9 to 6.4)
Ischemic stroke	Malaysia	1995	Age-standardized		9.0% (5.8 to 12.4%)	4.1 (2.7 to 5.9)
Ischemic stroke	Malaysia	2000	Age-standardized		9.0% (5.8 to 12.5%)	3.9 (2.4 to 5.5)
Ischemic stroke	Malaysia	2005	Age-standardized		9.9% (6.6 to 13.5%)	4.2 (2.8 to 5.9)
Ischemic stroke	Malaysia	2010	Age-standardized		8.8% (5.5 to 12.5%)	3.6 (2.2 to 5.1)
Ischemic stroke	Malaysia	2015	Age-standardized		8.8% (5.6 to 12.4%)	3.2 (2.0 to 4.7)
Hemorrhagic stroke	Malaysia	1990	All Ages	0.7 (0.5 to 0.9)	10.8% (8.2 to 13.6%)	3.8 (2.8 to 4.9)
Hemorrhagic stroke	Malaysia	1995	All Ages	0.8 (0.6 to 1.0)	10.9% (8.2 to 13.6%)	3.6 (2.7 to 4.6)
Hemorrhagic stroke	Malaysia	2000	All Ages	0.8 (0.6 to 1.0)	11.0% (8.4 to 13.8%)	3.4 (2.6 to 4.3)
Hemorrhagic stroke	Malaysia	2005	All Ages	1.0 (0.7 to 1.2)	12.1% (9.4 to 14.9%)	3.7 (2.8 to 4.7)
Hemorrhagic stroke	Malaysia	2010	All Ages	1.0 (0.7 to 1.2)	10.7% (8.0 to 13.5%)	3.4 (2.6 to 4.4)
Hemorrhagic stroke	Malaysia	2015	All Ages	1.1 (0.8 to 1.4)	10.8% (8.0 to 13.6%)	3.6 (2.7 to 4.7)
Hemorrhagic stroke	Malaysia	1990	Age-standardized		10.1% (7.5 to 12.9%)	8.6 (6.1 to 11.2)
Hemorrhagic stroke	Malaysia	1995	Age-standardized		10.1% (7.4 to 12.9%)	8.0 (5.7 to 10.4)
Hemorrhagic stroke	Malaysia	2000	Age-standardized		10.1% (7.4 to 12.9%)	7.1 (5.2 to 9.2)
Hemorrhagic stroke	Malaysia	2005	Age-standardized		11.0% (8.2 to 14.0%)	7.1 (5.3 to 9.2)
Hemorrhagic stroke	Malaysia	2010	Age-standardized		9.8% (7.0 to 12.7%)	6.0 (4.2 to 7.9)

Cause name	Location	Year	Age group	Number (in thousands)	PAF	Rate (per 100,000)
Hemorrhagic stroke	Malaysia	2015	Age-standardized		9.9% (7.2 to 12.8%)	5.4 (3.9 to 7.2)
Chronic obstructive pulmonary disease	Malaysia	1990	All Ages	0.4 (0.2 to 0.7)	15.1% (6.6 to 25.4%)	2.2 (1.0 to 3.7)
Chronic obstructive pulmonary disease	Malaysia	1995	All Ages	0.5 (0.2 to 0.8)	15.2% (6.6 to 25.4%)	2.4 (1.0 to 4.1)
Chronic obstructive pulmonary disease	Malaysia	2000	All Ages	0.5 (0.2 to 0.9)	15.4% (6.8 to 25.8%)	2.3 (1.0 to 3.8)
Chronic obstructive pulmonary disease	Malaysia	2005	All Ages	0.6 (0.3 to 1.0)	17.8% (8.2 to 28.2%)	2.4 (1.1 to 3.8)
Chronic obstructive pulmonary disease	Malaysia	2010	All Ages	0.6 (0.3 to 1.0)	15.0% (6.5 to 25.5%)	2.2 (0.9 to 3.6)
Chronic obstructive pulmonary disease	Malaysia	2015	All Ages	0.8 (0.3 to 1.3)	15.2% (6.6 to 25.5%)	2.5 (1.1 to 4.2)
Chronic obstructive pulmonary disease	Malaysia	1990	Age-standardized		15.2% (6.6 to 25.7%)	6.3 (2.7 to 10.9)
Chronic obstructive pulmonary disease	Malaysia	1995	Age-standardized		15.3% (6.7 to 25.6%)	7.1 (3.1 to 12.0)
Chronic obstructive pulmonary disease	Malaysia	2000	Age-standardized		15.5% (6.8 to 26.0%)	6.3 (2.8 to 10.5)
Chronic obstructive pulmonary disease	Malaysia	2005	Age-standardized		17.8% (8.3 to 28.4%)	5.9 (2.7 to 9.2)
Chronic obstructive pulmonary disease	Malaysia	2010	Age-standardized		15.1% (6.6 to 25.6%)	4.8 (2.0 to 8.0)
Chronic obstructive pulmonary disease	Malaysia	2015	Age-standardized		15.2% (6.7 to 25.5%)	4.8 (2.0 to 8.1)
All causes	Maldives	1990	All Ages	0.1 (0.1 to 0.1)	4.8% (3.5 to 6.4%)	31.4 (23.1 to 42.0)
All causes	Maldives	1995	All Ages	0.1 (0.1 to 0.1)	5.1% (3.8 to 6.5%)	25.0 (18.7 to 31.8)
All causes	Maldives	2000	All Ages	0.1 (0.0 to 0.1)	5.4% (4.2 to 6.8%)	20.0 (15.4 to 25.1)
All causes	Maldives	2005	All Ages	0.1 (0.0 to 0.1)	6.0% (4.7 to 7.4%)	17.9 (13.8 to 22.1)
All causes	Maldives	2010	All Ages	0.1 (0.0 to 0.1)	6.0% (4.6 to 7.5%)	16.6 (12.6 to 20.8)
All causes	Maldives	2015	All Ages	0.1 (0.1 to 0.1)	6.7% (5.2 to 8.3%)	18.9 (14.1 to 24.6)
All causes	Maldives	1990	Age-standardized		6.8% (5.4 to 8.5%)	80.8 (62.9 to 101.0)
All causes	Maldives	1995	Age-standardized		6.8% (5.2 to 8.4%)	68.1 (51.8 to 84.1)
All causes	Maldives	2000	Age-standardized		6.7% (5.2 to 8.4%)	54.2 (41.5 to 67.9)
All causes	Maldives	2005	Age-standardized		7.2% (5.5 to 9.0%)	44.7 (34.1 to 55.7)
All causes	Maldives	2010	Age-standardized		6.9% (5.3 to 8.7%)	36.7 (27.7 to 46.1)
All causes	Maldives	2015	Age-standardized		7.4% (5.7 to 9.3%)	37.4 (27.6 to 48.8)
Lower respiratory infections	Maldives	1990	All Ages	0.0 (0.0 to 0.0)	22.6% (11.8 to 33.6%)	9.8 (4.5 to 17.0)
Lower respiratory infections	Maldives	1995	All Ages	0.0 (0.0 to 0.0)	22.2% (9.7 to 32.8%)	6.1 (2.6 to 10.0)
Lower respiratory infections	Maldives	2000	All Ages	0.0 (0.0 to 0.0)	22.6% (11.4 to 34.0%)	4.1 (2.0 to 6.2)
Lower respiratory infections	Maldives	2005	All Ages	0.0 (0.0 to 0.0)	23.3% (12.8 to 34.4%)	2.9 (1.6 to 4.3)
Lower respiratory infections	Maldives	2010	All Ages	0.0 (0.0 to 0.0)	19.9% (9.1 to 31.0%)	2.2 (1.0 to 3.5)
Lower respiratory infections	Maldives	2015	All Ages	0.0 (0.0 to 0.0)	22.0% (10.5 to 33.4%)	2.4 (1.1 to 3.7)
Lower respiratory infections	Maldives	1990	Age-standardized		22.6% (11.8 to 33.6%)	12.7 (6.6 to 20.2)
Lower respiratory infections	Maldives	1995	Age-standardized		22.2% (9.7 to 32.8%)	10.2 (4.3 to 16.1)
Lower respiratory infections	Maldives	2000	Age-standardized		22.6% (11.4 to 34.0%)	8.3 (4.1 to 12.9)
Lower respiratory infections	Maldives	2005	Age-standardized		23.3% (12.8 to 34.4%)	6.4 (3.5 to 9.9)
Lower respiratory infections	Maldives	2010	Age-standardized		19.9% (9.1 to 31.0%)	4.5 (2.1 to 7.2)

Cause name	Location	Year	Age group	Number (in thousands)	PAF	Rate (per 100,000)
Lower respiratory infections	Maldives	2015	Age-standardized		22.0% (10.5 to 33.4%)	4.7 (2.1 to 7.3)
Tracheal, bronchus, and lung cancer	Maldives	1990	All Ages	0.0 (0.0 to 0.0)	16.0% (8.3 to 24.9%)	0.5 (0.3 to 0.8)
Tracheal, bronchus, and lung cancer	Maldives	1995	All Ages	0.0 (0.0 to 0.0)	15.8% (8.0 to 24.8%)	0.5 (0.3 to 0.8)
Tracheal, bronchus, and lung cancer	Maldives	2000	All Ages	0.0 (0.0 to 0.0)	16.1% (8.5 to 25.1%)	0.5 (0.3 to 0.8)
Tracheal, bronchus, and lung cancer	Maldives	2005	All Ages	0.0 (0.0 to 0.0)	16.6% (8.6 to 25.7%)	0.5 (0.2 to 0.7)
Tracheal, bronchus, and lung cancer	Maldives	2010	All Ages	0.0 (0.0 to 0.0)	14.0% (7.1 to 22.9%)	0.4 (0.2 to 0.6)
Tracheal, bronchus, and lung cancer	Maldives	2015	All Ages	0.0 (0.0 to 0.0)	15.7% (7.9 to 24.8%)	0.5 (0.2 to 0.8)
Tracheal, bronchus, and lung cancer	Maldives	1990	Age-standardized		16.1% (8.3 to 25.0%)	1.4 (0.7 to 2.2)
Tracheal, bronchus, and lung cancer	Maldives	1995	Age-standardized		15.8% (8.0 to 24.9%)	1.4 (0.7 to 2.3)
Tracheal, bronchus, and lung cancer	Maldives	2000	Age-standardized		16.2% (8.5 to 25.2%)	1.3 (0.7 to 2.1)
Tracheal, bronchus, and lung cancer	Maldives	2005	Age-standardized		16.7% (8.7 to 25.8%)	1.1 (0.5 to 1.7)
Tracheal, bronchus, and lung cancer	Maldives	2010	Age-standardized		14.0% (7.1 to 23.0%)	0.8 (0.4 to 1.2)
Tracheal, bronchus, and lung cancer	Maldives	2015	Age-standardized		15.7% (7.9 to 24.8%)	0.9 (0.4 to 1.4)
Cardiovascular diseases	Maldives	1990	All Ages	0.0 (0.0 to 0.1)	14.2% (11.7 to 17.0%)	16.7 (13.3 to 20.9)
Cardiovascular diseases	Maldives	1995	All Ages	0.0 (0.0 to 0.1)	14.0% (11.4 to 16.8%)	14.6 (11.7 to 18.1)
Cardiovascular diseases	Maldives	2000	All Ages	0.0 (0.0 to 0.0)	13.9% (11.2 to 16.8%)	12.4 (10.0 to 15.1)
Cardiovascular diseases	Maldives	2005	All Ages	0.0 (0.0 to 0.0)	14.0% (11.4 to 17.0%)	11.7 (9.3 to 14.3)
Cardiovascular diseases	Maldives	2010	All Ages	0.0 (0.0 to 0.1)	13.1% (10.4 to 16.2%)	11.4 (9.0 to 14.2)
Cardiovascular diseases	Maldives	2015	All Ages	0.1 (0.0 to 0.1)	13.5% (10.6 to 16.6%)	13.0 (9.8 to 16.8)
Cardiovascular diseases	Maldives	1990	Age-standardized		13.1% (10.5 to 16.1%)	52.3 (41.3 to 65.1)
Cardiovascular diseases	Maldives	1995	Age-standardized		12.9% (10.0 to 15.8%)	44.8 (34.5 to 55.7)
Cardiovascular diseases	Maldives	2000	Age-standardized		12.8% (10.0 to 15.7%)	35.7 (27.8 to 44.2)
Cardiovascular diseases	Maldives	2005	Age-standardized		13.1% (10.2 to 16.2%)	29.5 (22.8 to 36.9)
Cardiovascular diseases	Maldives	2010	Age-standardized		12.4% (9.4 to 15.4%)	25.3 (19.2 to 32.0)
Cardiovascular diseases	Maldives	2015	Age-standardized		12.9% (9.8 to 16.2%)	25.5 (18.8 to 33.6)
Ischemic heart disease	Maldives	1990	All Ages	0.0 (0.0 to 0.0)	19.0% (15.3 to 23.0%)	12.3 (9.7 to 15.6)
Ischemic heart disease	Maldives	1995	All Ages	0.0 (0.0 to 0.0)	18.5% (14.7 to 22.6%)	10.9 (8.5 to 13.7)
Ischemic heart disease	Maldives	2000	All Ages	0.0 (0.0 to 0.0)	18.1% (14.5 to 22.3%)	9.2 (7.2 to 11.4)
Ischemic heart disease	Maldives	2005	All Ages	0.0 (0.0 to 0.0)	17.6% (13.9 to 21.8%)	8.6 (6.7 to 10.7)
Ischemic heart disease	Maldives	2010	All Ages	0.0 (0.0 to 0.0)	15.9% (12.3 to 19.9%)	8.7 (6.7 to 10.9)
Ischemic heart disease	Maldives	2015	All Ages	0.0 (0.0 to 0.1)	16.4% (12.3 to 20.9%)	9.8 (7.1 to 12.8)
Ischemic heart disease	Maldives	1990	Age-standardized		16.5% (12.8 to 20.8%)	38.8 (29.7 to 49.3)
Ischemic heart disease	Maldives	1995	Age-standardized		16.3% (12.2 to 20.5%)	33.2 (24.9 to 42.4)
Ischemic heart disease	Maldives	2000	Age-standardized		16.1% (12.2 to 20.2%)	26.4 (19.6 to 33.4)
Ischemic heart disease	Maldives	2005	Age-standardized		16.1% (11.8 to 20.7%)	21.8 (16.0 to 28.2)
Ischemic heart disease	Maldives	2010	Age-standardized		14.9% (10.8 to 19.0%)	19.2 (13.7 to 24.7)

Cause name	Location	Year	Age group	Number (in thousands)	PAF	Rate (per 100,000)
Ischemic heart disease	Maldives	2015	Age-standardized		15.5% (11.1 to 20.1%)	19.2 (13.5 to 25.5)
Ischemic stroke	Maldives	1990	All Ages	0.0 (0.0 to 0.0)	13.6% (10.1 to 17.7%)	1.3 (0.9 to 1.8)
Ischemic stroke	Maldives	1995	All Ages	0.0 (0.0 to 0.0)	13.3% (9.5 to 17.1%)	1.2 (0.8 to 1.6)
Ischemic stroke	Maldives	2000	All Ages	0.0 (0.0 to 0.0)	13.1% (9.3 to 17.0%)	1.1 (0.8 to 1.4)
Ischemic stroke	Maldives	2005	All Ages	0.0 (0.0 to 0.0)	12.8% (8.8 to 16.6%)	1.0 (0.7 to 1.4)
Ischemic stroke	Maldives	2010	All Ages	0.0 (0.0 to 0.0)	11.3% (7.6 to 15.1%)	1.0 (0.7 to 1.3)
Ischemic stroke	Maldives	2015	All Ages	0.0 (0.0 to 0.0)	11.8% (7.7 to 15.6%)	1.1 (0.7 to 1.6)
Ischemic stroke	Maldives	1990	Age-standardized		12.1% (8.3 to 16.1%)	5.2 (3.5 to 7.3)
Ischemic stroke	Maldives	1995	Age-standardized		11.9% (7.9 to 16.2%)	4.5 (2.9 to 6.2)
Ischemic stroke	Maldives	2000	Age-standardized		11.9% (7.8 to 16.3%)	3.7 (2.4 to 5.0)
Ischemic stroke	Maldives	2005	Age-standardized		12.0% (7.8 to 16.4%)	3.0 (2.0 to 4.1)
Ischemic stroke	Maldives	2010	Age-standardized		10.8% (6.9 to 15.1%)	2.4 (1.5 to 3.4)
Ischemic stroke	Maldives	2015	Age-standardized		11.4% (7.2 to 15.8%)	2.4 (1.5 to 3.5)
Hemorrhagic stroke	Maldives	1990	All Ages	0.0 (0.0 to 0.0)	14.4% (11.0 to 18.5%)	3.0 (2.2 to 4.1)
Hemorrhagic stroke	Maldives	1995	All Ages	0.0 (0.0 to 0.0)	14.2% (10.5 to 18.0%)	2.6 (1.9 to 3.4)
Hemorrhagic stroke	Maldives	2000	All Ages	0.0 (0.0 to 0.0)	14.2% (10.5 to 17.9%)	2.2 (1.5 to 2.9)
Hemorrhagic stroke	Maldives	2005	All Ages	0.0 (0.0 to 0.0)	14.1% (10.4 to 17.7%)	2.0 (1.5 to 2.6)
Hemorrhagic stroke	Maldives	2010	All Ages	0.0 (0.0 to 0.0)	12.7% (9.3 to 16.3%)	1.8 (1.3 to 2.3)
Hemorrhagic stroke	Maldives	2015	All Ages	0.0 (0.0 to 0.0)	13.3% (9.7 to 17.0%)	2.1 (1.5 to 2.8)
Hemorrhagic stroke	Maldives	1990	Age-standardized		13.5% (10.2 to 17.3%)	8.3 (6.0 to 11.2)
Hemorrhagic stroke	Maldives	1995	Age-standardized		13.2% (9.4 to 17.0%)	7.1 (5.0 to 9.6)
Hemorrhagic stroke	Maldives	2000	Age-standardized		13.2% (9.4 to 17.0%)	5.7 (3.9 to 7.6)
Hemorrhagic stroke	Maldives	2005	Age-standardized		13.2% (9.4 to 17.0%)	4.8 (3.4 to 6.3)
Hemorrhagic stroke	Maldives	2010	Age-standardized		12.0% (8.2 to 15.7%)	3.7 (2.6 to 5.0)
Hemorrhagic stroke	Maldives	2015	Age-standardized		12.6% (8.7 to 16.3%)	3.9 (2.6 to 5.3)
Chronic obstructive pulmonary disease	Maldives	1990	All Ages	0.0 (0.0 to 0.0)	18.2% (8.6 to 29.2%)	4.4 (2.0 to 7.0)
Chronic obstructive pulmonary disease	Maldives	1995	All Ages	0.0 (0.0 to 0.0)	19.2% (9.6 to 30.0%)	3.7 (1.9 to 5.7)
Chronic obstructive pulmonary disease	Maldives	2000	All Ages	0.0 (0.0 to 0.0)	20.6% (9.9 to 32.3%)	3.0 (1.4 to 4.6)
Chronic obstructive pulmonary disease	Maldives	2005	All Ages	0.0 (0.0 to 0.0)	22.3% (10.9 to 33.7%)	2.9 (1.4 to 4.4)
Chronic obstructive pulmonary disease	Maldives	2010	All Ages	0.0 (0.0 to 0.0)	20.0% (9.6 to 31.4%)	2.6 (1.2 to 4.0)
Chronic obstructive pulmonary disease	Maldives	2015	All Ages	0.0 (0.0 to 0.0)	21.9% (10.3 to 34.2%)	3.0 (1.4 to 4.9)
Chronic obstructive pulmonary disease	Maldives	1990	Age-standardized		21.9% (10.3 to 34.2%)	14.4 (6.5 to 22.8)
Chronic obstructive pulmonary disease	Maldives	1995	Age-standardized		21.7% (11.0 to 33.6%)	11.6 (5.9 to 18.0)
Chronic obstructive pulmonary disease	Maldives	2000	Age-standardized		22.2% (10.7 to 34.5%)	8.8 (4.1 to 13.8)
Chronic obstructive pulmonary disease	Maldives	2005	Age-standardized		23.0% (11.2 to 35.0%)	7.7 (3.8 to 11.8)
Chronic obstructive pulmonary disease	Maldives	2010	Age-standardized		20.4% (9.7 to 32.1%)	6.1 (2.9 to 9.6)

Cause name	Location	Year	Age group	Number (in thousands)	PAF	Rate (per 100,000)
Chronic obstructive pulmonary disease	Maldives	2015	Age-standardized		22.2% (10.4 to 34.5%)	6.4 (3.0 to 10.3)
All causes	Mauritius	1990	All Ages	0.5 (0.4 to 0.6)	7.2% (6.0 to 8.4%)	44.7 (37.2 to 52.3)
All causes	Mauritius	1995	All Ages	0.5 (0.5 to 0.6)	7.4% (6.2 to 8.6%)	47.6 (40.0 to 55.7)
All causes	Mauritius	2000	All Ages	0.6 (0.5 to 0.7)	7.3% (6.2 to 8.5%)	48.0 (40.2 to 56.2)
All causes	Mauritius	2005	All Ages	0.5 (0.4 to 0.6)	6.1% (5.1 to 7.2%)	42.5 (35.2 to 50.1)
All causes	Mauritius	2010	All Ages	0.4 (0.3 to 0.5)	4.4% (3.6 to 5.2%)	32.1 (26.1 to 38.4)
All causes	Mauritius	2015	All Ages	0.5 (0.4 to 0.5)	4.5% (3.7 to 5.4%)	35.3 (28.7 to 42.3)
All causes	Mauritius	1990	Age-standardized		7.6% (6.3 to 9.0%)	88.0 (72.8 to 104.3)
All causes	Mauritius	1995	Age-standardized		7.7% (6.3 to 9.1%)	83.2 (68.0 to 98.7)
All causes	Mauritius	2000	Age-standardized		7.4% (6.2 to 8.8%)	73.6 (61.5 to 87.5)
All causes	Mauritius	2005	Age-standardized		6.2% (5.0 to 7.3%)	57.9 (47.3 to 68.5)
All causes	Mauritius	2010	Age-standardized		4.4% (3.6 to 5.3%)	37.6 (30.4 to 45.5)
All causes	Mauritius	2015	Age-standardized		4.5% (3.6 to 5.4%)	36.3 (29.0 to 44.1)
Lower respiratory infections	Mauritius	1990	All Ages	0.1 (0.0 to 0.1)	15.1% (6.5 to 25.8%)	4.3 (1.8 to 7.6)
Lower respiratory infections	Mauritius	1995	All Ages	0.1 (0.0 to 0.1)	15.3% (6.5 to 25.6%)	4.4 (1.9 to 7.5)
Lower respiratory infections	Mauritius	2000	All Ages	0.0 (0.0 to 0.1)	15.3% (6.6 to 26.2%)	3.8 (1.7 to 6.5)
Lower respiratory infections	Mauritius	2005	All Ages	0.0 (0.0 to 0.1)	15.7% (6.9 to 26.5%)	3.6 (1.6 to 6.2)
Lower respiratory infections	Mauritius	2010	All Ages	0.0 (0.0 to 0.1)	11.9% (3.8 to 22.8%)	2.7 (0.9 to 5.3)
Lower respiratory infections	Mauritius	2015	All Ages	0.0 (0.0 to 0.1)	12.9% (4.6 to 23.6%)	3.3 (1.2 to 6.2)
Lower respiratory infections	Mauritius	1990	Age-standardized		15.1% (6.5 to 25.8%)	9.4 (4.0 to 16.3)
Lower respiratory infections	Mauritius	1995	Age-standardized		15.3% (6.5 to 25.6%)	8.3 (3.5 to 14.2)
Lower respiratory infections	Mauritius	2000	Age-standardized		15.3% (6.6 to 26.2%)	6.3 (2.8 to 10.7)
Lower respiratory infections	Mauritius	2005	Age-standardized		15.7% (6.9 to 26.5%)	5.3 (2.3 to 9.0)
Lower respiratory infections	Mauritius	2010	Age-standardized		11.9% (3.8 to 22.8%)	3.5 (1.1 to 6.8)
Lower respiratory infections	Mauritius	2015	Age-standardized		12.9% (4.6 to 23.6%)	3.8 (1.4 to 7.0)
Tracheal, bronchus, and lung cancer	Mauritius	1990	All Ages	0.0 (0.0 to 0.0)	10.5% (5.2 to 16.6%)	0.9 (0.4 to 1.4)
Tracheal, bronchus, and lung cancer	Mauritius	1995	All Ages	0.0 (0.0 to 0.0)	10.6% (5.4 to 16.9%)	1.0 (0.5 to 1.5)
Tracheal, bronchus, and lung cancer	Mauritius	2000	All Ages	0.0 (0.0 to 0.0)	10.7% (5.3 to 17.0%)	1.0 (0.5 to 1.6)
Tracheal, bronchus, and lung cancer	Mauritius	2005	All Ages	0.0 (0.0 to 0.0)	11.0% (5.6 to 17.1%)	1.2 (0.6 to 1.8)
Tracheal, bronchus, and lung cancer	Mauritius	2010	All Ages	0.0 (0.0 to 0.0)	8.1% (4.0 to 13.7%)	1.0 (0.5 to 1.6)
Tracheal, bronchus, and lung cancer	Mauritius	2015	All Ages	0.0 (0.0 to 0.0)	8.9% (4.3 to 14.7%)	1.2 (0.6 to 2.1)
Tracheal, bronchus, and lung cancer	Mauritius	1990	Age-standardized		10.5% (5.2 to 16.7%)	1.7 (0.8 to 2.6)
Tracheal, bronchus, and lung cancer	Mauritius	1995	Age-standardized		10.6% (5.4 to 16.9%)	1.6 (0.8 to 2.6)
Tracheal, bronchus, and lung cancer	Mauritius	2000	Age-standardized		10.7% (5.3 to 17.0%)	1.5 (0.7 to 2.4)
Tracheal, bronchus, and lung cancer	Mauritius	2005	Age-standardized		11.0% (5.6 to 17.2%)	1.6 (0.8 to 2.5)
Tracheal, bronchus, and lung cancer	Mauritius	2010	Age-standardized		8.1% (4.0 to 13.7%)	1.1 (0.5 to 1.9)

Cause name	Location	Year	Age group	Number (in thousands)	PAF	Rate (per 100,000)
Tracheal, bronchus, and lung cancer	Mauritius	2015	Age-standardized		8.9% (4.3 to 14.7%)	1.2 (0.6 to 2.0)
Cardiovascular diseases	Mauritius	1990	All Ages	0.4 (0.3 to 0.5)	12.9% (10.7 to 15.2%)	36.4 (30.1 to 42.8)
Cardiovascular diseases	Mauritius	1995	All Ages	0.4 (0.4 to 0.5)	13.0% (11.0 to 15.2%)	39.4 (33.2 to 46.0)
Cardiovascular diseases	Mauritius	2000	All Ages	0.5 (0.4 to 0.6)	12.7% (10.7 to 14.9%)	40.1 (33.6 to 47.2)
Cardiovascular diseases	Mauritius	2005	All Ages	0.4 (0.4 to 0.5)	12.3% (10.1 to 14.4%)	34.9 (28.8 to 41.0)
Cardiovascular diseases	Mauritius	2010	All Ages	0.3 (0.3 to 0.4)	10.4% (8.6 to 12.5%)	26.2 (21.5 to 31.4)
Cardiovascular diseases	Mauritius	2015	All Ages	0.4 (0.3 to 0.4)	10.5% (8.5 to 12.5%)	28.0 (22.5 to 33.6)
Cardiovascular diseases	Mauritius	1990	Age-standardized		11.8% (9.7 to 14.1%)	69.7 (57.4 to 83.2)
Cardiovascular diseases	Mauritius	1995	Age-standardized		12.0% (9.9 to 14.2%)	67.2 (55.6 to 79.6)
Cardiovascular diseases	Mauritius	2000	Age-standardized		11.9% (9.8 to 14.2%)	60.3 (49.4 to 72.3)
Cardiovascular diseases	Mauritius	2005	Age-standardized		11.6% (9.4 to 13.8%)	46.7 (37.9 to 55.5)
Cardiovascular diseases	Mauritius	2010	Age-standardized		9.8% (7.9 to 11.9%)	30.1 (24.2 to 36.4)
Cardiovascular diseases	Mauritius	2015	Age-standardized		10.0% (8.0 to 12.1%)	28.3 (22.4 to 34.7)
Ischemic heart disease	Mauritius	1990	All Ages	0.3 (0.2 to 0.3)	15.9% (13.0 to 19.1%)	26.2 (21.4 to 31.5)
Ischemic heart disease	Mauritius	1995	All Ages	0.3 (0.3 to 0.4)	15.7% (12.9 to 18.7%)	28.5 (23.4 to 34.0)
Ischemic heart disease	Mauritius	2000	All Ages	0.3 (0.3 to 0.4)	15.5% (12.7 to 18.6%)	28.7 (23.2 to 34.5)
Ischemic heart disease	Mauritius	2005	All Ages	0.3 (0.2 to 0.4)	15.3% (12.2 to 18.3%)	25.1 (20.1 to 30.2)
Ischemic heart disease	Mauritius	2010	All Ages	0.2 (0.2 to 0.3)	13.6% (10.8 to 16.3%)	19.0 (15.2 to 23.0)
Ischemic heart disease	Mauritius	2015	All Ages	0.3 (0.2 to 0.3)	13.7% (10.8 to 16.7%)	20.3 (15.6 to 24.8)
Ischemic heart disease	Mauritius	1990	Age-standardized		14.5% (11.5 to 17.6%)	49.4 (39.3 to 60.2)
Ischemic heart disease	Mauritius	1995	Age-standardized		14.5% (11.5 to 17.6%)	47.9 (38.0 to 58.5)
Ischemic heart disease	Mauritius	2000	Age-standardized		14.5% (11.4 to 17.6%)	42.8 (33.7 to 52.4)
Ischemic heart disease	Mauritius	2005	Age-standardized		14.4% (11.2 to 17.5%)	33.3 (25.9 to 40.4)
Ischemic heart disease	Mauritius	2010	Age-standardized		12.8% (9.9 to 15.7%)	21.6 (16.7 to 26.8)
Ischemic heart disease	Mauritius	2015	Age-standardized		13.0% (9.9 to 16.2%)	20.3 (15.2 to 25.5)
Ischemic stroke	Mauritius	1990	All Ages	0.0 (0.0 to 0.0)	10.3% (7.2 to 13.3%)	3.1 (2.1 to 4.1)
Ischemic stroke	Mauritius	1995	All Ages	0.0 (0.0 to 0.1)	10.2% (7.0 to 13.1%)	3.3 (2.3 to 4.3)
Ischemic stroke	Mauritius	2000	All Ages	0.0 (0.0 to 0.1)	10.1% (7.0 to 13.1%)	3.5 (2.4 to 4.6)
Ischemic stroke	Mauritius	2005	All Ages	0.0 (0.0 to 0.1)	9.9% (6.6 to 13.3%)	3.1 (2.0 to 4.2)
Ischemic stroke	Mauritius	2010	All Ages	0.0 (0.0 to 0.0)	8.5% (5.4 to 12.1%)	2.2 (1.4 to 3.2)
Ischemic stroke	Mauritius	2015	All Ages	0.0 (0.0 to 0.0)	8.8% (5.5 to 12.4%)	2.5 (1.5 to 3.5)
Ischemic stroke	Mauritius	1990	Age-standardized		9.7% (6.5 to 13.1%)	7.2 (4.8 to 9.9)
Ischemic stroke	Mauritius	1995	Age-standardized		9.7% (6.4 to 13.1%)	6.7 (4.4 to 9.2)
Ischemic stroke	Mauritius	2000	Age-standardized		9.7% (6.3 to 13.3%)	6.1 (4.0 to 8.4)
Ischemic stroke	Mauritius	2005	Age-standardized		9.6% (6.2 to 13.3%)	4.7 (3.0 to 6.7)
Ischemic stroke	Mauritius	2010	Age-standardized		8.2% (4.9 to 12.1%)	2.9 (1.8 to 4.3)

Cause name	Location	Year	Age group	Number (in thousands)	PAF	Rate (per 100,000)
Ischemic stroke	Mauritius	2015	Age-standardized		8.5% (5.1 to 12.5%)	2.8 (1.7 to 4.1)
Hemorrhagic stroke	Mauritius	1990	All Ages	0.1 (0.1 to 0.1)	11.9% (8.8 to 14.9%)	7.1 (5.2 to 9.0)
Hemorrhagic stroke	Mauritius	1995	All Ages	0.1 (0.1 to 0.1)	11.8% (8.8 to 14.9%)	7.6 (5.7 to 9.6)
Hemorrhagic stroke	Mauritius	2000	All Ages	0.1 (0.1 to 0.1)	11.7% (8.8 to 14.6%)	7.9 (5.9 to 9.9)
Hemorrhagic stroke	Mauritius	2005	All Ages	0.1 (0.1 to 0.1)	11.7% (8.7 to 14.7%)	6.7 (5.0 to 8.6)
Hemorrhagic stroke	Mauritius	2010	All Ages	0.1 (0.0 to 0.1)	10.0% (7.2 to 12.9%)	4.9 (3.6 to 6.5)
Hemorrhagic stroke	Mauritius	2015	All Ages	0.1 (0.1 to 0.1)	10.1% (7.3 to 13.1%)	5.3 (3.8 to 6.8)
Hemorrhagic stroke	Mauritius	1990	Age-standardized		10.9% (8.0 to 13.9%)	13.1 (9.6 to 16.6)
Hemorrhagic stroke	Mauritius	1995	Age-standardized		10.9% (8.0 to 14.0%)	12.6 (9.2 to 16.1)
Hemorrhagic stroke	Mauritius	2000	Age-standardized		10.9% (8.1 to 13.9%)	11.5 (8.4 to 14.7)
Hemorrhagic stroke	Mauritius	2005	Age-standardized		10.9% (7.9 to 14.0%)	8.8 (6.4 to 11.4)
Hemorrhagic stroke	Mauritius	2010	Age-standardized		9.4% (6.6 to 12.5%)	5.5 (3.9 to 7.4)
Hemorrhagic stroke	Mauritius	2015	Age-standardized		9.7% (6.8 to 12.6%)	5.2 (3.7 to 7.0)
Chronic obstructive pulmonary disease	Mauritius	1990	All Ages	0.0 (0.0 to 0.1)	16.4% (7.4 to 27.1%)	3.0 (1.3 to 5.0)
Chronic obstructive pulmonary disease	Mauritius	1995	All Ages	0.0 (0.0 to 0.1)	16.6% (7.5 to 27.2%)	2.9 (1.3 to 4.8)
Chronic obstructive pulmonary disease	Mauritius	2000	All Ages	0.0 (0.0 to 0.1)	16.7% (7.6 to 26.9%)	3.1 (1.4 to 5.0)
Chronic obstructive pulmonary disease	Mauritius	2005	All Ages	0.0 (0.0 to 0.1)	17.1% (7.7 to 28.0%)	2.8 (1.3 to 4.6)
Chronic obstructive pulmonary disease	Mauritius	2010	All Ages	0.0 (0.0 to 0.1)	13.8% (5.7 to 24.0%)	2.2 (0.9 to 3.8)
Chronic obstructive pulmonary disease	Mauritius	2015	All Ages	0.0 (0.0 to 0.1)	14.8% (6.2 to 25.2%)	2.7 (1.1 to 4.6)
Chronic obstructive pulmonary disease	Mauritius	1990	Age-standardized		16.6% (7.5 to 27.4%)	7.2 (3.2 to 11.8)
Chronic obstructive pulmonary disease	Mauritius	1995	Age-standardized		16.7% (7.6 to 27.5%)	6.0 (2.7 to 10.0)
Chronic obstructive pulmonary disease	Mauritius	2000	Age-standardized		16.8% (7.6 to 27.1%)	5.5 (2.5 to 8.9)
Chronic obstructive pulmonary disease	Mauritius	2005	Age-standardized		17.1% (7.7 to 28.1%)	4.3 (2.0 to 7.1)
Chronic obstructive pulmonary disease	Mauritius	2010	Age-standardized		13.8% (5.7 to 24.0%)	2.8 (1.2 to 5.0)
Chronic obstructive pulmonary disease	Mauritius	2015	Age-standardized		14.8% (6.2 to 25.2%)	3.0 (1.3 to 5.1)
All causes	Myanmar	1990	All Ages	31.2 (22.7 to 41.3)	6.9% (5.7 to 8.2%)	74.2 (54.0 to 98.1)
All causes	Myanmar	1995	All Ages	31.3 (22.5 to 41.7)	6.8% (5.7 to 8.1%)	69.9 (50.1 to 93.1)
All causes	Myanmar	2000	All Ages	31.8 (23.0 to 42.5)	6.9% (5.7 to 8.1%)	66.7 (48.2 to 89.1)
All causes	Myanmar	2005	All Ages	31.5 (22.2 to 42.4)	7.1% (6.0 to 8.2%)	63.1 (44.4 to 84.9)
All causes	Myanmar	2010	All Ages	28.9 (19.6 to 40.5)	7.0% (5.9 to 8.0%)	55.8 (37.8 to 78.3)
All causes	Myanmar	2015	All Ages	31.1 (20.3 to 42.8)	7.6% (6.5 to 8.6%)	57.5 (37.6 to 79.2)
All causes	Myanmar	1990	Age-standardized		7.0% (5.9 to 8.2%)	122.4 (85.3 to 167.3)
All causes	Myanmar	1995	Age-standardized		7.1% (6.0 to 8.3%)	119.3 (84.4 to 162.4)
All causes	Myanmar	2000	Age-standardized		7.2% (6.1 to 8.4%)	113.7 (79.7 to 153.8)
All causes	Myanmar	2005	Age-standardized		7.6% (6.4 to 8.8%)	107.4 (74.4 to 145.6)
All causes	Myanmar	2010	Age-standardized		7.5% (6.3 to 8.7%)	94.3 (64.1 to 129.6)

Cause name	Location	Year	Age group	Number (in thousands)	PAF	Rate (per 100,000)
All causes	Myanmar	2015	Age-standardized		8.2% (6.9 to 9.4%)	93.7 (62.3 to 127.2)
Lower respiratory infections	Myanmar	1990	All Ages	15.2 (10.4 to 20.7)	28.9% (21.0 to 36.6%)	36.1 (24.7 to 49.3)
Lower respiratory infections	Myanmar	1995	All Ages	13.4 (9.3 to 18.3)	28.9% (21.1 to 36.6%)	29.8 (20.8 to 40.8)
Lower respiratory infections	Myanmar	2000	All Ages	12.3 (8.4 to 16.7)	29.1% (21.3 to 36.7%)	25.7 (17.7 to 35.1)
Lower respiratory infections	Myanmar	2005	All Ages	10.5 (7.4 to 13.9)	30.5% (22.8 to 37.9%)	21.1 (14.8 to 27.8)
Lower respiratory infections	Myanmar	2010	All Ages	7.4 (4.9 to 10.3)	29.7% (21.9 to 37.4%)	14.3 (9.5 to 19.8)
Lower respiratory infections	Myanmar	2015	All Ages	6.3 (4.0 to 8.9)	32.7% (24.9 to 40.0%)	11.7 (7.4 to 16.5)
Lower respiratory infections	Myanmar	1990	Age-standardized		28.9% (21.0 to 36.6%)	35.1 (24.2 to 48.5)
Lower respiratory infections	Myanmar	1995	Age-standardized		28.9% (21.1 to 36.6%)	31.8 (21.5 to 44.8)
Lower respiratory infections	Myanmar	2000	Age-standardized		29.1% (21.3 to 36.7%)	28.3 (18.7 to 40.9)
Lower respiratory infections	Myanmar	2005	Age-standardized		30.5% (22.8 to 37.9%)	24.5 (16.6 to 34.9)
Lower respiratory infections	Myanmar	2010	Age-standardized		29.7% (21.9 to 37.4%)	18.5 (12.0 to 27.9)
Lower respiratory infections	Myanmar	2015	Age-standardized		32.7% (24.9 to 40.0%)	16.4 (10.1 to 25.3)
Tracheal, bronchus, and lung cancer	Myanmar	1990	All Ages	1.4 (0.7 to 2.5)	20.8% (13.1 to 28.9%)	3.4 (1.7 to 5.9)
Tracheal, bronchus, and lung cancer	Myanmar	1995	All Ages	1.6 (0.8 to 2.6)	20.8% (13.3 to 28.7%)	3.5 (1.8 to 5.7)
Tracheal, bronchus, and lung cancer	Myanmar	2000	All Ages	1.8 (0.9 to 2.9)	20.9% (13.3 to 28.8%)	3.7 (1.9 to 6.1)
Tracheal, bronchus, and lung cancer	Myanmar	2005	All Ages	2.1 (1.1 to 3.5)	22.1% (14.1 to 30.2%)	4.2 (2.2 to 6.9)
Tracheal, bronchus, and lung cancer	Myanmar	2010	All Ages	2.3 (1.2 to 3.8)	21.5% (13.8 to 29.5%)	4.5 (2.3 to 7.3)
Tracheal, bronchus, and lung cancer	Myanmar	2015	All Ages	3.0 (1.6 to 4.9)	24.0% (15.9 to 32.2%)	5.6 (3.0 to 9.0)
Tracheal, bronchus, and lung cancer	Myanmar	1990	Age-standardized		20.9% (13.3 to 29.1%)	6.7 (3.4 to 11.4)
Tracheal, bronchus, and lung cancer	Myanmar	1995	Age-standardized		21.0% (13.4 to 28.9%)	6.6 (3.4 to 10.9)
Tracheal, bronchus, and lung cancer	Myanmar	2000	Age-standardized		21.1% (13.4 to 29.0%)	6.7 (3.6 to 11.1)
Tracheal, bronchus, and lung cancer	Myanmar	2005	Age-standardized		22.3% (14.2 to 30.4%)	7.3 (3.9 to 11.9)
Tracheal, bronchus, and lung cancer	Myanmar	2010	Age-standardized		21.6% (13.8 to 29.6%)	7.2 (3.8 to 11.5)
Tracheal, bronchus, and lung cancer	Myanmar	2015	Age-standardized		24.1% (16.0 to 32.3%)	8.1 (4.5 to 12.6)
Cardiovascular diseases	Myanmar	1990	All Ages	10.7 (6.6 to 15.8)	13.9% (11.9 to 15.8%)	25.6 (15.7 to 37.5)
Cardiovascular diseases	Myanmar	1995	All Ages	11.9 (7.5 to 17.4)	13.9% (11.9 to 15.8%)	26.6 (16.8 to 38.7)
Cardiovascular diseases	Myanmar	2000	All Ages	12.9 (8.0 to 18.8)	14.0% (11.9 to 15.8%)	27.0 (16.7 to 39.4)
Cardiovascular diseases	Myanmar	2005	All Ages	13.7 (8.4 to 20.0)	14.4% (12.4 to 16.3%)	27.3 (16.9 to 40.0)
Cardiovascular diseases	Myanmar	2010	All Ages	13.8 (8.8 to 20.3)	14.3% (12.3 to 16.1%)	26.7 (16.9 to 39.2)
Cardiovascular diseases	Myanmar	2015	All Ages	15.6 (9.8 to 22.4)	15.2% (13.2 to 17.1%)	28.8 (18.0 to 41.5)
Cardiovascular diseases	Myanmar	1990	Age-standardized		13.1% (11.1 to 15.1%)	56.5 (36.1 to 80.8)
Cardiovascular diseases	Myanmar	1995	Age-standardized		13.2% (11.1 to 15.1%)	56.2 (36.5 to 80.4)
Cardiovascular diseases	Myanmar	2000	Age-standardized		13.2% (11.1 to 15.2%)	54.5 (34.5 to 77.9)
Cardiovascular diseases	Myanmar	2005	Age-standardized		13.5% (11.4 to 15.5%)	52.1 (33.3 to 74.4)
Cardiovascular diseases	Myanmar	2010	Age-standardized		13.3% (11.1 to 15.3%)	47.1 (30.5 to 67.2)

Cause name	Location	Year	Age group	Number (in thousands)	PAF	Rate (per 100,000)
Cardiovascular diseases	Myanmar	2015	Age-standardized		14.1% (11.8 to 16.2%)	46.9 (30.0 to 65.7)
Ischemic heart disease	Myanmar	1990	All Ages	4.2 (2.5 to 6.4)	20.8% (17.6 to 24.4%)	10.1 (6.0 to 15.3)
Ischemic heart disease	Myanmar	1995	All Ages	4.7 (2.8 to 7.1)	20.7% (17.5 to 24.2%)	10.5 (6.3 to 15.9)
Ischemic heart disease	Myanmar	2000	All Ages	5.0 (3.0 to 7.5)	20.6% (17.3 to 24.2%)	10.5 (6.4 to 15.8)
Ischemic heart disease	Myanmar	2005	All Ages	5.3 (3.2 to 7.9)	21.0% (17.5 to 24.7%)	10.5 (6.3 to 15.8)
Ischemic heart disease	Myanmar	2010	All Ages	5.4 (3.2 to 7.9)	20.7% (17.5 to 24.5%)	10.4 (6.2 to 15.3)
Ischemic heart disease	Myanmar	2015	All Ages	6.0 (3.6 to 8.9)	21.7% (18.3 to 25.5%)	11.1 (6.6 to 16.4)
Ischemic heart disease	Myanmar	1990	Age-standardized		19.0% (15.5 to 23.0%)	22.0 (13.5 to 33.0)
Ischemic heart disease	Myanmar	1995	Age-standardized		19.0% (15.5 to 22.9%)	22.0 (13.4 to 32.8)
Ischemic heart disease	Myanmar	2000	Age-standardized		18.9% (15.4 to 22.9%)	21.0 (13.0 to 30.5)
Ischemic heart disease	Myanmar	2005	Age-standardized		19.2% (15.6 to 23.3%)	19.8 (12.1 to 29.2)
Ischemic heart disease	Myanmar	2010	Age-standardized		18.9% (15.3 to 23.1%)	17.9 (11.1 to 26.0)
Ischemic heart disease	Myanmar	2015	Age-standardized		19.7% (16.0 to 24.2%)	17.7 (10.8 to 25.5)
Ischemic stroke	Myanmar	1990	All Ages	2.0 (1.2 to 3.1)	15.2% (11.8 to 18.3%)	4.9 (2.9 to 7.4)
Ischemic stroke	Myanmar	1995	All Ages	2.3 (1.4 to 3.5)	15.1% (11.7 to 18.2%)	5.1 (3.1 to 7.8)
Ischemic stroke	Myanmar	2000	All Ages	2.6 (1.6 to 3.9)	15.0% (11.6 to 18.1%)	5.4 (3.3 to 8.1)
Ischemic stroke	Myanmar	2005	All Ages	2.8 (1.7 to 4.0)	15.3% (11.8 to 18.5%)	5.5 (3.4 to 8.1)
Ischemic stroke	Myanmar	2010	All Ages	2.7 (1.7 to 4.1)	15.0% (11.6 to 18.3%)	5.3 (3.3 to 7.8)
Ischemic stroke	Myanmar	2015	All Ages	3.1 (1.9 to 4.5)	16.0% (12.7 to 19.3%)	5.7 (3.5 to 8.4)
Ischemic stroke	Myanmar	1990	Age-standardized		14.2% (10.5 to 17.8%)	12.5 (7.5 to 19.0)
Ischemic stroke	Myanmar	1995	Age-standardized		14.1% (10.6 to 17.7%)	12.4 (7.6 to 18.3)
Ischemic stroke	Myanmar	2000	Age-standardized		14.2% (10.6 to 17.8%)	12.3 (7.5 to 18.4)
Ischemic stroke	Myanmar	2005	Age-standardized		14.5% (10.8 to 18.2%)	12.0 (7.4 to 18.0)
Ischemic stroke	Myanmar	2010	Age-standardized		14.2% (10.6 to 18.0%)	10.9 (6.7 to 16.5)
Ischemic stroke	Myanmar	2015	Age-standardized		15.1% (11.4 to 18.9%)	10.9 (6.7 to 16.1)
Hemorrhagic stroke	Myanmar	1990	All Ages	4.5 (2.7 to 6.7)	16.9% (14.1 to 20.0%)	10.6 (6.4 to 15.9)
Hemorrhagic stroke	Myanmar	1995	All Ages	4.9 (3.0 to 7.3)	16.9% (13.9 to 19.8%)	10.9 (6.6 to 16.4)
Hemorrhagic stroke	Myanmar	2000	All Ages	5.3 (3.1 to 8.2)	16.9% (13.8 to 19.9%)	11.1 (6.5 to 17.1)
Hemorrhagic stroke	Myanmar	2005	All Ages	5.7 (3.3 to 8.6)	17.4% (14.3 to 20.4%)	11.3 (6.6 to 17.3)
Hemorrhagic stroke	Myanmar	2010	All Ages	5.7 (3.5 to 8.7)	17.2% (14.2 to 20.1%)	11.0 (6.8 to 16.9)
Hemorrhagic stroke	Myanmar	2015	All Ages	6.5 (3.9 to 9.7)	18.4% (15.4 to 21.4%)	12.0 (7.2 to 18.0)
Hemorrhagic stroke	Myanmar	1990	Age-standardized		15.9% (13.0 to 19.0%)	22.0 (13.8 to 32.6)
Hemorrhagic stroke	Myanmar	1995	Age-standardized		15.9% (12.8 to 18.9%)	21.8 (13.4 to 32.2)
Hemorrhagic stroke	Myanmar	2000	Age-standardized		15.9% (12.6 to 19.0%)	21.1 (12.6 to 32.1)
Hemorrhagic stroke	Myanmar	2005	Age-standardized		16.3% (12.9 to 19.3%)	20.3 (12.3 to 30.4)
Hemorrhagic stroke	Myanmar	2010	Age-standardized		15.9% (12.8 to 19.0%)	18.3 (11.2 to 27.0)

Cause name	Location	Year	Age group	Number (in thousands)	PAF	Rate (per 100,000)
Hemorrhagic stroke	Myanmar	2015	Age-standardized		16.9% (13.7 to 19.8%)	18.3 (11.3 to 27.0)
Chronic obstructive pulmonary disease	Myanmar	1990	All Ages	3.9 (1.8 to 6.6)	25.9% (15.0 to 37.1%)	9.2 (4.3 to 15.7)
Chronic obstructive pulmonary disease	Myanmar	1995	All Ages	4.5 (2.1 to 7.5)	26.4% (15.6 to 37.9%)	10.0 (4.8 to 16.7)
Chronic obstructive pulmonary disease	Myanmar	2000	All Ages	4.9 (2.4 to 8.2)	26.7% (16.0 to 37.9%)	10.3 (4.9 to 17.3)
Chronic obstructive pulmonary disease	Myanmar	2005	All Ages	5.3 (2.7 to 8.7)	28.0% (17.1 to 39.9%)	10.5 (5.4 to 17.3)
Chronic obstructive pulmonary disease	Myanmar	2010	All Ages	5.3 (2.6 to 8.8)	27.7% (16.8 to 39.6%)	10.3 (5.1 to 17.0)
Chronic obstructive pulmonary disease	Myanmar	2015	All Ages	6.2 (3.1 to 9.8)	30.2% (18.9 to 42.5%)	11.5 (5.7 to 18.2)
Chronic obstructive pulmonary disease	Myanmar	1990	Age-standardized		27.1% (15.9 to 38.4%)	24.0 (11.6 to 40.1)
Chronic obstructive pulmonary disease	Myanmar	1995	Age-standardized		27.2% (16.1 to 38.7%)	24.7 (11.9 to 40.1)
Chronic obstructive pulmonary disease	Myanmar	2000	Age-standardized		27.4% (16.4 to 38.9%)	24.1 (11.5 to 39.1)
Chronic obstructive pulmonary disease	Myanmar	2005	Age-standardized		28.5% (17.4 to 40.4%)	23.5 (12.0 to 37.6)
Chronic obstructive pulmonary disease	Myanmar	2010	Age-standardized		28.0% (16.9 to 39.9%)	21.5 (10.7 to 35.1)
Chronic obstructive pulmonary disease	Myanmar	2015	Age-standardized		30.3% (19.0 to 42.8%)	22.3 (11.4 to 35.4)
All causes	Philippines	1990	All Ages	22.8 (17.6 to 28.6)	6.3% (4.9 to 7.9%)	36.9 (28.5 to 46.1)
All causes	Philippines	1995	All Ages	23.4 (19.0 to 28.2)	6.3% (5.1 to 7.6%)	33.4 (27.1 to 40.4)
All causes	Philippines	2000	All Ages	26.4 (22.1 to 31.2)	6.5% (5.4 to 7.7%)	33.8 (28.3 to 40.0)
All causes	Philippines	2005	All Ages	33.3 (28.6 to 38.5)	7.2% (6.2 to 8.4%)	38.7 (33.2 to 44.8)
All causes	Philippines	2010	All Ages	36.2 (30.8 to 42.1)	7.0% (5.9 to 8.2%)	38.7 (32.9 to 45.0)
All causes	Philippines	2015	All Ages	40.6 (34.2 to 47.5)	7.3% (6.2 to 8.5%)	40.3 (33.9 to 47.2)
All causes	Philippines	1990	Age-standardized		7.3% (6.1 to 8.7%)	77.1 (63.9 to 92.1)
All causes	Philippines	1995	Age-standardized		7.5% (6.3 to 8.9%)	77.5 (64.7 to 91.8)
All causes	Philippines	2000	Age-standardized		7.8% (6.5 to 9.1%)	81.2 (68.0 to 95.6)
All causes	Philippines	2005	Age-standardized		8.5% (7.2 to 10.0%)	92.0 (77.9 to 107.9)
All causes	Philippines	2010	Age-standardized		7.9% (6.6 to 9.4%)	79.7 (66.6 to 94.6)
All causes	Philippines	2015	Age-standardized		8.0% (6.7 to 9.4%)	77.3 (64.3 to 92.0)
Lower respiratory infections	Philippines	1990	All Ages	11.1 (6.1 to 17.2)	19.4% (11.1 to 29.2%)	18.0 (9.9 to 27.8)
Lower respiratory infections	Philippines	1995	All Ages	9.2 (5.1 to 13.9)	19.5% (11.2 to 29.1%)	13.2 (7.3 to 19.8)
Lower respiratory infections	Philippines	2000	All Ages	9.2 (5.3 to 13.6)	19.9% (11.5 to 29.6%)	11.8 (6.8 to 17.5)
Lower respiratory infections	Philippines	2005	All Ages	10.3 (6.3 to 14.7)	21.5% (13.2 to 30.8%)	12.0 (7.3 to 17.1)
Lower respiratory infections	Philippines	2010	All Ages	10.1 (5.6 to 15.4)	18.9% (10.6 to 28.8%)	10.7 (6.0 to 16.4)
Lower respiratory infections	Philippines	2015	All Ages	10.4 (5.7 to 15.8)	18.9% (10.5 to 28.6%)	10.3 (5.6 to 15.6)
Lower respiratory infections	Philippines	1990	Age-standardized		19.4% (11.1 to 29.2%)	26.3 (15.0 to 40.0)
Lower respiratory infections	Philippines	1995	Age-standardized		19.5% (11.2 to 29.1%)	24.1 (13.7 to 36.0)
Lower respiratory infections	Philippines	2000	Age-standardized		19.9% (11.5 to 29.6%)	25.3 (14.4 to 37.7)
Lower respiratory infections	Philippines	2005	Age-standardized		21.5% (13.2 to 30.8%)	28.6 (17.9 to 41.1)
Lower respiratory infections	Philippines	2010	Age-standardized		18.9% (10.6 to 28.8%)	23.8 (13.4 to 36.5)

Cause name	Location	Year	Age group	Number (in thousands)	PAF	Rate (per 100,000)
Lower respiratory infections	Philippines	2015	Age-standardized		18.9% (10.5 to 28.6%)	21.8 (11.9 to 33.4)
Tracheal, bronchus, and lung cancer	Philippines	1990	All Ages	0.7 (0.4 to 1.0)	13.6% (7.7 to 20.1%)	1.0 (0.6 to 1.6)
Tracheal, bronchus, and lung cancer	Philippines	1995	All Ages	0.9 (0.5 to 1.3)	13.6% (7.7 to 20.3%)	1.2 (0.7 to 1.8)
Tracheal, bronchus, and lung cancer	Philippines	2000	All Ages	1.1 (0.6 to 1.6)	14.0% (8.0 to 20.7%)	1.4 (0.8 to 2.0)
Tracheal, bronchus, and lung cancer	Philippines	2005	All Ages	1.4 (0.8 to 2.0)	15.2% (8.7 to 22.1%)	1.6 (0.9 to 2.3)
Tracheal, bronchus, and lung cancer	Philippines	2010	All Ages	1.4 (0.8 to 2.0)	13.2% (7.3 to 19.9%)	1.4 (0.8 to 2.2)
Tracheal, bronchus, and lung cancer	Philippines	2015	All Ages	1.6 (0.9 to 2.5)	13.2% (7.5 to 19.9%)	1.6 (0.9 to 2.4)
Tracheal, bronchus, and lung cancer	Philippines	1990	Age-standardized		13.7% (7.7 to 20.3%)	2.6 (1.4 to 3.8)
Tracheal, bronchus, and lung cancer	Philippines	1995	Age-standardized		13.7% (7.7 to 20.4%)	3.0 (1.6 to 4.4)
Tracheal, bronchus, and lung cancer	Philippines	2000	Age-standardized		14.0% (8.0 to 20.8%)	3.2 (1.8 to 4.8)
Tracheal, bronchus, and lung cancer	Philippines	2005	Age-standardized		15.2% (8.8 to 22.2%)	3.5 (2.0 to 5.2)
Tracheal, bronchus, and lung cancer	Philippines	2010	Age-standardized		13.3% (7.4 to 19.9%)	2.7 (1.5 to 4.1)
Tracheal, bronchus, and lung cancer	Philippines	2015	Age-standardized		13.2% (7.5 to 19.9%)	2.8 (1.6 to 4.3)
Cardiovascular diseases	Philippines	1990	All Ages	9.8 (8.6 to 11.1)	13.6% (11.9 to 15.3%)	15.9 (13.9 to 18.0)
Cardiovascular diseases	Philippines	1995	All Ages	11.8 (10.2 to 13.3)	13.7% (11.8 to 15.5%)	16.9 (14.6 to 19.1)
Cardiovascular diseases	Philippines	2000	All Ages	14.2 (12.3 to 16.0)	13.8% (12.0 to 15.7%)	18.2 (15.7 to 20.5)
Cardiovascular diseases	Philippines	2005	All Ages	18.9 (16.5 to 21.3)	14.3% (12.5 to 16.2%)	21.9 (19.2 to 24.7)
Cardiovascular diseases	Philippines	2010	All Ages	21.8 (18.9 to 24.7)	13.5% (11.8 to 15.3%)	23.2 (20.2 to 26.4)
Cardiovascular diseases	Philippines	2015	All Ages	24.8 (21.1 to 28.7)	13.4% (11.6 to 15.2%)	24.6 (21.0 to 28.4)
Cardiovascular diseases	Philippines	1990	Age-standardized		12.6% (10.7 to 14.6%)	41.7 (35.2 to 48.2)
Cardiovascular diseases	Philippines	1995	Age-standardized		12.6% (10.6 to 14.7%)	43.6 (36.8 to 50.8)
Cardiovascular diseases	Philippines	2000	Age-standardized		12.6% (10.6 to 14.7%)	45.2 (38.2 to 52.6)
Cardiovascular diseases	Philippines	2005	Age-standardized		12.9% (11.0 to 14.9%)	50.7 (43.0 to 58.6)
Cardiovascular diseases	Philippines	2010	Age-standardized		12.2% (10.3 to 14.1%)	45.3 (38.1 to 52.4)
Cardiovascular diseases	Philippines	2015	Age-standardized		12.1% (10.3 to 14.0%)	44.5 (37.0 to 52.2)
Ischemic heart disease	Philippines	1990	All Ages	6.7 (5.7 to 7.8)	17.8% (15.1 to 20.5%)	10.9 (9.1 to 12.6)
Ischemic heart disease	Philippines	1995	All Ages	8.8 (7.4 to 10.1)	17.8% (15.0 to 20.6%)	12.6 (10.5 to 14.5)
Ischemic heart disease	Philippines	2000	All Ages	10.4 (8.7 to 12.1)	18.1% (15.2 to 20.9%)	13.3 (11.2 to 15.4)
Ischemic heart disease	Philippines	2005	All Ages	12.2 (10.2 to 14.2)	18.9% (15.9 to 21.8%)	14.1 (11.8 to 16.5)
Ischemic heart disease	Philippines	2010	All Ages	13.8 (11.6 to 16.1)	17.7% (14.8 to 20.5%)	14.8 (12.4 to 17.2)
Ischemic heart disease	Philippines	2015	All Ages	16.1 (13.3 to 19.1)	17.5% (14.6 to 20.5%)	16.0 (13.2 to 19.0)
Ischemic heart disease	Philippines	1990	Age-standardized		15.9% (12.7 to 19.1%)	28.4 (22.9 to 34.1)
Ischemic heart disease	Philippines	1995	Age-standardized		15.9% (12.7 to 19.1%)	32.4 (26.0 to 38.9)
Ischemic heart disease	Philippines	2000	Age-standardized		16.1% (12.9 to 19.2%)	33.0 (26.5 to 39.8)
Ischemic heart disease	Philippines	2005	Age-standardized		16.6% (13.4 to 20.0%)	32.6 (26.2 to 39.3)
Ischemic heart disease	Philippines	2010	Age-standardized		15.8% (12.7 to 18.9%)	28.9 (23.2 to 34.9)

Cause name	Location	Year	Age group	Number (in thousands)	PAF	Rate (per 100,000)
Ischemic heart disease	Philippines	2015	Age-standardized		15.7% (12.7 to 18.8%)	29.1 (23.0 to 35.3)
Ischemic stroke	Philippines	1990	All Ages	0.8 (0.6 to 1.0)	11.6% (8.5 to 14.6%)	1.3 (0.9 to 1.6)
Ischemic stroke	Philippines	1995	All Ages	0.7 (0.5 to 0.9)	11.6% (8.6 to 14.6%)	1.1 (0.8 to 1.3)
Ischemic stroke	Philippines	2000	All Ages	0.9 (0.7 to 1.2)	11.9% (8.9 to 14.8%)	1.2 (0.9 to 1.5)
Ischemic stroke	Philippines	2005	All Ages	1.6 (1.2 to 2.0)	12.7% (9.7 to 15.6%)	1.9 (1.4 to 2.3)
Ischemic stroke	Philippines	2010	All Ages	1.9 (1.4 to 2.4)	11.9% (8.9 to 14.8%)	2.0 (1.5 to 2.5)
Ischemic stroke	Philippines	2015	All Ages	2.1 (1.5 to 2.7)	11.8% (8.7 to 14.6%)	2.0 (1.5 to 2.6)
Ischemic stroke	Philippines	1990	Age-standardized		10.9% (7.5 to 14.4%)	4.3 (3.0 to 5.7)
Ischemic stroke	Philippines	1995	Age-standardized		10.9% (7.5 to 14.4%)	3.6 (2.5 to 4.8)
Ischemic stroke	Philippines	2000	Age-standardized		11.1% (7.7 to 14.6%)	3.9 (2.8 to 5.2)
Ischemic stroke	Philippines	2005	Age-standardized		11.8% (8.5 to 15.1%)	5.6 (4.0 to 7.2)
Ischemic stroke	Philippines	2010	Age-standardized		11.0% (7.8 to 14.3%)	4.9 (3.5 to 6.3)
Ischemic stroke	Philippines	2015	Age-standardized		11.0% (7.8 to 14.3%)	4.5 (3.1 to 6.0)
Hemorrhagic stroke	Philippines	1990	All Ages	2.4 (1.9 to 2.8)	13.9% (11.3 to 16.6%)	3.8 (3.1 to 4.5)
Hemorrhagic stroke	Philippines	1995	All Ages	2.3 (1.8 to 2.7)	13.9% (11.3 to 16.6%)	3.3 (2.6 to 3.9)
Hemorrhagic stroke	Philippines	2000	All Ages	2.9 (2.3 to 3.4)	14.2% (11.5 to 16.9%)	3.7 (3.0 to 4.4)
Hemorrhagic stroke	Philippines	2005	All Ages	5.2 (4.2 to 6.2)	15.1% (12.4 to 18.1%)	6.0 (4.9 to 7.2)
Hemorrhagic stroke	Philippines	2010	All Ages	6.0 (4.8 to 7.4)	13.9% (11.2 to 16.9%)	6.5 (5.1 to 7.9)
Hemorrhagic stroke	Philippines	2015	All Ages	6.6 (5.2 to 8.2)	13.8% (11.0 to 16.8%)	6.6 (5.2 to 8.1)
Hemorrhagic stroke	Philippines	1990	Age-standardized		13.0% (10.3 to 15.9%)	9.0 (7.1 to 11.0)
Hemorrhagic stroke	Philippines	1995	Age-standardized		13.0% (10.2 to 15.8%)	7.6 (6.0 to 9.3)
Hemorrhagic stroke	Philippines	2000	Age-standardized		13.1% (10.3 to 15.9%)	8.2 (6.5 to 10.0)
Hemorrhagic stroke	Philippines	2005	Age-standardized		13.8% (11.0 to 16.7%)	12.5 (9.9 to 15.3)
Hemorrhagic stroke	Philippines	2010	Age-standardized		12.8% (10.0 to 15.7%)	11.5 (9.0 to 14.1)
Hemorrhagic stroke	Philippines	2015	Age-standardized		12.7% (10.0 to 15.6%)	10.9 (8.5 to 13.5)
Chronic obstructive pulmonary disease	Philippines	1990	All Ages	1.2 (0.6 to 1.9)	17.3% (8.6 to 26.9%)	1.9 (0.9 to 3.0)
Chronic obstructive pulmonary disease	Philippines	1995	All Ages	1.5 (0.7 to 2.3)	18.9% (9.4 to 29.1%)	2.1 (1.1 to 3.3)
Chronic obstructive pulmonary disease	Philippines	2000	All Ages	1.9 (1.0 to 2.9)	20.0% (10.0 to 30.8%)	2.5 (1.3 to 3.8)
Chronic obstructive pulmonary disease	Philippines	2005	All Ages	2.8 (1.5 to 4.1)	21.4% (11.1 to 32.3%)	3.2 (1.7 to 4.8)
Chronic obstructive pulmonary disease	Philippines	2010	All Ages	3.1 (1.6 to 4.8)	19.5% (9.6 to 30.1%)	3.3 (1.7 to 5.1)
Chronic obstructive pulmonary disease	Philippines	2015	All Ages	3.8 (1.9 to 5.9)	19.5% (9.5 to 30.2%)	3.8 (1.9 to 5.8)
Chronic obstructive pulmonary disease	Philippines	1990	Age-standardized		19.7% (9.8 to 30.3%)	6.5 (3.2 to 10.2)
Chronic obstructive pulmonary disease	Philippines	1995	Age-standardized		20.0% (9.9 to 30.8%)	6.8 (3.4 to 10.6)
Chronic obstructive pulmonary disease	Philippines	2000	Age-standardized		20.5% (10.2 to 31.6%)	7.5 (3.8 to 11.5)
Chronic obstructive pulmonary disease	Philippines	2005	Age-standardized		21.7% (11.3 to 32.8%)	9.2 (4.8 to 13.7)
Chronic obstructive pulmonary disease	Philippines	2010	Age-standardized		19.7% (9.7 to 30.4%)	7.8 (3.9 to 12.0)

Cause name	Location	Year	Age group	Number (in thousands)	PAF	Rate (per 100,000)
Chronic obstructive pulmonary disease	Philippines	2015	Age-standardized		19.7% (9.6 to 30.4%)	8.2 (4.1 to 12.7)
All causes	Sri Lanka	1990	All Ages	6.0 (5.1 to 6.8)	5.8% (5.0 to 6.6%)	34.4 (29.5 to 39.5)
All causes	Sri Lanka	1995	All Ages	6.2 (5.4 to 7.0)	5.6% (4.9 to 6.4%)	34.0 (29.6 to 38.6)
All causes	Sri Lanka	2000	All Ages	7.1 (6.2 to 8.1)	6.1% (5.3 to 7.0%)	37.8 (32.8 to 42.9)
All causes	Sri Lanka	2005	All Ages	8.2 (7.1 to 9.3)	6.7% (5.8 to 7.6%)	41.9 (36.5 to 47.6)
All causes	Sri Lanka	2010	All Ages	7.4 (6.4 to 8.5)	6.2% (5.3 to 7.2%)	36.8 (31.7 to 42.3)
All causes	Sri Lanka	2015	All Ages	7.7 (5.9 to 9.8)	6.7% (5.7 to 7.7%)	37.2 (28.4 to 47.0)
All causes	Sri Lanka	1990	Age-standardized		6.6% (5.6 to 7.7%)	59.2 (49.9 to 68.7)
All causes	Sri Lanka	1995	Age-standardized		6.1% (5.3 to 7.1%)	53.9 (46.5 to 62.0)
All causes	Sri Lanka	2000	Age-standardized		6.5% (5.6 to 7.5%)	56.9 (48.9 to 65.5)
All causes	Sri Lanka	2005	Age-standardized		6.9% (5.9 to 7.9%)	57.6 (49.5 to 66.0)
All causes	Sri Lanka	2010	Age-standardized		6.3% (5.4 to 7.4%)	47.1 (40.0 to 54.6)
All causes	Sri Lanka	2015	Age-standardized		6.6% (5.6 to 7.7%)	41.9 (32.0 to 53.0)
Lower respiratory infections	Sri Lanka	1990	All Ages	1.5 (0.9 to 2.0)	22.5% (14.6 to 31.9%)	8.3 (5.4 to 11.7)
Lower respiratory infections	Sri Lanka	1995	All Ages	1.1 (0.7 to 1.6)	22.5% (14.4 to 31.8%)	6.2 (4.0 to 8.7)
Lower respiratory infections	Sri Lanka	2000	All Ages	1.1 (0.7 to 1.5)	22.9% (14.7 to 32.0%)	5.8 (3.7 to 8.1)
Lower respiratory infections	Sri Lanka	2005	All Ages	1.2 (0.8 to 1.7)	22.7% (14.6 to 32.1%)	6.0 (3.8 to 8.5)
Lower respiratory infections	Sri Lanka	2010	All Ages	1.0 (0.6 to 1.4)	20.8% (12.6 to 30.5%)	4.7 (2.8 to 6.9)
Lower respiratory infections	Sri Lanka	2015	All Ages	1.0 (0.6 to 1.4)	21.4% (12.9 to 30.6%)	4.6 (2.7 to 6.9)
Lower respiratory infections	Sri Lanka	1990	Age-standardized		22.5% (14.6 to 31.9%)	13.6 (8.8 to 19.2)
Lower respiratory infections	Sri Lanka	1995	Age-standardized		22.5% (14.4 to 31.8%)	9.8 (6.3 to 13.7)
Lower respiratory infections	Sri Lanka	2000	Age-standardized		22.9% (14.7 to 32.0%)	8.6 (5.6 to 12.0)
Lower respiratory infections	Sri Lanka	2005	Age-standardized		22.7% (14.6 to 32.1%)	8.1 (5.2 to 11.5)
Lower respiratory infections	Sri Lanka	2010	Age-standardized		20.8% (12.6 to 30.5%)	6.0 (3.6 to 8.8)
Lower respiratory infections	Sri Lanka	2015	Age-standardized		21.4% (12.9 to 30.6%)	5.3 (3.1 to 8.0)
Tracheal, bronchus, and lung cancer	Sri Lanka	1990	All Ages	0.1 (0.1 to 0.1)	16.0% (9.3 to 23.2%)	0.6 (0.3 to 0.9)
Tracheal, bronchus, and lung cancer	Sri Lanka	1995	All Ages	0.1 (0.1 to 0.2)	15.9% (9.4 to 23.3%)	0.7 (0.4 to 1.0)
Tracheal, bronchus, and lung cancer	Sri Lanka	2000	All Ages	0.2 (0.1 to 0.2)	16.2% (9.4 to 23.1%)	0.8 (0.5 to 1.2)
Tracheal, bronchus, and lung cancer	Sri Lanka	2005	All Ages	0.2 (0.1 to 0.3)	16.2% (9.7 to 23.3%)	0.9 (0.6 to 1.4)
Tracheal, bronchus, and lung cancer	Sri Lanka	2010	All Ages	0.2 (0.1 to 0.3)	14.7% (8.4 to 21.7%)	0.9 (0.5 to 1.3)
Tracheal, bronchus, and lung cancer	Sri Lanka	2015	All Ages	0.2 (0.1 to 0.3)	15.2% (8.8 to 22.1%)	1.0 (0.5 to 1.6)
Tracheal, bronchus, and lung cancer	Sri Lanka	1990	Age-standardized		16.0% (9.3 to 23.3%)	1.0 (0.6 to 1.4)
Tracheal, bronchus, and lung cancer	Sri Lanka	1995	Age-standardized		16.0% (9.4 to 23.3%)	1.1 (0.6 to 1.5)
Tracheal, bronchus, and lung cancer	Sri Lanka	2000	Age-standardized		16.3% (9.4 to 23.1%)	1.2 (0.7 to 1.7)
Tracheal, bronchus, and lung cancer	Sri Lanka	2005	Age-standardized		16.2% (9.7 to 23.3%)	1.2 (0.7 to 1.7)
Tracheal, bronchus, and lung cancer	Sri Lanka	2010	Age-standardized		14.7% (8.4 to 21.7%)	1.1 (0.6 to 1.6)

Cause name	Location	Year	Age group	Number (in thousands)	PAF	Rate (per 100,000)
Tracheal, bronchus, and lung cancer	Sri Lanka	2015	Age-standardized		15.2% (8.8 to 22.1%)	1.0 (0.5 to 1.6)
Cardiovascular diseases	Sri Lanka	1990	All Ages	4.1 (3.5 to 4.8)	13.4% (11.5 to 15.5%)	23.8 (20.3 to 27.5)
Cardiovascular diseases	Sri Lanka	1995	All Ages	4.3 (3.7 to 5.0)	12.5% (10.6 to 14.3%)	23.8 (20.4 to 27.4)
Cardiovascular diseases	Sri Lanka	2000	All Ages	5.1 (4.4 to 5.9)	13.4% (11.4 to 15.4%)	27.2 (23.2 to 31.3)
Cardiovascular diseases	Sri Lanka	2005	All Ages	5.9 (5.0 to 6.8)	13.8% (11.8 to 15.9%)	30.1 (25.6 to 34.9)
Cardiovascular diseases	Sri Lanka	2010	All Ages	5.5 (4.7 to 6.4)	13.4% (11.3 to 15.5%)	27.3 (23.0 to 31.7)
Cardiovascular diseases	Sri Lanka	2015	All Ages	5.7 (4.2 to 7.2)	13.3% (11.2 to 15.6%)	27.3 (20.4 to 34.5)
Cardiovascular diseases	Sri Lanka	1990	Age-standardized		12.9% (10.7 to 15.2%)	41.4 (34.6 to 49.3)
Cardiovascular diseases	Sri Lanka	1995	Age-standardized		11.9% (10.0 to 14.0%)	37.6 (31.7 to 44.2)
Cardiovascular diseases	Sri Lanka	2000	Age-standardized		12.7% (10.6 to 14.9%)	40.7 (34.1 to 47.7)
Cardiovascular diseases	Sri Lanka	2005	Age-standardized		13.2% (11.0 to 15.5%)	41.0 (34.1 to 48.4)
Cardiovascular diseases	Sri Lanka	2010	Age-standardized		12.7% (10.6 to 15.0%)	34.8 (28.8 to 41.0)
Cardiovascular diseases	Sri Lanka	2015	Age-standardized		12.7% (10.4 to 15.0%)	30.5 (22.7 to 38.8)
Ischemic heart disease	Sri Lanka	1990	All Ages	3.5 (2.9 to 4.1)	17.7% (14.8 to 20.8%)	19.9 (16.6 to 23.5)
Ischemic heart disease	Sri Lanka	1995	All Ages	3.3 (2.7 to 3.9)	17.9% (14.9 to 21.1%)	17.9 (14.8 to 21.1)
Ischemic heart disease	Sri Lanka	2000	All Ages	3.8 (3.1 to 4.5)	18.0% (15.0 to 21.3%)	20.0 (16.6 to 23.8)
Ischemic heart disease	Sri Lanka	2005	All Ages	4.0 (3.3 to 4.8)	17.6% (14.4 to 20.8%)	20.6 (16.9 to 24.8)
Ischemic heart disease	Sri Lanka	2010	All Ages	3.9 (3.2 to 4.7)	16.8% (13.8 to 19.9%)	19.4 (15.9 to 23.0)
Ischemic heart disease	Sri Lanka	2015	All Ages	4.0 (2.9 to 5.2)	16.8% (13.8 to 20.1%)	19.4 (14.2 to 25.1)
Ischemic heart disease	Sri Lanka	1990	Age-standardized		16.6% (13.3 to 19.9%)	34.7 (27.7 to 41.9)
Ischemic heart disease	Sri Lanka	1995	Age-standardized		17.0% (13.6 to 20.3%)	27.8 (22.3 to 33.5)
Ischemic heart disease	Sri Lanka	2000	Age-standardized		16.9% (13.6 to 20.4%)	29.3 (23.5 to 35.2)
Ischemic heart disease	Sri Lanka	2005	Age-standardized		16.7% (13.2 to 20.0%)	27.5 (21.9 to 33.3)
Ischemic heart disease	Sri Lanka	2010	Age-standardized		15.9% (12.6 to 19.3%)	24.1 (19.0 to 29.4)
Ischemic heart disease	Sri Lanka	2015	Age-standardized		16.0% (12.6 to 19.4%)	21.1 (15.3 to 27.7)
Ischemic stroke	Sri Lanka	1990	All Ages	0.3 (0.2 to 0.4)	12.1% (8.8 to 15.5%)	1.6 (1.2 to 2.0)
Ischemic stroke	Sri Lanka	1995	All Ages	0.5 (0.3 to 0.6)	12.0% (8.5 to 15.4%)	2.6 (1.9 to 3.4)
Ischemic stroke	Sri Lanka	2000	All Ages	0.7 (0.5 to 0.9)	12.0% (8.5 to 15.5%)	3.6 (2.5 to 4.7)
Ischemic stroke	Sri Lanka	2005	All Ages	1.0 (0.7 to 1.4)	11.8% (8.3 to 15.5%)	5.3 (3.7 to 7.0)
Ischemic stroke	Sri Lanka	2010	All Ages	0.9 (0.6 to 1.2)	11.2% (7.8 to 15.1%)	4.4 (3.1 to 6.0)
Ischemic stroke	Sri Lanka	2015	All Ages	0.9 (0.6 to 1.3)	11.3% (7.7 to 15.2%)	4.4 (2.9 to 6.3)
Ischemic stroke	Sri Lanka	1990	Age-standardized		11.6% (8.0 to 15.6%)	3.2 (2.2 to 4.3)
Ischemic stroke	Sri Lanka	1995	Age-standardized		11.6% (8.0 to 15.5%)	4.8 (3.3 to 6.4)
Ischemic stroke	Sri Lanka	2000	Age-standardized		11.6% (8.0 to 15.6%)	6.2 (4.3 to 8.4)
Ischemic stroke	Sri Lanka	2005	Age-standardized		11.5% (7.8 to 15.7%)	8.2 (5.6 to 11.1)
Ischemic stroke	Sri Lanka	2010	Age-standardized		10.9% (7.3 to 15.0%)	6.4 (4.3 to 8.9)

Cause name	Location	Year	Age group	Number (in thousands)	PAF	Rate (per 100,000)
Ischemic stroke	Sri Lanka	2015	Age-standardized		11.0% (7.2 to 15.2%)	5.6 (3.6 to 8.2)
Hemorrhagic stroke	Sri Lanka	1990	All Ages	0.4 (0.3 to 0.5)	14.0% (11.5 to 16.5%)	2.2 (1.8 to 2.7)
Hemorrhagic stroke	Sri Lanka	1995	All Ages	0.6 (0.5 to 0.7)	14.1% (11.3 to 16.8%)	3.3 (2.6 to 4.0)
Hemorrhagic stroke	Sri Lanka	2000	All Ages	0.7 (0.5 to 0.8)	14.1% (11.3 to 16.9%)	3.6 (2.8 to 4.3)
Hemorrhagic stroke	Sri Lanka	2005	All Ages	0.8 (0.6 to 1.0)	13.9% (11.1 to 16.8%)	4.1 (3.3 to 5.0)
Hemorrhagic stroke	Sri Lanka	2010	All Ages	0.7 (0.6 to 0.9)	13.1% (10.4 to 16.0%)	3.5 (2.7 to 4.2)
Hemorrhagic stroke	Sri Lanka	2015	All Ages	0.7 (0.5 to 1.0)	13.2% (10.4 to 16.2%)	3.5 (2.6 to 4.7)
Hemorrhagic stroke	Sri Lanka	1990	Age-standardized		13.5% (10.8 to 16.3%)	3.6 (2.8 to 4.4)
Hemorrhagic stroke	Sri Lanka	1995	Age-standardized		13.4% (10.5 to 16.3%)	5.0 (3.9 to 6.2)
Hemorrhagic stroke	Sri Lanka	2000	Age-standardized		13.4% (10.4 to 16.3%)	5.1 (4.0 to 6.3)
Hemorrhagic stroke	Sri Lanka	2005	Age-standardized		13.3% (10.4 to 16.3%)	5.4 (4.1 to 6.7)
Hemorrhagic stroke	Sri Lanka	2010	Age-standardized		12.5% (9.6 to 15.4%)	4.3 (3.3 to 5.4)
Hemorrhagic stroke	Sri Lanka	2015	Age-standardized		12.6% (9.6 to 15.6%)	3.8 (2.7 to 5.2)
Chronic obstructive pulmonary disease	Sri Lanka	1990	All Ages	0.3 (0.1 to 0.5)	20.6% (11.2 to 31.0%)	1.7 (0.9 to 2.6)
Chronic obstructive pulmonary disease	Sri Lanka	1995	All Ages	0.6 (0.3 to 0.9)	22.0% (11.8 to 33.0%)	3.2 (1.7 to 5.0)
Chronic obstructive pulmonary disease	Sri Lanka	2000	All Ages	0.8 (0.4 to 1.1)	22.5% (12.1 to 33.5%)	4.0 (2.1 to 6.0)
Chronic obstructive pulmonary disease	Sri Lanka	2005	All Ages	1.0 (0.5 to 1.4)	22.6% (12.2 to 33.6%)	4.9 (2.6 to 7.3)
Chronic obstructive pulmonary disease	Sri Lanka	2010	All Ages	0.8 (0.4 to 1.2)	21.1% (10.9 to 32.0%)	3.9 (2.0 to 5.9)
Chronic obstructive pulmonary disease	Sri Lanka	2015	All Ages	0.9 (0.5 to 1.4)	21.6% (11.4 to 32.8%)	4.3 (2.2 to 6.6)
Chronic obstructive pulmonary disease	Sri Lanka	1990	Age-standardized		21.7% (11.7 to 32.4%)	3.2 (1.7 to 5.0)
Chronic obstructive pulmonary disease	Sri Lanka	1995	Age-standardized		22.3% (12.0 to 33.4%)	5.4 (2.8 to 8.4)
Chronic obstructive pulmonary disease	Sri Lanka	2000	Age-standardized		22.7% (12.2 to 33.7%)	6.4 (3.4 to 9.7)
Chronic obstructive pulmonary disease	Sri Lanka	2005	Age-standardized		22.7% (12.2 to 33.7%)	7.2 (3.8 to 10.7)
Chronic obstructive pulmonary disease	Sri Lanka	2010	Age-standardized		21.2% (10.9 to 32.1%)	5.2 (2.7 to 8.0)
Chronic obstructive pulmonary disease	Sri Lanka	2015	Age-standardized		21.6% (11.4 to 32.8%)	5.1 (2.6 to 7.8)
All causes	Seychelles	1990	All Ages	0.0 (0.0 to 0.1)	5.7% (3.4 to 8.4%)	45.6 (27.3 to 67.2)
All causes	Seychelles	1995	All Ages	0.0 (0.0 to 0.1)	5.5% (3.3 to 8.0%)	42.8 (25.1 to 62.1)
All causes	Seychelles	2000	All Ages	0.0 (0.0 to 0.1)	5.3% (3.1 to 7.7%)	41.9 (24.8 to 61.5)
All causes	Seychelles	2005	All Ages	0.0 (0.0 to 0.1)	5.2% (2.9 to 7.5%)	38.0 (22.0 to 55.8)
All causes	Seychelles	2010	All Ages	0.0 (0.0 to 0.1)	4.5% (2.4 to 6.8%)	32.6 (17.5 to 49.3)
All causes	Seychelles	2015	All Ages	0.0 (0.0 to 0.0)	4.1% (2.0 to 6.2%)	29.5 (15.0 to 45.1)
All causes	Seychelles	1990	Age-standardized		5.9% (3.6 to 8.7%)	67.4 (40.5 to 98.5)
All causes	Seychelles	1995	Age-standardized		5.7% (3.4 to 8.2%)	61.6 (36.1 to 89.4)
All causes	Seychelles	2000	Age-standardized		5.4% (3.2 to 7.9%)	54.7 (32.3 to 80.0)
All causes	Seychelles	2005	Age-standardized		5.3% (3.0 to 7.8%)	51.3 (29.6 to 75.4)
All causes	Seychelles	2010	Age-standardized		4.6% (2.5 to 7.0%)	40.5 (21.8 to 61.2)

Cause name	Location	Year	Age group	Number (in thousands)	PAF	Rate (per 100,000)
All causes	Seychelles	2015	Age-standardized		4.1% (2.1 to 6.3%)	34.6 (17.3 to 52.8)
Lower respiratory infections	Seychelles	1990	All Ages	0.0 (0.0 to 0.0)	16.0% (4.4 to 30.0%)	13.0 (3.7 to 24.5)
Lower respiratory infections	Seychelles	1995	All Ages	0.0 (0.0 to 0.0)	15.8% (3.6 to 29.2%)	11.4 (2.5 to 21.3)
Lower respiratory infections	Seychelles	2000	All Ages	0.0 (0.0 to 0.0)	15.7% (4.0 to 29.4%)	11.4 (3.0 to 21.6)
Lower respiratory infections	Seychelles	2005	All Ages	0.0 (0.0 to 0.0)	16.2% (3.9 to 30.0%)	11.2 (2.8 to 21.1)
Lower respiratory infections	Seychelles	2010	All Ages	0.0 (0.0 to 0.0)	13.4% (2.3 to 26.2%)	9.4 (1.6 to 19.0)
Lower respiratory infections	Seychelles	2015	All Ages	0.0 (0.0 to 0.0)	11.3% (1.3 to 24.1%)	7.9 (0.9 to 17.8)
Lower respiratory infections	Seychelles	1990	Age-standardized		16.0% (4.4 to 30.0%)	18.1 (5.1 to 34.1)
Lower respiratory infections	Seychelles	1995	Age-standardized		15.8% (3.6 to 29.2%)	16.0 (3.4 to 30.0)
Lower respiratory infections	Seychelles	2000	Age-standardized		15.7% (4.0 to 29.4%)	14.4 (3.8 to 27.5)
Lower respiratory infections	Seychelles	2005	Age-standardized		16.2% (3.9 to 30.0%)	14.9 (3.7 to 28.1)
Lower respiratory infections	Seychelles	2010	Age-standardized		13.4% (2.3 to 26.2%)	11.8 (2.0 to 23.9)
Lower respiratory infections	Seychelles	2015	Age-standardized		11.3% (1.3 to 24.1%)	9.5 (1.1 to 21.4)
Tracheal, bronchus, and lung cancer	Seychelles	1990	All Ages	0.0 (0.0 to 0.0)	11.2% (3.8 to 21.1%)	1.1 (0.4 to 2.1)
Tracheal, bronchus, and lung cancer	Seychelles	1995	All Ages	0.0 (0.0 to 0.0)	11.0% (3.6 to 21.1%)	1.5 (0.5 to 2.9)
Tracheal, bronchus, and lung cancer	Seychelles	2000	All Ages	0.0 (0.0 to 0.0)	11.0% (4.0 to 21.0%)	1.7 (0.6 to 3.3)
Tracheal, bronchus, and lung cancer	Seychelles	2005	All Ages	0.0 (0.0 to 0.0)	11.3% (3.9 to 21.7%)	1.6 (0.6 to 3.1)
Tracheal, bronchus, and lung cancer	Seychelles	2010	All Ages	0.0 (0.0 to 0.0)	9.2% (2.5 to 19.2%)	1.3 (0.4 to 2.7)
Tracheal, bronchus, and lung cancer	Seychelles	2015	All Ages	0.0 (0.0 to 0.0)	7.7% (1.9 to 16.5%)	1.2 (0.3 to 2.5)
Tracheal, bronchus, and lung cancer	Seychelles	1990	Age-standardized		11.2% (3.8 to 21.2%)	1.7 (0.6 to 3.1)
Tracheal, bronchus, and lung cancer	Seychelles	1995	Age-standardized		11.1% (3.6 to 21.2%)	2.2 (0.7 to 4.2)
Tracheal, bronchus, and lung cancer	Seychelles	2000	Age-standardized		11.0% (4.0 to 21.0%)	2.3 (0.8 to 4.4)
Tracheal, bronchus, and lung cancer	Seychelles	2005	Age-standardized		11.3% (3.9 to 21.7%)	2.2 (0.8 to 4.3)
Tracheal, bronchus, and lung cancer	Seychelles	2010	Age-standardized		9.3% (2.5 to 19.2%)	1.7 (0.4 to 3.5)
Tracheal, bronchus, and lung cancer	Seychelles	2015	Age-standardized		7.8% (1.9 to 16.5%)	1.4 (0.3 to 2.9)
Cardiovascular diseases	Seychelles	1990	All Ages	0.0 (0.0 to 0.0)	8.9% (6.2 to 11.8%)	27.9 (19.4 to 37.4)
Cardiovascular diseases	Seychelles	1995	All Ages	0.0 (0.0 to 0.0)	9.1% (6.2 to 12.1%)	26.1 (17.7 to 35.0)
Cardiovascular diseases	Seychelles	2000	All Ages	0.0 (0.0 to 0.0)	8.8% (6.1 to 11.9%)	24.8 (16.9 to 33.2)
Cardiovascular diseases	Seychelles	2005	All Ages	0.0 (0.0 to 0.0)	8.8% (6.0 to 11.7%)	21.8 (14.8 to 29.2)
Cardiovascular diseases	Seychelles	2010	All Ages	0.0 (0.0 to 0.0)	8.1% (5.2 to 11.1%)	19.1 (12.3 to 26.2)
Cardiovascular diseases	Seychelles	2015	All Ages	0.0 (0.0 to 0.0)	7.5% (4.6 to 10.3%)	18.0 (10.9 to 24.9)
Cardiovascular diseases	Seychelles	1990	Age-standardized		9.0% (6.3 to 11.9%)	42.1 (29.4 to 56.3)
Cardiovascular diseases	Seychelles	1995	Age-standardized		9.0% (6.1 to 12.1%)	37.8 (25.6 to 51.0)
Cardiovascular diseases	Seychelles	2000	Age-standardized		8.9% (6.1 to 11.9%)	32.7 (22.3 to 44.0)
Cardiovascular diseases	Seychelles	2005	Age-standardized		8.6% (5.9 to 11.6%)	29.3 (19.8 to 39.5)
Cardiovascular diseases	Seychelles	2010	Age-standardized		7.9% (5.1 to 10.8%)	23.5 (15.3 to 32.0)

Cause name	Location	Year	Age group	Number (in thousands)	PAF	Rate (per 100,000)
Cardiovascular diseases	Seychelles	2015	Age-standardized		7.2% (4.3 to 9.9%)	20.7 (12.1 to 28.6)
Ischemic heart disease	Seychelles	1990	All Ages	0.0 (0.0 to 0.0)	14.9% (10.4 to 19.6%)	19.5 (13.4 to 26.1)
Ischemic heart disease	Seychelles	1995	All Ages	0.0 (0.0 to 0.0)	15.0% (10.2 to 19.9%)	19.1 (12.9 to 25.4)
Ischemic heart disease	Seychelles	2000	All Ages	0.0 (0.0 to 0.0)	14.6% (9.8 to 19.8%)	18.5 (12.3 to 25.2)
Ischemic heart disease	Seychelles	2005	All Ages	0.0 (0.0 to 0.0)	14.8% (10.4 to 19.7%)	15.9 (10.8 to 21.6)
Ischemic heart disease	Seychelles	2010	All Ages	0.0 (0.0 to 0.0)	13.6% (8.7 to 18.6%)	14.0 (8.8 to 19.2)
Ischemic heart disease	Seychelles	2015	All Ages	0.0 (0.0 to 0.0)	12.6% (7.9 to 17.5%)	13.4 (8.2 to 18.8)
Ischemic heart disease	Seychelles	1990	Age-standardized		14.9% (10.5 to 19.6%)	29.4 (20.3 to 39.4)
Ischemic heart disease	Seychelles	1995	Age-standardized		14.9% (10.0 to 19.8%)	27.7 (18.6 to 36.9)
Ischemic heart disease	Seychelles	2000	Age-standardized		14.6% (9.7 to 19.8%)	24.4 (16.0 to 33.3)
Ischemic heart disease	Seychelles	2005	Age-standardized		14.5% (9.9 to 19.5%)	21.5 (14.5 to 29.2)
Ischemic heart disease	Seychelles	2010	Age-standardized		13.3% (8.5 to 18.2%)	17.2 (10.8 to 23.5)
Ischemic heart disease	Seychelles	2015	Age-standardized		12.2% (7.5 to 16.8%)	15.4 (9.2 to 21.6)
Ischemic stroke	Seychelles	1990	All Ages	0.0 (0.0 to 0.0)	10.0% (5.6 to 14.7%)	3.4 (1.9 to 5.1)
Ischemic stroke	Seychelles	1995	All Ages	0.0 (0.0 to 0.0)	10.0% (5.5 to 15.2%)	2.7 (1.5 to 4.2)
Ischemic stroke	Seychelles	2000	All Ages	0.0 (0.0 to 0.0)	9.9% (5.4 to 14.6%)	2.5 (1.4 to 3.8)
Ischemic stroke	Seychelles	2005	All Ages	0.0 (0.0 to 0.0)	10.0% (5.4 to 15.0%)	2.3 (1.2 to 3.5)
Ischemic stroke	Seychelles	2010	All Ages	0.0 (0.0 to 0.0)	9.0% (4.6 to 13.5%)	2.0 (1.0 to 3.1)
Ischemic stroke	Seychelles	2015	All Ages	0.0 (0.0 to 0.0)	8.2% (3.9 to 12.9%)	1.8 (0.8 to 2.9)
Ischemic stroke	Seychelles	1990	Age-standardized		10.0% (5.6 to 14.7%)	5.1 (2.8 to 7.6)
Ischemic stroke	Seychelles	1995	Age-standardized		10.0% (5.5 to 15.2%)	4.0 (2.2 to 6.2)
Ischemic stroke	Seychelles	2000	Age-standardized		9.9% (5.4 to 14.6%)	3.3 (1.8 to 5.0)
Ischemic stroke	Seychelles	2005	Age-standardized		10.0% (5.4 to 15.0%)	3.2 (1.7 to 5.0)
Ischemic stroke	Seychelles	2010	Age-standardized		9.0% (4.6 to 13.4%)	2.6 (1.3 to 4.0)
Ischemic stroke	Seychelles	2015	Age-standardized		8.1% (3.8 to 12.9%)	2.2 (1.0 to 3.6)
Hemorrhagic stroke	Seychelles	1990	All Ages	0.0 (0.0 to 0.0)	11.4% (7.0 to 16.1%)	5.1 (3.1 to 7.5)
Hemorrhagic stroke	Seychelles	1995	All Ages	0.0 (0.0 to 0.0)	11.6% (6.9 to 16.7%)	4.3 (2.5 to 6.2)
Hemorrhagic stroke	Seychelles	2000	All Ages	0.0 (0.0 to 0.0)	11.5% (7.1 to 16.3%)	3.8 (2.2 to 5.4)
Hemorrhagic stroke	Seychelles	2005	All Ages	0.0 (0.0 to 0.0)	11.8% (7.2 to 16.7%)	3.6 (2.1 to 5.2)
Hemorrhagic stroke	Seychelles	2010	All Ages	0.0 (0.0 to 0.0)	10.6% (6.2 to 15.1%)	3.1 (1.7 to 4.6)
Hemorrhagic stroke	Seychelles	2015	All Ages	0.0 (0.0 to 0.0)	9.6% (4.9 to 14.2%)	2.8 (1.4 to 4.3)
Hemorrhagic stroke	Seychelles	1990	Age-standardized		11.4% (7.0 to 16.3%)	7.7 (4.7 to 11.3)
Hemorrhagic stroke	Seychelles	1995	Age-standardized		11.5% (6.7 to 16.6%)	6.2 (3.5 to 8.9)
Hemorrhagic stroke	Seychelles	2000	Age-standardized		11.5% (7.0 to 16.3%)	5.0 (3.0 to 7.1)
Hemorrhagic stroke	Seychelles	2005	Age-standardized		11.5% (6.8 to 16.5%)	4.7 (2.8 to 6.8)
Hemorrhagic stroke	Seychelles	2010	Age-standardized		10.3% (5.7 to 14.6%)	3.7 (2.1 to 5.5)

Cause name	Location	Year	Age group	Number (in thousands)	PAF	Rate (per 100,000)
Hemorrhagic stroke	Seychelles	2015	Age-standardized		9.2% (4.6 to 13.6%)	3.1 (1.6 to 4.8)
Chronic obstructive pulmonary disease	Seychelles	1990	All Ages	0.0 (0.0 to 0.0)	17.2% (6.0 to 29.9%)	3.6 (1.3 to 6.5)
Chronic obstructive pulmonary disease	Seychelles	1995	All Ages	0.0 (0.0 to 0.0)	17.0% (5.4 to 30.4%)	3.9 (1.2 to 6.9)
Chronic obstructive pulmonary disease	Seychelles	2000	All Ages	0.0 (0.0 to 0.0)	17.1% (5.6 to 30.0%)	4.1 (1.3 to 7.2)
Chronic obstructive pulmonary disease	Seychelles	2005	All Ages	0.0 (0.0 to 0.0)	17.4% (6.2 to 32.0%)	3.4 (1.2 to 6.2)
Chronic obstructive pulmonary disease	Seychelles	2010	All Ages	0.0 (0.0 to 0.0)	15.1% (4.5 to 28.5%)	2.7 (0.8 to 5.2)
Chronic obstructive pulmonary disease	Seychelles	2015	All Ages	0.0 (0.0 to 0.0)	13.2% (3.3 to 25.4%)	2.4 (0.6 to 4.8)
Chronic obstructive pulmonary disease	Seychelles	1990	Age-standardized		17.3% (6.1 to 30.1%)	5.5 (1.9 to 9.7)
Chronic obstructive pulmonary disease	Seychelles	1995	Age-standardized		17.1% (5.4 to 30.5%)	5.7 (1.8 to 10.1)
Chronic obstructive pulmonary disease	Seychelles	2000	Age-standardized		17.2% (5.6 to 30.2%)	5.3 (1.7 to 9.5)
Chronic obstructive pulmonary disease	Seychelles	2005	Age-standardized		17.4% (6.3 to 32.1%)	4.8 (1.7 to 8.6)
Chronic obstructive pulmonary disease	Seychelles	2010	Age-standardized		15.1% (4.5 to 28.6%)	3.6 (1.0 to 6.8)
Chronic obstructive pulmonary disease	Seychelles	2015	Age-standardized		13.2% (3.3 to 25.4%)	3.0 (0.8 to 5.9)
All causes	Thailand	1990	All Ages	17.7 (14.9 to 20.8)	5.7% (4.8 to 6.7%)	31.3 (26.4 to 36.8)
All causes	Thailand	1995	All Ages	21.7 (18.4 to 25.0)	5.8% (5.0 to 6.7%)	36.5 (31.0 to 42.2)
All causes	Thailand	2000	All Ages	25.3 (21.4 to 29.7)	5.7% (4.8 to 6.6%)	40.3 (34.2 to 47.3)
All causes	Thailand	2005	All Ages	26.1 (21.2 to 31.8)	5.8% (4.8 to 7.1%)	39.7 (32.3 to 48.3)
All causes	Thailand	2010	All Ages	28.5 (22.5 to 35.5)	5.7% (4.5 to 7.0%)	42.8 (33.8 to 53.2)
All causes	Thailand	2015	All Ages	37.6 (29.1 to 46.6)	6.4% (5.3 to 7.8%)	55.3 (42.9 to 68.6)
All causes	Thailand	1990	Age-standardized		6.5% (5.4 to 7.7%)	60.1 (49.8 to 71.9)
All causes	Thailand	1995	Age-standardized		6.5% (5.5 to 7.6%)	62.2 (52.3 to 72.8)
All causes	Thailand	2000	Age-standardized		6.3% (5.2 to 7.5%)	60.9 (50.6 to 72.3)
All causes	Thailand	2005	Age-standardized		6.3% (5.1 to 7.7%)	52.4 (41.9 to 64.5)
All causes	Thailand	2010	Age-standardized		5.9% (4.7 to 7.4%)	47.0 (36.8 to 59.2)
All causes	Thailand	2015	Age-standardized		6.5% (5.3 to 8.0%)	51.4 (39.9 to 64.0)
Lower respiratory infections	Thailand	1990	All Ages	4.5 (2.5 to 7.0)	19.0% (10.5 to 29.1%)	8.0 (4.4 to 12.3)
Lower respiratory infections	Thailand	1995	All Ages	4.3 (2.4 to 6.6)	19.1% (10.6 to 29.1%)	7.3 (4.0 to 11.2)
Lower respiratory infections	Thailand	2000	All Ages	6.0 (3.3 to 9.4)	19.2% (10.6 to 29.1%)	9.6 (5.3 to 15.0)
Lower respiratory infections	Thailand	2005	All Ages	8.6 (4.3 to 13.8)	19.8% (11.3 to 29.6%)	13.1 (6.6 to 21.1)
Lower respiratory infections	Thailand	2010	All Ages	9.7 (4.4 to 16.2)	18.3% (9.8 to 28.4%)	14.5 (6.6 to 24.3)
Lower respiratory infections	Thailand	2015	All Ages	12.3 (6.4 to 19.4)	20.7% (12.1 to 30.4%)	18.0 (9.4 to 28.6)
Lower respiratory infections	Thailand	1990	Age-standardized		19.0% (10.5 to 29.1%)	15.7 (8.7 to 24.4)
Lower respiratory infections	Thailand	1995	Age-standardized		19.1% (10.6 to 29.1%)	13.2 (7.3 to 20.3)
Lower respiratory infections	Thailand	2000	Age-standardized		19.2% (10.6 to 29.1%)	15.8 (8.6 to 24.7)
Lower respiratory infections	Thailand	2005	Age-standardized		19.8% (11.3 to 29.6%)	18.5 (9.3 to 30.0)
Lower respiratory infections	Thailand	2010	Age-standardized		18.3% (9.8 to 28.4%)	16.8 (7.6 to 28.2)

Cause name	Location	Year	Age group	Number (in thousands)	PAF	Rate (per 100,000)
Lower respiratory infections	Thailand	2015	Age-standardized		20.7% (12.1 to 30.4%)	17.4 (9.1 to 27.6)
Tracheal, bronchus, and lung cancer	Thailand	1990	All Ages	0.9 (0.5 to 1.4)	13.3% (7.5 to 20.0%)	1.7 (0.9 to 2.5)
Tracheal, bronchus, and lung cancer	Thailand	1995	All Ages	1.3 (0.8 to 2.0)	13.4% (7.5 to 20.0%)	2.2 (1.3 to 3.4)
Tracheal, bronchus, and lung cancer	Thailand	2000	All Ages	2.0 (1.1 to 3.0)	13.4% (7.6 to 20.1%)	3.2 (1.8 to 4.8)
Tracheal, bronchus, and lung cancer	Thailand	2005	All Ages	2.4 (1.3 to 3.5)	13.9% (7.9 to 20.7%)	3.6 (2.0 to 5.3)
Tracheal, bronchus, and lung cancer	Thailand	2010	All Ages	2.8 (1.5 to 4.3)	12.9% (7.2 to 19.4%)	4.1 (2.3 to 6.5)
Tracheal, bronchus, and lung cancer	Thailand	2015	All Ages	3.9 (2.2 to 6.1)	14.6% (8.4 to 21.5%)	5.8 (3.2 to 8.9)
Tracheal, bronchus, and lung cancer	Thailand	1990	Age-standardized		13.4% (7.5 to 20.0%)	3.0 (1.7 to 4.6)
Tracheal, bronchus, and lung cancer	Thailand	1995	Age-standardized		13.4% (7.6 to 20.0%)	3.6 (2.0 to 5.3)
Tracheal, bronchus, and lung cancer	Thailand	2000	Age-standardized		13.5% (7.6 to 20.1%)	4.4 (2.4 to 6.6)
Tracheal, bronchus, and lung cancer	Thailand	2005	Age-standardized		13.9% (7.9 to 20.7%)	4.4 (2.4 to 6.5)
Tracheal, bronchus, and lung cancer	Thailand	2010	Age-standardized		12.9% (7.2 to 19.4%)	4.3 (2.4 to 6.7)
Tracheal, bronchus, and lung cancer	Thailand	2015	Age-standardized		14.6% (8.4 to 21.5%)	5.1 (2.8 to 7.9)
Cardiovascular diseases	Thailand	1990	All Ages	10.9 (9.2 to 12.6)	12.3% (10.6 to 14.1%)	19.2 (16.2 to 22.3)
Cardiovascular diseases	Thailand	1995	All Ages	13.6 (11.5 to 15.5)	12.5% (10.8 to 14.3%)	22.9 (19.4 to 26.2)
Cardiovascular diseases	Thailand	2000	All Ages	14.2 (12.0 to 16.5)	12.7% (10.9 to 14.5%)	22.6 (19.1 to 26.3)
Cardiovascular diseases	Thailand	2005	All Ages	12.4 (10.3 to 14.7)	12.6% (10.8 to 14.4%)	18.8 (15.7 to 22.4)
Cardiovascular diseases	Thailand	2010	All Ages	12.7 (10.4 to 15.6)	11.8% (9.9 to 13.6%)	19.1 (15.6 to 23.4)
Cardiovascular diseases	Thailand	2015	All Ages	16.6 (12.9 to 20.3)	12.3% (10.3 to 14.4%)	24.5 (19.0 to 30.0)
Cardiovascular diseases	Thailand	1990	Age-standardized		11.7% (9.6 to 13.6%)	36.2 (29.7 to 42.9)
Cardiovascular diseases	Thailand	1995	Age-standardized		11.7% (9.7 to 13.7%)	37.7 (31.2 to 44.4)
Cardiovascular diseases	Thailand	2000	Age-standardized		11.8% (9.8 to 13.9%)	32.7 (26.9 to 39.2)
Cardiovascular diseases	Thailand	2005	Age-standardized		11.8% (9.9 to 13.9%)	23.7 (19.1 to 28.9)
Cardiovascular diseases	Thailand	2010	Age-standardized		11.2% (9.3 to 13.2%)	20.2 (16.2 to 25.0)
Cardiovascular diseases	Thailand	2015	Age-standardized		11.9% (9.9 to 14.0%)	22.1 (17.0 to 27.3)
Ischemic heart disease	Thailand	1990	All Ages	6.8 (5.6 to 8.1)	17.5% (14.6 to 20.5%)	12.0 (9.8 to 14.3)
Ischemic heart disease	Thailand	1995	All Ages	9.0 (7.4 to 10.7)	17.2% (14.3 to 20.2%)	15.1 (12.4 to 18.0)
Ischemic heart disease	Thailand	2000	All Ages	9.3 (7.5 to 11.1)	16.6% (13.6 to 19.7%)	14.7 (11.9 to 17.7)
Ischemic heart disease	Thailand	2005	All Ages	7.4 (5.9 to 9.1)	16.4% (13.3 to 19.6%)	11.3 (8.9 to 13.9)
Ischemic heart disease	Thailand	2010	All Ages	7.5 (5.8 to 9.5)	15.5% (12.3 to 18.7%)	11.2 (8.7 to 14.3)
Ischemic heart disease	Thailand	2015	All Ages	9.8 (7.3 to 12.7)	16.0% (12.7 to 19.4%)	14.5 (10.7 to 18.6)
Ischemic heart disease	Thailand	1990	Age-standardized		16.0% (12.9 to 19.1%)	21.5 (16.9 to 26.1)
Ischemic heart disease	Thailand	1995	Age-standardized		15.6% (12.3 to 18.9%)	24.6 (19.3 to 30.4)
Ischemic heart disease	Thailand	2000	Age-standardized		15.3% (11.8 to 18.8%)	21.5 (16.7 to 26.7)
Ischemic heart disease	Thailand	2005	Age-standardized		15.4% (11.8 to 19.0%)	14.3 (10.9 to 18.2)
Ischemic heart disease	Thailand	2010	Age-standardized		14.8% (11.3 to 18.3%)	11.9 (9.0 to 15.5)

Cause name	Location	Year	Age group	Number (in thousands)	PAF	Rate (per 100,000)
Ischemic heart disease	Thailand	2015	Age-standardized		15.5% (12.0 to 19.1%)	13.1 (9.6 to 17.2)
Ischemic stroke	Thailand	1990	All Ages	0.8 (0.5 to 1.1)	11.0% (7.9 to 14.4%)	1.4 (0.9 to 1.9)
Ischemic stroke	Thailand	1995	All Ages	0.9 (0.6 to 1.3)	11.2% (8.1 to 14.6%)	1.5 (1.1 to 2.1)
Ischemic stroke	Thailand	2000	All Ages	1.2 (0.9 to 1.6)	11.5% (8.4 to 14.7%)	2.0 (1.4 to 2.6)
Ischemic stroke	Thailand	2005	All Ages	1.4 (1.0 to 1.8)	11.7% (8.5 to 14.9%)	2.1 (1.5 to 2.7)
Ischemic stroke	Thailand	2010	All Ages	1.5 (1.1 to 2.1)	11.0% (7.8 to 14.4%)	2.3 (1.6 to 3.1)
Ischemic stroke	Thailand	2015	All Ages	2.0 (1.3 to 2.8)	11.6% (8.2 to 15.1%)	2.9 (2.0 to 4.1)
Ischemic stroke	Thailand	1990	Age-standardized		10.5% (7.0 to 14.6%)	3.1 (2.0 to 4.6)
Ischemic stroke	Thailand	1995	Age-standardized		10.6% (7.1 to 14.7%)	3.1 (2.0 to 4.5)
Ischemic stroke	Thailand	2000	Age-standardized		10.8% (7.4 to 14.6%)	3.2 (2.2 to 4.5)
Ischemic stroke	Thailand	2005	Age-standardized		11.1% (7.8 to 14.8%)	2.8 (1.9 to 4.0)
Ischemic stroke	Thailand	2010	Age-standardized		10.7% (7.3 to 14.3%)	2.5 (1.7 to 3.6)
Ischemic stroke	Thailand	2015	Age-standardized		11.3% (7.8 to 15.1%)	2.8 (1.8 to 3.9)
Hemorrhagic stroke	Thailand	1990	All Ages	3.3 (2.6 to 4.1)	12.1% (9.6 to 14.9%)	5.9 (4.6 to 7.3)
Hemorrhagic stroke	Thailand	1995	All Ages	3.7 (2.9 to 4.4)	12.9% (10.3 to 15.5%)	6.2 (4.9 to 7.5)
Hemorrhagic stroke	Thailand	2000	All Ages	3.7 (2.9 to 4.5)	13.2% (10.6 to 15.8%)	5.9 (4.6 to 7.2)
Hemorrhagic stroke	Thailand	2005	All Ages	3.6 (2.8 to 4.5)	13.5% (10.8 to 16.2%)	5.5 (4.3 to 6.8)
Hemorrhagic stroke	Thailand	2010	All Ages	3.7 (2.9 to 4.8)	12.7% (9.9 to 15.5%)	5.6 (4.3 to 7.2)
Hemorrhagic stroke	Thailand	2015	All Ages	4.8 (3.5 to 6.2)	13.2% (10.3 to 16.1%)	7.1 (5.2 to 9.2)
Hemorrhagic stroke	Thailand	1990	Age-standardized		11.4% (8.5 to 14.6%)	11.5 (8.4 to 14.8)
Hemorrhagic stroke	Thailand	1995	Age-standardized		11.9% (9.1 to 14.9%)	10.0 (7.5 to 12.6)
Hemorrhagic stroke	Thailand	2000	Age-standardized		12.2% (9.3 to 15.2%)	8.0 (6.1 to 10.1)
Hemorrhagic stroke	Thailand	2005	Age-standardized		12.6% (9.8 to 15.6%)	6.5 (4.9 to 8.3)
Hemorrhagic stroke	Thailand	2010	Age-standardized		12.0% (9.2 to 15.0%)	5.7 (4.2 to 7.4)
Hemorrhagic stroke	Thailand	2015	Age-standardized		12.7% (9.8 to 15.8%)	6.2 (4.5 to 8.1)
Chronic obstructive pulmonary disease	Thailand	1990	All Ages	1.4 (0.7 to 2.2)	19.4% (9.5 to 30.1%)	2.5 (1.2 to 3.8)
Chronic obstructive pulmonary disease	Thailand	1995	All Ages	2.5 (1.2 to 3.8)	19.7% (9.8 to 30.4%)	4.1 (2.0 to 6.4)
Chronic obstructive pulmonary disease	Thailand	2000	All Ages	3.1 (1.5 to 4.8)	19.9% (9.8 to 30.8%)	4.9 (2.4 to 7.7)
Chronic obstructive pulmonary disease	Thailand	2005	All Ages	2.8 (1.3 to 4.3)	20.4% (10.2 to 31.4%)	4.2 (2.0 to 6.6)
Chronic obstructive pulmonary disease	Thailand	2010	All Ages	3.3 (1.6 to 5.2)	19.3% (9.4 to 30.3%)	5.0 (2.4 to 7.8)
Chronic obstructive pulmonary disease	Thailand	2015	All Ages	4.8 (2.4 to 7.4)	21.2% (10.8 to 32.2%)	7.0 (3.5 to 10.9)
Chronic obstructive pulmonary disease	Thailand	1990	Age-standardized		19.7% (9.7 to 30.6%)	5.2 (2.5 to 8.1)
Chronic obstructive pulmonary disease	Thailand	1995	Age-standardized		19.9% (9.8 to 30.7%)	7.7 (3.8 to 11.9)
Chronic obstructive pulmonary disease	Thailand	2000	Age-standardized		19.9% (9.9 to 30.9%)	8.0 (3.9 to 12.4)
Chronic obstructive pulmonary disease	Thailand	2005	Age-standardized		20.5% (10.2 to 31.5%)	5.9 (2.8 to 9.2)
Chronic obstructive pulmonary disease	Thailand	2010	Age-standardized		19.3% (9.4 to 30.3%)	5.7 (2.7 to 9.0)

Cause name	Location	Year	Age group	Number (in thousands)	PAF	Rate (per 100,000)
Chronic obstructive pulmonary disease	Thailand	2015	Age-standardized		21.2% (10.8 to 32.2%)	6.7 (3.4 to 10.4)
All causes	Timor-Leste	1990	All Ages	0.3 (0.2 to 0.5)	3.9% (2.1 to 6.2%)	40.2 (21.1 to 64.4)
All causes	Timor-Leste	1995	All Ages	0.3 (0.2 to 0.5)	3.9% (2.3 to 6.0%)	34.4 (20.5 to 52.9)
All causes	Timor-Leste	2000	All Ages	0.3 (0.2 to 0.4)	3.5% (2.1 to 5.3%)	32.1 (19.3 to 48.7)
All causes	Timor-Leste	2005	All Ages	0.3 (0.2 to 0.4)	4.3% (3.1 to 5.9%)	25.5 (17.6 to 35.3)
All causes	Timor-Leste	2010	All Ages	0.3 (0.2 to 0.4)	4.5% (3.4 to 6.0%)	24.8 (17.9 to 33.7)
All causes	Timor-Leste	2015	All Ages	0.3 (0.2 to 0.4)	4.7% (3.5 to 6.0%)	23.1 (16.7 to 31.0)
All causes	Timor-Leste	1990	Age-standardized		5.4% (4.1 to 7.1%)	79.4 (57.8 to 104.7)
All causes	Timor-Leste	1995	Age-standardized		5.5% (4.2 to 7.0%)	71.7 (54.0 to 91.7)
All causes	Timor-Leste	2000	Age-standardized		5.0% (3.8 to 6.4%)	65.9 (50.6 to 85.3)
All causes	Timor-Leste	2005	Age-standardized		5.8% (4.5 to 7.2%)	60.5 (45.5 to 77.5)
All causes	Timor-Leste	2010	Age-standardized		5.7% (4.4 to 7.0%)	49.3 (37.0 to 64.5)
All causes	Timor-Leste	2015	Age-standardized		5.7% (4.5 to 7.1%)	46.9 (34.5 to 61.3)
Lower respiratory infections	Timor-Leste	1990	All Ages	0.2 (0.1 to 0.4)	15.6% (8.1 to 25.3%)	26.8 (8.9 to 50.3)
Lower respiratory infections	Timor-Leste	1995	All Ages	0.2 (0.1 to 0.3)	15.6% (8.1 to 25.4%)	20.9 (7.7 to 39.4)
Lower respiratory infections	Timor-Leste	2000	All Ages	0.2 (0.1 to 0.3)	16.0% (8.7 to 25.4%)	19.0 (7.5 to 34.7)
Lower respiratory infections	Timor-Leste	2005	All Ages	0.1 (0.1 to 0.2)	15.8% (8.6 to 25.4%)	12.5 (5.7 to 21.8)
Lower respiratory infections	Timor-Leste	2010	All Ages	0.1 (0.1 to 0.2)	15.1% (7.8 to 24.4%)	9.5 (4.4 to 17.0)
Lower respiratory infections	Timor-Leste	2015	All Ages	0.1 (0.0 to 0.2)	15.0% (7.7 to 24.8%)	7.3 (3.3 to 13.1)
Lower respiratory infections	Timor-Leste	1990	Age-standardized		15.6% (8.1 to 25.3%)	26.5 (12.5 to 47.3)
Lower respiratory infections	Timor-Leste	1995	Age-standardized		15.6% (8.1 to 25.4%)	21.8 (10.3 to 37.6)
Lower respiratory infections	Timor-Leste	2000	Age-standardized		16.0% (8.7 to 25.4%)	19.7 (9.4 to 34.0)
Lower respiratory infections	Timor-Leste	2005	Age-standardized		15.8% (8.6 to 25.4%)	16.4 (8.1 to 28.2)
Lower respiratory infections	Timor-Leste	2010	Age-standardized		15.1% (7.8 to 24.4%)	12.3 (5.7 to 21.5)
Lower respiratory infections	Timor-Leste	2015	Age-standardized		15.0% (7.7 to 24.8%)	10.8 (5.0 to 19.1)
Tracheal, bronchus, and lung cancer	Timor-Leste	1990	All Ages	0.0 (0.0 to 0.0)	10.8% (5.8 to 16.8%)	0.4 (0.2 to 0.7)
Tracheal, bronchus, and lung cancer	Timor-Leste	1995	All Ages	0.0 (0.0 to 0.0)	10.8% (5.8 to 16.9%)	0.4 (0.2 to 0.8)
Tracheal, bronchus, and lung cancer	Timor-Leste	2000	All Ages	0.0 (0.0 to 0.0)	11.1% (5.7 to 17.2%)	0.4 (0.2 to 0.8)
Tracheal, bronchus, and lung cancer	Timor-Leste	2005	All Ages	0.0 (0.0 to 0.0)	10.9% (5.6 to 17.1%)	0.5 (0.2 to 0.8)
Tracheal, bronchus, and lung cancer	Timor-Leste	2010	All Ages	0.0 (0.0 to 0.0)	10.4% (5.4 to 16.5%)	0.6 (0.3 to 1.2)
Tracheal, bronchus, and lung cancer	Timor-Leste	2015	All Ages	0.0 (0.0 to 0.0)	10.3% (5.4 to 16.4%)	0.6 (0.3 to 1.2)
Tracheal, bronchus, and lung cancer	Timor-Leste	1990	Age-standardized		10.9% (5.8 to 17.0%)	1.3 (0.7 to 2.3)
Tracheal, bronchus, and lung cancer	Timor-Leste	1995	Age-standardized		10.9% (5.9 to 17.0%)	1.4 (0.7 to 2.4)
Tracheal, bronchus, and lung cancer	Timor-Leste	2000	Age-standardized		11.1% (5.7 to 17.2%)	1.2 (0.6 to 2.4)
Tracheal, bronchus, and lung cancer	Timor-Leste	2005	Age-standardized		11.0% (5.7 to 17.2%)	1.3 (0.6 to 2.4)
Tracheal, bronchus, and lung cancer	Timor-Leste	2010	Age-standardized		10.5% (5.5 to 16.5%)	1.3 (0.6 to 2.4)

Cause name	Location	Year	Age group	Number (in thousands)	PAF	Rate (per 100,000)
Tracheal, bronchus, and lung cancer	Timor-Leste	2015	Age-standardized		10.4% (5.4 to 16.4%)	1.3 (0.6 to 2.4)
Cardiovascular diseases	Timor-Leste	1990	All Ages	0.1 (0.1 to 0.1)	10.9% (9.0 to 13.0%)	11.8 (8.9 to 15.3)
Cardiovascular diseases	Timor-Leste	1995	All Ages	0.1 (0.1 to 0.1)	11.1% (9.2 to 13.0%)	11.8 (9.0 to 14.8)
Cardiovascular diseases	Timor-Leste	2000	All Ages	0.1 (0.1 to 0.1)	11.0% (9.0 to 13.0%)	11.2 (8.3 to 14.3)
Cardiovascular diseases	Timor-Leste	2005	All Ages	0.1 (0.1 to 0.1)	11.0% (9.0 to 13.1%)	11.0 (8.3 to 14.2)
Cardiovascular diseases	Timor-Leste	2010	All Ages	0.1 (0.1 to 0.2)	10.7% (8.6 to 12.6%)	12.7 (9.2 to 17.1)
Cardiovascular diseases	Timor-Leste	2015	All Ages	0.2 (0.1 to 0.2)	10.4% (8.5 to 12.5%)	13.1 (9.5 to 17.5)
Cardiovascular diseases	Timor-Leste	1990	Age-standardized		10.2% (8.3 to 12.2%)	44.7 (33.9 to 58.4)
Cardiovascular diseases	Timor-Leste	1995	Age-standardized		10.3% (8.3 to 12.3%)	41.7 (31.0 to 53.7)
Cardiovascular diseases	Timor-Leste	2000	Age-standardized		10.3% (8.4 to 12.3%)	38.0 (28.2 to 48.6)
Cardiovascular diseases	Timor-Leste	2005	Age-standardized		10.2% (8.1 to 12.3%)	36.1 (27.2 to 46.4)
Cardiovascular diseases	Timor-Leste	2010	Age-standardized		10.0% (8.0 to 12.0%)	30.0 (22.0 to 40.1)
Cardiovascular diseases	Timor-Leste	2015	Age-standardized		9.8% (7.7 to 11.9%)	29.2 (20.9 to 38.9)
Ischemic heart disease	Timor-Leste	1990	All Ages	0.1 (0.0 to 0.1)	16.2% (13.3 to 19.4%)	7.1 (5.3 to 9.3)
Ischemic heart disease	Timor-Leste	1995	All Ages	0.1 (0.1 to 0.1)	16.1% (13.1 to 19.1%)	7.1 (5.4 to 9.0)
Ischemic heart disease	Timor-Leste	2000	All Ages	0.1 (0.0 to 0.1)	15.7% (12.7 to 18.8%)	6.7 (4.9 to 8.8)
Ischemic heart disease	Timor-Leste	2005	All Ages	0.1 (0.1 to 0.1)	15.3% (12.2 to 18.4%)	6.6 (4.9 to 8.5)
Ischemic heart disease	Timor-Leste	2010	All Ages	0.1 (0.1 to 0.1)	14.4% (11.4 to 17.4%)	7.8 (5.5 to 10.6)
Ischemic heart disease	Timor-Leste	2015	All Ages	0.1 (0.1 to 0.1)	13.8% (10.9 to 16.9%)	8.1 (5.7 to 11.0)
Ischemic heart disease	Timor-Leste	1990	Age-standardized		13.5% (10.5 to 16.5%)	26.7 (19.8 to 35.1)
Ischemic heart disease	Timor-Leste	1995	Age-standardized		13.5% (10.4 to 16.4%)	25.2 (18.5 to 32.9)
Ischemic heart disease	Timor-Leste	2000	Age-standardized		13.5% (10.5 to 16.4%)	23.0 (16.8 to 29.9)
Ischemic heart disease	Timor-Leste	2005	Age-standardized		13.3% (10.2 to 16.6%)	21.6 (15.7 to 28.3)
Ischemic heart disease	Timor-Leste	2010	Age-standardized		13.0% (9.9 to 16.1%)	18.5 (13.0 to 25.0)
Ischemic heart disease	Timor-Leste	2015	Age-standardized		12.7% (9.4 to 15.9%)	18.0 (12.6 to 24.7)
Ischemic stroke	Timor-Leste	1990	All Ages	0.0 (0.0 to 0.0)	10.6% (7.8 to 13.5%)	1.0 (0.7 to 1.7)
Ischemic stroke	Timor-Leste	1995	All Ages	0.0 (0.0 to 0.0)	10.6% (7.8 to 13.7%)	1.1 (0.7 to 1.6)
Ischemic stroke	Timor-Leste	2000	All Ages	0.0 (0.0 to 0.0)	10.5% (7.7 to 13.5%)	1.1 (0.7 to 1.7)
Ischemic stroke	Timor-Leste	2005	All Ages	0.0 (0.0 to 0.0)	10.3% (7.5 to 13.4%)	1.2 (0.8 to 1.7)
Ischemic stroke	Timor-Leste	2010	All Ages	0.0 (0.0 to 0.0)	9.8% (6.9 to 13.0%)	1.4 (0.9 to 2.1)
Ischemic stroke	Timor-Leste	2015	All Ages	0.0 (0.0 to 0.0)	9.5% (6.3 to 12.8%)	1.5 (1.0 to 2.3)
Ischemic stroke	Timor-Leste	1990	Age-standardized		9.3% (6.1 to 12.6%)	5.9 (3.4 to 9.5)
Ischemic stroke	Timor-Leste	1995	Age-standardized		9.3% (6.2 to 12.7%)	5.5 (3.3 to 8.8)
Ischemic stroke	Timor-Leste	2000	Age-standardized		9.4% (6.4 to 12.7%)	5.2 (3.0 to 8.2)
Ischemic stroke	Timor-Leste	2005	Age-standardized		9.3% (6.1 to 12.7%)	5.1 (3.1 to 7.9)
Ischemic stroke	Timor-Leste	2010	Age-standardized		9.0% (6.0 to 12.5%)	4.0 (2.4 to 6.1)

Cause name	Location	Year	Age group	Number (in thousands)	PAF	Rate (per 100,000)
Ischemic stroke	Timor-Leste	2015	Age-standardized		8.8% (5.5 to 12.3%)	3.9 (2.3 to 6.2)
Hemorrhagic stroke	Timor-Leste	1990	All Ages	0.0 (0.0 to 0.0)	11.2% (8.5 to 14.2%)	3.7 (2.4 to 5.1)
Hemorrhagic stroke	Timor-Leste	1995	All Ages	0.0 (0.0 to 0.0)	11.4% (8.6 to 14.2%)	3.6 (2.4 to 4.9)
Hemorrhagic stroke	Timor-Leste	2000	All Ages	0.0 (0.0 to 0.0)	11.2% (8.6 to 14.3%)	3.3 (2.1 to 4.6)
Hemorrhagic stroke	Timor-Leste	2005	All Ages	0.0 (0.0 to 0.0)	11.2% (8.5 to 14.2%)	3.2 (2.1 to 4.4)
Hemorrhagic stroke	Timor-Leste	2010	All Ages	0.0 (0.0 to 0.1)	10.7% (7.9 to 13.6%)	3.4 (2.1 to 4.9)
Hemorrhagic stroke	Timor-Leste	2015	All Ages	0.0 (0.0 to 0.1)	10.5% (7.7 to 13.5%)	3.5 (2.2 to 5.0)
Hemorrhagic stroke	Timor-Leste	1990	Age-standardized		10.5% (7.8 to 13.3%)	12.1 (7.8 to 16.9)
Hemorrhagic stroke	Timor-Leste	1995	Age-standardized		10.5% (7.7 to 13.4%)	11.0 (7.1 to 15.2)
Hemorrhagic stroke	Timor-Leste	2000	Age-standardized		10.6% (7.9 to 13.4%)	9.9 (6.2 to 13.7)
Hemorrhagic stroke	Timor-Leste	2005	Age-standardized		10.5% (7.9 to 13.3%)	9.5 (6.0 to 13.1)
Hemorrhagic stroke	Timor-Leste	2010	Age-standardized		10.1% (7.5 to 13.1%)	7.5 (4.7 to 10.8)
Hemorrhagic stroke	Timor-Leste	2015	Age-standardized		9.9% (7.1 to 12.9%)	7.3 (4.6 to 10.4)
Chronic obstructive pulmonary disease	Timor-Leste	1990	All Ages	0.0 (0.0 to 0.0)	8.8% (2.8 to 17.4%)	1.2 (0.4 to 2.3)
Chronic obstructive pulmonary disease	Timor-Leste	1995	All Ages	0.0 (0.0 to 0.0)	10.2% (3.3 to 18.9%)	1.3 (0.4 to 2.5)
Chronic obstructive pulmonary disease	Timor-Leste	2000	All Ages	0.0 (0.0 to 0.0)	11.5% (4.3 to 20.6%)	1.5 (0.6 to 2.8)
Chronic obstructive pulmonary disease	Timor-Leste	2005	All Ages	0.0 (0.0 to 0.0)	13.0% (5.7 to 22.3%)	1.5 (0.6 to 2.8)
Chronic obstructive pulmonary disease	Timor-Leste	2010	All Ages	0.0 (0.0 to 0.0)	13.9% (6.5 to 23.1%)	2.0 (0.9 to 3.6)
Chronic obstructive pulmonary disease	Timor-Leste	2015	All Ages	0.0 (0.0 to 0.1)	14.5% (6.6 to 23.7%)	2.2 (0.9 to 3.8)
Chronic obstructive pulmonary disease	Timor-Leste	1990	Age-standardized		15.4% (7.0 to 25.0%)	6.9 (2.1 to 13.3)
Chronic obstructive pulmonary disease	Timor-Leste	1995	Age-standardized		15.7% (7.1 to 25.6%)	6.9 (2.3 to 12.8)
Chronic obstructive pulmonary disease	Timor-Leste	2000	Age-standardized		16.1% (7.6 to 25.7%)	6.9 (2.7 to 12.8)
Chronic obstructive pulmonary disease	Timor-Leste	2005	Age-standardized		16.2% (7.4 to 26.3%)	6.7 (2.7 to 12.2)
Chronic obstructive pulmonary disease	Timor-Leste	2010	Age-standardized		15.7% (7.5 to 25.5%)	5.7 (2.5 to 10.2)
Chronic obstructive pulmonary disease	Timor-Leste	2015	Age-standardized		15.7% (7.4 to 25.5%)	5.6 (2.3 to 9.8)
All causes	Vietnam	1990	All Ages	26.3 (21.3 to 32.3)	5.5% (4.6 to 6.6%)	38.6 (31.2 to 47.3)
All causes	Vietnam	1995	All Ages	29.0 (23.0 to 35.6)	5.8% (4.9 to 6.9%)	38.7 (30.6 to 47.4)
All causes	Vietnam	2000	All Ages	31.9 (25.8 to 38.4)	6.2% (5.3 to 7.3%)	39.6 (32.1 to 47.7)
All causes	Vietnam	2005	All Ages	35.8 (27.9 to 44.9)	6.6% (5.6 to 7.8%)	42.5 (33.1 to 53.2)
All causes	Vietnam	2010	All Ages	37.0 (28.4 to 45.8)	6.4% (5.4 to 7.6%)	41.9 (32.1 to 51.8)
All causes	Vietnam	2015	All Ages	42.2 (32.0 to 52.3)	6.8% (5.6 to 8.0%)	45.2 (34.3 to 55.9)
All causes	Vietnam	1990	Age-standardized		6.1% (5.2 to 7.3%)	63.0 (51.1 to 77.5)
All causes	Vietnam	1995	Age-standardized		6.4% (5.4 to 7.5%)	62.8 (49.6 to 77.3)
All causes	Vietnam	2000	Age-standardized		6.6% (5.6 to 7.7%)	61.8 (50.1 to 74.6)
All causes	Vietnam	2005	Age-standardized		6.9% (5.9 to 8.1%)	60.8 (47.0 to 75.9)
All causes	Vietnam	2010	Age-standardized		6.7% (5.6 to 7.9%)	55.0 (42.1 to 68.1)

Cause name	Location	Year	Age group	Number (in thousands)	PAF	Rate (per 100,000)
All causes	Vietnam	2015	Age-standardized		7.0% (5.8 to 8.2%)	54.7 (41.6 to 67.1)
Lower respiratory infections	Vietnam	1990	All Ages	6.0 (3.2 to 9.6)	20.7% (12.6 to 30.3%)	8.7 (4.6 to 14.1)
Lower respiratory infections	Vietnam	1995	All Ages	5.0 (2.8 to 7.7)	20.8% (12.7 to 30.3%)	6.6 (3.7 to 10.2)
Lower respiratory infections	Vietnam	2000	All Ages	4.3 (2.6 to 6.7)	20.9% (12.7 to 30.5%)	5.4 (3.2 to 8.3)
Lower respiratory infections	Vietnam	2005	All Ages	4.7 (2.9 to 7.1)	22.1% (14.0 to 31.6%)	5.6 (3.4 to 8.4)
Lower respiratory infections	Vietnam	2010	All Ages	4.6 (2.7 to 6.9)	20.7% (12.5 to 30.3%)	5.2 (3.1 to 7.8)
Lower respiratory infections	Vietnam	2015	All Ages	4.9 (2.8 to 7.5)	21.5% (13.5 to 31.1%)	5.2 (3.0 to 8.0)
Lower respiratory infections	Vietnam	1990	Age-standardized		20.7% (12.6 to 30.3%)	9.6 (5.4 to 15.1)
Lower respiratory infections	Vietnam	1995	Age-standardized		20.8% (12.7 to 30.3%)	8.3 (4.7 to 12.8)
Lower respiratory infections	Vietnam	2000	Age-standardized		20.9% (12.7 to 30.5%)	7.4 (4.4 to 11.6)
Lower respiratory infections	Vietnam	2005	Age-standardized		22.1% (14.0 to 31.6%)	7.4 (4.6 to 11.1)
Lower respiratory infections	Vietnam	2010	Age-standardized		20.7% (12.5 to 30.3%)	6.4 (3.8 to 9.7)
Lower respiratory infections	Vietnam	2015	Age-standardized		21.5% (13.5 to 31.1%)	6.1 (3.5 to 9.4)
Tracheal, bronchus, and lung cancer	Vietnam	1990	All Ages	2.1 (1.2 to 3.1)	14.6% (8.5 to 21.3%)	3.0 (1.7 to 4.6)
Tracheal, bronchus, and lung cancer	Vietnam	1995	All Ages	2.5 (1.4 to 3.9)	14.7% (8.5 to 21.6%)	3.3 (1.9 to 5.2)
Tracheal, bronchus, and lung cancer	Vietnam	2000	All Ages	3.1 (1.8 to 4.8)	14.7% (8.6 to 21.6%)	3.9 (2.2 to 5.9)
Tracheal, bronchus, and lung cancer	Vietnam	2005	All Ages	3.9 (2.1 to 6.0)	15.6% (9.1 to 22.6%)	4.6 (2.5 to 7.1)
Tracheal, bronchus, and lung cancer	Vietnam	2010	All Ages	4.2 (2.3 to 6.5)	14.6% (8.5 to 21.3%)	4.7 (2.6 to 7.3)
Tracheal, bronchus, and lung cancer	Vietnam	2015	All Ages	5.3 (2.9 to 8.0)	15.2% (8.9 to 22.2%)	5.6 (3.1 to 8.6)
Tracheal, bronchus, and lung cancer	Vietnam	1990	Age-standardized		14.6% (8.5 to 21.4%)	5.2 (2.9 to 7.9)
Tracheal, bronchus, and lung cancer	Vietnam	1995	Age-standardized		14.7% (8.5 to 21.7%)	5.6 (3.2 to 8.7)
Tracheal, bronchus, and lung cancer	Vietnam	2000	Age-standardized		14.8% (8.6 to 21.7%)	6.3 (3.6 to 9.5)
Tracheal, bronchus, and lung cancer	Vietnam	2005	Age-standardized		15.7% (9.2 to 22.7%)	6.9 (3.8 to 10.6)
Tracheal, bronchus, and lung cancer	Vietnam	2010	Age-standardized		14.6% (8.5 to 21.4%)	6.5 (3.6 to 10.0)
Tracheal, bronchus, and lung cancer	Vietnam	2015	Age-standardized		15.2% (9.0 to 22.3%)	7.0 (3.8 to 10.6)
Cardiovascular diseases	Vietnam	1990	All Ages	15.0 (12.0 to 18.8)	12.0% (10.0 to 14.1%)	22.0 (17.5 to 27.6)
Cardiovascular diseases	Vietnam	1995	All Ages	17.6 (13.4 to 22.2)	12.0% (9.9 to 14.0%)	23.4 (17.8 to 29.6)
Cardiovascular diseases	Vietnam	2000	All Ages	19.9 (15.6 to 25.1)	11.9% (9.8 to 13.9%)	24.7 (19.4 to 31.2)
Cardiovascular diseases	Vietnam	2005	All Ages	22.1 (16.5 to 28.7)	12.0% (9.9 to 14.3%)	26.2 (19.6 to 34.0)
Cardiovascular diseases	Vietnam	2010	All Ages	23.1 (16.9 to 29.7)	11.5% (9.2 to 13.7%)	26.1 (19.1 to 33.6)
Cardiovascular diseases	Vietnam	2015	All Ages	26.0 (19.3 to 33.1)	11.7% (9.4 to 14.1%)	27.9 (20.6 to 35.5)
Cardiovascular diseases	Vietnam	1990	Age-standardized		11.9% (9.8 to 13.9%)	39.2 (30.9 to 49.2)
Cardiovascular diseases	Vietnam	1995	Age-standardized		11.9% (9.9 to 14.0%)	39.7 (30.1 to 50.3)
Cardiovascular diseases	Vietnam	2000	Age-standardized		11.8% (9.8 to 14.0%)	39.2 (30.7 to 49.4)
Cardiovascular diseases	Vietnam	2005	Age-standardized		12.1% (9.9 to 14.2%)	37.7 (28.3 to 49.1)
Cardiovascular diseases	Vietnam	2010	Age-standardized		11.5% (9.3 to 13.7%)	34.3 (25.1 to 44.1)

Cause name	Location	Year	Age group	Number (in thousands)	PAF	Rate (per 100,000)
Cardiovascular diseases	Vietnam	2015	Age-standardized		11.7% (9.4 to 13.9%)	33.6 (25.0 to 42.7)
Ischemic heart disease	Vietnam	1990	All Ages	6.9 (5.2 to 8.8)	15.9% (12.5 to 19.2%)	10.1 (7.7 to 12.9)
Ischemic heart disease	Vietnam	1995	All Ages	8.2 (6.0 to 10.9)	15.7% (12.3 to 19.2%)	10.9 (8.0 to 14.5)
Ischemic heart disease	Vietnam	2000	All Ages	9.0 (6.5 to 11.8)	15.5% (12.0 to 19.1%)	11.2 (8.1 to 14.6)
Ischemic heart disease	Vietnam	2005	All Ages	9.5 (6.7 to 12.6)	15.7% (11.8 to 19.6%)	11.2 (8.0 to 14.9)
Ischemic heart disease	Vietnam	2010	All Ages	9.7 (6.8 to 13.1)	15.1% (11.2 to 19.1%)	10.9 (7.6 to 14.8)
Ischemic heart disease	Vietnam	2015	All Ages	11.3 (7.7 to 15.2)	15.3% (11.3 to 19.3%)	12.1 (8.2 to 16.2)
Ischemic heart disease	Vietnam	1990	Age-standardized		15.6% (12.1 to 19.0%)	18.0 (13.5 to 23.1)
Ischemic heart disease	Vietnam	1995	Age-standardized		15.6% (12.0 to 19.1%)	18.5 (13.4 to 24.8)
Ischemic heart disease	Vietnam	2000	Age-standardized		15.5% (12.0 to 19.0%)	17.7 (12.8 to 23.2)
Ischemic heart disease	Vietnam	2005	Age-standardized		15.7% (12.0 to 19.5%)	16.2 (11.5 to 21.4)
Ischemic heart disease	Vietnam	2010	Age-standardized		15.1% (11.3 to 19.0%)	14.4 (10.1 to 19.2)
Ischemic heart disease	Vietnam	2015	Age-standardized		15.2% (11.4 to 19.2%)	14.5 (10.0 to 19.3)
Ischemic stroke	Vietnam	1990	All Ages	2.8 (1.8 to 3.8)	11.4% (8.1 to 14.9%)	4.0 (2.6 to 5.6)
Ischemic stroke	Vietnam	1995	All Ages	3.3 (2.1 to 4.6)	11.3% (8.0 to 15.0%)	4.3 (2.7 to 6.2)
Ischemic stroke	Vietnam	2000	All Ages	3.9 (2.6 to 5.6)	11.2% (7.7 to 15.0%)	4.9 (3.2 to 6.9)
Ischemic stroke	Vietnam	2005	All Ages	4.6 (3.0 to 6.8)	11.4% (7.8 to 15.5%)	5.5 (3.5 to 8.0)
Ischemic stroke	Vietnam	2010	All Ages	5.0 (3.1 to 7.3)	10.9% (7.2 to 15.2%)	5.6 (3.5 to 8.2)
Ischemic stroke	Vietnam	2015	All Ages	5.5 (3.5 to 8.2)	11.1% (7.3 to 15.5%)	5.8 (3.7 to 8.7)
Ischemic stroke	Vietnam	1990	Age-standardized		11.2% (7.9 to 14.9%)	7.4 (4.8 to 10.5)
Ischemic stroke	Vietnam	1995	Age-standardized		11.2% (7.9 to 15.0%)	7.5 (4.7 to 10.6)
Ischemic stroke	Vietnam	2000	Age-standardized		11.2% (7.8 to 15.0%)	7.8 (5.1 to 11.1)
Ischemic stroke	Vietnam	2005	Age-standardized		11.5% (7.9 to 15.3%)	8.0 (5.2 to 11.6)
Ischemic stroke	Vietnam	2010	Age-standardized		11.0% (7.4 to 15.0%)	7.5 (4.7 to 10.8)
Ischemic stroke	Vietnam	2015	Age-standardized		11.2% (7.6 to 15.2%)	7.3 (4.7 to 10.5)
Hemorrhagic stroke	Vietnam	1990	All Ages	5.4 (4.0 to 7.1)	12.9% (10.2 to 15.8%)	7.9 (5.8 to 10.4)
Hemorrhagic stroke	Vietnam	1995	All Ages	6.1 (4.4 to 8.1)	12.9% (10.0 to 15.7%)	8.2 (5.9 to 10.8)
Hemorrhagic stroke	Vietnam	2000	All Ages	7.0 (5.3 to 9.4)	12.8% (9.9 to 15.7%)	8.7 (6.5 to 11.7)
Hemorrhagic stroke	Vietnam	2005	All Ages	8.0 (5.7 to 10.8)	13.2% (10.1 to 16.3%)	9.5 (6.8 to 12.8)
Hemorrhagic stroke	Vietnam	2010	All Ages	8.4 (5.8 to 11.6)	12.6% (9.6 to 15.7%)	9.6 (6.6 to 13.2)
Hemorrhagic stroke	Vietnam	2015	All Ages	9.3 (6.5 to 12.5)	12.9% (9.7 to 16.1%)	9.9 (7.0 to 13.3)
Hemorrhagic stroke	Vietnam	1990	Age-standardized		12.8% (9.9 to 15.8%)	13.8 (10.0 to 18.2)
Hemorrhagic stroke	Vietnam	1995	Age-standardized		12.8% (9.9 to 15.7%)	13.7 (9.9 to 18.3)
Hemorrhagic stroke	Vietnam	2000	Age-standardized		12.7% (9.8 to 15.7%)	13.7 (10.2 to 18.4)
Hemorrhagic stroke	Vietnam	2005	Age-standardized		13.1% (10.0 to 16.1%)	13.6 (9.7 to 18.2)
Hemorrhagic stroke	Vietnam	2010	Age-standardized		12.4% (9.4 to 15.5%)	12.5 (8.6 to 17.0)

Cause name	Location	Year	Age group	Number (in thousands)	PAF	Rate (per 100,000)
Hemorrhagic stroke	Vietnam	2015	Age-standardized		12.7% (9.6 to 15.8%)	11.9 (8.3 to 15.9)
Chronic obstructive pulmonary disease	Vietnam	1990	All Ages	3.3 (1.6 to 5.3)	20.6% (10.7 to 31.1%)	4.8 (2.3 to 7.8)
Chronic obstructive pulmonary disease	Vietnam	1995	All Ages	4.0 (1.9 to 6.3)	21.0% (10.9 to 31.7%)	5.3 (2.5 to 8.4)
Chronic obstructive pulmonary disease	Vietnam	2000	All Ages	4.5 (2.2 to 7.2)	21.2% (11.0 to 32.0%)	5.6 (2.8 to 8.9)
Chronic obstructive pulmonary disease	Vietnam	2005	All Ages	5.1 (2.6 to 8.0)	22.2% (11.7 to 33.0%)	6.0 (3.1 to 9.5)
Chronic obstructive pulmonary disease	Vietnam	2010	All Ages	5.2 (2.5 to 8.1)	21.1% (10.9 to 32.0%)	5.9 (2.8 to 9.2)
Chronic obstructive pulmonary disease	Vietnam	2015	All Ages	6.0 (3.0 to 9.6)	21.7% (11.4 to 32.8%)	6.5 (3.2 to 10.3)
Chronic obstructive pulmonary disease	Vietnam	1990	Age-standardized		21.0% (10.9 to 31.7%)	9.0 (4.2 to 14.6)
Chronic obstructive pulmonary disease	Vietnam	1995	Age-standardized		21.2% (11.0 to 32.0%)	9.1 (4.4 to 14.6)
Chronic obstructive pulmonary disease	Vietnam	2000	Age-standardized		21.3% (11.0 to 32.1%)	8.9 (4.5 to 14.3)
Chronic obstructive pulmonary disease	Vietnam	2005	Age-standardized		22.2% (11.7 to 33.0%)	8.8 (4.6 to 13.8)
Chronic obstructive pulmonary disease	Vietnam	2010	Age-standardized		21.1% (10.9 to 32.0%)	7.8 (3.8 to 12.2)
Chronic obstructive pulmonary disease	Vietnam	2015	Age-standardized		21.7% (11.4 to 32.8%)	7.9 (4.0 to 12.7)
All causes	American Samoa	1990	All Ages	0.0 (0.0 to 0.0)	1.0% (0.0 to 2.8%)	5.1 (0.2 to 13.6)
All causes	American Samoa	1995	All Ages	0.0 (0.0 to 0.0)	1.0% (0.0 to 2.7%)	4.7 (0.2 to 12.6)
All causes	American Samoa	2000	All Ages	0.0 (0.0 to 0.0)	1.0% (0.0 to 2.7%)	4.6 (0.2 to 12.4)
All causes	American Samoa	2005	All Ages	0.0 (0.0 to 0.0)	1.0% (0.0 to 2.6%)	4.4 (0.2 to 11.2)
All causes	American Samoa	2010	All Ages	0.0 (0.0 to 0.0)	0.4% (0.0 to 1.5%)	1.8 (0.0 to 6.2)
All causes	American Samoa	2015	All Ages	0.0 (0.0 to 0.0)	0.6% (0.0 to 2.2%)	2.6 (0.0 to 8.5)
All causes	American Samoa	1990	Age-standardized		1.1% (0.0 to 3.0%)	12.1 (0.5 to 32.4)
All causes	American Samoa	1995	Age-standardized		1.1% (0.0 to 2.9%)	11.3 (0.4 to 30.3)
All causes	American Samoa	2000	Age-standardized		1.1% (0.0 to 2.9%)	10.7 (0.3 to 29.7)
All causes	American Samoa	2005	Age-standardized		1.1% (0.0 to 2.7%)	10.2 (0.3 to 26.3)
All causes	American Samoa	2010	Age-standardized		0.5% (0.0 to 1.7%)	4.3 (0.0 to 16.2)
All causes	American Samoa	2015	Age-standardized		0.6% (0.0 to 2.2%)	6.0 (0.0 to 21.0)
Lower respiratory infections	American Samoa	1990	All Ages	0.0 (0.0 to 0.0)	1.6% (0.0 to 10.3%)	0.5 (0.0 to 3.2)
Lower respiratory infections	American Samoa	1995	All Ages	0.0 (0.0 to 0.0)	1.5% (0.0 to 9.8%)	0.4 (0.0 to 2.9)
Lower respiratory infections	American Samoa	2000	All Ages	0.0 (0.0 to 0.0)	1.5% (0.0 to 9.3%)	0.4 (0.0 to 2.4)
Lower respiratory infections	American Samoa	2005	All Ages	0.0 (0.0 to 0.0)	1.6% (0.0 to 10.6%)	0.3 (0.0 to 2.4)
Lower respiratory infections	American Samoa	2010	All Ages	0.0 (0.0 to 0.0)	0.7% (0.0 to 6.0%)	0.1 (0.0 to 1.1)
Lower respiratory infections	American Samoa	2015	All Ages	0.0 (0.0 to 0.0)	0.8% (0.0 to 6.3%)	0.1 (0.0 to 1.1)
Lower respiratory infections	American Samoa	1990	Age-standardized		1.6% (0.0 to 10.3%)	1.1 (0.0 to 7.0)
Lower respiratory infections	American Samoa	1995	Age-standardized		1.5% (0.0 to 9.8%)	0.9 (0.0 to 6.3)
Lower respiratory infections	American Samoa	2000	Age-standardized		1.5% (0.0 to 9.3%)	0.9 (0.0 to 5.5)
Lower respiratory infections	American Samoa	2005	Age-standardized		1.6% (0.0 to 10.6%)	0.8 (0.0 to 5.4)
Lower respiratory infections	American Samoa	2010	Age-standardized		0.7% (0.0 to 6.0%)	0.3 (0.0 to 2.9)

Cause name	Location	Year	Age group	Number (in thousands)	PAF	Rate (per 100,000)
Lower respiratory infections	American Samoa	2015	Age-standardized		0.8% (0.0 to 6.3%)	0.4 (0.0 to 2.9)
Tracheal, bronchus, and lung cancer	American Samoa	1990	All Ages	0.0 (0.0 to 0.0)	0.9% (0.0 to 4.3%)	0.1 (0.0 to 0.5)
Tracheal, bronchus, and lung cancer	American Samoa	1995	All Ages	0.0 (0.0 to 0.0)	0.9% (0.0 to 4.3%)	0.1 (0.0 to 0.5)
Tracheal, bronchus, and lung cancer	American Samoa	2000	All Ages	0.0 (0.0 to 0.0)	0.9% (0.0 to 4.2%)	0.1 (0.0 to 0.5)
Tracheal, bronchus, and lung cancer	American Samoa	2005	All Ages	0.0 (0.0 to 0.0)	0.9% (0.0 to 4.0%)	0.1 (0.0 to 0.5)
Tracheal, bronchus, and lung cancer	American Samoa	2010	All Ages	0.0 (0.0 to 0.0)	0.3% (0.0 to 2.6%)	0.0 (0.0 to 0.3)
Tracheal, bronchus, and lung cancer	American Samoa	2015	All Ages	0.0 (0.0 to 0.0)	0.5% (0.0 to 3.3%)	0.1 (0.0 to 0.3)
Tracheal, bronchus, and lung cancer	American Samoa	1990	Age-standardized		0.9% (0.0 to 4.3%)	0.3 (0.0 to 1.3)
Tracheal, bronchus, and lung cancer	American Samoa	1995	Age-standardized		0.9% (0.0 to 4.3%)	0.3 (0.0 to 1.3)
Tracheal, bronchus, and lung cancer	American Samoa	2000	Age-standardized		0.9% (0.0 to 4.2%)	0.2 (0.0 to 1.2)
Tracheal, bronchus, and lung cancer	American Samoa	2005	Age-standardized		0.9% (0.0 to 4.0%)	0.2 (0.0 to 1.1)
Tracheal, bronchus, and lung cancer	American Samoa	2010	Age-standardized		0.3% (0.0 to 2.6%)	0.1 (0.0 to 0.6)
Tracheal, bronchus, and lung cancer	American Samoa	2015	Age-standardized		0.5% (0.0 to 3.3%)	0.1 (0.0 to 0.8)
Cardiovascular diseases	American Samoa	1990	All Ages	0.0 (0.0 to 0.0)	2.7% (0.1 to 6.9%)	3.9 (0.2 to 10.2)
Cardiovascular diseases	American Samoa	1995	All Ages	0.0 (0.0 to 0.0)	2.6% (0.1 to 6.8%)	3.7 (0.2 to 9.8)
Cardiovascular diseases	American Samoa	2000	All Ages	0.0 (0.0 to 0.0)	2.8% (0.1 to 7.0%)	3.7 (0.1 to 9.5)
Cardiovascular diseases	American Samoa	2005	All Ages	0.0 (0.0 to 0.0)	2.8% (0.1 to 7.0%)	3.5 (0.1 to 8.8)
Cardiovascular diseases	American Samoa	2010	All Ages	0.0 (0.0 to 0.0)	1.3% (0.0 to 4.5%)	1.5 (0.0 to 5.2)
Cardiovascular diseases	American Samoa	2015	All Ages	0.0 (0.0 to 0.0)	1.8% (0.0 to 5.9%)	2.2 (0.0 to 6.9)
Cardiovascular diseases	American Samoa	1990	Age-standardized		2.5% (0.1 to 6.5%)	9.3 (0.4 to 24.3)
Cardiovascular diseases	American Samoa	1995	Age-standardized		2.4% (0.1 to 6.2%)	8.7 (0.3 to 22.5)
Cardiovascular diseases	American Samoa	2000	Age-standardized		2.5% (0.1 to 6.4%)	8.4 (0.2 to 22.6)
Cardiovascular diseases	American Samoa	2005	Age-standardized		2.5% (0.1 to 6.3%)	8.1 (0.2 to 20.5)
Cardiovascular diseases	American Samoa	2010	Age-standardized		1.1% (0.0 to 4.1%)	3.5 (0.0 to 12.7)
Cardiovascular diseases	American Samoa	2015	Age-standardized		1.6% (0.0 to 5.2%)	5.0 (0.0 to 16.5)
Ischemic heart disease	American Samoa	1990	All Ages	0.0 (0.0 to 0.0)	4.2% (0.0 to 10.8%)	2.6 (0.0 to 6.8)
Ischemic heart disease	American Samoa	1995	All Ages	0.0 (0.0 to 0.0)	4.1% (0.0 to 10.7%)	2.5 (0.0 to 6.6)
Ischemic heart disease	American Samoa	2000	All Ages	0.0 (0.0 to 0.0)	4.1% (0.0 to 10.6%)	2.6 (0.0 to 6.6)
Ischemic heart disease	American Samoa	2005	All Ages	0.0 (0.0 to 0.0)	4.3% (0.0 to 10.6%)	2.6 (0.0 to 6.6)
Ischemic heart disease	American Samoa	2010	All Ages	0.0 (0.0 to 0.0)	2.0% (0.0 to 7.1%)	1.1 (0.0 to 4.1)
Ischemic heart disease	American Samoa	2015	All Ages	0.0 (0.0 to 0.0)	2.8% (0.0 to 8.4%)	1.6 (0.0 to 5.1)
Ischemic heart disease	American Samoa	1990	Age-standardized		3.8% (0.0 to 9.9%)	6.1 (0.0 to 15.7)
Ischemic heart disease	American Samoa	1995	Age-standardized		3.7% (0.0 to 9.6%)	5.7 (0.0 to 15.1)
Ischemic heart disease	American Samoa	2000	Age-standardized		3.7% (0.0 to 9.6%)	5.7 (0.0 to 15.3)
Ischemic heart disease	American Samoa	2005	Age-standardized		3.8% (0.0 to 9.5%)	5.7 (0.0 to 14.3)
Ischemic heart disease	American Samoa	2010	Age-standardized		1.7% (0.0 to 6.8%)	2.5 (0.0 to 9.9)

Cause name	Location	Year	Age group	Number (in thousands)	PAF	Rate (per 100,000)
Ischemic heart disease	American Samoa	2015	Age-standardized		2.4% (0.0 to 7.7%)	3.6 (0.0 to 12.0)
Ischemic stroke	American Samoa	1990	All Ages	0.0 (0.0 to 0.0)	2.3% (0.0 to 6.9%)	0.4 (0.0 to 1.2)
Ischemic stroke	American Samoa	1995	All Ages	0.0 (0.0 to 0.0)	2.3% (0.0 to 6.7%)	0.4 (0.0 to 1.1)
Ischemic stroke	American Samoa	2000	All Ages	0.0 (0.0 to 0.0)	2.3% (0.0 to 6.5%)	0.3 (0.0 to 1.0)
Ischemic stroke	American Samoa	2005	All Ages	0.0 (0.0 to 0.0)	2.3% (0.0 to 6.7%)	0.3 (0.0 to 0.8)
Ischemic stroke	American Samoa	2010	All Ages	0.0 (0.0 to 0.0)	1.0% (0.0 to 4.2%)	0.1 (0.0 to 0.5)
Ischemic stroke	American Samoa	2015	All Ages	0.0 (0.0 to 0.0)	1.5% (0.0 to 5.4%)	0.2 (0.0 to 0.6)
Ischemic stroke	American Samoa	1990	Age-standardized		2.2% (0.0 to 6.9%)	1.1 (0.0 to 3.5)
Ischemic stroke	American Samoa	1995	Age-standardized		2.1% (0.0 to 6.4%)	1.1 (0.0 to 3.3)
Ischemic stroke	American Samoa	2000	Age-standardized		2.1% (0.0 to 6.4%)	1.0 (0.0 to 3.0)
Ischemic stroke	American Samoa	2005	Age-standardized		2.1% (0.0 to 6.4%)	0.9 (0.0 to 2.7)
Ischemic stroke	American Samoa	2010	Age-standardized		0.9% (0.0 to 4.4%)	0.4 (0.0 to 1.7)
Ischemic stroke	American Samoa	2015	Age-standardized		1.3% (0.0 to 5.2%)	0.5 (0.0 to 2.0)
Hemorrhagic stroke	American Samoa	1990	All Ages	0.0 (0.0 to 0.0)	2.5% (0.0 to 7.0%)	0.9 (0.0 to 2.5)
Hemorrhagic stroke	American Samoa	1995	All Ages	0.0 (0.0 to 0.0)	2.4% (0.0 to 6.9%)	0.8 (0.0 to 2.3)
Hemorrhagic stroke	American Samoa	2000	All Ages	0.0 (0.0 to 0.0)	2.5% (0.0 to 6.9%)	0.8 (0.0 to 2.2)
Hemorrhagic stroke	American Samoa	2005	All Ages	0.0 (0.0 to 0.0)	2.5% (0.0 to 6.9%)	0.7 (0.0 to 1.8)
Hemorrhagic stroke	American Samoa	2010	All Ages	0.0 (0.0 to 0.0)	1.1% (0.0 to 4.5%)	0.3 (0.0 to 1.0)
Hemorrhagic stroke	American Samoa	2015	All Ages	0.0 (0.0 to 0.0)	1.6% (0.0 to 5.8%)	0.4 (0.0 to 1.4)
Hemorrhagic stroke	American Samoa	1990	Age-standardized		2.4% (0.0 to 7.0%)	2.0 (0.0 to 5.9)
Hemorrhagic stroke	American Samoa	1995	Age-standardized		2.3% (0.0 to 6.7%)	1.9 (0.0 to 5.5)
Hemorrhagic stroke	American Samoa	2000	Age-standardized		2.3% (0.0 to 6.4%)	1.7 (0.0 to 5.0)
Hemorrhagic stroke	American Samoa	2005	Age-standardized		2.3% (0.0 to 6.4%)	1.5 (0.0 to 4.2)
Hemorrhagic stroke	American Samoa	2010	Age-standardized		1.0% (0.0 to 4.2%)	0.6 (0.0 to 2.6)
Hemorrhagic stroke	American Samoa	2015	Age-standardized		1.4% (0.0 to 5.3%)	0.9 (0.0 to 3.3)
Chronic obstructive pulmonary disease	American Samoa	1990	All Ages	0.0 (0.0 to 0.0)	2.3% (0.0 to 10.1%)	0.5 (0.0 to 2.4)
Chronic obstructive pulmonary disease	American Samoa	1995	All Ages	0.0 (0.0 to 0.0)	2.2% (0.0 to 9.9%)	0.5 (0.0 to 2.3)
Chronic obstructive pulmonary disease	American Samoa	2000	All Ages	0.0 (0.0 to 0.0)	2.2% (0.0 to 10.7%)	0.4 (0.0 to 2.0)
Chronic obstructive pulmonary disease	American Samoa	2005	All Ages	0.0 (0.0 to 0.0)	2.3% (0.0 to 10.3%)	0.4 (0.0 to 1.8)
Chronic obstructive pulmonary disease	American Samoa	2010	All Ages	0.0 (0.0 to 0.0)	0.9% (0.0 to 7.6%)	0.1 (0.0 to 1.0)
Chronic obstructive pulmonary disease	American Samoa	2015	All Ages	0.0 (0.0 to 0.0)	1.4% (0.0 to 8.8%)	0.2 (0.0 to 1.1)
Chronic obstructive pulmonary disease	American Samoa	1990	Age-standardized		2.4% (0.0 to 10.4%)	1.5 (0.0 to 6.8)
Chronic obstructive pulmonary disease	American Samoa	1995	Age-standardized		2.3% (0.0 to 10.3%)	1.4 (0.0 to 6.4)
Chronic obstructive pulmonary disease	American Samoa	2000	Age-standardized		2.3% (0.0 to 11.0%)	1.2 (0.0 to 5.6)
Chronic obstructive pulmonary disease	American Samoa	2005	Age-standardized		2.4% (0.0 to 10.5%)	1.1 (0.0 to 5.0)
Chronic obstructive pulmonary disease	American Samoa	2010	Age-standardized		0.9% (0.0 to 7.7%)	0.4 (0.0 to 3.0)

Cause name	Location	Year	Age group	Number (in thousands)	PAF	Rate (per 100,000)
Chronic obstructive pulmonary disease	American Samoa	2015	Age-standardized		1.4% (0.0 to 8.9%)	0.5 (0.0 to 3.3)
All causes	Federated States of Micronesia	1990	All Ages	0.0 (0.0 to 0.0)	3.1% (1.0 to 5.7%)	21.6 (7.0 to 41.2)
All causes	Federated States of Micronesia	1995	All Ages	0.0 (0.0 to 0.1)	3.2% (1.1 to 5.9%)	21.4 (6.8 to 42.4)
All causes	Federated States of Micronesia	2000	All Ages	0.0 (0.0 to 0.0)	3.2% (1.2 to 5.4%)	20.2 (6.9 to 38.5)
All causes	Federated States of Micronesia	2005	All Ages	0.0 (0.0 to 0.0)	3.3% (1.1 to 5.5%)	20.8 (6.5 to 38.7)
All causes	Federated States of Micronesia	2010	All Ages	0.0 (0.0 to 0.0)	2.6% (0.7 to 4.9%)	16.3 (4.2 to 35.7)
All causes	Federated States of Micronesia	2015	All Ages	0.0 (0.0 to 0.0)	3.0% (0.9 to 5.1%)	18.6 (5.3 to 37.2)
All causes	Federated States of Micronesia	1990	Age-standardized		3.6% (1.2 to 6.4%)	52.0 (16.8 to 99.0)
All causes	Federated States of Micronesia	1995	Age-standardized		3.6% (1.3 to 6.7%)	50.1 (15.5 to 98.0)
All causes	Federated States of Micronesia	2000	Age-standardized		3.6% (1.3 to 6.0%)	46.4 (16.6 to 88.5)
All causes	Federated States of Micronesia	2005	Age-standardized		3.5% (1.2 to 5.9%)	43.0 (13.6 to 79.1)
All causes	Federated States of Micronesia	2010	Age-standardized		2.7% (0.7 to 5.2%)	33.1 (8.3 to 72.0)
All causes	Federated States of Micronesia	2015	Age-standardized		3.1% (0.9 to 5.3%)	35.9 (10.6 to 70.3)
Lower respiratory infections	Federated States of Micronesia	1990	All Ages	0.0 (0.0 to 0.0)	7.3% (0.6 to 19.7%)	4.3 (0.4 to 11.7)
Lower respiratory infections	Federated States of Micronesia	1995	All Ages	0.0 (0.0 to 0.0)	7.6% (0.7 to 19.3%)	3.7 (0.3 to 9.8)
Lower respiratory infections	Federated States of Micronesia	2000	All Ages	0.0 (0.0 to 0.0)	7.4% (0.6 to 19.2%)	3.0 (0.2 to 8.1)
Lower respiratory infections	Federated States of Micronesia	2005	All Ages	0.0 (0.0 to 0.0)	7.3% (0.5 to 18.6%)	2.7 (0.2 to 7.4)
Lower respiratory infections	Federated States of Micronesia	2010	All Ages	0.0 (0.0 to 0.0)	5.2% (0.2 to 16.4%)	1.8 (0.1 to 6.0)
Lower respiratory infections	Federated States of Micronesia	2015	All Ages	0.0 (0.0 to 0.0)	6.0% (0.3 to 16.8%)	1.9 (0.1 to 5.8)
Lower respiratory infections	Federated States of Micronesia	1990	Age-standardized		7.3% (0.6 to 19.7%)	7.4 (0.7 to 20.6)
Lower respiratory infections	Federated States of Micronesia	1995	Age-standardized		7.6% (0.7 to 19.3%)	6.9 (0.6 to 17.8)
Lower respiratory infections	Federated States of Micronesia	2000	Age-standardized		7.4% (0.6 to 19.2%)	5.8 (0.4 to 16.3)
Lower respiratory infections	Federated States of Micronesia	2005	Age-standardized		7.3% (0.5 to 18.6%)	5.1 (0.4 to 14.1)
Lower respiratory infections	Federated States of Micronesia	2010	Age-standardized		5.2% (0.2 to 16.4%)	3.5 (0.1 to 12.2)
Lower respiratory infections	Federated States of Micronesia	2015	Age-standardized		6.0% (0.3 to 16.8%)	3.8 (0.2 to 11.3)
Tracheal, bronchus, and lung cancer	Federated States of Micronesia	1990	All Ages	0.0 (0.0 to 0.0)	4.8% (0.8 to 11.4%)	0.4 (0.1 to 1.0)
Tracheal, bronchus, and lung cancer	Federated States of Micronesia	1995	All Ages	0.0 (0.0 to 0.0)	5.0% (0.6 to 12.7%)	0.5 (0.1 to 1.2)
Tracheal, bronchus, and lung cancer	Federated States of Micronesia	2000	All Ages	0.0 (0.0 to 0.0)	4.9% (0.8 to 11.3%)	0.4 (0.1 to 1.1)
Tracheal, bronchus, and lung cancer	Federated States of Micronesia	2005	All Ages	0.0 (0.0 to 0.0)	4.9% (0.6 to 11.6%)	0.5 (0.1 to 1.2)
Tracheal, bronchus, and lung cancer	Federated States of Micronesia	2010	All Ages	0.0 (0.0 to 0.0)	3.3% (0.3 to 9.6%)	0.4 (0.0 to 1.1)
Tracheal, bronchus, and lung cancer	Federated States of Micronesia	2015	All Ages	0.0 (0.0 to 0.0)	3.9% (0.5 to 9.8%)	0.5 (0.0 to 1.2)
Tracheal, bronchus, and lung cancer	Federated States of Micronesia	1990	Age-standardized		4.9% (0.8 to 11.5%)	1.1 (0.2 to 2.7)
Tracheal, bronchus, and lung cancer	Federated States of Micronesia	1995	Age-standardized		5.1% (0.6 to 12.8%)	1.2 (0.1 to 2.9)
Tracheal, bronchus, and lung cancer	Federated States of Micronesia	2000	Age-standardized		4.9% (0.8 to 11.4%)	1.1 (0.2 to 2.6)
Tracheal, bronchus, and lung cancer	Federated States of Micronesia	2005	Age-standardized		4.9% (0.6 to 11.8%)	1.1 (0.2 to 2.6)
Tracheal, bronchus, and lung cancer	Federated States of Micronesia	2010	Age-standardized		3.4% (0.3 to 9.7%)	0.8 (0.1 to 2.2)

Cause name	Location	Year	Age group	Number (in thousands)	PAF	Rate (per 100,000)
Tracheal, bronchus, and lung cancer	Federated States of Micronesia	2015	Age-standardized		4.0% (0.5 to 9.9%)	0.9 (0.1 to 2.2)
Cardiovascular diseases	Federated States of Micronesia	1990	All Ages	0.0 (0.0 to 0.0)	6.6% (2.5 to 11.0%)	14.3 (4.8 to 26.0)
Cardiovascular diseases	Federated States of Micronesia	1995	All Ages	0.0 (0.0 to 0.0)	6.8% (2.7 to 11.3%)	14.9 (5.1 to 28.7)
Cardiovascular diseases	Federated States of Micronesia	2000	All Ages	0.0 (0.0 to 0.0)	6.9% (2.9 to 11.3%)	14.7 (5.5 to 27.3)
Cardiovascular diseases	Federated States of Micronesia	2005	All Ages	0.0 (0.0 to 0.0)	6.7% (2.5 to 10.7%)	15.5 (5.3 to 28.2)
Cardiovascular diseases	Federated States of Micronesia	2010	All Ages	0.0 (0.0 to 0.0)	5.4% (1.5 to 9.7%)	12.7 (3.2 to 27.0)
Cardiovascular diseases	Federated States of Micronesia	2015	All Ages	0.0 (0.0 to 0.0)	6.2% (2.0 to 10.1%)	14.5 (4.4 to 28.3)
Cardiovascular diseases	Federated States of Micronesia	1990	Age-standardized		6.3% (2.4 to 10.5%)	36.0 (11.9 to 65.1)
Cardiovascular diseases	Federated States of Micronesia	1995	Age-standardized		6.4% (2.5 to 10.7%)	35.5 (12.1 to 66.4)
Cardiovascular diseases	Federated States of Micronesia	2000	Age-standardized		6.5% (2.7 to 10.7%)	33.9 (13.1 to 61.4)
Cardiovascular diseases	Federated States of Micronesia	2005	Age-standardized		6.4% (2.3 to 10.5%)	32.1 (10.9 to 58.0)
Cardiovascular diseases	Federated States of Micronesia	2010	Age-standardized		5.0% (1.4 to 9.1%)	25.4 (6.5 to 53.8)
Cardiovascular diseases	Federated States of Micronesia	2015	Age-standardized		5.7% (1.9 to 9.5%)	27.5 (8.5 to 53.4)
Ischemic heart disease	Federated States of Micronesia	1990	All Ages	0.0 (0.0 to 0.0)	9.9% (3.7 to 16.0%)	9.5 (3.3 to 17.3)
Ischemic heart disease	Federated States of Micronesia	1995	All Ages	0.0 (0.0 to 0.0)	10.0% (3.9 to 16.5%)	10.0 (3.4 to 18.9)
Ischemic heart disease	Federated States of Micronesia	2000	All Ages	0.0 (0.0 to 0.0)	10.1% (4.1 to 16.4%)	10.2 (3.7 to 19.0)
Ischemic heart disease	Federated States of Micronesia	2005	All Ages	0.0 (0.0 to 0.0)	9.7% (3.6 to 15.3%)	10.9 (3.7 to 20.4)
Ischemic heart disease	Federated States of Micronesia	2010	All Ages	0.0 (0.0 to 0.0)	7.7% (2.1 to 13.8%)	9.2 (2.2 to 19.8)
Ischemic heart disease	Federated States of Micronesia	2015	All Ages	0.0 (0.0 to 0.0)	8.7% (2.9 to 14.3%)	10.5 (3.2 to 20.6)
Ischemic heart disease	Federated States of Micronesia	1990	Age-standardized		9.1% (3.4 to 15.0%)	23.6 (8.0 to 42.3)
Ischemic heart disease	Federated States of Micronesia	1995	Age-standardized		9.2% (3.4 to 15.2%)	23.6 (7.8 to 43.8)
Ischemic heart disease	Federated States of Micronesia	2000	Age-standardized		9.3% (3.9 to 15.4%)	23.1 (8.6 to 42.1)
Ischemic heart disease	Federated States of Micronesia	2005	Age-standardized		9.1% (3.4 to 14.6%)	22.3 (7.7 to 41.1)
Ischemic heart disease	Federated States of Micronesia	2010	Age-standardized		7.2% (1.9 to 13.0%)	18.0 (4.3 to 38.4)
Ischemic heart disease	Federated States of Micronesia	2015	Age-standardized		8.1% (2.6 to 13.2%)	19.6 (5.9 to 37.5)
Ischemic stroke	Federated States of Micronesia	1990	All Ages	0.0 (0.0 to 0.0)	6.2% (2.0 to 10.8%)	1.8 (0.5 to 3.6)
Ischemic stroke	Federated States of Micronesia	1995	All Ages	0.0 (0.0 to 0.0)	6.3% (2.1 to 11.5%)	1.8 (0.5 to 3.5)
Ischemic stroke	Federated States of Micronesia	2000	All Ages	0.0 (0.0 to 0.0)	6.2% (1.9 to 11.1%)	1.7 (0.5 to 3.3)
Ischemic stroke	Federated States of Micronesia	2005	All Ages	0.0 (0.0 to 0.0)	5.9% (1.8 to 10.5%)	1.7 (0.5 to 3.3)
Ischemic stroke	Federated States of Micronesia	2010	All Ages	0.0 (0.0 to 0.0)	4.6% (1.0 to 9.0%)	1.4 (0.3 to 3.0)
Ischemic stroke	Federated States of Micronesia	2015	All Ages	0.0 (0.0 to 0.0)	5.4% (1.5 to 9.8%)	1.5 (0.4 to 3.2)
Ischemic stroke	Federated States of Micronesia	1990	Age-standardized		5.8% (1.8 to 10.6%)	5.1 (1.5 to 10.1)
Ischemic stroke	Federated States of Micronesia	1995	Age-standardized		5.9% (1.8 to 10.8%)	4.9 (1.4 to 9.7)
Ischemic stroke	Federated States of Micronesia	2000	Age-standardized		5.8% (1.9 to 10.6%)	4.4 (1.4 to 8.6)
Ischemic stroke	Federated States of Micronesia	2005	Age-standardized		5.7% (1.8 to 10.4%)	4.0 (1.2 to 7.7)
Ischemic stroke	Federated States of Micronesia	2010	Age-standardized		4.4% (0.9 to 8.8%)	3.1 (0.6 to 6.9)

Cause name	Location	Year	Age group	Number (in thousands)	PAF	Rate (per 100,000)
Ischemic stroke	Federated States of Micronesia	2015	Age-standardized		5.0% (1.4 to 9.6%)	3.3 (0.9 to 6.9)
Hemorrhagic stroke	Federated States of Micronesia	1990	All Ages	0.0 (0.0 to 0.0)	6.4% (2.1 to 11.1%)	3.0 (1.0 to 5.9)
Hemorrhagic stroke	Federated States of Micronesia	1995	All Ages	0.0 (0.0 to 0.0)	6.6% (2.2 to 11.7%)	3.0 (0.9 to 6.0)
Hemorrhagic stroke	Federated States of Micronesia	2000	All Ages	0.0 (0.0 to 0.0)	6.7% (2.4 to 11.6%)	2.9 (0.9 to 5.8)
Hemorrhagic stroke	Federated States of Micronesia	2005	All Ages	0.0 (0.0 to 0.0)	6.5% (2.2 to 11.3%)	2.8 (0.9 to 5.6)
Hemorrhagic stroke	Federated States of Micronesia	2010	All Ages	0.0 (0.0 to 0.0)	5.1% (1.2 to 9.6%)	2.2 (0.5 to 4.7)
Hemorrhagic stroke	Federated States of Micronesia	2015	All Ages	0.0 (0.0 to 0.0)	5.8% (1.7 to 10.4%)	2.5 (0.7 to 5.0)
Hemorrhagic stroke	Federated States of Micronesia	1990	Age-standardized		6.3% (2.1 to 11.0%)	7.2 (2.3 to 14.1)
Hemorrhagic stroke	Federated States of Micronesia	1995	Age-standardized		6.4% (2.1 to 11.5%)	7.0 (2.1 to 13.8)
Hemorrhagic stroke	Federated States of Micronesia	2000	Age-standardized		6.4% (2.1 to 11.2%)	6.4 (2.1 to 12.7)
Hemorrhagic stroke	Federated States of Micronesia	2005	Age-standardized		6.3% (2.1 to 11.0%)	5.7 (1.7 to 11.1)
Hemorrhagic stroke	Federated States of Micronesia	2010	Age-standardized		4.8% (1.1 to 9.2%)	4.3 (0.9 to 9.3)
Hemorrhagic stroke	Federated States of Micronesia	2015	Age-standardized		5.5% (1.6 to 10.0%)	4.6 (1.2 to 9.2)
Chronic obstructive pulmonary disease	Federated States of Micronesia	1990	All Ages	0.0 (0.0 to 0.0)	8.1% (1.1 to 18.3%)	2.6 (0.4 to 6.2)
Chronic obstructive pulmonary disease	Federated States of Micronesia	1995	All Ages	0.0 (0.0 to 0.0)	8.5% (1.4 to 20.1%)	2.4 (0.4 to 5.8)
Chronic obstructive pulmonary disease	Federated States of Micronesia	2000	All Ages	0.0 (0.0 to 0.0)	8.5% (1.1 to 19.4%)	2.1 (0.3 to 5.2)
Chronic obstructive pulmonary disease	Federated States of Micronesia	2005	All Ages	0.0 (0.0 to 0.0)	8.5% (1.2 to 19.3%)	2.1 (0.3 to 5.0)
Chronic obstructive pulmonary disease	Federated States of Micronesia	2010	All Ages	0.0 (0.0 to 0.0)	6.2% (0.6 to 16.0%)	1.5 (0.2 to 4.1)
Chronic obstructive pulmonary disease	Federated States of Micronesia	2015	All Ages	0.0 (0.0 to 0.0)	7.3% (0.7 to 17.4%)	1.7 (0.1 to 4.4)
Chronic obstructive pulmonary disease	Federated States of Micronesia	1990	Age-standardized		8.6% (1.2 to 19.3%)	7.5 (1.1 to 18.0)
Chronic obstructive pulmonary disease	Federated States of Micronesia	1995	Age-standardized		8.9% (1.5 to 21.2%)	6.5 (1.1 to 15.6)
Chronic obstructive pulmonary disease	Federated States of Micronesia	2000	Age-standardized		8.8% (1.2 to 20.0%)	5.5 (0.7 to 13.4)
Chronic obstructive pulmonary disease	Federated States of Micronesia	2005	Age-standardized		8.7% (1.3 to 19.9%)	4.8 (0.7 to 11.6)
Chronic obstructive pulmonary disease	Federated States of Micronesia	2010	Age-standardized		6.4% (0.6 to 16.3%)	3.4 (0.3 to 9.2)
Chronic obstructive pulmonary disease	Federated States of Micronesia	2015	Age-standardized		7.4% (0.7 to 17.8%)	3.7 (0.3 to 9.5)
All causes	Fiji	1990	All Ages	0.1 (0.1 to 0.2)	3.1% (2.4 to 4.1%)	20.1 (14.6 to 27.1)
All causes	Fiji	1995	All Ages	0.2 (0.1 to 0.2)	3.2% (2.4 to 4.2%)	22.1 (16.2 to 29.5)
All causes	Fiji	2000	All Ages	0.2 (0.1 to 0.3)	3.2% (2.0 to 4.5%)	23.6 (15.2 to 33.6)
All causes	Fiji	2005	All Ages	0.2 (0.1 to 0.3)	3.0% (2.0 to 4.2%)	24.6 (15.8 to 34.8)
All causes	Fiji	2010	All Ages	0.2 (0.1 to 0.3)	2.2% (1.4 to 3.4%)	19.4 (11.5 to 29.1)
All causes	Fiji	2015	All Ages	0.2 (0.1 to 0.3)	2.5% (1.6 to 3.7%)	23.3 (14.2 to 33.9)
All causes	Fiji	1990	Age-standardized		3.2% (2.4 to 4.2%)	45.2 (32.8 to 60.4)
All causes	Fiji	1995	Age-standardized		3.2% (2.4 to 4.2%)	47.2 (34.7 to 62.5)
All causes	Fiji	2000	Age-standardized		3.1% (2.0 to 4.5%)	46.2 (29.6 to 65.9)
All causes	Fiji	2005	Age-standardized		2.9% (1.9 to 4.1%)	42.4 (27.1 to 59.7)
All causes	Fiji	2010	Age-standardized		2.2% (1.3 to 3.2%)	31.2 (18.5 to 46.1)

Cause name	Location	Year	Age group	Number (in thousands)	PAF	Rate (per 100,000)
All causes	Fiji	2015	Age-standardized		2.4% (1.5 to 3.6%)	33.3 (20.8 to 48.4)
Lower respiratory infections	Fiji	1990	All Ages	0.0 (0.0 to 0.1)	6.7% (2.0 to 15.0%)	3.4 (1.0 to 7.4)
Lower respiratory infections	Fiji	1995	All Ages	0.0 (0.0 to 0.1)	6.8% (2.4 to 14.7%)	3.5 (1.1 to 7.7)
Lower respiratory infections	Fiji	2000	All Ages	0.0 (0.0 to 0.1)	6.9% (1.8 to 16.2%)	3.5 (0.9 to 8.4)
Lower respiratory infections	Fiji	2005	All Ages	0.0 (0.0 to 0.1)	7.4% (2.1 to 16.4%)	3.7 (1.0 to 8.5)
Lower respiratory infections	Fiji	2010	All Ages	0.0 (0.0 to 0.1)	4.9% (1.0 to 13.3%)	2.4 (0.5 to 6.2)
Lower respiratory infections	Fiji	2015	All Ages	0.0 (0.0 to 0.1)	5.9% (1.3 to 14.4%)	2.8 (0.6 to 6.9)
Lower respiratory infections	Fiji	1990	Age-standardized		6.7% (2.0 to 15.0%)	6.7 (1.9 to 15.3)
Lower respiratory infections	Fiji	1995	Age-standardized		6.8% (2.4 to 14.7%)	6.8 (2.3 to 15.2)
Lower respiratory infections	Fiji	2000	Age-standardized		6.9% (1.8 to 16.2%)	6.5 (1.7 to 15.5)
Lower respiratory infections	Fiji	2005	Age-standardized		7.4% (2.1 to 16.4%)	6.3 (1.7 to 14.4)
Lower respiratory infections	Fiji	2010	Age-standardized		4.9% (1.0 to 13.3%)	3.9 (0.8 to 10.2)
Lower respiratory infections	Fiji	2015	Age-standardized		5.9% (1.3 to 14.4%)	4.2 (0.9 to 10.3)
Tracheal, bronchus, and lung cancer	Fiji	1990	All Ages	0.0 (0.0 to 0.0)	4.4% (1.9 to 8.1%)	0.1 (0.0 to 0.2)
Tracheal, bronchus, and lung cancer	Fiji	1995	All Ages	0.0 (0.0 to 0.0)	4.5% (1.9 to 8.1%)	0.1 (0.1 to 0.2)
Tracheal, bronchus, and lung cancer	Fiji	2000	All Ages	0.0 (0.0 to 0.0)	4.5% (1.5 to 8.9%)	0.2 (0.1 to 0.3)
Tracheal, bronchus, and lung cancer	Fiji	2005	All Ages	0.0 (0.0 to 0.0)	4.9% (1.8 to 9.7%)	0.2 (0.1 to 0.4)
Tracheal, bronchus, and lung cancer	Fiji	2010	All Ages	0.0 (0.0 to 0.0)	3.2% (1.0 to 6.7%)	0.2 (0.0 to 0.3)
Tracheal, bronchus, and lung cancer	Fiji	2015	All Ages	0.0 (0.0 to 0.0)	3.9% (1.3 to 8.1%)	0.2 (0.1 to 0.4)
Tracheal, bronchus, and lung cancer	Fiji	1990	Age-standardized		4.4% (2.0 to 8.2%)	0.3 (0.1 to 0.6)
Tracheal, bronchus, and lung cancer	Fiji	1995	Age-standardized		4.5% (1.9 to 8.2%)	0.3 (0.1 to 0.6)
Tracheal, bronchus, and lung cancer	Fiji	2000	Age-standardized		4.5% (1.6 to 9.0%)	0.3 (0.1 to 0.7)
Tracheal, bronchus, and lung cancer	Fiji	2005	Age-standardized		4.9% (1.8 to 9.7%)	0.4 (0.1 to 0.8)
Tracheal, bronchus, and lung cancer	Fiji	2010	Age-standardized		3.2% (1.0 to 6.8%)	0.3 (0.1 to 0.5)
Tracheal, bronchus, and lung cancer	Fiji	2015	Age-standardized		3.9% (1.3 to 8.2%)	0.3 (0.1 to 0.6)
Cardiovascular diseases	Fiji	1990	All Ages	0.1 (0.1 to 0.1)	7.1% (5.5 to 8.8%)	15.7 (11.6 to 20.9)
Cardiovascular diseases	Fiji	1995	All Ages	0.1 (0.1 to 0.2)	7.2% (5.5 to 9.1%)	17.5 (13.0 to 22.9)
Cardiovascular diseases	Fiji	2000	All Ages	0.1 (0.1 to 0.2)	7.1% (4.7 to 9.8%)	18.9 (12.4 to 26.1)
Cardiovascular diseases	Fiji	2005	All Ages	0.2 (0.1 to 0.2)	7.1% (4.7 to 9.5%)	19.6 (13.0 to 27.2)
Cardiovascular diseases	Fiji	2010	All Ages	0.1 (0.1 to 0.2)	5.4% (3.2 to 7.8%)	16.1 (9.5 to 23.4)
Cardiovascular diseases	Fiji	2015	All Ages	0.2 (0.1 to 0.3)	6.0% (3.9 to 8.5%)	19.2 (12.0 to 27.5)
Cardiovascular diseases	Fiji	1990	Age-standardized		6.2% (4.8 to 7.8%)	35.4 (26.5 to 46.3)
Cardiovascular diseases	Fiji	1995	Age-standardized		6.3% (4.8 to 7.9%)	37.3 (27.7 to 49.2)
Cardiovascular diseases	Fiji	2000	Age-standardized		6.2% (4.1 to 8.7%)	36.7 (24.1 to 50.7)
Cardiovascular diseases	Fiji	2005	Age-standardized		6.1% (4.1 to 8.3%)	33.2 (21.8 to 45.3)
Cardiovascular diseases	Fiji	2010	Age-standardized		4.7% (2.8 to 6.8%)	25.4 (15.2 to 36.8)

Cause name	Location	Year	Age group	Number (in thousands)	PAF	Rate (per 100,000)
Cardiovascular diseases	Fiji	2015	Age-standardized		5.4% (3.5 to 7.6%)	27.0 (17.1 to 38.6)
Ischemic heart disease	Fiji	1990	All Ages	0.1 (0.1 to 0.1)	10.4% (7.8 to 13.2%)	11.3 (8.0 to 15.4)
Ischemic heart disease	Fiji	1995	All Ages	0.1 (0.1 to 0.1)	10.5% (7.6 to 13.6%)	12.8 (9.0 to 17.2)
Ischemic heart disease	Fiji	2000	All Ages	0.1 (0.1 to 0.2)	10.5% (6.8 to 14.4%)	14.1 (8.8 to 19.7)
Ischemic heart disease	Fiji	2005	All Ages	0.1 (0.1 to 0.2)	10.7% (7.1 to 14.6%)	15.2 (9.8 to 21.5)
Ischemic heart disease	Fiji	2010	All Ages	0.1 (0.1 to 0.2)	8.1% (4.7 to 11.9%)	12.7 (7.5 to 19.0)
Ischemic heart disease	Fiji	2015	All Ages	0.1 (0.1 to 0.2)	9.0% (5.7 to 12.8%)	15.1 (9.3 to 22.2)
Ischemic heart disease	Fiji	1990	Age-standardized		9.1% (6.8 to 11.5%)	24.3 (17.3 to 32.5)
Ischemic heart disease	Fiji	1995	Age-standardized		9.1% (6.7 to 11.8%)	25.6 (18.2 to 34.2)
Ischemic heart disease	Fiji	2000	Age-standardized		9.2% (6.0 to 12.8%)	25.9 (16.6 to 36.3)
Ischemic heart disease	Fiji	2005	Age-standardized		9.3% (6.3 to 12.7%)	24.6 (16.3 to 34.3)
Ischemic heart disease	Fiji	2010	Age-standardized		7.2% (4.3 to 10.5%)	19.4 (11.3 to 28.5)
Ischemic heart disease	Fiji	2015	Age-standardized		8.1% (5.1 to 11.6%)	20.6 (12.5 to 29.8)
Ischemic stroke	Fiji	1990	All Ages	0.0 (0.0 to 0.0)	6.0% (4.0 to 8.3%)	1.5 (0.9 to 2.1)
Ischemic stroke	Fiji	1995	All Ages	0.0 (0.0 to 0.0)	6.1% (4.1 to 8.5%)	1.6 (1.0 to 2.2)
Ischemic stroke	Fiji	2000	All Ages	0.0 (0.0 to 0.0)	6.1% (3.7 to 9.1%)	1.6 (0.9 to 2.4)
Ischemic stroke	Fiji	2005	All Ages	0.0 (0.0 to 0.0)	6.4% (3.7 to 9.4%)	1.5 (0.9 to 2.2)
Ischemic stroke	Fiji	2010	All Ages	0.0 (0.0 to 0.0)	4.7% (2.5 to 7.5%)	1.2 (0.6 to 1.8)
Ischemic stroke	Fiji	2015	All Ages	0.0 (0.0 to 0.0)	5.4% (3.0 to 8.3%)	1.5 (0.8 to 2.3)
Ischemic stroke	Fiji	1990	Age-standardized		5.4% (3.3 to 8.0%)	4.5 (2.6 to 6.8)
Ischemic stroke	Fiji	1995	Age-standardized		5.4% (3.3 to 7.9%)	4.6 (2.8 to 7.0)
Ischemic stroke	Fiji	2000	Age-standardized		5.4% (3.1 to 8.5%)	4.2 (2.3 to 6.7)
Ischemic stroke	Fiji	2005	Age-standardized		5.6% (3.1 to 8.8%)	3.4 (1.8 to 5.3)
Ischemic stroke	Fiji	2010	Age-standardized		4.3% (2.1 to 7.1%)	2.4 (1.1 to 4.0)
Ischemic stroke	Fiji	2015	Age-standardized		4.9% (2.5 to 8.0%)	2.5 (1.2 to 4.2)
Hemorrhagic stroke	Fiji	1990	All Ages	0.0 (0.0 to 0.0)	6.5% (4.5 to 8.8%)	2.9 (1.9 to 4.1)
Hemorrhagic stroke	Fiji	1995	All Ages	0.0 (0.0 to 0.0)	6.6% (4.5 to 8.8%)	3.2 (2.2 to 4.4)
Hemorrhagic stroke	Fiji	2000	All Ages	0.0 (0.0 to 0.0)	6.6% (4.0 to 9.7%)	3.2 (1.9 to 4.8)
Hemorrhagic stroke	Fiji	2005	All Ages	0.0 (0.0 to 0.0)	6.8% (4.1 to 10.0%)	2.9 (1.7 to 4.2)
Hemorrhagic stroke	Fiji	2010	All Ages	0.0 (0.0 to 0.0)	5.0% (2.7 to 7.8%)	2.2 (1.2 to 3.3)
Hemorrhagic stroke	Fiji	2015	All Ages	0.0 (0.0 to 0.0)	5.7% (3.3 to 8.5%)	2.6 (1.5 to 4.0)
Hemorrhagic stroke	Fiji	1990	Age-standardized		5.8% (3.9 to 8.2%)	6.7 (4.2 to 9.8)
Hemorrhagic stroke	Fiji	1995	Age-standardized		5.9% (3.9 to 8.2%)	7.1 (4.6 to 10.2)
Hemorrhagic stroke	Fiji	2000	Age-standardized		5.9% (3.5 to 8.8%)	6.6 (3.8 to 10.0)
Hemorrhagic stroke	Fiji	2005	Age-standardized		6.1% (3.6 to 9.0%)	5.2 (3.0 to 7.8)
Hemorrhagic stroke	Fiji	2010	Age-standardized		4.5% (2.4 to 7.3%)	3.7 (1.9 to 6.0)

Cause name	Location	Year	Age group	Number (in thousands)	PAF	Rate (per 100,000)
Hemorrhagic stroke	Fiji	2015	Age-standardized		5.2% (2.9 to 8.1%)	3.9 (2.1 to 6.1)
Chronic obstructive pulmonary disease	Fiji	1990	All Ages	0.0 (0.0 to 0.0)	7.6% (2.5 to 14.8%)	0.9 (0.3 to 1.9)
Chronic obstructive pulmonary disease	Fiji	1995	All Ages	0.0 (0.0 to 0.0)	7.7% (2.6 to 15.1%)	1.0 (0.3 to 1.9)
Chronic obstructive pulmonary disease	Fiji	2000	All Ages	0.0 (0.0 to 0.0)	7.8% (2.5 to 15.6%)	1.1 (0.3 to 2.1)
Chronic obstructive pulmonary disease	Fiji	2005	All Ages	0.0 (0.0 to 0.0)	8.4% (2.8 to 16.3%)	1.1 (0.4 to 2.2)
Chronic obstructive pulmonary disease	Fiji	2010	All Ages	0.0 (0.0 to 0.0)	6.0% (1.5 to 13.1%)	0.8 (0.2 to 1.9)
Chronic obstructive pulmonary disease	Fiji	2015	All Ages	0.0 (0.0 to 0.0)	7.0% (2.0 to 14.8%)	1.1 (0.3 to 2.2)
Chronic obstructive pulmonary disease	Fiji	1990	Age-standardized		8.0% (2.6 to 15.5%)	2.8 (0.9 to 5.7)
Chronic obstructive pulmonary disease	Fiji	1995	Age-standardized		8.1% (2.7 to 15.8%)	2.8 (1.0 to 5.5)
Chronic obstructive pulmonary disease	Fiji	2000	Age-standardized		8.1% (2.6 to 16.2%)	2.7 (0.8 to 5.4)
Chronic obstructive pulmonary disease	Fiji	2005	Age-standardized		8.6% (2.9 to 16.8%)	2.5 (0.8 to 5.0)
Chronic obstructive pulmonary disease	Fiji	2010	Age-standardized		6.1% (1.5 to 13.4%)	1.7 (0.4 to 3.8)
Chronic obstructive pulmonary disease	Fiji	2015	Age-standardized		7.1% (2.1 to 15.0%)	1.8 (0.5 to 3.8)
All causes	Guam	1990	All Ages	0.0 (0.0 to 0.0)	3.3% (0.2 to 6.2%)	14.8 (0.9 to 28.2)
All causes	Guam	1995	All Ages	0.0 (0.0 to 0.0)	3.2% (0.2 to 5.9%)	15.2 (0.8 to 28.3)
All causes	Guam	2000	All Ages	0.0 (0.0 to 0.1)	3.8% (0.4 to 6.8%)	19.5 (2.1 to 34.9)
All causes	Guam	2005	All Ages	0.0 (0.0 to 0.1)	4.0% (0.3 to 7.0%)	24.1 (1.8 to 43.0)
All causes	Guam	2010	All Ages	0.0 (0.0 to 0.1)	3.5% (0.2 to 6.4%)	27.0 (1.5 to 50.0)
All causes	Guam	2015	All Ages	0.1 (0.0 to 0.1)	3.4% (0.1 to 6.5%)	29.3 (1.2 to 56.0)
All causes	Guam	1990	Age-standardized		3.5% (0.2 to 6.7%)	31.0 (1.6 to 59.3)
All causes	Guam	1995	Age-standardized		3.3% (0.2 to 6.2%)	29.1 (1.5 to 54.9)
All causes	Guam	2000	Age-standardized		3.9% (0.4 to 7.0%)	32.0 (3.3 to 57.3)
All causes	Guam	2005	Age-standardized		4.0% (0.3 to 7.1%)	35.2 (2.4 to 62.7)
All causes	Guam	2010	Age-standardized		3.5% (0.2 to 6.4%)	33.9 (1.5 to 63.0)
All causes	Guam	2015	Age-standardized		3.4% (0.1 to 6.4%)	32.5 (1.3 to 62.6)
Lower respiratory infections	Guam	1990	All Ages	0.0 (0.0 to 0.0)	6.4% (0.0 to 20.0%)	1.4 (0.0 to 4.4)
Lower respiratory infections	Guam	1995	All Ages	0.0 (0.0 to 0.0)	6.4% (0.0 to 20.6%)	1.4 (0.0 to 4.4)
Lower respiratory infections	Guam	2000	All Ages	0.0 (0.0 to 0.0)	6.5% (0.0 to 20.0%)	1.5 (0.0 to 4.8)
Lower respiratory infections	Guam	2005	All Ages	0.0 (0.0 to 0.0)	6.9% (0.0 to 20.5%)	1.8 (0.0 to 5.4)
Lower respiratory infections	Guam	2010	All Ages	0.0 (0.0 to 0.0)	5.6% (0.0 to 18.6%)	1.8 (0.0 to 6.1)
Lower respiratory infections	Guam	2015	All Ages	0.0 (0.0 to 0.0)	5.5% (0.0 to 19.0%)	1.9 (0.0 to 6.7)
Lower respiratory infections	Guam	1990	Age-standardized		6.4% (0.0 to 20.0%)	2.9 (0.0 to 9.0)
Lower respiratory infections	Guam	1995	Age-standardized		6.4% (0.0 to 20.6%)	2.8 (0.0 to 8.8)
Lower respiratory infections	Guam	2000	Age-standardized		6.5% (0.0 to 20.0%)	2.6 (0.0 to 8.1)
Lower respiratory infections	Guam	2005	Age-standardized		6.9% (0.0 to 20.5%)	2.7 (0.0 to 8.3)
Lower respiratory infections	Guam	2010	Age-standardized		5.6% (0.0 to 18.6%)	2.4 (0.0 to 8.0)

Cause name	Location	Year	Age group	Number (in thousands)	PAF	Rate (per 100,000)
Lower respiratory infections	Guam	2015	Age-standardized		5.5% (0.0 to 19.0%)	2.2 (0.0 to 7.8)
Tracheal, bronchus, and lung cancer	Guam	1990	All Ages	0.0 (0.0 to 0.0)	4.3% (0.0 to 12.0%)	0.9 (0.0 to 2.5)
Tracheal, bronchus, and lung cancer	Guam	1995	All Ages	0.0 (0.0 to 0.0)	4.3% (0.0 to 12.1%)	1.0 (0.0 to 2.7)
Tracheal, bronchus, and lung cancer	Guam	2000	All Ages	0.0 (0.0 to 0.0)	4.3% (0.0 to 12.2%)	1.1 (0.0 to 2.9)
Tracheal, bronchus, and lung cancer	Guam	2005	All Ages	0.0 (0.0 to 0.0)	4.6% (0.0 to 12.5%)	1.4 (0.0 to 3.7)
Tracheal, bronchus, and lung cancer	Guam	2010	All Ages	0.0 (0.0 to 0.0)	3.7% (0.0 to 10.5%)	1.4 (0.0 to 4.0)
Tracheal, bronchus, and lung cancer	Guam	2015	All Ages	0.0 (0.0 to 0.0)	3.5% (0.0 to 10.8%)	1.5 (0.0 to 4.8)
Tracheal, bronchus, and lung cancer	Guam	1990	Age-standardized		4.3% (0.0 to 12.1%)	1.8 (0.0 to 5.2)
Tracheal, bronchus, and lung cancer	Guam	1995	Age-standardized		4.3% (0.0 to 12.1%)	1.8 (0.0 to 5.0)
Tracheal, bronchus, and lung cancer	Guam	2000	Age-standardized		4.3% (0.0 to 12.2%)	1.7 (0.0 to 4.9)
Tracheal, bronchus, and lung cancer	Guam	2005	Age-standardized		4.6% (0.0 to 12.5%)	2.0 (0.0 to 5.3)
Tracheal, bronchus, and lung cancer	Guam	2010	Age-standardized		3.7% (0.0 to 10.5%)	1.7 (0.0 to 4.9)
Tracheal, bronchus, and lung cancer	Guam	2015	Age-standardized		3.5% (0.0 to 10.8%)	1.7 (0.0 to 5.2)
Cardiovascular diseases	Guam	1990	All Ages	0.0 (0.0 to 0.0)	6.5% (0.4 to 11.3%)	11.3 (0.7 to 19.6)
Cardiovascular diseases	Guam	1995	All Ages	0.0 (0.0 to 0.0)	6.4% (0.4 to 10.8%)	11.5 (0.7 to 19.8)
Cardiovascular diseases	Guam	2000	All Ages	0.0 (0.0 to 0.0)	7.4% (0.9 to 12.4%)	15.4 (2.0 to 25.9)
Cardiovascular diseases	Guam	2005	All Ages	0.0 (0.0 to 0.1)	7.7% (0.7 to 12.6%)	19.2 (1.6 to 31.9)
Cardiovascular diseases	Guam	2010	All Ages	0.0 (0.0 to 0.1)	6.8% (0.4 to 11.7%)	22.0 (1.3 to 38.2)
Cardiovascular diseases	Guam	2015	All Ages	0.0 (0.0 to 0.1)	6.5% (0.3 to 11.5%)	24.0 (1.1 to 43.1)
Cardiovascular diseases	Guam	1990	Age-standardized		5.8% (0.3 to 10.0%)	23.1 (1.4 to 40.0)
Cardiovascular diseases	Guam	1995	Age-standardized		5.6% (0.3 to 9.4%)	21.5 (1.2 to 37.3)
Cardiovascular diseases	Guam	2000	Age-standardized		6.8% (0.8 to 11.5%)	24.9 (3.1 to 42.2)
Cardiovascular diseases	Guam	2005	Age-standardized		7.2% (0.6 to 11.8%)	27.6 (2.3 to 46.0)
Cardiovascular diseases	Guam	2010	Age-standardized		6.4% (0.3 to 11.2%)	27.4 (1.3 to 48.2)
Cardiovascular diseases	Guam	2015	Age-standardized		6.3% (0.3 to 11.1%)	26.4 (1.0 to 48.0)
Ischemic heart disease	Guam	1990	All Ages	0.0 (0.0 to 0.0)	10.5% (0.6 to 17.8%)	8.5 (0.5 to 14.5)
Ischemic heart disease	Guam	1995	All Ages	0.0 (0.0 to 0.0)	10.4% (0.5 to 17.5%)	8.7 (0.4 to 14.6)
Ischemic heart disease	Guam	2000	All Ages	0.0 (0.0 to 0.0)	10.1% (1.0 to 16.7%)	12.0 (1.2 to 20.3)
Ischemic heart disease	Guam	2005	All Ages	0.0 (0.0 to 0.0)	10.1% (0.7 to 16.7%)	15.6 (1.1 to 26.2)
Ischemic heart disease	Guam	2010	All Ages	0.0 (0.0 to 0.1)	8.8% (0.4 to 15.2%)	18.1 (0.7 to 31.5)
Ischemic heart disease	Guam	2015	All Ages	0.0 (0.0 to 0.1)	8.5% (0.3 to 15.0%)	19.8 (0.6 to 35.6)
Ischemic heart disease	Guam	1990	Age-standardized		9.4% (0.5 to 15.9%)	17.0 (0.9 to 29.6)
Ischemic heart disease	Guam	1995	Age-standardized		9.2% (0.3 to 15.7%)	15.8 (0.6 to 27.1)
Ischemic heart disease	Guam	2000	Age-standardized		9.3% (0.9 to 15.7%)	19.3 (1.8 to 32.9)
Ischemic heart disease	Guam	2005	Age-standardized		9.5% (0.6 to 15.6%)	22.3 (1.4 to 37.3)
Ischemic heart disease	Guam	2010	Age-standardized		8.4% (0.2 to 14.5%)	22.4 (0.7 to 39.4)

Cause name	Location	Year	Age group	Number (in thousands)	PAF	Rate (per 100,000)
Ischemic heart disease	Guam	2015	Age-standardized		8.2% (0.2 to 14.5%)	21.6 (0.6 to 38.9)
Ischemic stroke	Guam	1990	All Ages	0.0 (0.0 to 0.0)	6.4% (0.1 to 12.3%)	1.1 (0.0 to 2.2)
Ischemic stroke	Guam	1995	All Ages	0.0 (0.0 to 0.0)	6.3% (0.3 to 11.9%)	1.2 (0.1 to 2.2)
Ischemic stroke	Guam	2000	All Ages	0.0 (0.0 to 0.0)	6.2% (0.3 to 11.5%)	1.5 (0.1 to 2.7)
Ischemic stroke	Guam	2005	All Ages	0.0 (0.0 to 0.0)	6.4% (0.5 to 11.9%)	1.6 (0.1 to 3.0)
Ischemic stroke	Guam	2010	All Ages	0.0 (0.0 to 0.0)	5.4% (0.0 to 10.8%)	1.8 (0.0 to 3.4)
Ischemic stroke	Guam	2015	All Ages	0.0 (0.0 to 0.0)	5.3% (0.1 to 10.9%)	2.0 (0.0 to 4.1)
Ischemic stroke	Guam	1990	Age-standardized		5.9% (0.1 to 11.3%)	2.9 (0.0 to 5.6)
Ischemic stroke	Guam	1995	Age-standardized		5.8% (0.2 to 11.4%)	2.7 (0.1 to 5.4)
Ischemic stroke	Guam	2000	Age-standardized		5.9% (0.2 to 11.3%)	2.7 (0.1 to 5.2)
Ischemic stroke	Guam	2005	Age-standardized		6.0% (0.4 to 11.7%)	2.6 (0.2 to 4.9)
Ischemic stroke	Guam	2010	Age-standardized		5.2% (0.0 to 10.6%)	2.4 (0.0 to 4.8)
Ischemic stroke	Guam	2015	Age-standardized		5.1% (0.1 to 10.9%)	2.3 (0.0 to 4.9)
Hemorrhagic stroke	Guam	1990	All Ages	0.0 (0.0 to 0.0)	6.9% (0.3 to 13.0%)	1.6 (0.1 to 3.1)
Hemorrhagic stroke	Guam	1995	All Ages	0.0 (0.0 to 0.0)	6.9% (0.4 to 12.7%)	1.7 (0.1 to 3.1)
Hemorrhagic stroke	Guam	2000	All Ages	0.0 (0.0 to 0.0)	6.8% (0.5 to 12.6%)	1.9 (0.1 to 3.5)
Hemorrhagic stroke	Guam	2005	All Ages	0.0 (0.0 to 0.0)	7.0% (0.6 to 12.6%)	2.0 (0.2 to 3.6)
Hemorrhagic stroke	Guam	2010	All Ages	0.0 (0.0 to 0.0)	6.0% (0.1 to 11.6%)	2.1 (0.0 to 4.2)
Hemorrhagic stroke	Guam	2015	All Ages	0.0 (0.0 to 0.0)	5.7% (0.1 to 11.6%)	2.2 (0.0 to 4.6)
Hemorrhagic stroke	Guam	1990	Age-standardized		6.4% (0.2 to 12.1%)	3.2 (0.1 to 6.1)
Hemorrhagic stroke	Guam	1995	Age-standardized		6.3% (0.3 to 12.1%)	3.0 (0.2 to 5.7)
Hemorrhagic stroke	Guam	2000	Age-standardized		6.4% (0.4 to 11.8%)	2.9 (0.2 to 5.3)
Hemorrhagic stroke	Guam	2005	Age-standardized		6.6% (0.5 to 12.2%)	2.7 (0.2 to 5.1)
Hemorrhagic stroke	Guam	2010	Age-standardized		5.7% (0.1 to 11.3%)	2.6 (0.0 to 5.1)
Hemorrhagic stroke	Guam	2015	Age-standardized		5.6% (0.1 to 11.3%)	2.4 (0.0 to 5.0)
Chronic obstructive pulmonary disease	Guam	1990	All Ages	0.0 (0.0 to 0.0)	8.1% (0.0 to 20.9%)	1.3 (0.0 to 3.4)
Chronic obstructive pulmonary disease	Guam	1995	All Ages	0.0 (0.0 to 0.0)	8.1% (0.0 to 20.4%)	1.4 (0.0 to 3.5)
Chronic obstructive pulmonary disease	Guam	2000	All Ages	0.0 (0.0 to 0.0)	8.2% (0.0 to 21.1%)	1.5 (0.0 to 3.9)
Chronic obstructive pulmonary disease	Guam	2005	All Ages	0.0 (0.0 to 0.0)	8.7% (0.0 to 21.1%)	1.8 (0.0 to 4.4)
Chronic obstructive pulmonary disease	Guam	2010	All Ages	0.0 (0.0 to 0.0)	7.3% (0.0 to 19.6%)	1.8 (0.0 to 5.0)
Chronic obstructive pulmonary disease	Guam	2015	All Ages	0.0 (0.0 to 0.0)	7.1% (0.0 to 19.5%)	1.9 (0.0 to 5.4)
Chronic obstructive pulmonary disease	Guam	1990	Age-standardized		8.3% (0.0 to 21.4%)	3.3 (0.0 to 8.4)
Chronic obstructive pulmonary disease	Guam	1995	Age-standardized		8.2% (0.0 to 20.7%)	3.1 (0.0 to 7.8)
Chronic obstructive pulmonary disease	Guam	2000	Age-standardized		8.3% (0.0 to 21.3%)	2.8 (0.0 to 7.3)
Chronic obstructive pulmonary disease	Guam	2005	Age-standardized		8.7% (0.0 to 21.3%)	2.9 (0.0 to 7.1)
Chronic obstructive pulmonary disease	Guam	2010	Age-standardized		7.3% (0.0 to 19.7%)	2.4 (0.0 to 6.8)

Cause name	Location	Year	Age group	Number (in thousands)	PAF	Rate (per 100,000)
Chronic obstructive pulmonary disease	Guam	2015	Age-standardized		7.1% (0.0 to 19.6%)	2.3 (0.0 to 6.4)
All causes	Kiribati	1990	All Ages	0.0 (0.0 to 0.0)	0.8% (0.3 to 1.7%)	8.9 (2.7 to 18.5)
All causes	Kiribati	1995	All Ages	0.0 (0.0 to 0.0)	0.9% (0.3 to 1.8%)	8.9 (2.6 to 18.3)
All causes	Kiribati	2000	All Ages	0.0 (0.0 to 0.0)	1.0% (0.1 to 2.5%)	9.2 (0.7 to 22.9)
All causes	Kiribati	2005	All Ages	0.0 (0.0 to 0.0)	0.9% (0.1 to 2.4%)	8.0 (0.5 to 22.0)
All causes	Kiribati	2010	All Ages	0.0 (0.0 to 0.0)	0.4% (0.0 to 1.5%)	3.6 (0.0 to 13.7)
All causes	Kiribati	2015	All Ages	0.0 (0.0 to 0.0)	0.5% (0.0 to 1.6%)	3.9 (0.0 to 14.4)
All causes	Kiribati	1990	Age-standardized		1.0% (0.3 to 2.0%)	18.9 (5.5 to 37.3)
All causes	Kiribati	1995	Age-standardized		1.0% (0.3 to 2.1%)	19.1 (5.4 to 37.8)
All causes	Kiribati	2000	Age-standardized		1.1% (0.1 to 2.7%)	19.6 (1.6 to 47.8)
All causes	Kiribati	2005	Age-standardized		1.0% (0.1 to 2.7%)	16.7 (0.9 to 45.3)
All causes	Kiribati	2010	Age-standardized		0.5% (0.0 to 1.7%)	7.3 (0.0 to 27.0)
All causes	Kiribati	2015	Age-standardized		0.5% (0.0 to 1.8%)	7.4 (0.0 to 27.1)
Lower respiratory infections	Kiribati	1990	All Ages	0.0 (0.0 to 0.0)	1.7% (0.0 to 8.6%)	1.2 (0.0 to 6.5)
Lower respiratory infections	Kiribati	1995	All Ages	0.0 (0.0 to 0.0)	1.8% (0.0 to 9.2%)	1.1 (0.0 to 6.2)
Lower respiratory infections	Kiribati	2000	All Ages	0.0 (0.0 to 0.0)	1.7% (0.0 to 10.2%)	1.2 (0.0 to 7.2)
Lower respiratory infections	Kiribati	2005	All Ages	0.0 (0.0 to 0.0)	1.5% (0.0 to 8.8%)	0.9 (0.0 to 5.4)
Lower respiratory infections	Kiribati	2010	All Ages	0.0 (0.0 to 0.0)	0.6% (0.0 to 4.8%)	0.3 (0.0 to 2.5)
Lower respiratory infections	Kiribati	2015	All Ages	0.0 (0.0 to 0.0)	0.6% (0.0 to 6.5%)	0.3 (0.0 to 2.9)
Lower respiratory infections	Kiribati	1990	Age-standardized		1.7% (0.0 to 8.6%)	1.8 (0.0 to 9.2)
Lower respiratory infections	Kiribati	1995	Age-standardized		1.8% (0.0 to 9.2%)	1.7 (0.0 to 9.2)
Lower respiratory infections	Kiribati	2000	Age-standardized		1.7% (0.0 to 10.2%)	1.8 (0.0 to 11.0)
Lower respiratory infections	Kiribati	2005	Age-standardized		1.5% (0.0 to 8.8%)	1.5 (0.0 to 9.0)
Lower respiratory infections	Kiribati	2010	Age-standardized		0.6% (0.0 to 4.8%)	0.6 (0.0 to 4.5)
Lower respiratory infections	Kiribati	2015	Age-standardized		0.6% (0.0 to 6.5%)	0.5 (0.0 to 5.6)
Tracheal, bronchus, and lung cancer	Kiribati	1990	All Ages	0.0 (0.0 to 0.0)	1.0% (0.0 to 3.8%)	0.1 (0.0 to 0.3)
Tracheal, bronchus, and lung cancer	Kiribati	1995	All Ages	0.0 (0.0 to 0.0)	1.0% (0.0 to 3.9%)	0.1 (0.0 to 0.3)
Tracheal, bronchus, and lung cancer	Kiribati	2000	All Ages	0.0 (0.0 to 0.0)	1.0% (0.0 to 4.5%)	0.1 (0.0 to 0.4)
Tracheal, bronchus, and lung cancer	Kiribati	2005	All Ages	0.0 (0.0 to 0.0)	0.9% (0.0 to 4.1%)	0.1 (0.0 to 0.4)
Tracheal, bronchus, and lung cancer	Kiribati	2010	All Ages	0.0 (0.0 to 0.0)	0.3% (0.0 to 2.5%)	0.0 (0.0 to 0.2)
Tracheal, bronchus, and lung cancer	Kiribati	2015	All Ages	0.0 (0.0 to 0.0)	0.3% (0.0 to 2.5%)	0.0 (0.0 to 0.2)
Tracheal, bronchus, and lung cancer	Kiribati	1990	Age-standardized		1.0% (0.0 to 3.9%)	0.2 (0.0 to 0.8)
Tracheal, bronchus, and lung cancer	Kiribati	1995	Age-standardized		1.0% (0.0 to 3.9%)	0.2 (0.0 to 0.7)
Tracheal, bronchus, and lung cancer	Kiribati	2000	Age-standardized		1.0% (0.0 to 4.6%)	0.2 (0.0 to 0.9)
Tracheal, bronchus, and lung cancer	Kiribati	2005	Age-standardized		0.9% (0.0 to 4.1%)	0.2 (0.0 to 0.8)
Tracheal, bronchus, and lung cancer	Kiribati	2010	Age-standardized		0.3% (0.0 to 2.5%)	0.1 (0.0 to 0.5)

Cause name	Location	Year	Age group	Number (in thousands)	PAF	Rate (per 100,000)
Tracheal, bronchus, and lung cancer	Kiribati	2015	Age-standardized		0.3% (0.0 to 2.5%)	0.1 (0.0 to 0.5)
Cardiovascular diseases	Kiribati	1990	All Ages	0.0 (0.0 to 0.0)	2.9% (0.9 to 5.3%)	6.9 (2.2 to 13.4)
Cardiovascular diseases	Kiribati	1995	All Ages	0.0 (0.0 to 0.0)	3.0% (0.9 to 5.5%)	7.1 (2.1 to 13.4)
Cardiovascular diseases	Kiribati	2000	All Ages	0.0 (0.0 to 0.0)	3.0% (0.2 to 7.4%)	7.5 (0.6 to 18.3)
Cardiovascular diseases	Kiribati	2005	All Ages	0.0 (0.0 to 0.0)	2.7% (0.2 to 7.0%)	6.6 (0.4 to 17.1)
Cardiovascular diseases	Kiribati	2010	All Ages	0.0 (0.0 to 0.0)	1.3% (0.0 to 4.5%)	3.1 (0.0 to 11.4)
Cardiovascular diseases	Kiribati	2015	All Ages	0.0 (0.0 to 0.0)	1.3% (0.0 to 4.5%)	3.4 (0.0 to 11.7)
Cardiovascular diseases	Kiribati	1990	Age-standardized		2.7% (0.9 to 5.2%)	15.3 (4.6 to 29.3)
Cardiovascular diseases	Kiribati	1995	Age-standardized		2.8% (0.7 to 5.2%)	15.5 (4.1 to 29.5)
Cardiovascular diseases	Kiribati	2000	Age-standardized		2.8% (0.2 to 6.9%)	16.3 (1.2 to 39.5)
Cardiovascular diseases	Kiribati	2005	Age-standardized		2.5% (0.1 to 6.4%)	13.9 (0.7 to 35.7)
Cardiovascular diseases	Kiribati	2010	Age-standardized		1.2% (0.0 to 4.3%)	6.2 (0.0 to 23.0)
Cardiovascular diseases	Kiribati	2015	Age-standardized		1.2% (0.0 to 4.1%)	6.4 (0.0 to 21.3)
Ischemic heart disease	Kiribati	1990	All Ages	0.0 (0.0 to 0.0)	4.7% (1.1 to 9.0%)	4.0 (0.9 to 7.6)
Ischemic heart disease	Kiribati	1995	All Ages	0.0 (0.0 to 0.0)	4.8% (1.1 to 9.1%)	4.1 (0.9 to 8.0)
Ischemic heart disease	Kiribati	2000	All Ages	0.0 (0.0 to 0.0)	4.8% (0.1 to 11.5%)	4.1 (0.1 to 9.9)
Ischemic heart disease	Kiribati	2005	All Ages	0.0 (0.0 to 0.0)	4.3% (0.0 to 11.2%)	3.7 (0.0 to 9.4)
Ischemic heart disease	Kiribati	2010	All Ages	0.0 (0.0 to 0.0)	2.0% (0.0 to 7.4%)	1.8 (0.0 to 6.5)
Ischemic heart disease	Kiribati	2015	All Ages	0.0 (0.0 to 0.0)	2.1% (0.0 to 7.8%)	2.0 (0.0 to 7.2)
Ischemic heart disease	Kiribati	1990	Age-standardized		4.3% (0.9 to 8.2%)	8.6 (1.9 to 16.8)
Ischemic heart disease	Kiribati	1995	Age-standardized		4.4% (1.0 to 8.5%)	8.8 (1.9 to 17.4)
Ischemic heart disease	Kiribati	2000	Age-standardized		4.3% (0.1 to 10.3%)	8.7 (0.2 to 20.8)
Ischemic heart disease	Kiribati	2005	Age-standardized		3.9% (0.0 to 10.1%)	7.6 (0.0 to 19.9)
Ischemic heart disease	Kiribati	2010	Age-standardized		1.8% (0.0 to 6.7%)	3.4 (0.0 to 12.5)
Ischemic heart disease	Kiribati	2015	Age-standardized		1.9% (0.0 to 7.0%)	3.6 (0.0 to 13.1)
Ischemic stroke	Kiribati	1990	All Ages	0.0 (0.0 to 0.0)	2.6% (0.5 to 5.6%)	1.1 (0.2 to 2.4)
Ischemic stroke	Kiribati	1995	All Ages	0.0 (0.0 to 0.0)	2.7% (0.5 to 5.7%)	1.1 (0.2 to 2.3)
Ischemic stroke	Kiribati	2000	All Ages	0.0 (0.0 to 0.0)	2.7% (0.1 to 7.2%)	1.3 (0.0 to 3.3)
Ischemic stroke	Kiribati	2005	All Ages	0.0 (0.0 to 0.0)	2.4% (0.0 to 6.8%)	1.1 (0.0 to 3.0)
Ischemic stroke	Kiribati	2010	All Ages	0.0 (0.0 to 0.0)	1.1% (0.0 to 4.5%)	0.5 (0.0 to 2.0)
Ischemic stroke	Kiribati	2015	All Ages	0.0 (0.0 to 0.0)	1.2% (0.0 to 4.4%)	0.5 (0.0 to 2.1)
Ischemic stroke	Kiribati	1990	Age-standardized		2.4% (0.4 to 5.6%)	2.9 (0.4 to 6.7)
Ischemic stroke	Kiribati	1995	Age-standardized		2.5% (0.3 to 5.6%)	2.8 (0.4 to 6.4)
Ischemic stroke	Kiribati	2000	Age-standardized		2.5% (0.0 to 7.0%)	3.2 (0.0 to 8.7)
Ischemic stroke	Kiribati	2005	Age-standardized		2.2% (0.0 to 6.3%)	2.6 (0.0 to 7.7)
Ischemic stroke	Kiribati	2010	Age-standardized		1.0% (0.0 to 4.3%)	1.2 (0.0 to 4.8)

Cause name	Location	Year	Age group	Number (in thousands)	PAF	Rate (per 100,000)
Ischemic stroke	Kiribati	2015	Age-standardized		1.1% (0.0 to 4.4%)	1.2 (0.0 to 4.9)
Hemorrhagic stroke	Kiribati	1990	All Ages	0.0 (0.0 to 0.0)	2.7% (0.6 to 5.5%)	1.8 (0.4 to 3.9)
Hemorrhagic stroke	Kiribati	1995	All Ages	0.0 (0.0 to 0.0)	2.8% (0.5 to 5.8%)	1.9 (0.3 to 4.0)
Hemorrhagic stroke	Kiribati	2000	All Ages	0.0 (0.0 to 0.0)	2.8% (0.1 to 7.5%)	2.2 (0.1 to 5.7)
Hemorrhagic stroke	Kiribati	2005	All Ages	0.0 (0.0 to 0.0)	2.5% (0.0 to 7.1%)	1.9 (0.0 to 5.4)
Hemorrhagic stroke	Kiribati	2010	All Ages	0.0 (0.0 to 0.0)	1.2% (0.0 to 4.7%)	0.9 (0.0 to 3.4)
Hemorrhagic stroke	Kiribati	2015	All Ages	0.0 (0.0 to 0.0)	1.2% (0.0 to 4.6%)	0.9 (0.0 to 3.4)
Hemorrhagic stroke	Kiribati	1990	Age-standardized		2.6% (0.5 to 5.6%)	3.8 (0.7 to 8.5)
Hemorrhagic stroke	Kiribati	1995	Age-standardized		2.7% (0.5 to 5.7%)	3.9 (0.7 to 8.5)
Hemorrhagic stroke	Kiribati	2000	Age-standardized		2.7% (0.1 to 7.2%)	4.4 (0.1 to 11.4)
Hemorrhagic stroke	Kiribati	2005	Age-standardized		2.4% (0.0 to 6.7%)	3.7 (0.0 to 10.7)
Hemorrhagic stroke	Kiribati	2010	Age-standardized		1.1% (0.0 to 4.5%)	1.6 (0.0 to 6.7)
Hemorrhagic stroke	Kiribati	2015	Age-standardized		1.1% (0.0 to 4.3%)	1.6 (0.0 to 6.1)
Chronic obstructive pulmonary disease	Kiribati	1990	All Ages	0.0 (0.0 to 0.0)	2.3% (0.0 to 9.0%)	0.6 (0.0 to 2.4)
Chronic obstructive pulmonary disease	Kiribati	1995	All Ages	0.0 (0.0 to 0.0)	2.5% (0.0 to 9.2%)	0.6 (0.0 to 2.1)
Chronic obstructive pulmonary disease	Kiribati	2000	All Ages	0.0 (0.0 to 0.0)	2.4% (0.0 to 10.1%)	0.4 (0.0 to 1.9)
Chronic obstructive pulmonary disease	Kiribati	2005	All Ages	0.0 (0.0 to 0.0)	2.1% (0.0 to 10.4%)	0.4 (0.0 to 2.0)
Chronic obstructive pulmonary disease	Kiribati	2010	All Ages	0.0 (0.0 to 0.0)	0.9% (0.0 to 6.4%)	0.2 (0.0 to 1.2)
Chronic obstructive pulmonary disease	Kiribati	2015	All Ages	0.0 (0.0 to 0.0)	1.0% (0.0 to 6.9%)	0.2 (0.0 to 1.3)
Chronic obstructive pulmonary disease	Kiribati	1990	Age-standardized		2.5% (0.0 to 9.7%)	1.7 (0.0 to 6.9)
Chronic obstructive pulmonary disease	Kiribati	1995	Age-standardized		2.6% (0.0 to 9.9%)	1.6 (0.0 to 5.9)
Chronic obstructive pulmonary disease	Kiribati	2000	Age-standardized		2.5% (0.0 to 10.8%)	1.2 (0.0 to 5.5)
Chronic obstructive pulmonary disease	Kiribati	2005	Age-standardized		2.3% (0.0 to 10.8%)	1.1 (0.0 to 5.5)
Chronic obstructive pulmonary disease	Kiribati	2010	Age-standardized		1.0% (0.0 to 6.8%)	0.4 (0.0 to 3.2)
Chronic obstructive pulmonary disease	Kiribati	2015	Age-standardized		1.0% (0.0 to 7.1%)	0.4 (0.0 to 3.2)
All causes	Marshall Islands	1990	All Ages	0.0 (0.0 to 0.0)	3.6% (0.1 to 7.8%)	23.3 (0.4 to 50.3)
All causes	Marshall Islands	1995	All Ages	0.0 (0.0 to 0.0)	3.7% (0.1 to 7.6%)	23.2 (0.7 to 47.5)
All causes	Marshall Islands	2000	All Ages	0.0 (0.0 to 0.0)	3.7% (0.0 to 7.8%)	24.6 (0.1 to 51.7)
All causes	Marshall Islands	2005	All Ages	0.0 (0.0 to 0.0)	3.7% (0.0 to 8.0%)	25.4 (0.0 to 55.0)
All causes	Marshall Islands	2010	All Ages	0.0 (0.0 to 0.0)	3.6% (0.0 to 7.6%)	24.5 (0.0 to 51.6)
All causes	Marshall Islands	2015	All Ages	0.0 (0.0 to 0.0)	3.7% (0.0 to 7.8%)	24.3 (0.0 to 51.1)
All causes	Marshall Islands	1990	Age-standardized		4.2% (0.1 to 9.0%)	59.3 (1.0 to 123.9)
All causes	Marshall Islands	1995	Age-standardized		4.2% (0.1 to 8.6%)	57.9 (1.8 to 117.3)
All causes	Marshall Islands	2000	Age-standardized		4.1% (0.0 to 8.5%)	58.7 (0.2 to 120.9)
All causes	Marshall Islands	2005	Age-standardized		4.0% (0.0 to 8.5%)	57.5 (0.0 to 123.6)
All causes	Marshall Islands	2010	Age-standardized		3.8% (0.0 to 7.9%)	52.8 (0.0 to 109.1)

Cause name	Location	Year	Age group	Number (in thousands)	PAF	Rate (per 100,000)
All causes	Marshall Islands	2015	Age-standardized		3.8% (0.0 to 8.0%)	49.1 (0.0 to 103.7)
Lower respiratory infections	Marshall Islands	1990	All Ages	0.0 (0.0 to 0.0)	9.6% (0.0 to 28.5%)	5.8 (0.0 to 17.6)
Lower respiratory infections	Marshall Islands	1995	All Ages	0.0 (0.0 to 0.0)	9.6% (0.0 to 27.0%)	4.7 (0.0 to 13.9)
Lower respiratory infections	Marshall Islands	2000	All Ages	0.0 (0.0 to 0.0)	10.0% (0.0 to 28.2%)	5.4 (0.0 to 16.2)
Lower respiratory infections	Marshall Islands	2005	All Ages	0.0 (0.0 to 0.0)	9.5% (0.0 to 30.7%)	4.6 (0.0 to 14.4)
Lower respiratory infections	Marshall Islands	2010	All Ages	0.0 (0.0 to 0.0)	9.2% (0.0 to 28.8%)	3.6 (0.0 to 11.8)
Lower respiratory infections	Marshall Islands	2015	All Ages	0.0 (0.0 to 0.0)	9.1% (0.0 to 28.1%)	3.1 (0.0 to 10.1)
Lower respiratory infections	Marshall Islands	1990	Age-standardized		9.6% (0.0 to 28.5%)	9.9 (0.0 to 29.9)
Lower respiratory infections	Marshall Islands	1995	Age-standardized		9.6% (0.0 to 27.0%)	8.9 (0.0 to 26.3)
Lower respiratory infections	Marshall Islands	2000	Age-standardized		10.0% (0.0 to 28.2%)	9.2 (0.0 to 27.4)
Lower respiratory infections	Marshall Islands	2005	Age-standardized		9.5% (0.0 to 30.7%)	8.2 (0.0 to 26.1)
Lower respiratory infections	Marshall Islands	2010	Age-standardized		9.2% (0.0 to 28.8%)	6.9 (0.0 to 22.2)
Lower respiratory infections	Marshall Islands	2015	Age-standardized		9.1% (0.0 to 28.1%)	6.3 (0.0 to 19.8)
Tracheal, bronchus, and lung cancer	Marshall Islands	1990	All Ages	0.0 (0.0 to 0.0)	6.6% (0.0 to 20.8%)	0.4 (0.0 to 1.4)
Tracheal, bronchus, and lung cancer	Marshall Islands	1995	All Ages	0.0 (0.0 to 0.0)	6.5% (0.0 to 18.6%)	0.5 (0.0 to 1.4)
Tracheal, bronchus, and lung cancer	Marshall Islands	2000	All Ages	0.0 (0.0 to 0.0)	6.7% (0.0 to 19.7%)	0.5 (0.0 to 1.5)
Tracheal, bronchus, and lung cancer	Marshall Islands	2005	All Ages	0.0 (0.0 to 0.0)	6.5% (0.0 to 21.0%)	0.6 (0.0 to 1.8)
Tracheal, bronchus, and lung cancer	Marshall Islands	2010	All Ages	0.0 (0.0 to 0.0)	6.3% (0.0 to 19.8%)	0.6 (0.0 to 1.9)
Tracheal, bronchus, and lung cancer	Marshall Islands	2015	All Ages	0.0 (0.0 to 0.0)	6.1% (0.0 to 20.3%)	0.6 (0.0 to 2.0)
Tracheal, bronchus, and lung cancer	Marshall Islands	1990	Age-standardized		6.7% (0.0 to 21.1%)	1.3 (0.0 to 4.0)
Tracheal, bronchus, and lung cancer	Marshall Islands	1995	Age-standardized		6.6% (0.0 to 18.9%)	1.3 (0.0 to 3.8)
Tracheal, bronchus, and lung cancer	Marshall Islands	2000	Age-standardized		6.8% (0.0 to 20.0%)	1.4 (0.0 to 4.1)
Tracheal, bronchus, and lung cancer	Marshall Islands	2005	Age-standardized		6.6% (0.0 to 21.2%)	1.4 (0.0 to 4.4)
Tracheal, bronchus, and lung cancer	Marshall Islands	2010	Age-standardized		6.4% (0.0 to 19.9%)	1.3 (0.0 to 4.3)
Tracheal, bronchus, and lung cancer	Marshall Islands	2015	Age-standardized		6.2% (0.0 to 20.4%)	1.3 (0.0 to 4.3)
Cardiovascular diseases	Marshall Islands	1990	All Ages	0.0 (0.0 to 0.0)	8.5% (0.2 to 15.5%)	14.6 (0.3 to 27.1)
Cardiovascular diseases	Marshall Islands	1995	All Ages	0.0 (0.0 to 0.0)	8.7% (0.3 to 15.6%)	15.6 (0.6 to 28.3)
Cardiovascular diseases	Marshall Islands	2000	All Ages	0.0 (0.0 to 0.0)	8.7% (0.1 to 15.9%)	16.5 (0.1 to 30.8)
Cardiovascular diseases	Marshall Islands	2005	All Ages	0.0 (0.0 to 0.0)	8.7% (0.0 to 16.4%)	18.0 (0.0 to 33.7)
Cardiovascular diseases	Marshall Islands	2010	All Ages	0.0 (0.0 to 0.0)	8.5% (0.0 to 15.9%)	18.2 (0.0 to 34.2)
Cardiovascular diseases	Marshall Islands	2015	All Ages	0.0 (0.0 to 0.0)	8.5% (0.0 to 16.0%)	18.5 (0.0 to 35.5)
Cardiovascular diseases	Marshall Islands	1990	Age-standardized		7.8% (0.2 to 14.1%)	39.8 (0.8 to 74.6)
Cardiovascular diseases	Marshall Islands	1995	Age-standardized		7.8% (0.3 to 14.0%)	40.0 (1.4 to 72.8)
Cardiovascular diseases	Marshall Islands	2000	Age-standardized		7.9% (0.0 to 14.4%)	41.3 (0.1 to 77.9)
Cardiovascular diseases	Marshall Islands	2005	Age-standardized		7.8% (0.0 to 15.0%)	41.6 (0.0 to 76.7)
Cardiovascular diseases	Marshall Islands	2010	Age-standardized		7.6% (0.0 to 14.4%)	38.9 (0.0 to 72.7)

Cause name	Location	Year	Age group	Number (in thousands)	PAF	Rate (per 100,000)
Cardiovascular diseases	Marshall Islands	2015	Age-standardized		7.6% (0.0 to 14.1%)	36.5 (0.0 to 69.7)
Ischemic heart disease	Marshall Islands	1990	All Ages	0.0 (0.0 to 0.0)	12.9% (0.2 to 23.0%)	9.5 (0.1 to 17.2)
Ischemic heart disease	Marshall Islands	1995	All Ages	0.0 (0.0 to 0.0)	13.0% (0.4 to 22.7%)	10.3 (0.4 to 18.2)
Ischemic heart disease	Marshall Islands	2000	All Ages	0.0 (0.0 to 0.0)	13.0% (0.0 to 23.1%)	11.2 (0.0 to 20.3)
Ischemic heart disease	Marshall Islands	2005	All Ages	0.0 (0.0 to 0.0)	12.7% (0.0 to 23.4%)	12.5 (0.0 to 23.2)
Ischemic heart disease	Marshall Islands	2010	All Ages	0.0 (0.0 to 0.0)	12.4% (0.0 to 22.8%)	12.9 (0.0 to 24.0)
Ischemic heart disease	Marshall Islands	2015	All Ages	0.0 (0.0 to 0.0)	12.2% (0.0 to 22.2%)	13.3 (0.0 to 25.5)
Ischemic heart disease	Marshall Islands	1990	Age-standardized		11.4% (0.1 to 20.2%)	25.0 (0.2 to 45.5)
Ischemic heart disease	Marshall Islands	1995	Age-standardized		11.4% (0.3 to 19.8%)	25.5 (0.7 to 44.7)
Ischemic heart disease	Marshall Islands	2000	Age-standardized		11.4% (0.0 to 20.6%)	27.2 (0.0 to 49.9)
Ischemic heart disease	Marshall Islands	2005	Age-standardized		11.2% (0.0 to 20.6%)	28.2 (0.0 to 52.9)
Ischemic heart disease	Marshall Islands	2010	Age-standardized		11.0% (0.0 to 20.1%)	26.8 (0.0 to 49.5)
Ischemic heart disease	Marshall Islands	2015	Age-standardized		10.8% (0.0 to 19.9%)	25.6 (0.0 to 48.5)
Ischemic stroke	Marshall Islands	1990	All Ages	0.0 (0.0 to 0.0)	8.2% (0.0 to 16.5%)	1.7 (0.0 to 3.5)
Ischemic stroke	Marshall Islands	1995	All Ages	0.0 (0.0 to 0.0)	8.2% (0.1 to 15.8%)	1.8 (0.0 to 3.6)
Ischemic stroke	Marshall Islands	2000	All Ages	0.0 (0.0 to 0.0)	8.3% (0.0 to 16.6%)	1.8 (0.0 to 3.8)
Ischemic stroke	Marshall Islands	2005	All Ages	0.0 (0.0 to 0.0)	8.0% (0.0 to 16.5%)	1.9 (0.0 to 3.9)
Ischemic stroke	Marshall Islands	2010	All Ages	0.0 (0.0 to 0.0)	7.8% (0.0 to 15.9%)	1.8 (0.0 to 3.8)
Ischemic stroke	Marshall Islands	2015	All Ages	0.0 (0.0 to 0.0)	7.6% (0.0 to 16.0%)	1.8 (0.0 to 4.0)
Ischemic stroke	Marshall Islands	1990	Age-standardized		7.4% (0.0 to 15.4%)	5.9 (0.0 to 12.5)
Ischemic stroke	Marshall Islands	1995	Age-standardized		7.4% (0.0 to 14.3%)	5.8 (0.0 to 11.5)
Ischemic stroke	Marshall Islands	2000	Age-standardized		7.4% (0.0 to 15.1%)	5.6 (0.0 to 11.5)
Ischemic stroke	Marshall Islands	2005	Age-standardized		7.3% (0.0 to 15.2%)	5.4 (0.0 to 11.5)
Ischemic stroke	Marshall Islands	2010	Age-standardized		7.0% (0.0 to 14.6%)	4.9 (0.0 to 10.4)
Ischemic stroke	Marshall Islands	2015	Age-standardized		6.9% (0.0 to 14.5%)	4.4 (0.0 to 9.8)
Hemorrhagic stroke	Marshall Islands	1990	All Ages	0.0 (0.0 to 0.0)	8.4% (0.1 to 16.8%)	3.4 (0.0 to 6.9)
Hemorrhagic stroke	Marshall Islands	1995	All Ages	0.0 (0.0 to 0.0)	8.6% (0.1 to 16.6%)	3.5 (0.0 to 6.8)
Hemorrhagic stroke	Marshall Islands	2000	All Ages	0.0 (0.0 to 0.0)	8.6% (0.0 to 17.1%)	3.6 (0.0 to 7.3)
Hemorrhagic stroke	Marshall Islands	2005	All Ages	0.0 (0.0 to 0.0)	8.5% (0.0 to 17.5%)	3.7 (0.0 to 7.5)
Hemorrhagic stroke	Marshall Islands	2010	All Ages	0.0 (0.0 to 0.0)	8.4% (0.0 to 17.3%)	3.5 (0.0 to 7.2)
Hemorrhagic stroke	Marshall Islands	2015	All Ages	0.0 (0.0 to 0.0)	8.4% (0.0 to 17.2%)	3.4 (0.0 to 7.0)
Hemorrhagic stroke	Marshall Islands	1990	Age-standardized		8.0% (0.0 to 16.2%)	8.9 (0.0 to 17.9)
Hemorrhagic stroke	Marshall Islands	1995	Age-standardized		8.0% (0.1 to 15.7%)	8.7 (0.1 to 16.6)
Hemorrhagic stroke	Marshall Islands	2000	Age-standardized		8.1% (0.0 to 16.2%)	8.5 (0.0 to 17.3)
Hemorrhagic stroke	Marshall Islands	2005	Age-standardized		7.9% (0.0 to 16.3%)	8.1 (0.0 to 17.0)
Hemorrhagic stroke	Marshall Islands	2010	Age-standardized		7.7% (0.0 to 15.8%)	7.2 (0.0 to 14.8)

Cause name	Location	Year	Age group	Number (in thousands)	PAF	Rate (per 100,000)
Hemorrhagic stroke	Marshall Islands	2015	Age-standardized		7.6% (0.0 to 15.8%)	6.5 (0.0 to 13.4)
Chronic obstructive pulmonary disease	Marshall Islands	1990	All Ages	0.0 (0.0 to 0.0)	10.1% (0.0 to 26.3%)	2.4 (0.0 to 6.4)
Chronic obstructive pulmonary disease	Marshall Islands	1995	All Ages	0.0 (0.0 to 0.0)	10.4% (0.0 to 26.4%)	2.4 (0.0 to 6.3)
Chronic obstructive pulmonary disease	Marshall Islands	2000	All Ages	0.0 (0.0 to 0.0)	10.4% (0.0 to 27.0%)	2.2 (0.0 to 5.7)
Chronic obstructive pulmonary disease	Marshall Islands	2005	All Ages	0.0 (0.0 to 0.0)	10.5% (0.0 to 28.1%)	2.2 (0.0 to 5.8)
Chronic obstructive pulmonary disease	Marshall Islands	2010	All Ages	0.0 (0.0 to 0.0)	10.3% (0.0 to 26.6%)	2.1 (0.0 to 5.7)
Chronic obstructive pulmonary disease	Marshall Islands	2015	All Ages	0.0 (0.0 to 0.0)	10.3% (0.0 to 28.4%)	2.1 (0.0 to 6.1)
Chronic obstructive pulmonary disease	Marshall Islands	1990	Age-standardized		11.1% (0.0 to 28.3%)	8.3 (0.0 to 21.9)
Chronic obstructive pulmonary disease	Marshall Islands	1995	Age-standardized		11.1% (0.0 to 28.0%)	7.7 (0.0 to 20.3)
Chronic obstructive pulmonary disease	Marshall Islands	2000	Age-standardized		11.2% (0.0 to 28.8%)	6.8 (0.0 to 17.8)
Chronic obstructive pulmonary disease	Marshall Islands	2005	Age-standardized		11.0% (0.0 to 29.3%)	6.3 (0.0 to 16.4)
Chronic obstructive pulmonary disease	Marshall Islands	2010	Age-standardized		10.6% (0.0 to 27.5%)	5.6 (0.0 to 14.8)
Chronic obstructive pulmonary disease	Marshall Islands	2015	Age-standardized		10.5% (0.0 to 29.0%)	5.1 (0.0 to 14.7)
All causes	Northern Mariana Islands	1990	All Ages	0.0 (0.0 to 0.0)	3.8% (2.2 to 5.6%)	12.5 (7.4 to 18.5)
All causes	Northern Mariana Islands	1995	All Ages	0.0 (0.0 to 0.0)	3.7% (2.1 to 5.2%)	10.1 (5.8 to 14.5)
All causes	Northern Mariana Islands	2000	All Ages	0.0 (0.0 to 0.0)	3.6% (2.1 to 5.1%)	8.5 (4.9 to 12.2)
All causes	Northern Mariana Islands	2005	All Ages	0.0 (0.0 to 0.0)	3.6% (2.3 to 5.1%)	8.3 (5.1 to 11.7)
All causes	Northern Mariana Islands	2010	All Ages	0.0 (0.0 to 0.0)	3.5% (1.9 to 4.9%)	7.9 (4.3 to 11.3)
All causes	Northern Mariana Islands	2015	All Ages	0.0 (0.0 to 0.0)	3.8% (2.5 to 5.3%)	8.9 (5.6 to 12.7)
All causes	Northern Mariana Islands	1990	Age-standardized		4.4% (2.5 to 6.5%)	42.1 (24.0 to 62.2)
All causes	Northern Mariana Islands	1995	Age-standardized		4.2% (2.4 to 6.2%)	36.0 (19.9 to 52.8)
All causes	Northern Mariana Islands	2000	Age-standardized		4.1% (2.3 to 5.9%)	32.3 (17.9 to 46.7)
All causes	Northern Mariana Islands	2005	Age-standardized		4.0% (2.4 to 5.8%)	30.6 (18.7 to 44.4)
All causes	Northern Mariana Islands	2010	Age-standardized		3.7% (2.0 to 5.3%)	27.4 (14.8 to 39.6)
All causes	Northern Mariana Islands	2015	Age-standardized		4.1% (2.6 to 5.7%)	30.3 (18.8 to 43.6)
Lower respiratory infections	Northern Mariana Islands	1990	All Ages	0.0 (0.0 to 0.0)	9.8% (1.2 to 22.7%)	1.7 (0.2 to 4.2)
Lower respiratory infections	Northern Mariana Islands	1995	All Ages	0.0 (0.0 to 0.0)	9.5% (1.2 to 21.9%)	1.4 (0.2 to 3.3)
Lower respiratory infections	Northern Mariana Islands	2000	All Ages	0.0 (0.0 to 0.0)	9.5% (0.9 to 21.5%)	1.1 (0.1 to 2.5)
Lower respiratory infections	Northern Mariana Islands	2005	All Ages	0.0 (0.0 to 0.0)	10.3% (1.7 to 23.3%)	1.0 (0.2 to 2.3)
Lower respiratory infections	Northern Mariana Islands	2010	All Ages	0.0 (0.0 to 0.0)	9.2% (1.3 to 21.4%)	0.8 (0.1 to 1.9)
Lower respiratory infections	Northern Mariana Islands	2015	All Ages	0.0 (0.0 to 0.0)	10.2% (1.9 to 23.1%)	0.9 (0.2 to 2.0)
Lower respiratory infections	Northern Mariana Islands	1990	Age-standardized		9.8% (1.2 to 22.7%)	5.5 (0.6 to 13.2)
Lower respiratory infections	Northern Mariana Islands	1995	Age-standardized		9.5% (1.2 to 21.9%)	4.6 (0.6 to 10.8)
Lower respiratory infections	Northern Mariana Islands	2000	Age-standardized		9.5% (0.9 to 21.5%)	4.1 (0.4 to 9.5)
Lower respiratory infections	Northern Mariana Islands	2005	Age-standardized		10.3% (1.7 to 23.3%)	4.0 (0.6 to 9.0)
Lower respiratory infections	Northern Mariana Islands	2010	Age-standardized		9.2% (1.3 to 21.4%)	3.3 (0.4 to 7.7)

Cause name	Location	Year	Age group	Number (in thousands)	PAF	Rate (per 100,000)
Lower respiratory infections	Northern Mariana Islands	2015	Age-standardized		10.2% (1.9 to 23.1%)	3.7 (0.6 to 8.1)
Tracheal, bronchus, and lung cancer	Northern Mariana Islands	1990	All Ages	0.0 (0.0 to 0.0)	6.6% (1.6 to 13.1%)	0.8 (0.2 to 1.7)
Tracheal, bronchus, and lung cancer	Northern Mariana Islands	1995	All Ages	0.0 (0.0 to 0.0)	6.4% (1.6 to 13.1%)	0.7 (0.2 to 1.5)
Tracheal, bronchus, and lung cancer	Northern Mariana Islands	2000	All Ages	0.0 (0.0 to 0.0)	6.4% (1.6 to 13.1%)	0.7 (0.2 to 1.4)
Tracheal, bronchus, and lung cancer	Northern Mariana Islands	2005	All Ages	0.0 (0.0 to 0.0)	7.0% (2.2 to 13.4%)	0.7 (0.2 to 1.4)
Tracheal, bronchus, and lung cancer	Northern Mariana Islands	2010	All Ages	0.0 (0.0 to 0.0)	6.2% (1.4 to 12.8%)	0.6 (0.1 to 1.3)
Tracheal, bronchus, and lung cancer	Northern Mariana Islands	2015	All Ages	0.0 (0.0 to 0.0)	7.0% (2.2 to 13.9%)	0.7 (0.2 to 1.4)
Tracheal, bronchus, and lung cancer	Northern Mariana Islands	1990	Age-standardized		6.7% (1.6 to 13.3%)	3.2 (0.8 to 6.5)
Tracheal, bronchus, and lung cancer	Northern Mariana Islands	1995	Age-standardized		6.5% (1.6 to 13.3%)	2.9 (0.7 to 5.8)
Tracheal, bronchus, and lung cancer	Northern Mariana Islands	2000	Age-standardized		6.5% (1.6 to 13.2%)	2.7 (0.7 to 5.7)
Tracheal, bronchus, and lung cancer	Northern Mariana Islands	2005	Age-standardized		7.0% (2.3 to 13.5%)	2.9 (0.9 to 5.7)
Tracheal, bronchus, and lung cancer	Northern Mariana Islands	2010	Age-standardized		6.3% (1.4 to 12.9%)	2.4 (0.5 to 5.0)
Tracheal, bronchus, and lung cancer	Northern Mariana Islands	2015	Age-standardized		7.0% (2.3 to 14.0%)	2.7 (0.9 to 5.4)
Cardiovascular diseases	Northern Mariana Islands	1990	All Ages	0.0 (0.0 to 0.0)	9.9% (6.4 to 13.3%)	8.4 (5.4 to 11.6)
Cardiovascular diseases	Northern Mariana Islands	1995	All Ages	0.0 (0.0 to 0.0)	9.7% (6.0 to 13.0%)	6.8 (4.2 to 9.2)
Cardiovascular diseases	Northern Mariana Islands	2000	All Ages	0.0 (0.0 to 0.0)	9.8% (6.3 to 13.2%)	5.8 (3.7 to 7.8)
Cardiovascular diseases	Northern Mariana Islands	2005	All Ages	0.0 (0.0 to 0.0)	10.2% (7.0 to 13.4%)	5.6 (3.8 to 7.4)
Cardiovascular diseases	Northern Mariana Islands	2010	All Ages	0.0 (0.0 to 0.0)	10.1% (6.3 to 13.4%)	5.7 (3.5 to 7.7)
Cardiovascular diseases	Northern Mariana Islands	2015	All Ages	0.0 (0.0 to 0.0)	10.8% (7.4 to 14.3%)	6.5 (4.4 to 8.8)
Cardiovascular diseases	Northern Mariana Islands	1990	Age-standardized		8.2% (5.2 to 11.1%)	27.2 (17.1 to 37.2)
Cardiovascular diseases	Northern Mariana Islands	1995	Age-standardized		7.9% (4.9 to 10.8%)	23.0 (13.9 to 31.6)
Cardiovascular diseases	Northern Mariana Islands	2000	Age-standardized		7.9% (5.0 to 10.7%)	20.7 (13.0 to 28.7)
Cardiovascular diseases	Northern Mariana Islands	2005	Age-standardized		8.1% (5.4 to 11.1%)	19.1 (12.8 to 25.9)
Cardiovascular diseases	Northern Mariana Islands	2010	Age-standardized		7.8% (4.8 to 10.6%)	18.0 (10.9 to 24.6)
Cardiovascular diseases	Northern Mariana Islands	2015	Age-standardized		8.3% (5.7 to 11.0%)	19.9 (13.6 to 27.0)
Ischemic heart disease	Northern Mariana Islands	1990	All Ages	0.0 (0.0 to 0.0)	14.7% (9.5 to 19.6%)	4.8 (3.1 to 6.7)
Ischemic heart disease	Northern Mariana Islands	1995	All Ages	0.0 (0.0 to 0.0)	14.5% (9.3 to 19.3%)	3.9 (2.4 to 5.3)
Ischemic heart disease	Northern Mariana Islands	2000	All Ages	0.0 (0.0 to 0.0)	14.4% (9.4 to 19.1%)	3.5 (2.2 to 4.7)
Ischemic heart disease	Northern Mariana Islands	2005	All Ages	0.0 (0.0 to 0.0)	15.2% (10.4 to 19.9%)	3.7 (2.5 to 4.8)
Ischemic heart disease	Northern Mariana Islands	2010	All Ages	0.0 (0.0 to 0.0)	14.9% (9.0 to 19.9%)	3.8 (2.3 to 5.2)
Ischemic heart disease	Northern Mariana Islands	2015	All Ages	0.0 (0.0 to 0.0)	15.8% (10.8 to 20.9%)	4.4 (2.9 to 6.0)
Ischemic heart disease	Northern Mariana Islands	1990	Age-standardized		11.8% (7.4 to 16.0%)	14.9 (9.3 to 20.4)
Ischemic heart disease	Northern Mariana Islands	1995	Age-standardized		11.5% (7.0 to 15.7%)	12.6 (7.6 to 17.4)
Ischemic heart disease	Northern Mariana Islands	2000	Age-standardized		11.4% (7.4 to 15.6%)	11.9 (7.6 to 16.3)
Ischemic heart disease	Northern Mariana Islands	2005	Age-standardized		11.9% (7.7 to 16.0%)	11.8 (7.7 to 16.0)
Ischemic heart disease	Northern Mariana Islands	2010	Age-standardized		11.5% (6.9 to 15.4%)	11.4 (6.8 to 15.6)

Cause name	Location	Year	Age group	Number (in thousands)	PAF	Rate (per 100,000)
Ischemic heart disease	Northern Mariana Islands	2015	Age-standardized		12.2% (8.3 to 16.3%)	12.7 (8.6 to 17.3)
Ischemic stroke	Northern Mariana Islands	1990	All Ages	0.0 (0.0 to 0.0)	9.0% (5.0 to 12.7%)	0.9 (0.5 to 1.3)
Ischemic stroke	Northern Mariana Islands	1995	All Ages	0.0 (0.0 to 0.0)	8.8% (4.9 to 12.8%)	0.7 (0.4 to 1.1)
Ischemic stroke	Northern Mariana Islands	2000	All Ages	0.0 (0.0 to 0.0)	8.8% (4.9 to 12.9%)	0.6 (0.3 to 0.9)
Ischemic stroke	Northern Mariana Islands	2005	All Ages	0.0 (0.0 to 0.0)	9.3% (5.5 to 13.5%)	0.5 (0.3 to 0.7)
Ischemic stroke	Northern Mariana Islands	2010	All Ages	0.0 (0.0 to 0.0)	9.0% (4.8 to 13.1%)	0.5 (0.2 to 0.7)
Ischemic stroke	Northern Mariana Islands	2015	All Ages	0.0 (0.0 to 0.0)	9.7% (5.9 to 13.7%)	0.5 (0.3 to 0.8)
Ischemic stroke	Northern Mariana Islands	1990	Age-standardized		7.7% (3.9 to 11.8%)	4.1 (2.1 to 6.5)
Ischemic stroke	Northern Mariana Islands	1995	Age-standardized		7.5% (3.6 to 12.0%)	3.5 (1.7 to 5.7)
Ischemic stroke	Northern Mariana Islands	2000	Age-standardized		7.5% (3.8 to 11.7%)	3.0 (1.5 to 4.9)
Ischemic stroke	Northern Mariana Islands	2005	Age-standardized		7.8% (4.1 to 12.1%)	2.5 (1.3 to 4.0)
Ischemic stroke	Northern Mariana Islands	2010	Age-standardized		7.4% (3.4 to 11.6%)	2.3 (1.0 to 3.7)
Ischemic stroke	Northern Mariana Islands	2015	Age-standardized		7.9% (4.1 to 12.0%)	2.6 (1.3 to 4.0)
Hemorrhagic stroke	Northern Mariana Islands	1990	All Ages	0.0 (0.0 to 0.0)	10.0% (5.6 to 14.2%)	2.7 (1.5 to 3.9)
Hemorrhagic stroke	Northern Mariana Islands	1995	All Ages	0.0 (0.0 to 0.0)	9.9% (5.5 to 14.2%)	2.1 (1.2 to 3.2)
Hemorrhagic stroke	Northern Mariana Islands	2000	All Ages	0.0 (0.0 to 0.0)	9.9% (5.6 to 14.3%)	1.7 (1.0 to 2.5)
Hemorrhagic stroke	Northern Mariana Islands	2005	All Ages	0.0 (0.0 to 0.0)	10.5% (6.1 to 14.9%)	1.5 (0.8 to 2.1)
Hemorrhagic stroke	Northern Mariana Islands	2010	All Ages	0.0 (0.0 to 0.0)	10.2% (5.5 to 14.6%)	1.4 (0.8 to 2.1)
Hemorrhagic stroke	Northern Mariana Islands	2015	All Ages	0.0 (0.0 to 0.0)	10.9% (6.4 to 15.2%)	1.6 (0.9 to 2.3)
Hemorrhagic stroke	Northern Mariana Islands	1990	Age-standardized		8.3% (4.5 to 12.2%)	8.1 (4.4 to 12.1)
Hemorrhagic stroke	Northern Mariana Islands	1995	Age-standardized		8.1% (4.4 to 12.2%)	6.8 (3.6 to 10.3)
Hemorrhagic stroke	Northern Mariana Islands	2000	Age-standardized		8.0% (4.4 to 12.2%)	5.7 (3.1 to 8.8)
Hemorrhagic stroke	Northern Mariana Islands	2005	Age-standardized		8.4% (4.8 to 12.5%)	4.8 (2.6 to 7.1)
Hemorrhagic stroke	Northern Mariana Islands	2010	Age-standardized		8.0% (4.1 to 12.0%)	4.3 (2.2 to 6.5)
Hemorrhagic stroke	Northern Mariana Islands	2015	Age-standardized		8.5% (4.9 to 12.3%)	4.7 (2.7 to 6.9)
Chronic obstructive pulmonary disease	Northern Mariana Islands	1990	All Ages	0.0 (0.0 to 0.0)	11.4% (3.2 to 21.8%)	1.5 (0.4 to 2.9)
Chronic obstructive pulmonary disease	Northern Mariana Islands	1995	All Ages	0.0 (0.0 to 0.0)	11.2% (2.4 to 21.8%)	1.2 (0.2 to 2.4)
Chronic obstructive pulmonary disease	Northern Mariana Islands	2000	All Ages	0.0 (0.0 to 0.0)	11.3% (2.1 to 22.2%)	1.0 (0.2 to 1.9)
Chronic obstructive pulmonary disease	Northern Mariana Islands	2005	All Ages	0.0 (0.0 to 0.0)	12.2% (3.0 to 23.0%)	0.9 (0.2 to 1.8)
Chronic obstructive pulmonary disease	Northern Mariana Islands	2010	All Ages	0.0 (0.0 to 0.0)	11.2% (2.7 to 21.6%)	0.7 (0.2 to 1.4)
Chronic obstructive pulmonary disease	Northern Mariana Islands	2015	All Ages	0.0 (0.0 to 0.0)	12.2% (3.6 to 23.2%)	0.8 (0.2 to 1.6)
Chronic obstructive pulmonary disease	Northern Mariana Islands	1990	Age-standardized		11.9% (3.3 to 22.6%)	6.3 (1.8 to 12.0)
Chronic obstructive pulmonary disease	Northern Mariana Islands	1995	Age-standardized		11.7% (2.5 to 22.7%)	5.5 (1.2 to 11.0)
Chronic obstructive pulmonary disease	Northern Mariana Islands	2000	Age-standardized		11.7% (2.2 to 22.8%)	4.8 (0.9 to 9.4)
Chronic obstructive pulmonary disease	Northern Mariana Islands	2005	Age-standardized		12.5% (3.1 to 23.6%)	4.6 (1.1 to 8.8)
Chronic obstructive pulmonary disease	Northern Mariana Islands	2010	Age-standardized		11.4% (2.8 to 22.0%)	3.7 (0.9 to 7.2)

Cause name	Location	Year	Age group	Number (in thousands)	PAF	Rate (per 100,000)
Chronic obstructive pulmonary disease	Northern Mariana Islands	2015	Age-standardized		12.5% (3.6 to 23.6%)	4.0 (1.1 to 7.8)
All causes	Papua New Guinea	1990	All Ages	2.0 (1.3 to 2.9)	4.6% (3.5 to 6.1%)	47.4 (31.5 to 68.3)
All causes	Papua New Guinea	1995	All Ages	2.1 (1.4 to 3.1)	4.6% (3.5 to 5.9%)	45.5 (30.3 to 65.7)
All causes	Papua New Guinea	2000	All Ages	2.5 (1.6 to 3.5)	4.7% (3.6 to 5.9%)	45.9 (29.4 to 65.7)
All causes	Papua New Guinea	2005	All Ages	2.6 (1.6 to 3.7)	4.3% (3.3 to 5.5%)	41.8 (26.9 to 60.2)
All causes	Papua New Guinea	2010	All Ages	2.4 (1.6 to 3.6)	3.8% (2.9 to 5.0%)	35.6 (22.6 to 53.0)
All causes	Papua New Guinea	2015	All Ages	3.0 (1.9 to 4.6)	4.5% (3.6 to 5.7%)	39.2 (24.6 to 60.5)
All causes	Papua New Guinea	1990	Age-standardized		5.6% (4.2 to 7.2%)	114.7 (76.3 to 159.4)
All causes	Papua New Guinea	1995	Age-standardized		5.5% (4.2 to 7.0%)	109.0 (71.5 to 154.8)
All causes	Papua New Guinea	2000	Age-standardized		5.5% (4.2 to 6.9%)	107.8 (69.3 to 153.4)
All causes	Papua New Guinea	2005	Age-standardized		5.0% (3.9 to 6.2%)	97.4 (63.4 to 139.2)
All causes	Papua New Guinea	2010	Age-standardized		4.3% (3.3 to 5.6%)	81.8 (53.2 to 121.9)
All causes	Papua New Guinea	2015	Age-standardized		5.0% (3.9 to 6.4%)	88.5 (57.6 to 131.0)
Lower respiratory infections	Papua New Guinea	1990	All Ages	0.7 (0.3 to 1.3)	12.3% (5.9 to 21.7%)	17.2 (8.1 to 30.9)
Lower respiratory infections	Papua New Guinea	1995	All Ages	0.7 (0.3 to 1.3)	12.3% (6.0 to 21.5%)	15.2 (7.0 to 27.7)
Lower respiratory infections	Papua New Guinea	2000	All Ages	0.8 (0.4 to 1.4)	12.8% (6.4 to 22.1%)	14.5 (6.7 to 26.2)
Lower respiratory infections	Papua New Guinea	2005	All Ages	0.7 (0.3 to 1.4)	11.2% (5.2 to 20.2%)	11.6 (5.0 to 22.9)
Lower respiratory infections	Papua New Guinea	2010	All Ages	0.6 (0.2 to 1.2)	9.3% (3.8 to 18.2%)	8.3 (3.0 to 17.7)
Lower respiratory infections	Papua New Guinea	2015	All Ages	0.6 (0.3 to 1.2)	11.2% (5.2 to 20.1%)	8.0 (3.4 to 16.2)
Lower respiratory infections	Papua New Guinea	1990	Age-standardized		12.3% (5.9 to 21.7%)	26.0 (12.3 to 47.5)
Lower respiratory infections	Papua New Guinea	1995	Age-standardized		12.3% (6.0 to 21.5%)	22.9 (10.5 to 40.9)
Lower respiratory infections	Papua New Guinea	2000	Age-standardized		12.8% (6.4 to 22.1%)	21.6 (10.2 to 38.5)
Lower respiratory infections	Papua New Guinea	2005	Age-standardized		11.2% (5.2 to 20.2%)	18.2 (7.6 to 34.7)
Lower respiratory infections	Papua New Guinea	2010	Age-standardized		9.3% (3.8 to 18.2%)	14.0 (5.2 to 28.7)
Lower respiratory infections	Papua New Guinea	2015	Age-standardized		11.2% (5.2 to 20.1%)	14.9 (6.4 to 30.1)
Tracheal, bronchus, and lung cancer	Papua New Guinea	1990	All Ages	0.0 (0.0 to 0.0)	8.3% (4.3 to 13.3%)	0.5 (0.2 to 0.9)
Tracheal, bronchus, and lung cancer	Papua New Guinea	1995	All Ages	0.0 (0.0 to 0.1)	8.3% (4.3 to 13.2%)	0.5 (0.2 to 1.0)
Tracheal, bronchus, and lung cancer	Papua New Guinea	2000	All Ages	0.0 (0.0 to 0.1)	8.6% (4.4 to 13.6%)	0.6 (0.3 to 1.1)
Tracheal, bronchus, and lung cancer	Papua New Guinea	2005	All Ages	0.0 (0.0 to 0.1)	7.5% (3.8 to 12.2%)	0.5 (0.2 to 1.0)
Tracheal, bronchus, and lung cancer	Papua New Guinea	2010	All Ages	0.0 (0.0 to 0.1)	6.2% (3.1 to 10.3%)	0.5 (0.2 to 0.9)
Tracheal, bronchus, and lung cancer	Papua New Guinea	2015	All Ages	0.1 (0.0 to 0.1)	7.6% (3.8 to 12.2%)	0.6 (0.3 to 1.1)
Tracheal, bronchus, and lung cancer	Papua New Guinea	1990	Age-standardized		8.4% (4.4 to 13.5%)	1.5 (0.7 to 2.7)
Tracheal, bronchus, and lung cancer	Papua New Guinea	1995	Age-standardized		8.4% (4.4 to 13.5%)	1.5 (0.7 to 2.7)
Tracheal, bronchus, and lung cancer	Papua New Guinea	2000	Age-standardized		8.8% (4.5 to 13.8%)	1.6 (0.7 to 2.9)
Tracheal, bronchus, and lung cancer	Papua New Guinea	2005	Age-standardized		7.6% (3.9 to 12.4%)	1.4 (0.6 to 2.6)
Tracheal, bronchus, and lung cancer	Papua New Guinea	2010	Age-standardized		6.3% (3.1 to 10.4%)	1.2 (0.5 to 2.2)

Cause name	Location	Year	Age group	Number (in thousands)	PAF	Rate (per 100,000)
Tracheal, bronchus, and lung cancer	Papua New Guinea	2015	Age-standardized		7.6% (3.9 to 12.3%)	1.4 (0.6 to 2.6)
Cardiovascular diseases	Papua New Guinea	1990	All Ages	0.8 (0.5 to 1.2)	9.8% (8.2 to 11.4%)	19.7 (12.3 to 28.9)
Cardiovascular diseases	Papua New Guinea	1995	All Ages	1.0 (0.6 to 1.5)	9.8% (8.2 to 11.4%)	20.2 (12.2 to 30.8)
Cardiovascular diseases	Papua New Guinea	2000	All Ages	1.2 (0.7 to 1.7)	10.2% (8.6 to 11.9%)	21.5 (12.9 to 32.3)
Cardiovascular diseases	Papua New Guinea	2005	All Ages	1.3 (0.8 to 1.9)	9.6% (7.9 to 11.2%)	21.2 (12.8 to 31.8)
Cardiovascular diseases	Papua New Guinea	2010	All Ages	1.3 (0.8 to 2.1)	8.6% (7.1 to 10.1%)	19.5 (11.7 to 30.3)
Cardiovascular diseases	Papua New Guinea	2015	All Ages	1.7 (1.0 to 2.5)	9.8% (8.3 to 11.4%)	21.9 (13.5 to 33.3)
Cardiovascular diseases	Papua New Guinea	1990	Age-standardized		8.9% (7.4 to 10.4%)	51.0 (33.5 to 73.1)
Cardiovascular diseases	Papua New Guinea	1995	Age-standardized		8.9% (7.3 to 10.4%)	51.2 (32.7 to 74.0)
Cardiovascular diseases	Papua New Guinea	2000	Age-standardized		9.2% (7.6 to 10.7%)	53.3 (33.3 to 76.1)
Cardiovascular diseases	Papua New Guinea	2005	Age-standardized		8.6% (7.1 to 10.1%)	50.4 (32.1 to 72.2)
Cardiovascular diseases	Papua New Guinea	2010	Age-standardized		7.7% (6.3 to 9.2%)	44.0 (28.0 to 64.9)
Cardiovascular diseases	Papua New Guinea	2015	Age-standardized		8.6% (7.2 to 10.3%)	47.0 (30.7 to 68.0)
Ischemic heart disease	Papua New Guinea	1990	All Ages	0.5 (0.3 to 0.7)	15.4% (12.5 to 18.4%)	11.0 (6.7 to 16.7)
Ischemic heart disease	Papua New Guinea	1995	All Ages	0.5 (0.3 to 0.8)	15.3% (12.2 to 18.3%)	11.4 (6.6 to 17.7)
Ischemic heart disease	Papua New Guinea	2000	All Ages	0.7 (0.4 to 1.1)	15.5% (12.4 to 18.5%)	12.7 (7.2 to 19.5)
Ischemic heart disease	Papua New Guinea	2005	All Ages	0.8 (0.5 to 1.2)	14.3% (11.3 to 17.2%)	12.9 (7.4 to 19.9)
Ischemic heart disease	Papua New Guinea	2010	All Ages	0.8 (0.5 to 1.3)	12.7% (9.9 to 15.6%)	12.3 (7.1 to 19.4)
Ischemic heart disease	Papua New Guinea	2015	All Ages	1.1 (0.6 to 1.7)	14.1% (11.2 to 17.1%)	14.0 (8.2 to 22.0)
Ischemic heart disease	Papua New Guinea	1990	Age-standardized		13.4% (10.9 to 16.0%)	27.2 (17.1 to 39.6)
Ischemic heart disease	Papua New Guinea	1995	Age-standardized		13.4% (10.7 to 16.0%)	27.6 (16.9 to 40.9)
Ischemic heart disease	Papua New Guinea	2000	Age-standardized		13.5% (10.9 to 16.2%)	30.1 (18.2 to 44.6)
Ischemic heart disease	Papua New Guinea	2005	Age-standardized		12.4% (9.9 to 15.0%)	29.7 (18.0 to 43.7)
Ischemic heart disease	Papua New Guinea	2010	Age-standardized		11.1% (8.6 to 13.5%)	26.7 (16.2 to 40.4)
Ischemic heart disease	Papua New Guinea	2015	Age-standardized		12.3% (9.8 to 14.9%)	28.9 (17.7 to 43.0)
Ischemic stroke	Papua New Guinea	1990	All Ages	0.1 (0.1 to 0.2)	9.9% (7.4 to 12.5%)	2.6 (1.5 to 4.2)
Ischemic stroke	Papua New Guinea	1995	All Ages	0.1 (0.1 to 0.2)	9.8% (7.3 to 12.5%)	2.6 (1.5 to 4.3)
Ischemic stroke	Papua New Guinea	2000	All Ages	0.1 (0.1 to 0.2)	9.9% (7.6 to 12.6%)	2.7 (1.5 to 4.3)
Ischemic stroke	Papua New Guinea	2005	All Ages	0.1 (0.1 to 0.2)	9.1% (6.8 to 11.5%)	2.5 (1.4 to 4.0)
Ischemic stroke	Papua New Guinea	2010	All Ages	0.1 (0.1 to 0.3)	8.0% (5.8 to 10.4%)	2.3 (1.3 to 3.7)
Ischemic stroke	Papua New Guinea	2015	All Ages	0.2 (0.1 to 0.3)	9.0% (6.7 to 11.4%)	2.5 (1.4 to 4.1)
Ischemic stroke	Papua New Guinea	1990	Age-standardized		8.7% (6.2 to 11.3%)	8.6 (5.0 to 13.4)
Ischemic stroke	Papua New Guinea	1995	Age-standardized		8.7% (6.2 to 11.3%)	8.6 (4.8 to 13.6)
Ischemic stroke	Papua New Guinea	2000	Age-standardized		8.8% (6.3 to 11.4%)	8.5 (4.7 to 13.1)
Ischemic stroke	Papua New Guinea	2005	Age-standardized		8.0% (5.6 to 10.7%)	7.7 (4.4 to 11.8)
Ischemic stroke	Papua New Guinea	2010	Age-standardized		7.0% (4.8 to 9.5%)	6.5 (3.7 to 10.4)

Cause name	Location	Year	Age group	Number (in thousands)	PAF	Rate (per 100,000)
Ischemic stroke	Papua New Guinea	2015	Age-standardized		7.9% (5.5 to 10.4%)	6.8 (3.9 to 10.9)
Hemorrhagic stroke	Papua New Guinea	1990	All Ages	0.3 (0.1 to 0.4)	10.0% (7.6 to 12.7%)	6.1 (3.5 to 9.5)
Hemorrhagic stroke	Papua New Guinea	1995	All Ages	0.3 (0.2 to 0.5)	10.0% (7.6 to 12.7%)	6.2 (3.5 to 9.8)
Hemorrhagic stroke	Papua New Guinea	2000	All Ages	0.3 (0.2 to 0.5)	10.2% (7.9 to 12.8%)	6.2 (3.3 to 9.6)
Hemorrhagic stroke	Papua New Guinea	2005	All Ages	0.4 (0.2 to 0.6)	9.5% (7.1 to 11.9%)	5.7 (3.1 to 8.9)
Hemorrhagic stroke	Papua New Guinea	2010	All Ages	0.3 (0.2 to 0.6)	8.4% (6.2 to 10.8%)	5.0 (2.8 to 8.2)
Hemorrhagic stroke	Papua New Guinea	2015	All Ages	0.4 (0.2 to 0.7)	9.6% (7.3 to 12.1%)	5.4 (3.0 to 8.7)
Hemorrhagic stroke	Papua New Guinea	1990	Age-standardized		9.5% (7.2 to 11.9%)	15.2 (8.8 to 23.1)
Hemorrhagic stroke	Papua New Guinea	1995	Age-standardized		9.5% (7.1 to 12.1%)	15.1 (8.5 to 23.1)
Hemorrhagic stroke	Papua New Guinea	2000	Age-standardized		9.6% (7.2 to 12.1%)	14.7 (8.2 to 22.0)
Hemorrhagic stroke	Papua New Guinea	2005	Age-standardized		8.8% (6.5 to 11.1%)	13.0 (7.3 to 19.7)
Hemorrhagic stroke	Papua New Guinea	2010	Age-standardized		7.7% (5.6 to 10.0%)	10.9 (6.4 to 17.3)
Hemorrhagic stroke	Papua New Guinea	2015	Age-standardized		8.7% (6.4 to 11.1%)	11.3 (6.5 to 17.6)
Chronic obstructive pulmonary disease	Papua New Guinea	1990	All Ages	0.4 (0.2 to 0.8)	12.5% (5.5 to 21.2%)	10.1 (4.1 to 17.9)
Chronic obstructive pulmonary disease	Papua New Guinea	1995	All Ages	0.5 (0.2 to 0.8)	12.4% (5.5 to 20.8%)	9.5 (3.8 to 17.2)
Chronic obstructive pulmonary disease	Papua New Guinea	2000	All Ages	0.5 (0.2 to 0.9)	12.5% (5.6 to 20.9%)	9.2 (3.6 to 17.0)
Chronic obstructive pulmonary disease	Papua New Guinea	2005	All Ages	0.5 (0.2 to 1.0)	11.5% (5.1 to 19.5%)	8.5 (3.3 to 15.7)
Chronic obstructive pulmonary disease	Papua New Guinea	2010	All Ages	0.5 (0.2 to 1.0)	10.1% (4.2 to 18.0%)	7.3 (2.7 to 14.1)
Chronic obstructive pulmonary disease	Papua New Guinea	2015	All Ages	0.7 (0.3 to 1.2)	12.0% (5.3 to 20.8%)	8.7 (3.4 to 16.1)
Chronic obstructive pulmonary disease	Papua New Guinea	1990	Age-standardized		13.5% (6.0 to 23.0%)	36.3 (15.2 to 63.5)
Chronic obstructive pulmonary disease	Papua New Guinea	1995	Age-standardized		13.5% (6.0 to 22.6%)	33.4 (14.0 to 61.0)
Chronic obstructive pulmonary disease	Papua New Guinea	2000	Age-standardized		13.8% (6.2 to 22.9%)	31.2 (12.7 to 56.8)
Chronic obstructive pulmonary disease	Papua New Guinea	2005	Age-standardized		12.3% (5.4 to 21.0%)	27.5 (11.1 to 49.2)
Chronic obstructive pulmonary disease	Papua New Guinea	2010	Age-standardized		10.6% (4.4 to 19.1%)	22.6 (8.4 to 43.2)
Chronic obstructive pulmonary disease	Papua New Guinea	2015	Age-standardized		12.4% (5.5 to 21.5%)	25.1 (9.9 to 47.2)
All causes	Samoa	1990	All Ages	0.0 (0.0 to 0.0)	1.1% (0.5 to 2.0%)	6.0 (2.6 to 10.8)
All causes	Samoa	1995	All Ages	0.0 (0.0 to 0.0)	1.2% (0.5 to 2.0%)	6.1 (2.6 to 10.8)
All causes	Samoa	2000	All Ages	0.0 (0.0 to 0.0)	1.2% (0.5 to 2.0%)	6.2 (2.8 to 10.7)
All causes	Samoa	2005	All Ages	0.0 (0.0 to 0.0)	1.2% (0.5 to 2.0%)	6.2 (2.9 to 10.9)
All causes	Samoa	2010	All Ages	0.0 (0.0 to 0.0)	0.5% (0.1 to 1.2%)	2.8 (0.7 to 6.3)
All causes	Samoa	2015	All Ages	0.0 (0.0 to 0.0)	0.8% (0.2 to 1.5%)	4.2 (1.3 to 8.2)
All causes	Samoa	1990	Age-standardized		1.3% (0.6 to 2.3%)	14.3 (6.1 to 26.2)
All causes	Samoa	1995	Age-standardized		1.3% (0.5 to 2.3%)	13.6 (5.8 to 24.6)
All causes	Samoa	2000	Age-standardized		1.3% (0.6 to 2.2%)	13.0 (5.5 to 22.7)
All causes	Samoa	2005	Age-standardized		1.2% (0.5 to 2.2%)	12.0 (5.3 to 21.5)
All causes	Samoa	2010	Age-standardized		0.5% (0.1 to 1.3%)	5.1 (1.3 to 12.0)

Cause name	Location	Year	Age group	Number (in thousands)	PAF	Rate (per 100,000)
All causes	Samoa	2015	Age-standardized		0.8% (0.2 to 1.6%)	7.1 (2.1 to 14.2)
Lower respiratory infections	Samoa	1990	All Ages	0.0 (0.0 to 0.0)	1.6% (0.0 to 8.4%)	0.6 (0.0 to 3.1)
Lower respiratory infections	Samoa	1995	All Ages	0.0 (0.0 to 0.0)	1.6% (0.0 to 8.2%)	0.6 (0.0 to 3.0)
Lower respiratory infections	Samoa	2000	All Ages	0.0 (0.0 to 0.0)	1.6% (0.0 to 8.3%)	0.5 (0.0 to 2.8)
Lower respiratory infections	Samoa	2005	All Ages	0.0 (0.0 to 0.0)	1.7% (0.0 to 8.6%)	0.5 (0.0 to 2.7)
Lower respiratory infections	Samoa	2010	All Ages	0.0 (0.0 to 0.0)	0.7% (0.0 to 4.5%)	0.2 (0.0 to 1.3)
Lower respiratory infections	Samoa	2015	All Ages	0.0 (0.0 to 0.0)	1.0% (0.0 to 6.4%)	0.3 (0.0 to 1.8)
Lower respiratory infections	Samoa	1990	Age-standardized		1.6% (0.0 to 8.4%)	1.2 (0.0 to 6.5)
Lower respiratory infections	Samoa	1995	Age-standardized		1.6% (0.0 to 8.2%)	1.1 (0.0 to 6.1)
Lower respiratory infections	Samoa	2000	Age-standardized		1.6% (0.0 to 8.3%)	1.1 (0.0 to 5.4)
Lower respiratory infections	Samoa	2005	Age-standardized		1.7% (0.0 to 8.6%)	1.0 (0.0 to 5.1)
Lower respiratory infections	Samoa	2010	Age-standardized		0.7% (0.0 to 4.5%)	0.4 (0.0 to 2.3)
Lower respiratory infections	Samoa	2015	Age-standardized		1.0% (0.0 to 6.4%)	0.5 (0.0 to 3.1)
Tracheal, bronchus, and lung cancer	Samoa	1990	All Ages	0.0 (0.0 to 0.0)	0.9% (0.0 to 3.3%)	0.0 (0.0 to 0.2)
Tracheal, bronchus, and lung cancer	Samoa	1995	All Ages	0.0 (0.0 to 0.0)	0.9% (0.0 to 3.5%)	0.0 (0.0 to 0.2)
Tracheal, bronchus, and lung cancer	Samoa	2000	All Ages	0.0 (0.0 to 0.0)	1.0% (0.0 to 3.5%)	0.0 (0.0 to 0.2)
Tracheal, bronchus, and lung cancer	Samoa	2005	All Ages	0.0 (0.0 to 0.0)	1.0% (0.0 to 3.5%)	0.0 (0.0 to 0.2)
Tracheal, bronchus, and lung cancer	Samoa	2010	All Ages	0.0 (0.0 to 0.0)	0.4% (0.0 to 2.1%)	0.0 (0.0 to 0.1)
Tracheal, bronchus, and lung cancer	Samoa	2015	All Ages	0.0 (0.0 to 0.0)	0.6% (0.0 to 2.6%)	0.0 (0.0 to 0.1)
Tracheal, bronchus, and lung cancer	Samoa	1990	Age-standardized		0.9% (0.0 to 3.4%)	0.1 (0.0 to 0.3)
Tracheal, bronchus, and lung cancer	Samoa	1995	Age-standardized		0.9% (0.0 to 3.6%)	0.1 (0.0 to 0.3)
Tracheal, bronchus, and lung cancer	Samoa	2000	Age-standardized		1.0% (0.0 to 3.6%)	0.1 (0.0 to 0.3)
Tracheal, bronchus, and lung cancer	Samoa	2005	Age-standardized		1.0% (0.0 to 3.5%)	0.1 (0.0 to 0.3)
Tracheal, bronchus, and lung cancer	Samoa	2010	Age-standardized		0.4% (0.0 to 2.1%)	0.0 (0.0 to 0.2)
Tracheal, bronchus, and lung cancer	Samoa	2015	Age-standardized		0.6% (0.0 to 2.6%)	0.0 (0.0 to 0.2)
Cardiovascular diseases	Samoa	1990	All Ages	0.0 (0.0 to 0.0)	2.8% (1.3 to 4.8%)	4.7 (2.1 to 8.4)
Cardiovascular diseases	Samoa	1995	All Ages	0.0 (0.0 to 0.0)	2.8% (1.3 to 4.6%)	4.9 (2.2 to 8.6)
Cardiovascular diseases	Samoa	2000	All Ages	0.0 (0.0 to 0.0)	2.8% (1.3 to 4.5%)	5.1 (2.1 to 8.5)
Cardiovascular diseases	Samoa	2005	All Ages	0.0 (0.0 to 0.0)	2.8% (1.3 to 4.5%)	5.1 (2.4 to 8.6)
Cardiovascular diseases	Samoa	2010	All Ages	0.0 (0.0 to 0.0)	1.3% (0.3 to 2.7%)	2.4 (0.6 to 5.2)
Cardiovascular diseases	Samoa	2015	All Ages	0.0 (0.0 to 0.0)	1.8% (0.6 to 3.4%)	3.6 (1.2 to 7.0)
Cardiovascular diseases	Samoa	1990	Age-standardized		2.6% (1.2 to 4.4%)	11.3 (4.6 to 20.0)
Cardiovascular diseases	Samoa	1995	Age-standardized		2.6% (1.2 to 4.5%)	10.9 (4.6 to 19.3)
Cardiovascular diseases	Samoa	2000	Age-standardized		2.7% (1.1 to 4.4%)	10.6 (4.4 to 18.5)
Cardiovascular diseases	Samoa	2005	Age-standardized		2.7% (1.2 to 4.5%)	9.9 (4.4 to 17.5)
Cardiovascular diseases	Samoa	2010	Age-standardized		1.2% (0.3 to 2.7%)	4.3 (1.0 to 9.6)

Cause name	Location	Year	Age group	Number (in thousands)	PAF	Rate (per 100,000)
Cardiovascular diseases	Samoa	2015	Age-standardized		1.7% (0.5 to 3.3%)	6.0 (1.9 to 12.2)
Ischemic heart disease	Samoa	1990	All Ages	0.0 (0.0 to 0.0)	4.2% (1.5 to 7.4%)	3.2 (1.1 to 5.9)
Ischemic heart disease	Samoa	1995	All Ages	0.0 (0.0 to 0.0)	4.2% (1.6 to 7.6%)	3.4 (1.3 to 6.3)
Ischemic heart disease	Samoa	2000	All Ages	0.0 (0.0 to 0.0)	4.2% (1.6 to 7.0%)	3.6 (1.3 to 6.1)
Ischemic heart disease	Samoa	2005	All Ages	0.0 (0.0 to 0.0)	4.1% (1.7 to 7.0%)	3.7 (1.5 to 6.5)
Ischemic heart disease	Samoa	2010	All Ages	0.0 (0.0 to 0.0)	1.9% (0.3 to 4.3%)	1.7 (0.3 to 3.9)
Ischemic heart disease	Samoa	2015	All Ages	0.0 (0.0 to 0.0)	2.6% (0.6 to 5.4%)	2.7 (0.6 to 5.8)
Ischemic heart disease	Samoa	1990	Age-standardized		3.9% (1.3 to 6.9%)	7.7 (2.5 to 14.1)
Ischemic heart disease	Samoa	1995	Age-standardized		3.9% (1.4 to 7.2%)	7.5 (2.7 to 13.8)
Ischemic heart disease	Samoa	2000	Age-standardized		3.9% (1.4 to 6.8%)	7.4 (2.6 to 13.0)
Ischemic heart disease	Samoa	2005	Age-standardized		3.9% (1.5 to 6.9%)	7.1 (2.7 to 12.7)
Ischemic heart disease	Samoa	2010	Age-standardized		1.8% (0.3 to 4.4%)	3.1 (0.5 to 7.7)
Ischemic heart disease	Samoa	2015	Age-standardized		2.5% (0.6 to 5.4%)	4.5 (1.0 to 10.1)
Ischemic stroke	Samoa	1990	All Ages	0.0 (0.0 to 0.0)	2.4% (0.7 to 5.1%)	0.5 (0.2 to 1.2)
Ischemic stroke	Samoa	1995	All Ages	0.0 (0.0 to 0.0)	2.4% (0.7 to 4.9%)	0.6 (0.2 to 1.2)
Ischemic stroke	Samoa	2000	All Ages	0.0 (0.0 to 0.0)	2.4% (0.6 to 4.9%)	0.6 (0.1 to 1.2)
Ischemic stroke	Samoa	2005	All Ages	0.0 (0.0 to 0.0)	2.4% (0.7 to 5.1%)	0.6 (0.2 to 1.2)
Ischemic stroke	Samoa	2010	All Ages	0.0 (0.0 to 0.0)	1.1% (0.1 to 3.1%)	0.2 (0.0 to 0.7)
Ischemic stroke	Samoa	2015	All Ages	0.0 (0.0 to 0.0)	1.5% (0.3 to 3.8%)	0.4 (0.1 to 1.0)
Ischemic stroke	Samoa	1990	Age-standardized		2.2% (0.6 to 4.9%)	1.5 (0.4 to 3.4)
Ischemic stroke	Samoa	1995	Age-standardized		2.3% (0.6 to 4.9%)	1.4 (0.4 to 3.1)
Ischemic stroke	Samoa	2000	Age-standardized		2.3% (0.5 to 5.1%)	1.3 (0.3 to 3.0)
Ischemic stroke	Samoa	2005	Age-standardized		2.3% (0.6 to 5.3%)	1.2 (0.3 to 2.7)
Ischemic stroke	Samoa	2010	Age-standardized		1.0% (0.1 to 3.2%)	0.5 (0.0 to 1.5)
Ischemic stroke	Samoa	2015	Age-standardized		1.4% (0.2 to 3.9%)	0.6 (0.1 to 1.8)
Hemorrhagic stroke	Samoa	1990	All Ages	0.0 (0.0 to 0.0)	2.5% (0.9 to 5.1%)	0.9 (0.3 to 1.9)
Hemorrhagic stroke	Samoa	1995	All Ages	0.0 (0.0 to 0.0)	2.5% (0.8 to 4.9%)	0.9 (0.3 to 1.9)
Hemorrhagic stroke	Samoa	2000	All Ages	0.0 (0.0 to 0.0)	2.6% (0.9 to 4.9%)	0.9 (0.3 to 1.8)
Hemorrhagic stroke	Samoa	2005	All Ages	0.0 (0.0 to 0.0)	2.6% (0.9 to 5.1%)	0.9 (0.3 to 1.8)
Hemorrhagic stroke	Samoa	2010	All Ages	0.0 (0.0 to 0.0)	1.1% (0.2 to 2.8%)	0.4 (0.1 to 1.0)
Hemorrhagic stroke	Samoa	2015	All Ages	0.0 (0.0 to 0.0)	1.6% (0.4 to 3.7%)	0.5 (0.1 to 1.3)
Hemorrhagic stroke	Samoa	1990	Age-standardized		2.4% (0.8 to 5.0%)	2.1 (0.7 to 4.6)
Hemorrhagic stroke	Samoa	1995	Age-standardized		2.4% (0.8 to 4.9%)	2.0 (0.6 to 4.2)
Hemorrhagic stroke	Samoa	2000	Age-standardized		2.5% (0.7 to 4.9%)	1.9 (0.5 to 3.9)
Hemorrhagic stroke	Samoa	2005	Age-standardized		2.5% (0.8 to 5.2%)	1.6 (0.5 to 3.6)
Hemorrhagic stroke	Samoa	2010	Age-standardized		1.1% (0.1 to 3.0%)	0.7 (0.1 to 1.9)

Cause name	Location	Year	Age group	Number (in thousands)	PAF	Rate (per 100,000)
Hemorrhagic stroke	Samoa	2015	Age-standardized		1.6% (0.3 to 3.6%)	0.9 (0.2 to 2.2)
Chronic obstructive pulmonary disease	Samoa	1990	All Ages	0.0 (0.0 to 0.0)	2.4% (0.0 to 9.1%)	0.6 (0.0 to 2.4)
Chronic obstructive pulmonary disease	Samoa	1995	All Ages	0.0 (0.0 to 0.0)	2.4% (0.0 to 9.1%)	0.6 (0.0 to 2.3)
Chronic obstructive pulmonary disease	Samoa	2000	All Ages	0.0 (0.0 to 0.0)	2.4% (0.0 to 8.9%)	0.5 (0.0 to 2.1)
Chronic obstructive pulmonary disease	Samoa	2005	All Ages	0.0 (0.0 to 0.0)	2.5% (0.0 to 9.2%)	0.5 (0.0 to 1.9)
Chronic obstructive pulmonary disease	Samoa	2010	All Ages	0.0 (0.0 to 0.0)	1.0% (0.0 to 5.6%)	0.2 (0.0 to 1.1)
Chronic obstructive pulmonary disease	Samoa	2015	All Ages	0.0 (0.0 to 0.0)	1.5% (0.0 to 7.1%)	0.3 (0.0 to 1.4)
Chronic obstructive pulmonary disease	Samoa	1990	Age-standardized		2.5% (0.0 to 9.5%)	1.7 (0.0 to 6.5)
Chronic obstructive pulmonary disease	Samoa	1995	Age-standardized		2.5% (0.0 to 9.3%)	1.5 (0.0 to 5.7)
Chronic obstructive pulmonary disease	Samoa	2000	Age-standardized		2.5% (0.0 to 9.0%)	1.2 (0.0 to 4.8)
Chronic obstructive pulmonary disease	Samoa	2005	Age-standardized		2.5% (0.0 to 9.4%)	1.0 (0.0 to 3.9)
Chronic obstructive pulmonary disease	Samoa	2010	Age-standardized		1.0% (0.0 to 5.7%)	0.4 (0.0 to 2.2)
Chronic obstructive pulmonary disease	Samoa	2015	Age-standardized		1.6% (0.0 to 7.2%)	0.5 (0.0 to 2.6)
All causes	Solomon Islands	1990	All Ages	0.1 (0.0 to 0.1)	2.3% (1.6 to 3.4%)	19.8 (11.5 to 32.2)
All causes	Solomon Islands	1995	All Ages	0.1 (0.0 to 0.1)	2.4% (1.6 to 3.6%)	18.6 (10.7 to 30.2)
All causes	Solomon Islands	2000	All Ages	0.1 (0.0 to 0.1)	2.4% (1.5 to 3.8%)	18.5 (9.9 to 32.9)
All causes	Solomon Islands	2005	All Ages	0.1 (0.1 to 0.2)	2.8% (1.9 to 4.2%)	22.6 (12.2 to 38.8)
All causes	Solomon Islands	2010	All Ages	0.1 (0.0 to 0.1)	2.0% (1.2 to 3.2%)	15.6 (8.1 to 27.5)
All causes	Solomon Islands	2015	All Ages	0.1 (0.1 to 0.2)	2.4% (1.4 to 3.7%)	17.4 (8.7 to 30.3)
All causes	Solomon Islands	1990	Age-standardized		2.8% (1.9 to 4.0%)	51.6 (30.6 to 83.5)
All causes	Solomon Islands	1995	Age-standardized		2.8% (1.9 to 4.1%)	49.0 (29.3 to 76.7)
All causes	Solomon Islands	2000	Age-standardized		2.8% (1.7 to 4.1%)	47.5 (26.1 to 81.8)
All causes	Solomon Islands	2005	Age-standardized		3.2% (2.1 to 4.7%)	54.5 (30.5 to 91.4)
All causes	Solomon Islands	2010	Age-standardized		2.2% (1.2 to 3.5%)	36.3 (19.0 to 62.8)
All causes	Solomon Islands	2015	Age-standardized		2.5% (1.5 to 3.8%)	39.1 (20.4 to 66.8)
Lower respiratory infections	Solomon Islands	1990	All Ages	0.0 (0.0 to 0.0)	5.3% (1.3 to 13.4%)	3.6 (0.9 to 9.0)
Lower respiratory infections	Solomon Islands	1995	All Ages	0.0 (0.0 to 0.0)	5.3% (1.3 to 13.0%)	3.0 (0.7 to 7.4)
Lower respiratory infections	Solomon Islands	2000	All Ages	0.0 (0.0 to 0.0)	5.2% (1.1 to 13.6%)	2.8 (0.5 to 7.7)
Lower respiratory infections	Solomon Islands	2005	All Ages	0.0 (0.0 to 0.0)	6.4% (1.7 to 15.3%)	3.3 (0.8 to 8.0)
Lower respiratory infections	Solomon Islands	2010	All Ages	0.0 (0.0 to 0.0)	4.0% (0.7 to 11.3%)	1.9 (0.3 to 5.6)
Lower respiratory infections	Solomon Islands	2015	All Ages	0.0 (0.0 to 0.0)	4.6% (0.9 to 12.9%)	1.9 (0.3 to 5.3)
Lower respiratory infections	Solomon Islands	1990	Age-standardized		5.3% (1.3 to 13.4%)	6.7 (1.6 to 16.2)
Lower respiratory infections	Solomon Islands	1995	Age-standardized		5.3% (1.3 to 13.0%)	6.1 (1.5 to 14.8)
Lower respiratory infections	Solomon Islands	2000	Age-standardized		5.2% (1.1 to 13.6%)	5.7 (1.1 to 15.8)
Lower respiratory infections	Solomon Islands	2005	Age-standardized		6.4% (1.7 to 15.3%)	6.6 (1.6 to 15.9)
Lower respiratory infections	Solomon Islands	2010	Age-standardized		4.0% (0.7 to 11.3%)	3.8 (0.6 to 11.3)

Cause name	Location	Year	Age group	Number (in thousands)	PAF	Rate (per 100,000)
Lower respiratory infections	Solomon Islands	2015	Age-standardized		4.6% (0.9 to 12.9%)	4.0 (0.7 to 11.4)
Tracheal, bronchus, and lung cancer	Solomon Islands	1990	All Ages	0.0 (0.0 to 0.0)	3.4% (1.3 to 6.9%)	0.3 (0.1 to 0.6)
Tracheal, bronchus, and lung cancer	Solomon Islands	1995	All Ages	0.0 (0.0 to 0.0)	3.5% (1.3 to 6.8%)	0.3 (0.1 to 0.6)
Tracheal, bronchus, and lung cancer	Solomon Islands	2000	All Ages	0.0 (0.0 to 0.0)	3.4% (1.2 to 7.2%)	0.3 (0.1 to 0.6)
Tracheal, bronchus, and lung cancer	Solomon Islands	2005	All Ages	0.0 (0.0 to 0.0)	4.2% (1.7 to 8.5%)	0.4 (0.1 to 0.8)
Tracheal, bronchus, and lung cancer	Solomon Islands	2010	All Ages	0.0 (0.0 to 0.0)	2.5% (0.8 to 5.6%)	0.2 (0.1 to 0.6)
Tracheal, bronchus, and lung cancer	Solomon Islands	2015	All Ages	0.0 (0.0 to 0.0)	3.0% (1.0 to 6.6%)	0.3 (0.1 to 0.7)
Tracheal, bronchus, and lung cancer	Solomon Islands	1990	Age-standardized		3.5% (1.3 to 7.0%)	0.8 (0.3 to 1.7)
Tracheal, bronchus, and lung cancer	Solomon Islands	1995	Age-standardized		3.5% (1.3 to 6.9%)	0.8 (0.3 to 1.7)
Tracheal, bronchus, and lung cancer	Solomon Islands	2000	Age-standardized		3.4% (1.2 to 7.3%)	0.8 (0.2 to 1.7)
Tracheal, bronchus, and lung cancer	Solomon Islands	2005	Age-standardized		4.2% (1.7 to 8.5%)	1.0 (0.3 to 2.2)
Tracheal, bronchus, and lung cancer	Solomon Islands	2010	Age-standardized		2.5% (0.8 to 5.7%)	0.6 (0.2 to 1.4)
Tracheal, bronchus, and lung cancer	Solomon Islands	2015	Age-standardized		3.0% (1.0 to 6.6%)	0.7 (0.2 to 1.7)
Cardiovascular diseases	Solomon Islands	1990	All Ages	0.0 (0.0 to 0.1)	5.6% (3.9 to 7.6%)	13.2 (7.7 to 21.2)
Cardiovascular diseases	Solomon Islands	1995	All Ages	0.1 (0.0 to 0.1)	5.6% (4.0 to 7.7%)	12.9 (7.2 to 20.4)
Cardiovascular diseases	Solomon Islands	2000	All Ages	0.1 (0.0 to 0.1)	5.7% (3.6 to 8.0%)	13.3 (7.0 to 22.3)
Cardiovascular diseases	Solomon Islands	2005	All Ages	0.1 (0.0 to 0.1)	6.5% (4.4 to 9.1%)	16.4 (8.9 to 27.3)
Cardiovascular diseases	Solomon Islands	2010	All Ages	0.1 (0.0 to 0.1)	4.6% (2.8 to 6.8%)	11.9 (6.0 to 20.7)
Cardiovascular diseases	Solomon Islands	2015	All Ages	0.1 (0.0 to 0.1)	5.3% (3.3 to 7.7%)	13.4 (6.8 to 23.4)
Cardiovascular diseases	Solomon Islands	1990	Age-standardized		5.1% (3.5 to 7.0%)	35.2 (21.1 to 54.5)
Cardiovascular diseases	Solomon Islands	1995	Age-standardized		5.1% (3.7 to 7.1%)	34.3 (20.3 to 52.5)
Cardiovascular diseases	Solomon Islands	2000	Age-standardized		5.2% (3.3 to 7.4%)	34.2 (18.9 to 55.5)
Cardiovascular diseases	Solomon Islands	2005	Age-standardized		5.9% (4.0 to 8.2%)	39.4 (22.3 to 63.3)
Cardiovascular diseases	Solomon Islands	2010	Age-standardized		4.2% (2.5 to 6.3%)	27.4 (13.9 to 47.6)
Cardiovascular diseases	Solomon Islands	2015	Age-standardized		4.8% (3.0 to 6.9%)	29.5 (16.0 to 50.2)
Ischemic heart disease	Solomon Islands	1990	All Ages	0.0 (0.0 to 0.0)	8.5% (5.8 to 11.8%)	8.2 (4.5 to 13.2)
Ischemic heart disease	Solomon Islands	1995	All Ages	0.0 (0.0 to 0.1)	8.5% (5.8 to 11.7%)	8.2 (4.5 to 13.5)
Ischemic heart disease	Solomon Islands	2000	All Ages	0.0 (0.0 to 0.1)	8.5% (5.3 to 12.1%)	8.6 (4.4 to 15.0)
Ischemic heart disease	Solomon Islands	2005	All Ages	0.1 (0.0 to 0.1)	9.6% (6.3 to 13.5%)	10.7 (5.6 to 17.9)
Ischemic heart disease	Solomon Islands	2010	All Ages	0.0 (0.0 to 0.1)	6.9% (3.9 to 10.4%)	7.9 (3.8 to 14.0)
Ischemic heart disease	Solomon Islands	2015	All Ages	0.1 (0.0 to 0.1)	7.8% (4.7 to 11.5%)	9.1 (4.5 to 15.8)
Ischemic heart disease	Solomon Islands	1990	Age-standardized		7.7% (5.2 to 10.6%)	21.4 (12.2 to 33.7)
Ischemic heart disease	Solomon Islands	1995	Age-standardized		7.5% (5.1 to 10.4%)	21.2 (11.9 to 33.5)
Ischemic heart disease	Solomon Islands	2000	Age-standardized		7.5% (4.7 to 10.8%)	21.7 (11.5 to 36.9)
Ischemic heart disease	Solomon Islands	2005	Age-standardized		8.5% (5.6 to 12.0%)	25.3 (13.7 to 41.4)
Ischemic heart disease	Solomon Islands	2010	Age-standardized		6.2% (3.5 to 9.3%)	18.0 (8.8 to 30.8)

Cause name	Location	Year	Age group	Number (in thousands)	PAF	Rate (per 100,000)
Ischemic heart disease	Solomon Islands	2015	Age-standardized		6.9% (4.2 to 10.3%)	19.6 (9.8 to 33.0)
Ischemic stroke	Solomon Islands	1990	All Ages	0.0 (0.0 to 0.0)	5.1% (3.2 to 7.7%)	1.6 (0.9 to 2.8)
Ischemic stroke	Solomon Islands	1995	All Ages	0.0 (0.0 to 0.0)	5.1% (3.2 to 7.6%)	1.5 (0.8 to 2.6)
Ischemic stroke	Solomon Islands	2000	All Ages	0.0 (0.0 to 0.0)	5.1% (2.9 to 7.9%)	1.5 (0.7 to 2.7)
Ischemic stroke	Solomon Islands	2005	All Ages	0.0 (0.0 to 0.0)	5.8% (3.5 to 8.7%)	1.9 (0.9 to 3.3)
Ischemic stroke	Solomon Islands	2010	All Ages	0.0 (0.0 to 0.0)	4.0% (2.2 to 6.6%)	1.3 (0.6 to 2.4)
Ischemic stroke	Solomon Islands	2015	All Ages	0.0 (0.0 to 0.0)	4.6% (2.4 to 7.2%)	1.4 (0.6 to 2.7)
Ischemic stroke	Solomon Islands	1990	Age-standardized		4.7% (2.7 to 7.4%)	5.3 (2.6 to 9.2)
Ischemic stroke	Solomon Islands	1995	Age-standardized		4.6% (2.7 to 7.3%)	5.0 (2.5 to 8.5)
Ischemic stroke	Solomon Islands	2000	Age-standardized		4.6% (2.4 to 7.4%)	4.8 (2.2 to 8.6)
Ischemic stroke	Solomon Islands	2005	Age-standardized		5.3% (3.0 to 8.3%)	5.4 (2.6 to 9.8)
Ischemic stroke	Solomon Islands	2010	Age-standardized		3.7% (1.8 to 6.4%)	3.6 (1.6 to 6.6)
Ischemic stroke	Solomon Islands	2015	Age-standardized		4.2% (2.1 to 6.8%)	3.9 (1.6 to 7.3)
Hemorrhagic stroke	Solomon Islands	1990	All Ages	0.0 (0.0 to 0.0)	5.5% (3.5 to 8.1%)	3.3 (1.8 to 5.7)
Hemorrhagic stroke	Solomon Islands	1995	All Ages	0.0 (0.0 to 0.0)	5.4% (3.5 to 8.2%)	3.2 (1.6 to 5.4)
Hemorrhagic stroke	Solomon Islands	2000	All Ages	0.0 (0.0 to 0.0)	5.5% (3.2 to 8.4%)	3.1 (1.5 to 5.7)
Hemorrhagic stroke	Solomon Islands	2005	All Ages	0.0 (0.0 to 0.0)	6.3% (3.9 to 9.4%)	3.8 (1.9 to 6.8)
Hemorrhagic stroke	Solomon Islands	2010	All Ages	0.0 (0.0 to 0.0)	4.4% (2.4 to 7.3%)	2.6 (1.2 to 4.8)
Hemorrhagic stroke	Solomon Islands	2015	All Ages	0.0 (0.0 to 0.0)	5.1% (2.9 to 8.0%)	2.9 (1.3 to 5.3)
Hemorrhagic stroke	Solomon Islands	1990	Age-standardized		5.2% (3.2 to 7.7%)	8.5 (4.5 to 14.2)
Hemorrhagic stroke	Solomon Islands	1995	Age-standardized		5.1% (3.2 to 7.6%)	8.1 (4.2 to 13.7)
Hemorrhagic stroke	Solomon Islands	2000	Age-standardized		5.1% (2.9 to 7.9%)	7.7 (3.6 to 13.7)
Hemorrhagic stroke	Solomon Islands	2005	Age-standardized		5.9% (3.4 to 8.8%)	8.7 (4.3 to 15.4)
Hemorrhagic stroke	Solomon Islands	2010	Age-standardized		4.1% (2.2 to 6.7%)	5.8 (2.7 to 10.4)
Hemorrhagic stroke	Solomon Islands	2015	Age-standardized		4.6% (2.5 to 7.4%)	6.1 (2.8 to 11.4)
Chronic obstructive pulmonary disease	Solomon Islands	1990	All Ages	0.0 (0.0 to 0.0)	6.2% (1.8 to 12.9%)	2.7 (0.8 to 6.0)
Chronic obstructive pulmonary disease	Solomon Islands	1995	All Ages	0.0 (0.0 to 0.0)	6.2% (1.8 to 13.0%)	2.4 (0.7 to 5.2)
Chronic obstructive pulmonary disease	Solomon Islands	2000	All Ages	0.0 (0.0 to 0.0)	6.1% (1.8 to 13.7%)	2.1 (0.6 to 5.0)
Chronic obstructive pulmonary disease	Solomon Islands	2005	All Ages	0.0 (0.0 to 0.0)	7.4% (2.3 to 15.6%)	2.6 (0.7 to 5.8)
Chronic obstructive pulmonary disease	Solomon Islands	2010	All Ages	0.0 (0.0 to 0.0)	4.9% (1.1 to 11.5%)	1.6 (0.3 to 4.0)
Chronic obstructive pulmonary disease	Solomon Islands	2015	All Ages	0.0 (0.0 to 0.0)	5.6% (1.6 to 13.1%)	1.8 (0.5 to 4.3)
Chronic obstructive pulmonary disease	Solomon Islands	1990	Age-standardized		6.5% (1.9 to 13.7%)	8.9 (2.5 to 19.4)
Chronic obstructive pulmonary disease	Solomon Islands	1995	Age-standardized		6.5% (1.9 to 13.7%)	7.8 (2.2 to 16.9)
Chronic obstructive pulmonary disease	Solomon Islands	2000	Age-standardized		6.5% (1.9 to 14.4%)	6.8 (1.8 to 15.8)
Chronic obstructive pulmonary disease	Solomon Islands	2005	Age-standardized		7.7% (2.4 to 16.2%)	7.5 (2.2 to 16.5)
Chronic obstructive pulmonary disease	Solomon Islands	2010	Age-standardized		5.0% (1.1 to 11.9%)	4.5 (1.0 to 11.1)

Cause name	Location	Year	Age group	Number (in thousands)	PAF	Rate (per 100,000)
Chronic obstructive pulmonary disease	Solomon Islands	2015	Age-standardized		5.8% (1.7 to 13.5%)	4.8 (1.2 to 11.3)
All causes	Tonga	1990	All Ages	0.0 (0.0 to 0.0)	1.3% (0.6 to 2.2%)	7.9 (3.5 to 13.5)
All causes	Tonga	1995	All Ages	0.0 (0.0 to 0.0)	1.3% (0.6 to 2.3%)	8.4 (3.8 to 14.4)
All causes	Tonga	2000	All Ages	0.0 (0.0 to 0.0)	1.3% (0.5 to 2.3%)	9.0 (3.5 to 15.4)
All causes	Tonga	2005	All Ages	0.0 (0.0 to 0.0)	1.4% (0.6 to 2.4%)	9.4 (3.9 to 16.6)
All causes	Tonga	2010	All Ages	0.0 (0.0 to 0.0)	0.7% (0.2 to 1.5%)	4.7 (1.2 to 10.3)
All causes	Tonga	2015	All Ages	0.0 (0.0 to 0.0)	0.8% (0.2 to 1.7%)	5.3 (1.4 to 11.4)
All causes	Tonga	1990	Age-standardized		1.5% (0.7 to 2.6%)	17.0 (7.4 to 29.0)
All causes	Tonga	1995	Age-standardized		1.4% (0.6 to 2.5%)	16.0 (6.9 to 27.9)
All causes	Tonga	2000	Age-standardized		1.5% (0.6 to 2.5%)	16.2 (6.1 to 28.1)
All causes	Tonga	2005	Age-standardized		1.5% (0.6 to 2.6%)	15.8 (6.3 to 28.2)
All causes	Tonga	2010	Age-standardized		0.7% (0.2 to 1.6%)	7.6 (1.8 to 16.8)
All causes	Tonga	2015	Age-standardized		0.8% (0.2 to 1.8%)	8.4 (2.3 to 17.7)
Lower respiratory infections	Tonga	1990	All Ages	0.0 (0.0 to 0.0)	2.4% (0.0 to 10.5%)	1.0 (0.0 to 4.4)
Lower respiratory infections	Tonga	1995	All Ages	0.0 (0.0 to 0.0)	2.3% (0.0 to 10.6%)	1.0 (0.0 to 4.7)
Lower respiratory infections	Tonga	2000	All Ages	0.0 (0.0 to 0.0)	2.4% (0.0 to 10.3%)	1.1 (0.0 to 4.8)
Lower respiratory infections	Tonga	2005	All Ages	0.0 (0.0 to 0.0)	2.3% (0.0 to 10.6%)	1.1 (0.0 to 5.0)
Lower respiratory infections	Tonga	2010	All Ages	0.0 (0.0 to 0.0)	1.0% (0.0 to 5.9%)	0.5 (0.0 to 2.9)
Lower respiratory infections	Tonga	2015	All Ages	0.0 (0.0 to 0.0)	1.2% (0.0 to 7.6%)	0.5 (0.0 to 3.4)
Lower respiratory infections	Tonga	1990	Age-standardized		2.4% (0.0 to 10.5%)	1.9 (0.0 to 9.0)
Lower respiratory infections	Tonga	1995	Age-standardized		2.3% (0.0 to 10.6%)	1.8 (0.0 to 8.4)
Lower respiratory infections	Tonga	2000	Age-standardized		2.4% (0.0 to 10.3%)	1.8 (0.0 to 8.1)
Lower respiratory infections	Tonga	2005	Age-standardized		2.3% (0.0 to 10.6%)	1.7 (0.0 to 8.0)
Lower respiratory infections	Tonga	2010	Age-standardized		1.0% (0.0 to 5.9%)	0.7 (0.0 to 4.5)
Lower respiratory infections	Tonga	2015	Age-standardized		1.2% (0.0 to 7.6%)	0.8 (0.0 to 5.1)
Tracheal, bronchus, and lung cancer	Tonga	1990	All Ages	0.0 (0.0 to 0.0)	1.4% (0.0 to 4.3%)	0.2 (0.0 to 0.7)
Tracheal, bronchus, and lung cancer	Tonga	1995	All Ages	0.0 (0.0 to 0.0)	1.4% (0.0 to 4.3%)	0.3 (0.0 to 0.8)
Tracheal, bronchus, and lung cancer	Tonga	2000	All Ages	0.0 (0.0 to 0.0)	1.4% (0.0 to 4.4%)	0.3 (0.0 to 0.8)
Tracheal, bronchus, and lung cancer	Tonga	2005	All Ages	0.0 (0.0 to 0.0)	1.4% (0.0 to 4.4%)	0.3 (0.0 to 0.9)
Tracheal, bronchus, and lung cancer	Tonga	2010	All Ages	0.0 (0.0 to 0.0)	0.6% (0.0 to 3.0%)	0.1 (0.0 to 0.6)
Tracheal, bronchus, and lung cancer	Tonga	2015	All Ages	0.0 (0.0 to 0.0)	0.7% (0.0 to 3.1%)	0.2 (0.0 to 0.6)
Tracheal, bronchus, and lung cancer	Tonga	1990	Age-standardized		1.4% (0.0 to 4.3%)	0.5 (0.0 to 1.5)
Tracheal, bronchus, and lung cancer	Tonga	1995	Age-standardized		1.4% (0.0 to 4.4%)	0.5 (0.0 to 1.5)
Tracheal, bronchus, and lung cancer	Tonga	2000	Age-standardized		1.4% (0.0 to 4.4%)	0.5 (0.0 to 1.5)
Tracheal, bronchus, and lung cancer	Tonga	2005	Age-standardized		1.4% (0.0 to 4.4%)	0.5 (0.0 to 1.6)
Tracheal, bronchus, and lung cancer	Tonga	2010	Age-standardized		0.6% (0.0 to 3.0%)	0.2 (0.0 to 1.0)

Cause name	Location	Year	Age group	Number (in thousands)	PAF	Rate (per 100,000)
Tracheal, bronchus, and lung cancer	Tonga	2015	Age-standardized		0.7% (0.0 to 3.1%)	0.2 (0.0 to 1.0)
Cardiovascular diseases	Tonga	1990	All Ages	0.0 (0.0 to 0.0)	4.0% (1.9 to 6.2%)	5.7 (2.7 to 9.2)
Cardiovascular diseases	Tonga	1995	All Ages	0.0 (0.0 to 0.0)	3.9% (1.7 to 6.2%)	6.2 (2.7 to 9.9)
Cardiovascular diseases	Tonga	2000	All Ages	0.0 (0.0 to 0.0)	3.9% (1.7 to 6.3%)	6.7 (3.0 to 11.0)
Cardiovascular diseases	Tonga	2005	All Ages	0.0 (0.0 to 0.0)	3.9% (1.7 to 6.3%)	7.0 (3.1 to 11.4)
Cardiovascular diseases	Tonga	2010	All Ages	0.0 (0.0 to 0.0)	2.0% (0.5 to 4.3%)	3.6 (0.9 to 7.7)
Cardiovascular diseases	Tonga	2015	All Ages	0.0 (0.0 to 0.0)	2.3% (0.6 to 4.7%)	4.2 (1.1 to 8.5)
Cardiovascular diseases	Tonga	1990	Age-standardized		3.8% (1.8 to 6.1%)	12.3 (5.6 to 20.2)
Cardiovascular diseases	Tonga	1995	Age-standardized		3.7% (1.5 to 5.9%)	11.7 (4.8 to 18.7)
Cardiovascular diseases	Tonga	2000	Age-standardized		3.8% (1.6 to 6.2%)	12.0 (5.0 to 20.0)
Cardiovascular diseases	Tonga	2005	Age-standardized		3.8% (1.6 to 6.3%)	11.9 (5.1 to 19.8)
Cardiovascular diseases	Tonga	2010	Age-standardized		2.0% (0.5 to 4.3%)	5.9 (1.4 to 12.7)
Cardiovascular diseases	Tonga	2015	Age-standardized		2.3% (0.6 to 4.7%)	6.5 (1.6 to 13.5)
Ischemic heart disease	Tonga	1990	All Ages	0.0 (0.0 to 0.0)	5.4% (2.4 to 9.0%)	4.1 (1.7 to 7.0)
Ischemic heart disease	Tonga	1995	All Ages	0.0 (0.0 to 0.0)	5.3% (2.2 to 8.9%)	4.4 (1.7 to 7.3)
Ischemic heart disease	Tonga	2000	All Ages	0.0 (0.0 to 0.0)	5.3% (2.0 to 9.0%)	4.8 (1.8 to 8.2)
Ischemic heart disease	Tonga	2005	All Ages	0.0 (0.0 to 0.0)	5.3% (2.0 to 8.7%)	5.1 (1.9 to 8.5)
Ischemic heart disease	Tonga	2010	All Ages	0.0 (0.0 to 0.0)	2.7% (0.6 to 5.8%)	2.7 (0.6 to 5.8)
Ischemic heart disease	Tonga	2015	All Ages	0.0 (0.0 to 0.0)	3.1% (0.6 to 6.6%)	3.1 (0.6 to 6.6)
Ischemic heart disease	Tonga	1990	Age-standardized		5.1% (2.1 to 8.5%)	8.6 (3.5 to 14.8)
Ischemic heart disease	Tonga	1995	Age-standardized		5.0% (1.9 to 8.5%)	8.3 (3.1 to 14.0)
Ischemic heart disease	Tonga	2000	Age-standardized		5.1% (1.9 to 8.7%)	8.6 (3.1 to 14.6)
Ischemic heart disease	Tonga	2005	Age-standardized		5.2% (1.8 to 8.7%)	8.6 (3.0 to 14.6)
Ischemic heart disease	Tonga	2010	Age-standardized		2.7% (0.5 to 5.8%)	4.4 (0.8 to 9.6)
Ischemic heart disease	Tonga	2015	Age-standardized		3.1% (0.6 to 6.7%)	4.8 (0.9 to 10.7)
Ischemic stroke	Tonga	1990	All Ages	0.0 (0.0 to 0.0)	3.1% (0.9 to 6.2%)	0.7 (0.2 to 1.3)
Ischemic stroke	Tonga	1995	All Ages	0.0 (0.0 to 0.0)	3.0% (0.8 to 6.0%)	0.7 (0.2 to 1.5)
Ischemic stroke	Tonga	2000	All Ages	0.0 (0.0 to 0.0)	3.0% (0.8 to 6.2%)	0.8 (0.2 to 1.6)
Ischemic stroke	Tonga	2005	All Ages	0.0 (0.0 to 0.0)	3.0% (0.8 to 5.9%)	0.8 (0.2 to 1.6)
Ischemic stroke	Tonga	2010	All Ages	0.0 (0.0 to 0.0)	1.5% (0.1 to 4.6%)	0.4 (0.0 to 1.3)
Ischemic stroke	Tonga	2015	All Ages	0.0 (0.0 to 0.0)	1.7% (0.2 to 4.8%)	0.5 (0.0 to 1.3)
Ischemic stroke	Tonga	1990	Age-standardized		3.0% (0.8 to 6.3%)	1.6 (0.4 to 3.5)
Ischemic stroke	Tonga	1995	Age-standardized		2.9% (0.7 to 6.1%)	1.5 (0.4 to 3.4)
Ischemic stroke	Tonga	2000	Age-standardized		2.9% (0.7 to 6.3%)	1.5 (0.3 to 3.2)
Ischemic stroke	Tonga	2005	Age-standardized		2.9% (0.8 to 6.2%)	1.4 (0.4 to 3.0)
Ischemic stroke	Tonga	2010	Age-standardized		1.5% (0.1 to 4.7%)	0.7 (0.1 to 2.2)

Cause name	Location	Year	Age group	Number (in thousands)	PAF	Rate (per 100,000)
Ischemic stroke	Tonga	2015	Age-standardized		1.7% (0.2 to 4.8%)	0.7 (0.1 to 2.0)
Hemorrhagic stroke	Tonga	1990	All Ages	0.0 (0.0 to 0.0)	3.2% (1.1 to 6.0%)	1.0 (0.3 to 1.9)
Hemorrhagic stroke	Tonga	1995	All Ages	0.0 (0.0 to 0.0)	3.1% (1.0 to 5.7%)	1.0 (0.3 to 2.0)
Hemorrhagic stroke	Tonga	2000	All Ages	0.0 (0.0 to 0.0)	3.2% (1.0 to 5.8%)	1.1 (0.3 to 2.1)
Hemorrhagic stroke	Tonga	2005	All Ages	0.0 (0.0 to 0.0)	3.1% (1.1 to 5.9%)	1.1 (0.4 to 2.1)
Hemorrhagic stroke	Tonga	2010	All Ages	0.0 (0.0 to 0.0)	1.6% (0.2 to 4.1%)	0.5 (0.1 to 1.5)
Hemorrhagic stroke	Tonga	2015	All Ages	0.0 (0.0 to 0.0)	1.8% (0.3 to 4.5%)	0.6 (0.1 to 1.5)
Hemorrhagic stroke	Tonga	1990	Age-standardized		3.2% (1.0 to 6.3%)	2.1 (0.7 to 4.2)
Hemorrhagic stroke	Tonga	1995	Age-standardized		3.1% (0.9 to 5.9%)	2.0 (0.6 to 4.0)
Hemorrhagic stroke	Tonga	2000	Age-standardized		3.1% (0.9 to 6.1%)	2.0 (0.6 to 4.1)
Hemorrhagic stroke	Tonga	2005	Age-standardized		3.1% (1.1 to 6.2%)	1.9 (0.6 to 3.8)
Hemorrhagic stroke	Tonga	2010	Age-standardized		1.6% (0.2 to 4.2%)	0.9 (0.1 to 2.4)
Hemorrhagic stroke	Tonga	2015	Age-standardized		1.8% (0.3 to 4.6%)	0.9 (0.1 to 2.4)
Chronic obstructive pulmonary disease	Tonga	1990	All Ages	0.0 (0.0 to 0.0)	3.4% (0.0 to 10.8%)	1.0 (0.0 to 3.2)
Chronic obstructive pulmonary disease	Tonga	1995	All Ages	0.0 (0.0 to 0.0)	3.3% (0.0 to 11.0%)	1.0 (0.0 to 3.3)
Chronic obstructive pulmonary disease	Tonga	2000	All Ages	0.0 (0.0 to 0.0)	3.4% (0.0 to 10.8%)	1.0 (0.0 to 3.3)
Chronic obstructive pulmonary disease	Tonga	2005	All Ages	0.0 (0.0 to 0.0)	3.4% (0.0 to 10.7%)	1.0 (0.0 to 3.2)
Chronic obstructive pulmonary disease	Tonga	2010	All Ages	0.0 (0.0 to 0.0)	1.6% (0.0 to 7.2%)	0.4 (0.0 to 2.0)
Chronic obstructive pulmonary disease	Tonga	2015	All Ages	0.0 (0.0 to 0.0)	1.9% (0.0 to 8.8%)	0.5 (0.0 to 2.2)
Chronic obstructive pulmonary disease	Tonga	1990	Age-standardized		3.5% (0.0 to 11.1%)	2.3 (0.0 to 7.4)
Chronic obstructive pulmonary disease	Tonga	1995	Age-standardized		3.4% (0.0 to 11.3%)	2.0 (0.0 to 6.9)
Chronic obstructive pulmonary disease	Tonga	2000	Age-standardized		3.5% (0.0 to 11.0%)	1.9 (0.0 to 6.2)
Chronic obstructive pulmonary disease	Tonga	2005	Age-standardized		3.5% (0.0 to 10.9%)	1.7 (0.0 to 5.5)
Chronic obstructive pulmonary disease	Tonga	2010	Age-standardized		1.6% (0.0 to 7.3%)	0.7 (0.0 to 3.3)
Chronic obstructive pulmonary disease	Tonga	2015	Age-standardized		1.9% (0.0 to 8.9%)	0.8 (0.0 to 3.6)
All causes	Vanuatu	1990	All Ages	0.1 (0.0 to 0.1)	3.7% (2.6 to 4.8%)	31.1 (20.0 to 47.2)
All causes	Vanuatu	1995	All Ages	0.1 (0.0 to 0.1)	3.7% (2.7 to 4.9%)	30.9 (19.4 to 47.3)
All causes	Vanuatu	2000	All Ages	0.1 (0.0 to 0.1)	3.6% (2.2 to 5.4%)	29.4 (15.6 to 49.3)
All causes	Vanuatu	2005	All Ages	0.1 (0.0 to 0.1)	3.7% (2.4 to 5.6%)	29.8 (16.6 to 49.5)
All causes	Vanuatu	2010	All Ages	0.1 (0.0 to 0.1)	2.9% (1.6 to 4.7%)	22.8 (11.2 to 41.9)
All causes	Vanuatu	2015	All Ages	0.1 (0.0 to 0.1)	3.2% (1.8 to 5.2%)	25.1 (12.5 to 46.8)
All causes	Vanuatu	1990	Age-standardized		4.2% (3.1 to 5.5%)	70.5 (45.8 to 104.8)
All causes	Vanuatu	1995	Age-standardized		4.2% (3.1 to 5.5%)	69.5 (44.0 to 102.6)
All causes	Vanuatu	2000	Age-standardized		4.1% (2.5 to 5.8%)	66.2 (36.3 to 108.3)
All causes	Vanuatu	2005	Age-standardized		4.1% (2.6 to 6.1%)	66.4 (36.9 to 107.3)
All causes	Vanuatu	2010	Age-standardized		3.1% (1.8 to 5.0%)	47.1 (23.9 to 83.0)

Cause name	Location	Year	Age group	Number (in thousands)	PAF	Rate (per 100,000)
All causes	Vanuatu	2015	Age-standardized		3.3% (1.9 to 5.4%)	48.5 (24.2 to 87.0)
Lower respiratory infections	Vanuatu	1990	All Ages	0.0 (0.0 to 0.0)	8.2% (2.8 to 17.0%)	5.5 (1.8 to 11.6)
Lower respiratory infections	Vanuatu	1995	All Ages	0.0 (0.0 to 0.0)	8.4% (3.0 to 17.1%)	5.3 (1.9 to 11.3)
Lower respiratory infections	Vanuatu	2000	All Ages	0.0 (0.0 to 0.0)	8.3% (2.5 to 17.9%)	5.4 (1.5 to 12.0)
Lower respiratory infections	Vanuatu	2005	All Ages	0.0 (0.0 to 0.0)	8.8% (2.7 to 19.1%)	5.3 (1.6 to 12.3)
Lower respiratory infections	Vanuatu	2010	All Ages	0.0 (0.0 to 0.0)	5.8% (1.1 to 15.0%)	3.2 (0.6 to 8.9)
Lower respiratory infections	Vanuatu	2015	All Ages	0.0 (0.0 to 0.0)	6.5% (1.2 to 17.1%)	3.2 (0.6 to 8.7)
Lower respiratory infections	Vanuatu	1990	Age-standardized		8.2% (2.8 to 17.0%)	9.5 (3.1 to 20.6)
Lower respiratory infections	Vanuatu	1995	Age-standardized		8.4% (3.0 to 17.1%)	9.3 (3.1 to 20.5)
Lower respiratory infections	Vanuatu	2000	Age-standardized		8.3% (2.5 to 17.9%)	9.1 (2.6 to 20.9)
Lower respiratory infections	Vanuatu	2005	Age-standardized		8.8% (2.7 to 19.1%)	9.4 (2.9 to 21.9)
Lower respiratory infections	Vanuatu	2010	Age-standardized		5.8% (1.1 to 15.0%)	5.7 (1.1 to 15.8)
Lower respiratory infections	Vanuatu	2015	Age-standardized		6.5% (1.2 to 17.1%)	5.8 (1.1 to 15.9)
Tracheal, bronchus, and lung cancer	Vanuatu	1990	All Ages	0.0 (0.0 to 0.0)	5.4% (2.4 to 9.6%)	0.5 (0.2 to 0.9)
Tracheal, bronchus, and lung cancer	Vanuatu	1995	All Ages	0.0 (0.0 to 0.0)	5.6% (2.6 to 9.6%)	0.5 (0.2 to 1.0)
Tracheal, bronchus, and lung cancer	Vanuatu	2000	All Ages	0.0 (0.0 to 0.0)	5.5% (2.2 to 11.2%)	0.5 (0.2 to 1.2)
Tracheal, bronchus, and lung cancer	Vanuatu	2005	All Ages	0.0 (0.0 to 0.0)	5.8% (2.3 to 12.1%)	0.6 (0.2 to 1.2)
Tracheal, bronchus, and lung cancer	Vanuatu	2010	All Ages	0.0 (0.0 to 0.0)	3.8% (1.2 to 8.6%)	0.4 (0.1 to 1.0)
Tracheal, bronchus, and lung cancer	Vanuatu	2015	All Ages	0.0 (0.0 to 0.0)	4.3% (1.3 to 10.6%)	0.5 (0.1 to 1.2)
Tracheal, bronchus, and lung cancer	Vanuatu	1990	Age-standardized		5.5% (2.4 to 9.6%)	1.2 (0.5 to 2.3)
Tracheal, bronchus, and lung cancer	Vanuatu	1995	Age-standardized		5.6% (2.6 to 9.6%)	1.3 (0.6 to 2.5)
Tracheal, bronchus, and lung cancer	Vanuatu	2000	Age-standardized		5.5% (2.2 to 11.3%)	1.3 (0.5 to 2.9)
Tracheal, bronchus, and lung cancer	Vanuatu	2005	Age-standardized		5.9% (2.4 to 12.2%)	1.4 (0.5 to 3.0)
Tracheal, bronchus, and lung cancer	Vanuatu	2010	Age-standardized		3.8% (1.2 to 8.7%)	0.9 (0.3 to 2.2)
Tracheal, bronchus, and lung cancer	Vanuatu	2015	Age-standardized		4.3% (1.4 to 10.6%)	1.0 (0.3 to 2.5)
Cardiovascular diseases	Vanuatu	1990	All Ages	0.0 (0.0 to 0.0)	7.3% (5.7 to 9.1%)	19.7 (12.2 to 30.1)
Cardiovascular diseases	Vanuatu	1995	All Ages	0.0 (0.0 to 0.1)	7.4% (5.7 to 9.3%)	20.1 (12.3 to 31.0)
Cardiovascular diseases	Vanuatu	2000	All Ages	0.0 (0.0 to 0.1)	7.5% (5.0 to 10.3%)	20.1 (10.9 to 33.6)
Cardiovascular diseases	Vanuatu	2005	All Ages	0.0 (0.0 to 0.1)	7.9% (5.2 to 10.8%)	20.8 (11.1 to 33.4)
Cardiovascular diseases	Vanuatu	2010	All Ages	0.0 (0.0 to 0.1)	6.2% (3.6 to 9.4%)	17.0 (8.4 to 29.6)
Cardiovascular diseases	Vanuatu	2015	All Ages	0.1 (0.0 to 0.1)	6.7% (3.9 to 10.1%)	19.0 (9.4 to 33.3)
Cardiovascular diseases	Vanuatu	1990	Age-standardized		7.0% (5.4 to 8.6%)	45.6 (29.2 to 67.6)
Cardiovascular diseases	Vanuatu	1995	Age-standardized		7.0% (5.4 to 8.9%)	46.1 (29.3 to 68.4)
Cardiovascular diseases	Vanuatu	2000	Age-standardized		7.1% (4.6 to 9.8%)	46.6 (26.2 to 74.5)
Cardiovascular diseases	Vanuatu	2005	Age-standardized		7.3% (4.6 to 10.1%)	47.2 (25.4 to 74.3)
Cardiovascular diseases	Vanuatu	2010	Age-standardized		5.7% (3.3 to 8.6%)	35.0 (17.9 to 59.0)

Cause name	Location	Year	Age group	Number<br>(in thousands)	PAF	Rate<br>(per 100,000)
Cardiovascular diseases	Vanuatu	2015	Age-standardized		6.1%<br>(3.5 to 9.3%)	36.1<br>(18.4 to 61.2)
Ischemic heart disease	Vanuatu	1990	All Ages	0.0<br>(0.0 to 0.0)	10.7%<br>(8.2 to 13.6%)	12.5<br>(7.6 to 19.4)
Ischemic heart disease	Vanuatu	1995	All Ages	0.0<br>(0.0 to 0.0)	10.8%<br>(8.1 to 13.8%)	12.8<br>(7.6 to 20.1)
Ischemic heart disease	Vanuatu	2000	All Ages	0.0<br>(0.0 to 0.0)	10.8%<br>(6.8 to 14.6%)	13.1<br>(7.0 to 21.8)
Ischemic heart disease	Vanuatu	2005	All Ages	0.0<br>(0.0 to 0.1)	11.2%<br>(7.3 to 15.4%)	13.9<br>(7.2 to 22.9)
Ischemic heart disease	Vanuatu	2010	All Ages	0.0<br>(0.0 to 0.1)	8.9%<br>(4.9 to 13.2%)	11.6<br>(5.6 to 20.7)
Ischemic heart disease	Vanuatu	2015	All Ages	0.0<br>(0.0 to 0.1)	9.4%<br>(5.2 to 14.2%)	13.0<br>(6.4 to 23.0)
Ischemic heart disease	Vanuatu	1990	Age-standardized		10.0%<br>(7.5 to 12.7%)	28.6<br>(17.7 to 43.8)
Ischemic heart disease	Vanuatu	1995	Age-standardized		10.0%<br>(7.4 to 12.8%)	29.0<br>(17.6 to 43.7)
Ischemic heart disease	Vanuatu	2000	Age-standardized		9.9%<br>(6.3 to 13.6%)	30.1<br>(16.7 to 48.7)
Ischemic heart disease	Vanuatu	2005	Age-standardized		10.1%<br>(6.4 to 13.9%)	30.9<br>(16.4 to 49.6)
Ischemic heart disease	Vanuatu	2010	Age-standardized		8.0%<br>(4.3 to 12.0%)	23.4<br>(11.9 to 40.4)
Ischemic heart disease	Vanuatu	2015	Age-standardized		8.5%<br>(4.7 to 13.0%)	24.4<br>(12.1 to 41.9)
Ischemic stroke	Vanuatu	1990	All Ages	0.0<br>(0.0 to 0.0)	6.7%<br>(4.5 to 9.1%)	2.6<br>(1.5 to 4.1)
Ischemic stroke	Vanuatu	1995	All Ages	0.0<br>(0.0 to 0.0)	6.7%<br>(4.4 to 9.4%)	2.6<br>(1.5 to 4.1)
Ischemic stroke	Vanuatu	2000	All Ages	0.0<br>(0.0 to 0.0)	6.7%<br>(3.9 to 10.2%)	2.5<br>(1.2 to 4.2)
Ischemic stroke	Vanuatu	2005	All Ages	0.0<br>(0.0 to 0.0)	7.1%<br>(4.1 to 10.8%)	2.4<br>(1.2 to 4.0)
Ischemic stroke	Vanuatu	2010	All Ages	0.0<br>(0.0 to 0.0)	5.4%<br>(2.7 to 9.1%)	1.9<br>(0.8 to 3.3)
Ischemic stroke	Vanuatu	2015	All Ages	0.0<br>(0.0 to 0.0)	5.8%<br>(3.1 to 9.5%)	2.1<br>(1.0 to 3.8)
Ischemic stroke	Vanuatu	1990	Age-standardized		6.3%<br>(4.1 to 9.0%)	6.7<br>(3.9 to 10.8)
Ischemic stroke	Vanuatu	1995	Age-standardized		6.4%<br>(4.0 to 9.0%)	6.7<br>(3.8 to 10.8)
Ischemic stroke	Vanuatu	2000	Age-standardized		6.3%<br>(3.5 to 9.7%)	6.5<br>(3.3 to 11.2)
Ischemic stroke	Vanuatu	2005	Age-standardized		6.5%<br>(3.6 to 10.1%)	6.5<br>(3.1 to 10.7)
Ischemic stroke	Vanuatu	2010	Age-standardized		4.9%<br>(2.3 to 8.5%)	4.6<br>(2.0 to 8.2)
Ischemic stroke	Vanuatu	2015	Age-standardized		5.3%<br>(2.6 to 9.0%)	4.7<br>(2.1 to 8.6)
Hemorrhagic stroke	Vanuatu	1990	All Ages	0.0<br>(0.0 to 0.0)	7.2%<br>(5.0 to 9.5%)	4.6<br>(2.6 to 7.4)
Hemorrhagic stroke	Vanuatu	1995	All Ages	0.0<br>(0.0 to 0.0)	7.3%<br>(5.1 to 9.8%)	4.7<br>(2.6 to 7.7)
Hemorrhagic stroke	Vanuatu	2000	All Ages	0.0<br>(0.0 to 0.0)	7.2%<br>(4.3 to 10.7%)	4.5<br>(2.2 to 7.8)
Hemorrhagic stroke	Vanuatu	2005	All Ages	0.0<br>(0.0 to 0.0)	7.6%<br>(4.6 to 11.4%)	4.6<br>(2.4 to 7.8)
Hemorrhagic stroke	Vanuatu	2010	All Ages	0.0<br>(0.0 to 0.0)	5.8%<br>(3.1 to 9.5%)	3.5<br>(1.6 to 6.4)
Hemorrhagic stroke	Vanuatu	2015	All Ages	0.0<br>(0.0 to 0.0)	6.3%<br>(3.4 to 10.3%)	3.9<br>(1.8 to 7.2)
Hemorrhagic stroke	Vanuatu	1990	Age-standardized		7.0%<br>(4.8 to 9.3%)	10.3<br>(5.8 to 16.1)
Hemorrhagic stroke	Vanuatu	1995	Age-standardized		7.0%<br>(4.7 to 9.7%)	10.3<br>(5.9 to 16.7)
Hemorrhagic stroke	Vanuatu	2000	Age-standardized		6.9%<br>(4.0 to 10.4%)	10.0<br>(5.1 to 17.2)
Hemorrhagic stroke	Vanuatu	2005	Age-standardized		7.1%<br>(4.2 to 10.9%)	9.8<br>(5.0 to 16.4)
Hemorrhagic stroke	Vanuatu	2010	Age-standardized		5.4%<br>(2.7 to 9.0%)	7.0<br>(3.1 to 12.3)

Cause name	Location	Year	Age group	Number (in thousands)	PAF	Rate (per 100,000)
Hemorrhagic stroke	Vanuatu	2015	Age-standardized		5.8% (3.1 to 9.6%)	7.1 (3.2 to 12.9)
Chronic obstructive pulmonary disease	Vanuatu	1990	All Ages	0.0 (0.0 to 0.0)	9.1% (3.3 to 17.1%)	5.4 (1.8 to 10.9)
Chronic obstructive pulmonary disease	Vanuatu	1995	All Ages	0.0 (0.0 to 0.0)	9.2% (3.4 to 17.0%)	4.9 (1.7 to 9.6)
Chronic obstructive pulmonary disease	Vanuatu	2000	All Ages	0.0 (0.0 to 0.0)	9.1% (2.9 to 18.1%)	3.4 (1.0 to 6.9)
Chronic obstructive pulmonary disease	Vanuatu	2005	All Ages	0.0 (0.0 to 0.0)	9.6% (3.1 to 18.8%)	3.1 (1.0 to 6.4)
Chronic obstructive pulmonary disease	Vanuatu	2010	All Ages	0.0 (0.0 to 0.0)	6.9% (1.6 to 15.4%)	2.2 (0.5 to 5.1)
Chronic obstructive pulmonary disease	Vanuatu	2015	All Ages	0.0 (0.0 to 0.0)	7.6% (2.1 to 16.6%)	2.4 (0.6 to 5.8)
Chronic obstructive pulmonary disease	Vanuatu	1990	Age-standardized		9.5% (3.5 to 17.7%)	14.1 (4.7 to 27.8)
Chronic obstructive pulmonary disease	Vanuatu	1995	Age-standardized		9.6% (3.7 to 17.8%)	12.8 (4.5 to 24.4)
Chronic obstructive pulmonary disease	Vanuatu	2000	Age-standardized		9.5% (3.0 to 18.8%)	9.1 (2.7 to 18.4)
Chronic obstructive pulmonary disease	Vanuatu	2005	Age-standardized		10.0% (3.3 to 19.8%)	8.4 (2.8 to 17.9)
Chronic obstructive pulmonary disease	Vanuatu	2010	Age-standardized		7.1% (1.7 to 16.1%)	5.5 (1.3 to 12.8)
Chronic obstructive pulmonary disease	Vanuatu	2015	Age-standardized		7.8% (2.2 to 16.9%)	5.6 (1.5 to 13.0)
All causes	Afghanistan	1990	All Ages	16.2 (11.2 to 21.3)	8.5% (6.0 to 10.9%)	131.7 (91.2 to 172.9)
All causes	Afghanistan	1995	All Ages	21.3 (15.7 to 27.0)	8.4% (6.4 to 10.3%)	128.1 (94.4 to 162.3)
All causes	Afghanistan	2000	All Ages	24.9 (18.6 to 31.2)	8.6% (6.7 to 10.4%)	126.3 (94.3 to 158.0)
All causes	Afghanistan	2005	All Ages	26.5 (21.0 to 32.4)	8.4% (7.1 to 10.0%)	108.3 (85.9 to 132.5)
All causes	Afghanistan	2010	All Ages	27.6 (22.0 to 34.6)	8.6% (7.3 to 10.0%)	98.7 (78.8 to 123.7)
All causes	Afghanistan	2015	All Ages	29.5 (23.0 to 36.8)	8.1% (6.9 to 9.5%)	90.4 (70.6 to 112.7)
All causes	Afghanistan	1990	Age-standardized		11.0% (9.6 to 12.5%)	268.5 (211.5 to 332.9)
All causes	Afghanistan	1995	Age-standardized		10.8% (9.5 to 12.3%)	271.5 (212.9 to 331.6)
All causes	Afghanistan	2000	Age-standardized		11.0% (9.6 to 12.4%)	276.6 (221.0 to 335.4)
All causes	Afghanistan	2005	Age-standardized		10.3% (9.1 to 11.7%)	249.3 (201.1 to 303.7)
All causes	Afghanistan	2010	Age-standardized		10.6% (9.3 to 12.0%)	243.7 (198.4 to 294.4)
All causes	Afghanistan	2015	Age-standardized		10.3% (9.0 to 11.6%)	232.5 (185.6 to 284.7)
Lower respiratory infections	Afghanistan	1990	All Ages	8.2 (3.6 to 12.5)	34.3% (26.4 to 41.5%)	66.8 (29.1 to 101.9)
Lower respiratory infections	Afghanistan	1995	All Ages	9.2 (4.6 to 13.2)	32.9% (25.2 to 40.1%)	55.1 (27.5 to 79.6)
Lower respiratory infections	Afghanistan	2000	All Ages	10.4 (5.5 to 14.8)	32.9% (25.1 to 40.1%)	52.6 (28.0 to 74.9)
Lower respiratory infections	Afghanistan	2005	All Ages	10.4 (6.7 to 14.2)	28.5% (20.8 to 36.1%)	42.4 (27.5 to 58.0)
Lower respiratory infections	Afghanistan	2010	All Ages	9.0 (5.9 to 12.4)	29.8% (22.2 to 37.3%)	32.0 (21.1 to 44.3)
Lower respiratory infections	Afghanistan	2015	All Ages	7.8 (5.3 to 10.9)	30.3% (22.6 to 37.6%)	24.0 (16.2 to 33.4)
Lower respiratory infections	Afghanistan	1990	Age-standardized		34.3% (26.4 to 41.5%)	49.2 (30.3 to 69.9)
Lower respiratory infections	Afghanistan	1995	Age-standardized		32.9% (25.2 to 40.1%)	45.0 (28.6 to 61.3)
Lower respiratory infections	Afghanistan	2000	Age-standardized		32.9% (25.1 to 40.1%)	42.8 (28.1 to 57.3)
Lower respiratory infections	Afghanistan	2005	Age-standardized		28.5% (20.8 to 36.1%)	36.4 (25.1 to 49.3)
Lower respiratory infections	Afghanistan	2010	Age-standardized		29.8% (22.2 to 37.3%)	32.5 (21.9 to 44.1)

Cause name	Location	Year	Age group	Number (in thousands)	PAF	Rate (per 100,000)
Lower respiratory infections	Afghanistan	2015	Age-standardized		30.3% (22.6 to 37.6%)	29.0 (20.1 to 39.2)
Tracheal, bronchus, and lung cancer	Afghanistan	1990	All Ages	0.1 (0.1 to 0.3)	25.6% (17.2 to 34.0%)	1.2 (0.6 to 2.0)
Tracheal, bronchus, and lung cancer	Afghanistan	1995	All Ages	0.2 (0.1 to 0.4)	24.3% (16.2 to 32.5%)	1.3 (0.6 to 2.2)
Tracheal, bronchus, and lung cancer	Afghanistan	2000	All Ages	0.3 (0.1 to 0.4)	24.3% (16.1 to 32.5%)	1.3 (0.6 to 2.1)
Tracheal, bronchus, and lung cancer	Afghanistan	2005	All Ages	0.3 (0.1 to 0.5)	20.5% (13.1 to 28.5%)	1.1 (0.5 to 1.9)
Tracheal, bronchus, and lung cancer	Afghanistan	2010	All Ages	0.4 (0.1 to 0.6)	21.6% (13.9 to 29.5%)	1.3 (0.5 to 2.1)
Tracheal, bronchus, and lung cancer	Afghanistan	2015	All Ages	0.5 (0.2 to 0.8)	22.0% (14.2 to 30.1%)	1.4 (0.6 to 2.4)
Tracheal, bronchus, and lung cancer	Afghanistan	1990	Age-standardized		25.8% (17.3 to 34.3%)	4.0 (2.1 to 6.3)
Tracheal, bronchus, and lung cancer	Afghanistan	1995	Age-standardized		24.5% (16.3 to 32.7%)	4.0 (2.0 to 6.4)
Tracheal, bronchus, and lung cancer	Afghanistan	2000	Age-standardized		24.5% (16.2 to 32.7%)	4.0 (2.0 to 6.4)
Tracheal, bronchus, and lung cancer	Afghanistan	2005	Age-standardized		20.7% (13.2 to 28.8%)	3.5 (1.7 to 5.8)
Tracheal, bronchus, and lung cancer	Afghanistan	2010	Age-standardized		21.8% (14.0 to 29.8%)	3.8 (1.8 to 6.2)
Tracheal, bronchus, and lung cancer	Afghanistan	2015	Age-standardized		22.2% (14.4 to 30.3%)	4.0 (1.9 to 6.6)
Cardiovascular diseases	Afghanistan	1990	All Ages	7.2 (5.4 to 9.4)	20.6% (18.3 to 23.3%)	58.3 (43.6 to 76.1)
Cardiovascular diseases	Afghanistan	1995	All Ages	10.9 (8.1 to 14.2)	20.1% (17.8 to 22.6%)	65.5 (48.6 to 85.5)
Cardiovascular diseases	Afghanistan	2000	All Ages	13.0 (9.8 to 16.7)	20.0% (17.6 to 22.4%)	65.8 (49.6 to 84.5)
Cardiovascular diseases	Afghanistan	2005	All Ages	14.4 (11.0 to 18.4)	18.4% (16.1 to 20.7%)	59.0 (45.2 to 75.2)
Cardiovascular diseases	Afghanistan	2010	All Ages	16.6 (12.6 to 21.4)	18.8% (16.5 to 21.3%)	59.4 (45.1 to 76.4)
Cardiovascular diseases	Afghanistan	2015	All Ages	19.2 (14.5 to 25.0)	18.9% (16.6 to 21.4%)	58.9 (44.6 to 76.7)
Cardiovascular diseases	Afghanistan	1990	Age-standardized		18.4% (16.1 to 20.9%)	192.6 (149.5 to 241.4)
Cardiovascular diseases	Afghanistan	1995	Age-standardized		18.1% (15.8 to 20.6%)	198.8 (153.4 to 246.5)
Cardiovascular diseases	Afghanistan	2000	Age-standardized		18.0% (15.8 to 20.5%)	203.9 (159.9 to 255.0)
Cardiovascular diseases	Afghanistan	2005	Age-standardized		16.5% (14.4 to 18.8%)	186.1 (147.7 to 228.5)
Cardiovascular diseases	Afghanistan	2010	Age-standardized		16.8% (14.6 to 19.2%)	183.9 (145.4 to 226.9)
Cardiovascular diseases	Afghanistan	2015	Age-standardized		16.9% (14.7 to 19.3%)	176.4 (137.3 to 219.0)
Ischemic heart disease	Afghanistan	1990	All Ages	5.1 (3.7 to 6.9)	24.8% (21.2 to 28.7%)	41.7 (30.2 to 56.0)
Ischemic heart disease	Afghanistan	1995	All Ages	7.6 (5.5 to 10.2)	24.2% (20.5 to 27.9%)	45.8 (33.3 to 61.4)
Ischemic heart disease	Afghanistan	2000	All Ages	8.8 (6.4 to 11.7)	24.2% (20.4 to 27.9%)	44.7 (32.5 to 59.4)
Ischemic heart disease	Afghanistan	2005	All Ages	9.7 (7.2 to 12.7)	22.5% (18.9 to 26.1%)	39.8 (29.3 to 52.0)
Ischemic heart disease	Afghanistan	2010	All Ages	11.3 (8.5 to 15.1)	22.9% (19.2 to 26.6%)	40.2 (30.5 to 53.9)
Ischemic heart disease	Afghanistan	2015	All Ages	13.0 (9.7 to 17.3)	23.0% (19.2 to 26.7%)	39.8 (29.6 to 53.0)
Ischemic heart disease	Afghanistan	1990	Age-standardized		21.9% (18.6 to 25.7%)	137.5 (102.4 to 177.8)
Ischemic heart disease	Afghanistan	1995	Age-standardized		21.5% (18.2 to 25.3%)	138.8 (103.1 to 178.4)
Ischemic heart disease	Afghanistan	2000	Age-standardized		21.6% (18.2 to 25.2%)	138.2 (103.1 to 177.7)
Ischemic heart disease	Afghanistan	2005	Age-standardized		20.0% (16.9 to 23.6%)	125.4 (95.2 to 159.9)
Ischemic heart disease	Afghanistan	2010	Age-standardized		20.4% (17.2 to 24.1%)	124.0 (94.7 to 159.1)

Cause name	Location	Year	Age group	Number (in thousands)	PAF	Rate (per 100,000)
Ischemic heart disease	Afghanistan	2015	Age-standardized		20.5% (17.0 to 24.2%)	119.0 (90.2 to 153.1)
Ischemic stroke	Afghanistan	1990	All Ages	0.9 (0.5 to 1.3)	19.4% (16.4 to 22.7%)	7.0 (4.4 to 10.5)
Ischemic stroke	Afghanistan	1995	All Ages	1.4 (0.9 to 2.0)	18.7% (15.7 to 21.8%)	8.2 (5.2 to 12.1)
Ischemic stroke	Afghanistan	2000	All Ages	1.7 (1.1 to 2.5)	18.6% (15.6 to 21.8%)	8.6 (5.6 to 12.8)
Ischemic stroke	Afghanistan	2005	All Ages	1.9 (1.3 to 2.7)	16.9% (13.9 to 19.8%)	7.8 (5.1 to 11.1)
Ischemic stroke	Afghanistan	2010	All Ages	2.2 (1.5 to 3.0)	17.2% (14.3 to 20.1%)	7.9 (5.4 to 10.9)
Ischemic stroke	Afghanistan	2015	All Ages	2.5 (1.7 to 3.4)	17.3% (14.4 to 20.3%)	7.7 (5.3 to 10.4)
Ischemic stroke	Afghanistan	1990	Age-standardized		17.0% (13.7 to 20.3%)	27.9 (17.9 to 41.4)
Ischemic stroke	Afghanistan	1995	Age-standardized		16.5% (13.3 to 19.7%)	29.8 (19.3 to 44.1)
Ischemic stroke	Afghanistan	2000	Age-standardized		16.5% (13.2 to 19.7%)	32.1 (21.0 to 47.5)
Ischemic stroke	Afghanistan	2005	Age-standardized		14.9% (11.7 to 18.1%)	29.8 (19.7 to 42.2)
Ischemic stroke	Afghanistan	2010	Age-standardized		15.3% (11.9 to 18.4%)	29.5 (20.1 to 40.6)
Ischemic stroke	Afghanistan	2015	Age-standardized		15.3% (11.9 to 18.7%)	28.2 (19.3 to 38.3)
Hemorrhagic stroke	Afghanistan	1990	All Ages	1.2 (0.8 to 1.7)	21.2% (18.2 to 24.6%)	9.7 (6.6 to 13.8)
Hemorrhagic stroke	Afghanistan	1995	All Ages	1.9 (1.3 to 2.7)	20.7% (17.6 to 23.9%)	11.5 (8.0 to 16.4)
Hemorrhagic stroke	Afghanistan	2000	All Ages	2.5 (1.7 to 3.4)	20.8% (17.6 to 23.9%)	12.5 (8.8 to 17.3)
Hemorrhagic stroke	Afghanistan	2005	All Ages	2.8 (1.9 to 4.0)	18.9% (15.9 to 22.0%)	11.4 (7.9 to 16.4)
Hemorrhagic stroke	Afghanistan	2010	All Ages	3.2 (2.2 to 4.5)	19.4% (16.4 to 22.5%)	11.3 (7.7 to 16.2)
Hemorrhagic stroke	Afghanistan	2015	All Ages	3.7 (2.5 to 5.2)	19.6% (16.6 to 22.7%)	11.3 (7.8 to 15.8)
Hemorrhagic stroke	Afghanistan	1990	Age-standardized		19.3% (16.3 to 22.5%)	27.2 (18.8 to 37.8)
Hemorrhagic stroke	Afghanistan	1995	Age-standardized		18.9% (15.9 to 21.9%)	30.2 (21.1 to 41.9)
Hemorrhagic stroke	Afghanistan	2000	Age-standardized		19.0% (16.0 to 22.0%)	33.6 (24.1 to 45.3)
Hemorrhagic stroke	Afghanistan	2005	Age-standardized		17.3% (14.4 to 20.3%)	30.8 (21.8 to 42.8)
Hemorrhagic stroke	Afghanistan	2010	Age-standardized		17.7% (14.8 to 20.6%)	30.3 (21.1 to 42.1)
Hemorrhagic stroke	Afghanistan	2015	Age-standardized		17.8% (14.8 to 20.7%)	29.3 (20.4 to 40.1)
Chronic obstructive pulmonary disease	Afghanistan	1990	All Ages	0.7 (0.3 to 1.2)	28.3% (17.7 to 40.5%)	5.3 (2.3 to 9.5)
Chronic obstructive pulmonary disease	Afghanistan	1995	All Ages	1.0 (0.5 to 1.7)	28.1% (17.4 to 40.3%)	6.1 (2.8 to 10.4)
Chronic obstructive pulmonary disease	Afghanistan	2000	All Ages	1.3 (0.6 to 2.2)	28.5% (17.7 to 40.2%)	6.6 (3.0 to 11.0)
Chronic obstructive pulmonary disease	Afghanistan	2005	All Ages	1.4 (0.7 to 2.3)	25.4% (15.0 to 36.0%)	5.8 (2.7 to 9.5)
Chronic obstructive pulmonary disease	Afghanistan	2010	All Ages	1.7 (0.9 to 2.7)	26.7% (16.3 to 37.5%)	6.0 (3.1 to 9.7)
Chronic obstructive pulmonary disease	Afghanistan	2015	All Ages	2.0 (1.1 to 3.1)	27.2% (16.5 to 38.6%)	6.1 (3.3 to 9.5)
Chronic obstructive pulmonary disease	Afghanistan	1990	Age-standardized		31.4% (19.9 to 44.5%)	22.8 (10.3 to 39.3)
Chronic obstructive pulmonary disease	Afghanistan	1995	Age-standardized		30.3% (19.0 to 43.0%)	23.7 (10.8 to 39.0)
Chronic obstructive pulmonary disease	Afghanistan	2000	Age-standardized		30.4% (19.1 to 43.1%)	26.0 (12.6 to 41.7)
Chronic obstructive pulmonary disease	Afghanistan	2005	Age-standardized		27.0% (16.0 to 38.4%)	23.3 (11.3 to 37.2)
Chronic obstructive pulmonary disease	Afghanistan	2010	Age-standardized		28.0% (17.1 to 39.7%)	23.5 (12.5 to 36.5)

Cause name	Location	Year	Age group	Number (in thousands)	PAF	Rate (per 100,000)
Chronic obstructive pulmonary disease	Afghanistan	2015	Age-standardized		28.4% (17.3 to 40.4%)	22.9 (12.6 to 35.3)
All causes	Algeria	1990	All Ages	6.8 (5.7 to 7.9)	5.2% (4.4 to 5.9%)	26.1 (22.1 to 30.2)
All causes	Algeria	1995	All Ages	7.0 (6.0 to 8.1)	5.4% (4.7 to 6.2%)	24.1 (20.6 to 27.9)
All causes	Algeria	2000	All Ages	7.6 (6.5 to 8.7)	5.9% (5.2 to 6.7%)	24.2 (20.8 to 27.8)
All causes	Algeria	2005	All Ages	8.0 (6.8 to 9.3)	5.7% (5.0 to 6.6%)	23.8 (20.3 to 27.8)
All causes	Algeria	2010	All Ages	8.8 (7.5 to 10.2)	5.5% (4.8 to 6.4%)	24.3 (20.8 to 28.2)
All causes	Algeria	2015	All Ages	12.1 (10.2 to 14.1)	6.5% (5.7 to 7.5%)	30.4 (25.8 to 35.6)
All causes	Algeria	1990	Age-standardized		7.0% (6.0 to 8.0%)	63.7 (53.9 to 73.9)
All causes	Algeria	1995	Age-standardized		6.9% (5.9 to 7.8%)	58.7 (49.5 to 68.6)
All causes	Algeria	2000	Age-standardized		6.8% (5.9 to 7.8%)	53.6 (45.5 to 62.4)
All causes	Algeria	2005	Age-standardized		6.4% (5.4 to 7.4%)	46.5 (39.4 to 55.2)
All causes	Algeria	2010	Age-standardized		6.1% (5.2 to 7.1%)	41.1 (34.8 to 48.6)
All causes	Algeria	2015	Age-standardized		7.0% (6.0 to 8.0%)	46.3 (39.0 to 54.8)
Lower respiratory infections	Algeria	1990	All Ages	1.7 (1.1 to 2.4)	23.8% (16.6 to 32.2%)	6.4 (4.2 to 9.1)
Lower respiratory infections	Algeria	1995	All Ages	1.4 (1.0 to 2.0)	23.5% (16.4 to 32.0%)	4.9 (3.3 to 6.9)
Lower respiratory infections	Algeria	2000	All Ages	1.3 (0.8 to 1.8)	23.0% (15.8 to 31.5%)	4.2 (2.7 to 5.8)
Lower respiratory infections	Algeria	2005	All Ages	1.2 (0.8 to 1.8)	21.0% (13.5 to 30.0%)	3.7 (2.3 to 5.4)
Lower respiratory infections	Algeria	2010	All Ages	1.3 (0.8 to 2.0)	19.9% (12.4 to 29.0%)	3.7 (2.2 to 5.4)
Lower respiratory infections	Algeria	2015	All Ages	1.9 (1.3 to 2.6)	24.1% (16.9 to 32.3%)	4.7 (3.2 to 6.6)
Lower respiratory infections	Algeria	1990	Age-standardized		23.8% (16.6 to 32.2%)	10.2 (6.8 to 14.6)
Lower respiratory infections	Algeria	1995	Age-standardized		23.5% (16.4 to 32.0%)	9.0 (5.9 to 12.9)
Lower respiratory infections	Algeria	2000	Age-standardized		23.0% (15.8 to 31.5%)	8.0 (5.2 to 11.3)
Lower respiratory infections	Algeria	2005	Age-standardized		21.0% (13.5 to 30.0%)	6.6 (4.1 to 9.6)
Lower respiratory infections	Algeria	2010	Age-standardized		19.9% (12.4 to 29.0%)	5.8 (3.5 to 8.6)
Lower respiratory infections	Algeria	2015	Age-standardized		24.1% (16.9 to 32.3%)	7.0 (4.7 to 9.8)
Tracheal, bronchus, and lung cancer	Algeria	1990	All Ages	0.2 (0.1 to 0.4)	17.1% (10.5 to 24.3%)	0.9 (0.5 to 1.3)
Tracheal, bronchus, and lung cancer	Algeria	1995	All Ages	0.3 (0.1 to 0.4)	16.9% (10.3 to 24.0%)	0.9 (0.5 to 1.3)
Tracheal, bronchus, and lung cancer	Algeria	2000	All Ages	0.2 (0.1 to 0.3)	16.5% (9.9 to 23.5%)	0.8 (0.5 to 1.1)
Tracheal, bronchus, and lung cancer	Algeria	2005	All Ages	0.3 (0.1 to 0.4)	14.9% (8.8 to 21.6%)	0.8 (0.5 to 1.2)
Tracheal, bronchus, and lung cancer	Algeria	2010	All Ages	0.3 (0.2 to 0.4)	14.1% (8.2 to 20.6%)	0.8 (0.5 to 1.2)
Tracheal, bronchus, and lung cancer	Algeria	2015	All Ages	0.4 (0.3 to 0.6)	17.5% (10.7 to 24.8%)	1.1 (0.7 to 1.6)
Tracheal, bronchus, and lung cancer	Algeria	1990	Age-standardized		17.2% (10.5 to 24.4%)	2.2 (1.3 to 3.2)
Tracheal, bronchus, and lung cancer	Algeria	1995	Age-standardized		16.9% (10.3 to 24.0%)	2.0 (1.2 to 2.9)
Tracheal, bronchus, and lung cancer	Algeria	2000	Age-standardized		16.5% (9.9 to 23.6%)	1.6 (0.9 to 2.2)
Tracheal, bronchus, and lung cancer	Algeria	2005	Age-standardized		14.9% (8.9 to 21.6%)	1.4 (0.8 to 2.1)
Tracheal, bronchus, and lung cancer	Algeria	2010	Age-standardized		14.2% (8.3 to 20.6%)	1.3 (0.7 to 1.9)

Cause name	Location	Year	Age group	Number (in thousands)	PAF	Rate (per 100,000)
Tracheal, bronchus, and lung cancer	Algeria	2015	Age-standardized		17.5% (10.7 to 24.8%)	1.6 (1.0 to 2.3)
Cardiovascular diseases	Algeria	1990	All Ages	4.6 (3.9 to 5.3)	12.8% (11.1 to 14.5%)	17.6 (15.0 to 20.5)
Cardiovascular diseases	Algeria	1995	All Ages	5.0 (4.2 to 5.8)	12.9% (11.2 to 14.6%)	17.2 (14.5 to 20.0)
Cardiovascular diseases	Algeria	2000	All Ages	5.7 (4.8 to 6.6)	12.8% (11.1 to 14.6%)	18.1 (15.4 to 21.1)
Cardiovascular diseases	Algeria	2005	All Ages	6.1 (5.1 to 7.2)	12.1% (10.4 to 14.0%)	18.2 (15.2 to 21.5)
Cardiovascular diseases	Algeria	2010	All Ages	6.7 (5.6 to 7.9)	11.5% (9.8 to 13.4%)	18.6 (15.6 to 21.9)
Cardiovascular diseases	Algeria	2015	All Ages	9.1 (7.5 to 10.8)	12.7% (10.8 to 14.6%)	23.0 (19.0 to 27.3)
Cardiovascular diseases	Algeria	1990	Age-standardized		12.7% (10.8 to 14.7%)	47.8 (39.7 to 56.2)
Cardiovascular diseases	Algeria	1995	Age-standardized		12.5% (10.6 to 14.6%)	44.4 (36.7 to 52.5)
Cardiovascular diseases	Algeria	2000	Age-standardized		12.3% (10.3 to 14.4%)	41.0 (34.0 to 49.1)
Cardiovascular diseases	Algeria	2005	Age-standardized		11.6% (9.7 to 13.7%)	35.9 (29.6 to 43.4)
Cardiovascular diseases	Algeria	2010	Age-standardized		11.2% (9.3 to 13.1%)	31.8 (26.1 to 38.0)
Cardiovascular diseases	Algeria	2015	Age-standardized		12.3% (10.3 to 14.4%)	35.2 (28.9 to 42.2)
Ischemic heart disease	Algeria	1990	All Ages	3.1 (2.5 to 3.6)	18.2% (15.2 to 21.3%)	11.8 (9.7 to 14.0)
Ischemic heart disease	Algeria	1995	All Ages	3.3 (2.7 to 4.0)	18.0% (15.0 to 21.0%)	11.5 (9.3 to 13.8)
Ischemic heart disease	Algeria	2000	All Ages	3.8 (3.1 to 4.5)	17.6% (14.7 to 20.8%)	12.0 (9.8 to 14.5)
Ischemic heart disease	Algeria	2005	All Ages	4.1 (3.3 to 4.9)	16.6% (13.6 to 19.8%)	12.1 (9.8 to 14.8)
Ischemic heart disease	Algeria	2010	All Ages	4.5 (3.6 to 5.4)	15.8% (12.9 to 18.8%)	12.3 (9.9 to 15.0)
Ischemic heart disease	Algeria	2015	All Ages	6.0 (4.8 to 7.3)	17.1% (13.9 to 20.6%)	15.1 (12.0 to 18.5)
Ischemic heart disease	Algeria	1990	Age-standardized		16.8% (13.5 to 20.2%)	31.9 (25.2 to 39.1)
Ischemic heart disease	Algeria	1995	Age-standardized		16.6% (13.2 to 20.0%)	29.5 (23.2 to 36.0)
Ischemic heart disease	Algeria	2000	Age-standardized		16.4% (13.1 to 19.8%)	27.1 (21.3 to 33.4)
Ischemic heart disease	Algeria	2005	Age-standardized		15.5% (12.2 to 18.9%)	23.9 (18.6 to 29.9)
Ischemic heart disease	Algeria	2010	Age-standardized		15.0% (11.9 to 18.3%)	21.0 (16.4 to 25.9)
Ischemic heart disease	Algeria	2015	Age-standardized		16.4% (13.0 to 20.0%)	22.9 (17.8 to 28.6)
Ischemic stroke	Algeria	1990	All Ages	0.7 (0.5 to 0.8)	13.2% (10.2 to 16.2%)	2.6 (1.9 to 3.3)
Ischemic stroke	Algeria	1995	All Ages	0.7 (0.6 to 0.9)	12.9% (9.9 to 16.0%)	2.6 (1.9 to 3.3)
Ischemic stroke	Algeria	2000	All Ages	0.9 (0.6 to 1.1)	12.6% (9.6 to 15.8%)	2.8 (2.0 to 3.6)
Ischemic stroke	Algeria	2005	All Ages	0.9 (0.7 to 1.2)	11.7% (8.8 to 14.8%)	2.8 (2.0 to 3.7)
Ischemic stroke	Algeria	2010	All Ages	1.1 (0.8 to 1.5)	11.2% (8.1 to 14.5%)	3.1 (2.2 to 4.1)
Ischemic stroke	Algeria	2015	All Ages	1.6 (1.1 to 2.1)	12.4% (9.2 to 16.0%)	4.0 (2.8 to 5.3)
Ischemic stroke	Algeria	1990	Age-standardized		12.3% (9.0 to 15.8%)	7.9 (5.5 to 10.5)
Ischemic stroke	Algeria	1995	Age-standardized		12.1% (8.9 to 15.6%)	7.5 (5.2 to 9.9)
Ischemic stroke	Algeria	2000	Age-standardized		11.9% (8.6 to 15.3%)	7.1 (5.0 to 9.5)
Ischemic stroke	Algeria	2005	Age-standardized		11.2% (7.8 to 14.8%)	6.1 (4.2 to 8.3)
Ischemic stroke	Algeria	2010	Age-standardized		10.8% (7.5 to 14.5%)	5.7 (3.9 to 7.9)

Cause name	Location	Year	Age group	Number (in thousands)	PAF	Rate (per 100,000)
Ischemic stroke	Algeria	2015	Age-standardized		12.1% (8.7 to 15.8%)	6.5 (4.4 to 8.8)
Hemorrhagic stroke	Algeria	1990	All Ages	0.8 (0.7 to 1.1)	13.3% (10.9 to 15.8%)	3.2 (2.5 to 4.0)
Hemorrhagic stroke	Algeria	1995	All Ages	0.9 (0.7 to 1.1)	13.6% (11.2 to 16.1%)	3.2 (2.5 to 3.9)
Hemorrhagic stroke	Algeria	2000	All Ages	1.0 (0.8 to 1.3)	13.7% (11.2 to 16.2%)	3.3 (2.6 to 4.2)
Hemorrhagic stroke	Algeria	2005	All Ages	1.1 (0.8 to 1.4)	13.0% (10.4 to 15.6%)	3.2 (2.5 to 4.1)
Hemorrhagic stroke	Algeria	2010	All Ages	1.2 (0.9 to 1.5)	12.4% (9.9 to 15.1%)	3.2 (2.5 to 4.0)
Hemorrhagic stroke	Algeria	2015	All Ages	1.6 (1.2 to 2.0)	14.0% (11.3 to 16.7%)	3.9 (3.0 to 5.0)
Hemorrhagic stroke	Algeria	1990	Age-standardized		13.6% (10.9 to 16.3%)	8.0 (6.0 to 10.3)
Hemorrhagic stroke	Algeria	1995	Age-standardized		13.5% (10.8 to 16.3%)	7.4 (5.6 to 9.5)
Hemorrhagic stroke	Algeria	2000	Age-standardized		13.3% (10.5 to 16.1%)	6.9 (5.2 to 8.9)
Hemorrhagic stroke	Algeria	2005	Age-standardized		12.4% (9.6 to 15.4%)	5.9 (4.4 to 7.5)
Hemorrhagic stroke	Algeria	2010	Age-standardized		12.0% (9.2 to 14.8%)	5.1 (3.8 to 6.6)
Hemorrhagic stroke	Algeria	2015	Age-standardized		13.4% (10.6 to 16.4%)	5.7 (4.3 to 7.4)
Chronic obstructive pulmonary disease	Algeria	1990	All Ages	0.3 (0.2 to 0.5)	21.6% (11.9 to 31.7%)	1.1 (0.6 to 1.8)
Chronic obstructive pulmonary disease	Algeria	1995	All Ages	0.3 (0.2 to 0.5)	21.9% (12.3 to 32.2%)	1.1 (0.6 to 1.7)
Chronic obstructive pulmonary disease	Algeria	2000	All Ages	0.4 (0.2 to 0.6)	21.9% (12.3 to 32.6%)	1.2 (0.6 to 1.8)
Chronic obstructive pulmonary disease	Algeria	2005	All Ages	0.4 (0.2 to 0.6)	20.6% (10.8 to 31.0%)	1.2 (0.6 to 1.8)
Chronic obstructive pulmonary disease	Algeria	2010	All Ages	0.4 (0.2 to 0.7)	19.9% (10.4 to 30.3%)	1.2 (0.6 to 1.9)
Chronic obstructive pulmonary disease	Algeria	2015	All Ages	0.6 (0.4 to 1.0)	23.4% (13.4 to 34.2%)	1.6 (0.9 to 2.4)
Chronic obstructive pulmonary disease	Algeria	1990	Age-standardized		23.1% (13.0 to 34.0%)	3.6 (1.9 to 5.5)
Chronic obstructive pulmonary disease	Algeria	1995	Age-standardized		22.9% (12.9 to 33.7%)	3.3 (1.7 to 5.1)
Chronic obstructive pulmonary disease	Algeria	2000	Age-standardized		22.6% (12.7 to 33.5%)	3.0 (1.6 to 4.6)
Chronic obstructive pulmonary disease	Algeria	2005	Age-standardized		21.0% (11.0 to 31.6%)	2.6 (1.3 to 4.0)
Chronic obstructive pulmonary disease	Algeria	2010	Age-standardized		20.2% (10.5 to 30.7%)	2.2 (1.1 to 3.5)
Chronic obstructive pulmonary disease	Algeria	2015	Age-standardized		23.6% (13.5 to 34.4%)	2.6 (1.5 to 4.0)
All causes	Bahrain	1990	All Ages	0.2 (0.1 to 0.2)	9.9% (8.6 to 11.1%)	36.0 (30.6 to 42.0)
All causes	Bahrain	1995	All Ages	0.2 (0.1 to 0.2)	9.3% (8.1 to 10.4%)	31.8 (27.1 to 37.2)
All causes	Bahrain	2000	All Ages	0.2 (0.2 to 0.2)	8.6% (7.5 to 9.7%)	28.6 (24.7 to 33.3)
All causes	Bahrain	2005	All Ages	0.2 (0.1 to 0.2)	7.9% (6.9 to 9.0%)	20.3 (17.5 to 24.0)
All causes	Bahrain	2010	All Ages	0.2 (0.2 to 0.2)	8.1% (7.1 to 9.1%)	16.3 (13.8 to 19.0)
All causes	Bahrain	2015	All Ages	0.2 (0.2 to 0.3)	7.6% (6.7 to 8.7%)	16.2 (13.0 to 19.8)
All causes	Bahrain	1990	Age-standardized		11.5% (9.9 to 13.3%)	107.8 (91.0 to 127.6)
All causes	Bahrain	1995	Age-standardized		10.6% (9.0 to 12.2%)	95.7 (80.6 to 112.5)
All causes	Bahrain	2000	Age-standardized		9.9% (8.6 to 11.5%)	85.3 (72.7 to 99.6)
All causes	Bahrain	2005	Age-standardized		8.9% (7.5 to 10.3%)	65.3 (55.0 to 76.4)
All causes	Bahrain	2010	Age-standardized		8.8% (7.5 to 10.2%)	51.3 (42.9 to 60.5)

Cause name	Location	Year	Age group	Number (in thousands)	PAF	Rate (per 100,000)
All causes	Bahrain	2015	Age-standardized		8.1% (6.9 to 9.5%)	46.2 (36.8 to 56.6)
Lower respiratory infections	Bahrain	1990	All Ages	0.0 (0.0 to 0.0)	35.2% (25.9 to 43.8%)	4.5 (3.3 to 5.8)
Lower respiratory infections	Bahrain	1995	All Ages	0.0 (0.0 to 0.0)	34.9% (26.1 to 43.0%)	3.8 (2.8 to 5.0)
Lower respiratory infections	Bahrain	2000	All Ages	0.0 (0.0 to 0.0)	35.1% (26.6 to 43.6%)	3.5 (2.5 to 4.5)
Lower respiratory infections	Bahrain	2005	All Ages	0.0 (0.0 to 0.0)	32.1% (23.3 to 40.5%)	2.6 (1.9 to 3.4)
Lower respiratory infections	Bahrain	2010	All Ages	0.0 (0.0 to 0.0)	36.2% (27.1 to 45.4%)	2.2 (1.6 to 2.8)
Lower respiratory infections	Bahrain	2015	All Ages	0.0 (0.0 to 0.0)	33.0% (24.4 to 41.3%)	2.1 (1.4 to 2.7)
Lower respiratory infections	Bahrain	1990	Age-standardized		35.2% (25.9 to 43.8%)	11.6 (8.5 to 15.5)
Lower respiratory infections	Bahrain	1995	Age-standardized		34.9% (26.1 to 43.0%)	11.0 (7.8 to 14.9)
Lower respiratory infections	Bahrain	2000	Age-standardized		35.1% (26.6 to 43.6%)	10.5 (7.5 to 13.8)
Lower respiratory infections	Bahrain	2005	Age-standardized		32.1% (23.3 to 40.5%)	9.1 (6.3 to 11.9)
Lower respiratory infections	Bahrain	2010	Age-standardized		36.2% (27.1 to 45.4%)	8.1 (5.8 to 10.6)
Lower respiratory infections	Bahrain	2015	Age-standardized		33.0% (24.4 to 41.3%)	7.2 (4.9 to 9.3)
Tracheal, bronchus, and lung cancer	Bahrain	1990	All Ages	0.0 (0.0 to 0.0)	26.2% (17.1 to 35.4%)	2.2 (1.4 to 3.0)
Tracheal, bronchus, and lung cancer	Bahrain	1995	All Ages	0.0 (0.0 to 0.0)	25.9% (16.9 to 35.1%)	2.0 (1.3 to 2.8)
Tracheal, bronchus, and lung cancer	Bahrain	2000	All Ages	0.0 (0.0 to 0.0)	26.2% (17.3 to 35.4%)	2.0 (1.3 to 2.8)
Tracheal, bronchus, and lung cancer	Bahrain	2005	All Ages	0.0 (0.0 to 0.0)	23.5% (14.8 to 32.6%)	1.5 (0.9 to 2.1)
Tracheal, bronchus, and lung cancer	Bahrain	2010	All Ages	0.0 (0.0 to 0.0)	27.1% (18.0 to 36.1%)	1.4 (0.9 to 1.9)
Tracheal, bronchus, and lung cancer	Bahrain	2015	All Ages	0.0 (0.0 to 0.0)	24.3% (15.4 to 33.3%)	1.4 (0.8 to 2.0)
Tracheal, bronchus, and lung cancer	Bahrain	1990	Age-standardized		26.4% (17.2 to 35.8%)	7.1 (4.6 to 9.9)
Tracheal, bronchus, and lung cancer	Bahrain	1995	Age-standardized		26.1% (17.0 to 35.4%)	6.2 (3.9 to 8.6)
Tracheal, bronchus, and lung cancer	Bahrain	2000	Age-standardized		26.3% (17.4 to 35.6%)	6.0 (3.9 to 8.2)
Tracheal, bronchus, and lung cancer	Bahrain	2005	Age-standardized		23.7% (14.9 to 32.9%)	4.6 (2.9 to 6.4)
Tracheal, bronchus, and lung cancer	Bahrain	2010	Age-standardized		27.3% (18.2 to 36.3%)	4.1 (2.7 to 5.7)
Tracheal, bronchus, and lung cancer	Bahrain	2015	Age-standardized		24.5% (15.4 to 33.5%)	3.6 (2.1 to 5.2)
Cardiovascular diseases	Bahrain	1990	All Ages	0.1 (0.1 to 0.2)	21.7% (18.9 to 24.5%)	26.7 (22.4 to 31.8)
Cardiovascular diseases	Bahrain	1995	All Ages	0.1 (0.1 to 0.2)	20.8% (18.3 to 23.8%)	23.2 (19.6 to 27.4)
Cardiovascular diseases	Bahrain	2000	All Ages	0.1 (0.1 to 0.2)	20.5% (17.8 to 23.3%)	20.5 (17.5 to 24.0)
Cardiovascular diseases	Bahrain	2005	All Ages	0.1 (0.1 to 0.1)	19.5% (16.8 to 22.4%)	14.2 (11.9 to 16.7)
Cardiovascular diseases	Bahrain	2010	All Ages	0.1 (0.1 to 0.2)	21.2% (18.5 to 24.1%)	11.3 (9.5 to 13.4)
Cardiovascular diseases	Bahrain	2015	All Ages	0.2 (0.1 to 0.2)	19.8% (17.2 to 22.7%)	11.5 (8.9 to 14.2)
Cardiovascular diseases	Bahrain	1990	Age-standardized		18.8% (15.9 to 22.4%)	77.9 (63.8 to 94.8)
Cardiovascular diseases	Bahrain	1995	Age-standardized		18.0% (15.3 to 21.3%)	66.8 (54.9 to 80.4)
Cardiovascular diseases	Bahrain	2000	Age-standardized		17.8% (15.1 to 21.1%)	58.0 (48.6 to 70.0)
Cardiovascular diseases	Bahrain	2005	Age-standardized		16.5% (13.7 to 19.8%)	42.5 (34.7 to 51.1)
Cardiovascular diseases	Bahrain	2010	Age-standardized		17.3% (14.3 to 20.7%)	32.7 (26.7 to 40.0)

Cause name	Location	Year	Age group	Number (in thousands)	PAF	Rate (per 100,000)
Cardiovascular diseases	Bahrain	2015	Age-standardized		16.2% (13.4 to 19.8%)	30.2 (23.2 to 38.0)
Ischemic heart disease	Bahrain	1990	All Ages	0.1 (0.1 to 0.1)	25.0% (21.5 to 28.6%)	23.0 (19.0 to 27.7)
Ischemic heart disease	Bahrain	1995	All Ages	0.1 (0.1 to 0.1)	24.4% (21.0 to 28.2%)	19.7 (16.4 to 23.6)
Ischemic heart disease	Bahrain	2000	All Ages	0.1 (0.1 to 0.1)	24.1% (20.4 to 27.9%)	17.3 (14.5 to 20.5)
Ischemic heart disease	Bahrain	2005	All Ages	0.1 (0.1 to 0.1)	23.2% (19.5 to 27.1%)	11.9 (9.8 to 14.3)
Ischemic heart disease	Bahrain	2010	All Ages	0.1 (0.1 to 0.1)	25.4% (21.7 to 29.2%)	9.4 (7.8 to 11.3)
Ischemic heart disease	Bahrain	2015	All Ages	0.1 (0.1 to 0.2)	23.8% (20.2 to 27.7%)	9.6 (7.3 to 12.1)
Ischemic heart disease	Bahrain	1990	Age-standardized		21.2% (17.5 to 26.0%)	66.7 (53.2 to 82.7)
Ischemic heart disease	Bahrain	1995	Age-standardized		20.8% (17.1 to 25.2%)	56.1 (45.1 to 68.7)
Ischemic heart disease	Bahrain	2000	Age-standardized		20.7% (16.9 to 25.3%)	48.4 (39.4 to 59.4)
Ischemic heart disease	Bahrain	2005	Age-standardized		19.5% (15.6 to 23.9%)	35.2 (27.8 to 43.3)
Ischemic heart disease	Bahrain	2010	Age-standardized		20.5% (16.6 to 25.2%)	26.6 (21.1 to 33.5)
Ischemic heart disease	Bahrain	2015	Age-standardized		19.5% (15.5 to 24.3%)	24.6 (18.6 to 31.7)
Ischemic stroke	Bahrain	1990	All Ages	0.0 (0.0 to 0.0)	18.1% (14.7 to 21.5%)	1.5 (1.2 to 1.9)
Ischemic stroke	Bahrain	1995	All Ages	0.0 (0.0 to 0.0)	17.8% (14.5 to 21.4%)	1.6 (1.2 to 2.0)
Ischemic stroke	Bahrain	2000	All Ages	0.0 (0.0 to 0.0)	17.7% (14.3 to 21.3%)	1.5 (1.2 to 1.9)
Ischemic stroke	Bahrain	2005	All Ages	0.0 (0.0 to 0.0)	16.9% (13.7 to 20.2%)	1.1 (0.9 to 1.3)
Ischemic stroke	Bahrain	2010	All Ages	0.0 (0.0 to 0.0)	18.8% (15.4 to 22.1%)	0.9 (0.7 to 1.1)
Ischemic stroke	Bahrain	2015	All Ages	0.0 (0.0 to 0.0)	17.2% (13.9 to 20.6%)	0.9 (0.7 to 1.2)
Ischemic stroke	Bahrain	1990	Age-standardized		16.2% (12.2 to 20.3%)	5.8 (4.4 to 7.6)
Ischemic stroke	Bahrain	1995	Age-standardized		15.8% (11.8 to 20.0%)	6.0 (4.4 to 7.9)
Ischemic stroke	Bahrain	2000	Age-standardized		15.9% (11.9 to 20.4%)	5.5 (4.1 to 7.3)
Ischemic stroke	Bahrain	2005	Age-standardized		14.8% (11.1 to 19.0%)	4.3 (3.1 to 5.7)
Ischemic stroke	Bahrain	2010	Age-standardized		16.0% (11.8 to 20.4%)	3.7 (2.7 to 4.8)
Ischemic stroke	Bahrain	2015	Age-standardized		14.9% (10.8 to 19.5%)	3.5 (2.4 to 4.8)
Hemorrhagic stroke	Bahrain	1990	All Ages	0.0 (0.0 to 0.0)	20.2% (17.1 to 23.4%)	2.1 (1.7 to 2.6)
Hemorrhagic stroke	Bahrain	1995	All Ages	0.0 (0.0 to 0.0)	20.2% (17.1 to 23.4%)	1.9 (1.5 to 2.4)
Hemorrhagic stroke	Bahrain	2000	All Ages	0.0 (0.0 to 0.0)	20.0% (16.8 to 23.2%)	1.7 (1.4 to 2.0)
Hemorrhagic stroke	Bahrain	2005	All Ages	0.0 (0.0 to 0.0)	19.3% (16.1 to 22.2%)	1.2 (1.0 to 1.5)
Hemorrhagic stroke	Bahrain	2010	All Ages	0.0 (0.0 to 0.0)	21.8% (18.6 to 25.1%)	1.0 (0.8 to 1.2)
Hemorrhagic stroke	Bahrain	2015	All Ages	0.0 (0.0 to 0.0)	20.0% (16.9 to 23.1%)	0.9 (0.7 to 1.2)
Hemorrhagic stroke	Bahrain	1990	Age-standardized		17.9% (14.6 to 21.2%)	5.4 (4.3 to 6.6)
Hemorrhagic stroke	Bahrain	1995	Age-standardized		17.6% (14.3 to 20.9%)	4.7 (3.6 to 5.9)
Hemorrhagic stroke	Bahrain	2000	Age-standardized		17.5% (14.2 to 20.9%)	4.1 (3.2 to 5.0)
Hemorrhagic stroke	Bahrain	2005	Age-standardized		16.3% (13.0 to 19.8%)	3.0 (2.4 to 3.8)
Hemorrhagic stroke	Bahrain	2010	Age-standardized		17.5% (14.0 to 21.2%)	2.4 (1.9 to 3.1)

Cause name	Location	Year	Age group	Number (in thousands)	PAF	Rate (per 100,000)
Hemorrhagic stroke	Bahrain	2015	Age-standardized		16.3% (12.7 to 20.0%)	2.1 (1.6 to 2.8)
Chronic obstructive pulmonary disease	Bahrain	1990	All Ages	0.0 (0.0 to 0.0)	29.6% (18.8 to 42.4%)	2.6 (1.6 to 3.9)
Chronic obstructive pulmonary disease	Bahrain	1995	All Ages	0.0 (0.0 to 0.0)	30.4% (19.1 to 43.1%)	2.8 (1.7 to 4.0)
Chronic obstructive pulmonary disease	Bahrain	2000	All Ages	0.0 (0.0 to 0.0)	31.1% (19.9 to 44.0%)	2.7 (1.7 to 3.8)
Chronic obstructive pulmonary disease	Bahrain	2005	All Ages	0.0 (0.0 to 0.0)	29.1% (18.1 to 41.4%)	2.0 (1.2 to 2.8)
Chronic obstructive pulmonary disease	Bahrain	2010	All Ages	0.0 (0.0 to 0.0)	32.4% (20.5 to 45.3%)	1.4 (0.8 to 1.9)
Chronic obstructive pulmonary disease	Bahrain	2015	All Ages	0.0 (0.0 to 0.0)	30.1% (18.7 to 42.2%)	1.3 (0.8 to 1.9)
Chronic obstructive pulmonary disease	Bahrain	1990	Age-standardized		32.0% (20.1 to 45.4%)	11.2 (6.8 to 16.3)
Chronic obstructive pulmonary disease	Bahrain	1995	Age-standardized		31.9% (20.0 to 44.7%)	11.7 (7.2 to 16.8)
Chronic obstructive pulmonary disease	Bahrain	2000	Age-standardized		32.2% (20.6 to 45.3%)	10.8 (6.8 to 15.4)
Chronic obstructive pulmonary disease	Bahrain	2005	Age-standardized		29.9% (18.6 to 42.5%)	9.0 (5.6 to 12.8)
Chronic obstructive pulmonary disease	Bahrain	2010	Age-standardized		33.1% (20.9 to 46.4%)	6.3 (3.9 to 9.2)
Chronic obstructive pulmonary disease	Bahrain	2015	Age-standardized		30.6% (19.0 to 42.8%)	5.3 (3.1 to 7.6)
All causes	Egypt	1990	All Ages	52.3 (45.7 to 59.4)	11.4% (9.9 to 12.9%)	92.8 (81.2 to 105.4)
All causes	Egypt	1995	All Ages	47.6 (42.1 to 53.0)	10.9% (9.7 to 12.2%)	76.1 (67.4 to 84.8)
All causes	Egypt	2000	All Ages	43.1 (38.6 to 47.9)	10.5% (9.4 to 11.6%)	63.0 (56.4 to 70.0)
All causes	Egypt	2005	All Ages	48.3 (43.4 to 53.6)	10.7% (9.6 to 11.8%)	64.5 (58.0 to 71.6)
All causes	Egypt	2010	All Ages	57.4 (51.8 to 63.5)	11.0% (9.9 to 12.2%)	69.7 (62.9 to 77.2)
All causes	Egypt	2015	All Ages	60.2 (54.3 to 66.5)	11.3% (10.2 to 12.5%)	66.0 (59.6 to 73.0)
All causes	Egypt	1990	Age-standardized		11.7% (10.4 to 13.1%)	139.5 (124.2 to 157.4)
All causes	Egypt	1995	Age-standardized		11.4% (10.1 to 12.8%)	128.5 (113.9 to 145.7)
All causes	Egypt	2000	Age-standardized		11.0% (9.8 to 12.5%)	111.1 (98.5 to 126.1)
All causes	Egypt	2005	Age-standardized		11.2% (9.9 to 12.7%)	113.7 (100.2 to 128.9)
All causes	Egypt	2010	Age-standardized		11.5% (10.2 to 13.1%)	120.2 (106.6 to 136.4)
All causes	Egypt	2015	Age-standardized		11.8% (10.4 to 13.4%)	113.5 (100.8 to 129.5)
Lower respiratory infections	Egypt	1990	All Ages	22.2 (16.5 to 28.2)	38.1% (29.7 to 45.8%)	39.5 (29.2 to 50.0)
Lower respiratory infections	Egypt	1995	All Ages	15.2 (11.5 to 18.8)	37.3% (29.3 to 44.9%)	24.3 (18.4 to 30.1)
Lower respiratory infections	Egypt	2000	All Ages	10.4 (7.9 to 12.9)	37.5% (29.2 to 45.0%)	15.2 (11.6 to 18.8)
Lower respiratory infections	Egypt	2005	All Ages	9.7 (7.3 to 12.4)	38.9% (30.4 to 46.7%)	12.9 (9.8 to 16.5)
Lower respiratory infections	Egypt	2010	All Ages	10.7 (8.1 to 13.5)	40.9% (32.2 to 49.3%)	12.9 (9.9 to 16.4)
Lower respiratory infections	Egypt	2015	All Ages	10.1 (7.5 to 13.3)	43.2% (34.1 to 52.1%)	11.1 (8.2 to 14.6)
Lower respiratory infections	Egypt	1990	Age-standardized		38.1% (29.7 to 45.8%)	31.6 (24.2 to 39.1)
Lower respiratory infections	Egypt	1995	Age-standardized		37.3% (29.3 to 44.9%)	23.8 (18.0 to 30.1)
Lower respiratory infections	Egypt	2000	Age-standardized		37.5% (29.2 to 45.0%)	17.3 (13.0 to 23.1)
Lower respiratory infections	Egypt	2005	Age-standardized		38.9% (30.4 to 46.7%)	16.0 (11.9 to 21.7)
Lower respiratory infections	Egypt	2010	Age-standardized		40.9% (32.2 to 49.3%)	16.4 (12.3 to 22.4)

Cause name	Location	Year	Age group	Number (in thousands)	PAF	Rate (per 100,000)
Lower respiratory infections	Egypt	2015	Age-standardized		43.2% (34.1 to 52.1%)	15.3 (11.4 to 20.7)
Tracheal, bronchus, and lung cancer	Egypt	1990	All Ages	0.6 (0.4 to 0.8)	29.0% (20.2 to 37.2%)	1.0 (0.7 to 1.4)
Tracheal, bronchus, and lung cancer	Egypt	1995	All Ages	0.7 (0.5 to 0.9)	28.2% (19.3 to 36.6%)	1.1 (0.8 to 1.5)
Tracheal, bronchus, and lung cancer	Egypt	2000	All Ages	0.8 (0.6 to 1.1)	28.4% (19.7 to 36.9%)	1.2 (0.8 to 1.6)
Tracheal, bronchus, and lung cancer	Egypt	2005	All Ages	1.1 (0.8 to 1.4)	29.7% (20.8 to 38.0%)	1.4 (1.0 to 1.9)
Tracheal, bronchus, and lung cancer	Egypt	2010	All Ages	1.5 (1.0 to 1.9)	32.0% (23.1 to 40.4%)	1.8 (1.3 to 2.3)
Tracheal, bronchus, and lung cancer	Egypt	2015	All Ages	1.8 (1.3 to 2.2)	34.7% (25.5 to 43.0%)	1.9 (1.4 to 2.4)
Tracheal, bronchus, and lung cancer	Egypt	1990	Age-standardized		29.2% (20.3 to 37.5%)	1.9 (1.3 to 2.4)
Tracheal, bronchus, and lung cancer	Egypt	1995	Age-standardized		28.4% (19.5 to 36.9%)	2.0 (1.4 to 2.6)
Tracheal, bronchus, and lung cancer	Egypt	2000	Age-standardized		28.6% (19.8 to 37.2%)	2.1 (1.4 to 2.7)
Tracheal, bronchus, and lung cancer	Egypt	2005	Age-standardized		30.0% (21.0 to 38.4%)	2.4 (1.7 to 3.1)
Tracheal, bronchus, and lung cancer	Egypt	2010	Age-standardized		32.2% (23.3 to 40.8%)	2.9 (2.1 to 3.7)
Tracheal, bronchus, and lung cancer	Egypt	2015	Age-standardized		34.8% (25.6 to 43.3%)	3.1 (2.2 to 3.9)
Cardiovascular diseases	Egypt	1990	All Ages	25.2 (22.4 to 28.6)	16.4% (14.6 to 18.6%)	44.7 (39.8 to 50.8)
Cardiovascular diseases	Egypt	1995	All Ages	27.4 (24.2 to 31.2)	16.7% (14.7 to 19.0%)	43.8 (38.8 to 50.0)
Cardiovascular diseases	Egypt	2000	All Ages	27.7 (24.4 to 31.7)	17.2% (15.2 to 19.5%)	40.5 (35.7 to 46.3)
Cardiovascular diseases	Egypt	2005	All Ages	32.9 (29.2 to 37.3)	17.6% (15.6 to 20.1%)	43.9 (39.1 to 49.9)
Cardiovascular diseases	Egypt	2010	All Ages	40.3 (35.7 to 45.6)	18.3% (16.3 to 20.7%)	48.9 (43.4 to 55.4)
Cardiovascular diseases	Egypt	2015	All Ages	43.2 (38.3 to 48.8)	19.1% (17.1 to 21.5%)	47.4 (42.1 to 53.5)
Cardiovascular diseases	Egypt	1990	Age-standardized		16.6% (14.4 to 19.1%)	90.3 (78.6 to 104.3)
Cardiovascular diseases	Egypt	1995	Age-standardized		16.4% (14.2 to 19.2%)	88.4 (76.5 to 103.4)
Cardiovascular diseases	Egypt	2000	Age-standardized		16.6% (14.4 to 19.4%)	79.2 (68.1 to 92.6)
Cardiovascular diseases	Egypt	2005	Age-standardized		17.1% (14.8 to 19.9%)	82.8 (72.0 to 96.1)
Cardiovascular diseases	Egypt	2010	Age-standardized		17.6% (15.3 to 20.6%)	89.0 (77.5 to 103.6)
Cardiovascular diseases	Egypt	2015	Age-standardized		18.1% (15.8 to 21.1%)	84.3 (73.4 to 98.5)
Ischemic heart disease	Egypt	1990	All Ages	17.8 (15.0 to 20.9)	22.9% (19.6 to 26.8%)	31.5 (26.6 to 37.2)
Ischemic heart disease	Egypt	1995	All Ages	19.5 (16.5 to 23.0)	22.3% (19.0 to 26.3%)	31.2 (26.3 to 36.8)
Ischemic heart disease	Egypt	2000	All Ages	19.7 (16.7 to 23.5)	22.4% (19.0 to 26.7%)	28.8 (24.4 to 34.3)
Ischemic heart disease	Egypt	2005	All Ages	23.3 (19.8 to 27.5)	23.0% (19.7 to 27.1%)	31.1 (26.4 to 36.7)
Ischemic heart disease	Egypt	2010	All Ages	28.8 (24.6 to 33.8)	23.9% (20.6 to 28.2%)	35.0 (29.9 to 41.1)
Ischemic heart disease	Egypt	2015	All Ages	31.4 (27.0 to 37.0)	24.9% (21.6 to 29.0%)	34.4 (29.6 to 40.6)
Ischemic heart disease	Egypt	1990	Age-standardized		21.5% (17.8 to 26.0%)	64.1 (53.0 to 77.4)
Ischemic heart disease	Egypt	1995	Age-standardized		20.9% (17.2 to 25.6%)	63.2 (51.3 to 77.4)
Ischemic heart disease	Egypt	2000	Age-standardized		21.1% (17.4 to 25.7%)	56.6 (46.2 to 68.8)
Ischemic heart disease	Egypt	2005	Age-standardized		21.7% (17.9 to 26.4%)	58.7 (48.5 to 71.8)
Ischemic heart disease	Egypt	2010	Age-standardized		22.4% (18.6 to 27.3%)	63.8 (53.0 to 78.5)

Cause name	Location	Year	Age group	Number (in thousands)	PAF	Rate (per 100,000)
Ischemic heart disease	Egypt	2015	Age-standardized		23.2% (19.5 to 28.3%)	61.3 (51.3 to 74.9)
Ischemic stroke	Egypt	1990	All Ages	2.9 (2.4 to 3.9)	18.4% (15.3 to 21.4%)	5.2 (4.2 to 6.9)
Ischemic stroke	Egypt	1995	All Ages	3.1 (2.5 to 4.0)	17.9% (14.9 to 21.2%)	5.0 (4.0 to 6.3)
Ischemic stroke	Egypt	2000	All Ages	3.1 (2.5 to 3.9)	18.1% (14.9 to 21.4%)	4.5 (3.6 to 5.6)
Ischemic stroke	Egypt	2005	All Ages	3.6 (2.9 to 4.5)	18.7% (15.5 to 22.1%)	4.8 (3.9 to 6.1)
Ischemic stroke	Egypt	2010	All Ages	4.2 (3.4 to 5.3)	19.5% (16.1 to 23.0%)	5.1 (4.2 to 6.5)
Ischemic stroke	Egypt	2015	All Ages	4.4 (3.6 to 5.6)	20.4% (17.0 to 24.2%)	4.8 (3.9 to 6.2)
Ischemic stroke	Egypt	1990	Age-standardized		17.5% (14.0 to 21.4%)	11.2 (8.7 to 15.3)
Ischemic stroke	Egypt	1995	Age-standardized		17.0% (13.4 to 20.8%)	10.7 (8.4 to 14.0)
Ischemic stroke	Egypt	2000	Age-standardized		17.2% (13.6 to 21.0%)	9.3 (7.3 to 11.9)
Ischemic stroke	Egypt	2005	Age-standardized		17.8% (14.1 to 21.5%)	9.7 (7.6 to 12.4)
Ischemic stroke	Egypt	2010	Age-standardized		18.5% (14.8 to 22.4%)	10.1 (8.0 to 13.1)
Ischemic stroke	Egypt	2015	Age-standardized		19.2% (15.4 to 23.3%)	9.4 (7.4 to 12.3)
Hemorrhagic stroke	Egypt	1990	All Ages	4.5 (3.8 to 5.5)	16.4% (14.0 to 19.0%)	8.0 (6.7 to 9.7)
Hemorrhagic stroke	Egypt	1995	All Ages	4.8 (4.0 to 5.8)	17.1% (14.6 to 19.7%)	7.6 (6.4 to 9.2)
Hemorrhagic stroke	Egypt	2000	All Ages	4.9 (4.1 to 6.0)	18.2% (15.4 to 20.8%)	7.2 (6.0 to 8.7)
Hemorrhagic stroke	Egypt	2005	All Ages	6.0 (5.1 to 7.2)	19.0% (16.3 to 21.7%)	8.0 (6.8 to 9.6)
Hemorrhagic stroke	Egypt	2010	All Ages	7.3 (6.2 to 8.7)	20.2% (17.4 to 23.1%)	8.8 (7.5 to 10.6)
Hemorrhagic stroke	Egypt	2015	All Ages	7.4 (6.3 to 9.3)	21.6% (18.6 to 24.6%)	8.2 (6.9 to 10.2)
Hemorrhagic stroke	Egypt	1990	Age-standardized		17.9% (15.0 to 20.7%)	15.1 (12.3 to 18.3)
Hemorrhagic stroke	Egypt	1995	Age-standardized		17.7% (14.7 to 20.6%)	14.5 (11.8 to 17.6)
Hemorrhagic stroke	Egypt	2000	Age-standardized		18.1% (15.1 to 21.2%)	13.3 (10.7 to 16.0)
Hemorrhagic stroke	Egypt	2005	Age-standardized		18.7% (15.6 to 21.9%)	14.4 (11.8 to 17.3)
Hemorrhagic stroke	Egypt	2010	Age-standardized		19.6% (16.5 to 23.0%)	15.1 (12.5 to 18.3)
Hemorrhagic stroke	Egypt	2015	Age-standardized		20.6% (17.4 to 24.2%)	13.7 (11.3 to 17.0)
Chronic obstructive pulmonary disease	Egypt	1990	All Ages	4.3 (2.8 to 6.1)	27.3% (17.9 to 39.0%)	7.6 (4.9 to 10.8)
Chronic obstructive pulmonary disease	Egypt	1995	All Ages	4.3 (2.8 to 6.0)	31.0% (20.6 to 44.5%)	6.8 (4.4 to 9.6)
Chronic obstructive pulmonary disease	Egypt	2000	All Ages	4.2 (2.7 to 5.9)	33.1% (21.7 to 46.8%)	6.1 (4.0 to 8.7)
Chronic obstructive pulmonary disease	Egypt	2005	All Ages	4.7 (3.1 to 6.6)	34.7% (23.3 to 49.2%)	6.3 (4.1 to 8.9)
Chronic obstructive pulmonary disease	Egypt	2010	All Ages	5.0 (3.3 to 7.1)	36.6% (25.0 to 51.8%)	6.1 (4.0 to 8.6)
Chronic obstructive pulmonary disease	Egypt	2015	All Ages	5.1 (3.5 to 7.3)	38.9% (27.2 to 54.7%)	5.6 (3.8 to 8.0)
Chronic obstructive pulmonary disease	Egypt	1990	Age-standardized		32.4% (21.4 to 46.3%)	15.8 (10.3 to 22.4)
Chronic obstructive pulmonary disease	Egypt	1995	Age-standardized		33.1% (22.0 to 46.9%)	14.4 (9.3 to 20.3)
Chronic obstructive pulmonary disease	Egypt	2000	Age-standardized		34.0% (22.2 to 47.9%)	12.5 (8.2 to 17.7)
Chronic obstructive pulmonary disease	Egypt	2005	Age-standardized		35.3% (23.7 to 50.0%)	12.6 (8.2 to 17.8)
Chronic obstructive pulmonary disease	Egypt	2010	Age-standardized		37.2% (25.4 to 52.5%)	11.9 (7.8 to 17.0)

Cause name	Location	Year	Age group	Number (in thousands)	PAF	Rate (per 100,000)
Chronic obstructive pulmonary disease	Egypt	2015	Age-standardized		39.3% (27.5 to 55.3%)	10.8 (7.4 to 15.4)
All causes	Iran	1990	All Ages	22.6 (18.9 to 26.6)	6.1% (5.2 to 7.0%)	40.3 (33.7 to 47.5)
All causes	Iran	1995	All Ages	21.7 (18.7 to 25.5)	7.4% (6.6 to 8.4%)	35.9 (30.8 to 42.1)
All causes	Iran	2000	All Ages	23.7 (20.8 to 27.3)	8.0% (7.2 to 8.9%)	36.0 (31.6 to 41.5)
All causes	Iran	2005	All Ages	27.4 (22.2 to 33.1)	7.6% (6.7 to 8.6%)	38.8 (31.4 to 46.9)
All causes	Iran	2010	All Ages	31.5 (24.9 to 38.8)	8.5% (7.6 to 9.6%)	42.4 (33.6 to 52.2)
All causes	Iran	2015	All Ages	33.3 (26.7 to 40.4)	8.6% (7.6 to 9.8%)	42.1 (33.7 to 51.1)
All causes	Iran	1990	Age-standardized		8.3% (7.2 to 9.5%)	92.4 (77.2 to 107.9)
All causes	Iran	1995	Age-standardized		9.0% (7.9 to 10.2%)	86.8 (73.8 to 101.6)
All causes	Iran	2000	Age-standardized		9.1% (8.0 to 10.4%)	82.8 (71.2 to 96.5)
All causes	Iran	2005	Age-standardized		8.5% (7.3 to 9.7%)	77.9 (62.6 to 94.6)
All causes	Iran	2010	Age-standardized		9.2% (8.0 to 10.6%)	77.6 (61.5 to 95.5)
All causes	Iran	2015	Age-standardized		9.0% (7.8 to 10.4%)	71.1 (56.9 to 86.1)
Lower respiratory infections	Iran	1990	All Ages	6.1 (4.0 to 8.8)	30.9% (23.1 to 38.2%)	11.0 (7.1 to 15.6)
Lower respiratory infections	Iran	1995	All Ages	3.5 (2.4 to 5.0)	30.1% (22.5 to 37.5%)	5.7 (3.9 to 8.2)
Lower respiratory infections	Iran	2000	All Ages	2.5 (1.8 to 3.5)	30.3% (22.6 to 37.7%)	3.9 (2.8 to 5.3)
Lower respiratory infections	Iran	2005	All Ages	2.3 (1.6 to 3.2)	26.0% (18.3 to 34.2%)	3.3 (2.2 to 4.5)
Lower respiratory infections	Iran	2010	All Ages	2.9 (2.0 to 3.9)	30.1% (22.6 to 37.5%)	3.9 (2.7 to 5.2)
Lower respiratory infections	Iran	2015	All Ages	2.8 (1.8 to 3.9)	28.5% (20.7 to 36.2%)	3.5 (2.3 to 4.9)
Lower respiratory infections	Iran	1990	Age-standardized		30.9% (23.1 to 38.2%)	10.9 (7.7 to 14.9)
Lower respiratory infections	Iran	1995	Age-standardized		30.1% (22.5 to 37.5%)	8.2 (5.8 to 10.9)
Lower respiratory infections	Iran	2000	Age-standardized		30.3% (22.6 to 37.7%)	6.9 (5.0 to 9.3)
Lower respiratory infections	Iran	2005	Age-standardized		26.0% (18.3 to 34.2%)	6.0 (4.0 to 8.3)
Lower respiratory infections	Iran	2010	Age-standardized		30.1% (22.6 to 37.5%)	6.9 (4.7 to 9.5)
Lower respiratory infections	Iran	2015	Age-standardized		28.5% (20.7 to 36.2%)	6.1 (4.0 to 8.7)
Tracheal, bronchus, and lung cancer	Iran	1990	All Ages	0.6 (0.3 to 0.8)	22.5% (14.7 to 30.6%)	1.0 (0.6 to 1.4)
Tracheal, bronchus, and lung cancer	Iran	1995	All Ages	0.7 (0.4 to 1.0)	21.9% (14.1 to 29.9%)	1.1 (0.7 to 1.6)
Tracheal, bronchus, and lung cancer	Iran	2000	All Ages	0.8 (0.5 to 1.1)	22.1% (14.2 to 30.1%)	1.2 (0.8 to 1.7)
Tracheal, bronchus, and lung cancer	Iran	2005	All Ages	0.8 (0.5 to 1.2)	18.6% (11.5 to 26.2%)	1.2 (0.7 to 1.7)
Tracheal, bronchus, and lung cancer	Iran	2010	All Ages	1.2 (0.7 to 1.7)	22.0% (14.3 to 30.0%)	1.7 (1.0 to 2.3)
Tracheal, bronchus, and lung cancer	Iran	2015	All Ages	1.4 (0.8 to 2.0)	20.7% (13.1 to 28.5%)	1.7 (1.0 to 2.5)
Tracheal, bronchus, and lung cancer	Iran	1990	Age-standardized		22.7% (14.8 to 30.8%)	2.6 (1.6 to 3.8)
Tracheal, bronchus, and lung cancer	Iran	1995	Age-standardized		22.0% (14.2 to 30.0%)	2.9 (1.8 to 4.0)
Tracheal, bronchus, and lung cancer	Iran	2000	Age-standardized		22.2% (14.3 to 30.3%)	2.8 (1.8 to 3.9)
Tracheal, bronchus, and lung cancer	Iran	2005	Age-standardized		18.7% (11.6 to 26.4%)	2.2 (1.3 to 3.3)
Tracheal, bronchus, and lung cancer	Iran	2010	Age-standardized		22.1% (14.4 to 30.2%)	2.9 (1.8 to 4.1)

Cause name	Location	Year	Age group	Number (in thousands)	PAF	Rate (per 100,000)
Tracheal, bronchus, and lung cancer	Iran	2015	Age-standardized		20.7% (13.1 to 28.6%)	2.8 (1.7 to 4.0)
Cardiovascular diseases	Iran	1990	All Ages	14.7 (12.2 to 17.8)	15.2% (13.4 to 17.2%)	26.3 (21.8 to 31.7)
Cardiovascular diseases	Iran	1995	All Ages	16.3 (13.8 to 19.4)	15.3% (13.4 to 17.2%)	27.0 (22.8 to 32.1)
Cardiovascular diseases	Iran	2000	All Ages	18.8 (16.1 to 22.0)	15.4% (13.7 to 17.4%)	28.6 (24.5 to 33.4)
Cardiovascular diseases	Iran	2005	All Ages	22.2 (17.8 to 27.2)	14.2% (12.2 to 16.3%)	31.5 (25.2 to 38.5)
Cardiovascular diseases	Iran	2010	All Ages	24.6 (19.3 to 30.7)	15.1% (13.1 to 17.4%)	33.1 (26.0 to 41.3)
Cardiovascular diseases	Iran	2015	All Ages	26.3 (20.7 to 32.4)	14.9% (12.9 to 17.2%)	33.3 (26.1 to 41.0)
Cardiovascular diseases	Iran	1990	Age-standardized		14.5% (12.5 to 16.9%)	72.3 (59.6 to 86.2)
Cardiovascular diseases	Iran	1995	Age-standardized		14.2% (12.2 to 16.5%)	69.7 (57.9 to 82.8)
Cardiovascular diseases	Iran	2000	Age-standardized		14.3% (12.3 to 16.6%)	67.0 (56.4 to 79.6)
Cardiovascular diseases	Iran	2005	Age-standardized		13.3% (11.2 to 15.7%)	63.4 (50.1 to 78.6)
Cardiovascular diseases	Iran	2010	Age-standardized		14.2% (12.0 to 16.7%)	60.5 (47.2 to 75.8)
Cardiovascular diseases	Iran	2015	Age-standardized		13.8% (11.7 to 16.3%)	55.6 (43.6 to 68.9)
Ischemic heart disease	Iran	1990	All Ages	10.7 (8.6 to 13.1)	21.2% (18.0 to 24.6%)	19.1 (15.3 to 23.5)
Ischemic heart disease	Iran	1995	All Ages	11.7 (9.6 to 14.1)	20.9% (17.8 to 24.3%)	19.4 (15.8 to 23.4)
Ischemic heart disease	Iran	2000	All Ages	13.7 (11.4 to 16.5)	20.8% (17.8 to 24.3%)	20.8 (17.4 to 25.0)
Ischemic heart disease	Iran	2005	All Ages	16.6 (12.8 to 20.7)	18.7% (15.7 to 22.2%)	23.5 (18.1 to 29.3)
Ischemic heart disease	Iran	2010	All Ages	18.3 (13.9 to 23.3)	19.9% (16.7 to 23.7%)	24.7 (18.8 to 31.3)
Ischemic heart disease	Iran	2015	All Ages	19.8 (15.3 to 24.9)	19.9% (16.6 to 23.4%)	25.0 (19.4 to 31.5)
Ischemic heart disease	Iran	1990	Age-standardized		19.4% (16.1 to 23.5%)	51.4 (40.6 to 64.1)
Ischemic heart disease	Iran	1995	Age-standardized		19.2% (15.7 to 23.2%)	49.1 (39.2 to 60.5)
Ischemic heart disease	Iran	2000	Age-standardized		19.2% (15.7 to 23.2%)	47.8 (38.6 to 58.6)
Ischemic heart disease	Iran	2005	Age-standardized		17.6% (14.2 to 21.3%)	46.5 (35.8 to 58.5)
Ischemic heart disease	Iran	2010	Age-standardized		18.7% (15.1 to 22.9%)	44.3 (33.5 to 57.2)
Ischemic heart disease	Iran	2015	Age-standardized		18.4% (14.8 to 22.4%)	40.9 (31.2 to 52.0)
Ischemic stroke	Iran	1990	All Ages	1.9 (1.5 to 2.4)	16.2% (13.0 to 19.1%)	3.4 (2.6 to 4.3)
Ischemic stroke	Iran	1995	All Ages	2.1 (1.6 to 2.7)	15.8% (12.6 to 18.8%)	3.6 (2.7 to 4.5)
Ischemic stroke	Iran	2000	All Ages	2.5 (1.8 to 3.0)	15.5% (12.3 to 18.5%)	3.7 (2.8 to 4.6)
Ischemic stroke	Iran	2005	All Ages	2.9 (2.0 to 3.8)	13.5% (10.1 to 16.7%)	4.1 (2.9 to 5.4)
Ischemic stroke	Iran	2010	All Ages	3.2 (2.2 to 4.4)	14.5% (11.0 to 18.1%)	4.3 (3.0 to 5.9)
Ischemic stroke	Iran	2015	All Ages	3.2 (2.2 to 4.3)	14.3% (10.7 to 17.6%)	4.1 (2.8 to 5.5)
Ischemic stroke	Iran	1990	Age-standardized		14.7% (11.1 to 18.4%)	11.2 (8.1 to 14.5)
Ischemic stroke	Iran	1995	Age-standardized		14.4% (10.7 to 18.1%)	10.9 (7.8 to 14.2)
Ischemic stroke	Iran	2000	Age-standardized		14.4% (10.7 to 18.1%)	10.2 (7.3 to 13.2)
Ischemic stroke	Iran	2005	Age-standardized		12.9% (9.2 to 16.6%)	9.2 (6.4 to 12.7)
Ischemic stroke	Iran	2010	Age-standardized		14.0% (10.2 to 18.0%)	8.8 (6.0 to 12.3)

Cause name	Location	Year	Age group	Number (in thousands)	PAF	Rate (per 100,000)
Ischemic stroke	Iran	2015	Age-standardized		13.6% (9.8 to 17.4%)	7.8 (5.2 to 10.9)
Hemorrhagic stroke	Iran	1990	All Ages	2.1 (1.6 to 2.6)	16.3% (13.7 to 18.8%)	3.8 (2.9 to 4.7)
Hemorrhagic stroke	Iran	1995	All Ages	2.4 (1.9 to 3.0)	16.6% (14.0 to 19.2%)	4.0 (3.1 to 5.0)
Hemorrhagic stroke	Iran	2000	All Ages	2.7 (2.2 to 3.3)	16.9% (14.1 to 19.6%)	4.1 (3.3 to 5.0)
Hemorrhagic stroke	Iran	2005	All Ages	2.8 (2.1 to 3.6)	15.0% (12.1 to 17.9%)	3.9 (2.9 to 5.1)
Hemorrhagic stroke	Iran	2010	All Ages	3.1 (2.3 to 4.1)	16.4% (13.5 to 19.2%)	4.1 (3.0 to 5.5)
Hemorrhagic stroke	Iran	2015	All Ages	3.3 (2.4 to 4.6)	16.2% (13.3 to 19.1%)	4.2 (3.1 to 5.8)
Hemorrhagic stroke	Iran	1990	Age-standardized		16.1% (13.1 to 18.9%)	9.7 (7.3 to 12.2)
Hemorrhagic stroke	Iran	1995	Age-standardized		15.9% (12.9 to 18.8%)	9.7 (7.4 to 12.2)
Hemorrhagic stroke	Iran	2000	Age-standardized		15.9% (12.9 to 18.9%)	9.1 (6.9 to 11.3)
Hemorrhagic stroke	Iran	2005	Age-standardized		14.2% (11.1 to 17.2%)	7.7 (5.6 to 10.1)
Hemorrhagic stroke	Iran	2010	Age-standardized		15.4% (12.3 to 18.5%)	7.3 (5.3 to 9.9)
Hemorrhagic stroke	Iran	2015	Age-standardized		15.0% (11.8 to 18.2%)	6.8 (4.8 to 9.7)
Chronic obstructive pulmonary disease	Iran	1990	All Ages	1.2 (0.7 to 1.7)	23.7% (13.7 to 34.2%)	2.0 (1.2 to 3.0)
Chronic obstructive pulmonary disease	Iran	1995	All Ages	1.2 (0.7 to 1.8)	25.4% (15.2 to 36.1%)	2.1 (1.2 to 3.0)
Chronic obstructive pulmonary disease	Iran	2000	All Ages	1.5 (0.9 to 2.2)	26.5% (16.2 to 37.6%)	2.3 (1.4 to 3.4)
Chronic obstructive pulmonary disease	Iran	2005	All Ages	2.0 (1.1 to 3.0)	24.4% (14.0 to 35.3%)	2.8 (1.5 to 4.3)
Chronic obstructive pulmonary disease	Iran	2010	All Ages	2.7 (1.6 to 4.1)	28.0% (17.1 to 39.9%)	3.7 (2.2 to 5.5)
Chronic obstructive pulmonary disease	Iran	2015	All Ages	2.8 (1.6 to 4.2)	26.9% (16.0 to 38.3%)	3.6 (2.0 to 5.3)
Chronic obstructive pulmonary disease	Iran	1990	Age-standardized		27.9% (17.0 to 39.5%)	6.5 (3.8 to 9.3)
Chronic obstructive pulmonary disease	Iran	1995	Age-standardized		27.6% (16.7 to 39.1%)	6.1 (3.6 to 8.8)
Chronic obstructive pulmonary disease	Iran	2000	Age-standardized		27.9% (17.1 to 39.6%)	6.0 (3.6 to 8.8)
Chronic obstructive pulmonary disease	Iran	2005	Age-standardized		25.0% (14.4 to 36.2%)	6.3 (3.4 to 9.4)
Chronic obstructive pulmonary disease	Iran	2010	Age-standardized		28.3% (17.3 to 40.3%)	7.3 (4.2 to 10.8)
Chronic obstructive pulmonary disease	Iran	2015	Age-standardized		27.1% (16.1 to 38.6%)	6.6 (3.7 to 9.8)
All causes	Iraq	1990	All Ages	9.7 (8.0 to 11.5)	7.9% (7.0 to 8.9%)	55.7 (45.9 to 65.9)
All causes	Iraq	1995	All Ages	11.5 (9.7 to 13.6)	8.1% (7.2 to 9.1%)	56.9 (47.8 to 67.1)
All causes	Iraq	2000	All Ages	13.0 (10.9 to 15.5)	8.3% (7.4 to 9.3%)	55.2 (46.2 to 65.7)
All causes	Iraq	2005	All Ages	14.3 (11.6 to 17.3)	8.0% (7.1 to 9.0%)	52.8 (43.0 to 64.0)
All causes	Iraq	2010	All Ages	17.5 (14.1 to 21.7)	9.7% (8.7 to 10.8%)	56.7 (45.7 to 70.1)
All causes	Iraq	2015	All Ages	15.7 (12.3 to 19.6)	7.4% (6.4 to 8.5%)	43.1 (33.8 to 53.8)
All causes	Iraq	1990	Age-standardized		9.8% (8.6 to 11.1%)	123.5 (101.3 to 148.8)
All causes	Iraq	1995	Age-standardized		10.0% (8.7 to 11.3%)	131.8 (108.6 to 156.5)
All causes	Iraq	2000	Age-standardized		10.1% (8.9 to 11.5%)	133.2 (110.4 to 159.5)
All causes	Iraq	2005	Age-standardized		10.0% (8.7 to 11.3%)	131.2 (107.0 to 159.7)
All causes	Iraq	2010	Age-standardized		11.8% (10.4 to 13.3%)	144.1 (115.0 to 178.0)

Cause name	Location	Year	Age group	Number (in thousands)	PAF	Rate (per 100,000)
All causes	Iraq	2015	Age-standardized		9.4% (8.1 to 10.7%)	111.3 (87.0 to 137.3)
Lower respiratory infections	Iraq	1990	All Ages	2.1 (1.4 to 2.8)	30.8% (23.3 to 37.9%)	11.9 (8.0 to 16.3)
Lower respiratory infections	Iraq	1995	All Ages	2.2 (1.5 to 2.9)	30.4% (22.9 to 37.7%)	10.7 (7.4 to 14.3)
Lower respiratory infections	Iraq	2000	All Ages	2.1 (1.4 to 2.8)	30.6% (23.2 to 37.8%)	8.9 (6.1 to 11.7)
Lower respiratory infections	Iraq	2005	All Ages	2.2 (1.6 to 2.9)	31.8% (24.4 to 38.9%)	8.2 (5.9 to 10.7)
Lower respiratory infections	Iraq	2010	All Ages	2.6 (1.9 to 3.4)	39.6% (31.1 to 47.3%)	8.5 (6.3 to 10.9)
Lower respiratory infections	Iraq	2015	All Ages	1.8 (1.3 to 2.4)	30.9% (23.4 to 38.0%)	5.1 (3.6 to 6.7)
Lower respiratory infections	Iraq	1990	Age-standardized		30.8% (23.3 to 37.9%)	12.6 (8.7 to 16.9)
Lower respiratory infections	Iraq	1995	Age-standardized		30.4% (22.9 to 37.7%)	12.3 (8.8 to 16.4)
Lower respiratory infections	Iraq	2000	Age-standardized		30.6% (23.2 to 37.8%)	11.3 (8.1 to 15.5)
Lower respiratory infections	Iraq	2005	Age-standardized		31.8% (24.4 to 38.9%)	10.9 (7.8 to 15.1)
Lower respiratory infections	Iraq	2010	Age-standardized		39.6% (31.1 to 47.3%)	12.2 (8.9 to 17.0)
Lower respiratory infections	Iraq	2015	Age-standardized		30.9% (23.4 to 38.0%)	8.3 (5.8 to 12.0)
Tracheal, bronchus, and lung cancer	Iraq	1990	All Ages	0.3 (0.2 to 0.4)	22.6% (14.8 to 30.7%)	1.7 (1.0 to 2.4)
Tracheal, bronchus, and lung cancer	Iraq	1995	All Ages	0.4 (0.2 to 0.5)	22.2% (14.6 to 30.1%)	1.7 (1.0 to 2.5)
Tracheal, bronchus, and lung cancer	Iraq	2000	All Ages	0.4 (0.3 to 0.6)	22.3% (14.5 to 30.4%)	1.8 (1.1 to 2.6)
Tracheal, bronchus, and lung cancer	Iraq	2005	All Ages	0.5 (0.3 to 0.7)	23.5% (15.6 to 31.4%)	1.9 (1.2 to 2.7)
Tracheal, bronchus, and lung cancer	Iraq	2010	All Ages	0.8 (0.5 to 1.1)	31.4% (22.7 to 39.5%)	2.6 (1.7 to 3.5)
Tracheal, bronchus, and lung cancer	Iraq	2015	All Ages	0.7 (0.4 to 1.0)	22.7% (15.0 to 30.8%)	1.9 (1.1 to 2.7)
Tracheal, bronchus, and lung cancer	Iraq	1990	Age-standardized		22.8% (14.9 to 31.1%)	4.0 (2.4 to 5.9)
Tracheal, bronchus, and lung cancer	Iraq	1995	Age-standardized		22.5% (14.8 to 30.5%)	4.2 (2.6 to 6.1)
Tracheal, bronchus, and lung cancer	Iraq	2000	Age-standardized		22.7% (14.7 to 30.8%)	4.6 (2.9 to 6.6)
Tracheal, bronchus, and lung cancer	Iraq	2005	Age-standardized		23.8% (15.8 to 31.8%)	4.9 (3.2 to 6.8)
Tracheal, bronchus, and lung cancer	Iraq	2010	Age-standardized		31.8% (23.0 to 39.9%)	6.6 (4.4 to 8.8)
Tracheal, bronchus, and lung cancer	Iraq	2015	Age-standardized		22.9% (15.2 to 31.1%)	4.7 (2.9 to 6.7)
Cardiovascular diseases	Iraq	1990	All Ages	7.1 (5.8 to 8.7)	16.0% (13.9 to 18.2%)	40.7 (33.1 to 49.7)
Cardiovascular diseases	Iraq	1995	All Ages	8.7 (7.2 to 10.5)	16.0% (14.0 to 18.3%)	42.9 (35.3 to 51.7)
Cardiovascular diseases	Iraq	2000	All Ages	10.1 (8.3 to 12.3)	16.3% (14.3 to 18.5%)	42.9 (35.2 to 52.1)
Cardiovascular diseases	Iraq	2005	All Ages	11.2 (9.0 to 13.8)	16.7% (14.7 to 18.9%)	41.2 (33.2 to 51.0)
Cardiovascular diseases	Iraq	2010	All Ages	13.6 (10.7 to 17.1)	19.3% (17.2 to 21.7%)	43.9 (34.5 to 55.3)
Cardiovascular diseases	Iraq	2015	All Ages	12.7 (9.6 to 16.2)	16.8% (14.6 to 19.0%)	34.9 (26.5 to 44.5)
Cardiovascular diseases	Iraq	1990	Age-standardized		15.6% (13.4 to 18.1%)	102.7 (82.6 to 125.9)
Cardiovascular diseases	Iraq	1995	Age-standardized		15.6% (13.4 to 18.0%)	110.9 (90.7 to 135.5)
Cardiovascular diseases	Iraq	2000	Age-standardized		15.7% (13.6 to 18.1%)	112.7 (91.4 to 137.1)
Cardiovascular diseases	Iraq	2005	Age-standardized		16.0% (13.7 to 18.4%)	110.9 (88.7 to 137.0)
Cardiovascular diseases	Iraq	2010	Age-standardized		18.2% (15.9 to 21.0%)	120.0 (95.4 to 149.6)

Cause name	Location	Year	Age group	Number (in thousands)	PAF	Rate (per 100,000)
Cardiovascular diseases	Iraq	2015	Age-standardized		15.6% (13.4 to 18.0%)	94.3 (72.5 to 118.4)
Ischemic heart disease	Iraq	1990	All Ages	5.2 (4.1 to 6.5)	20.3% (17.2 to 23.9%)	30.0 (23.7 to 37.5)
Ischemic heart disease	Iraq	1995	All Ages	6.4 (5.1 to 7.8)	20.4% (17.3 to 24.0%)	31.4 (25.1 to 38.6)
Ischemic heart disease	Iraq	2000	All Ages	7.4 (5.9 to 9.1)	20.6% (17.6 to 24.1%)	31.6 (25.2 to 38.8)
Ischemic heart disease	Iraq	2005	All Ages	8.2 (6.4 to 10.4)	21.1% (18.0 to 24.7%)	30.4 (23.7 to 38.4)
Ischemic heart disease	Iraq	2010	All Ages	9.9 (7.7 to 12.6)	24.1% (20.7 to 28.0%)	32.1 (24.9 to 40.7)
Ischemic heart disease	Iraq	2015	All Ages	9.5 (7.1 to 12.2)	21.2% (17.9 to 24.7%)	26.0 (19.4 to 33.4)
Ischemic heart disease	Iraq	1990	Age-standardized		19.3% (15.8 to 23.2%)	75.5 (59.0 to 95.2)
Ischemic heart disease	Iraq	1995	Age-standardized		19.3% (15.9 to 23.3%)	80.9 (63.4 to 100.5)
Ischemic heart disease	Iraq	2000	Age-standardized		19.4% (15.9 to 23.4%)	82.7 (65.2 to 102.1)
Ischemic heart disease	Iraq	2005	Age-standardized		19.6% (16.2 to 23.8%)	81.3 (63.2 to 102.9)
Ischemic heart disease	Iraq	2010	Age-standardized		22.2% (18.5 to 26.8%)	87.2 (67.6 to 109.5)
Ischemic heart disease	Iraq	2015	Age-standardized		19.3% (15.8 to 23.3%)	69.8 (52.1 to 88.8)
Ischemic stroke	Iraq	1990	All Ages	1.0 (0.7 to 1.3)	15.6% (12.4 to 18.5%)	5.5 (4.2 to 7.1)
Ischemic stroke	Iraq	1995	All Ages	1.2 (1.0 to 1.6)	15.5% (12.3 to 18.5%)	6.1 (4.7 to 7.7)
Ischemic stroke	Iraq	2000	All Ages	1.4 (1.1 to 1.8)	15.7% (12.5 to 18.8%)	6.0 (4.6 to 7.6)
Ischemic stroke	Iraq	2005	All Ages	1.5 (1.2 to 2.0)	16.2% (13.0 to 19.2%)	5.7 (4.3 to 7.3)
Ischemic stroke	Iraq	2010	All Ages	1.9 (1.4 to 2.4)	19.2% (16.0 to 22.6%)	6.1 (4.6 to 7.9)
Ischemic stroke	Iraq	2015	All Ages	1.6 (1.2 to 2.2)	16.0% (12.8 to 19.0%)	4.5 (3.3 to 6.0)
Ischemic stroke	Iraq	1990	Age-standardized		15.0% (11.6 to 18.2%)	15.0 (11.0 to 19.6)
Ischemic stroke	Iraq	1995	Age-standardized		14.9% (11.5 to 18.2%)	16.8 (12.7 to 21.7)
Ischemic stroke	Iraq	2000	Age-standardized		14.9% (11.6 to 18.2%)	17.0 (12.7 to 21.8)
Ischemic stroke	Iraq	2005	Age-standardized		15.3% (11.8 to 18.6%)	16.7 (12.4 to 21.8)
Ischemic stroke	Iraq	2010	Age-standardized		18.0% (14.4 to 22.0%)	18.5 (13.6 to 24.0)
Ischemic stroke	Iraq	2015	Age-standardized		14.9% (11.3 to 18.3%)	13.8 (9.9 to 18.6)
Hemorrhagic stroke	Iraq	1990	All Ages	0.9 (0.7 to 1.2)	16.6% (13.9 to 19.3%)	5.2 (4.0 to 6.6)
Hemorrhagic stroke	Iraq	1995	All Ages	1.1 (0.9 to 1.4)	16.7% (14.0 to 19.5%)	5.5 (4.3 to 6.9)
Hemorrhagic stroke	Iraq	2000	All Ages	1.3 (1.0 to 1.6)	17.0% (14.3 to 19.9%)	5.4 (4.2 to 6.8)
Hemorrhagic stroke	Iraq	2005	All Ages	1.4 (1.1 to 1.8)	17.7% (14.9 to 20.4%)	5.2 (4.1 to 6.6)
Hemorrhagic stroke	Iraq	2010	All Ages	1.8 (1.4 to 2.4)	21.2% (18.3 to 24.2%)	5.8 (4.4 to 7.7)
Hemorrhagic stroke	Iraq	2015	All Ages	1.6 (1.2 to 2.1)	17.8% (15.0 to 20.6%)	4.4 (3.2 to 5.9)
Hemorrhagic stroke	Iraq	1990	Age-standardized		16.6% (13.7 to 19.6%)	12.3 (9.4 to 15.7)
Hemorrhagic stroke	Iraq	1995	Age-standardized		16.6% (13.6 to 19.6%)	13.1 (10.2 to 16.4)
Hemorrhagic stroke	Iraq	2000	Age-standardized		16.7% (13.7 to 19.8%)	13.0 (10.1 to 16.6)
Hemorrhagic stroke	Iraq	2005	Age-standardized		17.0% (14.0 to 20.0%)	12.8 (9.7 to 16.3)
Hemorrhagic stroke	Iraq	2010	Age-standardized		20.1% (16.9 to 23.4%)	14.3 (10.8 to 18.8)

Cause name	Location	Year	Age group	Number (in thousands)	PAF	Rate (per 100,000)
Hemorrhagic stroke	Iraq	2015	Age-standardized		16.6% (13.7 to 19.5%)	10.7 (7.8 to 14.1)
Chronic obstructive pulmonary disease	Iraq	1990	All Ages	0.3 (0.1 to 0.4)	28.2% (17.3 to 40.4%)	1.5 (0.9 to 2.4)
Chronic obstructive pulmonary disease	Iraq	1995	All Ages	0.3 (0.2 to 0.5)	27.9% (17.1 to 40.0%)	1.5 (0.9 to 2.4)
Chronic obstructive pulmonary disease	Iraq	2000	All Ages	0.4 (0.2 to 0.6)	28.1% (17.2 to 40.1%)	1.5 (0.9 to 2.4)
Chronic obstructive pulmonary disease	Iraq	2005	All Ages	0.4 (0.2 to 0.6)	29.2% (18.3 to 41.1%)	1.5 (0.8 to 2.3)
Chronic obstructive pulmonary disease	Iraq	2010	All Ages	0.5 (0.3 to 0.8)	35.9% (24.5 to 51.1%)	1.7 (1.0 to 2.6)
Chronic obstructive pulmonary disease	Iraq	2015	All Ages	0.5 (0.3 to 0.7)	28.6% (17.7 to 40.8%)	1.3 (0.7 to 2.0)
Chronic obstructive pulmonary disease	Iraq	1990	Age-standardized		28.9% (17.9 to 41.0%)	4.2 (2.4 to 6.6)
Chronic obstructive pulmonary disease	Iraq	1995	Age-standardized		28.6% (17.6 to 40.9%)	4.4 (2.5 to 6.9)
Chronic obstructive pulmonary disease	Iraq	2000	Age-standardized		28.7% (17.6 to 41.0%)	4.5 (2.5 to 7.1)
Chronic obstructive pulmonary disease	Iraq	2005	Age-standardized		29.7% (18.7 to 42.1%)	4.5 (2.5 to 7.0)
Chronic obstructive pulmonary disease	Iraq	2010	Age-standardized		36.6% (24.9 to 52.0%)	5.4 (3.1 to 8.2)
Chronic obstructive pulmonary disease	Iraq	2015	Age-standardized		29.0% (17.9 to 41.3%)	4.0 (2.2 to 6.3)
All causes	Jordan	1990	All Ages	1.0 (0.8 to 1.2)	6.6% (5.7 to 7.6%)	29.8 (24.9 to 36.2)
All causes	Jordan	1995	All Ages	1.1 (0.9 to 1.3)	6.5% (5.7 to 7.4%)	25.6 (21.5 to 30.4)
All causes	Jordan	2000	All Ages	1.1 (0.9 to 1.3)	6.4% (5.6 to 7.2%)	23.2 (19.6 to 26.9)
All causes	Jordan	2005	All Ages	1.1 (1.0 to 1.3)	6.2% (5.4 to 7.0%)	20.8 (17.9 to 23.8)
All causes	Jordan	2010	All Ages	1.1 (1.0 to 1.3)	6.6% (5.8 to 7.4%)	17.4 (15.2 to 19.5)
All causes	Jordan	2015	All Ages	1.4 (1.2 to 1.6)	6.9% (6.1 to 7.8%)	18.3 (15.5 to 21.3)
All causes	Jordan	1990	Age-standardized		8.1% (7.0 to 9.3%)	74.4 (61.4 to 91.4)
All causes	Jordan	1995	Age-standardized		7.9% (6.8 to 9.0%)	69.6 (58.3 to 84.4)
All causes	Jordan	2000	Age-standardized		7.6% (6.5 to 8.7%)	64.6 (54.4 to 76.1)
All causes	Jordan	2005	Age-standardized		7.2% (6.2 to 8.3%)	57.3 (49.3 to 66.5)
All causes	Jordan	2010	Age-standardized		7.5% (6.5 to 8.5%)	43.7 (37.6 to 49.9)
All causes	Jordan	2015	Age-standardized		7.6% (6.6 to 8.7%)	42.8 (35.5 to 50.3)
Lower respiratory infections	Jordan	1990	All Ages	0.2 (0.1 to 0.3)	23.6% (14.9 to 32.7%)	6.0 (3.7 to 8.5)
Lower respiratory infections	Jordan	1995	All Ages	0.2 (0.1 to 0.3)	22.9% (14.5 to 32.2%)	4.4 (2.8 to 6.4)
Lower respiratory infections	Jordan	2000	All Ages	0.2 (0.1 to 0.3)	22.5% (14.0 to 31.9%)	3.8 (2.3 to 5.5)
Lower respiratory infections	Jordan	2005	All Ages	0.2 (0.1 to 0.2)	22.2% (14.0 to 31.6%)	3.1 (1.9 to 4.4)
Lower respiratory infections	Jordan	2010	All Ages	0.2 (0.1 to 0.3)	25.6% (17.5 to 33.8%)	2.9 (2.0 to 4.0)
Lower respiratory infections	Jordan	2015	All Ages	0.2 (0.1 to 0.3)	26.9% (19.1 to 34.9%)	2.9 (2.0 to 3.9)
Lower respiratory infections	Jordan	1990	Age-standardized		23.6% (14.9 to 32.7%)	9.1 (5.8 to 13.2)
Lower respiratory infections	Jordan	1995	Age-standardized		22.9% (14.5 to 32.2%)	8.0 (5.0 to 11.6)
Lower respiratory infections	Jordan	2000	Age-standardized		22.5% (14.0 to 31.9%)	7.3 (4.6 to 10.9)
Lower respiratory infections	Jordan	2005	Age-standardized		22.2% (14.0 to 31.6%)	6.3 (3.9 to 9.4)
Lower respiratory infections	Jordan	2010	Age-standardized		25.6% (17.5 to 33.8%)	5.8 (3.8 to 8.1)

Cause name	Location	Year	Age group	Number (in thousands)	PAF	Rate (per 100,000)
Lower respiratory infections	Jordan	2015	Age-standardized		26.9% (19.1 to 34.9%)	5.7 (3.8 to 8.0)
Tracheal, bronchus, and lung cancer	Jordan	1990	All Ages	0.0 (0.0 to 0.0)	16.7% (9.9 to 23.8%)	0.9 (0.5 to 1.3)
Tracheal, bronchus, and lung cancer	Jordan	1995	All Ages	0.0 (0.0 to 0.1)	16.2% (9.5 to 23.2%)	0.8 (0.5 to 1.3)
Tracheal, bronchus, and lung cancer	Jordan	2000	All Ages	0.0 (0.0 to 0.1)	15.9% (9.3 to 23.1%)	0.8 (0.5 to 1.2)
Tracheal, bronchus, and lung cancer	Jordan	2005	All Ages	0.1 (0.0 to 0.1)	15.7% (9.1 to 22.5%)	0.9 (0.5 to 1.3)
Tracheal, bronchus, and lung cancer	Jordan	2010	All Ages	0.1 (0.0 to 0.1)	18.3% (11.2 to 25.9%)	1.1 (0.6 to 1.5)
Tracheal, bronchus, and lung cancer	Jordan	2015	All Ages	0.1 (0.1 to 0.1)	19.3% (12.0 to 27.0%)	1.2 (0.7 to 1.7)
Tracheal, bronchus, and lung cancer	Jordan	1990	Age-standardized		16.8% (9.9 to 24.0%)	2.3 (1.3 to 3.4)
Tracheal, bronchus, and lung cancer	Jordan	1995	Age-standardized		16.3% (9.6 to 23.3%)	2.2 (1.3 to 3.3)
Tracheal, bronchus, and lung cancer	Jordan	2000	Age-standardized		16.0% (9.4 to 23.2%)	2.1 (1.2 to 3.1)
Tracheal, bronchus, and lung cancer	Jordan	2005	Age-standardized		15.7% (9.2 to 22.7%)	2.2 (1.3 to 3.2)
Tracheal, bronchus, and lung cancer	Jordan	2010	Age-standardized		18.4% (11.3 to 26.1%)	2.5 (1.5 to 3.5)
Tracheal, bronchus, and lung cancer	Jordan	2015	Age-standardized		19.4% (12.0 to 27.1%)	2.5 (1.5 to 3.6)
Cardiovascular diseases	Jordan	1990	All Ages	0.7 (0.6 to 0.8)	14.2% (12.2 to 16.3%)	20.7 (16.8 to 25.6)
Cardiovascular diseases	Jordan	1995	All Ages	0.8 (0.7 to 1.0)	14.3% (12.3 to 16.4%)	18.6 (15.2 to 22.5)
Cardiovascular diseases	Jordan	2000	All Ages	0.8 (0.7 to 1.0)	14.0% (12.0 to 16.0%)	17.0 (14.3 to 20.0)
Cardiovascular diseases	Jordan	2005	All Ages	0.8 (0.7 to 1.0)	13.6% (11.6 to 15.5%)	15.4 (12.9 to 17.9)
Cardiovascular diseases	Jordan	2010	All Ages	0.8 (0.7 to 0.9)	14.2% (12.3 to 16.3%)	12.3 (10.6 to 14.1)
Cardiovascular diseases	Jordan	2015	All Ages	1.0 (0.8 to 1.2)	14.5% (12.6 to 16.6%)	13.0 (10.9 to 15.6)
Cardiovascular diseases	Jordan	1990	Age-standardized		13.6% (11.4 to 15.7%)	56.4 (45.7 to 69.9)
Cardiovascular diseases	Jordan	1995	Age-standardized		13.2% (11.1 to 15.3%)	53.5 (43.9 to 65.2)
Cardiovascular diseases	Jordan	2000	Age-standardized		12.8% (10.7 to 15.0%)	49.8 (41.1 to 59.3)
Cardiovascular diseases	Jordan	2005	Age-standardized		12.4% (10.3 to 14.5%)	43.9 (36.3 to 52.6)
Cardiovascular diseases	Jordan	2010	Age-standardized		12.9% (10.8 to 15.1%)	32.0 (26.8 to 37.8)
Cardiovascular diseases	Jordan	2015	Age-standardized		13.2% (11.1 to 15.5%)	31.2 (25.3 to 37.5)
Ischemic heart disease	Jordan	1990	All Ages	0.5 (0.4 to 0.6)	18.6% (15.5 to 21.8%)	14.8 (11.6 to 18.5)
Ischemic heart disease	Jordan	1995	All Ages	0.6 (0.5 to 0.7)	18.8% (15.7 to 22.0%)	13.2 (10.5 to 16.4)
Ischemic heart disease	Jordan	2000	All Ages	0.6 (0.5 to 0.7)	18.6% (15.5 to 21.7%)	11.9 (9.6 to 14.5)
Ischemic heart disease	Jordan	2005	All Ages	0.6 (0.5 to 0.7)	18.2% (15.2 to 21.4%)	10.7 (8.7 to 12.9)
Ischemic heart disease	Jordan	2010	All Ages	0.6 (0.5 to 0.7)	19.6% (16.4 to 22.9%)	8.8 (7.2 to 10.4)
Ischemic heart disease	Jordan	2015	All Ages	0.7 (0.6 to 0.9)	20.0% (16.8 to 23.4%)	9.4 (7.6 to 11.4)
Ischemic heart disease	Jordan	1990	Age-standardized		17.6% (14.3 to 21.1%)	39.4 (30.6 to 49.7)
Ischemic heart disease	Jordan	1995	Age-standardized		17.2% (13.8 to 20.6%)	36.8 (28.5 to 46.0)
Ischemic heart disease	Jordan	2000	Age-standardized		16.8% (13.5 to 20.2%)	33.8 (26.4 to 41.5)
Ischemic heart disease	Jordan	2005	Age-standardized		16.5% (13.1 to 19.9%)	29.6 (23.2 to 36.3)
Ischemic heart disease	Jordan	2010	Age-standardized		17.8% (14.4 to 21.4%)	22.1 (17.6 to 27.0)

Cause name	Location	Year	Age group	Number (in thousands)	PAF	Rate (per 100,000)
Ischemic heart disease	Jordan	2015	Age-standardized		18.1% (14.7 to 22.0%)	21.6 (16.7 to 27.0)
Ischemic stroke	Jordan	1990	All Ages	0.1 (0.1 to 0.2)	13.1% (10.0 to 16.3%)	4.0 (2.9 to 5.3)
Ischemic stroke	Jordan	1995	All Ages	0.1 (0.1 to 0.2)	13.1% (10.0 to 16.1%)	3.6 (2.6 to 4.7)
Ischemic stroke	Jordan	2000	All Ages	0.2 (0.1 to 0.2)	13.0% (9.9 to 15.9%)	3.4 (2.4 to 4.3)
Ischemic stroke	Jordan	2005	All Ages	0.2 (0.1 to 0.2)	12.8% (9.7 to 15.7%)	3.2 (2.3 to 4.0)
Ischemic stroke	Jordan	2010	All Ages	0.2 (0.1 to 0.2)	13.7% (10.5 to 16.8%)	2.4 (1.8 to 3.0)
Ischemic stroke	Jordan	2015	All Ages	0.2 (0.1 to 0.2)	13.9% (10.5 to 17.1%)	2.5 (1.8 to 3.2)
Ischemic stroke	Jordan	1990	Age-standardized		12.6% (9.2 to 15.9%)	12.0 (8.5 to 16.0)
Ischemic stroke	Jordan	1995	Age-standardized		12.3% (8.9 to 15.7%)	11.9 (8.3 to 15.9)
Ischemic stroke	Jordan	2000	Age-standardized		12.1% (8.7 to 15.6%)	11.6 (8.0 to 15.5)
Ischemic stroke	Jordan	2005	Age-standardized		11.9% (8.4 to 15.4%)	10.4 (7.2 to 13.9)
Ischemic stroke	Jordan	2010	Age-standardized		12.9% (9.2 to 16.6%)	7.3 (5.1 to 9.5)
Ischemic stroke	Jordan	2015	Age-standardized		13.1% (9.5 to 17.0%)	7.1 (4.8 to 9.4)
Hemorrhagic stroke	Jordan	1990	All Ages	0.1 (0.1 to 0.1)	14.5% (11.7 to 17.4%)	1.9 (1.5 to 2.5)
Hemorrhagic stroke	Jordan	1995	All Ages	0.1 (0.1 to 0.1)	14.7% (12.0 to 17.6%)	1.8 (1.3 to 2.3)
Hemorrhagic stroke	Jordan	2000	All Ages	0.1 (0.1 to 0.1)	14.5% (11.7 to 17.4%)	1.7 (1.3 to 2.1)
Hemorrhagic stroke	Jordan	2005	All Ages	0.1 (0.1 to 0.1)	14.2% (11.3 to 17.0%)	1.5 (1.1 to 1.9)
Hemorrhagic stroke	Jordan	2010	All Ages	0.1 (0.1 to 0.1)	15.4% (12.5 to 18.2%)	1.1 (0.9 to 1.4)
Hemorrhagic stroke	Jordan	2015	All Ages	0.1 (0.1 to 0.1)	15.8% (13.0 to 18.5%)	1.1 (0.9 to 1.4)
Hemorrhagic stroke	Jordan	1990	Age-standardized		14.2% (11.3 to 17.1%)	5.0 (3.7 to 6.7)
Hemorrhagic stroke	Jordan	1995	Age-standardized		13.8% (10.8 to 16.8%)	4.7 (3.5 to 6.2)
Hemorrhagic stroke	Jordan	2000	Age-standardized		13.6% (10.6 to 16.5%)	4.5 (3.3 to 5.9)
Hemorrhagic stroke	Jordan	2005	Age-standardized		13.2% (10.2 to 16.3%)	3.8 (2.9 to 5.2)
Hemorrhagic stroke	Jordan	2010	Age-standardized		14.3% (11.3 to 17.3%)	2.6 (2.0 to 3.5)
Hemorrhagic stroke	Jordan	2015	Age-standardized		14.6% (11.5 to 17.6%)	2.5 (1.9 to 3.4)
Chronic obstructive pulmonary disease	Jordan	1990	All Ages	0.1 (0.0 to 0.1)	22.9% (12.3 to 33.8%)	2.2 (1.2 to 3.4)
Chronic obstructive pulmonary disease	Jordan	1995	All Ages	0.1 (0.0 to 0.1)	22.3% (11.7 to 33.2%)	1.8 (0.9 to 2.8)
Chronic obstructive pulmonary disease	Jordan	2000	All Ages	0.1 (0.0 to 0.1)	22.2% (11.6 to 33.1%)	1.6 (0.8 to 2.5)
Chronic obstructive pulmonary disease	Jordan	2005	All Ages	0.1 (0.0 to 0.1)	22.1% (11.7 to 33.0%)	1.5 (0.8 to 2.3)
Chronic obstructive pulmonary disease	Jordan	2010	All Ages	0.1 (0.0 to 0.1)	24.7% (14.0 to 36.1%)	1.1 (0.6 to 1.7)
Chronic obstructive pulmonary disease	Jordan	2015	All Ages	0.1 (0.1 to 0.1)	25.7% (14.9 to 37.1%)	1.2 (0.7 to 1.8)
Chronic obstructive pulmonary disease	Jordan	1990	Age-standardized		23.4% (12.5 to 34.4%)	6.7 (3.5 to 10.4)
Chronic obstructive pulmonary disease	Jordan	1995	Age-standardized		22.9% (12.0 to 34.0%)	6.0 (3.1 to 9.4)
Chronic obstructive pulmonary disease	Jordan	2000	Age-standardized		22.6% (11.9 to 33.7%)	5.5 (2.8 to 8.4)
Chronic obstructive pulmonary disease	Jordan	2005	Age-standardized		22.4% (11.9 to 33.4%)	4.9 (2.6 to 7.5)
Chronic obstructive pulmonary disease	Jordan	2010	Age-standardized		25.0% (14.1 to 36.5%)	3.4 (1.9 to 5.1)

Cause name	Location	Year	Age group	Number (in thousands)	PAF	Rate (per 100,000)
Chronic obstructive pulmonary disease	Jordan	2015	Age-standardized		25.9% (15.1 to 37.4%)	3.4 (1.9 to 5.1)
All causes	Kuwait	1990	All Ages	0.4 (0.3 to 0.4)	8.2% (6.9 to 9.7%)	17.2 (15.1 to 19.4)
All causes	Kuwait	1995	All Ages	0.4 (0.3 to 0.4)	9.9% (8.8 to 11.0%)	22.0 (19.6 to 24.5)
All causes	Kuwait	2000	All Ages	0.4 (0.4 to 0.4)	10.1% (9.0 to 11.2%)	20.2 (18.1 to 22.4)
All causes	Kuwait	2005	All Ages	0.5 (0.5 to 0.6)	10.7% (9.5 to 11.8%)	22.3 (20.0 to 24.7)
All causes	Kuwait	2010	All Ages	0.7 (0.6 to 0.8)	12.4% (11.2 to 13.7%)	22.6 (20.1 to 25.0)
All causes	Kuwait	2015	All Ages	0.7 (0.6 to 0.8)	11.3% (10.0 to 12.6%)	17.6 (14.5 to 21.1)
All causes	Kuwait	1990	Age-standardized		10.3% (8.9 to 11.8%)	60.9 (53.2 to 69.9)
All causes	Kuwait	1995	Age-standardized		11.1% (9.7 to 12.6%)	75.2 (65.6 to 85.7)
All causes	Kuwait	2000	Age-standardized		10.7% (9.4 to 12.1%)	65.0 (57.2 to 74.2)
All causes	Kuwait	2005	Age-standardized		11.3% (10.0 to 12.8%)	67.2 (59.4 to 75.8)
All causes	Kuwait	2010	Age-standardized		13.3% (11.9 to 14.9%)	76.0 (66.9 to 85.5)
All causes	Kuwait	2015	Age-standardized		11.5% (10.1 to 13.0%)	54.6 (46.3 to 64.8)
Lower respiratory infections	Kuwait	1990	All Ages	0.1 (0.1 to 0.1)	35.5% (26.9 to 43.3%)	3.0 (2.3 to 3.8)
Lower respiratory infections	Kuwait	1995	All Ages	0.1 (0.0 to 0.1)	35.1% (26.8 to 42.7%)	3.5 (2.7 to 4.4)
Lower respiratory infections	Kuwait	2000	All Ages	0.1 (0.0 to 0.1)	36.0% (27.7 to 43.6%)	2.9 (2.2 to 3.5)
Lower respiratory infections	Kuwait	2005	All Ages	0.1 (0.1 to 0.1)	35.9% (27.8 to 43.9%)	3.1 (2.4 to 3.8)
Lower respiratory infections	Kuwait	2010	All Ages	0.1 (0.1 to 0.1)	43.6% (33.6 to 52.8%)	4.0 (3.0 to 4.9)
Lower respiratory infections	Kuwait	2015	All Ages	0.1 (0.1 to 0.1)	36.4% (28.3 to 44.5%)	2.8 (2.1 to 3.6)
Lower respiratory infections	Kuwait	1990	Age-standardized		35.5% (26.9 to 43.3%)	11.3 (8.4 to 14.4)
Lower respiratory infections	Kuwait	1995	Age-standardized		35.1% (26.8 to 42.7%)	13.8 (10.3 to 17.2)
Lower respiratory infections	Kuwait	2000	Age-standardized		36.0% (27.7 to 43.6%)	12.4 (9.3 to 15.4)
Lower respiratory infections	Kuwait	2005	Age-standardized		35.9% (27.8 to 43.9%)	13.4 (10.2 to 16.7)
Lower respiratory infections	Kuwait	2010	Age-standardized		43.6% (33.6 to 52.8%)	19.2 (14.5 to 23.8)
Lower respiratory infections	Kuwait	2015	Age-standardized		36.4% (28.3 to 44.5%)	13.8 (10.3 to 17.6)
Tracheal, bronchus, and lung cancer	Kuwait	1990	All Ages	0.0 (0.0 to 0.0)	26.7% (17.9 to 35.4%)	0.8 (0.5 to 1.1)
Tracheal, bronchus, and lung cancer	Kuwait	1995	All Ages	0.0 (0.0 to 0.0)	26.3% (17.6 to 35.1%)	1.0 (0.6 to 1.3)
Tracheal, bronchus, and lung cancer	Kuwait	2000	All Ages	0.0 (0.0 to 0.0)	27.1% (18.7 to 35.7%)	0.9 (0.6 to 1.2)
Tracheal, bronchus, and lung cancer	Kuwait	2005	All Ages	0.0 (0.0 to 0.0)	27.1% (18.3 to 36.2%)	1.0 (0.6 to 1.3)
Tracheal, bronchus, and lung cancer	Kuwait	2010	All Ages	0.0 (0.0 to 0.0)	35.0% (25.3 to 43.9%)	1.0 (0.7 to 1.3)
Tracheal, bronchus, and lung cancer	Kuwait	2015	All Ages	0.0 (0.0 to 0.0)	27.5% (18.4 to 36.2%)	0.8 (0.5 to 1.1)
Tracheal, bronchus, and lung cancer	Kuwait	1990	Age-standardized		26.8% (18.0 to 35.5%)	3.2 (2.2 to 4.3)
Tracheal, bronchus, and lung cancer	Kuwait	1995	Age-standardized		26.4% (17.6 to 35.2%)	3.8 (2.5 to 5.1)
Tracheal, bronchus, and lung cancer	Kuwait	2000	Age-standardized		27.2% (18.7 to 35.7%)	3.3 (2.2 to 4.3)
Tracheal, bronchus, and lung cancer	Kuwait	2005	Age-standardized		27.1% (18.3 to 36.4%)	3.3 (2.2 to 4.4)
Tracheal, bronchus, and lung cancer	Kuwait	2010	Age-standardized		35.1% (25.4 to 44.1%)	4.0 (2.9 to 5.0)

Cause name	Location	Year	Age group	Number (in thousands)	PAF	Rate (per 100,000)
Tracheal, bronchus, and lung cancer	Kuwait	2015	Age-standardized		27.6% (18.4 to 36.3%)	2.8 (1.8 to 3.8)
Cardiovascular diseases	Kuwait	1990	All Ages	0.3 (0.2 to 0.3)	21.2% (18.6 to 23.9%)	13.0 (11.2 to 15.0)
Cardiovascular diseases	Kuwait	1995	All Ages	0.3 (0.2 to 0.3)	21.3% (18.6 to 24.1%)	17.0 (14.8 to 19.2)
Cardiovascular diseases	Kuwait	2000	All Ages	0.3 (0.3 to 0.4)	21.4% (18.9 to 24.0%)	15.9 (14.0 to 17.9)
Cardiovascular diseases	Kuwait	2005	All Ages	0.4 (0.4 to 0.5)	21.8% (19.2 to 24.5%)	17.6 (15.4 to 19.8)
Cardiovascular diseases	Kuwait	2010	All Ages	0.5 (0.5 to 0.6)	24.4% (21.7 to 27.2%)	16.9 (14.9 to 18.9)
Cardiovascular diseases	Kuwait	2015	All Ages	0.5 (0.4 to 0.6)	22.2% (19.5 to 25.1%)	13.5 (10.9 to 16.4)
Cardiovascular diseases	Kuwait	1990	Age-standardized		17.1% (14.6 to 20.2%)	44.2 (37.4 to 52.4)
Cardiovascular diseases	Kuwait	1995	Age-standardized		17.0% (14.5 to 20.1%)	54.7 (46.5 to 64.5)
Cardiovascular diseases	Kuwait	2000	Age-standardized		16.7% (14.4 to 19.7%)	46.7 (40.0 to 55.0)
Cardiovascular diseases	Kuwait	2005	Age-standardized		17.3% (14.9 to 20.1%)	47.6 (41.1 to 54.9)
Cardiovascular diseases	Kuwait	2010	Age-standardized		19.3% (16.9 to 22.2%)	49.7 (42.8 to 57.8)
Cardiovascular diseases	Kuwait	2015	Age-standardized		17.1% (14.7 to 20.0%)	35.8 (29.3 to 43.6)
Ischemic heart disease	Kuwait	1990	All Ages	0.2 (0.2 to 0.3)	26.0% (22.3 to 29.6%)	11.3 (9.5 to 13.1)
Ischemic heart disease	Kuwait	1995	All Ages	0.3 (0.2 to 0.3)	26.1% (22.4 to 29.8%)	14.8 (12.8 to 17.0)
Ischemic heart disease	Kuwait	2000	All Ages	0.3 (0.2 to 0.3)	26.8% (23.3 to 30.5%)	13.7 (11.7 to 15.6)
Ischemic heart disease	Kuwait	2005	All Ages	0.3 (0.3 to 0.4)	26.8% (22.9 to 30.6%)	14.3 (12.2 to 16.4)
Ischemic heart disease	Kuwait	2010	All Ages	0.4 (0.4 to 0.5)	29.8% (26.0 to 33.8%)	13.4 (11.5 to 15.3)
Ischemic heart disease	Kuwait	2015	All Ages	0.4 (0.3 to 0.5)	27.1% (23.2 to 31.2%)	11.1 (8.8 to 13.7)
Ischemic heart disease	Kuwait	1990	Age-standardized		21.0% (17.5 to 25.1%)	37.4 (30.9 to 45.6)
Ischemic heart disease	Kuwait	1995	Age-standardized		21.0% (17.3 to 25.2%)	46.5 (38.4 to 55.6)
Ischemic heart disease	Kuwait	2000	Age-standardized		21.5% (17.9 to 25.8%)	38.5 (32.0 to 46.5)
Ischemic heart disease	Kuwait	2005	Age-standardized		21.8% (18.3 to 25.9%)	36.5 (30.8 to 43.2)
Ischemic heart disease	Kuwait	2010	Age-standardized		24.0% (20.4 to 28.9%)	36.4 (30.7 to 43.9)
Ischemic heart disease	Kuwait	2015	Age-standardized		21.5% (18.0 to 25.9%)	27.2 (21.8 to 33.6)
Ischemic stroke	Kuwait	1990	All Ages	0.0 (0.0 to 0.0)	19.3% (16.2 to 22.2%)	0.9 (0.8 to 1.1)
Ischemic stroke	Kuwait	1995	All Ages	0.0 (0.0 to 0.0)	19.1% (16.0 to 22.1%)	1.2 (1.0 to 1.4)
Ischemic stroke	Kuwait	2000	All Ages	0.0 (0.0 to 0.0)	19.7% (16.6 to 22.7%)	1.4 (1.1 to 1.6)
Ischemic stroke	Kuwait	2005	All Ages	0.1 (0.0 to 0.1)	19.3% (16.2 to 22.4%)	2.0 (1.7 to 2.4)
Ischemic stroke	Kuwait	2010	All Ages	0.1 (0.1 to 0.1)	22.0% (18.6 to 25.4%)	2.2 (1.8 to 2.6)
Ischemic stroke	Kuwait	2015	All Ages	0.1 (0.0 to 0.1)	19.1% (15.9 to 22.1%)	1.4 (1.1 to 1.8)
Ischemic stroke	Kuwait	1990	Age-standardized		16.1% (12.4 to 20.1%)	4.6 (3.5 to 5.8)
Ischemic stroke	Kuwait	1995	Age-standardized		15.9% (12.1 to 19.9%)	5.7 (4.3 to 7.2)
Ischemic stroke	Kuwait	2000	Age-standardized		16.3% (12.4 to 20.2%)	6.1 (4.6 to 7.6)
Ischemic stroke	Kuwait	2005	Age-standardized		16.3% (12.6 to 20.3%)	8.5 (6.5 to 10.6)
Ischemic stroke	Kuwait	2010	Age-standardized		18.9% (14.9 to 23.6%)	10.3 (8.0 to 13.0)

Cause name	Location	Year	Age group	Number (in thousands)	PAF	Rate (per 100,000)
Ischemic stroke	Kuwait	2015	Age-standardized		16.2% (12.3 to 20.2%)	6.5 (4.8 to 8.3)
Hemorrhagic stroke	Kuwait	1990	All Ages	0.0 (0.0 to 0.0)	20.4% (17.5 to 23.6%)	0.8 (0.7 to 0.9)
Hemorrhagic stroke	Kuwait	1995	All Ages	0.0 (0.0 to 0.0)	20.8% (17.7 to 23.9%)	0.9 (0.8 to 1.1)
Hemorrhagic stroke	Kuwait	2000	All Ages	0.0 (0.0 to 0.0)	22.1% (19.0 to 25.4%)	0.9 (0.8 to 1.1)
Hemorrhagic stroke	Kuwait	2005	All Ages	0.0 (0.0 to 0.0)	22.6% (19.4 to 26.0%)	1.2 (1.1 to 1.5)
Hemorrhagic stroke	Kuwait	2010	All Ages	0.0 (0.0 to 0.1)	25.8% (22.4 to 29.4%)	1.3 (1.1 to 1.5)
Hemorrhagic stroke	Kuwait	2015	All Ages	0.0 (0.0 to 0.1)	22.7% (19.4 to 26.0%)	0.9 (0.7 to 1.2)
Hemorrhagic stroke	Kuwait	1990	Age-standardized		17.6% (14.5 to 20.5%)	2.2 (1.8 to 2.7)
Hemorrhagic stroke	Kuwait	1995	Age-standardized		17.5% (14.4 to 20.6%)	2.4 (2.0 to 2.9)
Hemorrhagic stroke	Kuwait	2000	Age-standardized		18.1% (15.0 to 21.2%)	2.1 (1.8 to 2.5)
Hemorrhagic stroke	Kuwait	2005	Age-standardized		18.6% (15.7 to 21.6%)	2.6 (2.2 to 3.1)
Hemorrhagic stroke	Kuwait	2010	Age-standardized		21.2% (17.9 to 24.8%)	3.0 (2.5 to 3.6)
Hemorrhagic stroke	Kuwait	2015	Age-standardized		18.2% (15.0 to 21.4%)	2.1 (1.6 to 2.7)
Chronic obstructive pulmonary disease	Kuwait	1990	All Ages	0.0 (0.0 to 0.0)	31.3% (20.2 to 44.5%)	0.4 (0.2 to 0.5)
Chronic obstructive pulmonary disease	Kuwait	1995	All Ages	0.0 (0.0 to 0.0)	31.3% (20.1 to 44.3%)	0.6 (0.4 to 0.8)
Chronic obstructive pulmonary disease	Kuwait	2000	All Ages	0.0 (0.0 to 0.0)	32.1% (20.6 to 44.9%)	0.5 (0.3 to 0.7)
Chronic obstructive pulmonary disease	Kuwait	2005	All Ages	0.0 (0.0 to 0.0)	32.2% (20.6 to 45.4%)	0.7 (0.4 to 0.9)
Chronic obstructive pulmonary disease	Kuwait	2010	All Ages	0.0 (0.0 to 0.0)	38.4% (26.7 to 54.1%)	0.6 (0.4 to 0.9)
Chronic obstructive pulmonary disease	Kuwait	2015	All Ages	0.0 (0.0 to 0.0)	32.7% (21.2 to 45.8%)	0.5 (0.3 to 0.7)
Chronic obstructive pulmonary disease	Kuwait	1990	Age-standardized		32.6% (21.1 to 46.4%)	2.1 (1.3 to 3.1)
Chronic obstructive pulmonary disease	Kuwait	1995	Age-standardized		32.3% (20.7 to 45.6%)	3.0 (1.9 to 4.3)
Chronic obstructive pulmonary disease	Kuwait	2000	Age-standardized		33.0% (21.1 to 46.1%)	2.7 (1.7 to 3.8)
Chronic obstructive pulmonary disease	Kuwait	2005	Age-standardized		32.9% (21.0 to 46.4%)	2.9 (1.9 to 4.1)
Chronic obstructive pulmonary disease	Kuwait	2010	Age-standardized		39.5% (27.5 to 55.6%)	3.2 (2.2 to 4.5)
Chronic obstructive pulmonary disease	Kuwait	2015	Age-standardized		33.3% (21.6 to 46.7%)	2.2 (1.4 to 3.1)
All causes	Lebanon	1990	All Ages	1.3 (1.0 to 1.6)	6.9% (5.8 to 7.9%)	47.5 (37.4 to 58.9)
All causes	Lebanon	1995	All Ages	1.2 (0.9 to 1.4)	7.2% (6.1 to 8.3%)	38.5 (31.1 to 46.5)
All causes	Lebanon	2000	All Ages	1.1 (0.9 to 1.3)	7.1% (6.0 to 8.2%)	33.2 (26.8 to 40.6)
All causes	Lebanon	2005	All Ages	1.3 (1.0 to 1.6)	7.2% (6.0 to 8.4%)	31.6 (24.3 to 40.2)
All causes	Lebanon	2010	All Ages	1.6 (1.2 to 2.0)	7.6% (6.4 to 8.9%)	36.4 (26.9 to 46.7)
All causes	Lebanon	2015	All Ages	2.1 (1.6 to 2.8)	7.9% (6.6 to 9.2%)	36.9 (26.9 to 48.0)
All causes	Lebanon	1990	Age-standardized		7.5% (6.3 to 8.7%)	76.1 (59.6 to 95.1)
All causes	Lebanon	1995	Age-standardized		7.5% (6.3 to 8.7%)	61.3 (49.6 to 74.6)
All causes	Lebanon	2000	Age-standardized		7.3% (6.0 to 8.6%)	50.5 (39.6 to 62.3)
All causes	Lebanon	2005	Age-standardized		7.1% (5.9 to 8.5%)	45.0 (34.4 to 57.5)
All causes	Lebanon	2010	Age-standardized		7.5% (6.3 to 8.9%)	46.1 (34.0 to 59.3)

Cause name	Location	Year	Age group	Number (in thousands)	PAF	Rate (per 100,000)
All causes	Lebanon	2015	Age-standardized		7.9% (6.5 to 9.4%)	45.0 (32.6 to 58.2)
Lower respiratory infections	Lebanon	1990	All Ages	0.1 (0.0 to 0.1)	19.7% (11.3 to 29.6%)	3.0 (1.6 to 4.7)
Lower respiratory infections	Lebanon	1995	All Ages	0.1 (0.0 to 0.1)	18.9% (10.2 to 28.9%)	2.1 (1.1 to 3.3)
Lower respiratory infections	Lebanon	2000	All Ages	0.1 (0.0 to 0.1)	19.6% (11.2 to 29.5%)	1.6 (0.9 to 2.5)
Lower respiratory infections	Lebanon	2005	All Ages	0.1 (0.0 to 0.1)	19.7% (11.1 to 29.7%)	1.2 (0.6 to 1.9)
Lower respiratory infections	Lebanon	2010	All Ages	0.1 (0.0 to 0.1)	21.8% (13.3 to 31.4%)	1.4 (0.7 to 2.2)
Lower respiratory infections	Lebanon	2015	All Ages	0.1 (0.1 to 0.1)	24.0% (15.6 to 33.1%)	1.4 (0.8 to 2.2)
Lower respiratory infections	Lebanon	1990	Age-standardized		19.7% (11.3 to 29.6%)	3.5 (1.9 to 5.5)
Lower respiratory infections	Lebanon	1995	Age-standardized		18.9% (10.2 to 28.9%)	2.7 (1.4 to 4.4)
Lower respiratory infections	Lebanon	2000	Age-standardized		19.6% (11.2 to 29.5%)	2.3 (1.2 to 3.6)
Lower respiratory infections	Lebanon	2005	Age-standardized		19.7% (11.1 to 29.7%)	1.8 (1.0 to 3.0)
Lower respiratory infections	Lebanon	2010	Age-standardized		21.8% (13.3 to 31.4%)	1.9 (1.0 to 3.0)
Lower respiratory infections	Lebanon	2015	Age-standardized		24.0% (15.6 to 33.1%)	1.8 (1.0 to 2.8)
Tracheal, bronchus, and lung cancer	Lebanon	1990	All Ages	0.1 (0.0 to 0.1)	13.8% (7.7 to 20.7%)	2.6 (1.4 to 4.2)
Tracheal, bronchus, and lung cancer	Lebanon	1995	All Ages	0.1 (0.0 to 0.1)	13.2% (7.4 to 20.0%)	2.1 (1.1 to 3.3)
Tracheal, bronchus, and lung cancer	Lebanon	2000	All Ages	0.1 (0.0 to 0.1)	13.8% (7.7 to 20.7%)	2.2 (1.2 to 3.5)
Tracheal, bronchus, and lung cancer	Lebanon	2005	All Ages	0.1 (0.1 to 0.2)	13.8% (7.8 to 20.7%)	2.7 (1.4 to 4.3)
Tracheal, bronchus, and lung cancer	Lebanon	2010	All Ages	0.1 (0.1 to 0.2)	15.4% (8.8 to 22.8%)	3.4 (1.8 to 5.5)
Tracheal, bronchus, and lung cancer	Lebanon	2015	All Ages	0.2 (0.1 to 0.3)	17.1% (10.2 to 24.3%)	3.6 (1.9 to 5.8)
Tracheal, bronchus, and lung cancer	Lebanon	1990	Age-standardized		13.9% (7.7 to 20.7%)	3.8 (2.0 to 6.2)
Tracheal, bronchus, and lung cancer	Lebanon	1995	Age-standardized		13.2% (7.4 to 20.1%)	3.0 (1.6 to 4.7)
Tracheal, bronchus, and lung cancer	Lebanon	2000	Age-standardized		13.8% (7.7 to 20.8%)	3.0 (1.6 to 4.8)
Tracheal, bronchus, and lung cancer	Lebanon	2005	Age-standardized		13.9% (7.8 to 20.7%)	3.5 (1.9 to 5.7)
Tracheal, bronchus, and lung cancer	Lebanon	2010	Age-standardized		15.5% (8.8 to 22.8%)	4.0 (2.1 to 6.4)
Tracheal, bronchus, and lung cancer	Lebanon	2015	Age-standardized		17.1% (10.2 to 24.4%)	4.3 (2.2 to 6.8)
Cardiovascular diseases	Lebanon	1990	All Ages	1.1 (0.8 to 1.3)	14.4% (12.2 to 16.6%)	38.9 (29.8 to 48.8)
Cardiovascular diseases	Lebanon	1995	All Ages	1.0 (0.8 to 1.2)	13.8% (11.6 to 16.0%)	31.9 (25.4 to 38.8)
Cardiovascular diseases	Lebanon	2000	All Ages	0.9 (0.7 to 1.1)	13.8% (11.6 to 16.1%)	27.3 (21.3 to 33.8)
Cardiovascular diseases	Lebanon	2005	All Ages	1.0 (0.8 to 1.3)	13.7% (11.4 to 16.0%)	25.6 (19.5 to 32.7)
Cardiovascular diseases	Lebanon	2010	All Ages	1.3 (0.9 to 1.6)	14.0% (11.8 to 16.6%)	29.0 (21.5 to 37.5)
Cardiovascular diseases	Lebanon	2015	All Ages	1.7 (1.2 to 2.2)	14.4% (11.9 to 17.2%)	29.1 (20.8 to 37.8)
Cardiovascular diseases	Lebanon	1990	Age-standardized		13.6% (11.4 to 16.1%)	63.3 (48.6 to 80.0)
Cardiovascular diseases	Lebanon	1995	Age-standardized		13.1% (10.7 to 15.5%)	51.3 (40.2 to 63.6)
Cardiovascular diseases	Lebanon	2000	Age-standardized		13.0% (10.6 to 15.5%)	41.6 (32.0 to 52.0)
Cardiovascular diseases	Lebanon	2005	Age-standardized		12.9% (10.5 to 15.6%)	36.4 (27.1 to 46.7)
Cardiovascular diseases	Lebanon	2010	Age-standardized		13.5% (10.9 to 16.4%)	36.8 (27.0 to 47.7)

Cause name	Location	Year	Age group	Number (in thousands)	PAF	Rate (per 100,000)
Cardiovascular diseases	Lebanon	2015	Age-standardized		14.1% (11.4 to 17.1%)	35.5 (25.3 to 46.3)
Ischemic heart disease	Lebanon	1990	All Ages	0.9 (0.7 to 1.1)	16.9% (14.0 to 20.0%)	32.3 (24.3 to 41.3)
Ischemic heart disease	Lebanon	1995	All Ages	0.8 (0.6 to 1.0)	16.3% (13.4 to 19.4%)	26.0 (20.3 to 32.2)
Ischemic heart disease	Lebanon	2000	All Ages	0.7 (0.6 to 0.9)	16.3% (13.2 to 19.4%)	22.1 (17.0 to 27.7)
Ischemic heart disease	Lebanon	2005	All Ages	0.8 (0.6 to 1.1)	16.2% (13.0 to 19.4%)	20.9 (15.5 to 27.1)
Ischemic heart disease	Lebanon	2010	All Ages	1.0 (0.7 to 1.4)	16.5% (13.3 to 19.8%)	23.9 (17.2 to 31.6)
Ischemic heart disease	Lebanon	2015	All Ages	1.4 (1.0 to 1.8)	16.9% (13.6 to 20.6%)	24.0 (16.7 to 32.0)
Ischemic heart disease	Lebanon	1990	Age-standardized		16.0% (12.8 to 19.2%)	52.3 (38.8 to 67.4)
Ischemic heart disease	Lebanon	1995	Age-standardized		15.4% (12.1 to 18.6%)	41.6 (31.8 to 52.7)
Ischemic heart disease	Lebanon	2000	Age-standardized		15.3% (11.8 to 18.8%)	33.5 (25.0 to 42.7)
Ischemic heart disease	Lebanon	2005	Age-standardized		15.3% (11.8 to 18.8%)	29.5 (21.0 to 38.5)
Ischemic heart disease	Lebanon	2010	Age-standardized		15.9% (12.3 to 19.5%)	30.0 (20.9 to 40.5)
Ischemic heart disease	Lebanon	2015	Age-standardized		16.5% (13.0 to 20.4%)	29.1 (20.1 to 38.8)
Ischemic stroke	Lebanon	1990	All Ages	0.1 (0.1 to 0.1)	11.8% (8.8 to 15.1%)	3.2 (2.2 to 4.5)
Ischemic stroke	Lebanon	1995	All Ages	0.1 (0.1 to 0.1)	11.5% (8.3 to 14.8%)	2.8 (1.8 to 3.9)
Ischemic stroke	Lebanon	2000	All Ages	0.1 (0.1 to 0.1)	11.6% (8.5 to 14.8%)	2.4 (1.5 to 3.5)
Ischemic stroke	Lebanon	2005	All Ages	0.1 (0.1 to 0.1)	11.4% (8.3 to 14.8%)	2.3 (1.5 to 3.3)
Ischemic stroke	Lebanon	2010	All Ages	0.1 (0.1 to 0.2)	11.8% (8.5 to 15.3%)	2.7 (1.7 to 3.8)
Ischemic stroke	Lebanon	2015	All Ages	0.2 (0.1 to 0.2)	12.3% (8.6 to 16.1%)	2.8 (1.7 to 4.1)
Ischemic stroke	Lebanon	1990	Age-standardized		11.2% (7.9 to 14.9%)	5.9 (3.7 to 8.3)
Ischemic stroke	Lebanon	1995	Age-standardized		10.8% (7.3 to 14.7%)	5.0 (3.1 to 7.3)
Ischemic stroke	Lebanon	2000	Age-standardized		10.9% (7.5 to 14.9%)	4.1 (2.4 to 6.1)
Ischemic stroke	Lebanon	2005	Age-standardized		10.9% (7.4 to 14.8%)	3.6 (2.2 to 5.3)
Ischemic stroke	Lebanon	2010	Age-standardized		11.4% (7.7 to 15.5%)	3.7 (2.2 to 5.5)
Ischemic stroke	Lebanon	2015	Age-standardized		12.0% (8.3 to 16.2%)	3.6 (2.2 to 5.3)
Hemorrhagic stroke	Lebanon	1990	All Ages	0.1 (0.1 to 0.1)	12.9% (10.3 to 15.5%)	3.3 (2.4 to 4.4)
Hemorrhagic stroke	Lebanon	1995	All Ages	0.1 (0.1 to 0.1)	12.6% (9.9 to 15.4%)	3.1 (2.2 to 4.2)
Hemorrhagic stroke	Lebanon	2000	All Ages	0.1 (0.1 to 0.1)	12.8% (10.1 to 15.6%)	2.7 (2.0 to 3.9)
Hemorrhagic stroke	Lebanon	2005	All Ages	0.1 (0.1 to 0.1)	12.7% (10.0 to 15.5%)	2.4 (1.7 to 3.4)
Hemorrhagic stroke	Lebanon	2010	All Ages	0.1 (0.1 to 0.1)	13.2% (10.5 to 16.1%)	2.5 (1.7 to 3.5)
Hemorrhagic stroke	Lebanon	2015	All Ages	0.1 (0.1 to 0.2)	13.8% (10.9 to 16.9%)	2.3 (1.5 to 3.3)
Hemorrhagic stroke	Lebanon	1990	Age-standardized		12.5% (9.7 to 15.3%)	5.2 (3.7 to 7.0)
Hemorrhagic stroke	Lebanon	1995	Age-standardized		12.1% (9.2 to 15.1%)	4.7 (3.3 to 6.5)
Hemorrhagic stroke	Lebanon	2000	Age-standardized		12.2% (9.3 to 15.2%)	4.0 (2.8 to 5.8)
Hemorrhagic stroke	Lebanon	2005	Age-standardized		12.1% (9.2 to 15.2%)	3.3 (2.2 to 4.8)
Hemorrhagic stroke	Lebanon	2010	Age-standardized		12.7% (9.7 to 15.9%)	3.1 (2.1 to 4.5)

Cause name	Location	Year	Age group	Number (in thousands)	PAF	Rate (per 100,000)
Hemorrhagic stroke	Lebanon	2015	Age-standardized		13.4% (10.3 to 16.8%)	2.8 (1.8 to 4.0)
Chronic obstructive pulmonary disease	Lebanon	1990	All Ages	0.1 (0.0 to 0.1)	19.9% (9.9 to 30.8%)	3.0 (1.5 to 4.8)
Chronic obstructive pulmonary disease	Lebanon	1995	All Ages	0.1 (0.0 to 0.1)	19.3% (9.7 to 30.0%)	2.4 (1.2 to 3.9)
Chronic obstructive pulmonary disease	Lebanon	2000	All Ages	0.1 (0.0 to 0.1)	20.1% (10.1 to 30.9%)	2.2 (1.0 to 3.5)
Chronic obstructive pulmonary disease	Lebanon	2005	All Ages	0.1 (0.0 to 0.1)	20.2% (9.9 to 30.9%)	2.1 (1.0 to 3.5)
Chronic obstructive pulmonary disease	Lebanon	2010	All Ages	0.1 (0.1 to 0.2)	22.0% (11.2 to 32.7%)	2.6 (1.3 to 4.2)
Chronic obstructive pulmonary disease	Lebanon	2015	All Ages	0.2 (0.1 to 0.3)	23.6% (12.8 to 35.0%)	2.7 (1.4 to 4.4)
Chronic obstructive pulmonary disease	Lebanon	1990	Age-standardized		20.2% (10.0 to 31.2%)	5.4 (2.6 to 8.6)
Chronic obstructive pulmonary disease	Lebanon	1995	Age-standardized		19.6% (9.8 to 30.3%)	4.3 (2.0 to 6.8)
Chronic obstructive pulmonary disease	Lebanon	2000	Age-standardized		20.3% (10.2 to 31.1%)	3.6 (1.7 to 5.9)
Chronic obstructive pulmonary disease	Lebanon	2005	Age-standardized		20.3% (9.9 to 31.0%)	3.3 (1.5 to 5.4)
Chronic obstructive pulmonary disease	Lebanon	2010	Age-standardized		22.0% (11.2 to 32.8%)	3.4 (1.7 to 5.6)
Chronic obstructive pulmonary disease	Lebanon	2015	Age-standardized		23.7% (12.9 to 35.1%)	3.4 (1.7 to 5.6)
All causes	Libya	1990	All Ages	1.2 (1.0 to 1.4)	7.6% (6.6 to 8.6%)	28.2 (23.6 to 32.8)
All causes	Libya	1995	All Ages	1.3 (1.1 to 1.5)	7.9% (6.9 to 8.9%)	26.5 (22.4 to 30.8)
All causes	Libya	2000	All Ages	1.5 (1.3 to 1.7)	8.2% (7.2 to 9.2%)	28.2 (24.5 to 32.4)
All causes	Libya	2005	All Ages	1.7 (1.5 to 1.9)	8.4% (7.4 to 9.4%)	29.3 (25.6 to 33.2)
All causes	Libya	2010	All Ages	2.0 (1.8 to 2.3)	9.2% (8.1 to 10.4%)	32.6 (28.4 to 36.9)
All causes	Libya	2015	All Ages	2.6 (2.2 to 3.1)	9.2% (8.0 to 10.5%)	41.5 (34.6 to 48.8)
All causes	Libya	1990	Age-standardized		9.8% (8.5 to 11.1%)	74.8 (63.2 to 87.4)
All causes	Libya	1995	Age-standardized		9.6% (8.4 to 10.9%)	68.7 (58.4 to 80.3)
All causes	Libya	2000	Age-standardized		9.6% (8.4 to 10.9%)	68.6 (59.3 to 79.5)
All causes	Libya	2005	Age-standardized		9.6% (8.4 to 10.9%)	66.8 (58.0 to 76.0)
All causes	Libya	2010	Age-standardized		10.3% (9.0 to 11.7%)	70.1 (60.3 to 80.0)
All causes	Libya	2015	Age-standardized		10.3% (9.0 to 11.8%)	79.6 (66.0 to 94.6)
Lower respiratory infections	Libya	1990	All Ages	0.3 (0.2 to 0.4)	32.5% (25.1 to 39.7%)	7.4 (5.2 to 9.9)
Lower respiratory infections	Libya	1995	All Ages	0.3 (0.2 to 0.4)	31.9% (24.3 to 39.1%)	5.4 (3.9 to 7.2)
Lower respiratory infections	Libya	2000	All Ages	0.3 (0.2 to 0.4)	31.8% (24.2 to 38.9%)	4.9 (3.5 to 6.5)
Lower respiratory infections	Libya	2005	All Ages	0.3 (0.2 to 0.4)	31.8% (24.3 to 38.7%)	4.8 (3.3 to 6.3)
Lower respiratory infections	Libya	2010	All Ages	0.3 (0.2 to 0.4)	35.4% (27.7 to 42.4%)	5.3 (3.7 to 6.8)
Lower respiratory infections	Libya	2015	All Ages	0.4 (0.3 to 0.5)	38.0% (29.9 to 45.4%)	6.1 (4.3 to 8.2)
Lower respiratory infections	Libya	1990	Age-standardized		32.5% (25.1 to 39.7%)	13.4 (9.4 to 18.6)
Lower respiratory infections	Libya	1995	Age-standardized		31.9% (24.3 to 39.1%)	11.3 (7.9 to 15.5)
Lower respiratory infections	Libya	2000	Age-standardized		31.8% (24.2 to 38.9%)	10.9 (7.5 to 15.2)
Lower respiratory infections	Libya	2005	Age-standardized		31.8% (24.3 to 38.7%)	10.5 (7.2 to 14.4)
Lower respiratory infections	Libya	2010	Age-standardized		35.4% (27.7 to 42.4%)	11.4 (7.8 to 15.4)

Cause name	Location	Year	Age group	Number (in thousands)	PAF	Rate (per 100,000)
Lower respiratory infections	Libya	2015	Age-standardized		38.0% (29.9 to 45.4%)	12.3 (8.4 to 17.2)
Tracheal, bronchus, and lung cancer	Libya	1990	All Ages	0.1 (0.1 to 0.1)	24.4% (16.2 to 32.6%)	2.1 (1.3 to 3.1)
Tracheal, bronchus, and lung cancer	Libya	1995	All Ages	0.1 (0.1 to 0.1)	23.8% (15.8 to 32.0%)	2.2 (1.4 to 3.1)
Tracheal, bronchus, and lung cancer	Libya	2000	All Ages	0.1 (0.1 to 0.2)	23.8% (15.6 to 31.8%)	2.4 (1.6 to 3.4)
Tracheal, bronchus, and lung cancer	Libya	2005	All Ages	0.1 (0.1 to 0.2)	23.7% (15.7 to 31.9%)	2.6 (1.7 to 3.5)
Tracheal, bronchus, and lung cancer	Libya	2010	All Ages	0.2 (0.1 to 0.3)	27.2% (18.6 to 35.6%)	3.2 (2.2 to 4.3)
Tracheal, bronchus, and lung cancer	Libya	2015	All Ages	0.3 (0.2 to 0.4)	29.8% (20.9 to 38.1%)	4.3 (2.8 to 5.8)
Tracheal, bronchus, and lung cancer	Libya	1990	Age-standardized		24.5% (16.3 to 32.6%)	5.6 (3.4 to 8.0)
Tracheal, bronchus, and lung cancer	Libya	1995	Age-standardized		23.9% (15.8 to 32.0%)	5.4 (3.3 to 7.6)
Tracheal, bronchus, and lung cancer	Libya	2000	Age-standardized		23.8% (15.6 to 31.8%)	5.5 (3.6 to 7.7)
Tracheal, bronchus, and lung cancer	Libya	2005	Age-standardized		23.8% (15.7 to 31.9%)	5.6 (3.7 to 7.7)
Tracheal, bronchus, and lung cancer	Libya	2010	Age-standardized		27.2% (18.6 to 35.6%)	6.6 (4.4 to 8.9)
Tracheal, bronchus, and lung cancer	Libya	2015	Age-standardized		29.8% (20.9 to 38.2%)	7.6 (5.0 to 10.3)
Cardiovascular diseases	Libya	1990	All Ages	0.7 (0.6 to 0.9)	15.0% (12.9 to 17.1%)	16.6 (13.4 to 19.7)
Cardiovascular diseases	Libya	1995	All Ages	0.8 (0.7 to 1.0)	15.6% (13.7 to 17.8%)	16.9 (13.8 to 20.0)
Cardiovascular diseases	Libya	2000	All Ages	1.0 (0.8 to 1.2)	16.1% (14.2 to 18.4%)	18.7 (16.0 to 21.9)
Cardiovascular diseases	Libya	2005	All Ages	1.1 (1.0 to 1.3)	16.4% (14.3 to 18.5%)	19.6 (16.9 to 22.8)
Cardiovascular diseases	Libya	2010	All Ages	1.4 (1.1 to 1.6)	17.6% (15.5 to 20.0%)	21.5 (18.2 to 25.1)
Cardiovascular diseases	Libya	2015	All Ages	1.8 (1.4 to 2.1)	18.8% (16.7 to 21.3%)	27.8 (22.7 to 33.8)
Cardiovascular diseases	Libya	1990	Age-standardized		15.6% (13.3 to 18.4%)	48.6 (39.5 to 58.8)
Cardiovascular diseases	Libya	1995	Age-standardized		15.5% (13.2 to 18.2%)	45.5 (37.6 to 54.8)
Cardiovascular diseases	Libya	2000	Age-standardized		15.6% (13.2 to 18.1%)	45.9 (38.6 to 54.9)
Cardiovascular diseases	Libya	2005	Age-standardized		15.6% (13.3 to 18.2%)	44.8 (37.3 to 53.0)
Cardiovascular diseases	Libya	2010	Age-standardized		16.7% (14.2 to 19.5%)	45.8 (37.6 to 54.4)
Cardiovascular diseases	Libya	2015	Age-standardized		17.5% (15.1 to 20.5%)	52.5 (42.4 to 65.0)
Ischemic heart disease	Libya	1990	All Ages	0.6 (0.4 to 0.7)	21.3% (18.1 to 24.6%)	12.7 (10.1 to 15.3)
Ischemic heart disease	Libya	1995	All Ages	0.6 (0.5 to 0.7)	21.1% (17.9 to 24.5%)	12.6 (9.9 to 15.1)
Ischemic heart disease	Libya	2000	All Ages	0.7 (0.6 to 0.9)	21.1% (17.9 to 24.6%)	13.7 (11.2 to 16.4)
Ischemic heart disease	Libya	2005	All Ages	0.8 (0.7 to 1.0)	21.0% (18.0 to 24.7%)	14.3 (11.9 to 17.1)
Ischemic heart disease	Libya	2010	All Ages	1.0 (0.8 to 1.2)	22.2% (18.9 to 26.0%)	15.7 (12.9 to 18.9)
Ischemic heart disease	Libya	2015	All Ages	1.3 (1.0 to 1.6)	23.2% (19.8 to 27.1%)	20.2 (15.9 to 25.1)
Ischemic heart disease	Libya	1990	Age-standardized		19.3% (15.8 to 23.7%)	36.6 (28.7 to 45.1)
Ischemic heart disease	Libya	1995	Age-standardized		19.1% (15.6 to 23.4%)	33.5 (26.3 to 41.6)
Ischemic heart disease	Libya	2000	Age-standardized		19.2% (15.7 to 23.5%)	33.1 (26.4 to 41.2)
Ischemic heart disease	Libya	2005	Age-standardized		19.3% (15.7 to 23.5%)	32.3 (26.0 to 39.7)
Ischemic heart disease	Libya	2010	Age-standardized		20.3% (16.7 to 24.5%)	33.0 (26.4 to 40.7)

Cause name	Location	Year	Age group	Number (in thousands)	PAF	Rate (per 100,000)
Ischemic heart disease	Libya	2015	Age-standardized		21.2% (17.5 to 25.8%)	37.7 (29.1 to 47.5)
Ischemic stroke	Libya	1990	All Ages	0.1 (0.1 to 0.1)	16.0% (12.9 to 18.8%)	1.8 (1.4 to 2.4)
Ischemic stroke	Libya	1995	All Ages	0.1 (0.1 to 0.1)	15.8% (12.6 to 18.7%)	2.0 (1.5 to 2.6)
Ischemic stroke	Libya	2000	All Ages	0.1 (0.1 to 0.2)	15.7% (12.6 to 18.7%)	2.4 (1.8 to 3.0)
Ischemic stroke	Libya	2005	All Ages	0.1 (0.1 to 0.2)	15.7% (12.5 to 18.7%)	2.6 (2.0 to 3.2)
Ischemic stroke	Libya	2010	All Ages	0.2 (0.1 to 0.2)	16.8% (13.5 to 20.3%)	2.9 (2.2 to 3.6)
Ischemic stroke	Libya	2015	All Ages	0.2 (0.2 to 0.3)	17.8% (14.4 to 21.2%)	3.8 (2.9 to 4.9)
Ischemic stroke	Libya	1990	Age-standardized		14.8% (11.2 to 18.7%)	6.5 (4.6 to 8.7)
Ischemic stroke	Libya	1995	Age-standardized		14.6% (10.8 to 18.4%)	6.5 (4.6 to 8.7)
Ischemic stroke	Libya	2000	Age-standardized		14.6% (10.9 to 18.5%)	6.9 (4.9 to 9.1)
Ischemic stroke	Libya	2005	Age-standardized		14.6% (11.0 to 18.4%)	6.8 (5.0 to 8.8)
Ischemic stroke	Libya	2010	Age-standardized		15.8% (12.0 to 19.8%)	7.0 (5.2 to 9.1)
Ischemic stroke	Libya	2015	Age-standardized		16.7% (12.8 to 20.8%)	8.3 (6.0 to 11.1)
Hemorrhagic stroke	Libya	1990	All Ages	0.1 (0.1 to 0.1)	11.0% (8.7 to 13.4%)	2.1 (1.6 to 2.6)
Hemorrhagic stroke	Libya	1995	All Ages	0.1 (0.1 to 0.1)	12.9% (10.6 to 15.2%)	2.3 (1.8 to 2.9)
Hemorrhagic stroke	Libya	2000	All Ages	0.1 (0.1 to 0.2)	14.5% (12.1 to 16.8%)	2.7 (2.1 to 3.3)
Hemorrhagic stroke	Libya	2005	All Ages	0.2 (0.1 to 0.2)	15.4% (12.9 to 17.7%)	2.8 (2.2 to 3.4)
Hemorrhagic stroke	Libya	2010	All Ages	0.2 (0.1 to 0.2)	17.3% (14.7 to 19.7%)	3.0 (2.4 to 3.7)
Hemorrhagic stroke	Libya	2015	All Ages	0.2 (0.2 to 0.3)	19.0% (16.3 to 21.7%)	3.8 (3.0 to 4.8)
Hemorrhagic stroke	Libya	1990	Age-standardized		14.5% (11.7 to 17.2%)	5.5 (4.1 to 7.2)
Hemorrhagic stroke	Libya	1995	Age-standardized		14.7% (11.8 to 17.5%)	5.6 (4.2 to 7.2)
Hemorrhagic stroke	Libya	2000	Age-standardized		15.1% (12.1 to 17.9%)	5.9 (4.6 to 7.6)
Hemorrhagic stroke	Libya	2005	Age-standardized		15.4% (12.5 to 18.3%)	5.7 (4.4 to 7.2)
Hemorrhagic stroke	Libya	2010	Age-standardized		16.8% (13.8 to 19.7%)	5.7 (4.4 to 7.3)
Hemorrhagic stroke	Libya	2015	Age-standardized		18.0% (14.8 to 21.1%)	6.5 (4.9 to 8.5)
Chronic obstructive pulmonary disease	Libya	1990	All Ages	0.1 (0.1 to 0.1)	28.0% (17.3 to 39.9%)	2.1 (1.2 to 3.2)
Chronic obstructive pulmonary disease	Libya	1995	All Ages	0.1 (0.1 to 0.1)	28.4% (17.7 to 40.3%)	2.0 (1.2 to 3.0)
Chronic obstructive pulmonary disease	Libya	2000	All Ages	0.1 (0.1 to 0.2)	28.8% (17.9 to 41.0%)	2.2 (1.3 to 3.2)
Chronic obstructive pulmonary disease	Libya	2005	All Ages	0.1 (0.1 to 0.2)	29.1% (18.2 to 41.5%)	2.3 (1.3 to 3.3)
Chronic obstructive pulmonary disease	Libya	2010	All Ages	0.2 (0.1 to 0.2)	32.2% (20.7 to 45.4%)	2.6 (1.6 to 3.8)
Chronic obstructive pulmonary disease	Libya	2015	All Ages	0.2 (0.1 to 0.3)	34.6% (22.9 to 49.1%)	3.3 (2.1 to 4.9)
Chronic obstructive pulmonary disease	Libya	1990	Age-standardized		30.0% (18.7 to 42.4%)	7.3 (4.3 to 11.0)
Chronic obstructive pulmonary disease	Libya	1995	Age-standardized		29.5% (18.4 to 42.0%)	6.4 (3.8 to 9.7)
Chronic obstructive pulmonary disease	Libya	2000	Age-standardized		29.6% (18.5 to 41.9%)	6.2 (3.7 to 9.2)
Chronic obstructive pulmonary disease	Libya	2005	Age-standardized		29.6% (18.6 to 42.3%)	5.9 (3.5 to 8.7)
Chronic obstructive pulmonary disease	Libya	2010	Age-standardized		32.7% (21.0 to 46.2%)	6.3 (3.9 to 9.4)

Cause name	Location	Year	Age group	Number (in thousands)	PAF	Rate (per 100,000)
Chronic obstructive pulmonary disease	Libya	2015	Age-standardized		34.9% (23.0 to 49.5%)	7.2 (4.5 to 10.6)
All causes	Morocco	1990	All Ages	6.6 (5.3 to 8.0)	4.3% (3.5 to 5.1%)	26.3 (21.1 to 32.1)
All causes	Morocco	1995	All Ages	6.8 (5.5 to 8.3)	4.5% (3.8 to 5.3%)	25.0 (20.2 to 30.7)
All causes	Morocco	2000	All Ages	6.9 (5.6 to 8.4)	4.7% (4.0 to 5.5%)	23.8 (19.3 to 29.1)
All causes	Morocco	2005	All Ages	7.5 (5.9 to 9.6)	4.8% (4.1 to 5.6%)	24.7 (19.3 to 31.4)
All causes	Morocco	2010	All Ages	8.1 (6.2 to 10.6)	4.7% (4.0 to 5.5%)	25.1 (19.1 to 32.9)
All causes	Morocco	2015	All Ages	9.8 (7.4 to 12.8)	5.2% (4.5 to 6.0%)	28.4 (21.5 to 37.3)
All causes	Morocco	1990	Age-standardized		5.3% (4.5 to 6.3%)	55.6 (45.6 to 67.3)
All causes	Morocco	1995	Age-standardized		5.3% (4.5 to 6.2%)	52.0 (41.8 to 63.8)
All causes	Morocco	2000	Age-standardized		5.3% (4.4 to 6.2%)	46.4 (37.0 to 56.6)
All causes	Morocco	2005	Age-standardized		5.2% (4.4 to 6.1%)	42.8 (33.5 to 54.0)
All causes	Morocco	2010	Age-standardized		5.1% (4.2 to 5.9%)	39.6 (29.9 to 52.1)
All causes	Morocco	2015	Age-standardized		5.5% (4.6 to 6.4%)	41.6 (31.3 to 54.3)
Lower respiratory infections	Morocco	1990	All Ages	2.0 (1.1 to 3.2)	18.1% (9.8 to 28.1%)	8.1 (4.4 to 12.9)
Lower respiratory infections	Morocco	1995	All Ages	1.7 (0.9 to 2.6)	17.9% (9.6 to 27.9%)	6.1 (3.2 to 9.7)
Lower respiratory infections	Morocco	2000	All Ages	1.4 (0.7 to 2.3)	17.7% (9.3 to 27.8%)	4.9 (2.5 to 7.9)
Lower respiratory infections	Morocco	2005	All Ages	1.4 (0.7 to 2.2)	17.5% (9.2 to 27.6%)	4.5 (2.3 to 7.2)
Lower respiratory infections	Morocco	2010	All Ages	1.4 (0.7 to 2.3)	16.5% (8.0 to 26.8%)	4.2 (2.0 to 7.0)
Lower respiratory infections	Morocco	2015	All Ages	1.6 (0.9 to 2.6)	18.6% (10.2 to 28.6%)	4.7 (2.5 to 7.5)
Lower respiratory infections	Morocco	1990	Age-standardized		18.1% (9.8 to 28.1%)	11.1 (6.0 to 17.5)
Lower respiratory infections	Morocco	1995	Age-standardized		17.9% (9.6 to 27.9%)	9.4 (4.9 to 15.3)
Lower respiratory infections	Morocco	2000	Age-standardized		17.7% (9.3 to 27.8%)	8.0 (4.2 to 13.1)
Lower respiratory infections	Morocco	2005	Age-standardized		17.5% (9.2 to 27.6%)	7.1 (3.7 to 11.6)
Lower respiratory infections	Morocco	2010	Age-standardized		16.5% (8.0 to 26.8%)	6.2 (3.1 to 10.5)
Lower respiratory infections	Morocco	2015	Age-standardized		18.6% (10.2 to 28.6%)	6.6 (3.5 to 10.8)
Tracheal, bronchus, and lung cancer	Morocco	1990	All Ages	0.3 (0.2 to 0.5)	12.6% (7.0 to 19.0%)	1.3 (0.7 to 2.1)
Tracheal, bronchus, and lung cancer	Morocco	1995	All Ages	0.4 (0.2 to 0.6)	12.4% (6.9 to 18.9%)	1.4 (0.7 to 2.1)
Tracheal, bronchus, and lung cancer	Morocco	2000	All Ages	0.4 (0.2 to 0.7)	12.4% (6.8 to 18.7%)	1.4 (0.8 to 2.2)
Tracheal, bronchus, and lung cancer	Morocco	2005	All Ages	0.5 (0.3 to 0.8)	12.2% (6.7 to 18.7%)	1.6 (0.8 to 2.5)
Tracheal, bronchus, and lung cancer	Morocco	2010	All Ages	0.6 (0.3 to 0.9)	11.4% (6.2 to 17.7%)	1.7 (0.9 to 2.9)
Tracheal, bronchus, and lung cancer	Morocco	2015	All Ages	0.8 (0.4 to 1.3)	13.0% (7.3 to 19.5%)	2.2 (1.2 to 3.8)
Tracheal, bronchus, and lung cancer	Morocco	1990	Age-standardized		12.7% (7.1 to 19.1%)	2.8 (1.5 to 4.4)
Tracheal, bronchus, and lung cancer	Morocco	1995	Age-standardized		12.5% (7.0 to 19.0%)	2.7 (1.4 to 4.1)
Tracheal, bronchus, and lung cancer	Morocco	2000	Age-standardized		12.4% (6.9 to 18.8%)	2.6 (1.4 to 4.0)
Tracheal, bronchus, and lung cancer	Morocco	2005	Age-standardized		12.3% (6.7 to 18.8%)	2.5 (1.3 to 4.0)
Tracheal, bronchus, and lung cancer	Morocco	2010	Age-standardized		11.5% (6.2 to 17.8%)	2.5 (1.3 to 4.2)

Cause name	Location	Year	Age group	Number (in thousands)	PAF	Rate (per 100,000)
Tracheal, bronchus, and lung cancer	Morocco	2015	Age-standardized		13.0% (7.3 to 19.6%)	3.0 (1.6 to 5.0)
Cardiovascular diseases	Morocco	1990	All Ages	4.0 (3.3 to 4.8)	11.0% (9.4 to 12.7%)	15.9 (13.1 to 19.3)
Cardiovascular diseases	Morocco	1995	All Ages	4.5 (3.6 to 5.5)	11.1% (9.5 to 12.8%)	16.6 (13.2 to 20.4)
Cardiovascular diseases	Morocco	2000	All Ages	4.8 (3.8 to 5.9)	11.1% (9.4 to 12.8%)	16.5 (13.2 to 20.5)
Cardiovascular diseases	Morocco	2005	All Ages	5.3 (4.1 to 6.9)	11.1% (9.4 to 12.7%)	17.5 (13.6 to 22.6)
Cardiovascular diseases	Morocco	2010	All Ages	5.8 (4.3 to 7.7)	10.8% (9.1 to 12.6%)	17.9 (13.3 to 23.9)
Cardiovascular diseases	Morocco	2015	All Ages	6.9 (5.1 to 9.3)	11.5% (9.7 to 13.5%)	20.1 (14.8 to 27.1)
Cardiovascular diseases	Morocco	1990	Age-standardized		10.9% (9.0 to 12.7%)	39.3 (31.5 to 47.7)
Cardiovascular diseases	Morocco	1995	Age-standardized		10.7% (8.9 to 12.6%)	37.5 (29.6 to 46.4)
Cardiovascular diseases	Morocco	2000	Age-standardized		10.7% (8.7 to 12.5%)	33.6 (26.4 to 42.0)
Cardiovascular diseases	Morocco	2005	Age-standardized		10.7% (8.7 to 12.5%)	31.1 (23.7 to 40.2)
Cardiovascular diseases	Morocco	2010	Age-standardized		10.5% (8.5 to 12.4%)	28.9 (21.2 to 38.2)
Cardiovascular diseases	Morocco	2015	Age-standardized		11.1% (9.0 to 13.1%)	29.8 (21.7 to 39.8)
Ischemic heart disease	Morocco	1990	All Ages	2.7 (2.2 to 3.4)	16.3% (13.4 to 19.4%)	10.8 (8.6 to 13.5)
Ischemic heart disease	Morocco	1995	All Ages	3.0 (2.4 to 3.8)	16.0% (13.2 to 19.2%)	11.2 (8.8 to 14.1)
Ischemic heart disease	Morocco	2000	All Ages	3.2 (2.5 to 4.1)	15.7% (12.8 to 18.8%)	11.1 (8.6 to 14.1)
Ischemic heart disease	Morocco	2005	All Ages	3.6 (2.7 to 4.7)	15.4% (12.5 to 18.4%)	11.7 (8.9 to 15.4)
Ischemic heart disease	Morocco	2010	All Ages	3.9 (2.8 to 5.3)	14.8% (11.9 to 17.8%)	12.1 (8.8 to 16.4)
Ischemic heart disease	Morocco	2015	All Ages	4.7 (3.3 to 6.4)	15.5% (12.3 to 18.6%)	13.6 (9.6 to 18.5)
Ischemic heart disease	Morocco	1990	Age-standardized		14.8% (11.6 to 18.0%)	26.6 (20.6 to 33.6)
Ischemic heart disease	Morocco	1995	Age-standardized		14.6% (11.3 to 17.9%)	25.3 (19.1 to 32.1)
Ischemic heart disease	Morocco	2000	Age-standardized		14.5% (11.2 to 17.8%)	22.6 (16.8 to 29.0)
Ischemic heart disease	Morocco	2005	Age-standardized		14.5% (11.2 to 17.8%)	20.9 (15.3 to 27.7)
Ischemic heart disease	Morocco	2010	Age-standardized		14.0% (10.9 to 17.3%)	19.5 (13.8 to 26.5)
Ischemic heart disease	Morocco	2015	Age-standardized		14.7% (11.2 to 18.1%)	20.2 (14.0 to 27.5)
Ischemic stroke	Morocco	1990	All Ages	0.7 (0.5 to 0.9)	11.7% (8.8 to 14.5%)	2.7 (1.9 to 3.7)
Ischemic stroke	Morocco	1995	All Ages	0.8 (0.6 to 1.0)	11.5% (8.5 to 14.4%)	2.9 (2.0 to 3.8)
Ischemic stroke	Morocco	2000	All Ages	0.8 (0.6 to 1.1)	11.3% (8.3 to 14.2%)	2.9 (2.1 to 3.9)
Ischemic stroke	Morocco	2005	All Ages	1.0 (0.7 to 1.3)	11.0% (8.0 to 14.0%)	3.1 (2.2 to 4.4)
Ischemic stroke	Morocco	2010	All Ages	1.0 (0.7 to 1.5)	10.5% (7.4 to 13.6%)	3.2 (2.1 to 4.6)
Ischemic stroke	Morocco	2015	All Ages	1.3 (0.8 to 1.8)	11.1% (7.9 to 14.4%)	3.6 (2.3 to 5.2)
Ischemic stroke	Morocco	1990	Age-standardized		10.7% (7.5 to 14.0%)	7.5 (5.1 to 10.4)
Ischemic stroke	Morocco	1995	Age-standardized		10.6% (7.3 to 14.0%)	7.2 (4.8 to 9.9)
Ischemic stroke	Morocco	2000	Age-standardized		10.5% (7.4 to 13.9%)	6.4 (4.3 to 9.0)
Ischemic stroke	Morocco	2005	Age-standardized		10.5% (7.2 to 13.8%)	6.0 (3.9 to 8.4)
Ischemic stroke	Morocco	2010	Age-standardized		10.1% (6.7 to 13.5%)	5.5 (3.5 to 8.1)

Cause name	Location	Year	Age group	Number (in thousands)	PAF	Rate (per 100,000)
Ischemic stroke	Morocco	2015	Age-standardized		10.7% (7.2 to 14.2%)	5.8 (3.6 to 8.4)
Hemorrhagic stroke	Morocco	1990	All Ages	0.6 (0.4 to 0.8)	11.6% (9.1 to 14.2%)	2.4 (1.8 to 3.1)
Hemorrhagic stroke	Morocco	1995	All Ages	0.7 (0.5 to 0.9)	11.9% (9.3 to 14.4%)	2.5 (1.8 to 3.3)
Hemorrhagic stroke	Morocco	2000	All Ages	0.7 (0.5 to 0.9)	12.0% (9.4 to 14.6%)	2.5 (1.8 to 3.2)
Hemorrhagic stroke	Morocco	2005	All Ages	0.8 (0.6 to 1.1)	12.1% (9.5 to 14.9%)	2.6 (1.9 to 3.5)
Hemorrhagic stroke	Morocco	2010	All Ages	0.8 (0.6 to 1.2)	11.7% (9.1 to 14.5%)	2.6 (1.8 to 3.6)
Hemorrhagic stroke	Morocco	2015	All Ages	1.0 (0.7 to 1.4)	12.6% (9.8 to 15.3%)	2.8 (1.9 to 4.0)
Hemorrhagic stroke	Morocco	1990	Age-standardized		11.7% (9.1 to 14.4%)	5.2 (3.8 to 6.8)
Hemorrhagic stroke	Morocco	1995	Age-standardized		11.6% (8.9 to 14.4%)	5.0 (3.6 to 6.6)
Hemorrhagic stroke	Morocco	2000	Age-standardized		11.6% (8.9 to 14.3%)	4.5 (3.2 to 6.0)
Hemorrhagic stroke	Morocco	2005	Age-standardized		11.6% (8.9 to 14.4%)	4.2 (3.0 to 5.7)
Hemorrhagic stroke	Morocco	2010	Age-standardized		11.2% (8.4 to 13.9%)	3.8 (2.6 to 5.3)
Hemorrhagic stroke	Morocco	2015	Age-standardized		11.9% (9.0 to 14.8%)	3.8 (2.6 to 5.5)
Chronic obstructive pulmonary disease	Morocco	1990	All Ages	0.2 (0.1 to 0.4)	17.5% (8.5 to 27.5%)	0.9 (0.4 to 1.6)
Chronic obstructive pulmonary disease	Morocco	1995	All Ages	0.3 (0.1 to 0.5)	17.7% (8.5 to 27.7%)	1.0 (0.5 to 1.7)
Chronic obstructive pulmonary disease	Morocco	2000	All Ages	0.3 (0.1 to 0.5)	17.9% (8.7 to 28.4%)	1.1 (0.5 to 1.8)
Chronic obstructive pulmonary disease	Morocco	2005	All Ages	0.4 (0.2 to 0.6)	18.0% (8.6 to 28.5%)	1.2 (0.6 to 1.9)
Chronic obstructive pulmonary disease	Morocco	2010	All Ages	0.4 (0.2 to 0.7)	17.3% (8.1 to 28.0%)	1.2 (0.6 to 2.1)
Chronic obstructive pulmonary disease	Morocco	2015	All Ages	0.5 (0.2 to 0.8)	19.1% (9.4 to 30.1%)	1.5 (0.7 to 2.5)
Chronic obstructive pulmonary disease	Morocco	1990	Age-standardized		18.7% (9.0 to 29.1%)	2.4 (1.1 to 4.2)
Chronic obstructive pulmonary disease	Morocco	1995	Age-standardized		18.5% (8.9 to 29.2%)	2.4 (1.1 to 4.1)
Chronic obstructive pulmonary disease	Morocco	2000	Age-standardized		18.5% (8.9 to 29.2%)	2.3 (1.1 to 3.8)
Chronic obstructive pulmonary disease	Morocco	2005	Age-standardized		18.4% (8.8 to 29.0%)	2.1 (1.1 to 3.5)
Chronic obstructive pulmonary disease	Morocco	2010	Age-standardized		17.6% (8.2 to 28.3%)	2.0 (0.9 to 3.4)
Chronic obstructive pulmonary disease	Morocco	2015	Age-standardized		19.3% (9.5 to 30.3%)	2.2 (1.0 to 3.7)
All causes	Palestine	1990	All Ages	0.4 (0.3 to 0.5)	4.6% (3.7 to 5.6%)	19.2 (14.4 to 24.9)
All causes	Palestine	1995	All Ages	0.4 (0.3 to 0.6)	5.0% (4.1 to 5.9%)	16.7 (13.1 to 21.0)
All causes	Palestine	2000	All Ages	0.5 (0.4 to 0.6)	4.9% (4.1 to 5.8%)	15.3 (12.5 to 18.3)
All causes	Palestine	2005	All Ages	0.6 (0.5 to 0.7)	5.3% (4.5 to 6.2%)	16.0 (13.3 to 19.0)
All causes	Palestine	2010	All Ages	0.8 (0.6 to 0.9)	6.0% (5.1 to 6.9%)	18.7 (15.1 to 22.8)
All causes	Palestine	2015	All Ages	1.0 (0.8 to 1.2)	6.6% (5.6 to 7.6%)	20.8 (16.0 to 26.0)
All causes	Palestine	1990	Age-standardized		7.2% (6.0 to 8.4%)	63.0 (48.5 to 81.8)
All causes	Palestine	1995	Age-standardized		7.3% (6.0 to 8.6%)	57.3 (44.7 to 72.6)
All causes	Palestine	2000	Age-standardized		7.0% (5.8 to 8.2%)	51.8 (42.4 to 61.7)
All causes	Palestine	2005	Age-standardized		7.0% (5.8 to 8.1%)	51.0 (42.2 to 60.5)
All causes	Palestine	2010	Age-standardized		7.3% (6.1 to 8.5%)	56.3 (45.0 to 68.9)

Cause name	Location	Year	Age group	Number (in thousands)	PAF	Rate (per 100,000)
All causes	Palestine	2015	Age-standardized		7.7% (6.5 to 9.0%)	58.7 (45.8 to 73.1)
Lower respiratory infections	Palestine	1990	All Ages	0.1 (0.0 to 0.1)	15.0% (6.4 to 25.6%)	4.2 (1.8 to 7.3)
Lower respiratory infections	Palestine	1995	All Ages	0.1 (0.0 to 0.1)	14.5% (6.1 to 25.2%)	2.9 (1.2 to 5.1)
Lower respiratory infections	Palestine	2000	All Ages	0.1 (0.0 to 0.1)	14.6% (6.1 to 25.2%)	2.4 (1.0 to 4.3)
Lower respiratory infections	Palestine	2005	All Ages	0.1 (0.0 to 0.1)	14.1% (5.9 to 24.9%)	2.2 (0.9 to 4.0)
Lower respiratory infections	Palestine	2010	All Ages	0.1 (0.0 to 0.2)	15.5% (7.0 to 25.9%)	2.3 (1.0 to 3.9)
Lower respiratory infections	Palestine	2015	All Ages	0.1 (0.1 to 0.2)	17.1% (8.5 to 27.4%)	2.4 (1.2 to 4.1)
Lower respiratory infections	Palestine	1990	Age-standardized		15.0% (6.4 to 25.6%)	7.2 (3.0 to 12.7)
Lower respiratory infections	Palestine	1995	Age-standardized		14.5% (6.1 to 25.2%)	6.2 (2.6 to 11.0)
Lower respiratory infections	Palestine	2000	Age-standardized		14.6% (6.1 to 25.2%)	5.7 (2.4 to 10.0)
Lower respiratory infections	Palestine	2005	Age-standardized		14.1% (5.9 to 24.9%)	5.3 (2.2 to 9.8)
Lower respiratory infections	Palestine	2010	Age-standardized		15.5% (7.0 to 25.9%)	5.7 (2.4 to 10.3)
Lower respiratory infections	Palestine	2015	Age-standardized		17.1% (8.5 to 27.4%)	5.8 (2.7 to 10.7)
Tracheal, bronchus, and lung cancer	Palestine	1990	All Ages	0.0 (0.0 to 0.0)	10.0% (5.2 to 15.6%)	0.5 (0.2 to 0.8)
Tracheal, bronchus, and lung cancer	Palestine	1995	All Ages	0.0 (0.0 to 0.0)	9.8% (5.1 to 15.5%)	0.5 (0.2 to 0.8)
Tracheal, bronchus, and lung cancer	Palestine	2000	All Ages	0.0 (0.0 to 0.0)	9.8% (5.1 to 15.6%)	0.5 (0.2 to 0.7)
Tracheal, bronchus, and lung cancer	Palestine	2005	All Ages	0.0 (0.0 to 0.0)	9.5% (4.9 to 15.2%)	0.5 (0.3 to 0.8)
Tracheal, bronchus, and lung cancer	Palestine	2010	All Ages	0.0 (0.0 to 0.0)	10.4% (5.5 to 16.3%)	0.7 (0.3 to 1.1)
Tracheal, bronchus, and lung cancer	Palestine	2015	All Ages	0.0 (0.0 to 0.1)	11.6% (6.3 to 17.8%)	0.8 (0.4 to 1.3)
Tracheal, bronchus, and lung cancer	Palestine	1990	Age-standardized		10.3% (5.3 to 16.1%)	1.6 (0.8 to 2.6)
Tracheal, bronchus, and lung cancer	Palestine	1995	Age-standardized		10.0% (5.2 to 15.9%)	1.5 (0.7 to 2.5)
Tracheal, bronchus, and lung cancer	Palestine	2000	Age-standardized		10.1% (5.2 to 15.9%)	1.5 (0.7 to 2.3)
Tracheal, bronchus, and lung cancer	Palestine	2005	Age-standardized		9.7% (5.0 to 15.6%)	1.5 (0.7 to 2.4)
Tracheal, bronchus, and lung cancer	Palestine	2010	Age-standardized		10.7% (5.7 to 16.6%)	1.8 (0.9 to 2.9)
Tracheal, bronchus, and lung cancer	Palestine	2015	Age-standardized		11.9% (6.5 to 18.2%)	2.1 (1.1 to 3.4)
Cardiovascular diseases	Palestine	1990	All Ages	0.3 (0.2 to 0.4)	12.5% (10.6 to 14.4%)	13.8 (10.4 to 18.3)
Cardiovascular diseases	Palestine	1995	All Ages	0.3 (0.3 to 0.4)	12.8% (10.9 to 14.7%)	12.7 (9.9 to 16.4)
Cardiovascular diseases	Palestine	2000	All Ages	0.4 (0.3 to 0.5)	13.0% (11.0 to 14.9%)	11.8 (9.7 to 14.2)
Cardiovascular diseases	Palestine	2005	All Ages	0.5 (0.4 to 0.5)	12.7% (10.9 to 14.7%)	12.7 (10.6 to 15.0)
Cardiovascular diseases	Palestine	2010	All Ages	0.6 (0.5 to 0.8)	13.0% (11.1 to 14.9%)	15.0 (12.0 to 18.3)
Cardiovascular diseases	Palestine	2015	All Ages	0.8 (0.6 to 1.0)	13.4% (11.5 to 15.3%)	16.7 (12.7 to 20.9)
Cardiovascular diseases	Palestine	1990	Age-standardized		11.5% (9.4 to 13.5%)	51.0 (38.3 to 66.3)
Cardiovascular diseases	Palestine	1995	Age-standardized		11.5% (9.4 to 13.6%)	46.6 (36.1 to 59.9)
Cardiovascular diseases	Palestine	2000	Age-standardized		11.6% (9.6 to 13.7%)	41.8 (34.1 to 50.7)
Cardiovascular diseases	Palestine	2005	Age-standardized		11.4% (9.4 to 13.4%)	41.5 (33.9 to 50.0)
Cardiovascular diseases	Palestine	2010	Age-standardized		11.6% (9.6 to 13.5%)	45.9 (36.4 to 56.3)

Cause name	Location	Year	Age group	Number (in thousands)	PAF	Rate (per 100,000)
Cardiovascular diseases	Palestine	2015	Age-standardized		12.1% (10.0 to 14.2%)	47.7 (36.7 to 59.3)
Ischemic heart disease	Palestine	1990	All Ages	0.2 (0.2 to 0.3)	16.3% (13.4 to 19.0%)	10.6 (7.8 to 14.4)
Ischemic heart disease	Palestine	1995	All Ages	0.3 (0.2 to 0.3)	16.2% (13.3 to 19.1%)	9.9 (7.5 to 13.0)
Ischemic heart disease	Palestine	2000	All Ages	0.3 (0.2 to 0.4)	16.2% (13.3 to 19.0%)	9.2 (7.4 to 11.3)
Ischemic heart disease	Palestine	2005	All Ages	0.4 (0.3 to 0.4)	16.0% (13.2 to 18.9%)	9.9 (8.1 to 12.0)
Ischemic heart disease	Palestine	2010	All Ages	0.5 (0.4 to 0.6)	16.5% (13.6 to 19.3%)	11.6 (9.0 to 14.5)
Ischemic heart disease	Palestine	2015	All Ages	0.6 (0.5 to 0.8)	17.1% (14.2 to 19.9%)	12.9 (9.6 to 16.4)
Ischemic heart disease	Palestine	1990	Age-standardized		14.4% (11.4 to 17.3%)	37.8 (27.4 to 50.3)
Ischemic heart disease	Palestine	1995	Age-standardized		14.2% (11.3 to 17.2%)	34.9 (25.7 to 45.5)
Ischemic heart disease	Palestine	2000	Age-standardized		14.2% (11.3 to 17.1%)	31.5 (24.4 to 38.6)
Ischemic heart disease	Palestine	2005	Age-standardized		14.2% (11.2 to 17.0%)	31.3 (24.5 to 38.6)
Ischemic heart disease	Palestine	2010	Age-standardized		14.7% (11.7 to 17.5%)	34.2 (26.2 to 42.8)
Ischemic heart disease	Palestine	2015	Age-standardized		15.3% (12.2 to 18.3%)	35.6 (26.7 to 45.2)
Ischemic stroke	Palestine	1990	All Ages	0.0 (0.0 to 0.0)	10.6% (7.7 to 13.4%)	0.7 (0.5 to 1.0)
Ischemic stroke	Palestine	1995	All Ages	0.0 (0.0 to 0.0)	10.4% (7.7 to 13.2%)	0.6 (0.4 to 0.9)
Ischemic stroke	Palestine	2000	All Ages	0.0 (0.0 to 0.0)	10.4% (7.5 to 13.3%)	0.6 (0.4 to 0.8)
Ischemic stroke	Palestine	2005	All Ages	0.0 (0.0 to 0.0)	10.2% (7.4 to 13.0%)	0.6 (0.5 to 0.9)
Ischemic stroke	Palestine	2010	All Ages	0.1 (0.0 to 0.1)	10.7% (7.8 to 13.6%)	1.2 (0.8 to 1.5)
Ischemic stroke	Palestine	2015	All Ages	0.1 (0.0 to 0.1)	11.2% (8.2 to 14.1%)	1.4 (0.9 to 1.9)
Ischemic stroke	Palestine	1990	Age-standardized		9.6% (6.3 to 13.1%)	3.3 (2.0 to 5.0)
Ischemic stroke	Palestine	1995	Age-standardized		9.5% (6.2 to 12.8%)	2.9 (1.8 to 4.4)
Ischemic stroke	Palestine	2000	Age-standardized		9.5% (6.2 to 13.0%)	2.6 (1.7 to 3.9)
Ischemic stroke	Palestine	2005	Age-standardized		9.3% (6.1 to 12.9%)	2.7 (1.7 to 4.0)
Ischemic stroke	Palestine	2010	Age-standardized		9.8% (6.5 to 13.3%)	4.5 (2.8 to 6.6)
Ischemic stroke	Palestine	2015	Age-standardized		10.3% (7.0 to 14.0%)	5.1 (3.2 to 7.3)
Hemorrhagic stroke	Palestine	1990	All Ages	0.1 (0.0 to 0.1)	10.4% (8.0 to 13.0%)	2.5 (1.7 to 3.5)
Hemorrhagic stroke	Palestine	1995	All Ages	0.1 (0.0 to 0.1)	10.5% (8.0 to 13.2%)	2.2 (1.6 to 3.0)
Hemorrhagic stroke	Palestine	2000	All Ages	0.1 (0.1 to 0.1)	10.6% (8.1 to 13.3%)	2.0 (1.5 to 2.6)
Hemorrhagic stroke	Palestine	2005	All Ages	0.1 (0.1 to 0.1)	10.6% (8.1 to 13.3%)	2.1 (1.6 to 2.7)
Hemorrhagic stroke	Palestine	2010	All Ages	0.1 (0.1 to 0.1)	11.3% (8.7 to 14.2%)	2.2 (1.6 to 2.9)
Hemorrhagic stroke	Palestine	2015	All Ages	0.1 (0.1 to 0.1)	12.0% (9.3 to 14.7%)	2.4 (1.7 to 3.1)
Hemorrhagic stroke	Palestine	1990	Age-standardized		10.1% (7.2 to 13.2%)	9.8 (6.4 to 14.1)
Hemorrhagic stroke	Palestine	1995	Age-standardized		10.0% (7.1 to 13.0%)	8.8 (5.9 to 12.6)
Hemorrhagic stroke	Palestine	2000	Age-standardized		10.0% (7.0 to 13.0%)	7.7 (5.3 to 10.4)
Hemorrhagic stroke	Palestine	2005	Age-standardized		9.9% (7.0 to 12.8%)	7.5 (5.2 to 9.9)
Hemorrhagic stroke	Palestine	2010	Age-standardized		10.5% (7.5 to 13.5%)	7.1 (4.9 to 9.6)

Cause name	Location	Year	Age group	Number (in thousands)	PAF	Rate (per 100,000)
Hemorrhagic stroke	Palestine	2015	Age-standardized		11.1% (8.1 to 14.1%)	7.0 (4.7 to 9.5)
Chronic obstructive pulmonary disease	Palestine	1990	All Ages	0.0 (0.0 to 0.0)	15.6% (7.1 to 25.5%)	0.7 (0.3 to 1.3)
Chronic obstructive pulmonary disease	Palestine	1995	All Ages	0.0 (0.0 to 0.0)	15.5% (7.0 to 25.8%)	0.7 (0.3 to 1.1)
Chronic obstructive pulmonary disease	Palestine	2000	All Ages	0.0 (0.0 to 0.0)	15.6% (7.2 to 25.8%)	0.6 (0.3 to 1.1)
Chronic obstructive pulmonary disease	Palestine	2005	All Ages	0.0 (0.0 to 0.0)	15.3% (6.7 to 25.5%)	0.7 (0.3 to 1.1)
Chronic obstructive pulmonary disease	Palestine	2010	All Ages	0.0 (0.0 to 0.1)	16.5% (7.7 to 26.9%)	0.8 (0.4 to 1.4)
Chronic obstructive pulmonary disease	Palestine	2015	All Ages	0.0 (0.0 to 0.1)	17.9% (8.5 to 28.7%)	0.9 (0.4 to 1.5)
Chronic obstructive pulmonary disease	Palestine	1990	Age-standardized		16.5% (7.5 to 27.0%)	3.3 (1.4 to 5.7)
Chronic obstructive pulmonary disease	Palestine	1995	Age-standardized		16.2% (7.3 to 26.9%)	3.0 (1.3 to 5.1)
Chronic obstructive pulmonary disease	Palestine	2000	Age-standardized		16.2% (7.5 to 26.9%)	2.7 (1.2 to 4.6)
Chronic obstructive pulmonary disease	Palestine	2005	Age-standardized		15.8% (6.9 to 26.4%)	2.6 (1.1 to 4.3)
Chronic obstructive pulmonary disease	Palestine	2010	Age-standardized		16.9% (7.9 to 27.7%)	2.9 (1.3 to 4.9)
Chronic obstructive pulmonary disease	Palestine	2015	Age-standardized		18.3% (8.7 to 29.4%)	3.1 (1.4 to 5.1)
All causes	Oman	1990	All Ages	0.5 (0.4 to 0.6)	6.8% (5.7 to 8.1%)	27.7 (21.3 to 35.0)
All causes	Oman	1995	All Ages	0.5 (0.4 to 0.6)	7.2% (6.3 to 8.2%)	22.5 (17.7 to 28.0)
All causes	Oman	2000	All Ages	0.5 (0.4 to 0.6)	8.0% (7.0 to 8.9%)	21.9 (18.1 to 26.1)
All causes	Oman	2005	All Ages	0.5 (0.5 to 0.6)	7.9% (7.0 to 8.8%)	20.9 (17.9 to 24.0)
All causes	Oman	2010	All Ages	0.7 (0.6 to 0.8)	7.9% (7.0 to 8.8%)	22.6 (19.5 to 25.9)
All causes	Oman	2015	All Ages	0.9 (0.7 to 1.1)	7.8% (6.9 to 8.7%)	20.0 (16.4 to 23.8)
All causes	Oman	1990	Age-standardized		9.7% (8.5 to 11.2%)	81.6 (62.5 to 102.8)
All causes	Oman	1995	Age-standardized		9.7% (8.4 to 11.1%)	75.7 (59.8 to 94.5)
All causes	Oman	2000	Age-standardized		9.8% (8.5 to 11.2%)	73.0 (59.9 to 87.0)
All causes	Oman	2005	Age-standardized		9.3% (8.0 to 10.5%)	67.2 (56.9 to 77.4)
All causes	Oman	2010	Age-standardized		9.4% (8.1 to 10.6%)	68.6 (58.8 to 79.8)
All causes	Oman	2015	Age-standardized		9.1% (7.9 to 10.5%)	62.8 (50.6 to 74.8)
Lower respiratory infections	Oman	1990	All Ages	0.2 (0.1 to 0.3)	35.1% (27.4 to 42.5%)	11.3 (7.4 to 16.7)
Lower respiratory infections	Oman	1995	All Ages	0.1 (0.1 to 0.2)	34.9% (27.0 to 42.2%)	6.8 (4.8 to 9.4)
Lower respiratory infections	Oman	2000	All Ages	0.1 (0.1 to 0.2)	35.1% (27.1 to 42.6%)	5.9 (4.2 to 7.7)
Lower respiratory infections	Oman	2005	All Ages	0.1 (0.1 to 0.2)	32.8% (25.2 to 40.0%)	5.2 (3.7 to 6.7)
Lower respiratory infections	Oman	2010	All Ages	0.2 (0.1 to 0.2)	32.1% (24.6 to 39.4%)	5.6 (3.9 to 7.3)
Lower respiratory infections	Oman	2015	All Ages	0.2 (0.1 to 0.3)	31.5% (23.9 to 38.6%)	4.7 (3.2 to 6.3)
Lower respiratory infections	Oman	1990	Age-standardized		35.1% (27.4 to 42.5%)	24.2 (15.5 to 36.0)
Lower respiratory infections	Oman	1995	Age-standardized		34.9% (27.0 to 42.2%)	20.6 (13.7 to 29.9)
Lower respiratory infections	Oman	2000	Age-standardized		35.1% (27.1 to 42.6%)	19.3 (13.3 to 26.6)
Lower respiratory infections	Oman	2005	Age-standardized		32.8% (25.2 to 40.0%)	17.3 (11.9 to 23.0)
Lower respiratory infections	Oman	2010	Age-standardized		32.1% (24.6 to 39.4%)	17.8 (12.0 to 23.8)

Cause name	Location	Year	Age group	Number (in thousands)	PAF	Rate (per 100,000)
Lower respiratory infections	Oman	2015	Age-standardized		31.5% (23.9 to 38.6%)	16.5 (10.8 to 23.0)
Tracheal, bronchus, and lung cancer	Oman	1990	All Ages	0.0 (0.0 to 0.0)	26.7% (18.1 to 35.0%)	0.5 (0.3 to 0.8)
Tracheal, bronchus, and lung cancer	Oman	1995	All Ages	0.0 (0.0 to 0.0)	26.6% (18.0 to 34.8%)	0.6 (0.4 to 1.0)
Tracheal, bronchus, and lung cancer	Oman	2000	All Ages	0.0 (0.0 to 0.0)	26.7% (18.4 to 35.2%)	0.7 (0.4 to 0.9)
Tracheal, bronchus, and lung cancer	Oman	2005	All Ages	0.0 (0.0 to 0.0)	24.5% (15.9 to 33.1%)	0.6 (0.4 to 0.9)
Tracheal, bronchus, and lung cancer	Oman	2010	All Ages	0.0 (0.0 to 0.0)	23.9% (15.8 to 32.4%)	0.7 (0.5 to 1.0)
Tracheal, bronchus, and lung cancer	Oman	2015	All Ages	0.0 (0.0 to 0.0)	23.4% (15.4 to 31.4%)	0.7 (0.4 to 1.0)
Tracheal, bronchus, and lung cancer	Oman	1990	Age-standardized		26.8% (18.2 to 35.1%)	1.6 (0.9 to 2.4)
Tracheal, bronchus, and lung cancer	Oman	1995	Age-standardized		26.6% (18.1 to 34.9%)	1.9 (1.2 to 2.7)
Tracheal, bronchus, and lung cancer	Oman	2000	Age-standardized		26.9% (18.5 to 35.3%)	1.9 (1.3 to 2.7)
Tracheal, bronchus, and lung cancer	Oman	2005	Age-standardized		24.6% (16.0 to 33.2%)	1.8 (1.2 to 2.5)
Tracheal, bronchus, and lung cancer	Oman	2010	Age-standardized		24.0% (15.8 to 32.5%)	1.9 (1.3 to 2.7)
Tracheal, bronchus, and lung cancer	Oman	2015	Age-standardized		23.5% (15.4 to 31.4%)	1.8 (1.2 to 2.6)
Cardiovascular diseases	Oman	1990	All Ages	0.3 (0.2 to 0.4)	12.8% (10.9 to 15.2%)	14.9 (10.7 to 19.5)
Cardiovascular diseases	Oman	1995	All Ages	0.3 (0.2 to 0.4)	14.1% (12.3 to 16.2%)	14.1 (10.7 to 18.1)
Cardiovascular diseases	Oman	2000	All Ages	0.3 (0.3 to 0.4)	14.4% (12.4 to 16.5%)	14.3 (11.7 to 17.5)
Cardiovascular diseases	Oman	2005	All Ages	0.4 (0.3 to 0.4)	13.9% (12.1 to 15.9%)	14.0 (11.9 to 16.4)
Cardiovascular diseases	Oman	2010	All Ages	0.4 (0.4 to 0.5)	15.0% (13.1 to 17.1%)	15.0 (12.8 to 17.5)
Cardiovascular diseases	Oman	2015	All Ages	0.6 (0.5 to 0.7)	15.2% (13.2 to 17.5%)	13.5 (10.8 to 16.4)
Cardiovascular diseases	Oman	1990	Age-standardized		13.6% (11.4 to 16.2%)	51.5 (38.1 to 66.9)
Cardiovascular diseases	Oman	1995	Age-standardized		13.6% (11.4 to 16.3%)	49.2 (38.3 to 63.6)
Cardiovascular diseases	Oman	2000	Age-standardized		13.6% (11.5 to 16.4%)	47.7 (38.5 to 59.0)
Cardiovascular diseases	Oman	2005	Age-standardized		13.1% (11.0 to 15.5%)	44.2 (36.5 to 53.0)
Cardiovascular diseases	Oman	2010	Age-standardized		13.8% (11.7 to 16.3%)	44.0 (36.7 to 52.7)
Cardiovascular diseases	Oman	2015	Age-standardized		13.4% (11.1 to 15.9%)	40.1 (31.4 to 49.1)
Ischemic heart disease	Oman	1990	All Ages	0.2 (0.2 to 0.3)	23.1% (19.8 to 27.0%)	12.1 (8.7 to 16.2)
Ischemic heart disease	Oman	1995	All Ages	0.3 (0.2 to 0.3)	23.3% (20.0 to 27.0%)	11.6 (8.8 to 14.9)
Ischemic heart disease	Oman	2000	All Ages	0.3 (0.2 to 0.3)	22.5% (19.2 to 26.2%)	11.6 (9.3 to 14.4)
Ischemic heart disease	Oman	2005	All Ages	0.3 (0.2 to 0.3)	21.7% (18.4 to 25.6%)	11.4 (9.4 to 13.6)
Ischemic heart disease	Oman	2010	All Ages	0.3 (0.3 to 0.4)	21.4% (18.2 to 25.2%)	11.6 (9.6 to 13.9)
Ischemic heart disease	Oman	2015	All Ages	0.5 (0.4 to 0.6)	21.8% (18.5 to 25.6%)	10.7 (8.4 to 13.2)
Ischemic heart disease	Oman	1990	Age-standardized		20.4% (16.8 to 25.0%)	41.9 (30.4 to 55.1)
Ischemic heart disease	Oman	1995	Age-standardized		20.4% (16.8 to 24.7%)	40.0 (30.3 to 52.2)
Ischemic heart disease	Oman	2000	Age-standardized		20.3% (16.6 to 24.6%)	38.5 (30.1 to 48.6)
Ischemic heart disease	Oman	2005	Age-standardized		19.5% (15.9 to 23.7%)	35.6 (28.2 to 44.1)
Ischemic heart disease	Oman	2010	Age-standardized		19.2% (15.7 to 23.5%)	33.7 (27.0 to 41.7)

Cause name	Location	Year	Age group	Number (in thousands)	PAF	Rate (per 100,000)
Ischemic heart disease	Oman	2015	Age-standardized		18.8% (15.1 to 23.3%)	31.0 (23.6 to 39.1)
Ischemic stroke	Oman	1990	All Ages	0.0 (0.0 to 0.0)	16.3% (12.8 to 20.0%)	0.4 (0.2 to 0.7)
Ischemic stroke	Oman	1995	All Ages	0.0 (0.0 to 0.0)	16.2% (12.5 to 20.1%)	0.4 (0.2 to 0.6)
Ischemic stroke	Oman	2000	All Ages	0.0 (0.0 to 0.0)	15.9% (12.1 to 19.9%)	0.5 (0.3 to 0.7)
Ischemic stroke	Oman	2005	All Ages	0.0 (0.0 to 0.0)	15.1% (11.2 to 19.0%)	0.5 (0.4 to 0.7)
Ischemic stroke	Oman	2010	All Ages	0.0 (0.0 to 0.0)	15.2% (11.5 to 18.8%)	1.0 (0.8 to 1.4)
Ischemic stroke	Oman	2015	All Ages	0.0 (0.0 to 0.1)	15.1% (11.6 to 18.8%)	0.8 (0.6 to 1.0)
Ischemic stroke	Oman	1990	Age-standardized		15.1% (10.8 to 19.7%)	2.0 (1.2 to 3.3)
Ischemic stroke	Oman	1995	Age-standardized		15.0% (10.6 to 19.7%)	1.9 (1.2 to 3.0)
Ischemic stroke	Oman	2000	Age-standardized		15.0% (10.7 to 19.7%)	2.1 (1.4 to 3.1)
Ischemic stroke	Oman	2005	Age-standardized		14.3% (10.0 to 18.8%)	2.0 (1.4 to 2.9)
Ischemic stroke	Oman	2010	Age-standardized		14.3% (10.2 to 18.7%)	4.0 (2.8 to 5.4)
Ischemic stroke	Oman	2015	Age-standardized		13.8% (9.8 to 18.5%)	3.4 (2.3 to 4.7)
Hemorrhagic stroke	Oman	1990	All Ages	0.0 (0.0 to 0.1)	16.5% (13.5 to 19.5%)	2.3 (1.6 to 3.4)
Hemorrhagic stroke	Oman	1995	All Ages	0.1 (0.0 to 0.1)	18.1% (15.2 to 20.9%)	2.2 (1.6 to 2.9)
Hemorrhagic stroke	Oman	2000	All Ages	0.1 (0.0 to 0.1)	18.3% (15.4 to 21.2%)	2.2 (1.7 to 2.9)
Hemorrhagic stroke	Oman	2005	All Ages	0.1 (0.0 to 0.1)	17.6% (14.7 to 20.3%)	2.2 (1.8 to 2.7)
Hemorrhagic stroke	Oman	2010	All Ages	0.1 (0.1 to 0.1)	17.5% (14.7 to 20.4%)	2.3 (1.9 to 2.9)
Hemorrhagic stroke	Oman	2015	All Ages	0.1 (0.1 to 0.1)	17.9% (15.0 to 20.7%)	2.1 (1.6 to 2.6)
Hemorrhagic stroke	Oman	1990	Age-standardized		17.0% (13.9 to 20.1%)	7.7 (5.2 to 11.4)
Hemorrhagic stroke	Oman	1995	Age-standardized		17.1% (13.8 to 20.3%)	7.3 (5.2 to 10.1)
Hemorrhagic stroke	Oman	2000	Age-standardized		17.2% (14.0 to 20.4%)	7.1 (5.4 to 9.3)
Hemorrhagic stroke	Oman	2005	Age-standardized		16.3% (13.1 to 19.5%)	6.6 (5.2 to 8.3)
Hemorrhagic stroke	Oman	2010	Age-standardized		16.1% (12.8 to 19.3%)	6.3 (4.9 to 8.0)
Hemorrhagic stroke	Oman	2015	Age-standardized		15.6% (12.3 to 18.7%)	5.8 (4.2 to 7.5)
Chronic obstructive pulmonary disease	Oman	1990	All Ages	0.0 (0.0 to 0.0)	28.9% (18.7 to 41.3%)	1.0 (0.6 to 1.5)
Chronic obstructive pulmonary disease	Oman	1995	All Ages	0.0 (0.0 to 0.0)	30.4% (19.7 to 43.2%)	0.9 (0.5 to 1.4)
Chronic obstructive pulmonary disease	Oman	2000	All Ages	0.0 (0.0 to 0.0)	31.3% (20.0 to 44.5%)	1.0 (0.6 to 1.6)
Chronic obstructive pulmonary disease	Oman	2005	All Ages	0.0 (0.0 to 0.0)	29.7% (19.0 to 42.2%)	1.0 (0.6 to 1.5)
Chronic obstructive pulmonary disease	Oman	2010	All Ages	0.0 (0.0 to 0.1)	29.2% (18.3 to 41.6%)	1.3 (0.8 to 2.0)
Chronic obstructive pulmonary disease	Oman	2015	All Ages	0.1 (0.0 to 0.1)	29.0% (18.1 to 41.5%)	1.1 (0.6 to 1.6)
Chronic obstructive pulmonary disease	Oman	1990	Age-standardized		32.0% (20.5 to 45.9%)	4.3 (2.5 to 6.7)
Chronic obstructive pulmonary disease	Oman	1995	Age-standardized		32.1% (20.8 to 45.3%)	3.9 (2.4 to 6.2)
Chronic obstructive pulmonary disease	Oman	2000	Age-standardized		32.4% (20.7 to 45.9%)	4.1 (2.5 to 6.1)
Chronic obstructive pulmonary disease	Oman	2005	Age-standardized		30.5% (19.4 to 43.3%)	3.9 (2.3 to 5.7)
Chronic obstructive pulmonary disease	Oman	2010	Age-standardized		29.9% (18.7 to 42.6%)	4.8 (2.8 to 7.1)

Cause name	Location	Year	Age group	Number (in thousands)	PAF	Rate (per 100,000)
Chronic obstructive pulmonary disease	Oman	2015	Age-standardized		29.5% (18.4 to 42.2%)	4.3 (2.5 to 6.4)
All causes	Qatar	1990	All Ages	0.1 (0.1 to 0.1)	8.1% (7.1 to 9.3%)	21.6 (18.4 to 25.2)
All causes	Qatar	1995	All Ages	0.1 (0.1 to 0.1)	8.8% (7.8 to 10.1%)	24.5 (20.6 to 28.8)
All causes	Qatar	2000	All Ages	0.2 (0.1 to 0.2)	9.3% (8.2 to 10.5%)	26.2 (22.5 to 30.6)
All causes	Qatar	2005	All Ages	0.2 (0.1 to 0.2)	8.6% (7.6 to 9.8%)	20.4 (17.4 to 24.3)
All causes	Qatar	2010	All Ages	0.2 (0.2 to 0.3)	7.7% (6.9 to 8.6%)	12.0 (10.0 to 14.2)
All causes	Qatar	2015	All Ages	0.3 (0.2 to 0.3)	7.5% (6.7 to 8.4%)	11.1 (8.5 to 14.3)
All causes	Qatar	1990	Age-standardized		11.1% (9.6 to 13.0%)	88.7 (74.5 to 105.2)
All causes	Qatar	1995	Age-standardized		11.3% (9.7 to 13.3%)	93.7 (78.6 to 111.7)
All causes	Qatar	2000	Age-standardized		11.0% (9.5 to 12.9%)	89.2 (75.3 to 105.4)
All causes	Qatar	2005	Age-standardized		10.2% (8.9 to 12.0%)	75.8 (64.1 to 91.2)
All causes	Qatar	2010	Age-standardized		9.9% (8.6 to 11.4%)	55.9 (46.8 to 65.9)
All causes	Qatar	2015	Age-standardized		9.3% (8.1 to 10.9%)	50.8 (39.7 to 64.5)
Lower respiratory infections	Qatar	1990	All Ages	0.0 (0.0 to 0.0)	44.1% (34.4 to 53.8%)	2.3 (1.7 to 3.0)
Lower respiratory infections	Qatar	1995	All Ages	0.0 (0.0 to 0.0)	43.8% (34.3 to 52.9%)	2.2 (1.6 to 2.9)
Lower respiratory infections	Qatar	2000	All Ages	0.0 (0.0 to 0.0)	43.9% (34.2 to 53.7%)	2.2 (1.6 to 2.9)
Lower respiratory infections	Qatar	2005	All Ages	0.0 (0.0 to 0.0)	43.3% (33.7 to 52.6%)	1.8 (1.3 to 2.3)
Lower respiratory infections	Qatar	2010	All Ages	0.0 (0.0 to 0.0)	46.0% (36.3 to 55.3%)	1.3 (0.9 to 1.7)
Lower respiratory infections	Qatar	2015	All Ages	0.0 (0.0 to 0.0)	44.0% (34.9 to 53.1%)	1.2 (0.8 to 1.6)
Lower respiratory infections	Qatar	1990	Age-standardized		44.1% (34.4 to 53.8%)	8.6 (6.2 to 11.5)
Lower respiratory infections	Qatar	1995	Age-standardized		43.8% (34.3 to 52.9%)	8.4 (6.1 to 11.1)
Lower respiratory infections	Qatar	2000	Age-standardized		43.9% (34.2 to 53.7%)	8.5 (5.9 to 11.2)
Lower respiratory infections	Qatar	2005	Age-standardized		43.3% (33.7 to 52.6%)	7.7 (5.5 to 10.5)
Lower respiratory infections	Qatar	2010	Age-standardized		46.0% (36.3 to 55.3%)	6.9 (4.7 to 9.4)
Lower respiratory infections	Qatar	2015	Age-standardized		44.0% (34.9 to 53.1%)	6.4 (4.2 to 8.8)
Tracheal, bronchus, and lung cancer	Qatar	1990	All Ages	0.0 (0.0 to 0.0)	35.6% (26.2 to 44.4%)	1.0 (0.7 to 1.3)
Tracheal, bronchus, and lung cancer	Qatar	1995	All Ages	0.0 (0.0 to 0.0)	35.2% (26.3 to 43.7%)	1.2 (0.8 to 1.5)
Tracheal, bronchus, and lung cancer	Qatar	2000	All Ages	0.0 (0.0 to 0.0)	35.3% (25.4 to 44.6%)	1.5 (1.0 to 2.0)
Tracheal, bronchus, and lung cancer	Qatar	2005	All Ages	0.0 (0.0 to 0.0)	34.8% (24.9 to 43.8%)	1.5 (1.0 to 2.0)
Tracheal, bronchus, and lung cancer	Qatar	2010	All Ages	0.0 (0.0 to 0.0)	37.9% (28.4 to 46.5%)	1.2 (0.8 to 1.5)
Tracheal, bronchus, and lung cancer	Qatar	2015	All Ages	0.0 (0.0 to 0.0)	35.5% (26.4 to 44.6%)	1.1 (0.8 to 1.7)
Tracheal, bronchus, and lung cancer	Qatar	1990	Age-standardized		35.7% (26.3 to 44.5%)	4.3 (3.1 to 5.7)
Tracheal, bronchus, and lung cancer	Qatar	1995	Age-standardized		35.3% (26.3 to 43.8%)	4.6 (3.3 to 6.1)
Tracheal, bronchus, and lung cancer	Qatar	2000	Age-standardized		35.4% (25.4 to 44.7%)	5.1 (3.5 to 6.8)
Tracheal, bronchus, and lung cancer	Qatar	2005	Age-standardized		34.8% (24.9 to 43.8%)	5.2 (3.6 to 7.1)
Tracheal, bronchus, and lung cancer	Qatar	2010	Age-standardized		38.0% (28.5 to 46.7%)	5.1 (3.7 to 6.7)

Cause name	Location	Year	Age group	Number (in thousands)	PAF	Rate (per 100,000)
Tracheal, bronchus, and lung cancer	Qatar	2015	Age-standardized		35.6% (26.5 to 44.7%)	4.9 (3.3 to 7.1)
Cardiovascular diseases	Qatar	1990	All Ages	0.1 (0.1 to 0.1)	24.4% (21.7 to 27.3%)	17.3 (14.5 to 20.5)
Cardiovascular diseases	Qatar	1995	All Ages	0.1 (0.1 to 0.1)	24.5% (21.7 to 27.4%)	20.1 (16.7 to 23.7)
Cardiovascular diseases	Qatar	2000	All Ages	0.1 (0.1 to 0.1)	25.0% (22.1 to 28.2%)	21.3 (18.0 to 25.2)
Cardiovascular diseases	Qatar	2005	All Ages	0.1 (0.1 to 0.2)	25.3% (22.2 to 28.6%)	16.3 (13.4 to 19.6)
Cardiovascular diseases	Qatar	2010	All Ages	0.2 (0.1 to 0.2)	26.7% (23.9 to 29.9%)	9.0 (7.4 to 10.9)
Cardiovascular diseases	Qatar	2015	All Ages	0.2 (0.1 to 0.2)	25.1% (22.4 to 27.9%)	8.2 (6.2 to 10.7)
Cardiovascular diseases	Qatar	1990	Age-standardized		20.5% (17.5 to 24.7%)	70.3 (57.9 to 86.2)
Cardiovascular diseases	Qatar	1995	Age-standardized		20.5% (17.5 to 24.7%)	75.0 (61.3 to 91.9)
Cardiovascular diseases	Qatar	2000	Age-standardized		20.5% (17.6 to 24.6%)	69.9 (57.9 to 85.1)
Cardiovascular diseases	Qatar	2005	Age-standardized		20.1% (17.1 to 24.3%)	57.7 (47.1 to 71.6)
Cardiovascular diseases	Qatar	2010	Age-standardized		20.5% (17.5 to 24.7%)	39.8 (32.5 to 48.6)
Cardiovascular diseases	Qatar	2015	Age-standardized		19.8% (16.6 to 23.9%)	35.7 (27.5 to 46.5)
Ischemic heart disease	Qatar	1990	All Ages	0.1 (0.1 to 0.1)	28.2% (24.3 to 32.4%)	13.5 (11.1 to 16.1)
Ischemic heart disease	Qatar	1995	All Ages	0.1 (0.1 to 0.1)	28.1% (24.4 to 32.2%)	15.6 (12.7 to 18.8)
Ischemic heart disease	Qatar	2000	All Ages	0.1 (0.1 to 0.1)	28.5% (24.7 to 32.7%)	16.8 (13.8 to 20.1)
Ischemic heart disease	Qatar	2005	All Ages	0.1 (0.1 to 0.1)	28.8% (24.6 to 33.1%)	13.1 (10.6 to 16.1)
Ischemic heart disease	Qatar	2010	All Ages	0.1 (0.1 to 0.1)	30.6% (26.5 to 34.9%)	6.9 (5.6 to 8.3)
Ischemic heart disease	Qatar	2015	All Ages	0.1 (0.1 to 0.2)	28.7% (24.8 to 32.8%)	6.3 (4.6 to 8.3)
Ischemic heart disease	Qatar	1990	Age-standardized		22.9% (18.8 to 28.6%)	56.7 (45.6 to 71.6)
Ischemic heart disease	Qatar	1995	Age-standardized		22.9% (18.8 to 28.5%)	60.4 (48.3 to 76.9)
Ischemic heart disease	Qatar	2000	Age-standardized		23.0% (19.1 to 28.6%)	56.1 (45.4 to 69.3)
Ischemic heart disease	Qatar	2005	Age-standardized		22.5% (18.4 to 28.3%)	46.2 (36.8 to 59.6)
Ischemic heart disease	Qatar	2010	Age-standardized		23.2% (19.2 to 29.3%)	30.4 (24.1 to 38.5)
Ischemic heart disease	Qatar	2015	Age-standardized		22.5% (18.3 to 28.5%)	27.5 (20.4 to 36.7)
Ischemic stroke	Qatar	1990	All Ages	0.0 (0.0 to 0.0)	21.8% (17.9 to 25.9%)	1.3 (1.0 to 1.7)
Ischemic stroke	Qatar	1995	All Ages	0.0 (0.0 to 0.0)	21.8% (18.1 to 25.9%)	1.6 (1.2 to 2.0)
Ischemic stroke	Qatar	2000	All Ages	0.0 (0.0 to 0.0)	21.9% (18.2 to 25.9%)	1.6 (1.3 to 2.0)
Ischemic stroke	Qatar	2005	All Ages	0.0 (0.0 to 0.0)	21.9% (18.3 to 25.9%)	1.1 (0.9 to 1.4)
Ischemic stroke	Qatar	2010	All Ages	0.0 (0.0 to 0.0)	23.7% (20.1 to 27.7%)	0.7 (0.5 to 0.9)
Ischemic stroke	Qatar	2015	All Ages	0.0 (0.0 to 0.0)	22.2% (18.8 to 25.9%)	0.6 (0.5 to 0.8)
Ischemic stroke	Qatar	1990	Age-standardized		18.3% (13.6 to 24.0%)	7.8 (5.6 to 10.9)
Ischemic stroke	Qatar	1995	Age-standardized		18.2% (13.6 to 23.5%)	8.4 (6.0 to 11.6)
Ischemic stroke	Qatar	2000	Age-standardized		18.2% (13.6 to 23.7%)	8.0 (5.7 to 10.9)
Ischemic stroke	Qatar	2005	Age-standardized		17.8% (13.2 to 23.1%)	6.8 (4.9 to 9.5)
Ischemic stroke	Qatar	2010	Age-standardized		18.6% (13.8 to 24.9%)	5.5 (4.0 to 7.7)

Cause name	Location	Year	Age group	Number (in thousands)	PAF	Rate (per 100,000)
Ischemic stroke	Qatar	2015	Age-standardized		17.9% (13.0 to 23.7%)	4.8 (3.2 to 6.8)
Hemorrhagic stroke	Qatar	1990	All Ages	0.0 (0.0 to 0.0)	25.1% (21.2 to 28.9%)	2.4 (1.9 to 3.3)
Hemorrhagic stroke	Qatar	1995	All Ages	0.0 (0.0 to 0.0)	25.7% (21.8 to 29.6%)	2.9 (2.2 to 3.8)
Hemorrhagic stroke	Qatar	2000	All Ages	0.0 (0.0 to 0.0)	26.1% (22.0 to 30.2%)	2.9 (2.3 to 3.8)
Hemorrhagic stroke	Qatar	2005	All Ages	0.0 (0.0 to 0.0)	26.2% (22.4 to 30.4%)	2.1 (1.6 to 2.8)
Hemorrhagic stroke	Qatar	2010	All Ages	0.0 (0.0 to 0.0)	28.8% (24.8 to 32.9%)	1.5 (1.2 to 2.0)
Hemorrhagic stroke	Qatar	2015	All Ages	0.0 (0.0 to 0.0)	27.3% (23.3 to 31.2%)	1.3 (0.9 to 1.7)
Hemorrhagic stroke	Qatar	1990	Age-standardized		20.9% (17.4 to 24.4%)	5.7 (4.3 to 8.2)
Hemorrhagic stroke	Qatar	1995	Age-standardized		20.9% (17.4 to 24.6%)	6.2 (4.8 to 8.7)
Hemorrhagic stroke	Qatar	2000	Age-standardized		21.1% (17.4 to 24.9%)	5.8 (4.5 to 8.4)
Hemorrhagic stroke	Qatar	2005	Age-standardized		20.5% (16.8 to 24.5%)	4.6 (3.5 to 6.9)
Hemorrhagic stroke	Qatar	2010	Age-standardized		21.3% (17.5 to 25.5%)	3.9 (3.0 to 5.7)
Hemorrhagic stroke	Qatar	2015	Age-standardized		20.5% (16.7 to 24.8%)	3.4 (2.4 to 5.0)
Chronic obstructive pulmonary disease	Qatar	1990	All Ages	0.0 (0.0 to 0.0)	37.1% (25.7 to 52.5%)	0.9 (0.6 to 1.4)
Chronic obstructive pulmonary disease	Qatar	1995	All Ages	0.0 (0.0 to 0.0)	37.4% (25.9 to 52.6%)	1.1 (0.7 to 1.5)
Chronic obstructive pulmonary disease	Qatar	2000	All Ages	0.0 (0.0 to 0.0)	37.8% (26.1 to 53.2%)	1.2 (0.8 to 1.7)
Chronic obstructive pulmonary disease	Qatar	2005	All Ages	0.0 (0.0 to 0.0)	37.6% (26.0 to 53.1%)	0.9 (0.6 to 1.3)
Chronic obstructive pulmonary disease	Qatar	2010	All Ages	0.0 (0.0 to 0.0)	40.0% (28.4 to 56.5%)	0.5 (0.4 to 0.8)
Chronic obstructive pulmonary disease	Qatar	2015	All Ages	0.0 (0.0 to 0.0)	38.8% (26.5 to 54.9%)	0.6 (0.3 to 0.8)
Chronic obstructive pulmonary disease	Qatar	1990	Age-standardized		39.7% (27.6 to 56.0%)	5.5 (3.6 to 8.0)
Chronic obstructive pulmonary disease	Qatar	1995	Age-standardized		39.4% (27.4 to 55.5%)	5.7 (3.7 to 8.2)
Chronic obstructive pulmonary disease	Qatar	2000	Age-standardized		39.5% (27.1 to 55.5%)	5.7 (3.7 to 8.3)
Chronic obstructive pulmonary disease	Qatar	2005	Age-standardized		39.1% (27.1 to 55.0%)	5.2 (3.3 to 7.6)
Chronic obstructive pulmonary disease	Qatar	2010	Age-standardized		41.7% (29.6 to 58.8%)	4.1 (2.7 to 6.0)
Chronic obstructive pulmonary disease	Qatar	2015	Age-standardized		39.9% (27.4 to 56.3%)	3.8 (2.4 to 5.7)
All causes	Saudi Arabia	1990	All Ages	4.2 (3.6 to 4.8)	6.7% (5.6 to 7.6%)	25.4 (21.9 to 29.1)
All causes	Saudi Arabia	1995	All Ages	4.6 (4.1 to 5.2)	7.8% (7.0 to 8.7%)	24.5 (21.5 to 27.7)
All causes	Saudi Arabia	2000	All Ages	5.2 (4.6 to 5.8)	8.9% (8.0 to 9.9%)	24.3 (21.7 to 27.1)
All causes	Saudi Arabia	2005	All Ages	5.8 (5.2 to 6.5)	9.5% (8.5 to 10.5%)	23.4 (21.1 to 26.2)
All causes	Saudi Arabia	2010	All Ages	7.0 (6.3 to 7.9)	10.7% (9.7 to 11.8%)	25.1 (22.5 to 28.1)
All causes	Saudi Arabia	2015	All Ages	8.0 (7.0 to 9.2)	11.1% (10.0 to 12.2%)	25.6 (22.4 to 29.3)
All causes	Saudi Arabia	1990	Age-standardized		11.2% (9.7 to 12.7%)	80.4 (69.2 to 92.0)
All causes	Saudi Arabia	1995	Age-standardized		11.5% (10.2 to 12.9%)	76.7 (66.6 to 87.6)
All causes	Saudi Arabia	2000	Age-standardized		11.9% (10.5 to 13.3%)	73.6 (64.8 to 82.9)
All causes	Saudi Arabia	2005	Age-standardized		11.7% (10.4 to 13.2%)	69.2 (61.2 to 77.9)
All causes	Saudi Arabia	2010	Age-standardized		12.8% (11.4 to 14.3%)	71.8 (63.2 to 80.9)

Cause name	Location	Year	Age group	Number (in thousands)	PAF	Rate (per 100,000)
All causes	Saudi Arabia	2015	Age-standardized		12.4% (11.0 to 13.9%)	66.4 (57.7 to 76.3)
Lower respiratory infections	Saudi Arabia	1990	All Ages	1.2 (0.9 to 1.6)	38.5% (30.2 to 46.2%)	7.5 (5.3 to 9.9)
Lower respiratory infections	Saudi Arabia	1995	All Ages	1.2 (0.9 to 1.5)	38.4% (30.1 to 46.0%)	6.2 (4.6 to 7.7)
Lower respiratory infections	Saudi Arabia	2000	All Ages	1.2 (0.9 to 1.5)	39.6% (31.1 to 47.4%)	5.7 (4.1 to 7.1)
Lower respiratory infections	Saudi Arabia	2005	All Ages	1.3 (0.9 to 1.6)	38.8% (30.4 to 46.3%)	5.2 (3.7 to 6.5)
Lower respiratory infections	Saudi Arabia	2010	All Ages	1.6 (1.1 to 2.0)	43.7% (34.8 to 52.1%)	5.7 (4.0 to 7.2)
Lower respiratory infections	Saudi Arabia	2015	All Ages	1.7 (1.2 to 2.2)	42.3% (33.3 to 51.0%)	5.5 (3.7 to 7.1)
Lower respiratory infections	Saudi Arabia	1990	Age-standardized		38.6% (30.2 to 46.2%)	20.5 (13.6 to 26.9)
Lower respiratory infections	Saudi Arabia	1995	Age-standardized		38.4% (30.0 to 46.0%)	18.8 (12.5 to 24.2)
Lower respiratory infections	Saudi Arabia	2000	Age-standardized		39.5% (31.0 to 47.3%)	17.6 (11.9 to 22.4)
Lower respiratory infections	Saudi Arabia	2005	Age-standardized		38.5% (30.1 to 46.1%)	16.3 (11.0 to 20.7)
Lower respiratory infections	Saudi Arabia	2010	Age-standardized		43.4% (34.5 to 51.9%)	17.8 (11.9 to 23.0)
Lower respiratory infections	Saudi Arabia	2015	Age-standardized		42.1% (33.2 to 50.8%)	16.6 (11.0 to 21.7)
Tracheal, bronchus, and lung cancer	Saudi Arabia	1990	All Ages	0.1 (0.1 to 0.1)	30.2% (21.4 to 38.7%)	0.6 (0.4 to 0.7)
Tracheal, bronchus, and lung cancer	Saudi Arabia	1995	All Ages	0.1 (0.1 to 0.1)	30.0% (21.2 to 38.7%)	0.6 (0.4 to 0.8)
Tracheal, bronchus, and lung cancer	Saudi Arabia	2000	All Ages	0.1 (0.1 to 0.2)	31.4% (22.6 to 39.9%)	0.7 (0.5 to 0.9)
Tracheal, bronchus, and lung cancer	Saudi Arabia	2005	All Ages	0.2 (0.1 to 0.2)	31.0% (22.2 to 39.4%)	0.7 (0.5 to 0.9)
Tracheal, bronchus, and lung cancer	Saudi Arabia	2010	All Ages	0.3 (0.2 to 0.3)	36.8% (27.8 to 45.2%)	0.9 (0.7 to 1.1)
Tracheal, bronchus, and lung cancer	Saudi Arabia	2015	All Ages	0.3 (0.2 to 0.4)	34.4% (25.3 to 43.0%)	1.0 (0.7 to 1.3)
Tracheal, bronchus, and lung cancer	Saudi Arabia	1990	Age-standardized		30.4% (21.5 to 39.0%)	1.8 (1.2 to 2.4)
Tracheal, bronchus, and lung cancer	Saudi Arabia	1995	Age-standardized		30.2% (21.3 to 38.9%)	1.9 (1.3 to 2.4)
Tracheal, bronchus, and lung cancer	Saudi Arabia	2000	Age-standardized		31.5% (22.7 to 40.1%)	2.0 (1.4 to 2.6)
Tracheal, bronchus, and lung cancer	Saudi Arabia	2005	Age-standardized		30.9% (22.0 to 39.4%)	2.0 (1.4 to 2.6)
Tracheal, bronchus, and lung cancer	Saudi Arabia	2010	Age-standardized		36.5% (27.5 to 45.0%)	2.4 (1.8 to 3.1)
Tracheal, bronchus, and lung cancer	Saudi Arabia	2015	Age-standardized		34.2% (25.2 to 42.8%)	2.3 (1.7 to 3.0)
Cardiovascular diseases	Saudi Arabia	1990	All Ages	2.7 (2.2 to 3.1)	20.1% (17.8 to 22.6%)	16.3 (13.6 to 19.3)
Cardiovascular diseases	Saudi Arabia	1995	All Ages	3.1 (2.7 to 3.7)	20.3% (18.1 to 22.8%)	16.6 (14.2 to 19.5)
Cardiovascular diseases	Saudi Arabia	2000	All Ages	3.6 (3.1 to 4.1)	20.6% (18.4 to 23.2%)	16.9 (14.8 to 19.4)
Cardiovascular diseases	Saudi Arabia	2005	All Ages	4.1 (3.6 to 4.7)	20.3% (18.1 to 22.8%)	16.5 (14.5 to 18.9)
Cardiovascular diseases	Saudi Arabia	2010	All Ages	4.9 (4.3 to 5.6)	22.0% (19.8 to 24.6%)	17.4 (15.3 to 20.0)
Cardiovascular diseases	Saudi Arabia	2015	All Ages	5.6 (4.8 to 6.6)	21.8% (19.5 to 24.5%)	17.9 (15.3 to 21.1)
Cardiovascular diseases	Saudi Arabia	1990	Age-standardized		18.7% (16.2 to 21.7%)	53.8 (44.8 to 64.4)
Cardiovascular diseases	Saudi Arabia	1995	Age-standardized		18.6% (16.1 to 21.6%)	52.2 (44.0 to 62.3)
Cardiovascular diseases	Saudi Arabia	2000	Age-standardized		18.8% (16.3 to 21.8%)	50.2 (42.7 to 59.1)
Cardiovascular diseases	Saudi Arabia	2005	Age-standardized		18.3% (15.9 to 21.3%)	47.3 (40.3 to 56.0)
Cardiovascular diseases	Saudi Arabia	2010	Age-standardized		19.5% (17.1 to 22.7%)	47.6 (40.7 to 56.1)

Cause name	Location	Year	Age group	Number (in thousands)	PAF	Rate (per 100,000)
Cardiovascular diseases	Saudi Arabia	2015	Age-standardized		18.9% (16.3 to 22.1%)	43.8 (36.7 to 52.4)
Ischemic heart disease	Saudi Arabia	1990	All Ages	1.9 (1.6 to 2.3)	24.3% (20.9 to 28.0%)	11.9 (9.7 to 14.3)
Ischemic heart disease	Saudi Arabia	1995	All Ages	2.3 (1.9 to 2.7)	24.3% (21.0 to 28.1%)	12.1 (10.1 to 14.4)
Ischemic heart disease	Saudi Arabia	2000	All Ages	2.6 (2.2 to 3.1)	24.7% (21.4 to 28.6%)	12.2 (10.4 to 14.4)
Ischemic heart disease	Saudi Arabia	2005	All Ages	3.0 (2.5 to 3.5)	24.5% (21.3 to 28.4%)	11.9 (10.2 to 14.1)
Ischemic heart disease	Saudi Arabia	2010	All Ages	3.5 (3.0 to 4.2)	26.5% (23.2 to 30.8%)	12.5 (10.7 to 14.9)
Ischemic heart disease	Saudi Arabia	2015	All Ages	4.1 (3.4 to 5.0)	26.3% (22.8 to 30.3%)	13.1 (10.9 to 15.8)
Ischemic heart disease	Saudi Arabia	1990	Age-standardized		21.9% (18.3 to 26.6%)	38.3 (30.8 to 47.3)
Ischemic heart disease	Saudi Arabia	1995	Age-standardized		21.8% (18.2 to 26.5%)	37.1 (30.2 to 45.2)
Ischemic heart disease	Saudi Arabia	2000	Age-standardized		22.2% (18.6 to 26.9%)	35.4 (29.0 to 42.8)
Ischemic heart disease	Saudi Arabia	2005	Age-standardized		21.9% (18.3 to 26.5%)	33.3 (27.3 to 40.6)
Ischemic heart disease	Saudi Arabia	2010	Age-standardized		23.4% (19.6 to 28.5%)	33.2 (27.3 to 40.9)
Ischemic heart disease	Saudi Arabia	2015	Age-standardized		22.7% (18.9 to 28.1%)	30.6 (24.6 to 38.5)
Ischemic stroke	Saudi Arabia	1990	All Ages	0.4 (0.3 to 0.5)	18.5% (15.1 to 21.8%)	2.2 (1.8 to 2.8)
Ischemic stroke	Saudi Arabia	1995	All Ages	0.4 (0.3 to 0.5)	18.4% (14.9 to 21.8%)	2.3 (1.8 to 2.8)
Ischemic stroke	Saudi Arabia	2000	All Ages	0.5 (0.4 to 0.6)	18.6% (14.9 to 22.2%)	2.4 (1.9 to 3.0)
Ischemic stroke	Saudi Arabia	2005	All Ages	0.6 (0.5 to 0.7)	18.1% (14.6 to 21.7%)	2.4 (1.9 to 2.9)
Ischemic stroke	Saudi Arabia	2010	All Ages	0.7 (0.6 to 0.9)	20.0% (16.3 to 24.1%)	2.5 (2.0 to 3.1)
Ischemic stroke	Saudi Arabia	2015	All Ages	0.8 (0.6 to 1.0)	19.7% (16.1 to 23.5%)	2.5 (2.0 to 3.1)
Ischemic stroke	Saudi Arabia	1990	Age-standardized		17.0% (13.0 to 21.2%)	9.0 (6.7 to 12.0)
Ischemic stroke	Saudi Arabia	1995	Age-standardized		16.9% (12.9 to 21.0%)	8.8 (6.6 to 11.5)
Ischemic stroke	Saudi Arabia	2000	Age-standardized		17.2% (13.3 to 21.5%)	8.7 (6.5 to 11.0)
Ischemic stroke	Saudi Arabia	2005	Age-standardized		16.8% (12.9 to 21.0%)	8.3 (6.2 to 10.6)
Ischemic stroke	Saudi Arabia	2010	Age-standardized		18.5% (14.3 to 23.2%)	8.7 (6.6 to 11.0)
Ischemic stroke	Saudi Arabia	2015	Age-standardized		17.9% (13.6 to 22.5%)	8.0 (6.0 to 10.4)
Hemorrhagic stroke	Saudi Arabia	1990	All Ages	0.4 (0.3 to 0.4)	19.4% (16.7 to 22.2%)	2.2 (1.8 to 2.7)
Hemorrhagic stroke	Saudi Arabia	1995	All Ages	0.4 (0.4 to 0.5)	20.1% (17.4 to 22.7%)	2.2 (1.8 to 2.6)
Hemorrhagic stroke	Saudi Arabia	2000	All Ages	0.5 (0.4 to 0.6)	20.9% (18.2 to 23.7%)	2.3 (1.9 to 2.6)
Hemorrhagic stroke	Saudi Arabia	2005	All Ages	0.5 (0.5 to 0.6)	21.0% (18.2 to 23.7%)	2.2 (1.8 to 2.6)
Hemorrhagic stroke	Saudi Arabia	2010	All Ages	0.7 (0.6 to 0.8)	23.5% (20.4 to 26.6%)	2.3 (2.0 to 2.8)
Hemorrhagic stroke	Saudi Arabia	2015	All Ages	0.7 (0.6 to 0.9)	23.0% (19.9 to 26.0%)	2.3 (1.9 to 2.8)
Hemorrhagic stroke	Saudi Arabia	1990	Age-standardized		18.7% (15.3 to 22.0%)	6.5 (5.1 to 8.2)
Hemorrhagic stroke	Saudi Arabia	1995	Age-standardized		18.7% (15.3 to 21.9%)	6.3 (5.0 to 7.8)
Hemorrhagic stroke	Saudi Arabia	2000	Age-standardized		19.1% (15.6 to 22.5%)	6.1 (5.0 to 7.5)
Hemorrhagic stroke	Saudi Arabia	2005	Age-standardized		18.7% (15.4 to 22.1%)	5.6 (4.5 to 6.9)
Hemorrhagic stroke	Saudi Arabia	2010	Age-standardized		20.6% (17.1 to 24.3%)	5.7 (4.7 to 7.1)

Cause name	Location	Year	Age group	Number (in thousands)	PAF	Rate (per 100,000)
Hemorrhagic stroke	Saudi Arabia	2015	Age-standardized		19.8% (16.2 to 23.4%)	5.2 (4.1 to 6.5)
Chronic obstructive pulmonary disease	Saudi Arabia	1990	All Ages	0.2 (0.1 to 0.3)	34.3% (23.2 to 48.6%)	1.1 (0.7 to 1.6)
Chronic obstructive pulmonary disease	Saudi Arabia	1995	All Ages	0.2 (0.1 to 0.3)	34.6% (23.3 to 48.8%)	1.1 (0.7 to 1.5)
Chronic obstructive pulmonary disease	Saudi Arabia	2000	All Ages	0.2 (0.1 to 0.3)	35.9% (24.4 to 50.4%)	1.1 (0.7 to 1.5)
Chronic obstructive pulmonary disease	Saudi Arabia	2005	All Ages	0.3 (0.2 to 0.4)	35.3% (23.9 to 49.8%)	1.1 (0.7 to 1.5)
Chronic obstructive pulmonary disease	Saudi Arabia	2010	All Ages	0.3 (0.2 to 0.5)	39.9% (28.1 to 55.4%)	1.2 (0.8 to 1.7)
Chronic obstructive pulmonary disease	Saudi Arabia	2015	All Ages	0.4 (0.3 to 0.5)	38.4% (26.7 to 53.7%)	1.2 (0.8 to 1.7)
Chronic obstructive pulmonary disease	Saudi Arabia	1990	Age-standardized		35.5% (23.9 to 50.3%)	4.2 (2.7 to 6.1)
Chronic obstructive pulmonary disease	Saudi Arabia	1995	Age-standardized		35.4% (23.7 to 49.8%)	4.0 (2.6 to 5.7)
Chronic obstructive pulmonary disease	Saudi Arabia	2000	Age-standardized		36.4% (24.8 to 51.2%)	3.8 (2.5 to 5.3)
Chronic obstructive pulmonary disease	Saudi Arabia	2005	Age-standardized		35.6% (24.0 to 50.2%)	3.6 (2.4 to 5.1)
Chronic obstructive pulmonary disease	Saudi Arabia	2010	Age-standardized		40.1% (28.2 to 55.8%)	4.0 (2.8 to 5.6)
Chronic obstructive pulmonary disease	Saudi Arabia	2015	Age-standardized		38.5% (26.8 to 53.9%)	3.7 (2.5 to 5.2)
All causes	Sudan	1990	All Ages	15.7 (12.7 to 19.2)	7.5% (6.2 to 8.9%)	78.5 (63.4 to 95.9)
All causes	Sudan	1995	All Ages	17.6 (14.0 to 21.6)	7.6% (6.3 to 8.9%)	71.8 (56.9 to 88.1)
All causes	Sudan	2000	All Ages	17.4 (13.6 to 21.4)	7.3% (6.2 to 8.5%)	61.6 (48.3 to 75.9)
All causes	Sudan	2005	All Ages	17.8 (13.9 to 22.2)	7.6% (6.5 to 8.7%)	55.6 (43.4 to 69.0)
All causes	Sudan	2010	All Ages	18.9 (14.3 to 23.9)	7.9% (6.9 to 9.1%)	52.1 (39.5 to 65.9)
All causes	Sudan	2015	All Ages	19.2 (14.4 to 24.7)	7.9% (6.8 to 8.9%)	47.5 (35.5 to 61.2)
All causes	Sudan	1990	Age-standardized		10.1% (8.8 to 11.5%)	149.2 (122.5 to 182.5)
All causes	Sudan	1995	Age-standardized		10.2% (9.0 to 11.6%)	145.4 (116.0 to 181.2)
All causes	Sudan	2000	Age-standardized		10.1% (8.9 to 11.4%)	134.8 (107.1 to 171.3)
All causes	Sudan	2005	Age-standardized		10.2% (9.0 to 11.4%)	126.2 (98.2 to 159.9)
All causes	Sudan	2010	Age-standardized		10.4% (9.1 to 11.7%)	121.0 (91.7 to 152.1)
All causes	Sudan	2015	Age-standardized		10.0% (8.8 to 11.3%)	110.7 (83.4 to 143.1)
Lower respiratory infections	Sudan	1990	All Ages	7.4 (5.1 to 10.3)	32.3% (25.0 to 39.2%)	37.2 (25.3 to 51.4)
Lower respiratory infections	Sudan	1995	All Ages	7.5 (5.1 to 10.2)	32.3% (24.9 to 39.3%)	30.4 (20.8 to 41.3)
Lower respiratory infections	Sudan	2000	All Ages	6.4 (4.4 to 8.8)	32.3% (24.9 to 39.1%)	22.8 (15.4 to 31.2)
Lower respiratory infections	Sudan	2005	All Ages	6.0 (4.1 to 8.2)	31.7% (24.2 to 38.9%)	18.6 (12.8 to 25.4)
Lower respiratory infections	Sudan	2010	All Ages	5.5 (3.7 to 7.5)	32.0% (24.6 to 38.9%)	15.0 (10.3 to 20.7)
Lower respiratory infections	Sudan	2015	All Ages	4.5 (3.0 to 6.5)	30.1% (22.6 to 37.2%)	11.2 (7.4 to 16.1)
Lower respiratory infections	Sudan	1990	Age-standardized		32.3% (25.0 to 39.2%)	31.9 (22.5 to 42.9)
Lower respiratory infections	Sudan	1995	Age-standardized		32.3% (24.9 to 39.3%)	28.5 (20.1 to 38.1)
Lower respiratory infections	Sudan	2000	Age-standardized		32.3% (24.9 to 39.1%)	24.7 (17.2 to 33.7)
Lower respiratory infections	Sudan	2005	Age-standardized		31.7% (24.2 to 38.9%)	22.3 (15.1 to 31.3)
Lower respiratory infections	Sudan	2010	Age-standardized		32.0% (24.6 to 38.9%)	20.2 (13.5 to 28.3)

Cause name	Location	Year	Age group	Number (in thousands)	PAF	Rate (per 100,000)
Lower respiratory infections	Sudan	2015	Age-standardized		30.1% (22.6 to 37.2%)	17.0 (11.2 to 24.0)
Tracheal, bronchus, and lung cancer	Sudan	1990	All Ages	0.2 (0.1 to 0.3)	24.2% (16.2 to 32.1%)	1.0 (0.6 to 1.5)
Tracheal, bronchus, and lung cancer	Sudan	1995	All Ages	0.3 (0.1 to 0.4)	24.2% (16.1 to 32.3%)	1.0 (0.6 to 1.5)
Tracheal, bronchus, and lung cancer	Sudan	2000	All Ages	0.3 (0.2 to 0.4)	24.2% (16.2 to 32.4%)	1.0 (0.6 to 1.6)
Tracheal, bronchus, and lung cancer	Sudan	2005	All Ages	0.3 (0.2 to 0.5)	23.7% (15.7 to 31.7%)	1.0 (0.6 to 1.6)
Tracheal, bronchus, and lung cancer	Sudan	2010	All Ages	0.4 (0.2 to 0.6)	23.9% (16.1 to 32.0%)	1.1 (0.7 to 1.7)
Tracheal, bronchus, and lung cancer	Sudan	2015	All Ages	0.4 (0.3 to 0.7)	22.2% (14.5 to 30.3%)	1.1 (0.6 to 1.7)
Tracheal, bronchus, and lung cancer	Sudan	1990	Age-standardized		24.4% (16.3 to 32.4%)	2.7 (1.6 to 3.9)
Tracheal, bronchus, and lung cancer	Sudan	1995	Age-standardized		24.4% (16.2 to 32.6%)	2.8 (1.7 to 4.1)
Tracheal, bronchus, and lung cancer	Sudan	2000	Age-standardized		24.3% (16.3 to 32.6%)	2.8 (1.7 to 4.1)
Tracheal, bronchus, and lung cancer	Sudan	2005	Age-standardized		23.9% (15.8 to 32.0%)	2.8 (1.6 to 4.2)
Tracheal, bronchus, and lung cancer	Sudan	2010	Age-standardized		24.1% (16.2 to 32.3%)	2.9 (1.7 to 4.4)
Tracheal, bronchus, and lung cancer	Sudan	2015	Age-standardized		22.4% (14.6 to 30.5%)	2.7 (1.6 to 4.2)
Cardiovascular diseases	Sudan	1990	All Ages	7.3 (5.8 to 9.2)	16.9% (14.8 to 19.1%)	36.3 (28.8 to 45.9)
Cardiovascular diseases	Sudan	1995	All Ages	8.9 (6.9 to 11.4)	17.1% (15.2 to 19.3%)	36.3 (27.9 to 46.4)
Cardiovascular diseases	Sudan	2000	All Ages	9.5 (7.2 to 12.5)	17.2% (15.3 to 19.4%)	33.9 (25.6 to 44.5)
Cardiovascular diseases	Sudan	2005	All Ages	10.2 (7.6 to 13.5)	17.1% (15.1 to 19.2%)	31.9 (23.7 to 42.1)
Cardiovascular diseases	Sudan	2010	All Ages	11.5 (8.3 to 14.9)	17.2% (15.2 to 19.5%)	31.7 (22.8 to 41.1)
Cardiovascular diseases	Sudan	2015	All Ages	12.5 (9.0 to 16.8)	16.8% (14.8 to 18.9%)	31.0 (22.3 to 41.6)
Cardiovascular diseases	Sudan	1990	Age-standardized		16.5% (14.2 to 18.9%)	101.2 (80.2 to 127.2)
Cardiovascular diseases	Sudan	1995	Age-standardized		16.6% (14.3 to 19.1%)	100.4 (78.4 to 129.1)
Cardiovascular diseases	Sudan	2000	Age-standardized		16.5% (14.3 to 18.9%)	94.4 (72.4 to 124.9)
Cardiovascular diseases	Sudan	2005	Age-standardized		16.2% (14.0 to 18.7%)	88.0 (66.2 to 114.2)
Cardiovascular diseases	Sudan	2010	Age-standardized		16.3% (14.0 to 18.7%)	84.4 (62.4 to 108.2)
Cardiovascular diseases	Sudan	2015	Age-standardized		15.7% (13.4 to 18.1%)	78.8 (57.8 to 103.7)
Ischemic heart disease	Sudan	1990	All Ages	5.2 (4.0 to 6.6)	22.3% (19.0 to 25.7%)	25.8 (20.0 to 33.1)
Ischemic heart disease	Sudan	1995	All Ages	6.2 (4.7 to 8.1)	22.3% (19.0 to 25.6%)	25.3 (19.0 to 33.0)
Ischemic heart disease	Sudan	2000	All Ages	6.5 (4.9 to 8.7)	22.2% (18.9 to 25.6%)	23.2 (17.3 to 31.0)
Ischemic heart disease	Sudan	2005	All Ages	7.0 (5.1 to 9.4)	21.9% (18.7 to 25.2%)	21.9 (16.0 to 29.2)
Ischemic heart disease	Sudan	2010	All Ages	7.9 (5.6 to 10.4)	22.0% (18.8 to 25.4%)	21.7 (15.5 to 28.6)
Ischemic heart disease	Sudan	2015	All Ages	8.6 (6.0 to 11.7)	21.2% (18.0 to 24.6%)	21.4 (15.0 to 29.1)
Ischemic heart disease	Sudan	1990	Age-standardized		20.4% (17.2 to 24.2%)	71.1 (54.8 to 91.8)
Ischemic heart disease	Sudan	1995	Age-standardized		20.3% (17.1 to 24.1%)	69.3 (52.5 to 91.2)
Ischemic heart disease	Sudan	2000	Age-standardized		20.2% (17.0 to 24.0%)	63.9 (48.3 to 85.5)
Ischemic heart disease	Sudan	2005	Age-standardized		19.9% (16.6 to 23.8%)	59.5 (44.2 to 79.2)
Ischemic heart disease	Sudan	2010	Age-standardized		20.0% (16.5 to 23.8%)	57.2 (41.5 to 74.9)

Cause name	Location	Year	Age group	Number (in thousands)	PAF	Rate (per 100,000)
Ischemic heart disease	Sudan	2015	Age-standardized		19.3% (15.8 to 23.2%)	53.5 (37.9 to 71.5)
Ischemic stroke	Sudan	1990	All Ages	1.0 (0.7 to 1.5)	16.8% (14.0 to 19.5%)	5.1 (3.3 to 7.3)
Ischemic stroke	Sudan	1995	All Ages	1.3 (0.8 to 1.8)	16.8% (13.8 to 19.5%)	5.2 (3.4 to 7.4)
Ischemic stroke	Sudan	2000	All Ages	1.4 (1.0 to 2.1)	16.7% (13.6 to 19.5%)	5.1 (3.4 to 7.3)
Ischemic stroke	Sudan	2005	All Ages	1.6 (1.0 to 2.2)	16.4% (13.3 to 19.3%)	4.9 (3.2 to 6.8)
Ischemic stroke	Sudan	2010	All Ages	1.8 (1.2 to 2.5)	16.4% (13.3 to 19.4%)	4.9 (3.2 to 6.9)
Ischemic stroke	Sudan	2015	All Ages	1.9 (1.3 to 2.8)	15.7% (12.7 to 18.6%)	4.8 (3.2 to 6.8)
Ischemic stroke	Sudan	1990	Age-standardized		15.5% (12.1 to 18.9%)	16.6 (10.6 to 24.0)
Ischemic stroke	Sudan	1995	Age-standardized		15.5% (12.0 to 18.8%)	17.0 (10.9 to 24.8)
Ischemic stroke	Sudan	2000	Age-standardized		15.4% (11.9 to 18.9%)	16.6 (10.7 to 23.8)
Ischemic stroke	Sudan	2005	Age-standardized		15.1% (11.6 to 18.6%)	15.7 (10.0 to 22.4)
Ischemic stroke	Sudan	2010	Age-standardized		15.2% (11.6 to 18.6%)	15.2 (9.9 to 21.7)
Ischemic stroke	Sudan	2015	Age-standardized		14.5% (11.0 to 18.0%)	14.1 (9.3 to 20.3)
Hemorrhagic stroke	Sudan	1990	All Ages	1.1 (0.8 to 1.5)	14.9% (12.3 to 17.6%)	5.4 (4.0 to 7.6)
Hemorrhagic stroke	Sudan	1995	All Ages	1.4 (1.0 to 2.0)	15.5% (13.0 to 18.3%)	5.7 (4.2 to 7.9)
Hemorrhagic stroke	Sudan	2000	All Ages	1.6 (1.1 to 2.2)	16.3% (13.8 to 18.9%)	5.6 (4.0 to 7.9)
Hemorrhagic stroke	Sudan	2005	All Ages	1.7 (1.2 to 2.4)	16.3% (13.7 to 19.0%)	5.2 (3.6 to 7.3)
Hemorrhagic stroke	Sudan	2010	All Ages	1.8 (1.2 to 2.6)	16.6% (14.0 to 19.4%)	5.1 (3.4 to 7.1)
Hemorrhagic stroke	Sudan	2015	All Ages	2.0 (1.3 to 2.9)	16.4% (13.8 to 19.1%)	4.9 (3.3 to 7.1)
Hemorrhagic stroke	Sudan	1990	Age-standardized		16.8% (14.0 to 19.4%)	13.5 (9.7 to 19.1)
Hemorrhagic stroke	Sudan	1995	Age-standardized		16.9% (14.1 to 19.6%)	14.1 (10.2 to 20.0)
Hemorrhagic stroke	Sudan	2000	Age-standardized		17.0% (14.1 to 19.7%)	13.9 (9.8 to 19.7)
Hemorrhagic stroke	Sudan	2005	Age-standardized		16.7% (13.7 to 19.6%)	12.7 (8.8 to 18.0)
Hemorrhagic stroke	Sudan	2010	Age-standardized		16.8% (13.8 to 19.7%)	12.1 (8.2 to 16.9)
Hemorrhagic stroke	Sudan	2015	Age-standardized		16.1% (13.2 to 19.0%)	11.2 (7.8 to 16.1)
Chronic obstructive pulmonary disease	Sudan	1990	All Ages	0.8 (0.4 to 1.4)	25.8% (15.7 to 37.1%)	4.0 (1.8 to 7.1)
Chronic obstructive pulmonary disease	Sudan	1995	All Ages	1.0 (0.5 to 1.7)	26.6% (16.8 to 37.9%)	4.2 (2.1 to 7.0)
Chronic obstructive pulmonary disease	Sudan	2000	All Ages	1.1 (0.6 to 1.8)	27.5% (17.2 to 38.9%)	3.9 (2.0 to 6.5)
Chronic obstructive pulmonary disease	Sudan	2005	All Ages	1.3 (0.7 to 2.2)	27.5% (17.1 to 39.0%)	4.1 (2.1 to 6.8)
Chronic obstructive pulmonary disease	Sudan	2010	All Ages	1.6 (0.8 to 2.6)	27.9% (17.4 to 39.6%)	4.3 (2.2 to 7.3)
Chronic obstructive pulmonary disease	Sudan	2015	All Ages	1.7 (0.8 to 2.9)	26.9% (16.2 to 38.3%)	4.1 (2.1 to 7.1)
Chronic obstructive pulmonary disease	Sudan	1990	Age-standardized		29.5% (18.5 to 41.9%)	13.5 (6.1 to 23.8)
Chronic obstructive pulmonary disease	Sudan	1995	Age-standardized		29.6% (18.8 to 42.2%)	13.7 (7.0 to 23.1)
Chronic obstructive pulmonary disease	Sudan	2000	Age-standardized		29.8% (18.9 to 42.2%)	12.9 (6.8 to 21.6)
Chronic obstructive pulmonary disease	Sudan	2005	Age-standardized		29.4% (18.5 to 42.0%)	13.1 (6.8 to 21.9)
Chronic obstructive pulmonary disease	Sudan	2010	Age-standardized		29.6% (18.4 to 42.1%)	13.4 (6.9 to 22.6)

Cause name	Location	Year	Age group	Number (in thousands)	PAF	Rate (per 100,000)
Chronic obstructive pulmonary disease	Sudan	2015	Age-standardized		28.1% (17.1 to 40.3%)	12.2 (6.2 to 20.3)
All causes	Syria	1990	All Ages	4.8 (4.0 to 5.8)	8.4% (7.2 to 9.5%)	38.7 (32.4 to 46.4)
All causes	Syria	1995	All Ages	4.7 (3.9 to 5.6)	8.5% (7.5 to 9.6%)	32.6 (27.3 to 39.2)
All causes	Syria	2000	All Ages	4.9 (4.1 to 5.8)	9.0% (7.8 to 10.1%)	30.1 (25.3 to 35.8)
All causes	Syria	2005	All Ages	5.1 (4.4 to 5.9)	9.0% (7.9 to 10.2%)	28.2 (24.4 to 32.3)
All causes	Syria	2010	All Ages	6.4 (5.6 to 7.3)	10.3% (9.0 to 11.6%)	31.0 (27.0 to 35.2)
All causes	Syria	2015	All Ages	6.6 (5.4 to 7.7)	5.8% (4.1 to 8.4%)	35.4 (28.8 to 41.5)
All causes	Syria	1990	Age-standardized		10.4% (9.1 to 11.8%)	99.0 (82.8 to 118.6)
All causes	Syria	1995	Age-standardized		10.2% (8.8 to 11.6%)	85.8 (70.8 to 105.5)
All causes	Syria	2000	Age-standardized		10.1% (8.6 to 11.7%)	78.0 (64.5 to 94.4)
All causes	Syria	2005	Age-standardized		9.9% (8.4 to 11.4%)	72.1 (60.6 to 83.8)
All causes	Syria	2010	Age-standardized		10.8% (9.2 to 12.5%)	72.7 (61.4 to 84.8)
All causes	Syria	2015	Age-standardized		7.5% (5.8 to 9.7%)	73.5 (59.3 to 87.7)
Lower respiratory infections	Syria	1990	All Ages	1.0 (0.6 to 1.5)	24.1% (16.8 to 32.7%)	8.1 (4.6 to 12.1)
Lower respiratory infections	Syria	1995	All Ages	0.7 (0.4 to 1.2)	23.4% (16.0 to 32.1%)	5.1 (3.1 to 8.1)
Lower respiratory infections	Syria	2000	All Ages	0.6 (0.4 to 1.0)	23.3% (15.7 to 31.7%)	3.7 (2.2 to 6.1)
Lower respiratory infections	Syria	2005	All Ages	0.5 (0.3 to 0.9)	22.7% (15.0 to 31.4%)	2.9 (1.7 to 5.1)
Lower respiratory infections	Syria	2010	All Ages	0.8 (0.5 to 1.3)	27.0% (19.5 to 35.0%)	3.7 (2.4 to 6.1)
Lower respiratory infections	Syria	2015	All Ages	0.7 (0.5 to 1.2)	27.2% (19.6 to 34.7%)	3.9 (2.4 to 6.5)
Lower respiratory infections	Syria	1990	Age-standardized		24.1% (16.8 to 32.7%)	10.3 (6.1 to 18.1)
Lower respiratory infections	Syria	1995	Age-standardized		23.4% (16.0 to 32.1%)	7.8 (4.3 to 15.3)
Lower respiratory infections	Syria	2000	Age-standardized		23.3% (15.7 to 31.7%)	6.5 (3.6 to 13.2)
Lower respiratory infections	Syria	2005	Age-standardized		22.7% (15.0 to 31.4%)	5.6 (3.0 to 11.9)
Lower respiratory infections	Syria	2010	Age-standardized		27.0% (19.5 to 35.0%)	6.8 (4.0 to 13.4)
Lower respiratory infections	Syria	2015	Age-standardized		27.2% (19.6 to 34.7%)	6.8 (3.8 to 13.3)
Tracheal, bronchus, and lung cancer	Syria	1990	All Ages	0.1 (0.0 to 0.1)	17.1% (10.4 to 24.4%)	0.4 (0.3 to 0.7)
Tracheal, bronchus, and lung cancer	Syria	1995	All Ages	0.1 (0.0 to 0.1)	16.5% (9.9 to 23.5%)	0.4 (0.2 to 0.7)
Tracheal, bronchus, and lung cancer	Syria	2000	All Ages	0.1 (0.0 to 0.1)	16.4% (9.8 to 23.3%)	0.4 (0.3 to 0.7)
Tracheal, bronchus, and lung cancer	Syria	2005	All Ages	0.1 (0.1 to 0.1)	16.0% (9.5 to 23.1%)	0.5 (0.3 to 0.7)
Tracheal, bronchus, and lung cancer	Syria	2010	All Ages	0.2 (0.1 to 0.2)	19.4% (12.0 to 26.9%)	0.8 (0.5 to 1.1)
Tracheal, bronchus, and lung cancer	Syria	2015	All Ages	0.2 (0.1 to 0.2)	19.6% (12.3 to 27.2%)	0.9 (0.5 to 1.3)
Tracheal, bronchus, and lung cancer	Syria	1990	Age-standardized		17.3% (10.5 to 24.7%)	1.3 (0.7 to 1.9)
Tracheal, bronchus, and lung cancer	Syria	1995	Age-standardized		16.7% (10.0 to 23.8%)	1.2 (0.7 to 1.8)
Tracheal, bronchus, and lung cancer	Syria	2000	Age-standardized		16.6% (9.9 to 23.7%)	1.2 (0.7 to 1.8)
Tracheal, bronchus, and lung cancer	Syria	2005	Age-standardized		16.1% (9.6 to 23.4%)	1.3 (0.7 to 1.8)
Tracheal, bronchus, and lung cancer	Syria	2010	Age-standardized		19.6% (12.1 to 27.1%)	1.9 (1.2 to 2.7)

Cause name	Location	Year	Age group	Number (in thousands)	PAF	Rate (per 100,000)
Tracheal, bronchus, and lung cancer	Syria	2015	Age-standardized		19.8% (12.4 to 27.4%)	1.8 (1.1 to 2.6)
Cardiovascular diseases	Syria	1990	All Ages	3.6 (3.0 to 4.3)	15.5% (13.7 to 17.4%)	28.8 (23.8 to 34.8)
Cardiovascular diseases	Syria	1995	All Ages	3.7 (3.1 to 4.5)	15.3% (13.4 to 17.3%)	25.9 (21.4 to 31.6)
Cardiovascular diseases	Syria	2000	All Ages	4.0 (3.4 to 4.9)	15.2% (13.2 to 17.3%)	24.8 (20.9 to 30.0)
Cardiovascular diseases	Syria	2005	All Ages	4.3 (3.7 to 5.0)	14.9% (13.0 to 17.0%)	23.7 (20.4 to 27.5)
Cardiovascular diseases	Syria	2010	All Ages	5.3 (4.5 to 6.0)	16.6% (14.5 to 18.9%)	25.4 (21.9 to 29.1)
Cardiovascular diseases	Syria	2015	All Ages	5.5 (4.4 to 6.5)	16.5% (14.3 to 18.9%)	29.3 (23.4 to 34.8)
Cardiovascular diseases	Syria	1990	Age-standardized		14.8% (12.7 to 17.0%)	82.3 (67.4 to 99.6)
Cardiovascular diseases	Syria	1995	Age-standardized		14.5% (12.3 to 16.7%)	72.6 (59.1 to 90.2)
Cardiovascular diseases	Syria	2000	Age-standardized		14.3% (12.2 to 16.7%)	66.6 (54.5 to 81.3)
Cardiovascular diseases	Syria	2005	Age-standardized		14.0% (11.8 to 16.2%)	61.9 (51.6 to 72.7)
Cardiovascular diseases	Syria	2010	Age-standardized		15.4% (12.9 to 18.1%)	61.0 (50.7 to 72.5)
Cardiovascular diseases	Syria	2015	Age-standardized		15.4% (13.0 to 18.0%)	61.8 (48.6 to 74.2)
Ischemic heart disease	Syria	1990	All Ages	2.5 (2.0 to 3.1)	18.9% (16.0 to 21.9%)	19.9 (16.0 to 24.5)
Ischemic heart disease	Syria	1995	All Ages	2.6 (2.1 to 3.2)	18.6% (15.7 to 21.5%)	17.8 (14.3 to 22.2)
Ischemic heart disease	Syria	2000	All Ages	2.8 (2.3 to 3.5)	18.4% (15.6 to 21.4%)	17.2 (14.0 to 21.2)
Ischemic heart disease	Syria	2005	All Ages	3.0 (2.5 to 3.6)	17.9% (15.0 to 20.9%)	16.7 (13.9 to 19.7)
Ischemic heart disease	Syria	2010	All Ages	4.0 (3.3 to 4.7)	19.4% (16.3 to 22.6%)	19.2 (16.0 to 22.8)
Ischemic heart disease	Syria	2015	All Ages	4.1 (3.2 to 5.0)	19.2% (16.0 to 22.6%)	22.1 (17.2 to 27.0)
Ischemic heart disease	Syria	1990	Age-standardized		17.6% (14.5 to 20.8%)	56.9 (44.9 to 71.4)
Ischemic heart disease	Syria	1995	Age-standardized		17.2% (14.1 to 20.5%)	49.9 (39.1 to 63.3)
Ischemic heart disease	Syria	2000	Age-standardized		17.0% (13.9 to 20.4%)	46.4 (36.8 to 58.2)
Ischemic heart disease	Syria	2005	Age-standardized		16.6% (13.4 to 19.9%)	43.5 (34.9 to 53.2)
Ischemic heart disease	Syria	2010	Age-standardized		17.8% (14.3 to 21.7%)	46.4 (36.7 to 56.8)
Ischemic heart disease	Syria	2015	Age-standardized		17.9% (14.3 to 21.6%)	46.5 (35.3 to 57.8)
Ischemic stroke	Syria	1990	All Ages	0.5 (0.3 to 0.6)	13.8% (10.9 to 16.5%)	3.6 (2.8 to 4.6)
Ischemic stroke	Syria	1995	All Ages	0.5 (0.4 to 0.6)	13.4% (10.5 to 16.0%)	3.4 (2.5 to 4.4)
Ischemic stroke	Syria	2000	All Ages	0.5 (0.4 to 0.7)	13.2% (10.2 to 16.1%)	3.2 (2.4 to 4.1)
Ischemic stroke	Syria	2005	All Ages	0.6 (0.4 to 0.7)	12.9% (10.0 to 15.7%)	3.0 (2.3 to 3.8)
Ischemic stroke	Syria	2010	All Ages	0.6 (0.4 to 0.7)	14.3% (11.2 to 17.3%)	2.7 (2.1 to 3.4)
Ischemic stroke	Syria	2015	All Ages	0.6 (0.5 to 0.8)	14.3% (11.1 to 17.3%)	3.3 (2.5 to 4.2)
Ischemic stroke	Syria	1990	Age-standardized		12.9% (9.6 to 16.1%)	11.8 (8.5 to 15.6)
Ischemic stroke	Syria	1995	Age-standardized		12.6% (9.2 to 15.7%)	10.8 (7.8 to 14.8)
Ischemic stroke	Syria	2000	Age-standardized		12.5% (9.1 to 15.7%)	9.7 (6.9 to 13.1)
Ischemic stroke	Syria	2005	Age-standardized		12.2% (8.9 to 15.6%)	8.9 (6.4 to 11.8)
Ischemic stroke	Syria	2010	Age-standardized		13.4% (10.0 to 16.9%)	7.3 (5.3 to 9.4)

Cause name	Location	Year	Age group	Number (in thousands)	PAF	Rate (per 100,000)
Ischemic stroke	Syria	2015	Age-standardized		13.5% (10.2 to 17.0%)	7.7 (5.5 to 10.2)
Hemorrhagic stroke	Syria	1990	All Ages	0.7 (0.5 to 0.8)	14.0% (11.5 to 16.4%)	5.3 (4.2 to 6.7)
Hemorrhagic stroke	Syria	1995	All Ages	0.7 (0.5 to 0.9)	13.9% (11.4 to 16.3%)	4.7 (3.7 to 6.0)
Hemorrhagic stroke	Syria	2000	All Ages	0.7 (0.6 to 0.9)	13.9% (11.4 to 16.4%)	4.4 (3.5 to 5.5)
Hemorrhagic stroke	Syria	2005	All Ages	0.7 (0.6 to 0.9)	13.6% (11.1 to 16.0%)	4.0 (3.2 to 4.8)
Hemorrhagic stroke	Syria	2010	All Ages	0.7 (0.6 to 0.9)	15.8% (13.2 to 18.5%)	3.5 (2.9 to 4.2)
Hemorrhagic stroke	Syria	2015	All Ages	0.7 (0.6 to 0.9)	15.7% (13.1 to 18.4%)	3.9 (3.0 to 4.9)
Hemorrhagic stroke	Syria	1990	Age-standardized		14.2% (11.5 to 16.9%)	13.6 (10.6 to 17.3)
Hemorrhagic stroke	Syria	1995	Age-standardized		13.9% (11.2 to 16.7%)	11.8 (9.1 to 15.1)
Hemorrhagic stroke	Syria	2000	Age-standardized		13.8% (10.9 to 16.7%)	10.5 (8.1 to 13.4)
Hemorrhagic stroke	Syria	2005	Age-standardized		13.3% (10.5 to 16.1%)	9.4 (7.3 to 11.7)
Hemorrhagic stroke	Syria	2010	Age-standardized		14.9% (11.8 to 17.8%)	7.4 (5.8 to 9.2)
Hemorrhagic stroke	Syria	2015	Age-standardized		15.0% (12.0 to 17.8%)	7.6 (5.7 to 9.6)
Chronic obstructive pulmonary disease	Syria	1990	All Ages	0.2 (0.1 to 0.3)	18.5% (10.0 to 27.9%)	1.4 (0.7 to 2.2)
Chronic obstructive pulmonary disease	Syria	1995	All Ages	0.2 (0.1 to 0.3)	19.7% (10.6 to 29.3%)	1.2 (0.6 to 1.9)
Chronic obstructive pulmonary disease	Syria	2000	All Ages	0.2 (0.1 to 0.3)	20.7% (11.2 to 31.0%)	1.1 (0.6 to 1.8)
Chronic obstructive pulmonary disease	Syria	2005	All Ages	0.2 (0.1 to 0.3)	21.1% (11.5 to 31.7%)	1.1 (0.6 to 1.7)
Chronic obstructive pulmonary disease	Syria	2010	All Ages	0.2 (0.1 to 0.3)	24.7% (14.5 to 35.7%)	1.0 (0.6 to 1.5)
Chronic obstructive pulmonary disease	Syria	2015	All Ages	0.2 (0.1 to 0.4)	25.0% (14.4 to 36.4%)	1.3 (0.7 to 2.0)
Chronic obstructive pulmonary disease	Syria	1990	Age-standardized		22.8% (12.6 to 33.2%)	5.1 (2.6 to 8.2)
Chronic obstructive pulmonary disease	Syria	1995	Age-standardized		22.5% (12.4 to 33.3%)	4.3 (2.2 to 6.8)
Chronic obstructive pulmonary disease	Syria	2000	Age-standardized		22.6% (12.3 to 33.6%)	3.7 (1.9 to 5.9)
Chronic obstructive pulmonary disease	Syria	2005	Age-standardized		22.3% (12.2 to 33.0%)	3.4 (1.7 to 5.2)
Chronic obstructive pulmonary disease	Syria	2010	Age-standardized		25.6% (15.2 to 36.9%)	3.0 (1.6 to 4.4)
Chronic obstructive pulmonary disease	Syria	2015	Age-standardized		25.8% (14.9 to 37.2%)	3.2 (1.7 to 4.9)
All causes	Tunisia	1990	All Ages	2.6 (2.3 to 3.0)	6.2% (5.4 to 7.1%)	31.9 (27.3 to 36.9)
All causes	Tunisia	1995	All Ages	3.0 (2.6 to 3.5)	6.7% (5.8 to 7.6%)	33.3 (28.6 to 38.7)
All causes	Tunisia	2000	All Ages	3.1 (2.6 to 3.6)	6.8% (5.9 to 7.7%)	31.7 (26.7 to 36.9)
All causes	Tunisia	2005	All Ages	3.0 (2.5 to 3.6)	6.2% (5.4 to 7.1%)	29.6 (24.3 to 35.5)
All causes	Tunisia	2010	All Ages	3.6 (2.9 to 4.4)	6.8% (5.9 to 7.8%)	33.6 (27.0 to 41.3)
All causes	Tunisia	2015	All Ages	4.5 (3.5 to 5.5)	7.7% (6.7 to 8.7%)	39.6 (31.3 to 49.3)
All causes	Tunisia	1990	Age-standardized		7.3% (6.3 to 8.3%)	62.9 (53.2 to 73.2)
All causes	Tunisia	1995	Age-standardized		7.3% (6.3 to 8.4%)	62.1 (52.3 to 72.7)
All causes	Tunisia	2000	Age-standardized		7.1% (6.1 to 8.1%)	51.5 (43.4 to 60.5)
All causes	Tunisia	2005	Age-standardized		6.3% (5.4 to 7.3%)	42.2 (34.5 to 50.9)
All causes	Tunisia	2010	Age-standardized		6.9% (6.0 to 8.0%)	43.8 (35.3 to 54.1)

Cause name	Location	Year	Age group	Number (in thousands)	PAF	Rate (per 100,000)
All causes	Tunisia	2015	Age-standardized		7.7% (6.7 to 8.8%)	47.9 (37.8 to 59.4)
Lower respiratory infections	Tunisia	1990	All Ages	0.6 (0.4 to 0.8)	26.3% (18.8 to 34.2%)	7.6 (5.2 to 10.3)
Lower respiratory infections	Tunisia	1995	All Ages	0.6 (0.4 to 0.8)	25.6% (17.9 to 33.8%)	6.2 (4.1 to 8.7)
Lower respiratory infections	Tunisia	2000	All Ages	0.5 (0.3 to 0.7)	25.3% (17.5 to 33.6%)	5.2 (3.5 to 7.4)
Lower respiratory infections	Tunisia	2005	All Ages	0.5 (0.3 to 0.7)	21.6% (13.3 to 31.1%)	4.5 (2.7 to 6.8)
Lower respiratory infections	Tunisia	2010	All Ages	0.6 (0.4 to 0.8)	25.0% (17.3 to 33.3%)	5.3 (3.5 to 7.7)
Lower respiratory infections	Tunisia	2015	All Ages	0.7 (0.5 to 1.0)	28.8% (21.3 to 36.5%)	6.4 (4.4 to 9.2)
Lower respiratory infections	Tunisia	1990	Age-standardized		26.3% (18.8 to 34.2%)	11.9 (7.9 to 16.9)
Lower respiratory infections	Tunisia	1995	Age-standardized		25.6% (17.9 to 33.8%)	10.5 (6.9 to 15.4)
Lower respiratory infections	Tunisia	2000	Age-standardized		25.3% (17.5 to 33.6%)	8.5 (5.5 to 12.4)
Lower respiratory infections	Tunisia	2005	Age-standardized		21.6% (13.3 to 31.1%)	6.5 (3.8 to 9.9)
Lower respiratory infections	Tunisia	2010	Age-standardized		25.0% (17.3 to 33.3%)	7.0 (4.6 to 10.4)
Lower respiratory infections	Tunisia	2015	Age-standardized		28.8% (21.3 to 36.5%)	7.9 (5.3 to 11.4)
Tracheal, bronchus, and lung cancer	Tunisia	1990	All Ages	0.3 (0.2 to 0.4)	18.9% (11.6 to 26.4%)	3.2 (2.0 to 4.7)
Tracheal, bronchus, and lung cancer	Tunisia	1995	All Ages	0.3 (0.2 to 0.5)	18.4% (11.3 to 26.0%)	3.6 (2.2 to 5.2)
Tracheal, bronchus, and lung cancer	Tunisia	2000	All Ages	0.4 (0.2 to 0.5)	18.1% (11.1 to 25.7%)	3.7 (2.2 to 5.4)
Tracheal, bronchus, and lung cancer	Tunisia	2005	All Ages	0.3 (0.2 to 0.5)	15.3% (8.9 to 22.4%)	3.4 (1.9 to 5.1)
Tracheal, bronchus, and lung cancer	Tunisia	2010	All Ages	0.5 (0.3 to 0.7)	18.0% (10.8 to 25.3%)	4.3 (2.5 to 6.4)
Tracheal, bronchus, and lung cancer	Tunisia	2015	All Ages	0.6 (0.4 to 1.0)	21.1% (13.5 to 29.2%)	5.7 (3.3 to 8.6)
Tracheal, bronchus, and lung cancer	Tunisia	1990	Age-standardized		18.9% (11.6 to 26.4%)	6.0 (3.6 to 8.7)
Tracheal, bronchus, and lung cancer	Tunisia	1995	Age-standardized		18.4% (11.3 to 26.1%)	6.1 (3.7 to 8.8)
Tracheal, bronchus, and lung cancer	Tunisia	2000	Age-standardized		18.2% (11.1 to 25.7%)	5.5 (3.3 to 8.1)
Tracheal, bronchus, and lung cancer	Tunisia	2005	Age-standardized		15.4% (8.9 to 22.5%)	4.5 (2.6 to 6.9)
Tracheal, bronchus, and lung cancer	Tunisia	2010	Age-standardized		18.0% (10.8 to 25.3%)	5.3 (3.1 to 8.0)
Tracheal, bronchus, and lung cancer	Tunisia	2015	Age-standardized		21.1% (13.5 to 29.2%)	6.5 (3.8 to 9.8)
Cardiovascular diseases	Tunisia	1990	All Ages	1.4 (1.2 to 1.7)	13.3% (11.6 to 15.2%)	17.4 (14.6 to 20.2)
Cardiovascular diseases	Tunisia	1995	All Ages	1.8 (1.5 to 2.1)	13.3% (11.5 to 15.2%)	19.3 (16.4 to 22.6)
Cardiovascular diseases	Tunisia	2000	All Ages	1.8 (1.5 to 2.2)	13.4% (11.6 to 15.3%)	18.9 (15.7 to 22.2)
Cardiovascular diseases	Tunisia	2005	All Ages	1.9 (1.5 to 2.3)	12.3% (10.5 to 14.1%)	18.3 (14.6 to 22.3)
Cardiovascular diseases	Tunisia	2010	All Ages	2.1 (1.7 to 2.7)	13.1% (11.2 to 15.0%)	20.1 (15.8 to 24.9)
Cardiovascular diseases	Tunisia	2015	All Ages	2.6 (2.0 to 3.3)	14.2% (12.1 to 16.4%)	23.2 (18.0 to 29.2)
Cardiovascular diseases	Tunisia	1990	Age-standardized		12.8% (10.7 to 15.0%)	36.4 (30.0 to 43.3)
Cardiovascular diseases	Tunisia	1995	Age-standardized		12.6% (10.5 to 14.7%)	36.6 (30.2 to 43.6)
Cardiovascular diseases	Tunisia	2000	Age-standardized		12.7% (10.7 to 14.8%)	30.8 (25.2 to 36.9)
Cardiovascular diseases	Tunisia	2005	Age-standardized		11.8% (9.9 to 13.7%)	26.0 (20.6 to 31.8)
Cardiovascular diseases	Tunisia	2010	Age-standardized		12.7% (10.6 to 14.7%)	26.2 (20.4 to 32.9)

Cause name	Location	Year	Age group	Number (in thousands)	PAF	Rate (per 100,000)
Cardiovascular diseases	Tunisia	2015	Age-standardized		13.7% (11.5 to 16.0%)	28.0 (21.5 to 35.3)
Ischemic heart disease	Tunisia	1990	All Ages	0.8 (0.7 to 1.0)	18.9% (15.9 to 22.3%)	9.7 (7.9 to 11.9)
Ischemic heart disease	Tunisia	1995	All Ages	1.0 (0.8 to 1.2)	18.4% (15.3 to 21.7%)	10.8 (8.8 to 13.2)
Ischemic heart disease	Tunisia	2000	All Ages	1.0 (0.8 to 1.3)	18.3% (15.3 to 21.7%)	10.7 (8.7 to 13.2)
Ischemic heart disease	Tunisia	2005	All Ages	1.1 (0.8 to 1.4)	16.8% (13.8 to 20.0%)	10.6 (8.2 to 13.3)
Ischemic heart disease	Tunisia	2010	All Ages	1.3 (1.0 to 1.6)	17.7% (14.4 to 21.2%)	11.7 (8.9 to 15.2)
Ischemic heart disease	Tunisia	2015	All Ages	1.5 (1.1 to 2.0)	18.7% (15.3 to 22.7%)	13.6 (10.1 to 17.6)
Ischemic heart disease	Tunisia	1990	Age-standardized		17.5% (13.9 to 21.4%)	19.9 (15.4 to 24.8)
Ischemic heart disease	Tunisia	1995	Age-standardized		17.1% (13.5 to 20.9%)	20.1 (15.5 to 24.9)
Ischemic heart disease	Tunisia	2000	Age-standardized		17.3% (13.7 to 21.0%)	17.2 (13.5 to 21.4)
Ischemic heart disease	Tunisia	2005	Age-standardized		16.1% (12.7 to 19.4%)	14.9 (11.4 to 18.9)
Ischemic heart disease	Tunisia	2010	Age-standardized		17.0% (13.5 to 20.8%)	15.1 (11.4 to 19.8)
Ischemic heart disease	Tunisia	2015	Age-standardized		18.0% (14.4 to 22.2%)	16.2 (12.0 to 21.0)
Ischemic stroke	Tunisia	1990	All Ages	0.3 (0.2 to 0.4)	13.9% (10.5 to 17.0%)	3.9 (2.9 to 5.0)
Ischemic stroke	Tunisia	1995	All Ages	0.4 (0.3 to 0.5)	13.4% (10.0 to 16.7%)	4.5 (3.3 to 5.7)
Ischemic stroke	Tunisia	2000	All Ages	0.4 (0.3 to 0.5)	13.3% (10.0 to 16.5%)	4.2 (3.1 to 5.4)
Ischemic stroke	Tunisia	2005	All Ages	0.4 (0.3 to 0.5)	11.9% (8.6 to 15.3%)	4.1 (2.8 to 5.4)
Ischemic stroke	Tunisia	2010	All Ages	0.5 (0.3 to 0.7)	12.8% (9.3 to 16.3%)	4.5 (3.2 to 6.2)
Ischemic stroke	Tunisia	2015	All Ages	0.6 (0.4 to 0.8)	13.9% (10.3 to 17.7%)	5.3 (3.7 to 7.2)
Ischemic stroke	Tunisia	1990	Age-standardized		13.0% (9.3 to 16.6%)	9.3 (6.5 to 12.5)
Ischemic stroke	Tunisia	1995	Age-standardized		12.6% (9.0 to 16.5%)	9.4 (6.5 to 12.5)
Ischemic stroke	Tunisia	2000	Age-standardized		12.6% (8.9 to 16.3%)	7.6 (5.3 to 10.0)
Ischemic stroke	Tunisia	2005	Age-standardized		11.5% (7.9 to 15.1%)	6.2 (4.2 to 8.4)
Ischemic stroke	Tunisia	2010	Age-standardized		12.4% (8.7 to 16.3%)	6.3 (4.3 to 8.6)
Ischemic stroke	Tunisia	2015	Age-standardized		13.6% (9.8 to 17.6%)	6.7 (4.6 to 9.3)
Hemorrhagic stroke	Tunisia	1990	All Ages	0.3 (0.2 to 0.4)	14.0% (11.4 to 16.6%)	3.7 (2.9 to 4.7)
Hemorrhagic stroke	Tunisia	1995	All Ages	0.4 (0.3 to 0.5)	14.2% (11.6 to 16.7%)	4.0 (3.1 to 5.1)
Hemorrhagic stroke	Tunisia	2000	All Ages	0.4 (0.3 to 0.5)	14.5% (11.7 to 17.1%)	3.9 (3.0 to 4.9)
Hemorrhagic stroke	Tunisia	2005	All Ages	0.4 (0.3 to 0.5)	13.2% (10.5 to 15.9%)	3.6 (2.7 to 4.7)
Hemorrhagic stroke	Tunisia	2010	All Ages	0.4 (0.3 to 0.6)	14.3% (11.5 to 17.1%)	3.9 (2.8 to 5.1)
Hemorrhagic stroke	Tunisia	2015	All Ages	0.5 (0.4 to 0.7)	15.6% (12.6 to 18.5%)	4.4 (3.2 to 5.8)
Hemorrhagic stroke	Tunisia	1990	Age-standardized		13.9% (11.1 to 16.8%)	7.2 (5.3 to 9.2)
Hemorrhagic stroke	Tunisia	1995	Age-standardized		13.7% (10.7 to 16.7%)	7.1 (5.4 to 9.1)
Hemorrhagic stroke	Tunisia	2000	Age-standardized		13.9% (10.8 to 16.9%)	6.0 (4.4 to 7.6)
Hemorrhagic stroke	Tunisia	2005	Age-standardized		12.7% (9.7 to 15.7%)	4.9 (3.5 to 6.5)
Hemorrhagic stroke	Tunisia	2010	Age-standardized		13.7% (10.7 to 16.8%)	4.8 (3.5 to 6.5)

Cause name	Location	Year	Age group	Number (in thousands)	PAF	Rate (per 100,000)
Hemorrhagic stroke	Tunisia	2015	Age-standardized		15.0% (11.8 to 18.1%)	5.1 (3.7 to 6.9)
Chronic obstructive pulmonary disease	Tunisia	1990	All Ages	0.3 (0.2 to 0.5)	24.7% (14.2 to 35.9%)	3.7 (2.1 to 5.5)
Chronic obstructive pulmonary disease	Tunisia	1995	All Ages	0.4 (0.2 to 0.6)	24.5% (14.0 to 35.7%)	4.2 (2.3 to 6.4)
Chronic obstructive pulmonary disease	Tunisia	2000	All Ages	0.4 (0.2 to 0.6)	24.4% (13.7 to 35.5%)	3.9 (2.1 to 5.7)
Chronic obstructive pulmonary disease	Tunisia	2005	All Ages	0.4 (0.2 to 0.6)	21.6% (11.6 to 32.4%)	3.5 (1.8 to 5.4)
Chronic obstructive pulmonary disease	Tunisia	2010	All Ages	0.4 (0.2 to 0.7)	24.3% (13.8 to 35.3%)	3.9 (2.1 to 6.1)
Chronic obstructive pulmonary disease	Tunisia	2015	All Ages	0.5 (0.3 to 0.8)	27.3% (16.5 to 39.0%)	4.4 (2.4 to 6.8)
Chronic obstructive pulmonary disease	Tunisia	1990	Age-standardized		25.3% (14.6 to 36.6%)	8.6 (4.8 to 13.1)
Chronic obstructive pulmonary disease	Tunisia	1995	Age-standardized		24.8% (14.1 to 36.2%)	8.8 (4.7 to 13.6)
Chronic obstructive pulmonary disease	Tunisia	2000	Age-standardized		24.6% (13.8 to 35.8%)	6.8 (3.7 to 10.2)
Chronic obstructive pulmonary disease	Tunisia	2005	Age-standardized		21.8% (11.7 to 32.6%)	5.2 (2.7 to 8.2)
Chronic obstructive pulmonary disease	Tunisia	2010	Age-standardized		24.4% (13.8 to 35.5%)	5.3 (2.8 to 8.4)
Chronic obstructive pulmonary disease	Tunisia	2015	Age-standardized		27.3% (16.5 to 39.1%)	5.5 (3.1 to 8.5)
All causes	Turkey	1990	All Ages	31.9 (27.1 to 37.2)	7.9% (6.8 to 9.1%)	58.5 (49.6 to 68.1)
All causes	Turkey	1995	All Ages	30.5 (26.0 to 35.4)	7.7% (6.6 to 8.8%)	51.5 (44.0 to 59.8)
All causes	Turkey	2000	All Ages	27.7 (23.7 to 31.9)	7.8% (6.7 to 8.9%)	43.3 (37.1 to 50.1)
All causes	Turkey	2005	All Ages	23.6 (20.1 to 27.2)	7.4% (6.3 to 8.5%)	34.6 (29.5 to 39.9)
All causes	Turkey	2010	All Ages	21.9 (18.8 to 25.2)	7.6% (6.4 to 8.7%)	30.2 (25.9 to 34.7)
All causes	Turkey	2015	All Ages	26.7 (22.8 to 30.9)	8.0% (6.9 to 9.2%)	34.0 (29.0 to 39.4)
All causes	Turkey	1990	Age-standardized		8.7% (7.4 to 10.0%)	87.9 (74.5 to 102.8)
All causes	Turkey	1995	Age-standardized		8.2% (7.1 to 9.5%)	76.5 (64.9 to 89.0)
All causes	Turkey	2000	Age-standardized		8.2% (6.9 to 9.5%)	62.2 (52.7 to 72.1)
All causes	Turkey	2005	Age-standardized		7.6% (6.4 to 8.8%)	48.9 (41.5 to 56.9)
All causes	Turkey	2010	Age-standardized		7.7% (6.5 to 9.0%)	40.8 (34.5 to 47.0)
All causes	Turkey	2015	Age-standardized		8.0% (6.9 to 9.3%)	41.6 (35.4 to 48.5)
Lower respiratory infections	Turkey	1990	All Ages	6.3 (3.9 to 9.2)	24.1% (15.9 to 33.1%)	11.5 (7.2 to 16.8)
Lower respiratory infections	Turkey	1995	All Ages	4.6 (2.7 to 7.0)	22.3% (13.9 to 32.0%)	7.7 (4.6 to 11.8)
Lower respiratory infections	Turkey	2000	All Ages	3.2 (1.9 to 4.9)	22.8% (14.4 to 32.3%)	5.0 (3.0 to 7.7)
Lower respiratory infections	Turkey	2005	All Ages	2.2 (1.3 to 3.4)	21.4% (13.0 to 31.2%)	3.2 (1.9 to 5.0)
Lower respiratory infections	Turkey	2010	All Ages	1.8 (1.2 to 2.6)	24.0% (15.9 to 33.0%)	2.5 (1.6 to 3.6)
Lower respiratory infections	Turkey	2015	All Ages	2.1 (1.4 to 2.9)	25.8% (17.9 to 34.0%)	2.6 (1.8 to 3.7)
Lower respiratory infections	Turkey	1990	Age-standardized		24.1% (15.9 to 33.1%)	10.4 (6.4 to 15.9)
Lower respiratory infections	Turkey	1995	Age-standardized		22.3% (13.9 to 32.0%)	7.8 (4.5 to 12.5)
Lower respiratory infections	Turkey	2000	Age-standardized		22.8% (14.4 to 32.3%)	5.6 (3.3 to 8.9)
Lower respiratory infections	Turkey	2005	Age-standardized		21.4% (13.0 to 31.2%)	4.0 (2.3 to 6.3)
Lower respiratory infections	Turkey	2010	Age-standardized		24.0% (15.9 to 33.0%)	3.3 (2.1 to 4.7)

Cause name	Location	Year	Age group	Number (in thousands)	PAF	Rate (per 100,000)
Lower respiratory infections	Turkey	2015	Age-standardized		25.8% (17.9 to 34.0%)	3.3 (2.2 to 4.6)
Tracheal, bronchus, and lung cancer	Turkey	1990	All Ages	2.7 (1.6 to 3.9)	17.1% (10.3 to 24.6%)	4.9 (2.9 to 7.1)
Tracheal, bronchus, and lung cancer	Turkey	1995	All Ages	2.7 (1.6 to 4.0)	15.8% (9.3 to 23.0%)	4.6 (2.6 to 6.8)
Tracheal, bronchus, and lung cancer	Turkey	2000	All Ages	2.9 (1.7 to 4.3)	16.2% (9.7 to 23.5%)	4.5 (2.7 to 6.7)
Tracheal, bronchus, and lung cancer	Turkey	2005	All Ages	2.8 (1.6 to 4.1)	15.2% (8.8 to 22.2%)	4.1 (2.4 to 6.0)
Tracheal, bronchus, and lung cancer	Turkey	2010	All Ages	3.0 (1.8 to 4.4)	17.1% (10.1 to 24.6%)	4.2 (2.5 to 6.0)
Tracheal, bronchus, and lung cancer	Turkey	2015	All Ages	3.7 (2.3 to 5.4)	18.5% (11.4 to 26.2%)	4.8 (2.9 to 6.8)
Tracheal, bronchus, and lung cancer	Turkey	1990	Age-standardized		17.2% (10.3 to 24.6%)	7.9 (4.6 to 11.5)
Tracheal, bronchus, and lung cancer	Turkey	1995	Age-standardized		15.8% (9.3 to 23.1%)	7.0 (4.0 to 10.4)
Tracheal, bronchus, and lung cancer	Turkey	2000	Age-standardized		16.2% (9.7 to 23.5%)	6.4 (3.8 to 9.5)
Tracheal, bronchus, and lung cancer	Turkey	2005	Age-standardized		15.2% (8.8 to 22.2%)	5.6 (3.2 to 8.1)
Tracheal, bronchus, and lung cancer	Turkey	2010	Age-standardized		17.1% (10.2 to 24.6%)	5.3 (3.1 to 7.6)
Tracheal, bronchus, and lung cancer	Turkey	2015	Age-standardized		18.5% (11.4 to 26.2%)	5.6 (3.4 to 8.0)
Cardiovascular diseases	Turkey	1990	All Ages	18.1 (15.2 to 21.6)	14.6% (12.5 to 17.0%)	33.2 (27.7 to 39.5)
Cardiovascular diseases	Turkey	1995	All Ages	18.3 (15.3 to 21.7)	14.1% (12.0 to 16.6%)	31.0 (25.8 to 36.7)
Cardiovascular diseases	Turkey	2000	All Ages	16.9 (14.2 to 19.8)	14.0% (11.9 to 16.3%)	26.4 (22.2 to 31.0)
Cardiovascular diseases	Turkey	2005	All Ages	14.3 (12.1 to 16.7)	13.2% (11.3 to 15.3%)	20.9 (17.7 to 24.5)
Cardiovascular diseases	Turkey	2010	All Ages	12.8 (11.0 to 15.0)	13.5% (11.6 to 15.6%)	17.7 (15.1 to 20.7)
Cardiovascular diseases	Turkey	2015	All Ages	15.8 (13.2 to 18.8)	13.7% (11.7 to 16.0%)	20.1 (16.8 to 23.9)
Cardiovascular diseases	Turkey	1990	Age-standardized		14.4% (12.1 to 16.9%)	54.3 (45.0 to 64.6)
Cardiovascular diseases	Turkey	1995	Age-standardized		13.8% (11.7 to 16.3%)	48.1 (39.7 to 57.5)
Cardiovascular diseases	Turkey	2000	Age-standardized		13.5% (11.3 to 15.9%)	38.8 (31.9 to 45.9)
Cardiovascular diseases	Turkey	2005	Age-standardized		12.6% (10.6 to 14.9%)	29.7 (24.6 to 35.4)
Cardiovascular diseases	Turkey	2010	Age-standardized		12.9% (10.8 to 15.3%)	23.9 (19.8 to 28.4)
Cardiovascular diseases	Turkey	2015	Age-standardized		13.3% (11.1 to 15.6%)	24.5 (20.1 to 29.5)
Ischemic heart disease	Turkey	1990	All Ages	15.1 (12.3 to 18.4)	18.3% (15.2 to 21.6%)	27.6 (22.6 to 33.6)
Ischemic heart disease	Turkey	1995	All Ages	15.3 (12.4 to 18.4)	17.7% (14.6 to 20.8%)	25.9 (20.9 to 31.0)
Ischemic heart disease	Turkey	2000	All Ages	13.8 (11.3 to 16.6)	17.6% (14.6 to 20.7%)	21.7 (17.7 to 26.0)
Ischemic heart disease	Turkey	2005	All Ages	11.0 (9.0 to 13.2)	17.1% (14.2 to 20.3%)	16.1 (13.2 to 19.4)
Ischemic heart disease	Turkey	2010	All Ages	9.2 (7.5 to 11.1)	17.7% (14.6 to 21.1%)	12.7 (10.4 to 15.4)
Ischemic heart disease	Turkey	2015	All Ages	11.3 (9.1 to 13.8)	18.0% (14.7 to 21.7%)	14.4 (11.6 to 17.5)
Ischemic heart disease	Turkey	1990	Age-standardized		17.8% (14.6 to 21.2%)	45.0 (36.3 to 55.3)
Ischemic heart disease	Turkey	1995	Age-standardized		17.2% (14.0 to 20.4%)	40.1 (32.0 to 48.5)
Ischemic heart disease	Turkey	2000	Age-standardized		17.0% (13.7 to 20.4%)	31.6 (25.2 to 38.3)
Ischemic heart disease	Turkey	2005	Age-standardized		16.4% (13.1 to 19.8%)	22.7 (17.9 to 27.6)
Ischemic heart disease	Turkey	2010	Age-standardized		16.9% (13.5 to 20.5%)	17.1 (13.4 to 20.9)

Cause name	Location	Year	Age group	Number (in thousands)	PAF	Rate (per 100,000)
Ischemic heart disease	Turkey	2015	Age-standardized		17.5% (14.0 to 21.3%)	17.4 (13.6 to 21.7)
Ischemic stroke	Turkey	1990	All Ages	1.6 (1.1 to 2.1)	12.4% (9.0 to 16.1%)	2.8 (2.0 to 3.8)
Ischemic stroke	Turkey	1995	All Ages	1.6 (1.1 to 2.2)	11.9% (8.4 to 15.6%)	2.6 (1.8 to 3.7)
Ischemic stroke	Turkey	2000	All Ages	1.6 (1.1 to 2.1)	12.0% (8.5 to 15.6%)	2.5 (1.7 to 3.4)
Ischemic stroke	Turkey	2005	All Ages	1.7 (1.2 to 2.2)	11.7% (8.3 to 15.2%)	2.4 (1.7 to 3.3)
Ischemic stroke	Turkey	2010	All Ages	1.8 (1.3 to 2.4)	12.5% (9.0 to 16.0%)	2.5 (1.8 to 3.4)
Ischemic stroke	Turkey	2015	All Ages	2.3 (1.7 to 3.1)	12.9% (9.3 to 16.6%)	3.0 (2.1 to 4.0)
Ischemic stroke	Turkey	1990	Age-standardized		12.3% (8.7 to 16.2%)	4.9 (3.4 to 6.7)
Ischemic stroke	Turkey	1995	Age-standardized		11.7% (8.1 to 15.6%)	4.3 (2.9 to 6.1)
Ischemic stroke	Turkey	2000	Age-standardized		11.7% (8.0 to 15.7%)	3.9 (2.6 to 5.5)
Ischemic stroke	Turkey	2005	Age-standardized		11.3% (7.8 to 15.3%)	3.8 (2.5 to 5.3)
Ischemic stroke	Turkey	2010	Age-standardized		12.1% (8.4 to 16.0%)	3.7 (2.5 to 5.0)
Ischemic stroke	Turkey	2015	Age-standardized		12.6% (8.9 to 16.5%)	3.8 (2.6 to 5.2)
Hemorrhagic stroke	Turkey	1990	All Ages	1.5 (1.1 to 2.0)	13.0% (10.5 to 15.6%)	2.7 (2.1 to 3.7)
Hemorrhagic stroke	Turkey	1995	All Ages	1.5 (1.1 to 1.9)	12.7% (10.1 to 15.3%)	2.5 (1.8 to 3.3)
Hemorrhagic stroke	Turkey	2000	All Ages	1.4 (1.1 to 1.9)	13.0% (10.4 to 15.7%)	2.3 (1.7 to 2.9)
Hemorrhagic stroke	Turkey	2005	All Ages	1.6 (1.2 to 2.0)	12.9% (10.4 to 15.7%)	2.3 (1.8 to 2.9)
Hemorrhagic stroke	Turkey	2010	All Ages	1.8 (1.4 to 2.1)	13.9% (11.2 to 16.8%)	2.4 (1.9 to 3.0)
Hemorrhagic stroke	Turkey	2015	All Ages	2.1 (1.7 to 2.6)	14.6% (11.7 to 17.4%)	2.7 (2.1 to 3.4)
Hemorrhagic stroke	Turkey	1990	Age-standardized		13.5% (10.6 to 16.3%)	4.3 (3.2 to 5.8)
Hemorrhagic stroke	Turkey	1995	Age-standardized		12.9% (10.1 to 15.9%)	3.7 (2.8 to 5.0)
Hemorrhagic stroke	Turkey	2000	Age-standardized		12.9% (10.0 to 16.0%)	3.3 (2.4 to 4.3)
Hemorrhagic stroke	Turkey	2005	Age-standardized		12.6% (9.7 to 15.5%)	3.2 (2.4 to 4.1)
Hemorrhagic stroke	Turkey	2010	Age-standardized		13.5% (10.6 to 16.5%)	3.2 (2.4 to 3.9)
Hemorrhagic stroke	Turkey	2015	Age-standardized		14.1% (11.1 to 17.2%)	3.2 (2.5 to 4.1)
Chronic obstructive pulmonary disease	Turkey	1990	All Ages	4.9 (2.6 to 7.3)	22.4% (12.2 to 32.9%)	8.9 (4.8 to 13.4)
Chronic obstructive pulmonary disease	Turkey	1995	All Ages	4.9 (2.5 to 7.3)	21.7% (11.4 to 32.3%)	8.2 (4.3 to 12.3)
Chronic obstructive pulmonary disease	Turkey	2000	All Ages	4.7 (2.5 to 7.0)	22.4% (12.0 to 33.3%)	7.4 (3.9 to 11.0)
Chronic obstructive pulmonary disease	Turkey	2005	All Ages	4.4 (2.3 to 6.6)	21.6% (11.2 to 32.3%)	6.4 (3.3 to 9.6)
Chronic obstructive pulmonary disease	Turkey	2010	All Ages	4.3 (2.3 to 6.3)	23.6% (12.9 to 34.8%)	5.9 (3.2 to 8.7)
Chronic obstructive pulmonary disease	Turkey	2015	All Ages	5.1 (2.9 to 7.6)	25.1% (14.2 to 36.3%)	6.5 (3.7 to 9.7)
Chronic obstructive pulmonary disease	Turkey	1990	Age-standardized		23.2% (12.6 to 34.2%)	15.3 (8.3 to 23.0)
Chronic obstructive pulmonary disease	Turkey	1995	Age-standardized		22.1% (11.6 to 33.0%)	13.5 (7.0 to 20.3)
Chronic obstructive pulmonary disease	Turkey	2000	Age-standardized		22.6% (12.1 to 33.7%)	11.4 (6.0 to 17.1)
Chronic obstructive pulmonary disease	Turkey	2005	Age-standardized		21.7% (11.3 to 32.6%)	9.6 (5.0 to 14.6)
Chronic obstructive pulmonary disease	Turkey	2010	Age-standardized		23.7% (12.9 to 34.9%)	8.4 (4.6 to 12.4)

Cause name	Location	Year	Age group	Number (in thousands)	PAF	Rate (per 100,000)
Chronic obstructive pulmonary disease	Turkey	2015	Age-standardized		25.1% (14.2 to 36.4%)	8.3 (4.7 to 12.3)
All causes	United Arab Emirates	1990	All Ages	0.5 (0.4 to 0.7)	8.6% (7.2 to 10.7%)	28.8 (22.1 to 37.9)
All causes	United Arab Emirates	1995	All Ages	0.6 (0.5 to 0.8)	9.3% (7.7 to 11.5%)	27.1 (21.7 to 34.4)
All causes	United Arab Emirates	2000	All Ages	0.7 (0.6 to 0.9)	9.2% (7.7 to 11.4%)	23.5 (19.2 to 29.5)
All causes	United Arab Emirates	2005	All Ages	0.8 (0.7 to 1.1)	8.6% (7.2 to 10.5%)	19.0 (15.7 to 23.5)
All causes	United Arab Emirates	2010	All Ages	1.7 (1.3 to 2.2)	9.6% (8.3 to 11.4%)	20.5 (16.1 to 26.2)
All causes	United Arab Emirates	2015	All Ages	2.6 (1.9 to 3.5)	10.7% (9.1 to 12.4%)	28.0 (20.3 to 38.3)
All causes	United Arab Emirates	1990	Age-standardized		11.6% (9.9 to 13.6%)	113.6 (88.7 to 139.6)
All causes	United Arab Emirates	1995	Age-standardized		11.8% (9.9 to 13.7%)	108.6 (89.0 to 129.2)
All causes	United Arab Emirates	2000	Age-standardized		11.6% (9.6 to 13.6%)	102.8 (84.5 to 121.7)
All causes	United Arab Emirates	2005	Age-standardized		10.8% (9.0 to 12.7%)	82.7 (67.7 to 100.0)
All causes	United Arab Emirates	2010	Age-standardized		11.4% (9.7 to 13.2%)	86.3 (71.6 to 104.4)
All causes	United Arab Emirates	2015	Age-standardized		11.2% (9.6 to 12.9%)	84.7 (66.9 to 107.8)
Lower respiratory infections	United Arab Emirates	1990	All Ages	0.1 (0.0 to 0.1)	38.3% (29.9 to 46.0%)	3.5 (2.3 to 4.8)
Lower respiratory infections	United Arab Emirates	1995	All Ages	0.1 (0.1 to 0.1)	38.0% (29.7 to 45.6%)	2.9 (1.9 to 3.9)
Lower respiratory infections	United Arab Emirates	2000	All Ages	0.1 (0.1 to 0.1)	38.2% (29.9 to 46.1%)	2.5 (1.7 to 3.4)
Lower respiratory infections	United Arab Emirates	2005	All Ages	0.1 (0.1 to 0.1)	36.2% (28.1 to 43.6%)	1.9 (1.3 to 2.5)
Lower respiratory infections	United Arab Emirates	2010	All Ages	0.1 (0.1 to 0.2)	37.9% (29.7 to 45.7%)	1.5 (1.1 to 2.0)
Lower respiratory infections	United Arab Emirates	2015	All Ages	0.2 (0.1 to 0.2)	35.4% (27.5 to 42.9%)	1.8 (1.2 to 2.5)
Lower respiratory infections	United Arab Emirates	1990	Age-standardized		38.3% (29.9 to 46.0%)	19.5 (9.2 to 29.8)
Lower respiratory infections	United Arab Emirates	1995	Age-standardized		38.0% (29.7 to 45.6%)	17.9 (8.4 to 26.8)
Lower respiratory infections	United Arab Emirates	2000	Age-standardized		38.2% (29.9 to 46.1%)	18.1 (8.2 to 27.7)
Lower respiratory infections	United Arab Emirates	2005	Age-standardized		36.2% (28.1 to 43.6%)	14.9 (6.5 to 22.0)
Lower respiratory infections	United Arab Emirates	2010	Age-standardized		37.9% (29.7 to 45.7%)	14.0 (6.2 to 21.0)
Lower respiratory infections	United Arab Emirates	2015	Age-standardized		35.4% (27.5 to 42.9%)	12.7 (5.3 to 19.5)
Tracheal, bronchus, and lung cancer	United Arab Emirates	1990	All Ages	0.0 (0.0 to 0.0)	29.4% (20.5 to 37.9%)	0.9 (0.6 to 1.2)
Tracheal, bronchus, and lung cancer	United Arab Emirates	1995	All Ages	0.0 (0.0 to 0.0)	29.1% (20.2 to 37.6%)	0.9 (0.6 to 1.2)
Tracheal, bronchus, and lung cancer	United Arab Emirates	2000	All Ages	0.0 (0.0 to 0.0)	29.4% (20.5 to 38.0%)	0.9 (0.6 to 1.2)
Tracheal, bronchus, and lung cancer	United Arab Emirates	2005	All Ages	0.0 (0.0 to 0.1)	27.4% (18.7 to 35.8%)	0.7 (0.5 to 1.0)
Tracheal, bronchus, and lung cancer	United Arab Emirates	2010	All Ages	0.1 (0.0 to 0.1)	29.0% (20.1 to 37.6%)	0.8 (0.5 to 1.1)
Tracheal, bronchus, and lung cancer	United Arab Emirates	2015	All Ages	0.1 (0.1 to 0.1)	26.7% (18.2 to 35.0%)	1.0 (0.6 to 1.5)
Tracheal, bronchus, and lung cancer	United Arab Emirates	1990	Age-standardized		29.5% (20.6 to 38.0%)	4.2 (2.7 to 5.9)
Tracheal, bronchus, and lung cancer	United Arab Emirates	1995	Age-standardized		29.2% (20.3 to 37.7%)	4.5 (3.0 to 6.1)
Tracheal, bronchus, and lung cancer	United Arab Emirates	2000	Age-standardized		29.5% (20.6 to 38.1%)	4.8 (3.2 to 6.4)
Tracheal, bronchus, and lung cancer	United Arab Emirates	2005	Age-standardized		27.5% (18.8 to 36.0%)	4.1 (2.7 to 5.5)
Tracheal, bronchus, and lung cancer	United Arab Emirates	2010	Age-standardized		29.1% (20.2 to 37.8%)	4.5 (3.0 to 6.1)

Cause name	Location	Year	Age group	Number (in thousands)	PAF	Rate (per 100,000)
Tracheal, bronchus, and lung cancer	United Arab Emirates	2015	Age-standardized		26.8% (18.2 to 35.1%)	4.1 (2.6 to 6.1)
Cardiovascular diseases	United Arab Emirates	1990	All Ages	0.4 (0.3 to 0.5)	23.1% (20.6 to 25.7%)	21.1 (15.5 to 30.1)
Cardiovascular diseases	United Arab Emirates	1995	All Ages	0.5 (0.4 to 0.7)	23.4% (20.9 to 26.0%)	20.2 (15.6 to 27.8)
Cardiovascular diseases	United Arab Emirates	2000	All Ages	0.5 (0.4 to 0.7)	23.4% (20.7 to 26.0%)	17.4 (13.6 to 23.7)
Cardiovascular diseases	United Arab Emirates	2005	All Ages	0.6 (0.5 to 0.8)	22.9% (20.2 to 25.5%)	14.2 (11.2 to 18.7)
Cardiovascular diseases	United Arab Emirates	2010	All Ages	1.3 (1.0 to 1.8)	24.8% (21.8 to 27.5%)	16.0 (12.2 to 21.1)
Cardiovascular diseases	United Arab Emirates	2015	All Ages	2.1 (1.5 to 2.9)	24.0% (21.0 to 26.8%)	22.5 (15.9 to 31.5)
Cardiovascular diseases	United Arab Emirates	1990	Age-standardized		18.9% (16.6 to 21.7%)	76.9 (58.9 to 100.2)
Cardiovascular diseases	United Arab Emirates	1995	Age-standardized		18.9% (16.5 to 21.6%)	74.4 (60.3 to 93.6)
Cardiovascular diseases	United Arab Emirates	2000	Age-standardized		18.5% (16.0 to 21.5%)	68.6 (55.4 to 85.4)
Cardiovascular diseases	United Arab Emirates	2005	Age-standardized		17.8% (15.5 to 20.7%)	54.9 (44.5 to 68.1)
Cardiovascular diseases	United Arab Emirates	2010	Age-standardized		18.4% (15.9 to 21.4%)	59.1 (48.1 to 72.8)
Cardiovascular diseases	United Arab Emirates	2015	Age-standardized		17.9% (15.6 to 20.8%)	59.8 (46.9 to 76.7)
Ischemic heart disease	United Arab Emirates	1990	All Ages	0.3 (0.2 to 0.4)	28.0% (24.1 to 32.0%)	14.2 (10.1 to 20.8)
Ischemic heart disease	United Arab Emirates	1995	All Ages	0.3 (0.2 to 0.5)	28.2% (24.3 to 32.2%)	13.8 (10.3 to 19.2)
Ischemic heart disease	United Arab Emirates	2000	All Ages	0.4 (0.3 to 0.5)	28.2% (24.4 to 32.2%)	11.9 (9.1 to 16.4)
Ischemic heart disease	United Arab Emirates	2005	All Ages	0.4 (0.3 to 0.6)	27.9% (23.9 to 31.9%)	9.9 (7.6 to 13.2)
Ischemic heart disease	United Arab Emirates	2010	All Ages	1.0 (0.7 to 1.3)	29.9% (25.5 to 34.4%)	11.4 (8.4 to 15.3)
Ischemic heart disease	United Arab Emirates	2015	All Ages	1.5 (1.0 to 2.1)	28.6% (24.3 to 33.0%)	16.3 (11.2 to 23.0)
Ischemic heart disease	United Arab Emirates	1990	Age-standardized		22.3% (18.7 to 26.5%)	52.8 (39.5 to 70.8)
Ischemic heart disease	United Arab Emirates	1995	Age-standardized		22.2% (18.6 to 26.5%)	51.0 (39.9 to 66.6)
Ischemic heart disease	United Arab Emirates	2000	Age-standardized		21.8% (18.1 to 26.2%)	46.6 (35.9 to 59.6)
Ischemic heart disease	United Arab Emirates	2005	Age-standardized		21.1% (17.5 to 25.7%)	37.4 (29.4 to 48.2)
Ischemic heart disease	United Arab Emirates	2010	Age-standardized		21.8% (18.1 to 26.3%)	40.6 (31.8 to 51.7)
Ischemic heart disease	United Arab Emirates	2015	Age-standardized		21.2% (17.7 to 25.5%)	41.4 (31.2 to 54.3)
Ischemic stroke	United Arab Emirates	1990	All Ages	0.0 (0.0 to 0.1)	22.3% (19.1 to 25.5%)	2.4 (1.7 to 3.3)
Ischemic stroke	United Arab Emirates	1995	All Ages	0.1 (0.0 to 0.1)	22.0% (18.8 to 25.2%)	2.2 (1.6 to 2.9)
Ischemic stroke	United Arab Emirates	2000	All Ages	0.1 (0.0 to 0.1)	21.7% (18.7 to 24.8%)	1.9 (1.4 to 2.5)
Ischemic stroke	United Arab Emirates	2005	All Ages	0.1 (0.1 to 0.1)	21.2% (18.3 to 24.3%)	1.5 (1.1 to 1.9)
Ischemic stroke	United Arab Emirates	2010	All Ages	0.1 (0.1 to 0.2)	23.2% (19.9 to 26.6%)	1.5 (1.1 to 2.0)
Ischemic stroke	United Arab Emirates	2015	All Ages	0.2 (0.1 to 0.3)	22.0% (18.7 to 25.3%)	2.1 (1.0 to 3.0)
Ischemic stroke	United Arab Emirates	1990	Age-standardized		17.7% (14.0 to 21.3%)	12.1 (8.7 to 16.1)
Ischemic stroke	United Arab Emirates	1995	Age-standardized		17.5% (13.9 to 21.2%)	11.6 (8.5 to 15.2)
Ischemic stroke	United Arab Emirates	2000	Age-standardized		17.1% (13.3 to 21.2%)	11.2 (7.9 to 15.0)
Ischemic stroke	United Arab Emirates	2005	Age-standardized		16.4% (12.6 to 20.4%)	9.1 (6.5 to 12.2)
Ischemic stroke	United Arab Emirates	2010	Age-standardized		16.9% (13.0 to 21.1%)	9.9 (7.1 to 13.1)

Cause name	Location	Year	Age group	Number (in thousands)	PAF	Rate (per 100,000)
Ischemic stroke	United Arab Emirates	2015	Age-standardized		16.2% (12.4 to 20.2%)	9.3 (5.3 to 13.1)
Hemorrhagic stroke	United Arab Emirates	1990	All Ages	0.1 (0.1 to 0.1)	25.1% (21.4 to 29.0%)	4.4 (3.1 to 6.5)
Hemorrhagic stroke	United Arab Emirates	1995	All Ages	0.1 (0.1 to 0.1)	25.3% (21.6 to 29.1%)	4.3 (3.1 to 6.2)
Hemorrhagic stroke	United Arab Emirates	2000	All Ages	0.1 (0.1 to 0.2)	25.2% (21.5 to 29.1%)	3.6 (2.7 to 5.2)
Hemorrhagic stroke	United Arab Emirates	2005	All Ages	0.1 (0.1 to 0.2)	24.6% (20.9 to 28.3%)	2.8 (2.1 to 4.0)
Hemorrhagic stroke	United Arab Emirates	2010	All Ages	0.3 (0.2 to 0.4)	26.3% (22.1 to 30.5%)	3.1 (2.2 to 4.3)
Hemorrhagic stroke	United Arab Emirates	2015	All Ages	0.4 (0.3 to 0.6)	24.7% (20.7 to 28.7%)	4.2 (2.8 to 6.1)
Hemorrhagic stroke	United Arab Emirates	1990	Age-standardized		19.9% (16.7 to 22.8%)	12.0 (8.7 to 16.1)
Hemorrhagic stroke	United Arab Emirates	1995	Age-standardized		19.7% (16.5 to 22.7%)	11.7 (8.9 to 15.5)
Hemorrhagic stroke	United Arab Emirates	2000	Age-standardized		19.3% (15.9 to 22.6%)	10.9 (8.4 to 14.3)
Hemorrhagic stroke	United Arab Emirates	2005	Age-standardized		18.4% (15.2 to 21.7%)	8.3 (6.4 to 11.0)
Hemorrhagic stroke	United Arab Emirates	2010	Age-standardized		19.0% (15.7 to 22.3%)	8.6 (6.5 to 11.6)
Hemorrhagic stroke	United Arab Emirates	2015	Age-standardized		18.2% (15.0 to 21.4%)	9.1 (6.2 to 13.7)
Chronic obstructive pulmonary disease	United Arab Emirates	1990	All Ages	0.1 (0.0 to 0.1)	33.1% (21.8 to 46.8%)	3.3 (1.9 to 5.2)
Chronic obstructive pulmonary disease	United Arab Emirates	1995	All Ages	0.1 (0.0 to 0.1)	33.4% (22.0 to 46.8%)	3.1 (1.9 to 4.5)
Chronic obstructive pulmonary disease	United Arab Emirates	2000	All Ages	0.1 (0.1 to 0.1)	33.7% (22.2 to 47.6%)	2.7 (1.7 to 3.9)
Chronic obstructive pulmonary disease	United Arab Emirates	2005	All Ages	0.1 (0.1 to 0.1)	32.1% (20.7 to 45.2%)	2.2 (1.3 to 3.1)
Chronic obstructive pulmonary disease	United Arab Emirates	2010	All Ages	0.2 (0.1 to 0.3)	33.7% (22.1 to 47.5%)	2.2 (1.3 to 3.3)
Chronic obstructive pulmonary disease	United Arab Emirates	2015	All Ages	0.3 (0.1 to 0.4)	32.2% (20.7 to 45.5%)	2.7 (1.5 to 4.3)
Chronic obstructive pulmonary disease	United Arab Emirates	1990	Age-standardized		34.6% (22.9 to 48.8%)	13.0 (7.6 to 20.0)
Chronic obstructive pulmonary disease	United Arab Emirates	1995	Age-standardized		34.5% (22.7 to 48.4%)	11.9 (7.2 to 17.6)
Chronic obstructive pulmonary disease	United Arab Emirates	2000	Age-standardized		34.7% (23.0 to 49.2%)	11.3 (7.1 to 16.4)
Chronic obstructive pulmonary disease	United Arab Emirates	2005	Age-standardized		33.1% (21.5 to 46.7%)	8.9 (5.5 to 13.0)
Chronic obstructive pulmonary disease	United Arab Emirates	2010	Age-standardized		34.5% (22.8 to 48.7%)	8.7 (5.6 to 12.9)
Chronic obstructive pulmonary disease	United Arab Emirates	2015	Age-standardized		32.5% (20.9 to 46.0%)	8.1 (5.0 to 12.0)
All causes	Yemen	1990	All Ages	10.0 (6.5 to 13.7)	7.4% (5.3 to 9.4%)	83.6 (54.7 to 114.8)
All causes	Yemen	1995	All Ages	12.0 (7.9 to 16.2)	7.8% (5.9 to 9.5%)	78.6 (52.2 to 106.5)
All causes	Yemen	2000	All Ages	11.2 (7.2 to 16.1)	7.8% (6.0 to 9.4%)	63.0 (40.5 to 90.3)
All causes	Yemen	2005	All Ages	10.0 (6.2 to 14.9)	7.2% (5.8 to 8.5%)	48.6 (30.0 to 72.7)
All causes	Yemen	2010	All Ages	11.0 (6.9 to 17.0)	8.1% (6.9 to 9.4%)	46.6 (29.0 to 71.9)
All causes	Yemen	2015	All Ages	12.5 (7.7 to 19.2)	7.7% (6.4 to 9.2%)	46.5 (28.7 to 71.4)
All causes	Yemen	1990	Age-standardized		10.6% (9.2 to 12.2%)	167.4 (112.1 to 233.9)
All causes	Yemen	1995	Age-standardized		10.7% (9.3 to 12.1%)	158.9 (106.1 to 228.9)
All causes	Yemen	2000	Age-standardized		10.7% (9.3 to 12.2%)	153.2 (99.5 to 222.7)
All causes	Yemen	2005	Age-standardized		9.8% (8.5 to 11.2%)	130.3 (81.4 to 194.2)
All causes	Yemen	2010	Age-standardized		10.5% (9.2 to 11.9%)	128.4 (80.6 to 195.6)

Cause name	Location	Year	Age group	Number (in thousands)	PAF	Rate (per 100,000)
All causes	Yemen	2015	Age-standardized		10.3% (8.9 to 11.8%)	128.0 (82.1 to 192.5)
Lower respiratory infections	Yemen	1990	All Ages	5.1 (2.7 to 7.6)	30.9% (23.6 to 38.1%)	43.0 (22.6 to 63.6)
Lower respiratory infections	Yemen	1995	All Ages	4.7 (2.3 to 6.9)	30.8% (23.6 to 37.8%)	30.8 (15.2 to 45.2)
Lower respiratory infections	Yemen	2000	All Ages	3.6 (1.8 to 5.3)	31.0% (23.7 to 38.0%)	20.0 (10.0 to 29.7)
Lower respiratory infections	Yemen	2005	All Ages	2.6 (1.4 to 3.9)	27.2% (20.0 to 34.4%)	12.6 (6.9 to 18.8)
Lower respiratory infections	Yemen	2010	All Ages	2.6 (1.7 to 3.7)	29.7% (22.5 to 36.9%)	10.9 (7.2 to 15.5)
Lower respiratory infections	Yemen	2015	All Ages	2.4 (1.6 to 3.5)	30.3% (22.8 to 37.4%)	9.0 (5.9 to 13.1)
Lower respiratory infections	Yemen	1990	Age-standardized		30.9% (23.6 to 38.1%)	32.5 (19.9 to 48.9)
Lower respiratory infections	Yemen	1995	Age-standardized		30.8% (23.6 to 37.8%)	27.6 (16.6 to 39.0)
Lower respiratory infections	Yemen	2000	Age-standardized		31.0% (23.7 to 38.0%)	24.4 (15.2 to 35.1)
Lower respiratory infections	Yemen	2005	Age-standardized		27.2% (20.0 to 34.4%)	19.1 (11.5 to 28.8)
Lower respiratory infections	Yemen	2010	Age-standardized		29.7% (22.5 to 36.9%)	18.7 (11.6 to 28.8)
Lower respiratory infections	Yemen	2015	Age-standardized		30.3% (22.8 to 37.4%)	17.5 (10.8 to 27.5)
Tracheal, bronchus, and lung cancer	Yemen	1990	All Ages	0.1 (0.0 to 0.2)	23.1% (15.1 to 31.0%)	0.8 (0.4 to 1.4)
Tracheal, bronchus, and lung cancer	Yemen	1995	All Ages	0.2 (0.1 to 0.3)	23.1% (15.5 to 31.1%)	1.1 (0.5 to 1.9)
Tracheal, bronchus, and lung cancer	Yemen	2000	All Ages	0.2 (0.1 to 0.3)	23.3% (15.1 to 31.3%)	1.1 (0.5 to 1.9)
Tracheal, bronchus, and lung cancer	Yemen	2005	All Ages	0.2 (0.1 to 0.3)	19.9% (12.7 to 27.8%)	0.9 (0.4 to 1.6)
Tracheal, bronchus, and lung cancer	Yemen	2010	All Ages	0.2 (0.1 to 0.4)	22.1% (14.2 to 30.0%)	1.0 (0.5 to 1.8)
Tracheal, bronchus, and lung cancer	Yemen	2015	All Ages	0.3 (0.1 to 0.6)	22.6% (14.8 to 30.6%)	1.1 (0.6 to 2.0)
Tracheal, bronchus, and lung cancer	Yemen	1990	Age-standardized		23.2% (15.2 to 31.2%)	2.4 (1.2 to 4.3)
Tracheal, bronchus, and lung cancer	Yemen	1995	Age-standardized		23.2% (15.6 to 31.3%)	2.9 (1.5 to 5.1)
Tracheal, bronchus, and lung cancer	Yemen	2000	Age-standardized		23.4% (15.2 to 31.6%)	3.1 (1.6 to 5.3)
Tracheal, bronchus, and lung cancer	Yemen	2005	Age-standardized		20.1% (12.8 to 28.0%)	2.7 (1.4 to 4.7)
Tracheal, bronchus, and lung cancer	Yemen	2010	Age-standardized		22.3% (14.3 to 30.3%)	3.0 (1.6 to 5.3)
Tracheal, bronchus, and lung cancer	Yemen	2015	Age-standardized		22.8% (14.9 to 30.8%)	3.1 (1.7 to 5.5)
Cardiovascular diseases	Yemen	1990	All Ages	4.3 (2.5 to 6.4)	16.6% (14.2 to 19.0%)	35.5 (21.3 to 53.5)
Cardiovascular diseases	Yemen	1995	All Ages	6.3 (3.9 to 9.7)	16.7% (14.5 to 19.1%)	41.6 (25.5 to 63.6)
Cardiovascular diseases	Yemen	2000	All Ages	6.7 (4.0 to 10.4)	17.1% (15.0 to 19.5%)	37.4 (22.6 to 58.4)
Cardiovascular diseases	Yemen	2005	All Ages	6.5 (3.6 to 10.3)	16.1% (14.0 to 18.4%)	31.5 (17.6 to 49.9)
Cardiovascular diseases	Yemen	2010	All Ages	7.4 (4.2 to 12.1)	17.0% (14.9 to 19.3%)	31.1 (17.7 to 51.1)
Cardiovascular diseases	Yemen	2015	All Ages	8.8 (5.2 to 14.1)	17.5% (15.3 to 19.8%)	32.7 (19.4 to 52.3)
Cardiovascular diseases	Yemen	1990	Age-standardized		16.5% (14.1 to 18.9%)	115.7 (73.0 to 167.7)
Cardiovascular diseases	Yemen	1995	Age-standardized		16.6% (14.4 to 19.0%)	112.8 (71.3 to 171.8)
Cardiovascular diseases	Yemen	2000	Age-standardized		16.4% (14.1 to 19.0%)	110.4 (68.9 to 166.9)
Cardiovascular diseases	Yemen	2005	Age-standardized		15.1% (12.9 to 17.6%)	96.1 (58.1 to 147.4)
Cardiovascular diseases	Yemen	2010	Age-standardized		15.8% (13.6 to 18.2%)	94.2 (56.9 to 147.8)

Cause name	Location	Year	Age group	Number (in thousands)	PAF	Rate (per 100,000)
Cardiovascular diseases	Yemen	2015	Age-standardized		15.9% (13.7 to 18.4%)	94.7 (60.0 to 145.3)
Ischemic heart disease	Yemen	1990	All Ages	3.2 (1.9 to 4.8)	21.8% (18.4 to 25.2%)	26.4 (15.6 to 39.9)
Ischemic heart disease	Yemen	1995	All Ages	4.6 (2.8 to 7.0)	21.1% (17.9 to 24.4%)	30.4 (18.7 to 46.4)
Ischemic heart disease	Yemen	2000	All Ages	4.8 (2.8 to 7.5)	21.4% (18.2 to 24.8%)	26.7 (15.9 to 42.0)
Ischemic heart disease	Yemen	2005	All Ages	4.6 (2.6 to 7.5)	20.2% (17.1 to 23.4%)	22.4 (12.5 to 36.4)
Ischemic heart disease	Yemen	2010	All Ages	5.2 (2.9 to 8.6)	21.4% (18.0 to 24.6%)	21.9 (12.1 to 36.1)
Ischemic heart disease	Yemen	2015	All Ages	6.1 (3.6 to 10.0)	21.8% (18.4 to 25.2%)	22.8 (13.5 to 37.3)
Ischemic heart disease	Yemen	1990	Age-standardized		19.9% (16.6 to 23.5%)	85.6 (53.3 to 125.9)
Ischemic heart disease	Yemen	1995	Age-standardized		19.9% (16.6 to 23.5%)	82.4 (52.6 to 125.2)
Ischemic heart disease	Yemen	2000	Age-standardized		19.8% (16.6 to 23.5%)	78.6 (48.1 to 119.8)
Ischemic heart disease	Yemen	2005	Age-standardized		18.4% (15.2 to 22.0%)	67.8 (39.7 to 105.7)
Ischemic heart disease	Yemen	2010	Age-standardized		19.2% (15.9 to 22.7%)	65.5 (39.3 to 103.4)
Ischemic heart disease	Yemen	2015	Age-standardized		19.4% (15.9 to 23.2%)	65.1 (40.6 to 101.2)
Ischemic stroke	Yemen	1990	All Ages	0.5 (0.3 to 0.8)	16.4% (13.5 to 19.3%)	4.1 (2.2 to 7.1)
Ischemic stroke	Yemen	1995	All Ages	0.8 (0.5 to 1.3)	15.7% (12.7 to 18.7%)	5.2 (3.0 to 8.4)
Ischemic stroke	Yemen	2000	All Ages	0.8 (0.5 to 1.3)	15.9% (13.0 to 18.9%)	4.8 (2.8 to 7.5)
Ischemic stroke	Yemen	2005	All Ages	0.8 (0.5 to 1.3)	14.7% (11.8 to 17.6%)	4.0 (2.3 to 6.4)
Ischemic stroke	Yemen	2010	All Ages	1.0 (0.6 to 1.6)	15.8% (12.8 to 18.8%)	4.1 (2.3 to 6.7)
Ischemic stroke	Yemen	2015	All Ages	1.2 (0.7 to 1.9)	16.2% (13.2 to 19.2%)	4.4 (2.5 to 7.2)
Ischemic stroke	Yemen	1990	Age-standardized		15.1% (11.7 to 18.4%)	15.8 (8.6 to 26.7)
Ischemic stroke	Yemen	1995	Age-standardized		15.0% (11.7 to 18.2%)	15.6 (9.0 to 24.8)
Ischemic stroke	Yemen	2000	Age-standardized		14.9% (11.6 to 18.4%)	16.2 (9.7 to 25.2)
Ischemic stroke	Yemen	2005	Age-standardized		13.6% (10.3 to 16.8%)	14.6 (8.6 to 23.0)
Ischemic stroke	Yemen	2010	Age-standardized		14.3% (10.9 to 18.0%)	14.9 (9.0 to 24.0)
Ischemic stroke	Yemen	2015	Age-standardized		14.5% (11.0 to 18.0%)	15.6 (9.5 to 24.8)
Hemorrhagic stroke	Yemen	1990	All Ages	0.6 (0.3 to 1.0)	14.1% (10.8 to 17.5%)	5.0 (2.8 to 8.1)
Hemorrhagic stroke	Yemen	1995	All Ages	0.9 (0.5 to 1.5)	15.3% (12.4 to 18.4%)	6.0 (3.5 to 9.7)
Hemorrhagic stroke	Yemen	2000	All Ages	1.1 (0.6 to 1.7)	16.3% (13.3 to 19.2%)	5.9 (3.4 to 9.4)
Hemorrhagic stroke	Yemen	2005	All Ages	1.0 (0.6 to 1.7)	15.4% (12.7 to 18.6%)	5.1 (2.7 to 8.4)
Hemorrhagic stroke	Yemen	2010	All Ages	1.2 (0.7 to 2.1)	16.7% (13.9 to 19.8%)	5.1 (2.8 to 8.8)
Hemorrhagic stroke	Yemen	2015	All Ages	1.5 (0.8 to 2.5)	17.4% (14.5 to 20.5%)	5.5 (3.1 to 9.3)
Hemorrhagic stroke	Yemen	1990	Age-standardized		16.5% (13.6 to 19.4%)	14.3 (8.5 to 22.8)
Hemorrhagic stroke	Yemen	1995	Age-standardized		16.6% (13.8 to 19.5%)	14.8 (8.7 to 23.4)
Hemorrhagic stroke	Yemen	2000	Age-standardized		16.7% (13.8 to 19.8%)	15.6 (9.3 to 24.6)
Hemorrhagic stroke	Yemen	2005	Age-standardized		15.3% (12.4 to 18.3%)	13.7 (7.8 to 21.9)
Hemorrhagic stroke	Yemen	2010	Age-standardized		16.2% (13.1 to 19.2%)	13.8 (7.9 to 22.9)

Cause name	Location	Year	Age group	Number (in thousands)	PAF	Rate (per 100,000)
Hemorrhagic stroke	Yemen	2015	Age-standardized		16.5% (13.5 to 19.5%)	14.0 (8.3 to 22.6)
Chronic obstructive pulmonary disease	Yemen	1990	All Ages	0.5 (0.2 to 1.0)	23.0% (12.8 to 34.3%)	4.4 (1.7 to 8.5)
Chronic obstructive pulmonary disease	Yemen	1995	All Ages	0.8 (0.4 to 1.4)	25.4% (15.6 to 37.5%)	5.2 (2.4 to 9.4)
Chronic obstructive pulmonary disease	Yemen	2000	All Ages	0.8 (0.4 to 1.6)	26.6% (16.5 to 38.5%)	4.5 (2.2 to 8.7)
Chronic obstructive pulmonary disease	Yemen	2005	All Ages	0.7 (0.3 to 1.4)	24.2% (14.2 to 34.8%)	3.5 (1.6 to 6.6)
Chronic obstructive pulmonary disease	Yemen	2010	All Ages	0.8 (0.4 to 1.5)	26.3% (16.4 to 37.4%)	3.5 (1.6 to 6.4)
Chronic obstructive pulmonary disease	Yemen	2015	All Ages	1.0 (0.5 to 1.8)	27.2% (16.9 to 39.0%)	3.7 (1.7 to 6.6)
Chronic obstructive pulmonary disease	Yemen	1990	Age-standardized		28.3% (17.3 to 40.5%)	16.8 (6.7 to 34.0)
Chronic obstructive pulmonary disease	Yemen	1995	Age-standardized		28.4% (17.7 to 40.8%)	15.6 (7.2 to 28.8)
Chronic obstructive pulmonary disease	Yemen	2000	Age-standardized		28.8% (17.9 to 41.2%)	15.3 (7.5 to 28.8)
Chronic obstructive pulmonary disease	Yemen	2005	Age-standardized		25.8% (15.5 to 37.4%)	12.5 (5.8 to 22.8)
Chronic obstructive pulmonary disease	Yemen	2010	Age-standardized		27.9% (17.5 to 39.8%)	12.5 (5.9 to 22.3)
Chronic obstructive pulmonary disease	Yemen	2015	Age-standardized		28.4% (17.6 to 40.9%)	12.6 (6.1 to 21.7)
All causes	Bangladesh	1990	All Ages	81.2 (66.9 to 95.5)	7.4% (6.2 to 8.7%)	76.6 (63.1 to 90.1)
All causes	Bangladesh	1995	All Ages	80.3 (66.8 to 94.5)	8.1% (6.9 to 9.3%)	67.8 (56.4 to 79.8)
All causes	Bangladesh	2000	All Ages	85.7 (72.8 to 98.5)	9.2% (8.0 to 10.4%)	65.2 (55.4 to 75.0)
All causes	Bangladesh	2005	All Ages	101.0 (88.5 to 113.8)	10.9% (9.7 to 12.2%)	70.7 (62.0 to 79.7)
All causes	Bangladesh	2010	All Ages	110.3 (96.6 to 126.0)	11.8% (10.5 to 13.2%)	72.7 (63.7 to 83.1)
All causes	Bangladesh	2015	All Ages	122.4 (103.2 to 144.4)	12.5% (11.0 to 14.1%)	76.0 (64.1 to 89.7)
All causes	Bangladesh	1990	Age-standardized		8.4% (7.0 to 9.9%)	130.4 (107.9 to 157.1)
All causes	Bangladesh	1995	Age-standardized		9.3% (7.9 to 10.8%)	126.1 (105.5 to 149.0)
All causes	Bangladesh	2000	Age-standardized		10.6% (9.1 to 12.1%)	125.2 (106.1 to 145.6)
All causes	Bangladesh	2005	Age-standardized		12.3% (10.8 to 13.8%)	135.8 (117.7 to 154.9)
All causes	Bangladesh	2010	Age-standardized		12.9% (11.4 to 14.7%)	135.8 (117.3 to 157.2)
All causes	Bangladesh	2015	Age-standardized		13.4% (11.7 to 15.3%)	133.2 (111.8 to 158.4)
Lower respiratory infections	Bangladesh	1990	All Ages	46.3 (34.6 to 58.6)	35.4% (27.5 to 42.7%)	43.7 (32.7 to 55.3)
Lower respiratory infections	Bangladesh	1995	All Ages	39.2 (29.7 to 50.0)	35.6% (27.7 to 42.9%)	33.1 (25.1 to 42.1)
Lower respiratory infections	Bangladesh	2000	All Ages	32.9 (25.1 to 40.9)	35.8% (27.9 to 43.1%)	25.1 (19.1 to 31.1)
Lower respiratory infections	Bangladesh	2005	All Ages	26.9 (20.4 to 33.0)	39.3% (30.5 to 47.3%)	18.8 (14.3 to 23.1)
Lower respiratory infections	Bangladesh	2010	All Ages	19.3 (14.5 to 24.4)	39.7% (30.9 to 47.8%)	12.7 (9.5 to 16.1)
Lower respiratory infections	Bangladesh	2015	All Ages	15.9 (11.8 to 20.9)	41.0% (32.1 to 49.5%)	9.9 (7.3 to 13.0)
Lower respiratory infections	Bangladesh	1990	Age-standardized		35.4% (27.5 to 42.7%)	37.5 (28.2 to 47.4)
Lower respiratory infections	Bangladesh	1995	Age-standardized		35.6% (27.7 to 42.9%)	32.3 (24.4 to 40.4)
Lower respiratory infections	Bangladesh	2000	Age-standardized		35.8% (27.9 to 43.1%)	26.9 (20.3 to 33.1)
Lower respiratory infections	Bangladesh	2005	Age-standardized		39.3% (30.5 to 47.3%)	21.6 (16.3 to 27.1)
Lower respiratory infections	Bangladesh	2010	Age-standardized		39.7% (30.9 to 47.8%)	16.1 (12.0 to 21.3)

Cause name	Location	Year	Age group	Number (in thousands)	PAF	Rate (per 100,000)
Lower respiratory infections	Bangladesh	2015	Age-standardized		41.0% (32.1 to 49.5%)	13.0 (9.5 to 17.9)
Tracheal, bronchus, and lung cancer	Bangladesh	1990	All Ages	2.1 (1.4 to 2.8)	26.6% (18.1 to 35.0%)	2.0 (1.3 to 2.7)
Tracheal, bronchus, and lung cancer	Bangladesh	1995	All Ages	2.5 (1.6 to 3.3)	26.8% (18.4 to 35.3%)	2.1 (1.4 to 2.8)
Tracheal, bronchus, and lung cancer	Bangladesh	2000	All Ages	3.0 (2.0 to 4.1)	27.0% (18.3 to 35.4%)	2.3 (1.6 to 3.1)
Tracheal, bronchus, and lung cancer	Bangladesh	2005	All Ages	4.1 (2.8 to 5.4)	30.3% (21.4 to 38.9%)	2.9 (2.0 to 3.8)
Tracheal, bronchus, and lung cancer	Bangladesh	2010	All Ages	5.4 (3.7 to 7.1)	30.7% (21.7 to 39.3%)	3.6 (2.5 to 4.7)
Tracheal, bronchus, and lung cancer	Bangladesh	2015	All Ages	8.2 (5.6 to 10.9)	32.3% (23.2 to 41.0%)	5.1 (3.5 to 6.7)
Tracheal, bronchus, and lung cancer	Bangladesh	1990	Age-standardized		26.7% (18.2 to 35.2%)	5.0 (3.3 to 6.9)
Tracheal, bronchus, and lung cancer	Bangladesh	1995	Age-standardized		26.9% (18.5 to 35.4%)	5.1 (3.4 to 7.0)
Tracheal, bronchus, and lung cancer	Bangladesh	2000	Age-standardized		27.1% (18.4 to 35.5%)	5.3 (3.6 to 7.1)
Tracheal, bronchus, and lung cancer	Bangladesh	2005	Age-standardized		30.4% (21.5 to 39.1%)	5.8 (4.1 to 7.7)
Tracheal, bronchus, and lung cancer	Bangladesh	2010	Age-standardized		30.8% (21.8 to 39.5%)	7.0 (4.8 to 9.2)
Tracheal, bronchus, and lung cancer	Bangladesh	2015	Age-standardized		32.3% (23.3 to 41.1%)	9.5 (6.5 to 12.6)
Cardiovascular diseases	Bangladesh	1990	All Ages	18.9 (16.1 to 22.1)	18.2% (16.2 to 20.4%)	17.9 (15.2 to 20.9)
Cardiovascular diseases	Bangladesh	1995	All Ages	23.3 (19.8 to 27.4)	18.2% (16.1 to 20.4%)	19.7 (16.7 to 23.1)
Cardiovascular diseases	Bangladesh	2000	All Ages	32.5 (27.6 to 37.5)	18.2% (16.2 to 20.4%)	24.8 (21.0 to 28.5)
Cardiovascular diseases	Bangladesh	2005	All Ages	50.3 (43.9 to 57.6)	19.9% (17.8 to 22.1%)	35.2 (30.7 to 40.3)
Cardiovascular diseases	Bangladesh	2010	All Ages	62.3 (53.8 to 72.1)	20.5% (18.4 to 22.8%)	41.1 (35.4 to 47.5)
Cardiovascular diseases	Bangladesh	2015	All Ages	68.4 (57.8 to 81.5)	21.4% (19.3 to 23.8%)	42.5 (35.9 to 50.6)
Cardiovascular diseases	Bangladesh	1990	Age-standardized		16.6% (14.3 to 18.8%)	48.8 (40.7 to 57.0)
Cardiovascular diseases	Bangladesh	1995	Age-standardized		16.6% (14.3 to 18.8%)	51.3 (42.8 to 60.1)
Cardiovascular diseases	Bangladesh	2000	Age-standardized		16.8% (14.5 to 19.2%)	58.5 (48.8 to 68.5)
Cardiovascular diseases	Bangladesh	2005	Age-standardized		18.6% (16.2 to 21.1%)	75.1 (64.3 to 86.8)
Cardiovascular diseases	Bangladesh	2010	Age-standardized		19.3% (17.0 to 21.8%)	79.1 (67.3 to 92.5)
Cardiovascular diseases	Bangladesh	2015	Age-standardized		20.2% (17.8 to 22.6%)	73.4 (61.3 to 87.7)
Ischemic heart disease	Bangladesh	1990	All Ages	7.0 (5.7 to 8.3)	23.9% (20.6 to 27.5%)	6.6 (5.4 to 7.8)
Ischemic heart disease	Bangladesh	1995	All Ages	8.1 (6.6 to 9.7)	24.0% (20.6 to 27.5%)	6.8 (5.6 to 8.2)
Ischemic heart disease	Bangladesh	2000	All Ages	11.6 (9.6 to 13.8)	23.6% (20.3 to 27.2%)	8.8 (7.3 to 10.5)
Ischemic heart disease	Bangladesh	2005	All Ages	21.6 (18.3 to 25.5)	24.8% (21.6 to 28.6%)	15.1 (12.8 to 17.9)
Ischemic heart disease	Bangladesh	2010	All Ages	30.5 (25.4 to 36.4)	24.7% (21.4 to 28.7%)	20.1 (16.8 to 24.0)
Ischemic heart disease	Bangladesh	2015	All Ages	35.3 (28.9 to 43.7)	25.1% (21.7 to 29.3%)	22.0 (17.9 to 27.1)
Ischemic heart disease	Bangladesh	1990	Age-standardized		21.1% (17.7 to 25.5%)	17.6 (14.3 to 21.5)
Ischemic heart disease	Bangladesh	1995	Age-standardized		21.2% (17.7 to 25.7%)	17.4 (14.2 to 21.2)
Ischemic heart disease	Bangladesh	2000	Age-standardized		21.2% (17.6 to 25.7%)	20.3 (16.4 to 24.7)
Ischemic heart disease	Bangladesh	2005	Age-standardized		22.8% (19.3 to 27.1%)	30.8 (25.3 to 37.7)
Ischemic heart disease	Bangladesh	2010	Age-standardized		23.0% (19.5 to 27.4%)	37.4 (30.9 to 45.6)

Cause name	Location	Year	Age group	Number (in thousands)	PAF	Rate (per 100,000)
Ischemic heart disease	Bangladesh	2015	Age-standardized		23.4% (20.0 to 28.0%)	37.2 (30.0 to 46.7)
Ischemic stroke	Bangladesh	1990	All Ages	4.7 (3.4 to 6.3)	18.3% (14.9 to 21.5%)	4.5 (3.2 to 5.9)
Ischemic stroke	Bangladesh	1995	All Ages	6.2 (4.4 to 8.0)	18.2% (14.7 to 21.4%)	5.2 (3.7 to 6.8)
Ischemic stroke	Bangladesh	2000	All Ages	8.7 (6.3 to 11.3)	17.8% (14.5 to 21.2%)	6.7 (4.8 to 8.6)
Ischemic stroke	Bangladesh	2005	All Ages	12.4 (9.2 to 16.0)	18.6% (15.3 to 22.2%)	8.7 (6.4 to 11.2)
Ischemic stroke	Bangladesh	2010	All Ages	13.7 (10.1 to 18.0)	18.5% (15.1 to 22.3%)	9.0 (6.6 to 11.9)
Ischemic stroke	Bangladesh	2015	All Ages	13.6 (10.0 to 18.3)	19.1% (15.5 to 22.9%)	8.5 (6.2 to 11.3)
Ischemic stroke	Bangladesh	1990	Age-standardized		16.5% (12.8 to 20.1%)	14.8 (10.6 to 19.3)
Ischemic stroke	Bangladesh	1995	Age-standardized		16.5% (12.8 to 20.1%)	16.3 (11.7 to 21.3)
Ischemic stroke	Bangladesh	2000	Age-standardized		16.5% (12.8 to 20.1%)	18.3 (13.3 to 23.9)
Ischemic stroke	Bangladesh	2005	Age-standardized		17.6% (13.8 to 21.4%)	21.3 (15.7 to 28.3)
Ischemic stroke	Bangladesh	2010	Age-standardized		17.7% (14.0 to 21.8%)	19.8 (14.4 to 26.4)
Ischemic stroke	Bangladesh	2015	Age-standardized		18.4% (14.6 to 22.4%)	16.7 (12.0 to 22.2)
Hemorrhagic stroke	Bangladesh	1990	All Ages	7.2 (5.6 to 9.1)	20.6% (17.4 to 23.8%)	6.8 (5.3 to 8.6)
Hemorrhagic stroke	Bangladesh	1995	All Ages	9.0 (6.9 to 11.5)	20.5% (17.4 to 23.8%)	7.6 (5.9 to 9.7)
Hemorrhagic stroke	Bangladesh	2000	All Ages	12.2 (9.5 to 15.2)	20.3% (17.2 to 23.4%)	9.3 (7.2 to 11.6)
Hemorrhagic stroke	Bangladesh	2005	All Ages	16.4 (12.8 to 20.2)	21.4% (18.3 to 24.5%)	11.5 (9.0 to 14.1)
Hemorrhagic stroke	Bangladesh	2010	All Ages	18.2 (14.2 to 22.2)	21.6% (18.5 to 24.6%)	12.0 (9.3 to 14.6)
Hemorrhagic stroke	Bangladesh	2015	All Ages	19.4 (15.0 to 24.4)	22.6% (19.5 to 25.6%)	12.1 (9.3 to 15.1)
Hemorrhagic stroke	Bangladesh	1990	Age-standardized		18.9% (15.7 to 22.0%)	16.4 (12.4 to 21.2)
Hemorrhagic stroke	Bangladesh	1995	Age-standardized		18.8% (15.7 to 21.8%)	17.6 (13.4 to 22.7)
Hemorrhagic stroke	Bangladesh	2000	Age-standardized		18.8% (15.7 to 21.9%)	19.9 (14.9 to 25.1)
Hemorrhagic stroke	Bangladesh	2005	Age-standardized		20.0% (16.8 to 23.1%)	22.9 (17.4 to 28.7)
Hemorrhagic stroke	Bangladesh	2010	Age-standardized		20.3% (17.1 to 23.4%)	21.9 (16.6 to 27.3)
Hemorrhagic stroke	Bangladesh	2015	Age-standardized		21.2% (18.1 to 24.4%)	19.6 (14.8 to 25.0)
Chronic obstructive pulmonary disease	Bangladesh	1990	All Ages	13.8 (7.2 to 21.8)	31.0% (19.9 to 43.8%)	13.1 (6.8 to 20.6)
Chronic obstructive pulmonary disease	Bangladesh	1995	All Ages	15.4 (8.4 to 23.3)	31.7% (20.4 to 44.8%)	13.0 (7.1 to 19.7)
Chronic obstructive pulmonary disease	Bangladesh	2000	All Ages	17.2 (10.5 to 25.0)	32.2% (20.7 to 45.4%)	13.1 (8.0 to 19.0)
Chronic obstructive pulmonary disease	Bangladesh	2005	All Ages	19.8 (12.8 to 28.2)	35.3% (23.8 to 49.9%)	13.8 (9.0 to 19.7)
Chronic obstructive pulmonary disease	Bangladesh	2010	All Ages	23.3 (15.4 to 33.4)	35.9% (24.3 to 50.8%)	15.3 (10.2 to 22.0)
Chronic obstructive pulmonary disease	Bangladesh	2015	All Ages	30.0 (19.6 to 42.5)	37.2% (25.5 to 52.5%)	18.6 (12.2 to 26.4)
Chronic obstructive pulmonary disease	Bangladesh	1990	Age-standardized		32.3% (20.8 to 45.6%)	39.0 (20.8 to 60.4)
Chronic obstructive pulmonary disease	Bangladesh	1995	Age-standardized		32.6% (21.1 to 46.1%)	37.4 (21.0 to 55.9)
Chronic obstructive pulmonary disease	Bangladesh	2000	Age-standardized		32.8% (21.2 to 46.3%)	34.5 (21.5 to 50.0)
Chronic obstructive pulmonary disease	Bangladesh	2005	Age-standardized		35.7% (24.1 to 50.5%)	33.2 (21.9 to 47.4)
Chronic obstructive pulmonary disease	Bangladesh	2010	Age-standardized		36.1% (24.5 to 51.2%)	33.6 (22.2 to 47.8)

Cause name	Location	Year	Age group	Number (in thousands)	PAF	Rate (per 100,000)
Chronic obstructive pulmonary disease	Bangladesh	2015	Age-standardized		37.4% (25.6 to 52.7%)	37.3 (24.5 to 53.0)
All causes	Bhutan	1990	All Ages	0.3 (0.2 to 0.3)	5.3% (4.2 to 6.6%)	48.0 (37.2 to 62.0)
All causes	Bhutan	1995	All Ages	0.2 (0.2 to 0.3)	5.8% (4.7 to 7.0%)	45.1 (35.6 to 56.9)
All causes	Bhutan	2000	All Ages	0.2 (0.2 to 0.3)	6.0% (5.0 to 7.2%)	40.6 (32.6 to 49.8)
All causes	Bhutan	2005	All Ages	0.3 (0.2 to 0.3)	7.4% (6.2 to 8.7%)	40.7 (33.2 to 49.3)
All causes	Bhutan	2010	All Ages	0.3 (0.2 to 0.4)	8.0% (6.8 to 9.2%)	40.5 (32.6 to 49.6)
All causes	Bhutan	2015	All Ages	0.4 (0.3 to 0.4)	9.0% (7.7 to 10.4%)	46.1 (36.4 to 57.4)
All causes	Bhutan	1990	Age-standardized		8.1% (6.6 to 9.7%)	107.5 (84.5 to 136.3)
All causes	Bhutan	1995	Age-standardized		8.4% (6.9 to 10.0%)	103.7 (82.6 to 129.7)
All causes	Bhutan	2000	Age-standardized		8.3% (6.8 to 9.8%)	91.4 (73.4 to 111.8)
All causes	Bhutan	2005	Age-standardized		9.4% (7.8 to 11.1%)	88.9 (72.0 to 108.1)
All causes	Bhutan	2010	Age-standardized		9.5% (7.9 to 11.0%)	81.2 (65.0 to 99.7)
All causes	Bhutan	2015	Age-standardized		10.1% (8.5 to 11.8%)	83.4 (65.6 to 103.7)
Lower respiratory infections	Bhutan	1990	All Ages	0.1 (0.1 to 0.1)	27.0% (19.7 to 34.7%)	18.7 (10.7 to 28.1)
Lower respiratory infections	Bhutan	1995	All Ages	0.1 (0.1 to 0.1)	27.1% (19.9 to 34.9%)	14.8 (9.3 to 22.1)
Lower respiratory infections	Bhutan	2000	All Ages	0.1 (0.0 to 0.1)	27.3% (19.7 to 34.7%)	11.6 (7.8 to 16.7)
Lower respiratory infections	Bhutan	2005	All Ages	0.1 (0.1 to 0.1)	31.1% (23.5 to 38.3%)	10.3 (7.1 to 14.3)
Lower respiratory infections	Bhutan	2010	All Ages	0.1 (0.0 to 0.1)	30.8% (23.2 to 38.0%)	8.8 (5.9 to 12.3)
Lower respiratory infections	Bhutan	2015	All Ages	0.1 (0.0 to 0.1)	32.5% (24.9 to 39.6%)	8.4 (5.6 to 12.0)
Lower respiratory infections	Bhutan	1990	Age-standardized		27.0% (19.7 to 34.7%)	20.4 (13.2 to 29.3)
Lower respiratory infections	Bhutan	1995	Age-standardized		27.1% (19.9 to 34.9%)	18.0 (12.0 to 25.8)
Lower respiratory infections	Bhutan	2000	Age-standardized		27.3% (19.7 to 34.7%)	16.0 (10.6 to 23.2)
Lower respiratory infections	Bhutan	2005	Age-standardized		31.1% (23.5 to 38.3%)	15.8 (10.7 to 22.8)
Lower respiratory infections	Bhutan	2010	Age-standardized		30.8% (23.2 to 38.0%)	14.0 (9.2 to 20.4)
Lower respiratory infections	Bhutan	2015	Age-standardized		32.5% (24.9 to 39.6%)	13.4 (8.7 to 19.3)
Tracheal, bronchus, and lung cancer	Bhutan	1990	All Ages	0.0 (0.0 to 0.0)	19.5% (12.1 to 26.9%)	0.5 (0.3 to 0.8)
Tracheal, bronchus, and lung cancer	Bhutan	1995	All Ages	0.0 (0.0 to 0.0)	19.6% (12.3 to 27.3%)	0.5 (0.3 to 0.8)
Tracheal, bronchus, and lung cancer	Bhutan	2000	All Ages	0.0 (0.0 to 0.0)	19.7% (12.4 to 27.6%)	0.5 (0.3 to 0.9)
Tracheal, bronchus, and lung cancer	Bhutan	2005	All Ages	0.0 (0.0 to 0.0)	22.8% (14.7 to 30.7%)	0.7 (0.4 to 1.2)
Tracheal, bronchus, and lung cancer	Bhutan	2010	All Ages	0.0 (0.0 to 0.0)	22.6% (14.4 to 30.5%)	0.7 (0.4 to 1.2)
Tracheal, bronchus, and lung cancer	Bhutan	2015	All Ages	0.0 (0.0 to 0.0)	24.2% (15.9 to 32.5%)	0.9 (0.5 to 1.5)
Tracheal, bronchus, and lung cancer	Bhutan	1990	Age-standardized		19.6% (12.2 to 27.1%)	1.3 (0.7 to 2.1)
Tracheal, bronchus, and lung cancer	Bhutan	1995	Age-standardized		19.7% (12.4 to 27.4%)	1.3 (0.7 to 2.1)
Tracheal, bronchus, and lung cancer	Bhutan	2000	Age-standardized		19.8% (12.4 to 27.7%)	1.2 (0.7 to 2.0)
Tracheal, bronchus, and lung cancer	Bhutan	2005	Age-standardized		22.9% (14.7 to 30.9%)	1.5 (0.8 to 2.5)
Tracheal, bronchus, and lung cancer	Bhutan	2010	Age-standardized		22.7% (14.4 to 30.6%)	1.5 (0.8 to 2.3)

Cause name	Location	Year	Age group	Number (in thousands)	PAF	Rate (per 100,000)
Tracheal, bronchus, and lung cancer	Bhutan	2015	Age-standardized		24.3% (16.0 to 32.6%)	1.7 (1.0 to 2.7)
Cardiovascular diseases	Bhutan	1990	All Ages	0.1 (0.1 to 0.1)	15.6% (13.6 to 17.6%)	18.3 (14.1 to 23.6)
Cardiovascular diseases	Bhutan	1995	All Ages	0.1 (0.1 to 0.1)	15.4% (13.3 to 17.5%)	19.0 (14.8 to 23.5)
Cardiovascular diseases	Bhutan	2000	All Ages	0.1 (0.1 to 0.1)	15.4% (13.3 to 17.6%)	18.4 (14.6 to 23.0)
Cardiovascular diseases	Bhutan	2005	All Ages	0.1 (0.1 to 0.2)	16.6% (14.3 to 18.8%)	19.4 (15.2 to 24.3)
Cardiovascular diseases	Bhutan	2010	All Ages	0.1 (0.1 to 0.2)	16.4% (14.2 to 18.9%)	20.4 (15.7 to 26.0)
Cardiovascular diseases	Bhutan	2015	All Ages	0.2 (0.1 to 0.2)	16.9% (14.5 to 19.5%)	24.1 (18.3 to 30.5)
Cardiovascular diseases	Bhutan	1990	Age-standardized		14.4% (12.2 to 16.8%)	50.4 (38.9 to 64.3)
Cardiovascular diseases	Bhutan	1995	Age-standardized		14.5% (12.3 to 16.9%)	50.7 (39.6 to 63.0)
Cardiovascular diseases	Bhutan	2000	Age-standardized		14.6% (12.4 to 17.2%)	45.8 (36.3 to 56.7)
Cardiovascular diseases	Bhutan	2005	Age-standardized		15.7% (13.3 to 18.5%)	44.5 (35.1 to 56.0)
Cardiovascular diseases	Bhutan	2010	Age-standardized		15.6% (13.2 to 18.5%)	41.7 (32.1 to 52.9)
Cardiovascular diseases	Bhutan	2015	Age-standardized		16.1% (13.6 to 19.1%)	43.3 (33.1 to 54.9)
Ischemic heart disease	Bhutan	1990	All Ages	0.1 (0.1 to 0.1)	20.0% (16.9 to 23.3%)	13.1 (9.9 to 17.0)
Ischemic heart disease	Bhutan	1995	All Ages	0.1 (0.1 to 0.1)	19.5% (16.5 to 22.7%)	13.7 (10.6 to 17.4)
Ischemic heart disease	Bhutan	2000	All Ages	0.1 (0.1 to 0.1)	19.3% (16.3 to 22.6%)	13.5 (10.6 to 17.0)
Ischemic heart disease	Bhutan	2005	All Ages	0.1 (0.1 to 0.1)	20.4% (17.2 to 23.9%)	14.2 (10.9 to 18.1)
Ischemic heart disease	Bhutan	2010	All Ages	0.1 (0.1 to 0.1)	20.0% (16.7 to 23.7%)	15.0 (11.2 to 19.5)
Ischemic heart disease	Bhutan	2015	All Ages	0.1 (0.1 to 0.2)	20.2% (16.7 to 24.1%)	17.8 (13.2 to 23.0)
Ischemic heart disease	Bhutan	1990	Age-standardized		17.8% (14.3 to 21.5%)	36.1 (27.4 to 46.6)
Ischemic heart disease	Bhutan	1995	Age-standardized		17.7% (14.3 to 21.5%)	36.8 (28.2 to 46.1)
Ischemic heart disease	Bhutan	2000	Age-standardized		17.7% (14.2 to 21.7%)	33.6 (25.6 to 43.1)
Ischemic heart disease	Bhutan	2005	Age-standardized		18.9% (15.2 to 23.2%)	32.7 (24.5 to 42.2)
Ischemic heart disease	Bhutan	2010	Age-standardized		18.7% (15.0 to 22.9%)	30.7 (22.8 to 39.6)
Ischemic heart disease	Bhutan	2015	Age-standardized		19.1% (15.3 to 23.6%)	32.0 (23.4 to 41.4)
Ischemic stroke	Bhutan	1990	All Ages	0.0 (0.0 to 0.0)	14.7% (11.7 to 17.7%)	1.9 (1.3 to 2.8)
Ischemic stroke	Bhutan	1995	All Ages	0.0 (0.0 to 0.0)	14.5% (11.4 to 17.2%)	2.0 (1.4 to 2.8)
Ischemic stroke	Bhutan	2000	All Ages	0.0 (0.0 to 0.0)	14.3% (11.1 to 17.3%)	1.9 (1.4 to 2.7)
Ischemic stroke	Bhutan	2005	All Ages	0.0 (0.0 to 0.0)	15.4% (12.0 to 18.6%)	2.1 (1.5 to 2.8)
Ischemic stroke	Bhutan	2010	All Ages	0.0 (0.0 to 0.0)	15.0% (11.5 to 18.3%)	2.2 (1.5 to 3.0)
Ischemic stroke	Bhutan	2015	All Ages	0.0 (0.0 to 0.0)	15.3% (11.7 to 19.0%)	2.7 (1.8 to 3.6)
Ischemic stroke	Bhutan	1990	Age-standardized		13.3% (9.9 to 16.8%)	6.5 (4.3 to 9.3)
Ischemic stroke	Bhutan	1995	Age-standardized		13.3% (9.8 to 16.8%)	6.4 (4.3 to 9.0)
Ischemic stroke	Bhutan	2000	Age-standardized		13.3% (9.8 to 17.0%)	5.6 (3.8 to 7.8)
Ischemic stroke	Bhutan	2005	Age-standardized		14.5% (10.7 to 18.3%)	5.5 (3.7 to 7.4)
Ischemic stroke	Bhutan	2010	Age-standardized		14.3% (10.6 to 18.2%)	5.1 (3.4 to 7.0)

Cause name	Location	Year	Age group	Number (in thousands)	PAF	Rate (per 100,000)
Ischemic stroke	Bhutan	2015	Age-standardized		14.7% (10.9 to 18.8%)	5.3 (3.6 to 7.2)
Hemorrhagic stroke	Bhutan	1990	All Ages	0.0 (0.0 to 0.0)	15.6% (12.9 to 18.6%)	3.3 (2.3 to 4.6)
Hemorrhagic stroke	Bhutan	1995	All Ages	0.0 (0.0 to 0.0)	15.5% (12.7 to 18.4%)	3.2 (2.3 to 4.4)
Hemorrhagic stroke	Bhutan	2000	All Ages	0.0 (0.0 to 0.0)	15.5% (12.8 to 18.3%)	3.0 (2.2 to 4.0)
Hemorrhagic stroke	Bhutan	2005	All Ages	0.0 (0.0 to 0.0)	17.2% (14.4 to 19.9%)	3.1 (2.2 to 4.2)
Hemorrhagic stroke	Bhutan	2010	All Ages	0.0 (0.0 to 0.0)	17.1% (14.4 to 19.8%)	3.2 (2.2 to 4.4)
Hemorrhagic stroke	Bhutan	2015	All Ages	0.0 (0.0 to 0.0)	17.8% (14.9 to 20.7%)	3.6 (2.5 to 5.0)
Hemorrhagic stroke	Bhutan	1990	Age-standardized		15.1% (12.2 to 17.9%)	7.8 (5.6 to 10.6)
Hemorrhagic stroke	Bhutan	1995	Age-standardized		15.1% (12.1 to 17.9%)	7.5 (5.5 to 10.2)
Hemorrhagic stroke	Bhutan	2000	Age-standardized		15.1% (12.3 to 18.0%)	6.6 (4.7 to 8.9)
Hemorrhagic stroke	Bhutan	2005	Age-standardized		16.5% (13.5 to 19.3%)	6.4 (4.4 to 8.6)
Hemorrhagic stroke	Bhutan	2010	Age-standardized		16.2% (13.3 to 19.1%)	5.9 (4.1 to 8.1)
Hemorrhagic stroke	Bhutan	2015	Age-standardized		16.8% (13.7 to 19.8%)	6.0 (4.2 to 8.3)
Chronic obstructive pulmonary disease	Bhutan	1990	All Ages	0.1 (0.0 to 0.1)	22.4% (12.9 to 32.5%)	10.4 (5.7 to 16.1)
Chronic obstructive pulmonary disease	Bhutan	1995	All Ages	0.1 (0.0 to 0.1)	23.2% (13.3 to 33.6%)	10.8 (5.9 to 16.1)
Chronic obstructive pulmonary disease	Bhutan	2000	All Ages	0.1 (0.0 to 0.1)	23.8% (13.9 to 34.5%)	10.0 (5.4 to 15.1)
Chronic obstructive pulmonary disease	Bhutan	2005	All Ages	0.1 (0.0 to 0.1)	27.3% (16.5 to 38.8%)	10.4 (5.7 to 15.4)
Chronic obstructive pulmonary disease	Bhutan	2010	All Ages	0.1 (0.0 to 0.1)	27.7% (17.0 to 39.0%)	10.5 (5.9 to 15.8)
Chronic obstructive pulmonary disease	Bhutan	2015	All Ages	0.1 (0.1 to 0.1)	29.6% (18.6 to 42.0%)	12.7 (7.2 to 19.0)
Chronic obstructive pulmonary disease	Bhutan	1990	Age-standardized		25.3% (14.8 to 36.4%)	35.4 (20.0 to 54.4)
Chronic obstructive pulmonary disease	Bhutan	1995	Age-standardized		25.4% (14.8 to 36.5%)	33.8 (18.6 to 50.1)
Chronic obstructive pulmonary disease	Bhutan	2000	Age-standardized		25.6% (14.9 to 36.8%)	28.4 (15.3 to 42.4)
Chronic obstructive pulmonary disease	Bhutan	2005	Age-standardized		28.6% (17.6 to 40.7%)	27.0 (14.7 to 39.9)
Chronic obstructive pulmonary disease	Bhutan	2010	Age-standardized		28.5% (17.5 to 40.1%)	24.0 (13.4 to 36.0)
Chronic obstructive pulmonary disease	Bhutan	2015	Age-standardized		30.0% (18.9 to 42.6%)	25.0 (14.3 to 37.5)
All causes	India	1990	All Ages	737.4 (634.8 to 859.0)	7.9% (6.8 to 9.1%)	84.7 (72.9 to 98.7)
All causes	India	1995	All Ages	795.2 (684.8 to 918.3)	8.3% (7.2 to 9.7%)	82.7 (71.2 to 95.5)
All causes	India	2000	All Ages	857.3 (732.9 to 988.1)	8.8% (7.5 to 10.1%)	81.4 (69.6 to 93.8)
All causes	India	2005	All Ages	895.9 (769.7 to 1,026.1)	9.1% (7.8 to 10.3%)	78.3 (67.3 to 89.7)
All causes	India	2010	All Ages	957.0 (822.6 to 1,092.1)	9.5% (8.2 to 10.8%)	77.8 (66.9 to 88.8)
All causes	India	2015	All Ages	1,090.4 (936.6 to 1,254.8)	10.6% (9.2 to 12.1%)	83.1 (71.4 to 95.7)
All causes	India	1990	Age-standardized		9.8% (8.2 to 11.5%)	163.7 (136.9 to 192.8)
All causes	India	1995	Age-standardized		10.2% (8.6 to 12.0%)	160.8 (134.4 to 188.3)
All causes	India	2000	Age-standardized		10.5% (8.8 to 12.2%)	154.9 (129.5 to 180.7)
All causes	India	2005	Age-standardized		10.6% (8.9 to 12.2%)	143.2 (120.9 to 165.5)
All causes	India	2010	Age-standardized		10.7% (9.0 to 12.3%)	135.0 (114.4 to 156.1)

Cause name	Location	Year	Age group	Number (in thousands)	PAF	Rate (per 100,000)
All causes	India	2015	Age-standardized		11.5% (9.8 to 13.3%)	133.5 (112.8 to 154.9)
Lower respiratory infections	India	1990	All Ages	264.4 (202.3 to 325.0)	35.5% (27.7 to 42.6%)	30.4 (23.2 to 37.3)
Lower respiratory infections	India	1995	All Ages	243.6 (190.4 to 300.4)	35.6% (27.8 to 42.7%)	25.3 (19.8 to 31.2)
Lower respiratory infections	India	2000	All Ages	225.8 (172.8 to 278.1)	35.5% (27.6 to 42.6%)	21.4 (16.4 to 26.4)
Lower respiratory infections	India	2005	All Ages	217.5 (165.2 to 266.1)	36.4% (28.5 to 43.7%)	19.0 (14.4 to 23.3)
Lower respiratory infections	India	2010	All Ages	196.5 (149.0 to 240.1)	35.9% (28.1 to 43.1%)	16.0 (12.1 to 19.5)
Lower respiratory infections	India	2015	All Ages	200.7 (151.9 to 246.4)	37.9% (29.9 to 45.2%)	15.3 (11.6 to 18.8)
Lower respiratory infections	India	1990	Age-standardized		35.1% (27.2 to 42.3%)	34.5 (26.1 to 42.3)
Lower respiratory infections	India	1995	Age-standardized		35.2% (27.4 to 42.4%)	30.6 (23.6 to 37.6)
Lower respiratory infections	India	2000	Age-standardized		35.1% (27.3 to 42.5%)	27.8 (21.2 to 34.2)
Lower respiratory infections	India	2005	Age-standardized		36.2% (28.2 to 43.5%)	26.6 (19.9 to 32.7)
Lower respiratory infections	India	2010	Age-standardized		35.7% (27.8 to 43.0%)	23.3 (17.4 to 28.6)
Lower respiratory infections	India	2015	Age-standardized		37.8% (29.7 to 45.1%)	22.1 (16.6 to 27.3)
Tracheal, bronchus, and lung cancer	India	1990	All Ages	8.7 (5.7 to 11.8)	24.2% (16.4 to 32.1%)	1.0 (0.7 to 1.4)
Tracheal, bronchus, and lung cancer	India	1995	All Ages	9.8 (6.4 to 13.1)	24.3% (16.5 to 32.4%)	1.0 (0.7 to 1.4)
Tracheal, bronchus, and lung cancer	India	2000	All Ages	11.3 (7.5 to 15.1)	24.5% (16.6 to 32.5%)	1.1 (0.7 to 1.4)
Tracheal, bronchus, and lung cancer	India	2005	All Ages	14.2 (9.6 to 18.8)	25.6% (17.5 to 33.7%)	1.2 (0.8 to 1.6)
Tracheal, bronchus, and lung cancer	India	2010	All Ages	17.0 (11.5 to 22.6)	25.2% (17.2 to 33.1%)	1.4 (0.9 to 1.8)
Tracheal, bronchus, and lung cancer	India	2015	All Ages	22.5 (15.6 to 29.5)	27.5% (19.2 to 35.7%)	1.7 (1.2 to 2.2)
Tracheal, bronchus, and lung cancer	India	1990	Age-standardized		24.4% (16.5 to 32.4%)	2.1 (1.4 to 2.8)
Tracheal, bronchus, and lung cancer	India	1995	Age-standardized		24.5% (16.6 to 32.5%)	2.0 (1.3 to 2.8)
Tracheal, bronchus, and lung cancer	India	2000	Age-standardized		24.6% (16.7 to 32.7%)	2.0 (1.4 to 2.7)
Tracheal, bronchus, and lung cancer	India	2005	Age-standardized		25.7% (17.6 to 33.8%)	2.2 (1.5 to 2.9)
Tracheal, bronchus, and lung cancer	India	2010	Age-standardized		25.3% (17.3 to 33.2%)	2.3 (1.6 to 3.0)
Tracheal, bronchus, and lung cancer	India	2015	Age-standardized		27.5% (19.3 to 35.8%)	2.6 (1.8 to 3.4)
Cardiovascular diseases	India	1990	All Ages	263.4 (232.3 to 297.3)	17.5% (15.6 to 19.6%)	30.3 (26.7 to 34.1)
Cardiovascular diseases	India	1995	All Ages	304.1 (268.5 to 342.7)	17.7% (15.8 to 19.9%)	31.6 (27.9 to 35.6)
Cardiovascular diseases	India	2000	All Ages	348.8 (307.1 to 391.5)	17.8% (15.8 to 20.0%)	33.1 (29.1 to 37.2)
Cardiovascular diseases	India	2005	All Ages	388.6 (341.7 to 438.8)	18.1% (16.1 to 20.3%)	34.0 (29.9 to 38.3)
Cardiovascular diseases	India	2010	All Ages	448.0 (392.3 to 509.1)	17.8% (15.7 to 19.9%)	36.4 (31.9 to 41.4)
Cardiovascular diseases	India	2015	All Ages	518.1 (452.6 to 583.5)	18.6% (16.5 to 20.9%)	39.5 (34.5 to 44.5)
Cardiovascular diseases	India	1990	Age-standardized		16.3% (14.2 to 18.5%)	66.0 (57.4 to 76.2)
Cardiovascular diseases	India	1995	Age-standardized		16.4% (14.3 to 18.7%)	66.0 (57.4 to 76.4)
Cardiovascular diseases	India	2000	Age-standardized		16.5% (14.4 to 18.9%)	65.0 (56.5 to 75.0)
Cardiovascular diseases	India	2005	Age-standardized		16.8% (14.7 to 19.2%)	62.4 (54.0 to 72.0)
Cardiovascular diseases	India	2010	Age-standardized		16.6% (14.5 to 18.9%)	61.9 (53.4 to 71.3)

Cause name	Location	Year	Age group	Number (in thousands)	PAF	Rate (per 100,000)
Cardiovascular diseases	India	2015	Age-standardized		17.4% (15.2 to 19.9%)	61.3 (53.0 to 70.8)
Ischemic heart disease	India	1990	All Ages	175.2 (147.3 to 203.9)	22.0% (18.8 to 25.3%)	20.1 (16.9 to 23.4)
Ischemic heart disease	India	1995	All Ages	203.8 (172.7 to 237.2)	22.0% (18.9 to 25.3%)	21.2 (18.0 to 24.7)
Ischemic heart disease	India	2000	All Ages	237.8 (201.9 to 277.6)	22.0% (18.9 to 25.3%)	22.6 (19.2 to 26.3)
Ischemic heart disease	India	2005	All Ages	268.5 (228.7 to 313.1)	22.3% (19.2 to 25.7%)	23.5 (20.0 to 27.4)
Ischemic heart disease	India	2010	All Ages	314.8 (266.2 to 366.9)	21.9% (18.7 to 25.3%)	25.6 (21.6 to 29.8)
Ischemic heart disease	India	2015	All Ages	365.6 (308.8 to 427.7)	22.7% (19.5 to 26.3%)	27.9 (23.5 to 32.6)
Ischemic heart disease	India	1990	Age-standardized		20.0% (16.7 to 23.9%)	43.8 (36.3 to 52.7)
Ischemic heart disease	India	1995	Age-standardized		20.1% (16.7 to 23.9%)	44.0 (36.3 to 53.0)
Ischemic heart disease	India	2000	Age-standardized		20.1% (16.8 to 23.9%)	44.1 (36.6 to 53.0)
Ischemic heart disease	India	2005	Age-standardized		20.5% (17.1 to 24.4%)	42.8 (35.7 to 51.6)
Ischemic heart disease	India	2010	Age-standardized		20.2% (16.8 to 24.1%)	43.2 (35.7 to 51.9)
Ischemic heart disease	India	2015	Age-standardized		21.0% (17.5 to 25.2%)	43.0 (35.5 to 51.5)
Ischemic stroke	India	1990	All Ages	35.1 (27.3 to 43.1)	16.7% (13.6 to 19.6%)	4.0 (3.1 to 4.9)
Ischemic stroke	India	1995	All Ages	40.1 (31.2 to 48.9)	16.7% (13.5 to 19.7%)	4.2 (3.2 to 5.1)
Ischemic stroke	India	2000	All Ages	44.8 (35.1 to 54.5)	16.7% (13.5 to 19.7%)	4.3 (3.3 to 5.2)
Ischemic stroke	India	2005	All Ages	49.5 (39.3 to 59.9)	17.1% (13.9 to 20.2%)	4.3 (3.4 to 5.2)
Ischemic stroke	India	2010	All Ages	55.6 (44.5 to 67.4)	16.8% (13.7 to 19.8%)	4.5 (3.6 to 5.5)
Ischemic stroke	India	2015	All Ages	64.8 (52.1 to 78.7)	17.6% (14.4 to 20.7%)	4.9 (4.0 to 6.0)
Ischemic stroke	India	1990	Age-standardized		15.4% (11.9 to 18.7%)	10.4 (7.8 to 12.8)
Ischemic stroke	India	1995	Age-standardized		15.5% (12.0 to 18.8%)	10.2 (7.7 to 12.6)
Ischemic stroke	India	2000	Age-standardized		15.5% (12.0 to 18.9%)	9.7 (7.4 to 12.1)
Ischemic stroke	India	2005	Age-standardized		16.0% (12.5 to 19.5%)	9.2 (7.1 to 11.4)
Ischemic stroke	India	2010	Age-standardized		15.7% (12.3 to 19.2%)	8.8 (6.8 to 10.9)
Ischemic stroke	India	2015	Age-standardized		16.6% (13.0 to 20.2%)	8.7 (6.7 to 10.8)
Hemorrhagic stroke	India	1990	All Ages	53.1 (44.1 to 63.6)	18.6% (15.7 to 21.4%)	6.1 (5.1 to 7.3)
Hemorrhagic stroke	India	1995	All Ages	60.3 (50.4 to 72.0)	18.8% (15.9 to 21.6%)	6.3 (5.2 to 7.5)
Hemorrhagic stroke	India	2000	All Ages	66.2 (55.3 to 78.5)	18.9% (16.0 to 21.7%)	6.3 (5.2 to 7.5)
Hemorrhagic stroke	India	2005	All Ages	70.6 (59.1 to 83.6)	19.5% (16.6 to 22.3%)	6.2 (5.2 to 7.3)
Hemorrhagic stroke	India	2010	All Ages	77.6 (64.5 to 92.3)	19.2% (16.3 to 22.2%)	6.3 (5.2 to 7.5)
Hemorrhagic stroke	India	2015	All Ages	87.7 (73.2 to 104.5)	20.2% (17.2 to 23.2%)	6.7 (5.6 to 8.0)
Hemorrhagic stroke	India	1990	Age-standardized		17.5% (14.7 to 20.4%)	11.9 (9.7 to 14.3)
Hemorrhagic stroke	India	1995	Age-standardized		17.6% (14.8 to 20.5%)	11.7 (9.7 to 14.2)
Hemorrhagic stroke	India	2000	Age-standardized		17.7% (14.9 to 20.5%)	11.2 (9.1 to 13.4)
Hemorrhagic stroke	India	2005	Age-standardized		18.2% (15.3 to 21.1%)	10.3 (8.5 to 12.3)
Hemorrhagic stroke	India	2010	Age-standardized		18.0% (15.0 to 20.8%)	9.8 (8.1 to 11.8)

Cause name	Location	Year	Age group	Number (in thousands)	PAF	Rate (per 100,000)
Hemorrhagic stroke	India	2015	Age-standardized		18.9% (15.9 to 21.9%)	9.6 (7.9 to 11.5)
Chronic obstructive pulmonary disease	India	1990	All Ages	200.9 (120.0 to 292.2)	29.6% (19.0 to 41.6%)	23.1 (13.8 to 33.6)
Chronic obstructive pulmonary disease	India	1995	All Ages	237.7 (144.2 to 339.6)	30.1% (19.3 to 42.2%)	24.7 (15.0 to 35.3)
Chronic obstructive pulmonary disease	India	2000	All Ages	271.3 (169.8 to 385.0)	30.5% (19.7 to 42.7%)	25.7 (16.1 to 36.5)
Chronic obstructive pulmonary disease	India	2005	All Ages	275.7 (177.8 to 387.9)	31.6% (20.4 to 44.1%)	24.1 (15.5 to 33.9)
Chronic obstructive pulmonary disease	India	2010	All Ages	295.5 (187.7 to 412.7)	31.3% (20.1 to 43.8%)	24.0 (15.3 to 33.5)
Chronic obstructive pulmonary disease	India	2015	All Ages	349.0 (227.7 to 482.8)	33.5% (22.1 to 46.9%)	26.6 (17.4 to 36.8)
Chronic obstructive pulmonary disease	India	1990	Age-standardized		30.7% (19.8 to 43.1%)	61.1 (36.4 to 88.7)
Chronic obstructive pulmonary disease	India	1995	Age-standardized		31.0% (19.9 to 43.4%)	62.1 (38.1 to 88.7)
Chronic obstructive pulmonary disease	India	2000	Age-standardized		31.1% (20.0 to 43.4%)	60.1 (37.8 to 85.5)
Chronic obstructive pulmonary disease	India	2005	Age-standardized		32.0% (20.7 to 44.6%)	52.1 (33.4 to 73.2)
Chronic obstructive pulmonary disease	India	2010	Age-standardized		31.6% (20.2 to 44.1%)	47.5 (30.3 to 66.2)
Chronic obstructive pulmonary disease	India	2015	Age-standardized		33.6% (22.2 to 47.0%)	47.5 (30.9 to 65.7)
All causes	Nepal	1990	All Ages	16.5 (13.5 to 19.8)	8.1% (6.7 to 9.6%)	88.0 (71.8 to 105.9)
All causes	Nepal	1995	All Ages	16.0 (13.2 to 19.1)	8.4% (7.0 to 9.8%)	75.0 (61.7 to 89.3)
All causes	Nepal	2000	All Ages	14.8 (12.5 to 17.3)	8.6% (7.3 to 10.0%)	62.5 (52.6 to 72.9)
All causes	Nepal	2005	All Ages	15.3 (12.7 to 18.0)	9.3% (7.9 to 10.7%)	59.9 (50.0 to 70.6)
All causes	Nepal	2010	All Ages	16.0 (13.0 to 19.3)	9.8% (8.3 to 11.2%)	59.7 (48.5 to 71.9)
All causes	Nepal	2015	All Ages	18.3 (14.6 to 22.5)	10.3% (8.7 to 12.0%)	64.2 (51.0 to 78.7)
All causes	Nepal	1990	Age-standardized		8.9% (7.5 to 10.5%)	135.5 (110.7 to 166.5)
All causes	Nepal	1995	Age-standardized		9.4% (7.9 to 11.0%)	127.8 (105.9 to 154.6)
All causes	Nepal	2000	Age-standardized		9.8% (8.3 to 11.4%)	114.8 (96.1 to 135.3)
All causes	Nepal	2005	Age-standardized		10.4% (8.7 to 12.1%)	110.9 (91.7 to 132.5)
All causes	Nepal	2010	Age-standardized		10.8% (9.1 to 12.6%)	109.7 (88.8 to 133.5)
All causes	Nepal	2015	Age-standardized		11.4% (9.5 to 13.3%)	113.2 (90.1 to 139.9)
Lower respiratory infections	Nepal	1990	All Ages	9.9 (7.4 to 12.8)	32.5% (25.1 to 39.5%)	52.8 (39.2 to 68.1)
Lower respiratory infections	Nepal	1995	All Ages	8.6 (6.4 to 11.0)	32.8% (25.1 to 39.7%)	40.0 (29.7 to 51.3)
Lower respiratory infections	Nepal	2000	All Ages	6.9 (5.1 to 8.9)	32.5% (24.9 to 39.5%)	29.0 (21.3 to 37.7)
Lower respiratory infections	Nepal	2005	All Ages	5.8 (4.3 to 7.6)	33.9% (26.1 to 41.0%)	22.8 (16.8 to 29.9)
Lower respiratory infections	Nepal	2010	All Ages	4.6 (3.2 to 6.3)	34.5% (26.6 to 41.7%)	17.2 (12.1 to 23.5)
Lower respiratory infections	Nepal	2015	All Ages	4.1 (2.8 to 5.7)	37.4% (29.2 to 44.8%)	14.5 (9.7 to 20.1)
Lower respiratory infections	Nepal	1990	Age-standardized		32.5% (25.1 to 39.5%)	41.8 (29.8 to 56.0)
Lower respiratory infections	Nepal	1995	Age-standardized		32.8% (25.1 to 39.7%)	35.2 (25.2 to 48.0)
Lower respiratory infections	Nepal	2000	Age-standardized		32.5% (24.9 to 39.5%)	29.0 (20.2 to 39.4)
Lower respiratory infections	Nepal	2005	Age-standardized		33.9% (26.1 to 41.0%)	26.2 (17.8 to 36.5)
Lower respiratory infections	Nepal	2010	Age-standardized		34.5% (26.6 to 41.7%)	23.0 (15.0 to 33.2)

Cause name	Location	Year	Age group	Number (in thousands)	PAF	Rate (per 100,000)
Lower respiratory infections	Nepal	2015	Age-standardized		37.4% (29.2 to 44.8%)	21.3 (13.4 to 31.0)
Tracheal, bronchus, and lung cancer	Nepal	1990	All Ages	0.1 (0.1 to 0.3)	24.2% (16.1 to 32.5%)	0.8 (0.4 to 1.4)
Tracheal, bronchus, and lung cancer	Nepal	1995	All Ages	0.2 (0.1 to 0.3)	24.5% (16.4 to 32.8%)	0.8 (0.4 to 1.3)
Tracheal, bronchus, and lung cancer	Nepal	2000	All Ages	0.2 (0.1 to 0.3)	24.3% (16.1 to 32.2%)	0.7 (0.4 to 1.2)
Tracheal, bronchus, and lung cancer	Nepal	2005	All Ages	0.2 (0.1 to 0.4)	25.5% (17.1 to 33.7%)	0.8 (0.5 to 1.4)
Tracheal, bronchus, and lung cancer	Nepal	2010	All Ages	0.3 (0.1 to 0.5)	26.1% (17.5 to 34.4%)	1.0 (0.6 to 1.7)
Tracheal, bronchus, and lung cancer	Nepal	2015	All Ages	0.4 (0.2 to 0.6)	28.9% (20.1 to 37.3%)	1.3 (0.7 to 2.2)
Tracheal, bronchus, and lung cancer	Nepal	1990	Age-standardized		24.3% (16.1 to 32.6%)	1.7 (0.9 to 3.2)
Tracheal, bronchus, and lung cancer	Nepal	1995	Age-standardized		24.6% (16.4 to 33.0%)	1.7 (0.9 to 3.1)
Tracheal, bronchus, and lung cancer	Nepal	2000	Age-standardized		24.4% (16.2 to 32.3%)	1.6 (0.9 to 2.9)
Tracheal, bronchus, and lung cancer	Nepal	2005	Age-standardized		25.5% (17.1 to 33.7%)	1.7 (0.9 to 2.9)
Tracheal, bronchus, and lung cancer	Nepal	2010	Age-standardized		26.1% (17.6 to 34.5%)	1.9 (1.0 to 3.2)
Tracheal, bronchus, and lung cancer	Nepal	2015	Age-standardized		29.0% (20.1 to 37.4%)	2.2 (1.2 to 3.7)
Cardiovascular diseases	Nepal	1990	All Ages	4.2 (3.3 to 5.4)	17.5% (15.5 to 19.8%)	22.5 (17.8 to 28.8)
Cardiovascular diseases	Nepal	1995	All Ages	4.7 (3.8 to 5.9)	17.6% (15.5 to 20.0%)	22.0 (17.8 to 27.4)
Cardiovascular diseases	Nepal	2000	All Ages	4.9 (3.9 to 6.0)	17.4% (15.3 to 19.7%)	20.6 (16.4 to 25.1)
Cardiovascular diseases	Nepal	2005	All Ages	5.7 (4.4 to 7.1)	17.8% (15.7 to 20.2%)	22.3 (17.3 to 28.0)
Cardiovascular diseases	Nepal	2010	All Ages	6.8 (5.2 to 8.8)	17.9% (15.7 to 20.4%)	25.3 (19.5 to 32.7)
Cardiovascular diseases	Nepal	2015	All Ages	8.4 (6.4 to 10.9)	18.9% (16.6 to 21.4%)	29.3 (22.3 to 38.1)
Cardiovascular diseases	Nepal	1990	Age-standardized		16.3% (14.0 to 18.8%)	56.3 (44.3 to 71.6)
Cardiovascular diseases	Nepal	1995	Age-standardized		16.4% (14.1 to 19.0%)	54.9 (44.1 to 68.4)
Cardiovascular diseases	Nepal	2000	Age-standardized		16.3% (14.0 to 18.9%)	49.8 (39.6 to 60.8)
Cardiovascular diseases	Nepal	2005	Age-standardized		16.6% (14.2 to 19.2%)	48.6 (37.7 to 60.4)
Cardiovascular diseases	Nepal	2010	Age-standardized		16.8% (14.4 to 19.5%)	49.7 (38.0 to 63.0)
Cardiovascular diseases	Nepal	2015	Age-standardized		17.7% (15.2 to 20.6%)	52.2 (40.3 to 67.3)
Ischemic heart disease	Nepal	1990	All Ages	3.0 (2.3 to 3.8)	22.0% (18.7 to 25.5%)	15.8 (12.3 to 20.3)
Ischemic heart disease	Nepal	1995	All Ages	3.3 (2.6 to 4.1)	21.8% (18.7 to 25.4%)	15.5 (12.3 to 19.4)
Ischemic heart disease	Nepal	2000	All Ages	3.5 (2.7 to 4.2)	21.5% (18.3 to 24.9%)	14.5 (11.5 to 17.9)
Ischemic heart disease	Nepal	2005	All Ages	4.0 (3.1 to 5.1)	21.7% (18.4 to 25.3%)	15.7 (12.2 to 19.8)
Ischemic heart disease	Nepal	2010	All Ages	4.8 (3.6 to 6.2)	21.7% (18.4 to 25.3%)	17.8 (13.5 to 23.2)
Ischemic heart disease	Nepal	2015	All Ages	5.8 (4.4 to 7.7)	22.6% (19.2 to 26.5%)	20.5 (15.3 to 26.8)
Ischemic heart disease	Nepal	1990	Age-standardized		19.8% (16.3 to 23.8%)	39.4 (30.3 to 51.3)
Ischemic heart disease	Nepal	1995	Age-standardized		19.8% (16.3 to 23.9%)	38.7 (30.3 to 48.6)
Ischemic heart disease	Nepal	2000	Age-standardized		19.6% (16.0 to 23.7%)	35.3 (27.5 to 44.0)
Ischemic heart disease	Nepal	2005	Age-standardized		20.0% (16.3 to 24.2%)	34.3 (26.3 to 43.1)
Ischemic heart disease	Nepal	2010	Age-standardized		20.1% (16.4 to 24.4%)	34.9 (26.0 to 45.1)

Cause name	Location	Year	Age group	Number (in thousands)	PAF	Rate (per 100,000)
Ischemic heart disease	Nepal	2015	Age-standardized		21.0% (17.2 to 25.6%)	36.4 (27.2 to 47.4)
Ischemic stroke	Nepal	1990	All Ages	0.5 (0.3 to 0.7)	16.9% (13.7 to 19.9%)	2.6 (1.8 to 3.7)
Ischemic stroke	Nepal	1995	All Ages	0.6 (0.4 to 0.7)	16.8% (13.6 to 19.9%)	2.6 (1.8 to 3.5)
Ischemic stroke	Nepal	2000	All Ages	0.6 (0.4 to 0.8)	16.5% (13.3 to 19.5%)	2.4 (1.7 to 3.2)
Ischemic stroke	Nepal	2005	All Ages	0.7 (0.5 to 0.9)	16.8% (13.5 to 20.1%)	2.7 (1.8 to 3.6)
Ischemic stroke	Nepal	2010	All Ages	0.8 (0.6 to 1.2)	16.9% (13.5 to 20.1%)	3.2 (2.2 to 4.3)
Ischemic stroke	Nepal	2015	All Ages	1.1 (0.7 to 1.5)	17.8% (14.4 to 21.2%)	3.7 (2.5 to 5.1)
Ischemic stroke	Nepal	1990	Age-standardized		15.4% (11.8 to 18.8%)	7.8 (5.2 to 10.8)
Ischemic stroke	Nepal	1995	Age-standardized		15.4% (11.9 to 19.0%)	7.5 (5.0 to 10.3)
Ischemic stroke	Nepal	2000	Age-standardized		15.2% (11.7 to 18.9%)	6.7 (4.6 to 9.1)
Ischemic stroke	Nepal	2005	Age-standardized		15.7% (11.9 to 19.4%)	6.7 (4.5 to 9.1)
Ischemic stroke	Nepal	2010	Age-standardized		15.8% (12.1 to 19.7%)	6.9 (4.7 to 9.4)
Ischemic stroke	Nepal	2015	Age-standardized		16.7% (12.8 to 20.6%)	7.4 (5.0 to 10.1)
Hemorrhagic stroke	Nepal	1990	All Ages	0.8 (0.5 to 1.1)	18.3% (15.4 to 21.2%)	4.1 (2.6 to 5.8)
Hemorrhagic stroke	Nepal	1995	All Ages	0.8 (0.5 to 1.2)	18.5% (15.6 to 21.3%)	4.0 (2.5 to 5.4)
Hemorrhagic stroke	Nepal	2000	All Ages	0.9 (0.5 to 1.2)	18.3% (15.5 to 21.1%)	3.6 (2.2 to 5.0)
Hemorrhagic stroke	Nepal	2005	All Ages	1.0 (0.6 to 1.4)	18.8% (15.9 to 21.7%)	3.9 (2.3 to 5.4)
Hemorrhagic stroke	Nepal	2010	All Ages	1.2 (0.7 to 1.7)	19.1% (16.1 to 22.1%)	4.4 (2.7 to 6.1)
Hemorrhagic stroke	Nepal	2015	All Ages	1.5 (1.0 to 2.0)	20.3% (17.2 to 23.4%)	5.1 (3.4 to 7.1)
Hemorrhagic stroke	Nepal	1990	Age-standardized		17.5% (14.5 to 20.4%)	9.0 (5.4 to 12.5)
Hemorrhagic stroke	Nepal	1995	Age-standardized		17.5% (14.6 to 20.4%)	8.8 (5.4 to 11.9)
Hemorrhagic stroke	Nepal	2000	Age-standardized		17.3% (14.4 to 20.3%)	7.8 (4.7 to 10.6)
Hemorrhagic stroke	Nepal	2005	Age-standardized		17.8% (14.6 to 20.8%)	7.6 (4.4 to 10.4)
Hemorrhagic stroke	Nepal	2010	Age-standardized		18.0% (14.9 to 21.1%)	7.9 (4.8 to 10.8)
Hemorrhagic stroke	Nepal	2015	Age-standardized		19.1% (15.9 to 22.2%)	8.4 (5.3 to 11.5)
Chronic obstructive pulmonary disease	Nepal	1990	All Ages	2.2 (1.1 to 3.6)	27.2% (17.0 to 38.5%)	11.9 (6.0 to 19.1)
Chronic obstructive pulmonary disease	Nepal	1995	All Ages	2.6 (1.4 to 4.0)	28.2% (17.4 to 39.9%)	12.2 (6.6 to 18.9)
Chronic obstructive pulmonary disease	Nepal	2000	All Ages	2.9 (1.7 to 4.3)	28.5% (17.5 to 40.5%)	12.2 (7.1 to 18.0)
Chronic obstructive pulmonary disease	Nepal	2005	All Ages	3.6 (2.1 to 5.3)	30.2% (19.0 to 42.6%)	13.9 (8.2 to 20.9)
Chronic obstructive pulmonary disease	Nepal	2010	All Ages	4.3 (2.5 to 6.6)	31.2% (20.0 to 44.3%)	16.1 (9.4 to 24.6)
Chronic obstructive pulmonary disease	Nepal	2015	All Ages	5.5 (3.2 to 8.1)	33.9% (22.3 to 48.1%)	19.1 (11.3 to 28.3)
Chronic obstructive pulmonary disease	Nepal	1990	Age-standardized		29.8% (18.6 to 42.0%)	35.7 (18.1 to 56.7)
Chronic obstructive pulmonary disease	Nepal	1995	Age-standardized		30.1% (18.8 to 42.7%)	36.0 (19.4 to 54.4)
Chronic obstructive pulmonary disease	Nepal	2000	Age-standardized		30.0% (18.8 to 42.6%)	34.3 (19.7 to 50.5)
Chronic obstructive pulmonary disease	Nepal	2005	Age-standardized		31.2% (19.8 to 44.3%)	34.4 (20.2 to 50.9)
Chronic obstructive pulmonary disease	Nepal	2010	Age-standardized		31.7% (20.3 to 45.2%)	35.2 (20.6 to 53.2)

Cause name	Location	Year	Age group	Number (in thousands)	PAF	Rate (per 100,000)
Chronic obstructive pulmonary disease	Nepal	2015	Age-standardized		34.2% (22.5 to 48.5%)	37.5 (22.5 to 55.9)
All causes	Pakistan	1990	All Ages	82.3 (69.3 to 96.1)	8.0% (6.9 to 9.2%)	76.2 (64.2 to 89.1)
All causes	Pakistan	1995	All Ages	98.2 (83.8 to 113.7)	8.6% (7.5 to 9.7%)	79.7 (68.0 to 92.3)
All causes	Pakistan	2000	All Ages	110.8 (94.9 to 127.0)	9.1% (8.0 to 10.2%)	79.9 (68.4 to 91.7)
All causes	Pakistan	2005	All Ages	115.7 (99.0 to 133.5)	8.5% (7.5 to 9.5%)	75.2 (64.3 to 86.7)
All causes	Pakistan	2010	All Ages	123.6 (104.4 to 144.6)	9.1% (8.0 to 10.2%)	72.6 (61.3 to 84.9)
All causes	Pakistan	2015	All Ages	135.1 (114.3 to 159.2)	9.5% (8.5 to 10.6%)	71.5 (60.5 to 84.2)
All causes	Pakistan	1990	Age-standardized		10.5% (9.1 to 12.0%)	138.6 (118.2 to 163.2)
All causes	Pakistan	1995	Age-standardized		10.9% (9.6 to 12.4%)	153.8 (129.7 to 180.1)
All causes	Pakistan	2000	Age-standardized		11.3% (10.0 to 12.8%)	157.7 (133.6 to 182.7)
All causes	Pakistan	2005	Age-standardized		10.6% (9.3 to 12.0%)	147.9 (125.9 to 171.7)
All causes	Pakistan	2010	Age-standardized		11.0% (9.6 to 12.4%)	140.2 (116.8 to 165.4)
All causes	Pakistan	2015	Age-standardized		11.3% (9.9 to 12.8%)	136.3 (113.7 to 163.5)
Lower respiratory infections	Pakistan	1990	All Ages	30.2 (22.0 to 38.9)	36.1% (28.1 to 43.6%)	28.0 (20.3 to 36.1)
Lower respiratory infections	Pakistan	1995	All Ages	28.6 (20.4 to 36.5)	36.0% (28.0 to 43.4%)	23.2 (16.6 to 29.6)
Lower respiratory infections	Pakistan	2000	All Ages	27.3 (19.8 to 34.9)	36.6% (28.6 to 44.3%)	19.7 (14.3 to 25.2)
Lower respiratory infections	Pakistan	2005	All Ages	24.8 (18.4 to 31.6)	35.0% (27.0 to 42.3%)	16.1 (12.0 to 20.5)
Lower respiratory infections	Pakistan	2010	All Ages	24.2 (17.7 to 31.1)	34.5% (26.7 to 41.7%)	14.2 (10.4 to 18.3)
Lower respiratory infections	Pakistan	2015	All Ages	22.4 (16.0 to 28.4)	35.5% (27.4 to 42.7%)	11.9 (8.5 to 15.0)
Lower respiratory infections	Pakistan	1990	Age-standardized		36.1% (28.1 to 43.6%)	21.2 (15.7 to 26.9)
Lower respiratory infections	Pakistan	1995	Age-standardized		36.0% (28.0 to 43.4%)	19.9 (14.7 to 25.1)
Lower respiratory infections	Pakistan	2000	Age-standardized		36.6% (28.6 to 44.3%)	19.0 (14.1 to 24.2)
Lower respiratory infections	Pakistan	2005	Age-standardized		35.0% (27.0 to 42.3%)	16.8 (12.5 to 21.4)
Lower respiratory infections	Pakistan	2010	Age-standardized		34.5% (26.7 to 41.7%)	15.1 (11.0 to 19.4)
Lower respiratory infections	Pakistan	2015	Age-standardized		35.5% (27.4 to 42.7%)	13.4 (9.6 to 17.2)
Tracheal, bronchus, and lung cancer	Pakistan	1990	All Ages	1.7 (1.1 to 2.3)	27.5% (18.9 to 35.9%)	1.6 (1.0 to 2.2)
Tracheal, bronchus, and lung cancer	Pakistan	1995	All Ages	2.3 (1.5 to 3.1)	27.3% (18.7 to 35.8%)	1.8 (1.2 to 2.5)
Tracheal, bronchus, and lung cancer	Pakistan	2000	All Ages	3.0 (2.0 to 4.1)	27.9% (19.3 to 36.3%)	2.2 (1.5 to 3.0)
Tracheal, bronchus, and lung cancer	Pakistan	2005	All Ages	3.3 (2.2 to 4.5)	26.5% (18.0 to 34.9%)	2.1 (1.4 to 2.9)
Tracheal, bronchus, and lung cancer	Pakistan	2010	All Ages	3.7 (2.4 to 5.2)	25.9% (17.5 to 34.3%)	2.2 (1.4 to 3.1)
Tracheal, bronchus, and lung cancer	Pakistan	2015	All Ages	4.4 (2.8 to 6.2)	26.8% (18.4 to 35.2%)	2.3 (1.5 to 3.3)
Tracheal, bronchus, and lung cancer	Pakistan	1990	Age-standardized		27.6% (19.0 to 35.9%)	3.4 (2.2 to 4.6)
Tracheal, bronchus, and lung cancer	Pakistan	1995	Age-standardized		27.4% (18.8 to 35.8%)	3.8 (2.5 to 5.2)
Tracheal, bronchus, and lung cancer	Pakistan	2000	Age-standardized		28.0% (19.3 to 36.4%)	4.5 (3.1 to 6.1)
Tracheal, bronchus, and lung cancer	Pakistan	2005	Age-standardized		26.5% (18.0 to 35.0%)	4.3 (2.9 to 5.8)
Tracheal, bronchus, and lung cancer	Pakistan	2010	Age-standardized		25.9% (17.5 to 34.3%)	4.2 (2.7 to 5.9)

Cause name	Location	Year	Age group	Number (in thousands)	PAF	Rate (per 100,000)
Tracheal, bronchus, and lung cancer	Pakistan	2015	Age-standardized		26.9% (18.4 to 35.3%)	4.3 (2.7 to 6.0)
Cardiovascular diseases	Pakistan	1990	All Ages	41.4 (34.1 to 49.8)	19.1% (16.9 to 21.5%)	38.3 (31.6 to 46.1)
Cardiovascular diseases	Pakistan	1995	All Ages	55.6 (46.4 to 65.6)	19.2% (17.0 to 21.8%)	45.1 (37.7 to 53.2)
Cardiovascular diseases	Pakistan	2000	All Ages	66.1 (56.0 to 78.1)	19.5% (17.2 to 22.1%)	47.7 (40.4 to 56.4)
Cardiovascular diseases	Pakistan	2005	All Ages	72.3 (60.8 to 85.5)	19.0% (16.7 to 21.6%)	46.9 (39.5 to 55.5)
Cardiovascular diseases	Pakistan	2010	All Ages	79.2 (64.8 to 95.3)	18.8% (16.5 to 21.4%)	46.5 (38.1 to 56.0)
Cardiovascular diseases	Pakistan	2015	All Ages	89.5 (73.8 to 109.1)	19.2% (16.9 to 22.0%)	47.4 (39.0 to 57.7)
Cardiovascular diseases	Pakistan	1990	Age-standardized		17.8% (15.4 to 20.8%)	91.1 (75.2 to 109.9)
Cardiovascular diseases	Pakistan	1995	Age-standardized		18.0% (15.6 to 20.9%)	104.6 (86.9 to 124.9)
Cardiovascular diseases	Pakistan	2000	Age-standardized		18.3% (15.9 to 21.3%)	107.7 (90.1 to 128.6)
Cardiovascular diseases	Pakistan	2005	Age-standardized		17.9% (15.5 to 20.8%)	102.4 (86.6 to 122.2)
Cardiovascular diseases	Pakistan	2010	Age-standardized		17.7% (15.3 to 20.6%)	97.9 (80.4 to 118.1)
Cardiovascular diseases	Pakistan	2015	Age-standardized		18.1% (15.6 to 21.0%)	96.0 (78.4 to 117.6)
Ischemic heart disease	Pakistan	1990	All Ages	29.7 (23.9 to 36.4)	22.7% (19.4 to 26.4%)	27.6 (22.1 to 33.8)
Ischemic heart disease	Pakistan	1995	All Ages	40.1 (32.9 to 48.2)	22.6% (19.5 to 26.3%)	32.5 (26.7 to 39.1)
Ischemic heart disease	Pakistan	2000	All Ages	48.2 (39.7 to 58.4)	22.8% (19.6 to 26.5%)	34.8 (28.6 to 42.1)
Ischemic heart disease	Pakistan	2005	All Ages	53.2 (43.7 to 64.3)	22.2% (19.0 to 25.9%)	34.6 (28.3 to 41.7)
Ischemic heart disease	Pakistan	2010	All Ages	58.6 (46.6 to 72.4)	22.1% (18.9 to 25.9%)	34.4 (27.3 to 42.5)
Ischemic heart disease	Pakistan	2015	All Ages	66.4 (53.5 to 82.2)	22.4% (19.1 to 26.3%)	35.1 (28.3 to 43.5)
Ischemic heart disease	Pakistan	1990	Age-standardized		20.9% (17.2 to 25.2%)	65.5 (52.1 to 81.3)
Ischemic heart disease	Pakistan	1995	Age-standardized		21.0% (17.5 to 25.3%)	75.4 (60.3 to 93.4)
Ischemic heart disease	Pakistan	2000	Age-standardized		21.2% (17.7 to 25.5%)	78.7 (63.7 to 97.2)
Ischemic heart disease	Pakistan	2005	Age-standardized		20.7% (17.2 to 24.9%)	75.5 (61.7 to 93.7)
Ischemic heart disease	Pakistan	2010	Age-standardized		20.5% (17.0 to 24.7%)	72.6 (57.0 to 91.0)
Ischemic heart disease	Pakistan	2015	Age-standardized		20.8% (17.3 to 25.2%)	71.3 (56.5 to 89.8)
Ischemic stroke	Pakistan	1990	All Ages	4.7 (3.4 to 6.3)	17.6% (14.3 to 20.8%)	4.4 (3.2 to 5.9)
Ischemic stroke	Pakistan	1995	All Ages	6.2 (4.6 to 8.3)	17.6% (14.3 to 20.7%)	5.0 (3.8 to 6.7)
Ischemic stroke	Pakistan	2000	All Ages	7.2 (5.4 to 9.4)	17.8% (14.5 to 21.0%)	5.2 (3.9 to 6.8)
Ischemic stroke	Pakistan	2005	All Ages	7.8 (5.8 to 9.8)	17.2% (13.9 to 20.3%)	5.0 (3.8 to 6.4)
Ischemic stroke	Pakistan	2010	All Ages	8.3 (6.4 to 10.6)	17.0% (13.7 to 20.1%)	4.9 (3.7 to 6.2)
Ischemic stroke	Pakistan	2015	All Ages	9.4 (7.0 to 12.0)	17.3% (14.0 to 20.5%)	4.9 (3.7 to 6.3)
Ischemic stroke	Pakistan	1990	Age-standardized		16.4% (12.7 to 20.2%)	11.9 (8.3 to 16.4)
Ischemic stroke	Pakistan	1995	Age-standardized		16.5% (12.8 to 20.2%)	13.3 (9.5 to 17.9)
Ischemic stroke	Pakistan	2000	Age-standardized		16.7% (13.1 to 20.4%)	13.3 (9.7 to 17.2)
Ischemic stroke	Pakistan	2005	Age-standardized		16.2% (12.7 to 19.8%)	12.3 (9.1 to 15.9)
Ischemic stroke	Pakistan	2010	Age-standardized		16.0% (12.4 to 19.7%)	11.6 (8.5 to 15.2)

Cause name	Location	Year	Age group	Number (in thousands)	PAF	Rate (per 100,000)
Ischemic stroke	Pakistan	2015	Age-standardized		16.3% (12.7 to 20.0%)	11.3 (8.2 to 14.6)
Hemorrhagic stroke	Pakistan	1990	All Ages	6.9 (4.9 to 9.4)	20.3% (17.3 to 23.3%)	6.4 (4.6 to 8.7)
Hemorrhagic stroke	Pakistan	1995	All Ages	9.3 (7.0 to 12.2)	20.3% (17.3 to 23.4%)	7.5 (5.7 to 9.9)
Hemorrhagic stroke	Pakistan	2000	All Ages	10.6 (8.1 to 13.7)	20.6% (17.6 to 23.7%)	7.7 (5.8 to 9.9)
Hemorrhagic stroke	Pakistan	2005	All Ages	11.3 (8.8 to 14.5)	20.0% (17.0 to 22.9%)	7.3 (5.7 to 9.4)
Hemorrhagic stroke	Pakistan	2010	All Ages	12.2 (9.4 to 15.6)	19.8% (16.8 to 22.7%)	7.2 (5.5 to 9.2)
Hemorrhagic stroke	Pakistan	2015	All Ages	13.8 (10.8 to 17.5)	20.3% (17.3 to 23.2%)	7.3 (5.7 to 9.2)
Hemorrhagic stroke	Pakistan	1990	Age-standardized		19.0% (15.9 to 22.0%)	13.8 (9.6 to 18.6)
Hemorrhagic stroke	Pakistan	1995	Age-standardized		19.0% (16.0 to 22.1%)	15.9 (11.8 to 20.8)
Hemorrhagic stroke	Pakistan	2000	Age-standardized		19.3% (16.3 to 22.5%)	15.8 (11.8 to 20.1)
Hemorrhagic stroke	Pakistan	2005	Age-standardized		18.7% (15.7 to 21.7%)	14.6 (11.2 to 18.6)
Hemorrhagic stroke	Pakistan	2010	Age-standardized		18.5% (15.5 to 21.5%)	13.8 (10.4 to 17.7)
Hemorrhagic stroke	Pakistan	2015	Age-standardized		18.9% (15.8 to 21.8%)	13.4 (10.3 to 17.1)
Chronic obstructive pulmonary disease	Pakistan	1990	All Ages	9.0 (5.2 to 13.4)	30.1% (19.6 to 42.5%)	8.3 (4.8 to 12.4)
Chronic obstructive pulmonary disease	Pakistan	1995	All Ages	11.8 (7.2 to 17.5)	30.7% (20.0 to 43.3%)	9.6 (5.9 to 14.2)
Chronic obstructive pulmonary disease	Pakistan	2000	All Ages	14.3 (9.0 to 20.7)	31.6% (20.4 to 44.6%)	10.3 (6.5 to 15.0)
Chronic obstructive pulmonary disease	Pakistan	2005	All Ages	15.4 (9.5 to 22.0)	30.6% (19.7 to 43.4%)	10.0 (6.2 to 14.3)
Chronic obstructive pulmonary disease	Pakistan	2010	All Ages	16.6 (10.1 to 23.7)	30.3% (19.3 to 42.8%)	9.7 (5.9 to 13.9)
Chronic obstructive pulmonary disease	Pakistan	2015	All Ages	18.8 (11.7 to 27.3)	31.3% (20.3 to 44.5%)	9.9 (6.2 to 14.4)
Chronic obstructive pulmonary disease	Pakistan	1990	Age-standardized		32.6% (21.2 to 46.1%)	22.9 (13.3 to 34.5)
Chronic obstructive pulmonary disease	Pakistan	1995	Age-standardized		32.6% (21.2 to 46.1%)	25.5 (15.7 to 38.1)
Chronic obstructive pulmonary disease	Pakistan	2000	Age-standardized		33.1% (21.4 to 46.4%)	26.4 (16.4 to 38.8)
Chronic obstructive pulmonary disease	Pakistan	2005	Age-standardized		31.9% (20.4 to 45.2%)	24.5 (15.0 to 35.4)
Chronic obstructive pulmonary disease	Pakistan	2010	Age-standardized		31.4% (20.0 to 44.6%)	23.0 (14.1 to 33.1)
Chronic obstructive pulmonary disease	Pakistan	2015	Age-standardized		32.3% (20.9 to 45.6%)	22.6 (13.8 to 32.8)
All causes	Botswana	1990	All Ages	0.4 (0.2 to 0.8)	3.9% (2.8 to 5.1%)	28.3 (12.5 to 59.7)
All causes	Botswana	1995	All Ages	0.5 (0.2 to 1.2)	2.9% (1.7 to 4.3%)	29.6 (10.9 to 74.1)
All causes	Botswana	2000	All Ages	0.6 (0.2 to 1.7)	2.1% (1.1 to 3.6%)	37.0 (11.6 to 99.9)
All causes	Botswana	2005	All Ages	0.7 (0.2 to 1.8)	2.2% (1.2 to 3.6%)	37.3 (11.6 to 98.6)
All causes	Botswana	2010	All Ages	0.6 (0.2 to 1.8)	2.4% (1.5 to 3.8%)	31.3 (11.0 to 88.2)
All causes	Botswana	2015	All Ages	0.7 (0.3 to 2.0)	2.6% (1.6 to 3.8%)	29.6 (11.3 to 86.3)
All causes	Botswana	1990	Age-standardized		5.5% (4.3 to 6.7%)	81.7 (36.0 to 166.4)
All causes	Botswana	1995	Age-standardized		4.4% (3.2 to 5.7%)	83.4 (33.0 to 191.6)
All causes	Botswana	2000	Age-standardized		3.4% (2.1 to 5.0%)	96.0 (33.8 to 227.8)
All causes	Botswana	2005	Age-standardized		3.5% (2.3 to 5.0%)	93.1 (32.9 to 219.0)
All causes	Botswana	2010	Age-standardized		3.7% (2.6 to 5.1%)	75.3 (29.8 to 187.2)

Cause name	Location	Year	Age group	Number (in thousands)	PAF	Rate (per 100,000)
All causes	Botswana	2015	Age-standardized		3.8% (2.7 to 4.9%)	66.7 (28.1 to 174.6)
Lower respiratory infections	Botswana	1990	All Ages	0.2 (0.1 to 0.3)	17.5% (9.6 to 27.2%)	11.8 (5.0 to 24.1)
Lower respiratory infections	Botswana	1995	All Ages	0.2 (0.1 to 0.4)	17.3% (9.5 to 26.9%)	10.8 (3.7 to 27.1)
Lower respiratory infections	Botswana	2000	All Ages	0.2 (0.1 to 0.6)	17.8% (9.9 to 27.3%)	12.6 (3.6 to 35.6)
Lower respiratory infections	Botswana	2005	All Ages	0.2 (0.1 to 0.6)	17.4% (9.5 to 27.1%)	12.0 (3.3 to 33.2)
Lower respiratory infections	Botswana	2010	All Ages	0.2 (0.1 to 0.6)	15.6% (8.1 to 25.6%)	9.6 (2.6 to 29.1)
Lower respiratory infections	Botswana	2015	All Ages	0.2 (0.1 to 0.6)	14.6% (6.9 to 24.4%)	8.7 (2.4 to 26.6)
Lower respiratory infections	Botswana	1990	Age-standardized		17.5% (9.6 to 27.2%)	23.8 (8.6 to 52.5)
Lower respiratory infections	Botswana	1995	Age-standardized		17.3% (9.5 to 26.9%)	23.7 (7.5 to 58.3)
Lower respiratory infections	Botswana	2000	Age-standardized		17.8% (9.9 to 27.3%)	26.9 (7.5 to 70.0)
Lower respiratory infections	Botswana	2005	Age-standardized		17.4% (9.5 to 27.1%)	25.4 (7.2 to 66.3)
Lower respiratory infections	Botswana	2010	Age-standardized		15.6% (8.1 to 25.6%)	20.2 (5.8 to 56.8)
Lower respiratory infections	Botswana	2015	Age-standardized		14.6% (6.9 to 24.4%)	17.4 (5.2 to 49.0)
Tracheal, bronchus, and lung cancer	Botswana	1990	All Ages	0.0 (0.0 to 0.0)	12.2% (6.9 to 18.4%)	0.7 (0.2 to 1.9)
Tracheal, bronchus, and lung cancer	Botswana	1995	All Ages	0.0 (0.0 to 0.0)	12.0% (6.7 to 18.4%)	0.9 (0.2 to 2.6)
Tracheal, bronchus, and lung cancer	Botswana	2000	All Ages	0.0 (0.0 to 0.1)	12.5% (6.9 to 18.9%)	1.2 (0.2 to 3.7)
Tracheal, bronchus, and lung cancer	Botswana	2005	All Ages	0.0 (0.0 to 0.1)	12.2% (6.8 to 18.3%)	1.3 (0.3 to 4.0)
Tracheal, bronchus, and lung cancer	Botswana	2010	All Ages	0.0 (0.0 to 0.1)	10.9% (5.8 to 16.8%)	1.2 (0.3 to 3.9)
Tracheal, bronchus, and lung cancer	Botswana	2015	All Ages	0.0 (0.0 to 0.1)	10.1% (5.4 to 15.8%)	1.2 (0.3 to 3.7)
Tracheal, bronchus, and lung cancer	Botswana	1990	Age-standardized		12.2% (6.9 to 18.4%)	2.1 (0.6 to 5.2)
Tracheal, bronchus, and lung cancer	Botswana	1995	Age-standardized		12.1% (6.7 to 18.5%)	2.4 (0.6 to 6.3)
Tracheal, bronchus, and lung cancer	Botswana	2000	Age-standardized		12.5% (6.9 to 18.9%)	2.9 (0.7 to 8.5)
Tracheal, bronchus, and lung cancer	Botswana	2005	Age-standardized		12.2% (6.8 to 18.3%)	3.1 (0.7 to 8.8)
Tracheal, bronchus, and lung cancer	Botswana	2010	Age-standardized		10.9% (5.8 to 16.8%)	2.6 (0.7 to 7.8)
Tracheal, bronchus, and lung cancer	Botswana	2015	Age-standardized		10.1% (5.4 to 15.8%)	2.4 (0.7 to 7.0)
Cardiovascular diseases	Botswana	1990	All Ages	0.2 (0.1 to 0.4)	10.6% (8.8 to 12.5%)	11.9 (4.3 to 28.1)
Cardiovascular diseases	Botswana	1995	All Ages	0.2 (0.1 to 0.6)	10.7% (8.9 to 12.5%)	13.9 (4.6 to 37.6)
Cardiovascular diseases	Botswana	2000	All Ages	0.3 (0.1 to 0.9)	11.0% (9.2 to 13.0%)	18.6 (5.4 to 51.6)
Cardiovascular diseases	Botswana	2005	All Ages	0.4 (0.1 to 1.0)	11.0% (9.2 to 12.9%)	19.4 (5.8 to 53.3)
Cardiovascular diseases	Botswana	2010	All Ages	0.3 (0.1 to 1.0)	10.4% (8.6 to 12.3%)	16.5 (5.7 to 46.7)
Cardiovascular diseases	Botswana	2015	All Ages	0.4 (0.1 to 1.1)	10.0% (8.3 to 11.9%)	16.0 (6.0 to 46.6)
Cardiovascular diseases	Botswana	1990	Age-standardized		9.9% (7.8 to 11.8%)	40.4 (16.2 to 86.5)
Cardiovascular diseases	Botswana	1995	Age-standardized		9.8% (7.8 to 11.7%)	43.0 (16.1 to 103.0)
Cardiovascular diseases	Botswana	2000	Age-standardized		10.2% (8.1 to 12.3%)	51.2 (17.1 to 126.5)
Cardiovascular diseases	Botswana	2005	Age-standardized		10.2% (8.2 to 12.2%)	50.5 (17.1 to 121.9)
Cardiovascular diseases	Botswana	2010	Age-standardized		9.6% (7.6 to 11.6%)	41.0 (15.8 to 102.8)

Cause name	Location	Year	Age group	Number (in thousands)	PAF	Rate (per 100,000)
Cardiovascular diseases	Botswana	2015	Age-standardized		9.2% (7.3 to 11.1%)	36.9 (15.3 to 97.2)
Ischemic heart disease	Botswana	1990	All Ages	0.1 (0.0 to 0.2)	16.3% (13.2 to 19.4%)	7.1 (2.6 to 17.0)
Ischemic heart disease	Botswana	1995	All Ages	0.1 (0.0 to 0.4)	16.4% (13.2 to 19.8%)	8.5 (2.8 to 23.4)
Ischemic heart disease	Botswana	2000	All Ages	0.2 (0.1 to 0.6)	16.7% (13.5 to 20.4%)	11.4 (3.2 to 32.6)
Ischemic heart disease	Botswana	2005	All Ages	0.2 (0.1 to 0.6)	16.5% (13.2 to 19.9%)	11.9 (3.4 to 32.9)
Ischemic heart disease	Botswana	2010	All Ages	0.2 (0.1 to 0.6)	15.5% (12.4 to 18.8%)	10.3 (3.3 to 29.7)
Ischemic heart disease	Botswana	2015	All Ages	0.2 (0.1 to 0.7)	15.0% (12.0 to 18.2%)	10.1 (3.6 to 30.0)
Ischemic heart disease	Botswana	1990	Age-standardized		14.4% (10.8 to 17.7%)	23.9 (9.4 to 52.3)
Ischemic heart disease	Botswana	1995	Age-standardized		14.5% (10.9 to 17.9%)	25.6 (9.3 to 63.3)
Ischemic heart disease	Botswana	2000	Age-standardized		15.0% (11.2 to 18.4%)	30.7 (9.8 to 76.9)
Ischemic heart disease	Botswana	2005	Age-standardized		14.8% (11.2 to 18.2%)	30.3 (9.8 to 73.0)
Ischemic heart disease	Botswana	2010	Age-standardized		13.9% (10.5 to 17.1%)	25.1 (9.1 to 63.0)
Ischemic heart disease	Botswana	2015	Age-standardized		13.4% (10.0 to 16.6%)	22.6 (8.9 to 59.7)
Ischemic stroke	Botswana	1990	All Ages	0.0 (0.0 to 0.1)	11.0% (8.0 to 14.1%)	1.8 (0.7 to 4.2)
Ischemic stroke	Botswana	1995	All Ages	0.0 (0.0 to 0.1)	11.0% (7.9 to 14.0%)	2.0 (0.7 to 5.3)
Ischemic stroke	Botswana	2000	All Ages	0.1 (0.0 to 0.1)	11.2% (8.1 to 14.4%)	2.6 (0.8 to 6.6)
Ischemic stroke	Botswana	2005	All Ages	0.1 (0.0 to 0.1)	11.0% (7.8 to 14.0%)	2.8 (0.9 to 6.8)
Ischemic stroke	Botswana	2010	All Ages	0.1 (0.0 to 0.1)	10.2% (7.3 to 13.1%)	2.3 (0.8 to 6.1)
Ischemic stroke	Botswana	2015	All Ages	0.1 (0.0 to 0.1)	9.7% (6.8 to 12.7%)	2.3 (0.9 to 5.9)
Ischemic stroke	Botswana	1990	Age-standardized		10.2% (6.9 to 13.5%)	7.6 (2.8 to 16.1)
Ischemic stroke	Botswana	1995	Age-standardized		10.1% (6.8 to 13.4%)	7.8 (2.7 to 18.2)
Ischemic stroke	Botswana	2000	Age-standardized		10.4% (7.1 to 13.8%)	9.0 (3.0 to 20.3)
Ischemic stroke	Botswana	2005	Age-standardized		10.2% (6.9 to 13.5%)	8.9 (2.9 to 19.9)
Ischemic stroke	Botswana	2010	Age-standardized		9.5% (6.3 to 13.0%)	7.2 (2.6 to 16.8)
Ischemic stroke	Botswana	2015	Age-standardized		9.1% (6.0 to 12.6%)	6.5 (2.5 to 15.6)
Hemorrhagic stroke	Botswana	1990	All Ages	0.0 (0.0 to 0.1)	12.5% (9.7 to 15.5%)	2.9 (0.9 to 7.2)
Hemorrhagic stroke	Botswana	1995	All Ages	0.1 (0.0 to 0.1)	12.5% (9.6 to 15.7%)	3.4 (1.0 to 9.6)
Hemorrhagic stroke	Botswana	2000	All Ages	0.1 (0.0 to 0.2)	12.8% (9.8 to 16.2%)	4.6 (1.1 to 13.6)
Hemorrhagic stroke	Botswana	2005	All Ages	0.1 (0.0 to 0.3)	12.5% (9.6 to 15.9%)	4.7 (1.2 to 13.6)
Hemorrhagic stroke	Botswana	2010	All Ages	0.1 (0.0 to 0.2)	11.7% (8.9 to 14.9%)	3.8 (1.1 to 11.8)
Hemorrhagic stroke	Botswana	2015	All Ages	0.1 (0.0 to 0.3)	11.2% (8.5 to 14.1%)	3.6 (1.2 to 11.4)
Hemorrhagic stroke	Botswana	1990	Age-standardized		11.4% (8.4 to 14.3%)	9.0 (3.0 to 20.3)
Hemorrhagic stroke	Botswana	1995	Age-standardized		11.4% (8.3 to 14.5%)	9.5 (3.0 to 24.4)
Hemorrhagic stroke	Botswana	2000	Age-standardized		11.7% (8.6 to 15.0%)	11.6 (3.3 to 31.3)
Hemorrhagic stroke	Botswana	2005	Age-standardized		11.5% (8.5 to 14.7%)	11.3 (3.3 to 30.0)
Hemorrhagic stroke	Botswana	2010	Age-standardized		10.7% (7.7 to 13.7%)	8.8 (2.9 to 24.5)

Cause name	Location	Year	Age group	Number (in thousands)	PAF	Rate (per 100,000)
Hemorrhagic stroke	Botswana	2015	Age-standardized		10.2% (7.4 to 13.1%)	7.8 (2.8 to 22.0)
Chronic obstructive pulmonary disease	Botswana	1990	All Ages	0.1 (0.0 to 0.1)	18.1% (8.8 to 28.5%)	3.9 (1.2 to 9.6)
Chronic obstructive pulmonary disease	Botswana	1995	All Ages	0.1 (0.0 to 0.2)	18.0% (8.8 to 28.5%)	3.9 (1.1 to 11.3)
Chronic obstructive pulmonary disease	Botswana	2000	All Ages	0.1 (0.0 to 0.2)	18.5% (9.2 to 28.9%)	4.6 (1.0 to 13.5)
Chronic obstructive pulmonary disease	Botswana	2005	All Ages	0.1 (0.0 to 0.3)	18.2% (8.8 to 28.3%)	4.6 (1.1 to 13.3)
Chronic obstructive pulmonary disease	Botswana	2010	All Ages	0.1 (0.0 to 0.2)	16.8% (7.8 to 27.0%)	4.0 (1.1 to 11.8)
Chronic obstructive pulmonary disease	Botswana	2015	All Ages	0.1 (0.0 to 0.3)	15.9% (7.4 to 26.2%)	3.7 (1.0 to 11.0)
Chronic obstructive pulmonary disease	Botswana	1990	Age-standardized		18.4% (8.9 to 28.9%)	15.4 (5.2 to 35.3)
Chronic obstructive pulmonary disease	Botswana	1995	Age-standardized		18.2% (8.9 to 28.9%)	14.3 (4.4 to 37.1)
Chronic obstructive pulmonary disease	Botswana	2000	Age-standardized		18.7% (9.3 to 29.3%)	14.9 (3.9 to 38.9)
Chronic obstructive pulmonary disease	Botswana	2005	Age-standardized		18.3% (8.9 to 28.6%)	14.0 (3.9 to 36.7)
Chronic obstructive pulmonary disease	Botswana	2010	Age-standardized		16.9% (7.8 to 27.1%)	11.4 (3.4 to 30.8)
Chronic obstructive pulmonary disease	Botswana	2015	Age-standardized		16.0% (7.4 to 26.3%)	10.0 (3.0 to 27.5)
All causes	Lesotho	1990	All Ages	0.7 (0.5 to 0.9)	4.4% (3.3 to 5.7%)	42.6 (30.5 to 56.6)
All causes	Lesotho	1995	All Ages	0.7 (0.5 to 1.0)	4.0% (3.1 to 5.2%)	41.9 (29.6 to 55.2)
All causes	Lesotho	2000	All Ages	0.9 (0.6 to 1.2)	3.3% (2.4 to 4.3%)	47.6 (33.3 to 64.4)
All causes	Lesotho	2005	All Ages	1.2 (0.9 to 1.6)	3.0% (2.3 to 4.0%)	61.3 (45.1 to 82.2)
All causes	Lesotho	2010	All Ages	1.1 (0.7 to 1.6)	3.0% (2.3 to 3.9%)	53.8 (36.0 to 78.0)
All causes	Lesotho	2015	All Ages	1.1 (0.7 to 1.8)	3.0% (2.3 to 4.0%)	53.5 (31.4 to 83.1)
All causes	Lesotho	1990	Age-standardized		5.3% (4.1 to 6.7%)	80.9 (58.5 to 107.9)
All causes	Lesotho	1995	Age-standardized		5.0% (3.9 to 6.3%)	80.8 (56.4 to 106.4)
All causes	Lesotho	2000	Age-standardized		4.2% (3.1 to 5.4%)	92.0 (65.4 to 126.6)
All causes	Lesotho	2005	Age-standardized		3.8% (2.9 to 4.9%)	117.3 (86.7 to 156.7)
All causes	Lesotho	2010	Age-standardized		3.9% (3.0 to 5.0%)	105.5 (70.0 to 151.6)
All causes	Lesotho	2015	Age-standardized		4.1% (3.1 to 5.2%)	111.0 (64.1 to 169.5)
Lower respiratory infections	Lesotho	1990	All Ages	0.3 (0.2 to 0.5)	19.0% (11.8 to 27.9%)	20.8 (12.5 to 31.7)
Lower respiratory infections	Lesotho	1995	All Ages	0.4 (0.2 to 0.5)	19.6% (12.7 to 28.5%)	20.0 (11.8 to 29.4)
Lower respiratory infections	Lesotho	2000	All Ages	0.4 (0.2 to 0.6)	20.2% (13.0 to 28.9%)	21.5 (12.8 to 32.1)
Lower respiratory infections	Lesotho	2005	All Ages	0.5 (0.3 to 0.7)	19.4% (12.0 to 28.0%)	24.2 (14.2 to 36.5)
Lower respiratory infections	Lesotho	2010	All Ages	0.4 (0.2 to 0.7)	17.3% (9.8 to 26.3%)	20.2 (10.7 to 32.5)
Lower respiratory infections	Lesotho	2015	All Ages	0.4 (0.2 to 0.7)	18.5% (10.8 to 27.6%)	19.1 (9.7 to 32.9)
Lower respiratory infections	Lesotho	1990	Age-standardized		19.0% (11.8 to 27.9%)	28.1 (16.1 to 44.4)
Lower respiratory infections	Lesotho	1995	Age-standardized		19.6% (12.7 to 28.5%)	28.4 (16.0 to 45.5)
Lower respiratory infections	Lesotho	2000	Age-standardized		20.2% (13.0 to 28.9%)	31.5 (18.7 to 48.2)
Lower respiratory infections	Lesotho	2005	Age-standardized		19.4% (12.0 to 28.0%)	36.1 (20.8 to 55.5)
Lower respiratory infections	Lesotho	2010	Age-standardized		17.3% (9.8 to 26.3%)	31.1 (16.4 to 51.1)

Cause name	Location	Year	Age group	Number (in thousands)	PAF	Rate (per 100,000)
Lower respiratory infections	Lesotho	2015	Age-standardized		18.5% (10.8 to 27.6%)	31.2 (15.1 to 54.8)
Tracheal, bronchus, and lung cancer	Lesotho	1990	All Ages	0.0 (0.0 to 0.0)	13.4% (7.9 to 19.6%)	1.0 (0.5 to 1.8)
Tracheal, bronchus, and lung cancer	Lesotho	1995	All Ages	0.0 (0.0 to 0.0)	13.9% (8.1 to 20.7%)	1.1 (0.5 to 1.9)
Tracheal, bronchus, and lung cancer	Lesotho	2000	All Ages	0.0 (0.0 to 0.0)	14.4% (8.2 to 21.7%)	1.2 (0.6 to 2.2)
Tracheal, bronchus, and lung cancer	Lesotho	2005	All Ages	0.0 (0.0 to 0.1)	13.8% (7.7 to 21.1%)	1.7 (0.8 to 2.8)
Tracheal, bronchus, and lung cancer	Lesotho	2010	All Ages	0.0 (0.0 to 0.1)	12.2% (6.7 to 18.9%)	1.5 (0.7 to 2.7)
Tracheal, bronchus, and lung cancer	Lesotho	2015	All Ages	0.0 (0.0 to 0.1)	13.0% (7.3 to 19.6%)	1.6 (0.7 to 3.0)
Tracheal, bronchus, and lung cancer	Lesotho	1990	Age-standardized		13.4% (7.9 to 19.6%)	2.1 (1.0 to 3.8)
Tracheal, bronchus, and lung cancer	Lesotho	1995	Age-standardized		13.9% (8.1 to 20.7%)	2.2 (1.1 to 4.0)
Tracheal, bronchus, and lung cancer	Lesotho	2000	Age-standardized		14.4% (8.2 to 21.7%)	2.6 (1.3 to 4.6)
Tracheal, bronchus, and lung cancer	Lesotho	2005	Age-standardized		13.8% (7.8 to 21.1%)	3.4 (1.8 to 5.7)
Tracheal, bronchus, and lung cancer	Lesotho	2010	Age-standardized		12.2% (6.7 to 19.0%)	3.2 (1.5 to 5.6)
Tracheal, bronchus, and lung cancer	Lesotho	2015	Age-standardized		13.1% (7.3 to 19.7%)	3.6 (1.5 to 6.6)
Cardiovascular diseases	Lesotho	1990	All Ages	0.2 (0.1 to 0.3)	10.1% (8.1 to 12.1%)	14.8 (8.9 to 21.0)
Cardiovascular diseases	Lesotho	1995	All Ages	0.3 (0.1 to 0.4)	10.2% (8.1 to 12.2%)	14.8 (8.6 to 21.3)
Cardiovascular diseases	Lesotho	2000	All Ages	0.3 (0.2 to 0.5)	10.4% (8.3 to 12.7%)	18.0 (11.1 to 26.6)
Cardiovascular diseases	Lesotho	2005	All Ages	0.5 (0.4 to 0.7)	10.5% (8.6 to 12.6%)	26.4 (18.7 to 36.5)
Cardiovascular diseases	Lesotho	2010	All Ages	0.5 (0.3 to 0.7)	9.9% (8.1 to 11.9%)	24.3 (15.8 to 35.9)
Cardiovascular diseases	Lesotho	2015	All Ages	0.5 (0.3 to 0.8)	10.4% (8.5 to 12.4%)	25.0 (13.7 to 40.1)
Cardiovascular diseases	Lesotho	1990	Age-standardized		9.7% (7.6 to 11.8%)	35.1 (21.2 to 49.4)
Cardiovascular diseases	Lesotho	1995	Age-standardized		9.8% (7.6 to 11.8%)	34.9 (20.6 to 49.9)
Cardiovascular diseases	Lesotho	2000	Age-standardized		10.1% (7.9 to 12.3%)	41.1 (25.3 to 59.8)
Cardiovascular diseases	Lesotho	2005	Age-standardized		10.3% (8.2 to 12.5%)	57.2 (40.9 to 78.7)
Cardiovascular diseases	Lesotho	2010	Age-standardized		9.8% (7.8 to 11.8%)	53.2 (35.0 to 78.2)
Cardiovascular diseases	Lesotho	2015	Age-standardized		10.2% (8.2 to 12.2%)	57.2 (32.4 to 90.7)
Ischemic heart disease	Lesotho	1990	All Ages	0.1 (0.1 to 0.2)	15.7% (12.8 to 18.8%)	8.3 (5.0 to 12.0)
Ischemic heart disease	Lesotho	1995	All Ages	0.1 (0.1 to 0.2)	15.8% (12.8 to 18.9%)	8.5 (4.8 to 12.2)
Ischemic heart disease	Lesotho	2000	All Ages	0.2 (0.1 to 0.3)	15.9% (12.9 to 19.3%)	10.4 (6.4 to 15.3)
Ischemic heart disease	Lesotho	2005	All Ages	0.3 (0.2 to 0.4)	15.8% (12.6 to 19.1%)	15.5 (10.8 to 21.2)
Ischemic heart disease	Lesotho	2010	All Ages	0.3 (0.2 to 0.4)	14.9% (11.9 to 18.1%)	14.5 (9.2 to 21.2)
Ischemic heart disease	Lesotho	2015	All Ages	0.3 (0.2 to 0.5)	15.5% (12.3 to 18.8%)	15.0 (8.0 to 23.9)
Ischemic heart disease	Lesotho	1990	Age-standardized		14.6% (11.4 to 17.8%)	19.7 (12.0 to 28.1)
Ischemic heart disease	Lesotho	1995	Age-standardized		14.8% (11.5 to 18.0%)	19.9 (11.1 to 28.7)
Ischemic heart disease	Lesotho	2000	Age-standardized		15.1% (11.8 to 18.5%)	23.7 (14.3 to 34.3)
Ischemic heart disease	Lesotho	2005	Age-standardized		15.2% (11.9 to 18.5%)	33.4 (23.1 to 45.0)
Ischemic heart disease	Lesotho	2010	Age-standardized		14.4% (11.4 to 17.7%)	31.5 (20.3 to 46.0)

Cause name	Location	Year	Age group	Number (in thousands)	PAF	Rate (per 100,000)
Ischemic heart disease	Lesotho	2015	Age-standardized		14.9% (11.6 to 18.2%)	34.2 (18.6 to 53.4)
Ischemic stroke	Lesotho	1990	All Ages	0.0 (0.0 to 0.1)	11.1% (8.1 to 14.0%)	2.8 (1.5 to 4.2)
Ischemic stroke	Lesotho	1995	All Ages	0.1 (0.0 to 0.1)	11.2% (8.1 to 14.0%)	2.8 (1.4 to 4.4)
Ischemic stroke	Lesotho	2000	All Ages	0.1 (0.0 to 0.1)	11.3% (8.2 to 14.4%)	3.4 (1.8 to 5.4)
Ischemic stroke	Lesotho	2005	All Ages	0.1 (0.1 to 0.1)	11.0% (7.9 to 14.3%)	4.7 (2.9 to 7.1)
Ischemic stroke	Lesotho	2010	All Ages	0.1 (0.1 to 0.1)	10.2% (7.2 to 13.3%)	4.2 (2.4 to 6.9)
Ischemic stroke	Lesotho	2015	All Ages	0.1 (0.1 to 0.2)	10.6% (7.5 to 14.0%)	4.3 (2.3 to 7.3)
Ischemic stroke	Lesotho	1990	Age-standardized		10.5% (7.3 to 13.7%)	7.3 (3.7 to 11.2)
Ischemic stroke	Lesotho	1995	Age-standardized		10.6% (7.4 to 13.9%)	7.2 (3.6 to 11.3)
Ischemic stroke	Lesotho	2000	Age-standardized		10.8% (7.5 to 14.1%)	8.3 (4.4 to 13.2)
Ischemic stroke	Lesotho	2005	Age-standardized		10.7% (7.4 to 14.0%)	10.9 (6.5 to 16.5)
Ischemic stroke	Lesotho	2010	Age-standardized		10.0% (6.8 to 13.2%)	9.8 (5.5 to 16.1)
Ischemic stroke	Lesotho	2015	Age-standardized		10.3% (7.1 to 13.8%)	10.4 (5.5 to 17.7)
Hemorrhagic stroke	Lesotho	1990	All Ages	0.1 (0.0 to 0.1)	12.3% (9.6 to 15.2%)	3.7 (2.0 to 5.7)
Hemorrhagic stroke	Lesotho	1995	All Ages	0.1 (0.0 to 0.1)	12.4% (9.7 to 15.4%)	3.5 (1.9 to 5.7)
Hemorrhagic stroke	Lesotho	2000	All Ages	0.1 (0.1 to 0.1)	12.6% (9.6 to 15.7%)	4.2 (2.4 to 6.9)
Hemorrhagic stroke	Lesotho	2005	All Ages	0.1 (0.1 to 0.2)	12.5% (9.6 to 15.6%)	6.2 (4.2 to 9.7)
Hemorrhagic stroke	Lesotho	2010	All Ages	0.1 (0.1 to 0.2)	11.6% (8.8 to 14.7%)	5.6 (3.3 to 9.0)
Hemorrhagic stroke	Lesotho	2015	All Ages	0.1 (0.1 to 0.2)	12.2% (9.3 to 15.2%)	5.7 (2.8 to 10.1)
Hemorrhagic stroke	Lesotho	1990	Age-standardized		11.7% (8.9 to 14.6%)	8.1 (4.4 to 12.5)
Hemorrhagic stroke	Lesotho	1995	Age-standardized		11.9% (9.1 to 14.7%)	7.8 (4.2 to 12.4)
Hemorrhagic stroke	Lesotho	2000	Age-standardized		12.2% (9.2 to 15.4%)	9.1 (5.3 to 14.8)
Hemorrhagic stroke	Lesotho	2005	Age-standardized		12.1% (9.2 to 15.2%)	12.9 (8.6 to 19.8)
Hemorrhagic stroke	Lesotho	2010	Age-standardized		11.3% (8.5 to 14.4%)	11.8 (6.9 to 19.3)
Hemorrhagic stroke	Lesotho	2015	Age-standardized		11.8% (8.9 to 14.9%)	12.6 (6.3 to 22.2)
Chronic obstructive pulmonary disease	Lesotho	1990	All Ages	0.1 (0.0 to 0.2)	19.1% (9.8 to 29.5%)	6.0 (2.7 to 10.7)
Chronic obstructive pulmonary disease	Lesotho	1995	All Ages	0.1 (0.1 to 0.2)	19.7% (10.4 to 30.2%)	6.0 (2.7 to 10.3)
Chronic obstructive pulmonary disease	Lesotho	2000	All Ages	0.1 (0.1 to 0.2)	20.2% (10.7 to 31.0%)	6.9 (3.2 to 11.6)
Chronic obstructive pulmonary disease	Lesotho	2005	All Ages	0.2 (0.1 to 0.3)	19.6% (10.6 to 30.3%)	9.1 (4.5 to 15.0)
Chronic obstructive pulmonary disease	Lesotho	2010	All Ages	0.2 (0.1 to 0.3)	17.8% (8.9 to 27.9%)	7.8 (3.5 to 13.7)
Chronic obstructive pulmonary disease	Lesotho	2015	All Ages	0.2 (0.1 to 0.3)	18.8% (9.4 to 28.6%)	7.7 (3.2 to 14.1)
Chronic obstructive pulmonary disease	Lesotho	1990	Age-standardized		19.3% (10.0 to 29.8%)	15.5 (7.3 to 27.5)
Chronic obstructive pulmonary disease	Lesotho	1995	Age-standardized		19.9% (10.5 to 30.4%)	15.2 (6.9 to 26.3)
Chronic obstructive pulmonary disease	Lesotho	2000	Age-standardized		20.4% (10.8 to 31.3%)	16.7 (7.7 to 28.4)
Chronic obstructive pulmonary disease	Lesotho	2005	Age-standardized		19.7% (10.7 to 30.5%)	20.6 (10.3 to 33.6)
Chronic obstructive pulmonary disease	Lesotho	2010	Age-standardized		18.0% (9.0 to 28.2%)	18.1 (8.1 to 31.4)

Cause name	Location	Year	Age group	Number (in thousands)	PAF	Rate (per 100,000)
Chronic obstructive pulmonary disease	Lesotho	2015	Age-standardized		18.9% (9.5 to 28.8%)	18.9 (8.1 to 34.0)
All causes	Namibia	1990	All Ages	0.6 (0.4 to 0.7)	4.8% (3.7 to 6.0%)	40.2 (30.0 to 51.6)
All causes	Namibia	1995	All Ages	0.6 (0.5 to 0.8)	4.4% (3.5 to 5.5%)	38.9 (29.7 to 49.5)
All causes	Namibia	2000	All Ages	0.9 (0.7 to 1.2)	3.9% (3.1 to 5.0%)	47.5 (35.4 to 61.7)
All causes	Namibia	2005	All Ages	0.8 (0.6 to 1.1)	3.2% (2.4 to 4.2%)	40.2 (27.9 to 56.1)
All causes	Namibia	2010	All Ages	0.6 (0.4 to 0.9)	3.4% (2.4 to 4.4%)	28.3 (18.1 to 41.2)
All causes	Namibia	2015	All Ages	0.6 (0.4 to 1.0)	3.4% (2.4 to 4.4%)	25.0 (15.0 to 39.5)
All causes	Namibia	1990	Age-standardized		6.3% (5.1 to 7.8%)	96.9 (74.8 to 122.0)
All causes	Namibia	1995	Age-standardized		6.0% (4.9 to 7.3%)	99.5 (77.3 to 124.7)
All causes	Namibia	2000	Age-standardized		5.6% (4.5 to 6.7%)	121.5 (91.8 to 154.9)
All causes	Namibia	2005	Age-standardized		4.8% (3.8 to 5.9%)	101.9 (73.2 to 140.7)
All causes	Namibia	2010	Age-standardized		4.9% (3.7 to 6.3%)	69.6 (45.2 to 101.6)
All causes	Namibia	2015	Age-standardized		4.8% (3.7 to 6.1%)	60.8 (38.2 to 94.2)
Lower respiratory infections	Namibia	1990	All Ages	0.2 (0.1 to 0.4)	19.9% (12.2 to 29.3%)	17.2 (10.2 to 26.2)
Lower respiratory infections	Namibia	1995	All Ages	0.2 (0.1 to 0.4)	19.4% (11.6 to 28.8%)	14.8 (8.5 to 22.5)
Lower respiratory infections	Namibia	2000	All Ages	0.3 (0.2 to 0.5)	20.5% (12.8 to 29.9%)	16.6 (9.9 to 25.1)
Lower respiratory infections	Namibia	2005	All Ages	0.3 (0.2 to 0.5)	19.6% (11.6 to 29.2%)	14.3 (8.0 to 22.8)
Lower respiratory infections	Namibia	2010	All Ages	0.2 (0.1 to 0.4)	18.0% (10.1 to 27.5%)	11.1 (5.7 to 18.9)
Lower respiratory infections	Namibia	2015	All Ages	0.2 (0.1 to 0.4)	17.1% (9.1 to 26.5%)	9.2 (4.3 to 16.8)
Lower respiratory infections	Namibia	1990	Age-standardized		19.9% (12.2 to 29.3%)	29.6 (17.1 to 46.2)
Lower respiratory infections	Namibia	1995	Age-standardized		19.4% (11.6 to 28.8%)	28.9 (16.2 to 44.7)
Lower respiratory infections	Namibia	2000	Age-standardized		20.5% (12.8 to 29.9%)	34.2 (20.4 to 52.7)
Lower respiratory infections	Namibia	2005	Age-standardized		19.6% (11.6 to 29.2%)	29.4 (16.3 to 47.4)
Lower respiratory infections	Namibia	2010	Age-standardized		18.0% (10.1 to 27.5%)	21.7 (10.4 to 37.9)
Lower respiratory infections	Namibia	2015	Age-standardized		17.1% (9.1 to 26.5%)	18.3 (8.3 to 33.7)
Tracheal, bronchus, and lung cancer	Namibia	1990	All Ages	0.0 (0.0 to 0.0)	14.0% (8.0 to 20.8%)	0.3 (0.1 to 0.5)
Tracheal, bronchus, and lung cancer	Namibia	1995	All Ages	0.0 (0.0 to 0.0)	13.6% (7.7 to 20.2%)	0.3 (0.2 to 0.5)
Tracheal, bronchus, and lung cancer	Namibia	2000	All Ages	0.0 (0.0 to 0.0)	14.5% (8.3 to 21.2%)	0.4 (0.2 to 0.6)
Tracheal, bronchus, and lung cancer	Namibia	2005	All Ages	0.0 (0.0 to 0.0)	13.8% (7.7 to 20.6%)	0.3 (0.2 to 0.6)
Tracheal, bronchus, and lung cancer	Namibia	2010	All Ages	0.0 (0.0 to 0.0)	12.6% (7.1 to 19.2%)	0.3 (0.1 to 0.4)
Tracheal, bronchus, and lung cancer	Namibia	2015	All Ages	0.0 (0.0 to 0.0)	11.9% (6.4 to 18.2%)	0.2 (0.1 to 0.4)
Tracheal, bronchus, and lung cancer	Namibia	1990	Age-standardized		14.0% (8.0 to 20.8%)	0.7 (0.4 to 1.2)
Tracheal, bronchus, and lung cancer	Namibia	1995	Age-standardized		13.7% (7.8 to 20.2%)	0.8 (0.4 to 1.2)
Tracheal, bronchus, and lung cancer	Namibia	2000	Age-standardized		14.5% (8.3 to 21.3%)	1.0 (0.5 to 1.5)
Tracheal, bronchus, and lung cancer	Namibia	2005	Age-standardized		13.8% (7.8 to 20.7%)	0.8 (0.4 to 1.4)
Tracheal, bronchus, and lung cancer	Namibia	2010	Age-standardized		12.6% (7.1 to 19.3%)	0.6 (0.3 to 1.1)

Cause name	Location	Year	Age group	Number (in thousands)	PAF	Rate (per 100,000)
Tracheal, bronchus, and lung cancer	Namibia	2015	Age-standardized		11.9% (6.4 to 18.2%)	0.6 (0.2 to 1.0)
Cardiovascular diseases	Namibia	1990	All Ages	0.2 (0.2 to 0.3)	11.4% (9.5 to 13.3%)	17.2 (12.4 to 21.7)
Cardiovascular diseases	Namibia	1995	All Ages	0.3 (0.2 to 0.4)	11.2% (9.5 to 13.1%)	18.2 (14.0 to 23.0)
Cardiovascular diseases	Namibia	2000	All Ages	0.5 (0.3 to 0.6)	11.9% (10.0 to 13.7%)	23.8 (17.6 to 30.9)
Cardiovascular diseases	Namibia	2005	All Ages	0.4 (0.3 to 0.6)	11.6% (9.8 to 13.5%)	19.9 (14.0 to 27.5)
Cardiovascular diseases	Namibia	2010	All Ages	0.3 (0.2 to 0.4)	10.8% (8.9 to 12.7%)	13.1 (8.1 to 19.8)
Cardiovascular diseases	Namibia	2015	All Ages	0.3 (0.2 to 0.5)	10.6% (8.7 to 12.5%)	12.1 (7.3 to 19.6)
Cardiovascular diseases	Namibia	1990	Age-standardized		10.8% (8.9 to 12.8%)	48.8 (35.3 to 62.4)
Cardiovascular diseases	Namibia	1995	Age-standardized		10.6% (8.8 to 12.5%)	51.9 (39.6 to 65.9)
Cardiovascular diseases	Namibia	2000	Age-standardized		11.2% (9.3 to 13.0%)	65.7 (49.1 to 84.4)
Cardiovascular diseases	Namibia	2005	Age-standardized		10.9% (9.0 to 12.9%)	54.5 (38.9 to 75.3)
Cardiovascular diseases	Namibia	2010	Age-standardized		10.2% (8.2 to 12.2%)	35.8 (22.4 to 53.3)
Cardiovascular diseases	Namibia	2015	Age-standardized		9.9% (7.9 to 11.9%)	31.9 (19.4 to 49.6)
Ischemic heart disease	Namibia	1990	All Ages	0.1 (0.1 to 0.2)	16.7% (13.7 to 19.8%)	10.8 (7.7 to 13.9)
Ischemic heart disease	Namibia	1995	All Ages	0.2 (0.1 to 0.2)	16.5% (13.6 to 19.6%)	11.4 (8.4 to 14.7)
Ischemic heart disease	Namibia	2000	All Ages	0.3 (0.2 to 0.4)	17.3% (14.2 to 20.4%)	14.8 (10.8 to 19.6)
Ischemic heart disease	Namibia	2005	All Ages	0.3 (0.2 to 0.4)	16.7% (13.6 to 19.8%)	12.5 (8.7 to 17.5)
Ischemic heart disease	Namibia	2010	All Ages	0.2 (0.1 to 0.3)	15.7% (12.8 to 18.9%)	8.4 (5.1 to 12.6)
Ischemic heart disease	Namibia	2015	All Ages	0.2 (0.1 to 0.3)	15.3% (12.3 to 18.5%)	7.9 (4.6 to 12.8)
Ischemic heart disease	Namibia	1990	Age-standardized		15.3% (12.1 to 18.5%)	30.5 (21.5 to 39.9)
Ischemic heart disease	Namibia	1995	Age-standardized		15.2% (11.9 to 18.4%)	32.1 (23.7 to 42.2)
Ischemic heart disease	Namibia	2000	Age-standardized		15.8% (12.5 to 19.1%)	40.5 (29.4 to 53.4)
Ischemic heart disease	Namibia	2005	Age-standardized		15.3% (12.0 to 18.7%)	33.8 (23.3 to 47.3)
Ischemic heart disease	Namibia	2010	Age-standardized		14.4% (11.2 to 17.8%)	22.4 (13.9 to 34.1)
Ischemic heart disease	Namibia	2015	Age-standardized		14.0% (10.8 to 17.4%)	20.3 (11.9 to 32.8)
Ischemic stroke	Namibia	1990	All Ages	0.0 (0.0 to 0.1)	11.7% (8.5 to 14.6%)	2.6 (1.7 to 3.6)
Ischemic stroke	Namibia	1995	All Ages	0.1 (0.0 to 0.1)	11.5% (8.3 to 14.4%)	2.7 (1.8 to 3.7)
Ischemic stroke	Namibia	2000	All Ages	0.1 (0.0 to 0.1)	11.9% (8.8 to 15.0%)	3.5 (2.3 to 4.8)
Ischemic stroke	Namibia	2005	All Ages	0.1 (0.0 to 0.1)	11.5% (8.2 to 14.6%)	2.9 (1.9 to 4.3)
Ischemic stroke	Namibia	2010	All Ages	0.0 (0.0 to 0.1)	10.7% (7.5 to 13.9%)	2.0 (1.2 to 3.0)
Ischemic stroke	Namibia	2015	All Ages	0.0 (0.0 to 0.1)	10.3% (7.2 to 13.3%)	1.8 (1.0 to 2.9)
Ischemic stroke	Namibia	1990	Age-standardized		11.0% (7.7 to 14.2%)	8.4 (5.4 to 11.6)
Ischemic stroke	Namibia	1995	Age-standardized		10.8% (7.5 to 14.1%)	8.9 (5.8 to 12.2)
Ischemic stroke	Namibia	2000	Age-standardized		11.3% (8.0 to 14.5%)	11.1 (7.1 to 15.5)
Ischemic stroke	Namibia	2005	Age-standardized		10.9% (7.5 to 14.4%)	9.2 (6.0 to 13.8)
Ischemic stroke	Namibia	2010	Age-standardized		10.1% (6.9 to 13.7%)	6.2 (3.6 to 9.5)

Cause name	Location	Year	Age group	Number (in thousands)	PAF	Rate (per 100,000)
Ischemic stroke	Namibia	2015	Age-standardized		9.8% (6.6 to 13.2%)	5.4 (3.0 to 9.0)
Hemorrhagic stroke	Namibia	1990	All Ages	0.1 (0.0 to 0.1)	13.0% (10.1 to 16.0%)	3.8 (2.3 to 5.3)
Hemorrhagic stroke	Namibia	1995	All Ages	0.1 (0.0 to 0.1)	13.0% (10.1 to 16.0%)	4.1 (2.7 to 5.6)
Hemorrhagic stroke	Namibia	2000	All Ages	0.1 (0.1 to 0.1)	13.6% (10.7 to 16.7%)	5.5 (3.7 to 7.7)
Hemorrhagic stroke	Namibia	2005	All Ages	0.1 (0.1 to 0.1)	13.0% (10.1 to 16.1%)	4.5 (2.9 to 6.7)
Hemorrhagic stroke	Namibia	2010	All Ages	0.1 (0.0 to 0.1)	12.0% (9.2 to 15.2%)	2.8 (1.6 to 4.5)
Hemorrhagic stroke	Namibia	2015	All Ages	0.1 (0.0 to 0.1)	11.7% (8.9 to 14.8%)	2.5 (1.4 to 4.4)
Hemorrhagic stroke	Namibia	1990	Age-standardized		12.2% (9.3 to 15.2%)	10.0 (6.1 to 14.2)
Hemorrhagic stroke	Namibia	1995	Age-standardized		12.1% (9.1 to 15.1%)	10.8 (7.1 to 15.1)
Hemorrhagic stroke	Namibia	2000	Age-standardized		12.7% (9.7 to 15.6%)	14.1 (9.6 to 19.8)
Hemorrhagic stroke	Namibia	2005	Age-standardized		12.2% (9.2 to 15.2%)	11.5 (7.6 to 17.0)
Hemorrhagic stroke	Namibia	2010	Age-standardized		11.3% (8.3 to 14.3%)	7.2 (4.1 to 11.3)
Hemorrhagic stroke	Namibia	2015	Age-standardized		10.9% (7.9 to 13.8%)	6.2 (3.4 to 10.5)
Chronic obstructive pulmonary disease	Namibia	1990	All Ages	0.1 (0.0 to 0.1)	20.0% (10.5 to 30.5%)	5.6 (2.7 to 9.0)
Chronic obstructive pulmonary disease	Namibia	1995	All Ages	0.1 (0.1 to 0.1)	19.7% (10.2 to 30.4%)	5.7 (2.8 to 8.9)
Chronic obstructive pulmonary disease	Namibia	2000	All Ages	0.1 (0.1 to 0.2)	20.6% (11.2 to 31.2%)	6.7 (3.3 to 10.6)
Chronic obstructive pulmonary disease	Namibia	2005	All Ages	0.1 (0.1 to 0.2)	19.9% (10.4 to 30.5%)	5.6 (2.8 to 9.3)
Chronic obstructive pulmonary disease	Namibia	2010	All Ages	0.1 (0.0 to 0.1)	18.6% (9.1 to 28.9%)	3.8 (1.7 to 6.6)
Chronic obstructive pulmonary disease	Namibia	2015	All Ages	0.1 (0.0 to 0.2)	17.9% (8.5 to 28.1%)	3.4 (1.4 to 6.4)
Chronic obstructive pulmonary disease	Namibia	1990	Age-standardized		20.3% (10.6 to 30.8%)	17.8 (8.7 to 28.6)
Chronic obstructive pulmonary disease	Namibia	1995	Age-standardized		19.9% (10.3 to 30.5%)	17.9 (9.0 to 28.2)
Chronic obstructive pulmonary disease	Namibia	2000	Age-standardized		20.8% (11.2 to 31.4%)	20.6 (10.2 to 32.0)
Chronic obstructive pulmonary disease	Namibia	2005	Age-standardized		20.1% (10.4 to 30.8%)	17.1 (8.5 to 28.3)
Chronic obstructive pulmonary disease	Namibia	2010	Age-standardized		18.7% (9.2 to 29.1%)	11.6 (5.2 to 20.0)
Chronic obstructive pulmonary disease	Namibia	2015	Age-standardized		18.0% (8.6 to 28.3%)	10.1 (4.2 to 18.6)
All causes	South Africa	1990	All Ages	20.6 (17.2 to 24.4)	5.9% (4.9 to 6.9%)	54.9 (45.9 to 64.8)
All causes	South Africa	1995	All Ages	22.4 (18.8 to 26.4)	5.8% (4.9 to 6.8%)	53.4 (44.8 to 63.1)
All causes	South Africa	2000	All Ages	28.4 (23.7 to 33.3)	4.9% (4.2 to 5.7%)	63.0 (52.5 to 73.9)
All causes	South Africa	2005	All Ages	28.0 (22.5 to 34.1)	3.8% (3.2 to 4.5%)	57.7 (46.4 to 70.4)
All causes	South Africa	2010	All Ages	23.3 (18.6 to 28.7)	3.2% (2.6 to 3.9%)	45.7 (36.5 to 56.5)
All causes	South Africa	2015	All Ages	24.9 (20.6 to 29.8)	4.2% (3.5 to 5.0%)	46.3 (38.3 to 55.5)
All causes	South Africa	1990	Age-standardized		6.9% (5.9 to 8.1%)	102.3 (86.8 to 120.4)
All causes	South Africa	1995	Age-standardized		6.8% (5.8 to 7.9%)	99.0 (83.5 to 116.2)
All causes	South Africa	2000	Age-standardized		6.1% (5.2 to 7.0%)	115.6 (96.0 to 136.4)
All causes	South Africa	2005	Age-standardized		4.9% (4.1 to 5.7%)	99.2 (80.5 to 119.9)
All causes	South Africa	2010	Age-standardized		4.1% (3.4 to 4.9%)	75.5 (60.8 to 92.8)

Cause name	Location	Year	Age group	Number (in thousands)	PAF	Rate (per 100,000)
All causes	South Africa	2015	Age-standardized		5.1% (4.3 to 6.0%)	73.9 (61.0 to 88.3)
Lower respiratory infections	South Africa	1990	All Ages	8.2 (5.6 to 11.1)	24.8% (16.8 to 33.4%)	21.7 (14.8 to 29.4)
Lower respiratory infections	South Africa	1995	All Ages	8.2 (5.6 to 11.0)	25.2% (17.5 to 33.6%)	19.5 (13.4 to 26.4)
Lower respiratory infections	South Africa	2000	All Ages	9.6 (6.5 to 13.2)	25.6% (17.9 to 33.8%)	21.3 (14.5 to 29.3)
Lower respiratory infections	South Africa	2005	All Ages	10.2 (6.7 to 14.3)	24.9% (17.0 to 33.3%)	21.1 (13.9 to 29.4)
Lower respiratory infections	South Africa	2010	All Ages	8.0 (4.8 to 11.8)	21.8% (13.5 to 31.3%)	15.8 (9.5 to 23.1)
Lower respiratory infections	South Africa	2015	All Ages	7.8 (4.7 to 11.3)	22.1% (13.8 to 31.6%)	14.5 (8.8 to 21.1)
Lower respiratory infections	South Africa	1990	Age-standardized		24.9% (16.9 to 33.4%)	31.0 (21.0 to 42.1)
Lower respiratory infections	South Africa	1995	Age-standardized		25.2% (17.4 to 33.6%)	29.5 (20.0 to 39.8)
Lower respiratory infections	South Africa	2000	Age-standardized		25.5% (17.7 to 33.7%)	33.2 (22.2 to 45.5)
Lower respiratory infections	South Africa	2005	Age-standardized		24.7% (16.8 to 33.3%)	31.3 (20.3 to 43.9)
Lower respiratory infections	South Africa	2010	Age-standardized		21.6% (13.4 to 31.2%)	23.4 (14.0 to 34.5)
Lower respiratory infections	South Africa	2015	Age-standardized		22.0% (13.8 to 31.5%)	21.4 (12.9 to 31.1)
Tracheal, bronchus, and lung cancer	South Africa	1990	All Ages	0.9 (0.5 to 1.3)	17.9% (10.9 to 25.3%)	2.4 (1.4 to 3.4)
Tracheal, bronchus, and lung cancer	South Africa	1995	All Ages	1.0 (0.6 to 1.5)	18.1% (11.2 to 25.5%)	2.4 (1.4 to 3.5)
Tracheal, bronchus, and lung cancer	South Africa	2000	All Ages	1.3 (0.8 to 1.8)	18.2% (11.1 to 25.7%)	2.8 (1.7 to 3.9)
Tracheal, bronchus, and lung cancer	South Africa	2005	All Ages	1.1 (0.7 to 1.7)	17.2% (10.4 to 24.6%)	2.3 (1.4 to 3.4)
Tracheal, bronchus, and lung cancer	South Africa	2010	All Ages	1.0 (0.6 to 1.5)	14.5% (8.4 to 21.5%)	1.9 (1.1 to 3.0)
Tracheal, bronchus, and lung cancer	South Africa	2015	All Ages	1.3 (0.8 to 1.9)	15.3% (9.0 to 22.4%)	2.4 (1.4 to 3.5)
Tracheal, bronchus, and lung cancer	South Africa	1990	Age-standardized		17.9% (10.9 to 25.4%)	4.8 (2.8 to 7.0)
Tracheal, bronchus, and lung cancer	South Africa	1995	Age-standardized		18.1% (11.2 to 25.5%)	4.7 (2.8 to 6.8)
Tracheal, bronchus, and lung cancer	South Africa	2000	Age-standardized		18.3% (11.1 to 25.7%)	5.1 (3.1 to 7.3)
Tracheal, bronchus, and lung cancer	South Africa	2005	Age-standardized		17.2% (10.4 to 24.7%)	4.0 (2.4 to 5.9)
Tracheal, bronchus, and lung cancer	South Africa	2010	Age-standardized		14.6% (8.4 to 21.6%)	3.2 (1.8 to 4.8)
Tracheal, bronchus, and lung cancer	South Africa	2015	Age-standardized		15.3% (9.0 to 22.5%)	3.8 (2.2 to 5.4)
Cardiovascular diseases	South Africa	1990	All Ages	8.5 (7.1 to 10.0)	12.0% (10.2 to 13.9%)	22.5 (18.9 to 26.5)
Cardiovascular diseases	South Africa	1995	All Ages	9.5 (7.9 to 11.2)	11.9% (10.1 to 13.8%)	22.7 (18.9 to 26.7)
Cardiovascular diseases	South Africa	2000	All Ages	13.1 (10.8 to 15.5)	12.3% (10.5 to 14.0%)	29.1 (24.0 to 34.3)
Cardiovascular diseases	South Africa	2005	All Ages	12.9 (10.3 to 15.7)	12.1% (10.2 to 13.9%)	26.5 (21.3 to 32.3)
Cardiovascular diseases	South Africa	2010	All Ages	11.1 (8.8 to 13.6)	11.1% (9.3 to 12.9%)	21.8 (17.4 to 26.7)
Cardiovascular diseases	South Africa	2015	All Ages	12.2 (10.1 to 14.5)	11.2% (9.4 to 13.1%)	22.7 (18.7 to 27.0)
Cardiovascular diseases	South Africa	1990	Age-standardized		11.8% (9.9 to 13.8%)	48.1 (39.8 to 57.2)
Cardiovascular diseases	South Africa	1995	Age-standardized		11.7% (9.7 to 13.7%)	46.1 (38.0 to 54.9)
Cardiovascular diseases	South Africa	2000	Age-standardized		12.0% (10.1 to 13.8%)	56.9 (46.5 to 67.6)
Cardiovascular diseases	South Africa	2005	Age-standardized		11.8% (9.8 to 13.6%)	48.7 (38.8 to 59.6)
Cardiovascular diseases	South Africa	2010	Age-standardized		10.8% (8.9 to 12.7%)	37.7 (29.8 to 46.2)

Cause name	Location	Year	Age group	Number (in thousands)	PAF	Rate (per 100,000)
Cardiovascular diseases	South Africa	2015	Age-standardized		11.0% (9.1 to 12.9%)	37.1 (30.2 to 44.7)
Ischemic heart disease	South Africa	1990	All Ages	5.0 (4.1 to 6.2)	17.4% (14.1 to 21.1%)	13.4 (10.8 to 16.5)
Ischemic heart disease	South Africa	1995	All Ages	5.7 (4.5 to 6.9)	17.3% (13.9 to 21.1%)	13.5 (10.8 to 16.6)
Ischemic heart disease	South Africa	2000	All Ages	7.7 (6.2 to 9.5)	17.9% (14.5 to 21.5%)	17.2 (13.7 to 21.1)
Ischemic heart disease	South Africa	2005	All Ages	7.7 (5.9 to 9.6)	17.7% (14.3 to 21.2%)	15.8 (12.2 to 19.7)
Ischemic heart disease	South Africa	2010	All Ages	6.7 (5.2 to 8.5)	16.4% (13.1 to 19.8%)	13.3 (10.1 to 16.7)
Ischemic heart disease	South Africa	2015	All Ages	7.5 (5.9 to 9.2)	16.7% (13.5 to 20.1%)	14.0 (11.0 to 17.1)
Ischemic heart disease	South Africa	1990	Age-standardized		16.8% (13.2 to 20.6%)	28.7 (22.6 to 35.7)
Ischemic heart disease	South Africa	1995	Age-standardized		16.7% (13.0 to 20.7%)	27.4 (21.4 to 34.1)
Ischemic heart disease	South Africa	2000	Age-standardized		17.1% (13.6 to 20.9%)	33.4 (25.8 to 41.5)
Ischemic heart disease	South Africa	2005	Age-standardized		17.0% (13.4 to 20.8%)	28.7 (21.7 to 36.3)
Ischemic heart disease	South Africa	2010	Age-standardized		15.8% (12.3 to 19.3%)	22.7 (17.1 to 28.7)
Ischemic heart disease	South Africa	2015	Age-standardized		16.0% (12.6 to 19.5%)	22.7 (17.5 to 27.9)
Ischemic stroke	South Africa	1990	All Ages	1.5 (1.1 to 1.9)	12.7% (9.3 to 16.2%)	3.9 (2.9 to 5.1)
Ischemic stroke	South Africa	1995	All Ages	1.7 (1.2 to 2.2)	12.7% (9.2 to 16.2%)	4.0 (2.9 to 5.2)
Ischemic stroke	South Africa	2000	All Ages	2.3 (1.6 to 2.9)	13.0% (9.6 to 16.3%)	5.0 (3.6 to 6.5)
Ischemic stroke	South Africa	2005	All Ages	2.3 (1.6 to 3.0)	12.7% (9.3 to 16.0%)	4.6 (3.3 to 6.1)
Ischemic stroke	South Africa	2010	All Ages	1.9 (1.3 to 2.6)	11.5% (8.1 to 15.0%)	3.8 (2.6 to 5.1)
Ischemic stroke	South Africa	2015	All Ages	2.1 (1.5 to 2.8)	11.7% (8.4 to 15.3%)	3.9 (2.7 to 5.2)
Ischemic stroke	South Africa	1990	Age-standardized		12.5% (9.0 to 16.1%)	8.8 (6.3 to 11.4)
Ischemic stroke	South Africa	1995	Age-standardized		12.4% (8.9 to 16.2%)	8.7 (6.2 to 11.3)
Ischemic stroke	South Africa	2000	Age-standardized		12.7% (9.2 to 16.4%)	10.6 (7.6 to 13.9)
Ischemic stroke	South Africa	2005	Age-standardized		12.4% (9.0 to 15.9%)	9.2 (6.6 to 12.2)
Ischemic stroke	South Africa	2010	Age-standardized		11.3% (7.9 to 14.9%)	7.1 (4.9 to 9.7)
Ischemic stroke	South Africa	2015	Age-standardized		11.5% (8.1 to 15.2%)	6.8 (4.8 to 9.3)
Hemorrhagic stroke	South Africa	1990	All Ages	2.0 (1.5 to 2.4)	14.5% (11.6 to 17.2%)	5.2 (4.1 to 6.4)
Hemorrhagic stroke	South Africa	1995	All Ages	2.2 (1.7 to 2.6)	14.4% (11.6 to 17.2%)	5.2 (4.0 to 6.3)
Hemorrhagic stroke	South Africa	2000	All Ages	3.1 (2.4 to 3.8)	14.9% (12.0 to 17.7%)	6.9 (5.3 to 8.5)
Hemorrhagic stroke	South Africa	2005	All Ages	3.0 (2.3 to 3.8)	14.4% (11.5 to 17.3%)	6.1 (4.6 to 7.8)
Hemorrhagic stroke	South Africa	2010	All Ages	2.4 (1.8 to 3.1)	13.1% (10.2 to 15.9%)	4.7 (3.5 to 6.1)
Hemorrhagic stroke	South Africa	2015	All Ages	2.6 (2.0 to 3.2)	13.4% (10.5 to 16.4%)	4.8 (3.7 to 6.0)
Hemorrhagic stroke	South Africa	1990	Age-standardized		14.0% (11.1 to 16.9%)	10.6 (8.1 to 13.1)
Hemorrhagic stroke	South Africa	1995	Age-standardized		13.9% (11.0 to 16.9%)	10.1 (7.7 to 12.4)
Hemorrhagic stroke	South Africa	2000	Age-standardized		14.2% (11.3 to 17.1%)	12.9 (9.8 to 16.0)
Hemorrhagic stroke	South Africa	2005	Age-standardized		13.8% (10.8 to 16.8%)	10.8 (8.2 to 14.0)
Hemorrhagic stroke	South Africa	2010	Age-standardized		12.6% (9.6 to 15.5%)	7.9 (5.7 to 10.3)

Cause name	Location	Year	Age group	Number (in thousands)	PAF	Rate (per 100,000)
Hemorrhagic stroke	South Africa	2015	Age-standardized		12.9% (9.9 to 15.7%)	7.6 (5.8 to 9.6)
Chronic obstructive pulmonary disease	South Africa	1990	All Ages	3.1 (1.7 to 4.6)	24.1% (13.4 to 35.2%)	8.2 (4.6 to 12.2)
Chronic obstructive pulmonary disease	South Africa	1995	All Ages	3.7 (2.0 to 5.4)	24.3% (13.8 to 35.5%)	8.8 (4.8 to 12.9)
Chronic obstructive pulmonary disease	South Africa	2000	All Ages	4.5 (2.5 to 6.5)	24.5% (13.8 to 35.7%)	9.9 (5.6 to 14.5)
Chronic obstructive pulmonary disease	South Africa	2005	All Ages	3.8 (2.1 to 5.7)	23.6% (12.9 to 34.6%)	7.8 (4.2 to 11.7)
Chronic obstructive pulmonary disease	South Africa	2010	All Ages	3.1 (1.6 to 4.8)	21.2% (10.9 to 32.0%)	6.2 (3.2 to 9.5)
Chronic obstructive pulmonary disease	South Africa	2015	All Ages	3.6 (1.9 to 5.5)	22.0% (11.6 to 32.9%)	6.7 (3.5 to 10.1)
Chronic obstructive pulmonary disease	South Africa	1990	Age-standardized		24.2% (13.5 to 35.4%)	18.4 (10.2 to 27.1)
Chronic obstructive pulmonary disease	South Africa	1995	Age-standardized		24.4% (13.8 to 35.7%)	18.7 (10.2 to 27.5)
Chronic obstructive pulmonary disease	South Africa	2000	Age-standardized		24.6% (13.9 to 35.8%)	20.5 (11.5 to 30.2)
Chronic obstructive pulmonary disease	South Africa	2005	Age-standardized		23.7% (12.9 to 34.8%)	15.2 (8.3 to 22.6)
Chronic obstructive pulmonary disease	South Africa	2010	Age-standardized		21.2% (10.9 to 32.1%)	11.3 (5.8 to 17.3)
Chronic obstructive pulmonary disease	South Africa	2015	Age-standardized		22.0% (11.6 to 32.9%)	11.6 (6.0 to 17.5)
All causes	Swaziland	1990	All Ages	0.3 (0.2 to 0.5)	4.6% (3.5 to 5.8%)	38.5 (27.3 to 53.1)
All causes	Swaziland	1995	All Ages	0.3 (0.2 to 0.4)	3.7% (2.7 to 4.9%)	32.5 (20.7 to 45.6)
All causes	Swaziland	2000	All Ages	0.5 (0.3 to 0.6)	3.0% (2.2 to 4.1%)	42.5 (28.6 to 59.7)
All causes	Swaziland	2005	All Ages	0.6 (0.4 to 0.8)	2.8% (2.0 to 3.7%)	54.5 (37.6 to 75.5)
All causes	Swaziland	2010	All Ages	0.6 (0.3 to 0.8)	2.8% (2.0 to 3.7%)	46.1 (27.4 to 71.1)
All causes	Swaziland	2015	All Ages	0.5 (0.3 to 0.8)	3.0% (2.1 to 4.0%)	40.3 (20.1 to 65.3)
All causes	Swaziland	1990	Age-standardized		6.1% (4.9 to 7.4%)	97.6 (68.2 to 131.9)
All causes	Swaziland	1995	Age-standardized		5.2% (4.0 to 6.6%)	83.4 (51.7 to 118.4)
All causes	Swaziland	2000	Age-standardized		4.4% (3.3 to 5.5%)	106.2 (71.2 to 148.5)
All causes	Swaziland	2005	Age-standardized		4.0% (3.1 to 5.0%)	129.0 (90.7 to 176.8)
All causes	Swaziland	2010	Age-standardized		3.9% (3.0 to 5.0%)	107.9 (65.2 to 161.8)
All causes	Swaziland	2015	Age-standardized		4.2% (3.2 to 5.3%)	95.6 (49.9 to 151.8)
Lower respiratory infections	Swaziland	1990	All Ages	0.2 (0.1 to 0.3)	20.0% (12.0 to 29.4%)	19.6 (11.4 to 30.5)
Lower respiratory infections	Swaziland	1995	All Ages	0.2 (0.1 to 0.3)	20.1% (12.6 to 29.5%)	16.2 (9.7 to 25.7)
Lower respiratory infections	Swaziland	2000	All Ages	0.2 (0.1 to 0.3)	20.6% (13.0 to 29.7%)	19.5 (11.3 to 29.1)
Lower respiratory infections	Swaziland	2005	All Ages	0.3 (0.1 to 0.4)	20.0% (12.4 to 29.2%)	23.3 (13.3 to 35.8)
Lower respiratory infections	Swaziland	2010	All Ages	0.2 (0.1 to 0.4)	17.6% (10.0 to 26.8%)	18.4 (9.6 to 30.7)
Lower respiratory infections	Swaziland	2015	All Ages	0.2 (0.1 to 0.3)	17.1% (9.3 to 26.7%)	14.1 (6.5 to 25.2)
Lower respiratory infections	Swaziland	1990	Age-standardized		20.0% (12.0 to 29.4%)	31.1 (17.1 to 50.8)
Lower respiratory infections	Swaziland	1995	Age-standardized		20.1% (12.6 to 29.5%)	27.9 (14.5 to 46.4)
Lower respiratory infections	Swaziland	2000	Age-standardized		20.6% (13.0 to 29.7%)	34.0 (18.8 to 55.0)
Lower respiratory infections	Swaziland	2005	Age-standardized		20.0% (12.4 to 29.2%)	38.8 (21.9 to 61.4)
Lower respiratory infections	Swaziland	2010	Age-standardized		17.6% (10.0 to 26.8%)	31.2 (15.5 to 53.8)

Cause name	Location	Year	Age group	Number (in thousands)	PAF	Rate (per 100,000)
Lower respiratory infections	Swaziland	2015	Age-standardized		17.1% (9.3 to 26.7%)	26.1 (11.5 to 47.2)
Tracheal, bronchus, and lung cancer	Swaziland	1990	All Ages	0.0 (0.0 to 0.0)	14.1% (8.1 to 21.1%)	0.8 (0.4 to 1.3)
Tracheal, bronchus, and lung cancer	Swaziland	1995	All Ages	0.0 (0.0 to 0.0)	14.2% (8.2 to 21.1%)	0.8 (0.3 to 1.4)
Tracheal, bronchus, and lung cancer	Swaziland	2000	All Ages	0.0 (0.0 to 0.0)	14.6% (8.6 to 21.5%)	1.1 (0.5 to 2.0)
Tracheal, bronchus, and lung cancer	Swaziland	2005	All Ages	0.0 (0.0 to 0.0)	14.1% (8.2 to 21.2%)	1.4 (0.6 to 2.4)
Tracheal, bronchus, and lung cancer	Swaziland	2010	All Ages	0.0 (0.0 to 0.0)	12.3% (6.7 to 18.7%)	1.3 (0.5 to 2.4)
Tracheal, bronchus, and lung cancer	Swaziland	2015	All Ages	0.0 (0.0 to 0.0)	11.9% (6.5 to 18.5%)	1.3 (0.4 to 2.6)
Tracheal, bronchus, and lung cancer	Swaziland	1990	Age-standardized		14.1% (8.1 to 21.1%)	2.3 (1.2 to 3.8)
Tracheal, bronchus, and lung cancer	Swaziland	1995	Age-standardized		14.3% (8.2 to 21.1%)	2.2 (1.0 to 4.0)
Tracheal, bronchus, and lung cancer	Swaziland	2000	Age-standardized		14.7% (8.6 to 21.6%)	3.1 (1.4 to 5.2)
Tracheal, bronchus, and lung cancer	Swaziland	2005	Age-standardized		14.2% (8.2 to 21.2%)	3.6 (1.7 to 6.2)
Tracheal, bronchus, and lung cancer	Swaziland	2010	Age-standardized		12.3% (6.7 to 18.7%)	3.1 (1.3 to 5.7)
Tracheal, bronchus, and lung cancer	Swaziland	2015	Age-standardized		12.0% (6.5 to 18.5%)	3.0 (1.1 to 6.1)
Cardiovascular diseases	Swaziland	1990	All Ages	0.1 (0.1 to 0.2)	10.8% (9.1 to 12.5%)	13.1 (8.5 to 18.2)
Cardiovascular diseases	Swaziland	1995	All Ages	0.1 (0.1 to 0.2)	10.7% (8.9 to 12.5%)	11.1 (6.0 to 16.7)
Cardiovascular diseases	Swaziland	2000	All Ages	0.2 (0.1 to 0.3)	11.2% (9.4 to 13.0%)	16.5 (10.4 to 24.2)
Cardiovascular diseases	Swaziland	2005	All Ages	0.3 (0.2 to 0.4)	11.3% (9.5 to 13.2%)	23.2 (15.3 to 33.8)
Cardiovascular diseases	Swaziland	2010	All Ages	0.3 (0.1 to 0.4)	10.6% (8.8 to 12.4%)	20.8 (11.3 to 33.2)
Cardiovascular diseases	Swaziland	2015	All Ages	0.3 (0.1 to 0.4)	10.5% (8.8 to 12.3%)	19.6 (9.0 to 33.0)
Cardiovascular diseases	Swaziland	1990	Age-standardized		10.2% (8.3 to 12.1%)	44.4 (29.7 to 61.5)
Cardiovascular diseases	Swaziland	1995	Age-standardized		10.1% (8.1 to 12.0%)	37.0 (21.3 to 54.6)
Cardiovascular diseases	Swaziland	2000	Age-standardized		10.6% (8.7 to 12.5%)	50.3 (32.4 to 72.2)
Cardiovascular diseases	Swaziland	2005	Age-standardized		10.8% (8.8 to 12.7%)	65.7 (44.9 to 91.8)
Cardiovascular diseases	Swaziland	2010	Age-standardized		10.1% (8.2 to 11.8%)	56.3 (32.7 to 86.2)
Cardiovascular diseases	Swaziland	2015	Age-standardized		10.0% (8.1 to 11.9%)	51.1 (25.7 to 84.0)
Ischemic heart disease	Swaziland	1990	All Ages	0.1 (0.0 to 0.1)	17.0% (13.9 to 20.1%)	7.4 (4.8 to 10.5)
Ischemic heart disease	Swaziland	1995	All Ages	0.1 (0.0 to 0.1)	16.8% (13.7 to 20.0%)	6.5 (3.6 to 9.9)
Ischemic heart disease	Swaziland	2000	All Ages	0.1 (0.1 to 0.1)	17.3% (14.2 to 20.4%)	9.7 (6.0 to 14.4)
Ischemic heart disease	Swaziland	2005	All Ages	0.1 (0.1 to 0.2)	17.2% (14.2 to 20.4%)	13.8 (8.8 to 20.4)
Ischemic heart disease	Swaziland	2010	All Ages	0.1 (0.1 to 0.2)	16.1% (13.2 to 19.2%)	12.6 (6.8 to 20.3)
Ischemic heart disease	Swaziland	2015	All Ages	0.2 (0.1 to 0.3)	15.8% (12.8 to 19.0%)	12.1 (5.5 to 20.8)
Ischemic heart disease	Swaziland	1990	Age-standardized		15.2% (11.8 to 18.6%)	24.8 (16.2 to 34.6)
Ischemic heart disease	Swaziland	1995	Age-standardized		15.2% (11.7 to 18.6%)	21.2 (12.1 to 31.8)
Ischemic heart disease	Swaziland	2000	Age-standardized		15.6% (12.3 to 18.9%)	29.2 (18.1 to 42.8)
Ischemic heart disease	Swaziland	2005	Age-standardized		15.7% (12.5 to 19.1%)	38.5 (25.0 to 55.4)
Ischemic heart disease	Swaziland	2010	Age-standardized		14.7% (11.6 to 17.7%)	33.6 (19.2 to 51.8)

Cause name	Location	Year	Age group	Number (in thousands)	PAF	Rate (per 100,000)
Ischemic heart disease	Swaziland	2015	Age-standardized		14.5% (11.4 to 17.8%)	31.1 (15.4 to 51.6)
Ischemic stroke	Swaziland	1990	All Ages	0.0 (0.0 to 0.0)	11.9% (8.8 to 14.8%)	2.1 (1.3 to 3.1)
Ischemic stroke	Swaziland	1995	All Ages	0.0 (0.0 to 0.0)	11.7% (8.5 to 14.7%)	1.8 (0.9 to 2.8)
Ischemic stroke	Swaziland	2000	All Ages	0.0 (0.0 to 0.0)	12.0% (8.7 to 15.0%)	2.6 (1.5 to 3.9)
Ischemic stroke	Swaziland	2005	All Ages	0.0 (0.0 to 0.1)	11.8% (8.6 to 14.9%)	3.5 (2.1 to 5.1)
Ischemic stroke	Swaziland	2010	All Ages	0.0 (0.0 to 0.1)	10.9% (7.7 to 14.0%)	3.1 (1.7 to 5.0)
Ischemic stroke	Swaziland	2015	All Ages	0.0 (0.0 to 0.1)	10.6% (7.6 to 13.7%)	2.9 (1.3 to 5.0)
Ischemic stroke	Swaziland	1990	Age-standardized		11.0% (7.6 to 14.2%)	8.6 (5.2 to 12.9)
Ischemic stroke	Swaziland	1995	Age-standardized		10.9% (7.5 to 14.4%)	7.1 (3.8 to 10.9)
Ischemic stroke	Swaziland	2000	Age-standardized		11.2% (7.8 to 14.5%)	9.3 (5.4 to 14.1)
Ischemic stroke	Swaziland	2005	Age-standardized		11.1% (7.8 to 14.4%)	11.5 (7.0 to 17.0)
Ischemic stroke	Swaziland	2010	Age-standardized		10.2% (7.1 to 13.6%)	9.8 (5.3 to 15.8)
Ischemic stroke	Swaziland	2015	Age-standardized		10.0% (6.9 to 13.3%)	8.8 (4.2 to 14.5)
Hemorrhagic stroke	Swaziland	1990	All Ages	0.0 (0.0 to 0.1)	13.3% (10.4 to 16.4%)	3.6 (2.1 to 5.4)
Hemorrhagic stroke	Swaziland	1995	All Ages	0.0 (0.0 to 0.0)	13.1% (10.2 to 16.1%)	2.9 (1.4 to 4.6)
Hemorrhagic stroke	Swaziland	2000	All Ages	0.0 (0.0 to 0.1)	13.5% (10.6 to 16.5%)	4.2 (2.5 to 6.7)
Hemorrhagic stroke	Swaziland	2005	All Ages	0.1 (0.0 to 0.1)	13.3% (10.3 to 16.5%)	5.9 (3.7 to 9.3)
Hemorrhagic stroke	Swaziland	2010	All Ages	0.1 (0.0 to 0.1)	12.3% (9.4 to 15.2%)	5.1 (2.6 to 8.6)
Hemorrhagic stroke	Swaziland	2015	All Ages	0.1 (0.0 to 0.1)	12.0% (9.2 to 15.0%)	4.6 (2.0 to 8.4)
Hemorrhagic stroke	Swaziland	1990	Age-standardized		12.3% (9.4 to 15.3%)	10.9 (6.5 to 16.2)
Hemorrhagic stroke	Swaziland	1995	Age-standardized		12.2% (9.2 to 15.3%)	8.7 (4.4 to 14.0)
Hemorrhagic stroke	Swaziland	2000	Age-standardized		12.6% (9.5 to 15.4%)	11.8 (7.2 to 18.3)
Hemorrhagic stroke	Swaziland	2005	Age-standardized		12.5% (9.5 to 15.5%)	15.6 (10.1 to 23.7)
Hemorrhagic stroke	Swaziland	2010	Age-standardized		11.5% (8.6 to 14.5%)	12.9 (6.9 to 21.1)
Hemorrhagic stroke	Swaziland	2015	Age-standardized		11.3% (8.3 to 14.3%)	11.3 (5.2 to 20.1)
Chronic obstructive pulmonary disease	Swaziland	1990	All Ages	0.0 (0.0 to 0.1)	20.0% (10.1 to 30.5%)	5.1 (2.3 to 8.8)
Chronic obstructive pulmonary disease	Swaziland	1995	All Ages	0.0 (0.0 to 0.1)	20.1% (10.8 to 30.5%)	4.3 (2.0 to 7.8)
Chronic obstructive pulmonary disease	Swaziland	2000	All Ages	0.1 (0.0 to 0.1)	20.5% (10.8 to 31.3%)	5.4 (2.5 to 9.7)
Chronic obstructive pulmonary disease	Swaziland	2005	All Ages	0.1 (0.0 to 0.1)	20.0% (10.2 to 30.1%)	6.6 (3.0 to 11.2)
Chronic obstructive pulmonary disease	Swaziland	2010	All Ages	0.1 (0.0 to 0.1)	18.1% (9.1 to 28.4%)	5.7 (2.5 to 10.7)
Chronic obstructive pulmonary disease	Swaziland	2015	All Ages	0.1 (0.0 to 0.1)	17.8% (8.8 to 27.9%)	5.3 (2.1 to 10.3)
Chronic obstructive pulmonary disease	Swaziland	1990	Age-standardized		20.3% (10.4 to 31.1%)	19.8 (8.9 to 33.4)
Chronic obstructive pulmonary disease	Swaziland	1995	Age-standardized		20.5% (11.0 to 31.1%)	16.3 (7.5 to 29.3)
Chronic obstructive pulmonary disease	Swaziland	2000	Age-standardized		20.8% (11.0 to 31.9%)	18.8 (8.7 to 33.0)
Chronic obstructive pulmonary disease	Swaziland	2005	Age-standardized		20.3% (10.3 to 30.6%)	21.0 (9.7 to 34.5)
Chronic obstructive pulmonary disease	Swaziland	2010	Age-standardized		18.3% (9.3 to 28.9%)	17.3 (8.1 to 31.6)

Cause name	Location	Year	Age group	Number (in thousands)	PAF	Rate (per 100,000)
Chronic obstructive pulmonary disease	Swaziland	2015	Age-standardized		18.0% (8.9 to 28.2%)	15.4 (6.3 to 29.0)
All causes	Zimbabwe	1990	All Ages	2.5 (1.8 to 3.4)	3.2% (2.4 to 4.2%)	23.9 (17.1 to 32.1)
All causes	Zimbabwe	1995	All Ages	2.6 (1.6 to 4.0)	2.2% (1.4 to 3.2%)	22.4 (13.6 to 34.2)
All causes	Zimbabwe	2000	All Ages	3.6 (2.3 to 5.5)	2.0% (1.3 to 2.9%)	28.7 (18.6 to 44.2)
All causes	Zimbabwe	2005	All Ages	4.5 (3.1 to 6.7)	2.1% (1.5 to 2.9%)	34.9 (23.6 to 51.4)
All causes	Zimbabwe	2010	All Ages	4.5 (2.9 to 6.3)	2.2% (1.6 to 3.1%)	32.0 (20.7 to 45.0)
All causes	Zimbabwe	2015	All Ages	3.8 (2.3 to 5.8)	2.7% (1.9 to 3.7%)	24.5 (14.8 to 37.3)
All causes	Zimbabwe	1990	Age-standardized		4.5% (3.5 to 5.6%)	60.6 (43.7 to 79.5)
All causes	Zimbabwe	1995	Age-standardized		3.4% (2.4 to 4.6%)	59.7 (36.7 to 90.7)
All causes	Zimbabwe	2000	Age-standardized		3.0% (2.2 to 4.2%)	75.7 (49.6 to 116.5)
All causes	Zimbabwe	2005	Age-standardized		3.1% (2.3 to 4.2%)	91.2 (62.7 to 133.1)
All causes	Zimbabwe	2010	Age-standardized		3.4% (2.5 to 4.4%)	82.1 (54.4 to 114.1)
All causes	Zimbabwe	2015	Age-standardized		4.0% (3.0 to 5.3%)	64.5 (38.5 to 98.9)
Lower respiratory infections	Zimbabwe	1990	All Ages	1.4 (0.8 to 2.1)	20.2% (12.0 to 29.5%)	13.0 (7.7 to 19.6)
Lower respiratory infections	Zimbabwe	1995	All Ages	1.4 (0.7 to 2.3)	19.3% (11.3 to 28.8%)	11.7 (6.1 to 19.5)
Lower respiratory infections	Zimbabwe	2000	All Ages	1.8 (0.9 to 3.1)	19.5% (11.8 to 28.9%)	14.0 (7.3 to 24.3)
Lower respiratory infections	Zimbabwe	2005	All Ages	2.2 (1.1 to 3.6)	19.6% (11.9 to 29.1%)	16.7 (8.7 to 27.9)
Lower respiratory infections	Zimbabwe	2010	All Ages	2.2 (1.2 to 3.6)	17.9% (10.2 to 27.3%)	15.8 (8.3 to 26.1)
Lower respiratory infections	Zimbabwe	2015	All Ages	1.9 (0.9 to 3.3)	18.0% (10.3 to 27.6%)	12.4 (6.1 to 21.5)
Lower respiratory infections	Zimbabwe	1990	Age-standardized		20.2% (12.0 to 29.5%)	24.5 (13.9 to 37.9)
Lower respiratory infections	Zimbabwe	1995	Age-standardized		19.3% (11.3 to 28.8%)	25.6 (12.8 to 44.4)
Lower respiratory infections	Zimbabwe	2000	Age-standardized		19.5% (11.8 to 28.9%)	31.5 (15.6 to 56.6)
Lower respiratory infections	Zimbabwe	2005	Age-standardized		19.6% (11.9 to 29.1%)	37.0 (18.2 to 64.1)
Lower respiratory infections	Zimbabwe	2010	Age-standardized		17.9% (10.2 to 27.3%)	33.6 (16.7 to 57.8)
Lower respiratory infections	Zimbabwe	2015	Age-standardized		18.0% (10.3 to 27.6%)	27.3 (12.2 to 48.7)
Tracheal, bronchus, and lung cancer	Zimbabwe	1990	All Ages	0.1 (0.1 to 0.2)	14.2% (8.3 to 21.0%)	1.3 (0.7 to 2.1)
Tracheal, bronchus, and lung cancer	Zimbabwe	1995	All Ages	0.1 (0.1 to 0.2)	13.6% (7.7 to 20.1%)	1.0 (0.4 to 1.9)
Tracheal, bronchus, and lung cancer	Zimbabwe	2000	All Ages	0.1 (0.1 to 0.3)	13.7% (7.9 to 20.3%)	1.2 (0.5 to 2.3)
Tracheal, bronchus, and lung cancer	Zimbabwe	2005	All Ages	0.2 (0.1 to 0.3)	13.8% (7.9 to 20.5%)	1.4 (0.7 to 2.6)
Tracheal, bronchus, and lung cancer	Zimbabwe	2010	All Ages	0.2 (0.1 to 0.3)	12.5% (7.1 to 18.9%)	1.3 (0.6 to 2.2)
Tracheal, bronchus, and lung cancer	Zimbabwe	2015	All Ages	0.2 (0.1 to 0.3)	12.6% (7.1 to 18.9%)	1.1 (0.5 to 2.0)
Tracheal, bronchus, and lung cancer	Zimbabwe	1990	Age-standardized		14.3% (8.3 to 21.0%)	4.0 (2.2 to 6.4)
Tracheal, bronchus, and lung cancer	Zimbabwe	1995	Age-standardized		13.6% (7.7 to 20.2%)	3.0 (1.4 to 5.6)
Tracheal, bronchus, and lung cancer	Zimbabwe	2000	Age-standardized		13.7% (7.9 to 20.4%)	3.5 (1.6 to 6.7)
Tracheal, bronchus, and lung cancer	Zimbabwe	2005	Age-standardized		13.9% (7.9 to 20.5%)	4.3 (2.0 to 7.7)
Tracheal, bronchus, and lung cancer	Zimbabwe	2010	Age-standardized		12.5% (7.1 to 18.9%)	3.9 (1.9 to 6.5)

Cause name	Location	Year	Age group	Number (in thousands)	PAF	Rate (per 100,000)
Tracheal, bronchus, and lung cancer	Zimbabwe	2015	Age-standardized		12.7% (7.1 to 18.9%)	3.4 (1.5 to 6.1)
Cardiovascular diseases	Zimbabwe	1990	All Ages	0.8 (0.6 to 1.1)	10.6% (8.9 to 12.2%)	7.7 (5.4 to 10.1)
Cardiovascular diseases	Zimbabwe	1995	All Ages	0.9 (0.6 to 1.5)	10.5% (8.7 to 12.2%)	7.8 (4.7 to 12.6)
Cardiovascular diseases	Zimbabwe	2000	All Ages	1.4 (0.9 to 2.1)	10.8% (9.2 to 12.6%)	11.1 (7.4 to 16.9)
Cardiovascular diseases	Zimbabwe	2005	All Ages	1.8 (1.2 to 2.6)	11.0% (9.3 to 12.7%)	13.8 (9.5 to 19.8)
Cardiovascular diseases	Zimbabwe	2010	All Ages	1.7 (1.2 to 2.4)	10.3% (8.7 to 11.8%)	12.2 (8.3 to 17.0)
Cardiovascular diseases	Zimbabwe	2015	All Ages	1.4 (0.8 to 2.2)	10.1% (8.3 to 11.7%)	9.0 (5.2 to 14.2)
Cardiovascular diseases	Zimbabwe	1990	Age-standardized		10.9% (9.0 to 12.9%)	25.1 (18.0 to 33.4)
Cardiovascular diseases	Zimbabwe	1995	Age-standardized		10.7% (8.8 to 12.8%)	24.6 (14.9 to 38.4)
Cardiovascular diseases	Zimbabwe	2000	Age-standardized		11.1% (9.2 to 13.1%)	33.1 (22.4 to 50.2)
Cardiovascular diseases	Zimbabwe	2005	Age-standardized		11.3% (9.4 to 13.3%)	40.7 (28.1 to 56.9)
Cardiovascular diseases	Zimbabwe	2010	Age-standardized		10.7% (8.9 to 12.6%)	36.2 (24.8 to 50.5)
Cardiovascular diseases	Zimbabwe	2015	Age-standardized		10.5% (8.6 to 12.4%)	27.2 (16.3 to 42.2)
Ischemic heart disease	Zimbabwe	1990	All Ages	0.5 (0.3 to 0.7)	16.0% (12.8 to 19.3%)	4.7 (3.3 to 6.2)
Ischemic heart disease	Zimbabwe	1995	All Ages	0.6 (0.3 to 0.9)	15.6% (12.4 to 18.7%)	4.9 (2.8 to 7.7)
Ischemic heart disease	Zimbabwe	2000	All Ages	0.9 (0.6 to 1.3)	15.7% (12.7 to 18.9%)	6.9 (4.4 to 10.6)
Ischemic heart disease	Zimbabwe	2005	All Ages	1.1 (0.7 to 1.6)	15.9% (12.8 to 19.0%)	8.5 (5.7 to 12.1)
Ischemic heart disease	Zimbabwe	2010	All Ages	1.0 (0.7 to 1.5)	15.2% (12.3 to 18.2%)	7.4 (4.9 to 10.6)
Ischemic heart disease	Zimbabwe	2015	All Ages	0.8 (0.5 to 1.4)	15.1% (12.0 to 18.2%)	5.5 (3.2 to 8.7)
Ischemic heart disease	Zimbabwe	1990	Age-standardized		14.8% (11.3 to 18.4%)	15.7 (10.8 to 21.0)
Ischemic heart disease	Zimbabwe	1995	Age-standardized		14.5% (10.8 to 18.1%)	15.7 (9.1 to 24.6)
Ischemic heart disease	Zimbabwe	2000	Age-standardized		14.9% (11.6 to 18.2%)	21.0 (13.9 to 32.3)
Ischemic heart disease	Zimbabwe	2005	Age-standardized		15.1% (11.8 to 18.3%)	25.6 (17.3 to 36.8)
Ischemic heart disease	Zimbabwe	2010	Age-standardized		14.5% (11.3 to 17.8%)	22.8 (15.0 to 32.5)
Ischemic heart disease	Zimbabwe	2015	Age-standardized		14.4% (11.0 to 17.6%)	17.2 (10.0 to 27.0)
Ischemic stroke	Zimbabwe	1990	All Ages	0.1 (0.1 to 0.2)	11.3% (8.1 to 14.6%)	1.1 (0.7 to 1.6)
Ischemic stroke	Zimbabwe	1995	All Ages	0.1 (0.1 to 0.2)	10.9% (7.8 to 14.1%)	1.1 (0.6 to 1.8)
Ischemic stroke	Zimbabwe	2000	All Ages	0.2 (0.1 to 0.3)	11.1% (8.0 to 14.2%)	1.5 (0.9 to 2.4)
Ischemic stroke	Zimbabwe	2005	All Ages	0.2 (0.1 to 0.4)	11.1% (8.0 to 14.2%)	1.9 (1.1 to 2.9)
Ischemic stroke	Zimbabwe	2010	All Ages	0.2 (0.1 to 0.4)	10.5% (7.4 to 13.5%)	1.6 (1.0 to 2.5)
Ischemic stroke	Zimbabwe	2015	All Ages	0.2 (0.1 to 0.3)	10.4% (7.4 to 13.6%)	1.2 (0.7 to 1.9)
Ischemic stroke	Zimbabwe	1990	Age-standardized		10.7% (7.3 to 14.4%)	4.1 (2.6 to 6.2)
Ischemic stroke	Zimbabwe	1995	Age-standardized		10.5% (7.1 to 14.3%)	3.9 (2.1 to 6.7)
Ischemic stroke	Zimbabwe	2000	Age-standardized		10.7% (7.5 to 14.3%)	5.1 (2.9 to 8.3)
Ischemic stroke	Zimbabwe	2005	Age-standardized		10.8% (7.6 to 14.2%)	6.1 (3.7 to 9.3)
Ischemic stroke	Zimbabwe	2010	Age-standardized		10.2% (7.0 to 13.7%)	5.4 (3.2 to 8.4)

Cause name	Location	Year	Age group	Number (in thousands)	PAF	Rate (per 100,000)
Ischemic stroke	Zimbabwe	2015	Age-standardized		10.2% (6.9 to 13.7%)	4.0 (2.2 to 6.7)
Hemorrhagic stroke	Zimbabwe	1990	All Ages	0.2 (0.1 to 0.3)	10.6% (8.4 to 13.0%)	1.9 (1.3 to 2.7)
Hemorrhagic stroke	Zimbabwe	1995	All Ages	0.2 (0.1 to 0.4)	10.9% (8.5 to 13.4%)	1.9 (1.0 to 3.2)
Hemorrhagic stroke	Zimbabwe	2000	All Ages	0.3 (0.2 to 0.6)	11.3% (8.9 to 13.9%)	2.7 (1.6 to 4.5)
Hemorrhagic stroke	Zimbabwe	2005	All Ages	0.5 (0.3 to 0.7)	11.6% (9.2 to 14.1%)	3.5 (2.2 to 5.4)
Hemorrhagic stroke	Zimbabwe	2010	All Ages	0.4 (0.3 to 0.7)	10.6% (8.4 to 13.1%)	3.1 (2.0 to 4.7)
Hemorrhagic stroke	Zimbabwe	2015	All Ages	0.4 (0.2 to 0.6)	10.5% (8.1 to 13.1%)	2.3 (1.2 to 3.8)
Hemorrhagic stroke	Zimbabwe	1990	Age-standardized		12.0% (9.3 to 14.8%)	5.3 (3.5 to 7.7)
Hemorrhagic stroke	Zimbabwe	1995	Age-standardized		11.8% (9.1 to 14.7%)	5.0 (2.7 to 8.3)
Hemorrhagic stroke	Zimbabwe	2000	Age-standardized		12.2% (9.6 to 15.0%)	7.0 (4.2 to 11.5)
Hemorrhagic stroke	Zimbabwe	2005	Age-standardized		12.5% (9.9 to 15.2%)	9.0 (5.8 to 13.6)
Hemorrhagic stroke	Zimbabwe	2010	Age-standardized		11.7% (9.1 to 14.3%)	8.1 (5.2 to 12.3)
Hemorrhagic stroke	Zimbabwe	2015	Age-standardized		11.6% (8.9 to 14.4%)	5.9 (3.2 to 9.9)
Chronic obstructive pulmonary disease	Zimbabwe	1990	All Ages	0.2 (0.1 to 0.3)	20.0% (10.7 to 30.5%)	2.0 (0.9 to 3.2)
Chronic obstructive pulmonary disease	Zimbabwe	1995	All Ages	0.2 (0.1 to 0.4)	19.5% (9.8 to 29.9%)	1.9 (0.8 to 3.6)
Chronic obstructive pulmonary disease	Zimbabwe	2000	All Ages	0.3 (0.1 to 0.6)	19.7% (9.7 to 30.3%)	2.4 (1.0 to 4.5)
Chronic obstructive pulmonary disease	Zimbabwe	2005	All Ages	0.4 (0.2 to 0.7)	19.8% (10.0 to 30.3%)	3.0 (1.4 to 5.1)
Chronic obstructive pulmonary disease	Zimbabwe	2010	All Ages	0.4 (0.2 to 0.6)	18.3% (9.1 to 28.7%)	2.7 (1.2 to 4.5)
Chronic obstructive pulmonary disease	Zimbabwe	2015	All Ages	0.3 (0.1 to 0.6)	18.4% (9.2 to 28.9%)	2.0 (0.9 to 3.7)
Chronic obstructive pulmonary disease	Zimbabwe	1990	Age-standardized		20.5% (10.9 to 31.2%)	7.0 (3.3 to 11.3)
Chronic obstructive pulmonary disease	Zimbabwe	1995	Age-standardized		19.8% (9.9 to 30.4%)	6.5 (2.8 to 11.9)
Chronic obstructive pulmonary disease	Zimbabwe	2000	Age-standardized		20.0% (9.9 to 30.7%)	7.7 (3.4 to 13.9)
Chronic obstructive pulmonary disease	Zimbabwe	2005	Age-standardized		20.1% (10.2 to 30.7%)	9.2 (4.3 to 15.5)
Chronic obstructive pulmonary disease	Zimbabwe	2010	Age-standardized		18.6% (9.3 to 29.2%)	8.4 (3.8 to 14.0)
Chronic obstructive pulmonary disease	Zimbabwe	2015	Age-standardized		18.8% (9.3 to 29.4%)	6.6 (2.9 to 11.8)
All causes	Benin	1990	All Ages	4.1 (2.9 to 5.4)	6.2% (4.4 to 8.1%)	82.1 (57.6 to 107.8)
All causes	Benin	1995	All Ages	4.5 (3.3 to 5.7)	6.2% (4.6 to 7.7%)	75.0 (55.5 to 95.2)
All causes	Benin	2000	All Ages	4.7 (3.4 to 6.0)	6.0% (4.5 to 7.5%)	67.5 (48.6 to 85.9)
All causes	Benin	2005	All Ages	4.1 (3.0 to 5.4)	5.0% (4.0 to 6.2%)	50.6 (37.0 to 65.7)
All causes	Benin	2010	All Ages	4.3 (2.9 to 6.0)	5.2% (4.1 to 6.2%)	45.2 (30.3 to 63.1)
All causes	Benin	2015	All Ages	4.3 (2.6 to 6.7)	5.0% (4.0 to 6.1%)	39.6 (24.0 to 61.6)
All causes	Benin	1990	Age-standardized		7.9% (6.6 to 9.2%)	121.3 (99.7 to 143.8)
All causes	Benin	1995	Age-standardized		7.9% (6.7 to 9.2%)	122.1 (98.3 to 147.2)
All causes	Benin	2000	Age-standardized		8.0% (6.8 to 9.2%)	125.4 (98.2 to 155.1)
All causes	Benin	2005	Age-standardized		7.2% (6.0 to 8.3%)	109.9 (80.3 to 144.7)
All causes	Benin	2010	Age-standardized		7.3% (6.2 to 8.5%)	106.1 (68.9 to 155.2)

Cause name	Location	Year	Age group	Number (in thousands)	PAF	Rate (per 100,000)
All causes	Benin	2015	Age-standardized		6.9% (5.8 to 8.1%)	96.1 (55.9 to 152.4)
Lower respiratory infections	Benin	1990	All Ages	3.1 (1.9 to 4.3)	33.4% (25.7 to 40.9%)	61.2 (37.1 to 86.1)
Lower respiratory infections	Benin	1995	All Ages	3.3 (2.2 to 4.5)	32.9% (25.1 to 39.8%)	55.2 (36.1 to 74.6)
Lower respiratory infections	Benin	2000	All Ages	3.3 (2.1 to 4.4)	32.6% (25.0 to 39.8%)	46.9 (30.5 to 63.4)
Lower respiratory infections	Benin	2005	All Ages	2.6 (1.8 to 3.5)	28.5% (20.8 to 36.0%)	31.2 (21.7 to 43.0)
Lower respiratory infections	Benin	2010	All Ages	2.4 (1.6 to 3.5)	27.4% (20.0 to 35.0%)	25.3 (16.8 to 36.3)
Lower respiratory infections	Benin	2015	All Ages	2.2 (1.3 to 3.3)	24.3% (17.0 to 32.5%)	20.1 (12.0 to 30.3)
Lower respiratory infections	Benin	1990	Age-standardized		33.4% (25.7 to 40.9%)	61.1 (42.9 to 80.3)
Lower respiratory infections	Benin	1995	Age-standardized		32.9% (25.1 to 39.8%)	63.3 (45.4 to 83.7)
Lower respiratory infections	Benin	2000	Age-standardized		32.6% (25.0 to 39.8%)	62.9 (43.1 to 82.2)
Lower respiratory infections	Benin	2005	Age-standardized		28.5% (20.8 to 36.0%)	50.0 (33.6 to 70.3)
Lower respiratory infections	Benin	2010	Age-standardized		27.4% (20.0 to 35.0%)	45.4 (27.0 to 67.2)
Lower respiratory infections	Benin	2015	Age-standardized		24.3% (17.0 to 32.5%)	38.4 (21.2 to 63.0)
Tracheal, bronchus, and lung cancer	Benin	1990	All Ages	0.0 (0.0 to 0.1)	25.3% (17.3 to 33.7%)	0.9 (0.6 to 1.2)
Tracheal, bronchus, and lung cancer	Benin	1995	All Ages	0.1 (0.0 to 0.1)	24.8% (16.8 to 32.7%)	0.8 (0.5 to 1.2)
Tracheal, bronchus, and lung cancer	Benin	2000	All Ages	0.1 (0.0 to 0.1)	24.6% (16.4 to 32.9%)	0.9 (0.5 to 1.2)
Tracheal, bronchus, and lung cancer	Benin	2005	All Ages	0.1 (0.0 to 0.1)	21.0% (13.3 to 28.5%)	0.8 (0.4 to 1.2)
Tracheal, bronchus, and lung cancer	Benin	2010	All Ages	0.1 (0.0 to 0.1)	19.9% (12.5 to 27.5%)	0.8 (0.4 to 1.5)
Tracheal, bronchus, and lung cancer	Benin	2015	All Ages	0.1 (0.0 to 0.2)	17.4% (10.8 to 24.8%)	0.8 (0.4 to 1.5)
Tracheal, bronchus, and lung cancer	Benin	1990	Age-standardized		25.4% (17.4 to 33.9%)	2.3 (1.5 to 3.1)
Tracheal, bronchus, and lung cancer	Benin	1995	Age-standardized		25.0% (16.9 to 32.9%)	2.3 (1.5 to 3.2)
Tracheal, bronchus, and lung cancer	Benin	2000	Age-standardized		24.8% (16.5 to 33.1%)	2.4 (1.5 to 3.4)
Tracheal, bronchus, and lung cancer	Benin	2005	Age-standardized		21.1% (13.4 to 28.7%)	2.2 (1.3 to 3.3)
Tracheal, bronchus, and lung cancer	Benin	2010	Age-standardized		20.1% (12.6 to 27.7%)	2.3 (1.2 to 4.0)
Tracheal, bronchus, and lung cancer	Benin	2015	Age-standardized		17.5% (10.8 to 25.0%)	2.2 (1.0 to 4.0)
Cardiovascular diseases	Benin	1990	All Ages	0.8 (0.7 to 1.0)	14.9% (13.1 to 16.8%)	16.2 (13.1 to 19.6)
Cardiovascular diseases	Benin	1995	All Ages	1.0 (0.8 to 1.2)	15.2% (13.2 to 17.1%)	16.0 (12.9 to 19.6)
Cardiovascular diseases	Benin	2000	All Ages	1.2 (0.9 to 1.5)	15.9% (14.0 to 17.9%)	17.1 (13.2 to 22.1)
Cardiovascular diseases	Benin	2005	All Ages	1.3 (0.9 to 1.8)	15.3% (13.4 to 17.2%)	16.3 (11.3 to 22.0)
Cardiovascular diseases	Benin	2010	All Ages	1.6 (1.0 to 2.5)	15.2% (13.2 to 17.2%)	16.7 (10.0 to 26.0)
Cardiovascular diseases	Benin	2015	All Ages	1.8 (0.9 to 3.1)	14.4% (12.4 to 16.3%)	16.5 (8.5 to 28.6)
Cardiovascular diseases	Benin	1990	Age-standardized		14.6% (12.5 to 16.8%)	46.4 (37.6 to 56.0)
Cardiovascular diseases	Benin	1995	Age-standardized		14.7% (12.6 to 17.0%)	47.1 (37.8 to 58.1)
Cardiovascular diseases	Benin	2000	Age-standardized		15.1% (13.0 to 17.4%)	51.5 (39.8 to 65.6)
Cardiovascular diseases	Benin	2005	Age-standardized		14.3% (12.1 to 16.4%)	49.9 (35.1 to 66.7)
Cardiovascular diseases	Benin	2010	Age-standardized		14.0% (11.8 to 16.2%)	50.5 (31.4 to 76.1)

Cause name	Location	Year	Age group	Number (in thousands)	PAF	Rate (per 100,000)
Cardiovascular diseases	Benin	2015	Age-standardized		13.2% (11.1 to 15.3%)	48.3 (27.0 to 79.5)
Ischemic heart disease	Benin	1990	All Ages	0.5 (0.4 to 0.6)	21.3% (18.0 to 24.9%)	9.1 (7.2 to 11.3)
Ischemic heart disease	Benin	1995	All Ages	0.6 (0.4 to 0.7)	21.4% (18.1 to 24.9%)	9.4 (7.3 to 11.7)
Ischemic heart disease	Benin	2000	All Ages	0.7 (0.5 to 0.9)	21.7% (18.4 to 25.1%)	10.0 (7.5 to 13.1)
Ischemic heart disease	Benin	2005	All Ages	0.8 (0.5 to 1.1)	20.4% (17.2 to 23.6%)	9.4 (6.4 to 13.2)
Ischemic heart disease	Benin	2010	All Ages	0.9 (0.6 to 1.4)	20.1% (17.0 to 23.5%)	9.5 (5.8 to 14.9)
Ischemic heart disease	Benin	2015	All Ages	1.0 (0.5 to 1.8)	19.1% (15.9 to 22.3%)	9.4 (4.9 to 16.3)
Ischemic heart disease	Benin	1990	Age-standardized		19.3% (15.8 to 23.7%)	26.7 (20.6 to 34.1)
Ischemic heart disease	Benin	1995	Age-standardized		19.3% (15.6 to 23.7%)	28.1 (21.4 to 35.9)
Ischemic heart disease	Benin	2000	Age-standardized		19.4% (15.7 to 23.7%)	30.5 (22.5 to 39.9)
Ischemic heart disease	Benin	2005	Age-standardized		18.1% (14.5 to 22.0%)	29.2 (20.4 to 41.0)
Ischemic heart disease	Benin	2010	Age-standardized		17.8% (14.2 to 21.6%)	29.1 (17.9 to 44.3)
Ischemic heart disease	Benin	2015	Age-standardized		16.8% (13.4 to 20.5%)	27.9 (15.5 to 45.5)
Ischemic stroke	Benin	1990	All Ages	0.1 (0.1 to 0.2)	16.8% (13.6 to 19.9%)	2.9 (2.1 to 3.7)
Ischemic stroke	Benin	1995	All Ages	0.2 (0.1 to 0.2)	16.6% (13.5 to 19.8%)	2.7 (2.0 to 3.5)
Ischemic stroke	Benin	2000	All Ages	0.2 (0.1 to 0.3)	16.7% (13.7 to 19.8%)	2.8 (2.0 to 3.8)
Ischemic stroke	Benin	2005	All Ages	0.2 (0.1 to 0.3)	15.3% (12.3 to 18.4%)	2.6 (1.7 to 3.7)
Ischemic stroke	Benin	2010	All Ages	0.3 (0.1 to 0.4)	14.9% (11.9 to 17.9%)	2.6 (1.5 to 4.2)
Ischemic stroke	Benin	2015	All Ages	0.3 (0.1 to 0.5)	13.8% (10.8 to 16.8%)	2.5 (1.2 to 4.2)
Ischemic stroke	Benin	1990	Age-standardized		15.5% (11.9 to 19.1%)	9.3 (6.7 to 12.2)
Ischemic stroke	Benin	1995	Age-standardized		15.3% (11.7 to 18.9%)	8.9 (6.5 to 12.0)
Ischemic stroke	Benin	2000	Age-standardized		15.3% (11.9 to 18.9%)	9.8 (6.8 to 13.6)
Ischemic stroke	Benin	2005	Age-standardized		13.9% (10.5 to 17.3%)	9.4 (6.2 to 13.7)
Ischemic stroke	Benin	2010	Age-standardized		13.5% (10.1 to 16.9%)	9.5 (5.5 to 14.9)
Ischemic stroke	Benin	2015	Age-standardized		12.5% (9.2 to 15.8%)	8.9 (4.6 to 15.0)
Hemorrhagic stroke	Benin	1990	All Ages	0.2 (0.2 to 0.3)	17.1% (14.2 to 20.0%)	4.2 (3.2 to 5.2)
Hemorrhagic stroke	Benin	1995	All Ages	0.2 (0.2 to 0.3)	17.3% (14.4 to 20.1%)	4.0 (3.1 to 5.0)
Hemorrhagic stroke	Benin	2000	All Ages	0.3 (0.2 to 0.4)	17.9% (15.0 to 20.7%)	4.4 (3.2 to 5.8)
Hemorrhagic stroke	Benin	2005	All Ages	0.4 (0.2 to 0.5)	16.7% (14.0 to 19.5%)	4.3 (2.9 to 6.2)
Hemorrhagic stroke	Benin	2010	All Ages	0.4 (0.3 to 0.7)	16.5% (13.7 to 19.4%)	4.6 (2.6 to 7.5)
Hemorrhagic stroke	Benin	2015	All Ages	0.5 (0.2 to 0.9)	15.5% (12.6 to 18.5%)	4.6 (2.2 to 8.5)
Hemorrhagic stroke	Benin	1990	Age-standardized		17.3% (14.4 to 20.3%)	10.4 (8.0 to 13.0)
Hemorrhagic stroke	Benin	1995	Age-standardized		17.3% (14.4 to 20.2%)	10.0 (7.6 to 12.7)
Hemorrhagic stroke	Benin	2000	Age-standardized		17.4% (14.4 to 20.4%)	11.3 (8.2 to 14.7)
Hemorrhagic stroke	Benin	2005	Age-standardized		15.9% (13.0 to 19.0%)	11.2 (7.4 to 16.1)
Hemorrhagic stroke	Benin	2010	Age-standardized		15.5% (12.5 to 18.3%)	11.9 (6.9 to 18.9)

Cause name	Location	Year	Age group	Number (in thousands)	PAF	Rate (per 100,000)
Hemorrhagic stroke	Benin	2015	Age-standardized		14.3% (11.4 to 17.3%)	11.6 (5.9 to 20.1)
Chronic obstructive pulmonary disease	Benin	1990	All Ages	0.2 (0.1 to 0.3)	30.0% (19.2 to 42.8%)	3.8 (2.3 to 5.8)
Chronic obstructive pulmonary disease	Benin	1995	All Ages	0.2 (0.1 to 0.3)	29.2% (18.5 to 41.7%)	3.0 (1.8 to 4.5)
Chronic obstructive pulmonary disease	Benin	2000	All Ages	0.2 (0.1 to 0.3)	28.8% (18.2 to 41.4%)	2.6 (1.5 to 4.0)
Chronic obstructive pulmonary disease	Benin	2005	All Ages	0.2 (0.1 to 0.3)	25.9% (15.8 to 37.4%)	2.3 (1.2 to 3.8)
Chronic obstructive pulmonary disease	Benin	2010	All Ages	0.2 (0.1 to 0.4)	25.2% (15.1 to 36.3%)	2.3 (1.1 to 4.0)
Chronic obstructive pulmonary disease	Benin	2015	All Ages	0.2 (0.1 to 0.5)	23.0% (12.8 to 34.0%)	2.2 (0.9 to 4.2)
Chronic obstructive pulmonary disease	Benin	1990	Age-standardized		31.0% (20.0 to 44.2%)	11.6 (7.1 to 17.4)
Chronic obstructive pulmonary disease	Benin	1995	Age-standardized		30.5% (19.5 to 43.5%)	9.5 (5.7 to 14.1)
Chronic obstructive pulmonary disease	Benin	2000	Age-standardized		30.3% (19.3 to 43.6%)	8.6 (5.0 to 13.2)
Chronic obstructive pulmonary disease	Benin	2005	Age-standardized		27.0% (16.5 to 38.7%)	7.9 (4.2 to 12.8)
Chronic obstructive pulmonary disease	Benin	2010	Age-standardized		26.1% (15.7 to 37.5%)	7.8 (4.0 to 13.6)
Chronic obstructive pulmonary disease	Benin	2015	Age-standardized		23.7% (13.2 to 34.8%)	7.2 (3.2 to 13.5)
All causes	Burkina Faso	1990	All Ages	7.7 (5.7 to 9.9)	5.6% (4.2 to 7.2%)	87.7 (65.2 to 112.8)
All causes	Burkina Faso	1995	All Ages	7.9 (6.0 to 10.2)	5.1% (3.9 to 6.5%)	78.4 (59.1 to 100.6)
All causes	Burkina Faso	2000	All Ages	8.2 (6.4 to 10.2)	5.0% (4.0 to 6.1%)	70.3 (55.1 to 87.9)
All causes	Burkina Faso	2005	All Ages	7.4 (5.8 to 9.1)	4.6% (3.7 to 5.6%)	55.1 (43.3 to 68.0)
All causes	Burkina Faso	2010	All Ages	7.3 (5.4 to 9.3)	4.7% (3.8 to 5.7%)	46.6 (34.8 to 59.8)
All causes	Burkina Faso	2015	All Ages	6.7 (4.6 to 9.4)	4.3% (3.4 to 5.4%)	37.0 (25.3 to 51.9)
All causes	Burkina Faso	1990	Age-standardized		6.7% (5.5 to 7.9%)	123.4 (98.0 to 150.2)
All causes	Burkina Faso	1995	Age-standardized		6.4% (5.2 to 7.5%)	119.2 (94.0 to 146.0)
All causes	Burkina Faso	2000	Age-standardized		6.6% (5.5 to 7.6%)	118.9 (95.6 to 146.3)
All causes	Burkina Faso	2005	Age-standardized		6.5% (5.5 to 7.6%)	104.9 (83.7 to 130.5)
All causes	Burkina Faso	2010	Age-standardized		6.9% (5.9 to 8.0%)	101.2 (74.4 to 135.2)
All causes	Burkina Faso	2015	Age-standardized		6.2% (5.2 to 7.3%)	86.2 (55.2 to 129.2)
Lower respiratory infections	Burkina Faso	1990	All Ages	6.3 (4.5 to 8.5)	35.0% (27.1 to 42.0%)	71.5 (50.7 to 96.1)
Lower respiratory infections	Burkina Faso	1995	All Ages	6.4 (4.5 to 8.6)	34.8% (27.0 to 42.0%)	63.5 (44.8 to 85.5)
Lower respiratory infections	Burkina Faso	2000	All Ages	6.4 (4.7 to 8.3)	34.7% (26.9 to 41.8%)	54.7 (40.3 to 71.3)
Lower respiratory infections	Burkina Faso	2005	All Ages	5.5 (4.1 to 7.2)	32.7% (25.3 to 39.7%)	40.8 (30.6 to 53.2)
Lower respiratory infections	Burkina Faso	2010	All Ages	5.0 (3.6 to 6.6)	33.1% (25.3 to 40.2%)	32.3 (23.4 to 42.1)
Lower respiratory infections	Burkina Faso	2015	All Ages	4.3 (2.9 to 6.0)	26.5% (19.1 to 33.9%)	23.9 (15.9 to 33.4)
Lower respiratory infections	Burkina Faso	1990	Age-standardized		35.0% (27.1 to 42.0%)	76.2 (55.8 to 98.9)
Lower respiratory infections	Burkina Faso	1995	Age-standardized		34.8% (27.0 to 42.0%)	73.4 (53.9 to 95.6)
Lower respiratory infections	Burkina Faso	2000	Age-standardized		34.7% (26.9 to 41.8%)	67.9 (49.4 to 88.4)
Lower respiratory infections	Burkina Faso	2005	Age-standardized		32.7% (25.3 to 39.7%)	54.5 (40.4 to 71.0)
Lower respiratory infections	Burkina Faso	2010	Age-standardized		33.1% (25.3 to 40.2%)	49.1 (34.4 to 67.9)

Cause name	Location	Year	Age group	Number (in thousands)	PAF	Rate (per 100,000)
Lower respiratory infections	Burkina Faso	2015	Age-standardized		26.5% (19.1 to 33.9%)	38.7 (23.9 to 59.2)
Tracheal, bronchus, and lung cancer	Burkina Faso	1990	All Ages	0.1 (0.0 to 0.1)	26.8% (18.5 to 34.8%)	0.6 (0.4 to 0.9)
Tracheal, bronchus, and lung cancer	Burkina Faso	1995	All Ages	0.1 (0.0 to 0.1)	26.7% (18.3 to 34.9%)	0.6 (0.4 to 0.8)
Tracheal, bronchus, and lung cancer	Burkina Faso	2000	All Ages	0.1 (0.0 to 0.1)	26.5% (18.1 to 34.8%)	0.6 (0.4 to 0.8)
Tracheal, bronchus, and lung cancer	Burkina Faso	2005	All Ages	0.1 (0.0 to 0.1)	24.6% (16.5 to 32.7%)	0.5 (0.3 to 0.7)
Tracheal, bronchus, and lung cancer	Burkina Faso	2010	All Ages	0.1 (0.0 to 0.1)	25.1% (16.8 to 33.2%)	0.5 (0.3 to 0.7)
Tracheal, bronchus, and lung cancer	Burkina Faso	2015	All Ages	0.1 (0.0 to 0.1)	19.1% (11.9 to 27.0%)	0.4 (0.2 to 0.7)
Tracheal, bronchus, and lung cancer	Burkina Faso	1990	Age-standardized		27.0% (18.5 to 34.9%)	1.7 (1.1 to 2.4)
Tracheal, bronchus, and lung cancer	Burkina Faso	1995	Age-standardized		26.8% (18.4 to 35.1%)	1.7 (1.1 to 2.4)
Tracheal, bronchus, and lung cancer	Burkina Faso	2000	Age-standardized		26.7% (18.2 to 35.1%)	1.7 (1.1 to 2.4)
Tracheal, bronchus, and lung cancer	Burkina Faso	2005	Age-standardized		24.8% (16.6 to 33.0%)	1.5 (0.9 to 2.1)
Tracheal, bronchus, and lung cancer	Burkina Faso	2010	Age-standardized		25.3% (16.9 to 33.4%)	1.6 (0.9 to 2.4)
Tracheal, bronchus, and lung cancer	Burkina Faso	2015	Age-standardized		19.3% (12.0 to 27.2%)	1.3 (0.7 to 2.3)
Cardiovascular diseases	Burkina Faso	1990	All Ages	1.2 (1.0 to 1.5)	14.0% (12.4 to 15.8%)	13.4 (10.8 to 16.6)
Cardiovascular diseases	Burkina Faso	1995	All Ages	1.3 (1.0 to 1.6)	14.0% (12.4 to 15.8%)	12.4 (9.6 to 15.5)
Cardiovascular diseases	Burkina Faso	2000	All Ages	1.5 (1.2 to 2.0)	14.7% (12.9 to 16.6%)	13.3 (10.6 to 16.8)
Cardiovascular diseases	Burkina Faso	2005	All Ages	1.7 (1.3 to 2.2)	14.4% (12.7 to 16.4%)	12.4 (9.5 to 16.2)
Cardiovascular diseases	Burkina Faso	2010	All Ages	1.9 (1.3 to 2.7)	14.6% (12.9 to 16.5%)	12.4 (8.4 to 17.4)
Cardiovascular diseases	Burkina Faso	2015	All Ages	2.1 (1.2 to 3.4)	13.1% (11.4 to 15.0%)	11.5 (6.5 to 18.6)
Cardiovascular diseases	Burkina Faso	1990	Age-standardized		13.6% (11.7 to 15.7%)	38.7 (31.0 to 48.0)
Cardiovascular diseases	Burkina Faso	1995	Age-standardized		13.6% (11.7 to 15.8%)	37.8 (29.3 to 47.6)
Cardiovascular diseases	Burkina Faso	2000	Age-standardized		14.1% (12.2 to 16.4%)	43.3 (33.8 to 55.0)
Cardiovascular diseases	Burkina Faso	2005	Age-standardized		13.7% (11.6 to 16.2%)	43.7 (33.2 to 57.0)
Cardiovascular diseases	Burkina Faso	2010	Age-standardized		13.8% (11.7 to 16.2%)	45.4 (31.2 to 63.0)
Cardiovascular diseases	Burkina Faso	2015	Age-standardized		12.3% (10.2 to 14.4%)	41.8 (25.2 to 65.8)
Ischemic heart disease	Burkina Faso	1990	All Ages	0.7 (0.6 to 0.9)	22.4% (19.2 to 26.0%)	8.3 (6.5 to 10.4)
Ischemic heart disease	Burkina Faso	1995	All Ages	0.8 (0.6 to 1.0)	22.3% (19.1 to 25.8%)	7.8 (5.9 to 9.9)
Ischemic heart disease	Burkina Faso	2000	All Ages	1.0 (0.8 to 1.3)	22.2% (19.0 to 25.8%)	8.7 (6.8 to 11.2)
Ischemic heart disease	Burkina Faso	2005	All Ages	1.1 (0.8 to 1.5)	21.3% (18.2 to 24.7%)	8.2 (6.1 to 10.8)
Ischemic heart disease	Burkina Faso	2010	All Ages	1.3 (0.8 to 1.8)	21.5% (18.2 to 24.9%)	8.2 (5.4 to 11.5)
Ischemic heart disease	Burkina Faso	2015	All Ages	1.4 (0.8 to 2.3)	19.4% (16.2 to 22.8%)	7.7 (4.4 to 12.5)
Ischemic heart disease	Burkina Faso	1990	Age-standardized		20.2% (16.7 to 24.5%)	24.8 (19.0 to 31.7)
Ischemic heart disease	Burkina Faso	1995	Age-standardized		20.1% (16.5 to 24.4%)	24.4 (18.0 to 31.4)
Ischemic heart disease	Burkina Faso	2000	Age-standardized		20.0% (16.5 to 24.4%)	29.2 (21.9 to 38.0)
Ischemic heart disease	Burkina Faso	2005	Age-standardized		19.1% (15.5 to 23.4%)	29.6 (21.6 to 39.1)
Ischemic heart disease	Burkina Faso	2010	Age-standardized		19.1% (15.4 to 23.4%)	30.7 (20.4 to 43.0)

Cause name	Location	Year	Age group	Number (in thousands)	PAF	Rate (per 100,000)
Ischemic heart disease	Burkina Faso	2015	Age-standardized		17.1% (13.4 to 21.0%)	28.7 (16.8 to 45.2)
Ischemic stroke	Burkina Faso	1990	All Ages	0.2 (0.1 to 0.2)	18.2% (14.9 to 21.7%)	2.0 (1.4 to 2.7)
Ischemic stroke	Burkina Faso	1995	All Ages	0.2 (0.1 to 0.3)	18.0% (14.7 to 21.2%)	1.8 (1.3 to 2.5)
Ischemic stroke	Burkina Faso	2000	All Ages	0.2 (0.1 to 0.3)	17.8% (14.8 to 21.1%)	1.8 (1.3 to 2.4)
Ischemic stroke	Burkina Faso	2005	All Ages	0.2 (0.2 to 0.3)	16.9% (13.9 to 20.2%)	1.7 (1.2 to 2.3)
Ischemic stroke	Burkina Faso	2010	All Ages	0.3 (0.2 to 0.4)	17.0% (13.8 to 20.4%)	1.6 (1.1 to 2.4)
Ischemic stroke	Burkina Faso	2015	All Ages	0.3 (0.1 to 0.4)	14.7% (11.7 to 17.8%)	1.4 (0.8 to 2.3)
Ischemic stroke	Burkina Faso	1990	Age-standardized		16.6% (13.1 to 20.0%)	6.4 (4.5 to 8.6)
Ischemic stroke	Burkina Faso	1995	Age-standardized		16.5% (12.9 to 19.9%)	6.2 (4.3 to 8.4)
Ischemic stroke	Burkina Faso	2000	Age-standardized		16.3% (12.8 to 19.8%)	6.5 (4.7 to 8.9)
Ischemic stroke	Burkina Faso	2005	Age-standardized		15.4% (12.0 to 18.9%)	6.6 (4.6 to 9.3)
Ischemic stroke	Burkina Faso	2010	Age-standardized		15.5% (12.1 to 19.0%)	6.9 (4.4 to 10.1)
Ischemic stroke	Burkina Faso	2015	Age-standardized		13.3% (10.0 to 16.7%)	6.0 (3.4 to 9.8)
Hemorrhagic stroke	Burkina Faso	1990	All Ages	0.3 (0.2 to 0.4)	19.8% (16.7 to 22.8%)	3.1 (2.3 to 4.0)
Hemorrhagic stroke	Burkina Faso	1995	All Ages	0.3 (0.2 to 0.4)	19.6% (16.6 to 22.6%)	2.8 (2.1 to 3.6)
Hemorrhagic stroke	Burkina Faso	2000	All Ages	0.3 (0.2 to 0.4)	19.5% (16.7 to 22.5%)	2.8 (2.1 to 3.6)
Hemorrhagic stroke	Burkina Faso	2005	All Ages	0.3 (0.3 to 0.5)	18.7% (15.9 to 21.6%)	2.6 (1.9 to 3.5)
Hemorrhagic stroke	Burkina Faso	2010	All Ages	0.4 (0.3 to 0.6)	19.0% (16.0 to 22.0%)	2.6 (1.7 to 3.9)
Hemorrhagic stroke	Burkina Faso	2015	All Ages	0.4 (0.2 to 0.7)	16.4% (13.5 to 19.5%)	2.4 (1.3 to 4.1)
Hemorrhagic stroke	Burkina Faso	1990	Age-standardized		19.1% (16.1 to 22.2%)	7.6 (5.7 to 9.7)
Hemorrhagic stroke	Burkina Faso	1995	Age-standardized		19.0% (16.0 to 22.0%)	7.2 (5.3 to 9.5)
Hemorrhagic stroke	Burkina Faso	2000	Age-standardized		18.8% (15.9 to 21.9%)	7.5 (5.6 to 9.9)
Hemorrhagic stroke	Burkina Faso	2005	Age-standardized		17.7% (14.8 to 20.9%)	7.5 (5.4 to 10.1)
Hemorrhagic stroke	Burkina Faso	2010	Age-standardized		17.7% (14.7 to 20.8%)	7.8 (5.2 to 11.6)
Hemorrhagic stroke	Burkina Faso	2015	Age-standardized		15.2% (12.2 to 18.1%)	7.1 (3.9 to 11.8)
Chronic obstructive pulmonary disease	Burkina Faso	1990	All Ages	0.2 (0.1 to 0.3)	29.2% (18.8 to 41.7%)	2.2 (1.2 to 3.3)
Chronic obstructive pulmonary disease	Burkina Faso	1995	All Ages	0.2 (0.1 to 0.3)	29.3% (18.7 to 42.0%)	1.9 (1.1 to 3.0)
Chronic obstructive pulmonary disease	Burkina Faso	2000	All Ages	0.2 (0.1 to 0.3)	29.3% (19.1 to 42.3%)	1.7 (1.0 to 2.6)
Chronic obstructive pulmonary disease	Burkina Faso	2005	All Ages	0.2 (0.1 to 0.3)	27.9% (17.7 to 40.1%)	1.4 (0.8 to 2.1)
Chronic obstructive pulmonary disease	Burkina Faso	2010	All Ages	0.2 (0.1 to 0.3)	28.3% (17.9 to 40.7%)	1.4 (0.7 to 2.2)
Chronic obstructive pulmonary disease	Burkina Faso	2015	All Ages	0.2 (0.1 to 0.4)	23.3% (13.8 to 34.4%)	1.2 (0.6 to 2.1)
Chronic obstructive pulmonary disease	Burkina Faso	1990	Age-standardized		32.0% (20.7 to 45.3%)	6.7 (3.8 to 10.1)
Chronic obstructive pulmonary disease	Burkina Faso	1995	Age-standardized		31.9% (20.7 to 45.5%)	6.3 (3.6 to 9.7)
Chronic obstructive pulmonary disease	Burkina Faso	2000	Age-standardized		31.9% (20.6 to 45.6%)	6.0 (3.5 to 9.3)
Chronic obstructive pulmonary disease	Burkina Faso	2005	Age-standardized		30.3% (19.2 to 42.7%)	5.2 (3.0 to 7.9)
Chronic obstructive pulmonary disease	Burkina Faso	2010	Age-standardized		30.6% (19.6 to 44.1%)	5.2 (2.8 to 8.5)

Cause name	Location	Year	Age group	Number (in thousands)	PAF	Rate (per 100,000)
Chronic obstructive pulmonary disease	Burkina Faso	2015	Age-standardized		25.2% (14.9 to 36.8%)	4.5 (2.2 to 8.2)
All causes	Cameroon	1990	All Ages	10.0 (7.6 to 12.4)	7.2% (5.6 to 8.8%)	82.3 (62.9 to 102.2)
All causes	Cameroon	1995	All Ages	11.6 (9.0 to 14.6)	7.0% (5.4 to 8.6%)	83.4 (64.6 to 104.9)
All causes	Cameroon	2000	All Ages	13.5 (10.3 to 17.0)	6.7% (5.3 to 8.1%)	84.6 (64.2 to 106.3)
All causes	Cameroon	2005	All Ages	12.8 (9.8 to 16.3)	5.8% (4.8 to 7.0%)	70.7 (54.1 to 89.6)
All causes	Cameroon	2010	All Ages	12.8 (9.5 to 16.8)	6.0% (4.9 to 7.1%)	62.0 (46.2 to 81.4)
All causes	Cameroon	2015	All Ages	12.9 (8.6 to 18.6)	5.8% (4.7 to 7.0%)	55.0 (36.8 to 79.4)
All causes	Cameroon	1990	Age-standardized		9.5% (8.2 to 10.9%)	143.9 (118.1 to 171.6)
All causes	Cameroon	1995	Age-standardized		9.3% (7.9 to 10.7%)	150.5 (121.1 to 181.8)
All causes	Cameroon	2000	Age-standardized		9.0% (7.6 to 10.4%)	163.9 (125.4 to 204.2)
All causes	Cameroon	2005	Age-standardized		8.2% (7.0 to 9.4%)	147.2 (113.3 to 190.0)
All causes	Cameroon	2010	Age-standardized		8.2% (7.0 to 9.4%)	131.1 (95.5 to 174.2)
All causes	Cameroon	2015	Age-standardized		8.0% (6.8 to 9.3%)	123.1 (78.9 to 185.6)
Lower respiratory infections	Cameroon	1990	All Ages	6.6 (4.4 to 8.8)	43.2% (34.1 to 51.8%)	54.4 (36.5 to 72.7)
Lower respiratory infections	Cameroon	1995	All Ages	7.9 (5.4 to 10.5)	42.8% (34.0 to 51.4%)	56.3 (38.5 to 75.4)
Lower respiratory infections	Cameroon	2000	All Ages	8.7 (6.1 to 11.6)	42.3% (33.5 to 50.7%)	54.4 (38.2 to 72.5)
Lower respiratory infections	Cameroon	2005	All Ages	7.7 (5.5 to 10.2)	39.0% (30.5 to 46.9%)	42.5 (30.3 to 56.2)
Lower respiratory infections	Cameroon	2010	All Ages	7.7 (5.5 to 10.2)	37.0% (28.8 to 44.5%)	37.3 (26.7 to 49.6)
Lower respiratory infections	Cameroon	2015	All Ages	7.2 (4.8 to 10.1)	35.8% (27.9 to 43.3%)	30.8 (20.7 to 43.0)
Lower respiratory infections	Cameroon	1990	Age-standardized		43.2% (34.1 to 51.8%)	67.9 (49.8 to 88.2)
Lower respiratory infections	Cameroon	1995	Age-standardized		42.8% (34.0 to 51.4%)	75.9 (55.2 to 98.5)
Lower respiratory infections	Cameroon	2000	Age-standardized		42.3% (33.5 to 50.7%)	80.3 (55.9 to 105.9)
Lower respiratory infections	Cameroon	2005	Age-standardized		39.0% (30.5 to 46.9%)	67.6 (46.7 to 91.5)
Lower respiratory infections	Cameroon	2010	Age-standardized		37.0% (28.8 to 44.5%)	59.7 (41.6 to 82.8)
Lower respiratory infections	Cameroon	2015	Age-standardized		35.8% (27.9 to 43.3%)	53.3 (33.1 to 79.8)
Tracheal, bronchus, and lung cancer	Cameroon	1990	All Ages	0.2 (0.1 to 0.3)	34.9% (25.9 to 43.5%)	1.6 (1.1 to 2.2)
Tracheal, bronchus, and lung cancer	Cameroon	1995	All Ages	0.2 (0.1 to 0.3)	34.5% (25.4 to 43.2%)	1.6 (1.0 to 2.2)
Tracheal, bronchus, and lung cancer	Cameroon	2000	All Ages	0.3 (0.2 to 0.4)	33.8% (25.0 to 42.3%)	1.7 (1.0 to 2.4)
Tracheal, bronchus, and lung cancer	Cameroon	2005	All Ages	0.3 (0.2 to 0.4)	30.4% (21.4 to 38.7%)	1.5 (0.9 to 2.3)
Tracheal, bronchus, and lung cancer	Cameroon	2010	All Ages	0.3 (0.2 to 0.5)	28.3% (19.7 to 36.5%)	1.4 (0.8 to 2.2)
Tracheal, bronchus, and lung cancer	Cameroon	2015	All Ages	0.3 (0.1 to 0.6)	27.1% (18.5 to 35.6%)	1.4 (0.6 to 2.6)
Tracheal, bronchus, and lung cancer	Cameroon	1990	Age-standardized		35.1% (26.0 to 43.7%)	4.0 (2.7 to 5.4)
Tracheal, bronchus, and lung cancer	Cameroon	1995	Age-standardized		34.6% (25.5 to 43.5%)	4.0 (2.6 to 5.6)
Tracheal, bronchus, and lung cancer	Cameroon	2000	Age-standardized		34.0% (25.2 to 42.5%)	4.3 (2.6 to 6.1)
Tracheal, bronchus, and lung cancer	Cameroon	2005	Age-standardized		30.5% (21.5 to 38.9%)	4.1 (2.5 to 6.0)
Tracheal, bronchus, and lung cancer	Cameroon	2010	Age-standardized		28.5% (19.9 to 36.7%)	3.8 (2.2 to 5.9)

Cause name	Location	Year	Age group	Number (in thousands)	PAF	Rate (per 100,000)
Tracheal, bronchus, and lung cancer	Cameroon	2015	Age-standardized		27.2% (18.6 to 35.7%)	3.9 (1.8 to 6.9)
Cardiovascular diseases	Cameroon	1990	All Ages	2.6 (2.0 to 3.1)	17.3% (15.4 to 19.4%)	21.1 (16.8 to 25.7)
Cardiovascular diseases	Cameroon	1995	All Ages	3.0 (2.4 to 3.7)	17.5% (15.5 to 19.6%)	21.3 (16.8 to 26.6)
Cardiovascular diseases	Cameroon	2000	All Ages	3.9 (2.8 to 5.2)	18.2% (16.2 to 20.4%)	24.7 (17.7 to 32.4)
Cardiovascular diseases	Cameroon	2005	All Ages	4.2 (3.0 to 5.6)	17.5% (15.5 to 19.8%)	23.1 (16.8 to 30.7)
Cardiovascular diseases	Cameroon	2010	All Ages	4.2 (2.8 to 6.0)	16.8% (14.8 to 19.0%)	20.2 (13.7 to 28.9)
Cardiovascular diseases	Cameroon	2015	All Ages	4.7 (2.7 to 7.8)	16.7% (14.6 to 18.8%)	19.9 (11.4 to 33.2)
Cardiovascular diseases	Cameroon	1990	Age-standardized		16.8% (14.6 to 19.3%)	57.2 (45.5 to 70.0)
Cardiovascular diseases	Cameroon	1995	Age-standardized		17.0% (14.7 to 19.5%)	58.4 (46.2 to 72.5)
Cardiovascular diseases	Cameroon	2000	Age-standardized		17.6% (15.3 to 20.2%)	67.9 (50.0 to 87.7)
Cardiovascular diseases	Cameroon	2005	Age-standardized		16.9% (14.5 to 19.3%)	65.0 (48.0 to 86.8)
Cardiovascular diseases	Cameroon	2010	Age-standardized		16.2% (14.0 to 18.6%)	58.1 (39.7 to 82.4)
Cardiovascular diseases	Cameroon	2015	Age-standardized		16.0% (13.7 to 18.5%)	56.8 (33.7 to 92.3)
Ischemic heart disease	Cameroon	1990	All Ages	1.3 (1.0 to 1.6)	24.6% (21.1 to 28.9%)	10.6 (8.2 to 13.3)
Ischemic heart disease	Cameroon	1995	All Ages	1.6 (1.2 to 2.0)	24.6% (21.1 to 28.9%)	11.2 (8.4 to 14.5)
Ischemic heart disease	Cameroon	2000	All Ages	2.1 (1.5 to 2.8)	24.6% (21.2 to 28.7%)	13.0 (9.2 to 17.2)
Ischemic heart disease	Cameroon	2005	All Ages	2.2 (1.5 to 3.0)	23.4% (20.0 to 27.5%)	12.0 (8.5 to 16.4)
Ischemic heart disease	Cameroon	2010	All Ages	2.2 (1.4 to 3.1)	22.6% (19.2 to 26.7%)	10.5 (6.9 to 15.1)
Ischemic heart disease	Cameroon	2015	All Ages	2.4 (1.4 to 4.0)	22.3% (18.8 to 26.3%)	10.3 (5.8 to 17.2)
Ischemic heart disease	Cameroon	1990	Age-standardized		22.5% (18.5 to 28.0%)	29.4 (22.2 to 37.7)
Ischemic heart disease	Cameroon	1995	Age-standardized		22.6% (18.7 to 27.8%)	31.2 (23.1 to 40.5)
Ischemic heart disease	Cameroon	2000	Age-standardized		22.7% (18.8 to 27.7%)	36.4 (25.9 to 49.2)
Ischemic heart disease	Cameroon	2005	Age-standardized		21.6% (17.8 to 26.3%)	34.1 (24.0 to 46.8)
Ischemic heart disease	Cameroon	2010	Age-standardized		20.8% (17.1 to 25.7%)	30.3 (20.2 to 43.3)
Ischemic heart disease	Cameroon	2015	Age-standardized		20.5% (16.7 to 25.0%)	29.8 (17.4 to 49.0)
Ischemic stroke	Cameroon	1990	All Ages	0.5 (0.3 to 0.7)	20.4% (17.0 to 24.4%)	3.9 (2.8 to 5.4)
Ischemic stroke	Cameroon	1995	All Ages	0.5 (0.4 to 0.7)	20.2% (16.9 to 24.2%)	3.9 (2.8 to 5.2)
Ischemic stroke	Cameroon	2000	All Ages	0.7 (0.5 to 1.0)	20.0% (16.5 to 23.8%)	4.5 (3.1 to 6.1)
Ischemic stroke	Cameroon	2005	All Ages	0.8 (0.6 to 1.1)	18.6% (15.2 to 22.0%)	4.4 (3.0 to 5.9)
Ischemic stroke	Cameroon	2010	All Ages	0.8 (0.5 to 1.1)	17.7% (14.3 to 21.0%)	3.9 (2.5 to 5.5)
Ischemic stroke	Cameroon	2015	All Ages	0.9 (0.5 to 1.4)	17.3% (13.9 to 20.5%)	3.7 (2.1 to 6.1)
Ischemic stroke	Cameroon	1990	Age-standardized		19.0% (15.0 to 23.5%)	12.1 (8.6 to 16.5)
Ischemic stroke	Cameroon	1995	Age-standardized		18.9% (15.0 to 23.1%)	11.9 (8.5 to 16.2)
Ischemic stroke	Cameroon	2000	Age-standardized		18.8% (14.8 to 22.9%)	14.0 (9.7 to 19.1)
Ischemic stroke	Cameroon	2005	Age-standardized		17.5% (13.6 to 21.3%)	13.9 (9.4 to 18.9)
Ischemic stroke	Cameroon	2010	Age-standardized		16.7% (12.9 to 20.4%)	12.7 (8.2 to 18.0)

Cause name	Location	Year	Age group	Number (in thousands)	PAF	Rate (per 100,000)
Ischemic stroke	Cameroon	2015	Age-standardized		16.2% (12.4 to 20.1%)	12.3 (7.1 to 19.5)
Hemorrhagic stroke	Cameroon	1990	All Ages	0.8 (0.6 to 1.0)	22.1% (19.1 to 25.0%)	6.6 (5.1 to 8.3)
Hemorrhagic stroke	Cameroon	1995	All Ages	0.9 (0.7 to 1.1)	21.9% (18.9 to 24.9%)	6.3 (4.7 to 8.1)
Hemorrhagic stroke	Cameroon	2000	All Ages	1.1 (0.8 to 1.6)	22.0% (19.0 to 25.2%)	7.2 (5.0 to 9.9)
Hemorrhagic stroke	Cameroon	2005	All Ages	1.2 (0.8 to 1.7)	20.6% (17.7 to 23.6%)	6.7 (4.6 to 9.5)
Hemorrhagic stroke	Cameroon	2010	All Ages	1.2 (0.8 to 1.9)	19.6% (16.6 to 22.4%)	5.9 (3.7 to 9.1)
Hemorrhagic stroke	Cameroon	2015	All Ages	1.4 (0.7 to 2.4)	19.3% (16.2 to 22.2%)	5.8 (3.0 to 10.4)
Hemorrhagic stroke	Cameroon	1990	Age-standardized		21.6% (18.4 to 24.7%)	15.8 (11.9 to 19.8)
Hemorrhagic stroke	Cameroon	1995	Age-standardized		21.5% (18.4 to 24.8%)	15.3 (11.4 to 19.8)
Hemorrhagic stroke	Cameroon	2000	Age-standardized		21.5% (18.4 to 24.9%)	17.5 (12.3 to 24.2)
Hemorrhagic stroke	Cameroon	2005	Age-standardized		20.0% (16.9 to 23.2%)	16.9 (11.8 to 23.8)
Hemorrhagic stroke	Cameroon	2010	Age-standardized		19.0% (16.0 to 22.1%)	15.1 (9.6 to 23.0)
Hemorrhagic stroke	Cameroon	2015	Age-standardized		18.5% (15.4 to 21.6%)	14.8 (7.9 to 25.4)
Chronic obstructive pulmonary disease	Cameroon	1990	All Ages	0.6 (0.4 to 1.0)	38.5% (26.9 to 54.3%)	5.2 (3.3 to 7.9)
Chronic obstructive pulmonary disease	Cameroon	1995	All Ages	0.6 (0.4 to 0.9)	37.8% (26.0 to 53.4%)	4.2 (2.7 to 6.4)
Chronic obstructive pulmonary disease	Cameroon	2000	All Ages	0.6 (0.4 to 1.0)	37.1% (25.6 to 52.1%)	3.9 (2.3 to 6.0)
Chronic obstructive pulmonary disease	Cameroon	2005	All Ages	0.6 (0.4 to 1.0)	34.4% (23.2 to 49.0%)	3.5 (2.1 to 5.6)
Chronic obstructive pulmonary disease	Cameroon	2010	All Ages	0.6 (0.4 to 1.0)	32.6% (21.5 to 45.9%)	3.1 (1.7 to 4.8)
Chronic obstructive pulmonary disease	Cameroon	2015	All Ages	0.7 (0.3 to 1.2)	31.7% (20.5 to 44.6%)	3.0 (1.4 to 5.2)
Chronic obstructive pulmonary disease	Cameroon	1990	Age-standardized		39.4% (27.4 to 55.3%)	14.8 (9.4 to 22.6)
Chronic obstructive pulmonary disease	Cameroon	1995	Age-standardized		39.0% (27.0 to 54.9%)	12.2 (7.7 to 18.5)
Chronic obstructive pulmonary disease	Cameroon	2000	Age-standardized		38.4% (26.7 to 53.8%)	11.5 (6.8 to 17.3)
Chronic obstructive pulmonary disease	Cameroon	2005	Age-standardized		35.5% (24.1 to 50.6%)	10.6 (6.2 to 16.5)
Chronic obstructive pulmonary disease	Cameroon	2010	Age-standardized		33.8% (22.1 to 47.9%)	9.5 (5.3 to 14.5)
Chronic obstructive pulmonary disease	Cameroon	2015	Age-standardized		32.8% (21.2 to 46.2%)	9.1 (4.5 to 15.6)
All causes	Cape Verde	1990	All Ages	0.2 (0.1 to 0.2)	6.8% (5.5 to 8.0%)	45.2 (37.3 to 53.3)
All causes	Cape Verde	1995	All Ages	0.2 (0.1 to 0.2)	7.4% (6.1 to 8.7%)	47.2 (36.9 to 58.3)
All causes	Cape Verde	2000	All Ages	0.2 (0.1 to 0.3)	7.5% (6.3 to 8.9%)	45.9 (34.1 to 59.5)
All causes	Cape Verde	2005	All Ages	0.2 (0.1 to 0.2)	6.5% (5.2 to 7.9%)	38.8 (28.6 to 50.7)
All causes	Cape Verde	2010	All Ages	0.2 (0.1 to 0.2)	6.7% (5.4 to 8.1%)	39.2 (31.4 to 47.9)
All causes	Cape Verde	2015	All Ages	0.2 (0.1 to 0.3)	7.1% (5.7 to 8.5%)	37.4 (28.2 to 48.7)
All causes	Cape Verde	1990	Age-standardized		8.6% (7.2 to 10.1%)	85.7 (71.5 to 100.9)
All causes	Cape Verde	1995	Age-standardized		9.1% (7.6 to 10.7%)	95.6 (74.2 to 119.3)
All causes	Cape Verde	2000	Age-standardized		8.9% (7.5 to 10.5%)	94.2 (69.8 to 123.2)
All causes	Cape Verde	2005	Age-standardized		7.5% (6.1 to 9.1%)	76.6 (56.3 to 100.1)
All causes	Cape Verde	2010	Age-standardized		7.6% (6.1 to 9.2%)	68.9 (55.0 to 84.6)

Cause name	Location	Year	Age group	Number (in thousands)	PAF	Rate (per 100,000)
All causes	Cape Verde	2015	Age-standardized		8.0% (6.4 to 9.6%)	65.0 (49.0 to 83.9)
Lower respiratory infections	Cape Verde	1990	All Ages	0.1 (0.0 to 0.1)	31.2% (23.0 to 39.4%)	16.6 (11.3 to 22.9)
Lower respiratory infections	Cape Verde	1995	All Ages	0.1 (0.1 to 0.1)	31.5% (22.8 to 39.8%)	17.9 (12.3 to 24.2)
Lower respiratory infections	Cape Verde	2000	All Ages	0.1 (0.1 to 0.1)	29.9% (21.8 to 38.5%)	18.0 (12.0 to 24.8)
Lower respiratory infections	Cape Verde	2005	All Ages	0.1 (0.0 to 0.1)	24.0% (15.3 to 33.0%)	13.2 (8.0 to 20.0)
Lower respiratory infections	Cape Verde	2010	All Ages	0.1 (0.0 to 0.1)	24.3% (15.3 to 33.9%)	13.1 (8.1 to 18.9)
Lower respiratory infections	Cape Verde	2015	All Ages	0.1 (0.0 to 0.1)	26.5% (17.7 to 35.6%)	12.8 (7.8 to 18.4)
Lower respiratory infections	Cape Verde	1990	Age-standardized		31.2% (23.0 to 39.4%)	22.2 (15.0 to 32.2)
Lower respiratory infections	Cape Verde	1995	Age-standardized		31.5% (22.8 to 39.8%)	28.3 (19.5 to 38.8)
Lower respiratory infections	Cape Verde	2000	Age-standardized		29.9% (21.8 to 38.5%)	32.2 (21.1 to 45.6)
Lower respiratory infections	Cape Verde	2005	Age-standardized		24.0% (15.3 to 33.0%)	23.9 (14.7 to 35.7)
Lower respiratory infections	Cape Verde	2010	Age-standardized		24.3% (15.3 to 33.9%)	21.5 (13.1 to 31.2)
Lower respiratory infections	Cape Verde	2015	Age-standardized		26.5% (17.7 to 35.6%)	20.7 (12.4 to 30.1)
Tracheal, bronchus, and lung cancer	Cape Verde	1990	All Ages	0.0 (0.0 to 0.0)	23.1% (14.2 to 31.9%)	1.5 (0.9 to 2.1)
Tracheal, bronchus, and lung cancer	Cape Verde	1995	All Ages	0.0 (0.0 to 0.0)	23.4% (14.4 to 33.0%)	1.7 (1.0 to 2.5)
Tracheal, bronchus, and lung cancer	Cape Verde	2000	All Ages	0.0 (0.0 to 0.0)	22.0% (13.7 to 31.3%)	1.8 (1.0 to 2.8)
Tracheal, bronchus, and lung cancer	Cape Verde	2005	All Ages	0.0 (0.0 to 0.0)	17.1% (10.2 to 25.7%)	1.6 (0.9 to 2.6)
Tracheal, bronchus, and lung cancer	Cape Verde	2010	All Ages	0.0 (0.0 to 0.0)	17.4% (10.0 to 26.2%)	1.7 (1.0 to 2.7)
Tracheal, bronchus, and lung cancer	Cape Verde	2015	All Ages	0.0 (0.0 to 0.0)	19.1% (11.0 to 27.5%)	1.6 (0.9 to 2.5)
Tracheal, bronchus, and lung cancer	Cape Verde	1990	Age-standardized		23.2% (14.3 to 32.0%)	3.1 (1.9 to 4.4)
Tracheal, bronchus, and lung cancer	Cape Verde	1995	Age-standardized		23.5% (14.5 to 33.1%)	3.7 (2.1 to 5.5)
Tracheal, bronchus, and lung cancer	Cape Verde	2000	Age-standardized		22.1% (13.7 to 31.4%)	3.8 (2.2 to 5.9)
Tracheal, bronchus, and lung cancer	Cape Verde	2005	Age-standardized		17.2% (10.2 to 25.8%)	3.1 (1.7 to 4.9)
Tracheal, bronchus, and lung cancer	Cape Verde	2010	Age-standardized		17.5% (10.0 to 26.2%)	3.1 (1.7 to 4.7)
Tracheal, bronchus, and lung cancer	Cape Verde	2015	Age-standardized		19.2% (11.0 to 27.6%)	3.0 (1.7 to 4.6)
Cardiovascular diseases	Cape Verde	1990	All Ages	0.1 (0.1 to 0.1)	13.6% (11.6 to 15.9%)	20.5 (17.2 to 24.0)
Cardiovascular diseases	Cape Verde	1995	All Ages	0.1 (0.1 to 0.1)	14.5% (12.2 to 16.9%)	22.6 (17.5 to 28.5)
Cardiovascular diseases	Cape Verde	2000	All Ages	0.1 (0.1 to 0.1)	14.9% (12.6 to 17.4%)	23.0 (16.7 to 31.1)
Cardiovascular diseases	Cape Verde	2005	All Ages	0.1 (0.1 to 0.1)	13.2% (10.9 to 15.5%)	21.3 (15.6 to 27.6)
Cardiovascular diseases	Cape Verde	2010	All Ages	0.1 (0.1 to 0.1)	13.0% (10.5 to 15.6%)	21.6 (17.5 to 26.0)
Cardiovascular diseases	Cape Verde	2015	All Ages	0.1 (0.1 to 0.1)	13.4% (10.9 to 15.9%)	20.6 (15.8 to 26.8)
Cardiovascular diseases	Cape Verde	1990	Age-standardized		13.8% (11.6 to 16.2%)	45.3 (37.8 to 53.3)
Cardiovascular diseases	Cape Verde	1995	Age-standardized		14.5% (11.9 to 17.0%)	51.9 (40.0 to 65.2)
Cardiovascular diseases	Cape Verde	2000	Age-standardized		14.6% (12.2 to 17.2%)	51.3 (37.2 to 68.3)
Cardiovascular diseases	Cape Verde	2005	Age-standardized		13.0% (10.4 to 15.5%)	44.0 (32.5 to 56.8)
Cardiovascular diseases	Cape Verde	2010	Age-standardized		13.0% (10.5 to 15.6%)	39.4 (31.6 to 47.7)

Cause name	Location	Year	Age group	Number (in thousands)	PAF	Rate (per 100,000)
Cardiovascular diseases	Cape Verde	2015	Age-standardized		13.4% (10.9 to 16.0%)	36.9 (28.0 to 47.6)
Ischemic heart disease	Cape Verde	1990	All Ages	0.0 (0.0 to 0.0)	19.5% (15.9 to 23.7%)	8.6 (6.9 to 10.5)
Ischemic heart disease	Cape Verde	1995	All Ages	0.0 (0.0 to 0.1)	19.6% (15.9 to 23.7%)	10.3 (7.8 to 13.7)
Ischemic heart disease	Cape Verde	2000	All Ages	0.1 (0.0 to 0.1)	19.1% (15.4 to 23.2%)	11.8 (8.4 to 16.2)
Ischemic heart disease	Cape Verde	2005	All Ages	0.1 (0.0 to 0.1)	17.1% (13.5 to 21.0%)	11.1 (7.9 to 14.7)
Ischemic heart disease	Cape Verde	2010	All Ages	0.1 (0.0 to 0.1)	16.7% (13.1 to 20.6%)	11.2 (8.7 to 14.1)
Ischemic heart disease	Cape Verde	2015	All Ages	0.1 (0.0 to 0.1)	17.1% (13.3 to 21.3%)	10.6 (7.8 to 14.0)
Ischemic heart disease	Cape Verde	1990	Age-standardized		18.9% (15.2 to 23.4%)	19.1 (15.0 to 23.8)
Ischemic heart disease	Cape Verde	1995	Age-standardized		18.9% (15.1 to 23.5%)	23.8 (17.7 to 31.5)
Ischemic heart disease	Cape Verde	2000	Age-standardized		18.3% (14.5 to 22.4%)	26.3 (18.3 to 36.5)
Ischemic heart disease	Cape Verde	2005	Age-standardized		16.5% (12.8 to 20.4%)	22.9 (16.3 to 30.7)
Ischemic heart disease	Cape Verde	2010	Age-standardized		16.5% (12.9 to 20.5%)	20.6 (15.8 to 26.0)
Ischemic heart disease	Cape Verde	2015	Age-standardized		17.0% (13.2 to 21.1%)	19.3 (14.1 to 25.3)
Ischemic stroke	Cape Verde	1990	All Ages	0.0 (0.0 to 0.0)	15.0% (11.3 to 18.7%)	5.1 (3.7 to 6.6)
Ischemic stroke	Cape Verde	1995	All Ages	0.0 (0.0 to 0.0)	15.2% (11.6 to 18.9%)	5.1 (3.6 to 6.9)
Ischemic stroke	Cape Verde	2000	All Ages	0.0 (0.0 to 0.0)	14.7% (11.0 to 18.3%)	4.5 (3.1 to 6.4)
Ischemic stroke	Cape Verde	2005	All Ages	0.0 (0.0 to 0.0)	12.7% (9.0 to 16.2%)	4.1 (2.7 to 5.7)
Ischemic stroke	Cape Verde	2010	All Ages	0.0 (0.0 to 0.0)	12.4% (8.8 to 16.2%)	4.3 (3.0 to 5.8)
Ischemic stroke	Cape Verde	2015	All Ages	0.0 (0.0 to 0.0)	12.8% (8.9 to 16.7%)	4.2 (2.8 to 6.0)
Ischemic stroke	Cape Verde	1990	Age-standardized		14.6% (10.8 to 18.6%)	11.7 (8.4 to 15.3)
Ischemic stroke	Cape Verde	1995	Age-standardized		14.7% (10.9 to 18.6%)	12.3 (8.5 to 16.8)
Ischemic stroke	Cape Verde	2000	Age-standardized		14.1% (10.3 to 18.1%)	10.8 (7.1 to 15.4)
Ischemic stroke	Cape Verde	2005	Age-standardized		12.2% (8.3 to 16.2%)	9.0 (5.9 to 12.5)
Ischemic stroke	Cape Verde	2010	Age-standardized		12.3% (8.6 to 16.4%)	8.0 (5.5 to 11.0)
Ischemic stroke	Cape Verde	2015	Age-standardized		12.9% (9.0 to 16.7%)	7.7 (5.0 to 10.9)
Hemorrhagic stroke	Cape Verde	1990	All Ages	0.0 (0.0 to 0.0)	16.3% (13.2 to 19.4%)	6.8 (5.4 to 8.3)
Hemorrhagic stroke	Cape Verde	1995	All Ages	0.0 (0.0 to 0.0)	16.5% (13.3 to 19.8%)	7.2 (5.3 to 9.3)
Hemorrhagic stroke	Cape Verde	2000	All Ages	0.0 (0.0 to 0.0)	16.0% (12.6 to 19.2%)	6.6 (4.5 to 9.3)
Hemorrhagic stroke	Cape Verde	2005	All Ages	0.0 (0.0 to 0.0)	14.0% (10.7 to 17.1%)	6.1 (4.2 to 8.2)
Hemorrhagic stroke	Cape Verde	2010	All Ages	0.0 (0.0 to 0.0)	13.9% (10.7 to 17.4%)	6.1 (4.6 to 7.8)
Hemorrhagic stroke	Cape Verde	2015	All Ages	0.0 (0.0 to 0.0)	14.7% (11.4 to 18.0%)	5.8 (4.2 to 8.0)
Hemorrhagic stroke	Cape Verde	1990	Age-standardized		16.6% (13.3 to 19.8%)	14.4 (11.1 to 17.7)
Hemorrhagic stroke	Cape Verde	1995	Age-standardized		16.6% (13.2 to 19.9%)	15.7 (11.6 to 20.4)
Hemorrhagic stroke	Cape Verde	2000	Age-standardized		15.8% (12.4 to 19.4%)	14.3 (9.7 to 20.1)
Hemorrhagic stroke	Cape Verde	2005	Age-standardized		13.8% (10.4 to 17.1%)	12.1 (8.3 to 16.5)
Hemorrhagic stroke	Cape Verde	2010	Age-standardized		13.8% (10.5 to 17.3%)	10.8 (8.0 to 13.6)

Cause name	Location	Year	Age group	Number (in thousands)	PAF	Rate (per 100,000)
Hemorrhagic stroke	Cape Verde	2015	Age-standardized		14.5% (11.1 to 17.9%)	9.9 (7.2 to 13.6)
Chronic obstructive pulmonary disease	Cape Verde	1990	All Ages	0.0 (0.0 to 0.0)	28.5% (16.9 to 41.0%)	6.7 (3.9 to 9.9)
Chronic obstructive pulmonary disease	Cape Verde	1995	All Ages	0.0 (0.0 to 0.0)	28.8% (17.5 to 41.4%)	5.0 (2.8 to 7.5)
Chronic obstructive pulmonary disease	Cape Verde	2000	All Ages	0.0 (0.0 to 0.0)	27.7% (16.4 to 41.1%)	3.0 (1.6 to 5.0)
Chronic obstructive pulmonary disease	Cape Verde	2005	All Ages	0.0 (0.0 to 0.0)	23.2% (12.4 to 34.5%)	2.7 (1.3 to 4.2)
Chronic obstructive pulmonary disease	Cape Verde	2010	All Ages	0.0 (0.0 to 0.0)	23.6% (12.8 to 35.1%)	2.7 (1.4 to 4.1)
Chronic obstructive pulmonary disease	Cape Verde	2015	All Ages	0.0 (0.0 to 0.0)	25.3% (14.1 to 37.5%)	2.4 (1.2 to 3.9)
Chronic obstructive pulmonary disease	Cape Verde	1990	Age-standardized		29.1% (17.4 to 42.0%)	15.2 (8.8 to 22.6)
Chronic obstructive pulmonary disease	Cape Verde	1995	Age-standardized		29.4% (17.8 to 42.3%)	11.8 (6.6 to 17.9)
Chronic obstructive pulmonary disease	Cape Verde	2000	Age-standardized		28.2% (16.7 to 41.8%)	6.9 (3.7 to 11.4)
Chronic obstructive pulmonary disease	Cape Verde	2005	Age-standardized		23.5% (12.6 to 34.9%)	5.6 (2.8 to 8.8)
Chronic obstructive pulmonary disease	Cape Verde	2010	Age-standardized		23.8% (13.0 to 35.5%)	4.9 (2.6 to 7.5)
Chronic obstructive pulmonary disease	Cape Verde	2015	Age-standardized		25.5% (14.2 to 37.8%)	4.4 (2.3 to 7.1)
All causes	Chad	1990	All Ages	4.9 (3.6 to 6.3)	5.3% (4.0 to 6.8%)	81.7 (61.1 to 105.1)
All causes	Chad	1995	All Ages	5.6 (4.2 to 7.2)	5.3% (4.0 to 6.8%)	80.1 (59.7 to 102.0)
All causes	Chad	2000	All Ages	7.0 (5.2 to 9.1)	5.4% (4.1 to 7.0%)	84.4 (62.6 to 109.5)
All causes	Chad	2005	All Ages	7.2 (5.2 to 9.5)	5.1% (3.9 to 6.5%)	71.9 (52.1 to 94.7)
All causes	Chad	2010	All Ages	8.1 (5.7 to 11.2)	5.6% (4.3 to 7.1%)	68.3 (48.0 to 94.0)
All causes	Chad	2015	All Ages	7.8 (5.2 to 11.0)	5.1% (3.9 to 6.5%)	55.5 (36.9 to 78.2)
All causes	Chad	1990	Age-standardized		6.7% (5.5 to 7.9%)	111.5 (90.8 to 136.2)
All causes	Chad	1995	Age-standardized		6.8% (5.6 to 8.0%)	116.9 (93.0 to 142.3)
All causes	Chad	2000	Age-standardized		7.0% (5.8 to 8.3%)	135.4 (105.9 to 170.4)
All causes	Chad	2005	Age-standardized		6.9% (5.7 to 8.1%)	126.5 (90.0 to 174.8)
All causes	Chad	2010	Age-standardized		7.4% (6.2 to 8.6%)	123.3 (77.9 to 180.8)
All causes	Chad	2015	Age-standardized		6.9% (5.8 to 8.1%)	110.0 (66.2 to 169.5)
Lower respiratory infections	Chad	1990	All Ages	3.9 (2.7 to 5.3)	32.8% (25.6 to 39.5%)	65.2 (45.8 to 88.3)
Lower respiratory infections	Chad	1995	All Ages	4.5 (3.1 to 6.0)	32.8% (25.4 to 39.4%)	64.4 (44.9 to 85.8)
Lower respiratory infections	Chad	2000	All Ages	5.5 (3.8 to 7.5)	33.1% (25.9 to 40.1%)	65.6 (45.9 to 89.8)
Lower respiratory infections	Chad	2005	All Ages	5.5 (3.8 to 7.5)	32.1% (24.8 to 38.9%)	54.2 (37.8 to 74.3)
Lower respiratory infections	Chad	2010	All Ages	6.1 (4.2 to 8.4)	31.3% (24.0 to 38.5%)	51.2 (35.3 to 70.9)
Lower respiratory infections	Chad	2015	All Ages	5.6 (3.7 to 7.9)	28.3% (21.3 to 35.7%)	39.6 (26.3 to 56.1)
Lower respiratory infections	Chad	1990	Age-standardized		32.8% (25.6 to 39.5%)	62.3 (45.4 to 82.6)
Lower respiratory infections	Chad	1995	Age-standardized		32.8% (25.4 to 39.4%)	67.5 (48.3 to 89.3)
Lower respiratory infections	Chad	2000	Age-standardized		33.1% (25.9 to 40.1%)	75.7 (52.6 to 102.5)
Lower respiratory infections	Chad	2005	Age-standardized		32.1% (24.8 to 38.9%)	67.1 (44.8 to 94.9)
Lower respiratory infections	Chad	2010	Age-standardized		31.3% (24.0 to 38.5%)	63.6 (39.4 to 93.2)

Cause name	Location	Year	Age group	Number (in thousands)	PAF	Rate (per 100,000)
Lower respiratory infections	Chad	2015	Age-standardized		28.3% (21.3 to 35.7%)	53.6 (32.1 to 81.1)
Tracheal, bronchus, and lung cancer	Chad	1990	All Ages	0.0 (0.0 to 0.1)	25.6% (17.5 to 33.5%)	0.7 (0.4 to 1.0)
Tracheal, bronchus, and lung cancer	Chad	1995	All Ages	0.1 (0.0 to 0.1)	25.5% (17.6 to 33.5%)	0.7 (0.4 to 1.0)
Tracheal, bronchus, and lung cancer	Chad	2000	All Ages	0.1 (0.0 to 0.1)	25.6% (17.7 to 33.8%)	0.7 (0.5 to 1.1)
Tracheal, bronchus, and lung cancer	Chad	2005	All Ages	0.1 (0.0 to 0.1)	24.4% (16.4 to 32.3%)	0.7 (0.4 to 1.1)
Tracheal, bronchus, and lung cancer	Chad	2010	All Ages	0.1 (0.0 to 0.1)	23.7% (15.7 to 31.6%)	0.7 (0.4 to 1.3)
Tracheal, bronchus, and lung cancer	Chad	2015	All Ages	0.1 (0.0 to 0.2)	20.9% (13.4 to 28.8%)	0.7 (0.3 to 1.3)
Tracheal, bronchus, and lung cancer	Chad	1990	Age-standardized		25.7% (17.6 to 33.7%)	1.8 (1.2 to 2.6)
Tracheal, bronchus, and lung cancer	Chad	1995	Age-standardized		25.7% (17.7 to 33.7%)	1.9 (1.2 to 2.7)
Tracheal, bronchus, and lung cancer	Chad	2000	Age-standardized		25.7% (17.8 to 34.0%)	2.2 (1.4 to 3.1)
Tracheal, bronchus, and lung cancer	Chad	2005	Age-standardized		24.6% (16.5 to 32.5%)	2.2 (1.3 to 3.4)
Tracheal, bronchus, and lung cancer	Chad	2010	Age-standardized		23.9% (15.8 to 31.8%)	2.4 (1.2 to 4.1)
Tracheal, bronchus, and lung cancer	Chad	2015	Age-standardized		21.0% (13.5 to 28.9%)	2.2 (1.0 to 4.2)
Cardiovascular diseases	Chad	1990	All Ages	0.8 (0.6 to 1.0)	14.1% (12.3 to 15.9%)	13.3 (10.5 to 16.6)
Cardiovascular diseases	Chad	1995	All Ages	0.9 (0.7 to 1.1)	14.4% (12.7 to 16.2%)	13.1 (10.2 to 16.3)
Cardiovascular diseases	Chad	2000	All Ages	1.3 (1.0 to 1.7)	15.3% (13.5 to 17.1%)	16.0 (12.1 to 20.5)
Cardiovascular diseases	Chad	2005	All Ages	1.5 (1.0 to 2.2)	15.2% (13.2 to 17.2%)	15.1 (9.7 to 22.2)
Cardiovascular diseases	Chad	2010	All Ages	1.7 (0.9 to 2.8)	14.9% (12.9 to 16.9%)	14.5 (7.6 to 23.1)
Cardiovascular diseases	Chad	2015	All Ages	1.9 (0.9 to 3.2)	14.1% (12.2 to 16.3%)	13.4 (6.7 to 23.0)
Cardiovascular diseases	Chad	1990	Age-standardized		13.9% (11.9 to 16.0%)	39.6 (31.3 to 49.4)
Cardiovascular diseases	Chad	1995	Age-standardized		14.1% (12.0 to 16.3%)	40.9 (31.9 to 50.7)
Cardiovascular diseases	Chad	2000	Age-standardized		14.9% (12.8 to 17.0%)	50.5 (38.5 to 64.4)
Cardiovascular diseases	Chad	2005	Age-standardized		14.8% (12.7 to 17.0%)	50.3 (32.9 to 72.5)
Cardiovascular diseases	Chad	2010	Age-standardized		14.6% (12.4 to 16.7%)	50.2 (27.7 to 78.4)
Cardiovascular diseases	Chad	2015	Age-standardized		13.8% (11.7 to 16.1%)	47.4 (25.3 to 77.6)
Ischemic heart disease	Chad	1990	All Ages	0.4 (0.3 to 0.5)	20.7% (17.5 to 24.4%)	6.7 (5.1 to 8.6)
Ischemic heart disease	Chad	1995	All Ages	0.5 (0.4 to 0.6)	20.8% (17.6 to 24.4%)	7.0 (5.3 to 9.1)
Ischemic heart disease	Chad	2000	All Ages	0.7 (0.5 to 1.0)	21.3% (18.0 to 24.9%)	8.6 (6.2 to 11.3)
Ischemic heart disease	Chad	2005	All Ages	0.8 (0.5 to 1.2)	20.8% (17.5 to 24.6%)	7.9 (4.9 to 11.8)
Ischemic heart disease	Chad	2010	All Ages	0.9 (0.5 to 1.5)	20.6% (17.2 to 24.2%)	7.6 (4.0 to 12.5)
Ischemic heart disease	Chad	2015	All Ages	1.0 (0.5 to 1.7)	19.6% (16.4 to 23.2%)	7.0 (3.5 to 12.0)
Ischemic heart disease	Chad	1990	Age-standardized		18.8% (15.1 to 23.2%)	20.9 (15.4 to 27.3)
Ischemic heart disease	Chad	1995	Age-standardized		18.9% (15.3 to 23.3%)	22.6 (16.9 to 29.3)
Ischemic heart disease	Chad	2000	Age-standardized		19.4% (15.7 to 23.7%)	27.9 (20.0 to 36.8)
Ischemic heart disease	Chad	2005	Age-standardized		19.0% (15.1 to 23.1%)	27.2 (17.0 to 39.6)
Ischemic heart disease	Chad	2010	Age-standardized		18.7% (14.8 to 22.9%)	27.0 (14.5 to 43.8)

Cause name	Location	Year	Age group	Number (in thousands)	PAF	Rate (per 100,000)
Ischemic heart disease	Chad	2015	Age-standardized		17.8% (14.0 to 21.7%)	25.5 (13.0 to 41.3)
Ischemic stroke	Chad	1990	All Ages	0.2 (0.1 to 0.2)	16.6% (13.4 to 19.7%)	2.8 (1.9 to 4.0)
Ischemic stroke	Chad	1995	All Ages	0.2 (0.1 to 0.3)	16.6% (13.5 to 19.7%)	2.6 (1.8 to 3.7)
Ischemic stroke	Chad	2000	All Ages	0.3 (0.2 to 0.4)	16.9% (13.8 to 20.0%)	3.1 (2.0 to 4.3)
Ischemic stroke	Chad	2005	All Ages	0.3 (0.2 to 0.5)	16.4% (13.3 to 19.5%)	2.9 (1.8 to 4.5)
Ischemic stroke	Chad	2010	All Ages	0.3 (0.2 to 0.5)	16.0% (13.0 to 19.1%)	2.7 (1.5 to 4.5)
Ischemic stroke	Chad	2015	All Ages	0.4 (0.2 to 0.6)	15.0% (11.9 to 18.1%)	2.5 (1.3 to 4.4)
Ischemic stroke	Chad	1990	Age-standardized		15.4% (11.8 to 18.9%)	9.1 (6.2 to 13.0)
Ischemic stroke	Chad	1995	Age-standardized		15.4% (11.9 to 19.0%)	9.0 (6.1 to 13.0)
Ischemic stroke	Chad	2000	Age-standardized		15.6% (12.1 to 19.0%)	10.9 (7.2 to 15.8)
Ischemic stroke	Chad	2005	Age-standardized		15.1% (11.7 to 18.7%)	11.0 (6.7 to 17.2)
Ischemic stroke	Chad	2010	Age-standardized		14.8% (11.3 to 18.3%)	10.9 (5.9 to 17.9)
Ischemic stroke	Chad	2015	Age-standardized		13.8% (10.4 to 17.3%)	10.2 (5.4 to 17.7)
Hemorrhagic stroke	Chad	1990	All Ages	0.2 (0.1 to 0.3)	17.1% (14.3 to 20.0%)	3.8 (2.6 to 5.1)
Hemorrhagic stroke	Chad	1995	All Ages	0.3 (0.2 to 0.3)	17.2% (14.5 to 20.0%)	3.5 (2.4 to 4.7)
Hemorrhagic stroke	Chad	2000	All Ages	0.4 (0.3 to 0.5)	17.9% (15.2 to 20.8%)	4.3 (3.1 to 5.9)
Hemorrhagic stroke	Chad	2005	All Ages	0.4 (0.3 to 0.7)	17.4% (14.7 to 20.5%)	4.3 (2.6 to 6.8)
Hemorrhagic stroke	Chad	2010	All Ages	0.5 (0.3 to 0.8)	16.8% (14.0 to 20.0%)	4.2 (2.1 to 7.1)
Hemorrhagic stroke	Chad	2015	All Ages	0.6 (0.3 to 1.0)	15.9% (12.9 to 19.0%)	3.9 (1.8 to 7.1)
Hemorrhagic stroke	Chad	1990	Age-standardized		17.3% (14.4 to 20.3%)	9.7 (6.4 to 12.8)
Hemorrhagic stroke	Chad	1995	Age-standardized		17.4% (14.5 to 20.5%)	9.3 (6.3 to 12.4)
Hemorrhagic stroke	Chad	2000	Age-standardized		17.9% (15.0 to 20.9%)	11.6 (8.1 to 15.9)
Hemorrhagic stroke	Chad	2005	Age-standardized		17.4% (14.4 to 20.3%)	12.0 (7.5 to 18.7)
Hemorrhagic stroke	Chad	2010	Age-standardized		17.0% (14.1 to 20.0%)	12.3 (6.6 to 20.2)
Hemorrhagic stroke	Chad	2015	Age-standardized		15.8% (12.8 to 18.9%)	11.7 (5.7 to 20.5)
Chronic obstructive pulmonary disease	Chad	1990	All Ages	0.1 (0.1 to 0.3)	29.6% (19.3 to 42.4%)	2.4 (1.0 to 4.5)
Chronic obstructive pulmonary disease	Chad	1995	All Ages	0.1 (0.1 to 0.3)	29.0% (18.8 to 41.2%)	2.0 (0.9 to 3.7)
Chronic obstructive pulmonary disease	Chad	2000	All Ages	0.2 (0.1 to 0.3)	28.7% (18.2 to 41.1%)	2.0 (0.9 to 3.6)
Chronic obstructive pulmonary disease	Chad	2005	All Ages	0.2 (0.1 to 0.4)	27.9% (17.9 to 40.0%)	1.9 (0.8 to 3.5)
Chronic obstructive pulmonary disease	Chad	2010	All Ages	0.2 (0.1 to 0.4)	27.2% (17.1 to 39.2%)	1.9 (0.8 to 3.5)
Chronic obstructive pulmonary disease	Chad	2015	All Ages	0.2 (0.1 to 0.5)	25.0% (15.0 to 36.1%)	1.7 (0.7 to 3.5)
Chronic obstructive pulmonary disease	Chad	1990	Age-standardized		30.9% (20.1 to 44.3%)	7.7 (3.2 to 14.2)
Chronic obstructive pulmonary disease	Chad	1995	Age-standardized		30.8% (19.9 to 44.0%)	6.6 (2.9 to 12.3)
Chronic obstructive pulmonary disease	Chad	2000	Age-standardized		30.9% (19.7 to 44.0%)	6.9 (3.2 to 12.5)
Chronic obstructive pulmonary disease	Chad	2005	Age-standardized		30.0% (19.1 to 42.7%)	6.9 (3.1 to 12.2)
Chronic obstructive pulmonary disease	Chad	2010	Age-standardized		29.3% (18.7 to 42.2%)	7.2 (3.3 to 12.9)

Cause name	Location	Year	Age group	Number (in thousands)	PAF	Rate (per 100,000)
Chronic obstructive pulmonary disease	Chad	2015	Age-standardized		26.9% (16.1 to 38.8%)	6.7 (2.9 to 13.0)
All causes	Cote d'Ivoire	1990	All Ages	6.1 (4.6 to 7.9)	4.1% (3.1 to 5.1%)	50.3 (37.7 to 64.9)
All causes	Cote d'Ivoire	1995	All Ages	8.0 (6.0 to 10.4)	4.1% (3.2 to 5.1%)	55.7 (41.5 to 72.6)
All causes	Cote d'Ivoire	2000	All Ages	8.7 (6.5 to 11.5)	3.9% (3.0 to 4.9%)	52.8 (39.4 to 69.7)
All causes	Cote d'Ivoire	2005	All Ages	7.7 (5.6 to 10.2)	3.4% (2.6 to 4.3%)	42.3 (30.8 to 56.1)
All causes	Cote d'Ivoire	2010	All Ages	7.8 (5.4 to 10.9)	3.6% (2.8 to 4.6%)	38.8 (26.8 to 54.2)
All causes	Cote d'Ivoire	2015	All Ages	7.7 (4.9 to 11.7)	3.6% (2.7 to 4.7%)	34.1 (21.4 to 51.8)
All causes	Cote d'Ivoire	1990	Age-standardized		5.9% (4.9 to 7.0%)	102.4 (80.8 to 127.0)
All causes	Cote d'Ivoire	1995	Age-standardized		5.8% (4.8 to 7.0%)	116.2 (86.5 to 147.5)
All causes	Cote d'Ivoire	2000	Age-standardized		5.6% (4.6 to 6.9%)	115.2 (87.4 to 150.9)
All causes	Cote d'Ivoire	2005	Age-standardized		5.1% (4.1 to 6.2%)	97.6 (72.9 to 128.1)
All causes	Cote d'Ivoire	2010	Age-standardized		5.4% (4.3 to 6.5%)	90.8 (63.5 to 127.7)
All causes	Cote d'Ivoire	2015	Age-standardized		5.2% (4.1 to 6.4%)	81.1 (50.8 to 121.7)
Lower respiratory infections	Cote d'Ivoire	1990	All Ages	3.7 (2.3 to 5.2)	23.6% (16.4 to 32.1%)	30.1 (19.1 to 42.7)
Lower respiratory infections	Cote d'Ivoire	1995	All Ages	4.6 (3.0 to 6.5)	23.3% (15.9 to 31.9%)	32.0 (21.1 to 45.3)
Lower respiratory infections	Cote d'Ivoire	2000	All Ages	4.9 (3.2 to 7.0)	23.0% (15.9 to 31.8%)	29.6 (19.2 to 42.7)
Lower respiratory infections	Cote d'Ivoire	2005	All Ages	4.0 (2.5 to 6.1)	20.7% (13.1 to 29.7%)	22.4 (13.6 to 33.5)
Lower respiratory infections	Cote d'Ivoire	2010	All Ages	4.1 (2.5 to 6.1)	21.2% (13.6 to 30.2%)	20.3 (12.3 to 30.2)
Lower respiratory infections	Cote d'Ivoire	2015	All Ages	3.8 (2.0 to 6.1)	18.4% (10.7 to 27.5%)	16.5 (9.0 to 26.9)
Lower respiratory infections	Cote d'Ivoire	1990	Age-standardized		23.6% (16.4 to 32.1%)	42.1 (27.5 to 58.9)
Lower respiratory infections	Cote d'Ivoire	1995	Age-standardized		23.3% (15.9 to 31.9%)	49.5 (31.5 to 70.7)
Lower respiratory infections	Cote d'Ivoire	2000	Age-standardized		23.0% (15.9 to 31.8%)	48.9 (30.8 to 71.9)
Lower respiratory infections	Cote d'Ivoire	2005	Age-standardized		20.7% (13.1 to 29.7%)	39.1 (24.0 to 59.4)
Lower respiratory infections	Cote d'Ivoire	2010	Age-standardized		21.2% (13.6 to 30.2%)	36.6 (21.9 to 57.1)
Lower respiratory infections	Cote d'Ivoire	2015	Age-standardized		18.4% (10.7 to 27.5%)	30.6 (16.0 to 52.8)
Tracheal, bronchus, and lung cancer	Cote d'Ivoire	1990	All Ages	0.1 (0.0 to 0.1)	16.9% (10.2 to 23.8%)	0.5 (0.3 to 0.8)
Tracheal, bronchus, and lung cancer	Cote d'Ivoire	1995	All Ages	0.1 (0.1 to 0.1)	16.7% (9.9 to 23.7%)	0.7 (0.4 to 1.0)
Tracheal, bronchus, and lung cancer	Cote d'Ivoire	2000	All Ages	0.1 (0.1 to 0.2)	16.4% (9.8 to 23.8%)	0.6 (0.4 to 1.0)
Tracheal, bronchus, and lung cancer	Cote d'Ivoire	2005	All Ages	0.1 (0.1 to 0.2)	14.6% (8.4 to 21.9%)	0.5 (0.3 to 0.9)
Tracheal, bronchus, and lung cancer	Cote d'Ivoire	2010	All Ages	0.1 (0.1 to 0.2)	15.0% (8.7 to 21.9%)	0.5 (0.3 to 0.9)
Tracheal, bronchus, and lung cancer	Cote d'Ivoire	2015	All Ages	0.1 (0.1 to 0.2)	12.8% (7.1 to 19.1%)	0.5 (0.2 to 1.0)
Tracheal, bronchus, and lung cancer	Cote d'Ivoire	1990	Age-standardized		17.0% (10.2 to 23.9%)	1.4 (0.8 to 2.2)
Tracheal, bronchus, and lung cancer	Cote d'Ivoire	1995	Age-standardized		16.7% (10.0 to 23.8%)	1.7 (1.0 to 2.7)
Tracheal, bronchus, and lung cancer	Cote d'Ivoire	2000	Age-standardized		16.5% (9.8 to 23.9%)	1.7 (1.0 to 2.8)
Tracheal, bronchus, and lung cancer	Cote d'Ivoire	2005	Age-standardized		14.7% (8.4 to 22.0%)	1.4 (0.8 to 2.3)
Tracheal, bronchus, and lung cancer	Cote d'Ivoire	2010	Age-standardized		15.0% (8.7 to 22.0%)	1.4 (0.7 to 2.5)

Cause name	Location	Year	Age group	Number (in thousands)	PAF	Rate (per 100,000)
Tracheal, bronchus, and lung cancer	Cote d'Ivoire	2015	Age-standardized		12.9% (7.2 to 19.2%)	1.3 (0.5 to 2.5)
Cardiovascular diseases	Cote d'Ivoire	1990	All Ages	2.0 (1.6 to 2.5)	13.4% (11.6 to 15.1%)	16.6 (12.9 to 20.7)
Cardiovascular diseases	Cote d'Ivoire	1995	All Ages	2.9 (2.0 to 3.8)	13.7% (11.9 to 15.5%)	20.0 (14.2 to 26.6)
Cardiovascular diseases	Cote d'Ivoire	2000	All Ages	3.3 (2.4 to 4.4)	13.9% (12.0 to 15.8%)	20.0 (14.5 to 27.0)
Cardiovascular diseases	Cote d'Ivoire	2005	All Ages	3.1 (2.3 to 4.2)	13.3% (11.4 to 15.3%)	17.3 (12.6 to 23.4)
Cardiovascular diseases	Cote d'Ivoire	2010	All Ages	3.2 (2.1 to 4.6)	13.4% (11.5 to 15.3%)	16.0 (10.5 to 23.0)
Cardiovascular diseases	Cote d'Ivoire	2015	All Ages	3.4 (1.9 to 5.6)	12.4% (10.5 to 14.4%)	15.2 (8.6 to 24.5)
Cardiovascular diseases	Cote d'Ivoire	1990	Age-standardized		12.4% (10.5 to 14.3%)	48.4 (37.5 to 59.3)
Cardiovascular diseases	Cote d'Ivoire	1995	Age-standardized		12.6% (10.7 to 14.6%)	55.4 (40.7 to 71.5)
Cardiovascular diseases	Cote d'Ivoire	2000	Age-standardized		12.7% (10.8 to 14.8%)	56.4 (41.9 to 74.1)
Cardiovascular diseases	Cote d'Ivoire	2005	Age-standardized		12.2% (10.3 to 14.2%)	50.1 (36.8 to 66.5)
Cardiovascular diseases	Cote d'Ivoire	2010	Age-standardized		12.3% (10.4 to 14.2%)	46.1 (31.2 to 64.5)
Cardiovascular diseases	Cote d'Ivoire	2015	Age-standardized		11.4% (9.4 to 13.4%)	43.1 (26.1 to 65.3)
Ischemic heart disease	Cote d'Ivoire	1990	All Ages	1.2 (0.9 to 1.5)	19.0% (15.9 to 22.3%)	9.6 (7.2 to 12.4)
Ischemic heart disease	Cote d'Ivoire	1995	All Ages	1.7 (1.2 to 2.3)	19.1% (15.8 to 22.4%)	12.0 (8.3 to 16.3)
Ischemic heart disease	Cote d'Ivoire	2000	All Ages	2.0 (1.4 to 2.7)	19.0% (15.7 to 22.2%)	11.9 (8.4 to 16.4)
Ischemic heart disease	Cote d'Ivoire	2005	All Ages	1.8 (1.3 to 2.5)	18.1% (14.8 to 21.4%)	10.0 (7.1 to 13.9)
Ischemic heart disease	Cote d'Ivoire	2010	All Ages	1.8 (1.2 to 2.7)	18.0% (15.0 to 21.0%)	9.1 (5.9 to 13.2)
Ischemic heart disease	Cote d'Ivoire	2015	All Ages	2.0 (1.1 to 3.2)	16.8% (13.8 to 19.9%)	8.7 (4.8 to 14.2)
Ischemic heart disease	Cote d'Ivoire	1990	Age-standardized		16.6% (13.1 to 20.0%)	28.9 (21.6 to 37.0)
Ischemic heart disease	Cote d'Ivoire	1995	Age-standardized		16.8% (13.3 to 20.1%)	33.8 (24.0 to 44.7)
Ischemic heart disease	Cote d'Ivoire	2000	Age-standardized		16.6% (13.1 to 20.0%)	34.1 (24.5 to 46.7)
Ischemic heart disease	Cote d'Ivoire	2005	Age-standardized		15.8% (12.4 to 19.1%)	29.6 (20.8 to 40.5)
Ischemic heart disease	Cote d'Ivoire	2010	Age-standardized		15.8% (12.5 to 19.0%)	26.8 (18.0 to 38.4)
Ischemic heart disease	Cote d'Ivoire	2015	Age-standardized		14.8% (11.6 to 18.0%)	25.1 (14.6 to 38.4)
Ischemic stroke	Cote d'Ivoire	1990	All Ages	0.3 (0.2 to 0.4)	13.9% (10.9 to 16.8%)	2.4 (1.7 to 3.2)
Ischemic stroke	Cote d'Ivoire	1995	All Ages	0.4 (0.3 to 0.5)	13.8% (10.8 to 16.8%)	2.7 (1.8 to 3.7)
Ischemic stroke	Cote d'Ivoire	2000	All Ages	0.4 (0.3 to 0.6)	13.7% (10.7 to 16.7%)	2.7 (1.9 to 3.8)
Ischemic stroke	Cote d'Ivoire	2005	All Ages	0.4 (0.3 to 0.6)	12.9% (9.9 to 16.0%)	2.4 (1.6 to 3.4)
Ischemic stroke	Cote d'Ivoire	2010	All Ages	0.5 (0.3 to 0.7)	12.9% (10.0 to 16.0%)	2.3 (1.4 to 3.5)
Ischemic stroke	Cote d'Ivoire	2015	All Ages	0.5 (0.3 to 0.8)	11.8% (8.8 to 14.7%)	2.2 (1.2 to 3.6)
Ischemic stroke	Cote d'Ivoire	1990	Age-standardized		12.4% (9.1 to 15.7%)	8.4 (5.9 to 11.5)
Ischemic stroke	Cote d'Ivoire	1995	Age-standardized		12.4% (9.1 to 15.7%)	9.1 (6.2 to 12.5)
Ischemic stroke	Cote d'Ivoire	2000	Age-standardized		12.2% (9.0 to 15.6%)	9.4 (6.3 to 13.1)
Ischemic stroke	Cote d'Ivoire	2005	Age-standardized		11.4% (8.3 to 14.7%)	8.6 (5.6 to 12.2)
Ischemic stroke	Cote d'Ivoire	2010	Age-standardized		11.5% (8.3 to 14.8%)	8.2 (5.0 to 11.9)

Cause name	Location	Year	Age group	Number (in thousands)	PAF	Rate (per 100,000)
Ischemic stroke	Cote d'Ivoire	2015	Age-standardized		10.6% (7.3 to 13.8%)	7.6 (4.2 to 12.3)
Hemorrhagic stroke	Cote d'Ivoire	1990	All Ages	0.6 (0.4 to 0.7)	15.1% (12.4 to 18.1%)	4.6 (3.4 to 6.1)
Hemorrhagic stroke	Cote d'Ivoire	1995	All Ages	0.8 (0.5 to 1.0)	15.2% (12.4 to 18.1%)	5.3 (3.7 to 7.2)
Hemorrhagic stroke	Cote d'Ivoire	2000	All Ages	0.9 (0.6 to 1.2)	15.1% (12.3 to 18.0%)	5.4 (3.8 to 7.5)
Hemorrhagic stroke	Cote d'Ivoire	2005	All Ages	0.9 (0.6 to 1.2)	14.2% (11.5 to 17.1%)	4.8 (3.3 to 6.9)
Hemorrhagic stroke	Cote d'Ivoire	2010	All Ages	0.9 (0.6 to 1.4)	14.2% (11.5 to 17.0%)	4.6 (2.8 to 6.9)
Hemorrhagic stroke	Cote d'Ivoire	2015	All Ages	1.0 (0.5 to 1.6)	13.1% (10.3 to 16.0%)	4.3 (2.3 to 7.2)
Hemorrhagic stroke	Cote d'Ivoire	1990	Age-standardized		14.2% (11.4 to 17.0%)	11.1 (8.3 to 14.7)
Hemorrhagic stroke	Cote d'Ivoire	1995	Age-standardized		14.3% (11.5 to 17.1%)	12.5 (8.9 to 16.6)
Hemorrhagic stroke	Cote d'Ivoire	2000	Age-standardized		14.1% (11.2 to 17.0%)	12.9 (9.0 to 17.6)
Hemorrhagic stroke	Cote d'Ivoire	2005	Age-standardized		13.2% (10.4 to 15.9%)	11.9 (8.2 to 16.7)
Hemorrhagic stroke	Cote d'Ivoire	2010	Age-standardized		13.2% (10.5 to 16.0%)	11.2 (7.1 to 16.2)
Hemorrhagic stroke	Cote d'Ivoire	2015	Age-standardized		12.1% (9.3 to 15.0%)	10.5 (5.9 to 16.9)
Chronic obstructive pulmonary disease	Cote d'Ivoire	1990	All Ages	0.4 (0.2 to 0.6)	22.5% (12.8 to 33.1%)	3.2 (1.7 to 5.0)
Chronic obstructive pulmonary disease	Cote d'Ivoire	1995	All Ages	0.4 (0.2 to 0.7)	22.1% (12.4 to 32.7%)	3.1 (1.6 to 5.0)
Chronic obstructive pulmonary disease	Cote d'Ivoire	2000	All Ages	0.4 (0.2 to 0.7)	21.7% (11.9 to 32.3%)	2.6 (1.3 to 4.2)
Chronic obstructive pulmonary disease	Cote d'Ivoire	2005	All Ages	0.4 (0.2 to 0.6)	20.1% (10.7 to 30.4%)	2.1 (1.0 to 3.6)
Chronic obstructive pulmonary disease	Cote d'Ivoire	2010	All Ages	0.4 (0.2 to 0.7)	20.4% (10.8 to 30.8%)	2.0 (1.0 to 3.6)
Chronic obstructive pulmonary disease	Cote d'Ivoire	2015	All Ages	0.4 (0.2 to 0.8)	18.3% (9.3 to 28.6%)	1.9 (0.8 to 3.7)
Chronic obstructive pulmonary disease	Cote d'Ivoire	1990	Age-standardized		23.2% (13.1 to 33.8%)	10.5 (5.6 to 16.7)
Chronic obstructive pulmonary disease	Cote d'Ivoire	1995	Age-standardized		22.9% (12.9 to 33.9%)	9.6 (5.0 to 15.5)
Chronic obstructive pulmonary disease	Cote d'Ivoire	2000	Age-standardized		22.6% (12.4 to 33.5%)	8.1 (4.2 to 13.2)
Chronic obstructive pulmonary disease	Cote d'Ivoire	2005	Age-standardized		20.8% (11.1 to 31.3%)	7.0 (3.5 to 11.5)
Chronic obstructive pulmonary disease	Cote d'Ivoire	2010	Age-standardized		21.1% (11.2 to 31.8%)	6.6 (3.3 to 11.3)
Chronic obstructive pulmonary disease	Cote d'Ivoire	2015	Age-standardized		18.9% (9.6 to 29.6%)	6.0 (2.6 to 11.5)
All causes	The Gambia	1990	All Ages	0.6 (0.4 to 0.8)	6.5% (4.7 to 8.5%)	59.3 (39.6 to 86.5)
All causes	The Gambia	1995	All Ages	0.6 (0.4 to 0.8)	6.1% (4.4 to 8.2%)	51.9 (34.1 to 73.8)
All causes	The Gambia	2000	All Ages	0.6 (0.4 to 0.8)	5.8% (4.3 to 7.7%)	44.9 (31.4 to 60.1)
All causes	The Gambia	2005	All Ages	0.5 (0.4 to 0.8)	5.3% (3.8 to 7.2%)	36.3 (25.4 to 51.6)
All causes	The Gambia	2010	All Ages	0.6 (0.4 to 0.8)	5.6% (4.0 to 7.4%)	33.3 (22.9 to 45.1)
All causes	The Gambia	2015	All Ages	0.6 (0.4 to 0.9)	5.8% (4.1 to 7.7%)	29.8 (19.4 to 43.4)
All causes	The Gambia	1990	Age-standardized		8.5% (6.5 to 10.8%)	115.3 (70.4 to 179.9)
All causes	The Gambia	1995	Age-standardized		8.3% (6.3 to 10.7%)	108.8 (69.5 to 160.2)
All causes	The Gambia	2000	Age-standardized		8.5% (6.4 to 11.0%)	107.7 (75.4 to 145.9)
All causes	The Gambia	2005	Age-standardized		7.6% (5.7 to 10.0%)	91.9 (64.5 to 127.7)
All causes	The Gambia	2010	Age-standardized		8.1% (6.1 to 10.7%)	92.3 (64.8 to 124.6)

Cause name	Location	Year	Age group	Number (in thousands)	PAF	Rate (per 100,000)
All causes	The Gambia	2015	Age-standardized		8.4% (6.2 to 10.8%)	89.2 (58.3 to 131.4)
Lower respiratory infections	The Gambia	1990	All Ages	0.4 (0.3 to 0.6)	32.7% (22.8 to 43.5%)	42.0 (27.0 to 60.6)
Lower respiratory infections	The Gambia	1995	All Ages	0.4 (0.2 to 0.6)	32.7% (22.5 to 44.5%)	36.0 (22.7 to 52.5)
Lower respiratory infections	The Gambia	2000	All Ages	0.4 (0.2 to 0.5)	33.1% (22.2 to 45.3%)	29.0 (18.8 to 40.7)
Lower respiratory infections	The Gambia	2005	All Ages	0.3 (0.2 to 0.5)	28.3% (18.0 to 40.2%)	22.5 (13.9 to 33.7)
Lower respiratory infections	The Gambia	2010	All Ages	0.3 (0.2 to 0.5)	30.7% (20.1 to 42.6%)	19.6 (12.1 to 28.1)
Lower respiratory infections	The Gambia	2015	All Ages	0.3 (0.2 to 0.5)	30.7% (19.5 to 42.6%)	16.6 (9.9 to 25.3)
Lower respiratory infections	The Gambia	1990	Age-standardized		32.7% (22.8 to 43.5%)	53.8 (32.2 to 85.7)
Lower respiratory infections	The Gambia	1995	Age-standardized		32.7% (22.5 to 44.5%)	52.1 (31.7 to 78.5)
Lower respiratory infections	The Gambia	2000	Age-standardized		33.1% (22.2 to 45.3%)	50.6 (31.6 to 73.9)
Lower respiratory infections	The Gambia	2005	Age-standardized		28.3% (18.0 to 40.2%)	41.4 (25.1 to 63.2)
Lower respiratory infections	The Gambia	2010	Age-standardized		30.7% (20.1 to 42.6%)	40.6 (24.3 to 60.7)
Lower respiratory infections	The Gambia	2015	Age-standardized		30.7% (19.5 to 42.6%)	38.4 (21.7 to 60.8)
Tracheal, bronchus, and lung cancer	The Gambia	1990	All Ages	0.0 (0.0 to 0.0)	25.2% (15.0 to 39.2%)	0.8 (0.4 to 1.6)
Tracheal, bronchus, and lung cancer	The Gambia	1995	All Ages	0.0 (0.0 to 0.0)	25.3% (15.0 to 39.9%)	0.8 (0.4 to 1.4)
Tracheal, bronchus, and lung cancer	The Gambia	2000	All Ages	0.0 (0.0 to 0.0)	25.8% (15.3 to 39.5%)	0.8 (0.4 to 1.3)
Tracheal, bronchus, and lung cancer	The Gambia	2005	All Ages	0.0 (0.0 to 0.0)	21.2% (12.0 to 34.4%)	0.6 (0.3 to 1.0)
Tracheal, bronchus, and lung cancer	The Gambia	2010	All Ages	0.0 (0.0 to 0.0)	23.5% (13.6 to 36.5%)	0.7 (0.4 to 1.1)
Tracheal, bronchus, and lung cancer	The Gambia	2015	All Ages	0.0 (0.0 to 0.0)	23.6% (12.7 to 37.7%)	0.7 (0.3 to 1.2)
Tracheal, bronchus, and lung cancer	The Gambia	1990	Age-standardized		25.6% (15.3 to 39.8%)	2.7 (1.4 to 5.2)
Tracheal, bronchus, and lung cancer	The Gambia	1995	Age-standardized		25.7% (15.3 to 40.5%)	2.7 (1.3 to 4.7)
Tracheal, bronchus, and lung cancer	The Gambia	2000	Age-standardized		26.2% (15.6 to 40.1%)	2.5 (1.4 to 4.2)
Tracheal, bronchus, and lung cancer	The Gambia	2005	Age-standardized		21.4% (12.1 to 34.9%)	2.1 (1.1 to 3.6)
Tracheal, bronchus, and lung cancer	The Gambia	2010	Age-standardized		23.8% (13.8 to 36.9%)	2.3 (1.3 to 3.9)
Tracheal, bronchus, and lung cancer	The Gambia	2015	Age-standardized		24.0% (12.9 to 38.2%)	2.3 (1.1 to 4.0)
Cardiovascular diseases	The Gambia	1990	All Ages	0.1 (0.1 to 0.2)	15.1% (12.1 to 18.4%)	13.1 (7.1 to 22.0)
Cardiovascular diseases	The Gambia	1995	All Ages	0.1 (0.1 to 0.2)	15.2% (12.3 to 18.6%)	12.4 (7.5 to 19.1)
Cardiovascular diseases	The Gambia	2000	All Ages	0.2 (0.1 to 0.2)	15.7% (12.5 to 19.5%)	13.0 (9.2 to 17.5)
Cardiovascular diseases	The Gambia	2005	All Ages	0.2 (0.1 to 0.2)	14.4% (11.4 to 17.9%)	11.4 (8.1 to 15.5)
Cardiovascular diseases	The Gambia	2010	All Ages	0.2 (0.1 to 0.3)	15.3% (12.2 to 19.0%)	11.3 (7.9 to 15.1)
Cardiovascular diseases	The Gambia	2015	All Ages	0.2 (0.1 to 0.3)	15.6% (12.3 to 19.0%)	10.9 (6.8 to 16.0)
Cardiovascular diseases	The Gambia	1990	Age-standardized		14.6% (11.4 to 17.9%)	45.9 (27.1 to 74.3)
Cardiovascular diseases	The Gambia	1995	Age-standardized		14.5% (11.6 to 17.9%)	43.9 (27.3 to 65.3)
Cardiovascular diseases	The Gambia	2000	Age-standardized		14.9% (11.6 to 18.8%)	46.1 (33.0 to 62.2)
Cardiovascular diseases	The Gambia	2005	Age-standardized		13.7% (10.5 to 17.2%)	41.5 (29.4 to 56.7)
Cardiovascular diseases	The Gambia	2010	Age-standardized		14.4% (11.2 to 18.1%)	42.1 (29.7 to 56.6)

Cause name	Location	Year	Age group	Number (in thousands)	PAF	Rate (per 100,000)
Cardiovascular diseases	The Gambia	2015	Age-standardized		14.4% (11.2 to 17.9%)	41.3 (26.9 to 59.1)
Ischemic heart disease	The Gambia	1990	All Ages	0.1 (0.0 to 0.1)	21.3% (17.1 to 25.7%)	7.3 (4.1 to 12.3)
Ischemic heart disease	The Gambia	1995	All Ages	0.1 (0.1 to 0.1)	21.1% (16.9 to 25.9%)	7.2 (4.4 to 10.9)
Ischemic heart disease	The Gambia	2000	All Ages	0.1 (0.1 to 0.1)	21.0% (16.5 to 25.8%)	7.4 (5.1 to 10.1)
Ischemic heart disease	The Gambia	2005	All Ages	0.1 (0.1 to 0.1)	19.3% (14.7 to 24.1%)	6.4 (4.6 to 8.7)
Ischemic heart disease	The Gambia	2010	All Ages	0.1 (0.1 to 0.1)	20.2% (15.9 to 25.5%)	6.2 (4.4 to 8.4)
Ischemic heart disease	The Gambia	2015	All Ages	0.1 (0.1 to 0.2)	20.4% (15.9 to 25.1%)	6.0 (3.8 to 8.7)
Ischemic heart disease	The Gambia	1990	Age-standardized		19.0% (14.8 to 23.8%)	26.5 (15.1 to 42.6)
Ischemic heart disease	The Gambia	1995	Age-standardized		18.9% (14.5 to 23.9%)	26.0 (15.9 to 39.1)
Ischemic heart disease	The Gambia	2000	Age-standardized		19.0% (14.3 to 24.1%)	27.1 (18.7 to 36.6)
Ischemic heart disease	The Gambia	2005	Age-standardized		17.4% (13.0 to 22.4%)	23.9 (16.8 to 32.1)
Ischemic heart disease	The Gambia	2010	Age-standardized		18.1% (13.7 to 23.5%)	23.7 (16.3 to 32.5)
Ischemic heart disease	The Gambia	2015	Age-standardized		18.1% (13.7 to 23.1%)	23.0 (14.8 to 33.0)
Ischemic stroke	The Gambia	1990	All Ages	0.0 (0.0 to 0.0)	16.8% (12.9 to 21.7%)	2.3 (1.2 to 3.9)
Ischemic stroke	The Gambia	1995	All Ages	0.0 (0.0 to 0.0)	16.7% (12.6 to 21.6%)	2.2 (1.3 to 3.5)
Ischemic stroke	The Gambia	2000	All Ages	0.0 (0.0 to 0.0)	16.6% (11.9 to 21.7%)	2.3 (1.5 to 3.3)
Ischemic stroke	The Gambia	2005	All Ages	0.0 (0.0 to 0.0)	14.7% (10.6 to 20.0%)	2.0 (1.3 to 2.9)
Ischemic stroke	The Gambia	2010	All Ages	0.0 (0.0 to 0.1)	15.6% (11.5 to 20.5%)	2.0 (1.3 to 2.8)
Ischemic stroke	The Gambia	2015	All Ages	0.0 (0.0 to 0.1)	15.7% (11.4 to 20.3%)	1.9 (1.1 to 2.9)
Ischemic stroke	The Gambia	1990	Age-standardized		15.3% (11.2 to 19.9%)	9.3 (5.1 to 15.3)
Ischemic stroke	The Gambia	1995	Age-standardized		15.2% (11.2 to 19.9%)	8.8 (5.2 to 13.8)
Ischemic stroke	The Gambia	2000	Age-standardized		15.3% (10.6 to 20.2%)	9.2 (5.9 to 13.4)
Ischemic stroke	The Gambia	2005	Age-standardized		13.6% (9.2 to 18.7%)	8.3 (5.2 to 12.3)
Ischemic stroke	The Gambia	2010	Age-standardized		14.3% (9.9 to 19.1%)	8.7 (5.4 to 12.8)
Ischemic stroke	The Gambia	2015	Age-standardized		14.3% (9.9 to 19.1%)	8.6 (5.1 to 13.5)
Hemorrhagic stroke	The Gambia	1990	All Ages	0.0 (0.0 to 0.1)	17.5% (13.5 to 22.3%)	3.4 (1.7 to 5.9)
Hemorrhagic stroke	The Gambia	1995	All Ages	0.0 (0.0 to 0.1)	17.5% (13.5 to 22.6%)	3.1 (1.8 to 4.8)
Hemorrhagic stroke	The Gambia	2000	All Ages	0.0 (0.0 to 0.1)	17.8% (13.5 to 23.3%)	3.2 (2.2 to 4.6)
Hemorrhagic stroke	The Gambia	2005	All Ages	0.0 (0.0 to 0.1)	16.0% (12.1 to 21.3%)	3.0 (2.0 to 4.2)
Hemorrhagic stroke	The Gambia	2010	All Ages	0.1 (0.0 to 0.1)	17.2% (13.2 to 22.1%)	3.1 (2.1 to 4.3)
Hemorrhagic stroke	The Gambia	2015	All Ages	0.1 (0.0 to 0.1)	17.5% (12.9 to 22.4%)	3.1 (1.8 to 4.7)
Hemorrhagic stroke	The Gambia	1990	Age-standardized		17.3% (13.3 to 22.1%)	10.1 (5.4 to 16.8)
Hemorrhagic stroke	The Gambia	1995	Age-standardized		17.2% (13.3 to 22.0%)	9.1 (5.4 to 14.4)
Hemorrhagic stroke	The Gambia	2000	Age-standardized		17.4% (12.8 to 22.6%)	9.8 (6.6 to 13.9)
Hemorrhagic stroke	The Gambia	2005	Age-standardized		15.3% (11.3 to 20.6%)	9.3 (6.1 to 13.1)
Hemorrhagic stroke	The Gambia	2010	Age-standardized		16.2% (12.2 to 21.1%)	9.8 (6.4 to 14.3)

Cause name	Location	Year	Age group	Number (in thousands)	PAF	Rate (per 100,000)
Hemorrhagic stroke	The Gambia	2015	Age-standardized		16.2% (11.9 to 20.7%)	9.7 (5.8 to 15.0)
Chronic obstructive pulmonary disease	The Gambia	1990	All Ages	0.0 (0.0 to 0.1)	29.8% (17.4 to 44.7%)	3.4 (1.6 to 6.5)
Chronic obstructive pulmonary disease	The Gambia	1995	All Ages	0.0 (0.0 to 0.1)	29.7% (17.7 to 44.3%)	2.7 (1.3 to 4.7)
Chronic obstructive pulmonary disease	The Gambia	2000	All Ages	0.0 (0.0 to 0.1)	30.1% (17.4 to 47.4%)	2.2 (1.2 to 3.7)
Chronic obstructive pulmonary disease	The Gambia	2005	All Ages	0.0 (0.0 to 0.0)	26.1% (14.3 to 40.4%)	1.7 (0.9 to 2.9)
Chronic obstructive pulmonary disease	The Gambia	2010	All Ages	0.0 (0.0 to 0.1)	28.2% (16.0 to 43.4%)	1.8 (0.9 to 2.9)
Chronic obstructive pulmonary disease	The Gambia	2015	All Ages	0.0 (0.0 to 0.1)	28.4% (16.1 to 43.8%)	1.7 (0.8 to 3.0)
Chronic obstructive pulmonary disease	The Gambia	1990	Age-standardized		30.8% (17.9 to 46.1%)	12.8 (6.0 to 23.9)
Chronic obstructive pulmonary disease	The Gambia	1995	Age-standardized		30.8% (18.3 to 45.9%)	10.0 (4.9 to 17.5)
Chronic obstructive pulmonary disease	The Gambia	2000	Age-standardized		31.2% (18.1 to 49.5%)	8.4 (4.6 to 13.9)
Chronic obstructive pulmonary disease	The Gambia	2005	Age-standardized		27.0% (14.7 to 41.8%)	6.9 (3.6 to 11.6)
Chronic obstructive pulmonary disease	The Gambia	2010	Age-standardized		29.1% (16.6 to 44.7%)	7.3 (3.8 to 11.9)
Chronic obstructive pulmonary disease	The Gambia	2015	Age-standardized		29.3% (16.5 to 45.2%)	7.2 (3.5 to 12.5)
All causes	Ghana	1990	All Ages	6.9 (5.1 to 9.3)	4.6% (3.7 to 5.6%)	47.0 (34.7 to 63.3)
All causes	Ghana	1995	All Ages	7.1 (5.4 to 9.2)	4.6% (3.7 to 5.7%)	42.3 (32.2 to 54.7)
All causes	Ghana	2000	All Ages	8.0 (6.0 to 10.4)	4.8% (3.9 to 5.8%)	42.6 (31.6 to 55.4)
All causes	Ghana	2005	All Ages	8.1 (5.6 to 11.2)	4.3% (3.3 to 5.4%)	37.9 (26.3 to 52.3)
All causes	Ghana	2010	All Ages	9.1 (5.6 to 13.7)	4.6% (3.5 to 5.8%)	37.4 (23.2 to 56.4)
All causes	Ghana	2015	All Ages	8.6 (4.8 to 13.9)	4.5% (3.4 to 5.8%)	31.5 (17.6 to 50.7)
All causes	Ghana	1990	Age-standardized		7.0% (5.9 to 8.3%)	113.5 (82.2 to 153.0)
All causes	Ghana	1995	Age-standardized		6.9% (5.7 to 8.1%)	100.5 (76.3 to 128.4)
All causes	Ghana	2000	Age-standardized		6.9% (5.8 to 8.2%)	102.9 (75.8 to 133.0)
All causes	Ghana	2005	Age-standardized		6.1% (4.9 to 7.4%)	88.2 (62.1 to 122.0)
All causes	Ghana	2010	Age-standardized		6.3% (5.1 to 7.6%)	84.6 (51.7 to 128.1)
All causes	Ghana	2015	Age-standardized		6.0% (4.8 to 7.4%)	74.8 (42.8 to 121.0)
Lower respiratory infections	Ghana	1990	All Ages	4.0 (2.7 to 5.7)	26.4% (18.8 to 34.6%)	27.2 (18.3 to 38.6)
Lower respiratory infections	Ghana	1995	All Ages	4.2 (2.8 to 5.8)	26.0% (18.3 to 34.1%)	24.8 (16.8 to 34.3)
Lower respiratory infections	Ghana	2000	All Ages	4.3 (2.9 to 5.9)	25.6% (17.9 to 33.8%)	22.7 (15.2 to 31.4)
Lower respiratory infections	Ghana	2005	All Ages	3.8 (2.2 to 6.0)	20.9% (12.7 to 30.2%)	18.0 (10.5 to 28.1)
Lower respiratory infections	Ghana	2010	All Ages	4.1 (2.3 to 6.6)	21.1% (12.8 to 30.7%)	16.7 (9.3 to 27.0)
Lower respiratory infections	Ghana	2015	All Ages	3.5 (1.7 to 6.4)	18.5% (10.1 to 28.4%)	12.9 (6.2 to 23.2)
Lower respiratory infections	Ghana	1990	Age-standardized		26.4% (18.8 to 34.6%)	50.4 (33.0 to 74.1)
Lower respiratory infections	Ghana	1995	Age-standardized		26.0% (18.3 to 34.1%)	46.8 (30.9 to 65.8)
Lower respiratory infections	Ghana	2000	Age-standardized		25.6% (17.9 to 33.8%)	44.9 (28.8 to 62.9)
Lower respiratory infections	Ghana	2005	Age-standardized		20.9% (12.7 to 30.2%)	35.1 (20.4 to 56.0)
Lower respiratory infections	Ghana	2010	Age-standardized		21.1% (12.8 to 30.7%)	32.2 (17.3 to 53.6)

Cause name	Location	Year	Age group	Number (in thousands)	PAF	Rate (per 100,000)
Lower respiratory infections	Ghana	2015	Age-standardized		18.5% (10.1 to 28.4%)	26.6 (12.4 to 48.8)
Tracheal, bronchus, and lung cancer	Ghana	1990	All Ages	0.1 (0.0 to 0.1)	18.9% (11.7 to 26.6%)	0.5 (0.3 to 0.8)
Tracheal, bronchus, and lung cancer	Ghana	1995	All Ages	0.1 (0.0 to 0.1)	18.6% (11.4 to 26.4%)	0.5 (0.3 to 0.7)
Tracheal, bronchus, and lung cancer	Ghana	2000	All Ages	0.1 (0.1 to 0.1)	18.3% (11.2 to 25.9%)	0.5 (0.3 to 0.7)
Tracheal, bronchus, and lung cancer	Ghana	2005	All Ages	0.1 (0.1 to 0.1)	14.7% (8.6 to 21.6%)	0.4 (0.2 to 0.7)
Tracheal, bronchus, and lung cancer	Ghana	2010	All Ages	0.1 (0.1 to 0.2)	14.9% (8.7 to 21.7%)	0.5 (0.2 to 0.8)
Tracheal, bronchus, and lung cancer	Ghana	2015	All Ages	0.1 (0.1 to 0.2)	13.0% (7.3 to 19.5%)	0.4 (0.2 to 0.9)
Tracheal, bronchus, and lung cancer	Ghana	1990	Age-standardized		19.0% (11.8 to 26.8%)	1.4 (0.7 to 2.2)
Tracheal, bronchus, and lung cancer	Ghana	1995	Age-standardized		18.7% (11.5 to 26.5%)	1.3 (0.7 to 1.9)
Tracheal, bronchus, and lung cancer	Ghana	2000	Age-standardized		18.4% (11.2 to 26.0%)	1.3 (0.7 to 1.9)
Tracheal, bronchus, and lung cancer	Ghana	2005	Age-standardized		14.8% (8.6 to 21.6%)	1.1 (0.6 to 1.8)
Tracheal, bronchus, and lung cancer	Ghana	2010	Age-standardized		14.9% (8.7 to 21.8%)	1.1 (0.6 to 2.0)
Tracheal, bronchus, and lung cancer	Ghana	2015	Age-standardized		13.0% (7.3 to 19.6%)	1.1 (0.5 to 2.0)
Cardiovascular diseases	Ghana	1990	All Ages	2.5 (1.7 to 3.7)	13.8% (12.0 to 15.7%)	17.2 (11.5 to 25.1)
Cardiovascular diseases	Ghana	1995	All Ages	2.6 (1.9 to 3.4)	13.4% (11.6 to 15.2%)	15.2 (11.1 to 20.2)
Cardiovascular diseases	Ghana	2000	All Ages	3.4 (2.4 to 4.4)	13.5% (11.7 to 15.4%)	17.8 (12.6 to 23.1)
Cardiovascular diseases	Ghana	2005	All Ages	3.8 (2.6 to 5.3)	13.2% (11.4 to 15.0%)	17.8 (12.0 to 24.7)
Cardiovascular diseases	Ghana	2010	All Ages	4.5 (2.5 to 7.0)	13.2% (11.2 to 15.1%)	18.5 (10.4 to 28.7)
Cardiovascular diseases	Ghana	2015	All Ages	4.6 (2.4 to 7.8)	12.6% (10.6 to 14.4%)	16.7 (8.8 to 28.4)
Cardiovascular diseases	Ghana	1990	Age-standardized		12.9% (10.9 to 15.0%)	54.7 (37.1 to 79.5)
Cardiovascular diseases	Ghana	1995	Age-standardized		12.6% (10.7 to 14.5%)	46.4 (34.2 to 60.8)
Cardiovascular diseases	Ghana	2000	Age-standardized		12.7% (10.7 to 14.8%)	51.3 (37.3 to 66.5)
Cardiovascular diseases	Ghana	2005	Age-standardized		12.4% (10.4 to 14.3%)	47.3 (31.8 to 64.0)
Cardiovascular diseases	Ghana	2010	Age-standardized		12.5% (10.5 to 14.5%)	46.5 (27.1 to 70.8)
Cardiovascular diseases	Ghana	2015	Age-standardized		11.6% (9.5 to 13.6%)	43.0 (23.7 to 70.6)
Ischemic heart disease	Ghana	1990	All Ages	1.3 (0.9 to 2.0)	20.0% (16.7 to 23.3%)	9.0 (5.9 to 13.4)
Ischemic heart disease	Ghana	1995	All Ages	1.4 (1.0 to 1.8)	19.5% (16.3 to 22.9%)	8.2 (5.9 to 10.9)
Ischemic heart disease	Ghana	2000	All Ages	1.9 (1.3 to 2.5)	19.7% (16.5 to 23.0%)	10.0 (7.0 to 13.3)
Ischemic heart disease	Ghana	2005	All Ages	2.1 (1.4 to 2.9)	17.7% (14.6 to 20.8%)	9.9 (6.6 to 13.6)
Ischemic heart disease	Ghana	2010	All Ages	2.5 (1.4 to 3.7)	17.3% (14.3 to 20.4%)	10.1 (5.8 to 15.4)
Ischemic heart disease	Ghana	2015	All Ages	2.6 (1.4 to 4.4)	16.7% (13.7 to 19.7%)	9.3 (5.0 to 15.9)
Ischemic heart disease	Ghana	1990	Age-standardized		17.5% (13.9 to 21.3%)	29.4 (19.6 to 42.0)
Ischemic heart disease	Ghana	1995	Age-standardized		17.3% (13.7 to 21.1%)	25.5 (18.1 to 34.4)
Ischemic heart disease	Ghana	2000	Age-standardized		17.5% (13.9 to 21.1%)	29.3 (20.2 to 38.6)
Ischemic heart disease	Ghana	2005	Age-standardized		16.0% (12.6 to 19.2%)	26.4 (17.6 to 36.1)
Ischemic heart disease	Ghana	2010	Age-standardized		15.9% (12.4 to 19.3%)	25.5 (14.8 to 38.6)

Cause name	Location	Year	Age group	Number (in thousands)	PAF	Rate (per 100,000)
Ischemic heart disease	Ghana	2015	Age-standardized		15.0% (11.6 to 18.3%)	24.3 (13.5 to 40.2)
Ischemic stroke	Ghana	1990	All Ages	0.5 (0.3 to 0.7)	15.0% (12.0 to 18.1%)	3.2 (2.0 to 4.8)
Ischemic stroke	Ghana	1995	All Ages	0.5 (0.3 to 0.7)	14.6% (11.5 to 17.5%)	2.9 (1.9 to 3.9)
Ischemic stroke	Ghana	2000	All Ages	0.6 (0.4 to 0.8)	14.6% (11.6 to 17.7%)	3.0 (2.0 to 4.2)
Ischemic stroke	Ghana	2005	All Ages	0.7 (0.5 to 1.0)	12.6% (9.6 to 15.5%)	3.4 (2.1 to 4.9)
Ischemic stroke	Ghana	2010	All Ages	0.9 (0.5 to 1.5)	12.4% (9.2 to 15.3%)	3.7 (2.0 to 6.1)
Ischemic stroke	Ghana	2015	All Ages	0.9 (0.4 to 1.5)	11.6% (8.5 to 14.6%)	3.1 (1.6 to 5.5)
Ischemic stroke	Ghana	1990	Age-standardized		13.4% (10.1 to 16.8%)	12.1 (7.5 to 17.9)
Ischemic stroke	Ghana	1995	Age-standardized		13.3% (9.9 to 16.7%)	10.1 (6.7 to 14.3)
Ischemic stroke	Ghana	2000	Age-standardized		13.2% (9.9 to 16.6%)	10.3 (6.7 to 14.2)
Ischemic stroke	Ghana	2005	Age-standardized		11.7% (8.4 to 15.0%)	10.4 (6.4 to 15.1)
Ischemic stroke	Ghana	2010	Age-standardized		11.6% (8.3 to 15.0%)	10.7 (5.6 to 17.3)
Ischemic stroke	Ghana	2015	Age-standardized		10.8% (7.5 to 14.2%)	9.5 (4.7 to 16.3)
Hemorrhagic stroke	Ghana	1990	All Ages	0.7 (0.5 to 1.1)	16.5% (13.6 to 19.5%)	5.0 (3.1 to 7.7)
Hemorrhagic stroke	Ghana	1995	All Ages	0.7 (0.5 to 1.0)	16.0% (13.3 to 18.9%)	4.2 (3.0 to 5.8)
Hemorrhagic stroke	Ghana	2000	All Ages	0.9 (0.6 to 1.2)	16.2% (13.3 to 19.1%)	4.7 (3.2 to 6.5)
Hemorrhagic stroke	Ghana	2005	All Ages	1.0 (0.6 to 1.5)	14.5% (11.8 to 17.4%)	4.6 (2.9 to 6.8)
Hemorrhagic stroke	Ghana	2010	All Ages	1.1 (0.6 to 1.9)	14.5% (11.7 to 17.3%)	4.7 (2.4 to 7.6)
Hemorrhagic stroke	Ghana	2015	All Ages	1.2 (0.6 to 2.0)	13.6% (10.8 to 16.6%)	4.2 (2.0 to 7.5)
Hemorrhagic stroke	Ghana	1990	Age-standardized		15.3% (12.4 to 18.3%)	13.2 (8.2 to 19.7)
Hemorrhagic stroke	Ghana	1995	Age-standardized		15.0% (12.2 to 17.9%)	10.8 (7.6 to 14.8)
Hemorrhagic stroke	Ghana	2000	Age-standardized		15.0% (12.2 to 18.0%)	11.7 (8.1 to 15.7)
Hemorrhagic stroke	Ghana	2005	Age-standardized		13.5% (10.7 to 16.3%)	10.5 (6.8 to 15.3)
Hemorrhagic stroke	Ghana	2010	Age-standardized		13.5% (10.7 to 16.5%)	10.3 (5.5 to 16.7)
Hemorrhagic stroke	Ghana	2015	Age-standardized		12.5% (9.7 to 15.4%)	9.2 (4.6 to 15.7)
Chronic obstructive pulmonary disease	Ghana	1990	All Ages	0.3 (0.1 to 0.5)	24.7% (14.2 to 35.7%)	2.1 (1.0 to 3.5)
Chronic obstructive pulmonary disease	Ghana	1995	All Ages	0.3 (0.2 to 0.5)	24.4% (13.9 to 35.4%)	1.8 (1.0 to 2.9)
Chronic obstructive pulmonary disease	Ghana	2000	All Ages	0.3 (0.2 to 0.5)	24.1% (13.7 to 34.9%)	1.7 (0.9 to 2.7)
Chronic obstructive pulmonary disease	Ghana	2005	All Ages	0.4 (0.2 to 0.6)	20.7% (10.8 to 31.1%)	1.6 (0.8 to 2.7)
Chronic obstructive pulmonary disease	Ghana	2010	All Ages	0.4 (0.2 to 0.7)	20.9% (10.7 to 31.6%)	1.8 (0.8 to 3.0)
Chronic obstructive pulmonary disease	Ghana	2015	All Ages	0.4 (0.2 to 0.8)	19.0% (9.1 to 29.6%)	1.5 (0.6 to 2.8)
Chronic obstructive pulmonary disease	Ghana	1990	Age-standardized		25.4% (14.6 to 36.8%)	7.1 (3.5 to 11.8)
Chronic obstructive pulmonary disease	Ghana	1995	Age-standardized		25.1% (14.3 to 36.4%)	6.0 (3.2 to 9.4)
Chronic obstructive pulmonary disease	Ghana	2000	Age-standardized		24.8% (14.2 to 35.9%)	5.5 (2.9 to 8.6)
Chronic obstructive pulmonary disease	Ghana	2005	Age-standardized		21.2% (11.0 to 31.8%)	4.8 (2.2 to 7.9)
Chronic obstructive pulmonary disease	Ghana	2010	Age-standardized		21.4% (10.9 to 32.2%)	4.7 (2.3 to 8.1)

Cause name	Location	Year	Age group	Number (in thousands)	PAF	Rate (per 100,000)
Chronic obstructive pulmonary disease	Ghana	2015	Age-standardized		19.3% (9.3 to 30.2%)	4.2 (1.7 to 7.5)
All causes	Guinea	1990	All Ages	4.3 (3.0 to 5.9)	4.5% (3.1 to 6.0%)	71.8 (49.9 to 97.5)
All causes	Guinea	1995	All Ages	5.0 (3.6 to 6.7)	4.4% (3.2 to 6.0%)	64.1 (45.7 to 86.3)
All causes	Guinea	2000	All Ages	5.1 (3.7 to 6.9)	4.3% (3.1 to 5.7%)	58.3 (42.2 to 77.9)
All causes	Guinea	2005	All Ages	4.4 (3.1 to 5.8)	3.8% (2.7 to 5.0%)	45.3 (32.0 to 60.1)
All causes	Guinea	2010	All Ages	5.0 (3.6 to 6.5)	4.2% (3.3 to 5.3%)	45.0 (32.8 to 59.0)
All causes	Guinea	2015	All Ages	4.6 (3.1 to 6.9)	3.8% (2.8 to 5.0%)	36.7 (24.4 to 54.7)
All causes	Guinea	1990	Age-standardized		5.6% (4.5 to 6.9%)	96.2 (76.0 to 122.0)
All causes	Guinea	1995	Age-standardized		5.7% (4.6 to 7.0%)	95.4 (75.5 to 119.6)
All causes	Guinea	2000	Age-standardized		5.8% (4.6 to 7.0%)	98.5 (76.3 to 124.9)
All causes	Guinea	2005	Age-standardized		5.3% (4.2 to 6.6%)	86.7 (65.2 to 111.9)
All causes	Guinea	2010	Age-standardized		6.0% (4.9 to 7.2%)	93.3 (70.7 to 119.4)
All causes	Guinea	2015	Age-standardized		5.3% (4.2 to 6.6%)	81.5 (53.7 to 119.2)
Lower respiratory infections	Guinea	1990	All Ages	3.3 (1.9 to 4.8)	22.3% (14.8 to 30.8%)	54.5 (32.0 to 80.2)
Lower respiratory infections	Guinea	1995	All Ages	3.6 (2.2 to 5.4)	22.3% (14.8 to 31.0%)	46.8 (28.8 to 69.0)
Lower respiratory infections	Guinea	2000	All Ages	3.5 (2.2 to 5.1)	22.3% (14.9 to 31.0%)	40.0 (24.6 to 58.2)
Lower respiratory infections	Guinea	2005	All Ages	2.8 (1.6 to 4.1)	20.0% (12.0 to 29.2%)	28.7 (16.6 to 42.7)
Lower respiratory infections	Guinea	2010	All Ages	2.9 (1.9 to 4.2)	21.8% (14.3 to 30.6%)	26.7 (17.0 to 37.9)
Lower respiratory infections	Guinea	2015	All Ages	2.4 (1.4 to 4.0)	17.9% (10.3 to 27.3%)	19.3 (10.7 to 31.5)
Lower respiratory infections	Guinea	1990	Age-standardized		22.3% (14.8 to 30.8%)	48.6 (31.5 to 69.9)
Lower respiratory infections	Guinea	1995	Age-standardized		22.3% (14.8 to 31.0%)	49.2 (31.9 to 71.9)
Lower respiratory infections	Guinea	2000	Age-standardized		22.3% (14.9 to 31.0%)	48.4 (30.1 to 69.8)
Lower respiratory infections	Guinea	2005	Age-standardized		20.0% (12.0 to 29.2%)	39.3 (22.7 to 58.8)
Lower respiratory infections	Guinea	2010	Age-standardized		21.8% (14.3 to 30.6%)	40.8 (25.6 to 59.0)
Lower respiratory infections	Guinea	2015	Age-standardized		17.9% (10.3 to 27.3%)	32.4 (17.3 to 54.3)
Tracheal, bronchus, and lung cancer	Guinea	1990	All Ages	0.0 (0.0 to 0.1)	15.8% (9.5 to 22.7%)	0.6 (0.3 to 0.9)
Tracheal, bronchus, and lung cancer	Guinea	1995	All Ages	0.1 (0.0 to 0.1)	15.7% (9.3 to 22.6%)	0.7 (0.4 to 1.1)
Tracheal, bronchus, and lung cancer	Guinea	2000	All Ages	0.1 (0.0 to 0.1)	15.8% (9.5 to 22.7%)	0.8 (0.4 to 1.2)
Tracheal, bronchus, and lung cancer	Guinea	2005	All Ages	0.1 (0.0 to 0.1)	14.0% (8.0 to 20.7%)	0.7 (0.3 to 1.0)
Tracheal, bronchus, and lung cancer	Guinea	2010	All Ages	0.1 (0.0 to 0.1)	15.4% (9.0 to 22.5%)	0.8 (0.4 to 1.3)
Tracheal, bronchus, and lung cancer	Guinea	2015	All Ages	0.1 (0.0 to 0.2)	12.4% (7.0 to 18.6%)	0.7 (0.3 to 1.4)
Tracheal, bronchus, and lung cancer	Guinea	1990	Age-standardized		15.8% (9.5 to 22.8%)	1.3 (0.8 to 2.0)
Tracheal, bronchus, and lung cancer	Guinea	1995	Age-standardized		15.8% (9.4 to 22.7%)	1.5 (0.9 to 2.2)
Tracheal, bronchus, and lung cancer	Guinea	2000	Age-standardized		15.9% (9.5 to 22.8%)	1.7 (0.9 to 2.6)
Tracheal, bronchus, and lung cancer	Guinea	2005	Age-standardized		14.1% (8.0 to 20.8%)	1.5 (0.8 to 2.3)
Tracheal, bronchus, and lung cancer	Guinea	2010	Age-standardized		15.5% (9.1 to 22.6%)	1.8 (0.9 to 2.9)

Cause name	Location	Year	Age group	Number (in thousands)	PAF	Rate (per 100,000)
Tracheal, bronchus, and lung cancer	Guinea	2015	Age-standardized		12.5% (7.0 to 18.8%)	1.5 (0.7 to 3.0)
Cardiovascular diseases	Guinea	1990	All Ages	0.8 (0.7 to 1.0)	12.0% (10.2 to 13.7%)	14.0 (11.1 to 17.3)
Cardiovascular diseases	Guinea	1995	All Ages	1.1 (0.9 to 1.4)	12.2% (10.6 to 14.1%)	14.3 (11.6 to 18.1)
Cardiovascular diseases	Guinea	2000	All Ages	1.4 (1.1 to 1.7)	12.8% (10.9 to 14.6%)	15.5 (12.2 to 19.8)
Cardiovascular diseases	Guinea	2005	All Ages	1.4 (1.0 to 1.8)	12.4% (10.6 to 14.2%)	14.1 (10.7 to 18.0)
Cardiovascular diseases	Guinea	2010	All Ages	1.7 (1.2 to 2.2)	13.1% (11.3 to 15.1%)	15.3 (11.1 to 20.2)
Cardiovascular diseases	Guinea	2015	All Ages	1.8 (1.1 to 2.8)	12.0% (10.2 to 13.8%)	14.6 (8.9 to 22.4)
Cardiovascular diseases	Guinea	1990	Age-standardized		11.6% (9.7 to 13.4%)	38.4 (30.0 to 47.4)
Cardiovascular diseases	Guinea	1995	Age-standardized		11.8% (9.9 to 13.7%)	38.3 (30.5 to 47.8)
Cardiovascular diseases	Guinea	2000	Age-standardized		12.1% (10.1 to 14.2%)	42.3 (33.1 to 54.0)
Cardiovascular diseases	Guinea	2005	Age-standardized		11.6% (9.7 to 13.5%)	40.2 (30.4 to 51.7)
Cardiovascular diseases	Guinea	2010	Age-standardized		12.2% (10.3 to 14.2%)	43.7 (32.2 to 57.4)
Cardiovascular diseases	Guinea	2015	Age-standardized		11.1% (9.1 to 13.0%)	41.3 (26.6 to 61.3)
Ischemic heart disease	Guinea	1990	All Ages	0.5 (0.4 to 0.6)	17.8% (14.7 to 21.0%)	7.8 (6.0 to 9.8)
Ischemic heart disease	Guinea	1995	All Ages	0.7 (0.5 to 0.8)	17.7% (14.8 to 20.9%)	8.4 (6.5 to 10.6)
Ischemic heart disease	Guinea	2000	All Ages	0.8 (0.6 to 1.0)	17.8% (14.8 to 20.9%)	9.1 (6.8 to 11.9)
Ischemic heart disease	Guinea	2005	All Ages	0.8 (0.6 to 1.0)	17.0% (14.1 to 20.2%)	8.1 (6.0 to 10.5)
Ischemic heart disease	Guinea	2010	All Ages	0.9 (0.7 to 1.3)	17.8% (14.8 to 21.0%)	8.5 (6.1 to 11.5)
Ischemic heart disease	Guinea	2015	All Ages	1.0 (0.6 to 1.6)	16.3% (13.3 to 19.4%)	8.2 (4.9 to 12.7)
Ischemic heart disease	Guinea	1990	Age-standardized		15.7% (12.3 to 19.2%)	22.3 (16.7 to 28.6)
Ischemic heart disease	Guinea	1995	Age-standardized		15.8% (12.4 to 19.2%)	23.1 (17.3 to 29.6)
Ischemic heart disease	Guinea	2000	Age-standardized		16.0% (12.4 to 19.3%)	25.4 (18.8 to 33.3)
Ischemic heart disease	Guinea	2005	Age-standardized		15.2% (11.8 to 18.5%)	23.5 (17.1 to 30.7)
Ischemic heart disease	Guinea	2010	Age-standardized		15.8% (12.4 to 19.2%)	24.9 (17.3 to 33.5)
Ischemic heart disease	Guinea	2015	Age-standardized		14.5% (11.2 to 17.7%)	23.7 (14.7 to 35.2)
Ischemic stroke	Guinea	1990	All Ages	0.1 (0.1 to 0.2)	13.3% (10.3 to 16.4%)	2.5 (1.7 to 3.5)
Ischemic stroke	Guinea	1995	All Ages	0.2 (0.1 to 0.3)	13.1% (10.1 to 16.2%)	2.4 (1.7 to 3.4)
Ischemic stroke	Guinea	2000	All Ages	0.2 (0.2 to 0.3)	13.1% (10.1 to 16.2%)	2.6 (1.8 to 3.7)
Ischemic stroke	Guinea	2005	All Ages	0.2 (0.1 to 0.3)	12.2% (9.2 to 15.2%)	2.3 (1.6 to 3.3)
Ischemic stroke	Guinea	2010	All Ages	0.3 (0.2 to 0.4)	12.9% (9.9 to 15.9%)	2.6 (1.7 to 3.7)
Ischemic stroke	Guinea	2015	All Ages	0.3 (0.2 to 0.5)	11.5% (8.6 to 14.4%)	2.4 (1.4 to 4.0)
Ischemic stroke	Guinea	1990	Age-standardized		12.0% (8.7 to 15.2%)	7.7 (5.2 to 11.1)
Ischemic stroke	Guinea	1995	Age-standardized		11.9% (8.6 to 15.1%)	7.3 (5.1 to 10.3)
Ischemic stroke	Guinea	2000	Age-standardized		11.9% (8.7 to 15.2%)	8.0 (5.3 to 11.4)
Ischemic stroke	Guinea	2005	Age-standardized		11.1% (8.0 to 14.4%)	7.8 (5.0 to 11.2)
Ischemic stroke	Guinea	2010	Age-standardized		11.7% (8.5 to 15.0%)	8.6 (5.7 to 12.5)

Cause name	Location	Year	Age group	Number (in thousands)	PAF	Rate (per 100,000)
Ischemic stroke	Guinea	2015	Age-standardized		10.4% (7.3 to 13.6%)	8.0 (4.7 to 12.9)
Hemorrhagic stroke	Guinea	1990	All Ages	0.2 (0.2 to 0.3)	13.2% (10.7 to 15.8%)	3.7 (2.7 to 4.8)
Hemorrhagic stroke	Guinea	1995	All Ages	0.3 (0.2 to 0.4)	13.4% (10.9 to 16.0%)	3.5 (2.6 to 4.5)
Hemorrhagic stroke	Guinea	2000	All Ages	0.3 (0.3 to 0.5)	13.9% (11.3 to 16.6%)	3.9 (2.8 to 5.1)
Hemorrhagic stroke	Guinea	2005	All Ages	0.4 (0.3 to 0.5)	13.2% (10.5 to 16.0%)	3.7 (2.6 to 4.9)
Hemorrhagic stroke	Guinea	2010	All Ages	0.5 (0.3 to 0.6)	14.1% (11.4 to 16.9%)	4.2 (3.0 to 5.8)
Hemorrhagic stroke	Guinea	2015	All Ages	0.5 (0.3 to 0.8)	12.6% (9.9 to 15.4%)	4.0 (2.3 to 6.4)
Hemorrhagic stroke	Guinea	1990	Age-standardized		13.3% (10.5 to 16.1%)	8.4 (6.1 to 11.1)
Hemorrhagic stroke	Guinea	1995	Age-standardized		13.4% (10.7 to 16.2%)	7.9 (5.8 to 10.5)
Hemorrhagic stroke	Guinea	2000	Age-standardized		13.6% (10.8 to 16.5%)	8.9 (6.4 to 11.9)
Hemorrhagic stroke	Guinea	2005	Age-standardized		12.7% (9.9 to 15.6%)	8.9 (6.3 to 12.0)
Hemorrhagic stroke	Guinea	2010	Age-standardized		13.3% (10.5 to 16.3%)	10.2 (7.0 to 14.1)
Hemorrhagic stroke	Guinea	2015	Age-standardized		11.9% (9.2 to 14.7%)	9.6 (5.7 to 14.9)
Chronic obstructive pulmonary disease	Guinea	1990	All Ages	0.2 (0.1 to 0.3)	21.3% (11.7 to 31.7%)	2.7 (1.3 to 4.4)
Chronic obstructive pulmonary disease	Guinea	1995	All Ages	0.2 (0.1 to 0.3)	21.1% (11.6 to 31.3%)	2.3 (1.1 to 3.7)
Chronic obstructive pulmonary disease	Guinea	2000	All Ages	0.2 (0.1 to 0.3)	21.1% (11.3 to 31.8%)	2.1 (1.0 to 3.3)
Chronic obstructive pulmonary disease	Guinea	2005	All Ages	0.2 (0.1 to 0.3)	19.5% (10.2 to 30.0%)	1.9 (0.9 to 3.0)
Chronic obstructive pulmonary disease	Guinea	2010	All Ages	0.3 (0.1 to 0.4)	21.0% (11.0 to 31.2%)	2.3 (1.1 to 3.7)
Chronic obstructive pulmonary disease	Guinea	2015	All Ages	0.3 (0.1 to 0.5)	18.0% (8.9 to 28.1%)	2.0 (0.8 to 3.9)
Chronic obstructive pulmonary disease	Guinea	1990	Age-standardized		22.1% (12.2 to 32.9%)	7.9 (3.8 to 12.9)
Chronic obstructive pulmonary disease	Guinea	1995	Age-standardized		22.0% (12.0 to 32.6%)	6.5 (3.2 to 10.4)
Chronic obstructive pulmonary disease	Guinea	2000	Age-standardized		22.0% (11.8 to 33.1%)	6.1 (3.0 to 9.7)
Chronic obstructive pulmonary disease	Guinea	2005	Age-standardized		20.3% (10.6 to 31.0%)	5.8 (2.9 to 9.4)
Chronic obstructive pulmonary disease	Guinea	2010	Age-standardized		21.7% (11.4 to 32.4%)	7.1 (3.5 to 11.4)
Chronic obstructive pulmonary disease	Guinea	2015	Age-standardized		18.6% (9.2 to 28.9%)	6.3 (2.7 to 11.7)
All causes	Guinea-Bissau	1990	All Ages	0.8 (0.5 to 1.4)	4.7% (3.5 to 6.1%)	77.0 (43.5 to 135.3)
All causes	Guinea-Bissau	1995	All Ages	0.8 (0.5 to 1.6)	4.7% (3.3 to 6.2%)	70.7 (38.6 to 134.4)
All causes	Guinea-Bissau	2000	All Ages	0.9 (0.5 to 1.7)	4.7% (3.3 to 6.1%)	67.1 (35.4 to 128.1)
All causes	Guinea-Bissau	2005	All Ages	0.8 (0.4 to 1.7)	4.1% (2.7 to 5.6%)	57.1 (27.0 to 113.6)
All causes	Guinea-Bissau	2010	All Ages	1.0 (0.5 to 2.1)	4.7% (3.2 to 6.3%)	60.2 (28.7 to 126.1)
All causes	Guinea-Bissau	2015	All Ages	1.0 (0.4 to 2.2)	4.4% (2.9 to 5.9%)	52.0 (23.3 to 117.7)
All causes	Guinea-Bissau	1990	Age-standardized		6.4% (5.1 to 7.7%)	120.7 (57.1 to 236.5)
All causes	Guinea-Bissau	1995	Age-standardized		6.5% (5.2 to 7.9%)	120.0 (55.7 to 246.7)
All causes	Guinea-Bissau	2000	Age-standardized		6.6% (5.1 to 8.1%)	122.4 (56.0 to 250.2)
All causes	Guinea-Bissau	2005	Age-standardized		5.7% (4.3 to 7.3%)	108.7 (45.5 to 228.0)
All causes	Guinea-Bissau	2010	Age-standardized		6.4% (4.9 to 8.0%)	117.9 (51.0 to 255.9)

Cause name	Location	Year	Age group	Number (in thousands)	PAF	Rate (per 100,000)
All causes	Guinea-Bissau	2015	Age-standardized		6.0% (4.5 to 7.8%)	107.5 (46.4 to 243.5)
Lower respiratory infections	Guinea-Bissau	1990	All Ages	0.5 (0.3 to 0.8)	27.0% (18.9 to 35.0%)	50.3 (30.8 to 78.3)
Lower respiratory infections	Guinea-Bissau	1995	All Ages	0.5 (0.3 to 0.9)	26.9% (19.3 to 34.9%)	46.0 (26.6 to 76.3)
Lower respiratory infections	Guinea-Bissau	2000	All Ages	0.6 (0.3 to 1.0)	26.8% (18.8 to 35.7%)	42.5 (24.4 to 73.2)
Lower respiratory infections	Guinea-Bissau	2005	All Ages	0.5 (0.3 to 0.9)	22.1% (13.6 to 31.4%)	34.0 (17.4 to 60.6)
Lower respiratory infections	Guinea-Bissau	2010	All Ages	0.6 (0.3 to 1.1)	24.9% (16.6 to 33.8%)	35.2 (18.6 to 64.2)
Lower respiratory infections	Guinea-Bissau	2015	All Ages	0.5 (0.3 to 1.0)	23.0% (14.5 to 32.7%)	27.7 (13.4 to 55.3)
Lower respiratory infections	Guinea-Bissau	1990	Age-standardized		27.0% (18.9 to 35.0%)	54.5 (27.1 to 100.9)
Lower respiratory infections	Guinea-Bissau	1995	Age-standardized		26.9% (19.3 to 34.9%)	57.0 (27.5 to 111.4)
Lower respiratory infections	Guinea-Bissau	2000	Age-standardized		26.8% (18.8 to 35.7%)	58.1 (26.9 to 117.4)
Lower respiratory infections	Guinea-Bissau	2005	Age-standardized		22.1% (13.6 to 31.4%)	47.8 (20.4 to 98.3)
Lower respiratory infections	Guinea-Bissau	2010	Age-standardized		24.9% (16.6 to 33.8%)	51.5 (22.9 to 109.5)
Lower respiratory infections	Guinea-Bissau	2015	Age-standardized		23.0% (14.5 to 32.7%)	44.0 (18.2 to 98.8)
Tracheal, bronchus, and lung cancer	Guinea-Bissau	1990	All Ages	0.0 (0.0 to 0.0)	19.7% (12.0 to 28.1%)	0.9 (0.3 to 2.3)
Tracheal, bronchus, and lung cancer	Guinea-Bissau	1995	All Ages	0.0 (0.0 to 0.0)	19.6% (12.1 to 27.6%)	0.9 (0.3 to 2.1)
Tracheal, bronchus, and lung cancer	Guinea-Bissau	2000	All Ages	0.0 (0.0 to 0.0)	19.5% (12.2 to 28.3%)	0.8 (0.3 to 2.1)
Tracheal, bronchus, and lung cancer	Guinea-Bissau	2005	All Ages	0.0 (0.0 to 0.0)	15.7% (8.8 to 23.0%)	0.7 (0.2 to 1.8)
Tracheal, bronchus, and lung cancer	Guinea-Bissau	2010	All Ages	0.0 (0.0 to 0.0)	17.9% (10.7 to 26.3%)	0.8 (0.2 to 2.3)
Tracheal, bronchus, and lung cancer	Guinea-Bissau	2015	All Ages	0.0 (0.0 to 0.0)	16.4% (9.4 to 24.4%)	0.8 (0.2 to 2.3)
Tracheal, bronchus, and lung cancer	Guinea-Bissau	1990	Age-standardized		19.8% (12.1 to 28.2%)	2.0 (0.7 to 4.8)
Tracheal, bronchus, and lung cancer	Guinea-Bissau	1995	Age-standardized		19.7% (12.2 to 27.8%)	2.0 (0.7 to 4.8)
Tracheal, bronchus, and lung cancer	Guinea-Bissau	2000	Age-standardized		19.6% (12.3 to 28.5%)	2.0 (0.7 to 4.8)
Tracheal, bronchus, and lung cancer	Guinea-Bissau	2005	Age-standardized		15.8% (8.8 to 23.2%)	1.8 (0.5 to 4.4)
Tracheal, bronchus, and lung cancer	Guinea-Bissau	2010	Age-standardized		18.0% (10.7 to 26.5%)	2.1 (0.7 to 5.4)
Tracheal, bronchus, and lung cancer	Guinea-Bissau	2015	Age-standardized		16.5% (9.4 to 24.6%)	2.0 (0.6 to 5.3)
Cardiovascular diseases	Guinea-Bissau	1990	All Ages	0.2 (0.1 to 0.5)	13.4% (11.2 to 15.8%)	20.6 (7.7 to 48.0)
Cardiovascular diseases	Guinea-Bissau	1995	All Ages	0.2 (0.1 to 0.6)	13.4% (11.3 to 15.8%)	19.9 (7.3 to 50.1)
Cardiovascular diseases	Guinea-Bissau	2000	All Ages	0.3 (0.1 to 0.7)	13.9% (11.7 to 16.5%)	20.7 (7.3 to 49.6)
Cardiovascular diseases	Guinea-Bissau	2005	All Ages	0.3 (0.1 to 0.7)	12.8% (10.6 to 15.4%)	19.8 (6.6 to 48.4)
Cardiovascular diseases	Guinea-Bissau	2010	All Ages	0.4 (0.1 to 0.9)	13.8% (11.4 to 16.6%)	21.3 (7.1 to 54.4)
Cardiovascular diseases	Guinea-Bissau	2015	All Ages	0.4 (0.1 to 1.1)	13.4% (10.9 to 16.3%)	20.6 (7.0 to 57.5)
Cardiovascular diseases	Guinea-Bissau	1990	Age-standardized		12.9% (10.6 to 15.2%)	50.9 (21.2 to 107.4)
Cardiovascular diseases	Guinea-Bissau	1995	Age-standardized		13.0% (10.7 to 15.3%)	50.4 (20.7 to 111.0)
Cardiovascular diseases	Guinea-Bissau	2000	Age-standardized		13.4% (11.1 to 15.9%)	53.5 (21.2 to 113.8)
Cardiovascular diseases	Guinea-Bissau	2005	Age-standardized		12.3% (9.8 to 14.7%)	51.8 (19.4 to 116.8)
Cardiovascular diseases	Guinea-Bissau	2010	Age-standardized		13.1% (10.7 to 15.7%)	55.7 (21.1 to 128.5)

Cause name	Location	Year	Age group	Number (in thousands)	PAF	Rate (per 100,000)
Cardiovascular diseases	Guinea-Bissau	2015	Age-standardized		12.6% (10.1 to 15.3%)	53.4 (20.6 to 129.1)
Ischemic heart disease	Guinea-Bissau	1990	All Ages	0.1 (0.0 to 0.3)	19.5% (16.1 to 23.3%)	11.3 (4.3 to 26.5)
Ischemic heart disease	Guinea-Bissau	1995	All Ages	0.1 (0.1 to 0.3)	19.4% (15.9 to 23.2%)	11.4 (4.2 to 27.7)
Ischemic heart disease	Guinea-Bissau	2000	All Ages	0.2 (0.1 to 0.4)	19.4% (16.0 to 23.4%)	11.8 (4.2 to 28.4)
Ischemic heart disease	Guinea-Bissau	2005	All Ages	0.2 (0.1 to 0.4)	17.9% (14.3 to 21.4%)	11.1 (3.7 to 27.2)
Ischemic heart disease	Guinea-Bissau	2010	All Ages	0.2 (0.1 to 0.5)	19.1% (15.5 to 23.0%)	11.8 (4.0 to 30.7)
Ischemic heart disease	Guinea-Bissau	2015	All Ages	0.2 (0.1 to 0.6)	18.4% (14.8 to 22.3%)	11.4 (3.9 to 31.6)
Ischemic heart disease	Guinea-Bissau	1990	Age-standardized		17.5% (13.7 to 21.8%)	28.7 (11.7 to 60.4)
Ischemic heart disease	Guinea-Bissau	1995	Age-standardized		17.5% (13.9 to 21.5%)	29.4 (11.9 to 64.8)
Ischemic heart disease	Guinea-Bissau	2000	Age-standardized		17.5% (13.9 to 21.6%)	31.2 (12.4 to 68.1)
Ischemic heart disease	Guinea-Bissau	2005	Age-standardized		16.0% (12.3 to 19.8%)	29.8 (11.1 to 66.0)
Ischemic heart disease	Guinea-Bissau	2010	Age-standardized		17.0% (13.1 to 21.0%)	31.4 (11.8 to 71.9)
Ischemic heart disease	Guinea-Bissau	2015	Age-standardized		16.3% (12.5 to 20.2%)	30.2 (11.6 to 71.8)
Ischemic stroke	Guinea-Bissau	1990	All Ages	0.0 (0.0 to 0.1)	14.8% (11.4 to 18.0%)	3.4 (1.3 to 7.5)
Ischemic stroke	Guinea-Bissau	1995	All Ages	0.0 (0.0 to 0.1)	14.6% (11.3 to 17.9%)	3.2 (1.2 to 7.2)
Ischemic stroke	Guinea-Bissau	2000	All Ages	0.0 (0.0 to 0.1)	14.5% (11.0 to 18.1%)	3.2 (1.1 to 7.2)
Ischemic stroke	Guinea-Bissau	2005	All Ages	0.0 (0.0 to 0.1)	12.9% (9.5 to 16.3%)	3.1 (1.0 to 7.1)
Ischemic stroke	Guinea-Bissau	2010	All Ages	0.1 (0.0 to 0.1)	14.0% (10.4 to 17.4%)	3.3 (1.1 to 8.0)
Ischemic stroke	Guinea-Bissau	2015	All Ages	0.1 (0.0 to 0.1)	13.3% (9.9 to 17.1%)	3.1 (1.1 to 7.9)
Ischemic stroke	Guinea-Bissau	1990	Age-standardized		13.5% (9.9 to 16.8%)	9.7 (4.0 to 19.3)
[truncated: 532,410 more chars]
